# Student engagement, assessed using heart rate, shows no reset following active learning sessions in lectures

**DOI:** 10.1371/journal.pone.0225709

**Published:** 2019-12-02

**Authors:** Diana K. Darnell, Paul A. Krieg

**Affiliations:** Department of Cellular and Molecular Medicine, University of Arizona College of Medicine, Tucson, Arizona, United States of America; University of South Australia, AUSTRALIA

## Abstract

Heart rate can be used as a measure of cognitive engagement. We measured average student heart rates during medical school lecture classes using wristwatch-style monitors. Analysis of 42 classes showed a steady decline in heart rate from the beginning to end of a lecture class. Active learning sessions (peer-discussion based problem solving) resulted in a significant uptick in heart rate, but this returned to the average level immediately following the active learning period. This is the first statistically robust assessment of changes in heart rate during the course of college lecture classes and indicates that personal heart rate monitors may be useful tools for assessment of different teaching modalities. The key findings suggest that the value of active learning within the classroom resides in the activity itself and not in an increase in engagement or reset in attention during the didactic period following an active learning session.

## Introduction

Teaching that involves active learning (AL) results in improved student outcomes [1]. Active learning can range from interactive studio or workshop course designs, or (as in our study) AL can be lecture interrupted by occasional student-centered activities such as small group problem solving, worksheets, and personal response challenges with or without peer discussion. [1]. What exactly are the features of AL that result in the improved outcomes? Numerous studies have shown that periods of AL where recently received information was manipulated or interpreted improved long-term retention [2–4]). This is especially the case if students enthusiastically embrace AL interactions and pedagogy [5–6]. Active learning can be beneficial in fully flipped classes [7], and when inserted into traditional lectures. Many speaker-training and faculty development publications, (including those for TED talks, University of Arizona, Cornell University Center for Teaching Excellence, Montana State University, Oregon Health Sciences University, West Virginia University, etc.), also base their inclusion and spacing of AL sessions within lectures on the “common knowledge” that attention span becomes a limiting factor in learning after 10–15 minutes of listening to a speaker [8–10]. The suggestion is that periods of AL will reset attention to its longer, start-of-lecture span [11–15] (and see Fig 6A), thereby enhancing retention of lecture material presented after the AL period. In the classroom, this would suggest that time spent in an activity, even a relatively unproductive AL exercise, might be repaid by increased attention after the break. This idea was originally proposed more than 40 years ago based on observations of a few faculty and their students in the context of an otherwise unrelated study [11]. The idea of attention reset is also based on a frequently cited, but hypothetical model by Bligh, proposing improved attention after a break in lecture [14]. Bligh’s studies showed that heart rate was an objective measure of engagement and correlated well with the observed drop in student attention across a lecture [14]. He also provided limited evidence that heart rate went up with questions and discussion but did not test the hypothesis that a lecture break restores attention. In this study, we have attempted to address this gap in knowledge by measuring how student heart rate changes over the course of medical school lectures in response active learning events and ascertain if attention span reset is a valid expectation on which to base course design.

A number of different approaches have been used to measure student engagement in the classroom. The most direct methods used self-reporting, or behavioral indicators such as posture, note taking, or fidgeting as measures of engagement; but such methods can be distracting, labor intensive and potentially subjective. Over the last few decades, objective physiological measurements have been used as indirect indicators of engagement or cognitive effort, mostly in laboratory settings. These methods include skin conductivity [16–18], blood pressure [19], heart rate [14, 19, 20–22] and pupil size [23–27]. Recently, heart rate variation, HRV, a gauge of the variation in time between consecutive heartbeats, has been used as a possible measure of attention and cognitive performance, and HRV has been seen to correlate with independent subjective measures of engagement [28–31]. Although indirect, application of these physiological methods to relatively large numbers of individuals in the classroom might provide non-subjective measures of physiological response to different teaching methods and act as a proxy measurement of classroom focus and engagement.

Use of heart rate to indicate engagement in a mental task has been studied for some time and correlates closely with independent measurements such as pupil size and skin resistance [32–33]. Subsequent studies have confirmed that heart rate and engagement in a task are closely correlated, with HR increasing during the superior learning performance [34]. In addition, increased heart rate correlates with greater cognitive effort and higher-order problem solving [22, 35–37], with the difference in heart rate between engaged/high effort and low cognitive engagement being about 5 bpm [22]. A similar rate difference has been reported between higher-order cognitive engagement (thinking slow) and rote-level engagement (thinking fast) [38]. Heart rate rise with cognitive demand is independent of blood glucose levels [39] and somatically matched control tasks. While informative, none of these studies took place in a classroom setting.

In a widely-cited study, Bligh employed heart rate as an objective measure to assess student engagement in the college lecture classroom [14]. Using results from four student traces from four classes (16 traces overall), this research indicated that attention span and HR decreased from the beginning of an 80-minute class to the end. Based on data from non-lecture-related attention assessments it was hypothesized that HR might increase in response to an interruption or break in the lecture, such as a rest, a student question or a short discussion [14]. The conclusion that students’ attention flags during lecture and his suggestions for making lecture more engaging are referenced by hundreds of articles in the active-learning literature. However, we have found no published attempts using heart rate measurement to replicate these seminal results nor test this hypothesis. Additionally, whereas the original studies were carried out in classes where the presentations were almost exclusively didactic, the prevalence of AL in the classroom now makes it possible to examine how engagement, measured by HR, changes over the course of classes that include periods of AL.

In our study, student heart rate was measured during lecture classes using wrist-worn HR monitors to assess the effects of different classroom events or activities on altering heart rate, as a surrogate measure of student engagement. The majority of activities involved adjacent students discussing and evaluating didactic material that had just been presented in the lecture, or students working together to solve problems or fill in worksheets together that involve didactic material. No other physical activity was involved in the AL events. Question-answer exchanges between individual students and the teacher were not considered to be AL for our studies and are not included in the analysis. Since different modes of active learning have not been specified in the seminal meta-analysis demonstrating the value of AL in student outcomes [1], we also pooled data from the variety of activities present in our classes. Similarly, the passive learning events involving short videos and animations were also pooled. Whereas most previous studies of attention looked at evidence for isolated lectures, we have monitored the same group of students during as many as 4 consecutive 50-minute lecture periods on a given day (with short breaks in between). This approach allows us to consider how time of day and/or the overall duration of didactic experience on a given day affect the ability of students to remain engaged.

## Methods

Fifteen student volunteers were recruited from the first year of medical school at a Public Medical School in the Southwestern US. All student provided informed written consent per the IRB requirements and received no compensation for participation. The University of Arizona Institutional Review Board specifically approved this study (IRB Protocol #1405330884). Our student population that year was 50% male/female, average age 25 (range 20–48), at least 55% white, <6% each African, African American, American Indian, Alaskan Native; Asian: Chinese, Japanese, Pakistani, Korean, Taiwanese, Vietnamese; and 22% declined to state. Ethnically, 8.6% self-identified as Hispanic/Chicanx/Latinx or of Spanish Origin. Average GPA 3.7 and Average MCAT 30.5. All students held Bachelor’s degrees and 21 also held Master’s degrees; 2 had a Doctorate. Twenty-five percent of our students report being ‘disadvantaged,’ which includes individuals who grew up in an area that was medically underserved or had insufficient access to social, economic, or educational opportunities. In Arizona that is typically students who are first in their family to attend a 4-year college or university, from a rural or U.S./Mexico border community, or are otherwise educationally or economically disadvantaged. Our volunteer sub-population was 70.5% female and 70.5% white (non-Hispanic), with the remainder West and East Asian and Hispanic. Other demographics were not recorded.

Volunteers were given Mio alpha heart rate monitors (Mioglobal.com/alpha 53P5C2L3) to wear. The Mio monitors report HR approximately one time per second, but do not provide measures of heart rate variability. Monitors were synchronized to the second with one another prior to distribution. Heart rate data were collected on individual devices using the Smokey Cat Software Heart Graph application, Simple Tracking. Graphic and spreadsheet data were collected from all participants and summarized in Microsoft Excel, where traces were aligned with a second-accurate timeline of the lecture. Twenty-eight classes with too few recording students were removed from further analysis. All students participating and present submitted their heart rate data in the form of .tcx files that were converted to .csv files. These were imported into excel and aligned by class start using the time/date stamp associated with the start of the file. Files were expanded to create one cell per second, which created occasional gaps of one cell in the data. These were filled by integrating flanking heart rate data. The resulting files contained heart rates for all attending students extending consistently from start to end of class. Class averages per second were calculated, as were grand average heart rates per student and per class. Student grand deviations from each class grand mean was added or subtracted per second to normalize each student’s trace to the class average without changing any scaling or individual variation in response. Individual responses that deviated from the class mean by greater than 2 SD for that second were replaced with the class mean to subtract non-class related spikes. The resulting normalized data was averaged and graphed directly and as a 40 second rolling average against a line trace representing classroom activities (S1 Dataset).

Bligh identified an initial "settling down period" in which heart rate dropped precipitously, and an uptick in heart rates at the end of the class [14]. While the precise duration is somewhat variable, inspection of multiple average HR graphs for our classes showed similar changes in slope apparently unrelated to instruction. Based on this observation, the first and last three minutes of the direct trace for each class was analyzed for its slope, and finding they were different from the core of the class (Fig 2, S2 Dataset), these data were cropped and the core of the class was used for additional analysis. For activities, average heart rates for the period during the activity was extracted, as were four minutes of heart rates preceding and following the activity (one-minute gap to account for the transition). Activities ranged in length from 40 second to 11 minutes with an average of 4 minutes 54 seconds.

Not all students participating in the study attended all classes, leaving unbalanced input data. Given the concern that students participating frequently might bias the data in some way correlated with the measurements, a multivariate analysis using before/activity/after, individual student and AL session as variables was done in R (S1 Table). First a one-way ANOVA and then a two-way ANOVA compared the effects on HR of 1) the activity to the period before, and after as a three-way comparison; 2) the period before to after as a two-way comparison; 3) the period before to the activity as a two-way comparison; and 4) the activity to the period after as a two-way comparison. The ANOVA function in the R package 'car' was used to take the unbalanced input data into consideration. While HR frequency was used as the measurement throughout the research, our study can be divided into two primary sections. First is replication of the results Bligh [14], including 1), decrease in average HR over the course of a class, 2) rapid reduction in HR and increase in HR at the beginning and end of classes, respectively; and 3), assessing if classroom events may result in a change in student HR. Second, the novel experiments in our study focused on the effects of modern classroom methods on student attention, as correlated with HR, including 1), the effects of AL sessions on HR and 2), the effect of videos on HR, and finally, a test of the dogma that AL resets student physiology to a higher level during subsequent didactics. Of the 22 AL events analyzed, ten were discussions with an immediate neighbor to review and develop understanding of didactic material (think-pair-share) and twelve involved application of previous didactic material to problem solving, again with neighbor discussion.

## Results

### Results using personal HR monitors replicate previous studies

**Fig 1:**
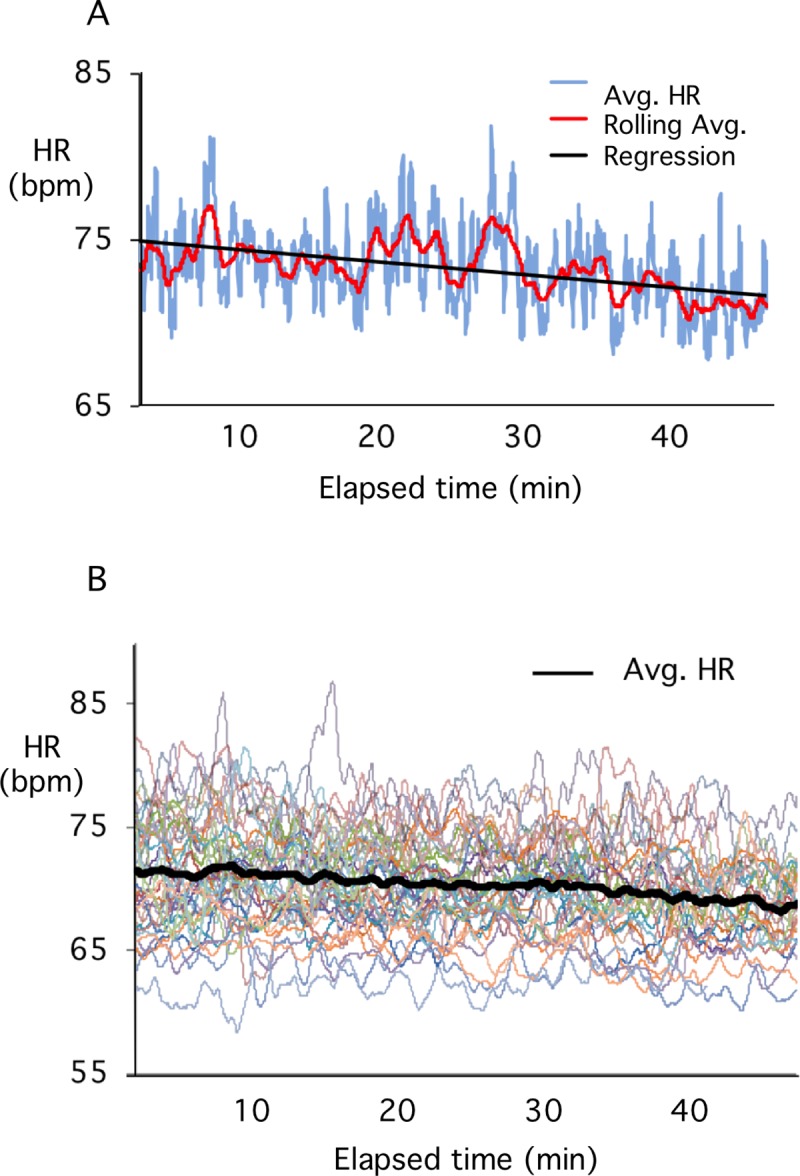
A). Average (blue) and rolling average of HR (red) for a total of 10 students over approximately 48 minutes of a lecture class. The first and last 3 minutes of the class were removed from the analysis (see text). The regression line for the HR over the duration of the class is shown in black. B). Average of HR activity (solid black line) for a total of 42 lecture classes (individual colored lines). Data includes class averages from 310 student traces at one second intervals. Note the steady decrease in HR between the beginning and end of the lecture period.

Our initial studies were directed towards confirming the core observations of classroom engagement reported by Bligh [14] with statistically rigorous methods. His observations may be summarized as follows—1) HR decreases from the beginning to end of a lecture, 2) the drop in HR is biphasic, with a greater decrease during the early stages of the lecture. Whereas Bligh recorded 950 readings (16 traces at 5 second intervals) to plot each 5-minute averaged data point [14], we recorded 93,000 readings (310 traces at 1 second intervals) in each 5-minute period and plotted the average of each second and a 40 second rolling average. We also tested his hypothesis that interruptions to the normal flow of lecture by student discussion or rest would result in increased heart rate/performance during and after the event, respectively. Student HR was assessed during a total of 42 medical school lecture classes. An example of an averaged HR trace from a typical single lecture class is presented in Fig 1A and shows the slight drop in HR from start to finish indicated by the regression line. In this case, the regression line starts at about 74 beats per minute (bpm) and drops to 70.5 bpm after 48 minutes for a total decrease of 3.5 bpm over the entire lecture. The result of averaging the HR over the entire 42 classes (310 student traces) is illustrated in Fig 1B. Calculation of the regression for the combined classes shows a statistically significant decrease in HR from an average of 71.8 at the start, to 68.9 by the end the lecture, a decrease of 2.9bpm (P<0.001).

This analysis confirms the original observation that HR decreases over the course of a standard lecture class. Our observations suggest a reduction of about 3.75 bpm in HR per hour, nearly identical to Bligh’s observations [14]. The biphasic shift from faster decline in the first 30 minutes to a slower decline for the remainder of the class was not confirmed.

**Fig 2:**
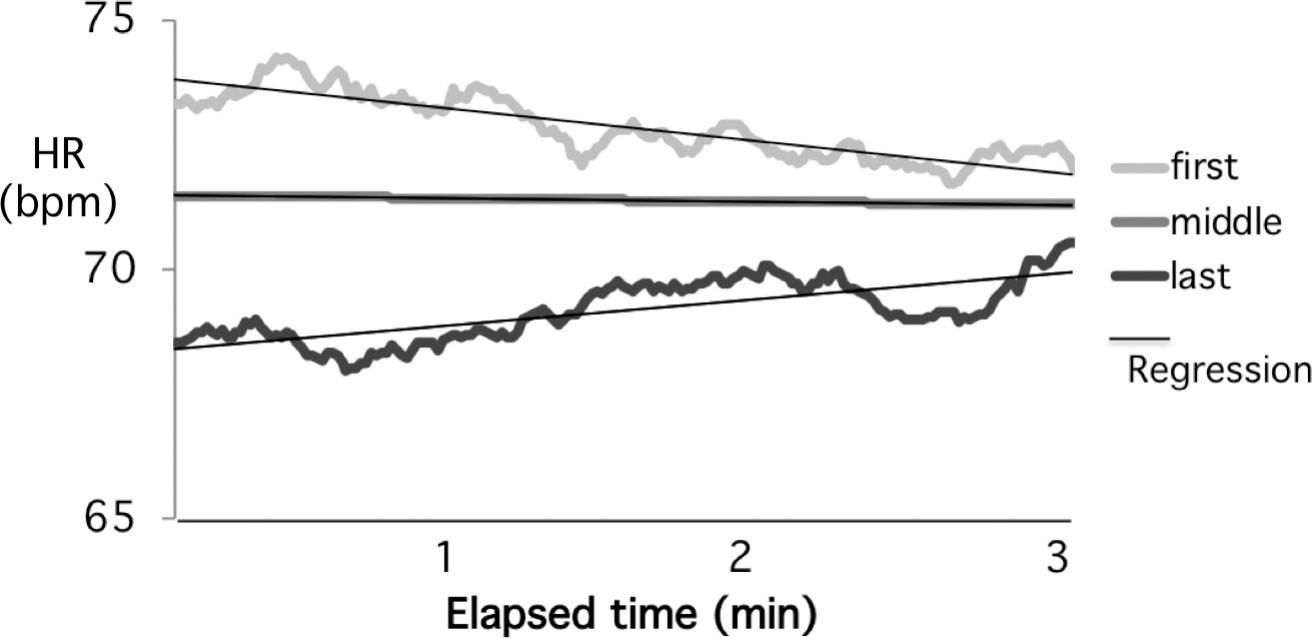
Average HR changes much more rapidly in the first and last 3 minutes (elapsed time) compared to the remainder of the class. The rates of change at the ends are approximately 10-fold the rate change during the class (contrast the absolute value of the slopes (bpm/h): Start -37.5, End +29.0, Middle -3.75).

Previous reports have suggested that student attention drops rapidly during the first few minutes of class and then rises again at the end [40–41] and this observation was supported by HR measurements which showed a rapid drop early in the class and and then an uptick at the end of the period [14]. To determine whether these changes were present in our data set, we analyzed the rate of HR change over the first three minutes and last three minutes of all classes compared to the HR change over the entire period between these time points. As shown in Fig 2, the HR averages and regression analysis confirm the rapid changes at the beginning and end of the lecture. Indeed, the rate of HR change is approximately 10-fold greater at the two ends of the lecture period compared to the middle. Although our alterations in HR go in opposite directions, we were concerned that these rapid changes might affect the slope analysis for the entire class and so all other regression calculations were carried out excluding the first and last three minutes.

**Fig 3:**
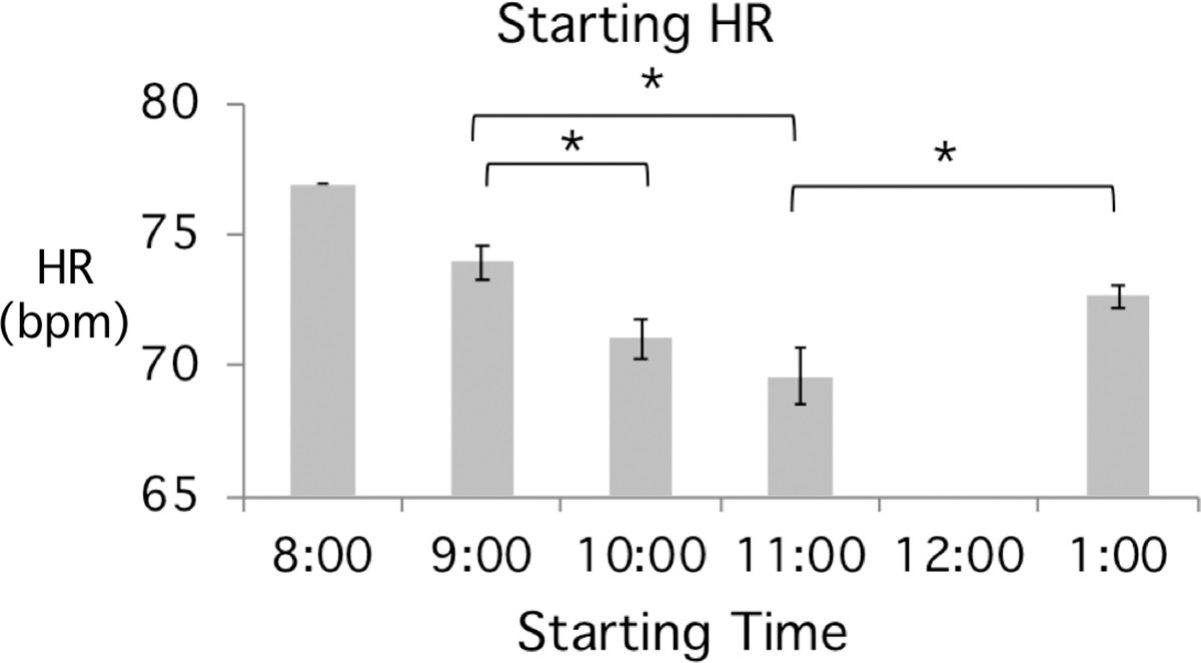
Average starting HR decreases as classes occur later during the morning. Statistically significant differences are indicated (Student T-test p<0.05). Bars show standard error of the mean. Classes analyzed at 8, 9, 10, 11 am and 1pm have n = 1, 12, 17, 9, 3, respectively.

It has been suggested that student attention/engagement might change with the time of day [20, 42–43]. We examined our data to determine whether the time of day when a lecture occurred made any difference to the average HR response (Fig 3). The results show that the starting HR, measured as the Y-intercept of the regression line at the time that the class commenced, decreased steadily during the morning, but that an apparent partial reset occurred after the lunch break. The differences in starting HR for 9 am and 10 am, and 9 am and 11 am classes are statistically significant, but there are insufficient numbers for the significance of the 8 am or 1pm classes to be determined.

### Activities during the lecture alter HR

**Fig 4:**
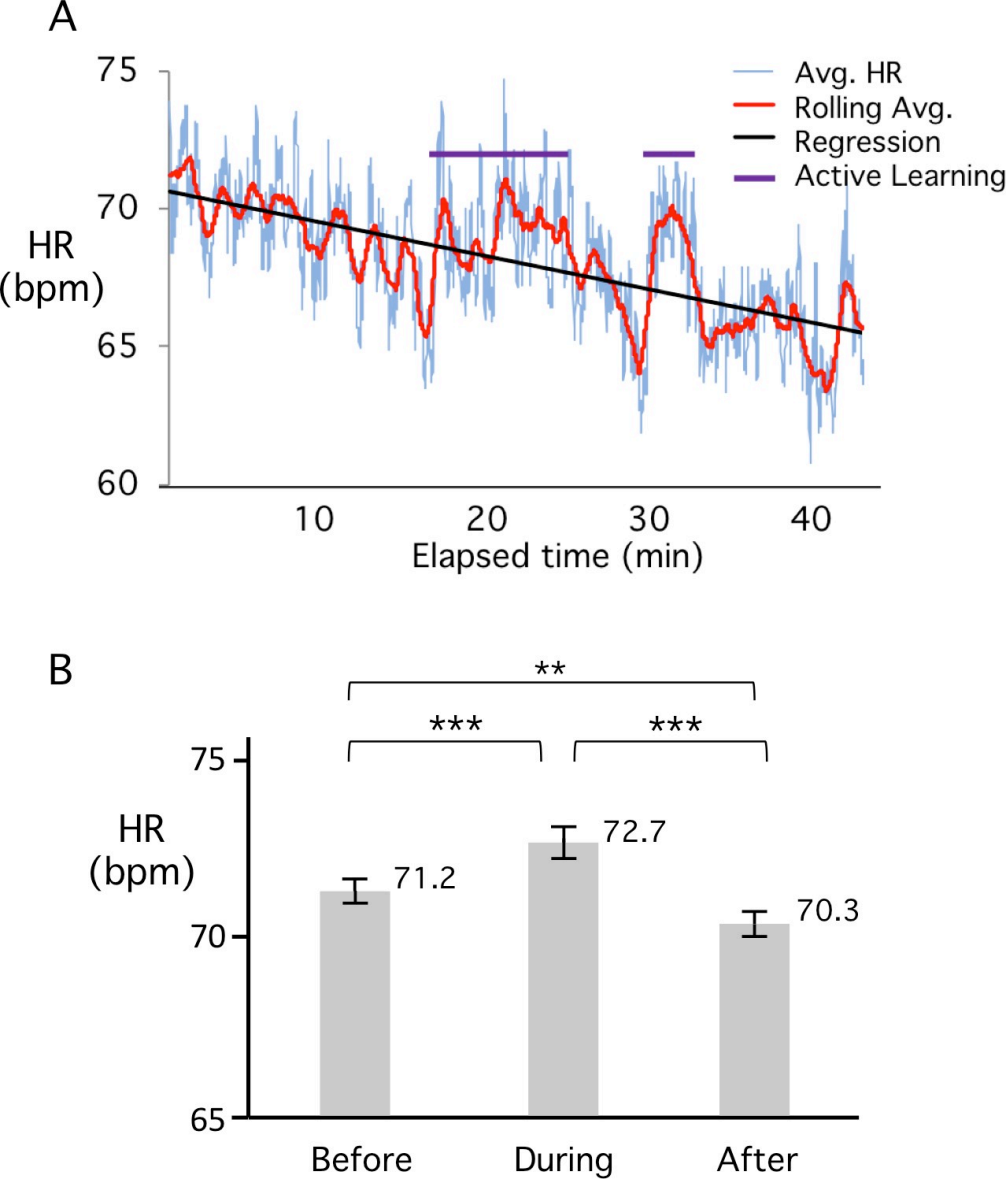
Average HR increases during AL activities. A. HR profile illustrating a typical lecture containing two AL sessions indicated by purple bars. In both cases, AL was a think-pair-share activity. B). Change in average HR during AL compared to adjacent lecture periods. A total of 22 different AL sessions were analyzed from 13 different classes. Average HR increased 1.5 bpm +/- SE during the activity. Statistical significance as determined by Two-Way Random Effects Analysis is indicated on the plot. (** P<0.01, *** P<0.001). Note that the average HR does not increase after the activity.

The primary objective of our studies was to determine whether different classroom activities, particularly those considered to be interactive, would result in alterations in student engagement, as correlated to HR frequency. To assess this question, we examined average HR during those times when students were involved in AL sessions during the lecture. AL modules included think-pair-share sessions, exercises in data analysis and paired solving of Board exam-style questions. An example of a typical averaged HR tracing during a class that contained 2 AL sessions is illustrated in Fig 4A. In this profile, the HR during the times when AL was taking place (purple overline), appears to be higher than the HR for other times in the class. To examine this matter more closely, we averaged HR during 22 different AL events and compared this to 4 minutes preceding and following the event (Fig 4B). The results show a statistically significant increase of about 1.5 bpm in the average HR during the activity. However, after the activity, the HR immediately returned to a level very similar to or even lower than the HR before. That is, there was no reset to a higher HR, correlating with increased attention, due to the insertion of an activity.

Previous studies have shown that a rest from an activity can allow a reset in engagement when the activity is recommenced [44–48]. Bligh [14,20] also suggests (but does not test) that a rest or an interactive session may be equally effective in re-setting heart rate. We have analyzed the HR at the end of one class and compared it to the starting HR of the immediately following class. In each case, lecture segments were separated by a 5–10 minute break of unstructured activity, for example, stretching, talking, standing up or checking texts. Analysis of HR on either side of 10 between-class breaks, with the same teacher on both sides of the break, shows no significant difference between the ending and starting HR (average reset in HR is +0.15 bpm, P = 0.80; S2 Table). This would appear to indicate that even a complete break from the structured classroom procedure is not sufficient to reset engagement as measured by HR frequency.

**Fig 5:**
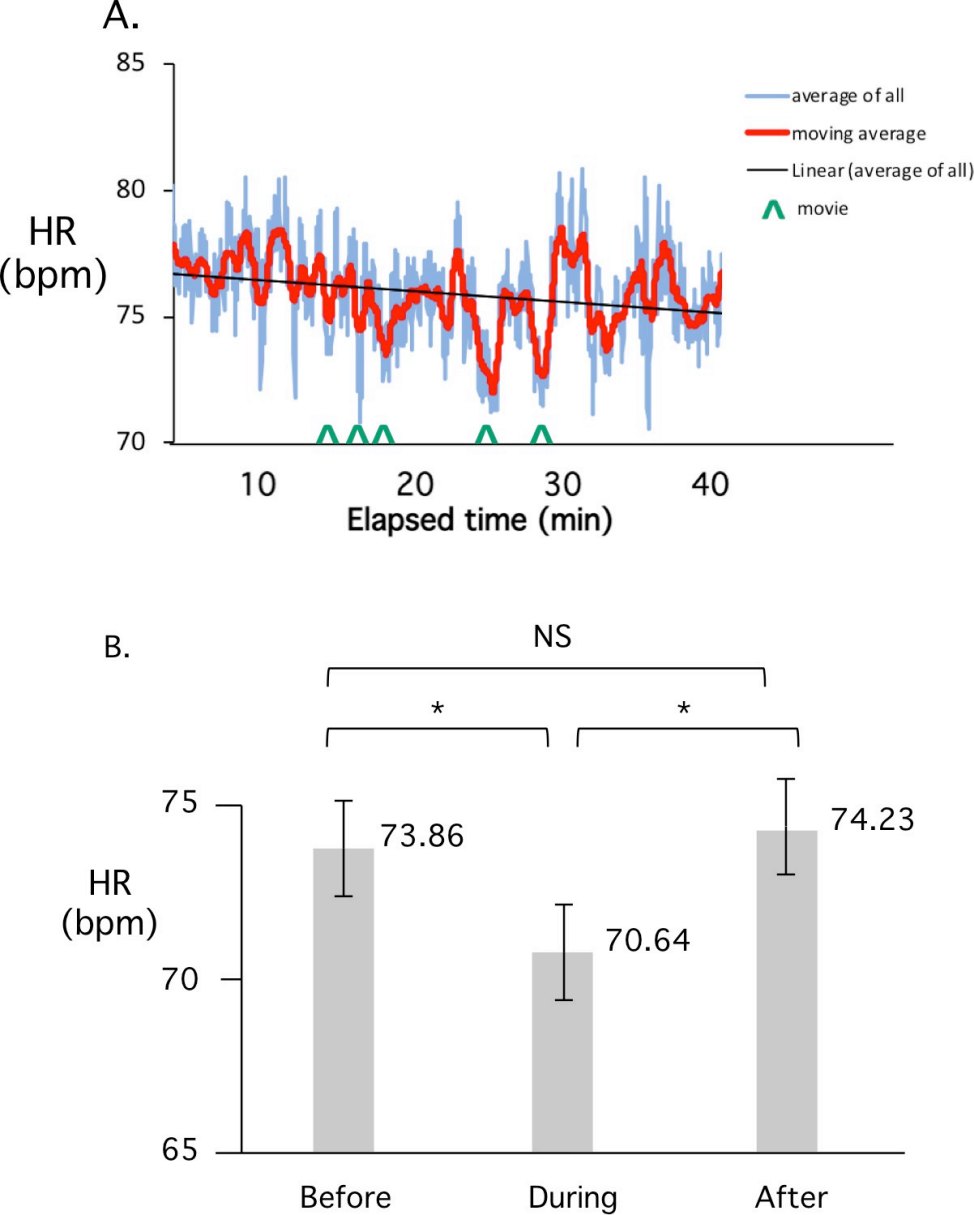
HR decreases during movies and returns to pre-movie levels afterwards. A). Average student HR during a lecture class containing 5 distinct short videos, indicated by vertical arrows. The beginning of each video corresponds to a rapid drop in HR. B). Peak to trough analysis of the HR before, during and after the video presentations. A total of 10 videos were analyzed. The decrease in HR during the movie is statistically significant (Paired T-test, * P < 0.05).

To assess a different type of teaching episode we examined the effect of short videos on average HR. The averaged HR profile of a class containing five short video presentations is illustrated in Fig 5A. We note that HR appears to decrease greatly during the time that each video is being shown and then rapidly resets afterwards. An analysis of HR during a total of 10 different videos, compared to the times immediately preceding and following the videos, is shown in Fig 5B. In this case, whereas an obvious drop in HR occurs during the videos, the rate resets to the preceding level immediately after the video stops.

The original report of HR traces during a lecture class showed a two to three beat increase in average HR in response to an “intervention” by a student [14] and a 20-beat increase for the student being monitored. In our HR studies, most of the classes contain many faculty and student questions. However, if any reliable change in average heart rates occurs in response to questions, it is less obvious in our data than in the profile reported by Bligh and will require additional analysis.

## Discussion

We have used HR as an objective surrogate for assessing student attention during lecture classes. It is important to re-state at this point that HR is only one of several possible physiological measures correlated with attention/engagement and so it will be important for this type of study to be repeated using other physiological parameters. Our results broadly replicate the results of the original studies [14]. As shown in Fig 1B, for a total of 42 classes, average student HR decreased by approximately 3bpm over the course of a 50-minute medical school lecture class. This is very similar to the rate of decrease originally reported by Bligh for an 80-minute class (3.75 vs 3.95 bpm/h).

Previous studies have reported that attention drops rapidly during the first few minutes of a lecture class and then rises again immediately before the end of the class [40–41] and we have confirmed these results using HR (Fig 2). The reasons for these rapid changes are not known, however, it seems likely that the initial drop is due to a simple reduction in physical activity (e.g. sitting down after walking to the lecture room). However, alternative explanations are also possible, for example that the lecture material is less engaging than whatever activity was being pursued up to that point. The increase in HR at the end of the lecture period is more difficult to explain but may be due to anticipation of the end of class.

It is well established that various physiological parameters, including HR, exhibit predictable alterations over the course of a day. These studies show a slight increase in HR during the morning with a broad peak around 10–11 AM [49–50]. Our student data indicate a decrease in starting HR from the earlier to later classes during the morning (Fig 3), a trend that runs counter to the expected circadian rhythm. Again, the reasons for the reduction in HR are not clear but could be due to the time since last food intake, an overall decline in activity, or perhaps task disengagement and mental fatigue as information from multiple classes accumulates [9, 24, 27, 51].

Our study also examined the effect of different in-class activities on average HR. The results show a statistically significant increase in HR during periods of student involvement and interaction, such as paired group discussions involving problem solving (Fig 4B). The average HR rose by 1.5 bpm during these activities and remained at an elevated level throughout the period that the activity continued. The longest activity in any class was only 11 minutes (average length ~5 minutes), so it is possible that HR would have decreased if the activity had continued for longer. The most surprising observation concerning student-involved activities was the immediate and rapid decrease in HR upon cessation of the activity. Not only did the HR decrease rapidly, but on average it declined to below the level it had been before the activity commenced (Fig 4B). If HR is indeed a surrogate measure of engagement, these observations are in contrast to previous studies that suggested attention should reset to close to the original level after an activity [44–45, 52]. Several more recent studies, some using student behavior as a measure of engagement, have also concluded that attention is restarted or reset after a period of active learning, but this is not evident in the 22 events analyzed in our HR data. Furthermore, our findings show a continuing decline in student HR with consecutive lecture periods over the course of a morning, even though the lectures are normally separated by a 5-10-minute break of unstructured time that is typically associated with a rapid rise in HR. We note however, that the lunch break, which is generally an hour of unstructured time including food, appears to result in a partial reset (~70 bpm at 11 am versus ~73bpm at 1 pm; Fig 3).

**Fig 6:**
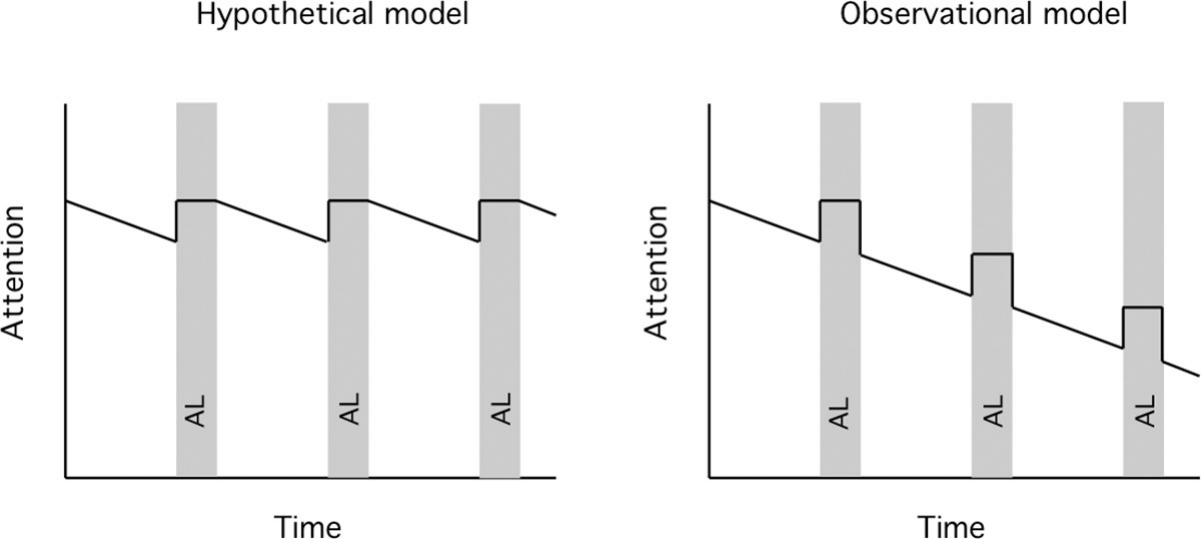
Models for attention reset following periods of active learning. A). The hypothetical model suggested that HR would rise during an interruption or activity within a didactic lecture and attention would then reset close to levels at the beginning of class. B). Based on observations, a revised model suggests that HR does indeed rise during an AL session, but HR and attention do not remain elevated and rapidly return to the level preceding the AL period.

To summarize, we have shown that personal HR monitors are effective tools for measuring the physiological response of students to different classroom activities. According to previous reports in the literature, such changes in HR also correlate with changes in engagement. Our data are the first to confirm the classic observations of Bligh [14], but utilizing larger sample sizes and supported by statistical validation. We find that increases in HR during AL sessions, or decreases in HR during videos, rapidly return to an average HR that steadily declines during the course of a class (modeled in Fig 6B). There is a widely held belief that AL sessions during a lecture presentation, in addition to providing the opportunity to integrate and manipulate new information, also result in a reset in attention [53]. Such a reset would provide the potential for improved attention span and concomitant improvement in retention of new material after the AL session. However, our results provide no evidence to support the expectation that AL interludes, spaced at 10-15-minute intervals, reset engagement and enhance student attention to didactic instruction. In view of the absence of HR reset after an AL session, our results suggest that the value of AL is restricted to the activities contained within that episode and not to an upward reset of student engagement after the active period.

## Supporting information

S1 Dataset Normalized HR data for all classes. (PDF)

S2 Dataset A. Normalized HR data for first 3 minutes of class; B. Normalized HR data for last 3 minutes of class.
(PDF)

S3 Dataset R formatted data for S1 Table ANOVA (and S1 Table attached). (PDF)

S1 Table Statistical output (R) from activities ANOVA (one-way, two-way). (PDF)

S2 Table Statistical comparison of start and endpoints for classes around breaks. (PDF)

## Abbreviations

HR: Heart Rate
AL: Active Learning
