# Supplementary material for: Student engagement, assessed using heart rate, shows no reset following active learning sessions in lectures
Source: PLoS One. 2019 Dec 2;14(12):e0225709. doi: 10.1371/journal.pone.0225709 (PMC6886849; doi:10.1371/journal.pone.0225709)
Supplement: S2 Dataset — A. Normalized HR data for first 3 minutes of class; B. Normalized HR data for last 3 minutes of class. (PDF) [file pone.0225709.s002.pdf]

1st 3 minutes			S2 Dataset A				1st 3 minutes			S2 I
time	91914.17		91914.18		92214.18		92214.2		92314.16	
	10:00-10:50		11:00-11:50		11-11:50		1-2:20		9am	
0:00:01	82.0721129	61	77.8069058	61	78.1395516	63	84.8538805	73	74.6681032	63
0:00:02	80.9471129	61	77.3130787	61	77.9173294	63	84.8538805	73	74.0014366	63
0:00:03	80.5096129	61	77.2329976	61	77.2506627	63	85.2538805	73	73.6681032	63
0:00:04	80.9314879	61	76.8996642	61	77.0284405	63	85.2538805	73	73.6828782	63
0:00:05	80.5096129	61	77.2329976	61	76.6951071	63	85.2538805	73	74.1114496	63
0:00:06	81.0721129	61	77.0107753	61	76.583996	63	85.4538805	73	74.2543067	63
0:00:07	81.9158629	61	77.0107753	61	76.2506627	63	87.024606	73	73.9685925	63
0:00:08	82.4783629	61	76.4552198	61	76.1395516	63	87.024606	73	74.1114496	63
0:00:09	82.0564879	61	75.7885531	61	76.2506627	63	87.024606	73	74.2543067	63
0:00:10	83.3221129	61	75.8996642	61	76.3617738	63	87.4134949	73	74.3971639	63
0:00:11	83.6033629	61	75.1218865	61	75.6951071	63	87.8023838	73	73.6828782	63
0:00:12	83.2908629	61	75.5663309	61	75.583996	63	87.4134949	73	73.6828782	63
0:00:13	81.2263973	61	76.0107753	61	75.583996	63	87.4134949	73	74.6828782	63
0:00:14	82.1346129	61	76.2329976	61	75.1428504	63	87.8023838	73	74.540021	63
0:00:15	82.2752379	61	77.1218865	61	75.3428504	63	88.1912727	73	74.3971639	63
0:00:16	82.1346129	61	77.3441087	61	75.4428504	63	88.1634949	73	74.8257353	63
0:00:17	81.5721129	61	77.5663309	61	75.4428504	63	90.3023838	73	75.2543067	63
0:00:18	81.0252379	61	77.8996642	61	75.8428504	63	96.1634949	73	75.540021	63
0:00:19	80.6971129	61	78.0107753	61	74.7149913	63	97.6912727	73	76.1114496	63
0:00:20	81.0564879	61	77.8996642	61	73.9449913	63	97.7190505	73	76.2543067	63
0:00:21	81.3377379	61	78.0107753	61	73.6459207	63	99.274606	73	75.8257353	63
0:00:22	81.3221129	61	77.8996642	61	74.4849399	63	99.6376194	73	76.1114496	63
0:00:23	80.7596129	61	78.0107753	61	73.3849913	63	100.248731	73	76.1114496	63
0:00:24	80.6189879	61	78.4552198	61	73.8249913	63	101.082064	73	75.8257353	63
0:00:25	80.4783629	61	77.7885531	61	73.7149913	63	97.7562139	73	76.8257353	63
0:00:26	80.6189879	61	76.1218865	61	73.8049913	63	96.5895472	73	77.2543067	63
0:00:27	79.9627379	61	75.677442	61	73.0349913	63	95.8117695	73	76.9685925	63
0:00:28	80.6658629	61	75.5663309	61	72.4749913	63	95.9784361	73	76.8257353	63
0:00:29	80.2439879	61	75.0107753	61	71.7049913	63	96.1451028	73	76.8257353	63
0:00:30	80.2439879	61	75.1218865	61	71.7049913	63	96.0895472	73	76.3971639	63
0:00:31	80.9273281	61	75.1218865	61	73.0428504	63	96.367325	73	76.540021	63
0:00:32	80.6773281	61	76.1218865	61	72.3428504	63	97.0895472	73	76.3971639	63
0:00:33	80.3023281	61	76.3441087	61	72.4428504	63	100.96905	73	76.8257353	63
0:00:34	80.3023281	61	76.3441087	61	72.9428504	63	100.385717	73	77.3971639	63
0:00:35	80.3023281	61	75.8996642	61	73.0428504	63	100.024606	73	76.9685925	63
0:00:36	79.1773281	61	75.0107753	61	72.0428504	63	99.0801616	73	77.2543067	63
0:00:37	79.4273281	61	74.8996642	61	71.5428504	63	99.0801616	73	76.9685925	63
0:00:38	79.3023281	61	74.5663309	61	71.5428504	63	97.1634949	73	76.9685925	63
0:00:39	79.5523281	61	74.5663309	61	72.1428504	63	97.774606	73	77.3971639	63

0:00:40	79.5523281	61	74.5663309	61	72.4428504	63	97.3857171	73	77.9685925	63
0:00:41	79.4273281	61	74.3441087	61	72.8428504	63	97.774606	73	76.9685925	63
0:00:42	78.6773281	61	74.677442	61	72.5428504	63	97.774606	73	77.1114496	63
0:00:43	78.9273281	61	73.677442	61	72.7428504	63	97.774606	73	76.8257353	63
0:00:44	78.9273281	61	73.0107753	61	72.3428504	63	98.1912727	73	77.540021	63
0:00:45	78.9273281	61	73.5663309	61	71.9428504	63	97.8023838	73	77.540021	63
0:00:46	78.8023281	61	73.3441087	61	71.9428504	63	97.8023838	73	78.1114496	63
0:00:47	78.4273281	61	70.216646	61	71.9428504	63	97.4412727	73	78.5735122	63
0:00:48	78.4273281	61	70.1055349	61	71.9428504	63	97.0523838	73	78.3235122	63
0:00:49	78.1773281	61	69.3524485	61	72.3428504	63	96.6634949	73	78.0735122	63
0:00:50	78.0523281	61	69.5993621	61	71.9428504	63	96.274606	73	77.5735122	63
0:00:51	78.8023281	61	69.7228189	61	72.0428504	63	96.0801616	73	76.9485122	63
0:00:52	78.9273281	61	69.8462757	61	71.7428504	63	95.6912727	73	76.8235122	63
0:00:53	79.0523281	61	70.4635596	61	71.4428504	63	95.3301616	73	76.6985122	63
0:00:54	76.9002379	61	72.5663309	61	71.2428504	63	95.7468282	73	76.4485122	63
0:00:55	79.3023281	61	73.7885531	61	71.1428504	63	96.7190505	73	76.9485122	63
0:00:56	79.5523281	61	73.8996642	61	71.0428504	63	97.5523838	73	76.8235122	63
0:00:57	79.8023281	61	74.0107753	61	71.2428504	63	97.5523838	73	77.0735122	63
0:00:58	79.1773281	61	74.677442	61	70.9428504	63	97.5523838	73	77.1985122	63
0:00:59	79.3023281	61	75.2329976	61	71.3428504	63	97.7468282	73	76.6985122	63
0:01:00	79.5523281	61	75.677442	61	71.6428504	63	98.3301616	73	75.9485122	63
0:01:01	79.5523281	61	75.677442	61	71.8428504	63	99.1079393	73	76.0735122	63
0:01:02	79.4273281	61	75.4552198	61	72.0428504	63	98.6912727	73	76.3235122	63
0:01:03	79.0523281	61	75.677442	61	71.8428504	63	99.274606	73	76.6985122	63
0:01:04	79.1773281	61	75.7885531	61	71.9428504	63	99.8579393	73	77.1985122	63
0:01:05	78.5523281	61	75.3441087	61	71.5428504	63	99.274606	73	77.4485122	63
0:01:06	78.8023281	61	75.0107753	61	71.1428504	63	99.274606	73	78.3235122	63
0:01:07	78.9273281	61	75.3441087	61	69.2649913	63	97.7190505	73	78.3235122	63
0:01:08	79.1773281	61	75.2329976	61	69.6049913	63	97.6079393	73	78.6985122	63
0:01:09	79.4273281	61	75.4552198	61	69.4949913	63	95.4690505	73	78.3235122	63
0:01:10	79.5523281	61	75.5663309	61	69.8428504	63	95.3857171	73	75.7575116	63
0:01:11	79.5523281	61	75.677442	61	69.9428504	63	94.1912727	73	75.6168866	63
0:01:12	79.1773281	61	75.0107753	61	69.9428504	63	92.2468282	73	75.6168866	63
0:01:13	79.3023281	61	74.677442	61	70.1428504	63	91.4690505	73	77.1985122	63
0:01:14	78.8023281	61	74.7885531	61	70.2428504	63	90.274606	73	76.8235122	63
0:01:15	80.1773281	61	74.8996642	61	70.2428504	63	89.6912727	73	76.8235122	63
0:01:16	80.1773281	61	74.7885531	61	69.7428504	63	89.6912727	73	76.9485122	63
0:01:17	80.4273281	61	74.5663309	61	69.6428504	63	88.3301616	73	76.9748892	63
0:01:18	80.0523281	61	74.4552198	61	69.4428504	63	88.3301616	73	76.9748892	63
0:01:19	80.0523281	61	73.8996642	61	69.1428504	63	86.9690505	73	75.9748892	63
0:01:20	80.6773281	69	73.2329976	61	69.2428504	63	86.774606	73	75.9748892	63
0:01:21	80.8023281	69	72.7885531	61	69.3428504	63	86.774606	73	76.0860003	63
0:01:22	80.3023281	69	72.0107753	61	69.5428504	63	86.3857171	73	75.9748892	63

0:01:23	80.5523281	69	71.8996642	61	70.0428504	63	86.3857171	73	76.3082225	63
0:01:24	80.8023281	69	71.8996642	61	70.3428504	63	81.524606	73	76.4193336	63
0:01:25	80.6773281	69	71.677442	61	70.4428504	63	81.1079393	73	76.3082225	63
0:01:26	79.8023281	69	71.5663309	61	70.5428504	63	81.4968282	73	76.4193336	63
0:01:27	79.6773281	69	71.3441087	61	70.4428504	63	82.274606	73	76.4193336	63
0:01:28	79.6773281	69	71.8996642	61	70.2428504	63	82.024606	73	76.3082225	63
0:01:29	79.4273281	69	71.8996642	61	70.5428504	63	82.4134949	73	76.0860003	63
0:01:30	79.4273281	69	72.1218865	61	70.4428504	63	83.3301616	73	76.1971114	63
0:01:31	79.5523281	69	72.8996642	61	70.4428504	63	82.524606	73	76.7526669	63
0:01:32	80.1773281	69	73.4552198	61	70.4428504	63	82.7190505	73	76.7526669	63
0:01:33	80.4273281	69	74.1218865	61	70.8428504	63	82.5801616	73	75.9748892	63
0:01:34	79.9273281	69	74.3441087	61	70.6428504	63	82.1912727	73	76.8637781	63
0:01:35	79.6773281	69	72.83393	61	71.4428504	63	82.5801616	73	76.8637781	63
0:01:36	79.3023281	69	72.7351646	61	71.1428504	63	82.4968282	73	76.4193336	63
0:01:37	79.4273281	69	72.3647942	61	70.7428504	63	82.8857171	73	76.3082225	63
0:01:38	78.9273281	69	72.6363991	61	70.5428504	63	83.5523838	73	75.3082225	63
0:01:39	78.0523281	69	72.7722016	61	70.3428504	63	83.6079393	73	75.8637781	63
0:01:40	77.9273281	69	72.6487448	61	70.1428504	63	82.8301616	73	75.9748892	63
0:01:41	77.9273281	69	72.7722016	61	70.1428504	63	82.2468282	73	75.6415558	63
0:01:42	78.3023281	69	73.0191152	61	70.4428504	63	81.8579393	73	75.5304447	63
0:01:43	78.3023281	69	73.3894856	61	70.3428504	63	81.6634949	73	75.4193336	63
0:01:44	78.1773281	69	73.142572	61	70.4428504	63	81.1357171	73	74.9748892	63
0:01:45	78.9273281	69	73.7598559	61	70.6428504	63	81.1357171	73	74.8821932	63
0:01:46	79.1773281	69	73.8833127	61	71.4428504	63	81.524606	73	74.6821932	63
0:01:47	77.5156461	69	73.6363991	61	72.2428504	63	81.9134949	73	74.8821932	63
0:01:48	76.6718961	69	73.7598559	61	72.0428504	63	82.3301616	73	74.9821932	63
0:01:49	76.2500211	69	74.6240535	61	72.2428504	63	82.1357171	73	75.2821932	63
0:01:50	76.2500211	69	74.3524485	61	71.7428504	63	82.524606	73	75.1821932	63
0:01:51	76.1093961	69	74.5993621	61	71.6428504	63	82.9412727	73	75.3821932	63
0:01:52	75.8281461	69	74.83393	61	71.4428504	63	82.2190505	73	75.2821932	63
0:01:53	75.5781461	69	74.9697325	61	71.9428504	63	81.8301616	73	75.9821932	63
0:01:54	75.7343961	69	74.1178806	61	71.8428504	63	81.8579393	73	76.3821932	63
0:01:55	75.1718961	69	72.326533	61	72.0428504	63	81.0801616	73	76.9821932	63
0:01:56	75.0468961	69	74.44165	61	72.4428504	63	80.1079393	73	77.5821932	63
0:01:57	75.0625211	69	74.5897981	61	72.6428504	63	79.1357171	73	78.1821932	63
0:01:58	72.8495261	69	74.7626376	61	73.1428504	63	78.7468282	73	78.5821932	63
0:01:59	72.8495261	69	74.5404154	61	73.0428504	63	78.5523838	73	79.0821932	63
0:02:00	74.7500211	69	74.6885635	61	72.8428504	63	77.774606	73	79.3821932	63
0:02:01	74.7500211	69	74.9478228	61	72.1428504	63	77.3857171	73	79.3821932	63
0:02:02	75.1718961	69	75.0218969	61	71.0428504	63	77.9412727	73	79.0821932	63
0:02:03	75.7343961	69	73.4252984	61	70.3428504	63	77.5523838	69	78.7821932	63
0:02:04	75.5937711	69	75.7749833	61	70.2428504	63	77.4690505	69	78.0821932	63
0:02:05	75.7343961	69	74.1536935	61	70.9428504	63	78.2468282	69	76.6821932	63

0:02:06	73.4120261	69	74.6104836	61	70.6428504	63	78.6079393	69	77.0821932	63
0:02:07	75.3125211	69	76.6638722	61	70.5428504	63	78.6079393	69	76.8821932	63
0:02:08	74.7500211	69	76.478687	61	70.2428504	63	78.2190505	69	77.1821932	63
0:02:09	74.8906461	69	76.2564648	61	70.2428504	63	78.5801616	69	76.4821932	63
0:02:10	75.1562711	69	75.9231314	61	69.4428504	63	78.5523838	69	76.4821932	63
0:02:11	75.4218961	69	74.0055453	61	69.2428504	71	77.774606	69	76.5821932	63
0:02:12	75.8281461	69	76.2070821	61	69.2428504	71	78.1357171	69	76.0821932	63
0:02:13	76.5312711	69	76.170045	61	69.5428504	71	80.6634949	69	75.4821932	63
0:02:14	77.0937711	69	76.0342425	61	70.4428504	71	80.6634949	69	75.8821932	63
0:02:15	77.2500211	69	74.0425824	61	71.0428504	71	80.4690505	69	75.8821932	63
0:02:16	76.5312711	69	73.635175	61	71.9428504	71	82.2190505	69	76.4821932	63
0:02:17	74.7557761	69	72.7709774	61	73.2428504	71	82.2190505	69	76.7821932	63
0:02:18	74.5995261	69	71.8450515	61	73.3428504	71	82.2190505	69	76.4821932	63
0:02:19	74.9120261	69	71.9685083	61	73.3428504	71	82.4412727	69	76.3821932	63
0:02:20	76.9687711	69	72.017891	61	73.2428504	71	83.0523838	69	74.9102608	63
0:02:21	77.6718961	69	72.017891	61	73.5428504	71	84.6079393	69	74.8002608	63
0:02:22	77.6718961	69	71.9561626	61	73.4428504	71	86.9412727	69	74.6902608	63
0:02:23	78.3906461	69	71.6845577	61	73.4428504	71	87.3023838	69	74.4702608	63
0:02:24	77.9531461	69	71.3141873	61	73.1428504	71	87.3023838	69	74.8002608	63
0:02:25	78.2343961	69	71.3882614	61	72.8428504	71	87.8857171	69	76.7821932	63
0:02:26	78.8125211	69	71.3018416	61	72.7428504	71	88.4690505	69	76.7821932	63
0:02:27	79.0937711	69	71.326533	61	72.4428504	71	88.0801616	69	75.9821932	63
0:02:28	79.3750211	69	71.3759157	61	73.5428504	71	87.3023838	69	75.3821932	63
0:02:29	81.9273281	69	72.1907305	61	73.6428504	71	87.8857171	69	75.2821932	63
0:02:30	80.3750211	69	72.4623355	61	74.0428504	71	87.8857171	69	74.9821932	63
0:02:31	82.8023281	69	72.8697429	61	74.2428504	71	88.0801616	69	75.1821932	63
0:02:32	82.6773281	69	73.1043108	61	74.4428504	71	86.7190505	69	74.9821932	63
0:02:33	82.9273281	69	73.3759157	61	73.5428504	71	86.7190505	69	74.9821932	63
0:02:34	82.6773281	69	74.4870268	61	73.0428504	71	86.524606	69	74.1821932	63
0:02:35	81.8023281	69	75.1660392	61	72.6428504	71	85.7468282	69	72.7821932	63
0:02:36	81.8023281	69	76.9685083	61	72.3428504	71	84.7468282	69	72.1821932	63
0:02:37	81.5523281	69	78.0425824	61	71.1428504	71	84.774606	69	71.6821932	63
0:02:38	81.0523281	69	79.4746811	61	70.0428504	71	84.024606	69	73.0821932	63
0:02:39	81.1773281	69	79.5240639	61	70.0428504	71	83.2468282	69	74.6821932	63
0:02:40	80.4273281	69	79.4129528	61	69.9428504	71	83.274606	69	75.5821932	63
0:02:41	80.6773281	69	79.5734466	61	69.6428504	71	83.274606	69	76.1821932	63
0:02:42	80.5523281	69	79.8450515	61	70.2428504	71	83.0523838	69	74.5802608	63
0:02:43	80.4273281	69	79.8697429	61	69.7428504	71	82.274606	69	74.1502608	63
0:02:44	80.3023281	69	79.0796194	61	69.6428504	71	82.6634949	69	74.2602608	63
0:02:45	80.0523281	69	78.943817	61	69.3428504	71	79.9134949	69	74.2602608	63
0:02:46	80.0523281	69	77.5611009	61	68.9428504	71	79.1357171	69	75.1402608	63
0:02:47	79.8023281	69	76.6104836	61	69.1428504	71	79.1357171	69	75.9821932	63
0:02:48	80.3023281	69	76.1783849	61	69.7428504	71	79.274606	69	75.9821932	63

0:02:49	80.4273281	69	75.6598663	61	69.8428504	71	79.2468282	69	75.1821932	63
0:02:50	81.0523281	69	75.4129528	61	69.8428504	63	79.9968282	69	73.7821932	63
0:02:51	81.3023281	69	75.1660392	61	69.3428504	63	80.774606	69	73.3821932	63
0:02:52	81.3023281	69	75.1413478	61	68.7428504	63	80.3857171	69	74.4821932	63
0:02:53	81.1773281	69	75.4129528	61	68.7428504	63	80.774606	69	74.8821932	63
0:02:54	81.0523281	69	75.6598663	61	68.7428504	63	81.1634949	69	75.2821932	63
0:02:55	80.9273281	69	75.6598663	61	69.9428504	63	83.1079393	69	75.2821932	63
0:02:56	80.3023281	69	75.635175	61	70.2428504	63	81.7468282	69	75.0821932	63
0:02:57	81.3023281	69	75.9067799	61	71.1428504	63	81.7468282	69	74.6821932	63
0:02:58	80.9273281	69	75.9931997	61	71.6428504	63	80.9412727	69	74.5821932	63
0:02:59	81.1773281	69	76.4006071	61	72.1428504	63	76.7589485	69	74.4821932	63
0:03:00	80.8023281	69	76.6228293	61	72.0428504	63	75.2589485	69	74.2821932	63

Dataset A				1st 3 minutes		S2 Dataset A				
92314.17		92414.15		92414.16		92414.17		92514.18		92514.2
10am		8.30-9.30		9:30-10:30		10:30-11:35		9-10:15		10:30-12
67.2120799	63	78.493489	61	75.6830694	61	69.3849147	61	79.4139558	60	63.4095318
66.9120799	63	79.1601557	61	75.8830694	63	69.5099147	61	79.595774	60	63.8857223
66.6120799	63	79.493489	61	76.2830694	63	69.6349147	61	78.7775921	60	64.0444525
65.6120799	63	79.8268223	61	76.8830694	63	69.5099147	61	78.5048649	60	63.9492144
65.6120799	63	80.8268223	61	74.7770893	63	70.2599147	61	78.1412285	60	63.9501513
65.5120799	63	81.8268223	61	75.0170893	63	70.3849147	61	78.2321376	60	65.5676978
65.5120799	63	81.1601557	61	75.2570893	63	70.5099147	61	77.8685012	60	66.0676978
65.3120799	63	82.493489	61	78.4830694	63	70.7599147	61	78.0503194	60	65.9426978
65.0120799	63	77.0245939	61	78.8830694	63	70.8849147	61	77.2321376	60	65.3176978
65.0120799	63	76.5801494	61	79.0830694	63	71.5099147	61	76.9594103	60	64.9426978
65.3120799	63	76.135705	61	80.0830694	63	70.7599147	61	77.1412285	60	64.4426978
65.1120799	63	75.3579272	61	80.4830694	63	70.8849147	61	77.5048649	60	64.4426978
65.0120799	63	74.0245939	61	80.6830694	63	68.2599147	61	77.7775921	60	64.1926978
64.8120799	63	74.4690383	61	80.4830694	63	67.7599147	61	78.2321376	60	64.6216908
64.8120799	63	74.3579272	61	80.2830694	63	68.6349147	61	78.5048649	60	64.7328019
65.1120799	63	75.2468161	61	78.6830694	63	68.5099147	61	78.6866831	60	65.0898244
65.5120799	63	76.5801494	61	78.4830694	63	68.0099147	61	78.595774	60	65.0898244
65.6120799	63	77.4690383	61	79.2830694	63	67.2599147	61	78.595774	60	64.9080063
65.7120799	63	77.0245939	61	79.4830694	63	64.6617634	61	78.9594103	60	64.9080063
65.7120799	63	82.8268223	61	79.2830694	63	64.3805134	61	79.0503194	60	64.9080063
65.7120799	63	82.493489	61	79.2830694	63	64.3961384	61	79.1412285	60	65.3625517
66.6120799	63	77.4690383	61	79.2830694	63	64.2555134	61	79.4139558	60	65.2716426
66.6120799	63	77.0245939	61	79.4830694	63	64.5367634	61	79.7775921	60	65.7261881
66.4120799	63	75.5801494	61	78.2830694	63	65.1305134	61	79.6866831	60	66.1807335
66.7120799	63	79.1601557	61	78.0830694	63	64.7242634	61	79.9594103	60	68.9989154
67.0120799	63	79.1601557	61	77.8830694	63	65.5680134	61	80.0503194	60	69.0898244
67.3120799	63	79.1601557	61	77.6830694	63	65.4273884	61	80.595774	60	70.7261881
67.6120799	63	72.7972013	61	77.8830694	63	65.5680134	61	80.8685012	60	71.8170972
67.9120799	63	72.1097013	61	77.8830694	63	66.2711384	61	80.595774	60	71.9989154
68.3120799	63	70.8597013	61	77.6830694	63	66.5523884	61	79.9623816	60	72.5443699
68.8120799	63	70.9847013	61	77.0830694	63	66.9742634	61	81.5048649	61	72.7261881
69.0120799	63	72.1844626	61	76.8830694	63	68.0992634	61	81.4139558	61	72.7261881
68.9120799	63	71.4344626	61	76.4830694	63	68.3805134	61	81.8685012	61	72.9989154
69.5120799	63	71.6844626	61	76.8830694	63	68.3805134	61	81.7775921	61	72.9989154
69.8120799	63	71.9344626	61	76.4830694	63	68.5211384	61	81.1412285	61	73.0898244
69.9120799	63	71.6844626	61	76.0830694	63	68.1148884	61	81.1412285	61	71.7261881
69.4120799	63	71.9344626	61	76.0830694	63	67.4273884	61	81.5048649	61	71.8170972
70.1120799	63	71.6844626	61	76.6830694	63	67.1617634	61	81.595774	61	71.1807335
70.4120799	63	71.1844626	61	77.6830694	63	67.1617634	61	81.9594103	63	71.0898244

70.8120799	63	71.9344626	61	78.0830694	63	66.7398884	61	82.1412285	63	70.7261881
71.2120799	63	67.9847013	61	77.6830694	63	66.8805134	61	81.9594103	63	70.8170972
70.5120799	63	67.6722013	61	77.6830694	63	66.1461384	61	81.6866831	63	71.0898244
70.5120799	63	67.9847013	61	77.8830694	63	66.0211384	61	82.1412285	63	71.0898244
70.6120799	63	67.9847013	61	77.8830694	63	66.1617634	61	82.0503194	63	70.5443699
70.6120799	63	68.0472013	61	78.0830694	63	66.3180134	61	82.2321376	63	70.4534608
70.9120799	63	67.7347013	61	78.4830694	63	66.3180134	61	82.2321376	63	70.5443699
71.1120799	63	66.7972013	61	78.8830694	63	67.0367634	61	82.2321376	63	70.4534608
70.6120799	63	67.1097013	61	80.2830694	63	67.0367634	61	82.9594103	63	69.8170972
69.9632566	63	67.1097013	61	80.6830694	63	67.5992634	61	81.4139558	63	69.5443699
69.5996202	63	67.1097013	61	80.4830694	63	68.7242634	61	80.9594103	63	69.635279
69.1450748	63	66.7972013	61	80.4830694	63	69.4117634	61	81.1412285	63	69.5443699
68.5996202	63	67.6722013	61	80.2830694	63	69.6461384	61	81.2321376	63	68.9989154
68.417802	63	67.9847013	61	79.4830694	63	69.7867634	61	81.0503194	63	69.3625517
67.5087111	63	68.3597013	61	78.8830694	63	69.2242634	61	81.6866831	63	69.2716426
67.1450748	63	68.3597013	61	78.2830694	63	68.2242634	61	81.595774	63	69.635279
66.9632566	63	70.0472013	61	78.4830694	63	67.0523884	61	81.2321376	63	69.635279
66.8723475	63	70.2972013	61	78.8830694	63	66.4898884	61	81.4139558	63	69.3625517
66.7814384	63	71.4847013	61	79.6830694	63	68.6349147	61	81.4139558	63	69.3625517
66.7814384	63	73.0129817	61	79.4830694	63	67.8849147	61	81.3230467	63	69.5443699
65.7814384	63	77.6830694	61	79.0830694	63	68.2599147	61	81.5048649	63	70.0898244
65.5087111	63	78.0830694	61	78.6830694	63	68.1349147	61	81.1412285	63	70.635279
64.9632566	63	78.6830694	61	78.2830694	63	68.1349147	61	80.8685012	63	70.9080063
64.6905293	63	79.0830694	61	76.4830694	63	68.1349147	61	80.6866831	63	70.635279
64.5087111	63	79.6830694	61	74.8830694	63	68.1349147	61	80.4139558	63	70.2716426
65.2359838	63	79.0830694	61	73.8830694	63	68.2599147	61	80.3230467	63	70.0898244
65.1450748	63	78.8830694	61	73.4830694	63	69.2599147	61	80.7775921	63	69.9989154
65.1450748	63	78.4830694	61	73.6830694	63	70.0099147	61	81.1412285	63	69.635279
65.417802	63	77.2830694	61	74.2830694	63	70.2599147	61	81.2321376	63	69.3625517
65.1450748	63	77.4830694	61	74.8830694	63	70.2599147	61	81.6866831	63	69.4534608
65.6905293	63	77.2830694	61	75.0830694	63	70.3849147	61	81.6866831	63	69.9080063
66.6905293	63	76.8830694	61	75.0830694	63	70.2599147	61	81.4139558	63	70.1807335
66.3268929	63	77.0830694	61	75.0830694	63	68.0099147	61	81.595774	63	71.3625517
66.7814384	63	77.6830694	61	75.2830694	63	68.0099147	61	81.3230467	63	71.5443699
67.1450748	63	77.6830694	61	75.4830694	63	67.7599147	61	78.3665685	63	71.2716426
66.7814384	63	77.6830694	61	74.6830694	63	67.8849147	61	78.0773123	63	71.635279
66.417802	63	77.6830694	61	75.6830694	63	67.1349147	61	77.8789652	63	71.7261881
66.0541657	63	77.2830694	61	76.2830694	63	67.0099147	61	78.267395	63	71.4534608
66.6905293	63	77.0830694	61	76.4830694	63	67.0099147	61	80.3230467	63	71.1807335
66.6905293	63	77.4830694	61	76.4830694	63	67.0099147	61	80.6866831	63	71.0898244
66.5996202	63	77.2830694	61	76.6830694	63	66.6349147	61	80.3230467	63	71.0898244
67.1450748	63	77.2830694	61	76.2830694	63	66.8849147	63	79.0690479	63	70.9080063
67.3268929	63	76.8830694	61	76.4830694	63	67.1349147	63	79.0690479	63	70.8170972

67.8723475	63	76.6830694	61	76.0830694	63	67.5099147	63	79.0690479	63	69.9989154	
68.1450748	63	76.8830694	61	76.0830694	63	67.2599147	63	79.6640892	63	69.2716426	
68.5996202	63	77.0830694	61	75.4830694	63	67.0099147	63	80.595774	63	69.1807335	
68.7814384	63	76.4830694	61	75.0830694	63	67.7599147	63	80.2321376	63	68.8170972	
68.8723475	63	76.0830694	61	74.8830694	63	67.8849147	63	80.0503194	63	68.8170972	
68.8723475	63	76.0830694	61	74.8830694	63	68.0099147	63	79.7775921	63	68.635279	
68.8723475	63	75.6830694	61	75.6830694	63	69.2599147	63	79.6866831	63	68.5443699	
68.9632566	63	75.4830694	61	75.4830694	63	69.5099147	63	80.4139558	63	68.0898244	
69.0541657	63	75.4830694	61	75.0830694	63	69.8849147	63	80.9594103	63	68.1807335	
69.0541657	63	75.4830694	61	75.0830694	63	69.7599147	63	80.1412285	63	68.2716426	
68.9632566	63	75.4830694	61	74.6830694	63	69.7599147	63	79.8685012	63	68.0898244	
69.1450748	63	75.4830694	61	75.2830694	63	68.6349147	63	79.5048649	63	67.9989154	
69.2359838	63	75.2830694	61	75.0830694	63	68.5099147	63	79.2321376	63	67.4534608	
69.0541657	63	75.2830694	61	75.0830694	63	65.5444656	63	79.0503194	63	67.7261881	
69.0541657	63	75.6830694	61	75.2830694	63	65.4350906	63	78.9594103	63	68.2716426	
68.7814384	63	75.6830694	61	74.6830694	63	65.4507156	63	79.2321376	63	68.5443699	
68.5996202	63	75.8830694	61	74.2830694	63	65.1694656	63	77.5048649	63	69.9989154	
68.5087111	63	75.8830694	61	73.8830694	63	65.1225906	63	77.595774	63	69.9989154	
69.0541657	63	75.8830694	61	74.0830694	63	65.2632156	63	77.595774	63	69.7261881	
68.7814384	63	75.4830694	61	73.5407084	63	65.1069656	63	77.3230467	63	69.4534608	
68.9632566	63	75.4830694	61	73.8740417	63	68.2599147	63	77.4139558	63	69.0898244	
68.5996202	63	75.4830694	61	74.0407084	63	68.3849147	63	77.3230467	63	69.2716426	
68.5996202	63	75.6830694	61	73.707375	63	68.3849147	63	77.595774	63	70.0898244	
68.7814384	63	76.0830694	61	74.0407084	63	68.8849147	63	77.595774	63	70.3625517	
68.9632566	63	76.0830694	61	74.707375	63	69.2599147	63	77.7775921	71	70.4534608	
69.5996202	63	76.0830694	61	75.5407084	63	69.7599147	63	78.1412285	71	71.635279	
70.1450748	63	75.6830694	61	75.707375	63	70.6349147	63	78.4139558	71	71.9989154	
70.3268929	63	75.6830694	61	75.0407084	63	70.5099147	63	79.595774	71	72.2716426	
70.5087111	63	75.6830694	61	75.5407084	63	69.6349147	63	80.4139558	71	72.9989154	
71.417802	63	75.4830694	61	74.8740417	63	69.3849147	63	79.9594103	71	72.9989154	
71.417802	63	75.4830694	61	75.707375	63	69.7599147	63	79.9594103	71	72.635279	
72.3268929	63	74.8830694	61	75.707375	63	69.7599147	63	79.3230467	71	71.9989154	
72.5996202	63	75.0830694	61	75.707375	63	70.1349147	63	79.1412285	71	72.0898244	
73.5996202	63	74.8830694	61	76.207375	63	70.0099147	63	79.0503194	71	71.2716426	
73.0541657	63	75.6830694	61	75.707375	63	69.8849147	63	79.2321376	71	70.8170972	
72.0541657	63	75.8830694	61	75.3740417	63	69.6349147	63	79.4139558	71	70.8170972	
71.2359838	63	76.2830694	61	75.0407084	63	69.6349147	63	79.7775921	71	69.9989154	
70.8723475	63	76.8830694	61	74.707375	63	70.0099147	63	80.2321376	71	69.8170972	
70.6905293	63	74.7770893	61	74.3740417	63	70.5099147	63	80.2321376	71	69.1807335	
70.2359838	63	75.0170893	61	74.707375	63	70.8849147	63	80.3230467	71	68.9080063	
70.5087111	63	75.2570893	61	74.207375	63	71.5099147	63	80.2321376	71	68.4534608	
70.417802	63	78.4830694	61	73.8740417	63	71.3849147	63	79.8685012	63	68.4534608	
70.417802	63	78.8830694	61	73.8740417	63	70.3849147	63	79.4139558	63	68.3625517	

70.3268929	63	79.0830694	61	73.8740417	63	70.3849147	63	78.6866831	63	67.9989154
70.5087111	63	80.0830694	61	74.3740417	63	69.3849147	63	78.595774	63	68.3625517
70.5087111	63	80.4830694	61	74.3740417	63	68.6349147	63	77.595774	63	68.5443699
70.0541657	63	80.6830694	63	74.207375	63	69.0099147	63	77.595774	63	68.2716426
69.5087111	63	80.4830694	63	73.8740417	63	68.3849147	63	77.6866831	63	68.635279
69.5087111	63	80.2830694	63	74.8740417	63	68.2599147	63	78.2321376	63	68.8170972
69.3268929	63	78.6830694	63	75.8740417	63	68.3849147	63	78.6866831	63	69.4534608
69.6905293	63	78.4830694	63	75.8740417	63	68.8849147	63	79.2321376	63	69.5443699
69.8723475	63	79.2830694	63	76.5407084	63	69.7599147	63	79.1412285	63	69.635279
69.0541657	63	79.4830694	63	76.207375	63	70.1349147	63	78.7775921	63	70.0898244
69.2359838	63	79.2830694	63	76.8656403	63	69.8849147	63	78.5048649	63	70.4534608
69.5996202	63	79.2830694	63	76.7406403	63	70.0099147	63	78.3230467	63	70.635279
69.5996202	63	79.2830694	63	76.9906403	63	69.6349147	63	78.5048649	63	71.2716426
69.7814384	63	79.4830694	63	77.3656403	63	69.2599147	63	78.3230467	63	71.9080063
70.1450748	63	78.2830694	63	77.3656403	63	69.2599147	63	78.2321376	63	71.5443699
70.1450748	63	78.0830694	63	77.6156403	63	70.0099147	63	78.595774	63	71.4534608
70.3268929	63	77.8830694	63	77.4906403	63	70.1349147	63	78.7775921	63	71.4534608
70.5087111	63	77.6830694	63	77.9906403	63	69.7599147	63	78.9594103	63	71.9080063
70.417802	63	77.8830694	63	77.8172418	63	69.6349147	63	79.1412285	63	72.8170972
70.7814384	63	77.8830694	63	77.5950196	63	69.0099147	63	79.3230467	63	73.3625517
70.7814384	63	77.6830694	63	77.7061307	63	69.1349147	63	79.2321376	63	73.2716426
70.7814384	63	77.0830694	63	77.8172418	63	69.1349147	63	79.1412285	63	72.7261881
70.8723475	63	76.8830694	63	77.9283529	63	68.3849147	63	78.1412285	63	72.7261881
71.1450748	63	76.4830694	63	77.7061307	63	67.6349147	63	77.6866831	63	71.9080063
71.2359838	63	76.8830694	63	77.7061307	63	67.2599147	63	77.0503194	63	71.8170972
71.2359838	63	76.4830694	63	77.4839085	63	66.8849147	63	77.0503194	63	70.9080063
70.9632566	63	76.0830694	63	77.7061307	63	66.7599147	63	76.595774	63	70.4534608
70.2359838	63	76.0830694	63	77.8172418	63	67.1349147	63	77.0503194	63	70.2716426
70.0541657	63	76.6830694	63	77.9283529	63	67.3849147	63	77.595774	63	70.4534608
69.7814384	63	77.6830694	63	77.5950196	63	67.1349147	63	77.595774	63	70.3625517
69.5996202	63	78.0830694	63	77.5950196	63	67.1349147	63	77.5048649	63	70.9080063
69.2359838	63	77.6830694	63	77.9283529	63	66.8849147	63	77.9594103	63	71.4534608
69.1450748	63	77.6830694	63	77.8172418	63	67.6349147	63	78.0503194	63	71.5443699
68.7814384	63	77.8830694	63	77.9283529	63	68.0099147	63	78.0503194	63	71.635279
67.9632566	63	77.8830694	63	77.7061307	63	68.1349147	63	77.8685012	63	71.5443699
66.5996202	63	78.0830694	63	77.4839085	63	68.0099147	63	77.8685012	63	71.635279
65.8723475	63	78.4830694	63	77.039464	63	67.6349147	63	77.9594103	63	70.5443699
66.0541657	63	78.8830694	63	76.8172418	63	67.7599147	63	79.1412285	63	69.5443699
65.9632566	63	80.2830694	63	76.5950196	63	67.3849147	63	79.1412285	63	69.8170972
66.3268929	63	80.6830694	63	76.5950196	63	67.6349147	63	79.0503194	63	69.635279
66.6905293	63	80.4830694	63	76.7061307	63	67.8849147	63	78.6866831	63	68.4969562
66.6905293	63	80.4830694	63	75.9283529	63	67.0099147	63	76.9623816	63	68.6939259
67.6905293	63	80.2830694	63	75.8172418	63	66.8849147	63	75.879737	63	70.0898244

68.1450748	63	79.4830694	63	75.8172418	63	66.8849147	63	75.0863485	63	70.2716426
68.5087111	63	78.8830694	63	76.2616862	63	66.8849147	63	74.6896543	63	70.1807335
68.3268929	63	78.2830694	63	76.039464	63	66.8849147	63	74.987175	63	70.0898244
67.6905293	63	78.4830694	63	76.1505751	63	66.7599147	63	74.7970923	63	69.7261881
67.6905293	63	78.8830694	63	76.039464	63	67.0099147	63	75.2929601	63	69.2716426
68.0541657	63	79.6830694	63	75.3727973	63	67.5099147	63	76.1937866	63	69.0898244
68.7814384	63	79.4830694	63	75.039464	63	67.3849147	63	77.3838692	63	68.7261881
68.8723475	63	79.0830694	63	74.9283529	63	67.2599147	63	77.7805634	63	69.1807335
69.5087111	63	78.6830694	63	74.5950196	63	67.0099147	63	78.0780841	63	69.7261881
69.3268929	63	78.2830694	63	74.5950196	63	66.6349147	63	77.7722989	63	70.1807335
69.1450748	63	76.4830694	63	74.5950196	63	66.6349147	63	77.4747783	63	70.5443699
69.3268929	63	74.8830694	63	74.7061307	63	67.2599147	63	76.9623816	63	70.3625517

1st 3 minutes S2 Dataset A 1st 3 minutes

	92614.17		92614.18		92914.17		92914.18		92914.2	
	10-11.		11-12.		9:30-10:46		11-11:50		1-2:00	
63	78.3805438	63	76.6666667	63	77.478866	63	69.3249208	71	72.2863593	63
63	79.0472104	63	76.6666667	63	76.978866	63	69.506739	71	72.2863593	63
63	79.5472104	63	76.7777778	63	76.1455327	63	69.3249208	71	71.9738593	63
63	79.5472104	63	77	63	76.7572916	63	69.5976481	71	71.6613593	63
63	79.7138771	63	77.4444444	63	76.9001488	63	68.8703754	71	71.7238593	63
63	79.5472104	63	78.1111111	63	76.3287202	63	69.0521935	71	71.5988593	63
63	79.5472104	63	78.3333333	63	76.4715773	63	68.3249208	71	72.0988593	63
63	79.8805438	63	78.3333333	63	76.3287202	63	67.8703754	71	72.8488593	63
63	79.7138771	63	79.1111111	63	76.3287202	63	67.8703754	71	72.8488593	63
63	80.0472104	63	79.2222222	63	76.1858631	63	68.1431026	71	72.8488593	63
63	79.3805438	63	79.5555556	63	75.7572916	63	68.0521935	71	73.2238593	63
63	78.8805438	63	80	63	75.6144345	63	69.3249208	71	73.5988593	63
63	78.7138771	63	80.3333333	63	76.7158641	63	69.1431026	71	73.2863593	63
63	78.7138771	63	81.1111111	63	76.9658641	63	68.7794663	71	72.5363593	63
63	80.0472104	63	81.5555556	63	77.2158641	63	69.1431026	71	72.4113593	63
63	80.3805438	63	80.4444444	63	77.0908641	63	68.0521935	71	72.4113593	63
63	80.3805438	63	79.5555556	63	77.5908641	63	67.4158299	71	72.4738593	63
63	80.7138771	63	78.7777778	63	77.7158641	63	66.9612845	71	73.5363593	63
63	81.0472104	63	78.3333333	63	77.8408641	63	66.9612845	71	73.3488593	63
63	80.5472104	63	78.2222222	63	77.2024518	63	66.9612845	71	72.9738593	63
63	80.7138771	63	78.8888889	63	77.2024518	63	68.0521935	71	72.5988593	63
63	80.5472104	63	78	63	76.9802296	63	68.0521935	71	72.2863593	71
63	80.7138771	63	77.2222222	63	77.9802296	63	67.6885572	71	72.2863593	71
63	81.2138771	63	77.2222222	63	78.0913407	63	67.4158299	71	72.2863593	71
63	81.0472104	63	75.5555556	63	77.7580074	63	66.8703754	71	71.4738593	71
63	80.8805438	63	75.1111111	63	77.9802296	63	66.8703754	71	72.0363593	71
63	80.2138771	63	75.6666667	63	77.4246741	63	66.2340117	71	71.6613593	71
63	80.0472104	63	76.2222222	63	77.0913407	63	66.2340117	71	71.4738593	71
63	80.3805438	63	75.8888889	63	76.5357852	63	65.8703754	71	71.2863593	71
63	80.3805438	63	75.1716368	63	76.2024518	63	66.2340117	71	71.3553542	71
63	80.5472104	63	75.5420072	63	76.2024518	63	66.2340117	71	71.5220209	71
63	80.5472104	63	75.7889207	63	76.313563	63	66.2340117	71	72.0220209	71
63	80.2138771	63	76.0358343	63	75.8691185	63	65.9612845	71	72.0220209	71
63	80.0472104	63	75.8012664	63	75.9802296	63	66.2340117	71	72.5220209	71
63	80.0472104	63	75.5543528	63	76.0913407	63	66.1431026	71	72.6886876	71
63	80.0472104	63	75.6901553	63	76.0913407	63	66.4158299	71	73.0220209	71
63	80.3805438	63	76.0605257	63	77.6468963	63	66.7794663	71	73.3553542	71
63	80.5472104	63	75.4308961	63	77.1033341	63	66.7794663	71	73.1886876	71
63	80.7138771	63	75.5543528	63	77.1527168	63	66.8703754	71	72.5220209	71

63	80.0472104	63	75.5555556	63	77.3996304	63	67.1431026	71	71.5220209	71
63	79.8805438	63	76.2222222	63	77.1897538	63	67.9612845	71	70.8553542	71
63	79.2138771	63	76.3333333	63	77.2020995	63	68.7794663	71	70.1886876	71
63	79.0472104	63	76.3333333	63	77.8193834	63	68.9612845	71	70.1886876	71
63	78.7138771	63	76.7777778	63	78.2144452	63	69.0521935	71	69.8553542	71
63	78.8805438	63	76.5555556	63	77.9798773	63	68.2340117	71	69.3553542	71
63	79.0472104	63	76.4444444	63	77.7453094	63	68.3249208	71	69.3553542	71
63	78.7138771	63	75.6666667	63	78.3625933	63	68.2340117	71	69.1886876	71
63	78.5472104	63	74.2222222	63	77.8687662	63	68.2340117	71	68.8553542	71
63	78.0472104	63	73.2222222	63	75.8934575	63	68.5976481	71	68.0220209	71
63	78.0472104	63	73	63	74.5971612	63	68.5976481	71	70.0248486	71
63	77.7138771	63	72.6666667	63	72.6218526	63	68.4158299	71	69.8819915	71
63	78.7138771	63	72.3333333	63	72.6218526	63	68.9612845	71	69.7391343	71
63	79.5472104	63	72	63	72.6218526	63	69.1431026	71	70.5962772	71
63	80.3805438	63	72.1111111	63	72.4983958	63	69.3249208	71	71.8819915	71
63	79.8805438	63	72	63	73.6468963	63	69.4158299	71	71.7391343	71
63	80.5472104	63	71.6666667	63	73.4246741	63	69.506739	71	71.7391343	71
63	81.5472104	63	71.7777778	63	71.7580074	63	69.6885572	71	71.3105629	71
63	82.3805438	63	72	63	70.2024518	63	69.5976481	71	71.4534201	71
63	83.2138771	63	71.8888889	63	69.9802296	63	69.2340117	71	72.1677058	71
63	84.2138771	63	71.7777778	63	70.2024518	63	69.4158299	71	71.8819915	71
63	84.8805438	63	72.1111111	63	69.9802296	63	69.0521935	71	71.5962772	71
63	85.8805438	63	72.2222222	63	70.0913407	63	68.6885572	71	71.3105629	71
63	85.7138771	63	72.3333333	63	69.6468963	63	68.9612845	63	71.4534201	71
63	85.7138771	63	73.4444444	63	69.8691185	63	69.1431026	63	71.0248486	71
63	86.8805438	63	73.8888889	63	69.6468963	63	69.2340117	63	71.0248486	71
63	87.2138771	63	73.8888889	63	69.7580074	63	69.6885572	63	71.0248486	71
63	87.5472104	63	74.5555556	63	69.7580074	63	70.1431026	63	70.4534201	71
63	87.5472104	63	74.1111111	63	70.2024518	63	69.9612845	63	70.4534201	71
63	87.3805438	63	74.2222222	63	70.0913407	63	70.0521935	63	70.1677058	71
63	83.3239293	63	74	63	70.0913407	63	70.506739	63	70.4534201	71
63	84.3805438	63	73.1111111	63	69.8691185	63	70.6885572	63	70.7391343	71
63	84.3805438	63	73.1111111	63	69.8691185	63	70.3249208	63	71.0248486	71
63	84.7138771	63	73	63	69.7580074	63	70.3249208	63	70.8819915	71
63	80.22584	63	72.6666667	63	69.5357852	63	70.8703754	63	71.1677058	71
63	80.22584	63	72.3333333	63	69.313563	63	70.2340117	63	70.8819915	71
63	79.72584	63	71.5555556	63	69.5357852	63	69.7794663	63	71.5962772	63
63	80.22584	63	71.7777778	63	69.7580074	63	69.4158299	63	72.1677058	63
63	79.9202845	63	71.7777778	63	69.8691185	63	69.4158299	63	72.3105629	63
63	79.7536178	63	71.5555556	63	69.6468963	63	68.7794663	63	72.3105629	63
63	79.1702845	63	72.6666667	63	69.0913407	63	66.506739	63	72.8819915	63
63	78.1980623	63	72.3333333	63	68.8691185	63	66.3249208	63	72.7391343	63
63	77.0591734	63	71.6666667	63	68.8691185	63	66.506739	63	71.7391343	63

63	76.6147289	63	71.8888889	63	68.4246741	63	66.7794663	63	70.8819915	63
63	80.0472104	63	71.4444444	63	68.5357852	63	67.5976481	63	70.8819915	63
63	80.2138771	63	72.3333333	63	68.9802296	63	67.9612845	63	71.1677058	63
63	79.7138771	63	72.6666667	63	69.2024518	63	68.2340117	63	70.4534201	63
63	80.0472104	63	72.7777778	63	69.4246741	63	68.506739	63	71.0248486	63
63	80.0472104	63	70.9112983	63	70.6468963	63	68.4158299	63	71.4534201	63
63	79.5472104	63	73.3333333	63	72.4246741	63	68.5976481	63	71.9990238	63
63	79.3805438	63	73.7777778	63	72.4246741	63	68.6885572	63	72.1656905	63
63	79.0472104	63	74.3333333	63	72.9802296	63	68.6885572	63	72.4434682	63
63	79.0472104	63	74.7777778	63	72.4246741	63	68.6885572	63	72.3323571	63
63	78.5472104	63	75	63	73.7580074	63	68.8703754	63	72.3323571	63
63	78.7138771	63	74.5555556	63	74.0913407	63	68.6885572	63	72.0545793	63
63	78.8805438	63	74.8888889	63	74.6468963	63	68.7794663	63	72.6101349	63
63	79.0472104	63	75.5555556	63	74.6468963	63	68.2340117	63	72.7490238	63
63	79.3805438	63	75.2222222	63	74.313563	63	68.3249208	63	73.3045793	63
63	80.7138771	63	75.2222222	63	74.313563	63	68.3249208	63	73.4434682	63
63	80.5472104	63	74.8888889	63	74.7580074	63	69.2340117	63	73.3601349	63
63	80.8805438	63	75	63	74.9802296	63	69.4158299	63	73.3601349	63
63	81.0472104	63	75.2222222	63	74.3338183	63	69.506739	63	71.4961336	63
63	80.5472104	63	74.7777778	63	74.2338183	63	69.6885572	63	71.6183559	63
63	80.5472104	63	74.4444444	63	74.1338183	63	70.1431026	63	71.8628003	63
63	80.5472104	63	74	63	74.6338183	63	70.4158299	63	72.229467	63
63	78.2368411	63	73.1111111	63	74.8338183	63	70.6885572	63	72.329467	63
63	78.0423967	63	72.5555556	63	75.4338183	63	70.9612845	63	72.5739114	63
63	80.3805438	63	72.2222222	63	75.2338183	63	71.2340117	63	72.7739114	63
63	79.7138771	63	73.2222222	63	75.6338183	63	70.7794663	63	72.8961336	63
63	78.8805438	63	74.2222222	63	75.4338183	63	69.7794663	63	72.729467	63
63	78.7138771	63	75.1111111	63	75.0338183	63	70.3249208	63	73.6617012	63
63	77.7138771	63	75	63	74.6338183	63	70.4158299	63	72.2172567	63
63	77.5472104	63	73.6678398	63	74.7338183	63	70.7794663	71	71.8839234	63
63	77.3805438	63	74.5320373	63	74.8338183	63	70.4158299	71	71.7728123	63
63	77.5472104	63	74.5320373	63	74.3338183	63	70.506739	63	71.55059	63
63	74.8672788	63	75.5555556	63	74.0338183	63	70.0521935	63	70.9950345	63
63	76.5472104	63	75.1111111	63	73.5338183	63	69.9612845	63	70.55059	63
63	76.7138771	63	75.3333333	63	73.4338183	63	69.3249208	63	70.55059	63
63	77.3805438	63	75.3333333	63	73.4338183	63	68.9612845	63	70.2172567	63
63	76.2561677	63	76.1111111	63	73.0338183	63	69.3249208	63	70.2172567	63
63	78.7138771	63	75.0258645	63	73.0338183	63	69.1431026	63	70.3283678	63
63	76.0617232	63	75.2727781	63	72.7338183	63	69.5976481	71	70.4394789	63
63	76.0617232	63	76.4444444	63	72.7338183	63	68.7794663	71	70.55059	63
63	77.0472104	63	76.5555556	63	72.6338183	63	68.8703754	71	70.7728123	63
63	76.7138771	63	75.6666667	63	72.6338183	63	69.0521935	71	70.9950345	63
63	77.3805438	63	72.6643847	63	72.7338183	63	68.8703754	71	71.6617012	63

63	77.3805438	63	74	63	73.2338183	63	68.6885572	71	71.6617012	63	
63	77.0472104	63	74	63	73.1338183	63	68.6885572	71	72.2172567	63	
63	76.7138771	63	74	63	73.2338183	63	69.0521935	71	72.3283678	63	
63	76.5472104	63	72.4174711	63	73.9338183	63	69.1431026	71	72.4394789	63	
63	76.7138771	63	72.0471008	63	74.2338183	63	69.1431026	71	72.6617012	63	
63	76.3805438	63	71.923644	63	74.7338183	63	69.1431026	71	73.2172567	63	
63	76.0472104	63	71.923644	63	74.6338183	63	68.4158299	71	73.2172567	63	
63	75.8805438	63	71.4174711	63	74.8338183	63	68.8703754	71	73.4394789	63	
63	75.5472104	63	71.1705576	63	75.5338183	63	69.3333333	71	73.3283678	63	
63	75.5472104	63	71.5409279	63	75.5338183	63	69.75	63	72.9950345	63	
63	76.2138771	63	73	63	75.1338183	63	70.0833333	63	72.8839234	63	
63	76.5472104	63	71.1498156	63	75.1338183	63	70.25	63	72.4394789	63	
63	76.5472104	63	70.2856181	63	75.1338183	63	70.25	63	72.6617012	63	
63	75.8805438	63	70.6559885	63	75.1338183	63	70	63	72.6617012	63	
63	75.7138771	63	72.2222222	63	75.1338183	63	69.5833333	63	72.55059	63	
63	75.7138771	63	72.2222222	63	75.7338183	63	69.1666667	63	72.9950345	63	
63	75.5472104	63	71.7777778	63	75.3338183	63	68.9166667	63	73.2172567	63	
63	75.3805438	63	72.1111111	63	75.1338183	63	68.9166667	63	71.786852	63	
63	75.3805438	63	72.2222222	63	75.0432037	63	68.75	63	72.0312965	63	
63	75.5472104	63	72	63	74.8338183	63	69	63	71.9090742	63	
63	76.0472104	63	71.5555556	63	74.9338183	63	68.5	63	74.3283678	63	
63	76.3805438	63	71.4444444	63	74.9338183	63	68.25	63	73.55059	63	
63	77.2138771	63	71.5555556	63	74.8338183	63	68.0833333	63	73.55059	63	
63	76.8805438	63	71.4444444	63	74.8338183	63	67.9166667	63	73.8839234	63	
63	77.8805438	63	71.2222222	63	75.0338183	63	67.6666667	63	73.7728123	63	
63	78.3805438	63	71.2222222	63	75.6338183	63	67.3333333	63	74.1061456	63	
63	79.2363953	63	71.4444444	63	75.7338183	63	67.4166667	63	74.2172567	63	
63	78.3792524	63	71.2222222	63	75.8338183	63	67.6666667	63	73.9950345	63	
63	78.0935381	63	70.7777778	63	75.7338183	63	67.9166667	63	73.8839234	63	
63	76.2363953	63	70	63	75.8338183	63	67.9166667	63	73.3283678	63	
63	76.0935381	63	69.7777778	63	75.8338183	63	68.1666667	63	74.1061456	63	
63	76.0935381	63	69.8888889	63	75.4338183	63	68.4166667	63	74.7728123	63	
63	75.950681	63	69.8888889	63	75.3338183	63	67.2689259	63	74.55059	63	
63	76.8078238	63	70.2222222	63	75.6338183	63	68.5416532	63	72.9933826	63	
63	76.950681	63	70.4444444	63	76.4338183	63	69.1780168	63	73.4489382	63	
63	77.5221096	63	70.3333333	63	76.1338183	63	69.6325623	63	73.7378271	63	
63	77.3792524	63	70.3333333	63	75.0338183	63	69.9052895	63	73.9044937	63	
63	77.5221096	63	70.7777778	63	74.8338183	63	69.7234714	63	74.2711604	63	
63	77.5221096	63	70.6666667	63	75.0338183	63	69.7234714	63	74.5378271	63	
63	77.8078238	63	70.5555556	63	75.1338183	63	69.7234714	63	75.5156048	63	
63	77.6649667	63	70.1111111	63	75.3338183	63	71.4166667	63	75.1489382	63	
63	77.6649667	63	69.5555556	63	75.6338183	63	71.25	63	74.6378271	63	
63	78.0935381	63	69.8888889	63	75.7338183	63	71.4166667	63	74.8822715	63	

63	78.3792524	63	70.6666667	63	76.5338183	63	71.4166667	63	74.8822715	63
63	79.0935381	63	70.5555556	63	77.1338183	63	71.3333333	63	74.5156048	63
63	78.950681	63	71.3333333	63	77.3338183	63	72.25	63	74.1489382	63
63	79.6649667	63	71.6666667	63	77.3338183	63	71.75	63	74.2489382	63
63	80.3792524	63	72.4444444	63	77.7338183	63	71.3333333	63	73.7600493	63
63	79.3792524	63	72.7777778	63	77.6338183	63	71.3333333	63	74.1267159	63
63	79.0935381	63	73.6666667	63	77.7338183	63	71.3333333	63	74.1044937	63
63	79.0935381	63	73.5555556	63	77.3338183	63	70.9166667	63	72.8822715	63
63	79.6649667	63	73.6666667	63	76.6338183	63	71.1666667	63	73.0044937	63
63	79.0935381	63	74.7777778	63	76.0338183	63	71.0833333	63	73.2489382	63
63	79.0935381	63	75.6666667	63	75.5338183	63	71.1666667	63	73.7489382	63
63	80.2363953	63	76.1111111	63	75.0338183	63	69.0439894	63	73.5044937	63

		S2 Dataset A				1st 3 minutes					S2 Dataset A
100114		100114		100314		100314		101314		101314	
11:00		10:00		10-11.		11-12.		9-10.		10-11.	
71.0445582	63	69.4502952	61	77.8882694	71	74.9643873	61	73.180476	63	65.5811285	
70.89641	63	69.4502952	61	77.2216027	71	75.5754984	61	72.9860315	63	65.7239857	
71.1433236	63	69.6419618	61	79.554936	71	75.5754984	61	72.6249204	63	65.8668428	
71.0445582	63	69.3169618	63	77.8882694	71	75.5643873	61	73.2082538	63	66.1525571	
71.0692495	63	68.6669618	63	77.8882694	71	75.2088317	61	73.5971427	63	66.2954143	
70.450997	63	68.2086285	63	77.8882694	71	76.4963334	61	73.680476	63	65.5811285	
70.41396	63	69.7106527	63	78.2216027	71	76.4963334	61	74.2638093	63	64.8668428	
70.8213674	63	69.7106527	63	79.2216027	71	76.6074445	61	74.0693649	63	64.2954143	
72.9257483	63	69.7106527	63	79.8882694	71	76.2741111	61	74.2638093	63	64.2954143	
73.1726619	63	69.6106527	63	81.2216027	71	76.7185556	61	74.4026982	63	64.7239857	
74.666489	63	69.4106527	63	80.554936	71	77.2741111	61	74.9860315	63	64.8668428	
74.666489	63	69.2106527	63	80.8882694	71	77.3852223	61	75.3749204	63	64.8668428	
73.9257483	63	68.8106527	63	80.8882694	71	78.0518889	61	76.1526982	63	65.1525571	
74.0492051	63	68.8106527	63	81.2216027	71	78.0518889	61	76.3471427	63	64.8668428	
74.1726619	63	68.7106527	63	81.2216027	71	77.9407778	61	76.5415871	63	64.5811285	
73.5553779	63	68.2106527	63	81.554936	71	77.7185556	61	76.5415871	63	64.8668428	
73.4566125	63	68.5106527	63	81.8882694	71	77.3852223	61	76.7360315	63	65.1525571	
73.5800693	63	68.6106527	63	82.8882694	71	77.163	61	76.5415871	63	65.4382714	
73.4813038	63	69.1106527	63	83.8882694	63	77.3852223	61	76.5415871	63	66.0097	
73.4813038	63	69.4106527	63	84.8882694	63	77.7185556	61	76.4582538	63	67.2954143	
73.2096989	63	69.4106527	63	85.554936	63	77.8296667	61	76.6526982	63	68.1525571	
73.2096989	63	70.1106527	63	85.2216027	63	77.3852223	61	76.6526982	63	68.8668428	
72.841176	63	70.1636822	63	85.554936	63	77.2741111	61	76.4582538	63	69.2954143	
73.5730874	63	70.3641494	63	84.554936	63	76.6799866	61	76.4582538	63	69.2954143	
73.5553779	63	69.8586285	63	82.8882694	63	76.9799866	61	75.2915871	63	69.1525571	
73.6788347	63	70.0169618	63	82.2216027	63	77.0799866	61	74.5744972	63	69.5811285	
73.2961186	63	69.6586285	63	81.2216027	63	77.1799866	61	76.2915871	63	67.634602	
72.8022915	63	69.5502952	63	81.2216027	63	77.4799866	61	76.2915871	63	67.3080714	
72.9134026	63	70.2002952	63	80.8507433	63	77.5799866	61	76.680476	63	67.1448061	
72.4195754	63	70.1419618	63	81.4757433	63	77.7799866	63	75.9026982	63	69.7239857	
72.2961186	63	70.0836285	63	81.4757433	63	78.0799866	63	73.3397988	63	69.5811285	
72.2961186	63	70.0836285	63	81.4132433	63	77.6799866	63	73.3397988	63	69.4382714	
71.7899458	63	70.4086285	63	81.7257433	63	77.1799866	63	73.0064654	63	69.5811285	
70.6911804	63	70.2106527	63	80.7882433	63	76.0799866	63	72.9509099	63	69.7239857	
72.2600502	63	70.0106527	63	80.4757433	63	75.3799866	63	73.2286876	63	70.5811285	
69.8949375	63	70.0106527	63	80.4132433	63	75.3799866	63	72.8397988	63	70.7239857	
71.7044947	63	70.2106527	63	80.7257433	63	75.4799866	63	73.3397988	63	70.7239857	
71.5933835	63	69.5106527	63	80.1007433	63	73.8012351	63	73.0064654	63	70.5811285	
71.1489391	63	69.9106527	63	79.7882433	63	73.5912351	63	72.1175765	63	70.4382714	

70.9267169	63	70.1106527	63	78.7882433	63	73.4912351	63	70.9252485	63	70.7239857
70.2600502	63	70.3106527	63	75.9468463	63	73.7112351	63	70.480804	63	70.4382714
68.9267169	63	70.3106527	63	75.9468463	63	73.7112351	63	69.5085818	63	70.7239857
68.9267169	63	69.8106527	63	75.6968463	63	73.4912351	63	69.0085818	63	71.1525571
69.1489391	63	69.6106527	63	75.9468463	63	73.2712351	63	68.8419151	63	71.0097
68.4822724	63	69.5106527	63	76.4468463	63	73.4912351	63	68.8419151	63	71.0097
68.5933835	63	69.1106527	63	74.9468463	63	72.4512351	63	68.8419151	63	70.8668428
68.3711613	63	68.7106527	63	74.9468463	63	73.3799866	63	68.8419151	63	70.5811285
68.037828	63	68.1106527	63	74.1968463	63	73.1799866	63	68.8419151	63	70.5811285
68.4822724	63	67.9106527	63	73.9468463	63	73.3799866	63	68.6474707	63	70.5811285
69.2600502	63	67.9106527	63	74.1968463	63	74.1799866	63	68.8419151	63	70.8668428
69.037828	63	67.8106527	63	74.4468463	63	74.4799866	63	69.0363596	63	71.2954143
69.2600502	63	67.4106527	63	76.6968463	63	74.4799866	63	68.6474707	63	71.2954143
69.5933835	63	67.2106527	63	76.6968463	63	75.4799866	63	69.0363596	63	71.5811285
69.5933835	63	66.9106527	63	78.4468463	63	75.5799866	63	73.5437035	63	70.5811285
70.1489391	63	66.6106527	63	78.4468463	63	75.9799866	63	73.7103701	63	70.1525571
71.8925569	63	66.7106527	63	82.4150475	63	76.3799866	63	75.7103701	63	68.1525571
72.0925569	63	66.5106527	63	81.8150475	63	76.3799866	63	75.8770368	63	67.7239857
71.7862202	63	66.8106527	63	81.8150475	63	76.3799866	63	75.3770368	63	66.7239857
71.5134929	63	67.3106527	63	81.8150475	63	76.0799866	63	76.3770368	63	65.5811285
71.604402	63	67.8106527	63	82.0150475	63	76.6799866	63	76.2103701	63	65.2954143
70.8771293	63	68.0106527	63	82.4150475	63	76.4799866	63	76.2103701	63	65.2954143
70.3316747	63	68.1106527	63	82.0150475	63	75.9799866	63	76.5437035	63	65.4382714
69.6953111	63	67.7106527	63	82.0150475	63	75.9799866	63	76.0437035	63	65.8668428
69.0589474	63	67.8106527	63	83.4150475	63	76.2799866	63	75.3770368	63	66.4382714
68.0589474	63	68.2106527	63	83.8150475	63	76.2799866	63	75.3770368	63	67.1525571
67.7862202	63	68.6106527	63	84.2150475	63	75.5799866	63	73.3770368	63	67.4382714
67.8771293	63	69.5106527	63	84.4150475	63	75.3799866	63	72.5437035	63	67.2954143
67.8771293	63	69.5106527	63	84.8150475	63	75.3799866	63	72.2103701	63	67.4382714
67.7862202	63	70.4106527	63	83.8150475	63	74.1799866	63	72.2103701	63	67.0097
67.1498565	63	70.4106527	63	84.8150475	63	74.6799866	63	72.0437035	63	66.7239857
66.9680384	63	71.3106527	63	86.0150475	63	75.0799866	63	71.2103701	63	67.0097
67.1498565	63	71.6106527	63	86.4150475	63	75.2799866	63	71.0437035	63	66.4382714
67.1498565	63	71.8106527	63	86.6150475	63	75.5799866	63	70.8770368	63	66.8668428
66.9680384	63	71.5106527	63	87.8150475	63	75.8799866	63	71.3770368	63	66.8668428
67.0589474	63	71.4106527	63	89.0150475	63	75.7799866	63	71.7103701	63	67.0097
66.9680384	63	70.7106527	63	89.6150475	63	75.9799866	63	71.7103701	63	67.0097
66.9680384	63	70.7324646	63	89.0150475	63	74.3086798	63	71.5437035	63	67.2954143
67.1498565	63	68.9245416	63	88.6150475	63	74.0786798	63	71.2103701	63	68.1525571
67.4225838	63	68.4106527	63	86.6150475	63	75.3799866	63	71.0437035	63	68.7239857
67.0589474	63	67.8106527	63	85.8150475	63	73.6901349	63	68.3744972	63	69.0097
66.9680384	63	67.7106527	63	86.0150475	63	73.8001349	63	70.7103701	63	68.7239857
67.0589474	63	67.1106527	63	86.0150475	63	73.5801349	63	71.3770368	63	66.6690679

67.604402	63	67.0106527	63	86.8150475	63	73.2501349	63	72.2103701	63	69.5811285
67.7862202	63	67.6106527	63	85.2150475	63	73.4601349	63	72.3770368	63	67.2404964
68.0589474	63	67.6106527	63	84.8150475	63	75.0799866	63	72.8770368	63	66.4649862
67.6953111	63	67.8106527	63	84.2150475	63	75.1799866	63	72.7103701	63	66.3425372
67.5134929	63	68.0106527	63	83.6150475	63	74.8799866	63	72.8770368	63	67.1996801
67.4225838	63	68.3106527	63	83.0150475	63	74.7799866	63	72.2103701	63	67.0772311
68.2407656	63	67.4754728	63	82.2150475	63	75.1799866	63	72.0437035	63	67.567027
68.604402	63	67.7421395	63	82.4150475	63	75.1799866	63	71.7103701	63	67.5874352
68.8771293	63	67.7421395	63	82.2150475	63	75.4799866	63	68.9026982	63	67.4853944
69.1498565	63	68.2838061	63	82.2150475	63	75.5799866	63	74.5437035	63	67.3629454
68.604402	63	68.5754728	63	82.2150475	63	75.6799866	63	71.6196929	63	66.8527413
67.0589474	63	68.9504728	63	81.6150475	63	75.8799866	63	71.480804	63	68.8668428
66.6953111	63	69.1671395	63	80.6150475	63	75.9799866	63	72.4530262	63	67.7239857
67.0589474	63	69.6004728	63	80.8150475	63	75.4799866	63	76.2103701	63	66.5811285
66.7862202	63	69.8171395	63	81.4150475	63	75.0799866	63	76.7103701	63	65.5811285
67.2407656	63	70.7921395	63	81.8150475	63	75.3799866	63	77.7103701	63	65.1525571
67.2407656	63	71.4421395	63	81.6150475	63	75.4799866	63	77.7103701	63	65.1525571
66.7862202	63	72.9106527	63	82.0150475	63	75.2799866	63	77.8770368	63	66.5811285
66.3316747	63	73.4106527	63	82.2150475	63	75.2799866	63	76.9717932	63	67.2954143
65.4225838	63	73.5106527	63	81.4447152	63	75.2799866	63	77.2575075	63	67.2954143
65.1498565	63	72.8106527	63	81.2780485	63	74.6799866	63	76.828936	63	67.0097
65.3316747	63	72.2106527	63	81.2780485	63	74.6799866	63	77.1146503	63	66.8668428
65.2407656	63	71.8106527	63	81.2780485	63	74.9799866	63	76.828936	63	67.0097
64.8771293	63	71.4106527	63	81.7780485	63	75.1799866	63	75.2575075	63	66.2954143
64.604402	63	71.0106527	63	81.7780485	63	75.0799866	63	71.6263729	63	66.8668428
64.8771293	63	70.7106527	63	81.7780485	63	75.4799866	63	71.136577	63	66.4382714
65.2407656	63	71.1106527	63	81.2780485	63	75.2799866	63	71.2998423	63	66.0097
66.0589474	63	71.3106527	63	80.9447152	63	75.2799866	63	70.4835158	63	65.8668428
66.3316747	63	71.2106527	63	80.6113819	63	75.6799866	63	70.3202505	63	65.1525571
66.0589474	63	71.2106527	63	80.4447152	63	75.4799866	63	70.4835158	63	64.8668428
66.5134929	63	70.7106527	63	80.9447152	63	75.0799866	63	70.8304545	63	65.2954143
66.604402	63	69.8848952	63	80.9447152	63	75.2799866	63	70.1773933	63	65.5811285
66.6953111	63	69.8015618	63	81.6551506	63	75.4799866	63	70.3406586	63	66.4382714
65.9680384	63	70.1265618	63	82.2265792	63	75.9799866	63	68.6875974	63	66.8668428
65.8771293	63	71.0106527	63	82.0837221	63	76.3799866	63	68.8508627	63	66.7239857
65.4225838	63	71.2106527	63	81.9408649	63	77.1799866	63	69.014128	63	66.5811285
65.3316747	63	70.6106527	63	81.3694363	63	77.4799866	63	69.5039239	63	67.4382714
65.1498565	63	70.1106527	63	81.6551506	63	77.6799866	63	69.6875974	63	67.5811285
65.2407656	63	69.3106527	63	81.6551506	63	77.9799866	63	69.8508627	63	67.7239857
65.5134929	63	69.3106527	63	81.6551506	63	78.3799866	63	70.6875974	63	68.4382714
65.1498565	63	68.9106527	63	82.0837221	63	76.7799866	63	71.1773933	63	68.5811285
65.2407656	63	68.8106527	63	81.5122935	63	76.0799866	63	71.014128	63	68.2954143
65.1498565	63	68.7106527	63	81.5122935	63	75.1799866	63	70.5243321	63	68.4382714

64.7862202	63	68.5106527	63	81.7980078	63	74.9799866	63	70.7080056	63	67.2954143
64.6953111	63	68.1106527	63	81.6551506	63	75.0799866	63	70.0549443	63	67.7239857
64.604402	63	67.7106527	63	81.2265792	63	74.7799866	63	69.5651484	63	67.5811285
64.5134929	63	67.6106527	63	81.0837221	63	74.8799866	63	69.5651484	63	67.5811285
64.3316747	63	66.8106527	63	80.6551506	63	74.8799866	63	69.2386178	63	67.8668428
64.3316747	63	66.8271593	63	80.5122935	63	74.8799866	63	69.0753525	63	68.2954143
64.0589474	63	65.8106527	63	79.5122935	63	76.4283771	63	68.9120872	63	68.8668428
63.8771293	63	65.9106527	63	79.2265792	63	76.6101953	63	68.9120872	63	68.5811285
63.8771293	63	65.9106527	63	78.5122935	63	77.2465589	63	69.1466133	63	68.4382714
63.7862202	63	66.6106527	63	78.5122935	63	77.337468	63	69.0168386	63	68.2954143
63.6953111	63	67.0106527	63	78.5122935	63	77.2465589	63	68.7488219	63	68.2954143
63.9680384	63	67.2106527	63	78.5122935	63	77.337468	63	68.7284137	63	65.4382714
64.0589474	63	67.3106527	63	77.7980078	63	77.7011044	63	68.891679	63	64.8668428
63.9680384	63	67.6106527	63	77.7980078	63	77.4283771	63	69.0345362	63	64.5811285
63.7862202	63	68.5106527	63	78.3694363	63	76.8829225	63	69.0345362	63	65.0097
63.8771293	63	68.4106527	63	78.0837221	63	76.8829225	63	69.2794341	63	65.0097
66.1498565	63	67.7106527	63	78.0837221	63	76.7920134	63	68.7896382	63	65.1525571
66.4225838	63	67.7106527	63	77.6551506	63	76.6101953	63	70.828936	63	64.8668428
66.604402	63	68.8106527	63	78.3694363	63	76.6101953	63	71.2575075	63	64.5811285
66.5134929	63	69.0106527	63	78.6551506	63	76.4283771	63	71.6860789	63	63.7239857
66.4225838	63	69.2106527	63	79.7980078	63	75.9738316	63	71.6860789	63	64.0097
66.8771293	63	69.4106527	63	79.6551506	63	75.8829225	63	71.1146503	63	64.1525571
66.8771293	63	69.2106527	63	79.5122935	63	75.9738316	63	71.2575075	63	64.1525571
67.0589474	63	69.1106527	63	77.0749688	63	75.1556498	63	68.9733117	63	64.2954143
67.0589474	63	69.1106527	63	76.9117035	63	74.9738316	63	69.7896382	63	64.7239857
66.6953111	63	69.0106527	63	75.7892545	63	75.2465589	63	70.136577	63	65.2954143
66.4225838	63	69.0106527	63	75.7892545	63	75.1556498	63	70.2998423	63	65.4382714
66.6953111	63	68.7106527	63	76.1157851	63	75.2465589	63	70.136577	63	65.4382714
66.3316747	63	68.0106527	63	76.2790504	63	75.5192862	63	70.136577	63	65.2954143
66.0589474	63	68.2106527	63	76.4423157	63	75.337468	63	69.9733117	63	65.7239857
66.2407656	63	68.5106527	63	76.2790504	63	75.1556498	63	69.6059647	63	66.0097
66.2407656	63	68.4106527	63	76.605581	63	75.4283771	63	72.1146503	63	66.2954143
65.9680384	63	68.3106527	63	76.605581	63	75.337468	63	73.1146503	63	66.5811285
64.6123737	63	68.3106527	63	76.605581	63	75.7920134	63	73.9717932	63	67.0097
64.4222911	63	68.3106527	63	80.6551506	63	75.6101953	63	74.828936	63	67.4382714
64.6123737	63	68.6106527	63	81.2265792	63	75.6101953	63	75.9717932	63	68.0097
65.8771293	63	69.4106527	63	81.7980078	63	75.7011044	63	74.828936	63	68.4382714
65.604402	63	69.8106527	63	82.0837221	63	75.7920134	63	75.4003646	63	67.8668428
65.6953111	63	69.7106527	63	82.5122935	63	76.2465589	63	75.2575075	63	67.7239857
65.6953111	63	69.8106527	63	82.9408649	63	76.7920134	63	75.1146503	63	67.7239857
65.3316747	63	69.7106527	63	83.2265792	63	76.6101953	63	74.6860789	63	67.5811285
65.1498565	63	69.3106527	63	83.2265792	63	76.9738316	63	74.9717932	63	67.0097
65.0589474	63	69.0106527	63	82.9408649	63	76.8829225	63	74.6860789	63	67.0097

64.7862202	63	69.1106527	63	82.6551506	63	76.6101953	63	74.4003646	63	67.0097
64.8771293	63	69.1106527	63	81.7980078	63	76.7011044	63	74.2575075	63	67.2954143
64.9680384	63	69.4106527	63	81.6551506	63	76.5192862	63	74.6860789	63	67.5811285
64.7862202	63	69.1106527	63	81.5122935	63	76.5192862	63	74.4003646	63	67.0097
65.1498565	63	69.0106527	63	82.2265792	63	76.8829225	63	70.9717932	63	66.8668428
65.2407656	63	68.4106527	63	82.9408649	63	76.337468	63	70.9717932	63	66.7239857
65.3316747	63	68.4106527	63	83.5122935	63	76.337468	63	71.4003646	63	66.7239857
65.6953111	63	69.0106527	63	84.0837221	63	76.1556498	63	72.4003646	63	66.2954143
65.8771293	63	69.3106527	63	84.9408649	63	76.337468	63	72.4003646	63	66.0097
65.6953111	63	69.9106527	63	84.7980078	63	76.6101953	63	69.6059647	63	66.0097
65.8771293	63	70.0106527	63	84.0837221	63	76.5192862	63	71.5432218	63	66.0097
66.0589474	63	70.2106527	63	81.5851729	63	76.6101953	63	71.2575075	63	66.1525571

1st 3 minutes S2 Dataset A

	101514		101614		101714		102014		102014	
	9-1030.		9-10.		9-1030.		1:00		10-11.	
63	79.2039708	61	67.034831	63	78.3861474	61	72.2627068	63	69.34662	61
63	80.8706374	61	67.034831	63	78.1861474	61	72.4293735	63	69.2474465	61
63	80.8706374	61	67.034831	63	78.5861474	61	72.4293735	63	69.7433142	61
63	78.5373041	61	68.534831	63	79.1861474	61	71.9293735	63	70.0490994	61
63	77.0928597	61	68.534831	63	79.3861474	61	71.7627068	63	70.8424878	63
63	77.0928597	61	69.034831	63	79.7861474	61	71.5960402	63	72.4198147	63
63	76.6484152	61	69.034831	63	79.7861474	61	72.7627068	63	71.6016329	63
63	75.8706374	61	70.034831	63	79.1861474	61	73.0960402	63	69.8424878	63
63	76.3150819	61	70.034831	63	77.9861474	61	73.9293735	63	70.692542	63
63	76.7595263	61	70.534831	63	76.9861474	61	75.7627068	63	69.7834511	63
63	81.2532676	61	70.034831	63	75.9861474	61	77.0960402	63	69.7834511	63
63	81.5866009	61	70.034831	63	76.7861474	61	78.5960402	63	69.4198147	63
63	75.7595263	61	70.534831	63	78.7861474	61	78.9293735	63	68.9652692	63
63	78.9199342	61	70.534831	63	78.7861474	61	79.0960402	63	68.692542	63
63	77.5866009	61	71.034831	63	79.1861474	61	78.9293735	63	66.1883722	63
63	79.2532676	61	71.534831	63	80.3861474	61	79.5960402	63	66.1222565	63
63	79.5866009	61	71.034831	63	80.3861474	61	79.7627068	63	66.1387855	63
63	79.5866009	61	71.034831	63	80.3861474	61	79.9293735	63	65.8577937	63
63	81.9199342	61	71.034831	63	80.5861474	61	77.9293735	63	65.9569673	63
63	82.5866009	61	71.534831	63	80.5861474	61	78.0960402	63	65.6594466	63
63	82.9199342	61	72.534831	63	79.9861474	61	75.0321146	63	65.5602731	63
63	82.5866009	61	74.534831	63	79.9861474	61	73.6710035	63	66.3619259	63
63	82.5866009	61	75.034831	63	79.5861474	61	73.8654479	63	65.9734962	63
63	82.5866009	61	75.034831	63	78.9861474	61	73.8654479	63	66.5602731	63
63	82.5866009	61	73.034831	63	76.7861474	61	74.1987812	63	66.6677111	63
63	82.5866009	61	72.034831	63	75.7861474	61	77.5960402	63	66.1718433	63
63	79.8911918	61	72.034831	63	75.9861474	61	77.5960402	63	65.0242685	63
63	80.1411918	61	71.034831	63	75.9861474	61	78.4293735	63	65.1317065	63
63	80.3911918	61	70.534831	63	75.9861474	61	78.9293735	63	64.9168305	63
63	80.6411918	61	70.534831	63	76.3861474	61	78.5960402	63	64.9912106	63
63	80.8911918	61	70.534831	63	76.5861474	61	78.4293735	63	64.8507148	63
63	80.6411918	61	70.534831	63	76.9861474	61	78.7627068	63	64.8341858	63
63	79.1411918	61	70.534831	63	77.1861474	61	77.7627068	63	64.7102189	63
63	78.6411918	61	70.034831	63	76.7861474	61	77.4293735	63	64.5118718	63
63	79.1411918	61	69.034831	63	76.9861474	61	74.1588015	63	64.4953429	63
63	79.8911918	61	68.034831	63	76.5861474	61	73.5754681	63	64.2930848	63
63	81.1411918	61	68.034831	63	76.5861474	61	72.9921348	63	65.6456889	63
63	81.3911918	61	67.534831	63	76.5861474	61	72.7976904	63	65.6441407	63
63	77.448595	61	67.534831	63	77.3861474	61	72.9921348	63	67.0561783	63

63	81.8911918	61	67.534831	63	77.9861474	61	72.9921348	63	67.1470874	63
63	81.8911918	61	67.034831	63	77.9861474	61	73.7699126	63	67.2379965	63
63	82.8911918	61	67.534831	63	77.9861474	61	74.3532459	63	67.3289056	63
63	82.8911918	61	67.534831	63	77.9861474	61	74.1588015	63	68.0561783	63
63	82.1411918	61	67.534831	63	78.5861474	61	77.5960402	63	67.5107238	63
63	82.6411918	61	67.534831	63	79.1861474	61	75.3532459	63	67.6016329	63
63	82.6411918	61	68.034831	63	79.3861474	61	77.5960402	63	68.0561783	63
63	82.8911918	61	67.534831	63	79.3861474	61	77.0960402	63	67.5107238	63
63	83.3911918	61	67.534831	63	81.1861474	61	76.7627068	63	67.5107238	63
63	82.8911918	61	67.034831	63	82.1861474	61	75.5960402	63	66.6016329	63
63	82.1411918	61	67.034831	63	81.3861474	61	75.4293735	63	66.5107238	63
63	81.1411918	61	67.034831	63	81.5861474	61	72.0960402	63	66.1470874	63
63	81.1411918	61	67.034831	63	79.7421587	61	71.5960402	63	65.692542	63
63	79.8911918	61	67.034831	63	79.9088254	61	71.5960402	63	65.4198147	63
63	79.8911918	61	66.034831	63	79.4088254	61	71.4293735	63	65.4198147	63
63	78.3911918	61	66.034831	63	79.4088254	61	69.9293735	63	65.6016329	63
63	77.8911918	61	66.034831	63	79.4088254	61	70.5960402	63	65.692542	63
63	79.0134889	61	66.034831	63	78.7421587	61	70.5960402	63	65.7834511	63
63	79.2134889	61	66.034831	63	78.7421587	61	70.7627068	63	66.0561783	63
63	79.0134889	61	66.034831	63	78.7421587	61	69.7627068	63	65.6016329	63
63	78.8134889	61	66.034831	63	79.0754921	63	69.0960402	63	64.9652692	63
63	78.6134889	61	66.034831	63	79.4088254	63	69.0960402	63	64.692542	63
63	78.4134889	61	66.034831	63	80.2421587	63	66.3163882	63	64.692542	63
63	76.0134889	61	66.034831	63	80.4088254	63	69.0960402	63	64.692542	63
63	76.2134889	61	65.534831	63	80.2421587	63	69.0960402	63	64.4198147	63
63	76.8134889	61	65.534831	63	80.0754921	63	65.8997215	63	64.6016329	63
63	76.6134889	61	66.534831	63	80.2421587	63	67.9293735	63	64.6016329	63
63	76.2134889	61	66.534831	63	79.0754921	63	67.4293735	63	64.8743601	63
63	75.2134889	61	66.534831	63	78.5754921	63	67.9293735	63	64.7834511	63
63	75.6134889	61	66.534831	63	78.0754921	63	68.0960402	63	64.8743601	63
63	75.4134889	61	66.534831	63	77.0754921	63	67.9293735	63	64.9652692	63
63	75.6134889	61	67.034831	63	75.9088254	63	67.9293735	63	65.3289056	63
63	75.8134889	61	67.534831	63	75.7421587	63	68.9293735	63	65.4198147	63
63	75.0134889	61	69.034831	63	75.7421587	63	65.8997215	63	65.8743601	63
63	75.6134889	61	69.534831	63	75.5754921	63	66.2052771	63	65.9652692	63
63	76.0134889	61	70.034831	63	75.9088254	63	66.6497215	63	65.692542	63
63	77.0134889	61	70.534831	63	75.7421587	63	66.2608327	63	66.1470874	63
63	78.2134889	61	69.534831	63	75.0754921	63	66.2608327	63	65.7834511	63
63	78.0134889	61	68.534831	63	74.9088254	63	66.4552771	63	66.4198147	63
63	78.6134889	61	69.034831	63	75.0754921	63	66.8163882	63	66.692542	63
63	80.0134889	61	68.534831	63	75.9088254	63	66.2330549	63	67.0561783	63
63	80.4134889	61	68.034831	63	75.9088254	63	66.0108327	63	66.8743601	63
63	81.2134889	61	68.534831	63	76.7421587	63	66.3163882	63	65.7834511	63

63	81.0134889	61	69.534831	63	76.9088254	63	68.9293735	63	65.5107238	63
63	81.0134889	61	70.034831	63	79.5754921	63	69.5960402	63	64.692542	63
63	80.4134889	61	70.034831	63	80.2421587	63	69.0960402	63	64.692542	63
63	79.4472432	61	69.534831	63	80.4088254	63	69.0960402	63	64.4198147	63
63	78.4472432	61	70.034831	63	80.5754921	63	68.5960402	63	64.7834511	63
63	77.6139099	61	70.034831	63	80.4088254	63	68.2627068	63	64.9652692	63
63	76.9472432	61	70.034831	63	79.4088254	63	67.5960402	63	65.1470874	63
63	77.6139099	61	69.534831	63	79.0754921	63	66.9293735	63	65.1470874	63
63	77.3788632	61	69.534831	63	78.7421587	63	67.2627068	63	66.0561783	63
63	77.093149	61	69.534831	63	78.0754921	63	67.2627068	63	66.2379965	63
63	77.093149	61	69.534831	63	77.2421587	63	66.9293735	63	66.2379965	63
63	76.8074347	61	69.534831	63	76.2421587	63	66.7627068	71	66.1470874	63
63	75.9502918	61	69.034831	63	76.5754921	63	66.5960402	71	66.9652692	63
63	75.9502918	61	69.534831	63	76.4088254	63	67.2627068	71	66.7834511	63
63	76.5217204	61	69.034831	63	76.4088254	63	67.2627068	63	66.692542	63
63	76.5217204	61	69.534831	63	77.2421587	63	66.2627068	63	67.0561783	63
63	76.3788632	61	69.034831	63	77.2421587	63	62.5386104	63	66.6016329	63
63	76.6645775	61	68.034831	63	77.2421587	63	62.3163882	63	66.692542	63
63	76.5217204	61	67.034831	63	77.5754921	63	62.3163882	63	66.9652692	63
63	75.3788632	61	66.534831	63	77.7421587	63	63.8719438	63	66.8743601	63
63	75.5217204	61	66.034831	63	77.7421587	63	64.3163882	63	66.692542	63
63	74.8074347	61	65.534831	63	77.7421587	63	64.8719438	63	67.0561783	63
63	74.093149	61	65.034831	63	77.5754921	63	66.4274993	63	67.0561783	63
63	75.2360061	61	64.534831	63	77.7421587	63	66.6497215	63	67.1470874	63
63	75.5217204	61	64.534831	63	76.7421587	63	66.7330549	63	66.7834511	63
63	74.6889155	61	64.534831	63	75.7421587	63	66.5386104	63	67.5107238	63
63	75.3139155	61	64.034831	63	75.5754921	63	67.1497215	63	67.2214301	63
63	75.5639155	61	63.534831	63	75.0754921	63	68.1497215	63	67.023083	63
63	75.3139155	61	63.534831	63	77.7421587	63	68.5177032	63	66.8247359	63
63	75.5639155	61	66.6271324	63	78.0754921	63	68.5779279	63	66.6677111	63
63	74.5639155	61	66.5938707	63	77.2421587	63	67.9552771	63	66.6677111	63
63	74.4389155	61	66.5606091	63	76.7421587	63	68.344166	63	66.3701904	63
63	73.9389155	61	66.5273474	63	77.4088254	63	69.1497215	63	65.0809342	63
63	74.0639155	61	66.4940857	63	77.0754921	63	70.6774993	63	64.8825871	63
63	73.6889155	61	66.4608241	63	76.9088254	63	72.4293735	63	64.68424	63
63	73.6889155	61	66.4275624	63	76.7421587	63	73.0960402	63	64.68424	63
63	73.0639155	61	66.3943007	63	77.0754921	63	71.9293735	63	64.5354797	63
63	72.8139155	61	66.3610391	63	76.9088254	63	71.4293735	63	64.6263888	63
63	72.9389155	61	66.3277774	63	77.0754921	63	71.5960402	63	64.5272152	63
63	72.8139155	63	66.2945157	63	77.2421587	63	71.5960402	63	63.7338268	63
63	72.8139155	63	66.261254	63	77.2421587	63	71.0960402	63	64.2214301	63
63	72.8139155	63	66.2279924	63	77.2421587	63	71.2627068	63	64.2049012	63
63	72.6889155	63	66.1947307	63	77.2421587	63	71.7627068	63	65.1470874	63

63	73.5639155	63	66.161469	63	78.2421587	63	71.9293735	63	64.6016329	63
63	73.5639155	63	66.1282074	63	78.0754921	63	71.9293735	63	64.692542	63
63	73.4389155	63	66.0949457	63	78.5754921	63	72.0960402	63	64.8743601	63
63	74.0639155	63	66.061684	63	78.7421587	63	72.0960402	63	65.3289056	63
63	73.9389155	63	66.0284223	63	78.5754921	63	72.0960402	63	65.4198147	63
63	73.8139155	63	65.9951607	63	78.9088254	63	71.9293735	63	65.7834511	63
63	72.6889155	63	65.961899	63	80.9088254	63	69.9293735	63	66.6016329	63
63	74.1889155	63	65.9286373	63	82.0754921	63	69.9293735	63	66.692542	63
63	73.8139155	63	65.8953757	63	82.0754921	63	69.9293735	63	65.9652692	63
63	74.0639155	63	65.862114	63	82.2421587	63	69.9293735	63	65.692542	63
63	70.0388615	63	65.8288523	63	81.5754921	63	70.2627068	63	65.4198147	63
63	72.7632822	63	65.7955906	63	81.0754921	63	69.4293735	69	65.7834511	63
63	72.9039072	63	65.762329	63	81.7421587	63	68.7627068	69	66.1470874	63
63	73.3257822	63	65.7290673	63	80.5754921	63	68.4293735	69	67.0561783	63
63	73.6070322	63	65.6958056	63	79.9088254	63	68.5960402	69	67.4198147	63
63	73.3570322	63	65.662544	63	79.0754921	63	68.2627068	69	67.4198147	63
63	73.3414072	63	65.6292823	63	78.5754921	63	68.0960402	69	67.2379965	63
63	74.6889155	63	65.5960206	63	77.7421587	63	67.4293735	69	66.8743601	63
63	72.5639155	63	65.562759	63	77.4088254	63	67.0960402	69	66.8743601	63
63	72.4389155	63	65.5294973	63	76.5754921	63	67.4293735	69	66.692542	63
63	72.0639155	63	65.4962356	63	76.4088254	63	67.0960402	69	66.9652692	63
63	72.0639155	63	65.4629739	63	76.7421587	63	67.0960402	69	67.692542	63
63	72.1889155	63	65.4297123	63	76.5754921	63	66.9293735	63	67.9652692	63
63	72.3139155	63	65.3964506	63	76.4088254	63	66.9293735	63	67.7834511	63
63	71.6889155	63	65.3631889	63	76.4088254	63	66.7627068	63	67.5107238	63
63	71.5639155	63	65.3299273	63	76.4088254	63	67.9293735	63	67.6016329	63
63	71.8139155	63	65.2966656	63	74.0367846	63	68.5960402	63	67.4198147	63
63	72.5639155	63	65.2634039	63	73.8939274	63	68.9293735	63	66.7834511	63
63	72.8139155	63	65.2301422	63	73.7510703	63	68.9293735	63	66.7834511	63
63	72.6889155	63	65.1968806	63	73.3224988	63	68.7627068	63	66.8743601	63
63	73.0639155	63	65.1636189	63	73.465356	63	69.0960402	63	66.692542	63
63	71.4045166	63	65.1303572	63	72.7510703	63	69.4293735	63	66.692542	63
63	73.6889155	63	65.0970956	63	72.7510703	63	69.5960402	63	66.692542	63
63	73.8139155	63	65.0638339	63	72.0367846	63	69.5960402	63	66.3289056	63
63	71.8767389	63	65.0305722	63	71.8939274	63	69.4293735	63	65.7834511	63
63	71.48785	63	64.9973106	63	72.465356	63	69.5960402	63	65.3289056	63
63	70.5434055	63	64.9640489	63	72.465356	63	69.9293735	63	65.2379965	63
63	70.7239611	63	64.9307872	63	72.1131737	63	69.7627068	63	65.2379965	63
63	70.86285	63	64.8975255	63	72.1131737	63	70.0960402	63	65.9652692	63
63	71.1545166	63	64.8642639	63	72.3631737	63	70.0960402	63	66.1470874	63
63	71.5711833	63	64.8310022	63	72.2381737	63	69.7627068	63	64.3030348	63
63	71.2934055	63	64.7977405	63	72.1131737	63	69.5960402	63	64.699729	63
63	71.4461833	63	64.7644789	63	72.2381737	63	69.7627068	63	65.022043	63

63	71.1406277	63	64.7312172	63	72.7381737	63	68.0960402	63	65.0303075	63
63	71.9739611	63	64.6979555	63	73.2381737	63	68.5960402	63	64.7410513	63
63	71.5434055	63	64.6646938	63	73.9881737	63	67.9293735	63	64.914605	63
63	71.2656277	63	64.6314322	63	74.3631737	63	67.5960402	63	65.0881588	63
63	70.8350722	63	64.5981705	63	75.3631737	63	67.7627068	63	64.3691505	63
63	71.11285	63	64.5649088	63	75.4881737	63	65.7627068	63	65.692542	63
63	71.3350722	63	64.5316472	63	76.1131737	63	65.5960402	63	65.4198147	63
63	72.5639155	63	64.4983855	63	76.7381737	63	65.2627068	63	64.4198147	63
63	73.8197358	63	64.4651238	63	76.6131737	63	65.0960402	63	63.692542	63
63	74.041958	63	64.4318621	63	76.6131737	63	64.9293735	63	63.6016329	63
63	74.1530691	63	64.3986005	63	76.7381737	63	64.7627068	63	63.6016329	63
63	74.3752914	63	64.3653388	63	76.7381737	63	64.9293735	63	63.4198147	63

1st 3 minutes S2 Dataset A

102014		102014		102114		102114		102214		102214
11-12.		9:00		9-10.		10-11.		9-10.		10-11.
62.5950535	61	68.5682219	61	75.3431299	71	70.5179799	63	69.8222677	61	64.2485032
63.150609	61	68.9015553	61	75.3431299	71	69.8679799	63	70.0222677	61	63.1127008
63.150609	61	68.2348886	61	75.6764632	71	70.6479799	63	70.2222677	61	64.9045393
62.9283868	61	70.9015553	61	76.3431299	71	71.0879799	63	70.4222677	61	64.4107121
63.150609	61	71.2348886	61	74.3431299	71	70.8679799	63	70.4222677	61	64.2749097
63.301845	61	71.5682219	61	74.0097966	71	70.9779799	63	70.6222677	61	63.2872553
63.150609	61	71.5682219	61	72.3431299	71	71.7579799	63	70.6222677	61	63.2872553
63.4839424	61	71.9015553	61	70.6764632	71	72.7379799	63	71.0222677	61	63.2872553
63.7061646	61	72.2348886	61	69.6764632	71	72.9579799	63	70.6222677	61	63.1637986
64.2617201	61	71.9015553	61	70.3431299	71	72.5179799	63	70.6222677	61	63.2872553
64.2617201	61	71.9015553	61	70.0097966	71	72.5079799	63	70.4222677	61	63.2872553
64.8053172	61	71.9015553	61	70.0097966	71	72.1679799	63	70.4222677	61	63.2872553
65.5950535	61	71.9015553	61	69.6764632	71	72.1679799	63	70.6222677	61	63.0403418
65.5950535	61	71.9015553	61	67.7959325	71	72.6079799	63	70.4222677	61	62.5341689
64.9283868	61	71.9015553	61	67.7959325	71	73.4879799	63	70.2222677	61	62.2872553
64.2617201	61	71.9015553	61	67.2959325	71	74.9585601	63	70.4222677	61	62.2872553
63.5950535	61	72.2348886	61	67.5459325	71	74.9585601	63	69.8222677	61	62.1761442
63.3728312	61	72.5682219	61	67.7959325	71	74.5585601	63	69.6222677	61	62.7934282
63.0394979	61	72.5682219	61	67.7959325	71	73.1585601	63	69.2222677	61	62.916885
62.4839424	61	72.2348886	61	67.7959325	61	72.5585601	63	69.4222677	61	62.6699714
62.4839424	61	73.2348886	61	67.7959325	61	72.2585601	63	69.4222677	61	62.8057739
62.5950535	61	73.2348886	61	67.5459325	61	70.6794007	63	69.6222677	61	63.1761442
62.8172757	61	74.2348886	61	68.2959325	61	72.9585601	63	69.4222677	61	63.0650331
62.5950535	61	74.9015553	61	69.0459325	61	70.8994007	63	69.6222677	61	63.1884899
63.0394979	61	75.2348886	61	69.0459325	61	72.995862	63	69.6222677	61	63.8057739
63.2617201	61	75.5682219	61	69.7959325	61	71.8847509	63	69.8222677	61	64.0526874
63.2617201	61	75.9015553	61	70.0459325	61	71.1069731	63	70.4222677	61	64.1761442
62.9283868	61	77.9015553	61	70.0459325	61	67.8601393	63	70.6222677	61	64.4230578
63.3728312	61	77.9015553	61	69.5459325	61	67.2428554	63	70.4222677	61	64.7810825
63.2617201	61	77.9015553	61	69.7959325	61	66.6132258	63	70.4222677	61	64.5218232
63.7061646	61	77.9015553	61	69.0459325	61	65.5021147	63	70.6222677	61	64.7687368
64.7061646	61	77.9015553	61	68.7959325	61	65.1317443	63	70.6222677	61	64.64528
64.7061646	61	81.6903142	61	69.0459325	61	64.7613739	63	70.2222677	61	64.64528
64.2617201	61	81.1903142	61	68.7959325	61	64.6379171	63	70.2222677	61	64.3860208
63.4839424	61	81.1903142	61	69.5459325	61	65.0082875	63	70.2222677	61	64.0033047
62.9283868	61	81.1903142	61	69.5459325	61	64.872485	63	70.0222677	61	64.0033047
62.9283868	61	80.9403142	61	69.7959325	61	64.6255714	63	69.8222677	61	64.225527
60.4839424	61	81.1903142	61	70.2959325	61	66.3291953	63	68.8222677	61	64.5958973
60.8172757	61	80.9403142	61	70.2959325	61	66.7736398	63	68.4222677	61	65.7070084

61.0394979	61	80.9403142	61	71.2959325	61	66.5514176	63	68.2222677	61	65.7070084
60.5950535	61	80.9403142	61	71.2959325	61	66.8847509	63	68.4222677	61	65.5835516
60.2617201	61	80.9403142	61	71.2959325	61	66.8847509	63	69.6222677	61	65.3242924
60.150609	61	80.9403142	61	72.2959325	61	67.7736398	63	70.0222677	61	64.8428109
60.150609	61	80.9403142	61	72.0459325	61	67.6625287	63	70.0222677	61	64.5958973
60.2617201	61	80.9403142	61	71.5613146	61	67.8847509	63	70.0222677	61	64.3489837
61.9852632	61	81.1903142	61	70.7613146	61	67.995862	63	69.6222677	61	64.8551566
62.0943541	61	81.1903142	61	70.7613146	61	66.0641034	63	69.0222677	61	65.2378726
62.1412072	61	81.4403142	61	70.5613146	61	66.002375	63	69.4222677	61	66.3138728
61.9412072	61	81.4403142	61	70.9613146	61	67.6443503	63	69.0222677	61	66.0916506
62.1412072	61	82.4403142	61	71.3613146	61	67.4344738	63	69.2222677	71	65.536095
62.4412072	61	82.4403142	61	73.7613146	61	67.0517577	63	69.4222677	71	65.0916506
63.1412072	61	82.4403142	61	74.7613146	61	67.0764491	63	70.0222677	71	65.0916506
64.6412072	61	81.6903142	61	74.5613146	61	67.2122516	63	69.6222677	71	65.0916506
64.8412072	61	76.911245	61	73.3613146	61	67.4838565	63	69.6222677	71	64.8694284
64.5412072	61	75.973745	61	72.5613146	61	67.27398	63	69.2222677	71	65.0916506
63.9412072	61	81.6903142	61	71.5613146	61	67.2863256	63	69.2222677	71	65.4249839
63.2412072	61	77.223745	61	71.5613146	61	67.2863256	63	69.0222677	71	65.536095
62.3412072	61	76.911245	61	71.5613146	61	67.2986713	63	68.6222677	71	65.7583173
61.8412072	61	77.098745	61	71.5613146	61	67.5455849	63	68.4222677	71	64.5444519
62.3412072	61	76.473745	61	71.5613146	61	69.27398	63	69.0222677	71	64.8284026
61.7412072	61	76.161245	61	71.7613146	61	69.5332392	63	70.0222677	71	65.4456865
61.9412072	61	75.536245	61	70.9613146	61	69.4097824	63	70.4222677	71	65.6802544
61.9412072	61	74.973745	61	71.5613146	61	69.6443503	63	70.6222677	71	67.6472062
62.4412072	61	74.348745	61	71.3613146	61	69.0147207	63	71.2222677	71	67.0916506
62.3849785	61	74.348745	61	70.5613146	61	68.5208935	63	71.4222677	71	66.6472062
61.8087777	61	72.473745	61	70.3613146	61	67.6073133	63	71.2222677	71	66.0916506
61.2412072	61	72.536245	61	71.1613146	61	67.3727454	63	71.6222677	71	65.8694284
60.6412072	61	72.661245	61	71.1613146	61	65.5580104	63	72.0222677	71	66.2027617
60.6412072	61	72.973745	61	72.3613146	61	66.6255714	63	72.8222677	71	65.9805395
59.4310619	61	72.973745	61	71.7613146	61	66.2552011	63	73.6222677	71	65.9805395
59.9946983	61	73.973745	61	71.9613146	61	68.1069731	63	73.6222677	71	65.6472062
62.1412072	61	74.286245	61	70.7613146	61	65.8895554	63	73.6222677	71	65.536095
62.6412072	61	74.598745	61	70.5613146	61	65.1317443	63	73.8222677	71	65.6472062
63.1412072	61	74.286245	61	69.1613146	61	65.5021147	63	72.8222677	71	65.3138728
63.2412072	61	72.911245	61	68.9613146	61	65.3786579	63	73.2222677	71	65.0916506
63.6412072	61	72.598745	61	69.3613146	61	67.1069731	63	73.2222677	71	66.3138728
63.6412072	61	72.286245	61	69.3613146	61	67.5514176	63	73.2222677	71	66.2027617
63.6412072	61	74.6903142	61	68.5613146	61	67.6625287	63	72.8222677	71	65.9805395
63.6412072	61	74.6903142	61	68.5613146	61	67.4403065	63	72.4222677	71	65.536095
63.9412072	71	74.4403142	61	68.5613146	61	66.995862	63	72.4222677	71	64.9805395
64.3412072	71	73.6903142	61	68.1613146	61	66.7736398	63	72.6222677	71	64.8694284
64.4412072	71	73.6903142	61	67.1613146	61	66.5514176	63	72.8222677	71	64.8694284

64.4412072	71	72.9403142	61	67.1613146	61	66.3291953	63	73.6222677	71	64.8694284
64.2412072	71	72.4403142	61	67.1613146	61	65.8847509	63	74.6222677	73	63.6472062
63.8412072	71	72.4403142	61	69.5333615	61	65.7736398	63	74.8222677	73	63.8694284
63.7412072	71	72.4403142	61	69.5333615	61	65.4403065	63	75.0222677	73	63.4249839
63.8412072	71	73.1903142	61	69.5333615	61	65.3291953	63	75.6222677	73	63.7583173
64.3412072	71	74.6903142	63	69.2000281	61	65.6625287	63	76.8222677	73	64.0916506
64.4412072	71	75.4403142	63	68.2000281	61	65.8847509	63	76.8222677	73	63.7583173
64.1412072	71	75.4403142	63	68.5333615	61	65.5514176	63	76.8222677	73	63.2027617
64.0412072	71	75.9403142	63	68.3666948	61	65.8847509	63	76.4222677	73	62.9805395
63.2412072	71	75.6903142	63	67.0333615	61	67.1069731	63	76.0222677	73	62.8694284
63.1412072	71	75.1903142	63	66.8666948	61	66.8847509	63	75.6222677	73	62.4249839
63.1412072	71	74.9403142	63	66.7000281	61	65.4221281	63	75.2222677	73	62.4249839
63.2412072	71	75.4403142	63	66.0333615	61	65.4344738	63	74.8222677	73	62.7583173
63.4412072	71	74.6903142	63	65.8666948	61	65.0887948	63	75.0222677	73	63.6472062
63.2412072	71	75.1903142	63	65.8666948	61	64.9776837	63	74.4222677	73	64.3138728
63.3412072	71	75.1903142	63	66.0333615	61	64.619659	63	72.8222677	73	64.4249839
63.5412072	71	77.1903142	63	66.2000281	61	64.2492886	63	72.8222677	73	64.2027617
63.9412072	71	76.4403142	63	66.0333615	61	64.619659	63	71.4222677	73	63.9805395
64.1412072	71	75.4403142	63	66.0333615	61	64.5085479	63	70.8222677	73	64.3138728
63.7412072	71	73.4403142	63	66.5333615	61	65.3727454	63	70.6222677	73	64.536095
63.0412072	71	72.9403142	63	66.8666948	61	65.9900293	63	70.6222677	73	65.3138728
62.2412072	71	72.6903142	63	67.0333615	61	66.27398	63	70.0222677	73	65.3138728
61.5412072	71	71.9403142	63	66.5333615	61	67.1505232	75	69.8222677	73	65.2027617
61.6412072	71	71.6903142	63	67.3666948	69	68.0394121	75	70.2222677	73	65.3138728
61.8412072	71	72.6903142	63	67.5333615	69	68.0394121	75	70.2222677	73	64.7583173
62.1412072	71	72.9403142	63	67.8666948	69	68.5332392	75	70.4222677	73	64.3138728
62.2412072	71	73.1903142	63	68.5333615	69	68.5455849	75	70.6222677	73	63.9805395
63.0412072	71	74.1903142	63	68.5333615	69	68.6690417	75	70.6222677	73	63.9805395
62.8412072	71	74.1903142	63	68.5333615	69	68.6690417	75	70.4222677	73	64.0916506
63.1412072	71	71.9403142	63	69.0333615	69	68.4221281	75	70.0222677	73	64.4249839
63.1412072	71	71.9403142	63	69.5333615	69	68.0517577	75	69.2222677	73	64.8694284
64.2412072	71	70.9403142	63	69.2000281	69	68.0641034	75	69.0222677	73	64.9805395
64.0412072	71	71.4403142	63	69.8666948	69	67.8048442	75	68.8222677	73	65.0916506
62.1144064	71	71.4403142	63	69.7000281	69	67.5579306	75	68.6222677	73	65.0916506
62.7689518	71	71.6903142	63	69.3666948	69	67.1875602	75	69.0222677	73	64.536095
62.75077	71	72.6903142	63	69.2000281	69	67.1875602	75	68.0222677	73	64.7583173
62.75077	71	73.6903142	63	69.2000281	69	67.5455849	75	67.4222677	73	64.8694284
61.998389	71	74.6903142	63	68.8666948	69	67.5208935	75	67.4222677	73	65.2027617
64.2412072	71	74.6903142	63	68.8666948	69	67.3233627	75	67.2222677	73	65.3138728
61.9412072	71	75.4403142	63	68.8666948	69	69.5514176	75	67.0222677	73	65.3138728
61.8412072	71	74.6903142	63	68.8666948	69	69.8847509	75	67.0222677	73	65.8694284
60.9412072	71	75.6903142	63	68.5333615	69	69.6625287	75	67.0222677	61	66.4249839
60.9412072	71	75.9403142	63	67.5333615	69	67.9406566	75	67.6222677	61	66.7583173

60.7412072	71	76.9403142	63	67.2000281	69	69.8847509	75	67.8222677	61	67.3138728
61.0412072	71	77.1903142	63	67.5333615	69	69.6625287	75	69.0222677	61	68.2027617
61.0412072	71	76.6903142	63	68.3666948	69	69.6625287	75	69.2222677	61	68.536095
61.3412072	71	76.6903142	63	69.0333615	69	69.5514176	75	70.2222677	61	68.536095
61.9412072	71	75.9403142	63	69.2000281	69	68.995862	75	70.6222677	61	68.6472062
61.9412072	71	75.9403142	63	69.5333615	69	68.8847509	75	71.4222677	61	68.3138728
62.1412072	71	75.9403142	63	69.7000281	69	68.8847509	75	72.0222677	61	68.3138728
62.0412072	71	75.9403142	63	69.7000281	69	68.6625287	75	72.0222677	61	67.9805395
62.2412072	71	74.6903142	63	70.0806473	69	68.1069731	75	72.6222677	61	67.536095
62.2412072	71	74.1903142	63	69.9377901	69	67.995862	75	73.0222677	61	67.3138728
62.7412072	71	73.4403142	63	69.9377901	69	67.2180842	75	74.2222677	61	67.8694284
62.9412072	71	72.1903142	63	70.6520759	69	66.5514176	75	73.6222677	61	68.0916506
63.3412072	71	71.4403142	63	71.0806473	69	65.4403065	75	70.6227018	61	68.3138728
63.0412072	71	71.4403142	63	70.6520759	69	64.995862	75	73.6222677	61	68.3138728
63.2412072	71	71.6903142	63	70.2235044	69	65.5514176	75	68.3027018	61	68.2027617
63.5412072	71	72.1903142	63	70.5092187	69	65.4403065	75	68.3027018	61	67.9805395
64.1412072	71	71.6903142	63	70.5092187	69	65.1069731	75	68.3027018	61	66.7583173
64.6412072	71	70.9403142	63	70.6520759	69	64.6625287	75	71.5251997	61	66.2027617
64.6412072	71	70.9403142	63	70.6520759	69	64.6625287	75	71.2196442	61	65.9805395
64.0412072	71	69.6903142	63	70.794933	69	65.2180842	75	71.4696442	61	65.9805395
64.0412072	71	69.6903142	63	67.4481668	69	65.2180842	75	71.6640886	61	66.4249839
61.7765164	71	69.4403142	63	67.6981668	69	65.3291953	75	71.8585331	61	66.536095
61.7765164	71	69.9403142	63	67.9481668	69	65.4403065	63	72.2474219	61	67.0916506
61.9037892	71	69.9403142	63	68.0731668	69	65.7736398	63	72.2474219	63	67.7604977
61.5765164	71	70.1903142	63	68.4481668	69	66.1069731	63	72.6085331	63	67.5876582
61.3583346	71	70.4403142	63	69.0731668	69	66.4403065	63	72.2196442	63	67.7234607
61.0310619	71	70.1903142	63	68.9481668	69	66.7736398	63	71.4418664	63	68.4395101
61.2674255	71	70.4403142	63	69.0731668	69	66.995862	63	71.2474219	63	68.5135842
61.1401528	71	70.4403142	63	69.1981668	69	66.4403065	63	70.7474219	63	67.9703743
61.5037892	71	70.9403142	63	68.8231668	69	66.5514176	63	70.9140886	63	66.0321027
62.1037892	71	70.9403142	63	68.4481668	69	66.5514176	63	69.7474219	63	65.3037076
61.9037892	71	70.9403142	63	68.4481668	69	66.995862	63	69.5529775	63	64.6987694
62.0310619	71	71.651788	63	68.9286951	69	67.6625287	63	69.5251997	63	64.6987694
61.8128801	71	71.451788	63	68.7064728	69	68.5514176	63	69.6918664	63	64.9333373
61.8674255	71	71.451788	63	68.817584	69	68.3291953	63	72.7435183	63	64.6246953
62.2310619	71	71.851788	63	69.3731395	69	67.7736398	63	72.910185	63	64.5876582
61.6492437	71	71.851788	63	70.817584	69	67.6625287	63	72.910185	63	64.7728434
61.9946983	71	71.651788	63	72.0398062	69	64.9406466	63	72.910185	63	64.6987694
62.6492437	71	72.451788	63	72.5953617	69	64.4591651	63	73.5768517	63	64.8469175
63.321971	63	72.651788	63	73.1509173	69	64.5949676	63	73.5768517	63	65.0444484
63.2128801	63	72.651788	63	73.3731395	69	65.1258318	63	70.9140886	63	65.1432138
63.4128801	63	73.051788	63	73.2620284	69	65.2863256	63	70.9140886	63	64.8345718
63.1946983	63	73.051788	63	72.817584	69	65.4097824	63	71.6918664	63	64.5629669

62.5583346	63	74.451788	63	73.0398062	69	66.1011404	63	71.6918664	63	65.7583173	
61.9037892	63	74.651788	63	73.7064728	69	66.3233627	63	74.5768517	63	65.7583173	
61.6492437	63	74.851788	63	74.0398062	69	66.1752145	63	74.7435183	63	64.7583173	
61.5401528	63	75.251788	63	74.2620284	69	67.4403065	63	74.5768517	63	64.536095	
61.636461	63	75.451788	63	74.3731395	69	67.1069731	63	74.5768517	63	64.7583173	
61.9236804	63	75.251788	63	72.8555963	69	66.5514176	63	74.410185	63	65.0916506	
62.9401528	63	75.651788	63	72.6210284	69	64.7743164	63	72.0807553	63	65.3138728	
63.6412072	63	75.651788	63	72.1272012	69	64.7743164	63	73.5768517	63	64.8694284	
64.6412072	63	75.651788	63	71.633374	69	64.1570324	63	73.410185	63	64.4249839	
64.1412072	63	75.251788	63	71.2630037	69	63.9101188	63	74.0768517	63	64.6472062	
63.6412072	63	75.651788	63	71.4975716	69	63.618652	63	74.2435183	63	64.6472062	
63.5412072	63	75.851788	63	70.156855	69	63.6298456	63	73.910185	63	65.2027617	

1st 3 minutes S2 Dataset A

	102214		102314		102414		102814		time	Average
	11-12.		9-10.30		9-10.		10-11.			
63	70.1221509	60	72.678859	63	79.1399432	63	75.1428571	63	0:00:01	73.2918191
63	70.4971509	60	72.678859	63	79.1399432	63	75.4285714	63	0:00:02	73.3035249
63	70.6846509	60	72.678859	63	78.6399432	63	75.5714286	63	0:00:03	73.4057722
63	66.3721509	60	72.678859	63	77.6399432	63	75.7142857	63	0:00:04	73.312347
63	66.3721509	60	72.678859	63	77.6399432	63	75.8571429	63	0:00:05	73.2218871
63	66.8721509	60	72.678859	63	76.6399432	63	75.2857143	63	0:00:06	73.3252288
63	65.9346509	60	72.678859	63	76.1399432	63	74.1428571	63	0:00:07	73.3176905
63	65.2471509	60	72.678859	63	75.1399432	63	73.7142857	63	0:00:08	73.3826564
71	65.2471509	60	71.678859	63	75.1399432	63	73.8571429	63	0:00:09	73.2487214
71	66.1846509	63	70.678859	63	75.1399432	63	73.5714286	63	0:00:10	73.4084973
71	65.5596509	63	70.678859	63	77.1399432	63	73.1428571	63	0:00:11	73.529039
71	65.4971509	63	70.678859	63	77.1399432	63	72.7142857	63	0:00:12	73.6265437
63	65.4971509	63	72.8072921	63	77.1399432	63	71.4285714	63	0:00:13	73.483611
63	65.4971509	63	72.3072921	63	77.1399432	63	71.2857143	63	0:00:14	73.535172
63	65.3096509	63	72.3072921	63	76.6399432	63	71.2857143	63	0:00:15	73.5482939
63	65.8096509	63	72.8072921	63	76.1399432	63	71.7142857	63	0:00:16	73.6366906
63	66.1846509	63	72.8072921	63	76.1399432	63	72.1428571	63	0:00:17	73.7154448
63	69.620477	63	73.3072921	63	75.6399432	63	71.8571429	63	0:00:18	74.0393786
63	69.9538104	63	73.3072921	63	75.1399432	63	71.4285714	63	0:00:19	73.9967851
63	70.4538104	63	72.8072921	63	73.2701234	63	71.2857143	63	0:00:20	74.0983881
63	71.120477	63	72.8072921	63	73.6034568	63	71.8571429	63	0:00:21	74.2208877
63	71.2871437	63	72.8072921	63	74.9367901	63	72.1428571	63	0:00:22	74.1687047
63	67.9971509	63	72.8072921	63	74.9367901	63	73.2857143	63	0:00:23	74.2168133
63	67.8096509	63	72.8072921	63	75.6034568	63	73.2857143	63	0:00:24	74.1899405
63	67.6221509	63	72.8072921	63	75.6034568	63	74	63	0:00:25	74.0995773
63	67.4346509	63	72.3072921	63	75.6034568	63	73.8571429	63	0:00:26	74.0796864
63	66.4346509	63	72.3072921	63	76.6034568	63	73.8571429	63	0:00:27	73.878187
63	66.0596509	63	72.8072921	63	76.2701234	63	73.5714286	63	0:00:28	73.7025103
63	66.0596509	63	72.8072921	63	75.2701234	63	73.7142857	63	0:00:29	73.6191671
63	68.2871437	63	72.8072921	63	75.2701234	63	70.6605211	63	0:00:30	73.5932579
63	68.2871437	63	74.8072921	63	75.6034568	63	70.6809292	63	0:00:31	73.7082848
63	68.2871437	63	74.8072921	63	76.6034568	63	71.1911333	63	0:00:32	73.8528541
63	68.2871437	63	74.8072921	63	76.9367901	63	71.1911333	63	0:00:33	73.9563361
63	66.7871437	63	77.3072921	63	76.6034568	63	71.5176639	63	0:00:34	73.8581525
63	66.620477	63	77.3072921	63	76.2701234	63	70.6605211	63	0:00:35	73.7245828
63	66.4538104	63	77.3072921	63	76.6034568	63	70.8237864	63	0:00:36	73.4564395
63	65.4538104	63	77.3072921	63	76.6034568	63	72.8571429	63	0:00:37	73.6612082
63	62.3721509	63	79.3072921	63	76.6034568	63	73.1428571	63	0:00:38	73.4243781
63	62.5596509	63	80.3072921	63	76.6034568	63	73.2857143	63	0:00:39	73.4827727

63	63.4971509	63	80.3072921	63	76.2701234	71	73.2857143	63	0:00:40	73.6122824
63	63.4971509	63	80.3072921	63	75.9367901	71	72.5714286	63	0:00:41	73.3812429
63	63.8096509	63	80.3072921	63	75.9367901	71	72	63	0:00:42	73.3215267
63	63.9971509	63	79.8072921	63	75.9367901	71	71.8571429	63	0:00:43	73.3585836
63	63.4346509	63	79.8072921	63	76.6034568	71	71.7142857	63	0:00:44	73.4064231
63	63.8096509	63	80.3072921	71	76.6034568	71	72.5714286	63	0:00:45	73.3767857
63	64.3721509	73	80.3072921	71	77.9367901	71	72.7142857	63	0:00:46	73.4975863
63	64.3721509	73	80.3072921	63	78.9367901	71	73.1428571	63	0:00:47	73.3422189
63	64.5596509	73	78.8072921	63	78.6034568	71	75.1428571	63	0:00:48	73.3439422
63	64.8721509	73	79.3072921	63	78.6034568	71	76.2857143	63	0:00:49	73.1928035
63	64.8721509	73	79.3072921	63	78.6034568	71	76.8571429	63	0:00:50	73.2366256
63	64.4971509	73	79.8072921	63	78.6034568	71	76.2857143	63	0:00:51	73.1063272
63	64.3096509	73	81.3072921	63	78.6034568	71	76.2857143	63	0:00:52	73.1856569
63	64.4971509	73	81.3072921	63	78.6034568	71	75.8571429	63	0:00:53	73.2301127
63	64.8721509	73	81.3072921	63	77.6034568	71	75.5714286	63	0:00:54	73.1707886
63	65.2471509	73	82.3072921	63	80.2701234	71	75.7142857	63	0:00:55	73.2552725
63	64.8721509	73	82.3072921	63	81.2701234	71	75.7142857	63	0:00:56	73.6038747
63	65.0596509	73	82.3072921	63	81.6034568	71	74.7142857	63	0:00:57	73.4380502
63	65.2471509	73	82.3072921	63	81.9367901	71	73.2857143	63	0:00:58	73.4463642
63	65.8721509	73	82.3072921	63	82.2701234	71	71.8571429	63	0:00:59	73.4051485
63	66.4971509	73	82.3072921	63	82.6034568	71	70.2857143	63	0:01:00	73.6173224
63	67.1221509	73	82.8072921	63	82.9367901	71	69.8571429	63	0:01:01	73.6914423
63	68.4971509	73	82.3072921	63	82.6034568	71	69.8571429	63	0:01:02	73.6029483
63	68.6846509	73	80.8072921	63	81.1018088	71	70.8571429	63	0:01:03	73.5612528
63	69.6221509	73	80.8072921	63	78.8518088	71	71.4285714	63	0:01:04	73.5441066
63	69.6846509	73	81.3072921	63	78.6018088	71	72	63	0:01:05	73.4327312
63	69.8096509	73	82.3072921	63	78.3518088	71	72.1428571	63	0:01:06	73.4265207
63	73.4538104	73	82.3072921	63	77.6018088	71	71.8571429	63	0:01:07	73.4136936
63	73.2871437	73	82.3072921	63	77.6018088	71	70.7142857	63	0:01:08	73.3205736
63	70.3721509	73	82.3072921	63	77.3518088	71	70.2857143	63	0:01:09	73.2249508
63	73.2871437	73	81.3072921	63	77.1018088	71	69.8571429	63	0:01:10	73.076639
63	68.8721509	73	81.3072921	63	76.8518088	71	70	63	0:01:11	73.081816
63	68.8721509	73	81.3072921	63	76.8518088	71	69.4285714	63	0:01:12	72.9794642
63	67.9346509	73	81.3072921	63	76.8518088	71	68.4285714	63	0:01:13	72.9589914
63	67.6846509	73	81.3072921	63	76.8518088	71	68.5714286	63	0:01:14	72.731398
63	68.2471509	73	81.3072921	63	76.6018088	63	68.7142857	63	0:01:15	72.744039
63	68.6221509	63	79.3072921	63	76.8518088	63	68.7142857	63	0:01:16	72.7972314
63	66.1513651	63	78.8072921	63	76.8518088	63	68.7142857	63	0:01:17	72.6259245
63	65.3180317	63	79.3072921	63	77.3518088	63	68.4285714	63	0:01:18	72.6363933
63	66.1846509	63	79.3072921	63	77.3518088	63	68.1428571	63	0:01:19	72.557697
63	65.9971509	63	79.8072921	63	76.1018088	63	67.7142857	63	0:01:20	72.3054227
63	66.0596509	63	80.3072921	63	76.1018088	63	68	63	0:01:21	72.268367
63	65.6221509	63	80.3072921	63	75.6018088	63	68.2857143	63	0:01:22	72.0952866

63	65.2471509	63	80.3072921	63	75.1018088	63	68.2857143	63		0:01:23	72.2613397
63	64.4971509	63	82.8072921	63	74.8518088	63	69	63		0:01:24	72.2853518
63	65.1846509	63	83.8072921	63	75.1018088	63	67.0269871	63		0:01:25	72.4218667
63	68.4538104	63	84.3072921	63	74.8518088	63	70.7142857	63		0:01:26	72.487706
63	70.2871437	63	84.3072921	63	74.8518088	63	71.4285714	63		0:01:27	72.627371
63	70.4538104	63	86.3072921	63	75.3518088	63	72.5714286	63		0:01:28	72.7427662
63	69.2871437	63	87.3072921	63	74.1018088	63	72.5714286	63		0:01:29	72.8019262
63	71.7871437	63	87.8072921	63	73.6018088	63	68.8637218	63		0:01:30	72.7889471
63	72.2871437	63	88.3072921	63	73.1018088	63	68.2106605	63		0:01:31	72.7981106
63	72.120477	63	87.8072921	63	73.1018088	63	69.4285714	63		0:01:32	72.9289731
63	74.120477	63	87.8072921	63	72.8518088	63	68.5714286	63		0:01:33	72.7937508
63	74.120477	63	87.8072921	63	73.3518088	63	68.8571429	71		0:01:34	72.6880304
63	73.7871437	63	87.8072921	63	73.3518088	63	69.5714286	71		0:01:35	72.6488013
63	73.620477	63	87.3072921	63	74.1018088	63	69.5714286	71		0:01:36	72.6517306
63	74.120477	63	86.8072921	63	75.8518088	63	70	71		0:01:37	72.7314412
63	73.7871437	63	84.3072921	63	75.6018088	63	71	71		0:01:38	72.7350415
63	72.4538104	63	83.8072921	63	74.6018088	63	71.1428571	71		0:01:39	72.5814853
63	72.2871437	63	83.8072921	63	74.6018088	63	71	71		0:01:40	72.5591083
63	72.120477	63	83.3072921	63	74.3518088	63	71	71		0:01:41	72.429724
63	71.9538104	63	83.8072921	63	74.3518088	63	71.2857143	71		0:01:42	72.3233708
63	72.4538104	63	83.8072921	63	74.1018088	63	71.4285714	71		0:01:43	72.3773952
63	73.9538104	63	83.8072921	63	74.3518088	63	70.8571429	71		0:01:44	72.347085
63	77.9538104	63	85.3072921	63	74.6018088	63	70.1428571	71		0:01:45	72.4362097
63	77.620477	63	87.8072921	63	74.6018088	63	71.2857143	71		0:01:46	72.6411708
63	77.620477	63	88.3072921	63	73.3518088	63	71.5714286	71		0:01:47	72.6080316
63	77.9538104	63	88.8072921	63	74.1018088	63	71.7142857	71		0:01:48	72.7303512
63	77.7871437	63	88.3072921	63	73.6018088	63	71.7142857	71		0:01:49	72.7622121
63	78.120477	63	88.3072921	63	74.3518088	63	72.1428571	71		0:01:50	72.9074708
63	77.120477	63	88.3072921	63	74.6018088	63	72.5714286	71		0:01:51	72.91959
63	77.120477	63	88.8072921	63	75.6018088	63	72.1428571	71		0:01:52	72.8932465
63	77.2871437	63	88.8072921	63	76.6018088	63	71.8571429	71		0:01:53	72.9164959
63	77.2871437	63	87.8072921	63	76.8518088	63	71.8571429	71		0:01:54	72.7996021
63	76.7871437	63	82.8072921	63	78.8518088	63	72	71		0:01:55	72.645769
63	76.4538104	63	79.8072921	63	79.1018088	63	72.2857143	71		0:01:56	72.5830242
63	76.4538104	63	77.8072921	63	78.6018088	63	72.8571429	71		0:01:57	72.560509
63	75.2871437	63	77.8072921	63	78.6018088	63	73.1428571	71		0:01:58	72.4871388
63	73.4538104	63	76.8072921	63	77.8518088	63	73.5714286	71		0:01:59	72.3480289
63	73.120477	63	76.3072921	63	77.8518088	63	73.4285714	71		0:02:00	72.3950971
63	73.2871437	63	76.3072921	63	77.6018088	63	72	71		0:02:01	72.2583596
63	72.9538104	63	75.3072921	63	77.3518088	63	72	71		0:02:02	72.2789422
63	72.620477	63	74.8072921	63	77.3518088	63	72.1428571	71		0:02:03	72.1973032
63	73.120477	63	75.3072921	63	77.3518088	63	72.1428571	71		0:02:04	72.2596822
63	73.620477	63	75.8072921	63	77.3518088	63	72.4285714	71		0:02:05	72.1324969

63	73.7871437	63	76.3072921	63	76.8518088	63	71.7142857	71	0:02:06	72.184174
63	73.9538104	63	76.3072921	63	76.6018088	63	71.2857143	71	0:02:07	72.3490786
63	73.9538104	63	76.8072921	63	76.1018088	63	70.8571429	71	0:02:08	72.2737501
63	73.4538104	63	76.3072921	63	75.6018088	63	70.5714286	71	0:02:09	72.259221
63	73.120477	63	77.3072921	63	75.1018088	63	71	71	0:02:10	72.1796541
63	72.620477	63	77.8072921	63	75.1018088	63	70.4285714	71	0:02:11	72.1823502
63	73.120477	63	77.8072921	63	74.8518088	63	70.2857143	71	0:02:12	72.2402808
63	72.7871437	63	77.8072921	63	75.3518088	63	70	71	0:02:13	72.3990937
63	73.4538104	63	78.3072921	63	75.1018088	63	70	71	0:02:14	72.4922635
63	76.2871437	63	78.3072921	63	75.3518088	63	69.5714286	71	0:02:15	72.5234759
63	75.4538104	63	77.3072921	63	75.6018088	63	69.1428571	71	0:02:16	72.4844072
63	75.2871437	63	77.3072921	63	75.1018088	63	69.4285714	71	0:02:17	72.3540221
63	73.2871437	63	77.3072921	63	74.3518088	63	69.1428571	71	0:02:18	72.1331175
63	71.9538104	63	77.3072921	63	73.6018088	63	69.8571429	71	0:02:19	72.1573596
63	72.4538104	63	77.3072921	63	72.6018088	63	69.2857143	71	0:02:20	72.0515601
63	72.7871437	63	77.3072921	63	72.1018088	63	70.1428571	71	0:02:21	72.1621603
63	73.4538104	63	77.3072921	63	72.1018088	63	69.7142857	71	0:02:22	72.1677387
63	69.1354317	63	78.4309396	63	72.3518088	63	69.2857143	71	0:02:23	72.1535842
63	69.1830507	63	77.7642729	63	71.8518088	63	69.1428571	71	0:02:24	72.0926749
63	68.9767015	63	77.7642729	63	71.8518088	63	69.2857143	71	0:02:25	72.1117659
63	69.2465428	63	77.7642729	63	71.6018088	63	69.4285714	63	0:02:26	72.1335655
63	69.5640031	63	77.4309396	63	71.8518088	63	69	63	0:02:27	72.0217636
63	72.1611686	63	77.4309396	63	72.1018088	63	68.2857143	63	0:02:28	72.0986049
63	72.0183114	63	77.0976062	63	72.6018088	63	68.1428571	63	0:02:29	72.0138505
63	72.0183114	63	76.7642729	63	73.1018088	63	68	63	0:02:30	71.9995235
63	71.3040257	63	76.0976062	63	73.6018088	63	67.4285714	63	0:02:31	72.0701141
63	71.7325971	63	75.4309396	63	74.1018088	63	66.2857143	63	0:02:32	71.9511085
63	72.1611686	63	75.8296353	63	74.1018088	63	66.5714286	63	0:02:33	71.9788256
63	73.7325971	63	75.0796353	63	73.6018088	63	66.4285714	63	0:02:34	71.9264709
63	74.0183114	63	75.0796353	63	73.8518088	63	66.2857143	63	0:02:35	71.7351526
63	73.8754543	63	74.8296353	63	74.1018088	63	66.2857143	63	0:02:36	71.7382929
63	73.7325971	63	74.5796353	63	74.1018088	63	66.7142857	63	0:02:37	71.7599185
63	73.8754543	63	74.5796353	63	74.3518088	63	66.8571429	63	0:02:38	71.9040492
71	73.8754543	63	74.5796353	63	75.3518088	63	66.7142857	63	0:02:39	71.9992183
71	74.0183114	63	74.8296353	63	76.3518088	63	66.7142857	63	0:02:40	72.19524
71	75.1611686	63	74.5796353	63	76.6018088	63	67.1428571	63	0:02:41	72.3383802
71	76.7325971	63	73.8296353	63	76.6018088	63	67.2857143	63	0:02:42	72.3154283
71	77.4468828	63	73.5796353	63	77.1018088	63	67.4285714	63	0:02:43	72.2949333
63	77.8754543	63	73.3296353	63	77.1018088	63	67.7142857	63	0:02:44	72.4405057
63	78.0183114	63	73.3296353	63	76.8518088	63	66.7142857	63	0:02:45	72.4630288
63	78.3040257	63	73.5796353	63	77.1018088	63	67.5714286	63	0:02:46	72.3320619
63	78.0183114	63	73.8296353	63	77.1018088	63	67.8571429	63	0:02:47	72.2133783
71	77.8754543	63	73.5796353	63	76.1018088	63	67.8571429	63	0:02:48	72.2461174

71	78.1513472	63	73.3296353	63	76.3518088	63	68.1428571	63		0:02:49	72.265178
63	77.9846805	63	73.8296353	71	75.6018088	63	68.5714286	63		0:02:50	72.3871055
63	77.8180139	63	73.8296353	71	74.8518088	63	69.1428571	63		0:02:51	72.3869437
63	77.6513472	63	73.5796353	63	74.8518088	63	69.5714286	63		0:02:52	72.3748807
71	78.1513472	63	73.8296353	63	74.3518088	63	69.1428571	63		0:02:53	72.3849867
71	77.6513472	63	73.8296353	63	73.6018088	63	69.2857143	63		0:02:54	72.3447223
71	77.8180139	63	73.8296353	63	72.8518088	63	69.1428571	63		0:02:55	72.4158753
71	77.3180139	63	74.3296353	63	73.1018088	63	69	63		0:02:56	72.4165018
71	76.4846805	63	74.5796353	63	72.8518088	63	69	63		0:02:57	72.4842181
71	74.8180139	63	74.8296353	63	72.6018088	63	69.7142857	63		0:02:58	72.3410522
71	74.6513472	63	74.3296353	63	72.8518088	63	69.1428571	63		0:02:59	72.2438451
71	73.8180139	63	73.8296353	63	73.3518088	63	68.2857143	63		0:03:00	71.9924524

S2B Dataset			Last 3 min						S2B Dataset	
time	91914.17		91914.18		92214.18		92214.2		92314.16	
	10:00-10:50		11:00-11:50		11-11:50		1-2:20		9am	
	1.00		2.00		3.00		4.00		5.00	
0:00:01	73.9273281	63	70.646412	65	73.0258374	65	70.6953836	71	66.1721197	63
0:00:02	74.0523281	63	70.5353009	65	72.9258374	65	70.6953836	71	66.0812106	63
0:00:03	73.9273281	63	71.6587577	65	72.6258374	65	69.8953836	71	65.9903015	63
0:00:04	73.8023281	63	71.0538194	65	72.5258374	65	70.4953836	71	66.2630288	63
0:00:05	73.6773281	63	71.0661651	65	72.7258374	65	70.6953836	71	66.7175742	63
0:00:06	73.3023281	63	72.4488811	65	72.8258374	65	70.6953836	71	66.8993924	63
0:00:07	73.0523281	63	73.4365354	65	72.8258374	65	72.0953836	71	67.8993924	63
0:00:08	73.3023281	63	74.2329976	65	72.0258374	65	72.2953836	71	68.1721197	63
0:00:09	73.0523281	63	74.0107753	65	71.9258374	65	72.0953836	71	68.1721197	63
0:00:10	73.3023281	63	71.1647878	65	71.6258374	65	72.2953836	71	68.535756	63
0:00:11	73.1773281	63	70.6833064	65	71.3258374	65	71.8953836	71	69.2630288	63
0:00:12	73.1773281	63	70.5598496	65	71.1258374	65	71.2953836	71	69.535756	63
0:00:13	73.9273281	63	70.5598496	65	71.0258374	65	72.0953836	71	69.6266651	63
0:00:14	73.5523281	63	70.5475039	65	71.0258374	65	72.8953836	71	69.9903015	63
0:00:15	73.5523281	63	71.1647878	65	71.1258374	65	74.2953836	71	70.3539378	63
0:00:16	74.3023281	63	71.4117014	65	71.1258374	65	74.4953836	71	70.3539378	63
0:00:17	74.3023281	63	72.677442	65	71.3258374	65	73.6953836	71	70.8993924	63
0:00:18	75.1773281	63	72.677442	65	71.2258374	65	72.2953836	71	71.0812106	63
0:00:19	75.1773281	63	72.8996642	65	70.9258374	65	71.2953836	71	69.9903015	63
0:00:20	75.1773281	63	71.7885531	65	70.8258374	65	71.6953836	71	69.2630288	63
0:00:21	75.3023281	63	72.4552198	65	70.8258374	65	71.8953836	71	69.1721197	63
0:00:22	75.0523281	63	72.677442	65	70.3258374	65	72.8953836	71	68.9903015	63
0:00:23	74.9273281	63	72.677442	65	70.2258374	65	73.2953836	71	68.9903015	63
0:00:24	74.1773281	63	73.4552198	65	70.3258374	65	73.2953836	71	68.6266651	63
0:00:25	72.9273281	63	73.3441087	65	70.3258374	65	72.0953836	71	68.2630288	63
0:00:26	72.1773281	63	73.0107753	65	70.6258374	65	70.6953836	71	68.3539378	63
0:00:27	72.1773281	63	73.1218865	65	70.9258374	65	69.8953836	71	67.9903015	63
0:00:28	72.4273281	63	72.5663309	65	70.6258374	65	69.6953836	71	68.3539378	63
0:00:29	72.6773281	63	72.8996642	65	69.8258374	65	69.4953836	71	67.8993924	63
0:00:30	73.6773281	63	72.3441087	65	69.8258374	65	68.8953836	71	67.8084833	63
0:00:31	74.0523281	63	72.3441087	65	69.9258374	65	68.4953836	71	67.4448469	63
0:00:32	74.1773281	63	71.8996642	65	70.1258374	65	68.0953836	71	67.4448469	63
0:00:33	72.4273281	63	71.4552198	65	70.8258374	65	67.6953836	71	67.535756	63
0:00:34	71.1773281	63	69.0414737	65	69.6766933	65	67.8953836	71	67.535756	63
0:00:35	71.0523281	63	68.0661651	65	69.6766933	65	67.6953836	71	67.7175742	63
0:00:36	70.6773281	63	67.3254243	65	69.5666933	65	67.4953836	71	67.8084833	63
0:00:37	70.8023281	63	66.8315972	65	69.3366933	65	67.2953836	71	67.8084833	63
0:00:38	70.3023281	63	67.2143132	65	71.0258374	65	66.8953836	71	67.9903015	63
0:00:39	70.8023281	63	67.5846836	65	71.0258374	65	67.0953836	71	67.9903015	63

0:00:40	70.9273281	63	69.2329976	65	70.5981292	65	67.6953836	71	67.8084833	63
0:00:41	70.4273281	63	68.4552198	65	71.9258374	65	67.8953836	71	68.2630288	63
0:00:42	70.3023281	63	68.3441087	65	72.3258374	65	68.6953836	71	68.3539378	63
0:00:43	70.0523281	63	68.4552198	65	72.2258374	65	70.0953836	71	67.9903015	63
0:00:44	69.9273281	63	68.3441087	65	71.8258374	65	70.0953836	71	67.8084833	63
0:00:45	70.0523281	63	67.2329976	65	72.2258374	65	70.4953836	71	66.9903015	63
0:00:46	70.6773281	63	66.8996642	65	72.4258374	65	70.0953836	71	66.8993924	63
0:00:47	71.3023281	63	67.0107753	65	72.4258374	65	69.2953836	71	66.9903015	63
0:00:48	71.8023281	63	66.5663309	65	72.0258374	65	70.4953836	71	67.0812106	63
0:00:49	72.0523281	63	66.7885531	65	72.2258374	65	70.8953836	71	66.7175742	63
0:00:50	72.4273281	63	67.1218865	65	72.8258374	65	71.2953836	71	66.8993924	63
0:00:51	72.4273281	63	67.3441087	65	73.3258374	65	70.6953836	71	66.7175742	63
0:00:52	72.8023281	63	66.8996642	65	73.2258374	65	67.8953836	71	67.0812106	63
0:00:53	73.0523281	63	66.8996642	65	73.5258374	65	63.6953836	71	67.1721197	63
0:00:54	73.6773281	63	67.0107753	65	73.4258374	65	66.0953836	71	67.2630288	63
0:00:55	73.8023281	63	66.7885531	65	74.2258374	65	66.6953836	71	67.3539378	63
0:00:56	74.0523281	63	66.7885531	65	74.0258374	65	66.2953836	71	67.4448469	63
0:00:57	73.3414297	63	66.8996642	65	74.0258374	65	67.0953836	71	67.4448469	63
0:00:58	73.4508047	63	66.677442	65	74.0258374	63	67.2953836	71	67.2630288	63
0:00:59	73.5758047	63	66.677442	65	73.8258374	63	67.8953836	71	66.8084833	63
0:01:00	74.1695547	63	66.7885531	63	73.8258374	63	67.8953836	71	66.8084833	63
0:01:01	74.1695547	63	67.0107753	63	73.5258374	63	68.0953836	71	66.9903015	63
0:01:02	73.9508047	63	67.677442	63	72.9258374	63	68.8953836	71	67.1721197	63
0:01:03	74.0914297	63	68.0107753	63	72.4258374	63	68.6953836	71	67.0812106	63
0:01:04	73.7789297	63	68.2329976	63	72.0258374	63	69.0953836	71	67.2630288	63
0:01:05	74.6226797	63	68.7885531	63	72.0258374	63	69.0953836	71	67.1721197	63
0:01:06	74.6226797	63	69.1218865	63	71.8258374	63	69.4953836	71	68.1721197	63
0:01:07	74.9195547	63	69.7885531	63	71.6258374	63	71.4953836	71	68.2630288	63
0:01:08	76.2008047	63	70.677442	63	71.4258374	63	71.2953836	71	68.0812106	63
0:01:09	75.7789297	63	70.4510888	63	71.1258374	63	73.6953836	71	67.2630288	63
0:01:10	75.5133047	63	70.327632	63	70.4258374	63	74.6953836	71	67.2630288	63
0:01:11	75.5133047	63	70.5745456	63	70.4258374	63	75.2953836	71	67.6266651	63
0:01:12	75.5133047	63	73.2329976	63	70.5258374	63	76.2953836	71	67.0812106	63
0:01:13	75.5289297	63	73.677442	63	70.6258374	63	75.4953836	71	67.9903015	63
0:01:14	75.6851797	63	73.4552198	63	70.7258374	63	75.4953836	71	66.9168146	63
0:01:15	75.7945547	63	72.7885531	63	71.4258374	63	75.0953836	71	66.7597898	63
0:01:16	75.7945547	63	72.677442	63	71.9258374	63	74.2953836	71	66.8084833	63
0:01:17	75.5758047	63	73.3441087	63	71.9258374	63	74.4953836	71	66.3539378	63
0:01:18	75.6851797	63	73.4552198	63	72.8258374	63	74.0953836	71	65.4448469	63
0:01:19	75.9508047	63	73.677442	63	73.0258374	63	73.8953836	71	65.3539378	63
0:01:20	76.2320547	63	73.8996642	63	72.6258374	63	73.8953836	71	65.7175742	63
0:01:21	76.6539297	63	73.3441087	63	72.4258374	63	72.0953836	71	65.8084833	63
0:01:22	76.6539297	63	73.2329976	63	73.5258374	63	74.2953836	71	65.3539378	63

0:01:23	76.7945547	63	73.2329976	63	73.7258374	63	74.2953836	71	65.8993924	67
0:01:24	78.6773281	63	73.3441087	63	73.7258374	63	74.4953836	71	66.1721197	67
0:01:25	78.5523281	63	73.3441087	63	73.6258374	63	74.0953836	71	66.4448469	67
0:01:26	78.3023281	63	73.5663309	63	74.0258374	63	74.2953836	71	66.535756	67
0:01:27	78.0523281	63	74.0107753	63	74.1258374	63	74.6953836	71	66.7175742	67
0:01:28	78.0523281	63	74.677442	63	74.7258374	63	73.8953836	71	67.1721197	67
0:01:29	77.4273281	63	74.1218865	63	75.1258374	63	74.6953836	71	67.1721197	67
0:01:30	76.9273281	63	74.1218865	63	75.4258374	63	74.0953836	71	67.0812106	67
0:01:31	76.5523281	63	74.1218865	63	75.4258374	63	73.6953836	71	66.6266651	67
0:01:32	76.8023281	63	74.1218865	63	75.5258374	63	74.0953836	71	66.535756	67
0:01:33	76.8023281	63	73.8996642	63	75.5258374	63	76.0953836	71	66.535756	67
0:01:34	76.8023281	63	73.4552198	63	75.5258374	63	75.6953836	71	66.535756	67
0:01:35	76.1773281	63	73.3441087	63	75.1258374	63	76.6953836	71	66.4448469	67
0:01:36	76.3023281	63	73.4552198	63	74.9258374	63	76.6953836	71	66.1721197	67
0:01:37	75.9273281	63	73.0107753	63	74.1258374	63	77.0953836	71	66.535756	67
0:01:38	75.3023281	63	72.1218865	63	73.5258374	63	76.8953836	71	66.7175742	67
0:01:39	75.3023281	63	71.1218865	63	73.8258374	63	71.6251951	71	66.9903015	67
0:01:40	75.4273281	63	70.677442	63	73.6258374	63	72.1851951	71	67.3539378	67
0:01:41	75.6773281	63	70.3441087	63	73.8258374	63	72.2251951	71	67.2630288	67
0:01:42	75.6773281	63	70.4552198	63	74.2258374	63	72.2251951	71	66.535756	67
0:01:43	75.6773281	63	70.4552198	63	74.5258374	63	71.2651951	71	66.2630288	67
0:01:44	75.1773281	63	70.5663309	63	74.6258374	63	70.3051951	71	65.7175742	67
0:01:45	74.6773281	63	68.7825148	63	75.0258374	63	70.3051951	71	64.535756	67
0:01:46	73.8023281	63	68.9183173	63	75.7258374	63	70.8251951	71	64.3539378	67
0:01:47	73.9273281	63	69.0541197	63	74.836277	63	71.5851951	71	64.2630288	67
0:01:48	74.0523281	63	69.1899222	63	74.226277	63	71.5851951	71	64.1721197	67
0:01:49	74.0523281	63	68.4491815	63	74.226277	63	69.9051951	71	64.7175742	67
0:01:50	74.0523281	63	68.0788111	63	74.886277	63	73.2651951	71	65.1721197	67
0:01:51	74.4273281	63	67.9677	63	75.176277	63	72.8251951	71	65.1721197	67
0:01:52	74.8023281	63	68.1158481	63	75.836277	63	71.6251951	71	65.8084833	67
0:01:53	75.1773281	63	68.4862185	63	76.166277	63	71.9051951	71	66.2630288	67
0:01:54	75.3023281	63	68.350416	63	75.946277	63	71.9051951	71	66.8084833	67
0:01:55	75.9273281	63	68.4738728	63	76.596277	63	72.8651951	71	66.4448469	67
0:01:56	76.1773281	63	69.1035024	63	76.256277	63	73.1051951	71	67.7175742	67
0:01:57	76.5523281	63	69.2516506	63	75.476277	63	74.3051951	71	68.2630288	67
0:01:58	77.3023281	63	68.7825148	63	74.486277	63	78.6953836	71	67.8993924	67
0:01:59	76.8023281	63	67.9430086	63	74.266277	63	79.6953836	71	67.8084833	67
0:02:00	76.8023281	63	67.4615271	63	74.686277	63	79.6953836	71	67.535756	67
0:02:01	75.6773281	63	66.8442432	63	76.1258374	63	75.2953836	71	67.7175742	67
0:02:02	75.5523281	63	66.2269592	63	76.0258374	63	74.0953836	71	67.6266651	67
0:02:03	75.5523281	63	66.5849839	63	75.6258374	63	73.8953836	71	68.3539378	67
0:02:04	75.6773281	63	65.9553543	63	75.1258374	63	74.6953836	71	68.3539378	67
0:02:05	75.8023281	63	65.8318975	63	74.7258374	63	74.8953836	71	68.8084833	67

0:02:06	76.1773281	63	65.041774	63	73.236277	63	74.8953836	71	68.6266651	67
0:02:07	76.8023281	63	64.5479469	63	72.926277	63	75.6953836	71	68.7175742	67
0:02:08	76.5523281	63	64.041774	63	72.506277	63	75.8953836	71	69.3539378	67
0:02:09	75.6773281	63	64.1405395	63	72.506277	63	75.6953836	71	69.6266651	67
0:02:10	75.0523281	63	64.2639963	63	72.396277	63	78.2953836	71	69.7175742	67
0:02:11	74.4273281	63	65.2269592	63	71.986277	63	78.2953836	71	69.4448469	67
0:02:12	74.3023281	63	65.7207864	63	72.426277	63	77.4953836	71	69.4448469	67
0:02:13	74.4273281	63	67.2329976	63	72.866277	63	78.2953836	71	68.9903015	67
0:02:14	73.6773281	63	66.3441087	63	73.106277	63	78.0953836	71	68.535756	67
0:02:15	73.6773281	63	65.7885531	63	73.766277	63	79.0953836	71	67.7175742	67
0:02:16	74.3023281	63	65.4552198	63	74.226277	63	79.0953836	71	67.2630288	67
0:02:17	74.4273281	63	65.8996642	63	74.566277	63	78.8953836	71	67.0812106	67
0:02:18	73.6773281	63	64.9059716	63	74.566277	63	78.6953836	71	66.8993924	67
0:02:19	72.8023281	63	65.2886876	63	74.9258374	63	77.2953836	71	67.0812106	67
0:02:20	73.1773281	63	65.1652308	63	74.1258374	63	75.0953836	71	68.1791799	67
0:02:21	72.6773281	63	64.3380703	63	73.5258374	63	74.0953836	71	68.6791799	67
0:02:22	73.6773281	63	64.1652308	63	73.8258374	63	73.2953836	71	68.8791799	67
0:02:23	74.5523281	63	64.041774	63	73.6258374	63	72.2953836	71	69.2791799	67
0:02:24	75.1773281	63	63.8072061	63	73.8258374	63	72.0953836	71	68.9791799	67
0:02:25	75.5523281	63	63.5602926	63	74.2258374	63	71.4953836	71	68.8791799	67
0:02:26	75.9273281	63	63.5602926	63	74.5258374	63	71.4953836	71	69.1791799	67
0:02:27	73.6909876	63	63.8072061	63	74.6258374	63	71.2953836	71	68.0791799	67
0:02:28	73.3941126	63	64.4368358	63	75.0258374	63	71.0953836	71	67.8791799	67
0:02:29	73.1128626	63	64.5602926	63	75.7258374	63	70.8953836	71	67.8791799	67
0:02:30	73.9273281	63	65.0664654	63	74.9898734	63	69.4953836	71	67.4791799	67
0:02:31	73.6773281	63	66.6714037	63	74.426237	63	68.8953836	71	67.4791799	67
0:02:32	73.8023281	63	65.2081322	63	74.426237	63	68.6953836	71	68.1791799	67
0:02:33	73.8023281	63	67.9183173	63	75.0807825	63	68.4953836	71	68.3791799	67
0:02:34	73.9273281	63	68.4121444	63	75.3353279	63	68.2953836	71	68.3791799	67
0:02:35	74.3023281	63	68.1652308	63	75.9898734	63	68.6953836	71	68.5791799	67
0:02:36	74.5523281	63	67.7825148	63	76.3171461	63	69.4953836	71	69.7791799	67
0:02:37	74.9273281	63	68.0294284	63	76.0989643	63	69.4953836	71	70.1791799	67
0:02:38	74.4273281	63	68.0788111	63	76.7353279	63	68.8953836	71	71.2791799	67
0:02:39	73.5523281	63	68.4491815	63	76.3898734	63	67.6953836	71	71.0791799	67
0:02:40	73.1773281	63	69.1218865	63	75.6080552	63	67.8953836	71	70.6791799	67
0:02:41	72.6773281	63	67.677442	63	74.626237	63	67.0953836	71	70.8791799	67
0:02:42	72.8023281	63	67.0107753	63	74.4080552	63	67.0953836	71	70.9791799	67
0:02:43	72.5523281	63	67.2329976	63	74.8080552	63	67.0953836	71	70.7791799	67
0:02:44	72.4273281	63	66.4552198	63	76.1258374	63	67.2953836	71	71.0791799	67
0:02:45	72.0523281	63	66.3441087	63	76.0258374	63	67.0953836	71	71.1791799	67
0:02:46	71.6773281	63	65.677442	63	75.6258374	63	69.0953836	71	70.7210154	67
0:02:47	71.8023281	63	65.2329976	63	75.1258374	63	74.2953836	71	70.8321265	67
0:02:48	72.0523281	63	64.8996642	63	74.7258374	63	74.2953836	71	71.4987932	67

0:02:49	72.3023281	63	65.0107753	63	73.3535098	63	67.3101721	71	71.7210154	67
0:02:50	72.8023281	63	65.2329976	63	73.0626007	63	66.2701721	71	71.9432376	67
0:02:51	72.8023281	63	65.1218865	63	72.6626007	63	71.2953836	71	72.276571	67
0:02:52	72.9273281	63	65.3441087	63	72.6626007	63	69.4953836	71	72.276571	67
0:02:53	73.5523281	63	65.2329976	63	72.5535098	63	70.0953836	71	71.9432376	67
0:02:54	73.5523281	63	65.3441087	63	72.1716916	63	70.2953836	71	71.4987932	67
0:02:55	74.8023281	63	65.4552198	63	72.6080552	63	69.8953836	71	71.8321265	67
0:02:56	75.0523281	63	65.5663309	63	73.0444189	63	70.4953836	71	71.9432376	67
0:02:57	75.6773281	63	65.677442	63	73.2989643	63	71.2953836	71	72.276571	67
0:02:58	75.8023281	63	65.4552198	63	73.9535098	63	72.6953836	71	72.276571	67
0:02:59	75.1773281	63	66.3441087	63	74.426237	63	72.6953836	71	70.9432376	60
0:03:00	74.6773281	63	66.3441087	63	74.7716916	63	74.0953836	71	71.0543487	60
							74.8953836	71		

Last 3 min S2B Dataset

92314.17		92414.15		92414.16		92414.17		92514.18		92514.2
10am		8.30-9.30		9:30-10:30		10:30-11:35		9-10:15		10:30-12
6.00		7.00		8.00		9.00		10.00		11.00
62.7396609	63	72.3514706	69	72.6614706	63	63.7647378	69	72.3282058	71	69.1398247
63.1446196	63	72.2414706	69	72.3514706	63	64.2647378	69	72.510024	71	68.2648247
63.3429667	63	71.7214706	69	72.2414706	63	64.9314045	69	72.8736604	71	67.5148247
63.9628014	63	71.1714706	69	71.7214706	63	65.2647378	69	72.8736604	71	67.5148247
64.5578428	63	71.1714706	69	71.1714706	63	65.5980711	69	73.3282058	71	66.7648247
64.5578428	63	70.7314706	69	71.1714706	63	66.0980711	69	73.6918422	71	66.2648247
64.0041237	63	70.0714706	69	70.7314706	63	66.0980711	69	73.8736604	71	65.6398247
64.2189998	63	70.4314706	69	70.0714706	63	66.0980711	69	72.2679925	71	65.3898247
64.3264378	63	70.2714706	69	70.4314706	63	65.9314045	69	71.9725379	71	65.2648247
64.5247849	63	70.0514706	69	70.2714706	63	66.2647378	69	72.3513258	71	65.3898247
64.8223056	63	69.0614706	69	70.0514706	63	64.9314045	69	73.2372968	71	65.2648247
65.2189998	63	68.9714706	69	69.0614706	63	64.2647378	69	74.3282058	71	65.7648247
65.9297436	63	68.9914706	69	68.9714706	63	63.5980711	69	74.6918422	71	66.1398247
65.83057	63	68.8814706	69	68.9914706	63	63.4314045	69	74.6009331	71	66.3898247
66.0289171	63	68.7714706	69	68.8814706	63	63.2647378	69	75.0554786	71	66.2648247
65.7313965	63	68.8814706	69	68.7714706	63	63.0980711	69	74.9645695	71	65.7648247
65.3925535	63	68.8814706	69	68.8814706	63	63.0980711	69	74.7827513	71	65.7648247
65.3016444	63	69.1014706	69	68.8814706	63	62.9314045	69	75.0554786	71	66.1398247
65.3347023	63	69.1014706	69	69.1014706	63	63.2647378	69	74.3282058	71	65.8898247
65.1859419	63	70.4214706	69	69.1014706	63	63.2647378	69	73.4191149	71	66.1398247
65.0371816	63	70.7514706	69	70.4214706	63	63.4314045	69	71.4339663	69	66.7648247
64.8553634	63	70.9814706	69	70.7514706	63	63.9314045	69	72.0400269	69	67.1398247
65.7479254	63	70.9814706	69	70.9814706	63	63.9314045	69	72.2369966	69	67.1398247
65.7479254	63	70.9914706	69	70.9814706	63	63.9314045	69	71.5476027	69	68.1398247
66.1446196	63	70.8814706	69	70.9914706	63	63.9314045	69	72.2372968	69	68.3898247
65.8553634	63	70.5514706	69	70.8814706	63	63.4314045	69	72.0554786	69	68.8898247
65.8801568	63	70.1114706	69	70.5514706	63	63.4314045	69	71.9645695	69	69.0148247
65.7809833	63	69.8914706	69	70.1114706	63	62.5836602	69	72.3282058	69	69.2648247
65.6818097	63	69.5614706	69	69.8914706	63	62.5836602	69	71.9645695	69	69.8898247
65.8718923	63	68.6814706	69	69.5614706	63	62.9836602	69	71.1463877	69	69.7648247
65.8223056	63	68.4614706	69	68.6814706	63	63.7836602	69	71.2372968	69	70.2648247
65.83057	63	69.2314706	69	68.4614706	63	64.3836602	69	70.8736604	69	70.2648247
65.83057	63	69.6714706	71	69.2314706	63	65.1836602	69	70.0554786	69	70.1398247
66.0289171	63	69.8814706	71	69.6714706	63	65.3836602	69	69.6009331	69	70.5148247
66.0371816	63	69.9914706	71	69.8814706	63	65.7836602	69	69.1463877	69	70.3898247
66.0371816	63	69.5514706	71	69.9914706	63	66.3836602	69	69.4191149	69	70.1398247
66.5330494	63	69.4314706	71	69.5514706	63	66.5836602	69	69.4191149	69	69.6398247
67.6074295	63	69.3214706	71	69.4314706	63	67.5836602	69	69.7827513	69	68.8898247
68.0123882	63	69.4314706	71	69.3214706	63	67.7836602	69	69.8736604	69	68.1398247

68.0041237	63	69.8614706	71	69.4314706	63	68.1836602	69	69.8736604	69	68.1398247
68.2024709	63	70.0814706	71	69.8614706	63	67.9836602	69	70.4191149	69	68.5148247
67.4090824	63	70.5214706	71	70.0814706	63	67.7836602	69	70.510024	69	68.7648247
67.2107353	63	71.2814706	71	70.5214706	63	66.1836602	69	70.9645695	69	67.7648247
67.8057766	63	72.1614706	71	71.2814706	63	65.9836602	69	70.9645695	69	68.2648247
67.8966857	63	72.3814706	71	72.1614706	63	63.9836602	69	70.3282058	69	68.2648247
68.3925535	63	72.7014706	71	72.3814706	63	63.7836602	69	69.8736604	69	68.1398247
68.6900742	63	73.1414706	71	72.7014706	63	63.9836602	69	69.8736604	69	68.0148247
69.0785039	63	73.6914706	71	73.1414706	63	64.1836602	69	70.0554786	69	68.1398247
68.9875948	63	74.2414706	71	73.6914706	63	64.9836602	69	69.9645695	69	68.6398247
68.9132147	63	73.6814706	71	74.2414706	63	64.3836602	69	70.6009331	69	69.0148247
68.5165204	63	73.5714706	71	73.6814706	63	62.9836602	69	71.3282058	69	69.0148247
68.0537105	63	73.6314706	71	73.5714706	63	62.1836602	69	71.8736604	69	69.2648247
67.5743717	63	74.7883718	71	73.6314706	63	62.1836602	69	71.9645695	71	69.6398247
67.6735452	63	75.0883718	71	74.7883718	63	62.9836602	69	72.0554786	71	69.6398247
67.6900742	63	74.0883718	71	75.0883718	63	62.9836602	69	72.1463877	71	70.1398247
67.6900742	63	73.4883718	71	74.0883718	63	64.7836602	69	71.8736604	71	70.5148247
67.1942064	63	73.1883718	71	73.4883718	63	65.3836602	69	72.6009331	71	70.8898247
67.0867684	63	72.5883718	71	73.1883718	63	66.5836602	69	72.9645695	71	70.8898247
66.8884213	63	72.6883718	71	72.5883718	63	66.3836602	69	71.9876895	71	70.7648247
66.8884213	63	72.6883718	71	72.6883718	63	66.5836602	69	72.2149622	71	71.2648247
66.8636279	63	73.4883718	71	72.6883718	63	66.5836602	69	72.313447	71	71.6398247
67.2520576	63	73.3883718	71	73.4883718	63	67.7836602	69	72.8058713	71	69.4148716
66.954537	63	73.4883718	71	73.3883718	63	67.7836602	69	73.3816288	71	69.4148716
66.8553634	63	72.7883718	71	73.4883718	63	66.1836602	69	74.8736604	71	70.4426494
67.0537105	63	72.0883718	71	72.7883718	63	65.9836602	69	74.8736604	71	70.9148716
67.615694	63	71.5883718	71	72.0883718	63	66.3836602	69	75.6918422	71	70.5537605
67.0206527	63	70.0538582	71	71.5883718	63	66.1836602	69	75.3282058	71	70.4148716
66.9049502	63	69.8638582	71	70.0538582	63	65.9836602	69	73.7827513	71	70.8898247
66.2107353	63	69.7538582	71	69.8638582	63	66.1836602	69	73.6009331	71	70.1398247
65.7148675	63	69.8838582	71	69.7538582	63	66.1836602	69	73.510024	71	70.0148247
65.615694	63	69.7838582	71	69.8838582	63	66.1836602	69	73.7827513	71	69.2648247
65.8140411	63	69.6838582	71	69.7838582	63	65.9836602	69	74.1463877	71	68.1398247
65.4090824	63	69.6838582	71	69.6838582	63	64.9836602	69	75.1463877	71	68.0148247
65.2107353	63	69.6938582	71	69.6838582	63	64.3836602	69	75.4191149	71	67.6398247
64.4090824	63	69.3738582	71	69.6938582	63	63.9836602	69	74.8736604	71	67.3898247
63.9710659	63	69.1938582	71	69.3738582	63	63.3836602	69	74.4191149	71	67.1398247
63.8553634	63	69.3438582	63	69.1938582	63	63.5836602	69	74.510024	71	66.6398247
63.723132	63	70.7883718	63	69.3438582	63	63.9836602	69	74.2372968	71	66.8898247
64.1115618	63	70.8883718	63	70.7883718	63	63.7836602	69	73.7827513	71	66.7648247
64.6074295	63	69.3994593	63	70.8883718	63	63.7836602	61	73.510024	71	66.5148247
65.1032973	63	69.1894593	63	69.3994593	63	64.5836602	61	73.8736604	71	66.3898247
65.5991651	63	69.1994593	63	69.1894593	63	65.5836602	61	73.7827513	71	66.8898247

66.1776775	63	69.0894593	63	69.1994593	63	66.1836602	61	73.510024	71	67.5148247
66.276851	63	68.7894593	63	69.0894593	63	66.9836602	61	73.8736604	71	67.7648247
66.0785039	63	68.4594593	63	68.7894593	63	68.1836602	61	73.7827513	71	67.8898247
65.8801568	63	68.0294593	63	68.4594593	63	68.9836602	61	73.7827513	71	68.0148247
65.9958593	63	67.8094593	63	68.0294593	63	69.3836602	61	73.1463877	71	67.7648247
66.0041237	63	67.9194593	63	67.8094593	63	69.5836602	61	73.2372968	71	68.0148247
66.400818	63	68.0294593	63	67.9194593	63	70.1836602	61	72.9645695	71	68.2648247
66.7975122	63	68.1394593	63	68.0294593	63	70.3836602	61	72.4191149	71	68.0148247
67.276851	63	68.5594593	63	68.1394593	63	70.5836602	61	72.4191149	71	68.1398247
67.2685866	63	68.7794593	63	68.5594593	63	70.3836602	61	72.6009331	71	68.1398247
67.3594956	63	68.7694593	63	68.7794593	63	70.3836602	61	72.7827513	71	67.5148247
67.5495783	63	68.0894593	63	68.7694593	63	69.9836602	61	72.7827513	71	67.2648247
68.1280907	63	68.1994593	63	68.0894593	63	69.1836602	61	71.4564457	71	67.0148247
67.9793304	63	68.1994593	63	68.1994593	63	68.1836602	61	71.6685669	71	67.2648247
69.6905293	63	68.0794593	63	68.1994593	63	68.1836602	61	72.1609911	71	67.6398247
68.9632566	63	68.4094593	63	68.0794593	63	68.9836602	61	72.1609911	71	67.6398247
69.0541657	63	69.9883718	63	68.4094593	63	70.9836602	61	72.259476	71	67.8898247
68.6905293	63	70.6883718	63	69.9883718	63	70.9836602	61	72.8655366	71	67.6398247
68.1450748	63	69.6883718	63	70.6883718	63	70.9836602	61	72.7670517	71	67.3898247
67.1450748	63	69.4883718	63	69.6883718	63	66.1697311	61	72.7670517	71	66.8898247
66.0541657	63	69.0883718	63	69.4883718	63	66.7297311	61	72.4867487	71	66.8898247
66.0541657	63	69.4883718	63	69.0883718	63	67.4897311	61	72.5852336	71	66.8898247
66.3268929	63	69.2883718	63	69.4883718	63	68.4097311	61	72.2064457	71	67.1398247
66.7814384	63	69.1883718	63	69.2883718	63	68.2897311	61	71.7140214	71	67.5148247
66.9632566	63	69.3883718	63	69.1883718	63	68.3297311	61	71.5170517	71	67.8898247
67.1450748	63	68.9883718	63	69.3883718	63	68.0897311	61	71.2215972	71	68.7648247
66.8723475	63	68.6883718	63	68.9883718	63	68.1697311	61	70.7140214	71	69.1398247
67.0541657	63	69.0883718	63	68.6883718	63	67.8097311	61	70.9109911	71	69.6398247
67.1450748	63	69.0883718	63	69.0883718	63	68.1297311	61	71.009476	71	69.7648247
67.3268929	63	69.0883718	63	69.0883718	63	68.3697311	61	70.6003851	71	69.8898247
67.5087111	63	69.5883718	63	69.0883718	63	68.2897311	61	70.4034154	71	69.3898247
67.5087111	63	69.7883718	63	69.5883718	63	68.4497311	61	71.4191149	71	69.7648247
67.5996202	63	69.5883718	63	69.7883718	63	68.4497311	61	71.4191149	71	70.1398247
68.3268929	63	69.3883718	63	69.5883718	63	66.5297311	61	71.4191149	71	71.0148247
68.0541657	63	69.0883718	63	69.3883718	63	66.6897311	61	71.8736604	71	71.0148247
68.7814384	63	69.6883718	63	69.0883718	63	66.2897311	61	72.1463877	71	71.3898247
65.9793304	63	70.0883718	63	69.6883718	63	69.5836602	61	72.6918422	71	71.1398247
66.0123882	63	70.6883718	63	70.0883718	63	68.1836602	61	72.6918422	71	71.2648247
66.0371816	63	71.2883718	63	70.6883718	63	65.3836602	61	72.4191149	71	71.0148247
66.4504047	63	71.6883718	63	71.2883718	63	64.9836602	61	72.6918422	71	71.3898247
66.5495783	63	71.3883718	63	71.6883718	63	63.3836602	61	72.6918422	71	71.7648247
65.6487518	63	70.9883718	63	71.3883718	63	63.5836602	61	73.0554786	71	72.2648247
65.8140411	63	70.7883718	63	70.9883718	63	63.3836602	61	73.6009331	71	72.8898247

65.9793304	63	68.6914706	63	70.7883718	63	62.3836602	61	73.8736604	71	72.8898247
68.2359838	63	68.6914706	63	68.6914706	63	61.9836602	61	73.8736604	71	73.2648247
68.5996202	63	68.5814706	63	68.6914706	63	62.1836602	61	74.0554786	71	73.3898247
68.7814384	63	68.3714706	63	68.5814706	63	62.1836602	61	74.0554786	71	73.5148247
68.417802	63	68.1814706	63	68.3714706	63	61.9836602	61	74.510024	71	73.8898247
68.417802	63	68.4714706	63	68.1814706	63	61.9836602	61	74.7827513	71	74.1398247
68.1450748	63	69.4883718	63	68.4714706	63	61.7836602	61	75.4191149	71	74.2648247
67.1450748	63	69.1883718	63	69.4883718	63	62.1836602	61	75.510024	71	74.5148247
66.8723475	63	68.9883718	63	69.1883718	63	62.5836602	61	75.2372968	71	74.5148247
66.3268929	63	68.7883718	63	68.9883718	63	62.5836602	61	74.8736604	71	74.5148247
66.0541657	63	68.4883718	63	68.7883718	63	62.1836602	61	74.4191149	71	72.5146274
65.3268929	63	68.2883718	63	68.4883718	63	62.1836602	61	74.6918422	71	72.2646274
65.2359838	63	68.1883718	63	68.2883718	63	61.9836602	61	74.8736604	71	72.4590718
65.9632566	63	68.6883718	63	68.1883718	63	62.3836602	61	74.9645695	71	72.2090718
65.8723475	63	68.1883718	63	68.6883718	63	62.1836602	61	75.0554786	71	73.4590718
65.5087111	63	67.9883718	63	68.1883718	63	61.9836602	61	75.2372968	71	73.0424052
65.5996202	63	68.0883718	63	67.9883718	63	61.5836602	61	75.2372968	71	73.1812941
64.9632566	63	67.9883718	63	68.0883718	63	61.5836602	61	75.7827513	71	72.7924052
64.8723475	63	68.1883718	63	67.9883718	63	61.7836602	61	76.0554786	71	73.070183
64.8723475	63	68.0883718	63	68.1883718	63	62.1836602	60	75.9645695	71	72.7924052
64.7814384	63	68.1883718	63	68.0883718	63	63.1836602	60	75.7827513	71	72.070183
65.1450748	63	68.3883718	63	68.1883718	63	63.5836602	60	73.9744046	71	71.6535163
65.0541657	63	68.5883718	63	68.3883718	63	63.5836602	60	73.9744046	71	70.7924052
65.3268929	63	68.4883718	63	68.5883718	63	63.7836602	60	72.8910712	71	70.2646274
65.417802	63	69.0883718	71	68.4883718	63	63.7836602	60	72.6941015	71	70.5146274
65.417802	63	68.0694593	71	69.0883718	63	63.9836602	60	72.7925864	71	72.3898247
65.417802	63	69.1794593	71	68.0694593	63	63.5836602	60	74.3282058	71	71.7648247
65.9632566	63	69.3994593	71	69.1794593	63	63.3836602	60	74.2372968	71	71.6398247
66.0541657	63	69.1794593	71	69.3994593	63	63.7836602	60	74.0554786	71	71.3898247
65.6905293	63	69.1794593	71	69.1794593	63	64.5836602	60	73.9645695	71	70.1398247
65.6905293	63	68.2994593	71	69.1794593	63	65.1836602	60	73.8736604	71	69.3898247
65.7814384	63	67.9694593	71	68.2994593	63	65.3836602	60	73.8736604	71	68.3898247
65.8723475	63	67.7494593	71	67.9694593	63	65.7836602	60	74.2372968	71	68.1398247
66.417802	63	67.8594593	71	67.7494593	63	64.9836602	60	74.1463877	71	67.8898247
66.3268929	63	67.8594593	71	67.8594593	63	64.3836602	60	74.2372968	71	68.0148247
65.9632566	63	67.4194593	71	67.8594593	63	65.1836602	60	74.2372968	71	68.0148247
65.1450748	63	67.5394593	71	67.4194593	63	65.3836602	60	75.510024	71	68.2648247
64.8723475	63	67.7594593	71	67.5394593	63	65.5836602	60	75.7827513	71	66.3757385
65.0541657	63	69.0694593	71	67.7594593	63	65.9836602	60	76.3282058	71	66.6535163
65.417802	63	70.6883718	71	69.0694593	63	66.1836602	60	76.9645695	71	66.5979607
65.3268929	63	70.4883718	71	70.6883718	63	66.5836602	60	77.4191149	71	66.5979607
65.1450748	63	70.5883718	71	70.4883718	63	67.3836602	60	77.4191149	71	66.8757385
65.1450748	63	69.0694593	71	70.5883718	63	67.5836602	60	76.2672997	71	66.6257385

65.1450748	63	70.4883718	71	69.0694593	63	67.5836602	60	76.3960876	61	66.5424052
65.1450748	63	70.7883718	71	70.4883718	63	67.1836602	60	76.3127542	61	66.9868496
64.5996202	63	71.0883718	71	70.7883718	63	67.1836602	60	76.5097239	61	66.5146274
64.1450748	63	71.1883718	71	71.0883718	63	67.1836602	60	76.3127542	61	67.320183
64.3268929	63	71.2883718	71	71.1883718	63	66.5836602	60	76.0324512	61	67.2646274
64.5996202	63	71.8883718	71	71.2883718	63	66.1836602	60	75.0627542	61	67.4590718
64.7814384	63	72.1883718	71	71.8883718	63	65.3836602	60	74.57033	61	67.8757385
65.2359838	63	72.7883718	71	72.1883718	63	64.7836602	60	73.7672997	61	67.9868496
64.8723475	63	72.9883718	71	72.7883718	63	65.1836602	60	73.7672997	61	68.1257385
64.6905293	63	72.7883718	71	72.9883718	63	65.7836602	60	73.57033	61	68.570183
64.3268929	63	72.3883718	71	72.7883718	63	66.3836602	60	73.57033	61	68.5146274
64.2359838	63	72.2883718	71	72.3883718	63	66.1836602	60	73.0627542	61	69.3757385
				72.2883718	60	66.3836602	60	73.6362287	61	69.5146274

Last 3 min S2B Dataset Las

	92614.17		92614.18		92914.17		92914.18		92914.2	
	10-11.		11-12.		9:30-10:46		11-11:50		1-2:00	
	12.00		13.00		14.00		15.00		16.00	
63	79.8888889	63	71.6666667	63	70.8621712	63	64.4458951	63	67.8863882	63
63	79.1111111	63	72.1111111	63	71.589444	63	64.2640769	63	67.7863882	63
69	78.5555556	63	72.2222222	63	71.8621712	63	64.1731678	63	67.1863882	63
69	78.4444444	63	73.1111111	63	72.4985349	63	63.7186223	63	67.1863882	63
69	78.4444444	63	71.557223	63	72.4076258	63	63.0822587	63	66.8863882	63
69	78.1111111	63	70.9275934	63	70.6922306	63	62.9913496	63	68.1863882	63
69	77.8888889	63	70.3103095	63	70.7914042	63	62.9913496	63	67.4863882	63
69	77.4444444	63	69.9399391	63	71.1054538	63	62.7186223	63	66.980092	63
69	77.3333333	63	69.6930255	63	71.1137182	63	62.5368042	63	66.030092	63
69	77.2222222	63	69.1991983	63	70.1219827	63	62.354986	63	66.160092	63
69	77	63	68.3350008	63	70.0228091	63	61.7186223	63	66.050092	63
69	77.1111111	63	67.9646304	63	69.7087596	63	61.2640769	63	66.500092	63
69	77.2222222	63	67.9769761	63	69.4938835	63	61.0822587	63	67.160092	63
69	77.6666667	63	68.8411737	63	70.589444	63	61.4458951	63	67.160092	63
69	78.2222222	63	69.4337662	63	70.1348985	63	62.1731678	63	67.710092	63
69	78.5555556	63	68.7794453	63	69.8621712	63	62.354986	63	67.490092	63
69	78.3333333	63	69.8888889	63	70.1348985	63	63.5368042	63	66.940092	63
69	77.8888889	63	69.5555556	63	70.4985349	63	63.9004405	63	67.160092	63
69	77.6666667	63	69.6666667	63	71.0439894	63	64.2640769	63	67.250092	63
69	77	63	70.2222222	63	70.4076258	63	64.6277133	63	68.8863882	63
69	77.1111111	63	70.1111111	63	69.8621712	63	64.8095314	63	69.8863882	63
69	76.7777778	63	70.8888889	63	69.589444	63	65.2640769	63	69.7863882	63
69	76.5555556	63	71.5555556	63	68.8621712	63	65.6277133	63	68.5863882	63
69	76	63	72.8888889	63	68.3167167	63	64.9913496	63	68.5863882	63
69	75.7777778	63	72.6666667	63	68.2258076	63	65.2640769	63	68.0863882	63
69	76	63	73.7777778	63	67.4076258	63	65.2640769	63	67.7863882	63
69	75.7777778	63	73.5555556	63	67.0439894	63	65.6277133	63	68.1863882	63
69	76	63	74.5555556	63	67.4076258	63	65.7186223	63	68.4863882	63
69	75.4444444	63	75	63	67.680353	63	66.0822587	63	69.4863882	63
69	75.5555556	63	75.7777778	63	68.0439894	63	66.5368042	63	70.1863882	63
69	75.7777778	63	77	63	68.2258076	63	66.8095314	63	69.8863882	63
69	75.6666667	63	77	63	69.2258076	63	67.0822587	63	69.1863882	63
69	75.6666667	63	75.8888889	63	69.4076258	63	67.2640769	63	68.6863882	63
69	75.8888889	63	73.8123195	63	69.4076258	63	67.8095314	63	68.2863882	63
69	76.1111111	63	73.3184923	63	69.4076258	63	67.9913496	63	67.7863882	63
69	76.1111111	63	73.5900972	63	69.4076258	63	67.7186223	63	67.9863882	63
69	76.6666667	63	73.6024429	63	69.8621712	63	67.6277133	63	67.5863882	63
69	77	63	74.2197269	63	69.9530803	63	67.0822587	63	67.3863882	63
69	77.2222222	63	74.7777778	63	69.7712621	63	66.9004405	63	66.9863882	63

69	77.3333333	63	75	63	69.7712621	63	67.1731678	63	66.8863882	63
69	77.6666667	63	74.6666667	63	69.8621712	63	67.6277133	63	66.9863882	63
69	77.8888889	63	74.3333333	63	69.8621712	63	68.354986	63	66.8863882	63
69	78.5555556	63	74.3333333	63	69.0233566	63	68.7186223	63	67.0863882	63
69	78.5555556	63	73.2222222	63	69.0729434	63	68.9913496	63	67.2863882	63
69	78.2222222	63	72.6666667	63	68.6927781	63	68.5368042	63	66.9863882	63
69	77.3333333	63	72.3333333	63	68.601869	63	68.0822587	63	67.1863882	63
69	77.4444444	63	72.5555556	63	68.494431	63	68.1731678	63	66.9863882	63
69	76.8888889	63	72.4444444	63	68.2960839	63	68.6277133	63	66.7863882	63
69	77.3333333	63	72.4444444	63	69.155588	71	67.9913496	63	66.7863882	63
69	77.2222222	63	72.8888889	63	69.4365798	71	67.9955496	63	66.2863882	63
69	77.3333333	63	72.8888889	63	71.1348985	71	66.9913496	63	66.1863882	63
69	77.4444444	63	72.5555556	63	69.9530803	71	66.2640769	63	65.9863882	63
69	77.3333333	63	72	63	69.1348985	71	65.9913496	63	66.1863882	63
69	76.5555556	63	72	63	68.680353	71	64.9913496	63	66.5863882	63
69	75.8888889	63	71	63	68.4985349	71	64.9004405	63	67.2863882	63
69	75.2222222	63	71	63	68.4076258	71	64.354986	63	67.6863882	63
69	74.7789509	63	70.8888889	63	68.3167167	71	64.354986	63	67.3863882	63
69	74.5690744	63	70.7777778	63	68.589444	71	64.8095314	63	66.7863882	63
69	74.3345065	63	71.6666667	63	68.4985349	67	64.9004405	63	66.5863882	63
69	74.3345065	63	71.8888889	63	69.0439894	67	64.8095314	63	66.6863882	63
69	74.4332719	63	72.3333333	63	69.589444	67	64.9004405	63	66.6863882	63
69	75.2851238	63	72.4444444	63	69.7712621	67	64.4458951	63	67.0863882	63
69	76.4444444	63	71.5538123	63	69.8621712	67	64.7186223	63	67.1863882	63
69	76.2222222	63	72.1710963	63	71.2258076	67	64.7186223	63	67.5863882	63
69	76.2222222	63	72.6896148	63	70.9530803	67	64.7186223	63	68.0863882	63
69	75.8888889	63	72.603195	63	70.7712621	69	65.1731678	63	68.6863882	63
69	75.5555556	63	72.7266518	63	71.1348985	69	66.354986	63	69.2863882	63
69	75.1111111	63	73.1217136	63	71.4985349	69	66.7186223	63	69.3863882	63
69	74.7777778	63	73.1464049	63	71.680353	69	66.9004405	63	69.5863882	63
69	74.8888889	63	73.2328247	63	71.7712621	69	67.3388729	63	69.3863882	63
69	75.2222222	63	73.4797382	63	71.589444	69	67.4388729	63	69.2863882	63
69	75.5555556	63	73.2081333	63	71.4076258	69	67.7388729	63	69.7863882	63
69	76.5555556	63	73.5538123	63	71.1348985	69	67.6388729	63	69.7863882	63
69	77.2222222	63	75	63	70.4985349	69	67.9388729	63	69.8863882	63
69	77.2222222	63	75.2222222	63	69.9530803	69	67.7388729	63	70.0863882	63
63	77.4444444	63	75.1111111	63	69.9530803	69	68.1388729	63	69.9863882	63
63	77.3333333	63	75.1111111	63	69.8621712	69	68.9388729	63	67.7024399	63
63	76.8888889	63	75.4444444	63	70.4076258	69	69.0388729	63	67.7024399	63
63	77	63	76.6666667	63	71.0439894	69	68.8388729	63	69.1863882	63
63	76.8888889	63	76.8888889	63	71.3058013	69	68.7388729	63	69.4863882	63
63	76.6666667	63	77	63	72.0058013	69	68.6388729	63	69.4863882	63
63	76.4444444	63	76.6666667	63	72.5058013	69	68.5388729	63	69.3863882	63

63	76.3333333	63	76.5555556	63	72.6058013	69	68.2388729	63	69.2863882	63
63	76.4444444	63	76.1111111	63	72.4058013	69	68.1388729	63	69.3863882	63
63	77.2222222	63	75.7777778	63	72.3058013	69	67.2388729	63	68.7863882	63
63	77.3333333	63	74.6666667	63	72.3058013	69	67.2388729	63	69.0863882	63
63	77.4444444	63	74.1111111	63	72.7058013	69	67.3388729	63	67.7863882	63
63	77.7777778	63	73.1111111	63	72.3058013	69	67.6388729	63	67.4863882	63
63	78	63	72.7777778	63	72.3058013	69	67.9388729	63	67.6863882	63
63	78	63	72.2222222	63	71.8058013	69	68.0388729	63	67.1863882	63
63	77.6666667	63	71.5555556	63	71.8058013	69	68.1388729	63	67.1863882	63
63	76.6666667	63	72	63	71.8058013	69	67.7388729	63	66.9863882	63
63	77.2222222	63	72.3333333	63	71.6058013	69	67.7388729	63	67.0863882	63
63	77.1111111	63	71.4444444	63	71.6058013	69	67.8388729	63	66.7863882	63
63	76.8888889	63	71.7777778	63	71.4058013	69	68.0388729	63	66.6863882	63
63	75.1200136	63	72	63	71.5058013	69	67.4388729	63	66.7863882	63
63	76.8888889	63	72.3333333	63	71.5058013	69	67.1388729	63	66.9863882	63
63	76.8888889	63	73	63	71.6058013	69	67.4388729	63	66.6863882	63
63	77	63	73.2222222	63	71.7058013	69	67.8388729	63	66.4863882	63
63	77.6666667	63	73.5555556	63	72.2058013	69	67.9388729	63	66.3863882	63
63	77.4444444	63	73.7777778	63	71.5058013	69	68.0388729	63	65.9863882	71
63	77.3333333	63	73.4444444	63	71.9058013	69	68.3388729	63	65.7863882	71
63	77.5555556	63	73.2222222	63	72.0058013	69	68.7388729	63	65.5863882	71
63	75.8273818	63	72	63	72.4058013	69	68.7388729	63	65.8863882	71
63	76.0742954	63	71.6666667	63	73.1058013	69	69.0388729	63	65.9863882	71
63	76.0002213	63	71.5555556	63	73.3058013	69	70.0388729	63	66.3863882	71
63	76.012567	63	71.6666667	63	73.4058013	69	70.5388729	63	66.1863882	71
63	76.012567	63	71.6666667	63	74.0058013	69	70.7388729	63	66.5863882	71
63	76.012567	63	71.2222222	63	74.0058013	69	70.9388729	63	66.5863882	71
63	76.049604	63	70.2222222	63	74.5058013	69	71.0388729	63	66.0863882	71
63	76.0619497	63	70.3333333	63	73.0058013	69	70.9388729	63	65.7863882	71
63	76.4570114	63	70.6666667	63	72.8058013	69	70.8388729	63	65.6863882	71
63	76.3459003	63	70.7777778	63	73.1058013	69	71.3388729	63	65.3863882	71
63	76.0866411	63	71	63	73.3058013	69	71.8388729	63	65.1863882	71
69	76.0619497	63	71	63	73.8058013	69	71.6388729	63	64.9863882	71
69	75.666888	63	71	63	73.7058013	69	71.9388729	63	64.5863882	71
69	75.358246	63	70.6666667	63	74.1058013	69	71.8388729	63	64.7863882	71
69	76.3333333	63	70.3333333	63	74.0058013	69	71.9388729	63	65.7863882	71
69	77.2222222	63	68.6554941	63	73.7058013	69	71.7388729	63	66.2863882	71
69	77.5555556	63	68.7912966	63	73.7058013	69	71.6388729	63	66.3863882	71
69	77.2222222	63	69.0382102	63	74.6058013	69	70.9388729	63	66.3863882	71
63	76.3333333	63	69.161667	63	74.9058013	69	71.4388729	63	66.8863882	71
63	76	63	69.3962349	63	75.1058013	69	71.5388729	63	66.8863882	71
63	75.6666667	63	70.7777778	63	75.8058013	69	68.7695188	63	66.3517855	71
63	75.6666667	63	70.2222222	63	75.6058013	69	68.9995188	63	66.4617855	71

63	75.5555556	63	70	63	75.1058013	69	68.8895188	63	66.6917855	71	
63	76.1111111	63	69.8888889	63	74.5058013	69	68.5495188	63	66.4817855	71	
63	76.3333333	63	70.1111111	63	73.8058013	69	70.7388729	63	66.2617855	71	
63	76.2222222	63	70.6666667	63	73.0058013	69	70.2388729	63	66.1517855	71	
63	76.5555556	63	70.4444444	63	72.5058013	69	70.3388729	63	66.7017855	71	
63	76	63	70.3333333	63	72.5058013	69	69.7388729	63	66.4617855	71	
63	75.1111111	63	70	63	72.7058013	69	69.9388729	63	68.2863882	71	
63	75.2222222	63	69.7777778	63	72.8058013	69	69.5388729	63	68.2863882	71	
63	75.4444444	63	70.1111111	63	72.8058013	69	69.1388729	63	65.7917855	71	
63	75	63	70.1111111	63	72.8058013	69	68.8388729	63	64.7017855	71	
63	75.2222222	63	70.1111111	63	72.3058013	69	68.5388729	63	64.3717855	71	
63	74.7777778	63	70.4444444	63	72.1058013	69	68.6388729	63	65.1417855	71	
63	74.6666667	63	70.2222222	63	71.4058013	69	68.4388729	63	64.3076364	71	
63	74.2222222	63	69.8888889	63	71.0058013	69	68.5388729	63	63.6409697	71	
63	74.5555556	63	71	63	70.9058013	69	68.6388729	63	63.3076364	71	
63	74.3333333	63	71.2222222	63	70.6058013	69	69.3388729	63	64.8631919	71	
63	74	63	71.1111111	63	71.2058013	69	68.7388729	63	65.8631919	71	
63	73.8888889	63	71.3333333	63	71.2058013	69	68.5388729	63	67.5298586	71	
63	73.8888889	63	70.4085806	63	71.8058013	69	68.5388729	63	67.6409697	71	
63	73.6666667	63	70.4085806	63	71.8058013	69	68.6388729	63	68.0854142	71	
63	73.3333333	63	70.9024077	63	72.0058013	69	68.5388729	63	68.3076364	71	
63	72.7777778	63	71.1493213	63	72.3058013	69	68.8388729	63	68.7520808	71	
63	72.6666667	63	73.2222222	63	72.4058013	69	68.7388729	63	69.4187475	71	
63	72.6666667	63	73	63	72.2058013	69	69.0388729	63	69.7520808	71	
63	72.8888889	63	71.1493213	63	72.3058013	69	69.0388729	63	69.7520808	71	
63	73.5555556	63	73.1111111	63	72.4058013	69	69.1388729	63	70.1965253	71	
63	74.3333333	63	73.1111111	63	72.9058013	69	69.4388729	63	70.5298586	71	
63	74.7777778	63	73.2222222	63	72.6058013	69	69.7388729	63	70.6409697	71	
63	75.2222222	63	71.9123775	63	72.2058013	69	69.7388729	63	70.3076364	71	
63	73.5325317	63	71.7889207	63	72.0058013	69	69.0388729	63	70.4187475	71	
63	72.7917909	63	71.9494146	63	72.1058013	69	69.1388729	63	69.0854142	71	
63	72.6683341	63	72.2086738	63	71.9058013	69	67.9388729	63	67.9743031	71	
63	72.6683341	63	72.3444763	63	72.2058013	69	67.9388729	63	67.8631919	71	
63	72.6683341	63	72.8506491	63	72.5058013	69	67.8388729	63	67.0854142	71	
63	72.7917909	63	73.2210195	63	72.6058013	69	68.1388729	63	67.3076364	71	
63	71.4461119	63	74.3321306	63	72.5058013	69	68.0388729	63	67.4187475	71	
63	70.5819144	63	74.3321306	63	73.0058013	69	68.4388729	63	67.5298586	71	
63	70.3226551	63	75.7777778	63	72.9058013	69	68.7388729	63	67.6409697	71	
63	70.1991983	63	76.6666667	63	72.7058013	69	69.4388729	63	67.5298586	71	
63	70.9275934	63	77	63	72.9058013	69	70.0388729	63	67.4187475	63	
63	71.4214206	63	77.7777778	63	72.8058013	69	70.6388729	63	67.4187475	63	
63	71.902902	63	78	63	73.0058013	69	69.884762	63	67.6409697	63	
63	74.1111111	63	78.1111111	63	72.6058013	69	70.9958731	63	67.7520808	63	

63	75.1111111	63	78.4444444	63	72.7058013	69	72.2180954	63	67.7520808	63
63	75.1111111	63	78.5555556	63	72.7058013	69	72.884762	63	69.1965253	63
63	75.4444444	63	78.6666667	63	72.9058013	69	74.1069843	63	71.2863882	63
63	76.4444444	63	79	63	73.6058013	69	74.4403176	63	71.6863882	63
63	76.8888889	63	78.7777778	63	73.7058013	69	74.3292065	63	71.4863882	63
63	76.8888889	63	78.8888889	63	74.4058013	69	74.4403176	63	71.5863882	63
63	77.1111111	63	79.2222222	63	74.1058013	69	74.4403176	63	71.0863882	63
63	77.1111111	63	80	63	73.9058013	61	74.6625398	63	70.9863882	63
63	76.7777778	63	80	63	74.3058013	61	74.884762	63	70.8863882	63
63	76.6666667	63	80.4444444	63	74.2058013	61	74.9958731	63	71.0863882	71
63	76.7777778	63	80.5555556	60	74.2058013	60	74.884762	63	71.4863882	71
63	74.9123775	63	79.7777778	60	73.7058013	60	74.884762	63	71.4863882	71
60	75.2827479	63	78.6666667	60	73.5058013	60	74.9958731	60	71.3863882	60

t 3 min S2B Dataset Last 3 min

100114		100114		100314		100314		101314		101314
11:00		10:00		10-11.		11-12.		9-10.		10-11.
17.00		18.00		19.00		20.00		21.00		22.00
67.9925569	63	68.4081496	63	70.1501363	63	70.7097112	63	64.9717932	67	69.6703045
67.1925569	63	68.2333244	63	70.6501363	63	70.4097112	63	65.4003646	67	69.1703045
67.5925569	63	68.2333244	63	70.7751363	63	70.6097112	63	65.2575075	67	69.0453045
67.6925569	63	68.0584992	63	70.5251363	63	70.6097112	63	64.9717932	67	69.5453045
67.7925569	63	68.2543034	63	70.2751363	63	70.8097112	63	64.9717932	67	68.9203045
67.8925569	63	67.7647929	63	70.4001363	63	70.7097112	63	64.9717932	67	68.6703045
67.6925569	63	67.5689887	63	70.5251363	63	70.3097112	63	64.5432218	67	68.6703045
68.3925569	63	66.7857719	63	70.5251363	63	70.2097112	63	64.4003646	67	68.2953045
68.4925569	63	66.2193384	63	71.2751363	63	70.1097112	63	64.5432218	67	68.2953045
68.9925569	63	67.1633943	63	71.5251363	63	70.3097112	63	64.828936	67	68.2953045
69.2925569	63	67.3801775	63	71.4001363	63	70.3097112	63	64.828936	67	68.4203045
69.2925569	63	66.890667	63	71.2751363	63	69.6097112	63	65.6860789	67	68.4203045
69.1925569	63	66.9885691	63	72.1501363	63	69.3097112	63	68.6860789	67	68.2953045
69.4925569	63	66.6948628	63	72.0251363	63	69.0097112	69	68.6860789	67	68.0453045
69.7925569	63	67.890667	63	73.1501363	63	68.2097112	69	68.828936	67	68.2953045
69.8925569	63	67.7927649	63	73.7751363	63	67.8097112	69	68.4003646	67	68.1703045
69.3925569	63	68.1633943	63	74.5251363	63	67.4097112	69	68.4003646	67	68.2953045
69.3925569	63	68.4377767	63	73.9001363	63	67.1097112	69	68.2575075	67	68.7953045
69.9925569	63	68.3303282	63	73.4001363	63	67.0097112	69	67.6860789	67	69.4203045
67.1385314	63	67.7997579	63	73.2751363	63	67.0097112	69	67.6860789	67	67.9618951
67.0985314	63	67.9381144	63	73.0251363	63	67.8097112	69	67.828936	67	68.3837701
66.7985314	63	67.2108417	63	72.4001363	63	67.9097112	69	67.828936	67	66.8993951
67.0385314	63	66.8472053	63	72.4001363	63	68.7097112	69	69.2575075	67	66.7587701
67.0485314	63	66.3926598	63	71.4001363	63	68.4097112	69	69.2575075	67	66.1962701
67.1585314	63	66.1199326	63	69.6501363	63	68.7097112	69	69.2575075	67	66.1962701
67.0485314	63	65.574478	63	68.9001363	63	69.2097112	69	69.5432218	67	67.0400201
67.0485314	63	64.9381144	63	68.2751363	63	69.3097112	69	69.9717932	67	66.6181451
66.9785314	63	65.0290235	63	67.2751363	63	69.4097112	69	70.2575075	67	66.2118951
66.8985314	63	64.3792765	63	67.1501363	63	69.2097112	63	70.2575075	67	65.2275201
66.8085314	63	64.6729828	63	67.4001363	63	69.3097112	63	70.6860789	67	64.6650201
66.9185314	63	65.0645912	63	67.2751363	63	69.2097112	63	71.2575075	67	64.3837701
66.8385314	63	65.5750807	63	67.7751363	63	69.3097112	63	68.5432218	67	63.7587701
66.2885314	63	66.1624933	63	68.0251363	63	70.0097112	63	68.2575075	67	63.9618951
65.3085314	63	66.3582975	63	68.5251363	63	70.4097112	63	67.828936	67	63.6962701
65.0985314	63	66.3582975	63	70.0251363	63	70.3097112	63	67.4003646	67	63.9775201
64.4385314	63	66.1275282	63	70.6501363	63	70.0097112	63	67.4003646	67	64.3368951
64.4385314	63	65.8338219	63	71.1501363	63	69.6097112	63	66.4003646	67	65.0712701
64.2185314	63	65.5191366	63	71.6501363	63	69.6097112	63	65.9717932	67	65.5243951
63.9985314	63	65.3233324	63	72.2751363	63	69.3097112	63	66.4003646	67	66.1025201

64.4285314	63	65.4212345	63	72.6501363	63	69.4097112	63	65.9717932	67	66.3837701
64.2085314	63	65.2603954	63	72.7751363	63	69.2097112	63	65.9717932	67	66.8993951
63.9885314	63	65.4561996	63	72.5251363	63	69.1097112	63	66.9717932	67	67.2118951
64.4085314	63	66.1415142	63	72.1501363	63	69.0097112	63	67.1146503	67	68.0712701
64.8085314	63	66.7562962	63	72.5251363	63	69.4097112	63	67.5432218	67	69.1962701
64.5785314	63	66.6653871	63	72.5251363	63	69.2097112	63	67.2575075	67	69.7587701
64.2385314	63	66.574478	63	72.7751363	63	69.0097112	63	67.5432218	67	70.3993951
64.1285314	63	65.8472053	63	72.2751363	63	69.0097112	63	65.2579204	67	69.6962701
63.7985314	63	66.9381144	63	70.9001363	63	69.2097112	63	65.6252673	67	69.3993951
63.3485314	63	67.4835689	63	70.9001363	63	70.1097112	63	65.0334306	67	68.9775201
63.5685314	63	67.8472053	63	68.4982738	63	70.633115	63	65.4007775	67	68.4150201
63.7785314	63	67.7562962	63	68.2170238	63	70.743115	63	65.5640428	67	68.2743951
64.4285314	63	67.7562962	63	69.7751363	63	70.953115	63	65.0946551	67	68.4150201
64.4285314	63	67.9381144	63	69.5251363	63	70.993115	63	65.2579204	67	68.4150201
64.4185314	63	68.3926598	63	69.5251363	63	71.103115	63	65.2375122	67	68.2743951
64.3085314	63	68.4835689	63	68.9001363	63	70.923115	63	65.1966959	67	68.9775201
64.1985314	63	68.7562962	63	68.9001363	63	70.933115	63	65.1966959	67	69.1181451
64.1985314	63	68.3017507	63	69.1501363	63	70.163115	63	65.1966959	67	69.1337701
64.1885314	63	67.574478	63	69.2751363	63	69.833115	63	65.1762877	67	69.5400201
64.6285314	63	67.0778292	63	68.7751363	63	70.0735976	63	65.3191449	67	69.5556451
64.9585314	63	67.3925145	63	69.0251363	63	70.6908815	63	65.3191449	67	69.4150201
65.0585314	63	68.1199326	63	68.9001363	63	70.8143383	63	65.7043493	67	69.3138547
65.2785314	63	67.8472053	63	68.9001363	63	70.8143383	63	65.4824102	67	68.7492213
65.1685314	63	67.574478	63	69.4001363	63	71.0612519	63	65.4415938	67	69.3429713
64.9385314	63	67.1199326	63	69.5251363	63	70.8143383	63	64.7477163	67	69.6554713
65.2685314	63	66.8472053	63	69.6501363	63	70.6785358	63	63.584451	67	69.6867213
64.9385314	63	67.3926598	63	69.6501363	63	70.0612519	63	66.2575075	67	69.6867213
65.1585314	63	67.574478	63	69.5251363	63	69.7402642	63	63.9722061	67	69.5617213
65.1585314	63	66.7562962	63	69.7751363	63	69.9871778	63	63.6864918	67	69.8429713
64.5885314	63	66.7562962	63	69.7751363	63	69.9871778	63	63.1966959	67	69.7492213
65.4385314	63	66.9381144	63	70.0251363	63	71.4182496	63	63.584451	67	70.3429713
65.4385314	63	66.6653871	63	69.6501363	63	71.5293607	63	64.0742469	67	70.5304713
67.0785314	63	66.5745986	63	69.6501363	63	71.5293607	63	63.9926143	67	70.0304713
67.3985314	63	66.1931528	63	69.6501363	63	70.9738052	63	64.1762877	67	70.1554713
67.1785314	63	66.1199326	63	69.5251363	63	70.9738052	63	63.6456755	67	70.3117213
67.4985314	63	66.8472053	63	69.6501363	63	69.9559497	63	62.9722061	67	70.4367213
67.3885314	63	67.1199326	63	69.9001363	63	67.1781719	63	62.4415938	67	70.5929713
67.3785314	63	66.574478	63	69.9001363	63	66.9312583	63	62.4415938	67	71.0617213
68.9925569	63	66.1199326	63	69.9001363	63	66.4003941	63	62.9109816	67	71.0617213
68.6925569	63	66.2108417	63	69.5251363	63	66.1164435	63	63.4007775	67	71.8742213
66.4685314	63	66.3017507	63	69.5251363	63	66.2275546	63	64.7069	67	72.4992213
68.6925569	63	65.3926598	63	69.1501363	63	66.215209	63	64.7069	67	72.4367213
68.4925569	63	65.0290235	63	69.0251363	63	65.9682954	63	64.5640428	67	74.4150201

68.4925569	63	64.7562962	63	69.2751363	63	65.9682954	63	64.4007775	67	74.1337701
68.3925569	63	64.6653871	63	69.4001363	63	66.0794065	63	64.584451	67	73.7275201
68.1925569	63	64.6653871	63	69.5251363	63	66.3263201	63	65.0946551	67	74.1650201
68.3925569	63	65.574478	63	69.6501363	63	65.8201472	63	65.584451	67	73.8837701
69.1925569	63	65.9381144	63	69.9001363	63	66.0670608	63	65.7681245	67	73.8837701
69.5925569	63	66.3926598	63	70.4001363	63	68.9131037	63	65.9313898	67	73.7431451
70.0925569	63	67.0290235	63	70.1501363	63	68.5427334	63	65.9313898	67	74.2900201
68.9925569	63	66.6653871	63	70.5251363	63	68.172363	63	66.2579204	67	73.3056451
68.9925569	63	67.3926598	63	71.0251363	63	67.8143383	63	66.6048592	67	72.8681451
68.6925569	63	67.6653871	63	71.5251363	63	66.5303877	63	66.9313898	67	72.4306451
68.7925569	63	67.1607359	63	71.7751363	63	67.0242149	63	66.9517979	67	71.5868951
68.8925569	63	67.6152814	63	71.9001363	63	66.7773013	63	67.6048592	67	71.4462701
68.8925569	63	67.3215751	63	71.6501363	63	67.1106346	63	68.0946551	67	71.6025201
68.7925569	63	67.1677289	63	71.2751363	63	66.863721	63	67.1150632	67	71.6025201
68.8925569	63	66.6012954	63	69.7416829	63	68.6404718	63	66.7477163	67	71.9150201
68.9925569	63	66.4264702	63	69.7416829	63	68.5293607	63	67.0742469	67	71.9306451
69.2925569	63	66.4264702	63	68.9604329	63	68.6404718	63	66.7885326	67	71.5087701
69.9925569	63	66.3635331	63	69.2885579	63	68.7515829	63	66.9722061	67	71.3681451
69.9925569	63	66.6572394	63	69.1791829	63	68.4182496	63	67.2987367	67	71.3681451
70.1925569	63	67.223673	63	69.1791829	63	68.3071385	63	67.4415938	67	71.5087701
70.2925569	63	67.5733233	63	70.2729329	63	68.0849163	63	67.6048592	67	71.9306451
71.0925569	63	68.8472053	63	70.5229329	63	68.1960274	63	68.0946551	67	72.0400201
70.9925569	63	69.1199326	63	70.7729329	63	68.3071385	63	68.7477163	67	71.9462701
71.4925569	63	69.3017507	63	71.6501363	63	68.3071385	63	68.5640428	67	71.9462701
71.4925569	63	69.3926598	63	71.7751363	63	67.9738052	63	69.0538387	67	71.8525201
71.4925569	63	70.1199326	63	70.7751363	63	67.6404718	63	69.0130224	67	71.6962701
71.1925569	63	70.0290235	63	70.7751363	63	67.3071385	63	69.1762877	67	72.3993951
70.9925569	63	66.3652905	63	71.1501363	63	67.0849163	63	68.8497571	67	69.9775201
71.1925569	63	66.1694863	63	72.4001363	63	67.4182496	63	68.8497571	67	69.8368951
71.2925569	63	66.0925632	63	73.0251363	63	68.4182496	63	67.7069	67	71.7953045
70.4925569	63	66.0715842	63	74.0251363	63	68.6404718	63	67.5436347	67	71.5453045
69.7925569	63	66.1694863	63	74.0251363	63	68.7515829	63	67.7330164	67	71.6703045
69.8925569	63	66.3233324	63	74.1501363	63	69.0849163	63	66.0742469	67	71.6703045
69.9925569	63	67.9381144	63	74.1501363	63	69.5293607	63	65.584451	67	69.4865334
69.8925569	63	67.8472053	63	73.5251363	63	69.5293607	63	65.2579204	67	72.2953045
70.1925569	63	67.8472053	63	73.7751363	63	70.1960274	63	65.0946551	67	72.6703045
68.5035923	63	67.9381144	63	73.4001363	63	70.0849163	61	64.7681245	67	72.7953045
66.5796321	63	67.7562962	63	73.1501363	63	70.7515829	61	65.2375122	67	72.9203045
66.5196321	63	67.574478	63	73.4001363	63	71.1960274	61	65.0538387	67	73.1703045
66.0396321	63	67.8472053	63	73.4001363	63	71.6404718	61	65.6864918	67	72.4203045
65.7196321	63	68.0290235	63	73.4001363	63	71.3071385	61	66.0130224	67	72.2953045
64.8796321	63	68.3926598	63	72.9001363	63	71.0849163	61	66.1762877	67	72.9203045
64.5196321	63	68.574478	63	72.6501363	63	71.3071385	61	66.5232265	67	73.1703045

64.7596321	63	68.4097438	63	72.2751363	63	71.4182496	61	66.5232265	67	73.2953045
65.1196321	63	67.9889752	63	71.9001363	63	71.6404718	61	66.7069	67	73.0453045
65.2396321	63	68.574478	63	71.7751363	63	71.6404718	61	66.5028183	67	73.4203045
65.5796321	63	69.6653871	63	71.6501363	63	71.9738052	61	66.3599612	67	73.4203045
65.9396321	63	69.6653871	63	71.2751363	63	72.1960274	61	66.5232265	67	73.5453045
65.6996321	63	69.6513219	63	71.2751363	63	72.862694	61	66.9109816	67	73.7953045
65.2596321	63	72.0290235	63	71.4001363	63	72.9738052	61	66.584451	67	74.0453045
65.6996321	63	72.574478	63	71.7751363	63	72.862694	61	66.7885326	67	73.5453045
65.9996321	63	72.6653871	63	71.9001363	63	73.1960274	61	66.9517979	67	73.4203045
67.2535923	63	72.7562962	63	72.0251363	63	73.3071385	61	67.2783285	67	73.2953045
66.9235923	63	73.0290235	63	72.0251363	63	73.3071385	61	67.2987367	67	73.0453045
66.0435923	63	71.2806925	63	71.7751363	63	73.0849163	61	66.9722061	67	73.2953045
66.0335923	63	71.0848883	63	72.6501363	63	73.1960274	61	66.9722061	67	73.2953045
66.1435923	63	70.2037694	63	72.4001363	63	73.1960274	61	66.3191449	67	73.4203045
65.9335923	63	69.5534198	63	72.4001363	63	73.3071385	61	65.5028183	67	73.5453045
65.6035923	63	67.1773011	63	72.1501363	63	70.8201472	61	65.1966959	67	73.1703045
65.5235923	63	66.5479305	63	71.4001363	63	70.2028633	61	65.0130224	67	73.2953045
65.7435923	63	66.5968815	63	71.1501363	63	70.1960274	61	64.3191449	67	71.3837701
66.0735923	63	68.6808768	63	70.7751363	63	69.6404718	61	64.3191449	67	71.3837701
66.7335923	63	68.7018559	63	70.1501363	63	69.7515829	61	63.9926143	67	71.8212701
67.2935923	63	68.5270307	63	69.7751363	63	69.6404718	61	62.9926143	67	71.8368951
67.8435923	63	68.3522055	63	69.9001363	63	69.7515829	61	63.2987367	67	72.1650201
68.1735923	63	68.3312265	63	69.5251363	63	69.7515829	61	63.462002	67	72.7587701
68.4935923	63	67.9396181	63	69.6501363	63	69.7515829	61	63.2579204	67	73.4618951
68.4535923	63	67.5270307	63	69.4001363	63	69.7515829	61	63.2579204	67	74.1650201
68.5435923	63	67.3312265	63	69.4001363	63	69.6404718	61	62.9722061	67	74.2743951
70.1925569	63	68.3926598	63	69.5251363	63	69.4182496	61	62.462002	67	74.2743951
69.7925569	63	67.574478	63	70.1501363	63	69.1960274	61	62.462002	67	74.1337701
69.8925569	63	67.3017507	63	70.2751363	63	68.8360716	61	62.6252673	67	74.4306451
69.8925569	63	67.1199326	63	70.4001363	63	68.7110716	61	62.1354714	67	74.0400201
69.5925569	63	66.9381144	63	71.0251363	63	65.8808195	61	61.6456755	67	73.9462701
69.4925569	63	66.9381144	63	71.4001363	63	65.1776945	61	61.3191449	67	73.5556451
69.9925569	63	66.3926598	63	70.9001363	63	64.9120695	61	61.1762877	67	72.3056451
69.9925569	63	66.2108417	63	70.4001363	63	65.0526945	61	60.8497571	67	72.0556451
70.4925569	63	66.4835689	63	70.0251363	63	65.5995695	61	61.0130224	67	72.0556451
70.4925569	63	66.574478	63	70.2751363	63	68.5860716	61	61.0130224	67	72.7587701
70.4925569	63	66.2108417	63	70.4001363	63	68.7110716	61	61.3191449	67	72.4931451
70.5925569	63	65.9381144	63	70.6501363	63	70.7110716	61	61.4824102	67	72.2118951
70.7925569	63	65.9381144	63	70.6501363	63	71.5860716	61	61.6456755	67	72.0712701
70.5925569	63	66.4835689	63	70.6501363	63	71.7110716	61	61.6252673	67	71.9462701
70.8925569	63	68.0290235	63	70.6501363	63	71.9610716	61	60.9722061	67	72.0868951
70.2925569	63	67.0515062	63	70.5251363	63	72.0860716	61	60.7681245	67	71.6650201
69.5925569	63	67.3452125	63	70.7751363	63	69.9827953	61	60.7681245	67	70.5400201

68.8925569	63	68.3926598	63	71.6501363	63	69.9984203	61	62.828936	67	69.7240186
68.6925569	63	68.7562962	63	71.6501363	63	72.0860716	61	62.828936	67	70.3837701
68.5925569	63	68.7562962	63	71.7751363	63	72.3360716	61	63.2575075	67	71.0868951
68.6925569	63	68.8472053	63	72.2751363	63	72.0860716	61	63.828936	67	70.9462701
67.6925569	63	68.8472053	63	72.4001363	63	72.5860716	61	63.9717932	67	70.6650201
67.2925569	63	69.2108417	63	72.6501363	63	72.5860716	61	64.5432218	67	70.5243951
67.2925569	63	69.2108417	63	72.7751363	63	72.3360716	61	64.5432218	67	70.1025201
67.2925569	63	69.4835689	63	72.7751363	63	72.2110716	61	64.9717932	67	70.2275201
65.4398382	63	69.1199326	63	72.7751363	63	71.9610716	61	65.1146503	67	70.3681451
65.2398382	63	68.4835689	63	72.4001363	63	71.8360716	61	65.6860789	67	70.3681451
65.0598382	63	68.3926598	63	73.2751363	63	72.3360716	61	66.2575075	67	70.6650201
64.6398382	63	68.3185049	63	73.4001363	63	72.2110716	61	69.4003646	67	70.3993951
64.5198382	63	68.0053678	63	73.6501363	63	72.6522851	61	68.0946551	67	70.1181451

S2B Dataset Last 3 min

	101514		101614		101714		102014		102014	
	9-1030.		9-10.		9-1030.		1:00		10-11.	
	23.00		24.00		25.00		26.00		27.00	
71	73.041958	71	66.375	63	69.2437021	63	66.9326031	69	66.8000474	63
63	73.9308469	71	66	63	69.2437021	63	66.9326031	69	66.6000474	63
63	74.1530691	71	66.125	63	68.8437021	63	67.3701031	69	66.4900474	63
63	74.5975136	63	64.5059463	63	68.5437021	63	67.7919781	69	65.9400474	63
63	75.041958	63	66	63	68.7437021	63	68.9169781	69	65.8300474	63
63	75.1530691	63	66.125	63	69.1437021	63	69.7607281	69	66.0500474	63
63	75.4864025	63	67.625	63	69.7437021	63	69.9013531	69	66.2700474	63
63	75.7086247	63	67.0601791	63	69.8437021	63	70.0419781	69	66.4900474	63
63	75.9308469	63	67.2008041	63	69.9437021	63	69.7451031	69	66.8200474	63
63	75.9308469	63	68.625	63	69.7437021	63	69.4638531	69	67.1500474	63
63	76.2641802	63	68.625	63	69.5437021	63	68.9013531	69	67.4700474	63
63	76.041958	63	68.125	63	68.8437021	63	70.955669	69	67.6800474	63
63	75.4864025	63	68	63	68.4437021	63	70.830669	69	67.6800474	63
63	75.1530691	63	67.625	63	68.0437021	63	69.830669	69	67.7900474	63
63	75.1530691	63	66.625	63	67.9437021	63	70.080669	69	68.0100474	63
63	75.041958	63	66.5	63	67.3437021	63	70.080669	69	68.3400474	63
63	74.3752914	63	66.5	63	67.5437021	63	69.830669	69	68.5600474	63
63	73.9308469	63	66.375	63	67.2437021	63	69.205669	69	68.7800474	63
63	73.9308469	63	65.875	63	67.3437021	63	68.580669	69	68.6700474	63
63	73.7086247	63	66.625	63	67.6437021	63	68.955669	69	68.6466491	63
63	73.4864025	63	66.75	63	67.9437021	63	68.455669	69	69.3400474	63
63	73.4864025	63	67	63	67.9437021	63	68.205669	69	69.3400474	63
63	74.041958	63	67.125	63	67.9437021	63	67.080669	69	69.9000474	63
63	73.9308469	63	67.5	63	67.6437021	63	66.455669	69	70.3400474	63
63	73.9308469	63	66.125	63	67.6437021	63	66.705669	69	70.4500474	63
63	73.3752914	63	65.75	63	67.6437021	63	66.580669	69	70.5600474	63
63	73.4864025	63	65.625	63	67.9437021	63	66.955669	69	69.9000474	63
63	73.5975136	63	65.375	63	68.5437021	63	66.705669	69	69.1300474	63
63	73.3752914	63	65.25	63	68.3437021	63	66.705669	69	68.9100474	63
63	73.2641802	63	64.5	63	68.4437021	63	66.205669	69	68.9100474	63
63	73.5975136	63	64.125	63	68.7437021	63	65.705669	69	68.9100474	63
63	73.2641802	63	64	63	68.5437021	63	65.330669	69	68.2500474	63
63	73.3752914	63	63.875	63	68.5437021	63	65.205669	69	68.5800474	63
63	73.5975136	63	64	63	68.6437021	63	65.205669	69	68.1400474	63
63	72.8197358	63	64.25	63	68.4437021	63	64.330669	69	67.9200474	63
63	73.7086247	63	65	63	68.4437021	63	64.455669	69	67.2600474	63
63	74.8197358	63	65.25	63	68.2437021	63	65.205669	69	67.3700474	63
63	75.8197358	63	65.25	63	68.4437021	63	65.455669	69	67.2700474	63
63	75.7086247	63	65	63	68.9437021	63	65.330669	69	67.8200474	63

63	75.4864025	63	65.875	63	69.2437021	63	65.205669	69	68.0400474	63
63	74.3752914	63	62.8498576	63	69.2437021	63	65.330669	69	68.1500474	63
63	73.3752914	63	63.6876613	63	69.5343684	63	65.330669	69	68.0300474	63
63	72.9308469	63	64.0001613	63	69.5437021	63	65.205669	69	67.9200474	63
63	73.3752914	63	64.0939113	63	69.3437021	63	64.830669	69	67.8100474	63
63	74.2641802	63	64.0939113	63	69.0437021	63	64.830669	69	67.7000474	63
63	73.8197358	63	63.3216129	63	68.7437021	63	64.955669	69	67.1500474	63
63	73.4864025	63	63.7189113	63	69.1437021	63	65.705669	69	67.2600474	63
63	73.4864025	63	63.8751613	63	69.7437021	63	66.705669	69	67.8000474	63
63	73.1530691	63	63.8751613	63	69.9437021	63	66.830669	69	67.6900474	63
63	72.5975136	63	64.8439113	63	70.2437021	63	66.455669	69	67.4600474	63
63	72.3752914	63	65.2189113	63	70.7437021	63	66.080669	69	67.1200474	63
63	71.9308469	63	65.5001613	63	70.5437021	63	66.080669	69	66.8700474	63
63	72.041958	63	65.0782863	63	70.3437021	63	65.330669	69	67.6412072	63
63	72.2641802	63	65.6095363	63	70.2437021	63	65.455669	69	67.2412072	63
63	72.8197358	63	65.7657863	63	69.6437021	63	65.330669	69	67.5412072	63
63	73.9308469	63	65.7501613	63	69.5437021	63	65.080669	69	67.1412072	63
63	74.3752914	63	65.7501613	63	69.6437021	63	65.330669	69	67.0412072	63
63	74.3752914	71	66.1564113	63	69.1437021	63	65.205669	69	67.2412072	63
63	74.3752914	71	66.4220363	63	68.7437021	63	65.330669	69	68.0412072	63
63	74.7086247	71	67.2657863	63	68.9437021	63	65.455669	69	66.4723162	63
63	75.041958	71	67.2657863	63	69.5437021	63	65.830669	69	65.21697	63
63	75.3752914	71	67.8282863	63	69.2437021	63	66.830669	69	65.995861	63
63	73.8011299	71	67.9689113	63	69.2437021	63	67.080669	69	66.105861	63
63	73.5511299	71	68.5314113	63	69.0437021	63	67.705669	63	63.51697	63
63	73.8011299	71	68.3907863	63	68.7437021	63	67.705669	63	63.57697	63
63	74.3011299	71	68.5314113	63	68.9437021	63	67.580669	63	65.155861	63
63	74.1761299	71	68.1095363	63	67.8437021	63	67.455669	63	63.59697	63
63	74.1761299	71	67.9532863	63	67.7437021	63	67.455669	63	63.63697	63
63	74.3011299	71	70.125	63	67.6437021	63	68.080669	63	63.65697	63
63	74.8011299	71	70.75	63	67.8437021	63	67.830669	63	65.2823162	63
63	74.6761299	71	69.610935	63	68.2437021	63	67.830669	63	65.0623162	63
63	74.9261299	71	69.610935	63	67.3437021	63	67.830669	63	64.9523162	63
63	75.1761299	71	69.62656	63	67.2437021	63	67.580669	63	65.0623162	63
63	75.9261299	71	71.5	63	67.6437021	63	67.830669	63	64.9595	63
63	75.5511299	71	71.625	63	67.6437021	63	67.580669	63	64.6963968	63
63	75.5511299	71	71.75	63	67.9437021	63	67.455669	63	64.6223162	63
63	73.4261299	71	71.5	63	68.2437021	63	67.455669	63	64.7323162	71
63	73.3011299	71	71.875	63	68.2437021	63	67.455669	63	64.4123162	71
63	72.5511299	71	71.375	63	68.4437021	63	67.080669	63	64.8523162	71
63	72.5511299	71	71.25	63	68.8437021	63	66.830669	63	65.1823162	71
63	71.8011299	71	71.125	63	69.2437021	63	67.330669	63	65.6223162	71
63	71.1761299	71	70.875	63	69.3437021	63	67.580669	63	65.5923162	71

63	70.9261299	71	70.75	63	70.3437021	63	70.205669	63	66.7412072	71
63	71.3011299	71	70.625	63	69.1437021	63	71.580669	63	66.3412072	71
63	69.1557797	71	70.375	63	68.8437021	63	72.705669	63	66.0412072	71
63	69.2946686	71	70.125	63	68.9437021	63	72.580669	63	66.5412072	71
63	69.2113352	71	69.375	63	69.8437021	63	72.455669	63	66.7412072	71
63	68.3780019	71	69	63	70.2437021	63	72.330669	63	67.4412072	71
63	68.739113	71	68.75	63	70.3437021	63	71.955669	63	67.7412072	71
63	68.8780019	71	68.875	63	71.0437021	63	71.205669	63	67.8412072	71
63	68.6002241	71	68.625	63	71.2437021	63	70.205669	63	68.1412072	71
63	68.6280019	71	68.25	63	71.5437021	63	69.830669	63	67.9412072	71
63	69.8780019	71	68.5	63	71.9437021	63	69.830669	63	67.6412072	71
63	70.4335574	71	68.5	63	72.0437021	63	69.580669	63	67.1412072	71
63	70.2946686	71	68.625	63	70.1939594	63	70.330669	63	67.1412072	71
63	70.1557797	71	68.25	63	72.2550582	63	70.330669	63	67.1412072	71
63	70.4335574	71	68.25	71	72.5437021	63	70.705669	63	67.2412072	71
63	70.4335574	71	69.125	71	72.1437021	63	70.455669	63	66.6412072	71
63	70.6835574	71	69.125	71	72.2437021	63	70.580669	63	66.3412072	71
63	71.0446686	71	68.2515996	71	72.3437021	63	70.830669	63	66.0412072	71
63	72.6761299	71	68.2984746	71	72.2437021	63	69.740402	63	66.3412072	71
63	73.4261299	71	68.8609746	71	72.7437021	63	70.052902	63	66.5412072	71
63	73.8011299	71	68.9390996	71	72.4559197	63	70.349777	63	67.9412072	71
63	73.1761299	71	69.4234746	71	73.0114753	63	70.349777	63	68.3412072	71
63	73.3011299	71	69.7203496	71	73.5670308	63	69.912277	63	68.7412072	71
63	72.5511299	71	69.6890996	71	73.2336975	63	69.771652	63	68.7412072	71
63	72.6761299	71	68.8140996	71	72.7892531	63	70.052902	63	69.8412072	71
63	73.0511299	71	68.0640996	71	73.0114753	63	70.615402	63	70.0412072	71
63	73.4261299	63	67.7672246	71	72.7892531	63	70.615402	63	69.8412072	71
63	73.3011299	63	67.2047246	71	72.7892531	63	70.052902	63	69.8412072	71
63	72.9261299	63	67.0640996	71	71.5670308	63	69.771652	63	69.9412072	71
63	72.9261299	63	67.2203496	71	70.4559197	63	69.615402	63	69.8412072	71
63	73.8011299	63	67.7828496	71	69.6781419	63	69.474777	63	69.8412072	71
63	74.0511299	63	68.0640996	71	68.5670308	63	68.177902	63	69.9412072	71
63	74.0511299	63	68.5015996	71	68.4559197	63	67.740402	63	68.2001549	71
63	74.8011299	63	68.5328496	71	67.3448086	63	68.955669	63	67.0201549	71
63	74.3011299	63	68.2984746	71	67.1225864	63	68.205669	63	66.4901549	71
63	73.8011299	63	70.2047246	71	67.3430467	63	67.080669	63	66.7201549	71
63	73.9261299	63	70.1422246	71	67.4601179	63	67.080669	63	66.6101549	71
63	73.9261299	63	69.8765996	71	67.8351179	63	66.955669	63	66.8301549	71
63	74.1761299	63	69.2984746	71	69.2101179	63	66.955669	63	66.7401549	71
63	73.9261299	63	68.9547246	71	69.5851179	63	67.455669	63	66.4101549	71
63	73.3011299	63	68.1422246	71	69.8351179	63	67.955669	63	66.1901549	71
63	73.4261299	63	67.8609746	71	69.7101179	63	68.205669	63	66.2301549	69
63	73.1761299	63	67.4390996	71	70.2101179	63	67.830669	63	66.6701549	69

63	73.0511299	63	67.1265996	71	70.4601179	63	66.955669	63	67.3301549	69
63	72.8011299	63	67.0953496	71	69.9601179	63	67.080669	63	67.8801549	69
75	72.5511299	63	67.4078496	71	70.7190065	63	67.080669	63	67.8066506	69
75	72.5511299	63	67.4078496	71	71.1475779	63	67.080669	63	69.4865165	69
75	72.5360259	71	68.75	71	71.7190065	63	66.705669	63	69.2001549	69
75	70.9261299	71	69	71	71.8618636	63	66.580669	63	69.6401549	69
75	70.6761299	71	69	71	71.2904351	63	66.330669	63	69.2001549	69
75	72.0511299	71	69.125	71	71.5761494	63	66.080669	63	69.5301549	69
75	72.4261299	71	68	71	71.1475779	63	66.080669	63	69.9701549	69
75	73.6761299	71	67	71	70.3714177	63	65.955669	63	69.8301549	69
75	74.3011299	71	66.375	71	70.204751	63	66.080669	63	69.9401549	69
75	74.8011299	71	66.125	71	70.204751	63	66.205669	63	70.0501549	69
75	75.1761299	71	65.875	71	70.0380843	63	66.330669	63	70.5901549	69
75	75.0511299	71	66.25	71	69.704751	63	66.455669	63	71.1201549	69
75	75.3011299	71	66.75	71	69.5380843	63	66.705669	63	71.0101549	69
75	75.3011299	71	67.625	71	70.3714177	63	66.955669	63	73.1412072	69
75	74.9261299	71	68	71	70.5380843	63	66.580669	63	73.8412072	69
75	75.1761299	71	69	71	70.5380843	63	66.580669	63	74.2412072	69
75	75.4261299	71	69.25	71	69.8714177	63	67.205669	63	74.1412072	69
75	75.5511299	71	69	71	70.204751	63	67.080669	63	74.2412072	69
75	75.5511299	71	69	71	69.8714177	63	66.955669	63	74.0412072	69
75	74.5511299	71	69.125	71	69.8714177	63	66.955669	63	73.6412072	69
75	73.6761299	71	69.25	71	69.8714177	63	67.205669	63	72.4412072	69
75	73.4261299	71	69.25	71	70.0950555	63	66.830669	63	72.7412072	69
75	72.9261299	71	69	71	70.3714177	63	66.830669	63	72.2412072	69
75	72.3011299	71	69.125	71	70.5380843	63	66.705669	63	70.108462	69
75	72.3011299	71	69.625	71	70.8714177	61	67.580669	63	69.678462	69
75	71.9261299	71	69.625	71	70.704751	61	67.705669	63	68.9731158	69
75	71.6761299	71	69.625	71	71.204751	61	68.0474893	63	68.6131158	69
75	71.6761299	71	69.875	71	71.704751	61	68.1903465	63	68.8531158	69
75	72.5511299	71	70	71	71.704751	61	68.3332036	63	70.435861	69
75	72.8011299	71	69.5	71	72.704751	61	68.6189179	63	68.8543227	69
75	72.3011299	71	69	71	72.5380843	61	68.6189179	63	68.4943227	69
75	71.8011299	71	68.875	71	73.5380843	61	69.4760608	63	68.4943227	69
75	71.6761299	71	68.5	71	74.0380843	69	69.3332036	63	69.0943227	69
75	71.4261299	71	68.25	71	75.8714177	69	69.9046322	63	69.5743227	69
75	71.3011299	71	67.5	71	75.8714177	69	70.4760608	63	69.6943227	69
75	71.1761299	71	67	71	75.8714177	69	70.3332036	63	70.895861	69
75	71.5511299	71	67.125	71	75.704751	69	70.761775	63	69.795861	69
75	71.8011299	71	66.875	71	74.8714177	69	70.3332036	63	69.685861	69
75	70.4301818	71	66.75	71	74.704751	69	70.9046322	63	69.245861	69
75	70.652404	71	66.625	71	74.5380843	69	71.0474893	63	69.245861	69
75	70.3746262	71	66.875	71	74.704751	69	70.0930789	63	68.805861	69

75	69.902404	71	67	71	74.704751	69	70.2597456	63	68.695861	69
75	69.7635151	71	67	71	75.204751	69	70.5930789	63	69.245861	69
75	69.7635151	71	67.125	71	75.3714177	69	70.4264122	63	69.135861	69
75	69.0690706	71	66.875	71	75.5380843	69	70.4264122	63	70.235861	69
75	67.9579595	71	67.125	71	75.5380843	69	70.4264122	63	70.675861	69
75	67.5412929	71	67.125	71	76.0380843	69	70.5930789	63	70.675861	69
75	67.5412929	71	67.125	71	75.704751	69	69.9264122	63	70.675861	69
75	67.9579595	71	67.625	71	75.204751	69	69.7597456	63	70.345861	69
75	68.3746262	71	68	71	78.5491162	69	69.861725	63	69.575861	69
75	67.8190706	71	67.875	71	77.1491162	69	71.261725	63	69.135861	69
75	67.7079595	71	67.5	71	76.3491162	69	72.261725	63	69.245861	69
75	67.8468484	71	67.5	71	76.1491162	69	73.261725	63	68.915861	69
75	67.8468484	71	67.25	71	76.1491162	69	74.261725	60	67.1625866	69

S2B Dataset Last 3 min

102014		102014		102114		102114		102214		102214
11-12.		9:00		9-10.		10-11.		9-10.		10-11.
28.00		29.00		30.00		31.00		32.00		33.00
62.9652692	63	71.4523292	63	65.4259273	63	62.5791422	71	66.7155498	63	63.9027222
63.2379965	63	71.4523292	63	65.2092606	63	62.2978922	71	66.6044387	63	63.9027222
63.6016329	63	71.4523292	63	64.8842606	71	62.1572672	71	66.3698708	63	63.7777222
63.8743601	63	71.5523292	63	65.3585601	71	64.4895621	71	66.0118461	63	63.7777222
63.7834511	63	72.0523292	63	64.8585601	71	64.9895621	71	65.8883893	63	63.4027222
63.692542	63	72.5523292	63	64.8585601	71	64.9895621	71	65.283451	63	63.4027222
64.2379965	63	72.6523292	63	65.2585601	71	65.8645621	71	65.5303646	63	63.4027222
64.0561783	63	72.5523292	63	65.1585601	63	65.7395621	71	65.1229572	63	62.7777222
63.692542	63	72.9523292	63	65.4585601	63	64.5169351	71	64.8019695	63	63.1527222
63.7834511	63	73.8523292	63	65.5585601	63	66.6145621	63	64.555056	63	63.7777222
63.8743601	63	73.8523292	63	65.7585601	63	65.3645621	63	64.4192535	63	63.9027222
64.1470874	63	73.7523292	63	65.9585601	63	64.7395621	63	64.283451	63	64.5277222
63.8743601	63	73.8523292	63	66.4585601	63	64.6145621	63	64.3822165	63	64.7777222
63.7834511	63	73.8523292	63	66.6585601	63	64.6145621	63	64.1229572	63	65.1527222
64.2379965	63	73.6523292	63	66.4585601	63	64.3645621	63	63.9871547	63	66.7777222
64.3289056	63	73.2523292	63	66.7585601	63	64.4895621	63	64.4809819	63	66.9027222
63.9652692	63	73.0523292	63	66.9585601	63	64.4895621	63	64.2217226	63	66.7777222
63.692542	63	72.5523292	63	66.9585601	63	64.2395621	63	63.6167844	63	66.7777222
64.0561783	63	71.5523292	63	67.0573697	63	64.6145621	63	63.8760436	63	66.7777222
63.9652692	63	71.5523292	63	66.9585601	63	64.9895621	63	64.1229572	63	66.4027222
64.1470874	63	70.7523292	63	66.3585601	63	65.1145621	63	64.4933276	63	64.2028292
64.4198147	63	70.2523292	63	66.2585601	63	65.3645621	63	64.62913	63	64.3434542
66.2379965	63	70.3523292	63	66.1585601	63	65.6145621	63	64.6538214	63	63.7965792
66.2379965	63	70.3523292	63	66.2585601	63	65.7395621	63	66.2330703	63	63.5153292
66.3289056	63	70.2523292	63	66.3585601	63	65.7395621	63	65.7886259	63	63.5153292
66.3289056	63	70.2523292	63	66.0585601	63	65.9895621	63	65.3441814	63	63.5153292
64.8743601	63	70.2523292	63	65.9585601	63	65.4895621	63	65.7886259	63	62.5465792
64.9652692	63	70.9523292	63	65.8585601	63	65.3645621	63	66.3441814	63	62.9528292
64.7834511	63	71.1523292	63	65.6585601	63	64.4895621	63	66.4552926	71	63.2340792
63.9652692	63	70.9523292	63	64.8585601	63	63.4895621	63	66.6775148	71	64.2184542
63.8743601	63	70.5523292	63	65.3585601	63	63.4895621	63	66.7886259	71	65.7653292
63.7834511	63	70.3523292	63	65.0585601	63	62.8645621	63	67.1219592	71	67.9027222
62.3045166	63	69.9523292	63	65.1585601	63	63.1145621	63	67.3441814	71	66.0314159
62.205343	63	69.7523292	63	64.9585601	63	63.2395621	63	67.6775148	71	66.3171302
61.4119546	63	69.6523292	63	64.6585601	63	62.9895621	63	67.7886259	71	65.1742731
63.3289056	63	69.3523292	63	64.6585601	63	62.7395621	63	67.4552926	71	64.7457016
63.3289056	63	69.3523292	63	64.6585601	63	62.8645621	63	67.4552926	63	64.7457016
63.4198147	63	69.2523292	63	64.0585601	63	63.1145621	63	67.1219592	63	64.8885588
63.2379965	63	68.6523292	63	64.4585601	63	63.7395621	63	67.0108481	63	64.6028445

63.2379965	63	68.2523292	63	64.9585601	63	63.4895621	63	66.2330703	63	64.7457016
63.8743601	63	68.0523292	63	64.9585601	63	63.3645621	63	66.0108481	63	64.4599873
63.6016329	63	67.6523292	63	65.0585601	63	63.2395621	63	65.899737	63	63.6028445
64.1470874	63	67.6523292	63	65.4585601	63	63.2395621	63	66.2330703	63	63.8885588
64.4198147	63	67.4523292	63	66.1585601	63	62.2395621	63	66.0108481	63	63.7457016
64.5107238	63	67.1523292	63	66.0585601	63	62.6145621	63	66.2330703	63	63.6028445
64.4198147	63	67.1523292	63	65.8585601	63	62.7395621	63	66.3441814	63	63.4599873
64.7834511	63	67.2523292	63	65.8585601	63	63.8645621	63	66.2330703	63	62.4599873
65.2379965	63	67.6523292	63	65.8585601	63	64.4895621	63	65.6775148	63	62.4599873
65.5107238	63	67.9523292	63	65.8585601	63	64.7395621	63	65.3441814	63	62.3171302
65.692542	63	68.5523292	63	66.0585601	63	65.2395621	63	65.0108481	63	61.3171302
65.7834511	63	68.9523292	63	66.2585601	63	64.9895621	63	65.2330703	63	61.4599873
66.0561783	63	68.9523292	63	66.7585601	63	65.1145621	63	65.4552926	63	61.4599873
65.692542	63	68.6523292	63	66.9585601	63	65.3645621	63	65.4552926	63	62.0314159
65.8743601	63	68.7523292	63	67.2585601	63	65.8645621	63	65.1219592	63	61.8885588
66.0561783	63	69.4523292	63	67.0585601	63	65.9895621	63	64.7886259	63	62.4599873
65.7834511	63	69.9523292	63	67.4585601	63	65.9895621	63	65.4552926	63	63.1742731
65.7834511	63	69.4523292	63	67.5585601	63	65.4895621	63	65.6775148	63	63.6028445
65.7834511	63	68.9523292	63	67.1585601	63	65.2395621	63	66.1219592	63	64.4599873
66.2379965	63	68.9523292	63	67.8585601	63	65.3645621	63	66.3441814	63	64.6028445
66.0561783	63	68.6523292	63	66.9585601	63	65.9895621	63	65.4552926	63	64.4599873
65.0561783	63	68.7523292	63	67.3585601	63	65.9895621	63	65.5664037	63	64.6028445
64.4198147	63	69.0523292	63	66.6585601	63	66.1145621	63	65.0108481	63	64.6028445
64.4198147	63	69.5523292	63	66.5585601	63	65.3645621	63	64.2330703	63	64.7457016
64.2379965	63	69.6523292	63	66.0585601	63	65.7395621	63	64.0108481	63	64.1742731
61.7906381	63	69.8523292	63	66.0585601	63	66.4895621	63	64.0108481	63	64.3171302
61.592291	63	69.4523292	63	65.7585601	63	66.8645621	63	63.6775148	63	63.620477
63.9652692	63	69.4523292	63	65.5585601	63	67.2395621	63	63.5664037	63	63.620477
63.6016329	63	69.4523292	63	65.3585601	63	67.1145621	63	63.899737	63	63.9538104
62.7834511	63	69.6523292	63	65.5585601	63	67.2395621	63	64.1219592	63	63.9538104
62.8743601	63	69.6523292	63	65.6585601	63	67.6145621	63	63.899737	63	63.2871437
62.9652692	63	70.0523292	63	65.5585601	63	67.8645621	63	64.5664037	63	62.2871437
62.3289056	63	70.7523292	63	65.6259487	63	67.8645621	63	64.5664037	63	61.9538104
62.5107238	63	71.1523292	63	66.1536332	63	68.3645621	63	64.4552926	63	62.620477
62.692542	63	71.5523292	63	65.8585601	63	68.2395621	63	64.3441814	63	62.7871437
62.4198147	63	71.4523292	63	65.8585601	63	68.1145621	63	64.4552926	63	64.620477
62.6016329	63	71.2523292	63	65.6585601	63	68.7395621	63	64.6775148	63	64.4538104
63.0561783	63	70.1222816	63	65.9585601	63	68.4895621	63	65.2330703	63	64.620477
63.3289056	63	69.3522816	63	65.5585601	63	68.9895621	63	65.3441814	63	64.620477
63.3289056	63	69.3522816	63	65.6585601	63	69.1145621	63	65.5664037	63	64.7871437
63.3289056	63	69.9523292	63	65.6585601	63	69.6145621	63	65.2330703	63	64.9538104
63.1470874	63	69.9523292	63	65.5585601	63	69.8645621	63	65.4552926	63	64.9538104
63.1470874	63	69.0523292	63	66.2585601	63	69.8645621	63	65.5664037	63	64.9538104

62.8743601	63	68.4523292	63	67.7585601	63	68.5956493	63	65.5664037	63	65.4538104
61.0821572	63	68.5523292	63	67.9585601	63	69.1737743	63	66.0108481	63	64.620477
61.0821572	63	69.7523292	63	68.0585601	63	69.1737743	63	66.0108481	63	64.2871437
61.1730663	63	70.1523292	63	68.4585601	63	69.6552068	63	65.1219592	63	63.620477
62.9652692	63	70.5523292	63	68.9585601	63	69.4706493	63	64.3441814	63	63.620477
62.9652692	71	70.3523292	63	69.2585601	63	69.3456493	63	63.6775148	63	63.7871437
63.0561783	71	70.4523292	63	69.2585601	63	69.9237743	63	63.4552926	63	63.9538104
63.5107238	71	69.8523292	63	69.3585601	63	69.6737743	63	63.3441814	63	64.4538104
63.4198147	71	69.6523292	63	69.4585601	63	69.1268993	63	63.3441814	63	64.620477
63.0561783	71	69.5523292	63	69.4585601	63	69.1268993	63	63.3441814	63	64.9538104
62.7834511	71	70.1523292	63	69.8585601	63	69.1268993	63	63.3441814	63	64.4538104
62.8743601	71	70.6523292	63	70.0585601	63	68.8456493	63	63.4552926	63	65.620477
63.0561783	71	71.2523292	63	70.0585601	63	68.9862743	63	64.4552926	63	65.7871437
63.1470874	71	71.6523292	63	69.4585601	63	68.5800243	63	64.899737	63	65.9538104
63.3289056	71	71.7523292	63	69.0585601	63	67.9706493	63	65.3441814	63	66.120477
63.5107238	71	71.8523292	63	69.3585601	63	68.1112743	63	64.1219592	63	66.4538104
63.7834511	71	72.7523292	63	69.2585601	63	68.2206493	63	64.1219592	63	66.4538104
63.9652692	71	71.660167	63	69.1585601	63	68.6268993	71	64.4552926	63	66.620477
64.0561783	71	71.370167	63	69.4585601	63	69.3143993	71	64.6775148	63	66.620477
63.9652692	71	70.490167	63	69.7585601	63	71.7395621	71	65.1219592	63	66.9538104
63.9652692	71	69.630167	63	69.6585601	63	71.8645621	71	65.6775148	63	66.9538104
63.9652692	71	69.100167	63	69.2585601	63	71.9895621	63	66.2330703	63	66.9538104
64.2379965	71	69.030167	63	67.6672451	63	71.4895621	63	66.5664037	63	67.620477
64.692542	71	68.280167	63	68.4585601	63	70.7395621	63	66.6775148	63	68.120477
65.8743601	71	68.310167	63	68.1585601	63	70.7395621	63	66.5664037	63	67.4538104
66.3289056	71	68.200167	63	67.0585601	63	70.3645621	63	66.4552926	63	67.4538104
66.8743601	71	68.480167	63	66.0585601	63	68.6145621	63	66.6775148	63	67.7871437
66.9652692	71	68.260167	63	65.4585601	63	67.9895621	63	66.3441814	63	67.620477
66.3289056	71	68.370167	63	65.4585601	63	66.4895621	63	66.3441814	63	67.4538104
65.6016329	71	68.390167	63	65.4585601	63	66.2395621	63	66.5664037	63	67.4538104
65.2379965	71	68.500167	69	65.8585601	63	64.7395621	63	66.4552926	63	67.120477
65.2379965	71	68.610167	69	66.2585601	63	64.2395621	63	67.3441814	63	66.7871437
65.3289056	71	68.400167	69	66.2585601	63	63.8645621	63	67.4552926	69	66.120477
65.4198147	71	68.070167	69	66.5585601	63	63.8645621	63	67.1219592	69	65.4538104
65.8743601	71	68.400167	69	66.8585601	63	63.9895621	63	66.6775148	69	64.9538104
66.2379965	71	69.180167	69	66.8585601	63	63.8645621	63	67.0108481	69	62.4538104
66.3289056	71	70.220167	69	66.9585601	63	63.8645621	63	67.6775148	69	62.4538104
66.2379965	71	70.770167	69	66.4585601	63	64.1145621	63	67.2330703	69	62.620477
66.0561783	71	71.210167	69	66.4585601	63	63.9895621	63	66.6775148	69	61.9538104
65.9652692	71	71.430167	69	67.1585601	63	63.9895621	63	66.4552926	69	62.120477
66.0561783	71	71.990167	69	67.2585601	63	64.4895621	63	66.0108481	69	62.120477
64.8082069	71	72.100167	69	67.4585601	63	64.2395621	63	65.6775148	69	62.120477
64.1139921	71	72.420167	69	67.8585601	63	63.9895621	63	65.5664037	69	62.620477

64.2131656	71	72.550167	69	67.9339915	63	64.6145621	63	65.5664037	69	62.7871437
64.7834511	71	72.550167	69	68.4603045	63	64.8645621	63	63.9944275	69	62.7871437
64.8743601	71	72.520167	69	68.1585601	63	65.1145621	63	64.2536868	69	62.9538104
64.1470874	71	72.070167	69	67.5585601	63	65.2395621	63	64.5252917	69	63.4538104
63.8743601	71	72.9523292	69	67.9585601	63	65.4895621	63	64.4018349	69	64.2871437
62.5880228	71	72.1523292	69	67.5585601	63	65.6145621	63	63.7351073	69	64.4538104
64.1470874	71	72.2523292	69	67.8585601	63	66.1145621	63	64.1594682	69	64.2871437
62.5962872	71	72.5523292	69	66.5263294	63	66.4895621	63	64.0594682	69	63.7871437
62.6294636	71	72.8523292	69	66.4179961	63	66.4895621	63	63.6594682	69	63.620477
62.2905021	71	72.8523292	69	66.0929961	63	66.4895621	63	63.9594682	69	63.620477
64.3289056	71	75.3523292	69	66.2013294	63	67.1145621	63	64.0594682	69	66.2871437
64.692542	71	75.5523292	69	67.0585601	63	67.2395621	63	64.0594682	69	66.7871437
64.8743601	71	76.7523292	69	65.1763294	63	67.2395621	63	64.5594682	69	66.2871437
65.2379965	71	76.4523292	69	66.6585601	63	67.2395621	63	63.9594682	69	65.7871437
65.3289056	71	75.8523292	69	65.1763294	63	66.9895621	63	64.1594682	69	65.4538104
64.8743601	71	75.5523292	69	66.0585601	63	65.6145621	63	64.7594682	69	65.4538104
64.7834511	71	75.2523292	69	66.2585601	63	65.1145621	63	64.9594682	69	65.120477
64.7834511	71	74.7939606	69	66.3585601	63	65.1145621	63	64.5594682	69	65.120477
64.5107238	71	74.5639606	69	66.1585601	63	65.6145621	63	64.7594682	69	65.120477
64.1470874	71	74.7839606	69	66.4585601	63	65.4895621	63	63.9594682	69	65.7871437
62.0928625	71	74.4539606	69	66.5585601	63	65.1145621	63	64.7594682	69	65.4538104
61.9121833	71	75.5439606	69	66.4585601	63	65.2395621	63	64.8594682	69	65.120477
61.8212742	71	75.5439606	69	66.6585601	63	64.7395621	63	64.8594682	69	64.2871437
62.0196213	71	75.9739606	69	66.6585601	63	64.3645621	63	65.3594682	69	63.4538104
61.763423	71	74.9094252	69	66.6585601	63	64.2395621	63	65.3594682	69	63.4538104
61.5650759	71	74.6694252	69	66.6585601	63	64.1145621	63	64.6594682	69	63.2871437
62.0609436	71	74.5294252	69	66.8585601	63	64.2395621	63	64.6594682	69	63.120477
62.4741668	71	73.6694252	69	66.7585601	63	64.6145621	63	64.5594682	69	63.120477
62.7799519	71	73.1094252	69	66.7585601	63	64.3645621	63	65.3594682	69	61.9538104
63.3006131	71	73.1094252	69	66.6585601	63	64.6145621	63	65.9594682	69	62.120477
63.4163155	71	72.8894252	69	66.4585601	63	64.6145621	63	66.1594682	69	61.7871437
64.6063982	71	73.5539606	69	66.2585601	63	64.4895621	63	66.4594682	69	61.7871437
65.4080511	71	73.5639606	69	66.0585601	63	64.6145621	63	67.1594682	69	61.9538104
66.209704	71	73.4539606	69	65.7585601	63	64.8645621	63	67.4594682	69	61.9538104
67.1022659	71	72.7939606	69	66.2585601	63	64.9895621	63	68.0594682	69	61.7871437
67.3997866	71	72.6839606	69	66.1585601	63	65.2395621	63	67.9594682	69	61.7871437
67.8956544	71	73.1139606	69	66.2585601	63	64.4669663	63	68.4594682	69	61.2871437
66.9007931	71	72.8939606	69	66.2585601	63	65.0607163	63	68.2594682	69	64.620477
68.9617701	71	72.7839606	69	66.3585601	69	65.3419663	63	68.2594682	69	65.4538104
68.1735203	71	71.7939606	69	66.6585601	69	65.7794663	63	66.9357101	69	65.4538104
69.185284	71	71.6839606	69	66.4585601	69	66.2013413	63	66.2757101	69	65.4538104
69.292722	71	72.6739606	69	66.5585601	69	66.3419663	63	66.2857101	69	66.120477
68.9952013	71	72.9939606	69	66.4585601	69	66.7638413	63	65.8457101	69	66.7871437

68.6976807	71	72.8839606	69	66.9585601	69	66.9044663	63	65.1857101	69	66.620477
68.4993336	71	73.2039606	69	67.0585601	69	68.8645621	63	64.7557101	69	67.120477
68.2761931	71	73.0939606	69	67.7585601	69	68.6145621	63	64.7857101	69	68.620477
68.8547055	71	73.5339606	69	68.5585601	69	68.6145621	63	64.4557101	69	68.4538104
68.7555319	71	73.6339606	69	68.5585601	69	68.4895621	63	64.3657101	69	68.620477
68.3588377	71	73.4139606	69	68.6585601	69	63.6625287	63	64.1257101	69	68.7871437
68.5571848	71	73.1939606	69	68.9585601	69	64.6625287	63	63.7957101	69	69.120477
68.846441	71	73.1939606	69	69.1585601	69	64.995862	63	63.4457101	69	69.4538104
68.4497468	71	73.3639606	69	68.9585601	69	65.3291953	63	63.1157101	69	69.9538104
68.4497468	71	73.4739606	69	69.0585601	69	65.4403065	63	62.5757101	69	69.9538104
68.1522261	71	73.2239606	69	69.2585601	69	65.6625287	63	62.3457101	69	69.9538104
69.0656283	71	73.3339606	69	69.2585601	69	65.2180842	63	62.3457101	69	70.120477
69.1648019	60	74.2328441	69	68.9337419	69	65.3291953	63	63.2594682	60	69.9538104

S2B Dataset Last 3 min

	102214		102314		102414		102814		time	Average
	11-12.		9-1030		9-10.		10-11.			
	34.00		35.00		36.00		37.00			
63	60.1611686	63	70.1980058	63	70.7813595	63	65.7417983	71	0:00:01	68.5519377
63	60.58974	63	71.0551486	63	70.7813595	63	65.3529094	71	0:00:02	68.5453171
63	60.58974	63	70.7694343	63	70.7813595	63	67.2276555	71	0:00:03	68.5629634
63	60.58974	63	71.1980058	63	71.352788	63	67.0609888	71	0:00:04	68.6393664
63	61.1611686	63	70.7694343	63	73.2099309	63	66.8943221	71	0:00:05	68.7140128
63	61.1611686	63	69.6265772	63	73.7813595	63	67.2276555	71	0:00:06	68.7246969
63	61.7325971	63	68.7694343	63	74.352788	63	67.8943221	71	0:00:07	68.8440146
63	62.0183114	63	67.9122915	63	74.4956452	63	68.0609888	71	0:00:08	68.7119722
63	62.8754543	63	67.3408629	63	74.7813595	63	68.5609888	71	0:00:09	68.6721908
63	62.58974	63	67.0551486	63	74.9242166	63	68.8943221	71	0:00:10	68.7694537
63	62.1611686	63	67.0551486	63	74.9242166	63	69.2276555	71	0:00:11	68.6436828
63	62.3040257	63	65.6265772	63	74.9242166	63	69.5609888	71	0:00:12	68.608051
63	62.1611686	63	65.9122915	63	73.7813595	63	69.8943221	71	0:00:13	68.7368916
63	62.0183114	63	66.0551486	63	72.352788	63	70.8943221	71	0:00:14	68.7497727
63	61.7325971	63	65.9122915	63	72.352788	63	71.5609888	73	0:00:15	68.9581111
63	61.4468828	63	66.3408629	63	71.9242166	63	70.5609888	73	0:00:16	68.9074721
63	62.3040257	63	66.3408629	63	72.0670737	63	70.8943221	73	0:00:17	68.9892749
63	62.3040257	63	66.3408629	63	71.2099309	63	68.8943221	73	0:00:18	68.8347813
63	62.8754543	63	66.0551486	63	70.7813595	63	68.0609888	73	0:00:19	68.7337015
63	63.0183114	63	66.4837201	63	70.6385023	63	67.5609888	73	0:00:20	68.6370357
63	63.0183114	63	66.3408629	63	70.7813595	63	68.3943221	73	0:00:21	68.6739214
63	63.8754543	63	65.9122915	63	69.7813595	63	67.8943221	73	0:00:22	68.6405967
63	63.8754543	63	66.4837201	63	69.2099309	63	67.7276555	73	0:00:23	68.7442329
63	64.0183114	63	66.3408629	63	69.2099309	63	67.3943221	73	0:00:24	68.6994279
63	64.0183114	63	66.3408629	63	69.2099309	63	67.2276555	73	0:00:25	68.5481059
63	63.8754543	63	66.0551486	63	68.6385023	63	67.7276555	73	0:00:26	68.426367
63	64.0183114	63	65.6265772	63	68.9242166	63	67.3943221	73	0:00:27	68.2757231
63	63.58974	63	66.0551486	63	69.352788	63	67.0609888	73	0:00:28	68.2921016
63	64.0183114	63	66.0551486	63	69.4956452	63	67.3943221	73	0:00:29	68.2299446
63	64.4468828	63	66.1980058	63	69.4956452	63	67.3943221	73	0:00:30	68.1952424
63	65.58974	71	66.3408629	63	69.352788	63	67.7276555	73	0:00:31	68.3309198
63	65.8754543	71	67.1980058	63	69.4956452	63	67.8943221	73	0:00:32	68.3158351
63	66.3040257	71	68.9122915	63	69.352788	63	67.8943221	73	0:00:33	68.2599898
63	66.4468828	71	69.4837201	63	69.2099309	63	68.5609888	73	0:00:34	68.1399579
63	64.4197552	71	69.1980058	63	69.2099309	63	69.8943221	73	0:00:35	67.9833552
63	63.9435647	71	68.7694343	63	69.4956452	63	71.0609888	73	0:00:36	68.015975
63	63.4673742	71	68.3408629	63	69.352788	63	71.0609888	73	0:00:37	68.0161841
63	62.9435647	71	68.0551486	63	69.0670737	63	71.2276555	73	0:00:38	68.0964408
63	62.7372155	71	68.9122915	63	68.9242166	63	68.7417983	73	0:00:39	68.1067406

63	62.261025	71	69.4837201	63	69.2099309	63	72.2276555	73	0:00:40	68.3280063
63	62.1022948	71	70.0551486	63	68.352788	63	71.5609888	73	0:00:41	68.2677247
63	63.0183114	71	70.0551486	63	68.6385023	63	71.8943221	73	0:00:42	68.3324365
63	59.3721361	71	70.1980058	63	69.352788	63	71.5609888	73	0:00:43	68.3260104
63	60.4468828	71	71.0551486	63	69.2099309	63	70.8943221	73	0:00:44	68.4572233
63	60.4468828	71	71.0551486	63	69.7813595	63	70.8943221	71	0:00:45	68.3620374
63	60.0183114	71	71.6265772	63	69.9242166	63	70.8943221	71	0:00:46	68.2912576
63	59.58974	71	70.0194122	63	71.4956452	63	70.3943221	71	0:00:47	68.2314692
63	59.4468828	71	70.7694343	63	71.352788	63	70.7276555	71	0:00:48	68.3908072
63	59.7325971	71	70.3408629	63	71.7813595	63	71.0609888	71	0:00:49	68.5103571
63	60.0183114	71	69.9122915	63	71.9242166	63	71.0609888	71	0:00:50	68.5401507
63	60.1611686	71	69.7694343	63	71.9242166	63	71.3943221	71	0:00:51	68.5389019
63	61.58974	71	70.3408629	63	71.7813595	63	71.2276555	71	0:00:52	68.5073372
63	61.58974	71	70.4837201	63	71.7813595	63	71.0609888	71	0:00:53	68.40102
63	63.0183114	71	70.9122915	63	71.9242166	63	70.77077	71	0:00:54	68.5855896
63	63.0183114	71	70.9122915	63	72.2099309	63	71.3541034	71	0:00:55	68.6423657
63	62.58974	71	71.6265772	63	72.352788	63	70.3818811	71	0:00:56	68.6765161
63	62.4468828	71	72.1980058	63	72.0670737	63	70.6874367	71	0:00:57	68.662896
63	62.3040257	71	72.1980058	63	71.9242166	63	69.9096589	71	0:00:58	68.6299428
63	62.7325971	63	73.1980058	63	72.0670737	63	68.77077	71	0:00:59	68.6633118
63	62.3040257	63	73.0551486	63	71.9242166	63	68.1874367	71	0:01:00	68.6829674
63	61.8754543	63	72.7694343	63	72.352788	63	68.02077	71	0:01:01	68.7693672
63	61.7325971	63	71.4837201	63	72.9242166	63	67.6318811	71	0:01:02	68.8044576
63	61.8754543	63	70.4837201	63	72.7813595	63	67.4652145	71	0:01:03	68.7608254
63	62.8754543	63	70.3408629	63	71.6385023	63	66.8818811	71	0:01:04	68.694937
63	63.3040257	63	70.3408629	63	71.6385023	63	65.5485478	71	0:01:05	68.6314052
69	64.1611686	63	69.9122915	63	70.4956452	63	64.3818811	71	0:01:06	68.7215895
69	63.8754543	63	69.7694343	63	70.2099309	63	64.2429923	71	0:01:07	68.6474383
69	64.0183114	63	70.6265772	63	70.2099309	63	64.8263256	71	0:01:08	68.6548673
69	64.0183114	63	71.1980058	63	71.352788	63	65.7985478	71	0:01:09	68.7485502
69	64.58974	63	71.0551486	63	72.352788	63	67.4096589	71	0:01:10	68.987941
69	64.58974	63	71.1980058	63	72.9242166	63	68.7985478	71	0:01:11	69.0563166
69	64.58974	63	70.7694343	63	72.4956452	63	68.9929923	71	0:01:12	69.1177345
69	63.7325971	63	70.7694343	63	72.4956452	63	68.7985478	71	0:01:13	69.1675432
69	63.8754543	63	70.4837201	63	72.9242166	63	67.8541034	71	0:01:14	69.2184357
69	63.58974	63	69.6265772	63	73.2099309	63	67.6874367	71	0:01:15	69.1163032
69	63.58974	63	70.1980058	63	73.9242166	63	66.5485478	73	0:01:16	69.005542
69	63.4468828	63	70.1980058	63	74.352788	63	66.6041034	73	0:01:17	68.9053253
69	62.4468828	63	70.4837201	63	75.0670737	63	66.5763256	73	0:01:18	68.9836863
69	62.58974	63	70.1980058	63	73.9242166	63	66.9652145	73	0:01:19	69.0909917
69	62.0183114	63	69.7694343	63	74.352788	63	67.1596589	65	0:01:20	69.1030126
69	62.4468828	63	69.9887373	63	73.7813595	63	67.7429923	65	0:01:21	69.1154986
69	62.58974	63	70.0839754	63	73.4956452	63	67.77077	65	0:01:22	69.2349555

69	62.8754543	63	70.0681024	63	73.4956452	63	68.5485478	65	0:01:23	69.4142061
69	64.3040257	63	70.703023	63	72.9242166	63	68.7429923	65	0:01:24	69.5025103
69	65.1611686	63	70.8617532	63	72.7813595	63	69.9096589	65	0:01:25	69.5336868
69	65.1611686	63	69.8458802	63	71.7813595	63	69.9096589	65	0:01:26	69.507499
69	65.1611686	63	69.8458802	63	71.7813595	63	70.4929923	65	0:01:27	69.5861543
69	64.4468828	63	69.68715	63	71.7813595	63	70.6874367	65	0:01:28	69.6693987
69	63.58974	63	71.1157215	63	71.4956452	63	70.4929923	65	0:01:29	69.766859
69	63.3040257	63	71.4331818	63	71.352788	63	70.8818811	65	0:01:30	69.6653191
69	62.8754543	63	71.2744516	63	70.9242166	63	70.6874367	65	0:01:31	69.5889479
69	63.1611686	63	71.2744516	63	70.9242166	63	70.8818811	65	0:01:32	69.5598271
69	64.0183114	63	71.4331818	63	70.4956452	63	71.0763256	65	0:01:33	69.6827797
69	64.8754543	63	70.9411183	63	70.4956452	63	71.4652145	65	0:01:34	69.6881221
69	64.8754543	63	72.4837201	63	70.4956452	63	71.4652145	65	0:01:35	69.6981235
69	62.7325971	63	72.0551486	63	71.2099309	63	71.6596589	65	0:01:36	69.5728538
69	62.1611686	63	70.9122915	63	71.7813595	63	71.8263256	65	0:01:37	69.6613896
69	61.7325971	63	70.1980058	63	72.0670737	63	71.6041034	65	0:01:38	69.5925445
69	61.58974	63	69.5626474	63	72.2099309	63	71.4096589	65	0:01:39	69.5583216
69	61.3040257	63	70.6265772	63	71.9242166	63	71.4096589	65	0:01:40	69.6994295
69	61.3040257	63	67.4479836	63	71.7813595	63	71.2152145	65	0:01:41	69.6002985
69	62.0183114	63	67.6543328	63	71.352788	63	71.1874367	65	0:01:42	69.5620711
69	62.1611686	63	67.3844916	63	71.0670737	63	71.5485478	65	0:01:43	69.599735
69	62.1611686	63	67.2733804	63	71.4956452	63	71.9374367	65	0:01:44	69.6299984
69	62.3040257	63	67.5908408	63	72.2099309	63	72.52077	65	0:01:45	69.687142
69	62.4468828	63	67.7495709	63	69.694646	63	73.7152145	65	0:01:46	69.7165685
69	62.4468828	63	67.5908408	63	70.0824011	63	74.4929923	65	0:01:47	69.7887281
69	62.8754543	63	68.0670312	63	71.0824011	63	76.27077	65	0:01:48	69.8786964
69	62.7325971	63	68.0670312	63	71.4089317	63	80.5485478	65	0:01:49	69.8718175
69	63.1611686	63	67.6384598	63	71.4089317	63	79.3818811	65	0:01:50	69.6861386
69	63.0183114	63	68.4321106	63	72.061993	63	81.52077	65	0:01:51	69.6901726
69	63.4468828	63	68.5273487	63	72.061993	63	83.0763256	65	0:01:52	69.7896988
69	64.0183114	63	68.9400471	63	72.3885236	63	83.5485478	65	0:01:53	69.8628512
69	64.3040257	63	69.1463963	63	72.3068909	63	84.52077	65	0:01:54	69.9445588
69	64.8754543	63	68.5114757	63	72.2864827	63	85.0763256	65	0:01:55	69.921837
69	64.7325971	63	68.5114757	63	72.4497481	63	84.6874367	65	0:01:56	69.8846224
69	64.58974	63	68.5590947	63	71.9599521	63	82.2991762	65	0:01:57	69.8281356
69	64.3040257	63	68.7178249	63	72.0824011	63	84.1325095	65	0:01:58	70.0734072
69	64.3040257	63	68.7178249	63	72.0824011	63	83.7436206	65	0:01:59	70.0636902
69	64.1611686	69	69.2892535	63	70.1232174	63	83.4658429	65	0:02:00	69.9860026
69	64.3040257	69	69.1305233	63	74.4956452	63	83.3547318	65	0:02:01	69.9353802
69	64.4468828	69	69.4956027	63	72.7813595	63	81.5213984	65	0:02:02	69.8777384
69	64.1611686	69	69.1305233	63	73.6385023	63	80.1880651	65	0:02:03	69.8280817
69	64.7325971	69	69.2892535	63	73.4956452	63	79.6325095	65	0:02:04	69.7148737
69	64.8754543	69	69.0035392	63	73.6385023	63	79.5213984	65	0:02:05	69.7341695

69	65.4468828	69	68.2098884	63	73.9242166	63	77.9102873	65	0:02:06	69.5564346
69	65.58974	69	68.0511582	63	74.0670737	71	78.1325095	65	0:02:07	69.5531639
69	65.4468828	69	67.79719	63	74.9242166	71	78.576954	65	0:02:08	69.6875856
69	66.3040257	69	67.6543328	63	74.4956452	71	78.4658429	65	0:02:09	69.7204643
69	66.0183114	69	69.0551486	63	74.7813595	71	77.6325095	65	0:02:10	69.8954854
69	65.7325971	69	68.1980058	63	75.2099309	71	76.8547318	65	0:02:11	69.7486247
69	65.8754543	69	68.1980058	63	75.4956452	71	76.076954	65	0:02:12	69.898899
69	64.0183114	69	68.3408629	63	75.4956452	71	77.4652145	65	0:02:13	69.9571274
69	62.0183114	69	68.3408629	63	75.7813595	71	74.52077	65	0:02:14	69.6898421
69	61.8754543	69	69.1980058	63	75.2099309	71	72.77077	65	0:02:15	69.5861387
69	61.0183114	69	68.7694343	63	75.0670737	71	71.4096589	65	0:02:16	69.6075458
69	60.8754543	69	69.1980058	63	75.352788	71	70.2152145	65	0:02:17	69.5700522
69	61.0183114	69	69.1980058	63	75.6385023	71	70.4096589	65	0:02:18	69.4921767
69	60.7325971	69	69.7694343	63	76.352788	71	69.5763256	65	0:02:19	69.4356321
69	60.8754543	69	69.9122915	63	75.9242166	71	69.3818811	65	0:02:20	69.356752
69	60.7325971	69	69.7694343	63	75.352788	71	68.9652145	65	0:02:21	69.1965882
69	60.4468828	69	69.3408629	63	74.6385023	71	69.3541034	65	0:02:22	69.1237484
69	60.0183114	69	68.1980058	63	74.6385023	71	68.8818811	65	0:02:23	69.0508891
69	59.58974	69	67.6265772	63	74.7813595	71	68.6596589	65	0:02:24	69.0886207
69	59.58974	69	67.0551486	63	74.6385023	71	69.2429923	65	0:02:25	69.102407
69	59.7325971	69	67.9122915	63	72.0670737	71	69.4374367	65	0:02:26	68.9983415
69	60.0183114	69	67.7694343	63	73.6385023	71	69.6318811	65	0:02:27	68.9703753
69	60.4468828	69	67.6265772	63	73.7813595	71	70.1874367	65	0:02:28	69.0017297
69	60.4468828	69	67.3408629	63	74.352788	71	69.9929923	65	0:02:29	69.0011159
69	62.3040257	69	67.7694343	63	74.6385023	71	70.77077	65	0:02:30	68.9695086
69	62.7325971	69	67.9122915	63	74.7813595	71	71.1596589	65	0:02:31	69.0210708
69	63.3040257	69	66.4548701	63	73.5109725	71	67.8541034	65	0:02:32	69.0239929
69	63.4468828	69	66.4548701	63	74.4089317	71	69.0457463	65	0:02:33	69.1692248
69	64.0183114	69	65.9786796	63	74.7354623	71	69.0457463	65	0:02:34	69.1413571
69	64.1611686	69	66.5024892	63	74.817095	71	68.5790796	65	0:02:35	69.1263741
69	65.58974	69	66.5501082	63	74.572197	69	68.8124129	65	0:02:36	69.1221052
69	62.613754	69	66.5024892	63	74.4089317	69	69.0457463	65	0:02:37	68.9516811
69	62.5026429	69	68.0551486	63	77.0670737	69	70.2124129	65	0:02:38	69.0653042
69	62.3439128	69	66.6265772	63	76.352788	69	70.9124129	65	0:02:39	68.9687459
69	62.8201032	69	67.3408629	63	75.9242166	69	70.9124129	65	0:02:40	69.0683336
69	62.9788334	69	66.6265772	63	75.2099309	69	71.1457463	65	0:02:41	69.115981
69	62.9788334	69	66.7694343	63	74.4956452	69	70.1457463	65	0:02:42	69.0753525
69	63.550262	69	66.4837201	63	74.7813595	69	68.4790796	65	0:02:43	69.18699
69	63.8677223	69	66.7694343	63	74.6385023	69	68.0457463	65	0:02:44	69.4290566
69	64.1851826	69	66.4837201	63	74.6385023	69	68.4252542	65	0:02:45	69.4868337
69	63.9788334	69	66.6265772	63	74.7813595	69	68.4252542	65	0:02:46	69.6124866
69	63.9788334	69	66.1980058	63	75.0670737	69	69.0252542	65	0:02:47	69.7746826
69	64.4074048	69	66.0551486	63	76.0670737	69	66.5834412	65	0:02:48	69.6524929

69	64.8359763	69	65.7694343	63	76.0670737	69	66.9501078	65	0:02:49	69.5696575
69	64.8359763	69	65.4837201	63	77.9242166	69	67.0834412	65	0:02:50	69.8839217
69	67.3040257	69	65.3408629	63	74.6538297	69	66.9834412	65	0:02:51	70.1705871
69	64.9947064	69	65.3408629	63	72.8579113	69	67.0501078	65	0:02:52	70.1799152
69	64.9947064	69	65.3408629	63	72.8579113	69	66.7501078	65	0:02:53	70.1487401
69	64.9947064	69	65.6265772	63	72.8579113	69	66.9834412	60	0:02:54	70.0487664
69	64.9312144	69	65.3408629	63	74.0007685	69	67.0167745	60	0:02:55	70.1096911
69	64.8677223	69	65.7694343	63	75.2048501	69	67.2501078	60	0:02:56	70.2583648
69	64.7089921	69	65.7694343	63	77.0670737	69	68.1834412	60	0:02:57	70.4553036
69	64.4550239	69	65.4837201	63	77.2099309	69	68.1834412	60	0:02:58	70.4733096
69	66.8754543	69	65.6265772	63	75.0670737	69	68.9834412	60	0:02:59	70.5315965
69	65.4468828	69	65.0551486	63	74.7813595	69	70.3167745	60	0:03:00	70.5467647
69	64.8754543	69	65.0551486	63	72.2366278	69	71.9167745	60		

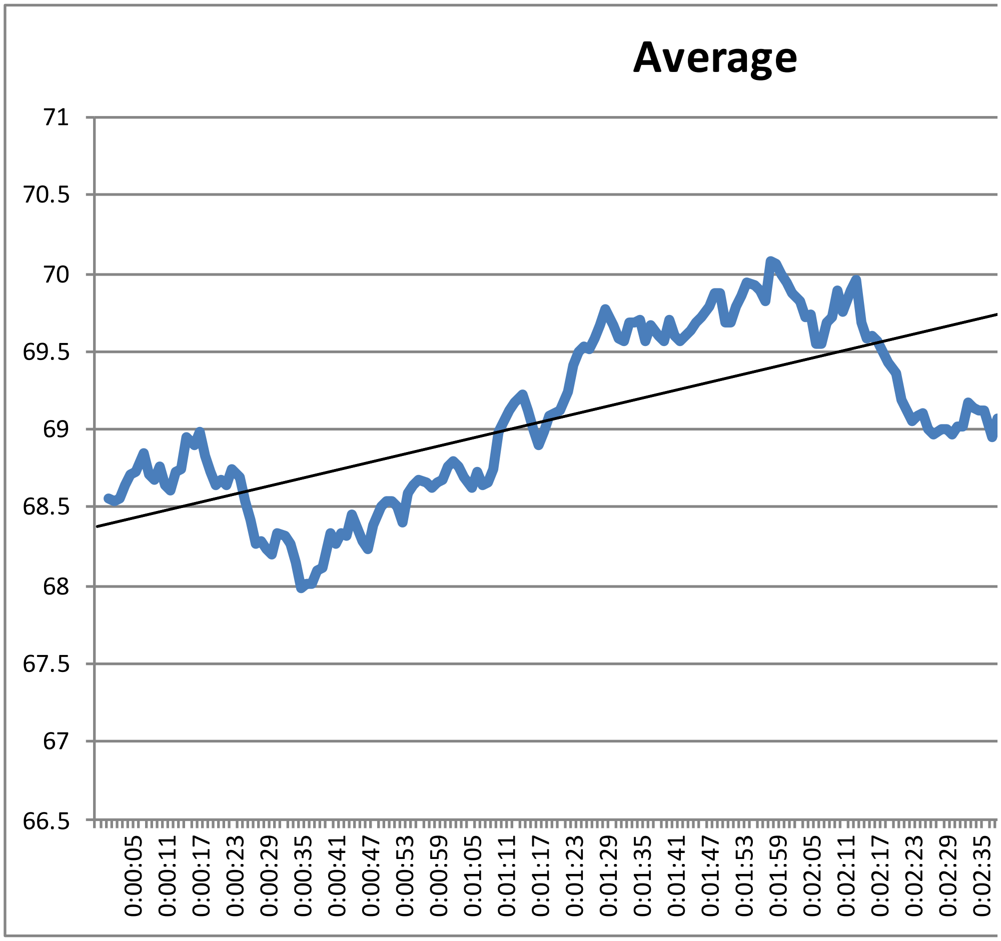

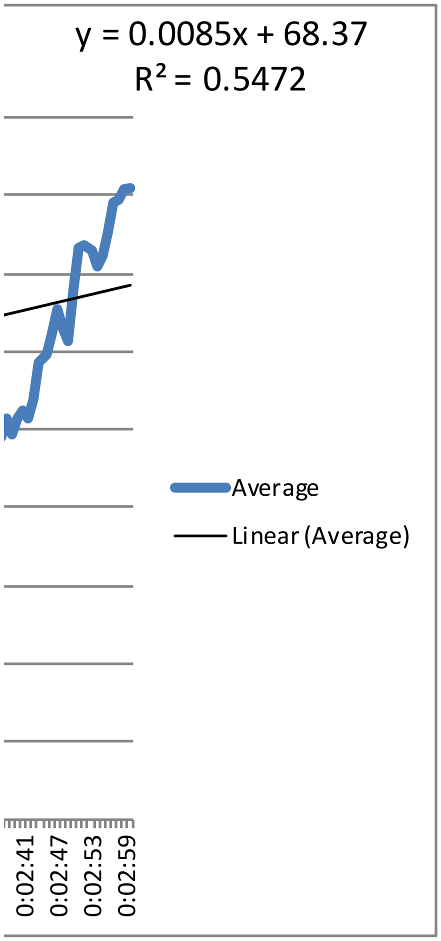

y = 0.0085x + 68.37
R^2 = 0.5472
Average
Linear (Average)
0:02:41
0:02:47
0:02:53
0:02:59
