# Supplementary material for: Student engagement, assessed using heart rate, shows no reset following active learning sessions in lectures
Source: PLoS One. 2019 Dec 2;14(12):e0225709. doi: 10.1371/journal.pone.0225709 (PMC6886849; doi:10.1371/journal.pone.0225709)
Supplement: S3 Dataset — (PDF) [file pone.0225709.s003.pdf]

S3 Dataset: R formatted data for S1 table, ANOVA

S3 Dataset is a data frame with 4 columns
column "who" is the person number (nuisance variable)
column "when" is the activity identifier (nuisance variable)
"what" is the variable of interest, taking values "Before", "Activity", or "After"
"rate" is the response variable, the average heartrate

**who**	**when**	**what**	**rate**
Person 1	Class 1	Before	78.293052442714
Person 1	Class 1	Activity	80.0241582800431
Person 1	Class 1	After	75.394297255992
Person 1	Class 10	Before	62.4322433474929
Person 1	Class 10	Activity	62.9641877919375
Person 1	Class 10	After	63.6155766808262
Person 1	Class 11	Before	67.5712774235763
Person 1	Class 11	Activity	63.9045647760643
Person 1	Class 11	After	63.5212238548866
Person 1	Class 12	Before	76.0479702064807
Person 1	Class 12	Activity	77.5345966894055
Person 1	Class 12	After	69.7433923900969
Person 1	Class 13	Before	74.0229702064807
Person 1	Class 13	Activity	74.4803420013525
Person 1	Class 13	After	73.1146368731474
Person 1	Class 14	Before	74.9628116362801
Person 1	Class 14	Activity	71.5986485010544
Person 1	Class 14	After	70.3229702064807
Person 1	Class 15	Before	66.0354702064807
Person 1	Class 15	Activity	70.6370901801737
Person 1	Class 15	After	70.7438035398141
Person 1	Class 16	Before	69.1010843981377
Person 1	Class 16	Activity	70.1422449919302
Person 1	Class 16	After	66.7494818340352
Person 1	Class 17	Before	78.57482100912
Person 1	Class 17	Activity	81.0856485944691
Person 1	Class 17	After	76.0398152611361
Person 1	Class 18	Before	70.2414891279482
Person 1	Class 18	Activity	65.3755013403559
Person 1	Class 18	After	68.7149389834475
Person 1	Class 19	Before	68.1789116795708
Person 1	Class 19	Activity	72.2473225322844
Person 1	Class 19	After	68.2455783462375
Person 1	Class 2	Before	68.3290357063121
Person 1	Class 2	Activity	74.7035533497033
Person 1	Class 2	After	67.4252049433571
Person 1	Class 20	Before	64.3633180655055
Person 1	Class 20	Activity	67.4748225184211

Person 1	Class 20	After	62.558137348068
Person 1	Class 21	Before	62.633137348068
Person 1	Class 21	Activity	68.4991296409389
Person 1	Class 21	After	64.3699138915565
Person 1	Class 22	Before	69.6370180268893
Person 1	Class 22	Activity	78.4856311412896
Person 1	Class 22	After	68.1830256977401
Person 1	Class 3	Before	69.1021915720857
Person 1	Class 3	Activity	71.8557172131114
Person 1	Class 3	After	71.4622711018229
Person 1	Class 4	Before	71.7063582387523
Person 1	Class 4	Activity	72.6171767152513
Person 1	Class 4	After	67.642864514903
Person 1	Class 5	Before	69.4051006451273
Person 1	Class 5	Activity	68.2709686283626
Person 1	Class 5	After	71.9813582387523
Person 1	Class 6	Before	71.7371187279782
Person 1	Class 6	Activity	71.1533840842435
Person 1	Class 6	After	72.1312628721224
Person 1	Class 7	Before	71.0562628721224
Person 1	Class 7	Activity	70.8999150983558
Person 1	Class 7	After	71.8437628721224
Person 1	Class 8	Before	67.3285050783578
Person 1	Class 8	Activity	72.3222152530749
Person 1	Class 8	After	70.4687628721224
Person 1	Class 9	Before	69.7604295387891
Person 1	Class 9	Activity	73.0648214306808
Person 1	Class 9	After	69.2270962054557
Person 10	Class 1	Before	82.7148834643726
Person 10	Class 1	Activity	83.5831085959588
Person 10	Class 1	After	76.1487181808318
Person 10	Class 10	Before	
Person 10	Class 10	Activity	
Person 10	Class 10	After	
Person 10	Class 11	Before	
Person 10	Class 11	Activity	
Person 10	Class 11	After	
Person 10	Class 12	Before	74.8550011875213
Person 10	Class 12	Activity	77.3786866358523
Person 10	Class 12	After	75.6800011875213
Person 10	Class 13	Before	73.6008345208546
Person 10	Class 13	Activity	70.8316037516238
Person 10	Class 13	After	68.0849113781286
Person 10	Class 14	Before	68.2258345208546
Person 10	Class 14	Activity	70.3140128154283

Person 10	Class 14	After	70.359167854188
Person 10	Class 15	Before	69.5731849954025
Person 10	Class 15	Activity	69.4856830057029
Person 10	Class 15	After	64.0050011875212
Person 10	Class 16	Before	
Person 10	Class 16	Activity	
Person 10	Class 16	After	
Person 10	Class 17	Before	70.2729961168916
Person 10	Class 17	Activity	69.8507738946694
Person 10	Class 17	After	73.2729961168916
Person 10	Class 18	Before	
Person 10	Class 18	Activity	
Person 10	Class 18	After	
Person 10	Class 19	Before	
Person 10	Class 19	Activity	
Person 10	Class 19	After	
Person 10	Class 2	Before	69.8715605912904
Person 10	Class 2	Activity	68.4505017439425
Person 10	Class 2	After	60.2773145938493
Person 10	Class 20	Before	63.5779195404307
Person 10	Class 20	Activity	66.169550794541
Person 10	Class 20	After	66.7194950694755
Person 10	Class 21	Before	67.1403284028089
Person 10	Class 21	Activity	72.4964732148608
Person 10	Class 21	After	63.6362528737641
Person 10	Class 22	Before	
Person 10	Class 22	Activity	
Person 10	Class 22	After	
Person 10	Class 3	Before	74.7058731268226
Person 10	Class 3	Activity	74.2433739161389
Person 10	Class 3	After	69.031460955315
Person 10	Class 4	Before	69.5475397934893
Person 10	Class 4	Activity	76.6457974920297
Person 10	Class 4	After	70.6411590403512
Person 10	Class 5	Before	70.2392064601559
Person 10	Class 5	Activity	77.4868255077752
Person 10	Class 5	After	69.2017064601559
Person 10	Class 6	Before	
Person 10	Class 6	Activity	
Person 10	Class 6	After	
Person 10	Class 7	Before	
Person 10	Class 7	Activity	
Person 10	Class 7	After	
Person 10	Class 8	Before	
Person 10	Class 8	Activity	

Person 10	Class 8	After	
Person 10	Class 9	Before	
Person 10	Class 9	Activity	
Person 10	Class 9	After	
Person 11	Class 1	Before	
Person 11	Class 1	Activity	
Person 11	Class 1	After	
Person 11	Class 10	Before	67.6368231841462
Person 11	Class 10	Activity	71.3132120730353
Person 11	Class 10	After	67.8118231841462
Person 11	Class 11	Before	61.5368231841463
Person 11	Class 11	Activity	64.4403946127177
Person 11	Class 11	After	61.7201565174796
Person 11	Class 12	Before	
Person 11	Class 12	Activity	
Person 11	Class 12	After	
Person 11	Class 13	Before	
Person 11	Class 13	Activity	
Person 11	Class 13	After	
Person 11	Class 14	Before	69.0956154200962
Person 11	Class 14	Activity	76.2643015141127
Person 11	Class 14	After	75.7640139176762
Person 11	Class 15	Before	69.1625385431794
Person 11	Class 15	Activity	71.099076100437
Person 11	Class 15	After	71.1729868752414
Person 11	Class 16	Before	73.8741994521297
Person 11	Class 16	Activity	81.3986658650391
Person 11	Class 16	After	64.3086002127193
Person 11	Class 17	Before	70.8259481725172
Person 11	Class 17	Activity	87.8099923543357
Person 11	Class 17	After	70.9055464744787
Person 11	Class 18	Before	66.7940960310643
Person 11	Class 18	Activity	64.6120038324832
Person 11	Class 18	After	71.0943605268625
Person 11	Class 19	Before	
Person 11	Class 19	Activity	
Person 11	Class 19	After	
Person 11	Class 2	Before	
Person 11	Class 2	Activity	
Person 11	Class 2	After	
Person 11	Class 20	Before	
Person 11	Class 20	Activity	
Person 11	Class 20	After	
Person 11	Class 21	Before	
Person 11	Class 21	Activity	

Person 11	Class 21	After	
Person 11	Class 22	Before	
Person 11	Class 22	Activity	
Person 11	Class 22	After	
Person 11	Class 3	Before	73.8931667033869
Person 11	Class 3	Activity	75.0176994566007
Person 11	Class 3	After	74.741674679899
Person 11	Class 4	Before	
Person 11	Class 4	Activity	
Person 11	Class 4	After	
Person 11	Class 5	Before	
Person 11	Class 5	Activity	
Person 11	Class 5	After	
Person 11	Class 6	Before	76.419237910764
Person 11	Class 6	Activity	77.9568136683395
Person 11	Class 6	After	74.7275712440973
Person 11	Class 7	Before	71.8425380689902
Person 11	Class 7	Activity	78.7671320142535
Person 11	Class 7	After	71.965369322848
Person 11	Class 8	Before	68.7110844335459
Person 11	Class 8	Activity	69.2773331488594
Person 11	Class 8	After	68.7650712440975
Person 11	Class 9	Before	65.7859045774308
Person 11	Class 9	Activity	72.5068505233763
Person 11	Class 9	After	66.8525712440975
Person 12	Class 1	Before	
Person 12	Class 1	Activity	
Person 12	Class 1	After	
Person 12	Class 10	Before	74.2164038174
Person 12	Class 10	Activity	73.3565602143833
Person 12	Class 10	After	70.0095556062072
Person 12	Class 11	Before	67.8071278418306
Person 12	Class 11	Activity	72.4862460071557
Person 12	Class 11	After	
Person 12	Class 12	Before	
Person 12	Class 12	Activity	
Person 12	Class 12	After	
Person 12	Class 13	Before	
Person 12	Class 13	Activity	
Person 12	Class 13	After	
Person 12	Class 14	Before	75.9036988258722
Person 12	Class 14	Activity	75.511445492404
Person 12	Class 14	After	74.0655179584117
Person 12	Class 15	Before	70.3536988258722
Person 12	Class 15	Activity	70.6177139773873

Person 12	Class 15	After	71.2578654925389
Person 12	Class 16	Before	
Person 12	Class 16	Activity	
Person 12	Class 16	After	
Person 12	Class 17	Before	
Person 12	Class 17	Activity	
Person 12	Class 17	After	
Person 12	Class 18	Before	
Person 12	Class 18	Activity	
Person 12	Class 18	After	
Person 12	Class 19	Before	
Person 12	Class 19	Activity	
Person 12	Class 19	After	
Person 12	Class 2	Before	74.7995608282869
Person 12	Class 2	Activity	71.0996333104827
Person 12	Class 2	After	69.6183283251455
Person 12	Class 20	Before	
Person 12	Class 20	Activity	
Person 12	Class 20	After	
Person 12	Class 21	Before	
Person 12	Class 21	Activity	
Person 12	Class 21	After	
Person 12	Class 22	Before	
Person 12	Class 22	Activity	
Person 12	Class 22	After	
Person 12	Class 3	Before	70.7885053644318
Person 12	Class 3	Activity	73.8176322398464
Person 12	Class 3	After	72.2133892352714
Person 12	Class 4	Before	
Person 12	Class 4	Activity	
Person 12	Class 4	After	
Person 12	Class 5	Before	
Person 12	Class 5	Activity	
Person 12	Class 5	After	
Person 12	Class 6	Before	74.3109936174174
Person 12	Class 6	Activity	76.6963198761741
Person 12	Class 6	After	70.4193269507508
Person 12	Class 7	Before	69.1734936174174
Person 12	Class 7	Activity	72.1302498421993
Person 12	Class 7	After	71.9943269507507
Person 12	Class 8	Before	71.372667503435
Person 12	Class 8	Activity	72.7059460234762
Person 12	Class 8	After	72.5358269758573
Person 12	Class 9	Before	67.7483269758572
Person 12	Class 9	Activity	71.928957606488

Person 12	Class 9	After	69.3649936425239
Person 13	Class 1	Before	
Person 13	Class 1	Activity	
Person 13	Class 1	After	
Person 13	Class 10	Before	70.679397574885
Person 13	Class 10	Activity	73.7960864826529
Person 13	Class 10	After	69.4600164179273
Person 13	Class 11	Before	66.4085642415517
Person 13	Class 11	Activity	73.2439809082184
Person 13	Class 11	After	64.5918975748851
Person 13	Class 12	Before	
Person 13	Class 12	Activity	
Person 13	Class 12	After	
Person 13	Class 13	Before	
Person 13	Class 13	Activity	
Person 13	Class 13	After	
Person 13	Class 14	Before	
Person 13	Class 14	Activity	
Person 13	Class 14	After	
Person 13	Class 15	Before	
Person 13	Class 15	Activity	
Person 13	Class 15	After	
Person 13	Class 16	Before	
Person 13	Class 16	Activity	
Person 13	Class 16	After	
Person 13	Class 17	Before	
Person 13	Class 17	Activity	
Person 13	Class 17	After	
Person 13	Class 18	Before	67.472207055093
Person 13	Class 18	Activity	67.963583886293
Person 13	Class 18	After	66.744967722466
Person 13	Class 19	Before	69.4436765338394
Person 13	Class 19	Activity	72.018192037715
Person 13	Class 19	After	69.2442643975122
Person 13	Class 2	Before	
Person 13	Class 2	Activity	
Person 13	Class 2	After	
Person 13	Class 20	Before	
Person 13	Class 20	Activity	
Person 13	Class 20	After	
Person 13	Class 21	Before	
Person 13	Class 21	Activity	
Person 13	Class 21	After	
Person 13	Class 22	Before	
Person 13	Class 22	Activity	

Person 13	Class 22	After	
Person 13	Class 3	Before	72.6965727928989
Person 13	Class 3	Activity	74.9480469052809
Person 13	Class 3	After	71.0649955168353
Person 13	Class 4	Before	
Person 13	Class 4	Activity	
Person 13	Class 4	After	
Person 13	Class 5	Before	
Person 13	Class 5	Activity	
Person 13	Class 5	After	
Person 13	Class 6	Before	73.1019617653679
Person 13	Class 6	Activity	70.4934011593073
Person 13	Class 6	After	73.4770473349085
Person 13	Class 7	Before	73.6025972618684
Person 13	Class 7	Activity	72.6025183237314
Person 13	Class 7	After	70.4769617653679
Person 13	Class 8	Before	68.5054090075739
Person 13	Class 8	Activity	70.2346998606059
Person 13	Class 8	After	68.7852950987012
Person 13	Class 9	Before	70.1519617653679
Person 13	Class 9	Activity	70.7311284320346
Person 13	Class 9	After	69.1019617653679
Person 14	Class 1	Before	
Person 14	Class 1	Activity	
Person 14	Class 1	After	
Person 14	Class 10	Before	
Person 14	Class 10	Activity	
Person 14	Class 10	After	
Person 14	Class 11	Before	
Person 14	Class 11	Activity	
Person 14	Class 11	After	
Person 14	Class 12	Before	
Person 14	Class 12	Activity	
Person 14	Class 12	After	
Person 14	Class 13	Before	
Person 14	Class 13	Activity	
Person 14	Class 13	After	
Person 14	Class 14	Before	
Person 14	Class 14	Activity	
Person 14	Class 14	After	
Person 14	Class 15	Before	
Person 14	Class 15	Activity	
Person 14	Class 15	After	
Person 14	Class 16	Before	
Person 14	Class 16	Activity	

Person 14	Class 16	After	
Person 14	Class 17	Before	80.1659910961744
Person 14	Class 17	Activity	78.4456024754891
Person 14	Class 17	After	77.7957220638606
Person 14	Class 18	Before	
Person 14	Class 18	Activity	
Person 14	Class 18	After	
Person 14	Class 19	Before	
Person 14	Class 19	Activity	
Person 14	Class 19	After	
Person 14	Class 2	Before	
Person 14	Class 2	Activity	
Person 14	Class 2	After	
Person 14	Class 20	Before	75.6003266441354
Person 14	Class 20	Activity	73.0918082583872
Person 14	Class 20	After	69.2857662171977
Person 14	Class 21	Before	69.3024328838644
Person 14	Class 21	Activity	68.8467817907887
Person 14	Class 21	After	65.2106496085516
Person 14	Class 22	Before	72.990098184948
Person 14	Class 22	Activity	68.3061836190884
Person 14	Class 22	After	75.6392765375884
Person 14	Class 3	Before	
Person 14	Class 3	Activity	
Person 14	Class 3	After	
Person 14	Class 4	Before	
Person 14	Class 4	Activity	
Person 14	Class 4	After	
Person 14	Class 5	Before	
Person 14	Class 5	Activity	
Person 14	Class 5	After	
Person 14	Class 6	Before	
Person 14	Class 6	Activity	
Person 14	Class 6	After	
Person 14	Class 7	Before	
Person 14	Class 7	Activity	
Person 14	Class 7	After	
Person 14	Class 8	Before	
Person 14	Class 8	Activity	
Person 14	Class 8	After	
Person 14	Class 9	Before	
Person 14	Class 9	Activity	
Person 14	Class 9	After	
Person 15	Class 1	Before	
Person 15	Class 1	Activity	

Person 15	Class 1	After	
Person 15	Class 10	Before	
Person 15	Class 10	Activity	
Person 15	Class 10	After	
Person 15	Class 11	Before	
Person 15	Class 11	Activity	
Person 15	Class 11	After	
Person 15	Class 12	Before	
Person 15	Class 12	Activity	
Person 15	Class 12	After	
Person 15	Class 13	Before	
Person 15	Class 13	Activity	
Person 15	Class 13	After	
Person 15	Class 14	Before	
Person 15	Class 14	Activity	
Person 15	Class 14	After	
Person 15	Class 15	Before	
Person 15	Class 15	Activity	
Person 15	Class 15	After	
Person 15	Class 16	Before	
Person 15	Class 16	Activity	
Person 15	Class 16	After	
Person 15	Class 17	Before	
Person 15	Class 17	Activity	
Person 15	Class 17	After	
Person 15	Class 18	Before	
Person 15	Class 18	Activity	
Person 15	Class 18	After	
Person 15	Class 19	Before	68.4950313405485
Person 15	Class 19	Activity	79.9431902552772
Person 15	Class 19	After	65.9484172190354
Person 15	Class 2	Before	
Person 15	Class 2	Activity	
Person 15	Class 2	After	
Person 15	Class 20	Before	67.170670356947
Person 15	Class 20	Activity	70.4823901083379
Person 15	Class 20	After	65.7518663188242
Person 15	Class 21	Before	65.7768663188242
Person 15	Class 21	Activity	69.3544674748937
Person 15	Class 21	After	64.0393663188242
Person 15	Class 22	Before	
Person 15	Class 22	Activity	
Person 15	Class 22	After	
Person 15	Class 3	Before	
Person 15	Class 3	Activity	

Person 15	Class 3	After
Person 15	Class 4	Before
Person 15	Class 4	Activity
Person 15	Class 4	After
Person 15	Class 5	Before
Person 15	Class 5	Activity
Person 15	Class 5	After
Person 15	Class 6	Before
Person 15	Class 6	Activity
Person 15	Class 6	After
Person 15	Class 7	Before
Person 15	Class 7	Activity
Person 15	Class 7	After
Person 15	Class 8	Before
Person 15	Class 8	Activity
Person 15	Class 8	After
Person 15	Class 9	Before
Person 15	Class 9	Activity
Person 15	Class 9	After
Person 16	Class 1	Before
Person 16	Class 1	Activity
Person 16	Class 1	After
Person 16	Class 10	Before
Person 16	Class 10	Activity
Person 16	Class 10	After
Person 16	Class 11	Before
Person 16	Class 11	Activity
Person 16	Class 11	After
Person 16	Class 12	Before
Person 16	Class 12	Activity
Person 16	Class 12	After
Person 16	Class 13	Before
Person 16	Class 13	Activity
Person 16	Class 13	After
Person 16	Class 14	Before
Person 16	Class 14	Activity
Person 16	Class 14	After
Person 16	Class 15	Before
Person 16	Class 15	Activity
Person 16	Class 15	After
Person 16	Class 16	Before
Person 16	Class 16	Activity
Person 16	Class 16	After
Person 16	Class 17	Before
Person 16	Class 17	Activity

Person 16	Class 17	After	
Person 16	Class 18	Before	
Person 16	Class 18	Activity	
Person 16	Class 18	After	
Person 16	Class 19	Before	69.7333133390235
Person 16	Class 19	Activity	67.8178504741574
Person 16	Class 19	After	66.9768621020641
Person 16	Class 2	Before	
Person 16	Class 2	Activity	
Person 16	Class 2	After	
Person 16	Class 20	Before	71.9664384394127
Person 16	Class 20	Activity	72.520818656927
Person 16	Class 20	After	70.9733595908164
Person 16	Class 21	Before	71.2400262574831
Person 16	Class 21	Activity	73.4191359345957
Person 16	Class 21	After	69.774771772746
Person 16	Class 22	Before	
Person 16	Class 22	Activity	
Person 16	Class 22	After	
Person 16	Class 3	Before	
Person 16	Class 3	Activity	
Person 16	Class 3	After	
Person 16	Class 4	Before	
Person 16	Class 4	Activity	
Person 16	Class 4	After	
Person 16	Class 5	Before	
Person 16	Class 5	Activity	
Person 16	Class 5	After	
Person 16	Class 6	Before	
Person 16	Class 6	Activity	
Person 16	Class 6	After	
Person 16	Class 7	Before	
Person 16	Class 7	Activity	
Person 16	Class 7	After	
Person 16	Class 8	Before	
Person 16	Class 8	Activity	
Person 16	Class 8	After	
Person 16	Class 9	Before	
Person 16	Class 9	Activity	
Person 16	Class 9	After	
Person 2	Class 1	Before	70.194688582191
Person 2	Class 1	Activity	78.1400253358625
Person 2	Class 1	After	77.4235960758404
Person 2	Class 10	Before	69.2517835343689
Person 2	Class 10	Activity	69.4740057565914

Person 2	Class 10	After	69.2917818948974
Person 2	Class 11	Before	69.1517835343689
Person 2	Class 11	Activity	72.1758786006031
Person 2	Class 11	After	69.1497312010784
Person 2	Class 12	Before	76.1525359973157
Person 2	Class 12	Activity	83.0927145687443
Person 2	Class 12	After	74.1677864152188
Person 2	Class 13	Before	80.148369330649
Person 2	Class 13	Activity	83.8211431904988
Person 2	Class 13	After	76.9192026639824
Person 2	Class 14	Before	67.0026122393056
Person 2	Class 14	Activity	65.1615476252228
Person 2	Class 14	After	61.6483693306492
Person 2	Class 15	Before	63.1317026639825
Person 2	Class 15	Activity	62.4453390276187
Person 2	Class 15	After	73.2779573456301
Person 2	Class 16	Before	75.271162175854
Person 2	Class 16	Activity	81.8148435407303
Person 2	Class 16	After	77.0874997160804
Person 2	Class 17	Before	74.0527575001513
Person 2	Class 17	Activity	75.555711086649
Person 2	Class 17	After	76.0057110866492
Person 2	Class 18	Before	65.3558325383314
Person 2	Class 18	Activity	64.2882948484269
Person 2	Class 18	After	65.9688799548098
Person 2	Class 19	Before	76.9075331202497
Person 2	Class 19	Activity	80.6564385599352
Person 2	Class 19	After	71.1670226873049
Person 2	Class 2	Before	74.660460131036
Person 2	Class 2	Activity	76.4736771643738
Person 2	Class 2	After	75.8018896410467
Person 2	Class 20	Before	69.9072443317421
Person 2	Class 20	Activity	69.6123541029236
Person 2	Class 20	After	69.8239109984088
Person 2	Class 21	Before	69.9572443317422
Person 2	Class 21	Activity	67.6204187055381
Person 2	Class 21	After	66.1734616493389
Person 2	Class 22	Before	76.1209557036733
Person 2	Class 22	Activity	85.1812472907688
Person 2	Class 22	After	69.7407343774044
Person 2	Class 3	Before	68.9134555580818
Person 2	Class 3	Activity	68.6945453016717
Person 2	Class 3	After	67.7863635802118
Person 2	Class 4	Before	
Person 2	Class 4	Activity	

Person 2	Class 4	After	
Person 2	Class 5	Before	
Person 2	Class 5	Activity	
Person 2	Class 5	After	
Person 2	Class 6	Before	73.943263065592
Person 2	Class 6	Activity	74.2555357928647
Person 2	Class 6	After	77.1265963989253
Person 2	Class 7	Before	72.7450536973548
Person 2	Class 7	Activity	72.9574327406822
Person 2	Class 7	After	69.4140963989254
Person 2	Class 8	Before	64.1984788929305
Person 2	Class 8	Activity	63.4368344941637
Person 2	Class 8	After	62.3992259709786
Person 2	Class 9	Before	63.7265963989254
Person 2	Class 9	Activity	57.745290092619
Person 2	Class 9	After	58.9599297322587
Person 3	Class 1	Before	76.6484536517059
Person 3	Class 1	Activity	79.4887314294834
Person 3	Class 1	After	74.7151894746658
Person 3	Class 10	Before	70.5230142830258
Person 3	Class 10	Activity	66.357117029751
Person 3	Class 10	After	70.7515614741956
Person 3	Class 11	Before	68.2140614741956
Person 3	Class 11	Activity	69.741144807529
Person 3	Class 11	After	67.6890614741956
Person 3	Class 12	Before	78.1160034208079
Person 3	Class 12	Activity	76.3258248493793
Person 3	Class 12	After	73.3118367541412
Person 3	Class 13	Before	76.7326700874745
Person 3	Class 13	Activity	75.7374777797822
Person 3	Class 13	After	74.6039541056672
Person 3	Class 14	Before	75.0410034208079
Person 3	Class 14	Activity	74.4498212502652
Person 3	Class 14	After	70.9694083003991
Person 3	Class 15	Before	70.3201700874745
Person 3	Class 15	Activity	72.2815337238382
Person 3	Class 15	After	67.4285034208079
Person 3	Class 16	Before	
Person 3	Class 16	Activity	
Person 3	Class 16	After	
Person 3	Class 17	Before	69.8310834165681
Person 3	Class 17	Activity	76.4130278610124
Person 3	Class 17	After	72.2394167499014
Person 3	Class 18	Before	69.9269007278808
Person 3	Class 18	Activity	65.0983546285901

Person 3	Class 18	After	67.0522668329262
Person 3	Class 19	Before	66.7452514612153
Person 3	Class 19	Activity	72.6412025249834
Person 3	Class 19	After	70.3471133776969
Person 3	Class 2	Before	74.3342974452903
Person 3	Class 2	Activity	70.1995289205232
Person 3	Class 2	After	70.8350927701633
Person 3	Class 20	Before	68.4614935426626
Person 3	Class 20	Activity	66.4822573027124
Person 3	Class 20	After	65.8531602093293
Person 3	Class 21	Before	65.8406602093293
Person 3	Class 21	Activity	63.3767632921809
Person 3	Class 21	After	67.4922840858608
Person 3	Class 22	Before	69.2997420024005
Person 3	Class 22	Activity	69.0521923644067
Person 3	Class 22	After	72.2023919326654
Person 3	Class 3	Before	76.6027052836384
Person 3	Class 3	Activity	74.2622348190465
Person 3	Class 3	After	71.5657928037505
Person 3	Class 4	Before	
Person 3	Class 4	Activity	
Person 3	Class 4	After	
Person 3	Class 5	Before	
Person 3	Class 5	Activity	
Person 3	Class 5	After	
Person 3	Class 6	Before	74.0772300443238
Person 3	Class 6	Activity	70.0969270140209
Person 3	Class 6	After	72.0063967109904
Person 3	Class 7	Before	75.8063967109905
Person 3	Class 7	Activity	74.372476735058
Person 3	Class 7	After	75.6438967109905
Person 3	Class 8	Before	68.6706353201034
Person 3	Class 8	Activity	71.1462776633714
Person 3	Class 8	After	68.3563967109904
Person 3	Class 9	Before	70.1480633776571
Person 3	Class 9	Activity	69.990293107387
Person 3	Class 9	After	69.8772300443237
Person 4	Class 1	Before	
Person 4	Class 1	Activity	
Person 4	Class 1	After	
Person 4	Class 10	Before	70.3625860598534
Person 4	Class 10	Activity	73.1994784228215
Person 4	Class 10	After	69.8619784228215
Person 4	Class 11	Before	65.6828117561548
Person 4	Class 11	Activity	67.6690771088518

Person 4	Class 11	After	61.2869784228215
Person 4	Class 12	Before	75.8753521060023
Person 4	Class 12	Activity	72.5066021060024
Person 4	Class 12	After	71.1086854393357
Person 4	Class 13	Before	67.7170187726691
Person 4	Class 13	Activity	73.651954670105
Person 4	Class 13	After	63.3961854393357
Person 4	Class 14	Before	73.7781183886549
Person 4	Class 14	Activity	75.4380791796237
Person 4	Class 14	After	71.7027750758237
Person 4	Class 15	Before	65.782255299096
Person 4	Class 15	Activity	69.3918369370779
Person 4	Class 15	After	70.6280886324294
Person 4	Class 16	Before	69.0747965165065
Person 4	Class 16	Activity	70.8090745192054
Person 4	Class 16	After	65.951399080609
Person 4	Class 17	Before	74.431703973253
Person 4	Class 17	Activity	77.466298022304
Person 4	Class 17	After	73.5454646889706
Person 4	Class 18	Before	67.1820432067147
Person 4	Class 18	Activity	66.7912630648703
Person 4	Class 18	After	66.848117108467
Person 4	Class 19	Before	70.0227622812286
Person 4	Class 19	Activity	74.5447584052601
Person 4	Class 19	After	67.8060956145619
Person 4	Class 2	Before	
Person 4	Class 2	Activity	
Person 4	Class 2	After	
Person 4	Class 20	Before	
Person 4	Class 20	Activity	
Person 4	Class 20	After	
Person 4	Class 21	Before	
Person 4	Class 21	Activity	
Person 4	Class 21	After	
Person 4	Class 22	Before	79.3478983761387
Person 4	Class 22	Activity	73.3890585993317
Person 4	Class 22	After	77.9202858491246
Person 4	Class 3	Before	72.9239854488027
Person 4	Class 3	Activity	74.9180669828402
Person 4	Class 3	After	72.5954652319464
Person 4	Class 4	Before	80.5302530202922
Person 4	Class 4	Activity	87.6285107188329
Person 4	Class 4	After	81.6238722671542
Person 4	Class 5	Before	81.2219196869589
Person 4	Class 5	Activity	88.4695387345783

Person 4	Class 5	After	80.1844196869589
Person 4	Class 6	Before	74.2698789419915
Person 4	Class 6	Activity	73.1500738563559
Person 4	Class 6	After	72.9973507857843
Person 4	Class 7	Before	69.1181841191176
Person 4	Class 7	Activity	73.9769128817543
Person 4	Class 7	After	69.9558051389471
Person 4	Class 8	Before	73.4640343403879
Person 4	Class 8	Activity	74.1035947799483
Person 4	Class 8	After	71.4708566847102
Person 4	Class 9	Before	73.3000233513769
Person 4	Class 9	Activity	71.6000233513769
Person 4	Class 9	After	72.0046957334166
Person 5	Class 1	Before	74.6145152987143
Person 5	Class 1	Activity	74.4547930764923
Person 5	Class 1	After	68.8494738049383
Person 5	Class 10	Before	
Person 5	Class 10	Activity	
Person 5	Class 10	After	
Person 5	Class 11	Before	
Person 5	Class 11	Activity	
Person 5	Class 11	After	
Person 5	Class 12	Before	78.5808611474832
Person 5	Class 12	Activity	75.3121684899476
Person 5	Class 12	After	72.8982413435549
Person 5	Class 13	Before	75.8919219808541
Person 5	Class 13	Activity	77.4829682703467
Person 5	Class 13	After	70.2305539416472
Person 5	Class 14	Before	68.968675064186
Person 5	Class 14	Activity	75.1811038166856
Person 5	Class 14	After	71.5652876502923
Person 5	Class 15	Before	66.7730993514014
Person 5	Class 15	Activity	69.8112357598288
Person 5	Class 15	After	66.649313528337
Person 5	Class 16	Before	
Person 5	Class 16	Activity	
Person 5	Class 16	After	
Person 5	Class 17	Before	70.2568440884269
Person 5	Class 17	Activity	71.2360107550933
Person 5	Class 17	After	72.5526774217602
Person 5	Class 18	Before	
Person 5	Class 18	Activity	
Person 5	Class 18	After	
Person 5	Class 19	Before	70.6251092168537
Person 5	Class 19	Activity	74.0403634610334

Person 5	Class 19	After	69.5765192800632
Person 5	Class 2	Before	64.0344943782121
Person 5	Class 2	Activity	75.9795147146808
Person 5	Class 2	After	71.0929429211129
Person 5	Class 20	Before	
Person 5	Class 20	Activity	
Person 5	Class 20	After	
Person 5	Class 21	Before	
Person 5	Class 21	Activity	
Person 5	Class 21	After	
Person 5	Class 22	Before	71.3097540514452
Person 5	Class 22	Activity	75.7315677107375
Person 5	Class 22	After	71.5278502857549
Person 5	Class 3	Before	69.7587965894864
Person 5	Class 3	Activity	73.8573384195688
Person 5	Class 3	After	74.566669199439
Person 5	Class 4	Before	80.638107650338
Person 5	Class 4	Activity	85.7763842575773
Person 5	Class 4	After	85.3126543727927
Person 5	Class 5	Before	81.4047743170047
Person 5	Class 5	Activity	85.3590246540156
Person 5	Class 5	After	76.5962238388089
Person 5	Class 6	Before	77.6591611542818
Person 5	Class 6	Activity	76.5043157921521
Person 5	Class 6	After	61.1548611705626
Person 5	Class 7	Before	70.258910845494
Person 5	Class 7	Activity	75.9953652348828
Person 5	Class 7	After	67.5173611705626
Person 5	Class 8	Before	65.1672112904669
Person 5	Class 8	Activity	70.5445714819327
Person 5	Class 8	After	65.1048611705625
Person 5	Class 9	Before	64.0381945038959
Person 5	Class 9	Activity	65.0722035129051
Person 5	Class 9	After	71.1006016547715
Person 6	Class 1	Before	80.8396208932654
Person 6	Class 1	Activity	77.4125375599321
Person 6	Class 1	After	74.6136527051603
Person 6	Class 10	Before	69.9208100785879
Person 6	Class 10	Activity	67.7666434119213
Person 6	Class 10	After	69.7833100785879
Person 6	Class 11	Before	66.7802690560205
Person 6	Class 11	Activity	65.7095005547784
Person 6	Class 11	After	60.0541434119212
Person 6	Class 12	Before	
Person 6	Class 12	Activity	

Person 6	Class 12	After	
Person 6	Class 13	Before	
Person 6	Class 13	Activity	
Person 6	Class 13	After	
Person 6	Class 14	Before	
Person 6	Class 14	Activity	
Person 6	Class 14	After	
Person 6	Class 15	Before	
Person 6	Class 15	Activity	
Person 6	Class 15	After	
Person 6	Class 16	Before	79.2758349438439
Person 6	Class 16	Activity	73.2055245969737
Person 6	Class 16	After	79.7625118549318
Person 6	Class 17	Before	76.1637379905985
Person 6	Class 17	Activity	81.1827421114594
Person 6	Class 17	After	73.2674643336817
Person 6	Class 18	Before	
Person 6	Class 18	Activity	
Person 6	Class 18	After	
Person 6	Class 19	Before	72.01441789765
Person 6	Class 19	Activity	80.9484233392289
Person 6	Class 19	After	71.7251546354084
Person 6	Class 2	Before	73.8833796101413
Person 6	Class 2	Activity	71.4861262696095
Person 6	Class 2	After	70.9391387996015
Person 6	Class 20	Before	71.444479850696
Person 6	Class 20	Activity	68.5064000733303
Person 6	Class 20	After	68.4368160944961
Person 6	Class 21	Before	68.5451494278295
Person 6	Class 21	Activity	66.8642775385574
Person 6	Class 21	After	66.781979850696
Person 6	Class 22	Before	78.6763707680432
Person 6	Class 22	Activity	81.7901098709215
Person 6	Class 22	After	75.7289160957978
Person 6	Class 3	Before	74.6546136561854
Person 6	Class 3	Activity	73.2247144098902
Person 6	Class 3	After	75.269697918774
Person 6	Class 4	Before	
Person 6	Class 4	Activity	
Person 6	Class 4	After	
Person 6	Class 5	Before	
Person 6	Class 5	Activity	
Person 6	Class 5	After	
Person 6	Class 6	Before	80.9704506740181
Person 6	Class 6	Activity	79.0165553995143

Person 6	Class 6	After	76.3579506740181
Person 6	Class 7	Before	75.5496173406847
Person 6	Class 7	Activity	76.3566871361119
Person 6	Class 7	After	75.7768989451169
Person 6	Class 8	Before	68.6352888035148
Person 6	Class 8	Activity	65.0593792454468
Person 6	Class 8	After	68.1412840073514
Person 6	Class 9	Before	66.9829506740181
Person 6	Class 9	Activity	69.2234912145586
Person 6	Class 9	After	66.9287840073515
Person 7	Class 1	Before	
Person 7	Class 1	Activity	
Person 7	Class 1	After	
Person 7	Class 10	Before	69.2813603736529
Person 7	Class 10	Activity	68.2210474104208
Person 7	Class 10	After	67.9113251881985
Person 7	Class 11	Before	67.8646773496618
Person 7	Class 11	Activity	67.275015664389
Person 7	Class 11	After	66.5907933092414
Person 7	Class 12	Before	78.5125048421154
Person 7	Class 12	Activity	75.4797198749722
Person 7	Class 12	After	72.189243684496
Person 7	Class 13	Before	71.4556724967193
Person 7	Class 13	Activity	77.0969359921883
Person 7	Class 13	After	73.1310251814802
Person 7	Class 14	Before	72.214243684496
Person 7	Class 14	Activity	71.0652066176133
Person 7	Class 14	After	70.6309103511626
Person 7	Class 15	Before	71.4723367179712
Person 7	Class 15	Activity	72.6494992220705
Person 7	Class 15	After	70.3434103511627
Person 7	Class 16	Before	67.9268231055055
Person 7	Class 16	Activity	64.5164317424822
Person 7	Class 16	After	69.7591949003773
Person 7	Class 17	Before	
Person 7	Class 17	Activity	
Person 7	Class 17	After	
Person 7	Class 18	Before	68.7892622215711
Person 7	Class 18	Activity	65.2260756064585
Person 7	Class 18	After	66.2432741384622
Person 7	Class 19	Before	
Person 7	Class 19	Activity	
Person 7	Class 19	After	
Person 7	Class 2	Before	
Person 7	Class 2	Activity	

Person 7	Class 2	After	
Person 7	Class 20	Before	
Person 7	Class 20	Activity	
Person 7	Class 20	After	
Person 7	Class 21	Before	
Person 7	Class 21	Activity	
Person 7	Class 21	After	
Person 7	Class 22	Before	
Person 7	Class 22	Activity	
Person 7	Class 22	After	
Person 7	Class 3	Before	
Person 7	Class 3	Activity	
Person 7	Class 3	After	
Person 7	Class 4	Before	72.7296592980314
Person 7	Class 4	Activity	71.4103454136446
Person 7	Class 4	After	77.6036481404303
Person 7	Class 5	Before	65.3046592980313
Person 7	Class 5	Activity	70.9346376530103
Person 7	Class 5	After	73.1635430275071
Person 7	Class 6	Before	
Person 7	Class 6	Activity	
Person 7	Class 6	After	
Person 7	Class 7	Before	68.8005509918678
Person 7	Class 7	Activity	73.3649561147224
Person 7	Class 7	After	68.3838843252012
Person 7	Class 8	Before	70.6613123827549
Person 7	Class 8	Activity	69.3170986109153
Person 7	Class 8	After	69.9443870283205
Person 7	Class 9	Before	76.9636535313953
Person 7	Class 9	Activity	71.7538167576336
Person 7	Class 9	After	69.6505509918678
Person 8	Class 1	Before	77.5457845956449
Person 8	Class 1	Activity	77.1409234845336
Person 8	Class 1	After	74.754498288591
Person 8	Class 10	Before	67.547443439296
Person 8	Class 10	Activity	67.2016101059627
Person 8	Class 10	After	63.659943439296
Person 8	Class 11	Before	64.8307767726293
Person 8	Class 11	Activity	64.8135148678674
Person 8	Class 11	After	64.2250480605045
Person 8	Class 12	Before	75.8674693607308
Person 8	Class 12	Activity	78.185620284379
Person 8	Class 12	After	72.1058641370019
Person 8	Class 13	Before	75.9344298081885
Person 8	Class 13	Activity	73.6305836543423

Person 8	Class 13	After	71.8677631415219
Person 8	Class 14	Before	71.8543036768814
Person 8	Class 14	Activity	75.1619491880335
Person 8	Class 14	After	77.4321003612233
Person 8	Class 15	Before	66.6677631415218
Person 8	Class 15	Activity	67.5177631415216
Person 8	Class 15	After	70.2805120591916
Person 8	Class 16	Before	72.5666302577261
Person 8	Class 16	Activity	72.6678448326249
Person 8	Class 16	After	74.0327000660353
Person 8	Class 17	Before	71.4701814707816
Person 8	Class 17	Activity	68.5271259152264
Person 8	Class 17	After	76.8701814707817
Person 8	Class 18	Before	71.4278747126253
Person 8	Class 18	Activity	72.4108802393307
Person 8	Class 18	After	69.5125646364938
Person 8	Class 19	Before	69.4647463838124
Person 8	Class 19	Activity	74.9562566405581
Person 8	Class 19	After	69.2335441635118
Person 8	Class 2	Before	69.1889668611604
Person 8	Class 2	Activity	70.7691716924192
Person 8	Class 2	After	69.2094209592814
Person 8	Class 20	Before	
Person 8	Class 20	Activity	
Person 8	Class 20	After	
Person 8	Class 21	Before	
Person 8	Class 21	Activity	
Person 8	Class 21	After	
Person 8	Class 22	Before	
Person 8	Class 22	Activity	
Person 8	Class 22	After	
Person 8	Class 3	Before	73.0061066774389
Person 8	Class 3	Activity	72.3208502671824
Person 8	Class 3	After	72.2049869779825
Person 8	Class 4	Before	
Person 8	Class 4	Activity	
Person 8	Class 4	After	
Person 8	Class 5	Before	
Person 8	Class 5	Activity	
Person 8	Class 5	After	
Person 8	Class 6	Before	
Person 8	Class 6	Activity	
Person 8	Class 6	After	
Person 8	Class 7	Before	
Person 8	Class 7	Activity	

Person 8	Class 7	After	
Person 8	Class 8	Before	
Person 8	Class 8	Activity	
Person 8	Class 8	After	
Person 8	Class 9	Before	
Person 8	Class 9	Activity	
Person 8	Class 9	After	
Person 9	Class 1	Before	74.6509594137401
Person 9	Class 1	Activity	73.9974871915176
Person 9	Class 1	After	71.6617062822329
Person 9	Class 10	Before	65.2905358692702
Person 9	Class 10	Activity	66.948869202604
Person 9	Class 10	After	69.3280358692702
Person 9	Class 11	Before	69.7238692026036
Person 9	Class 11	Activity	74.1085554330127
Person 9	Class 11	After	63.7863692026036
Person 9	Class 12	Before	
Person 9	Class 12	Activity	
Person 9	Class 12	After	
Person 9	Class 13	Before	
Person 9	Class 13	Activity	
Person 9	Class 13	After	
Person 9	Class 14	Before	71.2006734358114
Person 9	Class 14	Activity	72.2401411878199
Person 9	Class 14	After	73.9006734358114
Person 9	Class 15	Before	72.3708091103191
Person 9	Class 15	Activity	75.2999158600539
Person 9	Class 15	After	72.9356249294106
Person 9	Class 16	Before	
Person 9	Class 16	Activity	
Person 9	Class 16	After	
Person 9	Class 17	Before	
Person 9	Class 17	Activity	
Person 9	Class 17	After	
Person 9	Class 18	Before	
Person 9	Class 18	Activity	
Person 9	Class 18	After	
Person 9	Class 19	Before	
Person 9	Class 19	Activity	
Person 9	Class 19	After	
Person 9	Class 2	Before	69.8446658293608
Person 9	Class 2	Activity	74.9270809968239
Person 9	Class 2	After	75.8320326598752
Person 9	Class 20	Before	61.5700967149184
Person 9	Class 20	Activity	67.5450985316081

Person 9	Class 20	After	67.1700967149184
Person 9	Class 21	Before	67.2992633815851
Person 9	Class 21	Activity	66.7345475819705
Person 9	Class 21	After	62.4700967149184
Person 9	Class 22	Before	
Person 9	Class 22	Activity	
Person 9	Class 22	After	
Person 9	Class 3	Before	
Person 9	Class 3	Activity	
Person 9	Class 3	After	
Person 9	Class 4	Before	
Person 9	Class 4	Activity	
Person 9	Class 4	After	
Person 9	Class 5	Before	
Person 9	Class 5	Activity	
Person 9	Class 5	After	
Person 9	Class 6	Before	76.8528201082781
Person 9	Class 6	Activity	75.6659244758955
Person 9	Class 6	After	76.6861534416114
Person 9	Class 7	Before	74.1111534416114
Person 9	Class 7	Activity	77.4590174608654
Person 9	Class 7	After	72.7194867749447
Person 9	Class 8	Before	66.1579759835774
Person 9	Class 8	Activity	70.8864650656727
Person 9	Class 8	After	68.4486534416114
Person 9	Class 9	Before	64.7861534416113
Person 9	Class 9	Activity	71.2477212552086
Person 9	Class 9	After	62.815320108278
