# Supplementary material for: Student engagement, assessed using heart rate, shows no reset following active learning sessions in lectures
Source: PLoS One. 2019 Dec 2;14(12):e0225709. doi: 10.1371/journal.pone.0225709 (PMC6886849; doi:10.1371/journal.pone.0225709)
Supplement: S1 Table — (PDF) [file pone.0225709.s004.pdf]

S1 Table: Statistical output (R) from Activity ANOVAs

DATASET = S3 Dataset   # S3 Dataset is a data frame with 4 columns

column "who" is the person number (nuisance variable)

column "when" is the activity identifier (nuisance variable)

"what" is the variable of interest, taking values "Before", "Activity", or "After"

"rate" is the response variable, the average heartrate

one-way ANOVA for unbalanced data

ANOVA for three-way comparison of HR vs. Before/Activity/After

Response:

RATE	SumSq	Df	F-value	Pr(>F)	P>
(Intercept)	98784	1	80066.96	<2.2E-16	***
what	583	2	23.79	1.20E-10	***
who	578	15	3.15	5.65E-05	***
when	5394	21	20.98	<2.2E-16	***
Residuals	6894	563			

Interpretation: Heartrates are significantly different for 'what' values

two-way comparison: Activity vs. After (not Before)

Type III test

Response:

RATE	SumSq	Df	F-value	Pr(>F)	P>
(Intercept)	67223	1	5028.86	<2.2E-16	***
what	573	1	42.84	2.03E-10	***
who	389	15	1.94	1.90E-02	*
when	5394	21	20.98	<2.2E-16	***
Residuals	4852	363			

Interpretation: Heartrates are significantly different for Activity vs. After

two-way comparison: Before vs. After (not Activity)

Type III test

Response:

RATE	SumSq	Df	F-value	Pr(>F)	P>
(Intercept)	63119	1	6087.85	<2.2E-16	***
what	85	1	8.19	4.50E-03	**
who	541	15	3.48	1.40E-05	***
when	3308	21	15.19	<2.2E-16	***
Residuals	3764	363			

Interpretation: Heartrates are significantly different for Before vs. After

two-way comparison: Before vs. Activity (not After)

Type III test

Response:

RATE	SumSq	Df	F-value	Pr(>F)	P>
(Intercept)	68773	1	5236.05	<2.2E-16	***
what	218	1	16.57	5.75E-05	***
who	401	15	2.035	1.25E-02	*
when	3928	21	14.24	<2.2E-16	***
Residuals	4781	364			

Interpretation: Heartrates are significantly different for Before vs. Activity
