# Supplementary material for: Student engagement, assessed using heart rate, shows no reset following active learning sessions in lectures
Source: PLoS One. 2019 Dec 2;14(12):e0225709. doi: 10.1371/journal.pone.0225709 (PMC6886849; doi:10.1371/journal.pone.0225709)
Supplement: S2 Table — (PDF) [file pone.0225709.s005.pdf]

S2 Table Y intercepts for before and after breaks (same instructor).

FACULTY	Y intercept (I	slope/sec	total seconds	Y2 intercept	Drop	Between class
	cropped	cropped				
1A	79.061	-0.0018	2955	73.742	-5.319	0.648
1B	74.39	-0.0021	3206	67.6574	-6.7326	
2A	73.391	-0.0013	2820	69.725	-3.666	
2B	78.173	-0.0007	4853	74.7759	-3.3971	
3A	75.027	-0.002	3171	68.685	-6.342	0.837
3B	69.522	-0.0009	2770	67.029	-2.493	
4A	76.932	-0.0025	3234	68.847	-8.085	
4B	67.402	0.0003	3884	68.5672	1.1652	
5A	76.647	-0.0019	3103	70.7513	-5.8957	
5B	70.283	-0.0005	4368	68.099	-2.184	
6A	69.102	0.0008	**2448**	71.0604	1.9584	
6B	68.773	-0.0005	1644	67.951	-0.822	
7A	76.668	-0.0007	2409	74.9817	-1.6863	-1.9727
7B	73.009	-0.0019	2040	69.133	-3.876	
8A	75.86	-0.0012	4555	70.394	-5.466	
8B	75.945	-0.0016	2484	71.9706	-3.9744	
9A	71.732	-0.0004	1680	71.06	-0.672	
9B	69.599	-0.0015	3281	64.6775	-4.9215	
10A	71.33	-0.0013	3243	67.1141	-4.2159	
10B	70.026	-0.0012	3052	66.3636	-3.6624	0.6434
11A	67.007	-0.0002	3013	66.4044	-0.6026	
11B	79.531	-0.0031	2899	70.5441	-8.9869	3.4869
12A	74.031	-0.0018	3166	68.3322	-5.6988	
12B	72.395	-0.0014	2575	68.79	-3.605	-1.56
13A	67.23	0.0005	2810	68.635	1.405	
13B	74.384	-0.0007	3147	72.1811	-2.2029	
4A	75.526	-0.0014	3147	71.1202	-4.4058	
4B	69.672	0.0016	1887	72.6912	3.0192	
15A	70.959	-0.0009	3252	68.0322	-2.9268	
15B	74.095	-0.0014	4340	68.019	-6.076	
16A	76.457	-0.0035	2313	68.3615	-8.0955	
16B	70.016	-0.001	1639	68.377	-1.639	
17A	72.676	-0.0009	3105	69.8815	-2.7945	-2.3715
17B	67.51	-0.0006	3058	65.6752	-1.8348	
18A	64.034	-0.0003	3567	62.9639	-1.0701	
18B	68.356	-0.0006	3003	66.5542	-1.8018	
19A	69.004	-0.0006	3177	67.0978	-1.9062	-1.7258
19B	65.372	-0.0005	2974	63.885	-1.487	

20A	70.657	-0.0018	**3354**	64.6198	-6.0372		1.3112
20B	65.931	-0.0012	**3241**	62.0418	-3.8892		-0.7828
21A	61.259	0.0009	**2477**	63.4883	2.2293		
21B	74.782	-0.0009	**4862**	70.4062	-4.3758		
22A	74.84	-0.001	**3229**	71.611	-3.229		
22B	74.782	-0.0009	**1200**	73.702	-1.08		
23A	75.937	-0.001	**4189**	71.748	-4.189		
23B	76.991	-0.0022	**2545**	71.392	-5.599		
24A	75.937	-0.001	**1225**	74.712	-1.225		
24B	67.559	0.0006	**3306**	69.5426	1.9836		
							0.244375
						gray	
							-0.14863

within class

-2.2874

-0.2386

-1.4482

1.6545

3.171

4.545

all
0.89938333
red
