# Supplementary material for: Student engagement, assessed using heart rate, shows no reset following active learning sessions in lectures
Source: PLoS One. 2019 Dec 2;14(12):e0225709. doi: 10.1371/journal.pone.0225709 (PMC6886849; doi:10.1371/journal.pone.0225709)
Supplement: S1 Dataset — (PDF) [file pone.0225709.s001.pdf]

	0		Person 1	Person 6	Person 3	Person 5
Time	Time elapsed					
17:02:00	0:00:00		90.6930524	90.3812876	84.731787	68.2395153
17:02:01	0:00:01		89.6930524	91.3812876	84.731787	71.2395153
17:02:02	0:00:02		89.6930524	91.3812876	80.731787	71.2395153
17:02:03	0:00:03		89.6930524	91.3812876	77.731787	74.2395153
17:02:04	0:00:04		90.6930524	91.3812876	74.731787	76.2395153
17:02:05	0:00:05		89.6930524	92.3812876	75.731787	78.2395153
17:02:06	0:00:06		88.6930524	92.3812876	75.731787	78.2395153
17:02:07	0:00:07		86.6930524	92.3812876	76.731787	79.2395153
17:02:08	0:00:08		86.6930524	92.3812876	76.731787	79.2395153
17:02:09	0:00:09		85.6930524	92.3812876	76.731787	79.2395153
17:02:10	0:00:10		85.6930524	92.3812876	76.731787	80.2395153
17:02:11	0:00:11		83.6930524	93.3812876	79.731787	80.2395153
17:02:12	0:00:12		81.6930524	93.3812876	80.731787	84.2395153
17:02:13	0:00:13		81.6930524	93.3812876	80.731787	86.2395153
17:02:14	0:00:14		81.6930524	94.3812876	80.731787	86.2395153
17:02:15	0:00:15		81.6930524	94.3812876	80.731787	83.2395153
17:02:16	0:00:16		80.6930524	93.3812876	79.731787	82.2395153
17:02:17	0:00:17		81.6930524	93.3812876	79.731787	81.2395153
17:02:18	0:00:18		81.6930524	94.3812876	79.731787	79.2395153
17:02:19	0:00:19		81.6930524	94.3812876	79.731787	79.2395153
17:02:20	0:00:20		82.6930524	94.3812876	78.731787	76.2395153
17:02:21	0:00:21		82.6930524	94.3812876	80.731787	74.2395153
17:02:22	0:00:22		82.6930524	95.3812876	79.731787	72.2395153
17:02:23	0:00:23		83.6930524	95.3812876	79.731787	72.2395153
17:02:24	0:00:24		83.6930524	95.3812876	79.731787	72.2395153
17:02:25	0:00:25		84.6930524	96.3812876	78.731787	73.2395153
17:02:26	0:00:26		86.6930524	97.3812876	78.731787	73.2395153
17:02:27	0:00:27		88.6930524	97.3812876	78.731787	73.2395153
17:02:28	0:00:28		90.6930524	97.3812876	78.731787	75.2395153
17:02:29	0:00:29		91.6930524	101.381288	78.731787	75.2395153
17:02:30	0:00:30		91.6930524	100.381288	78.731787	76.2395153
17:02:31	0:00:31		91.6930524	100.381288	76.731787	82.2395153
17:02:32	0:00:32		92.6930524	100.381288	76.731787	82.2395153
17:02:33	0:00:33		93.6930524	100.381288	76.731787	82.2395153
17:02:34	0:00:34		93.6930524	98.3812876	73.731787	83.2395153
17:02:35	0:00:35		93.6930524	95.3812876	73.731787	83.2395153
17:02:36	0:00:36		93.6930524	93.3812876	73.731787	83.2395153
17:02:37	0:00:37		94.6930524	93.3812876	73.731787	81.2395153
17:02:38	0:00:38		94.6930524	91.3812876	73.731787	78.2395153
17:02:39	0:00:39		95.6930524	90.3812876	73.731787	78.2395153
17:02:40	0:00:40		95.6930524	87.3812876	73.731787	78.2395153
17:02:41	0:00:41		95.6930524	84.3812876	74.731787	80.2395153
17:02:42	0:00:42		95.6930524	84.3812876	83.731787	81.2395153

17:02:43	0:00:43		95.6930524	74.3812876	84.731787	81.2395153
17:02:44	0:00:44		95.6930524	74.3812876	84.731787	80.2395153
17:02:45	0:00:45		95.6930524	74.3812876	83.731787	80.2395153
17:02:46	0:00:46		94.6930524	74.3812876	79.731787	78.2395153
17:02:47	0:00:47		94.6930524	74.3812876	76.731787	78.2395153
17:02:48	0:00:48		94.6930524	75.3812876	76.731787	77.2395153
17:02:49	0:00:49		93.6930524	75.3812876	76.731787	76.2395153
17:02:50	0:00:50		93.6930524	78.3812876	77.731787	77.2395153
17:02:51	0:00:51		93.6930524	79.3812876	78.731787	78.2395153
17:02:52	0:00:52		92.6930524	82.3812876	80.731787	78.2395153
17:02:53	0:00:53		92.6930524	82.3812876	80.731787	78.2395153
17:02:54	0:00:54		93.6930524	84.3812876	80.731787	84.2395153
17:02:55	0:00:55		101.693052	87.3812876	79.731787	84.2395153
17:02:56	0:00:56		101.693052	89.3812876	79.731787	84.2395153
17:02:57	0:00:57		103.693052	89.3812876	79.731787	84.2395153
17:02:58	0:00:58		90.6930524	93.3812876	79.731787	84.2395153
17:02:59	0:00:59		90.6930524	93.3812876	79.731787	84.2395153
17:03:00	0:01:00		88.6930524	93.3812876	79.731787	85.2395153
17:03:01	0:01:01		88.6930524	90.3812876	79.731787	85.2395153
17:03:02	0:01:02		88.6930524	89.3812876	79.731787	85.2395153
17:03:03	0:01:03		88.6930524	89.3812876	76.731787	86.2395153
17:03:04	0:01:04		90.6930524	89.3812876	76.731787	87.2395153
17:03:05	0:01:05		91.6930524	89.3812876	76.731787	88.2395153
17:03:06	0:01:06		91.6930524	89.3812876	76.731787	88.2395153
17:03:07	0:01:07		92.6930524	89.3812876	76.731787	83.2395153
17:03:08	0:01:08		92.6930524	89.3812876	76.731787	83.2395153
17:03:09	0:01:09		92.6930524	88.3812876	75.731787	84.2395153
17:03:10	0:01:10		92.6930524	87.3812876	77.731787	84.2395153
17:03:11	0:01:11		92.6930524	84.3812876	77.731787	83.2395153
17:03:12	0:01:12		92.6930524	84.3812876	77.731787	83.2395153
17:03:13	0:01:13		88.6930524	85.3812876	77.731787	83.2395153
17:03:14	0:01:14		88.6930524	85.3812876	77.731787	83.2395153
17:03:15	0:01:15		84.6930524	85.3812876	76.731787	82.2395153
17:03:16	0:01:16		83.6930524	85.3812876	76.731787	82.2395153
17:03:17	0:01:17		82.6930524	85.3812876	75.731787	82.2395153
17:03:18	0:01:18		82.6930524	85.3812876	75.731787	82.2395153
17:03:19	0:01:19		82.6930524	85.3812876	75.731787	82.2395153
17:03:20	0:01:20		79.6930524	85.3812876	75.731787	79.2395153
17:03:21	0:01:21		78.6930524	89.3812876	75.731787	79.2395153
17:03:22	0:01:22		78.6930524	89.3812876	75.731787	76.2395153
17:03:23	0:01:23		79.6930524	89.3812876	75.731787	77.2395153
17:03:24	0:01:24		79.6930524	90.3812876	75.731787	77.2395153
17:03:25	0:01:25		79.6930524	90.3812876	74.731787	77.2395153
17:03:26	0:01:26		79.6930524	85.3812876	72.731787	77.2395153
17:03:27	0:01:27		80.6930524	85.3812876	72.731787	77.2395153

17:03:28	0:01:28		80.6930524	85.3812876	71.731787	75.2395153
17:03:29	0:01:29		80.6930524	83.3812876	72.731787	75.2395153
17:03:30	0:01:30		80.6930524	83.3812876	72.731787	73.2395153
17:03:31	0:01:31		79.6930524	83.3812876	72.731787	73.2395153
17:03:32	0:01:32		79.6930524	83.3812876	73.731787	73.2395153
17:03:33	0:01:33		78.6930524	84.3812876	73.731787	71.2395153
17:03:34	0:01:34		78.6930524	84.3812876	73.731787	70.2395153
17:03:35	0:01:35		79.6930524	85.3812876	73.731787	71.2395153
17:03:36	0:01:36		79.6930524	83.3812876	73.731787	71.2395153
17:03:37	0:01:37		79.6930524	83.3812876	72.731787	71.2395153
17:03:38	0:01:38		79.6930524	82.3812876	72.731787	71.2395153
17:03:39	0:01:39		79.6930524	82.3812876	73.731787	71.2395153
17:03:40	0:01:40		80.6930524	82.3812876	74.731787	70.2395153
17:03:41	0:01:41		80.6930524	82.3812876	74.731787	70.2395153
17:03:42	0:01:42		80.6930524	82.3812876	74.731787	69.2395153
17:03:43	0:01:43		80.6930524	82.3812876	75.731787	69.2395153
17:03:44	0:01:44		80.6930524	82.3812876	75.731787	69.2395153
17:03:45	0:01:45		79.6930524	82.3812876	75.731787	69.2395153
17:03:46	0:01:46		78.6930524	81.3812876	75.731787	70.2395153
17:03:47	0:01:47		78.6930524	81.3812876	75.731787	70.2395153
17:03:48	0:01:48		78.6930524	81.3812876	75.731787	70.2395153
17:03:49	0:01:49		78.6930524	81.3812876	76.731787	70.2395153
17:03:50	0:01:50		78.6930524	83.3812876	76.731787	72.2395153
17:03:51	0:01:51		78.6930524	83.3812876	76.731787	73.2395153
17:03:52	0:01:52		79.6930524	83.3812876	76.731787	74.2395153
17:03:53	0:01:53		79.6930524	83.3812876	76.731787	75.2395153
17:03:54	0:01:54		80.6930524	86.3812876	76.731787	75.2395153
17:03:55	0:01:55		80.6930524	86.3812876	76.731787	75.2395153
17:03:56	0:01:56		80.6930524	86.3812876	75.731787	75.2395153
17:03:57	0:01:57		80.6930524	87.3812876	75.731787	75.2395153
17:03:58	0:01:58		80.6930524	87.3812876	76.731787	76.2395153
17:03:59	0:01:59		80.6930524	87.3812876	76.731787	76.2395153
17:04:00	0:02:00		80.6930524	88.3812876	76.731787	77.2395153
17:04:01	0:02:01		80.6930524	90.3812876	77.731787	78.2395153
17:04:02	0:02:02		80.6930524	90.3812876	77.731787	80.2395153
17:04:03	0:02:03		80.6930524	90.3812876	77.731787	81.2395153
17:04:04	0:02:04		80.6930524	90.3812876	77.731787	81.2395153
17:04:05	0:02:05		80.6930524	89.3812876	77.731787	81.2395153
17:04:06	0:02:06		80.6930524	87.3812876	77.731787	80.2395153
17:04:07	0:02:07		80.6930524	85.3812876	81.731787	79.2395153
17:04:08	0:02:08		81.6930524	85.3812876	81.731787	79.2395153
17:04:09	0:02:09		80.6930524	85.3812876	81.731787	80.2395153
17:04:10	0:02:10		80.6930524	85.3812876	77.731787	81.2395153
17:04:11	0:02:11		80.6930524	86.3812876	76.731787	82.2395153
17:04:12	0:02:12		80.6930524	86.3812876	76.731787	82.2395153

17:04:13	0:02:13		81.6930524	85.3812876	75.731787	83.2395153
17:04:14	0:02:14		81.6930524	85.3812876	75.731787	83.2395153
17:04:15	0:02:15		81.6930524	85.3812876	75.731787	83.2395153
17:04:16	0:02:16		82.6930524	85.3812876	75.731787	83.2395153
17:04:17	0:02:17		83.6930524	86.3812876	75.731787	83.2395153
17:04:18	0:02:18		83.6930524	86.3812876	75.731787	81.2395153
17:04:19	0:02:19		83.6930524	86.3812876	74.731787	80.2395153
17:04:20	0:02:20		82.6930524	86.3812876	74.731787	79.2395153
17:04:21	0:02:21		82.6930524	86.3812876	74.731787	80.2395153
17:04:22	0:02:22		82.6930524	86.3812876	74.731787	80.2395153
17:04:23	0:02:23		81.6930524	86.3812876	74.731787	80.2395153
17:04:24	0:02:24		81.6930524	85.3812876	74.731787	79.2395153
17:04:25	0:02:25		81.6930524	85.3812876	74.731787	78.2395153
17:04:26	0:02:26		82.6930524	86.3812876	73.731787	78.2395153
17:04:27	0:02:27		82.6930524	87.3812876	73.731787	78.2395153
17:04:28	0:02:28		82.6930524	87.3812876	73.731787	78.2395153
17:04:29	0:02:29		82.6930524	88.3812876	73.731787	77.2395153
17:04:30	0:02:30		82.6930524	88.3812876	75.731787	78.2395153
17:04:31	0:02:31		83.6930524	90.3812876	76.731787	78.2395153
17:04:32	0:02:32		83.6930524	90.3812876	76.731787	77.2395153
17:04:33	0:02:33		83.6930524	90.3812876	76.731787	75.2395153
17:04:34	0:02:34		84.6930524	91.3812876	76.731787	74.2395153
17:04:35	0:02:35		84.6930524	91.3812876	75.731787	74.2395153
17:04:36	0:02:36		84.6930524	91.3812876	75.731787	74.2395153
17:04:37	0:02:37		84.6930524	91.3812876	74.731787	74.2395153
17:04:38	0:02:38		84.6930524	92.3812876	74.731787	74.2395153
17:04:39	0:02:39		84.6930524	93.3812876	74.731787	73.2395153
17:04:40	0:02:40		84.6930524	93.3812876	74.731787	73.2395153
17:04:41	0:02:41		87.6930524	93.3812876	73.731787	71.2395153
17:04:42	0:02:42		87.6930524	94.3812876	73.731787	69.2395153
17:04:43	0:02:43		87.6930524	94.3812876	73.731787	69.2395153
17:04:44	0:02:44		88.6930524	93.3812876	73.731787	68.2395153
17:04:45	0:02:45		88.6930524	92.3812876	73.731787	68.2395153
17:04:46	0:02:46		88.6930524	92.3812876	73.731787	68.2395153
17:04:47	0:02:47		89.6930524	92.3812876	73.731787	67.2395153
17:04:48	0:02:48		89.6930524	92.3812876	73.731787	66.2395153
17:04:49	0:02:49		89.6930524	94.3812876	73.731787	66.2395153
17:04:50	0:02:50		89.6930524	94.3812876	73.731787	66.2395153
17:04:51	0:02:51		90.6930524	93.3812876	73.731787	66.2395153
17:04:52	0:02:52		90.6930524	93.3812876	73.731787	65.2395153
17:04:53	0:02:53		90.6930524	93.3812876	74.731787	65.2395153
17:04:54	0:02:54		90.6930524	93.3812876	74.731787	67.2395153
17:04:55	0:02:55		89.6930524	93.3812876	77.731787	68.2395153
17:04:56	0:02:56		86.6930524	93.3812876	77.731787	70.2395153
17:04:57	0:02:57		86.6930524	93.3812876	81.731787	70.2395153

17:04:58	0:02:58		86.6930524	94.3812876	81.731787	72.2395153
17:04:59	0:02:59		86.6930524	94.3812876	81.731787	72.2395153
17:05:00	0:03:00		85.6930524	94.3812876	77.731787	72.2395153
17:05:01	0:03:01		85.6930524	95.3812876	76.731787	74.2395153
17:05:02	0:03:02		84.6930524	95.3812876	76.731787	74.2395153
17:05:03	0:03:03		84.6930524	95.3812876	76.731787	75.2395153
17:05:04	0:03:04		84.6930524	95.3812876	76.731787	75.2395153
17:05:05	0:03:05		84.6930524	93.3812876	75.731787	77.2395153
17:05:06	0:03:06		84.6930524	93.3812876	75.731787	77.2395153
17:05:07	0:03:07		87.6930524	93.3812876	72.731787	79.2395153
17:05:08	0:03:08		87.6930524	90.3812876	72.731787	79.2395153
17:05:09	0:03:09		87.6930524	89.3812876	73.731787	79.2395153
17:05:10	0:03:10		88.6930524	89.3812876	73.731787	78.2395153
17:05:11	0:03:11		88.6930524	89.3812876	74.731787	78.2395153
17:05:12	0:03:12		88.6930524	89.3812876	74.731787	79.2395153
17:05:13	0:03:13		86.6930524	88.3812876	74.731787	79.2395153
17:05:14	0:03:14		86.6930524	87.3812876	78.731787	79.2395153
17:05:15	0:03:15		84.6930524	87.3812876	82.731787	79.2395153
17:05:16	0:03:16		84.6930524	87.3812876	82.731787	77.2395153
17:05:17	0:03:17		85.6930524	87.3812876	83.731787	77.2395153
17:05:18	0:03:18		84.6930524	87.3812876	83.731787	76.2395153
17:05:19	0:03:19		81.6930524	86.3812876	83.731787	77.2395153
17:05:20	0:03:20		81.6930524	84.3812876	84.731787	77.2395153
17:05:21	0:03:21		80.6930524	82.3812876	85.731787	78.2395153
17:05:22	0:03:22		80.6930524	81.3812876	85.731787	78.2395153
17:05:23	0:03:23		80.6930524	81.3812876	86.731787	79.2395153
17:05:24	0:03:24		80.6930524	81.3812876	86.731787	79.2395153
17:05:25	0:03:25		79.6930524	82.3812876	87.731787	84.2395153
17:05:26	0:03:26		79.6930524	82.3812876	87.731787	85.2395153
17:05:27	0:03:27		79.6930524	81.3812876	87.731787	85.2395153
17:05:28	0:03:28		79.6930524	81.3812876	87.731787	84.2395153
17:05:29	0:03:29		80.6930524	81.3812876	84.731787	84.2395153
17:05:30	0:03:30		81.6930524	81.3812876	84.731787	84.2395153
17:05:31	0:03:31		81.6930524	81.3812876	84.731787	81.2395153
17:05:32	0:03:32		82.6930524	81.3812876	84.731787	80.2395153
17:05:33	0:03:33		82.6930524	82.3812876	83.731787	80.2395153
17:05:34	0:03:34		82.6930524	82.3812876	85.731787	79.2395153
17:05:35	0:03:35		82.6930524	83.3812876	85.731787	79.2395153
17:05:36	0:03:36		81.6930524	83.3812876	85.731787	78.2395153
17:05:37	0:03:37		80.6930524	83.3812876	85.731787	77.2395153
17:05:38	0:03:38		80.6930524	84.3812876	84.731787	75.2395153
17:05:39	0:03:39		79.6930524	84.3812876	84.731787	75.2395153
17:05:40	0:03:40		78.6930524	83.3812876	83.731787	73.2395153
17:05:41	0:03:41		78.6930524	91.3812876	82.731787	73.2395153
17:05:42	0:03:42		78.6930524	91.3812876	81.731787	73.2395153

17:05:43	0:03:43		78.6930524	94.3812876	81.731787	73.2395153
17:05:44	0:03:44		78.6930524	93.3812876	81.731787	73.2395153
17:05:45	0:03:45		79.6930524	89.3812876	81.731787	75.2395153
17:05:46	0:03:46		79.6930524	89.3812876	81.731787	76.2395153
17:05:47	0:03:47		79.6930524	86.3812876	81.731787	77.2395153
17:05:48	0:03:48		79.6930524	85.3812876	81.731787	77.2395153
17:05:49	0:03:49		79.6930524	85.3812876	81.731787	77.2395153
17:05:50	0:03:50		80.6930524	85.3812876	81.731787	77.2395153
17:05:51	0:03:51		80.6930524	85.3812876	80.731787	77.2395153
17:05:52	0:03:52		80.6930524	85.3812876	80.731787	78.2395153
17:05:53	0:03:53		80.6930524	85.3812876	80.731787	78.2395153
17:05:54	0:03:54		80.6930524	85.3812876	79.731787	78.2395153
17:05:55	0:03:55		82.6930524	85.3812876	80.731787	80.2395153
17:05:56	0:03:56		79.6930524	85.3812876	80.731787	80.2395153
17:05:57	0:03:57		78.6930524	85.3812876	80.731787	77.2395153
17:05:58	0:03:58		78.6930524	85.3812876	80.731787	75.2395153
17:05:59	0:03:59		79.6930524	85.3812876	80.731787	75.2395153
17:06:00	0:04:00		79.6930524	85.3812876	80.731787	75.2395153
17:06:01	0:04:01		79.6930524	86.3812876	80.731787	74.2395153
17:06:02	0:04:02		79.6930524	86.3812876	80.731787	74.2395153
17:06:03	0:04:03		78.6930524	86.3812876	79.731787	73.2395153
17:06:04	0:04:04		78.6930524	87.3812876	80.731787	71.2395153
17:06:05	0:04:05		77.6930524	87.3812876	80.731787	69.2395153
17:06:06	0:04:06		77.6930524	87.3812876	80.731787	69.2395153
17:06:07	0:04:07		77.6930524	87.3812876	80.731787	68.2395153
17:06:08	0:04:08		76.6930524	87.3812876	80.731787	68.2395153
17:06:09	0:04:09		76.6930524	87.3812876	80.731787	68.2395153
17:06:10	0:04:10		77.6930524	87.3812876	82.731787	68.2395153
17:06:11	0:04:11		78.6930524	83.3812876	82.731787	68.2395153
17:06:12	0:04:12		79.6930524	82.3812876	81.731787	67.2395153
17:06:13	0:04:13		79.6930524	82.3812876	81.731787	67.2395153
17:06:14	0:04:14		80.6930524	82.3812876	79.731787	67.2395153
17:06:15	0:04:15		80.6930524	81.3812876	79.731787	71.2395153
17:06:16	0:04:16		80.6930524	81.3812876	79.731787	72.2395153
17:06:17	0:04:17		79.6930524	81.3812876	79.731787	72.2395153
17:06:18	0:04:18		78.6930524	81.3812876	79.731787	73.2395153
17:06:19	0:04:19		79.6930524	80.3812876	80.731787	75.2395153
17:06:20	0:04:20		79.6930524	82.3812876	80.731787	75.2395153
17:06:21	0:04:21		79.6930524	82.3812876	80.731787	75.2395153
17:06:22	0:04:22		79.6930524	82.3812876	80.731787	75.2395153
17:06:23	0:04:23		79.6930524	82.3812876	81.731787	75.2395153
17:06:24	0:04:24		79.6930524	83.3812876	81.731787	76.2395153
17:06:25	0:04:25		79.6930524	83.3812876	81.731787	76.2395153
17:06:26	0:04:26		79.6930524	83.3812876	81.731787	76.2395153
17:06:27	0:04:27		79.6930524	81.3812876	86.731787	75.2395153

17:06:28	0:04:28		78.6930524	82.3812876	87.731787	73.2395153
17:06:29	0:04:29		77.6930524	82.3812876	87.731787	72.2395153
17:06:30	0:04:30		76.6930524	82.3812876	88.731787	72.2395153
17:06:31	0:04:31		75.6930524	81.3812876	89.731787	72.2395153
17:06:32	0:04:32		74.6930524	81.3812876	88.731787	72.2395153
17:06:33	0:04:33		74.6930524	81.3812876	87.731787	71.2395153
17:06:34	0:04:34		74.6930524	80.3812876	85.731787	68.2395153
17:06:35	0:04:35		74.6930524	80.3812876	84.731787	67.2395153
17:06:36	0:04:36		75.6930524	80.3812876	84.731787	69.2395153
17:06:37	0:04:37		75.6930524	80.3812876	84.731787	68.2395153
17:06:38	0:04:38		75.6930524	80.3812876	84.731787	68.2395153
17:06:39	0:04:39		75.6930524	81.3812876	84.731787	68.2395153
17:06:40	0:04:40		76.6930524	81.3812876	83.731787	68.2395153
17:06:41	0:04:41		76.6930524	81.3812876	83.731787	69.2395153
17:06:42	0:04:42		76.6930524	81.3812876	81.731787	69.2395153
17:06:43	0:04:43		76.6930524	81.3812876	81.731787	69.2395153
17:06:44	0:04:44		76.6930524	81.3812876	81.731787	71.2395153
17:06:45	0:04:45		78.6930524	81.3812876	82.731787	73.2395153
17:06:46	0:04:46		78.6930524	81.3812876	83.731787	73.2395153
17:06:47	0:04:47		78.6930524	81.3812876	84.731787	74.2395153
17:06:48	0:04:48		76.6930524	81.3812876	84.731787	74.2395153
17:06:49	0:04:49		76.6930524	81.3812876	86.731787	75.2395153
17:06:50	0:04:50		75.6930524	81.3812876	86.731787	75.2395153
17:06:51	0:04:51		74.6930524	81.3812876	86.731787	71.2395153
17:06:52	0:04:52		74.6930524	82.3812876	86.731787	71.2395153
17:06:53	0:04:53		74.6930524	85.3812876	85.731787	71.2395153
17:06:54	0:04:54		74.6930524	85.3812876	85.731787	71.2395153
17:06:55	0:04:55		75.6930524	87.3812876	84.731787	73.2395153
17:06:56	0:04:56		75.6930524	87.3812876	83.731787	73.2395153
17:06:57	0:04:57		75.6930524	87.3812876	81.731787	72.2395153
17:06:58	0:04:58		78.6930524	86.3812876	81.731787	72.2395153
17:06:59	0:04:59		76.6930524	86.3812876	81.731787	74.2395153
17:07:00	0:05:00		76.6930524	86.3812876	80.731787	75.2395153
17:07:01	0:05:01		76.6930524	86.3812876	80.731787	75.2395153
17:07:02	0:05:02		76.6930524	85.3812876	80.731787	76.2395153
17:07:03	0:05:03		75.6930524	85.3812876	79.731787	77.2395153
17:07:04	0:05:04		75.6930524	85.3812876	80.731787	78.2395153
17:07:05	0:05:05		75.6930524	85.3812876	83.731787	78.2395153
17:07:06	0:05:06		75.6930524	84.3812876	83.731787	78.2395153
17:07:07	0:05:07		75.6930524	83.3812876	82.731787	78.2395153
17:07:08	0:05:08		75.6930524	82.3812876	80.731787	76.2395153
17:07:09	0:05:09		75.6930524	82.3812876	80.731787	74.2395153
17:07:10	0:05:10		75.6930524	82.3812876	81.731787	74.2395153
17:07:11	0:05:11		76.6930524	82.3812876	81.731787	72.2395153
17:07:12	0:05:12		76.6930524	84.3812876	81.731787	72.2395153

17:07:13	0:05:13		76.6930524	86.3812876	81.731787	72.2395153
17:07:14	0:05:14		77.6930524	86.3812876	81.731787	71.2395153
17:07:15	0:05:15		78.6930524	87.3812876	81.731787	73.2395153
17:07:16	0:05:16		78.6930524	89.3812876	81.731787	73.2395153
17:07:17	0:05:17		78.6930524	89.3812876	81.731787	76.2395153
17:07:18	0:05:18		79.6930524	89.3812876	81.731787	77.2395153
17:07:19	0:05:19		79.6930524	88.3812876	80.731787	77.2395153
17:07:20	0:05:20		79.6930524	88.3812876	78.731787	77.2395153
17:07:21	0:05:21		79.6930524	88.3812876	77.731787	77.2395153
17:07:22	0:05:22		79.6930524	89.3812876	77.731787	77.2395153
17:07:23	0:05:23		79.6930524	89.3812876	77.731787	77.2395153
17:07:24	0:05:24		79.6930524	89.3812876	77.731787	78.2395153
17:07:25	0:05:25		80.6930524	89.3812876	78.731787	79.2395153
17:07:26	0:05:26		79.6930524	89.3812876	78.731787	80.2395153
17:07:27	0:05:27		79.6930524	89.3812876	78.731787	80.2395153
17:07:28	0:05:28		79.6930524	87.3812876	78.731787	83.2395153
17:07:29	0:05:29		79.6930524	87.3812876	78.731787	82.2395153
17:07:30	0:05:30		79.6930524	87.3812876	78.731787	82.2395153
17:07:31	0:05:31		79.6930524	86.3812876	78.731787	81.2395153
17:07:32	0:05:32		79.6930524	86.3812876	78.731787	81.2395153
17:07:33	0:05:33		80.6930524	87.3812876	77.731787	80.2395153
17:07:34	0:05:34		80.6930524	87.3812876	77.731787	82.2395153
17:07:35	0:05:35		80.6930524	87.3812876	78.731787	82.2395153
17:07:36	0:05:36		80.6930524	87.3812876	79.731787	82.2395153
17:07:37	0:05:37		80.6930524	87.3812876	80.731787	86.2395153
17:07:38	0:05:38		80.6930524	92.3812876	80.731787	86.2395153
17:07:39	0:05:39		79.6930524	93.3812876	80.731787	87.2395153
17:07:40	0:05:40		79.6930524	94.3812876	80.731787	85.2395153
17:07:41	0:05:41		79.6930524	94.3812876	80.731787	85.2395153
17:07:42	0:05:42		79.6930524	94.3812876	80.731787	83.2395153
17:07:43	0:05:43		78.6930524	94.3812876	79.731787	82.2395153
17:07:44	0:05:44		78.6930524	94.3812876	80.731787	80.2395153
17:07:45	0:05:45		78.6930524	94.3812876	80.731787	81.2395153
17:07:46	0:05:46		77.6930524	94.3812876	81.731787	81.2395153
17:07:47	0:05:47		77.6930524	94.3812876	82.731787	81.2395153
17:07:48	0:05:48		77.6930524	94.3812876	82.731787	81.2395153
17:07:49	0:05:49		79.6930524	94.3812876	84.731787	83.2395153
17:07:50	0:05:50		80.6930524	94.3812876	84.731787	83.2395153
17:07:51	0:05:51		80.6930524	94.3812876	83.731787	82.2395153
17:07:52	0:05:52		81.6930524	94.3812876	83.731787	80.2395153
17:07:53	0:05:53		80.6930524	94.3812876	82.731787	80.2395153
17:07:54	0:05:54		80.6930524	93.3812876	82.731787	79.2395153
17:07:55	0:05:55		80.6930524	93.3812876	81.731787	78.2395153
17:07:56	0:05:56		81.6930524	92.3812876	81.731787	78.2395153
17:07:57	0:05:57		80.6930524	91.3812876	81.731787	78.2395153

17:07:58	0:05:58		80.6930524	89.3812876	80.731787	78.2395153
17:07:59	0:05:59		80.6930524	89.3812876	78.731787	78.2395153
17:08:00	0:06:00		80.6930524	88.3812876	77.731787	78.2395153
17:08:01	0:06:01		79.6930524	87.3812876	77.731787	78.2395153
17:08:02	0:06:02		78.6930524	86.3812876	77.731787	78.2395153
17:08:03	0:06:03		82.6930524	85.3812876	77.731787	77.2395153
17:08:04	0:06:04		82.6930524	85.3812876	78.731787	76.2395153
17:08:05	0:06:05		82.6930524	85.3812876	79.731787	75.2395153
17:08:06	0:06:06		82.6930524	85.3812876	79.731787	75.2395153
17:08:07	0:06:07		82.6930524	85.3812876	79.731787	75.2395153
17:08:08	0:06:08		83.6930524	85.3812876	79.731787	75.2395153
17:08:09	0:06:09		83.6930524	84.3812876	79.731787	76.2395153
17:08:10	0:06:10		82.6930524	82.3812876	79.731787	77.2395153
17:08:11	0:06:11		81.6930524	82.3812876	79.731787	79.2395153
17:08:12	0:06:12		81.6930524	82.3812876	79.731787	77.2395153
17:08:13	0:06:13		81.6930524	82.3812876	79.731787	77.2395153
17:08:14	0:06:14		80.6930524	81.3812876	75.731787	76.2395153
17:08:15	0:06:15		80.6930524	81.3812876	74.731787	75.2395153
17:08:16	0:06:16		79.6930524	81.3812876	74.731787	75.2395153
17:08:17	0:06:17		79.6930524	80.3812876	76.731787	75.2395153
17:08:18	0:06:18		79.6930524	79.3812876	76.731787	74.2395153
17:08:19	0:06:19		79.6930524	79.3812876	76.731787	74.2395153
17:08:20	0:06:20		79.6930524	80.3812876	76.731787	74.2395153
17:08:21	0:06:21		79.6930524	80.3812876	76.731787	76.2395153
17:08:22	0:06:22		79.6930524	80.3812876	77.731787	76.2395153
17:08:23	0:06:23		79.6930524	76.3812876	77.731787	76.2395153
17:08:24	0:06:24		79.6930524	76.3812876	78.731787	76.2395153
17:08:25	0:06:25		78.6930524	77.3812876	80.731787	76.2395153
17:08:26	0:06:26		79.6930524	77.3812876	80.731787	77.2395153
17:08:27	0:06:27		79.6930524	80.3812876	79.731787	79.2395153
17:08:28	0:06:28		78.6930524	80.3812876	76.731787	80.2395153
17:08:29	0:06:29		78.6930524	82.3812876	76.731787	80.2395153
17:08:30	0:06:30		78.6930524	82.3812876	76.731787	80.2395153
17:08:31	0:06:31		78.6930524	83.3812876	76.731787	79.2395153
17:08:32	0:06:32		79.6930524	82.3812876	75.731787	78.2395153
17:08:33	0:06:33		79.6930524	82.3812876	74.731787	78.2395153
17:08:34	0:06:34		79.6930524	82.3812876	75.731787	78.2395153
17:08:35	0:06:35		79.6930524	83.3812876	77.731787	78.2395153
17:08:36	0:06:36		79.6930524	84.3812876	77.731787	78.2395153
17:08:37	0:06:37		79.6930524	84.3812876	77.731787	77.2395153
17:08:38	0:06:38		79.6930524	85.3812876	77.731787	75.2395153
17:08:39	0:06:39		78.6930524	85.3812876	77.731787	74.2395153
17:08:40	0:06:40		77.6930524	85.3812876	79.731787	75.2395153
17:08:41	0:06:41		77.6930524	85.3812876	79.731787	75.2395153
17:08:42	0:06:42		77.6930524	85.3812876	79.731787	75.2395153

17:08:43	0:06:43		77.6930524	86.3812876	80.731787	75.2395153
17:08:44	0:06:44		77.6930524	86.3812876	80.731787	75.2395153
17:08:45	0:06:45		77.6930524	86.3812876	80.731787	74.2395153
17:08:46	0:06:46		77.6930524	86.3812876	80.731787	73.2395153
17:08:47	0:06:47		78.6930524	87.3812876	80.731787	73.2395153
17:08:48	0:06:48		80.6930524	87.3812876	80.731787	74.2395153
17:08:49	0:06:49		81.6930524	87.3812876	80.731787	72.2395153
17:08:50	0:06:50		81.6930524	87.3812876	81.731787	71.2395153
17:08:51	0:06:51		82.6930524	88.3812876	82.731787	71.2395153
17:08:52	0:06:52		90.6930524	88.3812876	89.731787	71.2395153
17:08:53	0:06:53		79.6930524	88.3812876	91.731787	71.2395153
17:08:54	0:06:54		79.6930524	88.3812876	91.731787	71.2395153
17:08:55	0:06:55		78.6930524	88.3812876	91.731787	71.2395153
17:08:56	0:06:56		77.6930524	87.3812876	92.731787	71.2395153
17:08:57	0:06:57		76.6930524	87.3812876	92.731787	71.2395153
17:08:58	0:06:58		76.6930524	90.3812876	93.731787	71.2395153
17:08:59	0:06:59		77.6930524	90.3812876	94.731787	71.2395153
17:09:00	0:07:00		77.6930524	90.3812876	94.731787	71.2395153
17:09:01	0:07:01		78.6930524	89.3812876	94.731787	71.2395153
17:09:02	0:07:02		78.6930524	89.3812876	94.731787	71.2395153
17:09:03	0:07:03		78.6930524	89.3812876	94.731787	71.2395153
17:09:04	0:07:04		77.6930524	89.3812876	94.731787	70.2395153
17:09:05	0:07:05		78.6930524	89.3812876	94.731787	70.2395153
17:09:06	0:07:06		77.6930524	88.3812876	94.731787	70.2395153
17:09:07	0:07:07		76.6930524	88.3812876	90.731787	70.2395153
17:09:08	0:07:08		75.6930524	88.3812876	86.731787	70.2395153
17:09:09	0:07:09		76.6930524	85.3812876	86.731787	70.2395153
17:09:10	0:07:10		77.6930524	84.3812876	86.731787	70.2395153
17:09:11	0:07:11		77.6930524	84.3812876	86.731787	70.2395153
17:09:12	0:07:12		77.6930524	80.3812876	86.731787	69.2395153
17:09:13	0:07:13		78.6930524	80.3812876	85.731787	69.2395153
17:09:14	0:07:14		78.6930524	80.3812876	85.731787	70.2395153
17:09:15	0:07:15		78.6930524	80.3812876	89.731787	70.2395153
17:09:16	0:07:16		78.6930524	79.3812876	89.731787	74.2395153
17:09:17	0:07:17		77.6930524	79.3812876	92.731787	74.2395153
17:09:18	0:07:18		76.6930524	79.3812876	92.731787	74.2395153
17:09:19	0:07:19		77.6930524	79.3812876	91.731787	74.2395153
17:09:20	0:07:20		77.6930524	80.3812876	87.731787	74.2395153
17:09:21	0:07:21		78.6930524	82.3812876	83.731787	74.2395153
17:09:22	0:07:22		79.6930524	83.3812876	80.731787	76.2395153
17:09:23	0:07:23		80.6930524	83.3812876	79.731787	76.2395153
17:09:24	0:07:24		80.6930524	84.3812876	78.731787	76.2395153
17:09:25	0:07:25		80.6930524	84.3812876	78.731787	76.2395153
17:09:26	0:07:26		80.6930524	88.3812876	78.731787	76.2395153
17:09:27	0:07:27		80.6930524	89.3812876	78.731787	76.2395153

17:09:28	0:07:28		80.6930524	89.3812876	77.731787	76.2395153
17:09:29	0:07:29		80.6930524	88.3812876	77.731787	75.2395153
17:09:30	0:07:30		80.6930524	87.3812876	76.731787	75.2395153
17:09:31	0:07:31		80.6930524	87.3812876	77.731787	75.2395153
17:09:32	0:07:32		80.6930524	89.3812876	77.731787	75.2395153
17:09:33	0:07:33		80.6930524	89.3812876	78.731787	75.2395153
17:09:34	0:07:34		80.6930524	91.3812876	80.731787	74.2395153
17:09:35	0:07:35		80.6930524	91.3812876	81.731787	74.2395153
17:09:36	0:07:36		80.6930524	91.3812876	81.731787	74.2395153
17:09:37	0:07:37		80.6930524	91.3812876	82.731787	74.2395153
17:09:38	0:07:38		80.6930524	91.3812876	82.731787	74.2395153
17:09:39	0:07:39		79.6930524	91.3812876	83.731787	73.2395153
17:09:40	0:07:40		79.6930524	84.3812876	83.731787	73.2395153
17:09:41	0:07:41		78.6930524	81.3812876	83.731787	74.2395153
17:09:42	0:07:42		78.6930524	81.3812876	83.731787	75.2395153
17:09:43	0:07:43		78.6930524	80.3812876	83.731787	76.2395153
17:09:44	0:07:44		78.6930524	79.3812876	83.731787	76.2395153
17:09:45	0:07:45		78.6930524	79.3812876	83.731787	75.2395153
17:09:46	0:07:46		77.6930524	79.3812876	84.731787	75.2395153
17:09:47	0:07:47		77.6930524	79.3812876	84.731787	75.2395153
17:09:48	0:07:48		76.6930524	74.3812876	90.731787	75.2395153
17:09:49	0:07:49		77.6930524	73.3812876	90.731787	75.2395153
17:09:50	0:07:50		77.6930524	73.3812876	90.731787	75.2395153
17:09:51	0:07:51		77.6930524	72.3812876	91.731787	76.2395153
17:09:52	0:07:52		77.6930524	72.3812876	91.731787	77.2395153
17:09:53	0:07:53		78.6930524	76.3812876	92.731787	78.2395153
17:09:54	0:07:54		78.6930524	79.3812876	92.731787	78.2395153
17:09:55	0:07:55		78.6930524	79.3812876	92.731787	78.2395153
17:09:56	0:07:56		79.6930524	81.3812876	92.731787	77.2395153
17:09:57	0:07:57		81.6930524	81.3812876	91.731787	77.2395153
17:09:58	0:07:58		81.6930524	83.3812876	91.731787	76.2395153
17:09:59	0:07:59		81.6930524	83.3812876	91.731787	75.2395153
17:10:00	0:08:00		81.6930524	83.3812876	91.731787	75.2395153
17:10:01	0:08:01		82.6930524	83.3812876	91.731787	75.2395153
17:10:02	0:08:02		83.6930524	83.3812876	91.731787	75.2395153
17:10:03	0:08:03		83.6930524	83.3812876	89.731787	75.2395153
17:10:04	0:08:04		83.6930524	83.3812876	89.731787	75.2395153
17:10:05	0:08:05		85.6930524	84.3812876	88.731787	74.2395153
17:10:06	0:08:06		86.6930524	84.3812876	87.731787	74.2395153
17:10:07	0:08:07		86.6930524	84.3812876	87.731787	74.2395153
17:10:08	0:08:08		86.6930524	84.3812876	87.731787	73.2395153
17:10:09	0:08:09		84.6930524	84.3812876	85.731787	73.2395153
17:10:10	0:08:10		83.6930524	83.3812876	85.731787	73.2395153
17:10:11	0:08:11		83.6930524	84.3812876	81.731787	74.2395153
17:10:12	0:08:12		80.6930524	87.3812876	81.731787	74.2395153

17:10:13	0:08:13		79.6930524	88.3812876	80.731787	77.2395153
17:10:14	0:08:14		78.6930524	88.3812876	80.731787	78.2395153
17:10:15	0:08:15		78.6930524	88.3812876	80.731787	78.2395153
17:10:16	0:08:16		79.6930524	87.3812876	80.731787	78.2395153
17:10:17	0:08:17		79.6930524	87.3812876	80.731787	78.2395153
17:10:18	0:08:18		80.6930524	87.3812876	80.731787	81.2395153
17:10:19	0:08:19		83.6930524	87.3812876	80.731787	81.2395153
17:10:20	0:08:20		83.6930524	87.3812876	80.731787	82.2395153
17:10:21	0:08:21		83.6930524	87.3812876	80.731787	81.2395153
17:10:22	0:08:22		83.6930524	86.3812876	80.731787	80.2395153
17:10:23	0:08:23		82.6930524	86.3812876	79.731787	80.2395153
17:10:24	0:08:24		80.6930524	86.3812876	79.731787	77.2395153
17:10:25	0:08:25		80.6930524	85.3812876	78.731787	76.2395153
17:10:26	0:08:26		80.6930524	85.3812876	78.731787	75.2395153
17:10:27	0:08:27		79.6930524	85.3812876	78.731787	75.2395153
17:10:28	0:08:28		78.6930524	85.3812876	78.731787	73.2395153
17:10:29	0:08:29		76.6930524	85.3812876	78.731787	73.2395153
17:10:30	0:08:30		76.6930524	85.3812876	78.731787	73.2395153
17:10:31	0:08:31		76.6930524	85.3812876	78.731787	73.2395153
17:10:32	0:08:32		76.6930524	84.3812876	78.731787	72.2395153
17:10:33	0:08:33		76.6930524	83.3812876	79.731787	72.2395153
17:10:34	0:08:34		76.6930524	82.3812876	79.731787	72.2395153
17:10:35	0:08:35		76.6930524	82.3812876	80.731787	72.2395153
17:10:36	0:08:36		74.6930524	81.3812876	79.731787	72.2395153
17:10:37	0:08:37		74.6930524	79.3812876	79.731787	72.2395153
17:10:38	0:08:38		74.6930524	80.3812876	79.731787	72.2395153
17:10:39	0:08:39		75.6930524	81.3812876	79.731787	72.2395153
17:10:40	0:08:40		75.6930524	81.3812876	80.731787	72.2395153
17:10:41	0:08:41		75.6930524	81.3812876	80.731787	72.2395153
17:10:42	0:08:42		78.6930524	81.3812876	80.731787	72.2395153
17:10:43	0:08:43		80.6930524	81.3812876	80.731787	72.2395153
17:10:44	0:08:44		80.6930524	81.3812876	80.731787	73.2395153
17:10:45	0:08:45		81.6930524	81.3812876	80.731787	73.2395153
17:10:46	0:08:46		81.6930524	82.3812876	80.731787	73.2395153
17:10:47	0:08:47		81.6930524	82.3812876	79.731787	73.2395153
17:10:48	0:08:48		80.6930524	84.3812876	79.731787	74.2395153
17:10:49	0:08:49		80.6930524	85.3812876	79.731787	74.2395153
17:10:50	0:08:50		80.6930524	85.3812876	79.731787	74.2395153
17:10:51	0:08:51		79.6930524	88.3812876	79.731787	74.2395153
17:10:52	0:08:52		79.6930524	89.3812876	80.731787	74.2395153
17:10:53	0:08:53		79.6930524	92.3812876	80.731787	74.2395153
17:10:54	0:08:54		78.6930524	92.3812876	81.731787	75.2395153
17:10:55	0:08:55		78.6930524	94.3812876	81.731787	76.2395153
17:10:56	0:08:56		77.6930524	95.3812876	81.731787	76.2395153
17:10:57	0:08:57		78.6930524	95.3812876	81.731787	76.2395153

17:10:58	0:08:58		78.6930524	95.3812876	81.731787	77.2395153
17:10:59	0:08:59		78.6930524	95.3812876	80.731787	77.2395153
17:11:00	0:09:00		78.6930524	96.3812876	80.731787	77.2395153
17:11:01	0:09:01		79.6930524	96.3812876	80.731787	77.2395153
17:11:02	0:09:02		78.6930524	97.3812876	80.731787	77.2395153
17:11:03	0:09:03		78.6930524	97.3812876	80.731787	77.2395153
17:11:04	0:09:04		77.6930524	97.3812876	81.731787	77.2395153
17:11:05	0:09:05		76.6930524	97.3812876	82.731787	77.2395153
17:11:06	0:09:06		76.6930524	96.3812876	83.731787	77.2395153
17:11:07	0:09:07		75.6930524	96.3812876	83.731787	78.2395153
17:11:08	0:09:08		75.6930524	92.3812876	83.731787	73.2395153
17:11:09	0:09:09		76.6930524	92.3812876	81.731787	71.2395153
17:11:10	0:09:10		76.6930524	92.3812876	79.731787	71.2395153
17:11:11	0:09:11		77.6930524	91.3812876	78.731787	72.2395153
17:11:12	0:09:12		78.6930524	90.3812876	78.731787	72.2395153
17:11:13	0:09:13		78.6930524	89.3812876	77.731787	75.2395153
17:11:14	0:09:14		79.6930524	87.3812876	77.731787	74.2395153
17:11:15	0:09:15		79.6930524	85.3812876	78.731787	74.2395153
17:11:16	0:09:16		78.6930524	85.3812876	79.731787	74.2395153
17:11:17	0:09:17		78.6930524	84.3812876	79.731787	74.2395153
17:11:18	0:09:18		77.6930524	79.3812876	81.731787	73.2395153
17:11:19	0:09:19		76.6930524	77.3812876	81.731787	73.2395153
17:11:20	0:09:20		75.6930524	77.3812876	81.731787	73.2395153
17:11:21	0:09:21		74.6930524	77.3812876	80.731787	74.2395153
17:11:22	0:09:22		75.6930524	76.3812876	80.731787	75.2395153
17:11:23	0:09:23		75.6930524	76.3812876	82.731787	75.2395153
17:11:24	0:09:24		75.6930524	76.3812876	82.731787	75.2395153
17:11:25	0:09:25		76.6930524	75.3812876	85.731787	74.2395153
17:11:26	0:09:26		77.6930524	76.3812876	85.731787	74.2395153
17:11:27	0:09:27		78.6930524	76.3812876	85.731787	75.2395153
17:11:28	0:09:28		77.6930524	76.3812876	85.731787	75.2395153
17:11:29	0:09:29		77.6930524	77.3812876	85.731787	75.2395153
17:11:30	0:09:30		77.6930524	77.3812876	85.731787	75.2395153
17:11:31	0:09:31		76.6930524	77.3812876	85.731787	75.2395153
17:11:32	0:09:32		76.6930524	77.3812876	85.731787	75.2395153
17:11:33	0:09:33		76.6930524	78.3812876	84.731787	75.2395153
17:11:34	0:09:34		76.6930524	78.3812876	82.731787	75.2395153
17:11:35	0:09:35		76.6930524	78.3812876	81.731787	74.2395153
17:11:36	0:09:36		76.6930524	77.3812876	81.731787	74.2395153
17:11:37	0:09:37		76.6930524	77.3812876	81.731787	71.2395153
17:11:38	0:09:38		76.6930524	78.3812876	81.731787	71.2395153
17:11:39	0:09:39		76.6930524	78.3812876	81.731787	71.2395153
17:11:40	0:09:40		77.6930524	78.3812876	81.731787	71.2395153
17:11:41	0:09:41		76.6930524	83.3812876	81.731787	71.2395153
17:11:42	0:09:42		76.6930524	83.3812876	80.731787	71.2395153

17:11:43	0:09:43		76.6930524	83.3812876	79.731787	74.2395153
17:11:44	0:09:44		76.6930524	80.3812876	78.731787	75.2395153
17:11:45	0:09:45		77.6930524	78.3812876	78.731787	76.2395153
17:11:46	0:09:46		78.6930524	78.3812876	78.731787	76.2395153
17:11:47	0:09:47		79.6930524	79.3812876	78.731787	76.2395153
17:11:48	0:09:48		79.6930524	79.3812876	79.731787	76.2395153
17:11:49	0:09:49		79.6930524	82.3812876	80.731787	77.2395153
17:11:50	0:09:50		78.6930524	82.3812876	81.731787	77.2395153
17:11:51	0:09:51		77.6930524	83.3812876	82.731787	77.2395153
17:11:52	0:09:52		75.6930524	83.3812876	82.731787	77.2395153
17:11:53	0:09:53		75.6930524	83.3812876	82.731787	77.2395153
17:11:54	0:09:54		74.6930524	83.3812876	81.731787	75.2395153
17:11:55	0:09:55		75.6930524	83.3812876	81.731787	76.2395153
17:11:56	0:09:56		75.6930524	83.3812876	81.731787	77.2395153
17:11:57	0:09:57		75.6930524	83.3812876	81.731787	78.2395153
17:11:58	0:09:58		76.6930524	83.3812876	81.731787	78.2395153
17:11:59	0:09:59		77.6930524	83.3812876	81.731787	78.2395153
17:12:00	0:10:00		77.6930524	84.3812876	81.731787	78.2395153
17:12:01	0:10:01		79.6930524	87.3812876	81.731787	78.2395153
17:12:02	0:10:02		79.6930524	89.3812876	81.731787	78.2395153
17:12:03	0:10:03		79.6930524	92.3812876	81.731787	78.2395153
17:12:04	0:10:04		79.6930524	92.3812876	82.731787	78.2395153
17:12:05	0:10:05		79.6930524	94.3812876	82.731787	78.2395153
17:12:06	0:10:06		78.6930524	94.3812876	83.731787	78.2395153
17:12:07	0:10:07		77.6930524	94.3812876	83.731787	78.2395153
17:12:08	0:10:08		77.6930524	95.3812876	84.731787	77.2395153
17:12:09	0:10:09		77.6930524	95.3812876	84.731787	77.2395153
17:12:10	0:10:10		77.6930524	95.3812876	84.731787	77.2395153
17:12:11	0:10:11		77.6930524	95.3812876	86.731787	77.2395153
17:12:12	0:10:12		78.6930524	94.3812876	86.731787	77.2395153
17:12:13	0:10:13		78.6930524	94.3812876	86.731787	77.2395153
17:12:14	0:10:14		78.6930524	94.3812876	88.731787	77.2395153
17:12:15	0:10:15		78.6930524	93.3812876	88.731787	77.2395153
17:12:16	0:10:16		78.6930524	93.3812876	90.731787	77.2395153
17:12:17	0:10:17		78.6930524	93.3812876	92.731787	78.2395153
17:12:18	0:10:18		78.6930524	93.3812876	92.731787	78.2395153
17:12:19	0:10:19		77.6930524	93.3812876	92.731787	76.2395153
17:12:20	0:10:20		77.6930524	88.3812876	92.731787	76.2395153
17:12:21	0:10:21		77.6930524	88.3812876	92.731787	76.2395153
17:12:22	0:10:22		77.6930524	86.3812876	92.731787	76.2395153
17:12:23	0:10:23		76.6930524	77.3812876	92.731787	76.2395153
17:12:24	0:10:24		75.6930524	77.3812876	93.731787	76.2395153
17:12:25	0:10:25		75.6930524	77.3812876	93.731787	74.2395153
17:12:26	0:10:26		74.6930524	77.3812876	93.731787	74.2395153
17:12:27	0:10:27		74.6930524	76.3812876	93.731787	74.2395153

17:12:28	0:10:28		75.6930524	76.3812876	93.731787	74.2395153
17:12:29	0:10:29		75.6930524	76.3812876	93.731787	74.2395153
17:12:30	0:10:30		76.6930524	76.3812876	93.731787	76.2395153
17:12:31	0:10:31		77.6930524	75.3812876	78.731787	76.2395153
17:12:32	0:10:32		77.6930524	78.3812876	76.731787	77.2395153
17:12:33	0:10:33		76.6930524	78.3812876	75.731787	78.2395153
17:12:34	0:10:34		76.6930524	77.3812876	75.731787	78.2395153
17:12:35	0:10:35		75.6930524	76.3812876	75.731787	78.2395153
17:12:36	0:10:36		75.6930524	75.3812876	77.731787	77.2395153
17:12:37	0:10:37		74.6930524	76.3812876	78.731787	74.2395153
17:12:38	0:10:38		74.6930524	76.3812876	79.731787	74.2395153
17:12:39	0:10:39		74.6930524	76.3812876	80.731787	74.2395153
17:12:40	0:10:40		74.6930524	76.3812876	79.731787	73.2395153
17:12:41	0:10:41		74.6930524	76.3812876	79.731787	71.2395153
17:12:42	0:10:42		76.6930524	76.3812876	79.731787	72.2395153
17:12:43	0:10:43		76.6930524	76.3812876	78.731787	72.2395153
17:12:44	0:10:44		77.6930524	76.3812876	78.731787	72.2395153
17:12:45	0:10:45		78.6930524	77.3812876	79.731787	72.2395153
17:12:46	0:10:46		79.6930524	76.3812876	79.731787	72.2395153
17:12:47	0:10:47		79.6930524	76.3812876	80.731787	73.2395153
17:12:48	0:10:48		79.6930524	77.3812876	81.731787	73.2395153
17:12:49	0:10:49		79.6930524	76.3812876	81.731787	73.2395153
17:12:50	0:10:50		78.6930524	76.3812876	81.731787	73.2395153
17:12:51	0:10:51		79.6930524	77.3812876	81.731787	73.2395153
17:12:52	0:10:52		79.6930524	78.3812876	81.731787	75.2395153
17:12:53	0:10:53		79.6930524	78.3812876	81.731787	76.2395153
17:12:54	0:10:54		79.6930524	80.3812876	81.731787	77.2395153
17:12:55	0:10:55		80.6930524	81.3812876	81.731787	77.2395153
17:12:56	0:10:56		79.6930524	82.3812876	81.731787	77.2395153
17:12:57	0:10:57		78.6930524	82.3812876	81.731787	76.2395153
17:12:58	0:10:58		78.6930524	82.3812876	81.731787	75.2395153
17:12:59	0:10:59		78.6930524	82.3812876	82.731787	75.2395153
17:13:00	0:11:00		78.6930524	82.3812876	82.731787	74.2395153
17:13:01	0:11:01		78.6930524	82.3812876	82.731787	74.2395153
17:13:02	0:11:02		78.6930524	82.3812876	82.731787	74.2395153
17:13:03	0:11:03		78.6930524	85.3812876	83.731787	74.2395153
17:13:04	0:11:04		78.6930524	88.3812876	84.731787	74.2395153
17:13:05	0:11:05		78.6930524	90.3812876	84.731787	75.2395153
17:13:06	0:11:06		78.6930524	90.3812876	84.731787	75.2395153
17:13:07	0:11:07		78.6930524	90.3812876	84.731787	76.2395153
17:13:08	0:11:08		78.6930524	89.3812876	84.731787	76.2395153
17:13:09	0:11:09		78.6930524	88.3812876	85.731787	76.2395153
17:13:10	0:11:10		78.6930524	88.3812876	85.731787	76.2395153
17:13:11	0:11:11		79.6930524	87.3812876	85.731787	76.2395153
17:13:12	0:11:12		79.6930524	87.3812876	85.731787	76.2395153

17:13:13	0:11:13		79.6930524	88.3812876	84.731787	80.2395153
17:13:14	0:11:14		79.6930524	87.3812876	84.731787	80.2395153
17:13:15	0:11:15		79.6930524	87.3812876	84.731787	79.2395153
17:13:16	0:11:16		80.6930524	87.3812876	77.731787	79.2395153
17:13:17	0:11:17		80.6930524	86.3812876	76.731787	78.2395153
17:13:18	0:11:18		79.6930524	86.3812876	76.731787	76.2395153
17:13:19	0:11:19		78.6930524	86.3812876	76.731787	75.2395153
17:13:20	0:11:20		78.6930524	81.3812876	76.731787	74.2395153
17:13:21	0:11:21		78.6930524	79.3812876	76.731787	71.2395153
17:13:22	0:11:22		79.6930524	79.3812876	76.731787	70.2395153
17:13:23	0:11:23		79.6930524	75.3812876	75.731787	70.2395153
17:13:24	0:11:24		81.6930524	74.3812876	75.731787	70.2395153
17:13:25	0:11:25		81.6930524	77.3812876	76.731787	70.2395153
17:13:26	0:11:26		81.6930524	77.3812876	78.731787	71.2395153
17:13:27	0:11:27		82.6930524	77.3812876	78.731787	72.2395153
17:13:28	0:11:28		82.6930524	77.3812876	79.731787	72.2395153
17:13:29	0:11:29		81.6930524	77.3812876	79.731787	74.2395153
17:13:30	0:11:30		81.6930524	77.3812876	79.731787	74.2395153
17:13:31	0:11:31		82.6930524	77.3812876	79.731787	74.2395153
17:13:32	0:11:32		82.6930524	81.3812876	80.731787	70.2395153
17:13:33	0:11:33		82.6930524	84.3812876	81.731787	71.2395153
17:13:34	0:11:34		82.6930524	84.3812876	81.731787	71.2395153
17:13:35	0:11:35		82.6930524	84.3812876	82.731787	71.2395153
17:13:36	0:11:36		81.6930524	86.3812876	83.731787	71.2395153
17:13:37	0:11:37		79.6930524	86.3812876	84.731787	72.2395153
17:13:38	0:11:38		78.6930524	86.3812876	85.731787	71.2395153
17:13:39	0:11:39		76.6930524	86.3812876	85.731787	70.2395153
17:13:40	0:11:40		76.6930524	86.3812876	84.731787	69.2395153
17:13:41	0:11:41		76.6930524	86.3812876	83.731787	69.2395153
17:13:42	0:11:42		76.6930524	87.3812876	82.731787	69.2395153
17:13:43	0:11:43		76.6930524	87.3812876	82.731787	69.2395153
17:13:44	0:11:44		76.6930524	88.3812876	81.731787	69.2395153
17:13:45	0:11:45		76.6930524	88.3812876	81.731787	69.2395153
17:13:46	0:11:46		76.6930524	88.3812876	81.731787	70.2395153
17:13:47	0:11:47		76.6930524	85.3812876	81.731787	70.2395153
17:13:48	0:11:48		76.6930524	85.3812876	81.731787	71.2395153
17:13:49	0:11:49		76.6930524	85.3812876	81.731787	72.2395153
17:13:50	0:11:50		76.6930524	85.3812876	81.731787	72.2395153
17:13:51	0:11:51		76.6930524	85.3812876	80.731787	72.2395153
17:13:52	0:11:52		75.6930524	85.3812876	80.731787	72.2395153
17:13:53	0:11:53		75.6930524	87.3812876	80.731787	74.2395153
17:13:54	0:11:54		75.6930524	88.3812876	81.731787	74.2395153
17:13:55	0:11:55		75.6930524	88.3812876	82.731787	74.2395153
17:13:56	0:11:56		75.6930524	88.3812876	82.731787	73.2395153
17:13:57	0:11:57		74.6930524	88.3812876	81.731787	73.2395153

17:13:58	0:11:58		74.6930524	88.3812876	81.731787	73.2395153
17:13:59	0:11:59		74.6930524	85.3812876	81.731787	73.2395153
17:14:00	0:12:00		74.6930524	85.3812876	81.731787	74.2395153
17:14:01	0:12:01		74.6930524	84.3812876	80.731787	74.2395153
17:14:02	0:12:02		74.6930524	84.3812876	80.731787	74.2395153
17:14:03	0:12:03		74.6930524	83.3812876	80.731787	75.2395153
17:14:04	0:12:04		74.6930524	83.3812876	80.731787	76.2395153
17:14:05	0:12:05		74.6930524	83.3812876	80.731787	76.2395153
17:14:06	0:12:06		74.6930524	83.3812876	80.731787	76.2395153
17:14:07	0:12:07		73.6930524	81.3812876	81.731787	76.2395153
17:14:08	0:12:08		73.6930524	80.3812876	81.731787	76.2395153
17:14:09	0:12:09		73.6930524	79.3812876	82.731787	76.2395153
17:14:10	0:12:10		73.6930524	79.3812876	82.731787	76.2395153
17:14:11	0:12:11		73.6930524	82.3812876	82.731787	76.2395153
17:14:12	0:12:12		73.6930524	82.3812876	82.731787	76.2395153
17:14:13	0:12:13		73.6930524	82.3812876	82.731787	76.2395153
17:14:14	0:12:14		73.6930524	82.3812876	82.731787	76.2395153
17:14:15	0:12:15		73.6930524	82.3812876	82.731787	75.2395153
17:14:16	0:12:16		73.6930524	82.3812876	87.731787	74.2395153
17:14:17	0:12:17		74.6930524	84.3812876	89.731787	74.2395153
17:14:18	0:12:18		72.6930524	85.3812876	89.731787	74.2395153
17:14:19	0:12:19		70.6930524	85.3812876	89.731787	74.2395153
17:14:20	0:12:20		70.6930524	83.3812876	89.731787	74.2395153
17:14:21	0:12:21		70.6930524	83.3812876	88.731787	75.2395153
17:14:22	0:12:22		70.6930524	86.3812876	88.731787	75.2395153
17:14:23	0:12:23		71.6930524	87.3812876	87.731787	75.2395153
17:14:24	0:12:24		72.6930524	88.3812876	87.731787	76.2395153
17:14:25	0:12:25		73.6930524	88.3812876	87.731787	78.2395153
17:14:26	0:12:26		73.6930524	92.3812876	83.731787	78.2395153
17:14:27	0:12:27		73.6930524	95.3812876	83.731787	78.2395153
17:14:28	0:12:28		75.6930524	95.3812876	83.731787	78.2395153
17:14:29	0:12:29		76.6930524	94.3812876	81.731787	78.2395153
17:14:30	0:12:30		77.6930524	94.3812876	80.731787	77.2395153
17:14:31	0:12:31		77.6930524	90.3812876	80.731787	77.2395153
17:14:32	0:12:32		76.6930524	89.3812876	80.731787	77.2395153
17:14:33	0:12:33		75.6930524	88.3812876	80.731787	77.2395153
17:14:34	0:12:34		74.6930524	87.3812876	80.731787	78.2395153
17:14:35	0:12:35		73.6930524	87.3812876	81.731787	78.2395153
17:14:36	0:12:36		72.6930524	87.3812876	81.731787	76.2395153
17:14:37	0:12:37		72.6930524	89.3812876	81.731787	76.2395153
17:14:38	0:12:38		72.6930524	90.3812876	81.731787	74.2395153
17:14:39	0:12:39		73.6930524	90.3812876	80.731787	71.2395153
17:14:40	0:12:40		73.6930524	91.3812876	80.731787	71.2395153
17:14:41	0:12:41		74.6930524	90.3812876	80.731787	71.2395153
17:14:42	0:12:42		74.6930524	88.3812876	80.731787	71.2395153

17:14:43	0:12:43		75.6930524	89.3812876	80.731787	71.2395153
17:14:44	0:12:44		75.6930524	89.3812876	80.731787	71.2395153
17:14:45	0:12:45		75.6930524	89.3812876	81.731787	70.2395153
17:14:46	0:12:46		76.6930524	90.3812876	81.731787	67.2395153
17:14:47	0:12:47		75.6930524	90.3812876	81.731787	67.2395153
17:14:48	0:12:48		75.6930524	90.3812876	81.731787	66.2395153
17:14:49	0:12:49		75.6930524	90.3812876	81.731787	66.2395153
17:14:50	0:12:50		75.6930524	89.3812876	81.731787	67.2395153
17:14:51	0:12:51		76.6930524	88.3812876	81.731787	67.2395153
17:14:52	0:12:52		75.6930524	88.3812876	81.731787	67.2395153
17:14:53	0:12:53		75.6930524	86.3812876	80.731787	67.2395153
17:14:54	0:12:54		75.6930524	83.3812876	80.731787	65.2395153
17:14:55	0:12:55		75.6930524	83.3812876	80.731787	65.2395153
17:14:56	0:12:56		75.6930524	80.3812876	80.731787	65.2395153
17:14:57	0:12:57		75.6930524	80.3812876	80.731787	65.2395153
17:14:58	0:12:58		75.6930524	81.3812876	80.731787	63.2395153
17:14:59	0:12:59		76.6930524	81.3812876	81.731787	63.2395153
17:15:00	0:13:00		76.6930524	81.3812876	81.731787	63.2395153
17:15:01	0:13:01		77.6930524	81.3812876	81.731787	64.2395153
17:15:02	0:13:02		77.6930524	80.3812876	81.731787	64.2395153
17:15:03	0:13:03		77.6930524	78.3812876	82.731787	63.2395153
17:15:04	0:13:04		76.6930524	78.3812876	82.731787	63.2395153
17:15:05	0:13:05		76.6930524	78.3812876	82.731787	63.2395153
17:15:06	0:13:06		76.6930524	78.3812876	83.731787	67.2395153
17:15:07	0:13:07		77.6930524	78.3812876	83.731787	71.2395153
17:15:08	0:13:08		78.6930524	79.3812876	84.731787	72.2395153
17:15:09	0:13:09		78.6930524	79.3812876	84.731787	72.2395153
17:15:10	0:13:10		78.6930524	79.3812876	84.731787	72.2395153
17:15:11	0:13:11		77.6930524	80.3812876	85.731787	72.2395153
17:15:12	0:13:12		76.6930524	80.3812876	87.731787	73.2395153
17:15:13	0:13:13		75.6930524	80.3812876	84.731787	73.2395153
17:15:14	0:13:14		74.6930524	80.3812876	84.731787	73.2395153
17:15:15	0:13:15		74.6930524	80.3812876	83.731787	74.2395153
17:15:16	0:13:16		74.6930524	79.3812876	79.731787	75.2395153
17:15:17	0:13:17		74.6930524	79.3812876	78.731787	76.2395153
17:15:18	0:13:18		74.6930524	79.3812876	77.731787	76.2395153
17:15:19	0:13:19		75.6930524	79.3812876	76.731787	76.2395153
17:15:20	0:13:20		75.6930524	79.3812876	76.731787	74.2395153
17:15:21	0:13:21		76.6930524	79.3812876	77.731787	73.2395153
17:15:22	0:13:22		75.6930524	79.3812876	78.731787	73.2395153
17:15:23	0:13:23		76.6930524	80.3812876	79.731787	73.2395153
17:15:24	0:13:24		76.6930524	81.3812876	79.731787	72.2395153
17:15:25	0:13:25		75.6930524	82.3812876	79.731787	70.2395153
17:15:26	0:13:26		75.6930524	83.3812876	79.731787	70.2395153
17:15:27	0:13:27		75.6930524	84.3812876	79.731787	70.2395153

17:15:28	0:13:28		75.6930524	84.3812876	80.731787	70.2395153
17:15:29	0:13:29		76.6930524	83.3812876	80.731787	70.2395153
17:15:30	0:13:30		76.6930524	83.3812876	81.731787	68.2395153
17:15:31	0:13:31		76.6930524	83.3812876	81.731787	67.2395153
17:15:32	0:13:32		76.6930524	83.3812876	82.731787	66.2395153
17:15:33	0:13:33		76.6930524	82.3812876	82.731787	66.2395153
17:15:34	0:13:34		76.6930524	79.3812876	82.731787	63.2395153
17:15:35	0:13:35		76.6930524	78.3812876	81.731787	62.2395153
17:15:36	0:13:36		76.6930524	78.3812876	80.731787	62.2395153
17:15:37	0:13:37		75.6930524	78.3812876	80.731787	62.2395153
17:15:38	0:13:38		75.6930524	78.3812876	80.731787	65.2395153
17:15:39	0:13:39		75.6930524	78.3812876	79.731787	68.2395153
17:15:40	0:13:40		76.6930524	79.3812876	78.731787	69.2395153
17:15:41	0:13:41		76.6930524	79.3812876	78.731787	69.2395153
17:15:42	0:13:42		76.6930524	79.3812876	78.731787	69.2395153
17:15:43	0:13:43		75.6930524	79.3812876	78.731787	69.2395153
17:15:44	0:13:44		75.6930524	80.3812876	78.731787	69.2395153
17:15:45	0:13:45		78.6930524	80.3812876	78.731787	69.2395153
17:15:46	0:13:46		78.6930524	80.3812876	78.731787	69.2395153
17:15:47	0:13:47		78.6930524	80.3812876	78.731787	69.2395153
17:15:48	0:13:48		78.6930524	80.3812876	78.731787	69.2395153
17:15:49	0:13:49		79.6930524	80.3812876	77.731787	69.2395153
17:15:50	0:13:50		79.6930524	80.3812876	77.731787	69.2395153
17:15:51	0:13:51		79.6930524	81.3812876	77.731787	70.2395153
17:15:52	0:13:52		78.6930524	81.3812876	77.731787	70.2395153
17:15:53	0:13:53		77.6930524	82.3812876	77.731787	70.2395153
17:15:54	0:13:54		77.6930524	82.3812876	77.731787	70.2395153
17:15:55	0:13:55		76.6930524	83.3812876	77.731787	71.2395153
17:15:56	0:13:56		76.6930524	84.3812876	77.731787	71.2395153
17:15:57	0:13:57		75.6930524	85.3812876	77.731787	71.2395153
17:15:58	0:13:58		75.6930524	85.3812876	77.731787	71.2395153
17:15:59	0:13:59		75.6930524	91.3812876	76.731787	71.2395153
17:16:00	0:14:00		76.6930524	92.3812876	76.731787	71.2395153
17:16:01	0:14:01		77.6930524	91.3812876	72.731787	70.2395153
17:16:02	0:14:02		77.6930524	89.3812876	72.731787	70.2395153
17:16:03	0:14:03		77.6930524	88.3812876	72.731787	70.2395153
17:16:04	0:14:04		76.6930524	90.3812876	72.731787	70.2395153
17:16:05	0:14:05		76.6930524	89.3812876	73.731787	70.2395153
17:16:06	0:14:06		75.6930524	89.3812876	74.731787	71.2395153
17:16:07	0:14:07		75.6930524	89.3812876	74.731787	72.2395153
17:16:08	0:14:08		75.6930524	89.3812876	75.731787	72.2395153
17:16:09	0:14:09		75.6930524	89.3812876	74.731787	72.2395153
17:16:10	0:14:10		75.6930524	90.3812876	74.731787	73.2395153
17:16:11	0:14:11		75.6930524	90.3812876	74.731787	74.2395153
17:16:12	0:14:12		75.6930524	90.3812876	74.731787	74.2395153

17:16:13	0:14:13		75.6930524	90.3812876	74.731787	74.2395153
17:16:14	0:14:14		76.6930524	90.3812876	73.731787	72.2395153
17:16:15	0:14:15		76.6930524	90.3812876	72.731787	70.2395153
17:16:16	0:14:16		76.6930524	89.3812876	72.731787	69.2395153
17:16:17	0:14:17		76.6930524	88.3812876	73.731787	67.2395153
17:16:18	0:14:18		75.6930524	88.3812876	73.731787	67.2395153
17:16:19	0:14:19		74.6930524	88.3812876	73.731787	67.2395153
17:16:20	0:14:20		74.6930524	88.3812876	76.731787	67.2395153
17:16:21	0:14:21		74.6930524	88.3812876	77.731787	66.2395153
17:16:22	0:14:22		74.6930524	88.3812876	77.731787	67.2395153
17:16:23	0:14:23		74.6930524	87.3812876	76.731787	68.2395153
17:16:24	0:14:24		74.6930524	87.3812876	76.731787	69.2395153
17:16:25	0:14:25		74.6930524	71.3812876	75.731787	69.2395153
17:16:26	0:14:26		73.6930524	71.3812876	75.731787	69.2395153
17:16:27	0:14:27		73.6930524	71.3812876	76.731787	70.2395153
17:16:28	0:14:28		73.6930524	72.3812876	75.731787	72.2395153
17:16:29	0:14:29		74.6930524	79.3812876	75.731787	72.2395153
17:16:30	0:14:30		74.6930524	85.3812876	75.731787	72.2395153
17:16:31	0:14:31		74.6930524	85.3812876	75.731787	73.2395153
17:16:32	0:14:32		74.6930524	86.3812876	76.731787	74.2395153
17:16:33	0:14:33		75.6930524	86.3812876	75.731787	74.2395153
17:16:34	0:14:34		75.6930524	86.3812876	75.731787	75.2395153
17:16:35	0:14:35		76.6930524	86.3812876	75.731787	74.2395153
17:16:36	0:14:36		77.6930524	86.3812876	75.731787	73.2395153
17:16:37	0:14:37		77.6930524	86.3812876	75.731787	73.2395153
17:16:38	0:14:38		77.6930524	85.3812876	76.731787	73.2395153
17:16:39	0:14:39		77.6930524	85.3812876	76.731787	72.2395153
17:16:40	0:14:40		77.6930524	84.3812876	76.731787	72.2395153
17:16:41	0:14:41		77.6930524	84.3812876	76.731787	72.2395153
17:16:42	0:14:42		76.6930524	83.3812876	76.731787	72.2395153
17:16:43	0:14:43		76.6930524	81.3812876	76.731787	75.2395153
17:16:44	0:14:44		76.6930524	80.3812876	75.731787	76.2395153
17:16:45	0:14:45		77.6930524	80.3812876	74.731787	76.2395153
17:16:46	0:14:46		77.6930524	80.3812876	74.731787	76.2395153
17:16:47	0:14:47		77.6930524	80.3812876	73.731787	75.2395153
17:16:48	0:14:48		77.6930524	80.3812876	73.731787	75.2395153
17:16:49	0:14:49		78.6930524	80.3812876	74.731787	75.2395153
17:16:50	0:14:50		78.6930524	77.3812876	74.731787	74.2395153
17:16:51	0:14:51		78.6930524	73.3812876	77.731787	74.2395153
17:16:52	0:14:52		78.6930524	70.3812876	77.731787	74.2395153
17:16:53	0:14:53		78.6930524	69.3812876	76.731787	75.2395153
17:16:54	0:14:54		76.6930524	69.3812876	76.731787	75.2395153
17:16:55	0:14:55		75.6930524	68.3812876	76.731787	76.2395153
17:16:56	0:14:56		73.6930524	68.3812876	75.731787	79.2395153
17:16:57	0:14:57		73.6930524	68.3812876	75.731787	80.2395153

17:16:58	0:14:58		74.6930524	68.3812876	75.731787	80.2395153
17:16:59	0:14:59		74.6930524	68.3812876	76.731787	80.2395153
17:17:00	0:15:00		76.6930524	68.3812876	76.731787	78.2395153
17:17:01	0:15:01		76.6930524	68.3812876	76.731787	78.2395153
17:17:02	0:15:02		76.6930524	68.3812876	75.731787	77.2395153
17:17:03	0:15:03		76.6930524	68.3812876	75.731787	74.2395153
17:17:04	0:15:04		76.6930524	68.3812876	76.731787	70.2395153
17:17:05	0:15:05		76.6930524	69.3812876	78.731787	69.2395153
17:17:06	0:15:06		76.6930524	69.3812876	79.731787	69.2395153
17:17:07	0:15:07		77.6930524	70.3812876	80.731787	70.2395153
17:17:08	0:15:08		78.6930524	70.3812876	80.731787	71.2395153
17:17:09	0:15:09		78.6930524	68.3812876	80.731787	71.2395153
17:17:10	0:15:10		78.6930524	68.3812876	80.731787	70.2395153
17:17:11	0:15:11		78.6930524	68.3812876	80.731787	70.2395153
17:17:12	0:15:12		78.6930524	68.3812876	82.731787	70.2395153
17:17:13	0:15:13		78.6930524	68.3812876	82.731787	70.2395153
17:17:14	0:15:14		78.6930524	68.3812876	81.731787	69.2395153
17:17:15	0:15:15		78.6930524	68.3812876	79.731787	71.2395153
17:17:16	0:15:16		79.6930524	69.3812876	77.731787	71.2395153
17:17:17	0:15:17		79.6930524	69.3812876	76.731787	71.2395153
17:17:18	0:15:18		79.6930524	70.3812876	75.731787	71.2395153
17:17:19	0:15:19		80.6930524	70.3812876	75.731787	71.2395153
17:17:20	0:15:20		88.6930524	70.3812876	76.731787	71.2395153
17:17:21	0:15:21		88.6930524	71.3812876	76.731787	71.2395153
17:17:22	0:15:22		90.6930524	73.3812876	76.731787	71.2395153
17:17:23	0:15:23		89.6930524	73.3812876	76.731787	68.2395153
17:17:24	0:15:24		89.6930524	74.3812876	76.731787	68.2395153
17:17:25	0:15:25		89.6930524	74.3812876	76.731787	68.2395153
17:17:26	0:15:26		88.6930524	74.3812876	76.731787	68.2395153
17:17:27	0:15:27		88.6930524	76.3812876	77.731787	68.2395153
17:17:28	0:15:28		85.6930524	79.3812876	76.731787	68.2395153
17:17:29	0:15:29		86.6930524	79.3812876	76.731787	68.2395153
17:17:30	0:15:30		86.6930524	80.3812876	75.731787	67.2395153
17:17:31	0:15:31		87.6930524	80.3812876	75.731787	66.2395153
17:17:32	0:15:32		87.6930524	80.3812876	75.731787	66.2395153
17:17:33	0:15:33		87.6930524	80.3812876	75.731787	66.2395153
17:17:34	0:15:34		84.6930524	80.3812876	74.731787	66.2395153
17:17:35	0:15:35		83.6930524	80.3812876	74.731787	65.2395153
17:17:36	0:15:36		84.6930524	80.3812876	73.731787	65.2395153
17:17:37	0:15:37		84.6930524	79.3812876	74.731787	68.2395153
17:17:38	0:15:38		84.6930524	79.3812876	74.731787	69.2395153
17:17:39	0:15:39		84.6930524	79.3812876	74.731787	69.2395153
17:17:40	0:15:40		83.6930524	80.3812876	74.731787	69.2395153
17:17:41	0:15:41		82.6930524	81.3812876	74.731787	69.2395153
17:17:42	0:15:42		82.6930524	81.3812876	74.731787	69.2395153

17:17:43	0:15:43		80.6930524	81.3812876	76.731787	69.2395153
17:17:44	0:15:44		79.6930524	81.3812876	76.731787	69.2395153
17:17:45	0:15:45		79.6930524	79.3812876	76.731787	69.2395153
17:17:46	0:15:46		78.6930524	79.3812876	76.731787	69.2395153
17:17:47	0:15:47		78.6930524	79.3812876	74.731787	69.2395153
17:17:48	0:15:48		78.6930524	79.3812876	74.731787	73.2395153
17:17:49	0:15:49		77.6930524	82.3812876	74.731787	74.2395153
17:17:50	0:15:50		77.6930524	82.3812876	74.731787	74.2395153
17:17:51	0:15:51		77.6930524	84.3812876	75.731787	75.2395153
17:17:52	0:15:52		76.6930524	84.3812876	76.731787	75.2395153
17:17:53	0:15:53		76.6930524	85.3812876	76.731787	75.2395153
17:17:54	0:15:54		76.6930524	86.3812876	76.731787	74.2395153
17:17:55	0:15:55		76.6930524	86.3812876	74.731787	73.2395153
17:17:56	0:15:56		76.6930524	88.3812876	73.731787	73.2395153
17:17:57	0:15:57		77.6930524	89.3812876	72.731787	73.2395153
17:17:58	0:15:58		76.6930524	91.3812876	72.731787	72.2395153
17:17:59	0:15:59		75.6930524	92.3812876	72.731787	73.2395153
17:18:00	0:16:00		74.6930524	92.3812876	72.731787	73.2395153
17:18:01	0:16:01		73.6930524	92.3812876	73.731787	72.2395153
17:18:02	0:16:02		71.6930524	88.3812876	74.731787	73.2395153
17:18:03	0:16:03		71.6930524	83.3812876	75.731787	74.2395153
17:18:04	0:16:04		71.6930524	82.3812876	76.731787	74.2395153
17:18:05	0:16:05		72.6930524	82.3812876	75.731787	73.2395153
17:18:06	0:16:06		73.6930524	79.3812876	75.731787	73.2395153
17:18:07	0:16:07		73.6930524	79.3812876	75.731787	72.2395153
17:18:08	0:16:08		74.6930524	79.3812876	75.731787	72.2395153
17:18:09	0:16:09		74.6930524	79.3812876	75.731787	72.2395153
17:18:10	0:16:10		74.6930524	79.3812876	75.731787	72.2395153
17:18:11	0:16:11		73.6930524	78.3812876	75.731787	72.2395153
17:18:12	0:16:12		73.6930524	77.3812876	76.731787	72.2395153
17:18:13	0:16:13		73.6930524	77.3812876	77.731787	74.2395153
17:18:14	0:16:14		73.6930524	76.3812876	78.731787	74.2395153
17:18:15	0:16:15		72.6930524	76.3812876	78.731787	75.2395153
17:18:16	0:16:16		72.6930524	76.3812876	78.731787	76.2395153
17:18:17	0:16:17		72.6930524	76.3812876	78.731787	77.2395153
17:18:18	0:16:18		73.6930524	76.3812876	77.731787	77.2395153
17:18:19	0:16:19		73.6930524	77.3812876	77.731787	77.2395153
17:18:20	0:16:20		74.6930524	77.3812876	77.731787	77.2395153
17:18:21	0:16:21		74.6930524	77.3812876	77.731787	77.2395153
17:18:22	0:16:22		75.6930524	77.3812876	77.731787	78.2395153
17:18:23	0:16:23		74.6930524	77.3812876	77.731787	78.2395153
17:18:24	0:16:24		74.6930524	77.3812876	78.731787	79.2395153
17:18:25	0:16:25		74.6930524	77.3812876	78.731787	80.2395153
17:18:26	0:16:26		74.6930524	79.3812876	79.731787	80.2395153
17:18:27	0:16:27		74.6930524	79.3812876	78.731787	80.2395153

17:18:28	0:16:28		75.6930524	80.3812876	78.731787	80.2395153
17:18:29	0:16:29		75.6930524	80.3812876	78.731787	81.2395153
17:18:30	0:16:30		75.6930524	81.3812876	77.731787	81.2395153
17:18:31	0:16:31		75.6930524	81.3812876	77.731787	81.2395153
17:18:32	0:16:32		75.6930524	82.3812876	77.731787	83.2395153
17:18:33	0:16:33		75.6930524	83.3812876	77.731787	85.2395153
17:18:34	0:16:34		74.6930524	84.3812876	77.731787	88.2395153
17:18:35	0:16:35		75.6930524	84.3812876	77.731787	90.2395153
17:18:36	0:16:36		75.6930524	84.3812876	77.731787	90.2395153
17:18:37	0:16:37		74.6930524	84.3812876	85.731787	92.2395153
17:18:38	0:16:38		73.6930524	84.3812876	86.731787	91.2395153
17:18:39	0:16:39		71.6930524	84.3812876	86.731787	91.2395153
17:18:40	0:16:40		71.6930524	82.3812876	87.731787	91.2395153
17:18:41	0:16:41		74.6930524	82.3812876	88.731787	90.2395153
17:18:42	0:16:42		75.6930524	82.3812876	88.731787	90.2395153
17:18:43	0:16:43		74.6930524	82.3812876	87.731787	89.2395153
17:18:44	0:16:44		74.6930524	82.3812876	87.731787	89.2395153
17:18:45	0:16:45		74.6930524	82.3812876	85.731787	87.2395153
17:18:46	0:16:46		74.6930524	82.3812876	85.731787	85.2395153
17:18:47	0:16:47		75.6930524	83.3812876	81.731787	84.2395153
17:18:48	0:16:48		76.6930524	79.3812876	81.731787	84.2395153
17:18:49	0:16:49		76.6930524	79.3812876	80.731787	85.2395153
17:18:50	0:16:50		76.6930524	79.3812876	78.731787	85.2395153
17:18:51	0:16:51		76.6930524	79.3812876	76.731787	85.2395153
17:18:52	0:16:52		76.6930524	79.3812876	75.731787	84.2395153
17:18:53	0:16:53		76.6930524	79.3812876	74.731787	84.2395153
17:18:54	0:16:54		75.6930524	79.3812876	73.731787	84.2395153
17:18:55	0:16:55		75.6930524	79.3812876	74.731787	85.2395153
17:18:56	0:16:56		74.6930524	79.3812876	74.731787	85.2395153
17:18:57	0:16:57		74.6930524	78.3812876	74.731787	81.2395153
17:18:58	0:16:58		73.6930524	78.3812876	74.731787	81.2395153
17:18:59	0:16:59		73.6930524	78.3812876	74.731787	80.2395153
17:19:00	0:17:00		73.6930524	77.3812876	75.731787	78.2395153
17:19:01	0:17:01		74.6930524	77.3812876	76.731787	76.2395153
17:19:02	0:17:02		74.6930524	77.3812876	76.731787	76.2395153
17:19:03	0:17:03		75.6930524	77.3812876	76.731787	74.2395153
17:19:04	0:17:04		76.6930524	78.3812876	78.731787	71.2395153
17:19:05	0:17:05		76.6930524	78.3812876	78.731787	71.2395153
17:19:06	0:17:06		77.6930524	78.3812876	78.731787	68.2395153
17:19:07	0:17:07		77.6930524	78.3812876	78.731787	67.2395153
17:19:08	0:17:08		77.6930524	77.3812876	77.731787	69.2395153
17:19:09	0:17:09		76.6930524	77.3812876	77.731787	70.2395153
17:19:10	0:17:10		76.6930524	75.3812876	77.731787	72.2395153
17:19:11	0:17:11		75.6930524	75.3812876	78.731787	72.2395153
17:19:12	0:17:12		75.6930524	73.3812876	76.731787	72.2395153

17:19:13	0:17:13		74.6930524	76.3812876	75.731787	72.2395153
17:19:14	0:17:14		74.6930524	79.3812876	74.731787	71.2395153
17:19:15	0:17:15		73.6930524	79.3812876	74.731787	71.2395153
17:19:16	0:17:16		73.6930524	80.3812876	74.731787	71.2395153
17:19:17	0:17:17		73.6930524	80.3812876	74.731787	71.2395153
17:19:18	0:17:18		73.6930524	81.3812876	74.731787	71.2395153
17:19:19	0:17:19		73.6930524	80.3812876	74.731787	71.2395153
17:19:20	0:17:20		73.6930524	80.3812876	72.731787	72.2395153
17:19:21	0:17:21		73.6930524	79.3812876	72.731787	72.2395153
17:19:22	0:17:22		73.6930524	79.3812876	71.731787	72.2395153
17:19:23	0:17:23		73.6930524	79.3812876	71.731787	75.2395153
17:19:24	0:17:24		74.6930524	79.3812876	71.731787	77.2395153
17:19:25	0:17:25		74.6930524	75.3812876	72.731787	79.2395153
17:19:26	0:17:26		74.6930524	74.3812876	72.731787	79.2395153
17:19:27	0:17:27		75.6930524	74.3812876	72.731787	79.2395153
17:19:28	0:17:28		76.6930524	71.3812876	75.731787	80.2395153
17:19:29	0:17:29		76.6930524	71.3812876	80.731787	77.2395153
17:19:30	0:17:30		76.6930524	71.3812876	80.731787	77.2395153
17:19:31	0:17:31		76.6930524	73.3812876	82.731787	75.2395153
17:19:32	0:17:32		77.6930524	73.3812876	82.731787	75.2395153
17:19:33	0:17:33		77.6930524	75.3812876	81.731787	75.2395153
17:19:34	0:17:34		77.6930524	75.3812876	79.731787	75.2395153
17:19:35	0:17:35		78.6930524	75.3812876	77.731787	75.2395153
17:19:36	0:17:36		78.6930524	75.3812876	75.731787	74.2395153
17:19:37	0:17:37		79.6930524	75.3812876	75.731787	74.2395153
17:19:38	0:17:38		80.6930524	74.3812876	74.731787	74.2395153
17:19:39	0:17:39		80.6930524	73.3812876	73.731787	75.2395153
17:19:40	0:17:40		80.6930524	72.3812876	73.731787	75.2395153
17:19:41	0:17:41		80.6930524	71.3812876	73.731787	75.2395153
17:19:42	0:17:42		79.6930524	70.3812876	74.731787	75.2395153
17:19:43	0:17:43		78.6930524	70.3812876	75.731787	74.2395153
17:19:44	0:17:44		76.6930524	70.3812876	75.731787	74.2395153
17:19:45	0:17:45		76.6930524	70.3812876	75.731787	74.2395153
17:19:46	0:17:46		76.6930524	70.3812876	75.731787	74.2395153
17:19:47	0:17:47		76.6930524	70.3812876	75.731787	73.2395153
17:19:48	0:17:48		76.6930524	76.3812876	74.731787	72.2395153
17:19:49	0:17:49		76.6930524	81.3812876	73.731787	71.2395153
17:19:50	0:17:50		77.6930524	81.3812876	73.731787	71.2395153
17:19:51	0:17:51		76.6930524	81.3812876	74.731787	71.2395153
17:19:52	0:17:52		76.6930524	82.3812876	74.731787	71.2395153
17:19:53	0:17:53		76.6930524	82.3812876	74.731787	72.2395153
17:19:54	0:17:54		76.6930524	83.3812876	73.731787	73.2395153
17:19:55	0:17:55		76.6930524	83.3812876	73.731787	73.2395153
17:19:56	0:17:56		76.6930524	83.3812876	72.731787	74.2395153
17:19:57	0:17:57		77.6930524	84.3812876	72.731787	73.2395153

17:19:58	0:17:58		77.6930524	85.3812876	72.731787	70.2395153
17:19:59	0:17:59		77.6930524	86.3812876	72.731787	68.2395153
17:20:00	0:18:00		78.6930524	86.3812876	72.731787	68.2395153
17:20:01	0:18:01		77.6930524	86.3812876	72.731787	68.2395153
17:20:02	0:18:02		77.6930524	86.3812876	72.731787	68.2395153
17:20:03	0:18:03		77.6930524	86.3812876	72.731787	68.2395153
17:20:04	0:18:04		77.6930524	86.3812876	72.731787	68.2395153
17:20:05	0:18:05		77.6930524	86.3812876	72.731787	70.2395153
17:20:06	0:18:06		77.6930524	86.3812876	73.731787	71.2395153
17:20:07	0:18:07		76.6930524	86.3812876	75.731787	71.2395153
17:20:08	0:18:08		75.6930524	86.3812876	75.731787	73.2395153
17:20:09	0:18:09		76.6930524	86.3812876	76.731787	74.2395153
17:20:10	0:18:10		77.6930524	86.3812876	77.731787	74.2395153
17:20:11	0:18:11		81.6930524	85.3812876	77.731787	76.2395153
17:20:12	0:18:12		85.6930524	85.3812876	77.731787	76.2395153
17:20:13	0:18:13		86.6930524	84.3812876	77.731787	76.2395153
17:20:14	0:18:14		86.6930524	85.3812876	76.731787	77.2395153
17:20:15	0:18:15		86.6930524	85.3812876	76.731787	77.2395153
17:20:16	0:18:16		86.6930524	85.3812876	76.731787	76.2395153
17:20:17	0:18:17		86.6930524	85.3812876	75.731787	74.2395153
17:20:18	0:18:18		87.6930524	84.3812876	75.731787	74.2395153
17:20:19	0:18:19		88.6930524	84.3812876	75.731787	74.2395153
17:20:20	0:18:20		88.6930524	84.3812876	75.731787	74.2395153
17:20:21	0:18:21		88.6930524	83.3812876	75.731787	76.2395153
17:20:22	0:18:22		88.6930524	83.3812876	76.731787	76.2395153
17:20:23	0:18:23		83.6930524	83.3812876	75.731787	76.2395153
17:20:24	0:18:24		83.6930524	83.3812876	75.731787	76.2395153
17:20:25	0:18:25		79.6930524	83.3812876	75.731787	78.2395153
17:20:26	0:18:26		78.6930524	83.3812876	75.731787	78.2395153
17:20:27	0:18:27		78.6930524	83.3812876	75.731787	79.2395153
17:20:28	0:18:28		78.6930524	83.3812876	75.731787	80.2395153
17:20:29	0:18:29		78.6930524	83.3812876	77.731787	80.2395153
17:20:30	0:18:30		78.6930524	86.3812876	80.731787	78.2395153
17:20:31	0:18:31		78.6930524	87.3812876	81.731787	77.2395153
17:20:32	0:18:32		80.6930524	84.3812876	80.731787	76.2395153
17:20:33	0:18:33		83.6930524	84.3812876	79.731787	76.2395153
17:20:34	0:18:34		87.6930524	82.3812876	77.731787	74.2395153
17:20:35	0:18:35		86.6930524	82.3812876	76.731787	74.2395153
17:20:36	0:18:36		85.6930524	83.3812876	76.731787	74.2395153
17:20:37	0:18:37		83.6930524	83.3812876	75.731787	73.2395153
17:20:38	0:18:38		82.6930524	83.3812876	75.731787	72.2395153
17:20:39	0:18:39		82.6930524	83.3812876	75.731787	72.2395153
17:20:40	0:18:40		81.6930524	83.3812876	75.731787	72.2395153
17:20:41	0:18:41		80.6930524	83.3812876	75.731787	72.2395153
17:20:42	0:18:42		78.6930524	82.3812876	75.731787	72.2395153

17:20:43	0:18:43		77.6930524	82.3812876	75.731787	71.2395153
17:20:44	0:18:44		76.6930524	80.3812876	75.731787	71.2395153
17:20:45	0:18:45		74.6930524	80.3812876	75.731787	68.2395153
17:20:46	0:18:46		74.6930524	80.3812876	76.731787	68.2395153
17:20:47	0:18:47		74.6930524	80.3812876	76.731787	70.2395153
17:20:48	0:18:48		75.6930524	80.3812876	76.731787	70.2395153
17:20:49	0:18:49		77.6930524	80.3812876	77.731787	70.2395153
17:20:50	0:18:50		79.6930524	82.3812876	77.731787	70.2395153
17:20:51	0:18:51		80.6930524	82.3812876	77.731787	70.2395153
17:20:52	0:18:52		81.6930524	82.3812876	78.731787	71.2395153
17:20:53	0:18:53		81.6930524	82.3812876	83.731787	71.2395153
17:20:54	0:18:54		81.6930524	82.3812876	85.731787	72.2395153
17:20:55	0:18:55		81.6930524	81.3812876	84.731787	71.2395153
17:20:56	0:18:56		81.6930524	82.3812876	82.731787	71.2395153
17:20:57	0:18:57		82.6930524	82.3812876	78.731787	71.2395153
17:20:58	0:18:58		83.6930524	82.3812876	75.731787	71.2395153
17:20:59	0:18:59		79.6930524	85.3812876	74.731787	70.2395153
17:21:00	0:19:00		79.6930524	86.3812876	74.731787	68.2395153
17:21:01	0:19:01		79.6930524	87.3812876	75.731787	67.2395153
17:21:02	0:19:02		79.6930524	87.3812876	74.731787	67.2395153
17:21:03	0:19:03		80.6930524	87.3812876	74.731787	67.2395153
17:21:04	0:19:04		80.6930524	87.3812876	74.731787	67.2395153
17:21:05	0:19:05		81.6930524	87.3812876	73.731787	68.2395153
17:21:06	0:19:06		81.6930524	87.3812876	73.731787	68.2395153
17:21:07	0:19:07		82.6930524	86.3812876	73.731787	69.2395153
17:21:08	0:19:08		82.6930524	86.3812876	73.731787	69.2395153
17:21:09	0:19:09		82.6930524	81.3812876	73.731787	70.2395153
17:21:10	0:19:10		81.6930524	81.3812876	72.731787	70.2395153
17:21:11	0:19:11		79.6930524	81.3812876	72.731787	70.2395153
17:21:12	0:19:12		78.6930524	80.3812876	72.731787	70.2395153
17:21:13	0:19:13		78.6930524	80.3812876	72.731787	71.2395153
17:21:14	0:19:14		79.6930524	80.3812876	72.731787	71.2395153
17:21:15	0:19:15		79.6930524	80.3812876	72.731787	72.2395153
17:21:16	0:19:16		79.6930524	80.3812876	72.731787	72.2395153
17:21:17	0:19:17		79.6930524	80.3812876	71.731787	72.2395153
17:21:18	0:19:18		79.6930524	80.3812876	71.731787	72.2395153
17:21:19	0:19:19		78.6930524	78.3812876	69.731787	70.2395153
17:21:20	0:19:20		78.6930524	78.3812876	68.731787	63.2395153
17:21:21	0:19:21		78.6930524	78.3812876	71.731787	62.2395153
17:21:22	0:19:22		77.6930524	78.3812876	73.731787	62.2395153
17:21:23	0:19:23		77.6930524	79.3812876	73.731787	62.2395153
17:21:24	0:19:24		76.6930524	79.3812876	73.731787	63.2395153
17:21:25	0:19:25		75.6930524	78.3812876	73.731787	63.2395153
17:21:26	0:19:26		75.6930524	78.3812876	73.731787	64.2395153
17:21:27	0:19:27		77.6930524	77.3812876	73.731787	65.2395153

17:21:28	0:19:28		78.6930524	77.3812876	73.731787	65.2395153
17:21:29	0:19:29		79.6930524	77.3812876	73.731787	66.2395153
17:21:30	0:19:30		79.6930524	78.3812876	73.731787	69.2395153
17:21:31	0:19:31		79.6930524	79.3812876	74.731787	68.2395153
17:21:32	0:19:32		79.6930524	79.3812876	74.731787	68.2395153
17:21:33	0:19:33		78.6930524	86.3812876	74.731787	69.2395153
17:21:34	0:19:34		78.6930524	85.3812876	74.731787	69.2395153
17:21:35	0:19:35		79.6930524	83.3812876	74.731787	69.2395153
17:21:36	0:19:36		81.6930524	83.3812876	74.731787	69.2395153
17:21:37	0:19:37		84.6930524	81.3812876	76.731787	74.2395153
17:21:38	0:19:38		87.6930524	81.3812876	77.731787	74.2395153
17:21:39	0:19:39		88.6930524	80.3812876	78.731787	74.2395153
17:21:40	0:19:40		89.6930524	80.3812876	78.731787	72.2395153
17:21:41	0:19:41		89.6930524	80.3812876	79.731787	72.2395153
17:21:42	0:19:42		88.6930524	80.3812876	78.731787	72.2395153
17:21:43	0:19:43		87.6930524	80.3812876	78.731787	72.2395153
17:21:44	0:19:44		86.6930524	80.3812876	79.731787	72.2395153
17:21:45	0:19:45		85.6930524	80.3812876	79.731787	72.2395153
17:21:46	0:19:46		83.6930524	80.3812876	79.731787	73.2395153
17:21:47	0:19:47		82.6930524	80.3812876	79.731787	77.2395153
17:21:48	0:19:48		82.6930524	80.3812876	79.731787	79.2395153
17:21:49	0:19:49		81.6930524	79.3812876	79.731787	79.2395153
17:21:50	0:19:50		80.6930524	79.3812876	78.731787	79.2395153
17:21:51	0:19:51		80.6930524	79.3812876	78.731787	77.2395153
17:21:52	0:19:52		80.6930524	80.3812876	78.731787	76.2395153
17:21:53	0:19:53		79.6930524	80.3812876	79.731787	76.2395153
17:21:54	0:19:54		79.6930524	80.3812876	79.731787	76.2395153
17:21:55	0:19:55		78.6930524	80.3812876	79.731787	77.2395153
17:21:56	0:19:56		78.6930524	80.3812876	79.731787	77.2395153
17:21:57	0:19:57		78.6930524	76.3812876	79.731787	77.2395153
17:21:58	0:19:58		78.6930524	76.3812876	79.731787	79.2395153
17:21:59	0:19:59		78.6930524	76.3812876	79.731787	81.2395153
17:22:00	0:20:00		78.6930524	76.3812876	78.731787	81.2395153
17:22:01	0:20:01		77.6930524	77.3812876	77.731787	82.2395153
17:22:02	0:20:02		77.6930524	77.3812876	77.731787	84.2395153
17:22:03	0:20:03		76.6930524	78.3812876	78.731787	84.2395153
17:22:04	0:20:04		75.6930524	80.3812876	78.731787	85.2395153
17:22:05	0:20:05		75.6930524	80.3812876	78.731787	85.2395153
17:22:06	0:20:06		76.6930524	80.3812876	78.731787	85.2395153
17:22:07	0:20:07		77.6930524	80.3812876	79.731787	85.2395153
17:22:08	0:20:08		77.6930524	82.3812876	79.731787	85.2395153
17:22:09	0:20:09		77.6930524	83.3812876	79.731787	85.2395153
17:22:10	0:20:10		77.6930524	85.3812876	79.731787	85.2395153
17:22:11	0:20:11		77.6930524	85.3812876	79.731787	87.2395153
17:22:12	0:20:12		77.6930524	85.3812876	79.731787	87.2395153

17:22:13	0:20:13		76.6930524	88.3812876	79.731787	90.2395153
17:22:14	0:20:14		76.6930524	89.3812876	79.731787	92.2395153
17:22:15	0:20:15		76.6930524	90.3812876	79.731787	95.2395153
17:22:16	0:20:16		76.6930524	89.3812876	79.731787	95.2395153
17:22:17	0:20:17		76.6930524	89.3812876	79.731787	98.2395153
17:22:18	0:20:18		77.6930524	89.3812876	79.731787	98.2395153
17:22:19	0:20:19		79.6930524	88.3812876	79.731787	98.2395153
17:22:20	0:20:20		82.6930524	87.3812876	79.731787	82.2395153
17:22:21	0:20:21		86.6930524	87.3812876	79.731787	82.2395153
17:22:22	0:20:22		88.6930524	87.3812876	79.731787	73.2395153
17:22:23	0:20:23		89.6930524	85.3812876	79.731787	73.2395153
17:22:24	0:20:24		90.6930524	84.3812876	78.731787	73.2395153
17:22:25	0:20:25		90.6930524	84.3812876	77.731787	72.2395153
17:22:26	0:20:26		90.6930524	84.3812876	77.731787	72.2395153
17:22:27	0:20:27		90.6930524	83.3812876	77.731787	71.2395153
17:22:28	0:20:28		86.6930524	83.3812876	77.731787	71.2395153
17:22:29	0:20:29		82.6930524	82.3812876	76.731787	71.2395153
17:22:30	0:20:30		80.6930524	82.3812876	76.731787	71.2395153
17:22:31	0:20:31		80.6930524	80.3812876	76.731787	71.2395153
17:22:32	0:20:32		79.6930524	80.3812876	75.731787	71.2395153
17:22:33	0:20:33		79.6930524	80.3812876	73.731787	71.2395153
17:22:34	0:20:34		78.6930524	80.3812876	73.731787	70.2395153
17:22:35	0:20:35		78.6930524	80.3812876	73.731787	69.2395153
17:22:36	0:20:36		78.6930524	80.3812876	81.731787	69.2395153
17:22:37	0:20:37		77.6930524	80.3812876	81.731787	69.2395153
17:22:38	0:20:38		77.6930524	81.3812876	77.731787	68.2395153
17:22:39	0:20:39		77.6930524	80.3812876	76.731787	68.2395153
17:22:40	0:20:40		77.6930524	80.3812876	75.731787	63.2395153
17:22:41	0:20:41		77.6930524	81.3812876	74.731787	63.2395153
17:22:42	0:20:42		77.6930524	82.3812876	74.731787	63.2395153
17:22:43	0:20:43		77.6930524	82.3812876	73.731787	62.2395153
17:22:44	0:20:44		77.6930524	82.3812876	73.731787	59.2395153
17:22:45	0:20:45		76.6930524	82.3812876	73.731787	59.2395153
17:22:46	0:20:46		78.6930524	82.3812876	73.731787	58.2395153
17:22:47	0:20:47		79.6930524	82.3812876	73.731787	63.2395153
17:22:48	0:20:48		99.6930524	82.3812876	73.731787	66.2395153
17:22:49	0:20:49		107.693052	87.3812876	73.731787	66.2395153
17:22:50	0:20:50		111.693052	88.3812876	72.731787	68.2395153
17:22:51	0:20:51		111.693052	88.3812876	72.731787	70.2395153
17:22:52	0:20:52		112.693052	87.3812876	72.731787	70.2395153
17:22:53	0:20:53		114.693052	86.3812876	72.731787	70.2395153
17:22:54	0:20:54		117.693052	86.3812876	70.731787	70.2395153
17:22:55	0:20:55		117.693052	85.3812876	70.731787	70.2395153
17:22:56	0:20:56		117.693052	83.3812876	69.731787	70.2395153
17:22:57	0:20:57		115.693052	83.3812876	69.731787	71.2395153

17:22:58	0:20:58		111.693052	82.3812876	77.731787	71.2395153
17:22:59	0:20:59		107.693052	82.3812876	78.731787	72.2395153
17:23:00	0:21:00		91.6930524	82.3812876	78.731787	71.2395153
17:23:01	0:21:01		91.6930524	80.3812876	78.731787	71.2395153
17:23:02	0:21:02		91.6930524	79.3812876	78.731787	71.2395153
17:23:03	0:21:03		91.6930524	76.3812876	78.731787	71.2395153
17:23:04	0:21:04		91.6930524	76.3812876	77.731787	71.2395153
17:23:05	0:21:05		89.6930524	71.3812876	77.731787	71.2395153
17:23:06	0:21:06		89.6930524	71.3812876	77.731787	71.2395153
17:23:07	0:21:07		86.6930524	71.3812876	75.731787	71.2395153
17:23:08	0:21:08		82.6930524	71.3812876	75.731787	73.2395153
17:23:09	0:21:09		80.6930524	71.3812876	76.731787	75.2395153
17:23:10	0:21:10		80.6930524	71.3812876	77.731787	79.2395153
17:23:11	0:21:11		79.6930524	70.3812876	77.731787	79.2395153
17:23:12	0:21:12		79.6930524	70.3812876	77.731787	76.2395153
17:23:13	0:21:13		79.6930524	70.3812876	78.731787	76.2395153
17:23:14	0:21:14		79.6930524	70.3812876	78.731787	76.2395153
17:23:15	0:21:15		79.6930524	69.3812876	78.731787	75.2395153
17:23:16	0:21:16		80.6930524	69.3812876	78.731787	75.2395153
17:23:17	0:21:17		79.6930524	74.3812876	78.731787	73.2395153
17:23:18	0:21:18		79.6930524	76.3812876	78.731787	73.2395153
17:23:19	0:21:19		79.6930524	77.3812876	78.731787	72.2395153
17:23:20	0:21:20		79.6930524	77.3812876	78.731787	71.2395153
17:23:21	0:21:21		78.6930524	77.3812876	78.731787	71.2395153
17:23:22	0:21:22		78.6930524	77.3812876	80.731787	71.2395153
17:23:23	0:21:23		78.6930524	77.3812876	81.731787	71.2395153
17:23:24	0:21:24		78.6930524	78.3812876	81.731787	73.2395153
17:23:25	0:21:25		77.6930524	78.3812876	83.731787	73.2395153
17:23:26	0:21:26		77.6930524	80.3812876	83.731787	75.2395153
17:23:27	0:21:27		78.6930524	80.3812876	83.731787	75.2395153
17:23:28	0:21:28		78.6930524	80.3812876	80.731787	75.2395153
17:23:29	0:21:29		78.6930524	80.3812876	80.731787	75.2395153
17:23:30	0:21:30		77.6930524	80.3812876	79.731787	75.2395153
17:23:31	0:21:31		77.6930524	81.3812876	79.731787	75.2395153
17:23:32	0:21:32		78.6930524	81.3812876	79.731787	77.2395153
17:23:33	0:21:33		78.6930524	81.3812876	81.731787	77.2395153
17:23:34	0:21:34		79.6930524	82.3812876	81.731787	77.2395153
17:23:35	0:21:35		79.6930524	82.3812876	81.731787	77.2395153
17:23:36	0:21:36		78.6930524	82.3812876	82.731787	77.2395153
17:23:37	0:21:37		78.6930524	82.3812876	82.731787	77.2395153
17:23:38	0:21:38		78.6930524	82.3812876	82.731787	77.2395153
17:23:39	0:21:39		78.6930524	82.3812876	82.731787	77.2395153
17:23:40	0:21:40		78.6930524	82.3812876	82.731787	76.2395153
17:23:41	0:21:41		77.6930524	79.3812876	82.731787	76.2395153
17:23:42	0:21:42		76.6930524	79.3812876	82.731787	77.2395153

17:23:43	0:21:43		76.6930524	76.3812876	84.731787	77.2395153
17:23:44	0:21:44		76.6930524	74.3812876	84.731787	76.2395153
17:23:45	0:21:45		77.6930524	76.3812876	85.731787	74.2395153
17:23:46	0:21:46		78.6930524	77.3812876	85.731787	73.2395153
17:23:47	0:21:47		77.6930524	78.3812876	85.731787	71.2395153
17:23:48	0:21:48		77.6930524	78.3812876	85.731787	71.2395153
17:23:49	0:21:49		77.6930524	78.3812876	84.731787	71.2395153
17:23:50	0:21:50		77.6930524	79.3812876	84.731787	71.2395153
17:23:51	0:21:51		77.6930524	80.3812876	83.731787	71.2395153
17:23:52	0:21:52		80.6930524	80.3812876	80.731787	71.2395153
17:23:53	0:21:53		82.6930524	80.3812876	80.731787	75.2395153
17:23:54	0:21:54		82.6930524	80.3812876	80.731787	76.2395153
17:23:55	0:21:55		82.6930524	80.3812876	80.731787	75.2395153
17:23:56	0:21:56		90.6930524	81.3812876	80.731787	75.2395153
17:23:57	0:21:57		95.6930524	81.3812876	80.731787	75.2395153
17:23:58	0:21:58		95.6930524	81.3812876	79.731787	75.2395153
17:23:59	0:21:59		95.6930524	81.3812876	78.731787	75.2395153
17:24:00	0:22:00		95.6930524	81.3812876	78.731787	75.2395153
17:24:01	0:22:01		95.6930524	82.3812876	78.731787	75.2395153
17:24:02	0:22:02		95.6930524	82.3812876	76.731787	75.2395153
17:24:03	0:22:03		95.6930524	81.3812876	76.731787	75.2395153
17:24:04	0:22:04		95.6930524	80.3812876	76.731787	73.2395153
17:24:05	0:22:05		95.6930524	79.3812876	74.731787	73.2395153
17:24:06	0:22:06		95.6930524	79.3812876	74.731787	73.2395153
17:24:07	0:22:07		97.6930524	77.3812876	74.731787	72.2395153
17:24:08	0:22:08		97.6930524	77.3812876	73.731787	72.2395153
17:24:09	0:22:09		98.6930524	77.3812876	73.731787	72.2395153
17:24:10	0:22:10		98.6930524	77.3812876	73.731787	73.2395153
17:24:11	0:22:11		99.6930524	77.3812876	73.731787	73.2395153
17:24:12	0:22:12		99.6930524	77.3812876	73.731787	73.2395153
17:24:13	0:22:13		84.6930524	77.3812876	73.731787	73.2395153
17:24:14	0:22:14		81.6930524	77.3812876	73.731787	74.2395153
17:24:15	0:22:15		78.6930524	77.3812876	73.731787	73.2395153
17:24:16	0:22:16		78.6930524	77.3812876	73.731787	73.2395153
17:24:17	0:22:17		78.6930524	75.3812876	72.731787	72.2395153
17:24:18	0:22:18		78.6930524	75.3812876	72.731787	72.2395153
17:24:19	0:22:19		78.6930524	74.3812876	72.731787	72.2395153
17:24:20	0:22:20		78.6930524	74.3812876	74.731787	72.2395153
17:24:21	0:22:21		77.6930524	75.3812876	78.731787	72.2395153
17:24:22	0:22:22		76.6930524	76.3812876	81.731787	72.2395153
17:24:23	0:22:23		76.6930524	77.3812876	82.731787	72.2395153
17:24:24	0:22:24		75.6930524	77.3812876	82.731787	71.2395153
17:24:25	0:22:25		75.6930524	89.3812876	83.731787	71.2395153
17:24:26	0:22:26		75.6930524	90.3812876	83.731787	73.2395153
17:24:27	0:22:27		75.6930524	90.3812876	84.731787	73.2395153

17:24:28	0:22:28		76.6930524	90.3812876	84.731787	73.2395153
17:24:29	0:22:29		77.6930524	81.3812876	87.731787	74.2395153
17:24:30	0:22:30		77.6930524	81.3812876	88.731787	74.2395153
17:24:31	0:22:31		77.6930524	81.3812876	88.731787	74.2395153
17:24:32	0:22:32		76.6930524	81.3812876	88.731787	74.2395153
17:24:33	0:22:33		76.6930524	81.3812876	88.731787	74.2395153
17:24:34	0:22:34		76.6930524	81.3812876	89.731787	75.2395153
17:24:35	0:22:35		76.6930524	80.3812876	90.731787	75.2395153
17:24:36	0:22:36		75.6930524	76.3812876	90.731787	75.2395153
17:24:37	0:22:37		77.6930524	76.3812876	90.731787	75.2395153
17:24:38	0:22:38		77.6930524	72.3812876	90.731787	75.2395153
17:24:39	0:22:39		77.6930524	73.3812876	90.731787	74.2395153
17:24:40	0:22:40		77.6930524	75.3812876	91.731787	74.2395153
17:24:41	0:22:41		77.6930524	77.3812876	91.731787	73.2395153
17:24:42	0:22:42		77.6930524	78.3812876	91.731787	73.2395153
17:24:43	0:22:43		77.6930524	78.3812876	91.731787	73.2395153
17:24:44	0:22:44		77.6930524	80.3812876	91.731787	73.2395153
17:24:45	0:22:45		75.6930524	81.3812876	91.731787	73.2395153
17:24:46	0:22:46		74.6930524	81.3812876	91.731787	73.2395153
17:24:47	0:22:47		74.6930524	81.3812876	91.731787	74.2395153
17:24:48	0:22:48		74.6930524	81.3812876	91.731787	74.2395153
17:24:49	0:22:49		73.6930524	81.3812876	91.731787	73.2395153
17:24:50	0:22:50		73.6930524	81.3812876	91.731787	76.2395153
17:24:51	0:22:51		74.6930524	80.3812876	91.731787	77.2395153
17:24:52	0:22:52		74.6930524	80.3812876	90.731787	78.2395153
17:24:53	0:22:53		74.6930524	80.3812876	88.731787	78.2395153
17:24:54	0:22:54		74.6930524	80.3812876	88.731787	78.2395153
17:24:55	0:22:55		75.6930524	80.3812876	86.731787	77.2395153
17:24:56	0:22:56		75.6930524	80.3812876	86.731787	76.2395153
17:24:57	0:22:57		75.6930524	79.3812876	86.731787	75.2395153
17:24:58	0:22:58		75.6930524	81.3812876	86.731787	74.2395153
17:24:59	0:22:59		75.6930524	83.3812876	86.731787	73.2395153
17:25:00	0:23:00		77.6930524	83.3812876	85.731787	72.2395153
17:25:01	0:23:01		77.6930524	83.3812876	84.731787	72.2395153
17:25:02	0:23:02		85.6930524	81.3812876	84.731787	72.2395153
17:25:03	0:23:03		86.6930524	78.3812876	81.731787	73.2395153
17:25:04	0:23:04		87.6930524	78.3812876	80.731787	73.2395153
17:25:05	0:23:05		90.6930524	75.3812876	79.731787	73.2395153
17:25:06	0:23:06		90.6930524	74.3812876	79.731787	73.2395153
17:25:07	0:23:07		92.6930524	74.3812876	76.731787	73.2395153
17:25:08	0:23:08		92.6930524	74.3812876	76.731787	73.2395153
17:25:09	0:23:09		91.6930524	74.3812876	77.731787	73.2395153
17:25:10	0:23:10		90.6930524	74.3812876	76.731787	73.2395153
17:25:11	0:23:11		88.6930524	74.3812876	76.731787	73.2395153
17:25:12	0:23:12		87.6930524	74.3812876	78.731787	73.2395153

17:25:13	0:23:13		83.6930524	74.3812876	79.731787	73.2395153
17:25:14	0:23:14		81.6930524	75.3812876	78.731787	73.2395153
17:25:15	0:23:15		80.6930524	75.3812876	77.731787	74.2395153
17:25:16	0:23:16		80.6930524	76.3812876	76.731787	74.2395153
17:25:17	0:23:17		79.6930524	76.3812876	76.731787	74.2395153
17:25:18	0:23:18		79.6930524	76.3812876	75.731787	74.2395153
17:25:19	0:23:19		79.6930524	77.3812876	75.731787	73.2395153
17:25:20	0:23:20		79.6930524	78.3812876	75.731787	73.2395153
17:25:21	0:23:21		79.6930524	80.3812876	75.731787	73.2395153
17:25:22	0:23:22		79.6930524	80.3812876	75.731787	74.2395153
17:25:23	0:23:23		78.6930524	81.3812876	75.731787	74.2395153
17:25:24	0:23:24		77.6930524	81.3812876	75.731787	74.2395153
17:25:25	0:23:25		77.6930524	81.3812876	75.731787	76.2395153
17:25:26	0:23:26		77.6930524	80.3812876	76.731787	76.2395153
17:25:27	0:23:27		76.6930524	80.3812876	76.731787	76.2395153
17:25:28	0:23:28		77.6930524	80.3812876	76.731787	76.2395153
17:25:29	0:23:29		78.6930524	80.3812876	77.731787	76.2395153
17:25:30	0:23:30		77.6930524	80.3812876	80.731787	76.2395153
17:25:31	0:23:31		77.6930524	80.3812876	81.731787	80.2395153
17:25:32	0:23:32		78.6930524	80.3812876	82.731787	80.2395153
17:25:33	0:23:33		79.6930524	80.3812876	82.731787	83.2395153
17:25:34	0:23:34		79.6930524	80.3812876	82.731787	84.2395153
17:25:35	0:23:35		79.6930524	80.3812876	82.731787	84.2395153
17:25:36	0:23:36		79.6930524	80.3812876	81.731787	84.2395153
17:25:37	0:23:37		79.6930524	80.3812876	81.731787	84.2395153
17:25:38	0:23:38		79.6930524	80.3812876	82.731787	87.2395153
17:25:39	0:23:39		79.6930524	80.3812876	82.731787	87.2395153
17:25:40	0:23:40		77.6930524	78.3812876	81.731787	87.2395153
17:25:41	0:23:41		76.6930524	77.3812876	81.731787	88.2395153
17:25:42	0:23:42		76.6930524	77.3812876	81.731787	88.2395153
17:25:43	0:23:43		76.6930524	77.3812876	81.731787	88.2395153
17:25:44	0:23:44		76.6930524	77.3812876	81.731787	89.2395153
17:25:45	0:23:45		76.6930524	77.3812876	81.731787	89.2395153
17:25:46	0:23:46		76.6930524	81.3812876	81.731787	89.2395153
17:25:47	0:23:47		76.6930524	82.3812876	80.731787	89.2395153
17:25:48	0:23:48		76.6930524	82.3812876	80.731787	89.2395153
17:25:49	0:23:49		75.6930524	82.3812876	79.731787	89.2395153
17:25:50	0:23:50		75.6930524	82.3812876	78.731787	89.2395153
17:25:51	0:23:51		75.6930524	82.3812876	79.731787	86.2395153
17:25:52	0:23:52		74.6930524	82.3812876	79.731787	82.2395153
17:25:53	0:23:53		73.6930524	82.3812876	75.731787	81.2395153
17:25:54	0:23:54		73.6930524	83.3812876	74.731787	80.2395153
17:25:55	0:23:55		73.6930524	83.3812876	73.731787	79.2395153
17:25:56	0:23:56		72.6930524	83.3812876	73.731787	74.2395153
17:25:57	0:23:57		72.6930524	85.3812876	73.731787	71.2395153

17:25:58	0:23:58		72.6930524	85.3812876	73.731787	68.2395153
17:25:59	0:23:59		72.6930524	85.3812876	73.731787	66.2395153
17:26:00	0:24:00		72.6930524	85.3812876	75.731787	65.2395153
17:26:01	0:24:01		73.6930524	86.3812876	77.731787	64.2395153
17:26:02	0:24:02		78.6930524	86.3812876	77.731787	63.2395153
17:26:03	0:24:03		79.6930524	87.3812876	76.731787	65.2395153
17:26:04	0:24:04		79.6930524	88.3812876	74.731787	66.2395153
17:26:05	0:24:05		79.6930524	86.3812876	73.731787	67.2395153
17:26:06	0:24:06		79.6930524	80.3812876	72.731787	67.2395153
17:26:07	0:24:07		80.6930524	79.3812876	71.731787	67.2395153
17:26:08	0:24:08		77.6930524	79.3812876	71.731787	67.2395153
17:26:09	0:24:09		76.6930524	77.3812876	72.731787	67.2395153
17:26:10	0:24:10		76.6930524	77.3812876	73.731787	67.2395153
17:26:11	0:24:11		76.6930524	76.3812876	73.731787	67.2395153
17:26:12	0:24:12		76.6930524	75.3812876	73.731787	67.2395153
17:26:13	0:24:13		76.6930524	75.3812876	73.731787	68.2395153
17:26:14	0:24:14		77.6930524	71.3812876	73.731787	69.2395153
17:26:15	0:24:15		77.6930524	73.3812876	72.731787	70.2395153
17:26:16	0:24:16		77.6930524	75.3812876	72.731787	72.2395153
17:26:17	0:24:17		77.6930524	75.3812876	72.731787	72.2395153
17:26:18	0:24:18		77.6930524	75.3812876	72.731787	73.2395153
17:26:19	0:24:19		77.6930524	74.3812876	73.731787	76.2395153
17:26:20	0:24:20		77.6930524	74.3812876	74.731787	76.2395153
17:26:21	0:24:21		77.6930524	74.3812876	75.731787	77.2395153
17:26:22	0:24:22		76.6930524	78.3812876	75.731787	77.2395153
17:26:23	0:24:23		76.6930524	78.3812876	75.731787	77.2395153
17:26:24	0:24:24		76.6930524	77.3812876	75.731787	76.2395153
17:26:25	0:24:25		75.6930524	77.3812876	75.731787	76.2395153
17:26:26	0:24:26		75.6930524	75.3812876	76.731787	76.2395153
17:26:27	0:24:27		75.6930524	75.3812876	76.731787	76.2395153
17:26:28	0:24:28		75.6930524	75.3812876	76.731787	77.2395153
17:26:29	0:24:29		75.6930524	74.3812876	75.731787	77.2395153
17:26:30	0:24:30		76.6930524	74.3812876	75.731787	76.2395153
17:26:31	0:24:31		77.6930524	74.3812876	75.731787	75.2395153
17:26:32	0:24:32		77.6930524	74.3812876	75.731787	75.2395153
17:26:33	0:24:33		77.6930524	74.3812876	73.731787	75.2395153
17:26:34	0:24:34		77.6930524	72.3812876	73.731787	75.2395153
17:26:35	0:24:35		77.6930524	72.3812876	72.731787	75.2395153
17:26:36	0:24:36		77.6930524	71.3812876	72.731787	75.2395153
17:26:37	0:24:37		77.6930524	76.3812876	72.731787	73.2395153
17:26:38	0:24:38		77.6930524	76.3812876	72.731787	71.2395153
17:26:39	0:24:39		77.6930524	76.3812876	72.731787	72.2395153
17:26:40	0:24:40		78.6930524	76.3812876	71.731787	72.2395153
17:26:41	0:24:41		78.6930524	73.3812876	70.731787	73.2395153
17:26:42	0:24:42		78.6930524	73.3812876	70.731787	73.2395153

17:26:43	0:24:43		82.6930524	73.3812876	69.731787	73.2395153
17:26:44	0:24:44		82.6930524	72.3812876	68.731787	74.2395153
17:26:45	0:24:45		84.6930524	72.3812876	68.731787	75.2395153
17:26:46	0:24:46		84.6930524	72.3812876	65.731787	75.2395153
17:26:47	0:24:47		84.6930524	72.3812876	65.731787	75.2395153
17:26:48	0:24:48		86.6930524	72.3812876	64.731787	74.2395153
17:26:49	0:24:49		87.6930524	72.3812876	63.731787	73.2395153
17:26:50	0:24:50		87.6930524	73.3812876	62.731787	73.2395153
17:26:51	0:24:51		88.6930524	73.3812876	62.731787	71.2395153
17:26:52	0:24:52		84.6930524	73.3812876	63.731787	69.2395153
17:26:53	0:24:53		83.6930524	74.3812876	63.731787	67.2395153
17:26:54	0:24:54		83.6930524	74.3812876	64.731787	66.2395153
17:26:55	0:24:55		83.6930524	74.3812876	65.731787	66.2395153
17:26:56	0:24:56		82.6930524	74.3812876	67.731787	66.2395153
17:26:57	0:24:57		82.6930524	74.3812876	72.731787	67.2395153
17:26:58	0:24:58		82.6930524	76.3812876	77.731787	69.2395153
17:26:59	0:24:59		81.6930524	76.3812876	77.731787	70.2395153
17:27:00	0:25:00		80.6930524	74.3812876	79.731787	72.2395153
17:27:01	0:25:01		80.6930524	73.3812876	80.731787	75.2395153
17:27:02	0:25:02		78.6930524	77.3812876	81.731787	76.2395153
17:27:03	0:25:03		78.6930524	77.3812876	79.731787	78.2395153
17:27:04	0:25:04		78.6930524	78.3812876	79.731787	79.2395153
17:27:05	0:25:05		78.6930524	78.3812876	79.731787	80.2395153
17:27:06	0:25:06		78.6930524	79.3812876	79.731787	80.2395153
17:27:07	0:25:07		78.6930524	81.3812876	79.731787	80.2395153
17:27:08	0:25:08		79.6930524	83.3812876	79.731787	80.2395153
17:27:09	0:25:09		80.6930524	84.3812876	80.731787	78.2395153
17:27:10	0:25:10		80.6930524	83.3812876	80.731787	77.2395153
17:27:11	0:25:11		80.6930524	80.3812876	80.731787	76.2395153
17:27:12	0:25:12		80.6930524	80.3812876	77.731787	75.2395153
17:27:13	0:25:13		81.6930524	79.3812876	77.731787	75.2395153
17:27:14	0:25:14		82.6930524	77.3812876	77.731787	75.2395153
17:27:15	0:25:15		82.6930524	77.3812876	77.731787	75.2395153
17:27:16	0:25:16		82.6930524	76.3812876	75.731787	75.2395153
17:27:17	0:25:17		82.6930524	74.3812876	75.731787	76.2395153
17:27:18	0:25:18		82.6930524	73.3812876	76.731787	76.2395153
17:27:19	0:25:19		81.6930524	73.3812876	78.731787	76.2395153
17:27:20	0:25:20		81.6930524	72.3812876	81.731787	74.2395153
17:27:21	0:25:21		80.6930524	72.3812876	84.731787	74.2395153
17:27:22	0:25:22		80.6930524	72.3812876	87.731787	74.2395153
17:27:23	0:25:23		80.6930524	72.3812876	88.731787	75.2395153
17:27:24	0:25:24		80.6930524	70.3812876	88.731787	76.2395153
17:27:25	0:25:25		77.6930524	68.3812876	88.731787	75.2395153
17:27:26	0:25:26		76.6930524	64.3812876	88.731787	73.2395153
17:27:27	0:25:27		76.6930524	64.3812876	88.731787	71.2395153

17:27:28	0:25:28		77.6930524	64.3812876	88.731787	71.2395153
17:27:29	0:25:29		77.6930524	64.3812876	87.731787	71.2395153
17:27:30	0:25:30		78.6930524	64.3812876	87.731787	69.2395153
17:27:31	0:25:31		79.6930524	64.3812876	87.731787	68.2395153
17:27:32	0:25:32		79.6930524	64.3812876	86.731787	70.2395153
17:27:33	0:25:33		80.6930524	64.3812876	85.731787	70.2395153
17:27:34	0:25:34		80.6930524	64.3812876	85.731787	69.2395153
17:27:35	0:25:35		80.6930524	65.3812876	85.731787	69.2395153
17:27:36	0:25:36		80.6930524	65.3812876	85.731787	69.2395153
17:27:37	0:25:37		80.6930524	69.3812876	85.731787	70.2395153
17:27:38	0:25:38		80.6930524	69.3812876	85.731787	69.2395153
17:27:39	0:25:39		79.6930524	70.3812876	85.731787	69.2395153
17:27:40	0:25:40		79.6930524	70.3812876	86.731787	69.2395153
17:27:41	0:25:41		79.6930524	70.3812876	80.731787	68.2395153
17:27:42	0:25:42		74.6930524	70.3812876	80.731787	68.2395153
17:27:43	0:25:43		74.6930524	71.3812876	80.731787	68.2395153
17:27:44	0:25:44		74.6930524	71.3812876	80.731787	68.2395153
17:27:45	0:25:45		75.6930524	70.3812876	80.731787	69.2395153
17:27:46	0:25:46		76.6930524	70.3812876	80.731787	73.2395153
17:27:47	0:25:47		77.6930524	70.3812876	79.731787	73.2395153
17:27:48	0:25:48		77.6930524	70.3812876	77.731787	74.2395153
17:27:49	0:25:49		78.6930524	70.3812876	76.731787	74.2395153
17:27:50	0:25:50		79.6930524	71.3812876	76.731787	78.2395153
17:27:51	0:25:51		79.6930524	73.3812876	77.731787	81.2395153
17:27:52	0:25:52		80.6930524	73.3812876	77.731787	82.2395153
17:27:53	0:25:53		80.6930524	74.3812876	77.731787	82.2395153
17:27:54	0:25:54		80.6930524	74.3812876	77.731787	80.2395153
17:27:55	0:25:55		82.6930524	74.3812876	77.731787	80.2395153
17:27:56	0:25:56		82.6930524	74.3812876	77.731787	79.2395153
17:27:57	0:25:57		82.6930524	75.3812876	77.731787	77.2395153
17:27:58	0:25:58		77.6930524	76.3812876	76.731787	77.2395153
17:27:59	0:25:59		76.6930524	76.3812876	76.731787	75.2395153
17:28:00	0:26:00		75.6930524	76.3812876	76.731787	70.2395153
17:28:01	0:26:01		75.6930524	77.3812876	76.731787	69.2395153
17:28:02	0:26:02		75.6930524	77.3812876	76.731787	68.2395153
17:28:03	0:26:03		74.6930524	77.3812876	76.731787	67.2395153
17:28:04	0:26:04		74.6930524	77.3812876	76.731787	67.2395153
17:28:05	0:26:05		74.6930524	77.3812876	77.731787	67.2395153
17:28:06	0:26:06		74.6930524	77.3812876	77.731787	67.2395153
17:28:07	0:26:07		74.6930524	77.3812876	79.731787	67.2395153
17:28:08	0:26:08		73.6930524	77.3812876	81.731787	67.2395153
17:28:09	0:26:09		73.6930524	77.3812876	83.731787	66.2395153
17:28:10	0:26:10		74.6930524	77.3812876	84.731787	66.2395153
17:28:11	0:26:11		75.6930524	77.3812876	85.731787	66.2395153
17:28:12	0:26:12		76.6930524	78.3812876	85.731787	66.2395153

17:28:13	0:26:13		76.6930524	77.3812876	84.731787	66.2395153
17:28:14	0:26:14		76.6930524	77.3812876	79.731787	68.2395153
17:28:15	0:26:15		75.6930524	75.3812876	77.731787	69.2395153
17:28:16	0:26:16		75.6930524	75.3812876	76.731787	69.2395153
17:28:17	0:26:17		74.6930524	73.3812876	75.731787	70.2395153
17:28:18	0:26:18		74.6930524	72.3812876	75.731787	70.2395153
17:28:19	0:26:19		74.6930524	72.3812876	74.731787	69.2395153
17:28:20	0:26:20		74.6930524	73.3812876	74.731787	69.2395153
17:28:21	0:26:21		74.6930524	77.3812876	74.731787	68.2395153
17:28:22	0:26:22		74.6930524	77.3812876	74.731787	69.2395153
17:28:23	0:26:23		74.6930524	77.3812876	77.731787	69.2395153
17:28:24	0:26:24		74.6930524	76.3812876	81.731787	71.2395153
17:28:25	0:26:25		74.6930524	76.3812876	81.731787	71.2395153
17:28:26	0:26:26		74.6930524	77.3812876	80.731787	72.2395153
17:28:27	0:26:27		74.6930524	78.3812876	76.731787	72.2395153
17:28:28	0:26:28		75.6930524	77.3812876	76.731787	72.2395153
17:28:29	0:26:29		75.6930524	77.3812876	76.731787	73.2395153
17:28:30	0:26:30		75.6930524	77.3812876	76.731787	73.2395153
17:28:31	0:26:31		72.6930524	74.3812876	74.731787	73.2395153
17:28:32	0:26:32		71.6930524	72.3812876	74.731787	73.2395153
17:28:33	0:26:33		71.6930524	72.3812876	74.731787	74.2395153
17:28:34	0:26:34		71.6930524	72.3812876	74.731787	74.2395153
17:28:35	0:26:35		71.6930524	72.3812876	74.731787	74.2395153
17:28:36	0:26:36		71.6930524	71.3812876	74.731787	74.2395153
17:28:37	0:26:37		70.6930524	69.3812876	74.731787	74.2395153
17:28:38	0:26:38		70.6930524	69.3812876	73.731787	74.2395153
17:28:39	0:26:39		70.6930524	69.3812876	70.731787	74.2395153
17:28:40	0:26:40		70.6930524	68.3812876	68.731787	74.2395153
17:28:41	0:26:41		70.6930524	68.3812876	67.731787	73.2395153
17:28:42	0:26:42		72.6930524	73.3812876	66.731787	73.2395153
17:28:43	0:26:43		72.6930524	75.3812876	66.731787	73.2395153
17:28:44	0:26:44		72.6930524	78.3812876	65.731787	73.2395153
17:28:45	0:26:45		72.6930524	77.3812876	66.731787	73.2395153
17:28:46	0:26:46		72.6930524	77.3812876	67.731787	71.2395153
17:28:47	0:26:47		72.6930524	76.3812876	67.731787	68.2395153
17:28:48	0:26:48		72.6930524	73.3812876	70.731787	67.2395153
17:28:49	0:26:49		72.6930524	71.3812876	73.731787	67.2395153
17:28:50	0:26:50		72.6930524	71.3812876	74.731787	68.2395153
17:28:51	0:26:51		73.6930524	71.3812876	73.731787	69.2395153
17:28:52	0:26:52		73.6930524	71.3812876	71.731787	69.2395153
17:28:53	0:26:53		73.6930524	71.3812876	70.731787	68.2395153
17:28:54	0:26:54		74.6930524	71.3812876	70.731787	68.2395153
17:28:55	0:26:55		74.6930524	73.3812876	70.731787	68.2395153
17:28:56	0:26:56		74.6930524	74.3812876	70.731787	68.2395153
17:28:57	0:26:57		74.6930524	74.3812876	72.731787	68.2395153

17:28:58	0:26:58		73.6930524	74.3812876	72.731787	68.2395153
17:28:59	0:26:59		73.6930524	74.3812876	72.731787	68.2395153
17:29:00	0:27:00		73.6930524	73.3812876	72.731787	67.2395153
17:29:01	0:27:01		73.6930524	74.3812876	71.731787	68.2395153
17:29:02	0:27:02		73.6930524	73.3812876	71.731787	69.2395153
17:29:03	0:27:03		73.6930524	73.3812876	71.731787	70.2395153
17:29:04	0:27:04		73.6930524	73.3812876	71.731787	70.2395153
17:29:05	0:27:05		73.6930524	72.3812876	71.731787	69.2395153
17:29:06	0:27:06		73.6930524	73.3812876	71.731787	68.2395153
17:29:07	0:27:07		72.6930524	75.3812876	71.731787	67.2395153
17:29:08	0:27:08		72.6930524	75.3812876	71.731787	63.2395153
17:29:09	0:27:09		72.6930524	75.3812876	71.731787	62.2395153
17:29:10	0:27:10		72.6930524	74.3812876	72.731787	62.2395153
17:29:11	0:27:11		72.6930524	73.3812876	73.731787	62.2395153
17:29:12	0:27:12		73.6930524	70.3812876	73.731787	62.2395153
17:29:13	0:27:13		73.6930524	69.3812876	71.731787	62.2395153
17:29:14	0:27:14		73.6930524	69.3812876	70.731787	61.2395153
17:29:15	0:27:15		73.6930524	68.3812876	70.731787	60.2395153
17:29:16	0:27:16		73.6930524	68.3812876	71.731787	60.2395153
17:29:17	0:27:17		73.6930524	69.3812876	71.731787	62.2395153
17:29:18	0:27:18		73.6930524	73.3812876	71.731787	64.2395153
17:29:19	0:27:19		74.6930524	73.3812876	71.731787	66.2395153
17:29:20	0:27:20		74.6930524	72.3812876	72.731787	68.2395153
17:29:21	0:27:21		74.6930524	72.3812876	72.731787	69.2395153
17:29:22	0:27:22		73.6930524	71.3812876	73.731787	68.2395153
17:29:23	0:27:23		73.6930524	70.3812876	73.731787	68.2395153
17:29:24	0:27:24		73.6930524	70.3812876	73.731787	68.2395153
17:29:25	0:27:25		73.6930524	71.3812876	72.731787	68.2395153
17:29:26	0:27:26		73.6930524	72.3812876	72.731787	68.2395153
17:29:27	0:27:27		73.6930524	72.3812876	72.731787	71.2395153
17:29:28	0:27:28		73.6930524	72.3812876	72.731787	73.2395153
17:29:29	0:27:29		74.6930524	73.3812876	72.731787	74.2395153
17:29:30	0:27:30		74.6930524	73.3812876	72.731787	75.2395153
17:29:31	0:27:31		74.6930524	73.3812876	72.731787	75.2395153
17:29:32	0:27:32		74.6930524	74.3812876	72.731787	76.2395153
17:29:33	0:27:33		74.6930524	75.3812876	73.731787	76.2395153
17:29:34	0:27:34		74.6930524	77.3812876	73.731787	76.2395153
17:29:35	0:27:35		74.6930524	79.3812876	74.731787	76.2395153
17:29:36	0:27:36		74.6930524	79.3812876	74.731787	76.2395153
17:29:37	0:27:37		74.6930524	78.3812876	74.731787	75.2395153
17:29:38	0:27:38		74.6930524	78.3812876	75.731787	74.2395153
17:29:39	0:27:39		74.6930524	72.3812876	75.731787	74.2395153
17:29:40	0:27:40		74.6930524	70.3812876	76.731787	74.2395153
17:29:41	0:27:41		75.6930524	70.3812876	76.731787	73.2395153
17:29:42	0:27:42		75.6930524	68.3812876	76.731787	73.2395153

17:29:43	0:27:43		77.6930524	69.3812876	77.731787	73.2395153
17:29:44	0:27:44		78.6930524	69.3812876	77.731787	73.2395153
17:29:45	0:27:45		77.6930524	70.3812876	77.731787	73.2395153
17:29:46	0:27:46		77.6930524	70.3812876	77.731787	68.2395153
17:29:47	0:27:47		78.6930524	70.3812876	76.731787	67.2395153
17:29:48	0:27:48		77.6930524	75.3812876	76.731787	66.2395153
17:29:49	0:27:49		77.6930524	76.3812876	76.731787	66.2395153
17:29:50	0:27:50		77.6930524	76.3812876	75.731787	66.2395153
17:29:51	0:27:51		76.6930524	76.3812876	75.731787	65.2395153
17:29:52	0:27:52		75.6930524	76.3812876	77.731787	65.2395153
17:29:53	0:27:53		75.6930524	76.3812876	79.731787	65.2395153
17:29:54	0:27:54		75.6930524	75.3812876	79.731787	65.2395153
17:29:55	0:27:55		75.6930524	77.3812876	79.731787	65.2395153
17:29:56	0:27:56		75.6930524	78.3812876	76.731787	66.2395153
17:29:57	0:27:57		75.6930524	78.3812876	76.731787	66.2395153
17:29:58	0:27:58		75.6930524	78.3812876	73.731787	66.2395153
17:29:59	0:27:59		76.6930524	78.3812876	72.731787	65.2395153
17:30:00	0:28:00		76.6930524	72.3812876	72.731787	65.2395153
17:30:01	0:28:01		76.6930524	72.3812876	72.731787	65.2395153
17:30:02	0:28:02		76.6930524	71.3812876	72.731787	65.2395153
17:30:03	0:28:03		75.6930524	71.3812876	72.731787	65.2395153
17:30:04	0:28:04		75.6930524	71.3812876	72.731787	66.2395153
17:30:05	0:28:05		73.6930524	71.3812876	72.731787	68.2395153
17:30:06	0:28:06		73.6930524	70.3812876	72.731787	68.2395153
17:30:07	0:28:07		73.6930524	69.3812876	72.731787	70.2395153
17:30:08	0:28:08		74.6930524	66.3812876	71.731787	70.2395153
17:30:09	0:28:09		74.6930524	68.3812876	71.731787	67.2395153
17:30:10	0:28:10		75.6930524	69.3812876	71.731787	66.2395153
17:30:11	0:28:11		75.6930524	70.3812876	71.731787	66.2395153
17:30:12	0:28:12		76.6930524	70.3812876	71.731787	67.2395153
17:30:13	0:28:13		76.6930524	70.3812876	72.731787	69.2395153
17:30:14	0:28:14		76.6930524	70.3812876	72.731787	69.2395153
17:30:15	0:28:15		77.6930524	70.3812876	73.731787	69.2395153
17:30:16	0:28:16		76.6930524	71.3812876	73.731787	69.2395153
17:30:17	0:28:17		75.6930524	71.3812876	73.731787	70.2395153
17:30:18	0:28:18		75.6930524	71.3812876	73.731787	70.2395153
17:30:19	0:28:19		73.6930524	72.3812876	73.731787	66.2395153
17:30:20	0:28:20		71.6930524	73.3812876	74.731787	65.2395153
17:30:21	0:28:21		71.6930524	73.3812876	73.731787	65.2395153
17:30:22	0:28:22		70.6930524	73.3812876	72.731787	65.2395153
17:30:23	0:28:23		70.6930524	73.3812876	72.731787	65.2395153
17:30:24	0:28:24		71.6930524	73.3812876	71.731787	65.2395153
17:30:25	0:28:25		71.6930524	73.3812876	72.731787	65.2395153
17:30:26	0:28:26		71.6930524	73.3812876	72.731787	66.2395153
17:30:27	0:28:27		72.6930524	74.3812876	72.731787	67.2395153

17:30:28	0:28:28		72.6930524	74.3812876	72.731787	67.2395153
17:30:29	0:28:29		75.6930524	75.3812876	72.731787	67.2395153
17:30:30	0:28:30		75.6930524	76.3812876	73.731787	67.2395153
17:30:31	0:28:31		75.6930524	76.3812876	73.731787	67.2395153
17:30:32	0:28:32		75.6930524	78.3812876	73.731787	67.2395153
17:30:33	0:28:33		76.6930524	78.3812876	73.731787	69.2395153
17:30:34	0:28:34		77.6930524	78.3812876	73.731787	69.2395153
17:30:35	0:28:35		78.6930524	78.3812876	73.731787	70.2395153
17:30:36	0:28:36		78.6930524	83.3812876	73.731787	70.2395153
17:30:37	0:28:37		78.6930524	84.3812876	73.731787	70.2395153
17:30:38	0:28:38		75.6930524	86.3812876	78.731787	69.2395153
17:30:39	0:28:39		75.6930524	86.3812876	80.731787	68.2395153
17:30:40	0:28:40		75.6930524	86.3812876	82.731787	68.2395153
17:30:41	0:28:41		75.6930524	86.3812876	84.731787	69.2395153
17:30:42	0:28:42		75.6930524	82.3812876	84.731787	68.2395153
17:30:43	0:28:43		75.6930524	82.3812876	83.731787	69.2395153
17:30:44	0:28:44		75.6930524	77.3812876	82.731787	69.2395153
17:30:45	0:28:45		75.6930524	77.3812876	80.731787	69.2395153
17:30:46	0:28:46		75.6930524	77.3812876	78.731787	69.2395153
17:30:47	0:28:47		75.6930524	78.3812876	76.731787	69.2395153
17:30:48	0:28:48		74.6930524	78.3812876	74.731787	69.2395153
17:30:49	0:28:49		73.6930524	78.3812876	73.731787	69.2395153
17:30:50	0:28:50		73.6930524	78.3812876	73.731787	69.2395153
17:30:51	0:28:51		73.6930524	77.3812876	73.731787	68.2395153
17:30:52	0:28:52		73.6930524	77.3812876	74.731787	68.2395153
17:30:53	0:28:53		73.6930524	76.3812876	74.731787	68.2395153
17:30:54	0:28:54		75.6930524	75.3812876	74.731787	67.2395153
17:30:55	0:28:55		75.6930524	74.3812876	75.731787	67.2395153
17:30:56	0:28:56		75.6930524	74.3812876	75.731787	67.2395153
17:30:57	0:28:57		75.6930524	74.3812876	75.731787	67.2395153
17:30:58	0:28:58		75.6930524	72.3812876	75.731787	67.2395153
17:30:59	0:28:59		75.6930524	71.3812876	75.731787	67.2395153
17:31:00	0:29:00		75.6930524	71.3812876	74.731787	67.2395153
17:31:01	0:29:01		75.6930524	71.3812876	74.731787	67.2395153
17:31:02	0:29:02		75.6930524	72.3812876	74.731787	67.2395153
17:31:03	0:29:03		75.6930524	72.3812876	74.731787	67.2395153
17:31:04	0:29:04		75.6930524	72.3812876	74.731787	66.2395153
17:31:05	0:29:05		75.6930524	72.3812876	74.731787	64.2395153
17:31:06	0:29:06		74.6930524	72.3812876	74.731787	64.2395153
17:31:07	0:29:07		73.6930524	71.3812876	73.731787	64.2395153
17:31:08	0:29:08		73.6930524	71.3812876	73.731787	64.2395153
17:31:09	0:29:09		73.6930524	71.3812876	72.731787	65.2395153
17:31:10	0:29:10		74.6930524	71.3812876	72.731787	66.2395153
17:31:11	0:29:11		74.6930524	71.3812876	71.731787	67.2395153
17:31:12	0:29:12		74.6930524	71.3812876	71.731787	67.2395153

17:31:13	0:29:13		73.6930524	71.3812876	71.731787	69.2395153
17:31:14	0:29:14		73.6930524	71.3812876	71.731787	69.2395153
17:31:15	0:29:15		74.6930524	71.3812876	71.731787	68.2395153
17:31:16	0:29:16		75.6930524	71.3812876	72.731787	66.2395153
17:31:17	0:29:17		76.6930524	72.3812876	72.731787	66.2395153
17:31:18	0:29:18		76.6930524	72.3812876	73.731787	67.2395153
17:31:19	0:29:19		76.6930524	72.3812876	73.731787	67.2395153
17:31:20	0:29:20		76.6930524	74.3812876	72.731787	68.2395153
17:31:21	0:29:21		76.6930524	74.3812876	71.731787	71.2395153
17:31:22	0:29:22		76.6930524	75.3812876	71.731787	71.2395153
17:31:23	0:29:23		76.6930524	75.3812876	71.731787	71.2395153
17:31:24	0:29:24		76.6930524	75.3812876	71.731787	71.2395153
17:31:25	0:29:25		76.6930524	75.3812876	71.731787	69.2395153
17:31:26	0:29:26		73.6930524	75.3812876	72.731787	68.2395153
17:31:27	0:29:27		72.6930524	76.3812876	72.731787	68.2395153
17:31:28	0:29:28		72.6930524	76.3812876	73.731787	68.2395153
17:31:29	0:29:29		72.6930524	76.3812876	73.731787	67.2395153
17:31:30	0:29:30		72.6930524	77.3812876	73.731787	67.2395153
17:31:31	0:29:31		72.6930524	77.3812876	73.731787	68.2395153
17:31:32	0:29:32		72.6930524	77.3812876	73.731787	69.2395153
17:31:33	0:29:33		72.6930524	77.3812876	73.731787	69.2395153
17:31:34	0:29:34		72.6930524	78.3812876	73.731787	71.2395153
17:31:35	0:29:35		71.6930524	79.3812876	74.731787	71.2395153
17:31:36	0:29:36		71.6930524	80.3812876	75.731787	70.2395153
17:31:37	0:29:37		72.6930524	80.3812876	78.731787	69.2395153
17:31:38	0:29:38		73.6930524	79.3812876	79.731787	68.2395153
17:31:39	0:29:39		74.6930524	77.3812876	77.731787	69.2395153
17:31:40	0:29:40		74.6930524	76.3812876	76.731787	68.2395153
17:31:41	0:29:41		73.6930524	76.3812876	75.731787	69.2395153
17:31:42	0:29:42		72.6930524	76.3812876	74.731787	69.2395153
17:31:43	0:29:43		73.6930524	77.3812876	74.731787	69.2395153
17:31:44	0:29:44		73.6930524	77.3812876	74.731787	69.2395153
17:31:45	0:29:45		74.6930524	75.3812876	74.731787	70.2395153
17:31:46	0:29:46		74.6930524	75.3812876	75.731787	70.2395153
17:31:47	0:29:47		74.6930524	74.3812876	74.731787	70.2395153
17:31:48	0:29:48		74.6930524	73.3812876	73.731787	67.2395153
17:31:49	0:29:49		74.6930524	73.3812876	72.731787	66.2395153
17:31:50	0:29:50		74.6930524	73.3812876	72.731787	67.2395153
17:31:51	0:29:51		75.6930524	73.3812876	72.731787	69.2395153
17:31:52	0:29:52		75.6930524	73.3812876	73.731787	70.2395153
17:31:53	0:29:53		76.6930524	73.3812876	74.731787	70.2395153
17:31:54	0:29:54		76.6930524	73.3812876	74.731787	71.2395153
17:31:55	0:29:55		76.6930524	73.3812876	74.731787	72.2395153
17:31:56	0:29:56		76.6930524	73.3812876	74.731787	72.2395153
17:31:57	0:29:57		76.6930524	73.3812876	73.731787	69.2395153

17:31:58	0:29:58		76.6930524	73.3812876	73.731787	67.2395153
17:31:59	0:29:59		76.6930524	73.3812876	73.731787	66.2395153
17:32:00	0:30:00		76.6930524	73.3812876	72.731787	65.2395153
17:32:01	0:30:01		75.6930524	73.3812876	72.731787	65.2395153
17:32:02	0:30:02		75.6930524	73.3812876	73.731787	66.2395153
17:32:03	0:30:03		75.6930524	73.3812876	74.731787	66.2395153
17:32:04	0:30:04		75.6930524	73.3812876	75.731787	66.2395153
17:32:05	0:30:05		75.6930524	73.3812876	75.731787	66.2395153
17:32:06	0:30:06		75.6930524	73.3812876	75.731787	66.2395153
17:32:07	0:30:07		75.6930524	73.3812876	76.731787	66.2395153
17:32:08	0:30:08		75.6930524	73.3812876	75.731787	67.2395153
17:32:09	0:30:09		76.6930524	73.3812876	75.731787	67.2395153
17:32:10	0:30:10		76.6930524	78.3812876	74.731787	66.2395153
17:32:11	0:30:11		75.6930524	78.3812876	76.731787	66.2395153
17:32:12	0:30:12		76.6930524	78.3812876	77.731787	66.2395153
17:32:13	0:30:13		76.6930524	77.3812876	78.731787	66.2395153
17:32:14	0:30:14		77.6930524	75.3812876	78.731787	67.2395153
17:32:15	0:30:15		77.6930524	75.3812876	78.731787	67.2395153
17:32:16	0:30:16		77.6930524	75.3812876	78.731787	67.2395153
17:32:17	0:30:17		78.6930524	75.3812876	77.731787	67.2395153
17:32:18	0:30:18		78.6930524	75.3812876	77.731787	67.2395153
17:32:19	0:30:19		79.6930524	75.3812876	76.731787	67.2395153
17:32:20	0:30:20		80.6930524	75.3812876	76.731787	72.2395153
17:32:21	0:30:21		80.6930524	76.3812876	75.731787	73.2395153
17:32:22	0:30:22		81.6930524	76.3812876	75.731787	74.2395153
17:32:23	0:30:23		81.6930524	77.3812876	75.731787	74.2395153
17:32:24	0:30:24		81.6930524	78.3812876	78.731787	74.2395153
17:32:25	0:30:25		81.6930524	78.3812876	79.731787	74.2395153
17:32:26	0:30:26		80.6930524	78.3812876	79.731787	74.2395153
17:32:27	0:30:27		78.6930524	78.3812876	79.731787	76.2395153
17:32:28	0:30:28		77.6930524	78.3812876	79.731787	76.2395153
17:32:29	0:30:29		77.6930524	78.3812876	79.731787	76.2395153
17:32:30	0:30:30		76.6930524	78.3812876	79.731787	76.2395153
17:32:31	0:30:31		76.6930524	81.3812876	79.731787	76.2395153
17:32:32	0:30:32		76.6930524	81.3812876	79.731787	76.2395153
17:32:33	0:30:33		77.6930524	80.3812876	78.731787	76.2395153
17:32:34	0:30:34		77.6930524	80.3812876	78.731787	75.2395153
17:32:35	0:30:35		77.6930524	80.3812876	78.731787	76.2395153
17:32:36	0:30:36		77.6930524	80.3812876	77.731787	75.2395153
17:32:37	0:30:37		78.6930524	80.3812876	77.731787	74.2395153
17:32:38	0:30:38		78.6930524	73.3812876	77.731787	74.2395153
17:32:39	0:30:39		78.6930524	71.3812876	77.731787	74.2395153
17:32:40	0:30:40		78.6930524	71.3812876	77.731787	73.2395153
17:32:41	0:30:41		78.6930524	71.3812876	78.731787	73.2395153
17:32:42	0:30:42		78.6930524	70.3812876	78.731787	73.2395153

17:32:43	0:30:43		78.6930524	70.3812876	79.731787	73.2395153
17:32:44	0:30:44		77.6930524	70.3812876	79.731787	73.2395153
17:32:45	0:30:45		77.6930524	70.3812876	78.731787	73.2395153
17:32:46	0:30:46		80.6930524	70.3812876	78.731787	72.2395153
17:32:47	0:30:47		83.6930524	70.3812876	75.731787	73.2395153
17:32:48	0:30:48		84.6930524	69.3812876	74.731787	72.2395153
17:32:49	0:30:49		85.6930524	69.3812876	73.731787	72.2395153
17:32:50	0:30:50		88.6930524	69.3812876	73.731787	72.2395153
17:32:51	0:30:51		89.6930524	69.3812876	73.731787	72.2395153
17:32:52	0:30:52		89.6930524	70.3812876	73.731787	72.2395153
17:32:53	0:30:53		85.6930524	70.3812876	73.731787	71.2395153
17:32:54	0:30:54		85.6930524	70.3812876	73.731787	70.2395153
17:32:55	0:30:55		81.6930524	70.3812876	73.731787	69.2395153
17:32:56	0:30:56		80.6930524	72.3812876	73.731787	68.2395153
17:32:57	0:30:57		79.6930524	77.3812876	73.731787	69.2395153
17:32:58	0:30:58		78.6930524	79.3812876	73.731787	70.2395153
17:32:59	0:30:59		78.6930524	79.3812876	73.731787	71.2395153
17:33:00	0:31:00		78.6930524	79.3812876	73.731787	71.2395153
17:33:01	0:31:01		78.6930524	76.3812876	73.731787	73.2395153
17:33:02	0:31:02		78.6930524	76.3812876	74.731787	75.2395153
17:33:03	0:31:03		79.6930524	76.3812876	76.731787	76.2395153
17:33:04	0:31:04		79.6930524	76.3812876	77.731787	76.2395153
17:33:05	0:31:05		78.6930524	76.3812876	77.731787	75.2395153
17:33:06	0:31:06		78.6930524	75.3812876	75.731787	74.2395153
17:33:07	0:31:07		77.6930524	75.3812876	75.731787	73.2395153
17:33:08	0:31:08		77.6930524	75.3812876	75.731787	74.2395153
17:33:09	0:31:09		75.6930524	75.3812876	75.731787	74.2395153
17:33:10	0:31:10		75.6930524	75.3812876	74.731787	76.2395153
17:33:11	0:31:11		75.6930524	73.3812876	74.731787	76.2395153
17:33:12	0:31:12		74.6930524	68.3812876	73.731787	76.2395153
17:33:13	0:31:13		75.6930524	68.3812876	73.731787	76.2395153
17:33:14	0:31:14		75.6930524	67.3812876	73.731787	76.2395153
17:33:15	0:31:15		75.6930524	68.3812876	73.731787	74.2395153
17:33:16	0:31:16		74.6930524	73.3812876	73.731787	74.2395153
17:33:17	0:31:17		74.6930524	72.3812876	73.731787	75.2395153
17:33:18	0:31:18		74.6930524	72.3812876	74.731787	75.2395153
17:33:19	0:31:19		74.6930524	72.3812876	75.731787	75.2395153
17:33:20	0:31:20		74.6930524	72.3812876	76.731787	76.2395153
17:33:21	0:31:21		74.6930524	72.3812876	76.731787	77.2395153
17:33:22	0:31:22		74.6930524	71.3812876	76.731787	76.2395153
17:33:23	0:31:23		73.6930524	70.3812876	75.731787	75.2395153
17:33:24	0:31:24		73.6930524	70.3812876	74.731787	75.2395153
17:33:25	0:31:25		73.6930524	70.3812876	73.731787	77.2395153
17:33:26	0:31:26		73.6930524	69.3812876	72.731787	78.2395153
17:33:27	0:31:27		73.6930524	69.3812876	72.731787	78.2395153

17:33:28	0:31:28		73.6930524	69.3812876	72.731787	78.2395153
17:33:29	0:31:29		73.6930524	69.3812876	72.731787	78.2395153
17:33:30	0:31:30		73.6930524	69.3812876	72.731787	78.2395153
17:33:31	0:31:31		74.6930524	71.3812876	72.731787	78.2395153
17:33:32	0:31:32		75.6930524	72.3812876	73.731787	79.2395153
17:33:33	0:31:33		75.6930524	72.3812876	74.731787	78.2395153
17:33:34	0:31:34		75.6930524	78.3812876	74.731787	78.2395153
17:33:35	0:31:35		75.6930524	80.3812876	74.731787	77.2395153
17:33:36	0:31:36		75.6930524	81.3812876	74.731787	76.2395153
17:33:37	0:31:37		77.6930524	81.3812876	74.731787	74.2395153
17:33:38	0:31:38		77.6930524	81.3812876	73.731787	74.2395153
17:33:39	0:31:39		76.6930524	82.3812876	73.731787	74.2395153
17:33:40	0:31:40		76.6930524	82.3812876	73.731787	70.2395153
17:33:41	0:31:41		76.6930524	85.3812876	73.731787	70.2395153
17:33:42	0:31:42		77.6930524	85.3812876	73.731787	70.2395153
17:33:43	0:31:43		78.6930524	85.3812876	72.731787	72.2395153
17:33:44	0:31:44		79.6930524	85.3812876	69.731787	74.2395153
17:33:45	0:31:45		79.6930524	85.3812876	67.731787	76.2395153
17:33:46	0:31:46		78.6930524	85.3812876	66.731787	76.2395153
17:33:47	0:31:47		77.6930524	85.3812876	66.731787	75.2395153
17:33:48	0:31:48		77.6930524	85.3812876	66.731787	75.2395153
17:33:49	0:31:49		75.6930524	83.3812876	67.731787	74.2395153
17:33:50	0:31:50		75.6930524	73.3812876	67.731787	74.2395153
17:33:51	0:31:51		74.6930524	69.3812876	67.731787	76.2395153
17:33:52	0:31:52		74.6930524	69.3812876	70.731787	76.2395153
17:33:53	0:31:53		75.6930524	69.3812876	72.731787	77.2395153
17:33:54	0:31:54		75.6930524	69.3812876	72.731787	77.2395153
17:33:55	0:31:55		76.6930524	69.3812876	74.731787	78.2395153
17:33:56	0:31:56		78.6930524	69.3812876	74.731787	81.2395153
17:33:57	0:31:57		79.6930524	69.3812876	73.731787	81.2395153
17:33:58	0:31:58		79.6930524	69.3812876	73.731787	80.2395153
17:33:59	0:31:59		78.6930524	70.3812876	73.731787	79.2395153
17:34:00	0:32:00		78.6930524	71.3812876	72.731787	78.2395153
17:34:01	0:32:01		77.6930524	71.3812876	72.731787	78.2395153
17:34:02	0:32:02		77.6930524	77.3812876	71.731787	76.2395153
17:34:03	0:32:03		77.6930524	77.3812876	71.731787	77.2395153
17:34:04	0:32:04		77.6930524	77.3812876	72.731787	79.2395153
17:34:05	0:32:05		77.6930524	77.3812876	75.731787	80.2395153
17:34:06	0:32:06		77.6930524	81.3812876	75.731787	80.2395153
17:34:07	0:32:07		77.6930524	81.3812876	75.731787	80.2395153
17:34:08	0:32:08		76.6930524	82.3812876	74.731787	80.2395153
17:34:09	0:32:09		75.6930524	82.3812876	75.731787	79.2395153
17:34:10	0:32:10		75.6930524	82.3812876	77.731787	79.2395153
17:34:11	0:32:11		75.6930524	82.3812876	77.731787	76.2395153
17:34:12	0:32:12		75.6930524	82.3812876	76.731787	74.2395153

17:34:13	0:32:13		74.6930524	82.3812876	75.731787	74.2395153
17:34:14	0:32:14		74.6930524	81.3812876	74.731787	74.2395153
17:34:15	0:32:15		74.6930524	81.3812876	74.731787	74.2395153
17:34:16	0:32:16		74.6930524	79.3812876	74.731787	74.2395153
17:34:17	0:32:17		74.6930524	79.3812876	75.731787	74.2395153
17:34:18	0:32:18		74.6930524	79.3812876	76.731787	73.2395153
17:34:19	0:32:19		74.6930524	79.3812876	77.731787	73.2395153
17:34:20	0:32:20		74.6930524	76.3812876	77.731787	74.2395153
17:34:21	0:32:21		74.6930524	75.3812876	77.731787	75.2395153
17:34:22	0:32:22		78.6930524	75.3812876	78.731787	78.2395153
17:34:23	0:32:23		82.6930524	75.3812876	79.731787	79.2395153
17:34:24	0:32:24		85.6930524	72.3812876	79.731787	79.2395153
17:34:25	0:32:25		86.6930524	71.3812876	80.731787	80.2395153
17:34:26	0:32:26		83.6930524	70.3812876	81.731787	82.2395153
17:34:27	0:32:27		82.6930524	70.3812876	81.731787	82.2395153
17:34:28	0:32:28		82.6930524	69.3812876	79.731787	83.2395153
17:34:29	0:32:29		81.6930524	69.3812876	77.731787	83.2395153
17:34:30	0:32:30		81.6930524	69.3812876	76.731787	83.2395153
17:34:31	0:32:31		80.6930524	69.3812876	75.731787	81.2395153
17:34:32	0:32:32		80.6930524	71.3812876	75.731787	81.2395153
17:34:33	0:32:33		80.6930524	71.3812876	75.731787	81.2395153
17:34:34	0:32:34		81.6930524	71.3812876	75.731787	75.2395153
17:34:35	0:32:35		81.6930524	71.3812876	75.731787	69.2395153
17:34:36	0:32:36		82.6930524	72.3812876	75.731787	68.2395153
17:34:37	0:32:37		82.6930524	72.3812876	74.731787	66.2395153
17:34:38	0:32:38		83.6930524	74.3812876	74.731787	62.2395153
17:34:39	0:32:39		82.6930524	74.3812876	73.731787	61.2395153
17:34:40	0:32:40		81.6930524	74.3812876	73.731787	61.2395153
17:34:41	0:32:41		80.6930524	74.3812876	72.731787	60.2395153
17:34:42	0:32:42		79.6930524	74.3812876	71.731787	60.2395153
17:34:43	0:32:43		79.6930524	74.3812876	70.731787	60.2395153
17:34:44	0:32:44		75.6930524	74.3812876	70.731787	61.2395153
17:34:45	0:32:45		73.6930524	75.3812876	70.731787	61.2395153
17:34:46	0:32:46		71.6930524	75.3812876	69.731787	62.2395153
17:34:47	0:32:47		72.6930524	76.3812876	68.731787	62.2395153
17:34:48	0:32:48		72.6930524	76.3812876	68.731787	62.2395153
17:34:49	0:32:49		74.6930524	76.3812876	67.731787	63.2395153
17:34:50	0:32:50		74.6930524	76.3812876	67.731787	63.2395153
17:34:51	0:32:51		74.6930524	75.3812876	67.731787	64.2395153
17:34:52	0:32:52		74.6930524	75.3812876	68.731787	64.2395153
17:34:53	0:32:53		75.6930524	75.3812876	68.731787	64.2395153
17:34:54	0:32:54		75.6930524	75.3812876	70.731787	65.2395153
17:34:55	0:32:55		75.6930524	76.3812876	70.731787	65.2395153
17:34:56	0:32:56		75.6930524	76.3812876	70.731787	65.2395153
17:34:57	0:32:57		75.6930524	76.3812876	70.731787	65.2395153

17:34:58	0:32:58		75.6930524	76.3812876	70.731787	65.2395153
17:34:59	0:32:59		75.6930524	76.3812876	70.731787	66.2395153
17:35:00	0:33:00		75.6930524	76.3812876	70.731787	66.2395153
17:35:01	0:33:01		75.6930524	75.3812876	70.731787	64.2395153
17:35:02	0:33:02		75.6930524	75.3812876	70.731787	63.2395153
17:35:03	0:33:03		75.6930524	75.3812876	71.731787	63.2395153
17:35:04	0:33:04		75.6930524	76.3812876	72.731787	65.2395153
17:35:05	0:33:05		75.6930524	76.3812876	72.731787	68.2395153
17:35:06	0:33:06		75.6930524	76.3812876	72.731787	70.2395153
17:35:07	0:33:07		75.6930524	77.3812876	72.731787	70.2395153
17:35:08	0:33:08		75.6930524	77.3812876	72.731787	68.2395153
17:35:09	0:33:09		75.6930524	78.3812876	72.731787	68.2395153
17:35:10	0:33:10		75.6930524	80.3812876	71.731787	68.2395153
17:35:11	0:33:11		74.6930524	80.3812876	71.731787	68.2395153
17:35:12	0:33:12		74.6930524	80.3812876	71.731787	67.2395153
17:35:13	0:33:13		74.6930524	80.3812876	71.731787	67.2395153
17:35:14	0:33:14		74.6930524	80.3812876	71.731787	67.2395153
17:35:15	0:33:15		75.6930524	80.3812876	71.731787	64.2395153
17:35:16	0:33:16		75.6930524	82.3812876	72.731787	64.2395153
17:35:17	0:33:17		75.6930524	82.3812876	73.731787	64.2395153
17:35:18	0:33:18		77.6930524	82.3812876	73.731787	64.2395153
17:35:19	0:33:19		77.6930524	83.3812876	73.731787	64.2395153
17:35:20	0:33:20		77.6930524	83.3812876	73.731787	64.2395153
17:35:21	0:33:21		77.6930524	83.3812876	73.731787	64.2395153
17:35:22	0:33:22		77.6930524	84.3812876	73.731787	64.2395153
17:35:23	0:33:23		77.6930524	84.3812876	73.731787	64.2395153
17:35:24	0:33:24		78.6930524	84.3812876	73.731787	63.2395153
17:35:25	0:33:25		79.6930524	84.3812876	72.731787	63.2395153
17:35:26	0:33:26		83.6930524	88.3812876	73.731787	66.2395153
17:35:27	0:33:27		86.6930524	90.3812876	73.731787	68.2395153
17:35:28	0:33:28		88.6930524	91.3812876	78.731787	70.2395153
17:35:29	0:33:29		90.6930524	92.3812876	78.731787	70.2395153
17:35:30	0:33:30		90.6930524	92.3812876	80.731787	70.2395153
17:35:31	0:33:31		89.6930524	92.3812876	80.731787	70.2395153
17:35:32	0:33:32		85.6930524	90.3812876	80.731787	70.2395153
17:35:33	0:33:33		80.6930524	87.3812876	80.731787	69.2395153
17:35:34	0:33:34		79.6930524	87.3812876	79.731787	71.2395153
17:35:35	0:33:35		78.6930524	87.3812876	79.731787	71.2395153
17:35:36	0:33:36		78.6930524	87.3812876	79.731787	73.2395153
17:35:37	0:33:37		78.6930524	87.3812876	79.731787	73.2395153
17:35:38	0:33:38		78.6930524	86.3812876	79.731787	71.2395153
17:35:39	0:33:39		78.6930524	85.3812876	79.731787	71.2395153
17:35:40	0:33:40		79.6930524	85.3812876	78.731787	70.2395153
17:35:41	0:33:41		80.6930524	85.3812876	75.731787	69.2395153
17:35:42	0:33:42		80.6930524	85.3812876	75.731787	69.2395153

17:35:43	0:33:43		80.6930524	81.3812876	74.731787	70.2395153
17:35:44	0:33:44		80.6930524	81.3812876	74.731787	69.2395153
17:35:45	0:33:45		80.6930524	81.3812876	73.731787	69.2395153
17:35:46	0:33:46		80.6930524	81.3812876	73.731787	69.2395153
17:35:47	0:33:47		80.6930524	81.3812876	73.731787	68.2395153
17:35:48	0:33:48		79.6930524	81.3812876	73.731787	68.2395153
17:35:49	0:33:49		79.6930524	81.3812876	72.731787	68.2395153
17:35:50	0:33:50		74.6930524	81.3812876	72.731787	68.2395153
17:35:51	0:33:51		73.6930524	80.3812876	72.731787	68.2395153
17:35:52	0:33:52		73.6930524	79.3812876	73.731787	68.2395153
17:35:53	0:33:53		71.6930524	79.3812876	73.731787	67.2395153
17:35:54	0:33:54		71.6930524	80.3812876	73.731787	67.2395153
17:35:55	0:33:55		71.6930524	80.3812876	73.731787	66.2395153
17:35:56	0:33:56		71.6930524	79.3812876	73.731787	65.2395153
17:35:57	0:33:57		71.6930524	78.3812876	73.731787	65.2395153
17:35:58	0:33:58		73.6930524	78.3812876	73.731787	65.2395153
17:35:59	0:33:59		75.6930524	78.3812876	73.731787	65.2395153
17:36:00	0:34:00		75.6930524	77.3812876	73.731787	65.2395153
17:36:01	0:34:01		75.6930524	76.3812876	73.731787	65.2395153
17:36:02	0:34:02		75.6930524	75.3812876	72.731787	67.2395153
17:36:03	0:34:03		75.6930524	75.3812876	72.731787	67.2395153
17:36:04	0:34:04		75.6930524	76.3812876	72.731787	68.2395153
17:36:05	0:34:05		75.6930524	76.3812876	74.731787	68.2395153
17:36:06	0:34:06		75.6930524	76.3812876	74.731787	66.2395153
17:36:07	0:34:07		75.6930524	76.3812876	74.731787	66.2395153
17:36:08	0:34:08		76.6930524	76.3812876	73.731787	66.2395153
17:36:09	0:34:09		77.6930524	76.3812876	73.731787	66.2395153
17:36:10	0:34:10		79.6930524	76.3812876	73.731787	66.2395153
17:36:11	0:34:11		79.6930524	76.3812876	73.731787	65.2395153
17:36:12	0:34:12		79.6930524	74.3812876	73.731787	64.2395153
17:36:13	0:34:13		80.6930524	73.3812876	75.731787	65.2395153
17:36:14	0:34:14		80.6930524	73.3812876	75.731787	66.2395153
17:36:15	0:34:15		80.6930524	73.3812876	76.731787	65.2395153
17:36:16	0:34:16		80.6930524	73.3812876	76.731787	66.2395153
17:36:17	0:34:17		81.6930524	73.3812876	76.731787	66.2395153
17:36:18	0:34:18		82.6930524	73.3812876	76.731787	66.2395153
17:36:19	0:34:19		84.6930524	73.3812876	76.731787	66.2395153
17:36:20	0:34:20		84.6930524	73.3812876	76.731787	66.2395153
17:36:21	0:34:21		84.6930524	73.3812876	75.731787	66.2395153
17:36:22	0:34:22		85.6930524	75.3812876	75.731787	66.2395153
17:36:23	0:34:23		85.6930524	75.3812876	75.731787	68.2395153
17:36:24	0:34:24		85.6930524	78.3812876	75.731787	67.2395153
17:36:25	0:34:25		85.6930524	80.3812876	75.731787	67.2395153
17:36:26	0:34:26		85.6930524	80.3812876	75.731787	67.2395153
17:36:27	0:34:27		84.6930524	80.3812876	76.731787	67.2395153

17:36:28	0:34:28		84.6930524	80.3812876	75.731787	67.2395153
17:36:29	0:34:29		84.6930524	80.3812876	75.731787	66.2395153
17:36:30	0:34:30		83.6930524	82.3812876	74.731787	66.2395153
17:36:31	0:34:31		82.6930524	83.3812876	74.731787	66.2395153
17:36:32	0:34:32		81.6930524	83.3812876	74.731787	66.2395153
17:36:33	0:34:33		81.6930524	84.3812876	74.731787	66.2395153
17:36:34	0:34:34		80.6930524	77.3812876	74.731787	66.2395153
17:36:35	0:34:35		80.6930524	77.3812876	74.731787	66.2395153
17:36:36	0:34:36		80.6930524	77.3812876	74.731787	66.2395153
17:36:37	0:34:37		80.6930524	74.3812876	74.731787	67.2395153
17:36:38	0:34:38		80.6930524	73.3812876	74.731787	67.2395153
17:36:39	0:34:39		79.6930524	73.3812876	75.731787	67.2395153
17:36:40	0:34:40		79.6930524	73.3812876	75.731787	69.2395153
17:36:41	0:34:41		79.6930524	73.3812876	75.731787	69.2395153
17:36:42	0:34:42		80.6930524	72.3812876	76.731787	69.2395153
17:36:43	0:34:43		80.6930524	71.3812876	76.731787	69.2395153
17:36:44	0:34:44		80.6930524	70.3812876	76.731787	68.2395153
17:36:45	0:34:45		80.6930524	67.3812876	78.731787	67.2395153
17:36:46	0:34:46		80.6930524	67.3812876	84.731787	66.2395153
17:36:47	0:34:47		79.6930524	65.3812876	84.731787	65.2395153
17:36:48	0:34:48		79.6930524	65.3812876	81.731787	65.2395153
17:36:49	0:34:49		79.6930524	65.3812876	80.731787	65.2395153
17:36:50	0:34:50		79.6930524	65.3812876	79.731787	66.2395153
17:36:51	0:34:51		79.6930524	65.3812876	78.731787	66.2395153
17:36:52	0:34:52		79.6930524	65.3812876	78.731787	66.2395153
17:36:53	0:34:53		79.6930524	65.3812876	78.731787	66.2395153
17:36:54	0:34:54		79.6930524	66.3812876	78.731787	67.2395153
17:36:55	0:34:55		79.6930524	66.3812876	78.731787	71.2395153
17:36:56	0:34:56		78.6930524	68.3812876	78.731787	72.2395153
17:36:57	0:34:57		77.6930524	68.3812876	77.731787	73.2395153
17:36:58	0:34:58		76.6930524	69.3812876	77.731787	73.2395153
17:36:59	0:34:59		76.6930524	70.3812876	75.731787	71.2395153
17:37:00	0:35:00		76.6930524	71.3812876	76.731787	70.2395153
17:37:01	0:35:01		78.6930524	73.3812876	76.731787	70.2395153
17:37:02	0:35:02		80.6930524	73.3812876	76.731787	70.2395153
17:37:03	0:35:03		80.6930524	73.3812876	77.731787	70.2395153
17:37:04	0:35:04		80.6930524	73.3812876	78.731787	70.2395153
17:37:05	0:35:05		80.6930524	73.3812876	77.731787	72.2395153
17:37:06	0:35:06		80.6930524	73.3812876	76.731787	72.2395153
17:37:07	0:35:07		79.6930524	74.3812876	75.731787	72.2395153
17:37:08	0:35:08		78.6930524	74.3812876	73.731787	73.2395153
17:37:09	0:35:09		78.6930524	74.3812876	72.731787	73.2395153
17:37:10	0:35:10		77.6930524	74.3812876	72.731787	73.2395153
17:37:11	0:35:11		76.6930524	75.3812876	72.731787	73.2395153
17:37:12	0:35:12		75.6930524	75.3812876	72.731787	72.2395153

17:37:13	0:35:13		76.6930524	75.3812876	73.731787	72.2395153
17:37:14	0:35:14		76.6930524	75.3812876	74.731787	72.2395153
17:37:15	0:35:15		76.6930524	75.3812876	74.731787	72.2395153
17:37:16	0:35:16		75.6930524	74.3812876	74.731787	72.2395153
17:37:17	0:35:17		75.6930524	74.3812876	74.731787	72.2395153
17:37:18	0:35:18		75.6930524	77.3812876	74.731787	72.2395153
17:37:19	0:35:19		75.6930524	77.3812876	75.731787	72.2395153
17:37:20	0:35:20		75.6930524	77.3812876	75.731787	72.2395153
17:37:21	0:35:21		75.6930524	76.3812876	73.731787	73.2395153
17:37:22	0:35:22		76.6930524	75.3812876	73.731787	73.2395153
17:37:23	0:35:23		76.6930524	75.3812876	73.731787	72.2395153
17:37:24	0:35:24		76.6930524	75.3812876	73.731787	73.2395153
17:37:25	0:35:25		76.6930524	73.3812876	73.731787	73.2395153
17:37:26	0:35:26		76.6930524	73.3812876	73.731787	72.2395153
17:37:27	0:35:27		76.6930524	72.3812876	73.731787	72.2395153
17:37:28	0:35:28		76.6930524	71.3812876	73.731787	72.2395153
17:37:29	0:35:29		76.6930524	71.3812876	73.731787	72.2395153
17:37:30	0:35:30		76.6930524	71.3812876	73.731787	72.2395153
17:37:31	0:35:31		77.6930524	71.3812876	71.731787	70.2395153
17:37:32	0:35:32		77.6930524	72.3812876	71.731787	69.2395153
17:37:33	0:35:33		77.6930524	72.3812876	72.731787	69.2395153
17:37:34	0:35:34		78.6930524	72.3812876	72.731787	69.2395153
17:37:35	0:35:35		78.6930524	72.3812876	72.731787	69.2395153
17:37:36	0:35:36		77.6930524	72.3812876	73.731787	69.2395153
17:37:37	0:35:37		77.6930524	74.3812876	73.731787	69.2395153
17:37:38	0:35:38		77.6930524	75.3812876	75.731787	69.2395153
17:37:39	0:35:39		77.6930524	75.3812876	76.731787	69.2395153
17:37:40	0:35:40		76.6930524	77.3812876	77.731787	69.2395153
17:37:41	0:35:41		76.6930524	77.3812876	78.731787	73.2395153
17:37:42	0:35:42		76.6930524	77.3812876	78.731787	80.2395153
17:37:43	0:35:43		76.6930524	77.3812876	78.731787	83.2395153
17:37:44	0:35:44		77.6930524	77.3812876	77.731787	82.2395153
17:37:45	0:35:45		76.6930524	78.3812876	77.731787	82.2395153
17:37:46	0:35:46		76.6930524	78.3812876	76.731787	82.2395153
17:37:47	0:35:47		76.6930524	78.3812876	76.731787	82.2395153
17:37:48	0:35:48		75.6930524	73.3812876	76.731787	81.2395153
17:37:49	0:35:49		75.6930524	73.3812876	77.731787	81.2395153
17:37:50	0:35:50		75.6930524	72.3812876	78.731787	81.2395153
17:37:51	0:35:51		75.6930524	72.3812876	78.731787	81.2395153
17:37:52	0:35:52		75.6930524	72.3812876	78.731787	82.2395153
17:37:53	0:35:53		76.6930524	72.3812876	77.731787	82.2395153
17:37:54	0:35:54		76.6930524	72.3812876	77.731787	82.2395153
17:37:55	0:35:55		76.6930524	71.3812876	76.731787	82.2395153
17:37:56	0:35:56		77.6930524	71.3812876	75.731787	82.2395153
17:37:57	0:35:57		78.6930524	70.3812876	74.731787	83.2395153

17:37:58	0:35:58		78.6930524	70.3812876	75.731787	84.2395153
17:37:59	0:35:59		78.6930524	70.3812876	76.731787	84.2395153
17:38:00	0:36:00		78.6930524	70.3812876	77.731787	84.2395153
17:38:01	0:36:01		78.6930524	71.3812876	77.731787	83.2395153
17:38:02	0:36:02		78.6930524	71.3812876	78.731787	82.2395153
17:38:03	0:36:03		78.6930524	73.3812876	77.731787	82.2395153
17:38:04	0:36:04		78.6930524	73.3812876	76.731787	77.2395153
17:38:05	0:36:05		77.6930524	73.3812876	75.731787	75.2395153
17:38:06	0:36:06		77.6930524	72.3812876	75.731787	74.2395153
17:38:07	0:36:07		77.6930524	72.3812876	75.731787	73.2395153
17:38:08	0:36:08		77.6930524	73.3812876	75.731787	72.2395153
17:38:09	0:36:09		77.6930524	74.3812876	75.731787	72.2395153
17:38:10	0:36:10		77.6930524	73.3812876	75.731787	72.2395153
17:38:11	0:36:11		77.6930524	73.3812876	75.731787	72.2395153
17:38:12	0:36:12		77.6930524	73.3812876	75.731787	74.2395153
17:38:13	0:36:13		77.6930524	73.3812876	77.731787	76.2395153
17:38:14	0:36:14		78.6930524	73.3812876	79.731787	76.2395153
17:38:15	0:36:15		77.6930524	71.3812876	80.731787	76.2395153
17:38:16	0:36:16		76.6930524	71.3812876	80.731787	73.2395153
17:38:17	0:36:17		75.6930524	71.3812876	79.731787	73.2395153
17:38:18	0:36:18		75.6930524	72.3812876	78.731787	73.2395153
17:38:19	0:36:19		75.6930524	70.3812876	77.731787	73.2395153
17:38:20	0:36:20		76.6930524	70.3812876	77.731787	73.2395153
17:38:21	0:36:21		78.6930524	68.3812876	76.731787	73.2395153
17:38:22	0:36:22		79.6930524	67.3812876	76.731787	73.2395153
17:38:23	0:36:23		79.6930524	67.3812876	76.731787	73.2395153
17:38:24	0:36:24		79.6930524	67.3812876	76.731787	73.2395153
17:38:25	0:36:25		80.6930524	67.3812876	77.731787	81.2395153
17:38:26	0:36:26		80.6930524	67.3812876	76.731787	74.2395153
17:38:27	0:36:27		82.6930524	70.3812876	75.731787	73.2395153
17:38:28	0:36:28		82.6930524	71.3812876	75.731787	73.2395153
17:38:29	0:36:29		83.6930524	71.3812876	75.731787	71.2395153
17:38:30	0:36:30		85.6930524	71.3812876	74.731787	72.2395153
17:38:31	0:36:31		86.6930524	71.3812876	74.731787	72.2395153
17:38:32	0:36:32		86.6930524	72.3812876	74.731787	71.2395153
17:38:33	0:36:33		86.6930524	72.3812876	74.731787	71.2395153
17:38:34	0:36:34		90.6930524	73.3812876	73.731787	71.2395153
17:38:35	0:36:35		90.6930524	73.3812876	73.731787	70.2395153
17:38:36	0:36:36		90.6930524	73.3812876	73.731787	70.2395153
17:38:37	0:36:37		91.6930524	73.3812876	73.731787	70.2395153
17:38:38	0:36:38		91.6930524	74.3812876	75.731787	70.2395153
17:38:39	0:36:39		91.6930524	74.3812876	76.731787	70.2395153
17:38:40	0:36:40		90.6930524	74.3812876	76.731787	70.2395153
17:38:41	0:36:41		91.6930524	74.3812876	76.731787	70.2395153
17:38:42	0:36:42		78.6930524	74.3812876	76.731787	71.2395153

17:38:43	0:36:43		77.6930524	74.3812876	76.731787	72.2395153
17:38:44	0:36:44		77.6930524	74.3812876	76.731787	72.2395153
17:38:45	0:36:45		77.6930524	74.3812876	76.731787	73.2395153
17:38:46	0:36:46		77.6930524	75.3812876	76.731787	76.2395153
17:38:47	0:36:47		77.6930524	75.3812876	78.731787	76.2395153
17:38:48	0:36:48		78.6930524	74.3812876	79.731787	78.2395153
17:38:49	0:36:49		79.6930524	74.3812876	79.731787	78.2395153
17:38:50	0:36:50		79.6930524	74.3812876	78.731787	78.2395153
17:38:51	0:36:51		79.6930524	74.3812876	77.731787	79.2395153
17:38:52	0:36:52		79.6930524	74.3812876	77.731787	81.2395153
17:38:53	0:36:53		79.6930524	74.3812876	77.731787	81.2395153
17:38:54	0:36:54		79.6930524	75.3812876	77.731787	81.2395153
17:38:55	0:36:55		78.6930524	75.3812876	77.731787	81.2395153
17:38:56	0:36:56		78.6930524	75.3812876	76.731787	80.2395153
17:38:57	0:36:57		78.6930524	74.3812876	76.731787	80.2395153
17:38:58	0:36:58		78.6930524	74.3812876	75.731787	80.2395153
17:38:59	0:36:59		77.6930524	74.3812876	76.731787	80.2395153
17:39:00	0:37:00		76.6930524	73.3812876	77.731787	81.2395153
17:39:01	0:37:01		76.6930524	71.3812876	78.731787	82.2395153
17:39:02	0:37:02		75.6930524	71.3812876	79.731787	82.2395153
17:39:03	0:37:03		73.6930524	72.3812876	79.731787	82.2395153
17:39:04	0:37:04		72.6930524	72.3812876	79.731787	82.2395153
17:39:05	0:37:05		72.6930524	72.3812876	79.731787	82.2395153
17:39:06	0:37:06		77.6930524	73.3812876	79.731787	84.2395153
17:39:07	0:37:07		79.6930524	73.3812876	78.731787	86.2395153
17:39:08	0:37:08		80.6930524	73.3812876	78.731787	90.2395153
17:39:09	0:37:09		80.6930524	73.3812876	78.731787	90.2395153
17:39:10	0:37:10		79.6930524	74.3812876	78.731787	91.2395153
17:39:11	0:37:11		77.6930524	74.3812876	82.731787	92.2395153
17:39:12	0:37:12		76.6930524	74.3812876	85.731787	95.2395153
17:39:13	0:37:13		76.6930524	74.3812876	87.731787	97.2395153
17:39:14	0:37:14		75.6930524	76.3812876	88.731787	98.2395153
17:39:15	0:37:15		74.6930524	76.3812876	88.731787	99.2395153
17:39:16	0:37:16		74.6930524	76.3812876	88.731787	103.239515
17:39:17	0:37:17		74.6930524	76.3812876	86.731787	104.239515
17:39:18	0:37:18		74.6930524	76.3812876	86.731787	104.239515
17:39:19	0:37:19		73.6930524	78.3812876	84.731787	104.239515
17:39:20	0:37:20		74.6930524	78.3812876	82.731787	104.239515
17:39:21	0:37:21		75.6930524	79.3812876	80.731787	105.239515
17:39:22	0:37:22		75.6930524	81.3812876	78.731787	107.239515
17:39:23	0:37:23		75.6930524	81.3812876	77.731787	110.239515
17:39:24	0:37:24		76.6930524	84.3812876	76.731787	110.239515
17:39:25	0:37:25		76.6930524	84.3812876	76.731787	111.239515
17:39:26	0:37:26		75.6930524	84.3812876	77.731787	111.239515
17:39:27	0:37:27		75.6930524	85.3812876	78.731787	111.239515

17:39:28	0:37:28		74.6930524	85.3812876	80.731787	111.239515
17:39:29	0:37:29		74.6930524	85.3812876	82.731787	111.239515
17:39:30	0:37:30		74.6930524	85.3812876	84.731787	111.239515
17:39:31	0:37:31		75.6930524	85.3812876	84.731787	111.239515
17:39:32	0:37:32		75.6930524	85.3812876	83.731787	110.239515
17:39:33	0:37:33		75.6930524	86.3812876	83.731787	109.239515
17:39:34	0:37:34		76.6930524	86.3812876	82.731787	109.239515
17:39:35	0:37:35		75.6930524	88.3812876	81.731787	109.239515
17:39:36	0:37:36		75.6930524	88.3812876	81.731787	109.239515
17:39:37	0:37:37		75.6930524	88.3812876	80.731787	108.239515
17:39:38	0:37:38		75.6930524	88.3812876	80.731787	109.239515
17:39:39	0:37:39		75.6930524	88.3812876	80.731787	111.239515
17:39:40	0:37:40		75.6930524	88.3812876	80.731787	112.239515
17:39:41	0:37:41		75.6930524	88.3812876	80.731787	112.239515
17:39:42	0:37:42		75.6930524	87.3812876	80.731787	112.239515
17:39:43	0:37:43		75.6930524	87.3812876	79.731787	111.239515
17:39:44	0:37:44		76.6930524	87.3812876	76.731787	109.239515
17:39:45	0:37:45		75.6930524	86.3812876	75.731787	108.239515
17:39:46	0:37:46		75.6930524	84.3812876	74.731787	107.239515
17:39:47	0:37:47		76.6930524	82.3812876	74.731787	106.239515
17:39:48	0:37:48		76.6930524	82.3812876	74.731787	106.239515
17:39:49	0:37:49		76.6930524	82.3812876	74.731787	104.239515
17:39:50	0:37:50		76.6930524	77.3812876	74.731787	104.239515
17:39:51	0:37:51		76.6930524	75.3812876	74.731787	104.239515
17:39:52	0:37:52		76.6930524	75.3812876	74.731787	103.239515
17:39:53	0:37:53		76.6930524	75.3812876	74.731787	103.239515
17:39:54	0:37:54		76.6930524	78.3812876	73.731787	103.239515
17:39:55	0:37:55		76.6930524	74.3812876	73.731787	102.239515
17:39:56	0:37:56		75.6930524	74.3812876	73.731787	102.239515
17:39:57	0:37:57		75.6930524	72.3812876	73.731787	102.239515
17:39:58	0:37:58		75.6930524	71.3812876	73.731787	102.239515
17:39:59	0:37:59		75.6930524	72.3812876	73.731787	102.239515
17:40:00	0:38:00		75.6930524	72.3812876	73.731787	101.239515
17:40:01	0:38:01		75.6930524	72.3812876	73.731787	101.239515
17:40:02	0:38:02		75.6930524	72.3812876	73.731787	100.239515
17:40:03	0:38:03		74.6930524	72.3812876	73.731787	100.239515
17:40:04	0:38:04		73.6930524	72.3812876	73.731787	98.2395153
17:40:05	0:38:05		72.6930524	72.3812876	72.731787	98.2395153
17:40:06	0:38:06		71.6930524	75.3812876	72.731787	96.2395153
17:40:07	0:38:07		71.6930524	75.3812876	71.731787	96.2395153
17:40:08	0:38:08		71.6930524	75.3812876	71.731787	95.2395153
17:40:09	0:38:09		71.6930524	75.3812876	72.731787	95.2395153
17:40:10	0:38:10		71.6930524	74.3812876	75.731787	94.2395153
17:40:11	0:38:11		71.6930524	74.3812876	77.731787	94.2395153
17:40:12	0:38:12		71.6930524	74.3812876	77.731787	94.2395153

17:40:13	0:38:13		71.6930524	74.3812876	76.731787	86.2395153
17:40:14	0:38:14		70.6930524	74.3812876	74.731787	85.2395153
17:40:15	0:38:15		69.6930524	76.3812876	72.731787	85.2395153
17:40:16	0:38:16		70.6930524	76.3812876	70.731787	84.2395153
17:40:17	0:38:17		71.6930524	76.3812876	69.731787	84.2395153
17:40:18	0:38:18		72.6930524	76.3812876	69.731787	84.2395153
17:40:19	0:38:19		72.6930524	77.3812876	69.731787	84.2395153
17:40:20	0:38:20		73.6930524	77.3812876	69.731787	84.2395153
17:40:21	0:38:21		73.6930524	75.3812876	70.731787	84.2395153
17:40:22	0:38:22		73.6930524	74.3812876	72.731787	84.2395153
17:40:23	0:38:23		73.6930524	74.3812876	75.731787	84.2395153
17:40:24	0:38:24		73.6930524	77.3812876	76.731787	85.2395153
17:40:25	0:38:25		72.6930524	79.3812876	75.731787	85.2395153
17:40:26	0:38:26		72.6930524	79.3812876	76.731787	85.2395153
17:40:27	0:38:27		72.6930524	78.3812876	77.731787	85.2395153
17:40:28	0:38:28		72.6930524	74.3812876	77.731787	86.2395153
17:40:29	0:38:29		72.6930524	74.3812876	76.731787	86.2395153
17:40:30	0:38:30		72.6930524	74.3812876	75.731787	85.2395153
17:40:31	0:38:31		73.6930524	74.3812876	74.731787	85.2395153
17:40:32	0:38:32		74.6930524	73.3812876	74.731787	85.2395153
17:40:33	0:38:33		74.6930524	73.3812876	74.731787	82.2395153
17:40:34	0:38:34		74.6930524	72.3812876	74.731787	82.2395153
17:40:35	0:38:35		74.6930524	76.3812876	74.731787	79.2395153
17:40:36	0:38:36		74.6930524	78.3812876	74.731787	77.2395153
17:40:37	0:38:37		74.6930524	79.3812876	74.731787	76.2395153
17:40:38	0:38:38		74.6930524	79.3812876	74.731787	76.2395153
17:40:39	0:38:39		74.6930524	79.3812876	74.731787	76.2395153
17:40:40	0:38:40		75.6930524	79.3812876	75.731787	76.2395153
17:40:41	0:38:41		76.6930524	79.3812876	75.731787	76.2395153
17:40:42	0:38:42		76.6930524	79.3812876	74.731787	79.2395153
17:40:43	0:38:43		77.6930524	78.3812876	74.731787	79.2395153
17:40:44	0:38:44		77.6930524	77.3812876	75.731787	79.2395153
17:40:45	0:38:45		76.6930524	77.3812876	76.731787	79.2395153
17:40:46	0:38:46		76.6930524	77.3812876	76.731787	80.2395153
17:40:47	0:38:47		75.6930524	77.3812876	77.731787	79.2395153
17:40:48	0:38:48		75.6930524	74.3812876	77.731787	79.2395153
17:40:49	0:38:49		75.6930524	74.3812876	77.731787	79.2395153
17:40:50	0:38:50		75.6930524	74.3812876	76.731787	79.2395153
17:40:51	0:38:51		76.6930524	72.3812876	76.731787	79.2395153
17:40:52	0:38:52		77.6930524	72.3812876	75.731787	79.2395153
17:40:53	0:38:53		77.6930524	72.3812876	75.731787	80.2395153
17:40:54	0:38:54		78.6930524	71.3812876	74.731787	81.2395153
17:40:55	0:38:55		78.6930524	71.3812876	74.731787	81.2395153
17:40:56	0:38:56		78.6930524	71.3812876	73.731787	83.2395153
17:40:57	0:38:57		78.6930524	71.3812876	73.731787	83.2395153

17:40:58	0:38:58		78.6930524	71.3812876	73.731787	84.2395153
17:40:59	0:38:59		78.6930524	71.3812876	72.731787	85.2395153
17:41:00	0:39:00		78.6930524	71.3812876	72.731787	86.2395153
17:41:01	0:39:01		77.6930524	71.3812876	71.731787	86.2395153
17:41:02	0:39:02		76.6930524	68.3812876	71.731787	86.2395153
17:41:03	0:39:03		75.6930524	66.3812876	70.731787	86.2395153
17:41:04	0:39:04		75.6930524	65.3812876	70.731787	83.2395153
17:41:05	0:39:05		76.6930524	65.3812876	72.731787	82.2395153
17:41:06	0:39:06		76.6930524	65.3812876	72.731787	82.2395153
17:41:07	0:39:07		77.6930524	68.3812876	72.731787	82.2395153
17:41:08	0:39:08		77.6930524	68.3812876	73.731787	82.2395153
17:41:09	0:39:09		77.6930524	69.3812876	73.731787	81.2395153
17:41:10	0:39:10		77.6930524	69.3812876	74.731787	80.2395153
17:41:11	0:39:11		77.6930524	69.3812876	74.731787	80.2395153
17:41:12	0:39:12		76.6930524	69.3812876	74.731787	79.2395153
17:41:13	0:39:13		76.6930524	68.3812876	74.731787	78.2395153
17:41:14	0:39:14		75.6930524	69.3812876	74.731787	77.2395153
17:41:15	0:39:15		75.6930524	69.3812876	74.731787	75.2395153
17:41:16	0:39:16		75.6930524	69.3812876	74.731787	74.2395153
17:41:17	0:39:17		75.6930524	69.3812876	74.731787	73.2395153
17:41:18	0:39:18		75.6930524	69.3812876	74.731787	73.2395153
17:41:19	0:39:19		75.6930524	69.3812876	74.731787	75.2395153
17:41:20	0:39:20		76.6930524	69.3812876	74.731787	75.2395153
17:41:21	0:39:21		77.6930524	69.3812876	73.731787	76.2395153
17:41:22	0:39:22		77.6930524	69.3812876	70.731787	77.2395153
17:41:23	0:39:23		77.6930524	69.3812876	69.731787	77.2395153
17:41:24	0:39:24		77.6930524	67.3812876	69.731787	79.2395153
17:41:25	0:39:25		76.6930524	66.3812876	68.731787	80.2395153
17:41:26	0:39:26		75.6930524	66.3812876	68.731787	80.2395153
17:41:27	0:39:27		75.6930524	66.3812876	68.731787	80.2395153
17:41:28	0:39:28		75.6930524	68.3812876	67.731787	80.2395153
17:41:29	0:39:29		75.6930524	68.3812876	67.731787	80.2395153
17:41:30	0:39:30		75.6930524	68.3812876	66.731787	79.2395153
17:41:31	0:39:31		76.6930524	68.3812876	70.731787	77.2395153
17:41:32	0:39:32		76.6930524	69.3812876	74.731787	79.2395153
17:41:33	0:39:33		76.6930524	69.3812876	75.731787	79.2395153
17:41:34	0:39:34		76.6930524	69.3812876	76.731787	79.2395153
17:41:35	0:39:35		77.6930524	69.3812876	77.731787	79.2395153
17:41:36	0:39:36		77.6930524	70.3812876	77.731787	79.2395153
17:41:37	0:39:37		77.6930524	70.3812876	78.731787	79.2395153
17:41:38	0:39:38		77.6930524	70.3812876	79.731787	80.2395153
17:41:39	0:39:39		76.6930524	71.3812876	80.731787	80.2395153
17:41:40	0:39:40		76.6930524	71.3812876	80.731787	80.2395153
17:41:41	0:39:41		76.6930524	71.3812876	80.731787	80.2395153
17:41:42	0:39:42		76.6930524	71.3812876	80.731787	80.2395153

17:41:43	0:39:43		76.6930524	71.3812876	80.731787	79.2395153
17:41:44	0:39:44		77.6930524	71.3812876	81.731787	79.2395153
17:41:45	0:39:45		77.6930524	71.3812876	81.731787	79.2395153
17:41:46	0:39:46		83.6930524	71.3812876	81.731787	79.2395153
17:41:47	0:39:47		81.6930524	71.3812876	82.731787	79.2395153
17:41:48	0:39:48		81.6930524	71.3812876	82.731787	79.2395153
17:41:49	0:39:49		83.6930524	71.3812876	82.731787	81.2395153
17:41:50	0:39:50		87.6930524	71.3812876	78.731787	81.2395153
17:41:51	0:39:51		89.6930524	71.3812876	77.731787	81.2395153
17:41:52	0:39:52		90.6930524	70.3812876	77.731787	81.2395153
17:41:53	0:39:53		90.6930524	70.3812876	77.731787	81.2395153
17:41:54	0:39:54		90.6930524	70.3812876	77.731787	82.2395153
17:41:55	0:39:55		93.6930524	69.3812876	78.731787	82.2395153
17:41:56	0:39:56		96.6930524	69.3812876	79.731787	81.2395153
17:41:57	0:39:57		98.6930524	69.3812876	79.731787	79.2395153
17:41:58	0:39:58		99.6930524	70.3812876	80.731787	78.2395153
17:41:59	0:39:59		99.6930524	70.3812876	80.731787	78.2395153
17:42:00	0:40:00		99.6930524	70.3812876	80.731787	78.2395153
17:42:01	0:40:01		100.693052	71.3812876	81.731787	77.2395153
17:42:02	0:40:02		94.6930524	72.3812876	82.731787	77.2395153
17:42:03	0:40:03		86.6930524	73.3812876	83.731787	77.2395153
17:42:04	0:40:04		86.6930524	73.3812876	85.731787	78.2395153
17:42:05	0:40:05		82.6930524	72.3812876	86.731787	78.2395153
17:42:06	0:40:06		81.6930524	71.3812876	87.731787	78.2395153
17:42:07	0:40:07		80.6930524	71.3812876	84.731787	78.2395153
17:42:08	0:40:08		79.6930524	71.3812876	83.731787	79.2395153
17:42:09	0:40:09		79.6930524	71.3812876	82.731787	80.2395153
17:42:10	0:40:10		78.6930524	71.3812876	81.731787	80.2395153
17:42:11	0:40:11		78.6930524	71.3812876	80.731787	81.2395153
17:42:12	0:40:12		78.6930524	71.3812876	80.731787	81.2395153
17:42:13	0:40:13		78.6930524	70.3812876	79.731787	81.2395153
17:42:14	0:40:14		77.6930524	70.3812876	78.731787	83.2395153
17:42:15	0:40:15		77.6930524	70.3812876	77.731787	83.2395153
17:42:16	0:40:16		77.6930524	70.3812876	77.731787	84.2395153
17:42:17	0:40:17		77.6930524	70.3812876	77.731787	84.2395153
17:42:18	0:40:18		77.6930524	70.3812876	77.731787	84.2395153
17:42:19	0:40:19		75.6930524	70.3812876	76.731787	84.2395153
17:42:20	0:40:20		73.6930524	70.3812876	75.731787	85.2395153
17:42:21	0:40:21		73.6930524	70.3812876	75.731787	85.2395153
17:42:22	0:40:22		75.6930524	70.3812876	74.731787	86.2395153
17:42:23	0:40:23		77.6930524	70.3812876	74.731787	86.2395153
17:42:24	0:40:24		77.6930524	70.3812876	77.731787	87.2395153
17:42:25	0:40:25		78.6930524	70.3812876	80.731787	87.2395153
17:42:26	0:40:26		78.6930524	71.3812876	83.731787	87.2395153
17:42:27	0:40:27		78.6930524	71.3812876	84.731787	87.2395153

17:42:28	0:40:28		78.6930524	71.3812876	84.731787	87.2395153
17:42:29	0:40:29		78.6930524	71.3812876	82.731787	87.2395153
17:42:30	0:40:30		78.6930524	72.3812876	81.731787	88.2395153
17:42:31	0:40:31		78.6930524	72.3812876	80.731787	89.2395153
17:42:32	0:40:32		76.6930524	74.3812876	80.731787	90.2395153
17:42:33	0:40:33		75.6930524	76.3812876	78.731787	90.2395153
17:42:34	0:40:34		75.6930524	77.3812876	77.731787	91.2395153
17:42:35	0:40:35		74.6930524	77.3812876	76.731787	91.2395153
17:42:36	0:40:36		74.6930524	76.3812876	75.731787	92.2395153
17:42:37	0:40:37		74.6930524	76.3812876	75.731787	91.2395153
17:42:38	0:40:38		74.6930524	76.3812876	74.731787	88.2395153
17:42:39	0:40:39		74.6930524	77.3812876	73.731787	87.2395153
17:42:40	0:40:40		75.6930524	78.3812876	73.731787	85.2395153
17:42:41	0:40:41		76.6930524	81.3812876	72.731787	84.2395153
17:42:42	0:40:42		76.6930524	83.3812876	72.731787	82.2395153
17:42:43	0:40:43		74.6930524	83.3812876	72.731787	82.2395153
17:42:44	0:40:44		74.6930524	84.3812876	72.731787	81.2395153
17:42:45	0:40:45		73.6930524	84.3812876	72.731787	81.2395153
17:42:46	0:40:46		73.6930524	80.3812876	73.731787	80.2395153
17:42:47	0:40:47		74.6930524	80.3812876	74.731787	77.2395153
17:42:48	0:40:48		74.6930524	67.3812876	74.731787	77.2395153
17:42:49	0:40:49		73.6930524	64.3812876	74.731787	78.2395153
17:42:50	0:40:50		73.6930524	62.3812876	74.731787	78.2395153
17:42:51	0:40:51		74.6930524	62.3812876	73.731787	80.2395153
17:42:52	0:40:52		73.6930524	63.3812876	73.731787	88.2395153
17:42:53	0:40:53		72.6930524	63.3812876	73.731787	92.2395153
17:42:54	0:40:54		72.6930524	63.3812876	73.731787	92.2395153
17:42:55	0:40:55		70.6930524	63.3812876	73.731787	95.2395153
17:42:56	0:40:56		70.6930524	63.3812876	74.731787	95.2395153
17:42:57	0:40:57		70.6930524	63.3812876	74.731787	95.2395153
17:42:58	0:40:58		69.6930524	64.3812876	74.731787	95.2395153
17:42:59	0:40:59		65.6930524	64.3812876	75.731787	96.2395153
17:43:00	0:41:00		64.6930524	62.3812876	75.731787	96.2395153
17:43:01	0:41:01		64.6930524	62.3812876	79.731787	94.2395153
17:43:02	0:41:02		66.6930524	64.3812876	79.731787	79.2395153
17:43:03	0:41:03		67.6930524	64.3812876	79.731787	70.2395153
17:43:04	0:41:04		69.6930524	66.3812876	76.731787	66.2395153
17:43:05	0:41:05		70.6930524	69.3812876	75.731787	63.2395153
17:43:06	0:41:06		71.6930524	69.3812876	75.731787	63.2395153
17:43:07	0:41:07		71.6930524	69.3812876	75.731787	64.2395153
17:43:08	0:41:08		71.6930524	69.3812876	75.731787	64.2395153
17:43:09	0:41:09		71.6930524	70.3812876	75.731787	64.2395153
17:43:10	0:41:10		71.6930524	73.3812876	75.731787	64.2395153
17:43:11	0:41:11		72.6930524	73.3812876	75.731787	64.2395153
17:43:12	0:41:12		72.6930524	73.3812876	76.731787	65.2395153

17:43:13	0:41:13		72.6930524	74.3812876	77.731787	67.2395153
17:43:14	0:41:14		72.6930524	75.3812876	77.731787	67.2395153
17:43:15	0:41:15		72.6930524	73.3812876	77.731787	71.2395153
17:43:16	0:41:16		72.6930524	73.3812876	79.731787	73.2395153
17:43:17	0:41:17		73.6930524	72.3812876	79.731787	74.2395153
17:43:18	0:41:18		72.6930524	72.3812876	79.731787	75.2395153
17:43:19	0:41:19		72.6930524	72.3812876	81.731787	76.2395153
17:43:20	0:41:20		72.6930524	72.3812876	81.731787	76.2395153
17:43:21	0:41:21		72.6930524	71.3812876	77.731787	76.2395153
17:43:22	0:41:22		72.6930524	71.3812876	76.731787	74.2395153
17:43:23	0:41:23		72.6930524	71.3812876	76.731787	74.2395153
17:43:24	0:41:24		71.6930524	71.3812876	76.731787	74.2395153
17:43:25	0:41:25		71.6930524	70.3812876	76.731787	73.2395153
17:43:26	0:41:26		73.6930524	65.3812876	76.731787	72.2395153
17:43:27	0:41:27		74.6930524	65.3812876	76.731787	70.2395153
17:43:28	0:41:28		74.6930524	65.3812876	76.731787	69.2395153
17:43:29	0:41:29		74.6930524	66.3812876	76.731787	69.2395153
17:43:30	0:41:30		75.6930524	68.3812876	77.731787	69.2395153
17:43:31	0:41:31		76.6930524	68.3812876	80.731787	69.2395153
17:43:32	0:41:32		76.6930524	68.3812876	80.731787	68.2395153
17:43:33	0:41:33		76.6930524	68.3812876	80.731787	68.2395153
17:43:34	0:41:34		74.6930524	68.3812876	80.731787	68.2395153
17:43:35	0:41:35		74.6930524	68.3812876	78.731787	68.2395153
17:43:36	0:41:36		74.6930524	68.3812876	75.731787	68.2395153
17:43:37	0:41:37		74.6930524	68.3812876	74.731787	68.2395153
17:43:38	0:41:38		74.6930524	68.3812876	74.731787	68.2395153
17:43:39	0:41:39		73.6930524	69.3812876	73.731787	69.2395153
17:43:40	0:41:40		73.6930524	69.3812876	73.731787	69.2395153
17:43:41	0:41:41		73.6930524	69.3812876	73.731787	69.2395153
17:43:42	0:41:42		73.6930524	69.3812876	73.731787	69.2395153
17:43:43	0:41:43		73.6930524	70.3812876	73.731787	68.2395153
17:43:44	0:41:44		72.6930524	70.3812876	72.731787	69.2395153
17:43:45	0:41:45		73.6930524	70.3812876	72.731787	69.2395153
17:43:46	0:41:46		73.6930524	70.3812876	72.731787	69.2395153
17:43:47	0:41:47		74.6930524	70.3812876	72.731787	69.2395153
17:43:48	0:41:48		74.6930524	70.3812876	72.731787	69.2395153
17:43:49	0:41:49		74.6930524	70.3812876	72.731787	69.2395153
17:43:50	0:41:50		74.6930524	70.3812876	73.731787	66.2395153
17:43:51	0:41:51		74.6930524	70.3812876	73.731787	66.2395153
17:43:52	0:41:52		74.6930524	70.3812876	73.731787	62.2395153
17:43:53	0:41:53		74.6930524	69.3812876	73.731787	61.2395153
17:43:54	0:41:54		73.6930524	69.3812876	73.731787	61.2395153
17:43:55	0:41:55		73.6930524	68.3812876	73.731787	62.2395153
17:43:56	0:41:56		74.6930524	68.3812876	73.731787	64.2395153
17:43:57	0:41:57		74.6930524	65.3812876	74.731787	64.2395153

17:43:58	0:41:58		74.6930524	62.3812876	74.731787	66.2395153
17:43:59	0:41:59		74.6930524	60.3812876	74.731787	66.2395153
17:44:00	0:42:00		74.6930524	60.3812876	75.731787	64.2395153
17:44:01	0:42:01		74.6930524	55.3812876	75.731787	63.2395153
17:44:02	0:42:02		75.6930524	55.3812876	75.731787	63.2395153
17:44:03	0:42:03		76.6930524	55.3812876	75.731787	63.2395153
17:44:04	0:42:04		76.6930524	56.3812876	75.731787	62.2395153
17:44:05	0:42:05		77.6930524	56.3812876	75.731787	62.2395153
17:44:06	0:42:06		77.6930524	57.3812876	75.731787	62.2395153
17:44:07	0:42:07		78.6930524	57.3812876	74.731787	61.2395153
17:44:08	0:42:08		78.6930524	57.3812876	74.731787	61.2395153
17:44:09	0:42:09		78.6930524	61.3812876	74.731787	60.2395153
17:44:10	0:42:10		77.6930524	63.3812876	73.731787	59.2395153
17:44:11	0:42:11		76.6930524	63.3812876	72.731787	60.2395153
17:44:12	0:42:12		76.6930524	64.3812876	73.731787	61.2395153
17:44:13	0:42:13		75.6930524	64.3812876	74.731787	61.2395153
17:44:14	0:42:14		75.6930524	64.3812876	74.731787	62.2395153
17:44:15	0:42:15		75.6930524	64.3812876	74.731787	70.2395153
17:44:16	0:42:16		75.6930524	64.3812876	74.731787	73.2395153
17:44:17	0:42:17		76.6930524	64.3812876	74.731787	77.2395153
17:44:18	0:42:18		76.6930524	64.3812876	75.731787	77.2395153
17:44:19	0:42:19		76.6930524	64.3812876	75.731787	78.2395153
17:44:20	0:42:20		76.6930524	64.3812876	76.731787	79.2395153
17:44:21	0:42:21		76.6930524	66.3812876	76.731787	81.2395153
17:44:22	0:42:22		75.6930524	66.3812876	76.731787	81.2395153
17:44:23	0:42:23		73.6930524	66.3812876	77.731787	81.2395153
17:44:24	0:42:24		73.6930524	66.3812876	79.731787	81.2395153
17:44:25	0:42:25		72.6930524	66.3812876	79.731787	81.2395153
17:44:26	0:42:26		72.6930524	67.3812876	78.731787	82.2395153
17:44:27	0:42:27		72.6930524	67.3812876	78.731787	82.2395153
17:44:28	0:42:28		71.6930524	66.3812876	79.731787	83.2395153
17:44:29	0:42:29		71.6930524	65.3812876	79.731787	84.2395153
17:44:30	0:42:30		71.6930524	64.3812876	79.731787	86.2395153
17:44:31	0:42:31		71.6930524	64.3812876	78.731787	86.2395153
17:44:32	0:42:32		71.6930524	62.3812876	78.731787	86.2395153
17:44:33	0:42:33		71.6930524	62.3812876	78.731787	86.2395153
17:44:34	0:42:34		71.6930524	62.3812876	79.731787	86.2395153
17:44:35	0:42:35		71.6930524	62.3812876	80.731787	86.2395153
17:44:36	0:42:36		71.6930524	62.3812876	80.731787	86.2395153
17:44:37	0:42:37		71.6930524	63.3812876	78.731787	83.2395153
17:44:38	0:42:38		69.6930524	63.3812876	77.731787	82.2395153
17:44:39	0:42:39		69.6930524	63.3812876	76.731787	82.2395153
17:44:40	0:42:40		69.6930524	63.3812876	76.731787	81.2395153
17:44:41	0:42:41		69.6930524	63.3812876	76.731787	81.2395153
17:44:42	0:42:42		69.6930524	63.3812876	76.731787	81.2395153

17:44:43	0:42:43		71.6930524	65.3812876	77.731787	77.2395153
17:44:44	0:42:44		72.6930524	65.3812876	77.731787	77.2395153
17:44:45	0:42:45		73.6930524	69.3812876	76.731787	81.2395153
17:44:46	0:42:46		73.6930524	73.3812876	76.731787	82.2395153
17:44:47	0:42:47		74.6930524	73.3812876	75.731787	83.2395153
17:44:48	0:42:48		74.6930524	72.3812876	75.731787	83.2395153
17:44:49	0:42:49		74.6930524	72.3812876	74.731787	83.2395153
17:44:50	0:42:50		74.6930524	72.3812876	74.731787	83.2395153
17:44:51	0:42:51		74.6930524	72.3812876	71.731787	81.2395153
17:44:52	0:42:52		75.6930524	72.3812876	71.731787	79.2395153
17:44:53	0:42:53		75.6930524	70.3812876	71.731787	79.2395153
17:44:54	0:42:54		74.6930524	70.3812876	71.731787	78.2395153
17:44:55	0:42:55		74.6930524	69.3812876	72.731787	78.2395153
17:44:56	0:42:56		74.6930524	70.3812876	73.731787	78.2395153
17:44:57	0:42:57		74.6930524	70.3812876	74.731787	78.2395153
17:44:58	0:42:58		74.6930524	70.3812876	74.731787	78.2395153
17:44:59	0:42:59		74.6930524	71.3812876	76.731787	77.2395153
17:45:00	0:43:00		75.6930524	69.3812876	75.731787	78.2395153
17:45:01	0:43:01		75.6930524	68.3812876	74.731787	78.2395153
17:45:02	0:43:02		75.6930524	68.3812876	73.731787	78.2395153
17:45:03	0:43:03		75.6930524	67.3812876	72.731787	78.2395153
17:45:04	0:43:04		75.6930524	66.3812876	72.731787	77.2395153
17:45:05	0:43:05		77.6930524	66.3812876	72.731787	77.2395153
17:45:06	0:43:06		77.6930524	65.3812876	72.731787	77.2395153
17:45:07	0:43:07		77.6930524	65.3812876	72.731787	77.2395153
17:45:08	0:43:08		77.6930524	66.3812876	72.731787	77.2395153
17:45:09	0:43:09		77.6930524	66.3812876	71.731787	77.2395153
17:45:10	0:43:10		77.6930524	67.3812876	71.731787	77.2395153
17:45:11	0:43:11		76.6930524	67.3812876	71.731787	77.2395153
17:45:12	0:43:12		76.6930524	67.3812876	71.731787	77.2395153
17:45:13	0:43:13		77.6930524	67.3812876	70.731787	77.2395153
17:45:14	0:43:14		78.6930524	67.3812876	70.731787	77.2395153
17:45:15	0:43:15		77.6930524	68.3812876	71.731787	77.2395153
17:45:16	0:43:16		75.6930524	68.3812876	72.731787	77.2395153
17:45:17	0:43:17		75.6930524	75.3812876	72.731787	74.2395153
17:45:18	0:43:18		74.6930524	74.3812876	72.731787	72.2395153
17:45:19	0:43:19		74.6930524	73.3812876	72.731787	71.2395153
17:45:20	0:43:20		73.6930524	73.3812876	75.731787	70.2395153
17:45:21	0:43:21		73.6930524	73.3812876	79.731787	69.2395153
17:45:22	0:43:22		73.6930524	73.3812876	79.731787	69.2395153
17:45:23	0:43:23		74.6930524	73.3812876	79.731787	68.2395153
17:45:24	0:43:24		74.6930524	71.3812876	78.731787	68.2395153
17:45:25	0:43:25		74.6930524	70.3812876	76.731787	68.2395153
17:45:26	0:43:26		73.6930524	69.3812876	76.731787	68.2395153
17:45:27	0:43:27		74.6930524	69.3812876	76.731787	66.2395153

17:45:28	0:43:28		74.6930524	69.3812876	76.731787	66.2395153
17:45:29	0:43:29		73.6930524	70.3812876	76.731787	66.2395153
17:45:30	0:43:30		73.6930524	70.3812876	76.731787	66.2395153
17:45:31	0:43:31		72.6930524	71.3812876	76.731787	66.2395153
17:45:32	0:43:32		71.6930524	71.3812876	77.731787	65.2395153
17:45:33	0:43:33		71.6930524	72.3812876	78.731787	66.2395153
17:45:34	0:43:34		70.6930524	72.3812876	78.731787	67.2395153
17:45:35	0:43:35		70.6930524	73.3812876	78.731787	68.2395153
17:45:36	0:43:36		70.6930524	73.3812876	78.731787	68.2395153
17:45:37	0:43:37		71.6930524	72.3812876	75.731787	68.2395153
17:45:38	0:43:38		71.6930524	71.3812876	73.731787	70.2395153
17:45:39	0:43:39		72.6930524	70.3812876	73.731787	71.2395153
17:45:40	0:43:40		72.6930524	71.3812876	73.731787	71.2395153
17:45:41	0:43:41		72.6930524	71.3812876	74.731787	71.2395153
17:45:42	0:43:42		73.6930524	71.3812876	76.731787	71.2395153
17:45:43	0:43:43		73.6930524	71.3812876	77.731787	71.2395153
17:45:44	0:43:44		73.6930524	71.3812876	77.731787	71.2395153
17:45:45	0:43:45		73.6930524	71.3812876	77.731787	71.2395153
17:45:46	0:43:46		73.6930524	71.3812876	77.731787	71.2395153
17:45:47	0:43:47		73.6930524	73.3812876	76.731787	71.2395153
17:45:48	0:43:48		73.6930524	78.3812876	76.731787	71.2395153
17:45:49	0:43:49		72.6930524	78.3812876	77.731787	71.2395153
17:45:50	0:43:50		72.6930524	79.3812876	77.731787	72.2395153
17:45:51	0:43:51		72.6930524	79.3812876	78.731787	72.2395153
17:45:52	0:43:52		72.6930524	79.3812876	78.731787	72.2395153
17:45:53	0:43:53		72.6930524	80.3812876	78.731787	72.2395153
17:45:54	0:43:54		72.6930524	80.3812876	78.731787	71.2395153
17:45:55	0:43:55		74.6930524	80.3812876	82.731787	72.2395153
17:45:56	0:43:56		74.6930524	80.3812876	83.731787	72.2395153
17:45:57	0:43:57		74.6930524	80.3812876	83.731787	72.2395153
17:45:58	0:43:58		74.6930524	78.3812876	83.731787	75.2395153
17:45:59	0:43:59		74.6930524	77.3812876	83.731787	75.2395153
17:46:00	0:44:00		74.6930524	73.3812876	83.731787	73.2395153
17:46:01	0:44:01		73.6930524	70.3812876	84.731787	70.2395153
17:46:02	0:44:02		73.6930524	69.3812876	86.731787	69.2395153
17:46:03	0:44:03		72.6930524	69.3812876	85.731787	69.2395153
17:46:04	0:44:04		72.6930524	70.3812876	83.731787	68.2395153
17:46:05	0:44:05		72.6930524	71.3812876	81.731787	68.2395153
17:46:06	0:44:06		72.6930524	72.3812876	79.731787	67.2395153
17:46:07	0:44:07		73.6930524	73.3812876	79.731787	64.2395153
17:46:08	0:44:08		73.6930524	73.3812876	79.731787	63.2395153
17:46:09	0:44:09		75.6930524	73.3812876	78.731787	63.2395153
17:46:10	0:44:10		75.6930524	73.3812876	77.731787	63.2395153
17:46:11	0:44:11		75.6930524	73.3812876	75.731787	63.2395153
17:46:12	0:44:12		74.6930524	74.3812876	75.731787	63.2395153

17:46:13	0:44:13		74.6930524	74.3812876	74.731787	63.2395153
17:46:14	0:44:14		74.6930524	73.3812876	73.731787	64.2395153
17:46:15	0:44:15		69.6930524	71.3812876	73.731787	65.2395153
17:46:16	0:44:16		68.6930524	71.3812876	74.731787	65.2395153
17:46:17	0:44:17		68.6930524	70.3812876	75.731787	66.2395153
17:46:18	0:44:18		68.6930524	70.3812876	74.731787	67.2395153
17:46:19	0:44:19		68.6930524	67.3812876	74.731787	69.2395153
17:46:20	0:44:20		68.6930524	67.3812876	74.731787	69.2395153
17:46:21	0:44:21		67.6930524	70.3812876	74.731787	69.2395153
17:46:22	0:44:22		67.6930524	71.3812876	74.731787	70.2395153
17:46:23	0:44:23		68.6930524	72.3812876	74.731787	70.2395153
17:46:24	0:44:24		69.6930524	72.3812876	74.731787	70.2395153
17:46:25	0:44:25		72.6930524	72.3812876	74.731787	71.2395153
17:46:26	0:44:26		72.6930524	72.3812876	74.731787	71.2395153
17:46:27	0:44:27		72.6930524	73.3812876	74.731787	71.2395153
17:46:28	0:44:28		72.6930524	75.3812876	74.731787	71.2395153
17:46:29	0:44:29		73.6930524	75.3812876	74.731787	71.2395153
17:46:30	0:44:30		73.6930524	75.3812876	74.731787	71.2395153
17:46:31	0:44:31		75.6930524	74.3812876	76.731787	73.2395153
17:46:32	0:44:32		75.6930524	75.3812876	76.731787	73.2395153
17:46:33	0:44:33		75.6930524	75.3812876	76.731787	74.2395153
17:46:34	0:44:34		77.6930524	75.3812876	76.731787	74.2395153
17:46:35	0:44:35		78.6930524	74.3812876	76.731787	73.2395153
17:46:36	0:44:36		78.6930524	74.3812876	76.731787	73.2395153
17:46:37	0:44:37		78.6930524	74.3812876	77.731787	71.2395153
17:46:38	0:44:38		78.6930524	74.3812876	77.731787	68.2395153
17:46:39	0:44:39		78.6930524	76.3812876	79.731787	67.2395153
17:46:40	0:44:40		79.6930524	78.3812876	81.731787	67.2395153
17:46:41	0:44:41		79.6930524	78.3812876	81.731787	64.2395153
17:46:42	0:44:42		79.6930524	79.3812876	80.731787	64.2395153
17:46:43	0:44:43		79.6930524	80.3812876	74.731787	66.2395153
17:46:44	0:44:44		79.6930524	80.3812876	73.731787	68.2395153
17:46:45	0:44:45		77.6930524	80.3812876	72.731787	69.2395153
17:46:46	0:44:46		75.6930524	79.3812876	73.731787	69.2395153
17:46:47	0:44:47		74.6930524	77.3812876	73.731787	70.2395153
17:46:48	0:44:48		73.6930524	75.3812876	74.731787	71.2395153
17:46:49	0:44:49		73.6930524	75.3812876	74.731787	71.2395153
17:46:50	0:44:50		71.6930524	71.3812876	74.731787	70.2395153
17:46:51	0:44:51		71.6930524	70.3812876	75.731787	70.2395153
17:46:52	0:44:52		71.6930524	68.3812876	74.731787	70.2395153
17:46:53	0:44:53		72.6930524	68.3812876	73.731787	70.2395153
17:46:54	0:44:54		73.6930524	68.3812876	73.731787	70.2395153
17:46:55	0:44:55		74.6930524	69.3812876	73.731787	72.2395153
17:46:56	0:44:56		75.6930524	69.3812876	73.731787	74.2395153
17:46:57	0:44:57		75.6930524	72.3812876	73.731787	75.2395153

17:46:58	0:44:58		75.6930524	76.3812876	73.731787	78.2395153
17:46:59	0:44:59		75.6930524	77.3812876	74.731787	79.2395153
17:47:00	0:45:00		75.6930524	77.3812876	75.731787	79.2395153
17:47:01	0:45:01		75.6930524	78.3812876	76.731787	78.2395153
17:47:02	0:45:02		74.6930524	78.3812876	76.731787	78.2395153
17:47:03	0:45:03		74.6930524	79.3812876	76.731787	77.2395153
17:47:04	0:45:04		73.6930524	83.3812876	76.731787	77.2395153
17:47:05	0:45:05		73.6930524	83.3812876	76.731787	77.2395153
17:47:06	0:45:06		72.6930524	84.3812876	75.731787	77.2395153
17:47:07	0:45:07		72.6930524	85.3812876	75.731787	78.2395153
17:47:08	0:45:08		72.6930524	86.3812876	76.731787	80.2395153
17:47:09	0:45:09		72.6930524	86.3812876	76.731787	80.2395153
17:47:10	0:45:10		72.6930524	86.3812876	76.731787	80.2395153
17:47:11	0:45:11		72.6930524	87.3812876	76.731787	80.2395153
17:47:12	0:45:12		72.6930524	88.3812876	76.731787	80.2395153
17:47:13	0:45:13		73.6930524	80.3812876	77.731787	79.2395153
17:47:14	0:45:14		73.6930524	79.3812876	79.731787	78.2395153
17:47:15	0:45:15		74.6930524	79.3812876	80.731787	79.2395153
17:47:16	0:45:16		74.6930524	79.3812876	79.731787	79.2395153
17:47:17	0:45:17		74.6930524	78.3812876	77.731787	78.2395153
17:47:18	0:45:18		73.6930524	78.3812876	74.731787	78.2395153
17:47:19	0:45:19		73.6930524	78.3812876	73.731787	77.2395153
17:47:20	0:45:20		73.6930524	78.3812876	72.731787	77.2395153
17:47:21	0:45:21		73.6930524	78.3812876	72.731787	77.2395153
17:47:22	0:45:22		73.6930524	78.3812876	72.731787	77.2395153
17:47:23	0:45:23		72.6930524	78.3812876	74.731787	78.2395153
17:47:24	0:45:24		72.6930524	78.3812876	74.731787	78.2395153
17:47:25	0:45:25		72.6930524	78.3812876	75.731787	75.2395153
17:47:26	0:45:26		72.6930524	77.3812876	76.731787	75.2395153
17:47:27	0:45:27		72.6930524	77.3812876	76.731787	75.2395153
17:47:28	0:45:28		72.6930524	77.3812876	76.731787	75.2395153
17:47:29	0:45:29		72.6930524	77.3812876	76.731787	75.2395153
17:47:30	0:45:30		74.6930524	77.3812876	75.731787	74.2395153
17:47:31	0:45:31		77.6930524	77.3812876	75.731787	73.2395153
17:47:32	0:45:32		82.6930524	77.3812876	75.731787	72.2395153
17:47:33	0:45:33		82.6930524	77.3812876	76.731787	72.2395153
17:47:34	0:45:34		79.6930524	78.3812876	77.731787	69.2395153
17:47:35	0:45:35		78.6930524	78.3812876	77.731787	69.2395153
17:47:36	0:45:36		78.6930524	77.3812876	76.731787	68.2395153
17:47:37	0:45:37		76.6930524	77.3812876	76.731787	68.2395153
17:47:38	0:45:38		76.6930524	77.3812876	76.731787	68.2395153
17:47:39	0:45:39		75.6930524	78.3812876	76.731787	68.2395153
17:47:40	0:45:40		75.6930524	78.3812876	76.731787	68.2395153
17:47:41	0:45:41		75.6930524	78.3812876	76.731787	68.2395153
17:47:42	0:45:42		75.6930524	78.3812876	76.731787	66.2395153

17:47:43	0:45:43		74.6930524	78.3812876	75.731787	66.2395153
17:47:44	0:45:44		74.6930524	78.3812876	75.731787	65.2395153
17:47:45	0:45:45		74.6930524	80.3812876	74.731787	66.2395153
17:47:46	0:45:46		73.6930524	80.3812876	74.731787	65.2395153
17:47:47	0:45:47		73.6930524	81.3812876	74.731787	65.2395153
17:47:48	0:45:48		73.6930524	81.3812876	74.731787	65.2395153
17:47:49	0:45:49		73.6930524	81.3812876	75.731787	65.2395153
17:47:50	0:45:50		73.6930524	81.3812876	75.731787	63.2395153
17:47:51	0:45:51		73.6930524	81.3812876	75.731787	61.2395153
17:47:52	0:45:52		74.6930524	81.3812876	76.731787	61.2395153
17:47:53	0:45:53		74.6930524	81.3812876	76.731787	58.2395153
17:47:54	0:45:54		73.6930524	80.3812876	76.731787	56.2395153
17:47:55	0:45:55		73.6930524	79.3812876	77.731787	55.2395153
17:47:56	0:45:56		73.6930524	79.3812876	77.731787	55.2395153
17:47:57	0:45:57		73.6930524	78.3812876	77.731787	55.2395153
17:47:58	0:45:58		73.6930524	78.3812876	77.731787	54.2395153
17:47:59	0:45:59		73.6930524	75.3812876	77.731787	54.2395153
17:48:00	0:46:00		74.6930524	74.3812876	77.731787	53.2395153
17:48:01	0:46:01		74.6930524	74.3812876	77.731787	53.2395153
17:48:02	0:46:02		74.6930524	74.3812876	77.731787	53.2395153
17:48:03	0:46:03		74.6930524	75.3812876	77.731787	53.2395153
17:48:04	0:46:04		74.6930524	75.3812876	77.731787	53.2395153
17:48:05	0:46:05		74.6930524	73.3812876	77.731787	54.2395153
17:48:06	0:46:06		74.6930524	73.3812876	78.731787	54.2395153
17:48:07	0:46:07		74.6930524	72.3812876	78.731787	55.2395153
17:48:08	0:46:08		74.6930524	71.3812876	79.731787	55.2395153
17:48:09	0:46:09		75.6930524	71.3812876	79.731787	55.2395153
17:48:10	0:46:10		75.6930524	71.3812876	78.731787	55.2395153
17:48:11	0:46:11		76.6930524	74.3812876	78.731787	56.2395153
17:48:12	0:46:12		77.6930524	75.3812876	78.731787	57.2395153
17:48:13	0:46:13		78.6930524	74.3812876	78.731787	58.2395153
17:48:14	0:46:14		78.6930524	72.3812876	78.731787	59.2395153
17:48:15	0:46:15		77.6930524	72.3812876	78.731787	59.2395153
17:48:16	0:46:16		77.6930524	71.3812876	78.731787	60.2395153
17:48:17	0:46:17		77.6930524	72.3812876	78.731787	60.2395153
17:48:18	0:46:18		77.6930524	72.3812876	78.731787	60.2395153
17:48:19	0:46:19		77.6930524	72.3812876	78.731787	68.2395153
17:48:20	0:46:20		78.6930524	73.3812876	80.731787	68.2395153
17:48:21	0:46:21		78.6930524	73.3812876	81.731787	72.2395153
17:48:22	0:46:22		78.6930524	73.3812876	80.731787	73.2395153
17:48:23	0:46:23		77.6930524	74.3812876	80.731787	75.2395153
17:48:24	0:46:24		76.6930524	74.3812876	80.731787	76.2395153
17:48:25	0:46:25		76.6930524	74.3812876	80.731787	77.2395153
17:48:26	0:46:26		75.6930524	73.3812876	81.731787	78.2395153
17:48:27	0:46:27		74.6930524	73.3812876	81.731787	78.2395153

17:48:28	0:46:28		74.6930524	71.3812876	81.731787	79.2395153
17:48:29	0:46:29		74.6930524	71.3812876	81.731787	80.2395153
17:48:30	0:46:30		74.6930524	71.3812876	83.731787	80.2395153
17:48:31	0:46:31		74.6930524	72.3812876	83.731787	80.2395153
17:48:32	0:46:32		74.6930524	72.3812876	82.731787	80.2395153
17:48:33	0:46:33		74.6930524	72.3812876	82.731787	80.2395153
17:48:34	0:46:34		74.6930524	71.3812876	82.731787	80.2395153
17:48:35	0:46:35		74.6930524	71.3812876	82.731787	80.2395153
17:48:36	0:46:36		74.6930524	71.3812876	82.731787	85.2395153
17:48:37	0:46:37		75.6930524	71.3812876	82.731787	85.2395153
17:48:38	0:46:38		75.6930524	71.3812876	82.731787	85.2395153
17:48:39	0:46:39		75.6930524	71.3812876	79.731787	86.2395153
17:48:40	0:46:40		75.6930524	71.3812876	79.731787	86.2395153
17:48:41	0:46:41		76.6930524	72.3812876	79.731787	86.2395153
17:48:42	0:46:42		77.6930524	72.3812876	77.731787	86.2395153
17:48:43	0:46:43		78.6930524	72.3812876	76.731787	86.2395153
17:48:44	0:46:44		78.6930524	75.3812876	76.731787	86.2395153
17:48:45	0:46:45		80.6930524	75.3812876	76.731787	82.2395153
17:48:46	0:46:46		80.6930524	75.3812876	77.731787	82.2395153
17:48:47	0:46:47		80.6930524	75.3812876	77.731787	82.2395153
17:48:48	0:46:48		79.6930524	75.3812876	78.731787	81.2395153
17:48:49	0:46:49		78.6930524	74.3812876	78.731787	81.2395153
17:48:50	0:46:50		78.6930524	74.3812876	78.731787	82.2395153
17:48:51	0:46:51		78.6930524	73.3812876	77.731787	82.2395153
17:48:52	0:46:52		77.6930524	73.3812876	77.731787	81.2395153
17:48:53	0:46:53		77.6930524	74.3812876	76.731787	82.2395153
17:48:54	0:46:54		77.6930524	74.3812876	76.731787	82.2395153
17:48:55	0:46:55		76.6930524	74.3812876	75.731787	82.2395153
17:48:56	0:46:56		76.6930524	74.3812876	75.731787	81.2395153
17:48:57	0:46:57		76.6930524	73.3812876	75.731787	81.2395153
17:48:58	0:46:58		77.6930524	73.3812876	75.731787	81.2395153
17:48:59	0:46:59		77.6930524	73.3812876	73.731787	81.2395153
17:49:00	0:47:00		77.6930524	73.3812876	72.731787	82.2395153
17:49:01	0:47:01	1	77.6930524	73.3812876	71.731787	82.2395153
17:49:02	0:47:02	2	77.6930524	73.3812876	71.731787	81.2395153
17:49:03	0:47:03	3	77.6930524	73.3812876	71.731787	81.2395153
17:49:04	0:47:04	4	76.6930524	73.3812876	71.731787	81.2395153
17:49:05	0:47:05	5	74.6930524	73.3812876	71.731787	82.2395153
17:49:06	0:47:06	6	73.6930524	72.3812876	71.731787	82.2395153
17:49:07	0:47:07	7	70.6930524	72.3812876	72.731787	82.2395153
17:49:08	0:47:08	8	70.6930524	72.3812876	73.731787	82.2395153
17:49:09	0:47:09	9	71.6930524	72.3812876	72.731787	81.2395153
17:49:10	0:47:10	10	72.6930524	72.3812876	72.731787	81.2395153
17:49:11	0:47:11	11	72.6930524	72.3812876	72.731787	81.2395153
17:49:12	0:47:12	12	72.6930524	72.3812876	72.731787	81.2395153

17:49:13	0:47:13	13	74.6930524	72.3812876	73.731787	82.2395153
17:49:14	0:47:14	14	74.6930524	73.3812876	73.731787	82.2395153
17:49:15	0:47:15	15	74.6930524	73.3812876	73.731787	82.2395153
17:49:16	0:47:16	16	75.6930524	73.3812876	73.731787	83.2395153
17:49:17	0:47:17	17	76.6930524	73.3812876	73.731787	83.2395153
17:49:18	0:47:18	18	75.6930524	74.3812876	73.731787	85.2395153
17:49:19	0:47:19	19	75.6930524	74.3812876	73.731787	85.2395153
17:49:20	0:47:20	20	74.6930524	74.3812876	73.731787	86.2395153
17:49:21	0:47:21	21	74.6930524	74.3812876	72.731787	86.2395153
17:49:22	0:47:22	22	73.6930524	73.3812876	72.731787	85.2395153
17:49:23	0:47:23	23	73.6930524	72.3812876	72.731787	85.2395153
17:49:24	0:47:24	24	72.6930524	72.3812876	73.731787	84.2395153
17:49:25	0:47:25	25	72.6930524	72.3812876	74.731787	81.2395153
17:49:26	0:47:26	26	71.6930524	73.3812876	75.731787	79.2395153
17:49:27	0:47:27	27	71.6930524	73.3812876	75.731787	79.2395153
17:49:28	0:47:28	28	71.6930524	74.3812876	75.731787	79.2395153
17:49:29	0:47:29	29	72.6930524	74.3812876	76.731787	78.2395153
17:49:30	0:47:30	30	73.6930524	77.3812876	76.731787	78.2395153
17:49:31	0:47:31	31	73.6930524	77.3812876	76.731787	78.2395153
17:49:32	0:47:32	32	73.6930524	77.3812876	76.731787	78.2395153
17:49:33	0:47:33	33	73.6930524	69.3812876	76.731787	77.2395153
17:49:34	0:47:34	34	73.6930524	66.3812876	75.731787	77.2395153
17:49:35	0:47:35	35	74.6930524	66.3812876	75.731787	76.2395153
17:49:36	0:47:36	36	74.6930524	66.3812876	74.731787	76.2395153
17:49:37	0:47:37	37	74.6930524	67.3812876	73.731787	76.2395153
17:49:38	0:47:38	38	74.6930524	67.3812876	72.731787	74.2395153
17:49:39	0:47:39	39	74.6930524	69.3812876	72.731787	74.2395153
17:49:40	0:47:40	40	74.6930524	69.3812876	72.731787	74.2395153
17:49:41	0:47:41	41	73.6930524	69.3812876	72.731787	72.2395153
17:49:42	0:47:42	42	73.6930524	69.3812876	72.731787	72.2395153
17:49:43	0:47:43	43	73.6930524	69.3812876	72.731787	72.2395153
17:49:44	0:47:44	44	73.6930524	69.3812876	73.731787	72.2395153
17:49:45	0:47:45	45	73.6930524	70.3812876	74.731787	70.2395153
17:49:46	0:47:46	46	73.6930524	70.3812876	74.731787	70.2395153
17:49:47	0:47:47	47	73.6930524	71.3812876	74.731787	70.2395153
17:49:48	0:47:48	48	74.6930524	72.3812876	75.731787	72.2395153
17:49:49	0:47:49	49	74.6930524	72.3812876	74.731787	77.2395153
17:49:50	0:47:50	50	74.6930524	73.3812876	74.731787	77.2395153
17:49:51	0:47:51	51	74.6930524	73.3812876	72.731787	77.2395153
17:49:52	0:47:52	52	74.6930524	73.3812876	72.731787	77.2395153
17:49:53	0:47:53	53	75.6930524	73.3812876	72.731787	77.2395153
17:49:54	0:47:54	54	75.6930524	73.3812876	73.731787	78.2395153
17:49:55	0:47:55	55	75.6930524	73.3812876	73.731787	80.2395153
17:49:56	0:47:56	56	75.6930524	74.3812876	73.731787	80.2395153
17:49:57	0:47:57	57	75.6930524	74.3812876	73.731787	93.2395153

17:49:58	0:47:58	58	75.6930524	74.3812876	73.731787	109.239515
17:49:59	0:47:59	59	75.6930524	72.3812876	76.731787	117.239515
17:50:00	0:48:00	60	75.6930524	72.3812876	78.731787	119.239515
17:50:01	0:48:01	61	74.6930524	72.3812876	78.731787	119.239515
17:50:02	0:48:02	62	75.6930524	72.3812876	77.731787	114.239515
17:50:03	0:48:03	63	75.6930524	72.3812876	77.731787	114.239515
17:50:04	0:48:04	64	75.6930524	70.3812876	77.731787	112.239515
17:50:05	0:48:05	65	75.6930524	70.3812876	77.731787	112.239515
17:50:06	0:48:06	66	75.6930524	70.3812876	77.731787	112.239515
17:50:07	0:48:07	67	74.6930524	70.3812876	77.731787	122.239515
17:50:08	0:48:08	68	74.6930524	80.3812876	77.731787	123.239515
17:50:09	0:48:09	69	73.6930524	80.3812876	77.731787	123.239515
17:50:10	0:48:10	70	73.6930524	80.3812876	77.731787	124.239515
17:50:11	0:48:11	71	73.6930524	79.3812876	78.731787	124.239515
17:50:12	0:48:12	72	72.6930524	79.3812876	78.731787	124.239515
17:50:13	0:48:13	73	72.6930524	79.3812876	78.731787	125.239515
17:50:14	0:48:14	74	71.6930524	79.3812876	78.731787	126.239515
17:50:15	0:48:15	75	71.6930524	80.3812876	78.731787	115.239515
17:50:16	0:48:16	76	70.6930524	80.3812876	78.731787	106.239515
17:50:17	0:48:17	77	69.6930524	80.3812876	76.731787	101.239515
17:50:18	0:48:18	78	69.6930524	80.3812876	75.731787	99.2395153
17:50:19	0:48:19	79	68.6930524	82.3812876	75.731787	98.2395153
17:50:20	0:48:20	80	68.6930524	83.3812876	74.731787	98.2395153
17:50:21	0:48:21	81	67.6930524	83.3812876	76.731787	98.2395153
17:50:22	0:48:22	82	67.6930524	83.3812876	76.731787	98.2395153
17:50:23	0:48:23	83	67.6930524	83.3812876	76.731787	98.2395153
17:50:24	0:48:24	84	68.6930524	83.3812876	76.731787	93.2395153
17:50:25	0:48:25	85	68.6930524	83.3812876	76.731787	91.2395153
17:50:26	0:48:26	86	68.6930524	82.3812876	77.731787	91.2395153
17:50:27	0:48:27	87	69.6930524	82.3812876	76.731787	90.2395153
17:50:28	0:48:28	88	69.6930524	82.3812876	77.731787	90.2395153
17:50:29	0:48:29	89	69.6930524	79.3812876	77.731787	88.2395153
17:50:30	0:48:30	90	70.6930524	75.3812876	76.731787	87.2395153
17:50:31	0:48:31	91	70.6930524	70.3812876	76.731787	88.2395153
17:50:32	0:48:32	92	71.6930524	70.3812876	76.731787	88.2395153
17:50:33	0:48:33	93	73.6930524	70.3812876	76.731787	88.2395153
17:50:34	0:48:34	94	73.6930524	70.3812876	76.731787	88.2395153
17:50:35	0:48:35	95	74.6930524	68.3812876	76.731787	88.2395153
17:50:36	0:48:36	96	75.6930524	69.3812876	76.731787	89.2395153
17:50:37	0:48:37	97	79.6930524	69.3812876	75.731787	90.2395153
17:50:38	0:48:38	98	80.6930524	69.3812876	74.731787	90.2395153
17:50:39	0:48:39	99	80.6930524	71.3812876	74.731787	89.2395153
17:50:40	0:48:40	100	80.6930524	71.3812876	74.731787	89.2395153
17:50:41	0:48:41	101	80.6930524	71.3812876	74.731787	89.2395153
17:50:42	0:48:42	102	80.6930524	71.3812876	74.731787	89.2395153

17:50:43	0:48:43	103	80.6930524	71.3812876	75.731787	89.2395153
17:50:44	0:48:44	104	79.6930524	70.3812876	75.731787	89.2395153
17:50:45	0:48:45	105	79.6930524	70.3812876	76.731787	89.2395153
17:50:46	0:48:46	106	78.6930524	69.3812876	76.731787	88.2395153
17:50:47	0:48:47	107	78.6930524	69.3812876	77.731787	88.2395153
17:50:48	0:48:48	108	78.6930524	69.3812876	77.731787	88.2395153
17:50:49	0:48:49	109	78.6930524	68.3812876	77.731787	87.2395153
17:50:50	0:48:50	110	78.6930524	68.3812876	77.731787	86.2395153
17:50:51	0:48:51	111	78.6930524	68.3812876	77.731787	85.2395153
17:50:52	0:48:52	112	78.6930524	68.3812876	79.731787	85.2395153
17:50:53	0:48:53	113	76.6930524	68.3812876	81.731787	85.2395153
17:50:54	0:48:54	114	76.6930524	68.3812876	81.731787	85.2395153
17:50:55	0:48:55	115	76.6930524	68.3812876	83.731787	85.2395153
17:50:56	0:48:56	116	75.6930524	68.3812876	83.731787	86.2395153
17:50:57	0:48:57	117	75.6930524	67.3812876	84.731787	86.2395153
17:50:58	0:48:58	118	74.6930524	68.3812876	84.731787	86.2395153
17:50:59	0:48:59	119	74.6930524	67.3812876	83.731787	88.2395153
17:51:00	0:49:00	120	74.6930524	67.3812876	83.731787	88.2395153
17:51:01	0:49:01	121	74.6930524	65.3812876	82.731787	88.2395153
17:51:02	0:49:02	122	74.6930524	65.3812876	82.731787	86.2395153
17:51:03	0:49:03	123	74.6930524	65.3812876	81.731787	86.2395153
17:51:04	0:49:04	124	74.6930524	65.3812876	80.731787	86.2395153
17:51:05	0:49:05	125	74.6930524	65.3812876	81.731787	86.2395153
17:51:06	0:49:06	126	74.6930524	65.3812876	84.731787	86.2395153
17:51:07	0:49:07	127	74.6930524	66.3812876	88.731787	86.2395153
17:51:08	0:49:08	128	73.6930524	66.3812876	88.731787	86.2395153
17:51:09	0:49:09	129	73.6930524	66.3812876	86.731787	83.2395153
17:51:10	0:49:10	130	72.6930524	66.3812876	82.731787	82.2395153
17:51:11	0:49:11	131	72.6930524	66.3812876	79.731787	82.2395153
17:51:12	0:49:12	132	74.6930524	66.3812876	78.731787	81.2395153
17:51:13	0:49:13	133	74.6930524	66.3812876	77.731787	80.2395153
17:51:14	0:49:14	134	75.6930524	67.3812876	77.731787	72.2395153
17:51:15	0:49:15	135	75.6930524	67.3812876	78.731787	68.2395153
17:51:16	0:49:16	136	76.6930524	67.3812876	80.731787	67.2395153
17:51:17	0:49:17	137	77.6930524	67.3812876	81.731787	66.2395153
17:51:18	0:49:18	138	79.6930524	69.3812876	81.731787	65.2395153
17:51:19	0:49:19	139	80.6930524	68.3812876	81.731787	65.2395153
17:51:20	0:49:20	140	81.6930524	68.3812876	82.731787	66.2395153
17:51:21	0:49:21	141	81.6930524	69.3812876	82.731787	67.2395153
17:51:22	0:49:22	142	84.6930524	69.3812876	83.731787	69.2395153
17:51:23	0:49:23	143	87.6930524	70.3812876	85.731787	68.2395153
17:51:24	0:49:24	144	87.6930524	71.3812876	87.731787	68.2395153
17:51:25	0:49:25	145	89.6930524	71.3812876	87.731787	68.2395153
17:51:26	0:49:26	146	91.6930524	71.3812876	87.731787	69.2395153
17:51:27	0:49:27	147	92.6930524	71.3812876	88.731787	69.2395153

17:51:28	0:49:28	148	91.6930524	71.3812876	88.731787	69.2395153
17:51:29	0:49:29	149	91.6930524	71.3812876	87.731787	69.2395153
17:51:30	0:49:30	150	83.6930524	71.3812876	83.731787	69.2395153
17:51:31	0:49:31	151	78.6930524	73.3812876	83.731787	70.2395153
17:51:32	0:49:32	152	77.6930524	73.3812876	83.731787	70.2395153
17:51:33	0:49:33	153	77.6930524	73.3812876	83.731787	73.2395153
17:51:34	0:49:34	154	77.6930524	73.3812876	83.731787	73.2395153
17:51:35	0:49:35	155	77.6930524	73.3812876	83.731787	76.2395153
17:51:36	0:49:36	156	77.6930524	73.3812876	83.731787	76.2395153
17:51:37	0:49:37	157	77.6930524	73.3812876	83.731787	76.2395153
17:51:38	0:49:38	158	77.6930524	73.3812876	80.731787	76.2395153
17:51:39	0:49:39	159	77.6930524	73.3812876	77.731787	73.2395153
17:51:40	0:49:40	160	76.6930524	73.3812876	76.731787	72.2395153
17:51:41	0:49:41	161	73.6930524	73.3812876	75.731787	72.2395153
17:51:42	0:49:42	162	73.6930524	73.3812876	74.731787	74.2395153
17:51:43	0:49:43	163	73.6930524	72.3812876	73.731787	74.2395153
17:51:44	0:49:44	164	73.6930524	72.3812876	73.731787	75.2395153
17:51:45	0:49:45	165	72.6930524	72.3812876	73.731787	75.2395153
17:51:46	0:49:46	166	72.6930524	72.3812876	72.731787	75.2395153
17:51:47	0:49:47	167	72.6930524	72.3812876	72.731787	76.2395153
17:51:48	0:49:48	168	72.6930524	73.3812876	73.731787	76.2395153
17:51:49	0:49:49	169	73.6930524	73.3812876	73.731787	75.2395153
17:51:50	0:49:50	170	76.6930524	73.3812876	74.731787	75.2395153
17:51:51	0:49:51	171	76.6930524	73.3812876	74.731787	74.2395153
17:51:52	0:49:52	172	79.6930524	73.3812876	74.731787	74.2395153
17:51:53	0:49:53	173	79.6930524	73.3812876	74.731787	74.2395153
17:51:54	0:49:54	174	79.6930524	73.3812876	74.731787	74.2395153
17:51:55	0:49:55	175	78.6930524	74.3812876	75.731787	75.2395153
17:51:56	0:49:56	176	78.6930524	74.3812876	75.731787	75.2395153
17:51:57	0:49:57	177	78.6930524	74.3812876	75.731787	76.2395153
17:51:58	0:49:58	178	78.6930524	75.3812876	75.731787	75.2395153
17:51:59	0:49:59	179	77.6930524	75.3812876	74.731787	75.2395153
17:52:00	0:50:00	180	77.6930524	75.3812876	74.731787	75.2395153

Person 8	Person 2	Person 9	Person 10	behavior	average	annotation
79.8457846	81.9030219			69	82.6324081	
78.8457846	83.9030219		141.26905	69	91.5804998	
78.8457846	83.9030219		141.26905	69	91.0090713	
84.8457846	83.9030219		140.26905	69	91.723357	
83.8457846	83.9030219		140.26905	69	91.5804998	
82.8457846	83.9030219		140.26905	69	91.8662141	
82.8457846	84.9030219	75.3551261	140.26905	69	89.8023281	
82.8457846	84.9030219	75.3551261	140.26905	69	89.8023281	
82.8457846	84.9030219	77.3551261	140.26905	69	90.0523281	
83.8457846	84.9030219	77.3551261	140.26905	69	90.0523281	
83.8457846	84.9030219	77.3551261	139.26905	69	90.0523281	
83.8457846	84.9030219	77.3551261	139.26905	69	90.3023281	
84.8457846	83.9030219	76.3551261	139.26905	69	90.5523281	
87.8457846	81.9030219	75.3551261	138.26905	69	90.6773281	
87.8457846	81.9030219	75.3551261	137.26905	69	90.6773281	
87.8457846	80.9030219	75.3551261	136.26905	69	90.0523281	
87.8457846	80.9030219	75.3551261	135.26905	69	89.4273281	
87.8457846	80.9030219	76.3551261	135.26905	69	89.5523281	
87.8457846	80.9030219	76.3551261	133.26905	69	89.1773281	
86.8457846	82.9030219	77.3551261	132.26905	69	89.3023281	
86.8457846	83.9030219	77.3551261	131.26905	69	88.9273281	
86.8457846	84.9030219	77.3551261	131.26905	69	89.0523281	
85.8457846	85.9030219	77.3551261	131.26905	69	88.8023281	
82.8457846	86.9030219	77.3551261	131.26905	69	88.6773281	
82.8457846	86.9030219	77.3551261	131.26905	69	88.6773281	
81.8457846	91.9030219	77.3551261	131.26905	69	89.4273281	
81.8457846	91.9030219	77.3551261	127.26905	69	89.3023281	
82.8457846	92.9030219	75.3551261	126.26905	69	89.4273281	
82.8457846	92.9030219	75.3551261	126.26905	69	89.9273281	
82.8457846	92.9030219	75.3551261	126.26905	69	90.5523281	
82.8457846	93.9030219	73.3551261	125.26905	69	90.3023281	
81.8457846	93.9030219	74.3551261	125.26905	69	90.8023281	
81.8457846	93.9030219	74.3551261	125.26905	69	90.9273281	
81.8457846	93.9030219	74.3551261	124.26905	69	90.9273281	
82.8457846	93.9030219	74.3551261	124.26905	69	90.5523281	
82.8457846	93.9030219	74.3551261	124.26905	69	90.1773281	
82.8457846	93.9030219	74.3551261	124.26905	69	89.9273281	
84.8457846	93.9030219	74.3551261	122.26905	69	89.8023281	
86.8457846	93.9030219	74.3551261	122.26905	69	89.4273281	
86.8457846	91.9030219	74.3551261	121.26905	69	89.0523281	
90.8457846	90.9030219	74.3551261	120.26905	69	88.9273281	
90.8457846	89.9030219	74.3551261	118.26905	69	88.5523281	
90.8457846	87.9030219	74.3551261	115.26905	69	89.1773281	

85.8457846	87.9030219	74.3551261	115.26905	69	87.4273281	
79.8457846	87.9030219	74.3551261	112.26905	69	86.1773281	
74.8457846	87.9030219	74.3551261	112.26905	69	85.4273281	
74.8457846	87.9030219	73.3551261	112.26905	69	84.4273281	
74.8457846	87.9030219	73.3551261	111.26905	69	83.9273281	
78.8457846	86.9030219	73.3551261	111.26905	69	84.3023281	
78.8457846	85.9030219	73.3551261	111.26905	69	83.9273281	
81.8457846	83.9030219	71.3551261	111.26905	69	84.4273281	
84.8457846	83.9030219	71.3551261	111.26905	69	85.1773281	
84.8457846	82.9030219	72.3551261	111.26905	69	85.6773281	
82.8457846	81.9030219	72.3551261	111.26905	69	85.3023281	
82.8457846	81.9030219	72.3551261	111.26905	69	86.4273281	
82.8457846	73.9030219	72.3551261	111.26905	69	86.6773281	
78.8457846	73.9030219	72.3551261	109.26905	69	86.1773281	
78.8457846	73.9030219	71.3551261	108.26905	69	86.1773281	
78.8457846	73.9030219	71.3551261	107.26905	69	84.9273281	
78.8457846	73.9030219	72.3551261	107.26905	69	85.0523281	
78.8457846	73.9030219	72.3551261	107.26905	69	84.9273281	
78.8457846	73.9030219	71.3551261	107.26905	69	84.4273281	
77.8457846	71.9030219	71.3551261	108.26905	69	84.0523281	
78.8457846	70.9030219	71.3551261	105.26905	69	83.4273281	
79.8457846	70.9030219	70.3551261	101.26905	69	83.3023281	
79.8457846	70.9030219	70.3551261	101.26905	69	83.5523281	
79.8457846	70.9030219	70.3551261	100.26905	69	83.4273281	
79.8457846	70.9030219	70.3551261	100.26905	69	82.9273281	
78.8457846	70.9030219	70.3551261	100.26905	69	82.8023281	
78.8457846	70.9030219	70.3551261	100.26905	69	82.6773281	
78.8457846	70.9030219	70.3551261	100.26905	69	82.8023281	
79.8457846	69.9030219	69.3551261	103.26905	69	82.5523281	
80.8457846	73.9030219	69.3551261	103.26905	69	83.1773281	
80.8457846	73.9030219	69.3551261	103.26905	69	82.8023281	
79.8457846	74.9030219	69.3551261	103.26905	69	82.8023281	
78.8457846	74.9030219	69.3551261	95.2690501	69	80.9273281	
78.8457846	74.9030219	69.3551261	94.2690501	69	80.6773281	
78.8457846	74.9030219	69.3551261	93.2690501	69	80.3023281	
78.8457846	74.9030219	69.3551261	93.2690501	69	80.3023281	
79.8457846	72.9030219	69.3551261	94.2690501	69	80.3023281	
79.8457846	68.9030219	70.3551261	94.2690501	69	79.1773281	
81.8457846	65.9030219	71.3551261	93.2690501	69	79.4273281	
83.8457846	64.9030219	72.3551261	93.2690501	69	79.3023281	
83.8457846	64.9030219	72.3551261	93.2690501	69	79.5523281	
82.8457846	64.9030219	73.3551261	92.2690501	63	79.5523281	
81.8457846	65.9030219	73.3551261	92.2690501	63	79.4273281	
82.8457846	66.9030219	72.3551261	92.2690501	63	78.6773281	
82.8457846	67.9030219	72.3551261	92.2690501	63	78.9273281	

84.8457846	67.9030219	72.3551261	93.2690501	63	78.9273281	
85.8457846	67.9030219	72.3551261	93.2690501	63	78.9273281	
86.8457846	67.9030219	72.3551261	93.2690501	63	78.8023281	
85.8457846	67.9030219	72.3551261	92.2690501	63	78.4273281	
84.8457846	67.9030219	73.3551261	91.2690501	63	78.4273281	
83.8457846	67.9030219	74.3551261	91.2690501	63	78.1773281	
83.8457846	67.9030219	74.3551261	91.2690501	63	78.0523281	
84.8457846	68.9030219	75.3551261	91.2690501	63	78.8023281	
85.8457846	67.9030219	75.3551261	94.2690501	63	78.9273281	
86.8457846	67.9030219	75.3551261	95.2690501	63	79.0523281	
86.8457846	67.9030219	75.3551261	96.2690501	63	79.0523281	
87.8457846	67.9030219	77.3551261	94.2690501	63	79.3023281	
87.8457846	67.9030219	78.3551261	94.2690501	63	79.5523281	
89.8457846	68.9030219	79.3551261	92.2690501	63	79.8023281	
88.8457846	68.9030219	79.3551261	89.2690501	63	79.1773281	
87.8457846	69.9030219	80.3551261	88.2690501	63	79.3023281	
88.8457846	69.9030219	80.3551261	89.2690501	63	79.5523281	
88.8457846	69.9030219	80.3551261	90.2690501	63	79.5523281	
89.8457846	69.9030219	79.3551261	90.2690501	63	79.4273281	
89.8457846	69.9030219	76.3551261	90.2690501	63	79.0523281	
88.8457846	71.9030219	76.3551261	90.2690501	63	79.1773281	
85.8457846	71.9030219	73.3551261	90.2690501	63	78.5523281	
85.8457846	72.9030219	72.3551261	88.2690501	63	78.8023281	
85.8457846	74.9030219	72.3551261	86.2690501	63	78.9273281	
85.8457846	74.9030219	72.3551261	86.2690501	63	79.1773281	
86.8457846	75.9030219	71.3551261	86.2690501	63	79.4273281	
88.8457846	75.9030219	67.3551261	85.2690501	63	79.5523281	
88.8457846	75.9030219	67.3551261	85.2690501	63	79.5523281	
88.8457846	75.9030219	66.3551261	84.2690501	63	79.1773281	
88.8457846	75.9030219	66.3551261	84.2690501	63	79.3023281	
82.8457846	75.9030219	67.3551261	83.2690501	63	78.8023281	
80.8457846	79.9030219	75.3551261	84.2690501	63	80.1773281	
80.8457846	79.9030219	75.3551261	82.2690501	63	80.1773281	
80.8457846	77.9030219	75.3551261	82.2690501	63	80.4273281	
80.8457846	72.9030219	75.3551261	82.2690501	63	80.0523281	
81.8457846	70.9030219	75.3551261	82.2690501	63	80.0523281	
83.8457846	70.9030219	77.3551261	83.2690501	63	80.6773281	
83.8457846	67.9030219	80.3551261	85.2690501	63	80.8023281	
83.8457846	66.9030219	80.3551261	85.2690501	63	80.3023281	
83.8457846	66.9030219	80.3551261	86.2690501	63	80.5523281	
84.8457846	66.9030219	80.3551261	86.2690501	63	80.8023281	
84.8457846	66.9030219	78.3551261	87.2690501	63	80.6773281	
84.8457846	66.9030219	74.3551261	87.2690501	63	79.8023281	
85.8457846	65.9030219	72.3551261	87.2690501	63	79.6773281	
85.8457846	65.9030219	72.3551261	87.2690501	63	79.6773281	

85.8457846	64.9030219	71.3551261	87.2690501	63	79.4273281	
85.8457846	64.9030219	71.3551261	87.2690501	63	79.4273281	
85.8457846	63.9030219	71.3551261	89.2690501	63	79.5523281	
89.8457846	63.9030219	71.3551261	89.2690501	63	80.1773281	
89.8457846	63.9030219	71.3551261	89.2690501	63	80.4273281	
89.8457846	63.9030219	69.3551261	89.2690501	63	79.9273281	
90.8457846	63.9030219	69.3551261	88.2690501	63	79.6773281	
91.8457846	62.9030219	68.3551261	88.2690501	63	79.3023281	
92.8457846	62.9030219	68.3551261	87.2690501	63	79.4273281	
92.8457846	62.9030219	64.3551261	87.2690501	63	78.9273281	
92.8457846	62.9030219	58.3551261	87.2690501	63	78.0523281	
93.8457846	62.9030219	58.3551261	87.2690501	63	77.9273281	
93.8457846	62.9030219	58.3551261	88.2690501	63	77.9273281	
93.8457846	62.9030219	58.3551261	90.2690501	63	78.3023281	
93.8457846	62.9030219	57.3551261	90.2690501	63	78.3023281	
93.8457846	62.9030219	56.3551261	90.2690501	63	78.1773281	
94.8457846	65.9030219	56.3551261	92.2690501	63	78.9273281	
94.8457846	65.9030219	55.3551261	92.2690501	63	79.1773281	
95.8457846	65.9030219	54.3551261	91.2690501	63	79.5523281	
95.8457846	64.9030219	51.3551261	90.2690501	63	78.8023281	
95.8457846	64.9030219	51.3551261	89.2690501	63	78.4273281	
95.8457846	64.9030219	51.3551261	88.2690501	63	78.4273281	
95.8457846	65.9030219	51.3551261	87.2690501	63	78.3023281	
95.8457846	65.9030219	49.3551261	87.2690501	63	78.0523281	
97.8457846	65.9030219	49.3551261	86.2690501	63	78.0523281	
98.8457846	66.9030219	49.3551261	85.2690501	63	78.3023281	
98.8457846	66.9030219	45.3551261	85.2690501	63	77.8023281	
99.8457846	65.9030219	45.3551261	85.2690501	63	77.8023281	
100.845785	65.9030219	45.3551261	85.2690501	63	77.9273281	
100.845785	65.9030219	45.3551261	85.2690501	63	77.8023281	
100.845785	65.9030219	45.3551261	85.2690501	63	77.8023281	
98.8457846	65.9030219	45.3551261	85.2690501	63	77.4273281	
98.8457846	65.9030219	45.3551261	86.2690501	63	77.4273281	
98.8457846	65.9030219	45.3551261	89.2690501	63	77.8023281	
98.8457846	67.9030219	45.3551261	91.2690501	63	78.3023281	
98.8457846	67.9030219	45.3551261	91.2690501	63	78.1773281	
98.8457846	66.9030219	45.3551261	91.2690501	63	78.3023281	
98.8457846	66.9030219	43.3551261	91.2690501	63	78.0523281	
98.8457846	66.9030219	43.3551261	90.2690501	63	77.9273281	
98.8457846	66.9030219	43.3551261	87.2690501	63	77.4273281	
98.8457846	66.9030219	43.3551261	87.2690501	63	77.5523281	
97.8457846	66.9030219	43.3551261	87.2690501	63	77.6773281	
96.8457846	66.9030219	43.3551261	86.2690501	63	77.8023281	
95.8457846	68.9030219	43.3551261	88.2690501	63	78.0523281	
95.8457846	68.9030219	44.3551261	88.2690501	63	78.6773281	

95.8457846	68.9030219	44.3551261	89.2690501	63	79.1773281	
96.8457846	68.9030219	44.3551261	90.2690501	63	79.4273281	
95.8457846	68.9030219	44.3551261	90.2690501	63	78.6773281	
95.8457846	68.9030219	44.3551261	90.2690501	63	78.9273281	
95.8457846	68.9030219	44.3551261	90.2690501	63	78.8023281	
95.8457846	69.9030219	44.3551261	90.2690501	63	79.0523281	
96.8457846	69.9030219	44.3551261	90.2690501	63	79.1773281	
96.8457846	69.9030219	49.3551261	91.2690501	63	79.8023281	
96.8457846	69.9030219	49.3551261	91.2690501	63	79.8023281	
97.8457846	69.9030219	54.3551261	89.2690501	63	80.5523281	
96.8457846	69.9030219	55.3551261	88.2690501	63	80.0523281	
96.8457846	69.9030219	57.3551261	88.2690501	63	80.3023281	
97.8457846	69.9030219	61.3551261	88.2690501	63	80.9273281	
97.8457846	69.9030219	62.3551261	88.2690501	63	81.1773281	
97.8457846	68.9030219	62.3551261	90.2690501	63	81.4273281	
97.8457846	68.9030219	68.3551261	91.2690501	63	81.9273281	
98.8457846	69.9030219	67.3551261	91.2690501	63	82.4273281	
98.8457846	70.9030219	67.3551261	91.2690501	63	82.8023281	
98.8457846	71.9030219	67.3551261	91.2690501	63	82.6773281	
98.8457846	71.9030219	67.3551261	91.2690501	63	82.9273281	
98.8457846	71.9030219	68.3551261	90.2690501	63	82.6773281	
95.8457846	70.9030219	68.3551261	90.2690501	63	81.8023281	
95.8457846	69.9030219	69.3551261	91.2690501	63	81.8023281	
94.8457846	69.9030219	69.3551261	91.2690501	63	81.5523281	
93.8457846	68.9030219	69.3551261	90.2690501	63	81.0523281	
93.8457846	68.9030219	69.3551261	89.2690501	63	81.1773281	
87.8457846	69.9030219	69.3551261	88.2690501	63	80.4273281	
87.8457846	69.9030219	67.3551261	86.2690501	63	80.6773281	
87.8457846	69.9030219	66.3551261	85.2690501	63	80.5523281	
87.8457846	69.9030219	66.3551261	85.2690501	63	80.4273281	
86.8457846	69.9030219	67.3551261	85.2690501	63	80.3023281	
86.8457846	69.9030219	67.3551261	85.2690501	63	80.0523281	
85.8457846	69.9030219	67.3551261	85.2690501	63	80.0523281	
86.8457846	69.9030219	68.3551261	84.2690501	63	79.8023281	
88.8457846	70.9030219	68.3551261	85.2690501	63	80.3023281	
88.8457846	70.9030219	68.3551261	86.2690501	63	80.4273281	
90.8457846	69.9030219	70.3551261	87.2690501	63	81.0523281	
90.8457846	69.9030219	71.3551261	87.2690501	63	81.3023281	
91.8457846	70.9030219	71.3551261	87.2690501	63	81.3023281	
91.8457846	70.9030219	72.3551261	87.2690501	63	81.1773281	
90.8457846	70.9030219	73.3551261	88.2690501	63	81.0523281	
90.8457846	70.9030219	73.3551261	88.2690501	63	80.9273281	
90.8457846	70.9030219	73.3551261	88.2690501	63	80.3023281	
90.8457846	70.9030219	73.3551261	89.2690501	63	81.3023281	
89.8457846	69.9030219	73.3551261	89.2690501	63	80.9273281	

88.8457846	69.9030219	73.3551261	89.2690501	63	81.1773281	
87.8457846	69.9030219	73.3551261	88.2690501	63	80.8023281	
87.8457846	69.9030219	74.3551261	87.2690501	63	80.6773281	
87.8457846	69.9030219	74.3551261	87.2690501	63	80.8023281	
87.8457846	70.9030219	73.3551261	87.2690501	63	80.5523281	
88.8457846	71.9030219	72.3551261	87.2690501	63	80.5523281	
88.8457846	71.9030219	72.3551261	88.2690501	63	80.6773281	
88.8457846	71.9030219	72.3551261	88.2690501	63	80.8023281	
86.8457846	71.9030219	71.3551261	88.2690501	63	80.3023281	
85.8457846	71.9030219	72.3551261	86.2690501	63	80.1773281	
84.8457846	71.9030219	72.3551261	86.2690501	63	80.0523281	
84.8457846	71.9030219	72.3551261	86.2690501	63	79.9273281	
83.8457846	71.9030219	72.3551261	85.2690501	63	80.3023281	
83.8457846	71.9030219	72.3551261	84.2690501	63	79.8023281	
83.8457846	71.9030219	72.3551261	84.2690501	63	79.3023281	
84.8457846	71.9030219	73.3551261	84.2690501	63	79.3023281	
84.8457846	72.9030219	72.3551261	83.2690501	63	79.3023281	
84.8457846	72.9030219	72.3551261	83.2690501	63	79.3023281	
85.8457846	82.9030219	74.3551261	83.2690501	63	80.9273281	
86.8457846	82.9030219	74.3551261	83.2690501	63	81.0523281	
87.8457846	82.9030219	75.3551261	83.2690501	63	80.9273281	
87.8457846	82.9030219	75.3551261	84.2690501	63	81.0523281	
87.8457846	76.9030219	75.3551261	85.2690501	63	80.0523281	
87.8457846	76.9030219	75.3551261	86.2690501	63	80.1773281	
87.8457846	73.9030219	76.3551261	86.2690501	63	79.8023281	
87.8457846	71.9030219	75.3551261	86.2690501	63	79.3023281	
86.8457846	70.9030219	74.3551261	86.2690501	63	78.9273281	
82.8457846	70.9030219	72.3551261	86.2690501	63	78.5523281	
80.8457846	70.9030219	72.3551261	86.2690501	63	77.9273281	
77.8457846	70.9030219	72.3551261	85.2690501	63	77.1773281	
75.8457846	70.9030219	71.3551261	85.2690501	63	76.8023281	
75.8457846	69.9030219	70.3551261	86.2690501	63	76.5523281	
75.8457846	69.9030219	69.3551261	86.2690501	63	76.8023281	
75.8457846	69.9030219	69.3551261	86.2690501	63	76.9273281	
75.8457846	69.9030219	69.3551261	86.2690501	63	76.8023281	
75.8457846	69.9030219	69.3551261	85.2690501	63	76.6773281	
74.8457846	69.9030219	71.3551261	84.2690501	63	77.0523281	
74.8457846	69.9030219	71.3551261	84.2690501	63	77.3023281	
74.8457846	69.9030219	71.3551261	84.2690501	63	77.3023281	
74.8457846	69.9030219	71.3551261	84.2690501	63	77.3023281	
74.8457846	68.9030219	71.3551261	84.2690501	63	77.3023281	
74.8457846	68.9030219	71.3551261	85.2690501	63	77.6773281	
74.8457846	68.9030219	72.3551261	85.2690501	63	77.8023281	
74.8457846	68.9030219	73.3551261	86.2690501	63	78.0523281	
76.8457846	69.9030219	73.3551261	86.2690501	63	78.6773281	

76.8457846	69.9030219	74.3551261	85.2690501	63	78.5523281	
76.8457846	71.9030219	76.3551261	85.2690501	63	78.8023281	
76.8457846	69.9030219	76.3551261	84.2690501	63	78.4273281	
75.8457846	68.9030219	77.3551261	84.2690501	63	78.1773281	
73.8457846	68.9030219	77.3551261	84.2690501	63	77.6773281	
73.8457846	66.9030219	79.3551261	84.2690501	63	77.4273281	
73.8457846	64.9030219	79.3551261	85.2690501	63	76.5523281	
73.8457846	64.9030219	79.3551261	85.2690501	63	76.3023281	
72.8457846	64.9030219	75.3551261	85.2690501	63	76.0523281	
72.8457846	65.9030219	73.3551261	84.2690501	63	75.6773281	
72.8457846	70.9030219	73.3551261	83.2690501	63	76.1773281	
72.8457846	70.9030219	72.3551261	83.2690501	63	76.1773281	
74.8457846	72.9030219	71.3551261	83.2690501	63	76.5523281	
74.8457846	73.9030219	71.3551261	83.2690501	63	76.8023281	
74.8457846	73.9030219	71.3551261	82.2690501	63	76.4273281	
74.8457846	73.9030219	70.3551261	82.2690501	63	76.3023281	
75.8457846	72.9030219	70.3551261	81.2690501	63	76.4273281	
75.8457846	70.9030219	70.3551261	81.2690501	63	76.8023281	
74.8457846	69.9030219	70.3551261	85.2690501	63	77.1773281	
70.8457846	69.9030219	70.3551261	85.2690501	63	76.9273281	
66.8457846	70.9030219	70.3551261	85.2690501	63	76.3023281	
64.8457846	70.9030219	70.3551261	85.2690501	63	76.4273281	
64.8457846	70.9030219	70.3551261	84.2690501	63	76.1773281	
68.8457846	70.9030219	71.3551261	84.2690501	63	76.1773281	
70.8457846	70.9030219	72.3551261	84.2690501	63	76.6773281	
70.8457846	70.9030219	72.3551261	84.2690501	63	76.9273281	
70.8457846	70.9030219	72.3551261	84.2690501	63	76.9273281	
70.8457846	69.9030219	74.3551261	84.2690501	63	77.5523281	
71.8457846	68.9030219	74.3551261	84.2690501	63	77.4273281	
72.8457846	68.9030219	74.3551261	84.2690501	63	77.1773281	
73.8457846	68.9030219	74.3551261	84.2690501	63	77.5523281	
73.8457846	68.9030219	74.3551261	83.2690501	63	77.4273281	
73.8457846	69.9030219	74.3551261	83.2690501	63	77.5523281	
71.8457846	70.9030219	74.3551261	85.2690501	63	77.6773281	
70.8457846	70.9030219	75.3551261	87.2690501	63	77.9273281	
69.8457846	71.9030219	75.3551261	87.2690501	63	77.8023281	
69.8457846	71.9030219	75.3551261	86.2690501	63	77.9273281	
71.8457846	72.9030219	75.3551261	84.2690501	63	78.4273281	
75.8457846	72.9030219	75.3551261	84.2690501	63	78.8023281	
75.8457846	72.9030219	75.3551261	83.2690501	63	78.4273281	
75.8457846	72.9030219	76.3551261	83.2690501	63	77.9273281	
75.8457846	72.9030219	77.3551261	83.2690501	63	77.8023281	
75.8457846	74.9030219	77.3551261	83.2690501	63	78.1773281	
75.8457846	75.9030219	77.3551261	83.2690501	63	78.1773281	
75.8457846	75.9030219	79.3551261	87.2690501	63	79.1773281	

75.8457846	75.9030219	79.3551261	87.2690501	63	79.4273281	
75.8457846	75.9030219	77.3551261	89.2690501	63	79.4273281	
74.8457846	75.9030219	76.3551261	89.2690501	63	79.6773281	
73.8457846	75.9030219	74.3551261	88.2690501	63	79.4273281	
73.8457846	73.9030219	74.3551261	86.2690501	63	79.3023281	
73.8457846	72.9030219	74.3551261	86.2690501	63	79.4273281	
74.8457846	72.9030219	74.3551261	84.2690501	63	79.0523281	
74.8457846	72.9030219	74.3551261	84.2690501	63	78.8023281	
74.8457846	72.9030219	74.3551261	84.2690501	63	78.6773281	
71.8457846	72.9030219	74.3551261	85.2690501	63	78.5523281	
70.8457846	72.9030219	74.3551261	86.2690501	63	78.5523281	
70.8457846	73.9030219	74.3551261	86.2690501	63	78.8023281	
70.8457846	73.9030219	74.3551261	83.2690501	63	78.8023281	
70.8457846	73.9030219	75.3551261	82.2690501	63	78.8023281	
70.8457846	75.9030219	75.3551261	82.2690501	63	79.0523281	
70.8457846	75.9030219	75.3551261	82.2690501	63	79.1773281	
70.8457846	75.9030219	74.3551261	83.2690501	63	79.0523281	
71.8457846	74.9030219	74.3551261	83.2690501	63	79.0523281	
80.8457846	72.9030219	74.3551261	83.2690501	63	79.6773281	
83.8457846	71.9030219	74.3551261	84.2690501	63	80.0523281	
83.8457846	71.9030219	74.3551261	84.2690501	63	80.0523281	
82.8457846	70.9030219	74.3551261	85.2690501	63	80.1773281	
82.8457846	70.9030219	74.3551261	85.2690501	63	80.3023281	
83.8457846	70.9030219	73.3551261	85.2690501	63	80.4273281	
83.8457846	70.9030219	72.3551261	84.2690501	63	80.8023281	
84.8457846	70.9030219	71.3551261	84.2690501	63	81.4273281	
85.8457846	70.9030219	71.3551261	84.2690501	63	81.6773281	
84.8457846	70.9030219	71.3551261	83.2690501	63	81.3023281	
83.8457846	69.9030219	71.3551261	83.2690501	63	81.0523281	
79.8457846	69.9030219	71.3551261	83.2690501	63	80.3023281	
77.8457846	68.9030219	72.3551261	83.2690501	63	79.6773281	
77.8457846	68.9030219	72.3551261	83.2690501	63	79.5523281	
77.8457846	68.9030219	72.3551261	83.2690501	63	79.6773281	
77.8457846	68.9030219	72.3551261	83.2690501	63	79.6773281	
77.8457846	68.9030219	73.3551261	84.2690501	63	80.0523281	
77.8457846	68.9030219	73.3551261	84.2690501	63	80.0523281	
77.8457846	68.9030219	73.3551261	84.2690501	63	80.8023281	
76.8457846	69.9030219	73.3551261	84.2690501	63	80.9273281	
75.8457846	70.9030219	73.3551261	84.2690501	63	80.6773281	
69.8457846	70.9030219	73.3551261	85.2690501	63	79.9273281	
69.8457846	70.9030219	73.3551261	86.2690501	63	79.8023281	
69.8457846	70.9030219	73.3551261	86.2690501	63	79.5523281	
69.8457846	70.9030219	73.3551261	85.2690501	63	79.1773281	
69.8457846	70.9030219	73.3551261	86.2690501	63	79.3023281	
69.8457846	70.9030219	73.3551261	86.2690501	63	79.0523281	

70.8457846	70.9030219	73.3551261	89.2690501	63	79.1773281	
72.8457846	70.9030219	73.3551261	89.2690501	63	79.1773281	
72.8457846	70.9030219	73.3551261	88.2690501	63	78.8023281	
75.8457846	70.9030219	74.3551261	88.2690501	63	79.0523281	
74.8457846	70.9030219	75.3551261	86.2690501	63	78.5523281	
74.8457846	71.9030219	76.3551261	86.2690501	63	79.0523281	
74.8457846	73.9030219	76.3551261	87.2690501	63	79.4273281	
74.8457846	73.9030219	76.3551261	87.2690501	63	79.4273281	
73.8457846	75.9030219	75.3551261	89.2690501	63	79.6773281	
73.8457846	75.9030219	74.3551261	89.2690501	63	79.5523281	
73.8457846	75.9030219	73.3551261	91.2690501	63	79.8023281	
73.8457846	75.9030219	73.3551261	92.2690501	63	79.9273281	
73.8457846	74.9030219	72.3551261	92.2690501	63	79.4273281	
73.8457846	74.9030219	72.3551261	93.2690501	63	79.6773281	
73.8457846	73.9030219	73.3551261	93.2690501	63	79.4273281	
73.8457846	73.9030219	73.3551261	94.2690501	63	79.5523281	
73.8457846	72.9030219	75.3551261	94.2690501	63	78.8023281	
72.8457846	72.9030219	76.3551261	94.2690501	63	78.5523281	
72.8457846	72.9030219	76.3551261	95.2690501	63	78.5523281	
72.8457846	71.9030219	76.3551261	95.2690501	63	78.5523281	
70.8457846	71.9030219	76.3551261	95.2690501	63	78.0523281	
70.8457846	71.9030219	78.3551261	94.2690501	63	78.1773281	
69.8457846	71.9030219	78.3551261	94.2690501	63	78.1773281	
76.8457846	71.9030219	79.3551261	92.2690501	63	79.1773281	
76.8457846	72.9030219	79.3551261	92.2690501	63	79.4273281	
85.8457846	72.9030219	80.3551261	88.2690501	63	79.6773281	
87.8457846	71.9030219	80.3551261	87.2690501	63	79.8023281	
87.8457846	70.9030219	79.3551261	86.2690501	63	79.6773281	
87.8457846	70.9030219	79.3551261	86.2690501	63	79.9273281	
87.8457846	70.9030219	79.3551261	86.2690501	63	80.4273281	
87.8457846	70.9030219	78.3551261	89.2690501	63	80.3023281	
87.8457846	70.9030219	76.3551261	90.2690501	63	80.4273281	
86.8457846	69.9030219	76.3551261	92.2690501	63	80.4273281	
85.8457846	69.9030219	76.3551261	92.2690501	63	80.3023281	
84.8457846	68.9030219	76.3551261	93.2690501	63	79.9273281	
83.8457846	68.9030219	76.3551261	92.2690501	63	79.5523281	
81.8457846	68.9030219	76.3551261	91.2690501	63	79.3023281	
81.8457846	72.9030219	75.3551261	90.2690501	63	79.9273281	
74.8457846	72.9030219	75.3551261	90.2690501	63	79.1773281	
74.8457846	72.9030219	74.3551261	89.2690501	63	78.8023281	
73.8457846	72.9030219	73.3551261	88.2690501	63	78.3023281	
73.8457846	72.9030219	73.3551261	87.2690501	63	77.9273281	
74.8457846	72.9030219	73.3551261	88.2690501	63	78.4273281	
74.8457846	72.9030219	73.3551261	88.2690501	63	78.4273281	
74.8457846	72.9030219	73.3551261	88.2690501	63	78.4273281	

77.8457846	71.9030219	72.3551261	88.2690501	63	78.8023281	
77.8457846	71.9030219	72.3551261	88.2690501	63	78.8023281	
78.8457846	71.9030219	71.3551261	87.2690501	63	78.5523281	
80.8457846	74.9030219	70.3551261	87.2690501	63	78.9273281	
80.8457846	75.9030219	66.3551261	86.2690501	63	78.6773281	
79.8457846	75.9030219	66.3551261	86.2690501	63	78.9273281	
79.8457846	75.9030219	66.3551261	86.2690501	63	78.8023281	
80.8457846	77.9030219	68.3551261	88.2690501	63	79.6773281	
81.8457846	77.9030219	68.3551261	89.2690501	63	80.3023281	
82.8457846	77.9030219	68.3551261	90.2690501	63	82.4273281	
82.8457846	76.9030219	70.3551261	90.2690501	63	81.4273281	
82.8457846	76.9030219	70.3551261	90.2690501	63	81.4273281	
83.8457846	73.9030219	70.3551261	90.2690501	63	81.0523281	
85.8457846	72.9030219	71.3551261	90.2690501	63	81.1773281	
86.8457846	72.9030219	72.3551261	90.2690501	63	81.3023281	
87.8457846	71.9030219	73.3551261	90.2690501	63	81.9273281	
87.8457846	71.9030219	73.3551261	90.2690501	63	82.1773281	
87.8457846	71.9030219	73.3551261	90.2690501	63	82.1773281	
86.8457846	71.9030219	73.3551261	90.2690501	63	82.0523281	
86.8457846	71.9030219	73.3551261	89.2690501	63	81.9273281	
85.8457846	71.9030219	73.3551261	89.2690501	63	81.8023281	
85.8457846	72.9030219	73.3551261	89.2690501	63	81.6773281	
82.8457846	72.9030219	73.3551261	89.2690501	63	81.4273281	
82.8457846	72.9030219	74.3551261	90.2690501	63	81.4273281	
81.8457846	72.9030219	73.3551261	90.2690501	63	80.5523281	
81.8457846	73.9030219	73.3551261	90.2690501	63	80.0523281	
81.8457846	76.9030219	73.3551261	88.2690501	63	79.9273281	
81.8457846	77.9030219	73.3551261	87.2690501	63	79.9273281	
80.8457846	78.9030219	73.3551261	87.2690501	63	79.9273281	
78.8457846	78.9030219	75.3551261	86.2690501	63	79.1773281	
78.8457846	78.9030219	75.3551261	86.2690501	63	79.1773281	
78.8457846	79.9030219	75.3551261	86.2690501	63	79.4273281	
78.8457846	79.9030219	73.3551261	87.2690501	63	79.8023281	
79.8457846	79.9030219	72.3551261	86.2690501	63	80.0523281	
80.8457846	78.9030219	72.3551261	87.2690501	63	80.4273281	
80.8457846	78.9030219	72.3551261	88.2690501	63	80.4273281	
80.8457846	76.9030219	72.3551261	87.2690501	63	80.0523281	
80.8457846	75.9030219	72.3551261	87.2690501	63	79.5523281	
81.8457846	75.9030219	72.3551261	87.2690501	63	79.5523281	
84.8457846	73.9030219	72.3551261	85.2690501	63	79.5523281	
84.8457846	72.9030219	73.3551261	84.2690501	63	79.4273281	
84.8457846	72.9030219	73.3551261	84.2690501	63	79.4273281	
84.8457846	71.9030219	73.3551261	85.2690501	63	79.4273281	
84.8457846	75.9030219	73.3551261	84.2690501	63	80.3023281	
84.8457846	76.9030219	73.3551261	84.2690501	63	80.5523281	

83.8457846	76.9030219	73.3551261	84.2690501	63	80.3023281	
83.8457846	74.9030219	73.3551261	84.2690501	63	79.8023281	
83.8457846	72.9030219	73.3551261	83.2690501	63	79.1773281	
83.8457846	70.9030219	73.3551261	83.2690501	63	79.0523281	
82.8457846	68.9030219	73.3551261	83.2690501	63	78.9273281	
82.8457846	66.9030219	73.3551261	83.2690501	63	78.8023281	
82.8457846	65.9030219	73.3551261	83.2690501	63	79.0523281	
82.8457846	65.9030219	73.3551261	83.2690501	63	79.1773281	
82.8457846	65.9030219	73.3551261	83.2690501	63	79.1773281	
83.8457846	66.9030219	73.3551261	83.2690501	63	79.5523281	
84.8457846	67.9030219	73.3551261	88.2690501	63	80.4273281	
84.8457846	68.9030219	73.3551261	89.2690501	63	80.5523281	
84.8457846	69.9030219	71.3551261	89.2690501	63	79.5523281	
84.8457846	69.9030219	70.3551261	88.2690501	63	78.9273281	
83.8457846	69.9030219	70.3551261	86.2690501	63	78.6773281	
82.8457846	69.9030219	72.3551261	86.2690501	63	78.8023281	
79.8457846	68.9030219	72.3551261	85.2690501	63	78.0523281	
78.8457846	68.9030219	72.3551261	85.2690501	63	77.8023281	
78.8457846	68.9030219	72.3551261	85.2690501	63	77.8023281	
78.8457846	68.9030219	72.3551261	85.2690501	63	77.8023281	
76.8457846	68.9030219	72.3551261	87.2690501	63	77.8023281	
74.8457846	68.9030219	72.3551261	89.2690501	63	77.8023281	
74.8457846	69.9030219	72.3551261	90.2690501	63	78.0523281	
74.8457846	69.9030219	72.3551261	90.2690501	63	78.1773281	
73.8457846	70.9030219	73.3551261	90.2690501	63	78.4273281	
73.8457846	70.9030219	74.3551261	90.2690501	63	79.4273281	
73.8457846	70.9030219	74.3551261	90.2690501	63	79.8023281	
73.8457846	70.9030219	74.3551261	90.2690501	63	79.8023281	
73.8457846	70.9030219	74.3551261	91.2690501	63	80.1773281	
73.8457846	70.9030219	74.3551261	91.2690501	63	80.3023281	
73.8457846	73.9030219	74.3551261	92.2690501	63	80.9273281	
73.8457846	76.9030219	73.3551261	92.2690501	63	81.0523281	
73.8457846	78.9030219	73.3551261	92.2690501	63	81.3023281	
79.8457846	78.9030219	73.3551261	92.2690501	63	82.1773281	
84.8457846	78.9030219	73.3551261	92.2690501	63	82.9273281	
85.8457846	78.9030219	73.3551261	92.2690501	63	82.8023281	
85.8457846	78.9030219	73.3551261	90.2690501	63	82.5523281	
85.8457846	78.9030219	73.3551261	88.2690501	63	82.4273281	
85.8457846	78.9030219	75.3551261	88.2690501	63	82.6773281	
86.8457846	74.9030219	76.3551261	87.2690501	63	82.3023281	
86.8457846	73.9030219	76.3551261	87.2690501	63	82.0523281	
86.8457846	73.9030219	76.3551261	87.2690501	63	81.5523281	
82.8457846	72.9030219	76.3551261	87.2690501	63	80.6773281	
82.8457846	72.9030219	76.3551261	87.2690501	63	80.4273281	
81.8457846	73.9030219	76.3551261	87.2690501	63	80.4273281	

82.8457846	73.9030219	76.3551261	87.2690501	63	80.8023281	
82.8457846	73.9030219	76.3551261	87.2690501	63	80.8023281	
82.8457846	72.9030219	76.3551261	87.2690501	63	80.6773281	
82.8457846	72.9030219	76.3551261	83.2690501	63	80.1773281	
82.8457846	72.9030219	76.3551261	81.2690501	63	79.9273281	
82.8457846	72.9030219	78.3551261	82.2690501	63	80.8023281	
82.8457846	72.9030219	78.3551261	82.2690501	63	81.1773281	
82.8457846	72.9030219	73.3551261	83.2690501	63	80.8023281	
82.8457846	72.9030219	73.3551261	84.2690501	63	80.8023281	
82.8457846	71.9030219	73.3551261	83.2690501	63	80.3023281	
82.8457846	71.9030219	73.3551261	83.2690501	63	80.0523281	
82.8457846	71.9030219	73.3551261	83.2690501	63	79.4273281	
82.8457846	71.9030219	73.3551261	82.2690501	63	78.9273281	
80.8457846	71.9030219	72.3551261	82.2690501	63	78.4273281	
80.8457846	71.9030219	72.3551261	82.2690501	63	78.3023281	
79.8457846	71.9030219	72.3551261	82.2690501	63	77.8023281	
75.8457846	71.9030219	72.3551261	82.2690501	63	77.0523281	
75.8457846	71.9030219	72.3551261	82.2690501	63	77.0523281	
72.8457846	72.9030219	72.3551261	84.2690501	63	77.0523281	
72.8457846	72.9030219	72.3551261	85.2690501	63	76.9273281	
72.8457846	73.9030219	74.3551261	85.2690501	63	77.3023281	
71.8457846	73.9030219	74.3551261	85.2690501	63	77.0523281	
71.8457846	73.9030219	74.3551261	85.2690501	63	77.1773281	
71.8457846	72.9030219	74.3551261	85.2690501	63	76.5523281	
71.8457846	71.9030219	74.3551261	84.2690501	63	76.0523281	
72.8457846	71.9030219	74.3551261	84.2690501	63	76.3023281	
73.8457846	71.9030219	74.3551261	84.2690501	63	76.6773281	
77.8457846	72.9030219	74.3551261	84.2690501	63	77.4273281	
77.8457846	73.9030219	74.3551261	84.2690501	63	77.5523281	
78.8457846	74.9030219	74.3551261	87.2690501	63	78.5523281	
78.8457846	74.9030219	74.3551261	87.2690501	63	78.8023281	
78.8457846	76.9030219	74.3551261	88.2690501	63	79.3023281	
78.8457846	76.9030219	74.3551261	88.2690501	63	79.4273281	
78.8457846	76.9030219	73.3551261	90.2690501	63	79.6773281	
78.8457846	75.9030219	73.3551261	90.2690501	63	79.4273281	
78.8457846	75.9030219	72.3551261	90.2690501	63	79.5523281	
78.8457846	74.9030219	70.3551261	91.2690501	63	79.4273281	
78.8457846	73.9030219	67.3551261	91.2690501	63	78.9273281	
77.8457846	73.9030219	67.3551261	90.2690501	63	78.9273281	
76.8457846	73.9030219	65.3551261	89.2690501	63	78.6773281	
75.8457846	73.9030219	65.3551261	89.2690501	63	78.9273281	
75.8457846	74.9030219	65.3551261	87.2690501	63	78.9273281	
75.8457846	75.9030219	66.3551261	86.2690501	63	79.4273281	
73.8457846	75.9030219	67.3551261	86.2690501	63	79.3023281	
74.8457846	75.9030219	67.3551261	86.2690501	63	79.5523281	

74.8457846	75.9030219	68.3551261	86.2690501	63	79.8023281	
76.8457846	74.9030219	68.3551261	86.2690501	63	79.8023281	
77.8457846	74.9030219	69.3551261	88.2690501	63	80.4273281	
78.8457846	73.9030219	70.3551261	88.2690501	63	80.6773281	
78.8457846	72.9030219	69.3551261	87.2690501	63	80.3023281	
78.8457846	72.9030219	69.3551261	87.2690501	63	80.3023281	
78.8457846	72.9030219	75.3551261	87.2690501	63	81.0523281	
77.8457846	72.9030219	76.3551261	88.2690501	63	81.1773281	
77.8457846	72.9030219	76.3551261	89.2690501	63	81.3023281	
77.8457846	72.9030219	76.3551261	89.2690501	63	81.3023281	
78.8457846	72.9030219	76.3551261	89.2690501	63	80.3023281	
78.8457846	72.9030219	76.3551261	89.2690501	63	79.9273281	
78.8457846	71.9030219	78.3551261	89.2690501	63	79.8023281	
79.8457846	72.9030219	78.3551261	89.2690501	63	80.0523281	
80.8457846	72.9030219	80.3551261	89.2690501	63	80.4273281	
81.8457846	74.9030219	80.3551261	89.2690501	63	80.9273281	
82.8457846	74.9030219	80.3551261	90.2690501	63	80.9273281	
82.8457846	74.9030219	80.3551261	89.2690501	63	80.6773281	
80.8457846	74.9030219	80.3551261	89.2690501	63	80.4273281	
80.8457846	75.9030219	80.3551261	89.2690501	63	80.4273281	
80.8457846	75.9030219	80.3551261	89.2690501	63	79.8023281	
80.8457846	74.9030219	80.3551261	89.2690501	63	79.3023281	
80.8457846	74.9030219	81.3551261	90.2690501	63	79.4273281	
79.8457846	75.9030219	81.3551261	91.2690501	63	79.4273281	
79.8457846	76.9030219	83.3551261	91.2690501	63	79.9273281	
79.8457846	76.9030219	83.3551261	91.2690501	63	80.1773281	
79.8457846	76.9030219	83.3551261	91.2690501	63	80.1773281	
77.8457846	75.9030219	81.3551261	91.2690501	63	79.8023281	
77.8457846	75.9030219	81.3551261	90.2690501	63	79.9273281	
76.8457846	73.9030219	79.3551261	90.2690501	63	79.5523281	
74.8457846	71.9030219	79.3551261	90.2690501	63	78.9273281	
74.8457846	69.9030219	79.3551261	90.2690501	63	78.8023281	
74.8457846	69.9030219	79.3551261	90.2690501	63	78.8023281	
74.8457846	69.9030219	79.3551261	90.2690501	63	78.6773281	
74.8457846	69.9030219	79.3551261	90.2690501	63	78.6773281	
74.8457846	70.9030219	80.3551261	90.2690501	63	78.9273281	
75.8457846	71.9030219	81.3551261	90.2690501	63	79.0523281	
75.8457846	72.9030219	84.3551261	89.2690501	63	79.1773281	
76.8457846	73.9030219	84.3551261	89.2690501	63	79.3023281	
75.8457846	73.9030219	81.3551261	89.2690501	63	78.4273281	
75.8457846	72.9030219	78.3551261	89.2690501	63	78.0523281	
75.8457846	72.9030219	77.3551261	89.2690501	63	77.9273281	
75.8457846	72.9030219	76.3551261	89.2690501	63	77.9273281	
75.8457846	71.9030219	76.3551261	89.2690501	63	78.3023281	
74.8457846	68.9030219	74.3551261	90.2690501	63	77.5523281	

72.8457846	68.9030219	75.3551261	90.2690501	63	77.6773281	
71.8457846	68.9030219	76.3551261	90.2690501	63	77.3023281	
71.8457846	69.9030219	77.3551261	90.2690501	63	77.5523281	
70.8457846	69.9030219	77.3551261	90.2690501	63	77.5523281	
70.8457846	69.9030219	78.3551261	90.2690501	63	77.9273281	
70.8457846	69.9030219	77.3551261	88.2690501	63	77.6773281	
77.8457846	69.9030219	74.3551261	88.2690501	63	78.8023281	
78.8457846	69.9030219	72.3551261	89.2690501	63	78.8023281	
78.8457846	69.9030219	70.3551261	90.2690501	63	78.8023281	
78.8457846	69.9030219	69.3551261	90.2690501	63	78.4273281	
78.8457846	71.9030219	68.3551261	91.2690501	63	78.6773281	
77.8457846	71.9030219	66.3551261	90.2690501	63	77.6773281	
77.8457846	71.9030219	66.3551261	89.2690501	63	77.8023281	
77.8457846	72.9030219	65.3551261	89.2690501	63	77.9273281	
78.8457846	73.9030219	64.3551261	88.2690501	63	78.0523281	
81.8457846	74.9030219	64.3551261	88.2690501	63	78.6773281	
83.8457846	75.9030219	64.3551261	88.2690501	63	79.1773281	
79.8457846	75.9030219	64.3551261	87.2690501	63	78.6773281	
79.8457846	71.9030219	64.3551261	87.2690501	63	78.8023281	
76.8457846	70.9030219	64.3551261	87.2690501	63	78.5523281	
75.8457846	70.9030219	65.3551261	85.2690501	63	78.6773281	
76.8457846	70.9030219	65.3551261	85.2690501	63	78.9273281	
77.8457846	70.9030219	65.3551261	85.2690501	63	79.3023281	
79.8457846	71.9030219	65.3551261	85.2690501	63	79.6773281	
79.8457846	71.9030219	64.3551261	85.2690501	63	79.4273281	
79.8457846	71.9030219	64.3551261	84.2690501	63	79.4273281	
79.8457846	71.9030219	64.3551261	84.2690501	63	79.4273281	
79.8457846	72.9030219	64.3551261	87.2690501	63	79.9273281	
79.8457846	72.9030219	64.3551261	88.2690501	63	80.3023281	
80.8457846	72.9030219	64.3551261	88.2690501	63	80.4273281	
79.8457846	72.9030219	65.3551261	88.2690501	63	80.4273281	
79.8457846	73.9030219	68.3551261	88.2690501	63	81.1773281	
79.8457846	74.9030219	69.3551261	89.2690501	63	81.4273281	
79.8457846	74.9030219	69.3551261	90.2690501	63	81.8023281	
78.8457846	76.9030219	69.3551261	90.2690501	63	82.3023281	
78.8457846	76.9030219	69.3551261	90.2690501	63	82.3023281	
78.8457846	76.9030219	71.3551261	91.2690501	63	82.3023281	
78.8457846	78.9030219	69.3551261	92.2690501	63	81.8023281	
78.8457846	80.9030219	69.3551261	92.2690501	63	82.0523281	
78.8457846	81.9030219	69.3551261	92.2690501	63	81.9273281	
79.8457846	81.9030219	69.3551261	89.2690501	63	80.4273281	
79.8457846	81.9030219	67.3551261	89.2690501	63	80.1773281	
81.8457846	81.9030219	66.3551261	89.2690501	63	80.0523281	
81.8457846	81.9030219	66.3551261	87.2690501	63	79.6773281	
81.8457846	82.9030219	67.3551261	87.2690501	63	79.8023281	

81.8457846	83.9030219	67.3551261	87.2690501	63	80.0523281	
81.8457846	83.9030219	67.3551261	83.2690501	63	79.5523281	
81.8457846	83.9030219	68.3551261	83.2690501	63	80.0523281	
81.8457846	82.9030219	68.3551261	83.2690501	63	78.0523281	
82.8457846	81.9030219	69.3551261	84.2690501	63	78.5523281	
82.8457846	79.9030219	69.3551261	86.2690501	63	78.4273281	
84.8457846	79.9030219	71.3551261	86.2690501	63	78.8023281	
84.8457846	74.9030219	71.3551261	86.2690501	63	77.9273281	
85.8457846	70.9030219	71.3551261	86.2690501	63	77.5523281	
88.8457846	68.9030219	72.3551261	85.2690501	63	77.4273281	
88.8457846	68.9030219	72.3551261	85.2690501	63	77.5523281	
88.8457846	68.9030219	72.3551261	85.2690501	63	77.6773281	
88.8457846	68.9030219	72.3551261	84.2690501	63	77.3023281	
88.8457846	68.9030219	72.3551261	84.2690501	63	77.0523281	
89.8457846	67.9030219	72.3551261	84.2690501	63	77.4273281	
89.8457846	68.9030219	72.3551261	85.2690501	63	77.5523281	
90.8457846	71.9030219	72.3551261	86.2690501	63	78.3023281	
90.8457846	71.9030219	71.3551261	86.2690501	63	78.5523281	
89.8457846	73.9030219	70.3551261	88.2690501	63	78.8023281	
90.8457846	76.9030219	70.3551261	89.2690501	63	79.6773281	
90.8457846	76.9030219	70.3551261	89.2690501	63	79.9273281	
90.8457846	76.9030219	70.3551261	88.2690501	63	79.6773281	
90.8457846	77.9030219	70.3551261	88.2690501	63	79.6773281	
91.8457846	76.9030219	77.3551261	88.2690501	63	80.8023281	
91.8457846	74.9030219	78.3551261	89.2690501	63	81.1773281	
91.8457846	71.9030219	78.3551261	91.2690501	63	81.1773281	
91.8457846	71.9030219	78.3551261	93.2690501	63	81.8023281	
91.8457846	71.9030219	78.3551261	94.2690501	63	82.1773281	
92.8457846	71.9030219	77.3551261	95.2690501	63	82.3023281	
92.8457846	71.9030219	77.3551261	95.2690501	63	82.0523281	
92.8457846	70.9030219	77.3551261	95.2690501	63	81.8023281	
95.8457846	69.9030219	77.3551261	91.2690501	63	81.6773281	
96.8457846	69.9030219	78.3551261	89.2690501	63	81.5523281	
96.8457846	69.9030219	78.3551261	88.2690501	63	81.4273281	
93.8457846	68.9030219	78.3551261	87.2690501	63	80.8023281	
93.8457846	68.9030219	78.3551261	87.2690501	63	81.3023281	
92.8457846	67.9030219	78.3551261	86.2690501	63	81.4273281	
91.8457846	66.9030219	78.3551261	86.2690501	63	81.5523281	
91.8457846	66.9030219	78.3551261	85.2690501	63	81.4273281	
91.8457846	67.9030219	78.3551261	84.2690501	63	81.5523281	
90.8457846	67.9030219	78.3551261	83.2690501	63	81.1773281	
90.8457846	68.9030219	78.3551261	82.2690501	63	81.1773281	
90.8457846	68.9030219	80.3551261	81.2690501	63	81.3023281	
88.8457846	68.9030219	81.3551261	81.2690501	63	81.1773281	
88.8457846	68.9030219	81.3551261	81.2690501	63	81.1773281	

87.8457846	67.9030219	80.3551261	82.2690501	63	81.4273281	
86.8457846	67.9030219	80.3551261	82.2690501	63	81.1773281	
84.8457846	68.9030219	80.3551261	83.2690501	63	81.0523281	
84.8457846	68.9030219	76.3551261	82.2690501	63	79.6773281	
80.8457846	69.9030219	72.3551261	82.2690501	63	78.4273281	
82.8457846	69.9030219	71.3551261	81.2690501	63	78.0523281	
82.8457846	69.9030219	70.3551261	81.2690501	63	77.6773281	
82.8457846	68.9030219	70.3551261	82.2690501	63	76.9273281	
84.8457846	67.9030219	70.3551261	82.2690501	63	76.4273281	
84.8457846	67.9030219	70.3551261	83.2690501	63	76.5523281	
84.8457846	67.9030219	70.3551261	83.2690501	63	75.9273281	
84.8457846	67.9030219	70.3551261	83.2690501	63	76.0523281	
84.8457846	67.9030219	70.3551261	83.2690501	63	76.5523281	
84.8457846	68.9030219	70.3551261	83.2690501	63	77.0523281	
84.8457846	68.9030219	70.3551261	82.2690501	63	77.1773281	
84.8457846	68.9030219	69.3551261	82.2690501	63	77.1773281	
83.8457846	68.9030219	67.3551261	82.2690501	63	76.9273281	
83.8457846	68.9030219	67.3551261	82.2690501	63	76.9273281	
83.8457846	68.9030219	67.3551261	81.2690501	63	76.9273281	
83.8457846	68.9030219	67.3551261	81.2690501	63	77.0523281	
81.8457846	68.9030219	66.3551261	81.2690501	63	77.3023281	
78.8457846	68.9030219	65.3551261	81.2690501	63	76.8023281	
75.8457846	68.9030219	65.3551261	81.2690501	63	76.5523281	
75.8457846	67.9030219	65.3551261	81.2690501	63	76.6773281	
75.8457846	67.9030219	65.3551261	81.2690501	63	76.6773281	
75.8457846	67.9030219	65.3551261	80.2690501	63	76.4273281	
75.8457846	68.9030219	64.3551261	80.2690501	63	76.0523281	
75.8457846	68.9030219	64.3551261	80.2690501	63	75.8023281	
75.8457846	68.9030219	69.3551261	81.2690501	63	76.4273281	
74.8457846	68.9030219	71.3551261	82.2690501	63	76.6773281	
74.8457846	68.9030219	71.3551261	82.2690501	63	76.6773281	
75.8457846	68.9030219	71.3551261	82.2690501	63	76.8023281	
76.8457846	69.9030219	72.3551261	82.2690501	63	77.1773281	
76.8457846	69.9030219	73.3551261	82.2690501	63	77.4273281	
76.8457846	69.9030219	74.3551261	83.2690501	63	77.3023281	
77.8457846	71.9030219	74.3551261	83.2690501	63	77.8023281	
78.8457846	71.9030219	76.3551261	87.2690501	63	78.8023281	
80.8457846	72.9030219	76.3551261	88.2690501	63	79.3023281	
80.8457846	72.9030219	76.3551261	89.2690501	63	79.3023281	
80.8457846	72.9030219	76.3551261	89.2690501	63	79.1773281	
80.8457846	72.9030219	76.3551261	89.2690501	63	79.6773281	
80.8457846	72.9030219	77.3551261	89.2690501	63	80.0523281	
80.8457846	72.9030219	77.3551261	89.2690501	63	80.1773281	
80.8457846	72.9030219	77.3551261	90.2690501	63	80.1773281	
80.8457846	72.9030219	77.3551261	91.2690501	63	80.0523281	

78.8457846	72.9030219	77.3551261	91.2690501	63	79.8023281	
77.8457846	72.9030219	76.3551261	91.2690501	63	79.1773281	
77.8457846	72.9030219	75.3551261	90.2690501	63	79.0523281	
76.8457846	72.9030219	73.3551261	90.2690501	63	78.4273281	
76.8457846	72.9030219	72.3551261	87.2690501	63	77.9273281	
76.8457846	72.9030219	72.3551261	86.2690501	63	77.8023281	
76.8457846	72.9030219	71.3551261	86.2690501	63	77.8023281	
76.8457846	72.9030219	71.3551261	86.2690501	63	77.8023281	
76.8457846	72.9030219	71.3551261	86.2690501	63	77.8023281	
76.8457846	71.9030219	71.3551261	87.2690501	63	77.5523281	
76.8457846	71.9030219	71.3551261	87.2690501	63	77.4273281	
75.8457846	71.9030219	71.3551261	88.2690501	63	77.4273281	
75.8457846	75.9030219	71.3551261	88.2690501	63	77.9273281	
75.8457846	76.9030219	72.3551261	88.2690501	63	78.5523281	
75.8457846	76.9030219	74.3551261	88.2690501	63	78.8023281	
75.8457846	76.9030219	74.3551261	88.2690501	63	78.8023281	
75.8457846	76.9030219	76.3551261	87.2690501	63	78.9273281	
75.8457846	76.9030219	77.3551261	87.2690501	63	78.9273281	
75.8457846	76.9030219	77.3551261	87.2690501	63	79.4273281	
76.8457846	76.9030219	77.3551261	87.2690501	63	80.1773281	
76.8457846	76.9030219	78.3551261	87.2690501	63	80.1773281	
76.8457846	74.9030219	81.3551261	88.2690501	63	80.1773281	
76.8457846	73.9030219	81.3551261	88.2690501	63	79.8023281	
76.8457846	73.9030219	81.3551261	88.2690501	63	79.8023281	
76.8457846	73.9030219	81.3551261	86.2690501	63	79.9273281	
79.8457846	73.9030219	82.3551261	86.2690501	63	80.5523281	
79.8457846	73.9030219	83.3551261	86.2690501	63	81.0523281	
79.8457846	73.9030219	84.3551261	86.2690501	63	81.5523281	
79.8457846	73.9030219	86.3551261	85.2690501	63	81.6773281	
77.8457846	73.9030219	87.3551261	85.2690501	63	81.9273281	
75.8457846	73.9030219	87.3551261	85.2690501	63	81.9273281	
75.8457846	73.9030219	87.3551261	85.2690501	63	81.6773281	
78.8457846	73.9030219	87.3551261	85.2690501	63	81.9273281	
77.8457846	73.9030219	89.3551261	85.2690501	63	81.5523281	
76.8457846	74.9030219	86.3551261	86.2690501	63	81.0523281	
75.8457846	74.9030219	87.3551261	86.2690501	63	80.8023281	
75.8457846	74.9030219	87.3551261	86.2690501	63	80.6773281	
75.8457846	74.9030219	87.3551261	86.2690501	63	80.6773281	
75.8457846	75.9030219	88.3551261	86.2690501	63	80.5523281	
75.8457846	75.9030219	88.3551261	86.2690501	63	80.8023281	
76.8457846	75.9030219	88.3551261	86.2690501	63	80.8023281	
76.8457846	76.9030219	86.3551261	87.2690501	63	80.4273281	
78.8457846	76.9030219	79.3551261	87.2690501	63	79.9273281	
79.8457846	75.9030219	76.3551261	87.2690501	63	79.5523281	
79.8457846	74.9030219	73.3551261	87.2690501	63	78.8023281	

80.8457846	75.9030219	71.3551261	87.2690501	63	79.0523281	
80.8457846	75.9030219	71.3551261	86.2690501	63	78.9273281	
79.8457846	75.9030219	69.3551261	86.2690501	69	78.5523281	
79.8457846	75.9030219	69.3551261	86.2690501	69	78.4273281	
79.8457846	75.9030219	69.3551261	86.2690501	69	78.3023281	
79.8457846	79.9030219	68.3551261	88.2690501	69	78.8023281	
78.8457846	80.9030219	68.3551261	88.2690501	69	78.8023281	
78.8457846	80.9030219	69.3551261	87.2690501	69	78.8023281	
78.8457846	81.9030219	69.3551261	87.2690501	69	78.9273281	
76.8457846	81.9030219	68.3551261	87.2690501	69	78.4273281	
76.8457846	81.9030219	67.3551261	87.2690501	69	77.9273281	
75.8457846	80.9030219	67.3551261	87.2690501	69	77.0523281	
75.8457846	79.9030219	67.3551261	87.2690501	69	76.9273281	
75.8457846	78.9030219	67.3551261	87.2690501	69	76.4273281	
74.8457846	78.9030219	67.3551261	87.2690501	69	76.3023281	
74.8457846	73.9030219	67.3551261	86.2690501	69	75.4273281	
72.8457846	71.9030219	67.3551261	86.2690501	69	75.1773281	
72.8457846	71.9030219	67.3551261	86.2690501	69	75.1773281	
71.8457846	69.9030219	68.3551261	88.2690501	69	75.4273281	
71.8457846	69.9030219	68.3551261	89.2690501	69	75.4273281	
70.8457846	69.9030219	68.3551261	90.2690501	69	75.1773281	
71.8457846	68.9030219	67.3551261	91.2690501	69	75.0523281	
71.8457846	66.9030219	67.3551261	91.2690501	69	74.8023281	
72.8457846	66.9030219	68.3551261	93.2690501	69	75.9273281	
72.8457846	66.9030219	68.3551261	94.2690501	69	76.6773281	
76.8457846	64.9030219	68.3551261	94.2690501	69	77.4273281	
76.8457846	64.9030219	68.3551261	93.2690501	69	77.3023281	
76.8457846	63.9030219	69.3551261	92.2690501	69	77.1773281	
84.8457846	64.9030219	68.3551261	92.2690501	69	78.3023281	
84.8457846	64.9030219	68.3551261	92.2690501	69	78.5523281	
85.8457846	64.9030219	68.3551261	92.2690501	69	78.1773281	
85.8457846	64.9030219	68.3551261	92.2690501	69	78.0523281	
86.8457846	64.9030219	69.3551261	91.2690501	69	78.1773281	
86.8457846	65.9030219	69.3551261	91.2690501	69	77.8023281	
87.8457846	65.9030219	69.3551261	91.2690501	69	77.9273281	
89.8457846	66.9030219	69.3551261	89.2690501	69	77.9273281	
88.8457846	66.9030219	70.3551261	89.2690501	69	77.9273281	
88.8457846	66.9030219	70.3551261	88.2690501	69	77.5523281	
85.8457846	67.9030219	70.3551261	91.2690501	69	77.8023281	
79.8457846	68.9030219	70.3551261	91.2690501	69	77.1773281	
79.8457846	68.9030219	70.3551261	90.2690501	69	77.4273281	
79.8457846	68.9030219	70.3551261	90.2690501	69	77.4273281	
79.8457846	68.9030219	72.3551261	86.2690501	69	76.9273281	
77.8457846	68.9030219	72.3551261	85.2690501	69	76.6773281	
77.8457846	68.9030219	72.3551261	85.2690501	69	76.8023281	

78.8457846	68.9030219	72.3551261	84.2690501	69	76.9273281	
78.8457846	68.9030219	74.3551261	84.2690501	69	77.1773281	
78.8457846	68.9030219	76.3551261	84.2690501	69	77.3023281	
79.8457846	69.9030219	76.3551261	84.2690501	69	77.4273281	
79.8457846	69.9030219	76.3551261	84.2690501	69	77.4273281	
79.8457846	70.9030219	74.3551261	84.2690501	69	77.1773281	
78.8457846	70.9030219	73.3551261	83.2690501	69	76.0523281	
77.8457846	70.9030219	73.3551261	83.2690501	69	75.5523281	
79.8457846	69.9030219	73.3551261	83.2690501	69	75.5523281	
79.8457846	69.9030219	72.3551261	83.2690501	69	75.3023281	
79.8457846	69.9030219	72.3551261	83.2690501	69	75.6773281	
79.8457846	68.9030219	71.3551261	83.2690501	69	75.6773281	
78.8457846	68.9030219	71.3551261	81.2690501	69	75.5523281	
78.8457846	69.9030219	71.3551261	79.2690501	69	75.4273281	
79.8457846	69.9030219	71.3551261	79.2690501	69	75.5523281	
79.8457846	69.9030219	71.3551261	79.2690501	69	75.4273281	
79.8457846	69.9030219	71.3551261	79.2690501	69	75.5523281	
79.8457846	69.9030219	71.3551261	79.2690501	69	75.9273281	
79.8457846	70.9030219	70.3551261	79.2690501	69	75.9273281	
79.8457846	70.9030219	69.3551261	79.2690501	69	75.8023281	
83.8457846	70.9030219	68.3551261	80.2690501	69	76.3023281	
82.8457846	70.9030219	67.3551261	82.2690501	69	76.3023281	
82.8457846	71.9030219	67.3551261	82.2690501	69	76.4273281	
82.8457846	70.9030219	67.3551261	82.2690501	69	76.5523281	
82.8457846	68.9030219	67.3551261	82.2690501	69	76.1773281	
82.8457846	68.9030219	67.3551261	83.2690501	69	76.3023281	
82.8457846	68.9030219	67.3551261	83.2690501	63	76.3023281	
80.8457846	70.9030219	67.3551261	83.2690501	63	76.4273281	
80.8457846	71.9030219	67.3551261	82.2690501	63	76.5523281	
80.8457846	73.9030219	67.3551261	82.2690501	63	76.8023281	
80.8457846	73.9030219	67.3551261	78.2690501	63	76.3023281	
79.8457846	73.9030219	67.3551261	78.2690501	63	76.8023281	
79.8457846	72.9030219	67.3551261	79.2690501	63	77.0523281	
78.8457846	71.9030219	67.3551261	79.2690501	63	76.1773281	
77.8457846	71.9030219	67.3551261	81.2690501	63	76.0523281	
77.8457846	68.9030219	67.3551261	82.2690501	63	75.6773281	
77.8457846	67.9030219	67.3551261	82.2690501	63	75.6773281	
77.8457846	66.9030219	68.3551261	83.2690501	63	75.8023281	
77.8457846	64.9030219	71.3551261	83.2690501	63	76.0523281	
82.8457846	64.9030219	73.3551261	83.2690501	63	77.0523281	
85.8457846	65.9030219	73.3551261	83.2690501	63	77.6773281	
85.8457846	66.9030219	73.3551261	83.2690501	63	77.6773281	
85.8457846	66.9030219	74.3551261	83.2690501	63	78.0523281	
85.8457846	66.9030219	73.3551261	83.2690501	63	78.0523281	
85.8457846	67.9030219	72.3551261	83.2690501	63	78.0523281	

86.8457846	67.9030219	70.3551261	83.2690501	63	77.9273281	
86.8457846	67.9030219	70.3551261	85.2690501	63	77.9273281	
86.8457846	68.9030219	70.3551261	85.2690501	63	77.6773281	
87.8457846	68.9030219	69.3551261	86.2690501	63	77.5523281	
87.8457846	69.9030219	67.3551261	86.2690501	63	77.1773281	
87.8457846	68.9030219	67.3551261	86.2690501	63	76.9273281	
87.8457846	68.9030219	67.3551261	85.2690501	63	76.6773281	
86.8457846	67.9030219	67.3551261	82.2690501	63	76.4273281	
81.8457846	67.9030219	67.3551261	81.2690501	63	75.6773281	
78.8457846	65.9030219	66.3551261	81.2690501	63	75.0523281	
76.8457846	65.9030219	67.3551261	81.2690501	63	74.8023281	
76.8457846	65.9030219	67.3551261	82.2690501	63	75.0523281	
76.8457846	65.9030219	68.3551261	84.2690501	63	73.3023281	
76.8457846	65.9030219	68.3551261	84.2690501	63	73.1773281	
80.8457846	65.9030219	69.3551261	83.2690501	63	73.9273281	
80.8457846	64.9030219	69.3551261	81.2690501	63	73.8023281	
81.8457846	65.9030219	69.3551261	81.2690501	63	75.0523281	
79.8457846	65.9030219	70.3551261	81.2690501	63	75.6773281	
72.8457846	65.9030219	70.3551261	81.2690501	63	74.9273281	
68.8457846	65.9030219	70.3551261	81.2690501	63	74.8023281	
68.8457846	65.9030219	70.3551261	82.2690501	63	74.9273281	
64.8457846	66.9030219	70.3551261	82.2690501	63	74.6773281	
64.8457846	66.9030219	70.3551261	82.2690501	63	74.6773281	
64.8457846	66.9030219	70.3551261	82.2690501	63	74.6773281	
64.8457846	66.9030219	70.3551261	82.2690501	63	74.6773281	
65.8457846	66.9030219	70.3551261	81.2690501	63	74.6773281	
65.8457846	67.9030219	70.3551261	81.2690501	63	74.6773281	
69.8457846	67.9030219	68.3551261	82.2690501	63	74.9273281	
69.8457846	66.9030219	68.3551261	83.2690501	63	74.9273281	
72.8457846	66.9030219	69.3551261	84.2690501	63	75.3023281	
73.8457846	67.9030219	70.3551261	85.2690501	63	75.9273281	
73.8457846	67.9030219	70.3551261	85.2690501	63	75.8023281	
73.8457846	68.9030219	70.3551261	86.2690501	63	76.0523281	
72.8457846	69.9030219	70.3551261	84.2690501	63	75.8023281	
72.8457846	71.9030219	70.3551261	83.2690501	63	75.6773281	
72.8457846	71.9030219	70.3551261	83.2690501	63	75.6773281	
72.8457846	73.9030219	70.3551261	82.2690501	63	76.0523281	
72.8457846	73.9030219	70.3551261	82.2690501	63	75.5523281	
72.8457846	72.9030219	68.3551261	82.2690501	63	75.0523281	
71.8457846	72.9030219	68.3551261	83.2690501	63	74.6773281	
71.8457846	72.9030219	69.3551261	83.2690501	63	74.6773281	
72.8457846	72.9030219	69.3551261	83.2690501	63	74.5523281	
72.8457846	70.9030219	70.3551261	83.2690501	63	74.3023281	
75.8457846	70.9030219	70.3551261	82.2690501	63	74.5523281	
77.8457846	70.9030219	70.3551261	81.2690501	63	74.8023281	

77.8457846	70.9030219	70.3551261	81.2690501	63	74.9273281	
77.8457846	70.9030219	69.3551261	80.2690501	63	74.8023281	
77.8457846	70.9030219	70.3551261	80.2690501	63	74.9273281	
76.8457846	70.9030219	70.3551261	80.2690501	63	74.8023281	
76.8457846	70.9030219	70.3551261	79.2690501	63	74.4273281	
77.8457846	70.9030219	70.3551261	79.2690501	63	74.1773281	
78.8457846	70.9030219	70.3551261	79.2690501	63	73.9273281	
79.8457846	69.9030219	70.3551261	79.2690501	63	74.1773281	
79.8457846	67.9030219	70.3551261	80.2690501	63	74.1773281	
78.8457846	66.9030219	70.3551261	82.2690501	63	74.6773281	
77.8457846	66.9030219	70.3551261	82.2690501	63	74.8023281	
76.8457846	66.9030219	70.3551261	82.2690501	63	74.4273281	
76.8457846	66.9030219	70.3551261	81.2690501	63	74.1773281	
77.8457846	66.9030219	70.3551261	81.2690501	63	74.3023281	
78.8457846	66.9030219	70.3551261	81.2690501	63	74.6773281	
79.8457846	66.9030219	70.3551261	81.2690501	63	74.8023281	
79.8457846	66.9030219	69.3551261	81.2690501	63	74.4273281	
79.8457846	65.9030219	69.3551261	81.2690501	63	74.3023281	
79.8457846	64.9030219	69.3551261	81.2690501	63	74.1773281	
78.8457846	63.9030219	68.3551261	82.2690501	63	73.8023281	
77.8457846	63.9030219	68.3551261	80.2690501	63	73.4273281	
77.8457846	62.9030219	68.3551261	79.2690501	63	73.3023281	
77.8457846	63.9030219	67.3551261	79.2690501	63	74.4273281	
77.8457846	63.9030219	67.3551261	78.2690501	63	74.4273281	
77.8457846	63.9030219	67.3551261	78.2690501	63	74.9273281	
78.8457846	63.9030219	68.3551261	78.2690501	63	74.6773281	
79.8457846	63.9030219	69.3551261	78.2690501	63	75.0523281	
79.8457846	63.9030219	69.3551261	78.2690501	63	75.0523281	
80.8457846	63.9030219	69.3551261	79.2690501	63	75.1773281	
80.8457846	66.9030219	69.3551261	79.2690501	63	75.9273281	
80.8457846	66.9030219	69.3551261	79.2690501	63	75.8023281	
80.8457846	66.9030219	69.3551261	79.2690501	63	75.9273281	
80.8457846	66.9030219	69.3551261	79.2690501	63	75.8023281	
79.8457846	66.9030219	70.3551261	79.2690501	63	75.8023281	
79.8457846	67.9030219	71.3551261	84.2690501	63	76.6773281	
79.8457846	67.9030219	71.3551261	84.2690501	63	76.6773281	
79.8457846	68.9030219	71.3551261	85.2690501	63	76.4273281	
79.8457846	68.9030219	71.3551261	85.2690501	63	76.1773281	
79.8457846	68.9030219	73.3551261	85.2690501	63	76.4273281	
79.8457846	69.9030219	73.3551261	84.2690501	63	76.8023281	
79.8457846	69.9030219	75.3551261	82.2690501	63	76.9273281	
79.8457846	70.9030219	74.3551261	81.2690501	63	76.8023281	
79.8457846	71.9030219	74.3551261	79.2690501	63	76.6773281	
80.8457846	71.9030219	74.3551261	79.2690501	63	76.8023281	
80.8457846	71.9030219	74.3551261	79.2690501	63	76.8023281	

82.8457846	71.9030219	73.3551261	79.2690501	63	76.9273281	
80.8457846	66.9030219	73.3551261	79.2690501	63	75.9273281	
79.8457846	66.9030219	73.3551261	79.2690501	63	75.5523281	
77.8457846	65.9030219	73.3551261	80.2690501	63	75.1773281	
76.8457846	65.9030219	73.3551261	80.2690501	63	74.8023281	
77.8457846	65.9030219	73.3551261	80.2690501	63	75.4273281	
78.8457846	64.9030219	74.3551261	81.2690501	63	76.0523281	
77.8457846	64.9030219	74.3551261	82.2690501	63	76.0523281	
76.8457846	64.9030219	74.3551261	83.2690501	63	76.5523281	
76.8457846	63.9030219	74.3551261	83.2690501	63	76.4273281	
75.8457846	61.9030219	73.3551261	83.2690501	63	76.0523281	
75.8457846	61.9030219	73.3551261	83.2690501	63	76.0523281	
76.8457846	61.9030219	72.3551261	82.2690501	63	75.5523281	
76.8457846	62.9030219	71.3551261	81.2690501	63	75.5523281	
77.8457846	62.9030219	71.3551261	80.2690501	63	75.6773281	before 1
78.8457846	64.9030219	72.3551261	80.2690501	63	76.1773281	before 2
79.8457846	65.9030219	72.3551261	80.2690501	63	76.5523281	before 3
80.8457846	65.9030219	72.3551261	80.2690501	63	76.5523281	before 4
80.8457846	70.9030219	72.3551261	80.2690501	63	77.0523281	before 5
79.8457846	71.9030219	72.3551261	81.2690501	63	76.6773281	before 6
79.8457846	72.9030219	72.3551261	82.2690501	63	76.5523281	before 7
78.8457846	71.9030219	74.3551261	82.2690501	63	76.5523281	before 8
77.8457846	70.9030219	74.3551261	82.2690501	63	76.1773281	before 9
77.8457846	69.9030219	74.3551261	81.2690501	63	75.6773281	before 10
77.8457846	68.9030219	74.3551261	81.2690501	63	75.4273281	before 11
76.8457846	68.9030219	74.3551261	81.2690501	63	75.4273281	before 12
76.8457846	67.9030219	74.3551261	80.2690501	63	75.1773281	before 13
76.8457846	67.9030219	75.3551261	80.2690501	63	75.3023281	before 14
76.8457846	67.9030219	75.3551261	80.2690501	63	75.0523281	before 15
76.8457846	67.9030219	75.3551261	81.2690501	63	75.1773281	before 16
76.8457846	67.9030219	75.3551261	82.2690501	63	75.6773281	before 17
76.8457846	67.9030219	75.3551261	82.2690501	63	75.6773281	before 18
76.8457846	67.9030219	75.3551261	84.2690501	63	75.9273281	before 19
75.8457846	68.9030219	75.3551261	85.2690501	63	76.1773281	before 20
76.8457846	68.9030219	75.3551261	85.2690501	63	76.4273281	before 21
78.8457846	68.9030219	75.3551261	86.2690501	63	76.8023281	before 22
78.8457846	69.9030219	75.3551261	86.2690501	63	77.0523281	before 23
78.8457846	69.9030219	74.3551261	87.2690501	63	77.1773281	before 24
78.8457846	69.9030219	73.3551261	87.2690501	63	77.0523281	before 25
78.8457846	69.9030219	71.3551261	87.2690501	63	77.0523281	before 26
77.8457846	69.9030219	70.3551261	86.2690501	63	76.5523281	before 27
78.8457846	69.9030219	69.3551261	86.2690501	63	76.8023281	before 28
76.8457846	69.9030219	69.3551261	85.2690501	63	76.5523281	before 29
74.8457846	69.9030219	69.3551261	85.2690501	63	76.6773281	before 30
73.8457846	68.9030219	69.3551261	85.2690501	63	76.3023281	before 31

73.8457846	68.9030219	69.3551261	84.2690501	63	76.4273281	before 32
74.8457846	83.9030219	69.3551261	82.2690501	63	78.3023281	before 33
74.8457846	84.9030219	69.3551261	82.2690501	63	78.4273281	before 34
75.8457846	84.9030219	69.3551261	81.2690501	63	78.4273281	before 35
77.8457846	80.9030219	69.3551261	80.2690501	63	78.4273281	before 36
79.8457846	75.9030219	69.3551261	80.2690501	63	78.4273281	before 37
80.8457846	72.9030219	69.3551261	81.2690501	63	78.6773281	before 38
80.8457846	71.9030219	70.3551261	81.2690501	63	79.0523281	before 39
80.8457846	71.9030219	70.3551261	81.2690501	63	79.0523281	before 40
79.8457846	71.9030219	70.3551261	81.2690501	63	80.0523281	before 41
79.8457846	70.9030219	71.3551261	81.2690501	63	79.9273281	before 42
79.8457846	68.9030219	71.3551261	79.2690501	63	79.1773281	before 43
79.8457846	68.9030219	71.3551261	75.2690501	63	78.5523281	before 44
77.8457846	67.9030219	71.3551261	75.2690501	63	78.5523281	before 45
76.8457846	67.9030219	71.3551261	75.2690501	63	78.5523281	before 46
76.8457846	67.9030219	72.3551261	76.2690501	63	78.4273281	before 47
76.8457846	66.9030219	72.3551261	77.2690501	63	78.4273281	before 48
77.8457846	66.9030219	73.3551261	78.2690501	63	78.3023281	before 49
77.8457846	66.9030219	73.3551261	78.2690501	63	78.0523281	before 50
77.8457846	66.9030219	73.3551261	78.2690501	63	77.6773281	before 51
82.8457846	66.9030219	73.3551261	78.2690501	63	77.9273281	before 52
83.8457846	66.9030219	73.3551261	78.2690501	63	78.0523281	before 53
83.8457846	66.9030219	73.3551261	78.2690501	63	77.8023281	before 54
83.8457846	67.9030219	73.3551261	78.2690501	63	77.6773281	before 55
84.8457846	67.9030219	73.3551261	78.2690501	63	77.5523281	before 56
84.8457846	66.9030219	73.3551261	78.2690501	63	77.3023281	before 57
84.8457846	66.9030219	73.3551261	77.2690501	63	76.9273281	before 58
87.8457846	66.9030219	73.3551261	77.2690501	63	77.5523281	before 59
87.8457846	67.9030219	73.3551261	77.2690501	63	77.5523281	before 60
86.8457846	67.9030219	73.3551261	77.2690501	63	76.8023281	before 61
86.8457846	68.9030219	73.3551261	78.2690501	63	76.9273281	before 62
86.8457846	70.9030219	73.3551261	78.2690501	63	77.0523281	before 63
84.8457846	70.9030219	74.3551261	78.2690501	63	76.6773281	before 64
82.8457846	71.9030219	75.3551261	78.2690501	63	76.6773281	before 65
81.8457846	71.9030219	75.3551261	77.2690501	63	76.4273281	before 66
81.8457846	71.9030219	75.3551261	76.2690501	63	76.1773281	before 67
81.8457846	69.9030219	76.3551261	77.2690501	63	76.3023281	before 68
81.8457846	69.9030219	76.3551261	77.2690501	63	76.3023281	before 69
81.8457846	68.9030219	76.3551261	77.2690501	63	75.9273281	before 70
80.8457846	67.9030219	76.3551261	78.2690501	63	75.6773281	before 71
79.8457846	67.9030219	76.3551261	79.2690501	63	75.6773281	before 72
79.8457846	66.9030219	77.3551261	79.2690501	63	75.6773281	before 73
79.8457846	66.9030219	78.3551261	80.2690501	63	75.9273281	before 74
79.8457846	66.9030219	77.3551261	80.2690501	63	75.8023281	before 75
79.8457846	68.9030219	77.3551261	80.2690501	63	75.5523281	before 76

79.8457846	68.9030219	75.3551261	80.2690501	63	75.4273281	before 77
81.8457846	68.9030219	74.3551261	80.2690501	63	75.6773281	before 78
81.8457846	68.9030219	74.3551261	80.2690501	63	75.5523281	before 79
81.8457846	68.9030219	74.3551261	79.2690501	63	75.5523281	before 80
81.8457846	69.9030219	74.3551261	78.2690501	63	75.5523281	before 81
81.8457846	69.9030219	74.3551261	78.2690501	63	75.6773281	before 82
81.8457846	69.9030219	75.3551261	78.2690501	63	75.6773281	before 83
81.8457846	69.9030219	79.3551261	78.2690501	63	76.0523281	before 84
81.8457846	69.9030219	79.3551261	78.2690501	63	75.9273281	before 85
81.8457846	69.9030219	80.3551261	77.2690501	63	75.8023281	before 86
80.8457846	69.9030219	80.3551261	78.2690501	63	76.1773281	before 87
80.8457846	69.9030219	80.3551261	78.2690501	63	76.5523281	before 88
79.8457846	69.9030219	83.3551261	78.2690501	63	76.6773281	before 89
79.8457846	69.9030219	88.3551261	78.2690501	63	77.1773281	before 90
79.8457846	70.9030219	89.3551261	78.2690501	63	77.5523281	before 91
80.8457846	73.9030219	90.3551261	78.2690501	63	78.4273281	before 92
81.8457846	73.9030219	92.3551261	78.2690501	63	79.0523281	before 93
81.8457846	73.9030219	92.3551261	78.2690501	63	79.0523281	before 94
81.8457846	73.9030219	96.3551261	78.2690501	63	79.8023281	before 95
81.8457846	73.9030219	92.3551261	78.2690501	63	79.4273281	before 96
81.8457846	73.9030219	84.3551261	78.2690501	63	78.5523281	before 97
81.8457846	73.9030219	84.3551261	87.2690501	63	79.4273281	before 98
80.8457846	73.9030219	78.3551261	89.2690501	63	78.6773281	before 99
80.8457846	72.9030219	77.3551261	90.2690501	63	78.1773281	before 100
80.8457846	72.9030219	76.3551261	91.2690501	63	78.3023281	before 101
80.8457846	72.9030219	75.3551261	91.2690501	63	78.0523281	before 102
80.8457846	72.9030219	74.3551261	91.2690501	63	77.8023281	before 103
80.8457846	72.9030219	73.3551261	91.2690501	63	77.5523281	before 104
80.8457846	72.9030219	73.3551261	91.2690501	63	77.4273281	before 105
79.8457846	73.9030219	71.3551261	91.2690501	63	77.0523281	before 106
79.8457846	73.9030219	71.3551261	93.2690501	63	77.1773281	before 107
77.8457846	73.9030219	71.3551261	94.2690501	63	76.8023281	before 108
77.8457846	71.9030219	71.3551261	96.2690501	63	76.8023281	before 109
77.8457846	71.9030219	71.3551261	96.2690501	63	76.8023281	before 110
77.8457846	71.9030219	71.3551261	99.2690501	63	77.0523281	before 111
80.8457846	71.9030219	71.3551261	98.2690501	63	77.8023281	before 112
82.8457846	70.9030219	70.3551261	97.2690501	63	78.0523281	before 113
82.8457846	68.9030219	69.3551261	96.2690501	63	77.6773281	before 114
82.8457846	67.9030219	68.3551261	95.2690501	63	77.3023281	before 115
83.8457846	66.9030219	69.3551261	95.2690501	63	77.5523281	before 116
82.8457846	66.9030219	70.3551261	95.2690501	63	77.6773281	before 117
82.8457846	66.9030219	70.3551261	90.2690501	63	77.1773281	before 118
82.8457846	66.9030219	70.3551261	87.2690501	63	76.8023281	before 119
79.8457846	66.9030219	70.3551261	86.2690501	63	76.3023281	before 120
79.8457846	65.9030219	70.3551261	85.2690501	63	76.1773281	before 121

79.8457846	65.9030219	70.3551261	84.2690501	63	75.8023281	before 122
79.8457846	65.9030219	72.3551261	83.2690501	63	75.8023281	before 123
79.8457846	66.9030219	72.3551261	81.2690501	63	75.8023281	before 124
79.8457846	66.9030219	72.3551261	81.2690501	63	75.6773281	before 125
79.8457846	68.9030219	72.3551261	81.2690501	63	75.9273281	before 126
79.8457846	69.9030219	72.3551261	81.2690501	63	76.0523281	before 127
78.8457846	70.9030219	72.3551261	80.2690501	63	75.9273281	before 128
78.8457846	71.9030219	72.3551261	80.2690501	63	76.3023281	before 129
78.8457846	71.9030219	75.3551261	81.2690501	63	77.0523281	before 130
74.8457846	71.9030219	76.3551261	80.2690501	63	76.6773281	before 131
72.8457846	71.9030219	76.3551261	81.2690501	63	76.6773281	before 132
72.8457846	70.9030219	76.3551261	81.2690501	63	76.9273281	before 133
74.8457846	68.9030219	76.3551261	81.2690501	63	77.1773281	before 134
76.8457846	67.9030219	76.3551261	81.2690501	63	77.9273281	before 135
78.8457846	67.9030219	76.3551261	82.2690501	63	78.8023281	before 136
79.8457846	67.9030219	76.3551261	81.2690501	63	78.8023281	before 137
78.8457846	68.9030219	77.3551261	81.2690501	63	79.0523281	before 138
76.8457846	68.9030219	77.3551261	81.2690501	63	78.8023281	before 139
75.8457846	67.9030219	77.3551261	81.2690501	63	78.4273281	before 140
74.8457846	65.9030219	79.3551261	81.2690501	63	77.9273281	before 141
75.8457846	65.9030219	82.3551261	81.2690501	63	78.4273281	before 142
75.8457846	66.9030219	84.3551261	81.2690501	63	78.9273281	before 143
76.8457846	66.9030219	84.3551261	81.2690501	63	79.0523281	before 144
76.8457846	66.9030219	82.3551261	81.2690501	63	78.9273281	before 145
76.8457846	66.9030219	80.3551261	81.2690501	63	78.8023281	before 146
76.8457846	66.9030219	77.3551261	81.2690501	63	77.6773281	before 147
76.8457846	66.9030219	77.3551261	81.2690501	63	77.6773281	before 148
76.8457846	66.9030219	76.3551261	81.2690501	63	77.3023281	before 149
77.8457846	67.9030219	75.3551261	81.2690501	63	77.3023281	before 150
77.8457846	69.9030219	75.3551261	81.2690501	63	77.6773281	before 151
77.8457846	69.9030219	74.3551261	80.2690501	63	77.5523281	before 152
77.8457846	67.9030219	73.3551261	80.2690501	63	77.4273281	before 153
76.8457846	67.9030219	73.3551261	80.2690501	63	77.8023281	before 154
76.8457846	66.9030219	73.3551261	80.2690501	63	77.8023281	before 155
76.8457846	66.9030219	73.3551261	82.2690501	63	77.6773281	before 156
76.8457846	66.9030219	73.3551261	82.2690501	63	77.9273281	before 157
73.8457846	66.9030219	73.3551261	83.2690501	63	77.4273281	before 158
72.8457846	66.9030219	73.3551261	84.2690501	63	77.1773281	before 159
71.8457846	66.9030219	74.3551261	84.2690501	63	77.1773281	before 160
70.8457846	67.9030219	74.3551261	84.2690501	63	76.6773281	before 161
70.8457846	67.9030219	74.3551261	84.2690501	63	76.4273281	before 162
70.8457846	67.9030219	74.3551261	84.2690501	63	76.4273281	before 163
69.8457846	67.9030219	76.3551261	84.2690501	63	76.4273281	before 164
69.8457846	67.9030219	81.3551261	84.2690501	63	76.9273281	before 165
73.8457846	68.9030219	81.3551261	84.2690501	63	77.1773281	before 166

76.8457846	68.9030219	82.3551261	83.2690501	63	77.3023281	before 167
75.8457846	68.9030219	82.3551261	83.2690501	63	76.8023281	before 168
75.8457846	69.9030219	82.3551261	83.2690501	63	76.3023281	before 169
75.8457846	70.9030219	82.3551261	83.2690501	63	76.5523281	before 170
67.8457846	70.9030219	83.3551261	83.2690501	69	75.9273281	before 171
67.8457846	71.9030219	84.3551261	82.2690501	69	76.1773281	before 172
67.8457846	71.9030219	84.3551261	82.2690501	69	76.5523281	before 173
67.8457846	71.9030219	83.3551261	82.2690501	69	76.9273281	before 174
68.8457846	71.9030219	83.3551261	83.2690501	69	77.3023281	before 175
67.8457846	71.9030219	83.3551261	82.2690501	69	77.4273281	before 176
67.8457846	72.9030219	83.3551261	82.2690501	69	78.1773281	before 177
67.8457846	75.9030219	81.3551261	82.2690501	69	78.6773281	before 178
68.8457846	75.9030219	81.3551261	82.2690501	69	78.4273281	before 179
68.8457846	74.9030219	80.3551261	82.2690501	69	78.0523281	before 180
69.8457846	68.9030219	79.3551261	81.2690501	69	76.8023281	before 181
69.8457846	68.9030219	79.3551261	81.2690501	69	76.5523281	before 182
69.8457846	68.9030219	78.3551261	81.2690501	69	76.0523281	before 183
69.8457846	68.9030219	77.3551261	79.2690501	69	75.5523281	before 184
70.8457846	68.9030219	77.3551261	79.2690501	69	75.8023281	before 185
70.8457846	68.9030219	71.3551261	78.2690501	69	74.8023281	before 186
70.8457846	68.9030219	69.3551261	74.2690501	69	74.1773281	before 187
70.8457846	68.9030219	68.3551261	73.2690501	69	73.9273281	before 188
70.8457846	68.9030219	67.3551261	73.2690501	69	73.9273281	before 189
71.8457846	69.9030219	68.3551261	83.2690501	69	75.5523281	before 190
71.8457846	69.9030219	68.3551261	85.2690501	69	75.9273281	before 191
71.8457846	68.9030219	68.3551261	85.2690501	69	75.8023281	before 192
72.8457846	68.9030219	68.3551261	87.2690501	69	75.6773281	before 193
72.8457846	68.9030219	68.3551261	87.2690501	69	75.4273281	before 194
73.8457846	67.9030219	68.3551261	87.2690501	69	75.1773281	before 195
73.8457846	67.9030219	68.3551261	85.2690501	69	74.6773281	before 196
73.8457846	66.9030219	69.3551261	85.2690501	69	74.8023281	before 197
80.8457846	66.9030219	69.3551261	85.2690501	69	75.8023281	before 198
80.8457846	66.9030219	69.3551261	85.2690501	69	75.9273281	before 199
82.8457846	66.9030219	69.3551261	85.2690501	69	76.1773281	before 200
82.8457846	66.9030219	69.3551261	85.2690501	63	76.0523281	before 201
82.8457846	66.9030219	69.3551261	85.2690501	63	76.0523281	before 202
82.8457846	66.9030219	70.3551261	84.2690501	63	75.1773281	before 203
83.8457846	66.9030219	70.3551261	84.2690501	63	74.3023281	before 204
84.8457846	66.9030219	70.3551261	84.2690501	63	74.6773281	before 205
84.8457846	66.9030219	70.3551261	84.2690501	63	74.8023281	before 206
84.8457846	66.9030219	71.3551261	84.2690501	63	75.0523281	before 207
85.8457846	66.9030219	71.3551261	81.2690501	63	74.8023281	before 208
85.8457846	67.9030219	71.3551261	80.2690501	63	74.5523281	before 209
84.8457846	67.9030219	71.3551261	80.2690501	63	74.5523281	before 210
81.8457846	67.9030219	71.3551261	79.2690501	63	74.3023281	before 211

81.8457846	67.9030219	71.3551261	78.2690501	63	74.3023281	before 212
70.8457846	67.9030219	71.3551261	74.2690501	63	72.6773281	before 213
69.8457846	67.9030219	72.3551261	74.2690501	63	73.1773281	before 214
69.8457846	68.9030219	73.3551261	72.2690501	63	73.3023281	before 215
69.8457846	68.9030219	73.3551261	72.2690501	63	73.3023281	before 216
69.8457846	69.9030219	75.3551261	76.2690501	63	75.0523281	before 217
69.8457846	71.9030219	76.3551261	80.2690501	63	75.8023281	before 218
69.8457846	71.9030219	76.3551261	81.2690501	63	75.8023281	before 219
70.8457846	74.9030219	74.3551261	80.2690501	63	76.1773281	before 220
71.8457846	75.9030219	73.3551261	81.2690501	63	77.4273281	before 221
72.8457846	75.9030219	72.3551261	83.2690501	63	78.1773281	before 222
72.8457846	74.9030219	71.3551261	85.2690501	63	78.3023281	before 223
73.8457846	74.9030219	71.3551261	87.2690501	63	78.5523281	before 224
72.8457846	74.9030219	71.3551261	88.2690501	63	78.6773281	before 225
71.8457846	75.9030219	71.3551261	88.2690501	63	78.4273281	before 226
71.8457846	78.9030219	71.3551261	89.2690501	63	78.8023281	before 227
71.8457846	79.9030219	71.3551261	90.2690501	63	79.0523281	before 228
71.8457846	79.9030219	72.3551261	92.2690501	63	79.3023281	before 229
71.8457846	79.9030219	72.3551261	92.2690501	63	79.1773281	before 230
71.8457846	79.9030219	73.3551261	93.2690501	63	79.8023281	before 231
71.8457846	78.9030219	73.3551261	93.2690501	63	79.9273281	before 232
72.8457846	77.9030219	73.3551261	93.2690501	63	79.6773281	before 233
72.8457846	77.9030219	72.3551261	93.2690501	63	79.3023281	before 234
73.8457846	77.9030219	72.3551261	92.2690501	63	79.0523281	before 235
73.8457846	77.9030219	72.3551261	87.2690501	63	78.4273281	before 236
74.8457846	76.9030219	72.3551261	85.2690501	63	78.1773281	before 237
74.8457846	74.9030219	71.3551261	84.2690501	63	77.6773281	before 238
75.8457846	74.9030219	70.3551261	85.2690501	63	77.8023281	before 239
75.8457846	75.9030219	69.3551261	86.2690501	63	77.9273281	before 240
74.8457846	75.9030219	69.3551261	87.2690501	63	77.4273281	minute gap 1
74.8457846	76.9030219	69.3551261	87.2690501	63	77.8023281	minute gap 2
73.8457846	77.9030219	68.3551261	87.2690501	63	77.9273281	minute gap 3
73.8457846	77.9030219	67.3551261	86.2690501	63	77.5523281	minute gap 4
74.8457846	76.9030219	66.3551261	82.2690501	63	76.9273281	minute gap 5
74.8457846	76.9030219	65.3551261	81.2690501	63	76.9273281	minute gap 6
74.8457846	76.9030219	65.3551261	81.2690501	63	77.0523281	minute gap 7
73.8457846	76.9030219	65.3551261	81.2690501	63	77.1773281	minute gap 8
72.8457846	76.9030219	66.3551261	83.2690501	63	77.4273281	minute gap 9
71.8457846	75.9030219	66.3551261	84.2690501	63	77.4273281	minute gap 10
68.8457846	71.9030219	67.3551261	85.2690501	63	77.0523281	minute gap 11
68.8457846	71.9030219	67.3551261	85.2690501	63	77.3023281	minute gap 12
68.8457846	67.9030219	66.3551261	86.2690501	63	76.9273281	minute gap 13
68.8457846	65.9030219	66.3551261	86.2690501	63	76.9273281	minute gap 14
68.8457846	64.9030219	66.3551261	86.2690501	63	77.0523281	minute gap 15
68.8457846	64.9030219	66.3551261	86.2690501	63	77.0523281	minute gap 16

68.8457846	63.9030219	68.3551261	88.2690501	63	78.0523281	minute gap 17
69.8457846	63.9030219	68.3551261	89.2690501	63	78.6773281	minute gap 18
71.8457846	62.9030219	70.3551261	89.2690501	63	79.5523281	minute gap 19
72.8457846	62.9030219	70.3551261	89.2690501	63	79.5523281	minute gap 20
72.8457846	62.9030219	70.3551261	88.2690501	63	79.8023281	minute gap 21
72.8457846	63.9030219	71.3551261	87.2690501	63	80.0523281	minute gap 22
70.8457846	67.9030219	71.3551261	87.2690501	63	80.4273281	minute gap 23
68.8457846	68.9030219	71.3551261	87.2690501	63	78.5523281	minute gap 24
68.8457846	68.9030219	71.3551261	85.2690501	63	78.8023281	minute gap 25
68.8457846	69.9030219	70.3551261	85.2690501	63	77.9273281	minute gap 26
69.8457846	69.9030219	70.3551261	86.2690501	63	78.0523281	minute gap 27
70.8457846	69.9030219	70.3551261	85.2690501	63	77.9273281	minute gap 28
71.8457846	69.9030219	70.3551261	85.2690501	63	77.8023281	minute gap 29
71.8457846	69.9030219	70.3551261	85.2690501	63	77.8023281	minute gap 30
71.8457846	69.9030219	71.3551261	83.2690501	63	77.4273281	minute gap 31
71.8457846	69.9030219	72.3551261	81.2690501	63	76.8023281	minute gap 32
72.8457846	69.9030219	72.3551261	79.2690501	63	75.9273281	minute gap 33
73.8457846	68.9030219	72.3551261	78.2690501	63	75.5523281	minute gap 34
73.8457846	68.9030219	74.3551261	78.2690501	63	75.5523281	minute gap 35
73.8457846	66.9030219	74.3551261	78.2690501	63	75.0523281	minute gap 36
74.8457846	65.9030219	74.3551261	77.2690501	63	74.6773281	minute gap 37
75.8457846	64.9030219	74.3551261	77.2690501	63	74.4273281	minute gap 38
75.8457846	63.9030219	75.3551261	77.2690501	63	74.3023281	minute gap 39
74.8457846	63.9030219	75.3551261	77.2690501	63	75.1773281	minute gap 40
72.8457846	63.9030219	74.3551261	77.2690501	63	74.6773281	minute gap 41
70.8457846	63.9030219	73.3551261	77.2690501	63	73.8023281	minute gap 42
69.8457846	64.9030219	73.3551261	77.2690501	63	73.5523281	minute gap 43
69.8457846	64.9030219	73.3551261	77.2690501	63	72.8023281	minute gap 44
69.8457846	64.9030219	73.3551261	77.2690501	63	72.8023281	minute gap 45
69.8457846	64.9030219	71.3551261	77.2690501	63	72.6773281	minute gap 46
67.8457846	65.9030219	71.3551261	77.2690501	63	72.3023281	minute gap 47
67.8457846	65.9030219	71.3551261	78.2690501	63	72.0523281	minute gap 48
67.8457846	65.9030219	71.3551261	79.2690501	63	72.0523281	minute gap 49
67.8457846	65.9030219	71.3551261	79.2690501	63	72.1773281	minute gap 50
68.8457846	65.9030219	71.3551261	79.2690501	63	73.0523281	minute gap 51
68.8457846	65.9030219	70.3551261	78.2690501	63	75.6773281	minute gap 52
71.8457846	65.9030219	69.3551261	76.2690501	63	77.3023281	minute gap 53
71.8457846	65.9030219	69.3551261	74.2690501	63	77.8023281	minute gap 54
70.8457846	66.9030219	69.3551261	73.2690501	63	77.9273281	minute gap 55
70.8457846	67.9030219	69.3551261	73.2690501	63	78.0523281	minute gap 56
69.8457846	67.9030219	69.3551261	73.2690501	63	78.0523281	minute gap 57
70.8457846	67.9030219	70.3551261	75.2690501	63	78.6773281	minute gap 58
71.8457846	67.9030219	70.3551261	79.2690501	63	79.1773281	minute gap 59
71.8457846	67.9030219	70.3551261	81.2690501	63	79.0523281	minute gap 60
73.8457846	67.9030219	70.3551261	81.2690501	73	79.1773281	Activity

73.8457846	67.9030219	70.3551261	83.2690501	73	79.8023281	Activity
74.8457846	70.9030219	70.3551261	80.2690501	73	79.6773281	Activity
74.8457846	72.9030219	70.3551261	78.2690501	73	77.5523281	Activity
74.8457846	73.9030219	70.3551261	78.2690501	73	77.4273281	Activity
75.8457846	74.9030219	70.3551261	77.2690501	73	77.4273281	Activity
75.8457846	75.9030219	69.3551261	76.2690501	73	76.9273281	Activity
75.8457846	76.9030219	69.3551261	76.2690501	73	76.9273281	Activity
75.8457846	76.9030219	69.3551261	77.2690501	73	76.1773281	Activity
75.8457846	76.9030219	69.3551261	77.2690501	73	76.1773281	Activity
75.8457846	77.9030219	68.3551261	77.2690501	73	75.5523281	Activity
75.8457846	77.9030219	68.3551261	77.2690501	73	75.3023281	Activity
75.8457846	78.9030219	68.3551261	77.2690501	73	75.5523281	Activity
75.8457846	78.9030219	68.3551261	77.2690501	73	76.1773281	Activity
75.8457846	80.9030219	68.3551261	78.2690501	73	76.3023281	Activity
76.8457846	81.9030219	68.3551261	78.2690501	73	76.1773281	Activity
76.8457846	81.9030219	68.3551261	78.2690501	73	76.3023281	Activity
76.8457846	81.9030219	69.3551261	78.2690501	73	76.4273281	Activity
74.8457846	81.9030219	70.3551261	79.2690501	73	76.1773281	Activity
74.8457846	79.9030219	70.3551261	80.2690501	73	76.1773281	Activity
74.8457846	79.9030219	70.3551261	80.2690501	73	76.4273281	Activity
74.8457846	79.9030219	70.3551261	72.2690501	73	75.6773281	Activity
74.8457846	80.9030219	70.3551261	72.2690501	73	75.8023281	Activity
73.8457846	80.9030219	70.3551261	72.2690501	73	75.5523281	Activity
72.8457846	81.9030219	70.3551261	72.2690501	73	75.4273281	Activity
71.8457846	82.9030219	70.3551261	72.2690501	73	75.6773281	Activity
71.8457846	83.9030219	70.3551261	72.2690501	73	75.9273281	Activity
70.8457846	84.9030219	70.3551261	73.2690501	73	76.4273281	Activity
71.8457846	86.9030219	70.3551261	73.2690501	73	76.9273281	Activity
71.8457846	88.9030219	70.3551261	73.2690501	73	77.6773281	Activity
72.8457846	89.9030219	72.3551261	75.2690501	73	78.5523281	Activity
72.8457846	89.9030219	73.3551261	75.2690501	73	78.3023281	Activity
71.8457846	90.9030219	73.3551261	77.2690501	73	78.5523281	Activity
72.8457846	92.9030219	72.3551261	77.2690501	73	78.5523281	Activity
73.8457846	96.9030219	72.3551261	78.2690501	73	79.4273281	Activity
73.8457846	100.903022	72.3551261	79.2690501	73	80.4273281	Activity
73.8457846	100.903022	71.3551261	79.2690501	73	80.5523281	Activity
74.8457846	100.903022	71.3551261	79.2690501	73	80.9273281	Activity
74.8457846	100.903022	70.3551261	77.2690501	73	80.5523281	Activity
74.8457846	99.9030219	69.3551261	77.2690501	73	80.3023281	Activity
74.8457846	98.9030219	69.3551261	76.2690501	73	80.0523281	Activity
80.8457846	98.9030219	68.3551261	76.2690501	73	80.6773281	Activity
81.8457846	98.9030219	69.3551261	76.2690501	73	80.9273281	Activity
78.8457846	98.9030219	69.3551261	76.2690501	73	80.4273281	Activity
76.8457846	97.9030219	69.3551261	76.2690501	73	79.5523281	Activity
76.8457846	97.9030219	69.3551261	77.2690501	73	79.6773281	Activity

76.8457846	89.9030219	69.3551261	76.2690501	73	78.4273281	Activity
76.8457846	88.9030219	70.3551261	76.2690501	73	78.0523281	Activity
76.8457846	88.9030219	70.3551261	76.2690501	73	78.3023281	Activity
76.8457846	87.9030219	70.3551261	75.2690501	73	78.1773281	Activity
76.8457846	87.9030219	70.3551261	76.2690501	73	78.0523281	Activity
76.8457846	85.9030219	70.3551261	77.2690501	73	77.9273281	Activity
74.8457846	88.9030219	70.3551261	77.2690501	73	77.9273281	Activity
74.8457846	89.9030219	70.3551261	77.2690501	73	78.1773281	Activity
68.8457846	91.9030219	71.3551261	77.2690501	73	77.8023281	Activity
67.8457846	92.9030219	71.3551261	78.2690501	73	77.9273281	Activity
67.8457846	93.9030219	74.3551261	78.2690501	73	79.1773281	Activity
66.8457846	94.9030219	75.3551261	78.2690501	73	79.4273281	Activity
66.8457846	94.9030219	75.3551261	78.2690501	73	79.3023281	Activity
66.8457846	94.9030219	75.3551261	79.2690501	73	80.5523281	Activity
66.8457846	95.9030219	75.3551261	79.2690501	73	81.3023281	Activity
66.8457846	96.9030219	74.3551261	80.2690501	73	81.3023281	Activity
71.8457846	96.9030219	74.3551261	80.2690501	73	81.8023281	Activity
71.8457846	96.9030219	74.3551261	80.2690501	73	81.8023281	Activity
72.8457846	97.9030219	73.3551261	84.2690501	73	82.5523281	Activity
72.8457846	97.9030219	73.3551261	85.2690501	73	82.4273281	Activity
72.8457846	97.9030219	73.3551261	85.2690501	73	82.3023281	Activity
72.8457846	97.9030219	73.3551261	86.2690501	73	82.0523281	Activity
72.8457846	97.9030219	73.3551261	76.2690501	73	80.4273281	Activity
73.8457846	97.9030219	73.3551261	76.2690501	73	80.5523281	Activity
77.8457846	97.9030219	73.3551261	76.2690501	73	80.9273281	Activity
77.8457846	97.9030219	73.3551261	77.2690501	73	80.9273281	Activity
80.8457846	98.9030219	74.3551261	77.2690501	73	81.6773281	Activity
82.8457846	98.9030219	74.3551261	77.2690501	73	82.0523281	Activity
83.8457846	98.9030219	74.3551261	76.2690501	73	82.1773281	Activity
84.8457846	98.9030219	75.3551261	74.2690501	73	82.1773281	Activity
85.8457846	98.9030219	78.3551261	74.2690501	73	80.8023281	Activity
85.8457846	98.9030219	78.3551261	74.2690501	73	80.5523281	Activity
85.8457846	98.9030219	78.3551261	74.2690501	73	80.0523281	Activity
85.8457846	98.9030219	77.3551261	74.2690501	73	79.9273281	Activity
85.8457846	99.9030219	77.3551261	74.2690501	73	79.5523281	Activity
83.8457846	99.9030219	77.3551261	75.2690501	73	79.4273281	Activity
83.8457846	99.9030219	77.3551261	75.2690501	73	79.3023281	Activity
83.8457846	97.9030219	77.3551261	76.2690501	73	79.4273281	Activity
82.8457846	97.9030219	77.3551261	76.2690501	73	79.8023281	Activity
82.8457846	97.9030219	77.3551261	75.2690501	73	80.0523281	Activity
82.8457846	98.9030219	78.3551261	75.2690501	73	80.5523281	Activity
81.8457846	97.9030219	79.3551261	75.2690501	73	80.1773281	Activity
81.8457846	97.9030219	79.3551261	75.2690501	73	81.8023281	Activity
81.8457846	94.9030219	80.3551261	75.2690501	73	81.9273281	Activity
81.8457846	94.9030219	80.3551261	75.2690501	73	82.0523281	Activity

81.8457846	94.9030219	83.3551261	75.2690501	73	82.5523281	Activity
81.8457846	94.9030219	85.3551261	75.2690501	73	82.3023281	Activity
81.8457846	93.9030219	85.3551261	77.2690501	73	82.5523281	Activity
81.8457846	93.9030219	83.3551261	77.2690501	73	82.3023281	Activity
81.8457846	83.9030219	81.3551261	77.2690501	73	80.6773281	Activity
81.8457846	83.9030219	80.3551261	77.2690501	73	80.5523281	Activity
80.8457846	83.9030219	78.3551261	77.2690501	73	80.4273281	Activity
80.8457846	83.9030219	77.3551261	77.2690501	73	80.3023281	Activity
81.8457846	83.9030219	77.3551261	77.2690501	73	79.8023281	Activity
81.8457846	83.9030219	76.3551261	77.2690501	73	79.9273281	Activity
84.8457846	83.9030219	76.3551261	79.2690501	73	80.0523281	Activity
84.8457846	82.9030219	75.3551261	79.2690501	73	79.8023281	Activity
84.8457846	82.9030219	76.3551261	81.2690501	73	80.5523281	Activity
84.8457846	83.9030219	76.3551261	81.2690501	73	80.8023281	Activity
84.8457846	82.9030219	77.3551261	86.2690501	73	81.5523281	Activity
85.8457846	81.9030219	78.3551261	90.2690501	73	82.1773281	Activity
85.8457846	81.9030219	77.3551261	91.2690501	73	82.4273281	Activity
81.8457846	80.9030219	75.3551261	91.2690501	73	81.4273281	Activity
76.8457846	79.9030219	74.3551261	91.2690501	73	80.4273281	Activity
75.8457846	76.9030219	74.3551261	92.2690501	73	80.1773281	Activity
75.8457846	76.9030219	74.3551261	92.2690501	73	80.1773281	Activity
74.8457846	73.9030219	74.3551261	92.2690501	73	79.4273281	Activity
73.8457846	74.9030219	75.3551261	92.2690501	73	79.9273281	Activity
73.8457846	74.9030219	75.3551261	95.2690501	73	80.4273281	Activity
72.8457846	74.9030219	75.3551261	96.2690501	73	80.4273281	Activity
71.8457846	74.9030219	76.3551261	99.2690501	73	80.5523281	Activity
71.8457846	74.9030219	76.3551261	99.2690501	73	80.5523281	Activity
71.8457846	74.9030219	76.3551261	100.26905	73	80.4273281	Activity
70.8457846	75.9030219	74.3551261	101.26905	73	80.1773281	Activity
70.8457846	76.9030219	72.3551261	101.26905	73	79.8023281	Activity
70.8457846	76.9030219	71.3551261	100.26905	73	79.6773281	Activity
70.8457846	75.9030219	71.3551261	100.26905	73	79.6773281	Activity
71.8457846	75.9030219	70.3551261	99.2690501	73	79.5523281	Activity
71.8457846	75.9030219	70.3551261	98.2690501	73	79.3023281	Activity
70.8457846	75.9030219	70.3551261	97.2690501	73	79.8023281	Activity
71.8457846	74.9030219	70.3551261	96.2690501	73	79.1773281	Activity
71.8457846	73.9030219	70.3551261	94.2690501	73	78.8023281	Activity
71.8457846	68.9030219	70.3551261	92.2690501	73	77.8023281	Activity
72.8457846	68.9030219	70.3551261	90.2690501	73	77.5523281	Activity
74.8457846	66.9030219	70.3551261	89.2690501	73	77.3023281	Activity
72.8457846	66.9030219	71.3551261	89.2690501	73	77.1773281	Activity
71.8457846	66.9030219	71.3551261	89.2690501	73	77.0523281	Activity
71.8457846	66.9030219	71.3551261	89.2690501	73	76.8023281	Activity
71.8457846	66.9030219	71.3551261	89.2690501	73	76.5523281	Activity
70.8457846	66.9030219	71.3551261	91.2690501	73	76.8023281	Activity

71.8457846	66.9030219	71.3551261	91.2690501	73	76.5523281	Activity
71.8457846	67.9030219	73.3551261	91.2690501	73	76.6773281	Activity
73.8457846	67.9030219	72.3551261	91.2690501	73	76.6773281	Activity
73.8457846	68.9030219	72.3551261	90.2690501	73	76.6773281	Activity
73.8457846	71.9030219	72.3551261	87.2690501	73	76.5523281	Activity
73.8457846	71.9030219	74.3551261	86.2690501	73	76.5523281	Activity
71.8457846	73.9030219	74.3551261	86.2690501	73	76.5523281	Activity
71.8457846	74.9030219	74.3551261	85.2690501	73	76.6773281	Activity
71.8457846	76.9030219	74.3551261	85.2690501	73	77.1773281	Activity
69.8457846	76.9030219	74.3551261	85.2690501	73	77.0523281	Activity
66.8457846	79.9030219	74.3551261	85.2690501	73	77.0523281	Activity
64.8457846	86.9030219	74.3551261	85.2690501	73	77.5523281	Activity
63.8457846	92.9030219	74.3551261	85.2690501	73	78.4273281	Activity
63.8457846	84.9030219	74.3551261	87.2690501	73	77.6773281	Activity
63.8457846	84.9030219	75.3551261	88.2690501	73	77.8023281	Activity
63.8457846	78.9030219	75.3551261	88.2690501	73	77.1773281	Activity
63.8457846	75.9030219	75.3551261	88.2690501	73	77.0523281	Activity
63.8457846	75.9030219	75.3551261	88.2690501	73	77.3023281	Activity
63.8457846	72.9030219	74.3551261	95.2690501	73	78.3023281	Activity
63.8457846	72.9030219	74.3551261	98.2690501	73	78.9273281	Activity
63.8457846	72.9030219	74.3551261	98.2690501	73	79.4273281	Activity
64.8457846	77.9030219	73.3551261	98.2690501	73	80.1773281	Activity
64.8457846	74.9030219	73.3551261	98.2690501	73	79.8023281	Activity
64.8457846	74.9030219	73.3551261	98.2690501	73	79.6773281	Activity
64.8457846	72.9030219	75.3551261	96.2690501	73	79.4273281	Activity
64.8457846	70.9030219	75.3551261	95.2690501	73	79.5523281	Activity
64.8457846	69.9030219	75.3551261	90.2690501	73	78.8023281	Activity
68.8457846	69.9030219	75.3551261	88.2690501	73	78.4273281	Activity
67.8457846	69.9030219	75.3551261	88.2690501	73	78.1773281	Activity
67.8457846	69.9030219	75.3551261	88.2690501	73	78.1773281	Activity
67.8457846	71.9030219	75.3551261	87.2690501	73	78.3023281	Activity
67.8457846	71.9030219	74.3551261	87.2690501	73	78.3023281	Activity
69.8457846	72.9030219	74.3551261	86.2690501	73	78.5523281	Activity
70.8457846	73.9030219	74.3551261	80.2690501	73	78.5523281	Activity
70.8457846	76.9030219	74.3551261	79.2690501	73	78.8023281	Activity
70.8457846	76.9030219	74.3551261	79.2690501	73	78.8023281	Activity
71.8457846	77.9030219	74.3551261	78.2690501	73	78.6773281	Activity
72.8457846	77.9030219	71.3551261	78.2690501	73	78.3023281	Activity
72.8457846	76.9030219	71.3551261	81.2690501	73	78.3023281	Activity
73.8457846	75.9030219	73.3551261	81.2690501	73	77.9273281	Activity
73.8457846	74.9030219	73.3551261	88.2690501	73	77.9273281	Activity
73.8457846	74.9030219	73.3551261	88.2690501	73	77.8023281	Activity
72.8457846	73.9030219	74.3551261	89.2690501	73	77.5523281	Activity
73.8457846	72.9030219	76.3551261	89.2690501	73	77.0523281	Activity
75.8457846	78.9030219	77.3551261	90.2690501	73	78.1773281	Activity

76.8457846	80.9030219	78.3551261	90.2690501	73	78.3023281	Activity
76.8457846	84.9030219	78.3551261	90.2690501	73	78.5523281	Activity
76.8457846	86.9030219	77.3551261	91.2690501	73	78.9273281	Activity
79.8457846	86.9030219	76.3551261	91.2690501	73	79.5523281	Activity
81.8457846	88.9030219	76.3551261	91.2690501	73	80.5523281	Activity
81.8457846	98.9030219	76.3551261	91.2690501	73	82.1773281	Activity
81.8457846	100.903022	76.3551261	92.2690501	73	82.5523281	Activity
81.8457846	105.903022	76.3551261	92.2690501	73	82.9273281	Activity
81.8457846	107.903022	76.3551261	92.2690501	73	82.3023281	Activity
81.8457846	108.903022	76.3551261	92.2690501	73	82.3023281	Activity
82.8457846	108.903022	77.3551261	92.2690501	73	82.1773281	Activity
82.8457846	110.903022	76.3551261	92.2690501	73	82.0523281	Activity
82.8457846	110.903022	76.3551261	91.2690501	73	82.0523281	Activity
84.8457846	110.903022	76.3551261	91.2690501	73	82.1773281	Activity
84.8457846	109.903022	75.3551261	89.2690501	73	81.5523281	Activity
86.8457846	109.903022	75.3551261	86.2690501	73	81.5523281	Activity
86.8457846	109.903022	74.3551261	86.2690501	73	81.1773281	Activity
88.8457846	109.903022	73.3551261	86.2690501	73	81.5523281	Activity
89.8457846	108.903022	73.3551261	88.2690501	73	82.3023281	Activity
90.8457846	107.903022	73.3551261	88.2690501	73	82.3023281	Activity
91.8457846	107.903022	73.3551261	88.2690501	73	82.5523281	Activity
92.8457846	106.903022	73.3551261	85.2690501	73	82.5523281	Activity
93.8457846	107.903022	73.3551261	82.2690501	73	82.5523281	Activity
93.8457846	107.903022	73.3551261	82.2690501	73	82.8023281	Activity
93.8457846	107.903022	73.3551261	82.2690501	73	83.1773281	Activity
94.8457846	107.903022	73.3551261	80.2690501	73	83.0523281	Activity
94.8457846	106.903022	73.3551261	80.2690501	73	82.6773281	Activity
94.8457846	106.903022	73.3551261	81.2690501	73	82.6773281	Activity
92.8457846	105.903022	73.3551261	84.2690501	73	82.5523281	Activity
89.8457846	104.903022	73.3551261	84.2690501	73	82.0523281	Activity
87.8457846	105.903022	73.3551261	84.2690501	73	82.0523281	Activity
85.8457846	105.903022	73.3551261	84.2690501	73	81.5523281	Activity
84.8457846	105.903022	73.3551261	84.2690501	73	81.4273281	Activity
82.8457846	105.903022	73.3551261	84.2690501	73	81.1773281	Activity
80.8457846	98.9030219	73.3551261	85.2690501	73	80.1773281	Activity
80.8457846	89.9030219	73.3551261	85.2690501	73	78.8023281	Activity
80.8457846	80.9030219	73.3551261	85.2690501	73	77.4273281	Activity
80.8457846	74.9030219	73.3551261	84.2690501	73	76.4273281	Activity
81.8457846	72.9030219	73.3551261	84.2690501	73	76.1773281	Activity
81.8457846	71.9030219	73.3551261	84.2690501	73	76.4273281	Activity
82.8457846	71.9030219	74.3551261	84.2690501	73	76.4273281	Activity
82.8457846	70.9030219	71.3551261	87.2690501	73	76.4273281	Activity
82.8457846	69.9030219	71.3551261	89.2690501	73	76.5523281	Activity
82.8457846	67.9030219	72.3551261	91.2690501	73	76.3023281	Activity
82.8457846	67.9030219	72.3551261	91.2690501	73	76.3023281	Activity

82.8457846	67.9030219	74.3551261	91.2690501	73	76.9273281	Activity
82.8457846	65.9030219	74.3551261	91.2690501	73	76.5523281	Activity
82.8457846	64.9030219	74.3551261	91.2690501	73	76.8023281	Activity
82.8457846	64.9030219	74.3551261	91.2690501	73	76.4273281	Activity
81.8457846	64.9030219	74.3551261	85.2690501	73	75.5523281	Activity
81.8457846	64.9030219	74.3551261	85.2690501	73	75.5523281	Activity
80.8457846	65.9030219	74.3551261	85.2690501	73	75.4273281	Activity
81.8457846	66.9030219	72.3551261	86.2690501	73	75.5523281	Activity
81.8457846	67.9030219	72.3551261	85.2690501	73	75.4273281	Activity
81.8457846	67.9030219	72.3551261	86.2690501	73	74.9273281	Activity
81.8457846	67.9030219	72.3551261	86.2690501	73	74.6773281	Activity
81.8457846	67.9030219	75.3551261	86.2690501	73	75.0523281	Activity
81.8457846	67.9030219	77.3551261	87.2690501	73	75.5523281	Activity
82.8457846	68.9030219	78.3551261	87.2690501	73	76.0523281	Activity
82.8457846	68.9030219	78.3551261	87.2690501	73	76.8023281	Activity
83.8457846	68.9030219	77.3551261	88.2690501	73	78.0523281	Activity
83.8457846	69.9030219	77.3551261	88.2690501	73	78.1773281	Activity
83.8457846	69.9030219	75.3551261	89.2690501	73	78.1773281	Activity
83.8457846	69.9030219	74.3551261	89.2690501	73	78.4273281	Activity
83.8457846	70.9030219	73.3551261	89.2690501	73	78.9273281	Activity
82.8457846	70.9030219	72.3551261	89.2690501	73	78.6773281	Activity
82.8457846	70.9030219	73.3551261	89.2690501	73	79.0523281	Activity
82.8457846	70.9030219	74.3551261	90.2690501	73	79.4273281	Activity
82.8457846	70.9030219	75.3551261	90.2690501	73	79.6773281	Activity
81.8457846	69.9030219	75.3551261	90.2690501	73	79.6773281	Activity
81.8457846	69.9030219	76.3551261	94.2690501	73	80.6773281	Activity
78.8457846	68.9030219	77.3551261	93.2690501	73	80.3023281	Activity
73.8457846	68.9030219	77.3551261	93.2690501	73	79.4273281	Activity
73.8457846	68.9030219	77.3551261	93.2690501	73	78.9273281	Activity
73.8457846	68.9030219	77.3551261	93.2690501	73	78.4273281	Activity
72.8457846	69.9030219	77.3551261	93.2690501	73	78.4273281	Activity
72.8457846	73.9030219	75.3551261	93.2690501	73	78.5523281	Activity
72.8457846	74.9030219	74.3551261	93.2690501	73	78.5523281	Activity
73.8457846	74.9030219	74.3551261	93.2690501	73	78.3023281	Activity
73.8457846	74.9030219	74.3551261	95.2690501	73	78.4273281	Activity
73.8457846	75.9030219	74.3551261	95.2690501	73	78.5523281	Activity
73.8457846	75.9030219	74.3551261	95.2690501	73	78.6773281	Activity
73.8457846	75.9030219	74.3551261	91.2690501	73	78.1773281	Activity
73.8457846	75.9030219	73.3551261	86.2690501	73	77.6773281	Activity
77.8457846	75.9030219	73.3551261	86.2690501	73	78.5523281	Activity
80.8457846	75.9030219	73.3551261	86.2690501	73	79.1773281	Activity
80.8457846	75.9030219	75.3551261	88.2690501	73	79.5523281	Activity
80.8457846	75.9030219	78.3551261	88.2690501	73	79.1773281	Activity
80.8457846	75.9030219	80.3551261	88.2690501	73	78.5523281	Activity
80.8457846	75.9030219	79.3551261	92.2690501	73	78.6773281	Activity

80.8457846	75.9030219	78.3551261	92.2690501	73	78.6773281	Activity
78.8457846	75.9030219	77.3551261	92.2690501	73	78.1773281	Activity
75.8457846	71.9030219	77.3551261	92.2690501	73	77.1773281	Activity
72.8457846	67.9030219	77.3551261	92.2690501	73	76.3023281	Activity
71.8457846	67.9030219	77.3551261	92.2690501	73	76.3023281	Activity
71.8457846	65.9030219	77.3551261	92.2690501	73	76.0523281	Activity
71.8457846	64.9030219	76.3551261	86.2690501	73	74.9273281	Activity
71.8457846	64.9030219	75.3551261	85.2690501	73	74.8023281	Activity
71.8457846	64.9030219	75.3551261	85.2690501	73	74.8023281	Activity
71.8457846	65.9030219	75.3551261	86.2690501	73	75.6773281	Activity
71.8457846	66.9030219	75.3551261	86.2690501	73	75.6773281	Activity
72.8457846	67.9030219	75.3551261	86.2690501	73	75.9273281	Activity
79.8457846	67.9030219	71.3551261	86.2690501	73	76.4273281	Activity
84.8457846	65.9030219	69.3551261	86.2690501	73	75.6773281	Activity
86.8457846	65.9030219	69.3551261	86.2690501	73	75.3023281	Activity
85.8457846	65.9030219	68.3551261	86.2690501	73	75.1773281	Activity
83.8457846	65.9030219	68.3551261	86.2690501	73	74.9273281	End activity
80.8457846	65.9030219	69.3551261	90.2690501	63	75.3023281	minute gap 1
78.8457846	64.9030219	69.3551261	90.2690501	63	75.5523281	minute gap 2
77.8457846	63.9030219	69.3551261	90.2690501	63	75.3023281	minute gap 3
75.8457846	63.9030219	71.3551261	85.2690501	63	74.5523281	minute gap 4
74.8457846	64.9030219	71.3551261	85.2690501	63	74.5523281	minute gap 5
73.8457846	65.9030219	71.3551261	86.2690501	63	75.4273281	minute gap 6
71.8457846	65.9030219	70.3551261	87.2690501	63	75.9273281	minute gap 7
70.8457846	66.9030219	70.3551261	87.2690501	63	76.1773281	minute gap 8
69.8457846	66.9030219	70.3551261	88.2690501	63	76.3023281	minute gap 9
68.8457846	66.9030219	70.3551261	89.2690501	63	76.0523281	minute gap 10
68.8457846	66.9030219	70.3551261	89.2690501	63	76.3023281	minute gap 11
68.8457846	66.9030219	70.3551261	93.2690501	63	76.6773281	minute gap 12
69.8457846	66.9030219	71.3551261	93.2690501	63	76.8023281	minute gap 13
69.8457846	66.9030219	71.3551261	93.2690501	63	76.1773281	minute gap 14
70.8457846	66.9030219	71.3551261	91.2690501	63	75.6773281	minute gap 15
70.8457846	66.9030219	70.3551261	84.2690501	63	73.9273281	minute gap 16
70.8457846	66.9030219	69.3551261	84.2690501	63	73.8023281	minute gap 17
71.8457846	66.9030219	69.3551261	83.2690501	63	73.6773281	minute gap 18
71.8457846	66.9030219	69.3551261	82.2690501	63	73.3023281	minute gap 19
73.8457846	66.9030219	70.3551261	81.2690501	63	73.5523281	minute gap 20
73.8457846	65.9030219	70.3551261	80.2690501	63	73.4273281	minute gap 21
73.8457846	65.9030219	71.3551261	80.2690501	63	73.5523281	minute gap 22
73.8457846	68.9030219	72.3551261	80.2690501	63	74.3023281	minute gap 23
74.8457846	71.9030219	72.3551261	79.2690501	63	74.8023281	minute gap 24
74.8457846	70.9030219	73.3551261	77.2690501	63	74.6773281	minute gap 25
74.8457846	69.9030219	73.3551261	77.2690501	63	74.8023281	minute gap 26
73.8457846	67.9030219	73.3551261	77.2690501	63	74.6773281	minute gap 27
73.8457846	66.9030219	74.3551261	76.2690501	63	74.8023281	minute gap 28

73.8457846	66.9030219	75.3551261	73.2690501	63	74.3023281	minute gap 29
75.8457846	66.9030219	76.3551261	72.2690501	63	74.1773281	minute gap 30
75.8457846	66.9030219	79.3551261	72.2690501	63	74.0523281	minute gap 31
75.8457846	65.9030219	80.3551261	72.2690501	63	73.9273281	minute gap 32
75.8457846	66.9030219	80.3551261	72.2690501	63	73.6773281	minute gap 33
74.8457846	66.9030219	81.3551261	78.2690501	63	74.3023281	minute gap 34
74.8457846	66.9030219	81.3551261	85.2690501	63	74.9273281	minute gap 35
74.8457846	66.9030219	81.3551261	85.2690501	63	75.0523281	minute gap 36
74.8457846	66.9030219	82.3551261	91.2690501	63	76.3023281	minute gap 37
74.8457846	67.9030219	82.3551261	91.2690501	63	76.5523281	minute gap 38
73.8457846	67.9030219	82.3551261	91.2690501	63	76.8023281	minute gap 39
71.8457846	67.9030219	78.3551261	89.2690501	63	76.4273281	minute gap 40
71.8457846	67.9030219	78.3551261	89.2690501	63	76.4273281	minute gap 41
71.8457846	68.9030219	77.3551261	82.2690501	63	75.6773281	minute gap 42
71.8457846	68.9030219	76.3551261	79.2690501	63	74.8023281	minute gap 43
71.8457846	68.9030219	75.3551261	78.2690501	63	74.5523281	minute gap 44
70.8457846	68.9030219	74.3551261	77.2690501	63	74.3023281	minute gap 45
70.8457846	68.9030219	74.3551261	77.2690501	63	74.3023281	minute gap 46
72.8457846	69.9030219	73.3551261	76.2690501	63	73.4273281	minute gap 47
74.8457846	69.9030219	73.3551261	76.2690501	63	73.3023281	minute gap 48
75.8457846	69.9030219	74.3551261	76.2690501	63	73.6773281	minute gap 49
75.8457846	70.9030219	77.3551261	76.2690501	63	74.1773281	minute gap 50
75.8457846	70.9030219	82.3551261	76.2690501	63	74.8023281	minute gap 51
75.8457846	70.9030219	85.3551261	79.2690501	63	75.4273281	minute gap 52
76.8457846	72.9030219	88.3551261	80.2690501	63	75.9273281	minute gap 53
77.8457846	72.9030219	90.3551261	80.2690501	63	76.1773281	minute gap 54
77.8457846	72.9030219	90.3551261	80.2690501	63	75.8023281	minute gap 55
77.8457846	72.9030219	99.3551261	80.2690501	63	76.5523281	minute gap 56
77.8457846	72.9030219	111.355126	80.2690501	63	77.8023281	minute gap 57
77.8457846	72.9030219	117.355126	80.2690501	63	79.3023281	minute gap 58
77.8457846	72.9030219	117.355126	82.2690501	63	79.8023281	minute gap 59
77.8457846	72.9030219	117.355126	89.2690501	63	80.9273281	minute gap 60
77.8457846	72.9030219	119.355126	90.2690501	63	81.3023281	After 1
77.8457846	73.9030219	119.355126	89.2690501	63	81.1773281	After 2
77.8457846	73.9030219	129.355126	88.2690501	63	81.8023281	After 3
76.8457846	76.9030219	129.355126	88.2690501	63	81.9273281	After 4
70.8457846	78.9030219	129.355126	85.2690501	63	81.1773281	After 5
70.8457846	79.9030219	134.355126	85.2690501	63	82.1773281	After 6
66.8457846	81.9030219	135.355126	85.2690501	63	82.1773281	After 7
66.8457846	83.9030219	135.355126	85.2690501	63	82.1773281	After 8
66.8457846	83.9030219	138.355126	85.2690501	63	82.3023281	After 9
66.8457846	85.9030219	137.355126	85.2690501	63	82.5523281	After 10
66.8457846	85.9030219	137.355126	85.2690501	63	82.8023281	After 11
67.8457846	86.9030219	137.355126	84.2690501	63	83.0523281	After 12
68.8457846	86.9030219	137.355126	84.2690501	63	83.4273281	After 13

70.8457846	86.9030219	106.355126	85.2690501	63	79.8023281	After 14
71.8457846	86.9030219	90.3551261	85.2690501	63	77.9273281	After 15
72.8457846	85.9030219	80.3551261	84.2690501	63	76.3023281	After 16
73.8457846	84.9030219	75.3551261	83.2690501	63	75.6773281	After 17
73.8457846	84.9030219	74.3551261	82.2690501	63	75.4273281	After 18
75.8457846	84.9030219	74.3551261	85.2690501	63	76.1773281	After 19
76.8457846	85.9030219	73.3551261	85.2690501	63	76.3023281	After 20
76.8457846	85.9030219	72.3551261	85.2690501	63	75.9273281	After 21
76.8457846	87.9030219	72.3551261	82.2690501	63	75.8023281	After 22
76.8457846	85.9030219	71.3551261	80.2690501	63	75.1773281	After 23
76.8457846	84.9030219	71.3551261	79.2690501	63	74.4273281	After 24
76.8457846	84.9030219	71.3551261	79.2690501	63	74.3023281	After 25
77.8457846	84.9030219	71.3551261	78.2690501	63	74.3023281	After 26
77.8457846	84.9030219	71.3551261	78.2690501	63	74.3023281	After 27
76.8457846	84.9030219	71.3551261	78.2690501	63	73.9273281	After 28
75.8457846	84.9030219	71.3551261	81.2690501	63	73.8023281	After 29
73.8457846	87.9030219	71.3551261	81.2690501	63	73.6773281	After 30
72.8457846	88.9030219	71.3551261	81.2690501	63	73.4273281	After 31
71.8457846	89.9030219	71.3551261	81.2690501	63	73.5523281	After 32
70.8457846	90.9030219	70.3551261	79.2690501	63	73.5523281	After 33
70.8457846	91.9030219	70.3551261	79.2690501	63	74.4273281	After 34
70.8457846	92.9030219	69.3551261	78.2690501	63	74.6773281	After 35
69.8457846	92.9030219	69.3551261	78.2690501	63	74.8023281	After 36
69.8457846	93.9030219	69.3551261	78.2690501	63	75.0523281	After 37
70.8457846	93.9030219	68.3551261	79.2690501	63	74.9273281	After 38
71.8457846	91.9030219	68.3551261	79.2690501	63	74.6773281	After 39
71.8457846	91.9030219	68.3551261	79.2690501	63	74.6773281	After 40
72.8457846	90.9030219	68.3551261	81.2690501	63	74.9273281	After 41
74.8457846	90.9030219	68.3551261	81.2690501	63	75.3023281	After 42
74.8457846	87.9030219	68.3551261	81.2690501	63	75.3023281	After 43
74.8457846	87.9030219	68.3551261	81.2690501	63	75.5523281	After 44
76.8457846	87.9030219	68.3551261	83.2690501	63	76.4273281	After 45
77.8457846	87.9030219	69.3551261	82.2690501	63	76.6773281	After 46
80.8457846	87.9030219	69.3551261	82.2690501	63	77.0523281	After 47
81.8457846	87.9030219	72.3551261	82.2690501	63	77.8023281	After 48
82.8457846	86.9030219	74.3551261	82.2690501	63	78.3023281	After 49
82.8457846	85.9030219	75.3551261	82.2690501	63	78.5523281	After 50
82.8457846	86.9030219	75.3551261	81.2690501	63	78.9273281	After 51
83.8457846	87.9030219	75.3551261	81.2690501	63	79.1773281	After 52
83.8457846	89.9030219	75.3551261	80.2690501	63	79.0523281	After 53
81.8457846	89.9030219	75.3551261	79.2690501	63	78.6773281	After 54
74.8457846	89.9030219	71.3551261	78.2690501	63	76.4273281	After 55
71.8457846	91.9030219	70.3551261	78.2690501	63	76.0523281	After 56
70.8457846	91.9030219	69.3551261	78.2690501	63	75.8023281	After 57
71.8457846	92.9030219	69.3551261	78.2690501	63	75.8023281	After 58

71.8457846	92.9030219	69.3551261	78.2690501	63	76.3023281	After 59
71.8457846	92.9030219	69.3551261	78.2690501	63	76.4273281	After 60
74.8457846	92.9030219	69.3551261	78.2690501	63	76.8023281	After 61
75.8457846	91.9030219	68.3551261	78.2690501	63	76.0523281	After 62
75.8457846	91.9030219	69.3551261	80.2690501	63	76.3023281	After 63
75.8457846	92.9030219	69.3551261	80.2690501	63	76.8023281	After 64
70.8457846	92.9030219	68.3551261	80.2690501	63	76.1773281	After 65
69.8457846	93.9030219	68.3551261	80.2690501	63	76.0523281	After 66
70.8457846	93.9030219	68.3551261	80.2690501	63	75.9273281	After 67
71.8457846	93.9030219	62.3551261	80.2690501	63	75.4273281	After 68
73.8457846	93.9030219	64.3551261	80.2690501	63	76.1773281	After 69
73.8457846	93.9030219	65.3551261	79.2690501	63	76.0523281	After 70
75.8457846	93.9030219	65.3551261	78.2690501	63	76.4273281	After 71
76.8457846	93.9030219	66.3551261	78.2690501	63	76.5523281	After 72
77.8457846	93.9030219	66.3551261	77.2690501	63	76.5523281	After 73
77.8457846	93.9030219	65.3551261	77.2690501	63	76.0523281	After 74
76.8457846	92.9030219	65.3551261	77.2690501	63	75.6773281	After 75
76.8457846	89.9030219	65.3551261	77.2690501	63	74.5523281	After 76
73.8457846	84.9030219	65.3551261	77.2690501	63	73.5523281	After 77
72.8457846	83.9030219	64.3551261	78.2690501	63	73.1773281	After 78
72.8457846	82.9030219	64.3551261	78.2690501	63	72.9273281	After 79
73.8457846	81.9030219	64.3551261	78.2690501	63	73.0523281	After 80
73.8457846	81.9030219	64.3551261	78.2690501	63	73.0523281	After 81
73.8457846	81.9030219	64.3551261	78.2690501	63	72.9273281	After 82
73.8457846	81.9030219	64.3551261	78.2690501	63	73.0523281	After 83
72.8457846	81.9030219	64.3551261	79.2690501	63	72.6773281	After 84
70.8457846	81.9030219	64.3551261	80.2690501	63	72.4273281	After 85
70.8457846	81.9030219	64.3551261	80.2690501	63	72.5523281	After 86
69.8457846	80.9030219	64.3551261	80.2690501	63	72.4273281	After 87
69.8457846	80.9030219	64.3551261	78.2690501	63	72.4273281	After 88
67.8457846	81.9030219	64.3551261	76.2690501	63	72.4273281	After 89
66.8457846	81.9030219	64.3551261	75.2690501	63	72.1773281	After 90
64.8457846	84.9030219	64.3551261	75.2690501	63	72.5523281	After 91
64.8457846	84.9030219	64.3551261	75.2690501	63	72.5523281	After 92
64.8457846	84.9030219	64.3551261	75.2690501	63	72.5523281	After 93
64.8457846	84.9030219	64.3551261	75.2690501	63	72.5523281	After 94
67.8457846	120.903022	63.3551261	75.2690501	63	76.6773281	After 95
69.8457846	121.903022	63.3551261	74.2690501	63	76.8023281	After 96
71.8457846	113.903022	63.3551261	74.2690501	63	75.9273281	After 97
73.8457846	85.9030219	63.3551261	73.2690501	63	72.3023281	After 98
73.8457846	77.9030219	63.3551261	72.2690501	63	71.1773281	After 99
73.8457846	72.9030219	63.3551261	72.2690501	63	70.5523281	After 100
72.8457846	69.9030219	63.3551261	73.2690501	63	70.3023281	After 101
72.8457846	68.9030219	63.3551261	73.2690501	63	70.3023281	After 102
73.8457846	67.9030219	63.3551261	72.2690501	63	70.5523281	After 103

75.8457846	67.9030219	63.3551261	72.2690501	63	70.8023281	After 104
75.8457846	67.9030219	63.3551261	72.2690501	63	71.3023281	After 105
78.8457846	68.9030219	65.3551261	71.2690501	63	72.1773281	After 106
79.8457846	68.9030219	65.3551261	71.2690501	63	72.3023281	After 107
79.8457846	68.9030219	68.3551261	67.2690501	63	72.4273281	After 108
80.8457846	68.9030219	68.3551261	67.2690501	63	72.9273281	After 109
81.8457846	68.9030219	69.3551261	67.2690501	63	73.3023281	After 110
82.8457846	68.9030219	69.3551261	67.2690501	63	73.6773281	After 111
82.8457846	68.9030219	69.3551261	67.2690501	63	74.3023281	After 112
82.8457846	68.9030219	69.3551261	67.2690501	63	74.4273281	After 113
82.8457846	68.9030219	69.3551261	66.2690501	63	74.6773281	After 114
82.8457846	68.9030219	69.3551261	66.2690501	63	74.8023281	After 115
82.8457846	68.9030219	68.3551261	67.2690501	63	75.0523281	After 116
82.8457846	68.9030219	68.3551261	67.2690501	63	75.4273281	After 117
82.8457846	68.9030219	68.3551261	67.2690501	63	74.8023281	After 118
82.8457846	68.9030219	69.3551261	68.2690501	63	75.0523281	After 119
78.8457846	69.9030219	69.3551261	67.2690501	63	73.8023281	After 120
77.8457846	70.9030219	69.3551261	68.2690501	63	73.6773281	After 121
75.8457846	73.9030219	70.3551261	68.2690501	63	73.6773281	After 122
75.8457846	75.9030219	71.3551261	68.2690501	63	73.9273281	After 123
75.8457846	75.9030219	71.3551261	69.2690501	63	73.6773281	After 124
75.8457846	76.9030219	72.3551261	69.2690501	63	73.6773281	After 125
74.8457846	78.9030219	72.3551261	69.2690501	63	73.8023281	After 126
72.8457846	78.9030219	72.3551261	69.2690501	63	73.3023281	After 127
70.8457846	79.9030219	72.3551261	69.2690501	63	73.3023281	After 128
70.8457846	80.9030219	72.3551261	69.2690501	63	73.3023281	After 129
70.8457846	80.9030219	72.3551261	69.2690501	63	73.3023281	After 130
69.8457846	80.9030219	72.3551261	69.2690501	63	73.1773281	After 131
66.8457846	75.9030219	72.3551261	74.2690501	63	72.8023281	After 132
65.8457846	75.9030219	72.3551261	74.2690501	63	72.6773281	After 133
64.8457846	72.9030219	72.3551261	73.2690501	63	71.8023281	After 134
64.8457846	71.9030219	72.3551261	73.2690501	63	71.5523281	After 135
65.8457846	71.9030219	73.3551261	73.2690501	63	71.6773281	After 136
65.8457846	69.9030219	73.3551261	73.2690501	63	71.4273281	After 137
66.8457846	69.9030219	72.3551261	73.2690501	63	71.5523281	After 138
69.8457846	69.9030219	72.3551261	72.2690501	63	71.8023281	After 139
69.8457846	69.9030219	72.3551261	72.2690501	63	71.6773281	After 140
70.8457846	69.9030219	71.3551261	73.2690501	63	71.5523281	After 141
70.8457846	70.9030219	71.3551261	74.2690501	63	71.6773281	After 142
70.8457846	67.9030219	71.3551261	75.2690501	63	71.0523281	After 143
70.8457846	67.9030219	71.3551261	76.2690501	63	71.1773281	After 144
70.8457846	65.9030219	71.3551261	76.2690501	63	70.9273281	After 145
70.8457846	64.9030219	71.3551261	77.2690501	63	71.1773281	After 146
70.8457846	64.9030219	69.3551261	77.2690501	63	70.9273281	After 147
70.8457846	64.9030219	69.3551261	77.2690501	63	70.9273281	After 148

72.8457846	64.9030219	68.3551261	77.2690501	63	71.1773281	After 149
72.8457846	64.9030219	68.3551261	77.2690501	63	71.1773281	After 150
72.8457846	64.9030219	66.3551261	77.2690501	63	70.9273281	After 151
72.8457846	66.9030219	68.3551261	77.2690501	63	71.4273281	After 152
72.8457846	66.9030219	70.3551261	77.2690501	63	71.9273281	After 153
72.8457846	66.9030219	72.3551261	77.2690501	63	72.4273281	After 154
72.8457846	66.9030219	73.3551261	76.2690501	63	72.4273281	After 155
71.8457846	66.9030219	73.3551261	76.2690501	63	72.5523281	After 156
71.8457846	66.9030219	71.3551261	76.2690501	63	72.5523281	After 157
66.8457846	69.9030219	70.3551261	76.2690501	63	72.3023281	After 158
63.8457846	72.9030219	70.3551261	76.2690501	63	72.3023281	After 159
60.8457846	74.9030219	70.3551261	76.2690501	63	72.1773281	After 160
59.8457846	74.9030219	71.3551261	76.2690501	63	71.9273281	After 161
58.8457846	80.9030219	72.3551261	76.2690501	63	72.3023281	After 162
58.8457846	86.9030219	73.3551261	76.2690501	63	73.1773281	After 163
58.8457846	91.9030219	75.3551261	75.2690501	63	74.0523281	After 164
58.8457846	91.9030219	77.3551261	75.2690501	63	74.1773281	After 165
57.8457846	85.9030219	78.3551261	74.2690501	63	73.4273281	After 166
56.8457846	80.9030219	78.3551261	73.2690501	63	72.6773281	After 167
56.8457846	74.9030219	78.3551261	72.2690501	63	71.9273281	After 168
69.8457846	72.9030219	77.3551261	73.2690501	63	73.3023281	After 169
72.8457846	71.9030219	77.3551261	73.2690501	63	73.9273281	After 170
73.8457846	71.9030219	75.3551261	73.2690501	63	73.9273281	After 171
73.8457846	70.9030219	74.3551261	74.2690501	63	73.9273281	After 172
73.8457846	70.9030219	73.3551261	74.2690501	63	74.1773281	After 173
76.8457846	70.9030219	73.3551261	74.2690501	63	74.5523281	After 174
75.8457846	70.9030219	73.3551261	74.2690501	63	74.1773281	After 175
74.8457846	70.9030219	73.3551261	74.2690501	63	73.6773281	After 176
73.8457846	69.9030219	73.3551261	75.2690501	63	73.4273281	After 177
73.8457846	69.9030219	73.3551261	76.2690501	63	73.3023281	After 178
73.8457846	68.9030219	73.3551261	76.2690501	63	73.4273281	After 179
72.8457846	68.9030219	73.3551261	76.2690501	63	73.3023281	After 180
72.8457846	66.9030219	73.3551261	77.2690501	63	73.1773281	After 181
72.8457846	67.9030219	73.3551261	77.2690501	63	73.4273281	After 182
72.8457846	67.9030219	74.3551261	77.2690501	63	73.3023281	After 183
72.8457846	67.9030219	74.3551261	77.2690501	63	72.6773281	After 184
72.8457846	67.9030219	75.3551261	78.2690501	63	72.6773281	After 185
73.8457846	67.9030219	75.3551261	78.2690501	63	72.9273281	After 186
73.8457846	65.9030219	77.3551261	78.2690501	63	73.3023281	After 187
74.8457846	65.9030219	78.3551261	75.2690501	63	73.4273281	After 188
74.8457846	65.9030219	79.3551261	75.2690501	63	73.8023281	After 189
76.8457846	66.9030219	79.3551261	75.2690501	63	74.3023281	After 190
76.8457846	66.9030219	81.3551261	73.2690501	63	74.4273281	After 191
77.8457846	69.9030219	82.3551261	73.2690501	63	75.0523281	After 192
77.8457846	70.9030219	83.3551261	73.2690501	63	74.8023281	After 193

77.8457846	70.9030219	83.3551261	71.2690501	63	74.3023281	After 194
77.8457846	73.9030219	83.3551261	71.2690501	63	74.5523281	After 195
77.8457846	73.9030219	83.3551261	70.2690501	63	74.1773281	After 196
77.8457846	73.9030219	83.3551261	71.2690501	63	74.1773281	After 197
77.8457846	73.9030219	84.3551261	71.2690501	63	74.5523281	After 198
77.8457846	73.9030219	84.3551261	71.2690501	63	74.6773281	After 199
77.8457846	73.9030219	86.3551261	70.2690501	63	74.9273281	After 200
77.8457846	76.9030219	86.3551261	70.2690501	63	75.3023281	After 201
77.8457846	76.9030219	86.3551261	70.2690501	63	75.3023281	After 202
77.8457846	78.9030219	86.3551261	69.2690501	63	75.5523281	After 203
77.8457846	86.9030219	86.3551261	68.2690501	63	76.4273281	After 204
77.8457846	86.9030219	85.3551261	73.2690501	63	77.0523281	After 205
77.8457846	88.9030219	85.3551261	74.2690501	63	77.8023281	After 206
78.8457846	91.9030219	72.3551261	74.2690501	63	76.8023281	After 207
78.8457846	92.9030219	70.3551261	74.2690501	63	76.9273281	After 208
78.8457846	93.9030219	70.3551261	74.2690501	63	77.0523281	After 209
78.8457846	94.9030219	70.3551261	74.2690501	63	77.1773281	After 210
78.8457846	94.9030219	70.3551261	74.2690501	63	77.1773281	After 211
78.8457846	98.9030219	69.3551261	74.2690501	63	77.5523281	After 212
79.8457846	99.9030219	69.3551261	74.2690501	63	77.8023281	After 213
79.8457846	99.9030219	69.3551261	75.2690501	63	77.9273281	After 214
80.8457846	99.9030219	69.3551261	75.2690501	63	78.0523281	After 215
80.8457846	101.903022	70.3551261	74.2690501	63	79.0523281	After 216
80.8457846	103.903022	70.3551261	75.2690501	63	79.5523281	After 217
81.8457846	102.903022	70.3551261	74.2690501	63	79.6773281	After 218
82.8457846	97.9030219	71.3551261	74.2690501	63	79.4273281	After 219
82.8457846	97.9030219	72.3551261	74.2690501	63	80.0523281	After 220
82.8457846	97.9030219	73.3551261	74.2690501	63	80.3023281	After 221
82.8457846	96.9030219	76.3551261	74.2690501	63	80.4273281	After 222
82.8457846	97.9030219	71.3551261	74.2690501	63	79.9273281	After 223
84.8457846	97.9030219	69.3551261	75.2690501	63	79.9273281	After 224
84.8457846	100.903022	69.3551261	75.2690501	63	80.3023281	After 225
84.8457846	102.903022	68.3551261	75.2690501	63	80.3023281	After 226
84.8457846	104.903022	68.3551261	75.2690501	63	80.9273281	After 227
84.8457846	104.903022	68.3551261	75.2690501	63	80.9273281	After 228
84.8457846	103.903022	68.3551261	75.2690501	63	80.6773281	After 229
84.8457846	102.903022	68.3551261	73.2690501	63	80.1773281	After 230
85.8457846	101.903022	68.3551261	73.2690501	63	80.3023281	After 231
82.8457846	101.903022	68.3551261	73.2690501	63	79.6773281	After 232
82.8457846	100.903022	68.3551261	73.2690501	63	79.5523281	After 233
82.8457846	99.9030219	65.3551261	73.2690501	63	78.1773281	After 234
83.8457846	99.9030219	63.3551261	73.2690501	63	77.8023281	After 235
84.8457846	99.9030219	63.3551261	73.2690501	63	77.8023281	After 236
85.8457846	99.9030219	63.3551261	73.2690501	63	78.0523281	After 237
86.8457846	99.9030219	63.3551261	73.2690501	63	78.0523281	After 238

87.8457846	100.903022	64.3551261	73.2690501	63	78.5523281	After 239
88.8457846	100.903022	65.3551261	73.2690501	63	78.6773281	After 240
88.8457846	82.9030219	66.3551261	72.2690501	63	76.3023281	
88.8457846	73.9030219	66.3551261	72.2690501	63	75.4273281	
88.8457846	67.9030219	66.3551261	72.2690501	63	74.8023281	
89.8457846	64.9030219	66.3551261	75.2690501	63	74.6773281	
90.8457846	63.9030219	66.3551261	75.2690501	63	74.6773281	
89.8457846	63.9030219	66.3551261	75.2690501	63	74.9273281	
89.8457846	63.9030219	66.3551261	75.2690501	63	75.0523281	
89.8457846	63.9030219	66.3551261	75.2690501	63	75.1773281	
89.8457846	63.9030219	66.3551261	72.2690501	63	74.1773281	
89.8457846	61.9030219	66.3551261	69.2690501	63	73.4273281	
89.8457846	61.9030219	66.3551261	68.2690501	63	72.6773281	
89.8457846	61.9030219	67.3551261	68.2690501	63	72.8023281	
89.8457846	61.9030219	69.3551261	68.2690501	63	73.6773281	
89.8457846	62.9030219	69.3551261	68.2690501	63	74.0523281	
89.8457846	62.9030219	69.3551261	68.2690501	63	74.1773281	
89.8457846	62.9030219	69.3551261	68.2690501	63	74.1773281	
92.8457846	62.9030219	69.3551261	68.2690501	63	74.4273281	
92.8457846	63.9030219	69.3551261	68.2690501	63	74.9273281	
92.8457846	63.9030219	69.3551261	67.2690501	63	75.3023281	
92.8457846	62.9030219	69.3551261	67.2690501	63	75.3023281	
88.8457846	63.9030219	69.3551261	68.2690501	63	74.8023281	
84.8457846	66.9030219	69.3551261	69.2690501	63	74.3023281	
81.8457846	67.9030219	70.3551261	70.2690501	63	74.0523281	
80.8457846	65.9030219	70.3551261	70.2690501	63	73.8023281	
79.8457846	64.9030219	72.3551261	70.2690501	63	73.5523281	
79.8457846	62.9030219	73.3551261	69.2690501	63	73.4273281	
78.8457846	61.9030219	73.3551261	69.2690501	63	72.9273281	
77.8457846	60.9030219	73.3551261	69.2690501	63	71.8023281	
77.8457846	60.9030219	73.3551261	69.2690501	63	71.9273281	
77.8457846	60.9030219	73.3551261	69.2690501	63	71.8023281	
77.8457846	61.9030219	73.3551261	69.2690501	63	71.8023281	
77.8457846	62.9030219	73.3551261	69.2690501	63	72.4273281	
77.8457846	64.9030219	73.3551261	70.2690501	63	72.8023281	
78.8457846	64.9030219	72.3551261	69.2690501	63	72.8023281	
78.8457846	64.9030219	71.3551261	69.2690501	63	72.8023281	
78.8457846	64.9030219	73.3551261	69.2690501	63	73.3023281	
78.8457846	64.9030219	80.3551261	69.2690501	63	74.3023281	
76.8457846	64.9030219	80.3551261	69.2690501	63	73.8023281	
76.8457846	64.9030219	82.3551261	71.2690501	63	73.8023281	
75.8457846	65.9030219	82.3551261	71.2690501	63	73.6773281	
74.8457846	66.9030219	82.3551261	71.2690501	63	73.8023281	
74.8457846	66.9030219	82.3551261	73.2690501	63	73.9273281	
73.8457846	66.9030219	82.3551261	74.2690501	63	73.9273281	

73.8457846	66.9030219	82.3551261	76.2690501	63	74.1773281	
72.8457846	66.9030219	82.3551261	76.2690501	63	74.0523281	
71.8457846	66.9030219	82.3551261	75.2690501	63	73.8023281	
70.8457846	66.9030219	82.3551261	74.2690501	63	73.9273281	
70.8457846	66.9030219	83.3551261	74.2690501	63	74.5523281	
70.8457846	66.9030219	83.3551261	74.2690501	63	74.5523281	
69.8457846	69.9030219	83.3551261	74.2690501	63	75.5523281	
69.8457846	71.9030219	83.3551261	74.2690501	63	75.9273281	
70.8457846	73.9030219	83.3551261	74.2690501	63	76.3023281	
71.8457846	73.9030219	79.3551261	74.2690501	63	75.9273281	
72.8457846	73.9030219	75.3551261	74.2690501	63	75.4273281	
72.8457846	73.9030219	75.3551261	74.2690501	63	75.4273281	
75.8457846	76.9030219	73.3551261	74.2690501	63	75.4273281	
75.8457846	76.9030219	71.3551261	74.2690501	63	75.5523281	
75.8457846	74.9030219	71.3551261	73.2690501	63	75.3023281	
77.8457846	74.9030219	71.3551261	73.2690501	63	75.8023281	
77.8457846	73.9030219	71.3551261	73.2690501	63	75.6773281	
77.8457846	73.9030219	71.3551261	74.2690501	63	75.8023281	
77.8457846	73.9030219	71.3551261	74.2690501	63	75.5523281	
77.8457846	73.9030219	71.3551261	74.2690501	63	75.3023281	
77.8457846	73.9030219	71.3551261	73.2690501	63	75.1773281	
76.8457846	73.9030219	71.3551261	73.2690501	63	74.5523281	
76.8457846	74.9030219	70.3551261	73.2690501	63	73.3023281	
75.8457846	74.9030219	70.3551261	73.2690501	63	72.8023281	
75.8457846	74.9030219	70.3551261	73.2690501	63	73.1773281	
75.8457846	77.9030219	70.3551261	72.2690501	63	73.9273281	
75.8457846	77.9030219	70.3551261	72.2690501	63	73.9273281	
76.8457846	79.9030219	70.3551261	71.2690501	63	74.6773281	
76.8457846	80.9030219	70.3551261	70.2690501	63	75.3023281	
77.8457846	82.9030219	70.3551261	70.2690501	63	75.6773281	
77.8457846	84.9030219	72.3551261	70.2690501	63	76.0523281	
77.8457846	85.9030219	71.3551261	70.2690501	63	75.9273281	
77.8457846	85.9030219	71.3551261	69.2690501	69	75.6773281	
77.8457846	85.9030219	71.3551261	69.2690501	69	75.5523281	
77.8457846	85.9030219	72.3551261	67.2690501	69	75.8023281	
77.8457846	86.9030219	72.3551261	68.2690501	69	76.1773281	
76.8457846	87.9030219	75.3551261	68.2690501	69	76.9273281	
76.8457846	87.9030219	76.3551261	68.2690501	69	77.5523281	
76.8457846	87.9030219	79.3551261	68.2690501	69	78.4273281	
75.8457846	87.9030219	77.3551261	68.2690501	69	78.0523281	
74.8457846	87.9030219	76.3551261	68.2690501	69	77.6773281	
75.8457846	87.9030219	76.3551261	68.2690501	69	77.6773281	
78.8457846	89.9030219	75.3551261	68.2690501	69	78.4273281	
79.8457846	91.9030219	75.3551261	68.2690501	69	78.4273281	
80.8457846	92.9030219	74.3551261	69.2690501	69	78.3023281	

79.8457846	93.9030219	74.3551261	69.2690501	69	78.0523281	
77.8457846	95.9030219	73.3551261	71.2690501	69	77.9273281	
77.8457846	95.9030219	73.3551261	71.2690501	69	77.9273281	
79.8457846	95.9030219	73.3551261	71.2690501	69	77.9273281	
82.8457846	96.9030219	79.3551261	72.2690501	69	79.4273281	
86.8457846	96.9030219	81.3551261	72.2690501	69	80.1773281	
86.8457846	96.9030219	81.3551261	72.2690501	69	80.3023281	
88.8457846	96.9030219	80.3551261	72.2690501	69	80.1773281	
89.8457846	96.9030219	80.3551261	73.2690501	69	80.4273281	
89.8457846	97.9030219	80.3551261	73.2690501	69	81.5523281	
89.8457846	97.9030219	81.3551261	74.2690501	69	82.5523281	
89.8457846	98.9030219	81.3551261	77.2690501	69	83.0523281	
89.8457846	98.9030219	81.3551261	77.2690501	69	83.3023281	
89.8457846	99.9030219	85.3551261	77.2690501	69	83.8023281	
89.8457846	98.9030219	85.3551261	80.2690501	69	83.9273281	
87.8457846	98.9030219	86.3551261	78.2690501	69	83.3023281	
85.8457846	98.9030219	86.3551261	77.2690501	69	82.5523281	
85.8457846	98.9030219	86.3551261	77.2690501	69	82.4273281	
85.8457846	98.9030219	86.3551261	77.2690501	69	81.9273281	
83.8457846	99.9030219	87.3551261	77.2690501	69	82.1773281	
82.8457846	99.9030219	87.3551261	77.2690501	69	82.0523281	
82.8457846	100.903022	88.3551261	75.2690501	69	81.4273281	
81.8457846	100.903022	88.3551261	75.2690501	69	80.5523281	
80.8457846	101.903022	88.3551261	72.2690501	69	80.3023281	
80.8457846	102.903022	88.3551261	72.2690501	69	80.0523281	
80.8457846	103.903022	87.3551261	72.2690501	69	79.9273281	
79.8457846	104.903022	85.3551261	72.2690501	69	79.3023281	
80.8457846	104.903022	84.3551261	70.2690501	63	78.9273281	
80.8457846	104.903022	84.3551261	70.2690501	63	78.5523281	
82.8457846	104.903022	83.3551261	70.2690501	63	78.4273281	
82.8457846	105.903022	83.3551261	72.2690501	63	78.6773281	
84.8457846	105.903022	83.3551261	70.2690501	63	78.3023281	
84.8457846	104.903022	83.3551261	69.2690501	63	77.9273281	
84.8457846	103.903022	83.3551261	69.2690501	63	77.5523281	
84.8457846	103.903022	81.3551261	68.2690501	63	77.3023281	
84.8457846	103.903022	81.3551261	68.2690501	63	77.3023281	
81.8457846	104.903022	73.3551261	68.2690501	63	76.3023281	
81.8457846	104.903022	71.3551261	65.2690501	63	75.6773281	
80.8457846	104.903022	70.3551261	65.2690501	63	75.4273281	
80.8457846	104.903022	70.3551261	66.2690501	63	75.6773281	
80.8457846	104.903022	70.3551261	66.2690501	63	75.8023281	
75.8457846	104.903022	70.3551261	69.2690501	63	75.9273281	
72.8457846	104.903022	69.3551261	70.2690501	63	75.6773281	
72.8457846	104.903022	69.3551261	70.2690501	63	75.6773281	
71.8457846	104.903022	69.3551261	69.2690501	63	75.4273281	

71.8457846	104.903022	70.3551261	69.2690501	63	75.5523281	
71.8457846	103.903022	72.3551261	68.2690501	63	75.6773281	
71.8457846	102.903022	71.3551261	68.2690501	63	75.4273281	
70.8457846	101.903022	71.3551261	68.2690501	63	74.8023281	
70.8457846	98.9030219	71.3551261	69.2690501	63	74.4273281	
70.8457846	95.9030219	72.3551261	70.2690501	63	74.4273281	
70.8457846	95.9030219	73.3551261	70.2690501	63	75.0523281	
69.8457846	95.9030219	75.3551261	71.2690501	63	75.6773281	
68.8457846	95.9030219	78.3551261	71.2690501	63	76.1773281	
68.8457846	95.9030219	78.3551261	71.2690501	63	76.3023281	
68.8457846	96.9030219	78.3551261	71.2690501	63	76.1773281	
69.8457846	99.9030219	78.3551261	71.2690501	63	76.8023281	
69.8457846	107.903022	76.3551261	72.2690501	63	77.8023281	
69.8457846	106.903022	73.3551261	73.2690501	63	77.3023281	
69.8457846	106.903022	72.3551261	76.2690501	63	77.4273281	
70.8457846	106.903022	72.3551261	77.2690501	63	77.6773281	
71.8457846	108.903022	71.3551261	76.2690501	63	77.8023281	
78.8457846	108.903022	71.3551261	76.2690501	63	78.4273281	
79.8457846	111.903022	70.3551261	78.2690501	63	79.4273281	
79.8457846	111.903022	70.3551261	78.2690501	63	79.5523281	
81.8457846	111.903022	70.3551261	78.2690501	63	80.0523281	
83.8457846	112.903022	70.3551261	77.2690501	63	80.4273281	
83.8457846	112.903022	70.3551261	77.2690501	63	80.4273281	
82.8457846	113.903022	70.3551261	77.2690501	63	80.4273281	
80.8457846	113.903022	70.3551261	76.2690501	63	80.1773281	
80.8457846	113.903022	70.3551261	75.2690501	63	80.0523281	
79.8457846	113.903022	70.3551261	75.2690501	63	79.9273281	
78.8457846	113.903022	70.3551261	77.2690501	63	80.0523281	
78.8457846	108.903022	70.3551261	77.2690501	63	80.9273281	
73.8457846	89.9030219	70.3551261	81.2690501	63	79.3023281	
72.8457846	89.9030219	70.3551261	82.2690501	63	80.5523281	
72.8457846	74.9030219	70.3551261	83.2690501	63	79.1773281	
72.8457846	70.9030219	70.3551261	83.2690501	63	78.9273281	
73.8457846	68.9030219	70.3551261	83.2690501	63	78.6773281	
73.8457846	66.9030219	70.3551261	83.2690501	63	77.6773281	
73.8457846	66.9030219	70.3551261	83.2690501	63	76.5523281	
73.8457846	65.9030219	70.3551261	82.2690501	63	76.3023281	
73.8457846	64.9030219	70.3551261	79.2690501	63	75.6773281	
73.8457846	64.9030219	72.3551261	79.2690501	63	76.1773281	
72.8457846	63.9030219	74.3551261	79.2690501	63	76.1773281	
72.8457846	62.9030219	73.3551261	76.2690501	63	75.1773281	
73.8457846	62.9030219	73.3551261	75.2690501	63	75.0523281	
74.8457846	62.9030219	73.3551261	75.2690501	63	75.0523281	
74.8457846	62.9030219	73.3551261	75.2690501	63	74.6773281	
74.8457846	62.9030219	73.3551261	75.2690501	63	74.6773281	

76.8457846	62.9030219	74.3551261	74.2690501	63	74.4273281	
77.8457846	62.9030219	75.3551261	74.2690501	63	74.5523281	
78.8457846	62.9030219	76.3551261	75.2690501	63	74.8023281	
78.8457846	62.9030219	77.3551261	77.2690501	63	75.1773281	
78.8457846	62.9030219	77.3551261	76.2690501	63	74.9273281	
78.8457846	61.9030219	77.3551261	76.2690501	63	74.6773281	
78.8457846	61.9030219	77.3551261	76.2690501	63	74.5523281	
78.8457846	61.9030219	77.3551261	76.2690501	63	73.9273281	
79.8457846	61.9030219	77.3551261	76.2690501	63	73.8023281	
80.8457846	61.9030219	76.3551261	72.2690501	63	73.3023281	
82.8457846	61.9030219	74.3551261	70.2690501	63	72.6773281	
84.8457846	60.9030219	71.3551261	69.2690501	63	72.4273281	
86.8457846	60.9030219	71.3551261	69.2690501	63	72.5523281	
86.8457846	62.9030219	71.3551261	68.2690501	63	72.4273281	
86.8457846	62.9030219	71.3551261	68.2690501	63	72.3023281	
86.8457846	62.9030219	71.3551261	68.2690501	63	72.5523281	
86.8457846	62.9030219	72.3551261	68.2690501	63	72.9273281	
85.8457846	61.9030219	72.3551261	68.2690501	63	72.5523281	
85.8457846	61.9030219	72.3551261	68.2690501	63	72.4273281	
85.8457846	63.9030219	72.3551261	68.2690501	63	72.6773281	
83.8457846	65.9030219	72.3551261	68.2690501	63	72.6773281	
82.8457846	66.9030219	71.3551261	68.2690501	63	72.8023281	
82.8457846	68.9030219	71.3551261	69.2690501	63	73.4273281	
82.8457846	69.9030219	71.3551261	69.2690501	63	73.3023281	
79.8457846	69.9030219	71.3551261	69.2690501	63	72.9273281	
78.8457846	69.9030219	71.3551261	69.2690501	63	72.8023281	
78.8457846	68.9030219	76.3551261	69.2690501	63	73.4273281	
78.8457846	67.9030219	76.3551261	72.2690501	63	73.9273281	
77.8457846	67.9030219	76.3551261	73.2690501	63	73.8023281	
77.8457846	67.9030219	76.3551261	73.2690501	63	73.4273281	
78.8457846	67.9030219	76.3551261	73.2690501	63	73.9273281	
78.8457846	67.9030219	75.3551261	73.2690501	63	73.9273281	
76.8457846	67.9030219	75.3551261	74.2690501	63	73.8023281	
75.8457846	67.9030219	75.3551261	74.2690501	63	73.8023281	
74.8457846	67.9030219	75.3551261	74.2690501	63	73.8023281	
73.8457846	67.9030219	74.3551261	74.2690501	63	73.6773281	
73.8457846	67.9030219	74.3551261	74.2690501	63	73.9273281	
73.8457846	67.9030219	73.3551261	74.2690501	63	73.8023281	
74.8457846	66.9030219	73.3551261	75.2690501	63	73.8023281	
75.8457846	64.9030219	74.3551261	76.2690501	63	74.3023281	
77.8457846	64.9030219	74.3551261	76.2690501	63	74.8023281	
77.8457846	63.9030219	75.3551261	77.2690501	63	75.1773281	
76.8457846	63.9030219	75.3551261	77.2690501	63	75.3023281	
76.8457846	63.9030219	75.3551261	75.2690501	63	75.0523281	
76.8457846	63.9030219	75.3551261	70.2690501	63	74.4273281	

76.8457846	63.9030219	75.3551261	69.2690501	63	74.1773281	
76.8457846	64.9030219	74.3551261	68.2690501	63	73.9273281	
76.8457846	65.9030219	74.3551261	68.2690501	63	74.0523281	
76.8457846	65.9030219	74.3551261	68.2690501	63	74.0523281	
76.8457846	64.9030219	74.3551261	69.2690501	63	73.9273281	
76.8457846	64.9030219	74.3551261	71.2690501	63	74.3023281	
76.8457846	64.9030219	74.3551261	71.2690501	63	73.3023281	
76.8457846	64.9030219	74.3551261	71.2690501	63	73.3023281	
76.8457846	64.9030219	74.3551261	71.2690501	63	73.3023281	
75.8457846	64.9030219	74.3551261	71.2690501	63	72.9273281	
75.8457846	64.9030219	74.3551261	73.2690501	63	73.0523281	
74.8457846	64.9030219	74.3551261	71.2690501	63	72.6773281	
73.8457846	64.9030219	75.3551261	70.2690501	63	72.8023281	
73.8457846	64.9030219	76.3551261	70.2690501	63	72.9273281	
73.8457846	64.9030219	76.3551261	70.2690501	63	73.0523281	
73.8457846	64.9030219	74.3551261	70.2690501	63	72.6773281	
73.8457846	64.9030219	72.3551261	70.2690501	63	72.1773281	
73.8457846	64.9030219	70.3551261	70.2690501	63	71.6773281	
73.8457846	64.9030219	70.3551261	70.2690501	63	72.3023281	
74.8457846	64.9030219	70.3551261	69.2690501	63	71.8023281	
76.8457846	64.9030219	69.3551261	68.2690501	63	71.4273281	
76.8457846	63.9030219	69.3551261	68.2690501	63	71.1773281	
76.8457846	64.9030219	69.3551261	68.2690501	63	71.3023281	
76.8457846	64.9030219	69.3551261	68.2690501	63	71.1773281	
76.8457846	64.9030219	69.3551261	68.2690501	63	71.1773281	
76.8457846	64.9030219	69.3551261	68.2690501	63	71.1773281	
76.8457846	64.9030219	68.3551261	68.2690501	63	71.3023281	
76.8457846	64.9030219	67.3551261	69.2690501	63	71.8023281	
77.8457846	64.9030219	67.3551261	69.2690501	63	72.1773281	
78.8457846	64.9030219	67.3551261	69.2690501	63	72.1773281	
79.8457846	64.9030219	67.3551261	69.2690501	63	72.3023281	
80.8457846	65.9030219	67.3551261	69.2690501	63	72.1773281	
80.8457846	65.9030219	67.3551261	69.2690501	63	72.3023281	
80.8457846	66.9030219	67.3551261	69.2690501	63	72.9273281	
80.8457846	66.9030219	69.3551261	69.2690501	63	73.4273281	
81.8457846	66.9030219	69.3551261	70.2690501	63	73.8023281	
81.8457846	66.9030219	71.3551261	70.2690501	63	74.1773281	
81.8457846	66.9030219	71.3551261	70.2690501	63	74.3023281	
81.8457846	65.9030219	71.3551261	70.2690501	63	74.0523281	
81.8457846	65.9030219	71.3551261	72.2690501	63	74.1773281	
81.8457846	65.9030219	71.3551261	69.2690501	63	73.5523281	
81.8457846	65.9030219	77.3551261	69.2690501	63	74.1773281	
82.8457846	65.9030219	78.3551261	69.2690501	63	74.3023281	
87.8457846	65.9030219	78.3551261	70.2690501	63	75.0523281	
88.8457846	68.9030219	78.3551261	71.2690501	63	75.4273281	

90.8457846	69.9030219	78.3551261	72.2690501	63	76.1773281	
91.8457846	69.9030219	78.3551261	74.2690501	63	76.6773281	
90.8457846	71.9030219	79.3551261	74.2690501	63	76.9273281	
90.8457846	71.9030219	79.3551261	74.2690501	63	76.6773281	
89.8457846	71.9030219	79.3551261	74.2690501	63	76.5523281	
87.8457846	68.9030219	80.3551261	75.2690501	63	76.5523281	
86.8457846	66.9030219	81.3551261	76.2690501	63	76.5523281	
86.8457846	65.9030219	82.3551261	77.2690501	63	76.6773281	
86.8457846	64.9030219	82.3551261	78.2690501	63	76.4273281	
86.8457846	64.9030219	83.3551261	77.2690501	63	76.4273281	
86.8457846	64.9030219	83.3551261	76.2690501	63	76.1773281	
86.8457846	65.9030219	82.3551261	74.2690501	63	76.0523281	
86.8457846	65.9030219	81.3551261	74.2690501	63	75.6773281	
86.8457846	65.9030219	79.3551261	74.2690501	63	75.3023281	
87.8457846	66.9030219	78.3551261	73.2690501	63	75.1773281	
85.8457846	66.9030219	75.3551261	74.2690501	63	74.5523281	
83.8457846	65.9030219	74.3551261	74.2690501	63	74.0523281	
83.8457846	65.9030219	74.3551261	74.2690501	63	74.0523281	
81.8457846	65.9030219	74.3551261	74.2690501	63	73.4273281	
81.8457846	65.9030219	74.3551261	74.2690501	63	73.4273281	
81.8457846	64.9030219	73.3551261	74.2690501	63	73.3023281	
81.8457846	64.9030219	73.3551261	75.2690501	63	73.5523281	
81.8457846	64.9030219	72.3551261	75.2690501	63	73.4273281	
81.8457846	64.9030219	72.3551261	75.2690501	63	73.4273281	
81.8457846	64.9030219	72.3551261	75.2690501	63	73.6773281	
81.8457846	64.9030219	73.3551261	75.2690501	63	74.1773281	
81.8457846	64.9030219	73.3551261	77.2690501	63	74.5523281	
79.8457846	64.9030219	72.3551261	79.2690501	63	74.6773281	
77.8457846	65.9030219	71.3551261	81.2690501	63	75.3023281	
77.8457846	65.9030219	71.3551261	81.2690501	63	76.1773281	
77.8457846	65.9030219	71.3551261	81.2690501	63	76.5523281	
77.8457846	66.9030219	71.3551261	81.2690501	63	76.5523281	
77.8457846	72.9030219	71.3551261	79.2690501	63	77.0523281	
76.8457846	72.9030219	71.3551261	78.2690501	63	76.6773281	
76.8457846	89.9030219	71.3551261	77.2690501	63	78.6773281	
77.8457846	89.9030219	71.3551261	77.2690501	63	77.9273281	
78.8457846	109.903022	72.3551261	77.2690501	63	80.8023281	
79.8457846	95.9030219	72.3551261	76.2690501	63	79.0523281	
80.8457846	92.9030219	72.3551261	76.2690501	63	78.8023281	
80.8457846	92.9030219	72.3551261	75.2690501	63	78.8023281	
80.8457846	92.9030219	72.3551261	75.2690501	63	78.8023281	
80.8457846	76.9030219	73.3551261	75.2690501	63	76.9273281	
80.8457846	76.9030219	72.3551261	75.2690501	63	76.5523281	
80.8457846	74.9030219	71.3551261	75.2690501	63	76.1773281	
79.8457846	74.9030219	71.3551261	72.2690501	63	75.6773281	

78.8457846	74.9030219	71.3551261	72.2690501	63	75.8023281	
78.8457846	72.9030219	70.3551261	73.2690501	63	75.6773281	
74.8457846	73.9030219	68.3551261	74.2690501	63	75.3023281	
74.8457846	73.9030219	68.3551261	74.2690501	63	75.3023281	
74.8457846	75.9030219	68.3551261	74.2690501	63	75.5523281	
74.8457846	75.9030219	68.3551261	74.2690501	63	75.6773281	
73.8457846	76.9030219	67.3551261	73.2690501	63	74.6773281	
73.8457846	78.9030219	67.3551261	74.2690501	63	74.5523281	
73.8457846	81.9030219	67.3551261	74.2690501	63	74.6773281	
73.8457846	82.9030219	64.3551261	73.2690501	63	74.1773281	
75.8457846	82.9030219	64.3551261	73.2690501	63	74.4273281	
77.8457846	83.9030219	66.3551261	72.2690501	63	75.0523281	
78.8457846	83.9030219	67.3551261	71.2690501	63	75.0523281	
78.8457846	86.9030219	67.3551261	71.2690501	63	75.4273281	
78.8457846	86.9030219	67.3551261	71.2690501	63	75.6773281	
77.8457846	86.9030219	71.3551261	71.2690501	63	76.5523281	
77.8457846	88.9030219	72.3551261	72.2690501	63	77.4273281	
77.8457846	90.9030219	73.3551261	72.2690501	63	77.5523281	
77.8457846	90.9030219	76.3551261	72.2690501	63	77.4273281	
77.8457846	88.9030219	77.3551261	73.2690501	63	77.1773281	
77.8457846	88.9030219	77.3551261	73.2690501	63	77.1773281	
77.8457846	88.9030219	77.3551261	72.2690501	63	76.6773281	
77.8457846	75.9030219	79.3551261	72.2690501	63	75.4273281	
77.8457846	75.9030219	80.3551261	71.2690501	63	75.3023281	
76.8457846	74.9030219	81.3551261	71.2690501	63	75.1773281	
76.8457846	74.9030219	81.3551261	71.2690501	63	75.1773281	
76.8457846	74.9030219	81.3551261	71.2690501	63	75.1773281	
76.8457846	74.9030219	81.3551261	72.2690501	63	76.5523281	
76.8457846	74.9030219	81.3551261	71.2690501	63	75.4273281	
78.8457846	70.9030219	81.3551261	70.2690501	63	75.4273281	
79.8457846	70.9030219	81.3551261	70.2690501	63	75.6773281	
81.8457846	70.9030219	81.3551261	69.2690501	63	75.6773281	
82.8457846	72.9030219	81.3551261	68.2690501	63	76.1773281	
83.8457846	74.9030219	81.3551261	68.2690501	63	76.6773281	
83.8457846	75.9030219	81.3551261	68.2690501	63	76.8023281	
85.8457846	75.9030219	82.3551261	68.2690501	63	77.1773281	
86.8457846	78.9030219	81.3551261	68.2690501	63	78.0523281	
87.8457846	78.9030219	81.3551261	68.2690501	63	78.0523281	
87.8457846	78.9030219	82.3551261	69.2690501	63	78.3023281	
87.8457846	78.9030219	82.3551261	68.2690501	63	78.3023281	
87.8457846	78.9030219	82.3551261	68.2690501	63	78.6773281	
87.8457846	79.9030219	81.3551261	68.2690501	63	78.8023281	
87.8457846	81.9030219	81.3551261	68.2690501	63	78.9273281	
87.8457846	82.9030219	81.3551261	68.2690501	63	79.1773281	
87.8457846	84.9030219	80.3551261	72.2690501	63	78.3023281	

86.8457846	84.9030219	80.3551261	73.2690501	63	78.3023281	
85.8457846	85.9030219	75.3551261	73.2690501	63	77.6773281	
83.8457846	85.9030219	74.3551261	73.2690501	63	77.4273281	
83.8457846	86.9030219	74.3551261	73.2690501	63	78.0523281	
83.8457846	85.9030219	74.3551261	73.2690501	63	78.1773281	
83.8457846	85.9030219	75.3551261	75.2690501	63	78.9273281	
83.8457846	84.9030219	76.3551261	75.2690501	63	79.0523281	
83.8457846	84.9030219	77.3551261	84.2690501	63	80.1773281	
83.8457846	84.9030219	77.3551261	84.2690501	63	80.1773281	
83.8457846	83.9030219	77.3551261	85.2690501	63	80.4273281	
85.8457846	83.9030219	78.3551261	85.2690501	63	80.8023281	
90.8457846	83.9030219	77.3551261	78.2690501	63	80.5523281	
90.8457846	83.9030219	77.3551261	74.2690501	63	79.9273281	
90.8457846	83.9030219	77.3551261	72.2690501	63	79.4273281	
91.8457846	81.9030219	73.3551261	70.2690501	63	78.4273281	
91.8457846	81.9030219	72.3551261	69.2690501	63	78.0523281	
91.8457846	81.9030219	66.3551261	68.2690501	63	77.1773281	
91.8457846	81.9030219	65.3551261	69.2690501	63	77.1773281	
90.8457846	83.9030219	65.3551261	69.2690501	63	77.3023281	
86.8457846	83.9030219	71.3551261	69.2690501	63	77.5523281	
85.8457846	83.9030219	80.3551261	69.2690501	63	78.4273281	
85.8457846	83.9030219	82.3551261	69.2690501	63	78.5523281	
83.8457846	83.9030219	82.3551261	69.2690501	69	78.3023281	
82.8457846	83.9030219	82.3551261	70.2690501	69	79.3023281	
81.8457846	84.9030219	82.3551261	71.2690501	69	79.8023281	
81.8457846	85.9030219	83.3551261	71.2690501	69	80.6773281	
80.8457846	85.9030219	83.3551261	71.2690501	69	80.5523281	
77.8457846	90.9030219	71.3551261	75.2690501	69	79.9273281	
75.8457846	90.9030219	69.3551261	76.2690501	69	79.9273281	
75.8457846	91.9030219	68.3551261	77.2690501	69	80.6773281	
74.8457846	92.9030219	68.3551261	76.2690501	69	81.0523281	
74.8457846	92.9030219	69.3551261	75.2690501	69	81.4273281	
74.8457846	93.9030219	69.3551261	75.2690501	69	81.5523281	
74.8457846	92.9030219	71.3551261	74.2690501	69	82.0523281	
74.8457846	92.9030219	73.3551261	74.2690501	69	82.1773281	
74.8457846	92.9030219	73.3551261	74.2690501	69	82.1773281	
73.8457846	87.9030219	73.3551261	73.2690501	69	81.1773281	
74.8457846	86.9030219	74.3551261	73.2690501	63	81.1773281	
74.8457846	86.9030219	74.3551261	73.2690501	63	81.3023281	
74.8457846	85.9030219	74.3551261	73.2690501	63	81.4273281	
74.8457846	84.9030219	74.3551261	73.2690501	63	81.5523281	
74.8457846	84.9030219	75.3551261	73.2690501	63	82.0523281	
74.8457846	84.9030219	75.3551261	72.2690501	63	82.0523281	
73.8457846	84.9030219	75.3551261	70.2690501	63	81.6773281	
73.8457846	82.9030219	76.3551261	69.2690501	63	81.6773281	

73.8457846	69.9030219	76.3551261	69.2690501	63	80.1773281	
73.8457846	69.9030219	77.3551261	68.2690501	63	80.4273281	
73.8457846	65.9030219	77.3551261	68.2690501	63	80.1773281	
74.8457846	64.9030219	77.3551261	68.2690501	63	80.3023281	
75.8457846	64.9030219	77.3551261	67.2690501	63	80.0523281	
75.8457846	64.9030219	78.3551261	66.2690501	63	80.0523281	
76.8457846	64.9030219	78.3551261	65.2690501	63	80.0523281	
77.8457846	64.9030219	78.3551261	65.2690501	63	80.1773281	
77.8457846	63.9030219	76.3551261	65.2690501	63	79.8023281	
79.8457846	63.9030219	76.3551261	66.2690501	63	79.9273281	
80.8457846	63.9030219	76.3551261	71.2690501	63	80.8023281	
80.8457846	63.9030219	76.3551261	78.2690501	63	81.9273281	
80.8457846	63.9030219	76.3551261	81.2690501	63	82.4273281	
80.8457846	63.9030219	76.3551261	82.2690501	63	82.5523281	
81.8457846	61.9030219	76.3551261	82.2690501	63	82.3023281	
81.8457846	61.9030219	77.3551261	82.2690501	63	82.1773281	
82.8457846	61.9030219	77.3551261	81.2690501	63	81.6773281	
83.8457846	60.9030219	77.3551261	81.2690501	63	81.1773281	
86.8457846	67.9030219	79.3551261	81.2690501	63	82.1773281	
88.8457846	72.9030219	79.3551261	81.2690501	63	82.8023281	
89.8457846	79.9030219	79.3551261	81.2690501	63	83.8023281	
90.8457846	79.9030219	81.3551261	81.2690501	63	83.9273281	
91.8457846	83.9030219	81.3551261	81.2690501	63	83.9273281	
91.8457846	90.9030219	77.3551261	82.2690501	63	84.1773281	
91.8457846	93.9030219	75.3551261	81.2690501	63	84.0523281	
91.8457846	95.9030219	76.3551261	81.2690501	63	84.4273281	
92.8457846	95.9030219	77.3551261	80.2690501	63	84.8023281	
92.8457846	84.9030219	78.3551261	80.2690501	63	82.9273281	
92.8457846	76.9030219	78.3551261	80.2690501	63	81.8023281	
92.8457846	76.9030219	78.3551261	81.2690501	63	81.6773281	
90.8457846	76.9030219	79.3551261	81.2690501	63	81.4273281	
90.8457846	75.9030219	79.3551261	81.2690501	63	81.4273281	
86.8457846	73.9030219	79.3551261	79.2690501	63	80.3023281	
86.8457846	73.9030219	80.3551261	79.2690501	63	80.4273281	
86.8457846	73.9030219	80.3551261	78.2690501	63	80.1773281	
86.8457846	73.9030219	80.3551261	78.2690501	63	80.0523281	
87.8457846	71.9030219	81.3551261	78.2690501	63	79.6773281	
87.8457846	70.9030219	83.3551261	78.2690501	63	79.5523281	
90.8457846	70.9030219	86.3551261	79.2690501	63	80.4273281	
89.8457846	70.9030219	87.3551261	80.2690501	63	80.4273281	
86.8457846	70.9030219	87.3551261	80.2690501	63	79.9273281	
86.8457846	70.9030219	88.3551261	79.2690501	63	80.0523281	
83.8457846	72.9030219	88.3551261	76.2690501	63	79.6773281	
67.8457846	73.9030219	88.3551261	75.2690501	63	77.9273281	
67.8457846	73.9030219	88.3551261	75.2690501	63	77.9273281	

60.8457846	74.9030219	88.3551261	74.2690501	63	75.9273281	
58.8457846	74.9030219	89.3551261	74.2690501	63	75.3023281	
58.8457846	76.9030219	88.3551261	74.2690501	63	75.3023281	
56.8457846	81.9030219	88.3551261	74.2690501	63	75.4273281	
56.8457846	81.9030219	86.3551261	74.2690501	63	75.1773281	
55.8457846	81.9030219	86.3551261	74.2690501	63	75.1773281	
55.8457846	81.9030219	86.3551261	74.2690501	63	75.3023281	
55.8457846	81.9030219	84.3551261	74.2690501	63	75.1773281	
55.8457846	81.9030219	80.3551261	72.2690501	63	74.3023281	
55.8457846	79.9030219	80.3551261	69.2690501	63	73.8023281	
55.8457846	80.9030219	76.3551261	69.2690501	63	73.8023281	
57.8457846	80.9030219	76.3551261	69.2690501	63	74.6773281	
58.8457846	81.9030219	77.3551261	70.2690501	63	75.1773281	
58.8457846	81.9030219	76.3551261	73.2690501	63	75.5523281	
58.8457846	82.9030219	76.3551261	75.2690501	63	75.9273281	
64.8457846	82.9030219	76.3551261	77.2690501	63	76.5523281	
67.8457846	82.9030219	76.3551261	81.2690501	63	77.3023281	
68.8457846	82.9030219	76.3551261	84.2690501	63	77.5523281	
69.8457846	82.9030219	76.3551261	84.2690501	63	77.6773281	
69.8457846	74.9030219	76.3551261	85.2690501	63	76.8023281	
67.8457846	74.9030219	76.3551261	88.2690501	63	76.5523281	
66.8457846	70.9030219	77.3551261	88.2690501	63	75.9273281	
66.8457846	61.9030219	78.3551261	90.2690501	63	75.3023281	
68.8457846	59.9030219	77.3551261	89.2690501	63	75.0523281	
71.8457846	59.9030219	77.3551261	89.2690501	63	75.4273281	
74.8457846	58.9030219	77.3551261	85.2690501	63	75.1773281	
75.8457846	57.9030219	77.3551261	85.2690501	63	75.1773281	
75.8457846	58.9030219	77.3551261	85.2690501	63	75.5523281	
76.8457846	58.9030219	77.3551261	84.2690501	63	75.6773281	
76.8457846	58.9030219	77.3551261	84.2690501	63	75.9273281	
77.8457846	58.9030219	78.3551261	84.2690501	63	76.1773281	
77.8457846	59.9030219	78.3551261	84.2690501	63	76.3023281	
77.8457846	59.9030219	79.3551261	82.2690501	63	76.1773281	
78.8457846	59.9030219	79.3551261	82.2690501	63	76.4273281	
80.8457846	60.9030219	79.3551261	82.2690501	63	76.6773281	
81.8457846	60.9030219	79.3551261	70.2690501	63	74.9273281	
81.8457846	61.9030219	79.3551261	70.2690501	63	75.0523281	
82.8457846	62.9030219	82.3551261	68.2690501	63	75.3023281	
82.8457846	63.9030219	80.3551261	68.2690501	63	75.0523281	
81.8457846	63.9030219	79.3551261	68.2690501	63	74.8023281	
80.8457846	69.9030219	79.3551261	71.2690501	63	75.9273281	
80.8457846	73.9030219	78.3551261	71.2690501	63	76.3023281	
80.8457846	74.9030219	78.3551261	71.2690501	63	76.4273281	
80.8457846	73.9030219	78.3551261	71.2690501	63	76.4273281	
79.8457846	72.9030219	78.3551261	78.2690501	63	77.0523281	

79.8457846	72.9030219	78.3551261	78.2690501	63	77.1773281	
79.8457846	72.9030219	78.3551261	75.2690501	63	76.8023281	
79.8457846	74.9030219	78.3551261	74.2690501	63	77.0523281	
79.8457846	75.9030219	79.3551261	74.2690501	63	77.0523281	
79.8457846	75.9030219	80.3551261	74.2690501	63	76.6773281	
79.8457846	75.9030219	80.3551261	74.2690501	63	76.1773281	
80.8457846	76.9030219	80.3551261	74.2690501	63	75.9273281	
80.8457846	78.9030219	80.3551261	73.2690501	63	76.3023281	
80.8457846	78.9030219	80.3551261	73.2690501	63	76.3023281	
83.8457846	78.9030219	80.3551261	73.2690501	63	77.1773281	
83.8457846	77.9030219	79.3551261	73.2690501	63	77.0523281	
84.8457846	75.9030219	77.3551261	73.2690501	63	76.6773281	
84.8457846	75.9030219	76.3551261	73.2690501	63	76.5523281	
84.8457846	75.9030219	76.3551261	73.2690501	63	76.5523281	
83.8457846	75.9030219	77.3551261	72.2690501	63	76.1773281	
83.8457846	74.9030219	78.3551261	72.2690501	63	75.9273281	
82.8457846	71.9030219	78.3551261	70.2690501	63	75.0523281	
82.8457846	70.9030219	78.3551261	72.2690501	63	74.9273281	
82.8457846	70.9030219	78.3551261	72.2690501	63	74.8023281	
82.8457846	70.9030219	79.3551261	71.2690501	63	74.6773281	
82.8457846	70.9030219	79.3551261	71.2690501	63	74.6773281	
82.8457846	69.9030219	79.3551261	72.2690501	63	74.9273281	
82.8457846	69.9030219	79.3551261	75.2690501	63	75.4273281	
81.8457846	69.9030219	77.3551261	75.2690501	63	75.1773281	
81.8457846	68.9030219	77.3551261	75.2690501	63	74.8023281	
80.8457846	68.9030219	77.3551261	76.2690501	63	74.6773281	
79.8457846	67.9030219	79.3551261	76.2690501	63	74.6773281	
79.8457846	65.9030219	80.3551261	77.2690501	63	74.4273281	
79.8457846	63.9030219	81.3551261	77.2690501	63	74.1773281	
79.8457846	62.9030219	81.3551261	76.2690501	63	73.9273281	
78.8457846	61.9030219	82.3551261	76.2690501	63	73.9273281	
78.8457846	61.9030219	82.3551261	75.2690501	63	73.8023281	
77.8457846	60.9030219	83.3551261	75.2690501	63	73.4273281	
77.8457846	61.9030219	85.3551261	75.2690501	63	74.1773281	
77.8457846	62.9030219	86.3551261	74.2690501	63	75.1773281	
75.8457846	62.9030219	84.3551261	74.2690501	63	74.8023281	
76.8457846	63.9030219	81.3551261	76.2690501	63	75.0523281	
76.8457846	63.9030219	79.3551261	77.2690501	63	75.1773281	
76.8457846	63.9030219	78.3551261	77.2690501	63	75.1773281	
76.8457846	64.9030219	77.3551261	77.2690501	63	75.3023281	
77.8457846	63.9030219	76.3551261	76.2690501	63	75.3023281	
77.8457846	63.9030219	77.3551261	77.2690501	63	75.6773281	
76.8457846	63.9030219	76.3551261	77.2690501	63	75.4273281	
76.8457846	63.9030219	76.3551261	79.2690501	63	75.6773281	
77.8457846	63.9030219	76.3551261	79.2690501	63	75.8023281	

77.8457846	63.9030219	76.3551261	80.2690501	63	75.8023281	
76.8457846	64.9030219	76.3551261	81.2690501	63	76.1773281	
76.8457846	63.9030219	76.3551261	82.2690501	63	76.1773281	
76.8457846	63.9030219	76.3551261	84.2690501	63	77.1773281	
75.8457846	63.9030219	76.3551261	84.2690501	63	76.9273281	
75.8457846	62.9030219	76.3551261	84.2690501	63	76.8023281	
75.8457846	62.9030219	75.3551261	83.2690501	63	77.0523281	
75.8457846	62.9030219	75.3551261	82.2690501	63	76.9273281	
75.8457846	61.9030219	74.3551261	80.2690501	63	76.5523281	
76.8457846	62.9030219	74.3551261	78.2690501	63	76.5523281	
77.8457846	62.9030219	74.3551261	77.2690501	63	76.5523281	
77.8457846	62.9030219	74.3551261	77.2690501	63	76.6773281	
77.8457846	62.9030219	74.3551261	77.2690501	63	77.0523281	
77.8457846	61.9030219	74.3551261	77.2690501	63	77.3023281	
77.8457846	61.9030219	74.3551261	76.2690501	63	77.1773281	
77.8457846	61.9030219	74.3551261	77.2690501	63	77.5523281	
78.8457846	61.9030219	75.3551261	77.2690501	63	77.8023281	
78.8457846	61.9030219	75.3551261	77.2690501	63	77.8023281	
80.8457846	61.9030219	75.3551261	77.2690501	63	78.3023281	
80.8457846	61.9030219	75.3551261	76.2690501	63	77.6773281	
81.8457846	61.9030219	75.3551261	76.2690501	63	77.0523281	
82.8457846	61.9030219	75.3551261	76.2690501	63	77.5523281	
82.8457846	61.9030219	73.3551261	76.2690501	63	76.8023281	
85.8457846	61.9030219	71.3551261	76.2690501	63	76.8023281	
85.8457846	61.9030219	70.3551261	77.2690501	63	76.3023281	
86.8457846	61.9030219	70.3551261	78.2690501	63	76.4273281	
73.8457846	66.9030219	70.3551261	78.2690501	63	75.4273281	
70.8457846	67.9030219	70.3551261	79.2690501	63	75.0523281	
70.8457846	68.9030219	70.3551261	81.2690501	63	75.4273281	
70.8457846	68.9030219	70.3551261	81.2690501	63	75.4273281	
69.8457846	68.9030219	70.3551261	82.2690501	63	75.1773281	
69.8457846	68.9030219	70.3551261	83.2690501	63	75.3023281	
67.8457846	69.9030219	71.3551261	83.2690501	63	75.1773281	
66.8457846	69.9030219	71.3551261	83.2690501	63	75.1773281	
66.8457846	69.9030219	70.3551261	83.2690501	63	75.0523281	
65.8457846	69.9030219	70.3551261	83.2690501	63	74.9273281	
65.8457846	69.9030219	70.3551261	83.2690501	63	74.5523281	
65.8457846	70.9030219	71.3551261	84.2690501	63	74.6773281	
66.8457846	70.9030219	71.3551261	84.2690501	63	74.8023281	
70.8457846	70.9030219	70.3551261	84.2690501	63	75.4273281	
72.8457846	70.9030219	70.3551261	83.2690501	63	75.8023281	
72.8457846	70.9030219	70.3551261	81.2690501	63	76.0523281	
73.8457846	70.9030219	70.3551261	79.2690501	63	76.4273281	
73.8457846	65.9030219	70.3551261	78.2690501	63	76.1773281	
73.8457846	64.9030219	68.3551261	78.2690501	63	75.9273281	

73.8457846	62.9030219	68.3551261	77.2690501	63	75.5523281	
73.8457846	62.9030219	68.3551261	77.2690501	63	75.3023281	
73.8457846	62.9030219	68.3551261	79.2690501	63	75.6773281	
75.8457846	61.9030219	67.3551261	78.2690501	63	75.5523281	
75.8457846	61.9030219	65.3551261	78.2690501	63	75.4273281	
76.8457846	60.9030219	65.3551261	78.2690501	63	75.3023281	
76.8457846	60.9030219	63.3551261	76.2690501	63	74.9273281	
76.8457846	60.9030219	62.3551261	76.2690501	63	74.5523281	
76.8457846	60.9030219	62.3551261	74.2690501	63	74.1773281	
77.8457846	61.9030219	62.3551261	73.2690501	63	74.1773281	
77.8457846	60.9030219	62.3551261	72.2690501	63	73.4273281	
77.8457846	60.9030219	63.3551261	74.2690501	63	73.6773281	
77.8457846	60.9030219	63.3551261	74.2690501	63	73.6773281	
76.8457846	60.9030219	66.3551261	74.2690501	63	74.1773281	
76.8457846	60.9030219	68.3551261	74.2690501	63	74.4273281	
76.8457846	60.9030219	68.3551261	73.2690501	63	74.0523281	
76.8457846	60.9030219	68.3551261	72.2690501	63	73.9273281	
74.8457846	60.9030219	69.3551261	72.2690501	63	73.6773281	
74.8457846	60.9030219	69.3551261	72.2690501	63	73.1773281	
74.8457846	61.9030219	71.3551261	72.2690501	63	73.4273281	
74.8457846	61.9030219	70.3551261	74.2690501	63	71.9273281	
74.8457846	62.9030219	70.3551261	74.2690501	63	71.6773281	
74.8457846	63.9030219	70.3551261	74.2690501	63	71.5523281	
75.8457846	63.9030219	70.3551261	74.2690501	63	71.9273281	
76.8457846	63.9030219	69.3551261	74.2690501	63	72.9273281	
76.8457846	63.9030219	68.3551261	74.2690501	63	73.1773281	
76.8457846	63.9030219	68.3551261	74.2690501	63	73.1773281	
76.8457846	63.9030219	67.3551261	74.2690501	63	73.1773281	
76.8457846	68.9030219	68.3551261	76.2690501	63	74.3023281	
76.8457846	68.9030219	69.3551261	76.2690501	63	74.4273281	
77.8457846	68.9030219	70.3551261	80.2690501	63	75.1773281	
78.8457846	68.9030219	71.3551261	80.2690501	63	75.1773281	
77.8457846	68.9030219	72.3551261	80.2690501	63	74.8023281	
77.8457846	68.9030219	72.3551261	80.2690501	63	75.0523281	
77.8457846	68.9030219	72.3551261	82.2690501	63	73.9273281	
77.8457846	68.9030219	74.3551261	86.2690501	63	73.6773281	
76.8457846	68.9030219	76.3551261	87.2690501	63	73.5523281	
76.8457846	68.9030219	77.3551261	88.2690501	63	73.8023281	
76.8457846	68.9030219	77.3551261	88.2690501	63	73.9273281	
76.8457846	69.9030219	76.3551261	88.2690501	63	74.0523281	
76.8457846	70.9030219	75.3551261	88.2690501	63	74.0523281	
76.8457846	70.9030219	74.3551261	87.2690501	63	73.9273281	
75.8457846	72.9030219	72.3551261	87.2690501	63	74.1773281	
76.8457846	73.9030219	71.3551261	87.2690501	63	74.4273281	
76.8457846	73.9030219	69.3551261	88.2690501	63	74.5523281	

76.8457846	73.9030219	69.3551261	89.2690501	63	75.1773281	
74.8457846	77.9030219	69.3551261	91.2690501	63	75.8023281	
74.8457846	78.9030219	69.3551261	93.2690501	63	76.4273281	
74.8457846	80.9030219	69.3551261	94.2690501	63	77.3023281	
73.8457846	80.9030219	70.3551261	92.2690501	63	77.1773281	
73.8457846	80.9030219	70.3551261	92.2690501	63	77.1773281	
73.8457846	80.9030219	70.3551261	92.2690501	63	77.5523281	
73.8457846	80.9030219	70.3551261	88.2690501	63	77.0523281	
73.8457846	81.9030219	70.3551261	80.2690501	63	75.5523281	
73.8457846	84.9030219	69.3551261	79.2690501	63	75.3023281	
73.8457846	84.9030219	69.3551261	79.2690501	63	75.3023281	
73.8457846	85.9030219	69.3551261	79.2690501	63	75.3023281	
73.8457846	85.9030219	69.3551261	78.2690501	63	74.9273281	
72.8457846	86.9030219	70.3551261	78.2690501	63	74.5523281	
71.8457846	86.9030219	70.3551261	78.2690501	63	74.3023281	
70.8457846	86.9030219	70.3551261	78.2690501	63	74.0523281	
70.8457846	85.9030219	70.3551261	78.2690501	63	74.0523281	
69.8457846	85.9030219	70.3551261	78.2690501	63	74.4273281	
69.8457846	86.9030219	69.3551261	78.2690501	63	74.9273281	
71.8457846	86.9030219	68.3551261	78.2690501	63	74.9273281	
74.8457846	87.9030219	68.3551261	77.2690501	63	75.3023281	
73.8457846	89.9030219	67.3551261	77.2690501	63	75.0523281	
73.8457846	80.9030219	66.3551261	77.2690501	63	73.5523281	
73.8457846	74.9030219	66.3551261	77.2690501	63	72.4273281	
73.8457846	73.9030219	66.3551261	77.2690501	63	72.1773281	
73.8457846	73.9030219	66.3551261	77.2690501	63	72.1773281	
73.8457846	69.9030219	66.3551261	76.2690501	63	71.5523281	
74.8457846	69.9030219	66.3551261	75.2690501	63	71.5523281	
74.8457846	68.9030219	66.3551261	74.2690501	63	71.3023281	
74.8457846	68.9030219	66.3551261	73.2690501	63	71.1773281	
75.8457846	67.9030219	66.3551261	73.2690501	63	71.1773281	
75.8457846	66.9030219	66.3551261	73.2690501	63	70.9273281	
76.8457846	66.9030219	66.3551261	73.2690501	63	71.1773281	
77.8457846	66.9030219	66.3551261	74.2690501	63	71.4273281	
76.8457846	66.9030219	67.3551261	75.2690501	63	71.6773281	
76.8457846	66.9030219	67.3551261	75.2690501	63	71.6773281	
76.8457846	66.9030219	66.3551261	76.2690501	63	71.6773281	
76.8457846	66.9030219	66.3551261	76.2690501	63	71.4273281	
76.8457846	65.9030219	67.3551261	74.2690501	63	71.1773281	
75.8457846	65.9030219	67.3551261	74.2690501	63	70.5523281	
75.8457846	65.9030219	67.3551261	74.2690501	63	70.3023281	
76.8457846	64.9030219	69.3551261	74.2690501	63	70.4273281	
77.8457846	64.9030219	69.3551261	74.2690501	63	70.5523281	
77.8457846	69.9030219	70.3551261	74.2690501	63	71.6773281	
78.8457846	69.9030219	70.3551261	78.2690501	63	72.0523281	

80.8457846	69.9030219	71.3551261	77.2690501	63	72.1773281	
81.8457846	69.9030219	71.3551261	76.2690501	63	71.9273281	
81.8457846	69.9030219	71.3551261	76.2690501	63	71.8023281	
81.8457846	69.9030219	70.3551261	76.2690501	63	70.9273281	
81.8457846	68.9030219	67.3551261	76.2690501	63	70.5523281	
81.8457846	68.9030219	66.3551261	78.2690501	63	70.8023281	
81.8457846	67.9030219	66.3551261	78.2690501	63	70.6773281	
81.8457846	67.9030219	66.3551261	79.2690501	63	70.9273281	
77.8457846	67.9030219	67.3551261	79.2690501	63	70.6773281	
76.8457846	67.9030219	67.3551261	80.2690501	63	70.5523281	
76.8457846	67.9030219	66.3551261	80.2690501	63	70.4273281	
77.8457846	67.9030219	66.3551261	81.2690501	63	71.0523281	
77.8457846	70.9030219	66.3551261	81.2690501	63	71.3023281	
76.8457846	82.9030219	67.3551261	79.2690501	63	72.4273281	
76.8457846	82.9030219	67.3551261	78.2690501	63	72.6773281	
75.8457846	86.9030219	67.3551261	77.2690501	63	72.9273281	
75.8457846	86.9030219	67.3551261	78.2690501	63	73.1773281	
76.8457846	88.9030219	67.3551261	80.2690501	63	74.8023281	
76.8457846	95.9030219	67.3551261	82.2690501	63	76.3023281	
77.8457846	95.9030219	67.3551261	84.2690501	63	77.3023281	
77.8457846	95.9030219	67.3551261	85.2690501	63	77.5523281	
77.8457846	96.9030219	67.3551261	85.2690501	63	77.8023281	
78.8457846	98.9030219	67.3551261	86.2690501	63	78.5523281	
76.8457846	98.9030219	67.3551261	89.2690501	63	79.1773281	
77.8457846	98.9030219	67.3551261	89.2690501	63	79.1773281	
77.8457846	102.903022	67.3551261	88.2690501	63	79.4273281	
77.8457846	102.903022	68.3551261	87.2690501	63	79.6773281	
77.8457846	102.903022	68.3551261	84.2690501	63	79.1773281	
77.8457846	107.903022	69.3551261	81.2690501	63	79.6773281	
77.8457846	107.903022	69.3551261	80.2690501	63	79.5523281	
76.8457846	108.903022	69.3551261	79.2690501	63	79.4273281	
76.8457846	108.903022	69.3551261	81.2690501	63	79.6773281	
77.8457846	108.903022	69.3551261	82.2690501	63	80.0523281	
77.8457846	109.903022	69.3551261	79.2690501	63	79.6773281	
78.8457846	109.903022	69.3551261	78.2690501	63	79.4273281	
78.8457846	111.903022	69.3551261	76.2690501	63	79.4273281	
78.8457846	111.903022	69.3551261	74.2690501	63	79.3023281	
78.8457846	111.903022	69.3551261	74.2690501	63	79.4273281	
78.8457846	112.903022	70.3551261	74.2690501	63	79.6773281	
79.8457846	114.903022	70.3551261	74.2690501	63	79.5523281	
79.8457846	114.903022	71.3551261	75.2690501	63	79.3023281	
78.8457846	114.903022	71.3551261	76.2690501	63	79.1773281	
76.8457846	114.903022	70.3551261	76.2690501	63	78.6773281	
74.8457846	114.903022	70.3551261	76.2690501	63	78.4273281	
74.8457846	114.903022	70.3551261	76.2690501	63	78.4273281	

72.8457846	116.903022	70.3551261	76.2690501	63	78.5523281	
72.8457846	113.903022	71.3551261	76.2690501	63	78.4273281	
72.8457846	111.903022	71.3551261	76.2690501	63	79.1773281	
72.8457846	109.903022	73.3551261	76.2690501	63	79.8023281	
70.8457846	108.903022	74.3551261	76.2690501	63	79.6773281	
70.8457846	108.903022	76.3551261	77.2690501	63	79.9273281	
70.8457846	108.903022	74.3551261	77.2690501	63	79.5523281	
72.8457846	108.903022	74.3551261	76.2690501	63	79.6773281	
72.8457846	109.903022	74.3551261	75.2690501	63	79.0523281	
72.8457846	110.903022	74.3551261	75.2690501	63	79.0523281	
72.8457846	111.903022	74.3551261	70.2690501	63	78.3023281	
72.8457846	111.903022	73.3551261	67.2690501	63	77.5523281	
72.8457846	111.903022	71.3551261	72.2690501	63	77.9273281	
72.8457846	112.903022	71.3551261	72.2690501	63	78.3023281	
72.8457846	113.903022	71.3551261	74.2690501	63	78.8023281	
73.8457846	113.903022	71.3551261	76.2690501	63	79.1773281	
74.8457846	113.903022	71.3551261	75.2690501	63	79.4273281	
74.8457846	114.903022	71.3551261	75.2690501	63	79.4273281	
74.8457846	115.903022	71.3551261	74.2690501	63	79.1773281	
74.8457846	117.903022	71.3551261	72.2690501	63	79.0523281	
74.8457846	117.903022	71.3551261	72.2690501	63	78.8023281	
74.8457846	117.903022	71.3551261	71.2690501	63	78.4273281	
74.8457846	118.903022	72.3551261	71.2690501	63	78.9273281	
75.8457846	118.903022	72.3551261	70.2690501	63	78.8023281	
75.8457846	118.903022	73.3551261	70.2690501	63	78.9273281	
75.8457846	118.903022	73.3551261	71.2690501	63	79.1773281	
76.8457846	118.903022	73.3551261	72.2690501	63	79.3023281	
78.8457846	117.903022	73.3551261	72.2690501	63	79.5523281	
78.8457846	117.903022	75.3551261	72.2690501	63	79.6773281	
77.8457846	117.903022	76.3551261	73.2690501	63	79.8023281	
77.8457846	117.903022	77.3551261	77.2690501	63	80.4273281	
77.8457846	116.903022	78.3551261	77.2690501	63	80.5523281	
77.8457846	115.903022	78.3551261	79.2690501	63	80.8023281	
77.8457846	115.903022	78.3551261	78.2690501	63	80.5523281	
77.8457846	115.903022	79.3551261	78.2690501	63	81.1773281	
78.8457846	115.903022	79.3551261	78.2690501	63	80.8023281	
82.8457846	116.903022	79.3551261	78.2690501	63	81.1773281	
82.8457846	116.903022	80.3551261	79.2690501	63	81.5523281	
89.8457846	116.903022	80.3551261	80.2690501	63	82.9273281	
89.8457846	116.903022	80.3551261	81.2690501	63	83.0523281	
90.8457846	117.903022	80.3551261	83.2690501	63	83.5523281	
90.8457846	117.903022	80.3551261	84.2690501	63	83.3023281	
91.8457846	117.903022	79.3551261	84.2690501	63	82.9273281	
90.8457846	117.903022	78.3551261	83.2690501	63	82.3023281	
91.8457846	117.903022	77.3551261	81.2690501	63	81.9273281	

91.8457846	116.903022	76.3551261	79.2690501	63	81.4273281	
91.8457846	116.903022	74.3551261	78.2690501	63	81.0523281	
91.8457846	116.903022	73.3551261	77.2690501	63	80.8023281	
92.8457846	116.903022	73.3551261	77.2690501	63	80.9273281	
93.8457846	115.903022	73.3551261	78.2690501	63	80.9273281	
94.8457846	115.903022	73.3551261	79.2690501	63	81.5523281	
94.8457846	115.903022	73.3551261	82.2690501	63	81.9273281	
94.8457846	115.903022	73.3551261	85.2690501	63	82.5523281	
94.8457846	115.903022	73.3551261	85.2690501	63	82.5523281	
95.8457846	116.903022	73.3551261	85.2690501	63	82.4273281	
96.8457846	116.903022	73.3551261	89.2690501	63	82.9273281	
96.8457846	116.903022	73.3551261	89.2690501	63	83.0523281	
96.8457846	117.903022	73.3551261	89.2690501	63	83.3023281	
94.8457846	117.903022	73.3551261	89.2690501	63	83.1773281	
93.8457846	117.903022	73.3551261	89.2690501	63	83.4273281	
93.8457846	117.903022	73.3551261	89.2690501	63	83.5523281	
92.8457846	117.903022	73.3551261	86.2690501	63	83.0523281	
92.8457846	117.903022	75.3551261	84.2690501	63	83.0523281	
91.8457846	111.903022	78.3551261	81.2690501	63	82.1773281	
90.8457846	87.9030219	80.3551261	80.2690501	63	79.3023281	
89.8457846	76.9030219	80.3551261	79.2690501	63	78.3023281	
89.8457846	76.9030219	79.3551261	76.2690501	63	77.8023281	
87.8457846	66.9030219	79.3551261	76.2690501	63	76.5523281	
85.8457846	63.9030219	78.3551261	76.2690501	63	75.9273281	
84.8457846	61.9030219	78.3551261	76.2690501	63	75.5523281	
83.8457846	61.9030219	77.3551261	76.2690501	63	75.4273281	
83.8457846	61.9030219	77.3551261	76.2690501	63	75.3023281	
83.8457846	61.9030219	76.3551261	77.2690501	63	76.1773281	
82.8457846	61.9030219	76.3551261	77.2690501	63	76.1773281	
81.8457846	61.9030219	75.3551261	78.2690501	63	76.0523281	
81.8457846	61.9030219	74.3551261	77.2690501	63	75.9273281	
81.8457846	62.9030219	73.3551261	77.2690501	63	75.8023281	
80.8457846	62.9030219	72.3551261	76.2690501	63	74.6773281	
80.8457846	61.9030219	72.3551261	76.2690501	63	73.8023281	
79.8457846	62.9030219	72.3551261	76.2690501	63	73.8023281	
79.8457846	63.9030219	72.3551261	76.2690501	63	73.6773281	
79.8457846	66.9030219	72.3551261	78.2690501	63	74.0523281	
79.8457846	69.9030219	71.3551261	80.2690501	63	74.4273281	
80.8457846	70.9030219	71.3551261	80.2690501	63	74.4273281	
81.8457846	70.9030219	71.3551261	80.2690501	63	74.4273281	
81.8457846	70.9030219	71.3551261	81.2690501	63	74.4273281	
80.8457846	68.9030219	71.3551261	81.2690501	63	74.1773281	
78.8457846	67.9030219	70.3551261	81.2690501	63	73.5523281	
74.8457846	66.9030219	69.3551261	81.2690501	63	72.5523281	
72.8457846	66.9030219	68.3551261	81.2690501	63	72.1773281	

72.8457846	65.9030219	68.3551261	81.2690501	63	71.9273281	
72.8457846	65.9030219	69.3551261	81.2690501	63	71.9273281	
73.8457846	65.9030219	70.3551261	78.2690501	63	71.0523281	
74.8457846	65.9030219	70.3551261	74.2690501	63	70.6773281	
74.8457846	65.9030219	71.3551261	74.2690501	63	70.9273281	
74.8457846	65.9030219	71.3551261	74.2690501	63	70.9273281	
75.8457846	66.9030219	71.3551261	76.2690501	63	71.3023281	
76.8457846	69.9030219	71.3551261	78.2690501	63	72.0523281	
77.8457846	69.9030219	71.3551261	79.2690501	63	72.5523281	
77.8457846	69.9030219	71.3551261	79.2690501	63	72.8023281	
76.8457846	68.9030219	70.3551261	79.2690501	63	72.6773281	
76.8457846	67.9030219	70.3551261	79.2690501	63	72.6773281	
76.8457846	67.9030219	70.3551261	78.2690501	63	73.0523281	
75.8457846	67.9030219	70.3551261	77.2690501	63	72.8023281	
75.8457846	67.9030219	69.3551261	77.2690501	63	72.8023281	
75.8457846	66.9030219	68.3551261	77.2690501	63	72.8023281	
75.8457846	66.9030219	68.3551261	78.2690501	63	73.0523281	
75.8457846	66.9030219	68.3551261	78.2690501	63	73.0523281	
75.8457846	67.9030219	68.3551261	78.2690501	63	73.8023281	
75.8457846	67.9030219	68.3551261	78.2690501	63	73.9273281	
79.8457846	68.9030219	68.3551261	76.2690501	63	74.4273281	
80.8457846	69.9030219	68.3551261	74.2690501	63	74.6773281	
80.8457846	71.9030219	68.3551261	74.2690501	63	74.8023281	
80.8457846	71.9030219	68.3551261	72.2690501	63	74.5523281	
80.8457846	70.9030219	69.3551261	71.2690501	63	74.3023281	
79.8457846	69.9030219	70.3551261	71.2690501	63	73.8023281	
78.8457846	69.9030219	72.3551261	71.2690501	63	74.3023281	
77.8457846	69.9030219	72.3551261	71.2690501	63	74.8023281	
77.8457846	69.9030219	74.3551261	72.2690501	63	74.8023281	
77.8457846	69.9030219	73.3551261	72.2690501	63	74.6773281	
75.8457846	69.9030219	73.3551261	71.2690501	63	73.9273281	
74.8457846	69.9030219	72.3551261	70.2690501	63	73.6773281	
72.8457846	69.9030219	72.3551261	70.2690501	63	73.1773281	
72.8457846	69.9030219	72.3551261	70.2690501	63	72.9273281	
69.8457846	69.9030219	72.3551261	71.2690501	63	72.4273281	
69.8457846	69.9030219	72.3551261	71.2690501	63	72.3023281	
69.8457846	69.9030219	72.3551261	74.2690501	63	72.6773281	
70.8457846	68.9030219	72.3551261	75.2690501	63	71.9273281	
71.8457846	68.9030219	72.3551261	76.2690501	63	72.1773281	
71.8457846	68.9030219	71.3551261	76.2690501	63	71.6773281	
71.8457846	69.9030219	71.3551261	76.2690501	63	71.8023281	
71.8457846	69.9030219	71.3551261	76.2690501	63	71.9273281	
71.8457846	69.9030219	71.3551261	76.2690501	63	72.4273281	
72.8457846	69.9030219	71.3551261	76.2690501	63	72.9273281	
72.8457846	68.9030219	71.3551261	77.2690501	63	73.4273281	

72.8457846	68.9030219	71.3551261	76.2690501	63	74.1773281	
72.8457846	68.9030219	70.3551261	76.2690501	63	74.4273281	
72.8457846	67.9030219	70.3551261	76.2690501	63	74.4273281	
72.8457846	67.9030219	70.3551261	76.2690501	63	74.5523281	
74.8457846	67.9030219	70.3551261	76.2690501	63	74.6773281	
74.8457846	67.9030219	71.3551261	76.2690501	63	74.8023281	
75.8457846	67.9030219	72.3551261	76.2690501	63	75.4273281	
75.8457846	66.9030219	73.3551261	76.2690501	63	75.4273281	
75.8457846	67.9030219	74.3551261	75.2690501	63	75.4273281	
75.8457846	69.9030219	74.3551261	75.2690501	63	75.9273281	
75.8457846	72.9030219	74.3551261	74.2690501	63	76.6773281	
74.8457846	72.9030219	75.3551261	73.2690501	63	76.5523281	
73.8457846	72.9030219	77.3551261	73.2690501	63	76.6773281	
75.8457846	72.9030219	77.3551261	71.2690501	63	76.8023281	
76.8457846	69.9030219	79.3551261	71.2690501	63	76.9273281	
76.8457846	69.9030219	80.3551261	70.2690501	63	76.0523281	
78.8457846	68.9030219	80.3551261	69.2690501	63	76.0523281	
79.8457846	67.9030219	80.3551261	69.2690501	63	76.4273281	
82.8457846	67.9030219	80.3551261	69.2690501	63	76.6773281	
77.8457846	67.9030219	80.3551261	70.2690501	63	75.6773281	
77.8457846	67.9030219	80.3551261	70.2690501	63	75.1773281	
74.8457846	67.9030219	80.3551261	70.2690501	63	74.5523281	
72.8457846	67.9030219	81.3551261	70.2690501	63	74.3023281	
72.8457846	67.9030219	81.3551261	70.2690501	63	74.3023281	
72.8457846	66.9030219	80.3551261	72.2690501	63	74.3023281	
72.8457846	66.9030219	79.3551261	72.2690501	63	74.4273281	
72.8457846	66.9030219	79.3551261	72.2690501	63	74.4273281	
72.8457846	66.9030219	78.3551261	76.2690501	63	74.5523281	
72.8457846	66.9030219	78.3551261	79.2690501	63	74.9273281	
72.8457846	66.9030219	78.3551261	79.2690501	63	74.9273281	
72.8457846	66.9030219	78.3551261	81.2690501	63	75.1773281	
72.8457846	66.9030219	76.3551261	81.2690501	63	74.9273281	
70.8457846	71.9030219	75.3551261	81.2690501	63	75.1773281	
68.8457846	71.9030219	74.3551261	81.2690501	63	75.0523281	
68.8457846	70.9030219	74.3551261	81.2690501	63	75.4273281	
68.8457846	70.9030219	74.3551261	81.2690501	63	75.5523281	
68.8457846	69.9030219	74.3551261	81.2690501	63	74.9273281	
68.8457846	69.9030219	74.3551261	81.2690501	63	74.8023281	
68.8457846	69.9030219	73.3551261	81.2690501	63	74.3023281	
68.8457846	69.9030219	73.3551261	80.2690501	63	73.9273281	
71.8457846	69.9030219	73.3551261	80.2690501	63	74.3023281	
72.8457846	70.9030219	74.3551261	80.2690501	63	74.6773281	
73.8457846	70.9030219	74.3551261	78.2690501	63	74.5523281	
73.8457846	70.9030219	74.3551261	72.2690501	63	73.8023281	
73.8457846	70.9030219	74.3551261	72.2690501	63	73.5523281	

73.8457846	70.9030219	73.3551261	69.2690501	63	72.8023281	
73.8457846	70.9030219	74.3551261	67.2690501	63	72.5523281	
73.8457846	70.9030219	75.3551261	67.2690501	63	72.9273281	
74.8457846	68.9030219	75.3551261	65.2690501	63	72.3023281	
74.8457846	67.9030219	77.3551261	65.2690501	63	72.5523281	
75.8457846	67.9030219	79.3551261	65.2690501	63	72.9273281	
76.8457846	67.9030219	80.3551261	66.2690501	63	73.4273281	
76.8457846	68.9030219	80.3551261	67.2690501	63	73.4273281	
76.8457846	69.9030219	80.3551261	67.2690501	63	73.3023281	
76.8457846	69.9030219	79.3551261	67.2690501	63	73.4273281	
77.8457846	69.9030219	79.3551261	67.2690501	63	73.1773281	
78.8457846	69.9030219	78.3551261	67.2690501	63	72.6773281	
79.8457846	69.9030219	77.3551261	67.2690501	63	72.5523281	
79.8457846	69.9030219	77.3551261	67.2690501	63	72.5523281	
79.8457846	70.9030219	77.3551261	67.2690501	63	72.5523281	
79.8457846	70.9030219	76.3551261	67.2690501	63	72.3023281	
72.8457846	70.9030219	76.3551261	67.2690501	63	71.0523281	
69.8457846	70.9030219	76.3551261	70.2690501	63	70.9273281	
69.8457846	70.9030219	75.3551261	70.2690501	63	70.8023281	
69.8457846	70.9030219	75.3551261	70.2690501	63	70.8023281	
69.8457846	70.9030219	74.3551261	69.2690501	63	70.6773281	
69.8457846	71.9030219	74.3551261	70.2690501	63	70.9273281	
69.8457846	72.9030219	73.3551261	70.2690501	63	70.8023281	
71.8457846	72.9030219	73.3551261	70.2690501	63	71.1773281	
71.8457846	75.9030219	72.3551261	70.2690501	63	71.4273281	
69.8457846	74.9030219	72.3551261	70.2690501	63	71.0523281	
67.8457846	74.9030219	72.3551261	71.2690501	63	71.0523281	
67.8457846	73.9030219	73.3551261	71.2690501	63	70.9273281	
67.8457846	72.9030219	74.3551261	71.2690501	63	71.5523281	
67.8457846	67.9030219	74.3551261	71.2690501	63	71.3023281	
67.8457846	67.9030219	74.3551261	71.2690501	63	71.4273281	
67.8457846	67.9030219	74.3551261	71.2690501	63	71.3023281	
67.8457846	67.9030219	74.3551261	71.2690501	63	71.1773281	
68.8457846	67.9030219	75.3551261	71.2690501	63	71.4273281	
68.8457846	66.9030219	76.3551261	75.2690501	63	72.0523281	
68.8457846	66.9030219	76.3551261	75.2690501	63	72.0523281	
70.8457846	63.9030219	75.3551261	76.2690501	63	72.9273281	
71.8457846	62.9030219	76.3551261	76.2690501	63	73.5523281	
73.8457846	62.9030219	77.3551261	76.2690501	63	74.5523281	
74.8457846	62.9030219	77.3551261	76.2690501	63	74.6773281	
74.8457846	63.9030219	77.3551261	75.2690501	63	74.9273281	
74.8457846	63.9030219	77.3551261	72.2690501	63	74.5523281	
74.8457846	63.9030219	77.3551261	70.2690501	63	74.4273281	
74.8457846	63.9030219	77.3551261	69.2690501	63	74.3023281	
71.8457846	63.9030219	76.3551261	69.2690501	63	73.6773281	

69.8457846	64.9030219	76.3551261	69.2690501	63	73.4273281	
69.8457846	64.9030219	76.3551261	71.2690501	63	73.8023281	
69.8457846	64.9030219	76.3551261	71.2690501	63	74.0523281	
69.8457846	64.9030219	76.3551261	71.2690501	63	74.1773281	
69.8457846	64.9030219	76.3551261	70.2690501	63	73.9273281	
69.8457846	64.9030219	76.3551261	70.2690501	63	73.9273281	
69.8457846	62.9030219	76.3551261	70.2690501	63	73.5523281	
73.8457846	62.9030219	76.3551261	70.2690501	63	74.0523281	
73.8457846	62.9030219	77.3551261	70.2690501	63	74.8023281	
73.8457846	62.9030219	79.3551261	70.2690501	63	75.1773281	
73.8457846	62.9030219	80.3551261	71.2690501	63	75.4273281	
73.8457846	62.9030219	80.3551261	72.2690501	63	75.3023281	
74.8457846	61.9030219	79.3551261	72.2690501	63	75.1773281	
74.8457846	61.9030219	78.3551261	71.2690501	63	75.1773281	
80.8457846	57.9030219	78.3551261	70.2690501	63	75.1773281	
81.8457846	56.9030219	76.3551261	70.2690501	63	74.9273281	
82.8457846	56.9030219	75.3551261	71.2690501	63	75.4273281	
82.8457846	62.9030219	74.3551261	71.2690501	63	75.8023281	
82.8457846	63.9030219	75.3551261	72.2690501	63	76.3023281	
81.8457846	63.9030219	76.3551261	71.2690501	63	76.1773281	
81.8457846	65.9030219	76.3551261	70.2690501	63	76.1773281	
81.8457846	66.9030219	75.3551261	69.2690501	63	75.8023281	
81.8457846	67.9030219	74.3551261	69.2690501	63	75.9273281	
81.8457846	68.9030219	74.3551261	69.2690501	63	75.8023281	
82.8457846	68.9030219	74.3551261	70.2690501	63	75.8023281	
82.8457846	69.9030219	74.3551261	71.2690501	63	76.1773281	
80.8457846	69.9030219	73.3551261	71.2690501	63	75.8023281	
80.8457846	69.9030219	73.3551261	71.2690501	63	75.5523281	
77.8457846	66.9030219	74.3551261	71.2690501	63	74.8023281	
75.8457846	65.9030219	75.3551261	71.2690501	63	74.4273281	
74.8457846	64.9030219	75.3551261	71.2690501	63	74.3023281	
73.8457846	64.9030219	76.3551261	71.2690501	63	74.0523281	
72.8457846	65.9030219	76.3551261	71.2690501	63	74.0523281	
72.8457846	65.9030219	76.3551261	71.2690501	63	73.9273281	
72.8457846	65.9030219	77.3551261	72.2690501	63	74.0523281	
70.8457846	66.9030219	77.3551261	72.2690501	63	73.9273281	
70.8457846	66.9030219	77.3551261	72.2690501	63	73.8023281	
70.8457846	66.9030219	77.3551261	72.2690501	63	73.6773281	
70.8457846	66.9030219	77.3551261	71.2690501	63	73.3023281	
70.8457846	66.9030219	77.3551261	71.2690501		73.0523281	
71.8457846	66.9030219	78.3551261	70.2690501		73.3023281	
71.8457846	65.9030219	78.3551261	70.2690501		73.0523281	
72.8457846	64.9030219	79.3551261	70.2690501		73.3023281	
71.8457846	63.9030219	80.3551261	70.2690501		73.1773281	
71.8457846	63.9030219	80.3551261	70.2690501		73.1773281	

71.8457846	63.9030219	82.3551261	70.2690501		73.9273281	
67.8457846	63.9030219	82.3551261	70.2690501		73.5523281	
66.8457846	63.9030219	83.3551261	70.2690501		73.5523281	
66.8457846	64.9030219	85.3551261	71.2690501		74.3023281	
66.8457846	64.9030219	85.3551261	70.2690501		74.3023281	
66.8457846	68.9030219	86.3551261	70.2690501		75.1773281	
66.8457846	68.9030219	86.3551261	70.2690501		75.1773281	
65.8457846	68.9030219	86.3551261	71.2690501		75.1773281	
65.8457846	68.9030219	87.3551261	72.2690501		75.3023281	
65.8457846	68.9030219	88.3551261	72.2690501		75.0523281	
65.8457846	68.9030219	88.3551261	72.2690501		74.9273281	
65.8457846	68.9030219	84.3551261	71.2690501		74.1773281	
65.8457846	68.9030219	76.3551261	71.2690501		72.9273281	
65.8457846	68.9030219	72.3551261	70.2690501		72.1773281	
66.8457846	68.9030219	71.3551261	70.2690501		72.1773281	
67.8457846	68.9030219	70.3551261	71.2690501		72.4273281	
67.8457846	68.9030219	70.3551261	72.2690501		72.6773281	
70.8457846	69.9030219	69.3551261	73.2690501		73.6773281	
71.8457846	69.9030219	69.3551261	75.2690501		74.0523281	
72.8457846	69.9030219	69.3551261	75.2690501		74.1773281	
71.8457846	66.9030219	68.3551261	75.2690501		72.4273281	
70.8457846	64.9030219	67.3551261	73.2690501		71.1773281	
70.8457846	64.9030219	67.3551261	72.2690501		71.0523281	
71.8457846	63.9030219	67.3551261	70.2690501		70.6773281	
71.8457846	63.9030219	68.3551261	70.2690501		70.8023281	
71.8457846	63.9030219	68.3551261	69.2690501		70.3023281	
72.8457846	63.9030219	69.3551261	69.2690501		70.8023281	
72.8457846	64.9030219	69.3551261	69.2690501		70.9273281	
71.8457846	64.9030219	69.3551261	69.2690501		70.4273281	
69.8457846	64.9030219	69.3551261	70.2690501		70.3023281	
67.8457846	61.9030219	71.3551261	71.2690501		70.0523281	
66.8457846	60.9030219	71.3551261	71.2690501		69.9273281	
65.8457846	60.9030219	71.3551261	73.2690501		70.0523281	
65.8457846	63.9030219	70.3551261	76.2690501		70.6773281	
66.8457846	64.9030219	70.3551261	78.2690501		71.3023281	
66.8457846	64.9030219	68.3551261	79.2690501		71.8023281	
65.8457846	64.9030219	67.3551261	79.2690501		72.0523281	
67.8457846	64.9030219	67.3551261	79.2690501		72.4273281	
67.8457846	65.9030219	68.3551261	79.2690501		72.4273281	
69.8457846	65.9030219	69.3551261	79.2690501		72.8023281	
70.8457846	66.9030219	69.3551261	78.2690501		73.0523281	
72.8457846	66.9030219	70.3551261	78.2690501		73.6773281	
72.8457846	65.9030219	70.3551261	78.2690501		73.8023281	
73.8457846	65.9030219	70.3551261	78.2690501		74.0523281	
73.8457846	65.9030219	71.3551261	76.2690501		75.5523281	

75.8457846	63.9030219	71.3551261	75.2690501		77.4273281	
76.8457846	63.9030219	70.3551261	74.2690501		78.4273281	
76.8457846	64.9030219	71.3551261	74.2690501		79.1773281	
76.8457846	64.9030219	72.3551261	74.2690501		79.1773281	
76.8457846	64.9030219	73.3551261	72.2690501		78.4273281	
76.8457846	66.9030219	73.3551261	71.2690501		78.5523281	
75.8457846	66.9030219	73.3551261	72.2690501		78.0523281	
80.8457846	66.9030219	73.3551261	73.2690501		78.8023281	
80.8457846	66.9030219	73.3551261	73.2690501		78.8023281	
78.8457846	67.9030219	74.3551261	75.2690501		80.1773281	
77.8457846	67.9030219	74.3551261	75.2690501		81.4273281	
75.8457846	67.9030219	74.3551261	75.2690501		81.0523281	
74.8457846	67.9030219	74.3551261	74.2690501		80.9273281	
74.8457846	67.9030219	74.3551261	74.2690501		80.9273281	
75.8457846	67.9030219	75.3551261	73.2690501		80.9273281	
75.8457846	67.9030219	75.3551261	73.2690501		81.0523281	
75.8457846	67.9030219	76.3551261	74.2690501		81.3023281	
75.8457846	67.9030219	76.3551261	75.2690501		80.1773281	
75.8457846	68.9030219	77.3551261	75.2690501		79.1773281	
76.8457846	68.9030219	77.3551261	76.2690501		78.4273281	
76.8457846	69.9030219	78.3551261	76.2690501		78.3023281	
77.8457846	69.9030219	78.3551261	76.2690501		78.4273281	
78.8457846	69.9030219	78.3551261	77.2690501		78.6773281	
78.8457846	70.9030219	79.3551261	77.2690501		79.0523281	
78.8457846	70.9030219	79.3551261	77.2690501		79.0523281	
79.8457846	70.9030219	79.3551261	77.2690501		79.1773281	
79.8457846	70.9030219	79.3551261	77.2690501		78.6773281	
79.8457846	72.9030219	79.3551261	76.2690501		78.5523281	
79.8457846	73.9030219	79.3551261	73.2690501		78.3023281	
77.8457846	74.9030219	79.3551261	73.2690501		78.0523281	
75.8457846	74.9030219	80.3551261	73.2690501		78.0523281	
74.8457846	74.9030219	81.3551261	73.2690501		77.4273281	
74.8457846	75.9030219	81.3551261	73.2690501		76.9273281	
74.8457846	75.9030219	82.3551261	73.2690501		76.5523281	
75.8457846	75.9030219	82.3551261	73.2690501		76.8023281	
75.8457846	75.9030219	82.3551261	71.2690501		76.8023281	
76.8457846	75.9030219	82.3551261	70.2690501		76.8023281	
76.8457846	70.9030219	83.3551261	70.2690501		76.1773281	
74.8457846	70.9030219	83.3551261	70.2690501		76.3023281	
73.8457846	66.9030219	82.3551261	69.2690501		75.9273281	
71.8457846	65.9030219	80.3551261	69.2690501		75.3023281	
71.8457846	64.9030219	80.3551261	69.2690501		75.3023281	
72.8457846	64.9030219	80.3551261	69.2690501		75.4273281	
72.8457846	65.9030219	80.3551261	70.2690501		75.6773281	
73.8457846	64.9030219	80.3551261	70.2690501		75.6773281	

74.8457846	64.9030219	79.3551261	69.2690501		75.6773281	
74.8457846	64.9030219	79.3551261	67.2690501		75.1773281	
74.8457846	63.9030219	78.3551261	64.2690501		74.6773281	
73.8457846	63.9030219	77.3551261	62.2690501		73.8023281	
74.8457846	63.9030219	77.3551261	61.2690501		73.9273281	
76.8457846	62.9030219	77.3551261	61.2690501		74.0523281	
77.8457846	62.9030219	77.3551261	62.2690501		74.0523281	
77.8457846	62.9030219	77.3551261	63.2690501		74.0523281	
78.8457846	63.9030219	77.3551261	65.2690501		74.4273281	
78.8457846	63.9030219	77.3551261	66.2690501		74.8023281	
78.8457846	63.9030219	79.3551261	67.2690501		75.1773281	
78.8457846	64.9030219	79.3551261	67.2690501		75.3023281	
78.8457846	64.9030219	81.3551261	68.2690501		75.9273281	
80.8457846	64.9030219	82.3551261	67.2690501		76.1773281	
79.8457846	67.9030219	83.3551261	67.2690501		76.5523281	
79.8457846	73.9030219	84.3551261	66.2690501		77.3023281	
76.8457846	73.9030219	84.3551261	65.2690501		76.8023281	
76.8457846	73.9030219	84.3551261	65.2690501		76.8023281	
72.8457846	70.9030219	85.3551261	65.2690501		75.6773281	
72.8457846	70.9030219	85.3551261	66.2690501		75.5523281	
73.8457846	67.9030219	87.3551261	67.2690501		75.5523281	
74.8457846	67.9030219	87.3551261	68.2690501		75.6773281	
74.8457846	67.9030219	87.3551261	68.2690501		75.8023281	
74.8457846	67.9030219	87.3551261	68.2690501		76.1773281	
74.8457846	67.9030219	88.3551261	67.2690501		76.8023281	
74.8457846	67.9030219	88.3551261	66.2690501		76.5523281	
74.8457846	67.9030219	86.3551261	66.2690501		75.6773281	
76.8457846	68.9030219	85.3551261	65.2690501		75.0523281	
76.8457846	68.9030219	82.3551261	66.2690501		74.4273281	
76.8457846	69.9030219	80.3551261	66.2690501		74.3023281	
76.8457846	71.9030219	80.3551261	67.2690501		74.4273281	
77.8457846	71.9030219	79.3551261	67.2690501		73.6773281	
78.8457846	74.9030219	78.3551261	67.2690501		73.6773281	
79.8457846	76.9030219	78.3551261	67.2690501		74.3023281	
79.8457846	76.9030219	78.3551261	67.2690501		74.4273281	
67.8457846	79.9030219	78.3551261	67.2690501		73.6773281	
64.8457846	74.9030219	77.3551261	69.2690501		72.8023281	
64.8457846	74.9030219	77.3551261	69.2690501		73.1773281	
66.8457846	65.9030219	77.3551261	70.2690501		72.6773281	
68.8457846	64.9030219	77.3551261	71.2690501		73.6773281	
70.8457846	64.9030219	77.3551261	71.2690501		74.5523281	
72.8457846	63.9030219	77.3551261	72.2690501		75.1773281	
73.8457846	63.9030219	77.3551261	72.2690501		75.5523281	
74.8457846	63.9030219	78.3551261	70.2690501		75.9273281	
74.8457846	63.9030219	76.3551261	69.2690501		75.8023281	

75.8457846	63.9030219	74.3551261	68.2690501		75.4273281	
75.8457846	63.9030219	73.3551261	68.2690501		75.1773281	
76.8457846	63.9030219	73.3551261	69.2690501		73.9273281	
77.8457846	63.9030219	72.3551261	69.2690501		73.6773281	
78.8457846	63.9030219	72.3551261	70.2690501		73.8023281	
78.8457846	62.9030219	71.3551261	69.2690501		73.8023281	
79.8457846	61.9030219	72.3551261	69.2690501		73.9273281	
79.8457846	61.9030219	72.3551261	69.2690501		74.3023281	
79.8457846	61.9030219	74.3551261	69.2690501		74.5523281	
78.8457846	62.9030219	77.3551261	69.2690501		74.9273281	
77.8457846	62.9030219	77.3551261	69.2690501		74.4273281	
76.8457846	63.9030219	76.3551261	69.2690501		73.5523281	
76.8457846	65.9030219	74.3551261	69.2690501		73.1773281	
76.8457846	66.9030219	74.3551261	68.2690501		72.6773281	
76.8457846	66.9030219	74.3551261	68.2690501		72.8023281	
76.8457846	66.9030219	74.3551261	68.2690501		72.5523281	
75.8457846	66.9030219	74.3551261	67.2690501		72.4273281	
73.8457846	66.9030219	74.3551261	67.2690501		72.0523281	
71.8457846	67.9030219	73.3551261	67.2690501		71.6773281	
71.8457846	67.9030219	74.3551261	66.2690501		71.8023281	
70.8457846	67.9030219	76.3551261	65.2690501		72.0523281	
70.8457846	67.9030219	78.3551261	65.2690501		72.3023281	
70.8457846	67.9030219	78.3551261	65.2690501		72.8023281	
71.8457846	67.9030219	78.3551261	65.2690501		72.8023281	
71.8457846	65.9030219	78.3551261	65.2690501		72.9273281	
72.8457846	64.9030219	78.3551261	70.2690501		73.5523281	
72.8457846	64.9030219	78.3551261	70.2690501		73.5523281	
72.8457846	65.9030219	78.3551261	77.2690501		74.8023281	
72.8457846	65.9030219	80.3551261	77.2690501		75.0523281	
73.8457846	65.9030219	80.3551261	80.2690501		75.6773281	
73.8457846	66.9030219	80.3551261	80.2690501		75.8023281	
76.8457846	66.9030219	75.3551261	79.2690501		75.1773281	
73.8457846	66.9030219	75.3551261	78.2690501		74.6773281	

moving average	
87.1464035	
87.2787765	
87.4309412	
87.5685225	
87.6488122	
87.7082685	
87.7989748	
87.8865562	
88.0147625	
88.1596355	
88.3284668	
88.4785482	
88.6452962	
88.8026692	
88.9600422	
89.1163735	
89.2633298	
89.4040362	
89.5499508	
89.7114905	
89.8636552	
90.0210282	
89.9453239	
89.8995303	
89.7921296	
89.6570503	
89.4960781	
89.3617031	
89.2148281	
89.0710781	
88.9179531	
88.7773281	
88.6492031	
88.5273281	
88.3929531	
88.2867031	
88.2023281	
88.1210781	
88.0367031	
87.9304531	
87.8242031	
87.7242031	
87.6085781	

87.4898281	
87.3585781	
87.2242031	
87.0773281	
86.9304531	
86.7679531	
86.5898281	
86.3929531	
86.2054531	
85.9992031	
85.8054531	
85.6023281	
85.4085781	
85.1773281	
84.9460781	
84.7085781	
84.4804531	
84.2617031	
84.0179531	
83.7898281	
83.5429531	
83.3460781	
83.1804531	
83.0304531	
82.8867031	
82.7617031	
82.6273281	
82.5023281	
82.3617031	
82.1929531	
82.0117031	
81.8335781	
81.6242031	
81.4273281	
81.2460781	
81.0679531	
80.9210781	
80.7773281	
80.6429531	
80.5273281	
80.4054531	
80.3023281	
80.2085781	
80.1085781	
80.0085781	

79.9117031	
79.8210781	
79.7179531	
79.6179531	
79.5273281	
79.4273281	
79.3429531	
79.2617031	
79.2273281	
79.1898281	
79.1648281	
79.1273281	
79.1242031	
79.1492031	
79.1742031	
79.1929531	
79.2054531	
79.2335781	
79.2679531	
79.3085781	
79.3492031	
79.3960781	
79.4398281	
79.4648281	
79.4960781	
79.5273281	
79.5585781	
79.5929531	
79.6117031	
79.6429531	
79.6773281	
79.6992031	
79.7085781	
79.7023281	
79.6929531	
79.6867031	
79.6554531	
79.6148281	
79.5742031	
79.5460781	
79.5273281	
79.5023281	
79.5117031	
79.5210781	
79.5367031	

79.5273281	
79.5023281	
79.4742031	
79.4429531	
79.4148281	
79.3835781	
79.3710781	
79.3117031	
79.2523281	
79.1898281	
79.1335781	
79.0773281	
78.9960781	
78.9117031	
78.8492031	
78.7929531	
78.7273281	
78.6679531	
78.6242031	
78.5804531	
78.5242031	
78.4773281	
78.4335781	
78.3898281	
78.3367031	
78.2929531	
78.2742031	
78.2679531	
78.2523281	
78.2398281	
78.2367031	
78.2617031	
78.2929531	
78.3398281	
78.3773281	
78.4335781	
78.4804531	
78.5148281	
78.5585781	
78.5992031	
78.6648281	
78.7523281	
78.8523281	
78.9648281	
79.0804531	

79.2023281	
79.3117031	
79.4117031	
79.5117031	
79.6023281	
79.6835781	
79.7679531	
79.8429531	
79.9242031	
79.9929531	
80.0460781	
80.0992031	
80.1429531	
80.1929531	
80.2398281	
80.3117031	
80.3835781	
80.4679531	
80.5554531	
80.6367031	
80.6992031	
80.7460781	
80.7835781	
80.8242031	
80.8835781	
80.9367031	
80.9898281	
81.0304531	
81.0523281	
81.0773281	
81.0773281	
81.0898281	
81.0992031	
81.0960781	
81.0742031	
81.0429531	
80.9960781	
80.9335781	
80.8710781	
80.7992031	
80.7085781	
80.6242031	
80.5617031	
80.4992031	
80.4835781	

80.4835781	
80.4773281	
80.4929531	
80.4773281	
80.4679531	
80.4523281	
80.4273281	
80.3992031	
80.3617031	
80.3148281	
80.2367031	
80.1460781	
80.0335781	
79.9210781	
79.8117031	
79.7023281	
79.5929531	
79.4960781	
79.4210781	
79.3210781	
79.2304531	
79.1335781	
79.0554531	
78.9835781	
78.9148281	
78.8679531	
78.8179531	
78.7710781	
78.7117031	
78.6585781	
78.5960781	
78.5304531	
78.4460781	
78.3460781	
78.2523281	
78.1617031	
78.0835781	
78.0054531	
77.9367031	
77.8335781	
77.7179531	
77.6023281	
77.4867031	
77.4054531	
77.3304531	

77.2585781	
77.1835781	
77.1210781	
77.0617031	
77.0179531	
77.0054531	
77.0085781	
77.0179531	
77.0367031	
77.0492031	
77.0585781	
77.0804531	
77.0898281	
77.0960781	
77.1054531	
77.1210781	
77.1335781	
77.1398281	
77.1554531	
77.1742031	
77.1679531	
77.1523281	
77.1273281	
77.1210781	
77.1210781	
77.1585781	
77.2085781	
77.2804531	
77.3648281	
77.4492031	
77.5398281	
77.6210781	
77.6929531	
77.7492031	
77.7960781	
77.8492031	
77.9054531	
77.9648281	
78.0148281	
78.0554531	
78.1085781	
78.1804531	
78.2460781	
78.3179531	
78.4054531	

78.4898281	
78.5679531	
78.6492031	
78.7179531	
78.7929531	
78.8835781	
78.9804531	
79.0867031	
79.1804531	
79.2648281	
79.3242031	
79.3710781	
79.4117031	
79.4429531	
79.4648281	
79.5054531	
79.5585781	
79.6335781	
79.7023281	
79.7648281	
79.7835781	
79.7929531	
79.7960781	
79.7835781	
79.7804531	
79.7742031	
79.7679531	
79.7710781	
79.7710781	
79.7804531	
79.7804531	
79.7929531	
79.8085781	
79.8242031	
79.8460781	
79.8585781	
79.8742031	
79.8960781	
79.9054531	
79.9054531	
79.8898281	
79.8773281	
79.8429531	
79.7992031	
79.7523281	

79.6960781	
79.6117031	
79.5242031	
79.4460781	
79.3992031	
79.3773281	
79.3773281	
79.3835781	
79.3835781	
79.3898281	
79.3992031	
79.4054531	
79.3960781	
79.3835781	
79.3742031	
79.3742031	
79.3679531	
79.3617031	
79.3804531	
79.3773281	
79.3710781	
79.3492031	
79.3179531	
79.3085781	
79.2929531	
79.2898281	
79.2835781	
79.2679531	
79.2460781	
79.2273281	
79.2054531	
79.1835781	
79.1554531	
79.1617031	
79.1773281	
79.2523281	
79.2992031	
79.3648281	
79.4273281	
79.4929531	
79.5617031	
79.6585781	
79.7585781	
79.8585781	
79.9304531	

79.9929531	
80.0460781	
80.0929531	
80.1367031	
80.1742031	
80.1773281	
80.1710781	
80.1585781	
80.1460781	
80.1367031	
80.1179531	
80.1085781	
80.1117031	
80.1085781	
80.1304531	
80.1710781	
80.2242031	
80.2773281	
80.3054531	
80.3335781	
80.3617031	
80.3773281	
80.3929531	
80.4148281	
80.4492031	
80.4960781	
80.5304531	
80.5554531	
80.5429531	
80.5117031	
80.4242031	
80.3585781	
80.2992031	
80.2523281	
80.2023281	
80.1585781	
80.1210781	
80.0804531	
80.0148281	
79.9367031	
79.8554531	
79.7804531	
79.6898281	
79.5992031	
79.5085781	

79.4398281	
79.3835781	
79.3304531	
79.2835781	
79.2398281	
79.2210781	
79.2273281	
79.2367031	
79.2367031	
79.2398281	
79.2367031	
79.2492031	
79.2742031	
79.3179531	
79.3835781	
79.4679531	
79.5523281	
79.6304531	
79.7054531	
79.7648281	
79.8085781	
79.8523281	
79.8960781	
79.9335781	
79.9679531	
80.0054531	
80.0554531	
80.0992031	
80.1367031	
80.1617031	
80.1710781	
80.1804531	
80.1960781	
80.2273281	
80.2742031	
80.3148281	
80.3460781	
80.3804531	
80.4085781	
80.4242031	
80.4367031	
80.4367031	
80.4179531	
80.3929531	
80.3648281	

80.3273281	
80.2742031	
80.2054531	
80.1398281	
80.0492031	
79.9429531	
79.8273281	
79.7179531	
79.6210781	
79.5054531	
79.3960781	
79.2960781	
79.2148281	
79.1398281	
79.0648281	
78.9929531	
78.9304531	
78.8773281	
78.8335781	
78.7960781	
78.7523281	
78.7054531	
78.6585781	
78.6273281	
78.6054531	
78.5960781	
78.5710781	
78.5367031	
78.5273281	
78.5242031	
78.5242031	
78.5304531	
78.5710781	
78.6273281	
78.6992031	
78.7742031	
78.8367031	
78.9085781	
78.9773281	
79.0523281	
79.1398281	
79.2304531	
79.3273281	
79.4148281	
79.5117031	

79.6210781	
79.7085781	
79.7742031	
79.8242031	
79.8710781	
79.9054531	
79.9398281	
79.9617031	
79.9710781	
79.9773281	
79.9804531	
79.9648281	
79.9492031	
79.9460781	
79.9398281	
79.9398281	
79.9398281	
79.9429531	
79.9367031	
79.9367031	
79.9085781	
79.8648281	
79.8179531	
79.7554531	
79.6960781	
79.6273281	
79.5617031	
79.4679531	
79.3773281	
79.2835781	
79.1992031	
79.1335781	
79.1054531	
79.0804531	
79.0492031	
78.9992031	
78.9429531	
78.8617031	
78.7898281	
78.7273281	
78.6679531	
78.6398281	
78.6367031	
78.6179531	
78.6023281	

78.5679531	
78.5304531	
78.4992031	
78.4867031	
78.4804531	
78.4773281	
78.4898281	
78.5054531	
78.5335781	
78.5742031	
78.6179531	
78.6554531	
78.7085781	
78.7648281	
78.8273281	
78.9242031	
79.0304531	
79.1398281	
79.2367031	
79.3304531	
79.4398281	
79.5085781	
79.5804531	
79.6429531	
79.6960781	
79.7429531	
79.8023281	
79.8210781	
79.8523281	
79.8335781	
79.8367031	
79.8304531	
79.8585781	
79.8617031	
79.8523281	
79.8367031	
79.8085781	
79.7710781	
79.7367031	
79.6929531	
79.6648281	
79.6367031	
79.6210781	
79.6023281	
79.5804531	

79.5867031	
79.5992031	
79.6054531	
79.5992031	
79.6117031	
79.6304531	
79.6492031	
79.6648281	
79.6835781	
79.6960781	
79.6898281	
79.6773281	
79.6617031	
79.6554531	
79.6398281	
79.6117031	
79.6335781	
79.6648281	
79.7023281	
79.7460781	
79.7898281	
79.8179531	
79.8585781	
79.8898281	
79.9679531	
80.0335781	
80.1085781	
80.1679531	
80.2460781	
80.2992031	
80.3242031	
80.3367031	
80.3367031	
80.3273281	
80.3117031	
80.2898281	
80.2492031	
80.1929531	
80.1429531	
80.0992031	
80.0367031	
79.9679531	
79.8992031	
79.8304531	
79.7335781	

79.6304531	
79.5335781	
79.4085781	
79.2679531	
79.1273281	
78.9929531	
78.8585781	
78.7179531	
78.5742031	
78.4492031	
78.3460781	
78.2304531	
78.1148281	
78.0054531	
77.9054531	
77.7992031	
77.7148281	
77.6554531	
77.6054531	
77.5585781	
77.5085781	
77.4648281	
77.4367031	
77.4148281	
77.4273281	
77.4679531	
77.5117031	
77.5492031	
77.6023281	
77.6523281	
77.6867031	
77.7335781	
77.7773281	
77.8085781	
77.8273281	
77.8367031	
77.8429531	
77.8554531	
77.8804531	
77.9210781	
77.9648281	
78.0023281	
78.0554531	
78.1148281	
78.1835781	

78.2710781	
78.3648281	
78.4679531	
78.5679531	
78.6523281	
78.7335781	
78.8304531	
78.9367031	
79.0460781	
79.1523281	
79.2679531	
79.3710781	
79.4429531	
79.5085781	
79.5648281	
79.6117031	
79.6398281	
79.6554531	
79.6679531	
79.6773281	
79.6960781	
79.7210781	
79.7523281	
79.7742031	
79.8023281	
79.8242031	
79.8554531	
79.8835781	
79.9023281	
79.9179531	
79.9367031	
79.9710781	
80.0054531	
80.0273281	
80.0367031	
80.0273281	
80.0054531	
79.9585781	
79.9085781	
79.8335781	
79.7367031	
79.6179531	
79.4929531	
79.3773281	
79.2679531	

79.1554531	
79.0210781	
78.8710781	
78.7023281	
78.5585781	
78.4273281	
78.3148281	
78.2054531	
78.0867031	
78.0054531	
77.9429531	
77.8773281	
77.8117031	
77.7492031	
77.6804531	
77.6085781	
77.5367031	
77.4742031	
77.4148281	
77.3710781	
77.3304531	
77.2898281	
77.2523281	
77.2117031	
77.1679531	
77.1304531	
77.0835781	
77.0429531	
77.0054531	
76.9679531	
76.9429531	
76.9242031	
76.8992031	
76.8648281	
76.8429531	
76.8179531	
76.8242031	
76.8367031	
76.8460781	
76.8460781	
76.8492031	
76.8554531	
76.8679531	
76.8960781	
76.8960781	

76.8742031	
76.8460781	
76.8210781	
76.8023281	
76.7585781	
76.6992031	
76.6523281	
76.6085781	
76.5648281	
76.5335781	
76.5054531	
76.4648281	
76.4367031	
76.4242031	
76.3835781	
76.3554531	
76.3117031	
76.2679531	
76.2398281	
76.2242031	
76.2304531	
76.2492031	
76.2617031	
76.2804531	
76.2960781	
76.3117031	
76.3304531	
76.3773281	
76.4304531	
76.4804531	
76.5273281	
76.5585781	
76.5835781	
76.6054531	
76.6117031	
76.5992031	
76.5835781	
76.5710781	
76.5054531	
76.4367031	
76.3898281	
76.3273281	
76.2960781	
76.2773281	
76.2367031	

76.2023281	
76.1679531	
76.1273281	
76.0835781	
76.0367031	
75.9835781	
75.9429531	
75.8898281	
75.8367031	
75.8054531	
75.7867031	
75.7929531	
75.7960781	
75.8023281	
75.7960781	
75.7617031	
75.7117031	
75.6710781	
75.6085781	
75.5335781	
75.4492031	
75.3679531	
75.2835781	
75.1992031	
75.1242031	
75.0648281	
75.0148281	
74.9679531	
74.9304531	
74.9085781	
74.8929531	
74.8773281	
74.8492031	
74.8710781	
74.8960781	
74.9148281	
74.9398281	
74.9242031	
74.8867031	
74.8710781	
74.8679531	
74.8648281	
74.8585781	
74.8492031	
74.8367031	

74.8148281	
74.7835781	
74.7492031	
74.7367031	
74.7242031	
74.7148281	
74.6835781	
74.6648281	
74.6398281	
74.6242031	
74.6304531	
74.6335781	
74.6304531	
74.6367031	
74.6554531	
74.7054531	
74.7554531	
74.8023281	
74.8492031	
74.8960781	
74.9460781	
74.9960781	
75.0460781	
75.0898281	
75.1398281	
75.1992031	
75.2679531	
75.3179531	
75.3523281	
75.3773281	
75.3804531	
75.3960781	
75.4367031	
75.4835781	
75.5398281	
75.5835781	
75.6148281	
75.6554531	
75.6867031	
75.7210781	
75.7679531	
75.8367031	
75.9179531	
75.9710781	
76.0367031	

76.0804531	
76.1273281	
76.1648281	
76.1929531	
76.2054531	
76.1929531	
76.1835781	
76.1648281	
76.1523281	
76.1335781	
76.0960781	
76.0710781	
76.0523281	
76.0460781	
76.0398281	
76.0304531	
76.0273281	
76.0335781	
76.0460781	
76.0523281	
76.0585781	
76.0492031	
76.0710781	
76.0960781	
76.1335781	
76.1710781	
76.1960781	
76.2523281	
76.3117031	
76.3585781	
76.4085781	
76.4679531	
76.5335781	
76.6210781	
76.7085781	
76.8179531	
76.9117031	
76.9773281	
77.0273281	
77.0648281	
77.1117031	
77.1585781	
77.2054531	
77.2585781	
77.3179531	

77.3742031	
77.4367031	
77.5085781	
77.5710781	
77.6367031	
77.6960781	
77.7367031	
77.7679531	
77.8085781	
77.8429531	
77.8523281	
77.8554531	
77.8554531	
77.8429531	
77.8335781	
77.8179531	
77.8085781	
77.7960781	
77.7898281	
77.7710781	
77.7554531	
77.7367031	
77.6710781	
77.6085781	
77.5429531	
77.4710781	
77.3960781	
77.3210781	
77.2335781	
77.1460781	
77.0335781	
76.9273281	
76.8398281	
76.7773281	
76.7117031	
76.6429531	
76.5867031	
76.5398281	
76.4992031	
76.4773281	
76.4742031	
76.4867031	
76.5117031	
76.5429531	
76.5960781	

76.6429531	
76.6742031	
76.7367031	
76.7648281	
76.7804531	
76.8179531	
76.8460781	
76.8648281	
76.8867031	
76.9054531	
76.9210781	
76.9460781	
76.9585781	
76.9710781	
76.9929531	
77.0273281	
77.0804531	
77.1398281	
77.1835781	
77.2210781	
77.2710781	
77.3273281	
77.3648281	
77.3960781	
77.4148281	
77.4304531	
77.4335781	
77.4367031	
77.4304531	
77.4242031	
77.4273281	
77.4242031	
77.4085781	
77.3992031	
77.3960781	
77.3742031	
77.3304531	
77.2773281	
77.2304531	
77.1835781	
77.1679531	
77.1742031	
77.1648281	
77.1679531	
77.1742031	

77.1648281	
77.1742031	
77.2023281	
77.2398281	
77.2773281	
77.3210781	
77.3335781	
77.3554531	
77.3679531	
77.3804531	
77.3960781	
77.3898281	
77.3742031	
77.3773281	
77.3898281	
77.3929531	
77.3992031	
77.4054531	
77.4148281	
77.4367031	
77.4492031	
77.4648281	
77.4804531	
77.4960781	
77.5273281	
77.5585781	
77.5898281	
77.6117031	
77.6117031	
77.5992031	
77.5804531	
77.5679531	
77.5585781	
77.5523281	
77.5367031	
77.5023281	
77.4867031	
77.4773281	
77.4679531	
77.4585781	
77.4304531	
77.3835781	
77.3117031	
77.2242031	
77.1460781	

77.0460781	
76.9585781	
76.8648281	
76.7804531	
76.7367031	
76.6929531	
76.6492031	
76.6054531	
76.5460781	
76.4804531	
76.4054531	
76.3273281	
76.2867031	
76.2554531	
76.2304531	
76.2148281	
76.2054531	
76.1742031	
76.1210781	
76.0648281	
76.0054531	
75.9492031	
75.8992031	
75.8554531	
75.8054531	
75.7648281	
75.7179531	
75.6210781	
75.5273281	
75.4273281	
75.3242031	
75.2460781	
75.1742031	
75.1085781	
75.0617031	
75.0773281	
75.1179531	
75.1742031	
75.2492031	
75.3210781	
75.4117031	
75.5273281	
75.6554531	
75.7898281	
75.8804531	

75.9773281	
76.0804531	
76.1804531	
76.2773281	
76.3742031	
76.4679531	
76.5523281	
76.5992031	
76.6460781	
76.6898281	
76.7242031	
76.7679531	
76.8367031	
76.9179531	
76.9742031	
77.0273281	
77.0773281	
77.1367031	
77.2085781	
77.2804531	
77.3492031	
77.4242031	
77.5304531	
77.6242031	
77.7179531	
77.8117031	
77.8867031	
77.9585781	
78.0523281	
78.1367031	
78.1960781	
78.2429531	
78.2960781	
78.2960781	
78.2992031	
78.2867031	
78.2679531	
78.2398281	
78.2023281	
78.1679531	
78.1085781	
78.0304531	
77.9367031	
77.8429531	
77.7554531	

77.6710781	
77.5835781	
77.5023281	
77.4148281	
77.3460781	
77.2773281	
77.1773281	
77.0679531	
76.9492031	
76.8460781	
76.7398281	
76.6210781	
76.4929531	
76.3585781	
76.2273281	
76.1273281	
76.0867031	
76.0960781	
76.1179531	
76.1398281	
76.1648281	
76.1648281	
76.1648281	
76.1554531	
76.1429531	
76.1273281	
76.1210781	
76.1023281	
76.0773281	
76.0429531	
76.0304531	
76.0023281	
75.9773281	
75.9367031	
75.8960781	
75.8492031	
75.8117031	
75.8023281	
75.8179531	
75.8367031	
75.8648281	
75.9054531	
75.9554531	
76.0023281	
76.0273281	

76.0710781	
76.1179531	
76.1742031	
76.2429531	
76.3085781	
76.3835781	
76.4742031	
76.5835781	
76.7054531	
76.8429531	
76.9804531	
77.0460781	
77.0773281	
77.0960781	
77.1335781	
77.1929531	
77.2554531	
77.3117031	
77.3460781	
77.3773281	
77.3992031	
77.4210781	
77.4523281	
77.5242031	
77.5773281	
77.6335781	
77.6710781	
77.6992031	
77.7523281	
77.8023281	
77.8648281	
77.9304531	
77.9898281	
78.0398281	
78.0773281	
78.1210781	
78.1929531	
78.2679531	
78.3460781	
78.4554531	
78.5773281	
78.7179531	
78.8679531	
79.0242031	
79.2023281	

79.3710781	
79.5304531	
79.6710781	
79.7585781	
79.8304531	
79.8898281	
79.9554531	
80.0335781	
80.1210781	
80.1898281	
80.2335781	
80.2398281	
80.2304531	
80.2179531	
80.2085781	
80.1960781	
80.1648281	
80.1242031	
80.0992031	
80.1054531	
80.1148281	
80.1679531	
80.2210781	
80.3085781	
80.4023281	
80.5023281	
80.6179531	
80.7273281	
80.8367031	
80.9492031	
81.0179531	
81.0523281	
81.0773281	
81.1023281	
81.0835781	
81.0492031	
81.0179531	
80.9679531	
80.9367031	
80.8929531	
80.8710781	
80.8679531	
80.8773281	
80.9023281	
80.8992031	

80.8804531	
80.8617031	
80.8054531	
80.7523281	
80.7085781	
80.6648281	
80.6585781	
80.6585781	
80.6679531	
80.6742031	
80.6804531	
80.6867031	
80.6960781	
80.6992031	
80.6867031	
80.6804531	
80.6460781	
80.6117031	
80.5117031	
80.4023281	
80.2835781	
80.1492031	
80.0179531	
79.8742031	
79.7304531	
79.6335781	
79.5335781	
79.4398281	
79.3492031	
79.2710781	
79.1867031	
79.0992031	
79.0179531	
78.9210781	
78.8304531	
78.7179531	
78.5898281	
78.4679531	
78.3929531	
78.3242031	
78.2648281	
78.1898281	
78.1304531	
78.0648281	
78.0117031	

77.9742031	
77.9460781	
77.9367031	
77.9210781	
77.9085781	
77.8992031	
77.8960781	
77.8742031	
77.8460781	
77.8179531	
77.7773281	
77.7554531	
77.7429531	
77.7617031	
77.7867031	
77.8242031	
77.8648281	
77.9054531	
77.9429531	
77.9867031	
78.0148281	
78.0492031	
78.0773281	
78.0992031	
78.1085781	
78.1492031	
78.1929531	
78.2429531	
78.2992031	
78.3585781	
78.4460781	
78.5742031	
78.6992031	
78.8117031	
78.9273281	
79.0398281	
79.1648281	
79.2898281	
79.4085781	
79.5054531	
79.5710781	
79.6242031	
79.6492031	
79.6929531	
79.7585781	

79.8304531	
79.9054531	
79.9992031	
80.1023281	
80.2179531	
80.3429531	
80.4617031	
80.5710781	
80.6742031	
80.7742031	
80.8554531	
80.9367031	
81.0085781	
81.0867031	
81.1585781	
81.2148281	
81.2367031	
81.2273281	
81.1992031	
81.1773281	
81.1335781	
81.0867031	
81.0335781	
80.9742031	
80.8929531	
80.7867031	
80.6554531	
80.5054531	
80.3523281	
80.2054531	
80.0367031	
79.8710781	
79.7054531	
79.5429531	
79.3742031	
79.2085781	
79.0367031	
78.8835781	
78.7335781	
78.5773281	
78.4398281	
78.3273281	
78.2179531	
78.1085781	
77.9992031	

77.8929531	
77.7835781	
77.6929531	
77.6117031	
77.5398281	
77.4804531	
77.4460781	
77.4148281	
77.3648281	
77.3085781	
77.2648281	
77.2554531	
77.2835781	
77.3367031	
77.3898281	
77.4398281	
77.4929531	
77.5492031	
77.5898281	
77.6242031	
77.6804531	
77.7367031	
77.8117031	
77.8710781	
77.9242031	
78.0023281	
78.0804531	
78.1492031	
78.1898281	
78.2117031	
78.2460781	
78.2804531	
78.2773281	
78.2585781	
78.2273281	
78.1992031	
78.1398281	
78.0835781	
78.0398281	
77.9710781	
77.8804531	
77.7929531	
77.6898281	
77.5867031	
77.4835781	

77.3742031	
77.2210781	
77.0773281	
76.9773281	
76.9023281	
76.8460781	
76.7929531	
76.7304531	
76.6742031	
76.6335781	
76.5929531	
76.5335781	
76.4585781	
76.3523281	
76.2554531	
76.1335781	
75.9867031	
75.8367031	
75.6929531	
75.5679531	
75.4585781	
75.3617031	
75.2742031	
75.2148281	
75.1742031	
75.1367031	
75.0929531	
75.0742031	
75.0554531	
75.0335781	
74.9835781	
74.9492031	
74.9242031	
74.8898281	
74.9054531	
74.9367031	
74.9773281	
75.0148281	
75.0429531	
75.0460781	
75.0335781	
75.0335781	
75.0273281	
74.9992031	
74.9367031	

74.8648281	
74.7992031	
74.7523281	
74.7148281	
74.6835781	
74.6617031	
74.6617031	
74.6648281	
74.7304531	
74.8304531	
74.9710781	
75.1335781	
75.3179531	
75.5148281	
75.7054531	
75.8929531	
76.0710781	
76.2335781	
76.4179531	
76.6054531	
76.7898281	
76.9898281	
77.1992031	
77.4179531	
77.6460781	
77.8898281	
78.0273281	
78.1023281	
78.1335781	
78.1179531	
78.0898281	
78.0742031	
78.0710781	
78.0585781	
78.0617031	
78.0710781	
78.0679531	
78.0679531	
78.0679531	
78.0898281	
78.1054531	
78.1085781	
78.0960781	
78.0617031	
78.0148281	

77.9554531	
77.9117031	
77.8835781	
77.8398281	
77.7710781	
77.6617031	
77.5335781	
77.3773281	
77.2179531	
77.0710781	
76.9085781	
76.7492031	
76.6304531	
76.4929531	
76.3648281	
76.2554531	
76.1554531	
76.0554531	
75.9585781	
75.8617031	
75.7523281	
75.7242031	
75.6867031	
75.6804531	
75.6835781	
75.6929531	
75.6960781	
75.6992031	
75.7210781	
75.7273281	
75.7554531	
75.8148281	
75.8617031	
75.9054531	
75.9460781	
75.9835781	
76.0429531	
76.1023281	
76.1773281	
76.2523281	
76.3273281	
76.3679531	
76.3929531	
76.3867031	
76.3492031	

76.3054531	
76.2617031	
76.2210781	
76.1742031	
76.1148281	
76.0585781	
75.9867031	
75.8867031	
75.7835781	
75.6679531	
75.5335781	
75.3867031	
75.2273281	
75.0679531	
74.9023281	
74.7398281	
74.5867031	
74.5929531	
74.6117031	
74.6148281	
74.5273281	
74.3992031	
74.2523281	
74.0898281	
73.9460781	
73.8023281	
73.6523281	
73.5304531	
73.4335781	
73.3429531	
73.2679531	
73.1867031	
73.1179531	
73.0492031	
72.9929531	
72.9398281	
72.9054531	
72.8835781	
72.8960781	
72.9429531	
72.9835781	
73.0367031	
73.0554531	
73.0710781	
73.0898281	

73.1117031	
73.1367031	
73.1679531	
73.1992031	
73.2210781	
73.2429531	
73.2648281	
73.2929531	
73.3085781	
73.3148281	
73.3179531	
73.2992031	
73.1710781	
73.0429531	
72.9304531	
72.9117031	
72.9273281	
72.9554531	
72.9867031	
73.0210781	
73.0335781	
73.0429531	
73.0335781	
73.0085781	
72.9742031	
72.9367031	
72.8929531	
72.8398281	
72.7710781	
72.6992031	
72.6367031	
72.5804531	
72.5210781	
72.4585781	
72.3867031	
72.3242031	
72.2554531	
72.2148281	
72.1710781	
72.1367031	
72.1179531	
72.1273281	
72.1398281	
72.1304531	
72.1148281	

72.0804531	
72.0804531	
72.0960781	
72.1148281	
72.1429531	
72.1804531	
72.2492031	
72.3148281	
72.3648281	
72.4148281	
72.4585781	
72.4992031	
72.5398281	
72.5804531	
72.6242031	
72.6804531	
72.7179531	
72.7617031	
72.8054531	
72.8648281	
72.9273281	
72.9929531	
73.0710781	
73.1585781	
73.2492031	
73.3210781	
73.3679531	
73.4210781	
73.4617031	
73.5023281	
73.5585781	
73.6179531	
73.6867031	
73.7710781	
73.8460781	
73.9054531	
73.9648281	
74.0367031	
74.1460781	
74.2492031	
74.3742031	
74.4679531	
74.5492031	
74.6304531	
74.7210781	

74.8117031	
74.8960781	
74.9929531	
75.1273281	
75.2804531	
75.4398281	
75.5898281	
75.7585781	
75.9367031	
76.1117031	
76.2773281	
76.4585781	
76.6492031	
76.8335781	
77.0242031	
77.2117031	
77.3835781	
77.5304531	
77.6773281	
77.7929531	
77.9117031	
78.0085781	
78.0898281	
78.1804531	
78.2773281	
78.3648281	
78.4617031	
78.5554531	
78.5804531	
78.5835781	
78.5648281	
78.5210781	
78.4617031	
78.3898281	
78.3460781	
78.3023281	
78.2304531	
78.1367031	
78.0242031	
77.9054531	
77.8023281	
77.7054531	
77.6085781	
77.4867031	
77.3585781	

77.2398281	
77.1367031	
77.0179531	
76.8804531	
76.7273281	
76.5804531	
76.4273281	
76.2585781	
76.0867031	
75.8867031	
75.6585781	
75.4398281	
75.2304531	
75.0179531	
74.8367031	
74.6679531	
74.5335781	
74.4085781	
74.2960781	
74.2023281	
74.0960781	
73.9773281	
73.8523281	
73.7898281	
73.7523281	
73.7304531	
73.7179531	
73.7023281	
73.6742031	
73.6460781	
73.6304531	
73.6398281	
73.6929531	
73.7742031	
73.8617031	
73.9179531	
73.9523281	
73.9835781	
74.0148281	
74.0429531	
74.0523281	
74.0648281	
74.0742031	
74.0992031	
74.1304531	

74.1617031	
74.1960781	
74.2210781	
74.2179531	
74.2148281	
74.2492031	
74.2992031	
74.3523281	
74.4242031	
74.4960781	
74.5679531	
74.6492031	
74.7273281	
74.7867031	
74.8179531	
74.8679531	
74.9273281	
75.0085781	
75.1023281	
75.2148281	
75.3179531	
75.4054531	
75.4960781	
75.6117031	
75.7242031	
75.8179531	
75.9054531	
75.9648281	
76.0148281	
76.0554531	
76.1429531	
76.2617031	
76.3835781	
76.5023281	
76.6242031	
76.7804531	
76.9492031	
77.1335781	
77.3210781	
77.5273281	
77.7429531	
77.9460781	
78.1460781	
78.3742031	
78.6023281	

78.8273281	
79.0304531	
79.2179531	
79.3648281	
79.4898281	
79.5992031	
79.6960781	
79.7804531	
79.8617031	
79.9367031	
80.0023281	
80.0648281	
80.0992031	
80.1085781	
80.0867031	
80.0679531	
80.0585781	
80.0242031	
79.9554531	
79.8804531	
79.8148281	
79.7585781	
79.7085781	
79.6523281	
79.5960781	
79.4960781	
79.3804531	
79.2648281	
79.1460781	
79.0054531	
78.8273281	
78.6242031	
78.4242031	
78.2335781	
78.0429531	
77.8523281	
77.6742031	
77.5304531	
77.4148281	
77.2992031	
77.1804531	
77.0710781	
76.9804531	
76.9273281	
76.9054531	

76.8929531	
76.8960781	
76.9242031	
76.9617031	
77.0085781	
77.0523281	
77.0867031	
77.1273281	
77.1804531	
77.2648281	
77.3148281	
77.3960781	
77.4679531	
77.5492031	
77.6304531	
77.6804531	
77.6992031	
77.7085781	
77.7085781	
77.7210781	
77.7398281	
77.7304531	
77.7148281	
77.7054531	
77.7023281	
77.7085781	
77.7085781	
77.6960781	
77.6742031	
77.6492031	
77.6148281	
77.5773281	
77.5210781	
77.4242031	
77.3367031	
77.2335781	
77.1085781	
76.9742031	
76.8273281	
76.6523281	
76.4710781	
76.2835781	
76.0960781	
75.8992031	
75.6992031	

75.5117031	
75.3273281	
75.1492031	
74.9835781	
74.7929531	
74.6335781	
74.4398281	
74.2960781	
74.1710781	
74.0492031	
73.9429531	
73.8773281	
73.8179531	
73.7710781	
73.7117031	
73.6523281	
73.6148281	
73.5867031	
73.5554531	
73.5335781	
73.5242031	
73.5335781	
73.5492031	
73.5617031	
73.5585781	
73.5460781	
73.5335781	
73.5179531	
73.5210781	
73.5273281	
73.5429531	
73.5835781	
73.6054531	
73.6242031	
73.6460781	
73.6617031	
73.6742031	
73.6679531	
73.6742031	
73.6867031	
73.6960781	
73.6960781	
73.6804531	
73.6367031	
73.6117031	

73.5835781	
73.5492031	
73.4929531	
73.4273281	
73.3617031	
73.3054531	
73.2367031	
73.1710781	
73.1210781	
73.0804531	
73.0398281	
73.0054531	
72.9617031	
72.9242031	
72.9023281	
72.8804531	
72.8554531	
72.8304531	
72.8054531	
72.7804531	
72.7742031	
72.7585781	
72.7648281	
72.7710781	
72.7960781	
72.8335781	
72.8804531	
72.9648281	
73.0554531	
73.1398281	
73.2304531	
73.3179531	
73.4148281	
73.5117031	
73.5992031	
73.6835781	
73.7710781	
73.8679531	
73.9679531	
74.0429531	
74.1273281	
74.2054531	
74.2773281	
74.3460781	
74.4023281	

74.4585781	
74.5117031	
74.5679531	
74.6085781	
74.6398281	
74.6773281	
74.7242031	
74.7835781	
74.8429531	
74.9023281	
74.9710781	
75.0398281	
75.0992031	
75.1679531	
75.2335781	
75.3460781	
75.4554531	
75.6210781	
75.7398281	
75.8335781	
75.9179531	
75.9835781	
75.9898281	
75.9804531	
75.9679531	
75.9460781	
75.9273281	
75.9054531	
75.8710781	
75.8429531	
75.8210781	
75.8085781	
75.7742031	
75.7460781	
75.7304531	
75.7054531	
75.7023281	
75.7273281	
75.7523281	
75.8023281	
75.8585781	
75.9398281	
76.0367031	
76.1398281	
76.2398281	

76.3273281	
76.4023281	
76.4554531	
76.4742031	
76.4742031	
76.4492031	
76.4148281	
76.3804531	
76.3679531	
76.3367031	
76.2554531	
76.1992031	
76.0710781	
75.9992031	
75.9460781	
75.8960781	
75.8554531	
75.8835781	
75.9210781	
75.9742031	
76.0398281	
76.1117031	
76.1898281	
76.2804531	
76.3773281	
76.4460781	
76.5117031	
76.5867031	
76.6585781	
76.7429531	
76.8429531	
76.9554531	
77.0554531	
77.1835781	
77.3023281	
77.4210781	
77.5273281	
77.6054531	
77.6648281	
77.7148281	
77.7460781	
77.7679531	
77.7804531	
77.8242031	
77.8742031	

77.9335781	
78.0148281	
78.0992031	
78.1429531	
78.2398281	
78.3492031	
78.4742031	
78.5960781	
78.6898281	
78.7710781	
78.8679531	
78.9648281	
79.0492031	
79.1367031	
79.2304531	
79.3273281	
79.4148281	
79.4742031	
79.5304531	
79.5835781	
79.6617031	
79.7429531	
79.8523281	
79.9679531	
80.0585781	
80.1460781	
80.1773281	
80.2117031	
80.2117031	
80.2148281	
80.2054531	
80.1867031	
80.1742031	
80.1804531	
80.1898281	
80.2273281	
80.2960781	
80.4148281	
80.5460781	
80.6773281	
80.7960781	
80.8898281	
80.9679531	
81.0398281	
81.1117031	

81.1867031	
81.2648281	
81.3492031	
81.4492031	
81.5554531	
81.6398281	
81.7242031	
81.8085781	
81.8429531	
81.8367031	
81.8242031	
81.8054531	
81.8117031	
81.7898281	
81.7679531	
81.7367031	
81.6992031	
81.6398281	
81.5773281	
81.5460781	
81.5148281	
81.5085781	
81.4992031	
81.4867031	
81.4273281	
81.3742031	
81.2710781	
81.1523281	
81.0304531	
80.9210781	
80.8023281	
80.6617031	
80.4960781	
80.3148281	
80.1085781	
79.8960781	
79.6867031	
79.5117031	
79.3617031	
79.1960781	
79.0242031	
78.8429531	
78.6773281	
78.5179531	
78.3554531	

78.1742031	
77.9773281	
77.7554531	
77.5648281	
77.3960781	
77.2398281	
77.0835781	
76.9273281	
76.8085781	
76.6898281	
76.5835781	
76.4867031	
76.4023281	
76.3179531	
76.2179531	
76.1242031	
75.9992031	
75.8742031	
75.7648281	
75.6929531	
75.6148281	
75.6148281	
75.6398281	
75.6679531	
75.6929531	
75.7398281	
75.7898281	
75.8273281	
75.8742031	
75.9429531	
76.0148281	
76.0742031	
76.1054531	
76.1335781	
76.1523281	
76.1835781	
76.1960781	
76.1804531	
76.1554531	
76.1273281	
76.1117031	
76.0960781	
76.0742031	
76.0648281	
76.0585781	

76.0398281	
76.0273281	
76.0210781	
76.0179531	
76.0054531	
75.9773281	
75.9398281	
75.8992031	
75.8554531	
75.7992031	
75.7304531	
75.7054531	
75.6742031	
75.6273281	
75.6054531	
75.6148281	
75.5867031	
75.5554531	
75.5242031	
75.4929531	
75.4492031	
75.4023281	
75.3742031	
75.3335781	
75.2992031	
75.2773281	
75.2679531	
75.2742031	
75.2710781	
75.2929531	
75.2867031	
75.2804531	
75.2898281	
75.2992031	
75.2992031	
75.3085781	
75.3242031	
75.3648281	
75.4179531	
75.4804531	
75.5429531	
75.6148281	
75.6867031	
75.7460781	
75.8242031	

75.8960781	
75.9554531	
76.0273281	
76.0867031	
76.1523281	
76.2117031	
76.2742031	
76.3148281	
76.3554531	
76.3867031	
76.3929531	
76.4023281	
76.4085781	
76.4085781	
76.4085781	
76.4023281	
76.3929531	
76.3648281	
76.3460781	
76.3242031	
76.3148281	
76.3148281	
76.3117031	
76.3179531	
76.2929531	
76.2679531	
76.2367031	
76.1929531	
76.1617031	
76.1367031	
76.1085781	
76.0773281	
76.0335781	
75.9710781	
75.8929531	
75.8179531	
75.7148281	
75.6117031	
75.5085781	
75.4054531	
75.3242031	
75.2492031	
75.1585781	
75.0804531	
74.9898281	

74.9179531	
74.8054531	
74.7117031	
74.6242031	
74.5367031	
74.4742031	
74.4242031	
74.3710781	
74.3210781	
74.2992031	
74.2835781	
74.2898281	
74.3054531	
74.3085781	
74.3148281	
74.2773281	
74.2242031	
74.1617031	
74.0960781	
74.0398281	
73.9929531	
73.9554531	
73.9210781	
73.8835781	
73.8554531	
73.8335781	
73.8304531	
73.8523281	
73.8992031	
73.9773281	
74.0523281	
74.1460781	
74.2429531	
74.3273281	
74.3617031	
74.3835781	
74.4148281	
74.4492031	
74.4804531	
74.5148281	
74.5367031	
74.5898281	
74.6492031	
74.7210781	
74.7960781	

74.8460781	
74.8992031	
74.9460781	
74.9554531	
74.9085781	
74.8523281	
74.7773281	
74.6867031	
74.6054531	
74.5117031	
74.4429531	
74.3804531	
74.3148281	
74.2492031	
74.1867031	
74.1273281	
74.0679531	
74.0117031	
73.9429531	
73.8617031	
73.7617031	
73.6398281	
73.5054531	
73.3585781	
73.2179531	
73.0898281	
72.9648281	
72.8242031	
72.6929531	
72.5773281	
72.4585781	
72.3460781	
72.2304531	
72.1304531	
72.0335781	
71.9398281	
71.8492031	
71.7742031	
71.6960781	
71.6335781	
71.5773281	
71.5179531	
71.4710781	
71.5023281	
71.5992031	

71.7273281	
71.8617031	
72.0179531	
72.1929531	
72.3898281	
72.5898281	
72.7960781	
73.0148281	
73.2148281	
73.4210781	
73.6179531	
73.8117031	
74.0117031	
74.2273281	
74.4398281	
74.6617031	
74.8898281	
75.1117031	
75.3335781	
75.5335781	
75.7210781	
75.8992031	
76.0804531	
76.2523281	
76.4398281	
76.6367031	
76.8304531	
77.0242031	
77.2304531	
77.4585781	
77.6867031	
77.9242031	
78.1367031	
78.3460781	
78.5117031	
78.6710781	
78.8054531	
78.9148281	
78.9929531	
79.0429531	
79.0804531	
79.1210781	
79.1617031	
79.1835781	
79.1835781	

79.1804531	
79.1648281	
79.1335781	
79.1273281	
79.1054531	
79.0898281	
79.0835781	
79.0742031	
79.0617031	
79.0617031	
79.0710781	
79.0960781	
79.1273281	
79.1617031	
79.1835781	
79.2242031	
79.2617031	
79.3117031	
79.3835781	
79.4960781	
79.6117031	
79.7367031	
79.8585781	
79.9523281	
80.0148281	
80.0710781	
80.1085781	
80.1460781	
80.1742031	
80.2210781	
80.2679531	
80.3492031	
80.4585781	
80.5742031	
80.6804531	
80.7710781	
80.8648281	
80.9554531	
81.0523281	
81.1523281	
81.2617031	
81.3804531	
81.4960781	
81.5992031	
81.6835781	

81.6929531	
81.6710781	
81.6335781	
81.5585781	
81.4648281	
81.3585781	
81.2335781	
81.1023281	
80.9867031	
80.8773281	
80.7492031	
80.6273281	
80.4929531	
80.3210781	
80.0929531	
79.8617031	
79.6148281	
79.3835781	
79.1710781	
78.9742031	
78.7867031	
78.6117031	
78.4398281	
78.2585781	
78.0492031	
77.8304531	
77.5898281	
77.3398281	
77.0523281	
76.7554531	
76.4679531	
76.1679531	
75.8742031	
75.5929531	
75.3273281	
75.0617031	
74.7898281	
74.5304531	
74.2804531	
74.0460781	
73.8835781	
73.7460781	
73.6273281	
73.5398281	
73.4867031	

73.4460781	
73.4210781	
73.4054531	
73.3710781	
73.3304531	
73.2867031	
73.2335781	
73.1960781	
73.1992031	
73.2242031	
73.2460781	
73.2523281	
73.2429531	
73.2117031	
73.1742031	
73.1242031	
73.0710781	
73.0335781	
72.9929531	
72.9835781	
72.9710781	
72.9679531	
72.9679531	
73.0023281	
73.0585781	
73.1210781	
73.2023281	
73.2804531	
73.3398281	
73.3898281	
73.4367031	
73.4898281	
73.5585781	
73.6179531	
73.6835781	
73.7617031	
73.8585781	
73.9460781	
74.0367031	
74.1117031	
74.1867031	
74.2273281	
74.2617031	
74.3023281	
74.3554531	

74.3898281	
74.4242031	
74.4304531	
74.4179531	
74.4054531	
74.3960781	
74.4085781	
74.4273281	
74.4617031	
74.5117031	
74.5742031	
74.6460781	
74.7023281	
74.7835781	
74.8554531	
74.9492031	
75.0429531	
75.1179531	
75.1773281	
75.2117031	
75.2242031	
75.2273281	
75.2335781	
75.2367031	
75.2179531	
75.1898281	
75.1398281	
75.0679531	
75.0054531	
74.9273281	
74.8429531	
74.7492031	
74.6710781	
74.5898281	
74.5023281	
74.4148281	
74.3429531	
74.2585781	
74.1617031	
74.0585781	
73.9804531	
73.9085781	
73.8210781	
73.7367031	
73.6492031	

73.5617031	
73.4679531	
73.3804531	
73.2867031	
73.1929531	
73.1054531	
73.0023281	
72.9054531	
72.7992031	
72.7117031	
72.6085781	
72.5054531	
72.4148281	
72.3242031	
72.2523281	
72.2054531	
72.1492031	
72.1054531	
72.0804531	
72.0992031	
72.1273281	
72.1804531	
72.2304531	
72.2679531	
72.3179531	
72.3460781	
72.3585781	
72.3679531	
72.3835781	
72.4054531	
72.4179531	
72.4367031	
72.4585781	
72.4960781	
72.5523281	
72.6179531	
72.6960781	
72.8023281	
72.9085781	
73.0179531	
73.1273281	
73.2335781	
73.3460781	
73.4710781	
73.5992031	

73.7179531	
73.8460781	
73.9648281	
74.0898281	
74.1960781	
74.3085781	
74.4273281	
74.5398281	
74.6492031	
74.7335781	
74.7929531	
74.8492031	
74.8773281	
74.8898281	
74.8742031	
74.8585781	
74.8335781	
74.8148281	
74.7960781	
74.7710781	
74.7554531	
74.7523281	
74.7335781	
74.7148281	
74.6898281	
74.6710781	
74.6710781	
74.6710781	
74.6585781	
74.6460781	
74.6242031	
74.6179531	
74.6148281	
74.6148281	
74.6179531	
74.6148281	
74.6148281	
74.5835781	
74.5117031	
74.4085781	
74.3085781	
74.2148281	
74.1367031	
74.0804531	
74.0367031	

73.9960781	
73.9023281	
73.7867031	
73.6742031	
73.5710781	
73.4804531	
73.3804531	
73.2992031	
73.2210781	
73.1335781	
73.0398281	
72.9429531	
72.8460781	
72.7554531	
72.6898281	
72.6460781	
72.6085781	
72.5835781	
72.5617031	
72.5429531	
72.5335781	
72.5117031	
72.5148281	
72.5210781	
72.5148281	
72.5460781	
72.6023281	
72.6835781	
72.7835781	
72.8804531	
72.9648281	
73.0554531	
73.1523281	
73.2992031	
73.4648281	
73.6648281	
73.8898281	
74.0992031	
74.2804531	
74.4523281	
74.6210781	
74.8367031	
75.0898281	
75.3179531	
75.5304531	

75.7210781	
75.9210781	
76.1117031	
76.3054531	
76.5210781	
76.7398281	
76.9679531	
77.1867031	
77.3992031	
77.5898281	
77.7585781	
77.9148281	
78.0492031	
78.1617031	
78.2648281	
78.3648281	
78.4585781	
78.5367031	
78.5960781	
78.6523281	
78.6617031	
78.6085781	
78.5304531	
78.4367031	
78.3492031	
78.2804531	
78.2085781	
78.1367031	
78.0335781	
77.9085781	
77.7523281	
77.5679531	
77.3929531	
77.2210781	
77.0585781	
76.9054531	
76.7585781	
76.6085781	
76.5023281	
76.4273281	
76.3804531	
76.3554531	
76.3148281	
76.2679531	
76.1835781	

76.0960781	
76.0054531	
75.9304531	
75.8617031	
75.8085781	
75.7773281	
75.7398281	
75.6960781	
75.6492031	
75.5960781	
75.5335781	
75.4742031	
75.3960781	
75.3335781	
75.2835781	
75.2460781	
75.2054531	
75.1429531	
75.0867031	
75.0117031	
74.9617031	
74.9335781	
74.9335781	
74.9554531	
75.0085781	
75.0554531	
75.0898281	
75.1179531	
75.1148281	
75.0960781	
75.0710781	
75.0367031	
75.0023281	
74.9617031	
74.9210781	
74.8804531	
74.8085781	
74.7273281	
74.6367031	
74.5617031	
74.4929531	
74.4179531	
74.3367031	
74.2429531	
74.1304531	

74.0054531	
73.8929531	
73.8085781	
73.7523281	
73.7117031	
73.6773281	
73.6554531	
73.6523281	
73.6804531	
73.6992031	
73.7304531	
73.7835781	
73.8429531	
73.8804531	
73.9475042	
73.9895553	
73.9990171	
73.9999968	
73.98803	
73.9595454	
73.9520429	
73.950344	
73.9548951	
73.9835533	
74.0398902	
74.1002448	
74.1605995	
74.2106863	
74.2442552	
74.2608599	
74.2752324	
74.2985335	
74.3294239	

time GMT	elapsed	Person 1	Person 6	Person 3	Person 5	Person 8
17:59:31		77.6930524	71.3812876	70.731787	80.2395153	72.8457846
17:59:32		80.6930524	71.3812876	71.731787	80.2395153	72.8457846
17:59:33		81.6930524	72.3812876	71.731787	80.2395153	72.8457846
17:59:34		77.6930524	72.3812876	71.731787	81.2395153	72.8457846
17:59:35		75.6930524	73.3812876	71.731787	82.2395153	74.8457846
17:59:36		74.6930524	76.3812876	71.731787	82.2395153	75.8457846
17:59:37		74.6930524	78.3812876	71.731787	82.2395153	76.8457846
17:59:38		74.6930524	79.3812876	71.731787	82.2395153	77.8457846
17:59:39		74.6930524	79.3812876	72.731787	82.2395153	78.8457846
17:59:40		76.6930524	79.3812876	72.731787	82.2395153	78.8457846
17:59:41		80.6930524	79.3812876	70.731787	82.2395153	78.8457846
17:59:42		82.6930524	80.3812876	70.731787	83.2395153	76.8457846
17:59:43		83.6930524	80.3812876	70.731787	84.2395153	76.8457846
17:59:44		83.6930524	80.3812876	71.731787	84.2395153	77.8457846
17:59:45		85.6930524	80.3812876	73.731787	85.2395153	79.8457846
17:59:46		85.6930524	80.3812876	74.731787	88.2395153	80.8457846
17:59:47		85.6930524	80.3812876	74.731787	93.2395153	81.8457846
17:59:48		79.6930524	80.3812876	74.731787	95.2395153	82.8457846
17:59:49		79.6930524	80.3812876	74.731787	95.2395153	83.8457846
17:59:50		79.6930524	80.3812876	74.731787	96.2395153	83.8457846
17:59:51		80.6930524	79.3812876	74.731787	96.2395153	81.8457846
17:59:52		80.6930524	79.3812876	75.731787	95.2395153	77.8457846
17:59:53		80.6930524	79.3812876	74.731787	93.2395153	77.8457846
17:59:54		79.6930524	79.3812876	74.731787	93.2395153	75.8457846
17:59:55		78.6930524	79.3812876	74.731787	89.2395153	75.8457846
17:59:56		77.6930524	79.3812876	74.731787	88.2395153	75.8457846
17:59:57		76.6930524	79.3812876	74.731787	87.2395153	74.8457846
17:59:58		76.6930524	79.3812876	75.731787	87.2395153	74.8457846
17:59:59		76.6930524	79.3812876	75.731787	87.2395153	73.8457846
18:00:00		76.6930524	79.3812876	75.731787	87.2395153	73.8457846
18:00:01		75.6930524	78.3812876	77.731787	90.2395153	70.8457846
18:00:02		74.6930524	79.3812876	78.731787	90.2395153	69.8457846
18:00:03		74.6930524	79.3812876	78.731787	90.2395153	69.8457846
18:00:04		74.6930524	79.3812876	77.731787	89.2395153	69.8457846
18:00:05		74.6930524	78.3812876	77.731787	82.2395153	70.8457846
18:00:06		73.6930524	76.3812876	77.731787	82.2395153	70.8457846
18:00:07		73.6930524	67.3812876	77.731787	80.2395153	70.8457846
18:00:08		73.6930524	65.3812876	76.731787	77.2395153	70.8457846
18:00:09		72.6930524	65.3812876	76.731787	74.2395153	70.8457846
18:00:10		72.6930524	65.3812876	76.731787	74.2395153	70.8457846
18:00:11		73.6930524	67.3812876	76.731787	72.2395153	70.8457846
18:00:12		73.6930524	67.3812876	76.731787	72.2395153	70.8457846

18:00:13		74.6930524	67.3812876	76.731787	72.2395153	70.8457846
18:00:14		74.6930524	67.3812876	77.731787	72.2395153	78.8457846
18:00:15		74.6930524	67.3812876	78.731787	72.2395153	78.8457846
18:00:16		75.6930524	66.3812876	75.731787	72.2395153	81.8457846
18:00:17		74.6930524	65.3812876	73.731787	72.2395153	82.8457846
18:00:18		74.6930524	65.3812876	73.731787	74.2395153	82.8457846
18:00:19	0:00:00	74.6930524	65.3812876	74.731787	77.2395153	81.8457846
18:00:20	0:00:01	74.6930524	65.3812876	75.731787	80.2395153	81.8457846
18:00:21	0:00:02	74.6930524	63.3812876	75.731787	80.2395153	81.8457846
18:00:22	0:00:03	74.6930524	63.3812876	75.731787	81.2395153	81.8457846
18:00:23	0:00:04	73.6930524	63.3812876	75.731787	81.2395153	81.8457846
18:00:24	0:00:05	73.6930524	63.3812876	75.731787	82.2395153	81.8457846
18:00:25	0:00:06	71.6930524	61.3812876	76.731787	83.2395153	81.8457846
18:00:26	0:00:07	71.6930524	60.3812876	76.731787	84.2395153	81.8457846
18:00:27	0:00:08	72.6930524	59.3812876	76.731787	84.2395153	82.8457846
18:00:28	0:00:09	73.6930524	59.3812876	76.731787	85.2395153	82.8457846
18:00:29	0:00:10	75.6930524	59.3812876	76.731787	85.2395153	82.8457846
18:00:30	0:00:11	76.6930524	58.3812876	77.731787	85.2395153	81.8457846
18:00:31	0:00:12	78.6930524	58.3812876	82.731787	84.2395153	79.8457846
18:00:32	0:00:13	80.6930524	58.3812876	84.731787	85.2395153	78.8457846
18:00:33	0:00:14	80.6930524	58.3812876	84.731787	85.2395153	78.8457846
18:00:34	0:00:15	87.6930524	58.3812876	81.731787	86.2395153	77.8457846
18:00:35	0:00:16	88.6930524	58.3812876	77.731787	86.2395153	77.8457846
18:00:36	0:00:17	88.6930524	58.3812876	74.731787	86.2395153	76.8457846
18:00:37	0:00:18	88.6930524	60.3812876	72.731787	87.2395153	76.8457846
18:00:38	0:00:19	89.6930524	60.3812876	71.731787	87.2395153	76.8457846
18:00:39	0:00:20	89.6930524	61.3812876	71.731787	86.2395153	75.8457846
18:00:40	0:00:21	89.6930524	61.3812876	71.731787	84.2395153	75.8457846
18:00:41	0:00:22	90.6930524	61.3812876	71.731787	82.2395153	74.8457846
18:00:42	0:00:23	90.6930524	59.3812876	71.731787	82.2395153	74.8457846
18:00:43	0:00:24	88.6930524	58.3812876	71.731787	84.2395153	74.8457846
18:00:44	0:00:25	87.6930524	58.3812876	71.731787	79.2395153	74.8457846
18:00:45	0:00:26	86.6930524	57.3812876	71.731787	69.2395153	72.8457846
18:00:46	0:00:27	84.6930524	57.3812876	73.731787	66.2395153	70.8457846
18:00:47	0:00:28	84.6930524	57.3812876	73.731787	66.2395153	70.8457846
18:00:48	0:00:29	79.6930524	55.3812876	74.731787	66.2395153	70.8457846
18:00:49	0:00:30	78.6930524	55.3812876	77.731787	65.2395153	71.8457846
18:00:50	0:00:31	78.6930524	55.3812876	78.731787	63.2395153	72.8457846
18:00:51	0:00:32	78.6930524	55.3812876	79.731787	63.2395153	76.8457846
18:00:52	0:00:33	78.6930524	55.3812876	79.731787	63.2395153	78.8457846
18:00:53	0:00:34	78.6930524	56.3812876	79.731787	63.2395153	79.8457846
18:00:54	0:00:35	78.6930524	56.3812876	79.731787	63.2395153	79.8457846
18:00:55	0:00:36	78.6930524	56.3812876	80.731787	67.2395153	80.8457846
18:00:56	0:00:37	77.6930524	56.3812876	80.731787	69.2395153	81.8457846
18:00:57	0:00:38	78.6930524	56.3812876	81.731787	71.2395153	81.8457846

18:00:58	0:00:39	78.6930524	56.3812876	81.731787	72.2395153	82.8457846
18:00:59	0:00:40	78.6930524	56.3812876	81.731787	71.2395153	83.8457846
18:01:00	0:00:41	78.6930524	56.3812876	82.731787	71.2395153	82.8457846
18:01:01	0:00:42	80.6930524	57.3812876	79.731787	71.2395153	82.8457846
18:01:02	0:00:43	81.6930524	57.3812876	79.731787	70.2395153	82.8457846
18:01:03	0:00:44	81.6930524	57.3812876	75.731787	71.2395153	82.8457846
18:01:04	0:00:45	82.6930524	57.3812876	74.731787	72.2395153	82.8457846
18:01:05	0:00:46	81.6930524	58.3812876	74.731787	73.2395153	79.8457846
18:01:06	0:00:47	81.6930524	58.3812876	74.731787	73.2395153	75.8457846
18:01:07	0:00:48	81.6930524	58.3812876	74.731787	74.2395153	73.8457846
18:01:08	0:00:49	79.6930524	58.3812876	74.731787	75.2395153	71.8457846
18:01:09	0:00:50	79.6930524	58.3812876	74.731787	75.2395153	70.8457846
18:01:10	0:00:51	79.6930524	59.3812876	74.731787	74.2395153	70.8457846
18:01:11	0:00:52	78.6930524	60.3812876	73.731787	71.2395153	71.8457846
18:01:12	0:00:53	77.6930524	60.3812876	71.731787	71.2395153	72.8457846
18:01:13	0:00:54	77.6930524	61.3812876	70.731787	71.2395153	73.8457846
18:01:14	0:00:55	78.6930524	70.3812876	69.731787	71.2395153	74.8457846
18:01:15	0:00:56	78.6930524	71.3812876	69.731787	70.2395153	74.8457846
18:01:16	0:00:57	78.6930524	74.3812876	69.731787	70.2395153	74.8457846
18:01:17	0:00:58	78.6930524	77.3812876	69.731787	70.2395153	74.8457846
18:01:18	0:00:59	78.6930524	77.3812876	69.731787	69.2395153	74.8457846
18:01:19	0:01:00	77.6930524	81.3812876	70.731787	69.2395153	74.8457846
18:01:20	0:01:01	76.6930524	82.3812876	70.731787	68.2395153	74.8457846
18:01:21	0:01:02	75.6930524	82.3812876	70.731787	68.2395153	73.8457846
18:01:22	0:01:03	75.6930524	82.3812876	70.731787	69.2395153	73.8457846
18:01:23	0:01:04	74.6930524	83.3812876	70.731787	70.2395153	72.8457846
18:01:24	0:01:05	74.6930524	83.3812876	70.731787	71.2395153	72.8457846
18:01:25	0:01:06	74.6930524	83.3812876	70.731787	71.2395153	72.8457846
18:01:26	0:01:07	75.6930524	83.3812876	70.731787	72.2395153	72.8457846
18:01:27	0:01:08	75.6930524	82.3812876	70.731787	72.2395153	72.8457846
18:01:28	0:01:09	75.6930524	82.3812876	71.731787	72.2395153	72.8457846
18:01:29	0:01:10	75.6930524	79.3812876	71.731787	72.2395153	76.8457846
18:01:30	0:01:11	76.6930524	79.3812876	71.731787	72.2395153	77.8457846
18:01:31	0:01:12	76.6930524	69.3812876	72.731787	70.2395153	77.8457846
18:01:32	0:01:13	77.6930524	65.3812876	72.731787	70.2395153	77.8457846
18:01:33	0:01:14	78.6930524	64.3812876	72.731787	71.2395153	77.8457846
18:01:34	0:01:15	78.6930524	64.3812876	72.731787	72.2395153	77.8457846
18:01:35	0:01:16	79.6930524	62.3812876	72.731787	73.2395153	76.8457846
18:01:36	0:01:17	79.6930524	62.3812876	72.731787	73.2395153	75.8457846
18:01:37	0:01:18	78.6930524	60.3812876	73.731787	73.2395153	75.8457846
18:01:38	0:01:19	77.6930524	60.3812876	73.731787	73.2395153	72.8457846
18:01:39	0:01:20	77.6930524	60.3812876	73.731787	72.2395153	71.8457846
18:01:40	0:01:21	76.6930524	60.3812876	73.731787	73.2395153	69.8457846
18:01:41	0:01:22	76.6930524	61.3812876	73.731787	73.2395153	68.8457846
18:01:42	0:01:23	76.6930524	60.3812876	73.731787	74.2395153	68.8457846

18:01:43	0:01:24	76.6930524	58.3812876	73.731787	74.2395153	68.8457846
18:01:44	0:01:25	75.6930524	58.3812876	73.731787	74.2395153	67.8457846
18:01:45	0:01:26	75.6930524	58.3812876	73.731787	75.2395153	66.8457846
18:01:46	0:01:27	75.6930524	58.3812876	73.731787	72.2395153	66.8457846
18:01:47	0:01:28	75.6930524	59.3812876	74.731787	72.2395153	65.8457846
18:01:48	0:01:29	75.6930524	59.3812876	74.731787	72.2395153	65.8457846
18:01:49	0:01:30	76.6930524	59.3812876	76.731787	71.2395153	63.8457846
18:01:50	0:01:31	77.6930524	62.3812876	76.731787	71.2395153	63.8457846
18:01:51	0:01:32	77.6930524	62.3812876	76.731787	71.2395153	63.8457846
18:01:52	0:01:33	75.6930524	63.3812876	76.731787	70.2395153	63.8457846
18:01:53	0:01:34	74.6930524	62.3812876	76.731787	68.2395153	61.8457846
18:01:54	0:01:35	74.6930524	61.3812876	75.731787	67.2395153	61.8457846
18:01:55	0:01:36	74.6930524	61.3812876	75.731787	67.2395153	60.8457846
18:01:56	0:01:37	73.6930524	61.3812876	75.731787	67.2395153	60.8457846
18:01:57	0:01:38	73.6930524	61.3812876	75.731787	67.2395153	60.8457846
18:01:58	0:01:39	74.6930524	61.3812876	75.731787	67.2395153	60.8457846
18:01:59	0:01:40	74.6930524	61.3812876	73.731787	67.2395153	61.8457846
18:02:00	0:01:41	75.6930524	62.3812876	73.731787	66.2395153	61.8457846
18:02:01	0:01:42	75.6930524	62.3812876	73.731787	66.2395153	62.8457846
18:02:02	0:01:43	76.6930524	62.3812876	74.731787	66.2395153	63.8457846
18:02:03	0:01:44	76.6930524	62.3812876	74.731787	66.2395153	63.8457846
18:02:04	0:01:45	76.6930524	62.3812876	74.731787	66.2395153	64.8457846
18:02:05	0:01:46	76.6930524	63.3812876	74.731787	66.2395153	65.8457846
18:02:06	0:01:47	75.6930524	63.3812876	73.731787	67.2395153	66.8457846
18:02:07	0:01:48	75.6930524	63.3812876	73.731787	69.2395153	66.8457846
18:02:08	0:01:49	73.6930524	63.3812876	73.731787	70.2395153	68.8457846
18:02:09	0:01:50	73.6930524	61.3812876	72.731787	70.2395153	68.8457846
18:02:10	0:01:51	72.6930524	61.3812876	72.731787	71.2395153	70.8457846
18:02:11	0:01:52	72.6930524	61.3812876	72.731787	71.2395153	70.8457846
18:02:12	0:01:53	73.6930524	61.3812876	72.731787	71.2395153	69.8457846
18:02:13	0:01:54	73.6930524	62.3812876	72.731787	72.2395153	68.8457846
18:02:14	0:01:55	73.6930524	64.3812876	72.731787	71.2395153	68.8457846
18:02:15	0:01:56	73.6930524	67.3812876	72.731787	71.2395153	68.8457846
18:02:16	0:01:57	73.6930524	67.3812876	72.731787	71.2395153	68.8457846
18:02:17	0:01:58	73.6930524	68.3812876	73.731787	71.2395153	68.8457846
18:02:18	0:01:59	73.6930524	68.3812876	73.731787	71.2395153	68.8457846
18:02:19	0:02:00	72.6930524	68.3812876	74.731787	71.2395153	69.8457846
18:02:20	0:02:01	73.6930524	70.3812876	74.731787	71.2395153	68.8457846
18:02:21	0:02:02	72.6930524	70.3812876	74.731787	72.2395153	67.8457846
18:02:22	0:02:03	72.6930524	69.3812876	75.731787	73.2395153	66.8457846
18:02:23	0:02:04	73.6930524	69.3812876	76.731787	73.2395153	68.8457846
18:02:24	0:02:05	73.6930524	69.3812876	76.731787	72.2395153	72.8457846
18:02:25	0:02:06	74.6930524	70.3812876	77.731787	71.2395153	73.8457846
18:02:26	0:02:07	74.6930524	71.3812876	76.731787	71.2395153	74.8457846
18:02:27	0:02:08	74.6930524	70.3812876	74.731787	71.2395153	73.8457846

18:02:28	0:02:09	74.6930524	70.3812876	73.731787	69.2395153	73.8457846
18:02:29	0:02:10	73.6930524	66.3812876	73.731787	69.2395153	73.8457846
18:02:30	0:02:11	73.6930524	66.3812876	73.731787	69.2395153	73.8457846
18:02:31	0:02:12	73.6930524	65.3812876	73.731787	71.2395153	73.8457846
18:02:32	0:02:13	73.6930524	65.3812876	73.731787	71.2395153	73.8457846
18:02:33	0:02:14	73.6930524	65.3812876	73.731787	72.2395153	72.8457846
18:02:34	0:02:15	72.6930524	65.3812876	72.731787	72.2395153	72.8457846
18:02:35	0:02:16	73.6930524	65.3812876	71.731787	71.2395153	70.8457846
18:02:36	0:02:17	73.6930524	64.3812876	70.731787	70.2395153	69.8457846
18:02:37	0:02:18	73.6930524	64.3812876	69.731787	69.2395153	66.8457846
18:02:38	0:02:19	74.6930524	64.3812876	69.731787	69.2395153	66.8457846
18:02:39	0:02:20	74.6930524	64.3812876	68.731787	70.2395153	67.8457846
18:02:40	0:02:21	74.6930524	64.3812876	67.731787	71.2395153	67.8457846
18:02:41	0:02:22	74.6930524	64.3812876	66.731787	71.2395153	67.8457846
18:02:42	0:02:23	74.6930524	64.3812876	66.731787	71.2395153	67.8457846
18:02:43	0:02:24	74.6930524	64.3812876	66.731787	71.2395153	67.8457846
18:02:44	0:02:25	75.6930524	64.3812876	64.731787	72.2395153	68.8457846
18:02:45	0:02:26	75.6930524	63.3812876	64.731787	72.2395153	68.8457846
18:02:46	0:02:27	74.6930524	62.3812876	64.731787	75.2395153	68.8457846
18:02:47	0:02:28	73.6930524	62.3812876	64.731787	75.2395153	69.8457846
18:02:48	0:02:29	73.6930524	62.3812876	64.731787	75.2395153	76.8457846
18:02:49	0:02:30	73.6930524	63.3812876	64.731787	75.2395153	77.8457846
18:02:50	0:02:31	73.6930524	63.3812876	64.731787	78.2395153	76.8457846
18:02:51	0:02:32	73.6930524	64.3812876	63.731787	81.2395153	75.8457846
18:02:52	0:02:33	74.6930524	65.3812876	62.731787	82.2395153	75.8457846
18:02:53	0:02:34	74.6930524	65.3812876	62.731787	82.2395153	74.8457846
18:02:54	0:02:35	74.6930524	65.3812876	62.731787	86.2395153	74.8457846
18:02:55	0:02:36	74.6930524	66.3812876	62.731787	88.2395153	75.8457846
18:02:56	0:02:37	74.6930524	66.3812876	62.731787	89.2395153	77.8457846
18:02:57	0:02:38	74.6930524	68.3812876	62.731787	91.2395153	78.8457846
18:02:58	0:02:39	73.6930524	69.3812876	62.731787	91.2395153	79.8457846
18:02:59	0:02:40	72.6930524	69.3812876	62.731787	91.2395153	80.8457846
18:03:00	0:02:41	70.6930524	69.3812876	63.731787	92.2395153	81.8457846
18:03:01	0:02:42	70.6930524	69.3812876	67.731787	92.2395153	80.8457846
18:03:02	0:02:43	69.6930524	69.3812876	71.731787	93.2395153	78.8457846
18:03:03	0:02:44	68.6930524	69.3812876	72.731787	93.2395153	77.8457846
18:03:04	0:02:45	68.6930524	69.3812876	72.731787	92.2395153	77.8457846
18:03:05	0:02:46	68.6930524	69.3812876	73.731787	90.2395153	77.8457846
18:03:06	0:02:47	69.6930524	70.3812876	75.731787	88.2395153	77.8457846
18:03:07	0:02:48	69.6930524	69.3812876	75.731787	88.2395153	76.8457846
18:03:08	0:02:49	69.6930524	69.3812876	75.731787	86.2395153	74.8457846
18:03:09	0:02:50	69.6930524	70.3812876	76.731787	84.2395153	72.8457846
18:03:10	0:02:51	69.6930524	70.3812876	76.731787	83.2395153	72.8457846
18:03:11	0:02:52	69.6930524	70.3812876	75.731787	83.2395153	72.8457846
18:03:12	0:02:53	70.6930524	68.3812876	74.731787	83.2395153	72.8457846

18:03:13	0:02:54	70.6930524	68.3812876	74.731787	83.2395153	72.8457846
18:03:14	0:02:55	70.6930524	68.3812876	74.731787	83.2395153	72.8457846
18:03:15	0:02:56	70.6930524	68.3812876	74.731787	80.2395153	73.8457846
18:03:16	0:02:57	70.6930524	69.3812876	74.731787	80.2395153	73.8457846
18:03:17	0:02:58	70.6930524	69.3812876	73.731787	80.2395153	73.8457846
18:03:18	0:02:59	71.6930524	69.3812876	73.731787	79.2395153	75.8457846
18:03:19	0:03:00	72.6930524	69.3812876	73.731787	79.2395153	75.8457846
18:03:20	0:03:01	73.6930524	69.3812876	73.731787	79.2395153	74.8457846
18:03:21	0:03:02	74.6930524	70.3812876	73.731787	78.2395153	74.8457846
18:03:22	0:03:03	74.6930524	70.3812876	73.731787	77.2395153	74.8457846
18:03:23	0:03:04	71.6930524	71.3812876	73.731787	77.2395153	74.8457846
18:03:24	0:03:05	70.6930524	71.3812876	73.731787	75.2395153	75.8457846
18:03:25	0:03:06	69.6930524	72.3812876	73.731787	73.2395153	75.8457846
18:03:26	0:03:07	69.6930524	74.3812876	74.731787	73.2395153	75.8457846
18:03:27	0:03:08	69.6930524	74.3812876	70.731787	74.2395153	75.8457846
18:03:28	0:03:09	69.6930524	74.3812876	70.731787	74.2395153	76.8457846
18:03:29	0:03:10	68.6930524	74.3812876	68.731787	74.2395153	76.8457846
18:03:30	0:03:11	68.6930524	74.3812876	68.731787	74.2395153	76.8457846
18:03:31	0:03:12	68.6930524	75.3812876	68.731787	72.2395153	78.8457846
18:03:32	0:03:13	68.6930524	75.3812876	69.731787	72.2395153	78.8457846
18:03:33	0:03:14	68.6930524	76.3812876	69.731787	69.2395153	78.8457846
18:03:34	0:03:15	68.6930524	76.3812876	69.731787	67.2395153	77.8457846
18:03:35	0:03:16	72.6930524	76.3812876	69.731787	67.2395153	76.8457846
18:03:36	0:03:17	72.6930524	76.3812876	69.731787	67.2395153	76.8457846
18:03:37	0:03:18	72.6930524	75.3812876	69.731787	67.2395153	73.8457846
18:03:38	0:03:19	72.6930524	75.3812876	69.731787	68.2395153	73.8457846
18:03:39	0:03:20	72.6930524	74.3812876	67.731787	67.2395153	72.8457846
18:03:40	0:03:21	73.6930524	74.3812876	67.731787	66.2395153	72.8457846
18:03:41	0:03:22	73.6930524	72.3812876	67.731787	66.2395153	72.8457846
18:03:42	0:03:23	73.6930524	72.3812876	67.731787	66.2395153	72.8457846
18:03:43	0:03:24	72.6930524	72.3812876	67.731787	66.2395153	72.8457846
18:03:44	0:03:25	72.6930524	72.3812876	69.731787	65.2395153	71.8457846
18:03:45	0:03:26	71.6930524	72.3812876	70.731787	69.2395153	71.8457846
18:03:46	0:03:27	70.6930524	73.3812876	71.731787	70.2395153	71.8457846
18:03:47	0:03:28	70.6930524	72.3812876	71.731787	69.2395153	71.8457846
18:03:48	0:03:29	69.6930524	71.3812876	71.731787	69.2395153	71.8457846
18:03:49	0:03:30	69.6930524	71.3812876	71.731787	69.2395153	70.8457846
18:03:50	0:03:31	68.6930524	71.3812876	71.731787	69.2395153	70.8457846
18:03:51	0:03:32	68.6930524	71.3812876	71.731787	70.2395153	70.8457846
18:03:52	0:03:33	69.6930524	71.3812876	71.731787	70.2395153	71.8457846
18:03:53	0:03:34	69.6930524	71.3812876	71.731787	70.2395153	71.8457846
18:03:54	0:03:35	69.6930524	71.3812876	71.731787	69.2395153	71.8457846
18:03:55	0:03:36	69.6930524	70.3812876	73.731787	69.2395153	71.8457846
18:03:56	0:03:37	69.6930524	71.3812876	73.731787	68.2395153	73.8457846
18:03:57	0:03:38	69.6930524	71.3812876	73.731787	68.2395153	73.8457846

18:03:58	0:03:39	69.6930524	71.3812876	73.731787	67.2395153	75.8457846
18:03:59	0:03:40	69.6930524	71.3812876	72.731787	67.2395153	75.8457846
18:04:00	0:03:41	69.6930524	71.3812876	71.731787	66.2395153	75.8457846
18:04:01	0:03:42	69.6930524	71.3812876	70.731787	65.2395153	75.8457846
18:04:02	0:03:43	70.6930524	71.3812876	70.731787	65.2395153	75.8457846
18:04:03	0:03:44	70.6930524	72.3812876	70.731787	63.2395153	74.8457846
18:04:04	0:03:45	70.6930524	72.3812876	71.731787	62.2395153	74.8457846
18:04:05	0:03:46	71.6930524	75.3812876	71.731787	62.2395153	72.8457846
18:04:06	0:03:47	71.6930524	75.3812876	71.731787	62.2395153	71.8457846
18:04:07	0:03:48	71.6930524	75.3812876	71.731787	62.2395153	67.8457846
18:04:08	0:03:49	70.6930524	75.3812876	72.731787	62.2395153	67.8457846
18:04:09	0:03:50	70.6930524	74.3812876	72.731787	62.2395153	67.8457846
18:04:10	0:03:51	70.6930524	74.3812876	72.731787	64.2395153	67.8457846
18:04:11	0:03:52	70.6930524	74.3812876	72.731787	64.2395153	67.8457846
18:04:12	0:03:53	70.6930524	74.3812876	72.731787	65.2395153	68.8457846
18:04:13	0:03:54	70.6930524	74.3812876	72.731787	65.2395153	69.8457846
18:04:14	0:03:55	71.6930524	73.3812876	72.731787	65.2395153	69.8457846
18:04:15	0:03:56	71.6930524	73.3812876	72.731787	65.2395153	70.8457846
18:04:16	0:03:57	72.6930524	70.3812876	71.731787	66.2395153	70.8457846
18:04:17	0:03:58	72.6930524	70.3812876	71.731787	67.2395153	70.8457846
18:04:18	0:03:59	72.6930524	70.3812876	71.731787	67.2395153	70.8457846
18:04:19	0:04:00	72.6930524	70.3812876	71.731787	68.2395153	71.8457846
18:04:20	0:04:01	72.6930524	70.3812876	73.731787	68.2395153	72.8457846
18:04:21	0:04:02	72.6930524	74.3812876	74.731787	72.2395153	73.8457846
18:04:22	0:04:03	73.6930524	75.3812876	74.731787	74.2395153	73.8457846
18:04:23	0:04:04	73.6930524	76.3812876	73.731787	75.2395153	73.8457846
18:04:24	0:04:05	73.6930524	76.3812876	73.731787	75.2395153	73.8457846
18:04:25	0:04:06	73.6930524	76.3812876	72.731787	75.2395153	73.8457846
18:04:26	0:04:07	74.6930524	76.3812876	72.731787	76.2395153	73.8457846
18:04:27	0:04:08	74.6930524	76.3812876	72.731787	79.2395153	73.8457846
18:04:28	0:04:09	73.6930524	76.3812876	72.731787	79.2395153	73.8457846
18:04:29	0:04:10	73.6930524	75.3812876	72.731787	79.2395153	73.8457846
18:04:30	0:04:11	73.6930524	75.3812876	72.731787	79.2395153	73.8457846
18:04:31	0:04:12	70.6930524	75.3812876	72.731787	80.2395153	74.8457846
18:04:32	0:04:13	70.6930524	75.3812876	71.731787	80.2395153	73.8457846
18:04:33	0:04:14	68.6930524	75.3812876	71.731787	82.2395153	73.8457846
18:04:34	0:04:15	68.6930524	75.3812876	71.731787	83.2395153	72.8457846
18:04:35	0:04:16	68.6930524	75.3812876	71.731787	83.2395153	71.8457846
18:04:36	0:04:17	68.6930524	75.3812876	70.731787	83.2395153	72.8457846
18:04:37	0:04:18	68.6930524	76.3812876	70.731787	83.2395153	73.8457846
18:04:38	0:04:19	68.6930524	82.3812876	70.731787	84.2395153	73.8457846
18:04:39	0:04:20	68.6930524	82.3812876	71.731787	84.2395153	74.8457846
18:04:40	0:04:21	71.6930524	80.3812876	71.731787	84.2395153	75.8457846
18:04:41	0:04:22	71.6930524	79.3812876	71.731787	84.2395153	77.8457846
18:04:42	0:04:23	71.6930524	79.3812876	70.731787	84.2395153	78.8457846

18:04:43	0:04:24	71.6930524	80.3812876	70.731787	84.2395153	78.8457846
18:04:44	0:04:25	71.6930524	79.3812876	71.731787	84.2395153	79.8457846
18:04:45	0:04:26	71.6930524	77.3812876	71.731787	84.2395153	80.8457846
18:04:46	0:04:27	71.6930524	77.3812876	71.731787	84.2395153	82.8457846
18:04:47	0:04:28	70.6930524	74.3812876	71.731787	84.2395153	83.8457846
18:04:48	0:04:29	70.6930524	73.3812876	71.731787	84.2395153	85.8457846
18:04:49	0:04:30	70.6930524	72.3812876	72.731787	84.2395153	85.8457846
18:04:50	0:04:31	69.6930524	72.3812876	72.731787	82.2395153	85.8457846
18:04:51	0:04:32	69.6930524	68.3812876	72.731787	79.2395153	85.8457846
18:04:52	0:04:33	69.6930524	66.3812876	71.731787	79.2395153	85.8457846
18:04:53	0:04:34	69.6930524	67.3812876	71.731787	77.2395153	83.8457846
18:04:54	0:04:35	69.6930524	71.3812876	70.731787	75.2395153	82.8457846
18:04:55	0:04:36	69.6930524	73.3812876	70.731787	74.2395153	82.8457846
18:04:56	0:04:37	69.6930524	73.3812876	70.731787	74.2395153	78.8457846
18:04:57	0:04:38	69.6930524	73.3812876	71.731787	75.2395153	75.8457846
18:04:58	0:04:39	69.6930524	73.3812876	71.731787	76.2395153	74.8457846
18:04:59	0:04:40	69.6930524	70.3812876	71.731787	76.2395153	74.8457846
18:05:00	0:04:41	68.6930524	69.3812876	71.731787	76.2395153	74.8457846
18:05:01	0:04:42	68.6930524	69.3812876	71.731787	79.2395153	74.8457846
18:05:02	0:04:43	68.6930524	72.3812876	71.731787	79.2395153	76.8457846
18:05:03	0:04:44	68.6930524	74.3812876	71.731787	81.2395153	77.8457846
18:05:04	0:04:45	68.6930524	74.3812876	71.731787	82.2395153	77.8457846
18:05:05	0:04:46	67.6930524	74.3812876	71.731787	82.2395153	77.8457846
18:05:06	0:04:47	67.6930524	73.3812876	73.731787	82.2395153	77.8457846
18:05:07	0:04:48	67.6930524	73.3812876	73.731787	81.2395153	78.8457846
18:05:08	0:04:49	67.6930524	73.3812876	73.731787	81.2395153	78.8457846
18:05:09	0:04:50	67.6930524	72.3812876	71.731787	80.2395153	79.8457846
18:05:10	0:04:51	67.6930524	72.3812876	71.731787	79.2395153	80.8457846
18:05:11	0:04:52	67.6930524	72.3812876	70.731787	78.2395153	80.8457846
18:05:12	0:04:53	67.6930524	70.3812876	70.731787	77.2395153	81.8457846
18:05:13	0:04:54	67.6930524	70.3812876	69.731787	77.2395153	80.8457846
18:05:14	0:04:55	67.6930524	68.3812876	70.731787	77.2395153	80.8457846
18:05:15	0:04:56	68.6930524	67.3812876	72.731787	77.2395153	78.8457846
18:05:16	0:04:57	68.6930524	66.3812876	71.731787	78.2395153	77.8457846
18:05:17	0:04:58	69.6930524	66.3812876	71.731787	79.2395153	77.8457846
18:05:18	0:04:59	69.6930524	65.3812876	71.731787	81.2395153	76.8457846
18:05:19	0:05:00	69.6930524	64.3812876	71.731787	81.2395153	75.8457846
18:05:20	0:05:01	69.6930524	64.3812876	71.731787	83.2395153	73.8457846
18:05:21	0:05:02	69.6930524	64.3812876	72.731787	84.2395153	69.8457846
18:05:22	0:05:03	68.6930524	64.3812876	72.731787	84.2395153	67.8457846
18:05:23	0:05:04	68.6930524	64.3812876	72.731787	84.2395153	65.8457846
18:05:24	0:05:05	68.6930524	64.3812876	72.731787	84.2395153	65.8457846
18:05:25	0:05:06	68.6930524	64.3812876	73.731787	84.2395153	64.8457846
18:05:26	0:05:07	69.6930524	68.3812876	73.731787	85.2395153	64.8457846
18:05:27	0:05:08	69.6930524	69.3812876	73.731787	85.2395153	64.8457846

18:05:28	0:05:09	70.6930524	69.3812876	77.731787	85.2395153	63.8457846
18:05:29	0:05:10	70.6930524	70.3812876	77.731787	86.2395153	63.8457846
18:05:30	0:05:11	70.6930524	70.3812876	77.731787	86.2395153	63.8457846
18:05:31	0:05:12	71.6930524	70.3812876	75.731787	86.2395153	63.8457846
18:05:32	0:05:13	70.6930524	71.3812876	75.731787	87.2395153	62.8457846
18:05:33	0:05:14	70.6930524	71.3812876	77.731787	87.2395153	61.8457846
18:05:34	0:05:15	70.6930524	71.3812876	76.731787	86.2395153	62.8457846
18:05:35	0:05:16	71.6930524	70.3812876	75.731787	85.2395153	64.8457846
18:05:36	0:05:17	70.6930524	69.3812876	74.731787	84.2395153	66.8457846
18:05:37	0:05:18	70.6930524	69.3812876	73.731787	84.2395153	68.8457846
18:05:38	0:05:19	71.6930524	68.3812876	74.731787	84.2395153	70.8457846
18:05:39	0:05:20	72.6930524	74.3812876	75.731787	82.2395153	70.8457846
18:05:40	0:05:21	72.6930524	74.3812876	76.731787	80.2395153	70.8457846
18:05:41	0:05:22	72.6930524	74.3812876	76.731787	80.2395153	70.8457846
18:05:42	0:05:23	72.6930524	74.3812876	76.731787	79.2395153	69.8457846
18:05:43	0:05:24	72.6930524	72.3812876	75.731787	79.2395153	70.8457846
18:05:44	0:05:25	72.6930524	72.3812876	74.731787	78.2395153	71.8457846
18:05:45	0:05:26	72.6930524	70.3812876	74.731787	77.2395153	71.8457846
18:05:46	0:05:27	72.6930524	70.3812876	74.731787	72.2395153	70.8457846
18:05:47	0:05:28	72.6930524	70.3812876	73.731787	71.2395153	69.8457846
18:05:48	0:05:29	73.6930524	69.3812876	73.731787	71.2395153	69.8457846
18:05:49	0:05:30	73.6930524	69.3812876	73.731787	71.2395153	69.8457846
18:05:50	0:05:31	73.6930524	68.3812876	72.731787	71.2395153	68.8457846
18:05:51	0:05:32	73.6930524	68.3812876	72.731787	71.2395153	67.8457846
18:05:52	0:05:33	74.6930524	68.3812876	72.731787	66.2395153	66.8457846
18:05:53	0:05:34	75.6930524	68.3812876	72.731787	65.2395153	66.8457846
18:05:54	0:05:35	76.6930524	71.3812876	72.731787	65.2395153	66.8457846
18:05:55	0:05:36	76.6930524	73.3812876	73.731787	65.2395153	66.8457846
18:05:56	0:05:37	77.6930524	75.3812876	73.731787	63.2395153	66.8457846
18:05:57	0:05:38	76.6930524	75.3812876	73.731787	61.2395153	61.8457846
18:05:58	0:05:39	76.6930524	75.3812876	72.731787	62.2395153	62.8457846
18:05:59	0:05:40	75.6930524	75.3812876	72.731787	62.2395153	62.8457846
18:06:00	0:05:41	75.6930524	75.3812876	71.731787	64.2395153	62.8457846
18:06:01	0:05:42	74.6930524	75.3812876	71.731787	67.2395153	62.8457846
18:06:02	0:05:43	74.6930524	72.3812876	71.731787	69.2395153	62.8457846
18:06:03	0:05:44	73.6930524	73.3812876	71.731787	69.2395153	65.8457846
18:06:04	0:05:45	73.6930524	73.3812876	72.731787	69.2395153	68.8457846
18:06:05	0:05:46	73.6930524	73.3812876	73.731787	72.2395153	68.8457846
18:06:06	0:05:47	73.6930524	73.3812876	74.731787	75.2395153	68.8457846
18:06:07	0:05:48	73.6930524	73.3812876	75.731787	75.2395153	68.8457846
18:06:08	0:05:49	73.6930524	73.3812876	75.731787	80.2395153	68.8457846
18:06:09	0:05:50	74.6930524	72.3812876	75.731787	84.2395153	68.8457846
18:06:10	0:05:51	74.6930524	69.3812876	75.731787	86.2395153	69.8457846
18:06:11	0:05:52	74.6930524	67.3812876	75.731787	86.2395153	69.8457846
18:06:12	0:05:53	74.6930524	67.3812876	75.731787	75.5451505	72.8457846

18:06:13	0:05:54	74.6930524	66.3812876	75.731787	75.5451505	73.8457846
18:06:14	0:05:55	73.6930524	65.3812876	75.731787	75.2951505	73.8457846
18:06:15	0:05:56	73.6930524	64.3812876	76.731787	75.4201505	73.8457846
18:06:16	0:05:57	73.6930524	64.3812876	75.731787	75.0451505	73.8457846
18:06:17	0:05:58	74.6930524	64.3812876	74.731787	75.0451505	73.8457846
18:06:18	0:05:59	74.6930524	64.3812876	74.731787	75.0451505	73.8457846
18:06:19	0:06:00	74.6930524	69.3812876	74.731787	75.7951505	74.8457846
18:06:20	0:06:01	74.6930524	69.3812876	74.731787	75.7951505	74.8457846
18:06:21	0:06:02	74.6930524	69.3812876	74.731787	75.9201505	74.8457846
18:06:22	0:06:03	73.6930524	69.3812876	74.731787	76.2951505	74.8457846
18:06:23	0:06:04	73.6930524	67.3812876	75.731787	76.6701505	73.8457846
18:06:24	0:06:05	74.6930524	67.3812876	75.731787	76.4201505	70.8457846
18:06:25	0:06:06	73.6930524	68.3812876	74.731787	76.2951505	70.8457846
18:06:26	0:06:07	74.6930524	69.3812876	74.731787	92.2395153	70.8457846
18:06:27	0:06:08	74.6930524	71.3812876	74.731787	90.2395153	70.8457846
18:06:28	0:06:09	74.6930524	71.3812876	74.731787	90.2395153	70.8457846
18:06:29	0:06:10	74.6930524	72.3812876	74.731787	90.2395153	70.8457846
18:06:30	0:06:11	74.6930524	72.3812876	74.731787	90.2395153	70.8457846
18:06:31	0:06:12	74.6930524	72.3812876	72.731787	90.2395153	70.8457846
18:06:32	0:06:13	74.6930524	73.3812876	72.731787	88.2395153	70.8457846
18:06:33	0:06:14	74.6930524	73.3812876	73.731787	87.2395153	69.8457846
18:06:34	0:06:15	74.6930524	73.3812876	73.731787	87.2395153	69.8457846
18:06:35	0:06:16	75.6930524	73.3812876	73.731787	87.2395153	69.8457846
18:06:36	0:06:17	74.6930524	74.3812876	73.731787	83.2395153	69.8457846
18:06:37	0:06:18	74.6930524	75.3812876	75.731787	79.2395153	69.8457846
18:06:38	0:06:19	74.6930524	75.3812876	76.731787	79.2395153	68.8457846
18:06:39	0:06:20	74.6930524	74.3812876	77.731787	73.2395153	68.8457846
18:06:40	0:06:21	74.6930524	73.3812876	78.731787	74.2395153	68.8457846
18:06:41	0:06:22	74.6930524	73.3812876	82.731787	74.2395153	69.8457846
18:06:42	0:06:23	74.6930524	72.3812876	82.731787	74.2395153	69.8457846
18:06:43	0:06:24	73.6930524	72.3812876	81.731787	74.2395153	70.8457846
18:06:44	0:06:25	74.6930524	72.3812876	79.731787	74.2395153	70.8457846
18:06:45	0:06:26	74.6930524	72.3812876	78.731787	74.2395153	69.8457846
18:06:46	0:06:27	74.6930524	73.3812876	78.731787	74.2395153	69.8457846
18:06:47	0:06:28	74.6930524	73.3812876	77.731787	74.2395153	70.8457846
18:06:48	0:06:29	74.6930524	73.3812876	77.731787	74.2395153	70.8457846
18:06:49	0:06:30	75.6930524	73.3812876	77.731787	74.2395153	70.8457846
18:06:50	0:06:31	75.6930524	74.3812876	77.731787	74.2395153	69.8457846
18:06:51	0:06:32	75.6930524	74.3812876	77.731787	74.2395153	67.8457846
18:06:52	0:06:33	75.6930524	74.3812876	76.731787	75.2395153	67.8457846
18:06:53	0:06:34	75.6930524	74.3812876	76.731787	76.2395153	67.8457846
18:06:54	0:06:35	75.6930524	74.3812876	76.731787	76.2395153	67.8457846
18:06:55	0:06:36	74.6930524	74.3812876	76.731787	77.2395153	67.8457846
18:06:56	0:06:37	74.6930524	74.3812876	76.731787	77.2395153	66.8457846
18:06:57	0:06:38	74.6930524	75.3812876	74.731787	78.2395153	67.8457846

18:06:58	0:06:39	74.6930524	75.3812876	72.731787	78.2395153	69.8457846
18:06:59	0:06:40	74.6930524	75.3812876	71.731787	79.2395153	69.8457846
18:07:00	0:06:41	74.6930524	70.3812876	71.731787	79.2395153	69.8457846
18:07:01	0:06:42	74.6930524	70.3812876	71.731787	79.2395153	69.8457846
18:07:02	0:06:43	75.6930524	69.3812876	72.731787	80.2395153	68.8457846
18:07:03	0:06:44	76.6930524	68.3812876	74.731787	84.2395153	69.8457846
18:07:04	0:06:45	76.6930524	68.3812876	74.731787	86.2395153	69.8457846
18:07:05	0:06:46	76.6930524	68.3812876	74.731787	76.2395153	69.8457846
18:07:06	0:06:47	76.6930524	67.3812876	74.731787	72.2395153	69.8457846
18:07:07	0:06:48	76.6930524	67.3812876	75.731787	72.2395153	69.8457846
18:07:08	0:06:49	76.6930524	67.3812876	75.731787	71.2395153	69.8457846
18:07:09	0:06:50	76.6930524	67.3812876	75.731787	71.2395153	71.8457846
18:07:10	0:06:51	76.6930524	67.3812876	75.731787	71.2395153	72.8457846
18:07:11	0:06:52	75.6930524	66.3812876	75.731787	71.2395153	72.8457846
18:07:12	0:06:53	75.6930524	66.3812876	75.731787	71.2395153	72.8457846
18:07:13	0:06:54	75.6930524	65.3812876	76.731787	72.2395153	72.8457846
18:07:14	0:06:55	75.6930524	65.3812876	75.731787	72.2395153	73.8457846
18:07:15	0:06:56	74.6930524	67.3812876	74.731787	74.2395153	73.8457846
18:07:16	0:06:57	74.6930524	67.3812876	74.731787	75.2395153	72.8457846
18:07:17	0:06:58	74.6930524	68.3812876	73.731787	76.2395153	72.8457846
18:07:18	0:06:59	73.6930524	68.3812876	73.731787	76.2395153	73.8457846
18:07:19	0:07:00	73.6930524	68.3812876	73.731787	76.2395153	73.8457846
18:07:20	0:07:01	73.6930524	69.3812876	73.731787	77.2395153	75.8457846
18:07:21	0:07:02	73.6930524	69.3812876	73.731787	77.2395153	75.8457846
18:07:22	0:07:03	73.6930524	70.3812876	73.731787	76.2395153	75.8457846
18:07:23	0:07:04	73.6930524	69.3812876	73.731787	76.2395153	75.8457846
18:07:24	0:07:05	73.6930524	68.3812876	72.731787	76.2395153	75.8457846
18:07:25	0:07:06	73.6930524	68.3812876	71.731787	76.2395153	76.8457846
18:07:26	0:07:07	73.6930524	68.3812876	71.731787	76.2395153	76.8457846
18:07:27	0:07:08	73.6930524	68.3812876	72.731787	77.2395153	77.8457846
18:07:28	0:07:09	72.6930524	68.3812876	72.731787	77.2395153	77.8457846
18:07:29	0:07:10	72.6930524	68.3812876	73.731787	77.2395153	77.8457846
18:07:30	0:07:11	72.6930524	68.3812876	73.731787	77.2395153	77.8457846
18:07:31	0:07:12	72.6930524	68.3812876	73.731787	77.2395153	77.8457846
18:07:32	0:07:13	72.6930524	68.3812876	73.731787	77.2395153	77.8457846
18:07:33	0:07:14	72.6930524	71.3812876	73.731787	77.2395153	77.8457846
18:07:34	0:07:15	72.6930524	71.3812876	73.731787	77.2395153	76.8457846
18:07:35	0:07:16	72.6930524	71.3812876	73.731787	77.2395153	76.8457846
18:07:36	0:07:17	72.6930524	71.3812876	73.731787	78.2395153	75.8457846
18:07:37	0:07:18	71.6930524	71.3812876	72.731787	78.2395153	74.8457846
18:07:38	0:07:19	71.6930524	71.3812876	72.731787	78.2395153	74.8457846
18:07:39	0:07:20	74.6930524	71.3812876	73.731787	78.2395153	74.8457846
18:07:40	0:07:21	75.6930524	72.3812876	73.731787	77.2395153	73.8457846
18:07:41	0:07:22	76.6930524	72.3812876	72.731787	77.2395153	72.8457846
18:07:42	0:07:23	76.6930524	73.3812876	70.731787	77.2395153	73.8457846

18:07:43	0:07:24	76.6930524	73.3812876	70.731787	76.2395153	73.8457846
18:07:44	0:07:25	76.6930524	74.3812876	69.731787	76.2395153	73.8457846
18:07:45	0:07:26	75.6930524	74.3812876	69.731787	76.2395153	73.8457846
18:07:46	0:07:27	76.6930524	74.3812876	69.731787	76.2395153	73.8457846
18:07:47	0:07:28	75.6930524	74.3812876	70.731787	76.2395153	74.8457846
18:07:48	0:07:29	76.6930524	74.3812876	71.731787	76.2395153	74.8457846
18:07:49	0:07:30	76.6930524	74.3812876	71.731787	76.2395153	75.8457846
18:07:50	0:07:31	76.6930524	74.3812876	72.731787	77.2395153	75.8457846
18:07:51	0:07:32	75.6930524	74.3812876	72.731787	78.2395153	75.8457846
18:07:52	0:07:33	75.6930524	74.3812876	72.731787	78.2395153	75.8457846
18:07:53	0:07:34	74.6930524	74.3812876	72.731787	78.2395153	75.8457846
18:07:54	0:07:35	72.6930524	74.3812876	72.731787	79.2395153	74.8457846
18:07:55	0:07:36	71.6930524	74.3812876	72.731787	81.2395153	74.8457846
18:07:56	0:07:37	71.6930524	74.3812876	72.731787	83.2395153	73.8457846
18:07:57	0:07:38	71.6930524	75.3812876	72.731787	82.2395153	73.8457846
18:07:58	0:07:39	72.6930524	75.3812876	72.731787	82.2395153	72.8457846
18:07:59	0:07:40	72.6930524	75.3812876	72.731787	82.2395153	72.8457846
18:08:00	0:07:41	73.6930524	77.3812876	72.731787	82.2395153	69.8457846
18:08:01	0:07:42	74.6930524	81.3812876	72.731787	82.2395153	69.8457846
18:08:02	0:07:43	73.6930524	81.3812876	71.731787	83.2395153	67.8457846
18:08:03	0:07:44	73.6930524	81.3812876	71.731787	85.2395153	66.8457846
18:08:04	0:07:45	73.6930524	81.3812876	71.731787	85.2395153	65.8457846
18:08:05	0:07:46	74.6930524	80.3812876	71.731787	89.2395153	64.8457846
18:08:06	0:07:47	74.6930524	80.3812876	71.731787	89.2395153	64.8457846
18:08:07	0:07:48	74.6930524	80.3812876	70.731787	91.2395153	63.8457846
18:08:08	0:07:49	74.6930524	79.3812876	69.731787	91.2395153	64.8457846
18:08:09	0:07:50	73.6930524	79.3812876	69.731787	93.2395153	65.8457846
18:08:10	0:07:51	74.6930524	79.3812876	70.731787	93.2395153	65.8457846
18:08:11	0:07:52	76.6930524	78.3812876	71.731787	93.2395153	67.8457846
18:08:12	0:07:53	77.6930524	79.3812876	72.731787	93.2395153	71.8457846
18:08:13	0:07:54	76.6930524	79.3812876	72.731787	93.2395153	71.8457846
18:08:14	0:07:55	75.6930524	81.3812876	72.731787	93.2395153	82.8457846
18:08:15	0:07:56	74.6930524	82.3812876	72.731787	91.2395153	84.8457846
18:08:16	0:07:57	74.6930524	83.3812876	72.731787	89.2395153	85.8457846
18:08:17	0:07:58	74.6930524	83.3812876	72.731787	89.2395153	85.8457846
18:08:18	0:07:59	75.6930524	84.3812876	72.731787	89.2395153	84.8457846
18:08:19	0:08:00	76.6930524	84.3812876	73.731787	85.2395153	84.8457846
18:08:20	0:08:01	76.6930524	84.3812876	76.731787	85.2395153	84.8457846
18:08:21	0:08:02	75.6930524	79.3812876	78.731787	79.2395153	84.8457846
18:08:22	0:08:03	75.6930524	76.3812876	79.731787	76.2395153	84.8457846
18:08:23	0:08:04	75.6930524	74.3812876	79.731787	76.2395153	84.8457846
18:08:24	0:08:05	75.6930524	72.3812876	79.731787	75.2395153	84.8457846
18:08:25	0:08:06	75.6930524	72.3812876	79.731787	74.2395153	84.8457846
18:08:26	0:08:07	75.6930524	74.3812876	80.731787	74.2395153	84.8457846
18:08:27	0:08:08	75.6930524	74.3812876	81.731787	74.2395153	83.8457846

18:08:28	0:08:09	75.6930524	74.3812876	81.731787	74.2395153	82.8457846
18:08:29	0:08:10	75.6930524	75.3812876	84.731787	74.2395153	82.8457846
18:08:30	0:08:11	76.6930524	75.3812876	84.731787	73.2395153	81.8457846
18:08:31	0:08:12	76.6930524	76.3812876	84.731787	71.2395153	80.8457846
18:08:32	0:08:13	75.6930524	76.3812876	84.731787	70.2395153	80.8457846
18:08:33	0:08:14	75.6930524	74.3812876	84.731787	70.2395153	78.8457846
18:08:34	0:08:15	75.6930524	73.3812876	84.731787	70.2395153	78.8457846
18:08:35	0:08:16	74.6930524	73.3812876	80.731787	70.2395153	77.8457846
18:08:36	0:08:17	74.6930524	73.3812876	75.731787	70.2395153	76.8457846
18:08:37	0:08:18	73.6930524	73.3812876	71.731787	70.2395153	76.8457846
18:08:38	0:08:19	73.6930524	73.3812876	71.731787	70.2395153	75.8457846
18:08:39	0:08:20	73.6930524	73.3812876	70.731787	70.2395153	73.8457846
18:08:40	0:08:21	73.6930524	73.3812876	70.731787	70.2395153	72.8457846
18:08:41	0:08:22	73.6930524	75.3812876	70.731787	71.2395153	72.8457846
18:08:42	0:08:23	73.6930524	77.3812876	70.731787	72.2395153	71.8457846
18:08:43	0:08:24	74.6930524	78.3812876	70.731787	72.2395153	71.8457846
18:08:44	0:08:25	75.6930524	78.3812876	70.731787	72.2395153	70.8457846
18:08:45	0:08:26	75.6930524	78.3812876	70.731787	77.2395153	70.8457846
18:08:46	0:08:27	75.6930524	78.3812876	70.731787	78.2395153	69.8457846
18:08:47	0:08:28	75.6930524	80.3812876	70.731787	78.2395153	69.8457846
18:08:48	0:08:29	75.6930524	81.3812876	70.731787	78.2395153	67.8457846
18:08:49	0:08:30	76.6930524	81.3812876	71.731787	77.2395153	67.8457846
18:08:50	0:08:31	75.6930524	83.3812876	71.731787	76.2395153	67.8457846
18:08:51	0:08:32	75.6930524	83.3812876	71.731787	75.2395153	67.8457846
18:08:52	0:08:33	75.6930524	84.3812876	72.731787	75.2395153	67.8457846
18:08:53	0:08:34	75.6930524	84.3812876	72.731787	75.2395153	69.8457846
18:08:54	0:08:35	74.6930524	84.3812876	72.731787	74.2395153	70.8457846
18:08:55	0:08:36	73.6930524	84.3812876	72.731787	70.2395153	71.8457846
18:08:56	0:08:37	73.6930524	84.3812876	72.731787	69.2395153	71.8457846
18:08:57	0:08:38	73.6930524	85.3812876	72.731787	67.2395153	71.8457846
18:08:58	0:08:39	75.6930524	85.3812876	72.731787	65.2395153	71.8457846
18:08:59	0:08:40	75.6930524	86.3812876	72.731787	63.2395153	72.8457846
18:09:00	0:08:41	75.6930524	86.3812876	72.731787	63.2395153	72.8457846
18:09:01	0:08:42	75.6930524	86.3812876	72.731787	62.2395153	72.8457846
18:09:02	0:08:43	75.6930524	88.3812876	75.731787	62.2395153	73.8457846
18:09:03	0:08:44	76.6930524	88.3812876	83.731787	62.2395153	75.8457846
18:09:04	0:08:45	74.6930524	88.3812876	85.731787	61.2395153	75.8457846
18:09:05	0:08:46	74.6930524	83.3812876	86.731787	61.2395153	71.8457846
18:09:06	0:08:47	74.6930524	83.3812876	88.731787	60.2395153	71.8457846
18:09:07	0:08:48	75.6930524	83.3812876	87.731787	60.2395153	71.8457846
18:09:08	0:08:49	75.6930524	83.3812876	87.731787	60.2395153	70.8457846
18:09:09	0:08:50	74.6930524	82.3812876	86.731787	59.2395153	70.8457846
18:09:10	0:08:51	75.6930524	78.3812876	86.731787	59.2395153	70.8457846
18:09:11	0:08:52	75.6930524	77.3812876	86.731787	60.2395153	69.8457846
18:09:12	0:08:53	75.6930524	76.3812876	86.731787	60.2395153	69.8457846

18:09:13	0:08:54	75.6930524	75.3812876	84.731787	55.2395153	69.8457846
18:09:14	0:08:55	75.6930524	74.3812876	77.731787	54.2395153	69.8457846
18:09:15	0:08:56	75.6930524	74.3812876	72.731787	53.2395153	69.8457846
18:09:16	0:08:57	74.6930524	72.3812876	70.731787	52.2395153	69.8457846
18:09:17	0:08:58	74.6930524	72.3812876	69.731787	52.2395153	69.8457846
18:09:18	0:08:59	74.6930524	71.3812876	69.731787	52.2395153	69.8457846
18:09:19	0:09:00	73.6930524	71.3812876	69.731787	52.2395153	69.8457846
18:09:20	0:09:01	73.6930524	71.3812876	68.731787	52.2395153	69.8457846
18:09:21	0:09:02	73.6930524	71.3812876	67.731787	53.2395153	69.8457846
18:09:22	0:09:03	73.6930524	71.3812876	67.731787	54.2395153	68.8457846
18:09:23	0:09:04	73.6930524	71.3812876	68.731787	55.2395153	68.8457846
18:09:24	0:09:05	73.6930524	71.3812876	68.731787	55.2395153	68.8457846
18:09:25	0:09:06	73.6930524	73.3812876	69.731787	55.2395153	66.8457846
18:09:26	0:09:07	73.6930524	73.3812876	69.731787	55.2395153	66.8457846
18:09:27	0:09:08	73.6930524	73.3812876	69.731787	56.2395153	65.8457846
18:09:28	0:09:09	72.6930524	73.3812876	69.731787	57.2395153	64.8457846
18:09:29	0:09:10	72.6930524	73.3812876	69.731787	58.2395153	64.8457846
18:09:30	0:09:11	72.6930524	73.3812876	69.731787	57.2395153	66.8457846
18:09:31	0:09:12	73.6930524	73.3812876	68.731787	57.2395153	66.8457846
18:09:32	0:09:13	73.6930524	73.3812876	68.731787	58.2395153	66.8457846
18:09:33	0:09:14	74.6930524	73.3812876	69.731787	59.2395153	67.8457846
18:09:34	0:09:15	75.6930524	74.3812876	71.731787	59.2395153	67.8457846
18:09:35	0:09:16	75.6930524	74.3812876	71.731787	58.2395153	67.8457846
18:09:36	0:09:17	76.6930524	74.3812876	71.731787	58.2395153	67.8457846
18:09:37	0:09:18	76.6930524	75.3812876	71.731787	58.2395153	67.8457846
18:09:38	0:09:19	76.6930524	76.3812876	71.731787	56.2395153	66.8457846
18:09:39	0:09:20	76.6930524	76.3812876	71.731787	55.2395153	66.8457846
18:09:40	0:09:21	76.6930524	73.3812876	71.731787	55.2395153	64.8457846
18:09:41	0:09:22	77.6930524	73.3812876	72.731787	55.2395153	62.8457846
18:09:42	0:09:23	77.6930524	73.3812876	74.731787	56.2395153	60.8457846
18:09:43	0:09:24	78.6930524	74.3812876	74.731787	58.2395153	60.8457846
18:09:44	0:09:25	77.6930524	74.3812876	75.731787	58.2395153	60.8457846
18:09:45	0:09:26	77.6930524	74.3812876	75.731787	60.2395153	60.8457846
18:09:46	0:09:27	77.6930524	74.3812876	75.731787	64.2395153	60.8457846
18:09:47	0:09:28	77.6930524	74.3812876	75.731787	63.2395153	60.8457846
18:09:48	0:09:29	78.6930524	74.3812876	76.731787	63.2395153	60.8457846
18:09:49	0:09:30	78.6930524	74.3812876	76.731787	64.2395153	60.8457846
18:09:50	0:09:31	78.6930524	75.3812876	76.731787	65.2395153	59.8457846
18:09:51	0:09:32	78.6930524	76.3812876	77.731787	65.2395153	59.8457846
18:09:52	0:09:33	79.6930524	78.3812876	77.731787	66.2395153	59.8457846
18:09:53	0:09:34	79.6930524	78.3812876	78.731787	66.2395153	59.8457846
18:09:54	0:09:35	79.6930524	78.3812876	78.731787	66.2395153	59.8457846
18:09:55	0:09:36	77.6930524	79.3812876	79.731787	70.2395153	59.8457846
18:09:56	0:09:37	77.6930524	79.3812876	79.731787	70.2395153	61.8457846
18:09:57	0:09:38	76.6930524	79.3812876	79.731787	73.2395153	66.8457846

18:09:58	0:09:39	76.6930524	79.3812876	79.731787	72.2395153	66.8457846
18:09:59	0:09:40	76.6930524	77.3812876	77.731787	72.2395153	67.8457846
18:10:00	0:09:41	76.6930524	75.3812876	77.731787	71.2395153	70.8457846
18:10:01	0:09:42	76.6930524	73.3812876	77.731787	70.2395153	71.8457846
18:10:02	0:09:43	76.6930524	74.3812876	75.731787	69.2395153	71.8457846
18:10:03	0:09:44	75.6930524	74.3812876	75.731787	69.2395153	71.8457846
18:10:04	0:09:45	75.6930524	74.3812876	75.731787	69.2395153	71.8457846
18:10:05	0:09:46	75.6930524	74.3812876	75.731787	69.2395153	71.8457846
18:10:06	0:09:47	75.6930524	73.3812876	75.731787	69.2395153	71.8457846
18:10:07	0:09:48	75.6930524	72.3812876	75.731787	69.2395153	71.8457846
18:10:08	0:09:49	75.6930524	72.3812876	73.731787	69.2395153	68.8457846
18:10:09	0:09:50	75.6930524	72.3812876	73.731787	71.2395153	68.8457846
18:10:10	0:09:51	75.6930524	71.3812876	73.731787	74.2395153	68.8457846
18:10:11	0:09:52	75.6930524	71.3812876	74.731787	74.2395153	68.8457846
18:10:12	0:09:53	75.6930524	71.3812876	73.731787	75.2395153	67.8457846
18:10:13	0:09:54	79.6930524	71.3812876	73.731787	75.2395153	67.8457846
18:10:14	0:09:55	82.6930524	71.3812876	73.731787	74.2395153	67.8457846
18:10:15	0:09:56	85.6930524	71.3812876	73.731787	74.2395153	67.8457846
18:10:16	0:09:57	87.6930524	71.3812876	73.731787	74.2395153	68.8457846
18:10:17	0:09:58	86.6930524	71.3812876	72.731787	74.2395153	69.8457846
18:10:18	0:09:59	86.6930524	70.3812876	72.731787	75.2395153	70.8457846
18:10:19	0:10:00	85.6930524	74.3812876	72.731787	76.2395153	71.8457846
18:10:20	0:10:01	83.6930524	74.3812876	74.731787	75.2395153	72.8457846
18:10:21	0:10:02	81.6930524	74.3812876	75.731787	75.2395153	72.8457846
18:10:22	0:10:03	80.6930524	75.3812876	75.731787	75.2395153	72.8457846
18:10:23	0:10:04	80.6930524	75.3812876	76.731787	75.2395153	72.8457846
18:10:24	0:10:05	81.6930524	75.3812876	75.731787	72.2395153	72.8457846
18:10:25	0:10:06	81.6930524	75.3812876	75.731787	72.2395153	73.8457846
18:10:26	0:10:07	81.6930524	75.3812876	75.731787	72.2395153	74.8457846
18:10:27	0:10:08	79.6930524	75.3812876	76.731787	72.2395153	74.8457846
18:10:28	0:10:09	75.6930524	74.3812876	77.731787	71.2395153	74.8457846
18:10:29	0:10:10	72.6930524	74.3812876	78.731787	71.2395153	75.8457846
18:10:30	0:10:11	71.6930524	74.3812876	78.731787	71.2395153	76.8457846
18:10:31	0:10:12	70.6930524	74.3812876	79.731787	69.2395153	77.8457846
18:10:32	0:10:13	70.6930524	74.3812876	79.731787	69.2395153	77.8457846
18:10:33	0:10:14	70.6930524	74.3812876	79.731787	69.2395153	77.8457846
18:10:34	0:10:15	70.6930524	74.3812876	80.731787	69.2395153	81.8457846
18:10:35	0:10:16	70.6930524	74.3812876	79.731787	69.2395153	80.8457846
18:10:36	0:10:17	70.6930524	75.3812876	78.731787	70.2395153	78.8457846
18:10:37	0:10:18	68.6930524	75.3812876	77.731787	71.2395153	78.8457846
18:10:38	0:10:19	68.6930524	74.3812876	75.731787	72.2395153	77.8457846
18:10:39	0:10:20	67.6930524	74.3812876	74.731787	77.2395153	77.8457846
18:10:40	0:10:21	67.6930524	74.3812876	73.731787	79.2395153	78.8457846
18:10:41	0:10:22	66.6930524	75.3812876	73.731787	78.2395153	78.8457846
18:10:42	0:10:23	65.6930524	75.3812876	72.731787	78.2395153	79.8457846

18:10:43	0:10:24	65.6930524	75.3812876	71.731787	78.2395153	79.8457846
18:10:44	0:10:25	65.6930524	75.3812876	71.731787	78.2395153	79.8457846
18:10:45	0:10:26	64.6930524	74.3812876	72.731787	77.2395153	79.8457846
18:10:46	0:10:27	65.6930524	74.3812876	73.731787	76.2395153	79.8457846
18:10:47	0:10:28	65.6930524	75.3812876	72.731787	74.2395153	79.8457846
18:10:48	0:10:29	65.6930524	75.3812876	72.731787	74.2395153	79.8457846
18:10:49	0:10:30	65.6930524	74.3812876	69.731787	73.2395153	80.8457846
18:10:50	0:10:31	67.6930524	74.3812876	68.731787	73.2395153	80.8457846
18:10:51	0:10:32	67.6930524	74.3812876	67.731787	73.2395153	81.8457846
18:10:52	0:10:33	67.6930524	73.3812876	66.731787	74.2395153	81.8457846
18:10:53	0:10:34	67.6930524	72.3812876	63.731787	77.2395153	81.8457846
18:10:54	0:10:35	67.6930524	73.3812876	63.731787	77.2395153	76.8457846
18:10:55	0:10:36	64.6930524	73.3812876	62.731787	77.2395153	76.8457846
18:10:56	0:10:37	64.6930524	73.3812876	62.731787	77.2395153	76.8457846
18:10:57	0:10:38	64.6930524	73.3812876	62.731787	77.2395153	76.8457846
18:10:58	0:10:39	64.6930524	73.3812876	61.731787	77.2395153	76.8457846
18:10:59	0:10:40	64.6930524	73.3812876	61.731787	77.2395153	76.8457846
18:11:00	0:10:41	65.6930524	71.3812876	61.731787	77.2395153	76.8457846
18:11:01	0:10:42	65.6930524	69.3812876	61.731787	78.2395153	78.8457846
18:11:02	0:10:43	65.6930524	69.3812876	61.731787	78.2395153	78.8457846
18:11:03	0:10:44	69.6930524	69.3812876	62.731787	78.2395153	78.8457846
18:11:04	0:10:45	69.6930524	69.3812876	68.731787	78.2395153	78.8457846
18:11:05	0:10:46	70.6930524	70.3812876	73.731787	78.2395153	78.8457846
18:11:06	0:10:47	70.6930524	70.3812876	74.731787	78.2395153	78.8457846
18:11:07	0:10:48	69.6930524	70.3812876	75.731787	78.2395153	74.8457846
18:11:08	0:10:49	70.6930524	71.3812876	75.731787	78.2395153	73.8457846
18:11:09	0:10:50	70.6930524	72.3812876	75.731787	78.2395153	73.8457846
18:11:10	0:10:51	70.6930524	72.3812876	75.731787	77.2395153	71.8457846
18:11:11	0:10:52	70.6930524	72.3812876	75.731787	76.2395153	70.8457846
18:11:12	0:10:53	69.6930524	72.3812876	74.731787	76.2395153	70.8457846
18:11:13	0:10:54	69.6930524	71.3812876	74.731787	75.2395153	70.8457846
18:11:14	0:10:55	69.6930524	71.3812876	73.731787	75.2395153	69.8457846
18:11:15	0:10:56	69.6930524	70.3812876	72.731787	74.2395153	71.8457846
18:11:16	0:10:57	71.6930524	71.3812876	71.731787	70.2395153	71.8457846
18:11:17	0:10:58	71.6930524	71.3812876	71.731787	69.2395153	73.8457846
18:11:18	0:10:59	71.6930524	71.3812876	71.731787	69.2395153	74.8457846
18:11:19	0:11:00	71.6930524	71.3812876	71.731787	69.2395153	75.8457846
18:11:20	0:11:01	71.6930524	71.3812876	71.731787	69.2395153	76.8457846
18:11:21	0:11:02	70.6930524	71.3812876	70.731787	69.2395153	76.8457846
18:11:22	0:11:03	70.6930524	71.3812876	70.731787	69.2395153	76.8457846
18:11:23	0:11:04	71.6930524	70.3812876	70.731787	70.2395153	80.8457846
18:11:24	0:11:05	72.6930524	71.3812876	70.731787	70.2395153	80.8457846
18:11:25	0:11:06	72.6930524	71.3812876	70.731787	70.2395153	85.8457846
18:11:26	0:11:07	72.6930524	71.3812876	70.731787	70.2395153	87.8457846
18:11:27	0:11:08	71.6930524	71.3812876	72.731787	73.2395153	87.8457846

18:11:28	0:11:09	71.6930524	71.3812876	73.731787	73.2395153	88.8457846
18:11:29	0:11:10	73.6930524	71.3812876	73.731787	74.2395153	88.8457846
18:11:30	0:11:11	75.6930524	70.3812876	73.731787	74.2395153	88.8457846
18:11:31	0:11:12	76.6930524	69.3812876	73.731787	74.2395153	88.8457846
18:11:32	0:11:13	76.6930524	69.3812876	73.731787	74.2395153	88.8457846
18:11:33	0:11:14	75.6930524	68.3812876	73.731787	74.2395153	87.8457846
18:11:34	0:11:15	75.6930524	68.3812876	73.731787	74.2395153	86.8457846
18:11:35	0:11:16	75.6930524	66.3812876	74.731787	75.2395153	86.8457846
18:11:36	0:11:17	74.6930524	66.3812876	74.731787	75.2395153	86.8457846
18:11:37	0:11:18	74.6930524	66.3812876	74.731787	75.2395153	85.8457846
18:11:38	0:11:19	73.6930524	65.3812876	74.731787	75.2395153	88.8457846
18:11:39	0:11:20	74.6930524	66.3812876	75.731787	84.2395153	88.8457846
18:11:40	0:11:21	74.6930524	66.3812876	75.731787	89.2395153	86.8457846
18:11:41	0:11:22	74.6930524	67.3812876	76.731787	75.6701505	86.8457846
18:11:42	0:11:23	74.6930524	67.3812876	76.731787	75.6701505	86.8457846
18:11:43	0:11:24	75.6930524	69.3812876	74.731787	75.6701505	86.8457846
18:11:44	0:11:25	75.6930524	69.3812876	73.731787	75.4201505	86.8457846
18:11:45	0:11:26	75.6930524	71.3812876	73.731787	75.6701505	86.8457846
18:11:46	0:11:27	75.6930524	73.3812876	73.731787	92.2395153	85.8457846
18:11:47	0:11:28	75.6930524	73.3812876	73.731787	75.9201505	84.8457846
18:11:48	0:11:29	76.6930524	72.3812876	73.731787	75.9201505	84.8457846
18:11:49	0:11:30	74.6930524	72.3812876	72.731787	75.6701505	83.8457846
18:11:50	0:11:31	72.6930524	71.3812876	72.731787	75.4201505	82.8457846
18:11:51	0:11:32	72.6930524	70.3812876	72.731787	74.1701505	70.8457846
18:11:52	0:11:33	72.6930524	71.3812876	72.731787	73.6701505	65.8457846
18:11:53	0:11:34	72.6930524	71.3812876	71.731787	73.6701505	67.8457846
18:11:54	0:11:35	72.6930524	70.3812876	70.731787	73.7951505	70.8457846
18:11:55	0:11:36	72.6930524	71.3812876	71.731787	73.9201505	70.8457846
18:11:56	0:11:37	72.6930524	70.3812876	72.731787	74.2951505	71.8457846
18:11:57	0:11:38	72.6930524	70.3812876	71.731787	74.4201505	72.8457846
18:11:58	0:11:39	72.6930524	70.3812876	71.731787	74.2951505	73.8457846
18:11:59	0:11:40	71.6930524	70.3812876	71.731787	74.0451505	73.8457846
18:12:00	0:11:41	69.6930524	70.3812876	71.731787	73.9201505	73.8457846
18:12:01	0:11:42	69.6930524	69.3812876	71.731787	73.9201505	73.8457846
18:12:02	0:11:43	69.6930524	68.3812876	71.731787	73.7951505	70.8457846
18:12:03	0:11:44	74.6930524	73.3812876	71.731787	75.0451505	70.8457846
18:12:04	0:11:45	79.6930524	73.3812876	72.731787	76.1701505	70.8457846
18:12:05	0:11:46	80.6930524	74.3812876	73.731787	76.6701505	70.8457846
18:12:06	0:11:47	80.6930524	75.3812876	72.731787	76.9201505	72.8457846
18:12:07	0:11:48	86.6930524	75.3812876	71.731787	77.7951505	73.8457846
18:12:08	0:11:49	87.6930524	76.3812876	70.731787	78.0451505	73.8457846
18:12:09	0:11:50	89.6930524	84.3812876	70.731787	79.1701505	73.8457846
18:12:10	0:11:51	94.6930524	84.3812876	70.731787	79.5451505	73.8457846
18:12:11	0:11:52	95.6930524	84.3812876	71.731787	79.7951505	73.8457846
18:12:12	0:11:53	95.6930524	84.3812876	71.731787	79.7951505	72.8457846

18:12:13	0:11:54	78.6930524	84.3812876	71.731787	77.1701505	74.8457846
18:12:14	0:11:55	76.6930524	84.3812876	70.731787	83.2395153	74.8457846
18:12:15	0:11:56	75.6930524	84.3812876	70.731787	77.2395153	73.8457846
18:12:16	0:11:57	74.6930524	84.3812876	70.731787	77.2395153	72.8457846
18:12:17	0:11:58	74.6930524	84.3812876	70.731787	74.2395153	72.8457846
18:12:18	0:11:59	73.6930524	85.3812876	71.731787	73.2395153	72.8457846
18:12:19	0:12:00	73.6930524	85.3812876	71.731787	73.2395153	72.8457846
18:12:20	0:12:01	73.6930524	84.3812876	71.731787	73.2395153	72.8457846
18:12:21	0:12:02	73.6930524	84.3812876	71.731787	72.2395153	72.8457846
18:12:22	0:12:03	73.6930524	84.3812876	71.731787	73.2395153	72.8457846
18:12:23	0:12:04	72.6930524	84.3812876	71.731787	73.2395153	72.8457846
18:12:24	0:12:05	72.6930524	84.3812876	71.731787	72.2395153	73.8457846
18:12:25	0:12:06	70.6930524	84.3812876	71.731787	69.2395153	73.8457846
18:12:26	0:12:07	68.6930524	82.3812876	71.731787	69.2395153	75.8457846
18:12:27	0:12:08	67.6930524	81.3812876	71.731787	68.2395153	75.8457846
18:12:28	0:12:09	67.6930524	81.3812876	72.731787	68.2395153	75.8457846
18:12:29	0:12:10	67.6930524	77.3812876	73.731787	68.2395153	75.8457846
18:12:30	0:12:11	67.6930524	77.3812876	74.731787	68.2395153	75.8457846
18:12:31	0:12:12	68.6930524	77.3812876	74.731787	68.2395153	76.8457846
18:12:32	0:12:13	70.6930524	77.3812876	74.731787	69.2395153	76.8457846
18:12:33	0:12:14	71.6930524	78.3812876	74.731787	70.2395153	76.8457846
18:12:34	0:12:15	71.6930524	79.3812876	74.731787	70.2395153	76.8457846
18:12:35	0:12:16	71.6930524	79.3812876	75.731787	71.2395153	78.8457846
18:12:36	0:12:17	71.6930524	79.3812876	75.731787	71.2395153	78.8457846
18:12:37	0:12:18	69.6930524	79.3812876	77.731787	75.2395153	78.8457846
18:12:38	0:12:19	69.6930524	79.3812876	77.731787	75.2395153	77.8457846
18:12:39	0:12:20	69.6930524	74.3812876	77.731787	76.2395153	77.8457846
18:12:40	0:12:21	68.6930524	74.3812876	77.731787	76.2395153	77.8457846
18:12:41	0:12:22	68.6930524	74.3812876	76.731787	77.2395153	75.8457846
18:12:42	0:12:23	68.6930524	74.3812876	75.731787	77.2395153	73.8457846
18:12:43	0:12:24	68.6930524	74.3812876	75.731787	78.2395153	71.8457846
18:12:44	0:12:25	68.6930524	69.3812876	74.731787	78.2395153	70.8457846
18:12:45	0:12:26	68.6930524	69.3812876	73.731787	79.2395153	69.8457846
18:12:46	0:12:27	68.6930524	68.3812876	73.731787	80.2395153	69.8457846
18:12:47	0:12:28	68.6930524	67.3812876	71.731787	80.2395153	70.8457846
18:12:48	0:12:29	69.6930524	67.3812876	70.731787	79.2395153	70.8457846
18:12:49	0:12:30	69.6930524	70.3812876	69.731787	80.2395153	70.8457846
18:12:50	0:12:31	69.6930524	71.3812876	67.731787	80.2395153	71.8457846
18:12:51	0:12:32	70.6930524	71.3812876	65.731787	80.2395153	73.8457846
18:12:52	0:12:33	70.6930524	72.3812876	64.731787	79.2395153	73.8457846
18:12:53	0:12:34	71.6930524	72.3812876	63.731787	75.2395153	73.8457846
18:12:54	0:12:35	71.6930524	72.3812876	63.731787	72.2395153	74.8457846
18:12:55	0:12:36	71.6930524	72.3812876	63.731787	72.2395153	75.8457846
18:12:56	0:12:37	72.6930524	71.3812876	63.731787	72.2395153	75.8457846
18:12:57	0:12:38	71.6930524	71.3812876	63.731787	72.2395153	75.8457846

18:12:58	0:12:39	71.6930524	71.3812876	63.731787	59.2395153	74.8457846
18:12:59	0:12:40	71.6930524	71.3812876	63.731787	59.2395153	73.8457846
18:13:00	0:12:41	69.6930524	71.3812876	63.731787	59.2395153	73.8457846
18:13:01	0:12:42	69.6930524	69.3812876	63.731787	60.2395153	73.8457846
18:13:02	0:12:43	70.6930524	69.3812876	64.731787	61.2395153	73.8457846
18:13:03	0:12:44	71.6930524	69.3812876	66.731787	66.2395153	73.8457846
18:13:04	0:12:45	71.6930524	69.3812876	67.731787	67.2395153	73.8457846
18:13:05	0:12:46	74.6930524	69.3812876	67.731787	68.2395153	73.8457846
18:13:06	0:12:47	75.6930524	68.3812876	67.731787	68.2395153	73.8457846
18:13:07	0:12:48	75.6930524	68.3812876	66.731787	68.2395153	75.8457846
18:13:08	0:12:49	74.6930524	67.3812876	66.731787	69.2395153	78.8457846
18:13:09	0:12:50	73.6930524	67.3812876	66.731787	65.2395153	78.8457846
18:13:10	0:12:51	72.6930524	68.3812876	66.731787	64.2395153	78.8457846
18:13:11	0:12:52	72.6930524	73.3812876	66.731787	64.2395153	79.8457846
18:13:12	0:12:53	72.6930524	73.3812876	66.731787	64.2395153	80.8457846
18:13:13	0:12:54	72.6930524	73.3812876	66.731787	65.2395153	81.8457846
18:13:14	0:12:55	72.6930524	72.3812876	66.731787	65.2395153	82.8457846
18:13:15	0:12:56	73.6930524	72.3812876	66.731787	66.2395153	83.8457846
18:13:16	0:12:57	73.6930524	72.3812876	66.731787	66.2395153	83.8457846
18:13:17	0:12:58	73.6930524	72.3812876	66.731787	66.2395153	83.8457846
18:13:18	0:12:59	74.6930524	71.3812876	66.731787	66.2395153	83.8457846
18:13:19	0:13:00	73.6930524	71.3812876	66.731787	66.2395153	83.8457846
18:13:20	0:13:01	73.6930524	71.3812876	66.731787	66.2395153	83.8457846
18:13:21	0:13:02	72.6930524	71.3812876	65.731787	66.2395153	83.8457846
18:13:22	0:13:03	70.6930524	70.3812876	64.731787	64.2395153	85.8457846
18:13:23	0:13:04	69.6930524	68.3812876	67.731787	63.2395153	84.8457846
18:13:24	0:13:05	69.6930524	68.3812876	69.731787	63.2395153	84.8457846
18:13:25	0:13:06	68.6930524	68.3812876	69.731787	63.2395153	84.8457846
18:13:26	0:13:07	68.6930524	69.3812876	69.731787	65.2395153	83.8457846
18:13:27	0:13:08	67.6930524	69.3812876	70.731787	66.2395153	83.8457846
18:13:28	0:13:09	67.6930524	71.3812876	71.731787	65.2395153	82.8457846
18:13:29	0:13:10	67.6930524	71.3812876	71.731787	64.2395153	83.8457846
18:13:30	0:13:11	67.6930524	71.3812876	71.731787	64.2395153	83.8457846
18:13:31	0:13:12	68.6930524	70.3812876	72.731787	63.2395153	83.8457846
18:13:32	0:13:13	70.6930524	70.3812876	72.731787	63.2395153	82.8457846
18:13:33	0:13:14	72.6930524	71.3812876	72.731787	63.2395153	81.8457846
18:13:34	0:13:15	73.6930524	71.3812876	72.731787	63.2395153	80.8457846
18:13:35	0:13:16	73.6930524	71.3812876	73.731787	63.2395153	81.8457846
18:13:36	0:13:17	75.6930524	68.3812876	72.731787	63.2395153	82.8457846
18:13:37	0:13:18	75.6930524	68.3812876	72.731787	63.2395153	83.8457846
18:13:38	0:13:19	75.6930524	68.3812876	72.731787	62.2395153	83.8457846
18:13:39	0:13:20	73.6930524	68.3812876	71.731787	61.2395153	83.8457846
18:13:40	0:13:21	71.6930524	68.3812876	70.731787	61.2395153	83.8457846
18:13:41	0:13:22	71.6930524	69.3812876	70.731787	62.2395153	83.8457846
18:13:42	0:13:23	69.6930524	69.3812876	69.731787	63.2395153	83.8457846

18:13:43	0:13:24	67.6930524	70.3812876	69.731787	63.2395153	83.8457846
18:13:44	0:13:25	67.6930524	72.3812876	69.731787	63.2395153	83.8457846
18:13:45	0:13:26	67.6930524	72.3812876	68.731787	64.2395153	83.8457846
18:13:46	0:13:27	67.6930524	72.3812876	69.731787	65.2395153	83.8457846
18:13:47	0:13:28	66.6930524	72.3812876	69.731787	65.2395153	83.8457846
18:13:48	0:13:29	66.6930524	72.3812876	69.731787	66.2395153	78.8457846
18:13:49	0:13:30	65.6930524	73.3812876	69.731787	66.2395153	77.8457846
18:13:50	0:13:31	66.6930524	73.3812876	70.731787	67.2395153	77.8457846
18:13:51	0:13:32	67.6930524	73.3812876	71.731787	68.2395153	77.8457846
18:13:52	0:13:33	67.6930524	74.3812876	69.731787	69.2395153	79.8457846
18:13:53	0:13:34	69.6930524	76.3812876	68.731787	71.2395153	84.8457846
18:13:54	0:13:35	73.6930524	79.3812876	67.731787	76.2395153	84.8457846
18:13:55	0:13:36	75.6930524	82.3812876	67.731787	79.2395153	82.8457846
18:13:56	0:13:37	77.6930524	82.3812876	67.731787	84.2395153	80.8457846
18:13:57	0:13:38	76.6930524	83.3812876	67.731787	90.2395153	78.8457846
18:13:58	0:13:39	75.6930524	84.3812876	67.731787	74.5451505	76.8457846
18:13:59	0:13:40	74.6930524	85.3812876	66.731787	74.4201505	75.8457846
18:14:00	0:13:41	73.6930524	85.3812876	66.731787	74.1701505	75.8457846
18:14:01	0:13:42	72.6930524	85.3812876	65.731787	84.2395153	72.8457846
18:14:02	0:13:43	71.6930524	85.3812876	66.731787	82.2395153	72.8457846
18:14:03	0:13:44	70.6930524	85.3812876	66.731787	81.2395153	72.8457846
18:14:04	0:13:45	69.6930524	85.3812876	66.731787	77.2395153	72.8457846
18:14:05	0:13:46	69.6930524	85.3812876	64.731787	75.2395153	71.8457846
18:14:06	0:13:47	68.6930524	84.3812876	62.731787	75.2395153	74.8457846
18:14:07	0:13:48	68.6930524	83.3812876	61.731787	71.2395153	75.8457846
18:14:08	0:13:49	68.6930524	83.3812876	60.731787	68.2395153	76.8457846
18:14:09	0:13:50	67.6930524	83.3812876	60.731787	67.2395153	78.8457846
18:14:10	0:13:51	67.6930524	83.3812876	60.731787	66.2395153	78.8457846
18:14:11	0:13:52	68.6930524	82.3812876	61.731787	66.2395153	83.8457846
18:14:12	0:13:53	69.6930524	82.3812876	63.731787	65.2395153	84.8457846
18:14:13	0:13:54	70.6930524	79.3812876	63.731787	64.2395153	84.8457846
18:14:14	0:13:55	70.6930524	76.3812876	63.731787	63.2395153	84.8457846
18:14:15	0:13:56	70.6930524	76.3812876	63.731787	62.2395153	84.8457846
18:14:16	0:13:57	70.6930524	75.3812876	63.731787	62.2395153	84.8457846
18:14:17	0:13:58	70.6930524	74.3812876	64.731787	62.2395153	84.8457846
18:14:18	0:13:59	70.6930524	74.3812876	65.731787	62.2395153	83.8457846
18:14:19	0:14:00	70.6930524	74.3812876	66.731787	62.2395153	83.8457846
18:14:20	0:14:01	71.6930524	73.3812876	67.731787	60.2395153	82.8457846
18:14:21	0:14:02	71.6930524	73.3812876	69.731787	58.2395153	82.8457846
18:14:22	0:14:03	72.6930524	72.3812876	69.731787	57.2395153	79.8457846
18:14:23	0:14:04	72.6930524	70.3812876	69.731787	57.2395153	79.8457846
18:14:24	0:14:05	72.6930524	70.3812876	69.731787	57.2395153	79.8457846
18:14:25	0:14:06	71.6930524	70.3812876	71.731787	58.2395153	80.8457846
18:14:26	0:14:07	71.6930524	68.3812876	73.731787	58.2395153	80.8457846
18:14:27	0:14:08	71.6930524	68.3812876	73.731787	58.2395153	80.8457846

18:14:28	0:14:09	72.6930524	68.3812876	73.731787	59.2395153	80.8457846
18:14:29	0:14:10	72.6930524	67.3812876	73.731787	59.2395153	80.8457846
18:14:30	0:14:11	72.6930524	68.3812876	73.731787	60.2395153	80.8457846
18:14:31	0:14:12	72.6930524	68.3812876	73.731787	60.2395153	81.8457846
18:14:32	0:14:13	72.6930524	68.3812876	73.731787	60.2395153	81.8457846
18:14:33	0:14:14	72.6930524	66.3812876	74.731787	60.2395153	81.8457846
18:14:34	0:14:15	75.6930524	66.3812876	74.731787	61.2395153	82.8457846
18:14:35	0:14:16	81.6930524	66.3812876	77.731787	61.2395153	82.8457846
18:14:36	0:14:17	88.6930524	66.3812876	78.731787	63.2395153	82.8457846
18:14:37	0:14:18	81.6930524	66.3812876	78.731787	63.2395153	81.8457846
18:14:38	0:14:19	78.6930524	66.3812876	78.731787	63.2395153	79.8457846
18:14:39	0:14:20	75.6930524	66.3812876	78.731787	63.2395153	77.8457846
18:14:40	0:14:21	75.6930524	66.3812876	78.731787	64.2395153	75.8457846
18:14:41	0:14:22	71.6930524	66.3812876	78.731787	64.2395153	75.8457846
18:14:42	0:14:23	69.6930524	66.3812876	78.731787	65.2395153	77.8457846
18:14:43	0:14:24	68.6930524	67.3812876	79.731787	68.2395153	77.8457846
18:14:44	0:14:25	68.6930524	67.3812876	79.731787	69.2395153	78.8457846
18:14:45	0:14:26	67.6930524	67.3812876	79.731787	69.2395153	79.8457846
18:14:46	0:14:27	67.6930524	68.3812876	79.731787	68.2395153	79.8457846
18:14:47	0:14:28	67.6930524	69.3812876	78.731787	67.2395153	79.8457846
18:14:48	0:14:29	67.6930524	70.3812876	79.731787	67.2395153	80.8457846
18:14:49	0:14:30	67.6930524	71.3812876	79.731787	67.2395153	80.8457846
18:14:50	0:14:31	67.6930524	72.3812876	78.731787	66.2395153	80.8457846
18:14:51	0:14:32	68.6930524	72.3812876	73.731787	66.2395153	80.8457846
18:14:52	0:14:33	67.6930524	72.3812876	71.731787	66.2395153	79.8457846
18:14:53	0:14:34	66.6930524	73.3812876	70.731787	69.2395153	79.8457846
18:14:54	0:14:35	66.6930524	74.3812876	69.731787	71.2395153	79.8457846
18:14:55	0:14:36	66.6930524	74.3812876	68.731787	71.2395153	79.8457846
18:14:56	0:14:37	65.6930524	74.3812876	67.731787	71.2395153	79.8457846
18:14:57	0:14:38	65.6930524	74.3812876	67.731787	70.2395153	79.8457846
18:14:58	0:14:39	65.6930524	74.3812876	67.731787	70.2395153	79.8457846
18:14:59	0:14:40	65.6930524	74.3812876	67.731787	68.2395153	79.8457846
18:15:00	0:14:41	65.6930524	74.3812876	66.731787	68.2395153	79.8457846
18:15:01	0:14:42	65.6930524	75.3812876	66.731787	68.2395153	80.8457846
18:15:02	0:14:43	65.6930524	76.3812876	66.731787	68.2395153	80.8457846
18:15:03	0:14:44	64.6930524	76.3812876	69.731787	67.2395153	82.8457846
18:15:04	0:14:45	64.6930524	76.3812876	70.731787	68.2395153	83.8457846
18:15:05	0:14:46	64.6930524	75.3812876	71.731787	72.2395153	84.8457846
18:15:06	0:14:47	65.6930524	75.3812876	73.731787	76.2395153	86.8457846
18:15:07	0:14:48	64.6930524	75.3812876	74.731787	77.2395153	86.8457846
18:15:08	0:14:49	63.6930524	75.3812876	74.731787	77.2395153	86.8457846
18:15:09	0:14:50	63.6930524	75.3812876	73.731787	77.2395153	86.8457846
18:15:10	0:14:51	63.6930524	75.3812876	72.731787	77.2395153	86.8457846
18:15:11	0:14:52	64.6930524	75.3812876	73.731787	77.2395153	86.8457846
18:15:12	0:14:53	65.6930524	74.3812876	75.731787	77.2395153	86.8457846

18:15:13	0:14:54	65.6930524	74.3812876	77.731787	77.2395153	86.8457846
18:15:14	0:14:55	66.6930524	72.3812876	77.731787	77.2395153	86.8457846
18:15:15	0:14:56	67.6930524	72.3812876	77.731787	77.2395153	86.8457846
18:15:16	0:14:57	68.6930524	63.3812876	77.731787	77.2395153	86.8457846
18:15:17	0:14:58	68.6930524	64.3812876	77.731787	75.2395153	86.8457846
18:15:18	0:14:59	68.6930524	64.3812876	77.731787	75.2395153	86.8457846
18:15:19	0:15:00	68.6930524	64.3812876	78.731787	65.2395153	69.0451505
18:15:20	0:15:01	68.6930524	63.3812876	79.731787	63.2395153	68.7951505
18:15:21	0:15:02	68.6930524	63.3812876	80.731787	63.2395153	68.9201505
18:15:22	0:15:03	68.6930524	63.3812876	80.731787	61.2395153	68.6701505
18:15:23	0:15:04	70.6930524	63.3812876	80.731787	59.2395153	69.0451505
18:15:24	0:15:05	70.6930524	63.3812876	81.731787	59.2395153	69.1701505
18:15:25	0:15:06	70.6930524	63.3812876	82.731787	58.2395153	83.8457846
18:15:26	0:15:07	70.6930524	64.3812876	81.731787	59.2395153	78.8457846
18:15:27	0:15:08	70.6930524	64.3812876	81.731787	59.2395153	78.8457846
18:15:28	0:15:09	70.6930524	64.3812876	81.731787	59.2395153	78.8457846
18:15:29	0:15:10	73.6930524	65.3812876	81.731787	59.2395153	78.8457846
18:15:30	0:15:11	75.6930524	65.3812876	81.731787	59.2395153	78.8457846
18:15:31	0:15:12	75.6930524	65.3812876	81.731787	60.2395153	78.8457846
18:15:32	0:15:13	75.6930524	65.3812876	80.731787	61.2395153	78.8457846
18:15:33	0:15:14	72.6930524	65.3812876	80.731787	62.2395153	79.8457846
18:15:34	0:15:15	71.6930524	65.3812876	79.731787	62.2395153	80.8457846
18:15:35	0:15:16	70.6930524	65.3812876	79.731787	62.2395153	81.8457846
18:15:36	0:15:17	69.6930524	65.3812876	80.731787	62.2395153	81.8457846
18:15:37	0:15:18	68.6930524	66.3812876	81.731787	62.2395153	81.8457846
18:15:38	0:15:19	68.6930524	66.3812876	81.731787	63.2395153	81.8457846
18:15:39	0:15:20	69.6930524	67.3812876	81.731787	63.2395153	83.8457846
18:15:40	0:15:21	70.6930524	67.3812876	81.731787	63.2395153	84.8457846
18:15:41	0:15:22	70.6930524	66.3812876	81.731787	63.2395153	84.8457846
18:15:42	0:15:23	70.6930524	66.3812876	81.731787	63.2395153	84.8457846
18:15:43	0:15:24	70.6930524	65.3812876	82.731787	63.2395153	85.8457846
18:15:44	0:15:25	71.6930524	64.3812876	82.731787	63.2395153	85.8457846
18:15:45	0:15:26	71.6930524	64.3812876	82.731787	62.2395153	85.8457846
18:15:46	0:15:27	71.6930524	63.3812876	82.731787	60.2395153	86.8457846
18:15:47	0:15:28	71.6930524	64.3812876	81.731787	59.2395153	88.8457846
18:15:48	0:15:29	72.6930524	67.3812876	79.731787	60.2395153	84.8457846
18:15:49	0:15:30	72.6930524	67.3812876	79.731787	60.2395153	80.8457846
18:15:50	0:15:31	72.6930524	67.3812876	78.731787	60.2395153	78.8457846
18:15:51	0:15:32	71.6930524	67.3812876	78.731787	60.2395153	76.8457846
18:15:52	0:15:33	70.6930524	67.3812876	78.731787	60.2395153	78.8457846
18:15:53	0:15:34	69.6930524	66.3812876	77.731787	59.2395153	79.8457846
18:15:54	0:15:35	69.6930524	65.3812876	75.731787	59.2395153	79.8457846
18:15:55	0:15:36	68.6930524	65.3812876	72.731787	60.2395153	79.8457846
18:15:56	0:15:37	68.6930524	65.3812876	71.731787	61.2395153	78.8457846
18:15:57	0:15:38	69.6930524	66.3812876	70.731787	62.2395153	77.8457846

18:15:58	0:15:39	69.6930524	69.3812876	70.731787	64.2395153	77.8457846
18:15:59	0:15:40	70.6930524	69.3812876	70.731787	64.2395153	77.8457846
18:16:00	0:15:41	70.6930524	69.3812876	70.731787	66.2395153	77.8457846
18:16:01	0:15:42	71.6930524	69.3812876	70.731787	68.2395153	77.8457846
18:16:02	0:15:43	71.6930524	69.3812876	70.731787	69.2395153	77.8457846
18:16:03	0:15:44	71.6930524	70.3812876	70.731787	69.2395153	76.8457846
18:16:04	0:15:45	70.6930524	70.3812876	70.731787	68.2395153	75.8457846
18:16:05	0:15:46	69.6930524	70.3812876	70.731787	69.2395153	75.8457846
18:16:06	0:15:47	69.6930524	70.3812876	70.731787	68.2395153	75.8457846
18:16:07	0:15:48	69.6930524	70.3812876	70.731787	68.2395153	75.8457846
18:16:08	0:15:49	68.6930524	70.3812876	70.731787	67.2395153	76.8457846
18:16:09	0:15:50	69.6930524	70.3812876	70.731787	66.2395153	78.8457846
18:16:10	0:15:51	70.6930524	71.3812876	70.731787	65.2395153	80.8457846
18:16:11	0:15:52	70.6930524	71.3812876	71.731787	65.2395153	82.8457846
18:16:12	0:15:53	70.6930524	71.3812876	71.731787	65.2395153	82.8457846
18:16:13	0:15:54	72.6930524	71.3812876	71.731787	61.2395153	84.8457846
18:16:14	0:15:55	72.6930524	71.3812876	71.731787	60.2395153	84.8457846
18:16:15	0:15:56	72.6930524	72.3812876	72.731787	60.2395153	82.8457846
18:16:16	0:15:57	72.6930524	72.3812876	73.731787	60.2395153	81.8457846
18:16:17	0:15:58	72.6930524	73.3812876	73.731787	58.2395153	81.8457846
18:16:18	0:15:59	71.6930524	73.3812876	73.731787	58.2395153	81.8457846
18:16:19	0:16:00	70.6930524	73.3812876	74.731787	58.2395153	81.8457846
18:16:20	0:16:01	69.6930524	75.3812876	74.731787	58.2395153	81.8457846
18:16:21	0:16:02	69.6930524	75.3812876	74.731787	57.2395153	81.8457846
18:16:22	0:16:03	69.6930524	74.3812876	74.731787	56.2395153	81.8457846
18:16:23	0:16:04	68.6930524	73.3812876	73.731787	56.2395153	81.8457846
18:16:24	0:16:05	68.6930524	71.3812876	72.731787	56.2395153	81.8457846
18:16:25	0:16:06	68.6930524	71.3812876	72.731787	55.2395153	81.8457846
18:16:26	0:16:07	68.6930524	70.3812876	70.731787	55.2395153	80.8457846
18:16:27	0:16:08	68.6930524	69.3812876	70.731787	57.2395153	81.8457846
18:16:28	0:16:09	68.6930524	69.3812876	68.731787	57.2395153	81.8457846
18:16:29	0:16:10	68.6930524	69.3812876	67.731787	59.2395153	70.0451505
18:16:30	0:16:11	68.6930524	68.3812876	67.731787	61.2395153	70.0451505
18:16:31	0:16:12	67.6930524	68.3812876	67.731787	61.2395153	85.8457846
18:16:32	0:16:13	67.6930524	68.3812876	67.731787	65.2395153	84.8457846
18:16:33	0:16:14	67.6930524	68.3812876	67.731787	66.2395153	84.8457846
18:16:34	0:16:15	67.6930524	69.3812876	67.731787	66.2395153	84.8457846
18:16:35	0:16:16	67.6930524	69.3812876	68.731787	68.2395153	83.8457846
18:16:36	0:16:17	67.6930524	71.3812876	68.731787	69.2395153	85.8457846
18:16:37	0:16:18	67.6930524	71.3812876	68.731787	69.2395153	84.8457846
18:16:38	0:16:19	68.6930524	71.3812876	69.731787	70.2395153	84.8457846
18:16:39	0:16:20	69.6930524	71.3812876	68.731787	70.2395153	84.8457846
18:16:40	0:16:21	69.6930524	72.3812876	68.731787	70.2395153	84.8457846
18:16:41	0:16:22	68.6930524	72.3812876	68.731787	71.2395153	84.8457846
18:16:42	0:16:23	68.6930524	72.3812876	68.731787	71.2395153	84.8457846

18:16:43	0:16:24	68.6930524	72.3812876	68.731787	72.2395153	81.8457846
18:16:44	0:16:25	67.6930524	72.3812876	69.731787	73.2395153	79.8457846
18:16:45	0:16:26	67.6930524	71.3812876	70.731787	73.2395153	79.8457846
18:16:46	0:16:27	67.6930524	71.3812876	69.731787	74.2395153	78.8457846
18:16:47	0:16:28	67.6930524	71.3812876	69.731787	74.2395153	79.8457846
18:16:48	0:16:29	67.6930524	71.3812876	69.731787	75.2395153	79.8457846
18:16:49	0:16:30	67.6930524	71.3812876	69.731787	75.2395153	79.8457846
18:16:50	0:16:31	67.6930524	68.3812876	69.731787	75.2395153	80.8457846
18:16:51	0:16:32	69.6930524	68.3812876	68.731787	75.2395153	80.8457846
18:16:52	0:16:33	70.6930524	67.3812876	68.731787	75.2395153	79.8457846
18:16:53	0:16:34	72.6930524	67.3812876	67.731787	75.2395153	78.8457846
18:16:54	0:16:35	72.6930524	68.3812876	67.731787	75.2395153	77.8457846
18:16:55	0:16:36	72.6930524	69.3812876	67.731787	76.2395153	77.8457846
18:16:56	0:16:37	72.6930524	70.3812876	64.731787	76.2395153	78.8457846
18:16:57	0:16:38	72.6930524	70.3812876	62.731787	77.2395153	79.8457846
18:16:58	0:16:39	71.6930524	69.3812876	62.731787	77.2395153	81.8457846
18:16:59	0:16:40	71.6930524	69.3812876	63.731787	78.2395153	83.8457846
18:17:00	0:16:41	70.6930524	69.3812876	64.731787	81.2395153	84.8457846
18:17:01	0:16:42	69.6930524	70.3812876	64.731787	83.2395153	84.8457846
18:17:02	0:16:43	68.6930524	70.3812876	68.731787	83.2395153	85.8457846
18:17:03	0:16:44	67.6930524	70.3812876	68.731787	84.2395153	85.8457846
18:17:04	0:16:45	67.6930524	71.3812876	68.731787	84.2395153	86.8457846
18:17:05	0:16:46	67.6930524	72.3812876	69.731787	86.2395153	86.8457846
18:17:06	0:16:47	67.6930524	72.3812876	69.731787	86.2395153	86.8457846
18:17:07	0:16:48	68.6930524	73.3812876	71.731787	86.2395153	86.8457846
18:17:08	0:16:49	69.6930524	74.3812876	72.731787	85.2395153	86.8457846
18:17:09	0:16:50	69.6930524	75.3812876	73.731787	85.2395153	86.8457846
18:17:10	0:16:51	70.6930524	75.3812876	74.731787	86.2395153	86.8457846
18:17:11	0:16:52	70.6930524	75.3812876	74.731787	86.2395153	85.8457846
18:17:12	0:16:53	70.6930524	75.3812876	74.731787	86.2395153	82.8457846
18:17:13	0:16:54	70.6930524	75.3812876	80.731787	85.2395153	80.8457846
18:17:14	0:16:55	70.6930524	75.3812876	80.731787	85.2395153	79.8457846
18:17:15	0:16:56	69.6930524	73.3812876	79.731787	84.2395153	76.8457846
18:17:16	0:16:57	68.6930524	73.3812876	77.731787	83.2395153	75.8457846
18:17:17	0:16:58	68.6930524	73.3812876	74.731787	83.2395153	75.8457846
18:17:18	0:16:59	68.6930524	73.3812876	71.731787	84.2395153	82.8457846
18:17:19	0:17:00	67.6930524	73.3812876	69.731787	85.2395153	84.8457846
18:17:20	0:17:01	67.6930524	73.3812876	69.731787	86.2395153	84.8457846
18:17:21	0:17:02	67.6930524	78.3812876	69.731787	87.2395153	84.8457846
18:17:22	0:17:03	67.6930524	79.3812876	69.731787	85.2395153	85.8457846
18:17:23	0:17:04	67.6930524	79.3812876	68.731787	74.2395153	85.8457846
18:17:24	0:17:05	68.6930524	81.3812876	68.731787	69.2395153	86.8457846
18:17:25	0:17:06	68.6930524	81.3812876	67.731787	69.2395153	85.8457846
18:17:26	0:17:07	69.6930524	79.3812876	65.731787	70.2395153	84.8457846
18:17:27	0:17:08	69.6930524	79.3812876	65.731787	71.2395153	83.8457846

18:17:28	0:17:09	70.6930524	74.3812876	64.731787	71.2395153	83.8457846
18:17:29	0:17:10	70.6930524	71.3812876	66.731787	71.2395153	82.8457846
18:17:30	0:17:11	69.6930524	71.3812876	67.731787	71.2395153	82.8457846
18:17:31	0:17:12	68.6930524	68.3812876	67.731787	72.2395153	83.8457846
18:17:32	0:17:13	68.6930524	68.3812876	68.731787	73.2395153	84.8457846
18:17:33	0:17:14	67.6930524	67.3812876	68.731787	73.2395153	84.8457846
18:17:34	0:17:15	66.6930524	67.3812876	69.731787	74.2395153	85.8457846
18:17:35	0:17:16	66.6930524	67.3812876	70.731787	76.2395153	86.8457846
18:17:36	0:17:17	65.6930524	67.3812876	71.731787	77.2395153	86.8457846
18:17:37	0:17:18	65.6930524	66.3812876	71.731787	78.2395153	86.8457846
18:17:38	0:17:19	65.6930524	64.3812876	71.731787	79.2395153	86.8457846
18:17:39	0:17:20	65.6930524	64.3812876	71.731787	81.2395153	86.8457846
18:17:40	0:17:21	66.6930524	64.3812876	71.731787	84.2395153	87.8457846
18:17:41	0:17:22	66.6930524	64.3812876	71.731787	84.2395153	86.8457846
18:17:42	0:17:23	67.6930524	63.3812876	72.731787	86.2395153	86.8457846
18:17:43	0:17:24	67.6930524	63.3812876	72.731787	79.2395153	86.8457846
18:17:44	0:17:25	67.6930524	63.3812876	71.731787	78.2395153	86.8457846
18:17:45	0:17:26	67.6930524	66.3812876	71.731787	76.2395153	84.8457846
18:17:46	0:17:27	66.6930524	66.3812876	71.731787	76.2395153	83.8457846
18:17:47	0:17:28	66.6930524	66.3812876	70.731787	74.2395153	83.8457846
18:17:48	0:17:29	65.6930524	67.3812876	69.731787	72.2395153	83.8457846
18:17:49	0:17:30	65.6930524	69.3812876	67.731787	71.2395153	83.8457846
18:17:50	0:17:31	65.6930524	69.3812876	66.731787	69.2395153	83.8457846
18:17:51	0:17:32	65.6930524	72.3812876	66.731787	68.2395153	83.8457846
18:17:52	0:17:33	65.6930524	73.3812876	65.731787	68.2395153	83.8457846
18:17:53	0:17:34	65.6930524	73.3812876	65.731787	68.2395153	83.8457846
18:17:54	0:17:35	65.6930524	75.3812876	62.731787	68.2395153	82.8457846
18:17:55	0:17:36	65.6930524	77.3812876	61.731787	67.2395153	81.8457846
18:17:56	0:17:37	64.6930524	77.3812876	61.731787	69.2395153	81.8457846
18:17:57	0:17:38	63.6930524	77.3812876	61.731787	70.2395153	81.8457846
18:17:58	0:17:39	63.6930524	76.3812876	63.731787	70.2395153	81.8457846
18:17:59	0:17:40	63.6930524	72.3812876	64.731787	71.2395153	81.8457846
18:18:00	0:17:41	63.6930524	72.3812876	64.731787	71.2395153	81.8457846
18:18:01	0:17:42	63.6930524	72.3812876	64.731787	72.2395153	78.8457846
18:18:02	0:17:43	63.6930524	70.3812876	64.731787	72.2395153	78.8457846
18:18:03	0:17:44	62.6930524	70.3812876	64.731787	74.2395153	78.8457846
18:18:04	0:17:45	62.6930524	70.3812876	64.731787	75.2395153	78.8457846
18:18:05	0:17:46	61.6930524	70.3812876	67.731787	75.2395153	81.8457846
18:18:06	0:17:47	61.6930524	70.3812876	74.731787	75.2395153	81.8457846
18:18:07	0:17:48	61.6930524	70.3812876	75.731787	75.2395153	84.8457846
18:18:08	0:17:49	62.6930524	69.3812876	76.731787	75.2395153	85.8457846
18:18:09	0:17:50	63.6930524	69.3812876	77.731787	75.2395153	85.8457846
18:18:10	0:17:51	64.6930524	69.3812876	76.731787	74.2395153	85.8457846
18:18:11	0:17:52	64.6930524	69.3812876	75.731787	74.2395153	85.8457846
18:18:12	0:17:53	65.6930524	68.3812876	75.731787	74.2395153	85.8457846

18:18:13	0:17:54	65.6930524	69.3812876	75.731787	73.2395153	85.8457846
18:18:14	0:17:55	65.6930524	69.3812876	75.731787	73.2395153	82.8457846
18:18:15	0:17:56	65.6930524	69.3812876	75.731787	73.2395153	79.8457846
18:18:16	0:17:57	66.6930524	69.3812876	75.731787	74.2395153	76.8457846
18:18:17	0:17:58	68.6930524	68.3812876	74.731787	74.2395153	73.8457846
18:18:18	0:17:59	69.6930524	68.3812876	74.731787	74.2395153	70.8457846
18:18:19	0:18:00	71.6930524	67.3812876	74.731787	74.2395153	67.8457846
18:18:20	0:18:01	72.6930524	67.3812876	74.731787	74.2395153	66.8457846
18:18:21	0:18:02	74.6930524	66.3812876	73.731787	74.2395153	67.8457846
18:18:22	0:18:03	74.6930524	66.3812876	73.731787	74.2395153	67.8457846
18:18:23	0:18:04	74.6930524	66.3812876	72.731787	76.2395153	67.8457846
18:18:24	0:18:05	72.6930524	66.3812876	72.731787	77.2395153	67.8457846
18:18:25	0:18:06	71.6930524	66.3812876	73.731787	80.2395153	67.8457846
18:18:26	0:18:07	70.6930524	66.3812876	73.731787	86.2395153	67.8457846
18:18:27	0:18:08	69.6930524	65.3812876	72.731787	88.2395153	67.8457846
18:18:28	0:18:09	70.6930524	65.3812876	72.731787	89.2395153	67.8457846
18:18:29	0:18:10	71.6930524	64.3812876	72.731787	89.2395153	68.8457846
18:18:30	0:18:11	71.6930524	64.3812876	72.731787	73.4201505	69.8457846
18:18:31	0:18:12	71.6930524	63.3812876	72.731787	73.2951505	69.8457846
18:18:32	0:18:13	71.6930524	63.3812876	72.731787	73.4201505	70.8457846
18:18:33	0:18:14	71.6930524	63.3812876	72.731787	73.9201505	73.8457846
18:18:34	0:18:15	70.6930524	66.3812876	72.731787	74.1701505	73.8457846
18:18:35	0:18:16	70.6930524	67.3812876	72.731787	74.2951505	73.8457846
18:18:36	0:18:17	69.6930524	67.3812876	70.731787	74.1701505	74.8457846
18:18:37	0:18:18	69.6930524	67.3812876	70.731787	74.5451505	74.8457846
18:18:38	0:18:19	68.6930524	69.3812876	70.731787	74.7951505	74.8457846
18:18:39	0:18:20	67.6930524	69.3812876	70.731787	74.7951505	75.8457846
18:18:40	0:18:21	66.6930524	74.3812876	69.731787	90.2395153	75.8457846
18:18:41	0:18:22	66.6930524	77.3812876	69.731787	89.2395153	76.8457846
18:18:42	0:18:23	66.6930524	78.3812876	70.731787	88.2395153	77.8457846
18:18:43	0:18:24	66.6930524	78.3812876	70.731787	88.2395153	77.8457846
18:18:44	0:18:25	66.6930524	77.3812876	71.731787	87.2395153	77.8457846
18:18:45	0:18:26	67.6930524	77.3812876	72.731787	87.2395153	77.8457846
18:18:46	0:18:27	68.6930524	77.3812876	72.731787	87.2395153	76.8457846
18:18:47	0:18:28	69.6930524	78.3812876	73.731787	87.2395153	76.8457846
18:18:48	0:18:29	69.6930524	79.3812876	75.731787	87.2395153	76.8457846
18:18:49	0:18:30	69.6930524	79.3812876	75.731787	87.2395153	75.8457846
18:18:50	0:18:31	69.6930524	81.3812876	75.731787	87.2395153	75.8457846
18:18:51	0:18:32	69.6930524	82.3812876	75.731787	87.2395153	75.8457846
18:18:52	0:18:33	69.6930524	83.3812876	75.731787	84.2395153	74.8457846
18:18:53	0:18:34	69.6930524	83.3812876	73.731787	80.2395153	74.8457846
18:18:54	0:18:35	69.6930524	83.3812876	73.731787	80.2395153	74.8457846
18:18:55	0:18:36	69.6930524	83.3812876	69.731787	76.2395153	72.8457846
18:18:56	0:18:37	69.6930524	75.3812876	69.731787	75.2395153	72.8457846
18:18:57	0:18:38	69.6930524	74.3812876	69.731787	74.2395153	72.8457846

18:18:58	0:18:39	70.6930524	73.3812876	68.731787	73.2395153	73.8457846
18:18:59	0:18:40	70.6930524	73.3812876	68.731787	72.2395153	72.8457846
18:19:00	0:18:41	70.6930524	72.3812876	68.731787	69.2395153	72.8457846
18:19:01	0:18:42	71.6930524	72.3812876	68.731787	68.2395153	72.8457846
18:19:02	0:18:43	74.6930524	72.3812876	68.731787	68.2395153	72.8457846
18:19:03	0:18:44	75.6930524	72.3812876	71.731787	68.2395153	72.8457846
18:19:04	0:18:45	75.6930524	72.3812876	71.731787	68.2395153	71.8457846
18:19:05	0:18:46	74.6930524	72.3812876	71.731787	68.2395153	70.8457846
18:19:06	0:18:47	74.6930524	72.3812876	71.731787	68.2395153	69.8457846
18:19:07	0:18:48	73.6930524	72.3812876	71.731787	68.2395153	70.8457846
18:19:08	0:18:49	73.6930524	73.3812876	72.731787	69.2395153	70.8457846
18:19:09	0:18:50	73.6930524	74.3812876	72.731787	70.2395153	70.8457846
18:19:10	0:18:51	74.6930524	74.3812876	72.731787	71.2395153	70.8457846
18:19:11	0:18:52	73.6930524	74.3812876	72.731787	74.2395153	70.8457846
18:19:12	0:18:53	73.6930524	74.3812876	73.731787	76.2395153	70.8457846
18:19:13	0:18:54	72.6930524	75.3812876	73.731787	77.2395153	69.8457846
18:19:14	0:18:55	72.6930524	75.3812876	73.731787	78.2395153	69.8457846
18:19:15	0:18:56	75.6930524	76.3812876	73.731787	80.2395153	70.8457846
18:19:16	0:18:57	76.6930524	77.3812876	72.731787	80.2395153	72.8457846
18:19:17	0:18:58	73.6930524	77.3812876	72.731787	80.2395153	74.8457846
18:19:18	0:18:59	73.6930524	77.3812876	72.731787	80.2395153	73.8457846
18:19:19	0:19:00	72.6930524	77.3812876	72.731787	80.2395153	72.8457846
18:19:20	0:19:01	70.6930524	78.3812876	72.731787	80.2395153	70.8457846
18:19:21	0:19:02	69.6930524	79.3812876	71.731787	78.2395153	69.8457846
18:19:22	0:19:03	68.6930524	79.3812876	71.731787	77.2395153	68.8457846
18:19:23	0:19:04	67.6930524	79.3812876	71.731787	77.2395153	65.8457846
18:19:24	0:19:05	66.6930524	80.3812876	71.731787	77.2395153	64.8457846
18:19:25	0:19:06	66.6930524	80.3812876	71.731787	78.2395153	64.8457846
18:19:26	0:19:07	66.6930524	80.3812876	71.731787	78.2395153	65.8457846
18:19:27	0:19:08	67.6930524	82.3812876	72.731787	79.2395153	65.8457846
18:19:28	0:19:09	68.6930524	82.3812876	72.731787	80.2395153	63.8457846
18:19:29	0:19:10	70.6930524	82.3812876	73.731787	81.2395153	61.8457846
18:19:30	0:19:11	71.6930524	83.3812876	73.731787	82.2395153	61.8457846
18:19:31	0:19:12	71.6930524	85.3812876	73.731787	82.2395153	61.8457846
18:19:32	0:19:13	71.6930524	86.3812876	74.731787	82.2395153	61.8457846
18:19:33	0:19:14	69.6930524	87.3812876	74.731787	81.2395153	61.8457846
18:19:34	0:19:15	68.6930524	87.3812876	74.731787	81.2395153	60.8457846
18:19:35	0:19:16	67.6930524	87.3812876	74.731787	80.2395153	61.8457846
18:19:36	0:19:17	67.6930524	87.3812876	74.731787	80.2395153	63.8457846
18:19:37	0:19:18	67.6930524	87.3812876	74.731787	80.2395153	64.8457846
18:19:38	0:19:19	67.6930524	87.3812876	74.731787	83.2395153	65.8457846
18:19:39	0:19:20	67.6930524	87.3812876	73.731787	86.2395153	65.8457846
18:19:40	0:19:21	67.6930524	86.3812876	73.731787	75.7951505	65.8457846
18:19:41	0:19:22	66.6930524	87.3812876	73.731787	75.9201505	65.8457846
18:19:42	0:19:23	70.6930524	87.3812876	73.731787	76.7951505	66.8457846

18:19:43	0:19:24	71.6930524	87.3812876	74.731787	77.1701505	66.8457846
18:19:44	0:19:25	71.6930524	86.3812876	73.731787	77.0451505	67.8457846
18:19:45	0:19:26	70.6930524	86.3812876	73.731787	77.0451505	68.8457846
18:19:46	0:19:27	71.6930524	86.3812876	74.731787	77.2951505	68.8457846
18:19:47	0:19:28	73.6930524	86.3812876	74.731787	77.7951505	68.8457846
18:19:48	0:19:29	73.6930524	86.3812876	74.731787	77.7951505	68.8457846
18:19:49	0:19:30	73.6930524	86.3812876	75.731787	77.9201505	68.8457846
18:19:50	0:19:31	73.6930524	86.3812876	74.731787	85.2395153	68.8457846
18:19:51	0:19:32	72.6930524	86.3812876	74.731787	73.2395153	70.8457846
18:19:52	0:19:33	71.6930524	87.3812876	74.731787	73.2395153	73.8457846
18:19:53	0:19:34	70.6930524	89.3812876	75.731787	72.2395153	75.8457846
18:19:54	0:19:35	69.6930524	89.3812876	75.731787	72.2395153	76.8457846
18:19:55	0:19:36	68.6930524	90.3812876	74.731787	68.2395153	75.8457846
18:19:56	0:19:37	68.6930524	75.0451505	74.731787	68.2395153	72.8457846
18:19:57	0:19:38	68.6930524	74.9201505	74.731787	67.2395153	71.8457846
18:19:58	0:19:39	69.6930524	74.9201505	74.731787	66.2395153	70.8457846
18:19:59	0:19:40	69.6930524	75.0451505	74.731787	65.2395153	72.8457846
18:20:00	0:19:41	68.6930524	73.2951505	74.731787	65.2395153	72.8457846
18:20:01	0:19:42	69.6930524	73.2951505	74.731787	65.2395153	69.8457846
18:20:02	0:19:43	69.6930524	71.7951505	74.731787	65.2395153	67.8457846
18:20:03	0:19:44	68.6930524	71.6701505	75.731787	66.2395153	65.8457846
18:20:04	0:19:45	67.6930524	72.4201505	77.731787	69.2395153	64.8457846
18:20:05	0:19:46	67.6930524	72.7951505	79.731787	69.2395153	64.8457846
18:20:06	0:19:47	67.6930524	83.3812876	79.731787	71.2395153	65.8457846
18:20:07	0:19:48	67.6930524	77.3812876	78.731787	72.2395153	67.8457846
18:20:08	0:19:49	67.6930524	77.3812876	74.731787	74.2395153	68.8457846
18:20:09	0:19:50	68.6930524	77.3812876	73.731787	78.2395153	69.8457846
18:20:10	0:19:51	69.6930524	77.3812876	73.731787	78.2395153	69.8457846
18:20:11	0:19:52	68.6930524	77.3812876	72.731787	79.2395153	69.8457846
18:20:12	0:19:53	68.6930524	76.3812876	71.731787	80.2395153	69.8457846
18:20:13	0:19:54	67.6930524	76.3812876	71.731787	82.2395153	70.8457846
18:20:14	0:19:55	66.6930524	68.3812876	71.731787	82.2395153	70.8457846
18:20:15	0:19:56	64.6930524	68.3812876	71.731787	82.2395153	67.8457846
18:20:16	0:19:57	63.6930524	68.3812876	71.731787	82.2395153	65.8457846
18:20:17	0:19:58	63.6930524	67.3812876	71.731787	82.2395153	64.8457846
18:20:18	0:19:59	62.6930524	65.3812876	71.731787	82.2395153	64.8457846
18:20:19	0:20:00	61.6930524	62.3812876	70.731787	81.2395153	64.8457846
18:20:20	0:20:01	62.6930524	62.3812876	70.731787	81.2395153	67.8457846
18:20:21	0:20:02	62.6930524	61.3812876	70.731787	81.2395153	68.8457846
18:20:22	0:20:03	62.6930524	61.3812876	70.731787	81.2395153	68.8457846
18:20:23	0:20:04	63.6930524	56.3812876	70.731787	81.2395153	66.8457846
18:20:24	0:20:05	64.6930524	54.3812876	71.731787	81.2395153	63.8457846
18:20:25	0:20:06	64.6930524	51.3812876	71.731787	81.2395153	62.8457846
18:20:26	0:20:07	64.6930524	51.3812876	71.731787	82.2395153	62.8457846
18:20:27	0:20:08	64.6930524	51.3812876	71.731787	82.2395153	63.8457846

18:20:28	0:20:09	64.6930524	59.3812876	71.731787	83.2395153	63.8457846
18:20:29	0:20:10	64.6930524	64.3812876	71.731787	84.2395153	64.8457846
18:20:30	0:20:11	64.6930524	64.3812876	71.731787	84.2395153	64.8457846
18:20:31	0:20:12	65.6930524	64.3812876	71.731787	85.2395153	64.8457846
18:20:32	0:20:13	65.6930524	64.3812876	72.731787	85.2395153	64.8457846
18:20:33	0:20:14	66.6930524	66.3812876	72.731787	82.2395153	64.8457846
18:20:34	0:20:15	67.6930524	68.3812876	73.731787	78.2395153	64.8457846
18:20:35	0:20:16	67.6930524	68.3812876	73.731787	73.2395153	64.8457846
18:20:36	0:20:17	68.6930524	68.3812876	73.731787	73.2395153	63.8457846
18:20:37	0:20:18	69.6930524	69.3812876	73.731787	72.2395153	63.8457846
18:20:38	0:20:19	70.6930524	82.3812876	73.731787	72.2395153	63.8457846
18:20:39	0:20:20	70.6930524	82.3812876	73.731787	71.2395153	64.8457846
18:20:40	0:20:21	73.6930524	83.3812876	73.731787	71.2395153	65.8457846
18:20:41	0:20:22	69.6930524	83.3812876	73.731787	69.2395153	65.8457846
18:20:42	0:20:23	69.6930524	83.3812876	73.731787	69.2395153	65.8457846
18:20:43	0:20:24	68.6930524	83.3812876	73.731787	67.2395153	68.8457846
18:20:44	0:20:25	68.6930524	83.3812876	72.731787	67.2395153	63.8457846
18:20:45	0:20:26	69.6930524	80.3812876	71.731787	66.2395153	61.8457846
18:20:46	0:20:27	71.6930524	79.3812876	72.731787	65.2395153	60.8457846
18:20:47	0:20:28	71.6930524	79.3812876	72.731787	65.2395153	60.8457846
18:20:48	0:20:29	72.6930524	79.3812876	71.731787	65.2395153	61.8457846
18:20:49	0:20:30	73.6930524	78.3812876	71.731787	64.2395153	61.8457846
18:20:50	0:20:31	73.6930524	77.3812876	71.731787	64.2395153	63.8457846
18:20:51	0:20:32	73.6930524	77.3812876	71.731787	63.2395153	64.8457846
18:20:52	0:20:33	73.6930524	76.3812876	70.731787	63.2395153	65.8457846
18:20:53	0:20:34	73.6930524	76.3812876	71.731787	63.2395153	64.8457846
18:20:54	0:20:35	73.6930524	72.3812876	71.731787	63.2395153	64.8457846
18:20:55	0:20:36	73.6930524	71.3812876	71.731787	63.2395153	64.8457846
18:20:56	0:20:37	73.6930524	71.3812876	71.731787	63.2395153	64.8457846
18:20:57	0:20:38	73.6930524	71.3812876	71.731787	62.2395153	63.8457846
18:20:58	0:20:39	73.6930524	71.3812876	71.731787	62.2395153	63.8457846
18:20:59	0:20:40	73.6930524	71.3812876	71.731787	62.2395153	63.8457846
18:21:00	0:20:41	73.6930524	69.3812876	71.731787	64.2395153	64.8457846
18:21:01	0:20:42	73.6930524	69.3812876	71.731787	66.2395153	64.8457846
18:21:02	0:20:43	74.6930524	68.3812876	71.731787	67.2395153	65.8457846
18:21:03	0:20:44	74.6930524	67.3812876	70.731787	67.2395153	65.8457846
18:21:04	0:20:45	74.6930524	66.3812876	69.731787	67.2395153	65.8457846
18:21:05	0:20:46	74.6930524	66.3812876	67.731787	66.2395153	65.8457846
18:21:06	0:20:47	74.6930524	66.3812876	67.731787	65.2395153	64.8457846
18:21:07	0:20:48	73.6930524	66.3812876	67.731787	65.2395153	65.8457846
18:21:08	0:20:49	73.6930524	65.3812876	67.731787	65.2395153	65.8457846
18:21:09	0:20:50	73.6930524	64.3812876	67.731787	67.2395153	65.8457846
18:21:10	0:20:51	74.6930524	64.3812876	67.731787	70.2395153	66.8457846
18:21:11	0:20:52	74.6930524	63.3812876	67.731787	73.2395153	66.8457846
18:21:12	0:20:53	74.6930524	63.3812876	67.731787	73.2395153	66.8457846

18:21:13	0:20:54	71.6930524	63.3812876	68.731787	73.2395153	65.8457846
18:21:14	0:20:55	70.6930524	63.3812876	68.731787	73.2395153	65.8457846
18:21:15	0:20:56	70.6930524	64.3812876	68.731787	73.2395153	65.8457846
18:21:16	0:20:57	70.6930524	64.3812876	68.731787	74.2395153	66.8457846
18:21:17	0:20:58	71.6930524	67.3812876	68.731787	74.2395153	66.8457846
18:21:18	0:20:59	71.6930524	68.3812876	68.731787	73.2395153	66.8457846
18:21:19	0:21:00	71.6930524	68.3812876	68.731787	72.2395153	66.8457846
18:21:20	0:21:01	71.6930524	68.3812876	67.731787	73.2395153	66.8457846
18:21:21	0:21:02	71.6930524	68.3812876	67.731787	73.2395153	66.8457846
18:21:22	0:21:03	68.6930524	68.3812876	68.731787	74.2395153	66.8457846
18:21:23	0:21:04	67.6930524	69.3812876	68.731787	75.2395153	67.8457846
18:21:24	0:21:05	67.6930524	70.3812876	69.731787	76.2395153	67.8457846
18:21:25	0:21:06	67.6930524	71.3812876	69.731787	76.2395153	67.8457846
18:21:26	0:21:07	67.6930524	73.3812876	69.731787	76.2395153	67.8457846
18:21:27	0:21:08	67.6930524	73.3812876	69.731787	77.2395153	67.8457846
18:21:28	0:21:09	67.6930524	75.3812876	70.731787	77.2395153	67.8457846
18:21:29	0:21:10	67.6930524	76.3812876	70.731787	77.2395153	67.8457846
18:21:30	0:21:11	67.6930524	76.3812876	70.731787	76.2395153	67.8457846
18:21:31	0:21:12	67.6930524	77.3812876	70.731787	76.2395153	68.8457846
18:21:32	0:21:13	67.6930524	76.3812876	70.731787	73.2395153	68.8457846
18:21:33	0:21:14	67.6930524	76.3812876	69.731787	72.2395153	68.8457846
18:21:34	0:21:15	69.6930524	76.3812876	69.731787	71.2395153	67.8457846
18:21:35	0:21:16	70.6930524	76.3812876	69.731787	69.2395153	67.8457846
18:21:36	0:21:17	70.6930524	76.3812876	69.731787	69.2395153	67.8457846
18:21:37	0:21:18	69.6930524	74.3812876	68.731787	62.2395153	66.8457846
18:21:38	0:21:19	69.6930524	74.3812876	68.731787	61.2395153	66.8457846
18:21:39	0:21:20	68.6930524	74.3812876	68.731787	58.2395153	66.8457846
18:21:40	0:21:21	68.6930524	73.3812876	68.731787	56.2395153	66.8457846
18:21:41	0:21:22	67.6930524	73.3812876	67.731787	55.2395153	66.8457846
18:21:42	0:21:23	67.6930524	73.3812876	67.731787	56.2395153	66.8457846
18:21:43	0:21:24	67.6930524	73.3812876	67.731787	54.2395153	67.8457846
18:21:44	0:21:25	66.6930524	72.3812876	67.731787	54.2395153	68.8457846
18:21:45	0:21:26	66.6930524	73.3812876	67.731787	53.2395153	68.8457846
18:21:46	0:21:27	66.6930524	73.3812876	65.731787	53.2395153	68.8457846
18:21:47	0:21:28	66.6930524	74.3812876	65.731787	53.2395153	68.8457846
18:21:48	0:21:29	66.6930524	76.3812876	68.731787	53.2395153	68.8457846
18:21:49	0:21:30	66.6930524	75.3812876	70.731787	53.2395153	68.8457846
18:21:50	0:21:31	66.6930524	75.3812876	71.731787	53.2395153	68.8457846
18:21:51	0:21:32	66.6930524	76.3812876	72.731787	53.2395153	68.8457846
18:21:52	0:21:33	66.6930524	76.3812876	74.731787	53.2395153	68.8457846
18:21:53	0:21:34	67.6930524	75.3812876	76.731787	52.2395153	68.8457846
18:21:54	0:21:35	68.6930524	75.3812876	77.731787	52.2395153	69.8457846
18:21:55	0:21:36	70.6930524	76.3812876	78.731787	53.2395153	69.8457846
18:21:56	0:21:37	71.6930524	76.3812876	78.731787	53.2395153	69.8457846
18:21:57	0:21:38	70.6930524	75.3812876	78.731787	54.2395153	70.8457846

18:21:58	0:21:39	69.6930524	75.3812876	78.731787	53.2395153	70.8457846
18:21:59	0:21:40	68.6930524	75.3812876	78.731787	52.2395153	71.8457846
18:22:00	0:21:41	68.6930524	74.3812876	78.731787	52.2395153	71.8457846
18:22:01	0:21:42	67.6930524	72.3812876	71.731787	53.2395153	72.8457846
18:22:02	0:21:43	67.6930524	70.3812876	70.731787	53.2395153	72.8457846
18:22:03	0:21:44	67.6930524	68.3812876	70.731787	53.2395153	72.8457846
18:22:04	0:21:45	67.6930524	67.3812876	70.731787	53.2395153	73.8457846
18:22:05	0:21:46	67.6930524	67.3812876	71.731787	53.2395153	73.8457846
18:22:06	0:21:47	67.6930524	68.3812876	71.731787	54.2395153	73.8457846
18:22:07	0:21:48	68.6930524	69.3812876	71.731787	54.2395153	77.8457846
18:22:08	0:21:49	69.6930524	69.3812876	71.731787	55.2395153	77.8457846
18:22:09	0:21:50	70.6930524	72.3812876	70.731787	55.2395153	83.8457846
18:22:10	0:21:51	70.6930524	71.3812876	69.731787	54.2395153	85.8457846
18:22:11	0:21:52	70.6930524	71.3812876	69.731787	53.2395153	71.7951505
18:22:12	0:21:53	70.6930524	71.3812876	69.731787	53.2395153	71.9201505
18:22:13	0:21:54	70.6930524	71.3812876	69.731787	53.2395153	72.2951505
18:22:14	0:21:55	70.6930524	71.3812876	69.731787	54.2395153	72.6701505
18:22:15	0:21:56	70.6930524	71.3812876	69.731787	56.2395153	73.0451505
18:22:16	0:21:57	71.6930524	71.3812876	74.731787	56.2395153	73.5451505
18:22:17	0:21:58	71.6930524	71.3812876	76.731787	56.2395153	73.6701505
18:22:18	0:21:59	72.6930524	73.3812876	76.731787	56.2395153	78.8457846
18:22:19	0:22:00	73.6930524	73.3812876	77.731787	56.2395153	70.8457846
18:22:20	0:22:01	73.6930524	73.3812876	78.731787	56.2395153	67.8457846
18:22:21	0:22:02	73.6930524	73.3812876	78.731787	56.2395153	67.8457846
18:22:22	0:22:03	72.6930524	74.3812876	78.731787	55.2395153	65.8457846
18:22:23	0:22:04	72.6930524	74.3812876	79.731787	55.2395153	64.8457846
18:22:24	0:22:05	72.6930524	76.3812876	81.731787	55.2395153	64.8457846
18:22:25	0:22:06	72.6930524	78.3812876	80.731787	56.2395153	61.8457846
18:22:26	0:22:07	72.6930524	79.3812876	76.731787	56.2395153	62.8457846
18:22:27	0:22:08	72.6930524	79.3812876	74.731787	56.2395153	65.8457846
18:22:28	0:22:09	72.6930524	78.3812876	72.731787	56.2395153	67.8457846
18:22:29	0:22:10	72.6930524	77.3812876	72.731787	55.2395153	67.8457846
18:22:30	0:22:11	72.6930524	77.3812876	72.731787	55.2395153	67.8457846
18:22:31	0:22:12	70.6930524	77.3812876	70.731787	57.2395153	71.8457846
18:22:32	0:22:13	69.6930524	77.3812876	69.731787	59.2395153	68.8457846
18:22:33	0:22:14	68.6930524	77.3812876	69.731787	60.2395153	67.8457846
18:22:34	0:22:15	68.6930524	78.3812876	68.731787	60.2395153	66.8457846
18:22:35	0:22:16	68.6930524	78.3812876	68.731787	61.2395153	65.8457846
18:22:36	0:22:17	69.6930524	79.3812876	68.731787	61.2395153	64.8457846
18:22:37	0:22:18	69.6930524	80.3812876	67.731787	61.2395153	64.8457846
18:22:38	0:22:19	70.6930524	80.3812876	68.731787	61.2395153	64.8457846
18:22:39	0:22:20	70.6930524	80.3812876	68.731787	61.2395153	64.8457846
18:22:40	0:22:21	71.6930524	80.3812876	68.731787	61.2395153	64.8457846
18:22:41	0:22:22	72.6930524	80.3812876	69.731787	59.2395153	65.8457846
18:22:42	0:22:23	72.6930524	80.3812876	69.731787	58.2395153	65.8457846

18:22:43	0:22:24	72.6930524	79.3812876	69.731787	58.2395153	65.8457846
18:22:44	0:22:25	72.6930524	79.3812876	69.731787	57.2395153	65.8457846
18:22:45	0:22:26	71.6930524	80.3812876	72.731787	57.2395153	65.8457846
18:22:46	0:22:27	71.6930524	80.3812876	72.731787	53.2395153	65.8457846
18:22:47	0:22:28	70.6930524	80.3812876	73.731787	53.2395153	68.8457846
18:22:48	0:22:29	70.6930524	80.3812876	74.731787	54.2395153	69.8457846
18:22:49	0:22:30	69.6930524	80.3812876	75.731787	54.2395153	66.8457846
18:22:50	0:22:31	68.6930524	80.3812876	74.731787	54.2395153	64.8457846
18:22:51	0:22:32	68.6930524	80.3812876	74.731787	54.2395153	63.8457846
18:22:52	0:22:33	68.6930524	78.3812876	73.731787	54.2395153	63.8457846
18:22:53	0:22:34	68.6930524	78.3812876	73.731787	54.2395153	65.8457846
18:22:54	0:22:35	68.6930524	77.3812876	73.731787	55.2395153	67.8457846
18:22:55	0:22:36	68.6930524	77.3812876	73.731787	56.2395153	68.8457846
18:22:56	0:22:37	71.6930524	77.3812876	74.731787	56.2395153	68.8457846
18:22:57	0:22:38	77.6930524	77.3812876	74.731787	56.2395153	67.8457846
18:22:58	0:22:39	78.6930524	77.3812876	75.731787	57.2395153	67.8457846
18:22:59	0:22:40	79.6930524	77.3812876	76.731787	58.2395153	65.8457846
18:23:00	0:22:41	79.6930524	77.3812876	76.731787	58.2395153	63.8457846
18:23:01	0:22:42	79.6930524	77.3812876	76.731787	58.2395153	63.8457846
18:23:02	0:22:43	79.6930524	78.3812876	76.731787	58.2395153	63.8457846
18:23:03	0:22:44	79.6930524	78.3812876	75.731787	58.2395153	63.8457846
18:23:04	0:22:45	78.6930524	78.3812876	71.731787	60.2395153	63.8457846
18:23:05	0:22:46	77.6930524	78.3812876	68.731787	61.2395153	65.8457846
18:23:06	0:22:47	77.6930524	79.3812876	68.731787	61.2395153	65.8457846
18:23:07	0:22:48	77.6930524	79.3812876	66.731787	64.2395153	64.8457846
18:23:08	0:22:49	77.6930524	79.3812876	66.731787	64.2395153	64.8457846
18:23:09	0:22:50	77.6930524	78.3812876	66.731787	64.2395153	64.8457846
18:23:10	0:22:51	77.6930524	78.3812876	67.731787	64.2395153	65.8457846
18:23:11	0:22:52	77.6930524	79.3812876	67.731787	65.2395153	67.8457846
18:23:12	0:22:53	77.6930524	79.3812876	67.731787	65.2395153	69.8457846
18:23:13	0:22:54	78.6930524	79.3812876	70.731787	68.2395153	70.8457846
18:23:14	0:22:55	78.6930524	79.3812876	70.731787	68.2395153	71.8457846
18:23:15	0:22:56	78.6930524	81.3812876	70.731787	68.2395153	72.8457846
18:23:16	0:22:57	78.6930524	81.3812876	71.731787	68.2395153	72.8457846
18:23:17	0:22:58	78.6930524	81.3812876	71.731787	68.2395153	72.8457846
18:23:18	0:22:59	77.6930524	81.3812876	71.731787	68.2395153	72.8457846
18:23:19	0:23:00	77.6930524	81.3812876	68.731787	68.2395153	73.8457846
18:23:20	0:23:01	77.6930524	81.3812876	67.731787	63.2395153	74.8457846
18:23:21	0:23:02	76.6930524	81.3812876	67.731787	63.2395153	75.8457846
18:23:22	0:23:03	76.6930524	81.3812876	66.731787	63.2395153	76.8457846
18:23:23	0:23:04	75.6930524	81.3812876	66.731787	64.2395153	75.8457846
18:23:24	0:23:05	75.6930524	81.3812876	66.731787	64.2395153	75.8457846
18:23:25	0:23:06	74.6930524	81.3812876	69.731787	65.2395153	71.8457846
18:23:26	0:23:07	74.6930524	81.3812876	71.731787	66.2395153	66.8457846
18:23:27	0:23:08	73.6930524	81.3812876	71.731787	67.2395153	64.8457846

18:23:28	0:23:09	73.6930524	81.3812876	72.731787	68.2395153	63.8457846
18:23:29	0:23:10	72.6930524	80.3812876	71.731787	69.2395153	63.8457846
18:23:30	0:23:11	72.6930524	79.3812876	71.731787	69.2395153	62.8457846
18:23:31	0:23:12	72.6930524	77.3812876	69.731787	69.2395153	62.8457846
18:23:32	0:23:13	72.6930524	77.3812876	68.731787	70.2395153	62.8457846
18:23:33	0:23:14	69.6930524	77.3812876	68.731787	72.2395153	60.8457846
18:23:34	0:23:15	69.6930524	76.3812876	68.731787	76.2395153	62.8457846
18:23:35	0:23:16	69.6930524	76.3812876	68.731787	76.2395153	63.8457846
18:23:36	0:23:17	69.6930524	75.3812876	70.731787	78.2395153	62.8457846
18:23:37	0:23:18	69.6930524	75.3812876	71.731787	79.2395153	61.8457846
18:23:38	0:23:19	70.6930524	75.3812876	71.731787	78.2395153	62.8457846
18:23:39	0:23:20	70.6930524	75.3812876	71.731787	77.2395153	64.8457846
18:23:40	0:23:21	75.6930524	75.3812876	71.731787	76.2395153	65.8457846
18:23:41	0:23:22	80.6930524	74.3812876	71.731787	75.2395153	66.8457846
18:23:42	0:23:23	81.6930524	72.3812876	72.731787	75.2395153	66.8457846
18:23:43	0:23:24	81.6930524	72.3812876	73.731787	75.2395153	67.8457846
18:23:44	0:23:25	76.6930524	71.3812876	73.731787	75.2395153	66.8457846
18:23:45	0:23:26	76.6930524	71.3812876	73.731787	75.2395153	66.8457846
18:23:46	0:23:27	72.6930524	71.3812876	75.731787	75.2395153	66.8457846
18:23:47	0:23:28	70.6930524	71.3812876	76.731787	75.2395153	66.8457846
18:23:48	0:23:29	69.6930524	71.3812876	78.731787	76.2395153	67.8457846
18:23:49	0:23:30	69.6930524	71.3812876	79.731787	75.2395153	68.8457846
18:23:50	0:23:31	70.6930524	71.3812876	79.731787	74.2395153	69.8457846
18:23:51	0:23:32	70.6930524	71.3812876	79.731787	73.2395153	69.8457846
18:23:52	0:23:33	70.6930524	73.3812876	79.731787	71.2395153	69.8457846
18:23:53	0:23:34	71.6930524	73.3812876	80.731787	71.2395153	68.8457846
18:23:54	0:23:35	72.6930524	73.3812876	80.731787	71.2395153	68.8457846
18:23:55	0:23:36	71.6930524	73.3812876	80.731787	71.2395153	68.8457846
18:23:56	0:23:37	71.6930524	71.3812876	80.731787	70.2395153	68.8457846
18:23:57	0:23:38	64.6930524	69.3812876	80.731787	65.2395153	68.8457846
18:23:58	0:23:39	61.6930524	66.3812876	80.731787	65.2395153	68.8457846
18:23:59	0:23:40	60.6930524	66.3812876	82.731787	65.2395153	68.8457846
18:24:00	0:23:41	59.6930524	66.3812876	84.731787	64.2395153	68.8457846
18:24:01	0:23:42	58.6930524	64.3812876	85.731787	64.2395153	68.8457846
18:24:02	0:23:43	58.6930524	63.3812876	83.731787	64.2395153	68.8457846
18:24:03	0:23:44	58.6930524	63.3812876	81.731787	64.2395153	69.8457846
18:24:04	0:23:45	55.6930524	63.3812876	81.731787	63.2395153	68.8457846
18:24:05	0:23:46	55.6930524	63.3812876	79.731787	61.2395153	68.8457846
18:24:06	0:23:47	55.6930524	62.3812876	79.731787	59.2395153	68.8457846
18:24:07	0:23:48	55.6930524	63.3812876	78.731787	59.2395153	69.8457846
18:24:08	0:23:49	54.6930524	63.3812876	78.731787	59.2395153	70.8457846
18:24:09	0:23:50	54.6930524	64.3812876	76.731787	59.2395153	71.8457846
18:24:10	0:23:51	54.6930524	64.3812876	73.731787	59.2395153	71.8457846
18:24:11	0:23:52	54.6930524	64.3812876	71.731787	59.2395153	71.8457846
18:24:12	0:23:53	55.6930524	64.3812876	71.731787	59.2395153	70.8457846

18:24:13	0:23:54	56.6930524	65.3812876	71.731787	60.2395153	69.8457846
18:24:14	0:23:55	56.6930524	65.3812876	71.731787	59.2395153	69.8457846
18:24:15	0:23:56	55.6930524	66.3812876	72.731787	59.2395153	70.8457846
18:24:16	0:23:57	55.6930524	66.3812876	74.731787	59.2395153	70.8457846
18:24:17	0:23:58	53.6930524	67.3812876	74.731787	59.2395153	70.8457846
18:24:18	0:23:59	53.6930524	67.3812876	74.731787	59.2395153	70.8457846
18:24:19	0:24:00	52.6930524	70.3812876	73.731787	60.2395153	69.8457846
18:24:20	0:24:01	51.6930524	70.3812876	71.731787	61.2395153	69.8457846
18:24:21	0:24:02	51.6930524	70.3812876	70.731787	61.2395153	69.8457846
18:24:22	0:24:03	51.6930524	70.3812876	69.731787	62.2395153	69.8457846
18:24:23	0:24:04	53.6930524	71.3812876	69.731787	62.2395153	69.8457846
18:24:24	0:24:05	54.6930524	71.3812876	69.731787	63.2395153	69.8457846
18:24:25	0:24:06	54.6930524	71.3812876	69.731787	64.2395153	69.8457846
18:24:26	0:24:07	55.6930524	71.3812876	69.731787	64.2395153	69.8457846
18:24:27	0:24:08	55.6930524	71.3812876	69.731787	63.2395153	69.8457846
18:24:28	0:24:09	55.6930524	71.3812876	68.731787	61.2395153	70.8457846
18:24:29	0:24:10	55.6930524	71.3812876	67.731787	61.2395153	71.8457846
18:24:30	0:24:11	57.6930524	71.3812876	67.731787	61.2395153	72.8457846
18:24:31	0:24:12	57.6930524	71.3812876	67.731787	64.2395153	72.8457846
18:24:32	0:24:13	59.6930524	71.3812876	67.731787	66.2395153	74.8457846
18:24:33	0:24:14	60.6930524	71.3812876	68.731787	68.2395153	77.8457846
18:24:34	0:24:15	60.6930524	71.3812876	68.731787	69.2395153	80.8457846
18:24:35	0:24:16	65.6930524	73.3812876	68.731787	69.2395153	78.8457846
18:24:36	0:24:17	65.6930524	73.3812876	68.731787	70.2395153	77.8457846
18:24:37	0:24:18	65.6930524	73.3812876	68.731787	70.2395153	77.8457846
18:24:38	0:24:19	65.6930524	73.3812876	68.731787	70.2395153	74.8457846
18:24:39	0:24:20	65.6930524	73.3812876	68.731787	70.2395153	72.8457846
18:24:40	0:24:21	65.6930524	72.3812876	68.731787	70.2395153	72.8457846
18:24:41	0:24:22	63.6930524	73.3812876	70.731787	71.2395153	72.8457846
18:24:42	0:24:23	62.6930524	73.3812876	70.731787	71.2395153	71.8457846
18:24:43	0:24:24	62.6930524	74.3812876	72.731787	72.2395153	71.8457846
18:24:44	0:24:25	62.6930524	82.3812876	72.731787	73.2395153	71.8457846
18:24:45	0:24:26	62.6930524	82.3812876	75.731787	73.2395153	71.8457846
18:24:46	0:24:27	62.6930524	82.3812876	75.731787	74.2395153	71.8457846
18:24:47	0:24:28	63.6930524	83.3812876	75.731787	74.2395153	71.8457846
18:24:48	0:24:29	65.6930524	83.3812876	76.731787	74.2395153	71.8457846
18:24:49	0:24:30	66.6930524	84.3812876	78.731787	75.2395153	71.8457846
18:24:50	0:24:31	66.6930524	78.3812876	78.731787	76.2395153	71.8457846
18:24:51	0:24:32	67.6930524	77.3812876	78.731787	76.2395153	71.8457846
18:24:52	0:24:33	67.6930524	76.3812876	78.731787	79.2395153	71.8457846
18:24:53	0:24:34	67.6930524	76.3812876	79.731787	82.2395153	72.8457846
18:24:54	0:24:35	67.6930524	76.3812876	79.731787	85.2395153	72.8457846
18:24:55	0:24:36	66.6930524	75.3812876	80.731787	87.2395153	72.8457846
18:24:56	0:24:37	67.6930524	74.3812876	80.731787	87.2395153	72.8457846
18:24:57	0:24:38	68.6930524	72.3812876	80.731787	86.2395153	73.8457846

18:24:58	0:24:39	70.6930524	72.3812876	82.731787	85.2395153	72.8457846
18:24:59	0:24:40	71.6930524	71.3812876	82.731787	84.2395153	72.8457846
18:25:00	0:24:41	70.6930524	73.3812876	81.731787	83.2395153	71.8457846
18:25:01	0:24:42	69.6930524	73.3812876	76.731787	83.2395153	71.8457846
18:25:02	0:24:43	69.6930524	73.3812876	75.731787	81.2395153	70.8457846
18:25:03	0:24:44	69.6930524	73.3812876	75.731787	78.2395153	69.8457846
18:25:04	0:24:45	68.6930524	74.3812876	75.731787	75.2395153	69.8457846
18:25:05	0:24:46	68.6930524	74.3812876	75.731787	75.2395153	69.8457846
18:25:06	0:24:47	69.6930524	74.3812876	75.731787	71.2395153	68.8457846
18:25:07	0:24:48	70.6930524	75.3812876	75.731787	69.2395153	67.8457846
18:25:08	0:24:49	70.6930524	76.3812876	75.731787	68.2395153	67.8457846
18:25:09	0:24:50	71.6930524	76.3812876	73.731787	67.2395153	67.8457846
18:25:10	0:24:51	71.6930524	76.3812876	73.731787	64.2395153	67.8457846
18:25:11	0:24:52	70.6930524	76.3812876	71.731787	64.2395153	67.8457846
18:25:12	0:24:53	70.6930524	76.3812876	71.731787	64.2395153	67.8457846
18:25:13	0:24:54	67.6930524	74.3812876	71.731787	60.2395153	67.8457846
18:25:14	0:24:55	66.6930524	74.3812876	70.731787	59.2395153	67.8457846
18:25:15	0:24:56	66.6930524	74.3812876	70.731787	57.2395153	67.8457846
18:25:16	0:24:57	66.6930524	74.3812876	70.731787	56.2395153	67.8457846
18:25:17	0:24:58	66.6930524	74.3812876	70.731787	55.2395153	66.8457846
18:25:18	0:24:59	66.6930524	74.3812876	71.731787	55.2395153	64.8457846
18:25:19	0:25:00	65.6930524	75.3812876	71.731787	55.2395153	64.8457846
18:25:20	0:25:01	63.6930524	73.3812876	72.731787	55.2395153	64.8457846
18:25:21	0:25:02	64.6930524	73.3812876	72.731787	55.2395153	65.8457846
18:25:22	0:25:03	64.6930524	73.3812876	74.731787	55.2395153	67.8457846
18:25:23	0:25:04	65.6930524	72.3812876	75.731787	58.2395153	68.8457846
18:25:24	0:25:05	67.6930524	73.3812876	75.731787	59.2395153	69.8457846
18:25:25	0:25:06	68.6930524	73.3812876	75.731787	59.2395153	69.8457846
18:25:26	0:25:07	69.6930524	75.3812876	76.731787	59.2395153	69.8457846
18:25:27	0:25:08	68.6930524	75.3812876	76.731787	59.2395153	69.8457846
18:25:28	0:25:09	69.6930524	75.3812876	77.731787	59.2395153	69.8457846
18:25:29	0:25:10	68.6930524	74.3812876	77.731787	59.2395153	69.8457846
18:25:30	0:25:11	66.6930524	71.3812876	77.731787	60.2395153	69.8457846
18:25:31	0:25:12	66.6930524	71.3812876	78.731787	60.2395153	70.8457846
18:25:32	0:25:13	67.6930524	70.3812876	78.731787	60.2395153	73.8457846
18:25:33	0:25:14	68.6930524	70.3812876	79.731787	61.2395153	74.8457846
18:25:34	0:25:15	68.6930524	72.3812876	80.731787	60.2395153	77.8457846
18:25:35	0:25:16	68.6930524	72.3812876	80.731787	60.2395153	77.8457846
18:25:36	0:25:17	68.6930524	73.3812876	80.731787	60.2395153	76.8457846
18:25:37	0:25:18	67.6930524	73.3812876	80.731787	60.2395153	76.8457846
18:25:38	0:25:19	67.6930524	73.3812876	80.731787	60.2395153	76.8457846
18:25:39	0:25:20	67.6930524	72.3812876	80.731787	60.2395153	75.8457846
18:25:40	0:25:21	66.6930524	72.3812876	82.731787	58.2395153	75.8457846
18:25:41	0:25:22	66.6930524	72.3812876	82.731787	57.2395153	74.8457846
18:25:42	0:25:23	66.6930524	73.3812876	82.731787	57.2395153	73.8457846

18:25:43	0:25:24	67.6930524	73.3812876	82.731787	57.2395153	73.8457846
18:25:44	0:25:25	68.6930524	73.3812876	82.731787	57.2395153	73.8457846
18:25:45	0:25:26	69.6930524	71.3812876	82.731787	57.2395153	72.8457846
18:25:46	0:25:27	69.6930524	71.3812876	82.731787	57.2395153	66.8457846
18:25:47	0:25:28	68.6930524	71.3812876	82.731787	58.2395153	64.8457846
18:25:48	0:25:29	68.6930524	71.3812876	81.731787	59.2395153	63.8457846
18:25:49	0:25:30	68.6930524	71.3812876	78.731787	59.2395153	63.8457846
18:25:50	0:25:31	68.6930524	69.3812876	78.731787	61.2395153	64.8457846
18:25:51	0:25:32	67.6930524	67.3812876	78.731787	62.2395153	63.8457846
18:25:52	0:25:33	66.6930524	67.3812876	78.731787	63.2395153	61.8457846
18:25:53	0:25:34	66.6930524	67.3812876	79.731787	62.2395153	60.8457846
18:25:54	0:25:35	65.6930524	66.3812876	79.731787	62.2395153	59.8457846
18:25:55	0:25:36	64.6930524	65.3812876	76.731787	62.2395153	59.8457846
18:25:56	0:25:37	64.6930524	64.3812876	75.731787	62.2395153	59.8457846
18:25:57	0:25:38	63.6930524	64.3812876	74.731787	62.2395153	60.8457846
18:25:58	0:25:39	61.6930524	64.3812876	74.731787	62.2395153	62.8457846
18:25:59	0:25:40	62.6930524	64.3812876	74.731787	62.2395153	62.8457846
18:26:00	0:25:41	62.6930524	63.3812876	74.731787	62.2395153	63.8457846
18:26:01	0:25:42	62.6930524	63.3812876	74.731787	63.2395153	63.8457846
18:26:02	0:25:43	68.6930524	61.3812876	72.731787	63.2395153	63.8457846
18:26:03	0:25:44	69.6930524	61.3812876	71.731787	63.2395153	63.8457846
18:26:04	0:25:45	68.6930524	61.3812876	69.731787	67.2395153	66.8457846
18:26:05	0:25:46	67.6930524	61.3812876	68.731787	67.2395153	66.8457846
18:26:06	0:25:47	67.6930524	61.3812876	68.731787	67.2395153	66.8457846
18:26:07	0:25:48	66.6930524	61.3812876	68.731787	67.2395153	69.8457846
18:26:08	0:25:49	64.6930524	61.3812876	65.731787	68.2395153	69.8457846
18:26:09	0:25:50	64.6930524	62.3812876	64.731787	68.2395153	69.8457846
18:26:10	0:25:51	64.6930524	62.3812876	64.731787	69.2395153	69.8457846
18:26:11	0:25:52	64.6930524	62.3812876	64.731787	70.2395153	69.8457846
18:26:12	0:25:53	63.6930524	62.3812876	63.731787	72.2395153	69.8457846
18:26:13	0:25:54	63.6930524	62.3812876	63.731787	72.2395153	69.8457846
18:26:14	0:25:55	64.6930524	60.3812876	63.731787	73.2395153	69.8457846
18:26:15	0:25:56	63.6930524	59.3812876	63.731787	73.2395153	69.8457846
18:26:16	0:25:57	63.6930524	59.3812876	65.731787	73.2395153	72.8457846
18:26:17	0:25:58	63.6930524	60.3812876	65.731787	74.2395153	72.8457846
18:26:18	0:25:59	63.6930524	60.3812876	66.731787	75.2395153	69.8457846
18:26:19	0:26:00	63.6930524	60.3812876	66.731787	75.2395153	67.8457846
18:26:20	0:26:01	62.6930524	61.3812876	67.731787	75.2395153	67.8457846
18:26:21	0:26:02	65.6930524	64.3812876	67.731787	75.2395153	67.8457846
18:26:22	0:26:03	66.6930524	70.3812876	67.731787	75.2395153	67.8457846
18:26:23	0:26:04	66.6930524	73.3812876	67.731787	75.2395153	67.8457846
18:26:24	0:26:05	67.6930524	74.3812876	66.731787	75.2395153	67.8457846
18:26:25	0:26:06	69.6930524	74.3812876	66.731787	73.2395153	68.8457846
18:26:26	0:26:07	70.6930524	74.3812876	66.731787	73.2395153	70.8457846
18:26:27	0:26:08	70.6930524	71.3812876	65.731787	73.2395153	70.8457846

18:26:28	0:26:09	70.6930524	70.3812876	65.731787	72.2395153	70.8457846
18:26:29	0:26:10	69.6930524	69.3812876	65.731787	72.2395153	71.8457846
18:26:30	0:26:11	68.6930524	70.3812876	66.731787	72.2395153	71.8457846
18:26:31	0:26:12	68.6930524	71.3812876	66.731787	74.2395153	71.8457846
18:26:32	0:26:13	66.6930524	71.3812876	66.731787	76.2395153	70.8457846
18:26:33	0:26:14	65.6930524	73.3812876	67.731787	76.2395153	69.8457846
18:26:34	0:26:15	65.6930524	74.3812876	67.731787	76.2395153	68.8457846
18:26:35	0:26:16	65.6930524	75.3812876	67.731787	77.2395153	67.8457846
18:26:36	0:26:17	65.6930524	75.3812876	67.731787	78.2395153	67.8457846
18:26:37	0:26:18	65.6930524	75.3812876	68.731787	80.2395153	67.8457846
18:26:38	0:26:19	66.6930524	75.3812876	69.731787	80.2395153	67.8457846
18:26:39	0:26:20	66.6930524	75.3812876	69.731787	80.2395153	67.8457846
18:26:40	0:26:21	66.6930524	74.3812876	70.731787	79.2395153	66.8457846
18:26:41	0:26:22	66.6930524	74.3812876	71.731787	79.2395153	64.8457846
18:26:42	0:26:23	66.6930524	70.3812876	71.731787	79.2395153	62.8457846
18:26:43	0:26:24	66.6930524	69.3812876	71.731787	78.2395153	63.8457846
18:26:44	0:26:25	66.6930524	68.3812876	71.731787	77.2395153	64.8457846
18:26:45	0:26:26	73.6930524	67.3812876	70.731787	77.2395153	64.8457846
18:26:46	0:26:27	76.6930524	67.3812876	70.731787	76.2395153	64.8457846
18:26:47	0:26:28	77.6930524	67.3812876	70.731787	76.2395153	63.8457846
18:26:48	0:26:29	79.6930524	67.3812876	70.731787	76.2395153	61.8457846
18:26:49	0:26:30	81.6930524	68.3812876	69.731787	71.2395153	61.8457846
18:26:50	0:26:31	82.6930524	65.3812876	68.731787	69.2395153	61.8457846
18:26:51	0:26:32	86.6930524	65.3812876	68.731787	68.2395153	64.8457846
18:26:52	0:26:33	70.4201505	64.3812876	68.731787	66.2395153	64.8457846
18:26:53	0:26:34	70.6701505	64.3812876	67.731787	65.2395153	64.8457846
18:26:54	0:26:35	70.2951505	65.3812876	65.731787	65.2395153	64.8457846
18:26:55	0:26:36	70.0451505	65.3812876	63.731787	65.2395153	64.8457846
18:26:56	0:26:37	69.9201505	65.3812876	62.731787	65.2395153	64.8457846
18:26:57	0:26:38	70.2951505	65.3812876	62.731787	65.2395153	64.8457846
18:26:58	0:26:39	70.4201505	65.3812876	62.731787	67.2395153	64.8457846
18:26:59	0:26:40	70.2951505	65.3812876	62.731787	68.2395153	64.8457846
18:27:00	0:26:41	70.2951505	65.3812876	62.731787	68.2395153	64.8457846
18:27:01	0:26:42	85.6930524	65.3812876	63.731787	69.2395153	70.8457846
18:27:02	0:26:43	84.6930524	65.3812876	63.731787	70.2395153	72.8457846
18:27:03	0:26:44	83.6930524	67.3812876	63.731787	70.2395153	70.8457846
18:27:04	0:26:45	81.6930524	71.3812876	63.731787	71.2395153	70.8457846
18:27:05	0:26:46	80.6930524	72.3812876	64.731787	71.2395153	66.8457846
18:27:06	0:26:47	79.6930524	72.3812876	65.731787	72.2395153	66.8457846
18:27:07	0:26:48	77.6930524	72.3812876	65.731787	72.2395153	66.8457846
18:27:08	0:26:49	71.6930524	72.3812876	65.731787	72.2395153	66.8457846
18:27:09	0:26:50	68.6930524	72.3812876	65.731787	74.2395153	66.8457846
18:27:10	0:26:51	67.6930524	72.3812876	65.731787	75.2395153	66.8457846
18:27:11	0:26:52	67.6930524	72.3812876	65.731787	75.2395153	66.8457846
18:27:12	0:26:53	67.6930524	73.3812876	65.731787	77.2395153	66.8457846

18:27:13	0:26:54	67.6930524	73.3812876	65.731787	77.2395153	66.8457846
18:27:14	0:26:55	67.6930524	73.3812876	65.731787	86.2395153	69.8457846
18:27:15	0:26:56	66.6930524	73.3812876	65.731787	87.2395153	67.8457846
18:27:16	0:26:57	66.6930524	73.3812876	65.731787	71.4201505	67.8457846
18:27:17	0:26:58	66.6930524	74.3812876	65.731787	71.6701505	67.8457846
18:27:18	0:26:59	66.6930524	75.3812876	65.731787	72.2951505	70.8457846
18:27:19	0:27:00	65.6930524	75.3812876	65.731787	72.5451505	70.8457846
18:27:20	0:27:01	65.6930524	75.3812876	65.731787	72.5451505	70.8457846
18:27:21	0:27:02	65.6930524	75.3812876	65.731787	71.9201505	70.8457846
18:27:22	0:27:03	67.6930524	75.3812876	66.731787	87.2395153	70.8457846
18:27:23	0:27:04	67.6930524	75.3812876	66.731787	80.2395153	71.8457846
18:27:24	0:27:05	67.6930524	77.3812876	68.731787	79.2395153	71.8457846
18:27:25	0:27:06	67.6930524	77.3812876	71.731787	77.2395153	73.8457846
18:27:26	0:27:07	67.6930524	77.3812876	71.731787	75.2395153	73.8457846
18:27:27	0:27:08	68.6930524	77.3812876	71.731787	73.2395153	73.8457846
18:27:28	0:27:09	67.6930524	77.3812876	71.731787	72.2395153	73.8457846
18:27:29	0:27:10	67.6930524	77.3812876	72.731787	70.2395153	73.8457846
18:27:30	0:27:11	67.6930524	77.3812876	72.731787	70.2395153	73.8457846
18:27:31	0:27:12	66.6930524	77.3812876	72.731787	76.2395153	73.8457846
18:27:32	0:27:13	66.6930524	77.3812876	73.731787	80.2395153	74.8457846
18:27:33	0:27:14	65.6930524	77.3812876	73.731787	80.2395153	77.8457846
18:27:34	0:27:15	65.6930524	77.3812876	73.731787	81.2395153	78.8457846
18:27:35	0:27:16	65.6930524	77.3812876	73.731787	81.2395153	77.8457846
18:27:36	0:27:17	67.6930524	77.3812876	72.731787	84.2395153	73.8457846
18:27:37	0:27:18	69.6930524	77.3812876	70.731787	84.2395153	71.8457846
18:27:38	0:27:19	69.6930524	79.3812876	68.731787	79.2395153	69.8457846
18:27:39	0:27:20	69.6930524	80.3812876	68.731787	79.2395153	68.8457846
18:27:40	0:27:21	69.6930524	82.3812876	68.731787	77.2395153	67.8457846
18:27:41	0:27:22	69.6930524	82.3812876	68.731787	71.2395153	67.8457846
18:27:42	0:27:23	69.6930524	82.3812876	68.731787	69.2395153	67.8457846
18:27:43	0:27:24	69.6930524	82.3812876	69.731787	69.2395153	67.8457846
18:27:44	0:27:25	69.6930524	82.3812876	69.731787	64.2395153	67.8457846
18:27:45	0:27:26	69.6930524	81.3812876	70.731787	63.2395153	67.8457846
18:27:46	0:27:27	70.6930524	81.3812876	70.731787	63.2395153	68.8457846
18:27:47	0:27:28	71.6930524	80.3812876	68.731787	64.2395153	68.8457846
18:27:48	0:27:29	71.6930524	80.3812876	67.731787	69.2395153	68.8457846
18:27:49	0:27:30	71.6930524	81.3812876	66.731787	73.2395153	69.8457846
18:27:50	0:27:31	72.6930524	81.3812876	65.731787	77.2395153	67.8457846
18:27:51	0:27:32	73.6930524	81.3812876	64.731787	89.2395153	66.8457846
18:27:52	0:27:33	76.6930524	81.3812876	63.731787	74.2951505	67.8457846
18:27:53	0:27:34	76.6930524	83.3812876	63.731787	75.6701505	67.8457846
18:27:54	0:27:35	76.6930524	83.3812876	63.731787	75.6701505	67.8457846
18:27:55	0:27:36	76.6930524	84.3812876	63.731787	75.9201505	65.8457846
18:27:56	0:27:37	76.6930524	84.3812876	62.731787	75.7951505	65.8457846
18:27:57	0:27:38	76.6930524	84.3812876	62.731787	75.9201505	65.8457846

18:27:58	0:27:39	76.6930524	83.3812876	63.731787	76.0451505	66.8457846
18:27:59	0:27:40	75.6930524	83.3812876	63.731787	76.2951505	67.8457846
18:28:00	0:27:41	76.6930524	84.3812876	64.731787	76.7951505	67.8457846
18:28:01	0:27:42	80.6930524	85.3812876	64.731787	77.4201505	67.8457846
18:28:02	0:27:43	72.6930524	85.3812876	65.731787	76.7951505	70.8457846
18:28:03	0:27:44	70.6930524	85.3812876	65.731787	76.5451505	71.8457846
18:28:04	0:27:45	70.6930524	85.3812876	65.731787	76.6701505	72.8457846
18:28:05	0:27:46	70.6930524	86.3812876	66.731787	76.9201505	72.8457846
18:28:06	0:27:47	73.6930524	86.3812876	68.731787	77.7951505	72.8457846
18:28:07	0:27:48	78.6930524	86.3812876	68.731787	79.6701505	72.8457846
18:28:08	0:27:49	85.6930524	86.3812876	68.731787	80.9201505	71.8457846
18:28:09	0:27:50	98.6930524	86.3812876	69.731787	82.7951505	71.8457846
18:28:10	0:27:51	84.6701505	86.3812876	69.731787	84.6701505	71.8457846
18:28:11	0:27:52	84.4201505	85.3812876	68.731787	84.4201505	72.8457846
18:28:12	0:27:53	84.5451505	85.3812876	69.731787	84.5451505	72.8457846
18:28:13	0:27:54	85.5451505	85.3812876	72.731787	85.5451505	73.8457846
18:28:14	0:27:55	92.6930524	85.3812876	72.731787	83.0451505	77.8457846
18:28:15	0:27:56	88.6930524	85.3812876	72.731787	83.1701505	77.8457846
18:28:16	0:27:57	79.6930524	85.3812876	72.731787	82.4201505	77.8457846
18:28:17	0:27:58	75.6930524	82.3812876	71.731787	81.4201505	72.8457846
18:28:18	0:27:59	73.6930524	82.3812876	70.731787	80.5451505	69.8457846
18:28:19	0:28:00	72.6930524	80.3812876	70.731787	79.9201505	68.8457846
18:28:20	0:28:01	72.6930524	80.3812876	70.731787	79.9201505	70.8457846
18:28:21	0:28:02	72.6930524	79.3812876	70.731787	79.9201505	71.8457846
18:28:22	0:28:03	72.6930524	78.3812876	70.731787	80.0451505	71.8457846
18:28:23	0:28:04	72.6930524	78.3812876	72.731787	80.4201505	71.8457846
18:28:24	0:28:05	71.6930524	77.3812876	72.731787	79.7951505	71.8457846
18:28:25	0:28:06	71.6930524	75.3812876	70.731787	79.5451505	71.8457846
18:28:26	0:28:07	72.6930524	75.3812876	70.731787	79.7951505	71.8457846
18:28:27	0:28:08	72.6930524	75.3812876	70.731787	80.0451505	71.8457846
18:28:28	0:28:09	72.6930524	75.3812876	70.731787	80.4201505	71.8457846
18:28:29	0:28:10	72.6930524	75.3812876	70.731787	80.0451505	70.8457846
18:28:30	0:28:11	73.6930524	75.3812876	70.731787	80.2951505	70.8457846
18:28:31	0:28:12	75.6930524	77.3812876	70.731787	80.6701505	70.8457846
18:28:32	0:28:13	76.6930524	78.3812876	70.731787	81.0451505	70.8457846
18:28:33	0:28:14	76.6930524	78.3812876	70.731787	80.7951505	70.8457846
18:28:34	0:28:15	77.6930524	78.3812876	70.731787	81.4201505	69.8457846
18:28:35	0:28:16	77.6930524	78.3812876	70.731787	81.4201505	70.8457846
18:28:36	0:28:17	77.6930524	78.3812876	70.731787	79.6701505	70.8457846
18:28:37	0:28:18	77.6930524	80.3812876	67.731787	79.1701505	71.8457846
18:28:38	0:28:19	77.6930524	80.3812876	66.731787	78.7951505	71.8457846
18:28:39	0:28:20	77.6930524	80.3812876	65.731787	78.6701505	72.8457846
18:28:40	0:28:21	77.6930524	77.3812876	65.731787	78.1701505	75.8457846
18:28:41	0:28:22	77.6930524	76.3812876	65.731787	78.1701505	77.8457846
18:28:42	0:28:23	76.6930524	76.3812876	65.731787	77.5451505	77.8457846

18:28:43	0:28:24	74.6930524	75.3812876	66.731787	77.0451505	77.8457846
18:28:44	0:28:25	72.6930524	75.3812876	66.731787	76.9201505	77.8457846
18:28:45	0:28:26	71.6930524	73.3812876	66.731787	76.7951505	77.8457846
18:28:46	0:28:27	72.6930524	71.3812876	66.731787	77.2951505	77.8457846
18:28:47	0:28:28	72.6930524	71.3812876	66.731787	77.5451505	78.8457846
18:28:48	0:28:29	72.6930524	71.3812876	66.731787	77.5451505	78.8457846
18:28:49	0:28:30	72.6930524	70.3812876	67.731787	78.6701505	81.8457846
18:28:50	0:28:31	72.6930524	68.3812876	67.731787	78.6701505	81.8457846
18:28:51	0:28:32	72.6930524	68.3812876	67.731787	78.5451505	81.8457846
18:28:52	0:28:33	72.6930524	67.3812876	68.731787	78.2951505	81.8457846
18:28:53	0:28:34	72.6930524	65.3812876	74.731787	78.4201505	80.8457846
18:28:54	0:28:35	74.6930524	65.3812876	76.731787	78.7951505	78.8457846
18:28:55	0:28:36	75.6930524	65.3812876	77.731787	78.7951505	76.8457846
18:28:56	0:28:37	76.6930524	65.3812876	77.731787	78.6701505	74.8457846
18:28:57	0:28:38	77.6930524	64.3812876	77.731787	78.4201505	72.8457846
18:28:58	0:28:39	77.6930524	64.3812876	77.731787	78.5451505	70.8457846
18:28:59	0:28:40	79.6930524	64.3812876	77.731787	78.6701505	69.8457846
18:29:00	0:28:41	81.6930524	64.3812876	76.731787	78.5451505	69.8457846
18:29:01	0:28:42	82.6930524	63.3812876	76.731787	78.2951505	69.8457846
18:29:02	0:28:43	84.6930524	63.3812876	76.731787	78.4201505	69.8457846
18:29:03	0:28:44	84.6930524	62.3812876	76.731787	78.4201505	70.8457846
18:29:04	0:28:45	86.6930524	62.3812876	76.731787	78.4201505	70.8457846
18:29:05	0:28:46	86.6930524	62.3812876	76.731787	78.2951505	70.8457846
18:29:06	0:28:47	86.6930524	62.3812876	76.731787	78.2951505	70.8457846
18:29:07	0:28:48	85.6930524	61.3812876	75.731787	78.1701505	70.8457846
18:29:08	0:28:49	83.6930524	61.3812876	75.731787	78.2951505	73.8457846
18:29:09	0:28:50	82.6930524	61.3812876	75.731787	78.4201505	73.8457846
18:29:10	0:28:51	82.6930524	61.3812876	75.731787	78.1701505	73.8457846
18:29:11	0:28:52	82.6930524	61.3812876	75.731787	77.5451505	73.8457846
18:29:12	0:28:53	82.6930524	61.3812876	75.731787	77.1701505	73.8457846
18:29:13	0:28:54	86.6930524	61.3812876	75.731787	77.5451505	73.8457846
18:29:14	0:28:55	90.6930524	63.3812876	73.731787	77.7951505	72.8457846
18:29:15	0:28:56	84.6930524	65.3812876	74.731787	77.4201505	72.8457846
18:29:16	0:28:57	84.6930524	65.3812876	74.731787	77.5451505	72.8457846
18:29:17	0:28:58	85.6930524	66.3812876	74.731787	78.0451505	72.8457846
18:29:18	0:28:59	85.6930524	66.3812876	74.731787	78.0451505	72.8457846
18:29:19	0:29:00	86.6930524	66.3812876	74.731787	78.0451505	72.8457846
18:29:20	0:29:01	87.6930524	67.3812876	73.731787	77.6701505	71.8457846
18:29:21	0:29:02	88.6930524	68.3812876	72.731787	77.2951505	71.8457846
18:29:22	0:29:03	88.6930524	68.3812876	73.731787	77.4201505	71.8457846
18:29:23	0:29:04	88.6930524	68.3812876	73.731787	76.9201505	70.8457846
18:29:24	0:29:05	88.6930524	69.3812876	72.731787	76.7951505	70.8457846
18:29:25	0:29:06	88.6930524	69.3812876	72.731787	77.0451505	69.8457846
18:29:26	0:29:07	89.6930524	69.3812876	69.731787	77.0451505	69.8457846
18:29:27	0:29:08	92.6930524	69.3812876	68.731787	77.9201505	69.8457846

18:29:28	0:29:09	93.6930524	69.3812876	66.731787	78.1701505	69.8457846
18:29:29	0:29:10	93.6930524	70.3812876	66.731787	78.4201505	69.8457846
18:29:30	0:29:11	91.6930524	70.3812876	66.731787	78.0451505	70.8457846
18:29:31	0:29:12	91.6930524	70.3812876	66.731787	77.5451505	71.8457846
18:29:32	0:29:13	90.6930524	70.3812876	68.731787	77.9201505	71.8457846
18:29:33	0:29:14	88.6930524	70.3812876	70.731787	77.6701505	71.8457846
18:29:34	0:29:15	87.6930524	70.3812876	71.731787	77.9201505	71.8457846
18:29:35	0:29:16	85.6930524	70.3812876	71.731787	78.0451505	70.8457846
18:29:36	0:29:17	79.6930524	70.3812876	71.731787	77.6701505	70.8457846
18:29:37	0:29:18	79.6930524	70.3812876	72.731787	78.1701505	71.8457846
18:29:38	0:29:19	82.6930524	72.3812876	72.731787	79.2951505	72.8457846
18:29:39	0:29:20	83.6930524	72.3812876	72.731787	79.4201505	72.8457846
18:29:40	0:29:21	83.6930524	72.3812876	72.731787	79.5451505	72.8457846
18:29:41	0:29:22	83.6930524	72.3812876	73.731787	79.9201505	72.8457846
18:29:42	0:29:23	83.6930524	72.3812876	73.731787	79.9201505	72.8457846
18:29:43	0:29:24	84.6930524	73.3812876	73.731787	80.0451505	70.8457846
18:29:44	0:29:25	84.6930524	73.3812876	73.731787	80.5451505	70.8457846
18:29:45	0:29:26	86.6930524	73.3812876	73.731787	80.9201505	70.8457846
18:29:46	0:29:27	84.6930524	76.3812876	73.731787	81.0451505	70.8457846
18:29:47	0:29:28	83.6930524	78.3812876	73.731787	80.9201505	70.8457846
18:29:48	0:29:29	83.6930524	78.3812876	73.731787	80.5451505	70.8457846
18:29:49	0:29:30	81.6930524	78.3812876	73.731787	80.5451505	70.8457846
18:29:50	0:29:31	80.6930524	72.3812876	73.731787	79.6701505	71.8457846
18:29:51	0:29:32	78.6930524	69.3812876	73.731787	79.1701505	71.8457846
18:29:52	0:29:33	79.6930524	66.3812876	72.731787	78.6701505	71.8457846
18:29:53	0:29:34	77.6930524	65.3812876	73.731787	78.4201505	72.8457846
18:29:54	0:29:35	77.6930524	65.3812876	75.731787	78.6701505	72.8457846
18:29:55	0:29:36	77.6930524	65.3812876	75.731787	78.6701505	72.8457846
18:29:56	0:29:37	77.6930524	65.3812876	75.731787	78.4201505	73.8457846
18:29:57	0:29:38	76.6930524	64.3812876	72.731787	77.2951505	73.8457846
18:29:58	0:29:39	76.6930524	64.3812876	70.731787	76.2951505	70.8457846
18:29:59	0:29:40	76.6930524	63.3812876	70.731787	75.2951505	69.8457846
18:30:00	0:29:41	70.6930524	63.3812876	70.731787	74.4201505	70.8457846
18:30:01	0:29:42	69.6930524	63.3812876	70.731787	74.4201505	72.8457846
18:30:02	0:29:43	70.6930524	65.3812876	70.731787	74.7951505	72.8457846
18:30:03	0:29:44	70.6930524	65.3812876	70.731787	74.6701505	72.8457846
18:30:04	0:29:45	70.6930524	62.3812876	71.731787	74.4201505	72.8457846
18:30:05	0:29:46	70.6930524	60.3812876	70.731787	73.9201505	70.8457846
18:30:06	0:29:47	71.6930524	60.3812876	70.731787	74.0451505	70.8457846
18:30:07	0:29:48	70.6930524	59.3812876	70.731787	73.7951505	70.8457846
18:30:08	0:29:49	70.6930524	59.3812876	70.731787	74.0451505	70.8457846
18:30:09	0:29:50	69.6930524	59.3812876	66.731787	73.9201505	70.8457846
18:30:10	0:29:51	67.6930524	59.3812876	66.731787	73.7951505	70.8457846
18:30:11	0:29:52	66.6930524	59.3812876	66.731787	73.9201505	69.8457846
18:30:12	0:29:53	66.6930524	59.3812876	67.731787	74.0451505	69.8457846

18:30:13	0:29:54	66.6930524	59.3812876	67.731787	74.1701505	70.8457846
18:30:14	0:29:55	66.6930524	61.3812876	67.731787	73.9201505	67.8457846
18:30:15	0:29:56	66.6930524	61.3812876	68.731787	74.0451505	64.8457846
18:30:16	0:29:57	66.6930524	61.3812876	68.731787	73.9201505	63.8457846
18:30:17	0:29:58	66.6930524	61.3812876	68.731787	72.6701505	62.8457846
18:30:18	0:29:59	66.6930524	61.3812876	66.731787	72.2951505	61.8457846
18:30:19	0:30:00	67.6930524	61.3812876	66.731787	72.7951505	61.8457846
18:30:20	0:30:01	67.6930524	61.3812876	66.731787	73.0451505	61.8457846
18:30:21	0:30:02	67.6930524	60.3812876	66.731787	72.5451505	61.8457846
18:30:22	0:30:03	67.6930524	61.3812876	67.731787	72.7951505	61.8457846
18:30:23	0:30:04	68.6930524	62.3812876	67.731787	73.1701505	61.8457846
18:30:24	0:30:05	68.6930524	62.3812876	67.731787	73.0451505	61.8457846
18:30:25	0:30:06	68.6930524	62.3812876	67.731787	72.9201505	61.8457846
18:30:26	0:30:07	68.6930524	62.3812876	68.731787	73.0451505	61.8457846
18:30:27	0:30:08	68.6930524	62.3812876	68.731787	72.7951505	61.8457846
18:30:28	0:30:09	68.6930524	62.3812876	68.731787	72.2951505	61.8457846
18:30:29	0:30:10	67.6930524	62.3812876	68.731787	71.9201505	61.8457846
18:30:30	0:30:11	67.6930524	62.3812876	68.731787	71.7951505	61.8457846
18:30:31	0:30:12	67.6930524	64.3812876	67.731787	72.2951505	64.8457846
18:30:32	0:30:13	67.6930524	65.3812876	67.731787	73.1701505	68.8457846
18:30:33	0:30:14	67.6930524	65.3812876	68.731787	73.6701505	70.8457846
18:30:34	0:30:15	67.6930524	66.3812876	68.731787	74.4201505	70.8457846
18:30:35	0:30:16	68.6930524	66.3812876	68.731787	75.0451505	70.8457846
18:30:36	0:30:17	68.6930524	66.3812876	68.731787	75.0451505	70.8457846
18:30:37	0:30:18	69.6930524	67.3812876	68.731787	75.4201505	70.8457846
18:30:38	0:30:19	71.6930524	67.3812876	68.731787	75.6701505	70.8457846
18:30:39	0:30:20	71.6930524	68.3812876	68.731787	74.9201505	70.8457846
18:30:40	0:30:21	72.6930524	68.3812876	68.731787	74.5451505	71.8457846
18:30:41	0:30:22	71.6930524	66.3812876	68.731787	74.1701505	72.8457846
18:30:42	0:30:23	71.6930524	66.3812876	68.731787	74.0451505	72.8457846
18:30:43	0:30:24	68.6930524	66.3812876	69.731787	73.2951505	73.8457846
18:30:44	0:30:25	68.6930524	66.3812876	69.731787	72.5451505	73.8457846
18:30:45	0:30:26	67.6930524	66.3812876	69.731787	72.2951505	73.8457846
18:30:46	0:30:27	67.6930524	66.3812876	69.731787	72.1701505	74.8457846
18:30:47	0:30:28	67.6930524	66.3812876	70.731787	72.2951505	73.8457846
18:30:48	0:30:29	67.6930524	66.3812876	70.731787	72.2951505	72.8457846
18:30:49	0:30:30	67.6930524	66.3812876	70.731787	72.6701505	72.8457846
18:30:50	0:30:31	66.6930524	67.3812876	70.731787	73.0451505	72.8457846
18:30:51	0:30:32	66.6930524	67.3812876	70.731787	73.6701505	72.8457846
18:30:52	0:30:33	66.6930524	67.3812876	70.731787	74.0451505	73.8457846
18:30:53	0:30:34	66.6930524	68.3812876	70.731787	73.9201505	73.8457846
18:30:54	0:30:35	66.6930524	68.3812876	69.731787	73.7951505	72.8457846
18:30:55	0:30:36	68.6930524	68.3812876	69.731787	73.9201505	71.8457846
18:30:56	0:30:37	68.6930524	69.3812876	69.731787	73.1701505	66.8457846
18:30:57	0:30:38	68.6930524	69.3812876	69.731787	72.5451505	63.8457846

18:30:58	0:30:39	68.6930524	68.3812876	70.731787	71.7951505	61.8457846
18:30:59	0:30:40	68.6930524	68.3812876	70.731787	83.2395153	60.8457846
18:31:00	0:30:41	68.6930524	67.3812876	71.731787	67.2395153	60.8457846
18:31:01	0:30:42	67.6930524	65.3812876	71.731787	62.2395153	60.8457846
18:31:02	0:30:43	67.6930524	65.3812876	71.731787	61.2395153	61.8457846
18:31:03	0:30:44	67.6930524	65.3812876	71.731787	61.2395153	61.8457846
18:31:04	0:30:45	66.6930524	65.3812876	71.731787	61.2395153	66.8457846
18:31:05	0:30:46	66.6930524	63.3812876	71.731787	61.2395153	67.8457846
18:31:06	0:30:47	66.6930524	64.3812876	71.731787	61.2395153	67.8457846
18:31:07	0:30:48	66.6930524	65.3812876	71.731787	61.2395153	67.8457846
18:31:08	0:30:49	66.6930524	66.3812876	71.731787	61.2395153	68.8457846
18:31:09	0:30:50	66.6930524	66.3812876	72.731787	61.2395153	68.8457846
18:31:10	0:30:51	68.6930524	66.3812876	72.731787	62.2395153	68.8457846
18:31:11	0:30:52	67.6930524	67.3812876	72.731787	62.2395153	69.8457846
18:31:12	0:30:53	67.6930524	68.3812876	69.731787	63.2395153	69.8457846
18:31:13	0:30:54	66.6930524	68.3812876	67.731787	59.2395153	69.8457846
18:31:14	0:30:55	66.6930524	68.3812876	67.731787	59.2395153	69.8457846
18:31:15	0:30:56	66.6930524	69.3812876	66.731787	60.2395153	69.8457846
18:31:16	0:30:57	66.6930524	69.3812876	66.731787	60.2395153	70.8457846
18:31:17	0:30:58	64.6930524	69.3812876	67.731787	60.2395153	70.8457846
18:31:18	0:30:59	64.6930524	69.3812876	68.731787	60.2395153	70.8457846
18:31:19	0:31:00	65.6930524	70.3812876	69.731787	60.2395153	70.8457846
18:31:20	0:31:01	65.6930524	70.3812876	69.731787	60.2395153	73.8457846
18:31:21	0:31:02	65.6930524	70.3812876	69.731787	60.2395153	75.8457846
18:31:22	0:31:03	67.6930524	70.3812876	70.731787	60.2395153	75.8457846
18:31:23	0:31:04	67.6930524	70.3812876	70.731787	61.2395153	75.8457846
18:31:24	0:31:05	67.6930524	68.3812876	70.731787	61.2395153	78.8457846
18:31:25	0:31:06	67.6930524	68.3812876	71.731787	63.2395153	79.8457846
18:31:26	0:31:07	67.6930524	68.3812876	71.731787	63.2395153	79.8457846
18:31:27	0:31:08	69.6930524	68.3812876	73.731787	63.2395153	79.8457846
18:31:28	0:31:09	69.6930524	68.3812876	73.731787	63.2395153	79.8457846
18:31:29	0:31:10	69.6930524	68.3812876	79.731787	62.2395153	79.8457846
18:31:30	0:31:11	69.6930524	68.3812876	79.731787	62.2395153	79.8457846
18:31:31	0:31:12	71.6930524	65.3812876	83.731787	62.2395153	73.8457846
18:31:32	0:31:13	73.6930524	64.3812876	83.731787	62.2395153	69.8457846
18:31:33	0:31:14	73.6930524	64.3812876	83.731787	62.2395153	66.8457846
18:31:34	0:31:15	73.6930524	64.3812876	83.731787	62.2395153	66.8457846
18:31:35	0:31:16	71.6930524	63.3812876	83.731787	62.2395153	67.8457846
18:31:36	0:31:17	71.6930524	63.3812876	84.731787	62.2395153	69.8457846
18:31:37	0:31:18	71.6930524	63.3812876	84.731787	62.2395153	69.8457846
18:31:38	0:31:19	71.6930524	63.3812876	84.731787	62.2395153	67.8457846
18:31:39	0:31:20	71.6930524	63.3812876	84.731787	62.2395153	65.8457846
18:31:40	0:31:21	69.6930524	63.3812876	84.731787	62.2395153	64.8457846
18:31:41	0:31:22	69.6930524	61.3812876	83.731787	63.2395153	64.8457846
18:31:42	0:31:23	69.6930524	61.3812876	83.731787	63.2395153	65.8457846

18:31:43	0:31:24	68.6930524	61.3812876	83.731787	65.2395153	67.8457846
18:31:44	0:31:25	68.6930524	61.3812876	82.731787	65.2395153	69.8457846
18:31:45	0:31:26	68.6930524	61.3812876	82.731787	66.2395153	69.8457846
18:31:46	0:31:27	68.6930524	61.3812876	83.731787	69.2395153	68.8457846
18:31:47	0:31:28	69.6930524	61.3812876	83.731787	69.2395153	68.8457846
18:31:48	0:31:29	69.6930524	62.3812876	84.731787	69.2395153	67.8457846
18:31:49	0:31:30	69.6930524	62.3812876	85.731787	69.2395153	67.8457846
18:31:50	0:31:31	69.6930524	63.3812876	85.731787	69.2395153	68.8457846
18:31:51	0:31:32	69.6930524	64.3812876	85.731787	69.2395153	71.8457846
18:31:52	0:31:33	68.6930524	64.3812876	70.2951505	69.2395153	72.8457846
18:31:53	0:31:34	68.6930524	64.3812876	70.1701505	68.2395153	72.8457846
18:31:54	0:31:35	67.6930524	68.3812876	86.731787	69.2395153	72.8457846
18:31:55	0:31:36	67.6930524	68.3812876	73.731787	69.2395153	73.8457846
18:31:56	0:31:37	67.6930524	69.3812876	69.731787	70.2395153	75.8457846
18:31:57	0:31:38	66.6930524	71.3812876	67.731787	72.2395153	75.8457846
18:31:58	0:31:39	67.6930524	71.3812876	67.731787	71.2395153	75.8457846
18:31:59	0:31:40	67.6930524	71.3812876	67.731787	71.2395153	73.8457846
18:32:00	0:31:41	67.6930524	74.3812876	67.731787	72.2395153	71.8457846
18:32:01	0:31:42	67.6930524	74.3812876	67.731787	73.2395153	71.8457846
18:32:02	0:31:43	67.6930524	74.3812876	67.731787	73.2395153	70.8457846
18:32:03	0:31:44	67.6930524	76.3812876	67.731787	74.2395153	71.8457846
18:32:04	0:31:45	67.6930524	76.3812876	67.731787	76.2395153	72.8457846
18:32:05	0:31:46	66.6930524	76.3812876	67.731787	78.2395153	74.8457846
18:32:06	0:31:47	66.6930524	74.3812876	67.731787	81.2395153	76.8457846
18:32:07	0:31:48	67.6930524	74.3812876	67.731787	81.2395153	77.8457846
18:32:08	0:31:49	67.6930524	72.3812876	67.731787	81.2395153	77.8457846
18:32:09	0:31:50	68.6930524	72.3812876	67.731787	80.2395153	76.8457846
18:32:10	0:31:51	68.6930524	72.3812876	67.731787	80.2395153	76.8457846
18:32:11	0:31:52	68.6930524	71.3812876	67.731787	76.2395153	76.8457846
18:32:12	0:31:53	68.6930524	70.3812876	67.731787	76.2395153	76.8457846
18:32:13	0:31:54	68.6930524	70.3812876	67.731787	73.2395153	74.8457846
18:32:14	0:31:55	68.6930524	70.3812876	67.731787	70.2395153	73.8457846
18:32:15	0:31:56	67.6930524	70.3812876	67.731787	70.2395153	73.8457846
18:32:16	0:31:57	67.6930524	67.3812876	67.731787	68.2395153	73.8457846
18:32:17	0:31:58	67.6930524	66.3812876	66.731787	68.2395153	74.8457846
18:32:18	0:31:59	67.6930524	66.3812876	67.731787	67.2395153	74.8457846
18:32:19	0:32:00	66.6930524	65.3812876	67.731787	67.2395153	74.8457846
18:32:20	0:32:01	65.6930524	64.3812876	68.731787	68.2395153	74.8457846
18:32:21	0:32:02	63.6930524	64.3812876	68.731787	68.2395153	74.8457846
18:32:22	0:32:03	65.6930524	64.3812876	68.731787	70.2395153	74.8457846
18:32:23	0:32:04	65.6930524	63.3812876	67.731787	73.2395153	74.8457846
18:32:24	0:32:05	65.6930524	63.3812876	67.731787	73.2395153	74.8457846
18:32:25	0:32:06	65.6930524	62.3812876	68.731787	75.2395153	79.8457846
18:32:26	0:32:07	65.6930524	62.3812876	68.731787	76.2395153	78.8457846
18:32:27	0:32:08	65.6930524	62.3812876	68.731787	76.2395153	77.8457846

18:32:28	0:32:09	65.6930524	62.3812876	69.731787	80.2395153	76.8457846
18:32:29	0:32:10	65.6930524	62.3812876	70.731787	82.2395153	76.8457846
18:32:30	0:32:11	65.6930524	62.3812876	70.731787	83.2395153	74.8457846
18:32:31	0:32:12	65.6930524	62.3812876	70.731787	83.2395153	74.8457846
18:32:32	0:32:13	65.6930524	62.3812876	70.731787	84.2395153	75.8457846
18:32:33	0:32:14	65.6930524	62.3812876	70.731787	69.6701505	75.8457846
18:32:34	0:32:15	65.6930524	63.3812876	70.731787	69.7951505	75.8457846
18:32:35	0:32:16	64.6930524	63.3812876	68.731787	69.2951505	75.8457846
18:32:36	0:32:17	64.6930524	63.3812876	68.731787	69.2951505	75.8457846
18:32:37	0:32:18	64.6930524	64.3812876	68.731787	80.2395153	74.8457846
18:32:38	0:32:19	65.6930524	64.3812876	68.731787	80.2395153	74.8457846
18:32:39	0:32:20	65.6930524	65.3812876	68.731787	80.2395153	74.8457846
18:32:40	0:32:21	66.6930524	65.3812876	67.731787	79.2395153	74.8457846
18:32:41	0:32:22	67.6930524	65.3812876	68.731787	79.2395153	74.8457846
18:32:42	0:32:23	67.6930524	65.3812876	70.731787	75.2395153	74.8457846
18:32:43	0:32:24	69.6930524	65.3812876	76.731787	72.2395153	76.8457846
18:32:44	0:32:25	69.6930524	66.3812876	77.731787	68.2395153	68.8457846
18:32:45	0:32:26	72.6930524	67.3812876	77.731787	66.2395153	68.8457846
18:32:46	0:32:27	73.6930524	67.3812876	78.731787	66.2395153	63.8457846
18:32:47	0:32:28	71.2951505	67.3812876	78.731787	66.2395153	62.8457846
18:32:48	0:32:29	72.5451505	67.3812876	78.731787	66.2395153	63.8457846
18:32:49	0:32:30	72.6701505	67.3812876	77.731787	64.2395153	63.8457846
18:32:50	0:32:31	73.0451505	67.3812876	77.731787	63.2395153	66.8457846
18:32:51	0:32:32	73.5451505	67.3812876	76.731787	63.2395153	68.8457846
18:32:52	0:32:33	74.0451505	67.3812876	75.731787	63.2395153	68.8457846
18:32:53	0:32:34	74.2951505	68.3812876	75.731787	63.2395153	68.8457846
18:32:54	0:32:35	75.6701505	77.3812876	74.731787	63.2395153	68.8457846
18:32:55	0:32:36	76.1701505	79.3812876	73.731787	63.2395153	68.8457846
18:32:56	0:32:37	76.0451505	81.3812876	73.731787	63.2395153	67.8457846
18:32:57	0:32:38	76.2951505	82.3812876	73.731787	63.2395153	68.8457846
18:32:58	0:32:39	76.6701505	83.3812876	73.731787	63.2395153	68.8457846
18:32:59	0:32:40	76.7951505	83.3812876	72.731787	63.2395153	68.8457846
18:33:00	0:32:41	76.5451505	83.3812876	72.731787	64.2395153	67.8457846
18:33:01	0:32:42	75.1701505	83.3812876	72.731787	64.2395153	67.8457846
18:33:02	0:32:43	74.1701505	79.3812876	72.731787	64.2395153	66.8457846
18:33:03	0:32:44	73.9201505	78.3812876	72.731787	66.2395153	66.8457846
18:33:04	0:32:45	73.1701505	77.3812876	72.731787	66.2395153	66.8457846
18:33:05	0:32:46	72.6701505	77.3812876	72.731787	66.2395153	66.8457846
18:33:06	0:32:47	87.6930524	77.3812876	72.731787	70.2395153	67.8457846
18:33:07	0:32:48	86.6930524	76.3812876	72.731787	70.2395153	66.8457846
18:33:08	0:32:49	86.6930524	76.3812876	72.731787	70.2395153	66.8457846
18:33:09	0:32:50	85.6930524	76.3812876	72.731787	69.2395153	66.8457846
18:33:10	0:32:51	85.6930524	73.3812876	72.731787	68.2395153	66.8457846
18:33:11	0:32:52	70.2951505	70.3812876	72.731787	68.2395153	67.8457846
18:33:12	0:32:53	86.6930524	70.3812876	73.731787	68.2395153	68.8457846

18:33:13	0:32:54	73.0451505	70.3812876	76.731787	70.2395153	69.8457846
18:33:14	0:32:55	74.0451505	70.3812876	75.731787	72.2395153	74.8457846
18:33:15	0:32:56	74.0451505	69.3812876	76.731787	72.2395153	75.8457846
18:33:16	0:32:57	86.6930524	69.3812876	76.731787	72.2395153	75.8457846
18:33:17	0:32:58	84.6930524	69.3812876	76.731787	68.2395153	74.8457846
18:33:18	0:32:59	84.6930524	69.3812876	76.731787	68.2395153	74.8457846
18:33:19	0:33:00	82.6930524	68.3812876	77.731787	68.2395153	71.8457846
18:33:20	0:33:01	81.6930524	67.3812876	77.731787	68.2395153	71.8457846
18:33:21	0:33:02	81.6930524	67.3812876	78.731787	69.2395153	70.8457846
18:33:22	0:33:03	79.6930524	67.3812876	77.731787	69.2395153	69.8457846
18:33:23	0:33:04	77.6930524	65.3812876	76.731787	70.2395153	69.8457846
18:33:24	0:33:05	77.6930524	65.3812876	75.731787	70.2395153	68.8457846
18:33:25	0:33:06	74.6930524	65.3812876	75.731787	70.2395153	68.8457846
18:33:26	0:33:07	73.6930524	68.3812876	75.731787	70.2395153	67.8457846
18:33:27	0:33:08	72.6930524	69.3812876	74.731787	70.2395153	67.8457846
18:33:28	0:33:09	72.6930524	69.3812876	74.731787	70.2395153	66.8457846
18:33:29	0:33:10	72.6930524	69.3812876	72.731787	71.2395153	66.8457846
18:33:30	0:33:11	71.6930524	69.3812876	72.731787	72.2395153	66.8457846
18:33:31	0:33:12	72.6930524	69.3812876	72.731787	73.2395153	64.8457846
18:33:32	0:33:13	71.6930524	69.3812876	73.731787	74.2395153	63.8457846
18:33:33	0:33:14	71.6930524	69.3812876	73.731787	73.2395153	63.8457846
18:33:34	0:33:15	71.6930524	69.3812876	73.731787	72.2395153	64.8457846
18:33:35	0:33:16	70.6930524	69.3812876	73.731787	72.2395153	65.8457846
18:33:36	0:33:17	70.6930524	70.3812876	73.731787	72.2395153	66.8457846
18:33:37	0:33:18	70.6930524	71.3812876	73.731787	72.2395153	66.8457846
18:33:38	0:33:19	70.6930524	71.3812876	74.731787	72.2395153	66.8457846
18:33:39	0:33:20	70.6930524	71.3812876	74.731787	72.2395153	64.8457846
18:33:40	0:33:21	69.6930524	72.3812876	74.731787	71.2395153	64.8457846
18:33:41	0:33:22	69.6930524	72.3812876	74.731787	71.2395153	64.8457846
18:33:42	0:33:23	70.6930524	72.3812876	75.731787	71.2395153	65.8457846
18:33:43	0:33:24	69.6930524	73.3812876	75.731787	71.2395153	65.8457846
18:33:44	0:33:25	69.6930524	71.3812876	75.731787	70.2395153	66.8457846
18:33:45	0:33:26	69.6930524	71.3812876	73.731787	70.2395153	67.8457846
18:33:46	0:33:27	68.6930524	71.3812876	73.731787	71.2395153	66.8457846
18:33:47	0:33:28	68.6930524	71.3812876	72.731787	71.2395153	66.8457846
18:33:48	0:33:29	68.6930524	71.3812876	72.731787	71.2395153	66.8457846
18:33:49	0:33:30	68.6930524	71.3812876	72.731787	68.2395153	67.8457846
18:33:50	0:33:31	68.6930524	72.3812876	72.731787	67.2395153	67.8457846
18:33:51	0:33:32	68.6930524	72.3812876	72.731787	67.2395153	66.8457846
18:33:52	0:33:33	68.6930524	72.3812876	71.731787	66.2395153	66.8457846
18:33:53	0:33:34	68.6930524	73.3812876	71.731787	65.2395153	67.8457846
18:33:54	0:33:35	69.6930524	75.3812876	71.731787	65.2395153	67.8457846
18:33:55	0:33:36	70.6930524	75.3812876	69.731787	66.2395153	67.8457846
18:33:56	0:33:37	70.6930524	75.3812876	68.731787	66.2395153	65.8457846
18:33:57	0:33:38	71.6930524	72.3812876	68.731787	66.2395153	65.8457846

18:33:58	0:33:39	71.6930524	71.3812876	68.731787	66.2395153	65.8457846
18:33:59	0:33:40	71.6930524	71.3812876	68.731787	66.2395153	65.8457846
18:34:00	0:33:41	71.6930524	71.3812876	67.731787	66.2395153	65.8457846
18:34:01	0:33:42	71.6930524	71.3812876	66.731787	67.2395153	64.8457846
18:34:02	0:33:43	71.6930524	72.3812876	66.731787	68.2395153	64.8457846
18:34:03	0:33:44	71.6930524	72.3812876	69.731787	68.2395153	63.8457846
18:34:04	0:33:45	70.6930524	72.3812876	67.731787	68.2395153	61.8457846
18:34:05	0:33:46	70.6930524	72.3812876	66.731787	70.2395153	61.8457846
18:34:06	0:33:47	70.6930524	72.3812876	65.731787	71.2395153	56.8457846
18:34:07	0:33:48	70.6930524	72.3812876	65.731787	70.2395153	55.8457846
18:34:08	0:33:49	69.6930524	72.3812876	65.731787	70.2395153	55.8457846
18:34:09	0:33:50	70.6930524	72.3812876	65.731787	67.2395153	55.8457846
18:34:10	0:33:51	71.6930524	72.3812876	65.731787	66.2395153	54.8457846
18:34:11	0:33:52	71.6930524	71.3812876	65.731787	66.2395153	53.8457846
18:34:12	0:33:53	71.6930524	71.3812876	65.731787	66.2395153	53.8457846
18:34:13	0:33:54	70.6930524	71.3812876	65.731787	65.2395153	52.8457846
18:34:14	0:33:55	71.6930524	71.3812876	65.731787	65.2395153	51.8457846
18:34:15	0:33:56	71.6930524	71.3812876	65.731787	65.2395153	51.8457846
18:34:16	0:33:57	71.6930524	72.3812876	65.731787	66.2395153	50.8457846
18:34:17	0:33:58	71.6930524	72.3812876	65.731787	68.2395153	50.8457846
18:34:18	0:33:59	71.6930524	73.3812876	65.731787	70.2395153	50.8457846
18:34:19	0:34:00	71.6930524	73.3812876	65.731787	70.2395153	49.8457846
18:34:20	0:34:01	72.6930524	73.3812876	65.731787	71.2395153	48.8457846
18:34:21	0:34:02	72.6930524	71.3812876	65.731787	74.2395153	48.8457846
18:34:22	0:34:03	73.6930524	69.3812876	65.731787	75.2395153	48.8457846
18:34:23	0:34:04	73.6930524	68.3812876	65.731787	76.2395153	45.8457846
18:34:24	0:34:05	72.6930524	67.3812876	65.731787	77.2395153	58.8457846
18:34:25	0:34:06	72.6930524	67.3812876	65.731787	77.2395153	70.8457846
18:34:26	0:34:07	72.6930524	67.3812876	65.731787	77.2395153	70.8457846
18:34:27	0:34:08	72.6930524	67.3812876	66.731787	77.2395153	75.8457846
18:34:28	0:34:09	72.6930524	67.3812876	66.731787	77.2395153	77.8457846
18:34:29	0:34:10	67.6930524	67.3812876	65.731787	77.2395153	78.8457846
18:34:30	0:34:11	64.6930524	67.3812876	66.731787	76.2395153	78.8457846
18:34:31	0:34:12	63.6930524	66.3812876	66.731787	76.2395153	78.8457846
18:34:32	0:34:13	62.6930524	66.3812876	69.731787	74.2395153	78.8457846
18:34:33	0:34:14	62.6930524	67.3812876	69.731787	74.2395153	78.8457846
18:34:34	0:34:15	62.6930524	66.3812876	69.731787	74.2395153	79.8457846
18:34:35	0:34:16	61.6930524	66.3812876	69.731787	74.2395153	81.8457846
18:34:36	0:34:17	61.6930524	66.3812876	70.731787	74.2395153	81.8457846
18:34:37	0:34:18	61.6930524	70.3812876	70.731787	74.2395153	76.8457846
18:34:38	0:34:19	60.6930524	70.3812876	70.731787	74.2395153	74.8457846
18:34:39	0:34:20	59.6930524	76.3812876	70.731787	75.2395153	74.8457846
18:34:40	0:34:21	59.6930524	75.3812876	70.731787	75.2395153	74.8457846
18:34:41	0:34:22	59.6930524	75.3812876	71.731787	75.2395153	74.8457846
18:34:42	0:34:23	59.6930524	75.3812876	69.731787	75.2395153	70.8457846

18:34:43	0:34:24	59.6930524	75.3812876	68.731787	75.2395153	68.8457846
18:34:44	0:34:25	59.6930524	75.3812876	67.731787	75.2395153	68.8457846
18:34:45	0:34:26	59.6930524	77.3812876	65.731787	74.2395153	68.8457846
18:34:46	0:34:27	59.6930524	77.3812876	65.731787	73.2395153	68.8457846
18:34:47	0:34:28	58.6930524	78.3812876	63.731787	73.2395153	65.8457846
18:34:48	0:34:29	58.6930524	78.3812876	63.731787	73.2395153	63.8457846
18:34:49	0:34:30	57.6930524	77.3812876	62.731787	72.2395153	63.8457846
18:34:50	0:34:31	56.6930524	75.3812876	62.731787	69.2395153	63.8457846
18:34:51	0:34:32	56.6930524	75.3812876	62.731787	69.2395153	63.8457846
18:34:52	0:34:33	56.6930524	74.3812876	60.731787	69.2395153	64.8457846
18:34:53	0:34:34	56.6930524	74.3812876	59.731787	67.2395153	65.8457846
18:34:54	0:34:35	57.6930524	74.3812876	58.731787	67.2395153	65.8457846
18:34:55	0:34:36	57.6930524	73.3812876	58.731787	67.2395153	66.8457846
18:34:56	0:34:37	58.6930524	73.3812876	57.731787	67.2395153	68.8457846
18:34:57	0:34:38	58.6930524	73.3812876	55.731787	67.2395153	71.8457846
18:34:58	0:34:39	59.6930524	73.3812876	55.731787	68.2395153	72.8457846
18:34:59	0:34:40	59.6930524	73.3812876	61.731787	69.2395153	73.8457846
18:35:00	0:34:41	59.6930524	73.3812876	64.731787	70.2395153	70.8457846
18:35:01	0:34:42	59.6930524	73.3812876	65.731787	70.2395153	67.8457846
18:35:02	0:34:43	59.6930524	73.3812876	66.731787	70.2395153	67.8457846
18:35:03	0:34:44	59.6930524	71.3812876	66.731787	70.2395153	67.8457846
18:35:04	0:34:45	59.6930524	70.3812876	66.731787	70.2395153	67.8457846
18:35:05	0:34:46	59.6930524	69.3812876	66.731787	71.2395153	67.8457846
18:35:06	0:34:47	59.6930524	69.3812876	66.731787	71.2395153	67.8457846
18:35:07	0:34:48	59.6930524	69.3812876	66.731787	72.2395153	68.8457846
18:35:08	0:34:49	59.6930524	68.3812876	66.731787	72.2395153	70.8457846
18:35:09	0:34:50	58.6930524	71.3812876	67.731787	72.2395153	71.8457846
18:35:10	0:34:51	58.6930524	72.3812876	67.731787	72.2395153	74.8457846
18:35:11	0:34:52	58.6930524	73.3812876	67.731787	73.2395153	75.8457846
18:35:12	0:34:53	58.6930524	75.3812876	67.731787	73.2395153	76.8457846
18:35:13	0:34:54	57.6930524	76.3812876	67.731787	75.2395153	76.8457846
18:35:14	0:34:55	57.6930524	78.3812876	68.731787	75.2395153	76.8457846
18:35:15	0:34:56	57.6930524	78.3812876	69.731787	75.2395153	75.8457846
18:35:16	0:34:57	57.6930524	78.3812876	69.731787	77.2395153	74.8457846
18:35:17	0:34:58	57.6930524	78.3812876	69.731787	77.2395153	72.8457846
18:35:18	0:34:59	57.6930524	74.3812876	71.731787	79.2395153	70.8457846
18:35:19	0:35:00	57.6930524	73.3812876	71.731787	79.2395153	71.8457846
18:35:20	0:35:01	57.6930524	71.3812876	72.731787	79.2395153	72.8457846
18:35:21	0:35:02	57.6930524	71.3812876	75.731787	80.2395153	72.8457846
18:35:22	0:35:03	57.6930524	70.3812876	77.731787	80.2395153	69.8457846
18:35:23	0:35:04	57.6930524	70.3812876	78.731787	81.2395153	69.8457846
18:35:24	0:35:05	57.6930524	69.3812876	78.731787	81.2395153	69.8457846
18:35:25	0:35:06	57.6930524	69.3812876	78.731787	82.2395153	70.8457846
18:35:26	0:35:07	57.6930524	69.3812876	80.731787	83.2395153	71.8457846
18:35:27	0:35:08	57.6930524	69.3812876	80.731787	83.2395153	71.8457846

18:35:28	0:35:09	56.6930524	69.3812876	81.731787	82.2395153	71.8457846
18:35:29	0:35:10	55.6930524	70.3812876	83.731787	82.2395153	71.8457846
18:35:30	0:35:11	55.6930524	73.3812876	84.731787	82.2395153	71.8457846
18:35:31	0:35:12	56.6930524	73.3812876	85.731787	82.2395153	71.8457846
18:35:32	0:35:13	56.6930524	74.3812876	85.731787	85.2395153	70.8457846
18:35:33	0:35:14	56.6930524	74.3812876	86.731787	86.2395153	68.8457846
18:35:34	0:35:15	57.6930524	74.3812876	74.731787	70.2951505	67.8457846
18:35:35	0:35:16	57.6930524	67.3812876	73.731787	69.9201505	67.8457846
18:35:36	0:35:17	57.6930524	67.3812876	72.731787	69.9201505	67.8457846
18:35:37	0:35:18	57.6930524	67.3812876	71.731787	70.1701505	69.8457846
18:35:38	0:35:19	57.6930524	67.3812876	71.731787	69.6701505	71.8457846
18:35:39	0:35:20	57.6930524	67.3812876	71.731787	82.2395153	71.8457846
18:35:40	0:35:21	59.6930524	67.3812876	70.731787	82.2395153	69.8457846
18:35:41	0:35:22	61.6930524	67.3812876	69.731787	81.2395153	64.8457846
18:35:42	0:35:23	62.6930524	70.3812876	69.731787	78.2395153	62.8457846
18:35:43	0:35:24	64.6930524	70.3812876	69.731787	73.2395153	61.8457846
18:35:44	0:35:25	68.6930524	70.3812876	69.731787	65.2395153	62.8457846
18:35:45	0:35:26	70.6930524	71.3812876	70.731787	61.2395153	64.8457846
18:35:46	0:35:27	70.6930524	73.3812876	70.731787	60.2395153	65.8457846
18:35:47	0:35:28	70.6930524	75.3812876	70.731787	58.2395153	67.8457846
18:35:48	0:35:29	70.6930524	75.3812876	70.731787	59.2395153	69.8457846
18:35:49	0:35:30	70.6930524	73.3812876	70.731787	60.2395153	70.8457846
18:35:50	0:35:31	70.6930524	73.3812876	71.731787	61.2395153	71.8457846
18:35:51	0:35:32	71.6930524	73.3812876	71.731787	61.2395153	71.8457846
18:35:52	0:35:33	71.6930524	73.3812876	71.731787	61.2395153	71.8457846
18:35:53	0:35:34	71.6930524	73.3812876	71.731787	62.2395153	73.8457846
18:35:54	0:35:35	72.6930524	71.3812876	71.731787	62.2395153	73.8457846
18:35:55	0:35:36	72.6930524	70.3812876	73.731787	63.2395153	74.8457846
18:35:56	0:35:37	72.6930524	70.3812876	72.731787	64.2395153	74.8457846
18:35:57	0:35:38	68.6930524	70.3812876	71.731787	65.2395153	74.8457846
18:35:58	0:35:39	66.6930524	70.3812876	71.731787	65.2395153	74.8457846
18:35:59	0:35:40	66.6930524	70.3812876	72.731787	65.2395153	74.8457846
18:36:00	0:35:41	66.6930524	70.3812876	72.731787	65.2395153	74.8457846
18:36:01	0:35:42	66.6930524	69.3812876	70.731787	65.2395153	74.8457846
18:36:02	0:35:43	66.6930524	69.3812876	69.731787	65.2395153	74.8457846
18:36:03	0:35:44	67.6930524	69.3812876	68.731787	64.2395153	74.8457846
18:36:04	0:35:45	68.6930524	69.3812876	68.731787	63.2395153	74.8457846
18:36:05	0:35:46	67.6930524	70.3812876	68.731787	62.2395153	72.8457846
18:36:06	0:35:47	67.6930524	70.3812876	69.731787	62.2395153	71.8457846
18:36:07	0:35:48	66.6930524	70.3812876	69.731787	62.2395153	71.8457846
18:36:08	0:35:49	65.6930524	70.3812876	69.731787	62.2395153	71.8457846
18:36:09	0:35:50	64.6930524	71.3812876	70.731787	62.2395153	71.8457846
18:36:10	0:35:51	64.6930524	71.3812876	71.731787	62.2395153	72.8457846
18:36:11	0:35:52	63.6930524	71.3812876	72.731787	62.2395153	72.8457846
18:36:12	0:35:53	63.6930524	72.3812876	72.731787	64.2395153	72.8457846

18:36:13	0:35:54	62.6930524	75.3812876	72.731787	66.2395153	72.8457846
18:36:14	0:35:55	62.6930524	75.3812876	73.731787	66.2395153	73.8457846
18:36:15	0:35:56	62.6930524	74.3812876	74.731787	66.2395153	72.8457846
18:36:16	0:35:57	62.6930524	74.3812876	75.731787	66.2395153	67.8457846
18:36:17	0:35:58	62.6930524	74.3812876	75.731787	66.2395153	66.8457846
18:36:18	0:35:59	61.6930524	74.3812876	75.731787	64.2395153	67.8457846
18:36:19	0:36:00	57.6930524	69.3812876	75.731787	64.2395153	70.8457846
18:36:20	0:36:01	56.6930524	67.3812876	76.731787	64.2395153	71.8457846
18:36:21	0:36:02	56.6930524	66.3812876	76.731787	63.2395153	71.8457846
18:36:22	0:36:03	56.6930524	66.3812876	75.731787	62.2395153	70.8457846
18:36:23	0:36:04	56.6930524	66.3812876	74.731787	62.2395153	70.8457846
18:36:24	0:36:05	56.6930524	67.3812876	74.731787	63.2395153	69.8457846
18:36:25	0:36:06	56.6930524	67.3812876	73.731787	64.2395153	69.8457846
18:36:26	0:36:07	56.6930524	67.3812876	72.731787	65.2395153	69.8457846
18:36:27	0:36:08	56.6930524	67.3812876	71.731787	66.2395153	69.8457846
18:36:28	0:36:09	57.6930524	67.3812876	71.731787	66.2395153	69.8457846
18:36:29	0:36:10	57.6930524	67.3812876	70.731787	66.2395153	71.8457846
18:36:30	0:36:11	57.6930524	67.3812876	70.731787	67.2395153	71.8457846
18:36:31	0:36:12	57.6930524	67.3812876	70.731787	68.2395153	72.8457846
18:36:32	0:36:13	55.6930524	67.3812876	70.731787	69.2395153	70.8457846
18:36:33	0:36:14	55.6930524	67.3812876	70.731787	69.2395153	67.8457846
18:36:34	0:36:15	55.6930524	67.3812876	70.731787	72.2395153	64.8457846
18:36:35	0:36:16	55.6930524	67.3812876	70.731787	72.2395153	62.8457846
18:36:36	0:36:17	56.6930524	67.3812876	71.731787	72.2395153	60.8457846
18:36:37	0:36:18	57.6930524	67.3812876	71.731787	72.2395153	60.8457846
18:36:38	0:36:19	57.6930524	67.3812876	69.731787	72.2395153	62.8457846
18:36:39	0:36:20	57.6930524	67.3812876	68.731787	72.2395153	64.8457846
18:36:40	0:36:21	57.6930524	67.3812876	68.731787	66.2395153	63.8457846
18:36:41	0:36:22	58.6930524	67.3812876	68.731787	65.2395153	62.8457846
18:36:42	0:36:23	58.6930524	65.3812876	68.731787	65.2395153	61.8457846
18:36:43	0:36:24	57.6930524	65.3812876	68.731787	66.2395153	61.8457846
18:36:44	0:36:25	57.6930524	63.3812876	69.731787	66.2395153	61.8457846
18:36:45	0:36:26	57.6930524	62.3812876	70.731787	65.2395153	61.8457846
18:36:46	0:36:27	57.6930524	62.3812876	70.731787	65.2395153	63.8457846
18:36:47	0:36:28	57.6930524	59.3812876	69.731787	65.2395153	64.8457846
18:36:48	0:36:29	57.6930524	59.3812876	68.731787	65.2395153	65.8457846
18:36:49	0:36:30	57.6930524	59.3812876	68.731787	64.2395153	65.8457846
18:36:50	0:36:31	57.6930524	61.3812876	68.731787	64.2395153	66.8457846
18:36:51	0:36:32	57.6930524	62.3812876	68.731787	64.2395153	68.8457846
18:36:52	0:36:33	58.6930524	63.3812876	68.731787	65.2395153	66.8457846
18:36:53	0:36:34	58.6930524	63.3812876	67.731787	66.2395153	65.8457846
18:36:54	0:36:35	58.6930524	63.3812876	67.731787	66.2395153	65.8457846
18:36:55	0:36:36	58.6930524	66.3812876	66.731787	69.2395153	65.8457846
18:36:56	0:36:37	58.6930524	66.3812876	64.731787	70.2395153	66.8457846
18:36:57	0:36:38	58.6930524	67.3812876	64.731787	71.2395153	66.8457846

18:36:58	0:36:39	58.6930524	67.3812876	64.731787	71.2395153	66.8457846
18:36:59	0:36:40	58.6930524	67.3812876	63.731787	71.2395153	67.8457846
18:37:00	0:36:41	58.6930524	68.3812876	62.731787	71.2395153	68.8457846
18:37:01	0:36:42	58.6930524	68.3812876	62.731787	70.2395153	69.8457846
18:37:02	0:36:43	59.6930524	71.3812876	62.731787	70.2395153	69.8457846
18:37:03	0:36:44	59.6930524	71.3812876	62.731787	70.2395153	69.8457846
18:37:04	0:36:45	66.6930524	71.3812876	62.731787	69.2395153	69.8457846
18:37:05	0:36:46	72.1701505	72.3812876	70.731787	70.2395153	70.8457846
18:37:06	0:36:47	74.0451505	72.3812876	70.731787	70.2395153	70.8457846
18:37:07	0:36:48	75.4201505	72.3812876	71.731787	70.2395153	72.8457846
18:37:08	0:36:49	75.6701505	72.3812876	72.731787	68.2395153	72.8457846
18:37:09	0:36:50	76.1701505	72.3812876	73.731787	67.2395153	71.8457846
18:37:10	0:36:51	76.6701505	72.3812876	74.731787	67.2395153	70.8457846
18:37:11	0:36:52	77.2951505	72.3812876	74.731787	67.2395153	71.8457846
18:37:12	0:36:53	77.4201505	71.3812876	74.731787	67.2395153	71.8457846
18:37:13	0:36:54	77.9201505	71.3812876	73.731787	67.2395153	71.8457846
18:37:14	0:36:55	78.0451505	71.3812876	72.731787	66.2395153	71.8457846
18:37:15	0:36:56	77.6701505	69.3812876	72.731787	66.2395153	71.8457846
18:37:16	0:36:57	77.4201505	69.3812876	71.731787	65.2395153	73.8457846
18:37:17	0:36:58	77.5451505	69.3812876	70.731787	64.2395153	76.8457846
18:37:18	0:36:59	77.2951505	67.3812876	69.731787	64.2395153	78.8457846
18:37:19	0:37:00	76.6701505	63.3812876	68.731787	64.2395153	78.8457846
18:37:20	0:37:01	75.9201505	60.3812876	67.731787	64.2395153	76.8457846
18:37:21	0:37:02	75.2951505	60.3812876	67.731787	64.2395153	73.8457846
18:37:22	0:37:03	75.0451505	60.3812876	67.731787	64.2395153	71.8457846
18:37:23	0:37:04	74.7951505	59.3812876	67.731787	64.2395153	70.8457846
18:37:24	0:37:05	74.5451505	58.3812876	67.731787	64.2395153	69.8457846
18:37:25	0:37:06	74.4201505	58.3812876	67.731787	64.2395153	70.8457846
18:37:26	0:37:07	74.4201505	59.3812876	67.731787	67.2395153	69.8457846
18:37:27	0:37:08	75.2951505	68.3812876	67.731787	69.2395153	67.8457846
18:37:28	0:37:09	75.5451505	68.3812876	68.731787	75.2395153	67.8457846
18:37:29	0:37:10	74.6701505	68.3812876	68.731787	75.2395153	65.8457846
18:37:30	0:37:11	75.1701505	68.3812876	68.731787	81.2395153	65.8457846
18:37:31	0:37:12	75.1701505	67.3812876	67.731787	83.2395153	65.8457846
18:37:32	0:37:13	86.6930524	67.3812876	66.731787	85.2395153	66.8457846
18:37:33	0:37:14	82.6930524	67.3812876	63.731787	86.2395153	67.8457846
18:37:34	0:37:15	79.6930524	67.3812876	63.731787	87.2395153	67.8457846
18:37:35	0:37:16	75.6930524	66.3812876	66.731787	72.9201505	67.8457846
18:37:36	0:37:17	72.6930524	64.3812876	66.731787	72.9201505	67.8457846
18:37:37	0:37:18	70.6930524	64.3812876	67.731787	73.0451505	67.8457846
18:37:38	0:37:19	67.6930524	61.3812876	67.731787	72.6701505	68.8457846
18:37:39	0:37:20	66.6930524	61.3812876	67.731787	72.6701505	68.8457846
18:37:40	0:37:21	66.6930524	61.3812876	67.731787	72.6701505	68.8457846
18:37:41	0:37:22	63.6930524	59.3812876	68.731787	71.4201505	68.8457846
18:37:42	0:37:23	61.6930524	59.3812876	68.731787	70.4201505	68.8457846

18:37:43	0:37:24	61.6930524	59.3812876	68.731787	69.9201505	68.8457846
18:37:44	0:37:25	60.6930524	58.3812876	69.731787	69.0451505	67.8457846
18:37:45	0:37:26	59.6930524	58.3812876	69.731787	69.0451505	67.8457846
18:37:46	0:37:27	59.6930524	58.3812876	69.731787	68.9201505	67.8457846
18:37:47	0:37:28	59.6930524	58.3812876	69.731787	69.0451505	67.8457846
18:37:48	0:37:29	59.6930524	58.3812876	69.731787	69.0451505	67.8457846
18:37:49	0:37:30	59.6930524	58.3812876	69.731787	68.6701505	67.8457846
18:37:50	0:37:31	59.6930524	60.3812876	69.731787	68.9201505	66.8457846
18:37:51	0:37:32	59.6930524	63.3812876	68.731787	68.9201505	65.8457846
18:37:52	0:37:33	59.6930524	63.3812876	68.731787	68.9201505	65.8457846
18:37:53	0:37:34	59.6930524	64.3812876	70.731787	85.2395153	66.8457846
18:37:54	0:37:35	58.6930524	64.3812876	71.731787	83.2395153	65.8457846
18:37:55	0:37:36	58.6930524	64.3812876	71.731787	83.2395153	66.8457846
18:37:56	0:37:37	58.6930524	70.3812876	71.731787	82.2395153	66.8457846
18:37:57	0:37:38	58.6930524	71.3812876	71.731787	81.2395153	65.8457846
18:37:58	0:37:39	58.6930524	71.3812876	74.731787	81.2395153	65.8457846
18:37:59	0:37:40	58.6930524	72.3812876	75.731787	81.2395153	65.8457846
18:38:00	0:37:41	58.6930524	72.3812876	74.731787	81.2395153	64.8457846
18:38:01	0:37:42	58.6930524	72.3812876	73.731787	81.2395153	64.8457846
18:38:02	0:37:43	59.6930524	74.3812876	71.731787	81.2395153	63.8457846
18:38:03	0:37:44	60.6930524	75.3812876	70.731787	83.2395153	62.8457846
18:38:04	0:37:45	60.6930524	78.3812876	70.731787	84.2395153	62.8457846
18:38:05	0:37:46	60.6930524	78.3812876	71.731787	86.2395153	62.8457846
18:38:06	0:37:47	60.6930524	79.3812876	71.731787	87.2395153	63.8457846
18:38:07	0:37:48	60.6930524	79.3812876	72.731787	87.2395153	64.8457846
18:38:08	0:37:49	61.6930524	79.3812876	72.731787	88.2395153	65.8457846
18:38:09	0:37:50	61.6930524	79.3812876	75.731787	88.2395153	66.8457846
18:38:10	0:37:51	62.6930524	78.3812876	75.731787	88.2395153	66.8457846
18:38:11	0:37:52	62.6930524	78.3812876	76.731787	88.2395153	66.8457846
18:38:12	0:37:53	62.6930524	70.3812876	76.731787	88.2395153	66.8457846
18:38:13	0:37:54	62.6930524	68.3812876	76.731787	88.2395153	66.8457846
18:38:14	0:37:55	61.6930524	66.3812876	72.731787	88.2395153	66.8457846
18:38:15	0:37:56	61.6930524	65.3812876	71.731787	85.2395153	67.8457846
18:38:16	0:37:57	61.6930524	65.3812876	71.731787	80.2395153	68.8457846
18:38:17	0:37:58	61.6930524	65.3812876	71.731787	75.2395153	69.8457846
18:38:18	0:37:59	61.6930524	66.3812876	71.731787	75.2395153	69.8457846
18:38:19	0:38:00	61.6930524	67.3812876	71.731787	75.2395153	69.8457846
18:38:20	0:38:01	61.6930524	65.3812876	71.731787	75.2395153	68.8457846
18:38:21	0:38:02	61.6930524	65.3812876	71.731787	75.2395153	68.8457846
18:38:22	0:38:03	60.6930524	63.3812876	71.731787	74.2395153	68.8457846
18:38:23	0:38:04	60.6930524	62.3812876	72.731787	74.2395153	69.8457846
18:38:24	0:38:05	60.6930524	61.3812876	72.731787	74.2395153	69.8457846
18:38:25	0:38:06	60.6930524	61.3812876	72.731787	74.2395153	70.8457846
18:38:26	0:38:07	60.6930524	60.3812876	72.731787	76.2395153	70.8457846
18:38:27	0:38:08	60.6930524	61.3812876	73.731787	83.2395153	69.8457846

18:38:28	0:38:09	59.6930524	63.3812876	74.731787	84.2395153	68.8457846
18:38:29	0:38:10	59.6930524	65.3812876	75.731787	85.2395153	68.8457846
18:38:30	0:38:11	59.6930524	65.3812876	75.731787	87.2395153	67.8457846
18:38:31	0:38:12	59.6930524	66.3812876	74.731787	88.2395153	67.8457846
18:38:32	0:38:13	59.6930524	67.3812876	74.731787	72.4201505	67.8457846
18:38:33	0:38:14	59.6930524	67.3812876	73.731787	72.0451505	66.8457846
18:38:34	0:38:15	58.6930524	67.3812876	73.731787	71.4201505	65.8457846
18:38:35	0:38:16	56.6930524	68.3812876	72.731787	70.9201505	64.8457846
18:38:36	0:38:17	56.6930524	68.3812876	72.731787	71.1701505	66.8457846
18:38:37	0:38:18	56.6930524	68.3812876	72.731787	71.4201505	68.8457846
18:38:38	0:38:19	57.6930524	68.3812876	71.731787	71.4201505	68.8457846
18:38:39	0:38:20	58.6930524	67.3812876	71.731787	71.4201505	68.8457846
18:38:40	0:38:21	58.6930524	67.3812876	70.731787	71.6701505	69.8457846
18:38:41	0:38:22	58.6930524	66.3812876	69.731787	71.5451505	70.8457846
18:38:42	0:38:23	58.6930524	66.3812876	69.731787	71.5451505	70.8457846
18:38:43	0:38:24	58.6930524	66.3812876	67.731787	70.7951505	69.8457846
18:38:44	0:38:25	59.6930524	66.3812876	67.731787	70.6701505	69.8457846
18:38:45	0:38:26	59.6930524	66.3812876	67.731787	84.2395153	69.8457846
18:38:46	0:38:27	59.6930524	66.3812876	68.731787	83.2395153	69.8457846
18:38:47	0:38:28	60.6930524	64.3812876	68.731787	81.2395153	68.8457846
18:38:48	0:38:29	60.6930524	63.3812876	68.731787	78.2395153	69.8457846
18:38:49	0:38:30	60.6930524	64.3812876	68.731787	76.2395153	69.8457846
18:38:50	0:38:31	61.6930524	65.3812876	73.731787	75.2395153	69.8457846
18:38:51	0:38:32	61.6930524	65.3812876	74.731787	74.2395153	69.8457846
18:38:52	0:38:33	62.6930524	65.3812876	75.731787	74.2395153	69.8457846
18:38:53	0:38:34	63.6930524	66.3812876	75.731787	73.2395153	68.8457846
18:38:54	0:38:35	63.6930524	73.3812876	75.731787	73.2395153	67.8457846
18:38:55	0:38:36	62.6930524	79.3812876	74.731787	72.2395153	67.8457846
18:38:56	0:38:37	62.6930524	79.3812876	72.731787	67.2395153	67.8457846
18:38:57	0:38:38	62.6930524	79.3812876	72.731787	67.2395153	67.8457846
18:38:58	0:38:39	62.6930524	79.3812876	72.731787	67.2395153	67.8457846
18:38:59	0:38:40	61.6930524	78.3812876	71.731787	66.2395153	67.8457846
18:39:00	0:38:41	61.6930524	77.3812876	71.731787	65.2395153	65.8457846
18:39:01	0:38:42	60.6930524	77.3812876	71.731787	64.2395153	64.8457846
18:39:02	0:38:43	60.6930524	75.3812876	71.731787	64.2395153	63.8457846
18:39:03	0:38:44	60.6930524	75.3812876	71.731787	64.2395153	62.8457846
18:39:04	0:38:45	61.6930524	75.3812876	71.731787	64.2395153	62.8457846
18:39:05	0:38:46	61.6930524	74.3812876	71.731787	64.2395153	63.8457846
18:39:06	0:38:47	60.6930524	74.3812876	70.731787	66.2395153	63.8457846
18:39:07	0:38:48	60.6930524	74.3812876	70.731787	67.2395153	64.8457846
18:39:08	0:38:49	59.6930524	73.3812876	70.731787	67.2395153	64.8457846
18:39:09	0:38:50	59.6930524	73.3812876	71.731787	67.2395153	65.8457846
18:39:10	0:38:51	59.6930524	74.3812876	71.731787	68.2395153	65.8457846
18:39:11	0:38:52	59.6930524	74.3812876	71.731787	68.2395153	65.8457846
18:39:12	0:38:53	59.6930524	78.3812876	71.731787	67.2395153	65.8457846

18:39:13	0:38:54	59.6930524	77.3812876	71.731787	67.2395153	66.8457846
18:39:14	0:38:55	59.6930524	75.3812876	71.731787	67.2395153	67.8457846
18:39:15	0:38:56	59.6930524	75.3812876	71.731787	65.2395153	67.8457846
18:39:16	0:38:57	58.6930524	73.3812876	72.731787	65.2395153	67.8457846
18:39:17	0:38:58	58.6930524	70.3812876	72.731787	65.2395153	66.8457846
18:39:18	0:38:59	58.6930524	70.3812876	72.731787	67.2395153	66.8457846
18:39:19	0:39:00	58.6930524	69.3812876	72.731787	67.2395153	67.8457846
18:39:20	0:39:01	58.6930524	70.3812876	72.731787	67.2395153	67.8457846
18:39:21	0:39:02	58.6930524	71.3812876	72.731787	69.2395153	67.8457846
18:39:22	0:39:03	60.6930524	71.3812876	72.731787	70.2395153	67.8457846
18:39:23	0:39:04	61.6930524	71.3812876	72.731787	71.2395153	67.8457846
18:39:24	0:39:05	61.6930524	71.3812876	72.731787	72.2395153	67.8457846
18:39:25	0:39:06	61.6930524	71.3812876	72.731787	72.2395153	69.8457846
18:39:26	0:39:07	60.6930524	72.3812876	71.731787	72.2395153	68.8457846
18:39:27	0:39:08	60.6930524	72.3812876	71.731787	72.2395153	66.8457846
18:39:28	0:39:09	60.6930524	72.3812876	71.731787	73.2395153	65.8457846
18:39:29	0:39:10	59.6930524	73.3812876	71.731787	74.2395153	65.8457846
18:39:30	0:39:11	59.6930524	75.3812876	71.731787	74.2395153	64.8457846
18:39:31	0:39:12	59.6930524	76.3812876	71.731787	74.2395153	64.8457846
18:39:32	0:39:13	58.6930524	71.3812876	71.731787	74.2395153	63.8457846
18:39:33	0:39:14	61.6930524	72.3812876	72.731787	74.2395153	63.8457846
18:39:34	0:39:15	62.6930524	73.3812876	72.731787	77.2395153	65.8457846
18:39:35	0:39:16	62.6930524	74.3812876	74.731787	77.2395153	65.8457846
18:39:36	0:39:17	62.6930524	74.3812876	74.731787	77.2395153	65.8457846
18:39:37	0:39:18	62.6930524	75.3812876	75.731787	77.2395153	65.8457846
18:39:38	0:39:19	62.6930524	75.3812876	77.731787	77.2395153	65.8457846
18:39:39	0:39:20	60.6930524	77.3812876	77.731787	76.2395153	62.8457846
18:39:40	0:39:21	60.6930524	78.3812876	77.731787	75.2395153	63.8457846
18:39:41	0:39:22	60.6930524	78.3812876	77.731787	75.2395153	63.8457846
18:39:42	0:39:23	60.6930524	79.3812876	77.731787	74.2395153	62.8457846
18:39:43	0:39:24	60.6930524	78.3812876	77.731787	74.2395153	63.8457846
18:39:44	0:39:25	60.6930524	74.3812876	76.731787	74.2395153	63.8457846
18:39:45	0:39:26	60.6930524	73.3812876	74.731787	74.2395153	63.8457846
18:39:46	0:39:27	60.6930524	73.3812876	74.731787	74.2395153	63.8457846
18:39:47	0:39:28	60.6930524	73.3812876	73.731787	74.2395153	63.8457846
18:39:48	0:39:29	60.6930524	71.3812876	72.731787	74.2395153	63.8457846
18:39:49	0:39:30	60.6930524	71.3812876	71.731787	71.2395153	63.8457846
18:39:50	0:39:31	59.6930524	70.3812876	70.731787	70.2395153	65.8457846
18:39:51	0:39:32	59.6930524	70.3812876	68.731787	70.2395153	66.8457846
18:39:52	0:39:33	60.6930524	70.3812876	65.731787	67.2395153	67.8457846
18:39:53	0:39:34	60.6930524	71.3812876	65.731787	63.2395153	67.8457846
18:39:54	0:39:35	60.6930524	72.3812876	65.731787	62.2395153	68.8457846
18:39:55	0:39:36	60.6930524	72.3812876	65.731787	62.2395153	68.8457846
18:39:56	0:39:37	61.6930524	72.3812876	65.731787	62.2395153	68.8457846
18:39:57	0:39:38	61.6930524	72.3812876	65.731787	62.2395153	70.8457846

18:39:58	0:39:39	61.6930524	72.3812876	65.731787	62.2395153	70.8457846
18:39:59	0:39:40	63.6930524	73.3812876	65.731787	62.2395153	70.8457846
18:40:00	0:39:41	63.6930524	73.3812876	65.731787	62.2395153	70.8457846
18:40:01	0:39:42	62.6930524	73.3812876	65.731787	62.2395153	71.8457846
18:40:02	0:39:43	63.6930524	75.3812876	65.731787	62.2395153	72.8457846
18:40:03	0:39:44	63.6930524	76.3812876	65.731787	63.2395153	72.8457846
18:40:04	0:39:45	63.6930524	77.3812876	65.731787	63.2395153	72.8457846
18:40:05	0:39:46	63.6930524	77.3812876	65.731787	63.2395153	72.8457846
18:40:06	0:39:47	63.6930524	79.3812876	65.731787	62.2395153	73.8457846
18:40:07	0:39:48	63.6930524	82.3812876	65.731787	62.2395153	74.8457846
18:40:08	0:39:49	63.6930524	82.3812876	65.731787	62.2395153	75.8457846
18:40:09	0:39:50	63.6930524	82.3812876	65.731787	62.2395153	76.8457846
18:40:10	0:39:51	63.6930524	82.3812876	65.731787	63.2395153	76.8457846
18:40:11	0:39:52	64.6930524	83.3812876	65.731787	63.2395153	76.8457846
18:40:12	0:39:53	64.6930524	84.3812876	65.731787	64.2395153	76.8457846
18:40:13	0:39:54	64.6930524	82.3812876	65.731787	64.2395153	76.8457846
18:40:14	0:39:55	64.6930524	82.3812876	65.731787	65.2395153	77.8457846
18:40:15	0:39:56	65.6930524	82.3812876	65.731787	64.2395153	78.8457846
18:40:16	0:39:57	65.6930524	82.3812876	65.731787	63.2395153	78.8457846
18:40:17	0:39:58	65.6930524	82.3812876	65.731787	63.2395153	78.8457846
18:40:18	0:39:59	65.6930524	82.3812876	65.731787	63.2395153	79.8457846
18:40:19	0:40:00	65.6930524	82.3812876	65.731787	63.2395153	79.8457846
18:40:20	0:40:01	66.6930524	82.3812876	65.731787	62.2395153	79.8457846
18:40:21	0:40:02	65.6930524	84.3812876	67.731787	62.2395153	79.8457846
18:40:22	0:40:03	65.6930524	84.3812876	70.731787	62.2395153	79.8457846
18:40:23	0:40:04	65.6930524	84.3812876	70.731787	62.2395153	79.8457846
18:40:24	0:40:05	65.6930524	84.3812876	70.731787	62.2395153	79.8457846
18:40:25	0:40:06	65.6930524	85.3812876	69.731787	62.2395153	78.8457846
18:40:26	0:40:07	65.6930524	85.3812876	69.731787	62.2395153	78.8457846
18:40:27	0:40:08	65.6930524	85.3812876	69.731787	62.2395153	78.8457846
18:40:28	0:40:09	65.6930524	84.3812876	69.731787	62.2395153	78.8457846
18:40:29	0:40:10	65.6930524	83.3812876	70.731787	62.2395153	78.8457846
18:40:30	0:40:11	65.6930524	83.3812876	70.731787	62.2395153	78.8457846
18:40:31	0:40:12	64.6930524	82.3812876	70.731787	62.2395153	80.8457846
18:40:32	0:40:13	64.6930524	76.3812876	70.731787	62.2395153	81.8457846
18:40:33	0:40:14	64.6930524	76.3812876	71.731787	61.2395153	81.8457846
18:40:34	0:40:15	64.6930524	76.3812876	71.731787	61.2395153	82.8457846
18:40:35	0:40:16	64.6930524	75.3812876	71.731787	61.2395153	81.8457846
18:40:36	0:40:17	64.6930524	75.3812876	71.731787	61.2395153	81.8457846
18:40:37	0:40:18	65.6930524	75.3812876	71.731787	61.2395153	80.8457846
18:40:38	0:40:19	65.6930524	75.3812876	70.731787	61.2395153	79.8457846
18:40:39	0:40:20	65.6930524	75.3812876	69.731787	61.2395153	79.8457846
18:40:40	0:40:21	65.6930524	75.3812876	68.731787	61.2395153	79.8457846
18:40:41	0:40:22	66.6930524	75.3812876	68.731787	61.2395153	79.8457846
18:40:42	0:40:23	66.6930524	76.3812876	68.731787	61.2395153	79.8457846

18:40:43	0:40:24	67.6930524	75.3812876	69.731787	60.2395153	79.8457846
18:40:44	0:40:25	68.6930524	75.3812876	69.731787	62.2395153	79.8457846
18:40:45	0:40:26	69.6930524	74.3812876	69.731787	62.2395153	79.8457846
18:40:46	0:40:27	70.6930524	73.3812876	69.731787	62.2395153	79.8457846
18:40:47	0:40:28	70.6930524	73.3812876	68.731787	62.2395153	79.8457846
18:40:48	0:40:29	68.6930524	73.3812876	68.731787	63.2395153	79.8457846
18:40:49	0:40:30	65.6930524	72.3812876	68.731787	63.2395153	78.8457846
18:40:50	0:40:31	65.6930524	71.3812876	68.731787	60.2395153	77.8457846
18:40:51	0:40:32	63.6930524	71.3812876	69.731787	60.2395153	77.8457846
18:40:52	0:40:33	63.6930524	71.3812876	69.731787	60.2395153	77.8457846
18:40:53	0:40:34	62.6930524	71.3812876	69.731787	60.2395153	77.8457846
18:40:54	0:40:35	62.6930524	71.3812876	68.731787	60.2395153	77.8457846
18:40:55	0:40:36	62.6930524	71.3812876	68.731787	60.2395153	75.8457846
18:40:56	0:40:37	63.6930524	71.3812876	68.731787	62.2395153	75.8457846
18:40:57	0:40:38	63.6930524	71.3812876	69.731787	64.2395153	74.8457846
18:40:58	0:40:39	64.6930524	70.3812876	70.731787	64.2395153	74.8457846
18:40:59	0:40:40	66.6930524	71.3812876	71.731787	64.2395153	74.8457846
18:41:00	0:40:41	68.6930524	71.3812876	72.731787	66.2395153	73.8457846
18:41:01	0:40:42	69.6930524	71.3812876	72.731787	67.2395153	73.8457846
18:41:02	0:40:43	72.6930524	70.3812876	73.731787	68.2395153	73.8457846
18:41:03	0:40:44	76.6930524	69.3812876	73.731787	68.2395153	72.8457846
18:41:04	0:40:45	77.6930524	68.3812876	74.731787	68.2395153	72.8457846
18:41:05	0:40:46	77.6930524	66.3812876	75.731787	69.2395153	71.8457846
18:41:06	0:40:47	73.6930524	66.3812876	76.731787	69.2395153	71.8457846
18:41:07	0:40:48	69.6930524	64.3812876	76.731787	72.2395153	69.8457846
18:41:08	0:40:49	68.6930524	64.3812876	74.731787	72.2395153	69.8457846
18:41:09	0:40:50	68.6930524	63.3812876	73.731787	74.2395153	73.8457846
18:41:10	0:40:51	68.6930524	63.3812876	73.731787	75.2395153	74.8457846
18:41:11	0:40:52	67.6930524	63.3812876	73.731787	75.2395153	73.8457846
18:41:12	0:40:53	67.6930524	63.3812876	73.731787	75.2395153	73.8457846
18:41:13	0:40:54	67.6930524	64.3812876	73.731787	76.2395153	72.8457846
18:41:14	0:40:55	66.6930524	67.3812876	71.731787	76.2395153	71.8457846
18:41:15	0:40:56	66.6930524	67.3812876	70.731787	79.2395153	71.8457846
18:41:16	0:40:57	64.6930524	70.3812876	70.731787	79.2395153	71.8457846
18:41:17	0:40:58	64.6930524	70.3812876	69.731787	79.2395153	69.8457846
18:41:18	0:40:59	64.6930524	70.3812876	67.731787	80.2395153	68.8457846
18:41:19	0:41:00	65.6930524	70.3812876	67.731787	79.2395153	69.8457846
18:41:20	0:41:01	65.6930524	71.3812876	66.731787	79.2395153	69.8457846
18:41:21	0:41:02	65.6930524	71.3812876	64.731787	79.2395153	70.8457846
18:41:22	0:41:03	65.6930524	71.3812876	62.731787	79.2395153	70.8457846
18:41:23	0:41:04	66.6930524	71.3812876	62.731787	79.2395153	71.8457846
18:41:24	0:41:05	66.6930524	71.3812876	62.731787	79.2395153	71.8457846
18:41:25	0:41:06	69.6930524	71.3812876	62.731787	79.2395153	71.8457846
18:41:26	0:41:07	72.6930524	71.3812876	63.731787	79.2395153	70.8457846
18:41:27	0:41:08	73.6930524	71.3812876	63.731787	79.2395153	68.8457846

18:41:28	0:41:09	74.6930524	73.3812876	65.731787	78.2395153	68.8457846
18:41:29	0:41:10	75.6930524	73.3812876	66.731787	78.2395153	68.8457846
18:41:30	0:41:11	76.6930524	74.3812876	67.731787	78.2395153	67.8457846
18:41:31	0:41:12	77.6930524	73.3812876	68.731787	77.2395153	66.8457846
18:41:32	0:41:13	77.6930524	72.3812876	68.731787	65.2395153	65.8457846
18:41:33	0:41:14	77.6930524	71.3812876	68.731787	62.2395153	65.8457846
18:41:34	0:41:15	80.6930524	71.3812876	68.731787	62.2395153	65.8457846
18:41:35	0:41:16	79.6930524	70.3812876	67.731787	62.2395153	65.8457846
18:41:36	0:41:17	74.6930524	69.3812876	66.731787	62.2395153	66.8457846
18:41:37	0:41:18	74.6930524	69.3812876	66.731787	62.2395153	67.8457846
18:41:38	0:41:19	75.6930524	71.3812876	66.731787	62.2395153	67.8457846
18:41:39	0:41:20	75.6930524	71.3812876	66.731787	62.2395153	69.8457846
18:41:40	0:41:21	76.6930524	71.3812876	66.731787	62.2395153	70.8457846
18:41:41	0:41:22	77.6930524	71.3812876	67.731787	63.2395153	70.8457846
18:41:42	0:41:23	77.6930524	71.3812876	67.731787	63.2395153	70.8457846
18:41:43	0:41:24	76.6930524	71.3812876	67.731787	63.2395153	70.8457846
18:41:44	0:41:25	76.6930524	71.3812876	69.731787	63.2395153	70.8457846
18:41:45	0:41:26	73.6930524	71.3812876	68.731787	63.2395153	70.8457846
18:41:46	0:41:27	67.6930524	71.3812876	68.731787	63.2395153	70.8457846
18:41:47	0:41:28	65.6930524	72.3812876	68.731787	64.2395153	70.8457846
18:41:48	0:41:29	65.6930524	72.3812876	68.731787	65.2395153	70.8457846
18:41:49	0:41:30	63.6930524	72.3812876	68.731787	67.2395153	72.8457846
18:41:50	0:41:31	61.6930524	72.3812876	68.731787	73.2395153	74.8457846
18:41:51	0:41:32	61.6930524	72.3812876	68.731787	74.2395153	74.8457846
18:41:52	0:41:33	61.6930524	72.3812876	68.731787	75.2395153	75.8457846
18:41:53	0:41:34	61.6930524	72.3812876	68.731787	76.2395153	75.8457846
18:41:54	0:41:35	60.6930524	73.3812876	68.731787	77.2395153	75.8457846
18:41:55	0:41:36	60.6930524	73.3812876	68.731787	78.2395153	75.8457846
18:41:56	0:41:37	60.6930524	73.3812876	68.731787	80.2395153	75.8457846
18:41:57	0:41:38	60.6930524	73.3812876	68.731787	80.2395153	75.8457846
18:41:58	0:41:39	60.6930524	73.3812876	68.731787	81.2395153	75.8457846
18:41:59	0:41:40	59.6930524	74.3812876	68.731787	81.2395153	75.8457846
18:42:00	0:41:41	59.6930524	73.3812876	69.731787	81.2395153	75.8457846
18:42:01	0:41:42	59.6930524	73.3812876	70.731787	74.2395153	75.8457846
18:42:02	0:41:43	59.6930524	73.3812876	70.731787	73.2395153	74.8457846
18:42:03	0:41:44	59.6930524	74.3812876	72.731787	73.2395153	74.8457846
18:42:04	0:41:45	58.6930524	73.3812876	73.731787	73.2395153	72.8457846
18:42:05	0:41:46	57.6930524	73.3812876	73.731787	73.2395153	71.8457846
18:42:06	0:41:47	56.6930524	74.3812876	73.731787	73.2395153	71.8457846
18:42:07	0:41:48	56.6930524	74.3812876	73.731787	73.2395153	71.8457846
18:42:08	0:41:49	56.6930524	74.3812876	73.731787	73.2395153	71.8457846
18:42:09	0:41:50	56.6930524	74.3812876	72.731787	74.2395153	69.8457846
18:42:10	0:41:51	56.6930524	70.3812876	69.731787	74.2395153	69.8457846
18:42:11	0:41:52	56.6930524	67.3812876	68.731787	75.2395153	68.8457846
18:42:12	0:41:53	56.6930524	66.3812876	67.731787	75.2395153	66.8457846

18:42:13	0:41:54	56.6930524	66.3812876	67.731787	78.2395153	66.8457846
18:42:14	0:41:55	56.6930524	65.3812876	66.731787	77.2395153	66.8457846
18:42:15	0:41:56	56.6930524	66.3812876	65.731787	72.2395153	66.8457846
18:42:16	0:41:57	56.6930524	66.3812876	66.731787	66.2395153	66.8457846
18:42:17	0:41:58	56.6930524	66.3812876	66.731787	66.2395153	66.8457846
18:42:18	0:41:59	56.6930524	66.3812876	66.731787	66.2395153	66.8457846
18:42:19	0:42:00	56.6930524	65.3812876	67.731787	65.2395153	68.8457846
18:42:20	0:42:01	57.6930524	65.3812876	66.731787	65.2395153	69.8457846
18:42:21	0:42:02	57.6930524	65.3812876	65.731787	66.2395153	70.8457846
18:42:22	0:42:03	57.6930524	64.3812876	64.731787	66.2395153	70.8457846
18:42:23	0:42:04	57.6930524	64.3812876	64.731787	71.2395153	70.8457846
18:42:24	0:42:05	57.6930524	64.3812876	64.731787	73.2395153	70.8457846
18:42:25	0:42:06	57.6930524	64.3812876	65.731787	73.2395153	70.8457846
18:42:26	0:42:07	57.6930524	65.3812876	65.731787	73.2395153	66.8457846
18:42:27	0:42:08	57.6930524	64.3812876	66.731787	73.2395153	64.8457846
18:42:28	0:42:09	58.6930524	64.3812876	66.731787	73.2395153	63.8457846
18:42:29	0:42:10	58.6930524	64.3812876	66.731787	74.2395153	63.8457846
18:42:30	0:42:11	58.6930524	64.3812876	66.731787	74.2395153	63.8457846
18:42:31	0:42:12	57.6930524	64.3812876	65.731787	75.2395153	63.8457846
18:42:32	0:42:13	57.6930524	64.3812876	65.731787	77.2395153	63.8457846
18:42:33	0:42:14	57.6930524	64.3812876	65.731787	80.2395153	64.8457846
18:42:34	0:42:15	57.6930524	66.3812876	64.731787	81.2395153	64.8457846
18:42:35	0:42:16	57.6930524	67.3812876	66.731787	81.2395153	65.8457846
18:42:36	0:42:17	58.6930524	68.3812876	66.731787	78.2395153	69.8457846
18:42:37	0:42:18	58.6930524	68.3812876	66.731787	75.2395153	69.8457846
18:42:38	0:42:19	58.6930524	69.3812876	66.731787	75.2395153	69.8457846
18:42:39	0:42:20	58.6930524	69.3812876	66.731787	74.2395153	69.8457846
18:42:40	0:42:21	58.6930524	69.3812876	66.731787	74.2395153	69.8457846
18:42:41	0:42:22	59.6930524	69.3812876	67.731787	74.2395153	67.8457846
18:42:42	0:42:23	58.6930524	69.3812876	67.731787	75.2395153	67.8457846
18:42:43	0:42:24	58.6930524	69.3812876	67.731787	75.2395153	67.8457846
18:42:44	0:42:25	58.6930524	69.3812876	67.731787	75.2395153	68.8457846
18:42:45	0:42:26	58.6930524	68.3812876	67.731787	76.2395153	68.8457846
18:42:46	0:42:27	58.6930524	68.3812876	67.731787	77.2395153	68.8457846
18:42:47	0:42:28	57.6930524	67.3812876	67.731787	77.2395153	68.8457846
18:42:48	0:42:29	58.6930524	67.3812876	67.731787	78.2395153	69.8457846
18:42:49	0:42:30	58.6930524	67.3812876	67.731787	78.2395153	70.8457846
18:42:50	0:42:31	58.6930524	68.3812876	66.731787	77.2395153	69.8457846
18:42:51	0:42:32	57.6930524	69.3812876	70.731787	76.2395153	68.8457846
18:42:52	0:42:33	57.6930524	69.3812876	70.731787	76.2395153	68.8457846
18:42:53	0:42:34	56.6930524	70.3812876	70.731787	75.2395153	68.8457846
18:42:54	0:42:35	55.6930524	70.3812876	69.731787	75.2395153	68.8457846
18:42:55	0:42:36	55.6930524	70.3812876	69.731787	75.2395153	67.8457846
18:42:56	0:42:37	55.6930524	70.3812876	69.731787	77.2395153	67.8457846
18:42:57	0:42:38	55.6930524	70.3812876	69.731787	78.2395153	66.8457846

18:42:58	0:42:39	54.6930524	70.3812876	69.731787	78.2395153	66.8457846
18:42:59	0:42:40	54.6930524	70.3812876	69.731787	80.2395153	66.8457846
18:43:00	0:42:41	55.6930524	70.3812876	70.731787	83.2395153	66.8457846
18:43:01	0:42:42	56.6930524	70.3812876	70.731787	83.2395153	65.8457846
18:43:02	0:42:43	56.6930524	71.3812876	71.731787	78.2395153	65.8457846
18:43:03	0:42:44	56.6930524	71.3812876	70.731787	78.2395153	65.8457846
18:43:04	0:42:45	57.6930524	71.3812876	67.731787	70.2395153	66.8457846
18:43:05	0:42:46	57.6930524	71.3812876	64.731787	69.2395153	66.8457846
18:43:06	0:42:47	57.6930524	71.3812876	63.731787	68.2395153	68.8457846
18:43:07	0:42:48	57.6930524	71.3812876	63.731787	67.2395153	70.8457846
18:43:08	0:42:49	57.6930524	71.3812876	63.731787	67.2395153	70.8457846
18:43:09	0:42:50	57.6930524	71.3812876	63.731787	67.2395153	71.8457846
18:43:10	0:42:51	56.6930524	71.3812876	64.731787	67.2395153	73.8457846
18:43:11	0:42:52	56.6930524	71.3812876	64.731787	67.2395153	73.8457846
18:43:12	0:42:53	56.6930524	71.3812876	64.731787	67.2395153	73.8457846
18:43:13	0:42:54	58.6930524	71.3812876	67.731787	65.2395153	73.8457846
18:43:14	0:42:55	60.6930524	74.3812876	67.731787	64.2395153	74.8457846
18:43:15	0:42:56	62.6930524	74.3812876	66.731787	64.2395153	74.8457846
18:43:16	0:42:57	63.6930524	74.3812876	66.731787	66.2395153	74.8457846
18:43:17	0:42:58	64.6930524	72.3812876	66.731787	66.2395153	74.8457846
18:43:18	0:42:59	66.6930524	70.3812876	66.731787	66.2395153	74.8457846
18:43:19	0:43:00	66.6930524	70.3812876	66.731787	67.2395153	75.8457846
18:43:20	0:43:01	65.6930524	72.3812876	66.731787	68.2395153	75.8457846
18:43:21	0:43:02	65.6930524	74.3812876	66.731787	69.2395153	75.8457846
18:43:22	0:43:03	64.6930524	74.3812876	66.731787	69.2395153	75.8457846
18:43:23	0:43:04	64.6930524	74.3812876	66.731787	70.2395153	75.8457846
18:43:24	0:43:05	64.6930524	74.3812876	66.731787	70.2395153	75.8457846
18:43:25	0:43:06	62.6930524	75.3812876	68.731787	70.2395153	75.8457846
18:43:26	0:43:07	61.6930524	75.3812876	68.731787	70.2395153	75.8457846
18:43:27	0:43:08	60.6930524	75.3812876	69.731787	70.2395153	75.8457846
18:43:28	0:43:09	59.6930524	75.3812876	69.731787	70.2395153	75.8457846
18:43:29	0:43:10	58.6930524	76.3812876	69.731787	71.2395153	74.8457846
18:43:30	0:43:11	58.6930524	76.3812876	70.731787	71.2395153	74.8457846
18:43:31	0:43:12	58.6930524	76.3812876	75.731787	71.2395153	72.8457846
18:43:32	0:43:13	59.6930524	76.3812876	75.731787	72.2395153	72.8457846
18:43:33	0:43:14	59.6930524	76.3812876	74.731787	72.2395153	72.8457846
18:43:34	0:43:15	60.6930524	76.3812876	74.731787	72.2395153	73.8457846
18:43:35	0:43:16	60.6930524	76.3812876	74.731787	72.2395153	74.8457846
18:43:36	0:43:17	61.6930524	77.3812876	73.731787	75.2395153	75.8457846
18:43:37	0:43:18	60.6930524	78.3812876	73.731787	75.2395153	74.8457846
18:43:38	0:43:19	60.6930524	80.3812876	73.731787	76.2395153	73.8457846
18:43:39	0:43:20	60.6930524	80.3812876	73.731787	76.2395153	73.8457846
18:43:40	0:43:21	59.6930524	80.3812876	73.731787	76.2395153	72.8457846
18:43:41	0:43:22	59.6930524	80.3812876	73.731787	75.2395153	72.8457846
18:43:42	0:43:23	60.6930524	80.3812876	72.731787	74.2395153	72.8457846

18:43:43	0:43:24	61.6930524	80.3812876	72.731787	74.2395153	76.8457846
18:43:44	0:43:25	61.6930524	81.3812876	72.731787	73.2395153	76.8457846
18:43:45	0:43:26	62.6930524	81.3812876	71.731787	72.2395153	77.8457846
18:43:46	0:43:27	63.6930524	82.3812876	71.731787	63.2395153	77.8457846
18:43:47	0:43:28	63.6930524	82.3812876	71.731787	61.2395153	78.8457846
18:43:48	0:43:29	66.6930524	83.3812876	71.731787	60.2395153	78.8457846
18:43:49	0:43:30	68.6930524	83.3812876	70.731787	60.2395153	78.8457846
18:43:50	0:43:31	66.6930524	85.3812876	70.731787	60.2395153	78.8457846
18:43:51	0:43:32	64.6930524	85.3812876	69.731787	61.2395153	78.8457846
18:43:52	0:43:33	63.6930524	68.9201505	69.731787	61.2395153	78.8457846
18:43:53	0:43:34	63.6930524	80.3812876	69.731787	61.2395153	78.8457846
18:43:54	0:43:35	63.6930524	74.3812876	69.731787	62.2395153	78.8457846
18:43:55	0:43:36	63.6930524	74.3812876	69.731787	62.2395153	78.8457846
18:43:56	0:43:37	63.6930524	73.3812876	69.731787	66.2395153	78.8457846
18:43:57	0:43:38	63.6930524	72.3812876	69.731787	67.2395153	78.8457846
18:43:58	0:43:39	63.6930524	72.3812876	70.731787	69.2395153	79.8457846
18:43:59	0:43:40	63.6930524	71.3812876	70.731787	71.2395153	78.8457846
18:44:00	0:43:41	63.6930524	71.3812876	68.731787	71.2395153	76.8457846
18:44:01	0:43:42	64.6930524	71.3812876	68.731787	72.2395153	74.8457846
18:44:02	0:43:43	64.6930524	71.3812876	68.731787	72.2395153	74.8457846
18:44:03	0:43:44	65.6930524	68.3812876	68.731787	73.2395153	74.8457846
18:44:04	0:43:45	66.6930524	68.3812876	68.731787	73.2395153	74.8457846
18:44:05	0:43:46	66.6930524	69.3812876	68.731787	73.2395153	73.8457846
18:44:06	0:43:47	66.6930524	69.3812876	68.731787	73.2395153	73.8457846
18:44:07	0:43:48	69.6930524	69.3812876	69.731787	75.2395153	71.8457846
18:44:08	0:43:49	69.6930524	72.3812876	69.731787	75.2395153	70.8457846
18:44:09	0:43:50	65.6930524	75.3812876	70.731787	76.2395153	70.8457846
18:44:10	0:43:51	65.6930524	75.3812876	70.731787	77.2395153	70.8457846
18:44:11	0:43:52	66.6930524	76.3812876	70.731787	80.2395153	70.8457846
18:44:12	0:43:53	66.6930524	76.3812876	69.731787	83.2395153	70.8457846
18:44:13	0:43:54	67.6930524	76.3812876	70.731787	84.2395153	70.8457846
18:44:14	0:43:55	68.6930524	76.3812876	74.731787	85.2395153	70.8457846
18:44:15	0:43:56	69.6930524	75.3812876	74.731787	86.2395153	65.8457846
18:44:16	0:43:57	69.6930524	75.3812876	74.731787	71.0451505	64.8457846
18:44:17	0:43:58	68.6930524	73.3812876	74.731787	71.1701505	64.8457846
18:44:18	0:43:59	68.6930524	73.3812876	75.731787	71.0451505	62.8457846
18:44:19	0:44:00	67.6930524	73.3812876	72.731787	70.6701505	62.8457846
18:44:20	0:44:01	66.6930524	73.3812876	71.731787	70.2951505	62.8457846
18:44:21	0:44:02	66.6930524	73.3812876	71.731787	70.4201505	62.8457846
18:44:22	0:44:03	65.6930524	73.3812876	72.731787	70.6701505	63.8457846
18:44:23	0:44:04	65.6930524	74.3812876	71.731787	71.1701505	64.8457846
18:44:24	0:44:05	65.6930524	73.3812876	71.731787	71.0451505	65.8457846
18:44:25	0:44:06	65.6930524	73.3812876	72.731787	71.0451505	65.8457846
18:44:26	0:44:07	65.6930524	75.3812876	72.731787	71.2951505	65.8457846
18:44:27	0:44:08	65.6930524	75.3812876	72.731787	71.1701505	65.8457846

18:44:28	0:44:09	65.6930524	75.3812876	71.731787	71.0451505	65.8457846
18:44:29	0:44:10	65.6930524	77.3812876	70.731787	71.2951505	66.8457846
18:44:30	0:44:11	65.6930524	77.3812876	69.731787	71.7951505	70.8457846
18:44:31	0:44:12	65.6930524	77.3812876	69.731787	72.0451505	70.8457846
18:44:32	0:44:13	64.6930524	75.3812876	68.731787	72.1701505	71.8457846
18:44:33	0:44:14	64.6930524	75.3812876	68.731787	72.5451505	73.8457846
18:44:34	0:44:15	63.6930524	75.3812876	68.731787	72.9201505	74.8457846
18:44:35	0:44:16	62.6930524	73.3812876	69.731787	73.2951505	77.8457846
18:44:36	0:44:17	62.6930524	72.3812876	70.731787	73.5451505	76.8457846
18:44:37	0:44:18	62.6930524	73.3812876	70.731787	73.6701505	75.8457846
18:44:38	0:44:19	62.6930524	73.3812876	71.731787	73.7951505	74.8457846
18:44:39	0:44:20	62.6930524	73.3812876	70.731787	73.7951505	74.8457846
18:44:40	0:44:21	62.6930524	73.3812876	69.731787	73.5451505	73.8457846
18:44:41	0:44:22	63.6930524	63.3812876	69.731787	72.9201505	74.8457846
18:44:42	0:44:23	63.6930524	63.3812876	68.731787	73.0451505	74.8457846
18:44:43	0:44:24	64.6930524	62.3812876	68.731787	72.9201505	74.8457846
18:44:44	0:44:25	64.6930524	62.3812876	67.731787	72.6701505	74.8457846
18:44:45	0:44:26	64.6930524	62.3812876	67.731787	71.1701505	67.8457846
18:44:46	0:44:27	67.6930524	62.3812876	67.731787	71.1701505	67.8457846
18:44:47	0:44:28	66.6930524	62.3812876	68.731787	71.7951505	67.8457846
18:44:48	0:44:29	66.6930524	62.3812876	69.731787	71.4201505	67.8457846
18:44:49	0:44:30	66.6930524	62.3812876	69.731787	71.5451505	67.8457846
18:44:50	0:44:31	66.6930524	62.3812876	71.731787	71.7951505	66.8457846
18:44:51	0:44:32	66.6930524	62.3812876	71.731787	71.2951505	66.8457846
18:44:52	0:44:33	66.6930524	62.3812876	72.731787	71.1701505	66.8457846
18:44:53	0:44:34	66.6930524	63.3812876	72.731787	70.9201505	66.8457846
18:44:54	0:44:35	66.6930524	63.3812876	72.731787	70.9201505	67.8457846
18:44:55	0:44:36	66.6930524	63.3812876	72.731787	70.5451505	67.8457846
18:44:56	0:44:37	67.6930524	67.3812876	72.731787	71.1701505	68.8457846
18:44:57	0:44:38	67.6930524	67.3812876	73.731787	71.0451505	68.8457846
18:44:58	0:44:39	67.6930524	70.3812876	73.731787	71.4201505	68.8457846
18:44:59	0:44:40	67.6930524	71.3812876	73.731787	71.5451505	69.8457846
18:45:00	0:44:41	67.6930524	71.3812876	73.731787	71.5451505	69.8457846
18:45:01	0:44:42	68.6930524	72.3812876	71.731787	71.4201505	70.8457846
18:45:02	0:44:43	67.6930524	72.3812876	70.731787	71.4201505	70.8457846
18:45:03	0:44:44	67.6930524	73.3812876	69.731787	71.7951505	71.8457846
18:45:04	0:44:45	67.6930524	73.3812876	69.731787	72.0451505	72.8457846
18:45:05	0:44:46	67.6930524	73.3812876	69.731787	72.1701505	73.8457846
18:45:06	0:44:47	67.6930524	72.3812876	68.731787	72.1701505	73.8457846
18:45:07	0:44:48	67.6930524	72.3812876	68.731787	72.2951505	73.8457846
18:45:08	0:44:49	66.6930524	72.3812876	67.731787	71.6701505	73.8457846
18:45:09	0:44:50	66.6930524	73.3812876	67.731787	71.6701505	73.8457846
18:45:10	0:44:51	67.6930524	74.3812876	67.731787	71.7951505	72.8457846
18:45:11	0:44:52	67.6930524	74.3812876	67.731787	71.6701505	72.8457846
18:45:12	0:44:53	67.6930524	74.3812876	68.731787	71.6701505	70.8457846

18:45:13	0:44:54	66.6930524	74.3812876	68.731787	71.5451505	70.8457846
18:45:14	0:44:55	66.6930524	72.3812876	69.731787	71.5451505	69.8457846
18:45:15	0:44:56	66.6930524	71.3812876	69.731787	71.7951505	68.8457846
18:45:16	0:44:57	66.6930524	71.3812876	70.731787	71.9201505	68.8457846
18:45:17	0:44:58	66.6930524	71.3812876	72.731787	72.0451505	67.8457846
18:45:18	0:44:59	66.6930524	71.3812876	72.731787	71.7951505	67.8457846
18:45:19	0:45:00	66.6930524	71.3812876	72.731787	71.7951505	67.8457846
18:45:20	0:45:01	66.6930524	71.3812876	73.731787	71.6701505	67.8457846
18:45:21	0:45:02	66.6930524	66.3812876	73.731787	71.0451505	67.8457846
18:45:22	0:45:03	66.6930524	66.3812876	73.731787	70.4201505	67.8457846
18:45:23	0:45:04	67.6930524	65.3812876	73.731787	70.2951505	67.8457846
18:45:24	0:45:05	67.6930524	65.3812876	73.731787	87.2395153	68.8457846
18:45:25	0:45:06	67.6930524	65.3812876	72.731787	84.2395153	67.8457846
18:45:26	0:45:07	66.6930524	65.3812876	72.731787	80.2395153	68.8457846
18:45:27	0:45:08	66.6930524	65.3812876	72.731787	80.2395153	68.8457846
18:45:28	0:45:09	66.6930524	65.3812876	72.731787	78.2395153	68.8457846
18:45:29	0:45:10	66.6930524	65.3812876	70.731787	78.2395153	68.8457846
18:45:30	0:45:11	66.6930524	66.3812876	69.731787	77.2395153	69.8457846
18:45:31	0:45:12	66.6930524	67.3812876	69.731787	77.2395153	69.8457846
18:45:32	0:45:13	66.6930524	67.3812876	69.731787	77.2395153	69.8457846
18:45:33	0:45:14	66.6930524	67.3812876	69.731787	71.2395153	69.8457846
18:45:34	0:45:15	67.6930524	68.3812876	70.731787	70.2395153	70.8457846
18:45:35	0:45:16	67.6930524	69.3812876	71.731787	69.2395153	70.8457846
18:45:36	0:45:17	67.6930524	70.3812876	72.731787	68.2395153	70.8457846
18:45:37	0:45:18	67.6930524	70.3812876	72.731787	67.2395153	70.8457846
18:45:38	0:45:19	66.6930524	70.3812876	72.731787	66.2395153	70.8457846
18:45:39	0:45:20	67.6930524	70.3812876	72.731787	66.2395153	71.8457846
18:45:40	0:45:21	67.6930524	69.3812876	71.731787	64.2395153	72.8457846
18:45:41	0:45:22	67.6930524	69.3812876	70.731787	65.2395153	72.8457846
18:45:42	0:45:23	68.6930524	69.3812876	71.731787	66.2395153	72.8457846
18:45:43	0:45:24	68.6930524	69.3812876	71.731787	68.2395153	72.8457846
18:45:44	0:45:25	71.6930524	70.3812876	71.731787	68.2395153	72.8457846
18:45:45	0:45:26	70.6930524	70.3812876	71.731787	68.2395153	71.8457846
18:45:46	0:45:27	69.6930524	70.3812876	71.731787	68.2395153	71.8457846
18:45:47	0:45:28	68.6930524	70.3812876	71.731787	67.2395153	71.8457846
18:45:48	0:45:29	69.6930524	70.3812876	72.731787	67.2395153	71.8457846
18:45:49	0:45:30	69.6930524	70.3812876	73.731787	66.2395153	71.8457846
18:45:50	0:45:31	69.6930524	71.3812876	73.731787	66.2395153	71.8457846
18:45:51	0:45:32	69.6930524	71.3812876	73.731787	66.2395153	68.8457846
18:45:52	0:45:33	68.6930524	72.3812876	73.731787	66.2395153	68.8457846
18:45:53	0:45:34	68.6930524	73.3812876	73.731787	65.2395153	68.8457846
18:45:54	0:45:35	68.6930524	73.3812876	73.731787	65.2395153	68.8457846
18:45:55	0:45:36	68.6930524	74.3812876	74.731787	65.2395153	69.8457846
18:45:56	0:45:37	67.6930524	74.3812876	74.731787	65.2395153	69.8457846
18:45:57	0:45:38	67.6930524	74.3812876	74.731787	63.2395153	69.8457846

18:45:58	0:45:39	66.6930524	74.3812876	72.731787	63.2395153	69.8457846
18:45:59	0:45:40	66.6930524	74.3812876	71.731787	60.2395153	69.8457846
18:46:00	0:45:41	67.6930524	74.3812876	71.731787	60.2395153	70.8457846
18:46:01	0:45:42	67.6930524	70.3812876	70.731787	59.2395153	71.8457846
18:46:02	0:45:43	67.6930524	80.3812876	68.731787	59.2395153	71.8457846
18:46:03	0:45:44	67.6930524	80.3812876	67.731787	59.2395153	71.8457846
18:46:04	0:45:45	68.6930524	80.3812876	66.731787	59.2395153	71.8457846
18:46:05	0:45:46	68.6930524	80.3812876	66.731787	59.2395153	71.8457846
18:46:06	0:45:47	68.6930524	79.3812876	66.731787	58.2395153	65.8457846
18:46:07	0:45:48	68.6930524	79.3812876	66.731787	58.2395153	62.8457846
18:46:08	0:45:49	67.6930524	80.3812876	66.731787	57.2395153	59.8457846
18:46:09	0:45:50	67.6930524	80.3812876	68.731787	57.2395153	58.8457846
18:46:10	0:45:51	68.6930524	80.3812876	68.731787	63.2395153	57.8457846
18:46:11	0:45:52	68.6930524	80.3812876	68.731787	63.2395153	56.8457846
18:46:12	0:45:53	68.6930524	80.3812876	68.731787	64.2395153	56.8457846
18:46:13	0:45:54	68.6930524	80.3812876	68.731787	64.2395153	56.8457846
18:46:14	0:45:55	68.6930524	80.3812876	68.731787	64.2395153	56.8457846
18:46:15	0:45:56	68.6930524	80.3812876	68.731787	62.2395153	56.8457846
18:46:16	0:45:57	68.6930524	80.3812876	69.731787	62.2395153	56.8457846
18:46:17	0:45:58	69.6930524	80.3812876	69.731787	62.2395153	56.8457846
18:46:18	0:45:59	69.6930524	80.3812876	69.731787	61.2395153	57.8457846
18:46:19	0:46:00	69.6930524	80.3812876	69.731787	61.2395153	59.8457846
18:46:20	0:46:01	68.6930524	80.3812876	69.731787	61.2395153	59.8457846
18:46:21	0:46:02	68.6930524	80.3812876	69.731787	61.2395153	59.8457846
18:46:22	0:46:03	68.6930524	80.3812876	69.731787	61.2395153	59.8457846
18:46:23	0:46:04	68.6930524	80.3812876	68.731787	62.2395153	59.8457846
18:46:24	0:46:05	68.6930524	79.3812876	68.731787	62.2395153	59.8457846
18:46:25	0:46:06	68.6930524	77.3812876	68.731787	61.2395153	59.8457846
18:46:26	0:46:07	68.6930524	77.3812876	67.731787	61.2395153	59.8457846
18:46:27	0:46:08	69.6930524	74.3812876	67.731787	60.2395153	60.8457846
18:46:28	0:46:09	68.6930524	73.3812876	67.731787	60.2395153	60.8457846
18:46:29	0:46:10	68.6930524	73.3812876	67.731787	59.2395153	62.8457846
18:46:30	0:46:11	68.6930524	73.3812876	67.731787	60.2395153	62.8457846
18:46:31	0:46:12	68.6930524	73.3812876	67.731787	60.2395153	63.8457846
18:46:32	0:46:13	69.6930524	73.3812876	67.731787	60.2395153	62.8457846
18:46:33	0:46:14	69.6930524	73.3812876	67.731787	60.2395153	62.8457846
18:46:34	0:46:15	70.6930524	73.3812876	67.731787	59.2395153	62.8457846
18:46:35	0:46:16	71.6930524	73.3812876	67.731787	59.2395153	62.8457846
18:46:36	0:46:17	72.6930524	71.3812876	70.731787	59.2395153	64.8457846
18:46:37	0:46:18	71.6930524	71.3812876	72.731787	59.2395153	66.8457846
18:46:38	0:46:19	71.6930524	71.3812876	72.731787	59.2395153	67.8457846
18:46:39	0:46:20	70.6930524	72.3812876	72.731787	59.2395153	67.8457846
18:46:40	0:46:21	70.6930524	76.3812876	72.731787	59.2395153	67.8457846
18:46:41	0:46:22	71.6930524	79.3812876	72.731787	59.2395153	68.8457846
18:46:42	0:46:23	71.6930524	78.3812876	72.731787	59.2395153	68.8457846

18:46:43	0:46:24	72.6930524	76.3812876	71.731787	59.2395153	68.8457846
18:46:44	0:46:25	73.6930524	76.3812876	70.731787	60.2395153	66.8457846
18:46:45	0:46:26	73.6930524	76.3812876	69.731787	63.2395153	66.8457846
18:46:46	0:46:27	72.6930524	77.3812876	68.731787	64.2395153	65.8457846
18:46:47	0:46:28	72.6930524	77.3812876	67.731787	65.2395153	65.8457846
18:46:48	0:46:29	72.6930524	77.3812876	67.731787	65.2395153	65.8457846
18:46:49	0:46:30	70.6930524	76.3812876	67.731787	67.2395153	64.8457846
18:46:50	0:46:31	69.6930524	76.3812876	68.731787	68.2395153	63.8457846
18:46:51	0:46:32	69.6930524	76.3812876	68.731787	70.2395153	63.8457846
18:46:52	0:46:33	69.6930524	76.3812876	68.731787	70.2395153	66.8457846
18:46:53	0:46:34	68.6930524	76.3812876	68.731787	74.2395153	69.8457846
18:46:54	0:46:35	68.6930524	75.3812876	66.731787	75.2395153	70.8457846
18:46:55	0:46:36	68.6930524	75.3812876	66.731787	75.2395153	71.8457846
18:46:56	0:46:37	67.6930524	75.3812876	66.731787	75.2395153	71.8457846
18:46:57	0:46:38	66.6930524	75.3812876	66.731787	74.2395153	71.8457846
18:46:58	0:46:39	66.6930524	75.3812876	67.731787	73.2395153	71.8457846
18:46:59	0:46:40	66.6930524	72.3812876	67.731787	73.2395153	71.8457846
18:47:00	0:46:41	67.6930524	69.3812876	68.731787	74.2395153	72.8457846
18:47:01	0:46:42	67.6930524	69.3812876	70.731787	74.2395153	72.8457846
18:47:02	0:46:43	67.6930524	68.3812876	71.731787	74.2395153	72.8457846
18:47:03	0:46:44	68.6930524	66.3812876	71.731787	74.2395153	72.8457846
18:47:04	0:46:45	68.6930524	65.3812876	71.731787	74.2395153	72.8457846
18:47:05	0:46:46	69.6930524	65.3812876	71.731787	74.2395153	72.8457846
18:47:06	0:46:47	69.6930524	65.3812876	71.731787	73.2395153	73.8457846
18:47:07	0:46:48	69.6930524	64.3812876	71.731787	73.2395153	73.8457846
18:47:08	0:46:49	69.6930524	64.3812876	71.731787	72.2395153	73.8457846
18:47:09	0:46:50	69.6930524	68.3812876	70.731787	72.2395153	73.8457846
18:47:10	0:46:51	69.6930524	70.3812876	69.731787	72.2395153	73.8457846
18:47:11	0:46:52	69.6930524	71.3812876	69.731787	72.2395153	73.8457846
18:47:12	0:46:53	68.6930524	71.3812876	69.731787	71.2395153	73.8457846
18:47:13	0:46:54	67.6930524	72.3812876	69.731787	71.2395153	73.8457846
18:47:14	0:46:55	67.6930524	75.3812876	68.731787	70.2395153	73.8457846
18:47:15	0:46:56	67.6930524	75.3812876	68.731787	69.2395153	71.8457846
18:47:16	0:46:57	67.6930524	76.3812876	67.731787	66.2395153	71.8457846
18:47:17	0:46:58	69.6930524	75.3812876	67.731787	64.2395153	71.8457846
18:47:18	0:46:59	69.6930524	75.3812876	67.731787	63.2395153	71.8457846
18:47:19	0:47:00	69.6930524	75.3812876	67.731787	63.2395153	71.8457846
18:47:20	0:47:01	69.6930524	75.3812876	67.731787	63.2395153	71.8457846
18:47:21	0:47:02	69.6930524	75.3812876	67.731787	63.2395153	71.8457846
18:47:22	0:47:03	69.6930524	75.3812876	67.731787	64.2395153	71.8457846
18:47:23	0:47:04	69.6930524	75.3812876	67.731787	65.2395153	71.8457846
18:47:24	0:47:05	69.6930524	75.3812876	68.731787	64.2395153	72.8457846
18:47:25	0:47:06	69.6930524	75.3812876	68.731787	64.2395153	72.8457846
18:47:26	0:47:07	68.6930524	75.3812876	68.731787	64.2395153	73.8457846
18:47:27	0:47:08	68.6930524	79.3812876	68.731787	64.2395153	73.8457846

18:47:28	0:47:09	68.6930524	79.3812876	69.731787	63.2395153	75.8457846
18:47:29	0:47:10	68.6930524	79.3812876	69.731787	63.2395153	75.8457846
18:47:30	0:47:11	66.6930524	78.3812876	69.731787	65.2395153	76.8457846
18:47:31	0:47:12	66.6930524	77.3812876	68.731787	66.2395153	76.8457846
18:47:32	0:47:13	66.6930524	76.3812876	66.731787	66.2395153	76.8457846
18:47:33	0:47:14	66.6930524	75.3812876	66.731787	66.2395153	78.8457846
18:47:34	0:47:15	66.6930524	74.3812876	66.731787	67.2395153	79.8457846
18:47:35	0:47:16	66.6930524	74.3812876	66.731787	67.2395153	79.8457846
18:47:36	0:47:17	66.6930524	74.3812876	66.731787	68.2395153	79.8457846
18:47:37	0:47:18	66.6930524	73.3812876	67.731787	69.2395153	78.8457846
18:47:38	0:47:19	66.6930524	73.3812876	67.731787	69.2395153	76.8457846
18:47:39	0:47:20	66.6930524	73.3812876	68.731787	69.2395153	75.8457846
18:47:40	0:47:21	66.6930524	73.3812876	69.731787	71.2395153	75.8457846
18:47:41	0:47:22	66.6930524	73.3812876	69.731787	71.2395153	75.8457846
18:47:42	0:47:23	66.6930524	73.3812876	69.731787	71.2395153	75.8457846
18:47:43	0:47:24	66.6930524	73.3812876	70.731787	74.2395153	76.8457846
18:47:44	0:47:25	66.6930524	72.3812876	70.731787	74.2395153	76.8457846
18:47:45	0:47:26	66.6930524	72.3812876	70.731787	74.2395153	77.8457846
18:47:46	0:47:27	65.6930524	72.3812876	70.731787	74.2395153	77.8457846
18:47:47	0:47:28	65.6930524	68.3812876	70.731787	75.2395153	76.8457846
18:47:48	0:47:29	64.6930524	68.3812876	70.731787	75.2395153	76.8457846
18:47:49	0:47:30	63.6930524	67.3812876	70.731787	76.2395153	75.8457846
18:47:50	0:47:31	63.6930524	67.3812876	68.731787	76.2395153	75.8457846
18:47:51	0:47:32	63.6930524	67.3812876	70.731787	76.2395153	75.8457846
18:47:52	0:47:33	64.6930524	67.3812876	69.731787	76.2395153	75.8457846
18:47:53	0:47:34	64.6930524	66.3812876	69.731787	76.2395153	76.8457846
18:47:54	0:47:35	64.6930524	67.3812876	69.731787	77.2395153	76.8457846
18:47:55	0:47:36	65.6930524	67.3812876	70.731787	77.2395153	75.8457846
18:47:56	0:47:37	65.6930524	67.3812876	70.731787	77.2395153	75.8457846
18:47:57	0:47:38	65.6930524	68.3812876	70.731787	77.2395153	75.8457846
18:47:58	0:47:39	64.6930524	69.3812876	70.731787	77.2395153	75.8457846
18:47:59	0:47:40	64.6930524	69.3812876	70.731787	77.2395153	75.8457846
18:48:00	0:47:41	63.6930524	70.3812876	71.731787	77.2395153	75.8457846
18:48:01	0:47:42	62.6930524	70.3812876	71.731787	79.2395153	75.8457846
18:48:02	0:47:43	61.6930524	71.3812876	72.731787	79.2395153	75.8457846
18:48:03	0:47:44	61.6930524	70.3812876	72.731787	79.2395153	76.8457846
18:48:04	0:47:45	61.6930524	68.3812876	72.731787	78.2395153	76.8457846
18:48:05	0:47:46	61.6930524	68.3812876	72.731787	77.2395153	76.8457846
18:48:06	0:47:47	60.6930524	65.3812876	72.731787	77.2395153	76.8457846
18:48:07	0:47:48	60.6930524	65.3812876	72.731787	77.2395153	75.8457846
18:48:08	0:47:49	60.6930524	65.3812876	72.731787	74.2395153	74.8457846
18:48:09	0:47:50	60.6930524	69.3812876	72.731787	74.2395153	74.8457846
18:48:10	0:47:51	60.6930524	72.3812876	72.731787	74.2395153	74.8457846
18:48:11	0:47:52	61.6930524	73.3812876	71.731787	73.2395153	74.8457846
18:48:12	0:47:53	61.6930524	73.3812876	71.731787	74.2395153	73.8457846

18:48:13	0:47:54	61.6930524	69.3812876	71.731787	74.2395153	72.8457846
18:48:14	0:47:55	62.6930524	69.3812876	70.731787	74.2395153	72.8457846
18:48:15	0:47:56	62.6930524	66.3812876	69.731787	75.2395153	71.8457846
18:48:16	0:47:57	62.6930524	65.3812876	69.731787	76.2395153	70.8457846
18:48:17	0:47:58	62.6930524	64.3812876	68.731787	76.2395153	70.8457846
18:48:18	0:47:59	62.6930524	64.3812876	67.731787	76.2395153	70.8457846
18:48:19	0:48:00	62.6930524	64.3812876	67.731787	76.2395153	70.8457846
18:48:20	0:48:01	62.6930524	64.3812876	67.731787	76.2395153	69.8457846
18:48:21	0:48:02	62.6930524	64.3812876	68.731787	78.2395153	69.8457846
18:48:22	0:48:03	62.6930524	63.3812876	68.731787	78.2395153	72.8457846
18:48:23	0:48:04	62.6930524	63.3812876	69.731787	81.2395153	75.8457846
18:48:24	0:48:05	63.6930524	63.3812876	69.731787	81.2395153	76.8457846
18:48:25	0:48:06	63.6930524	63.3812876	70.731787	83.2395153	75.8457846
18:48:26	0:48:07	74.6930524	65.3812876	69.731787	83.2395153	75.8457846
18:48:27	0:48:08	75.6930524	65.3812876	68.731787	83.2395153	74.8457846
18:48:28	0:48:09	76.6930524	65.3812876	68.731787	83.2395153	73.8457846
18:48:29	0:48:10	77.6930524	65.3812876	68.731787	83.2395153	72.8457846
18:48:30	0:48:11	77.6930524	65.3812876	68.731787	83.2395153	71.8457846
18:48:31	0:48:12	77.6930524	65.3812876	68.731787	77.2395153	71.8457846
18:48:32	0:48:13	76.6930524	62.3812876	68.731787	76.2395153	72.8457846
18:48:33	0:48:14	75.6930524	61.3812876	68.731787	74.2395153	72.8457846
18:48:34	0:48:15	74.6930524	62.3812876	70.731787	72.2395153	72.8457846
18:48:35	0:48:16	73.6930524	61.3812876	71.731787	72.2395153	72.8457846
18:48:36	0:48:17	73.6930524	61.3812876	71.731787	72.2395153	72.8457846
18:48:37	0:48:18	73.6930524	61.3812876	71.731787	73.2395153	72.8457846
18:48:38	0:48:19	72.6930524	62.3812876	69.731787	66.2395153	72.8457846
18:48:39	0:48:20	72.6930524	63.3812876	68.731787	64.2395153	72.8457846
18:48:40	0:48:21	73.6930524	65.3812876	67.731787	62.2395153	72.8457846
18:48:41	0:48:22	75.6930524	70.3812876	66.731787	62.2395153	73.8457846
18:48:42	0:48:23	75.6930524	71.3812876	66.731787	62.2395153	73.8457846
18:48:43	0:48:24	75.6930524	71.3812876	66.731787	62.2395153	73.8457846
18:48:44	0:48:25	74.6930524	70.3812876	66.731787	61.2395153	73.8457846
18:48:45	0:48:26	73.6930524	69.3812876	65.731787	61.2395153	72.8457846
18:48:46	0:48:27	72.6930524	69.3812876	65.731787	61.2395153	72.8457846
18:48:47	0:48:28	70.6930524	68.3812876	65.731787	61.2395153	72.8457846
18:48:48	0:48:29	69.6930524	68.3812876	64.731787	61.2395153	72.8457846
18:48:49	0:48:30	69.6930524	68.3812876	64.731787	61.2395153	74.8457846
18:48:50	0:48:31	68.6930524	68.3812876	64.731787	61.2395153	74.8457846
18:48:51	0:48:32	68.6930524	68.3812876	64.731787	61.2395153	75.8457846
18:48:52	0:48:33	67.6930524	68.3812876	65.731787	60.2395153	75.8457846
18:48:53	0:48:34	67.6930524	68.3812876	64.731787	60.2395153	75.8457846
18:48:54	0:48:35	67.6930524	68.3812876	64.731787	60.2395153	76.8457846
18:48:55	0:48:36	67.6930524	67.3812876	64.731787	63.2395153	76.8457846
18:48:56	0:48:37	67.6930524	68.3812876	64.731787	67.2395153	76.8457846
18:48:57	0:48:38	67.6930524	68.3812876	64.731787	69.2395153	85.8457846

18:48:58	0:48:39	67.6930524	70.3812876	69.731787	71.2395153	85.8457846
18:48:59	0:48:40	67.6930524	69.3812876	69.731787	72.2395153	85.8457846
18:49:00	0:48:41	66.6930524	69.3812876	69.731787	72.2395153	83.8457846
18:49:01	0:48:42	65.6930524	66.3812876	68.731787	72.2395153	71.8457846
18:49:02	0:48:43	65.6930524	66.3812876	68.731787	73.2395153	66.8457846
18:49:03	0:48:44	64.6930524	66.3812876	68.731787	73.2395153	65.8457846
18:49:04	0:48:45	64.6930524	66.3812876	69.731787	73.2395153	64.8457846
18:49:05	0:48:46	64.6930524	66.3812876	70.731787	73.2395153	64.8457846
18:49:06	0:48:47	64.6930524	66.3812876	71.731787	73.2395153	62.8457846
18:49:07	0:48:48	65.6930524	67.3812876	71.731787	73.2395153	62.8457846
18:49:08	0:48:49	65.6930524	67.3812876	71.731787	73.2395153	62.8457846
18:49:09	0:48:50	66.6930524	67.3812876	70.731787	74.2395153	63.8457846
18:49:10	0:48:51	67.6930524	68.3812876	68.731787	74.2395153	65.8457846
18:49:11	0:48:52	67.6930524	68.3812876	68.731787	74.2395153	65.8457846
18:49:12	0:48:53	66.6930524	68.3812876	67.731787	75.2395153	65.8457846
18:49:13	0:48:54	67.6930524	67.3812876	68.731787	75.2395153	62.8457846
18:49:14	0:48:55	67.6930524	64.3812876	68.731787	78.2395153	62.8457846
18:49:15	0:48:56	66.6930524	64.3812876	68.731787	79.2395153	61.8457846
18:49:16	0:48:57	66.6930524	63.3812876	69.731787	79.2395153	61.8457846
18:49:17	0:48:58	66.6930524	64.3812876	70.731787	78.2395153	61.8457846
18:49:18	0:48:59	66.6930524	65.3812876	70.731787	67.2395153	61.8457846
18:49:19	0:49:00	66.6930524	65.3812876	71.731787	67.2395153	60.8457846
18:49:20	0:49:01	66.6930524	65.3812876	72.731787	62.2395153	60.8457846
18:49:21	0:49:02	66.6930524	67.3812876	72.731787	62.2395153	60.8457846
18:49:22	0:49:03	66.6930524	68.3812876	73.731787	62.2395153	60.8457846
18:49:23	0:49:04	66.6930524	69.3812876	74.731787	62.2395153	60.8457846
18:49:24	0:49:05	66.6930524	69.3812876	75.731787	61.2395153	61.8457846
18:49:25	0:49:06	66.6930524	71.3812876	77.731787	60.2395153	61.8457846
18:49:26	0:49:07	66.6930524	71.3812876	79.731787	60.2395153	61.8457846
18:49:27	0:49:08	66.6930524	71.3812876	82.731787	60.2395153	65.8457846
18:49:28	0:49:09	66.6930524	71.3812876	82.731787	58.2395153	70.8457846
18:49:29	0:49:10	66.6930524	71.3812876	67.2951505	55.2395153	71.8457846
18:49:30	0:49:11	66.6930524	71.3812876	67.2951505	55.2395153	71.8457846
18:49:31	0:49:12	69.6930524	70.3812876	82.731787	50.2395153	68.8457846
18:49:32	0:49:13	71.6930524	70.3812876	82.731787	50.2395153	67.8457846
18:49:33	0:49:14	71.6930524	70.3812876	82.731787	51.2395153	67.8457846
18:49:34	0:49:15	72.6930524	69.3812876	77.731787	51.2395153	67.8457846
18:49:35	0:49:16	71.6930524	69.3812876	75.731787	53.2395153	67.8457846
18:49:36	0:49:17	71.6930524	69.3812876	74.731787	55.2395153	69.8457846
18:49:37	0:49:18	71.6930524	68.3812876	74.731787	56.2395153	70.8457846
18:49:38	0:49:19	71.6930524	66.3812876	74.731787	58.2395153	69.8457846
18:49:39	0:49:20	71.6930524	66.3812876	73.731787	61.2395153	67.8457846
18:49:40	0:49:21	71.6930524	66.3812876	72.731787	61.2395153	65.8457846
18:49:41	0:49:22	71.6930524	66.3812876	71.731787	62.2395153	65.8457846
18:49:42	0:49:23	71.6930524	66.3812876	70.731787	63.2395153	66.8457846

18:49:43	0:49:24	70.6930524	66.3812876	69.731787	63.2395153	66.8457846
18:49:44	0:49:25	70.6930524	66.3812876	68.731787	64.2395153	67.8457846
18:49:45	0:49:26	70.6930524	65.3812876	68.731787	66.2395153	67.8457846
18:49:46	0:49:27	70.6930524	65.3812876	68.731787	67.2395153	67.8457846
18:49:47	0:49:28	70.6930524	65.3812876	69.731787	68.2395153	67.8457846
18:49:48	0:49:29	69.6930524	65.3812876	70.731787	69.2395153	67.8457846
18:49:49	0:49:30	69.6930524	64.3812876	69.731787	70.2395153	67.8457846
18:49:50	0:49:31	69.6930524	64.3812876	69.731787	69.2395153	67.8457846
18:49:51	0:49:32	69.6930524	64.3812876	70.731787	67.2395153	68.8457846
18:49:52	0:49:33	69.6930524	64.3812876	69.731787	61.2395153	70.8457846
18:49:53	0:49:34	69.6930524	64.3812876	69.731787	60.2395153	69.8457846
18:49:54	0:49:35	68.6930524	65.3812876	69.731787	59.2395153	68.8457846
18:49:55	0:49:36	68.6930524	66.3812876	69.731787	59.2395153	67.8457846
18:49:56	0:49:37	70.6930524	66.3812876	70.731787	59.2395153	67.8457846
18:49:57	0:49:38	71.6930524	69.3812876	70.731787	59.2395153	66.8457846
18:49:58	0:49:39	71.6930524	69.3812876	70.731787	59.2395153	65.8457846
18:49:59	0:49:40	70.6930524	69.3812876	71.731787	59.2395153	66.8457846
18:50:00	0:49:41	70.6930524	69.3812876	72.731787	58.2395153	68.8457846
18:50:01	0:49:42	71.6930524	69.3812876	72.731787	58.2395153	69.8457846
18:50:02	0:49:43	70.6930524	69.3812876	78.731787	56.2395153	69.8457846
18:50:03	0:49:44	69.6930524	69.3812876	78.731787	56.2395153	69.8457846
18:50:04	0:49:45	68.6930524	69.3812876	78.731787	56.2395153	69.8457846
18:50:05	0:49:46	67.6930524	69.3812876	79.731787	56.2395153	70.8457846
18:50:06	0:49:47	67.6930524	69.3812876	80.731787	57.2395153	70.8457846
18:50:07	0:49:48	67.6930524	69.3812876	80.731787	58.2395153	70.8457846
18:50:08	0:49:49	67.6930524	67.3812876	82.731787	60.2395153	70.8457846
18:50:09	0:49:50	66.6930524	67.3812876	82.731787	61.2395153	67.8457846
18:50:10	0:49:51	66.6930524	66.3812876	82.731787	61.2395153	65.8457846
18:50:11	0:49:52	70.6930524	66.3812876	82.731787	61.2395153	64.8457846
18:50:12	0:49:53	70.6930524	66.3812876	82.731787	61.2395153	64.8457846
18:50:13	0:49:54	71.6930524	65.3812876	81.731787	60.2395153	65.8457846
18:50:14	0:49:55	71.6930524	62.3812876	81.731787	59.2395153	67.8457846
18:50:15	0:49:56	71.6930524	62.3812876	80.731787	58.2395153	68.8457846
18:50:16	0:49:57	71.6930524	62.3812876	80.731787	57.2395153	70.8457846
18:50:17	0:49:58	70.6930524	62.3812876	80.731787	57.2395153	70.8457846
18:50:18	0:49:59	69.6930524	61.3812876	80.731787	57.2395153	69.8457846
18:50:19	0:50:00	70.6930524	60.3812876	75.731787	57.2395153	69.8457846
18:50:20	0:50:01	70.6930524	60.3812876	75.731787	58.2395153	69.8457846
18:50:21	0:50:02	70.6930524	60.3812876	73.731787	59.2395153	69.8457846
18:50:22	0:50:03	70.6930524	60.3812876	73.731787	59.2395153	69.8457846
18:50:23	0:50:04	70.6930524	62.3812876	73.731787	60.2395153	69.8457846
18:50:24	0:50:05	70.6930524	62.3812876	73.731787	60.2395153	69.8457846
18:50:25	0:50:06	71.6930524	63.3812876	74.731787	64.2395153	69.8457846
18:50:26	0:50:07	71.6930524	63.3812876	74.731787	66.2395153	68.8457846
18:50:27	0:50:08	70.6930524	63.3812876	74.731787	66.2395153	68.8457846

18:50:28	0:50:09	69.6930524	61.3812876	74.731787	67.2395153	68.8457846
18:50:29	0:50:10	68.6930524	61.3812876	72.731787	67.2395153	67.8457846
18:50:30	0:50:11	67.6930524	61.3812876	72.731787	67.2395153	67.8457846
18:50:31	0:50:12	67.6930524	61.3812876	71.731787	68.2395153	67.8457846
18:50:32	0:50:13	66.6930524	61.3812876	72.731787	69.2395153	67.8457846
18:50:33	0:50:14	66.6930524	61.3812876	75.731787	70.2395153	68.8457846
18:50:34	0:50:15	66.6930524	61.3812876	76.731787	69.2395153	69.8457846
18:50:35	0:50:16	66.6930524	62.3812876	76.731787	69.2395153	69.8457846
18:50:36	0:50:17	66.6930524	62.3812876	75.731787	69.2395153	69.8457846
18:50:37	0:50:18	66.6930524	62.3812876	70.731787	70.2395153	68.8457846
18:50:38	0:50:19	66.6930524	62.3812876	68.731787	73.2395153	70.8457846
18:50:39	0:50:20	66.6930524	62.3812876	68.731787	75.2395153	71.8457846
18:50:40	0:50:21	66.6930524	59.3812876	67.731787	77.2395153	73.8457846
18:50:41	0:50:22	66.6930524	59.3812876	66.731787	80.2395153	76.8457846
18:50:42	0:50:23	66.6930524	58.3812876	66.731787	80.2395153	78.8457846
18:50:43	0:50:24	65.6930524	58.3812876	67.731787	81.2395153	79.8457846
18:50:44	0:50:25	65.6930524	58.3812876	68.731787	84.2395153	78.8457846
18:50:45	0:50:26	66.6930524	58.3812876	68.731787	84.2395153	76.8457846
18:50:46	0:50:27	68.6930524	57.3812876	68.731787	84.2395153	76.8457846
18:50:47	0:03:00	68.6930524	56.3812876	68.731787	85.2395153	75.8457846
18:50:48	0:02:59	68.6930524	56.3812876	69.731787	85.2395153	73.8457846
18:50:49	0:02:58	69.6930524	64.3812876	69.731787	84.2395153	73.8457846
18:50:50	0:02:57	70.6930524	64.3812876	67.731787	83.2395153	70.8457846
18:50:51	0:02:56	70.6930524	64.3812876	66.731787	83.2395153	70.8457846
18:50:52	0:02:55	72.6930524	65.3812876	67.731787	83.2395153	70.8457846
18:50:53	0:02:54	73.6930524	65.3812876	67.731787	82.2395153	66.8457846
18:50:54	0:02:53	75.6930524	64.3812876	67.731787	83.2395153	64.8457846
18:50:55	0:02:52	73.6930524	64.3812876	68.731787	83.2395153	64.8457846
18:50:56	0:02:51	72.6930524	64.3812876	68.731787	84.2395153	64.8457846
18:50:57	0:02:50	72.6930524	64.3812876	68.731787	83.2395153	64.8457846
18:50:58	0:02:49	72.6930524	64.3812876	66.731787	83.2395153	66.8457846
18:50:59	0:02:48	71.6930524	64.3812876	66.731787	85.2395153	66.8457846
18:51:00	0:02:47	71.6930524	64.3812876	66.731787	85.2395153	66.8457846
18:51:01	0:02:46	70.6930524	70.3812876	66.731787	85.2395153	66.8457846
18:51:02	0:02:45	70.6930524	70.3812876	66.731787	85.2395153	66.8457846
18:51:03	0:02:44	70.6930524	71.3812876	65.731787	86.2395153	66.8457846
18:51:04	0:02:43	71.6930524	71.3812876	65.731787	86.2395153	65.8457846
18:51:05	0:02:42	71.6930524	72.3812876	65.731787	86.2395153	64.8457846
18:51:06	0:02:41	70.6930524	72.3812876	65.731787	86.2395153	63.8457846
18:51:07	0:02:40	70.6930524	72.3812876	66.731787	86.2395153	64.8457846
18:51:08	0:02:39	70.6930524	72.3812876	66.731787	87.2395153	64.8457846
18:51:09	0:02:38	69.6930524	73.3812876	66.731787	87.2395153	65.8457846
18:51:10	0:02:37	69.6930524	73.3812876	66.731787	87.2395153	67.8457846
18:51:11	0:02:36	69.6930524	73.3812876	66.731787	83.2395153	68.8457846
18:51:12	0:02:35	68.6930524	74.3812876	67.731787	79.2395153	68.8457846

18:51:13	0:02:34	68.6930524	74.3812876	67.731787	78.2395153	66.8457846
18:51:14	0:02:33	68.6930524	74.3812876	66.731787	77.2395153	65.8457846
18:51:15	0:02:32	68.6930524	74.3812876	66.731787	76.2395153	66.8457846
18:51:16	0:02:31	68.6930524	73.3812876	66.731787	76.2395153	67.8457846
18:51:17	0:02:30	68.6930524	73.3812876	66.731787	76.2395153	69.8457846
18:51:18	0:02:29	68.6930524	73.3812876	66.731787	76.2395153	69.8457846
18:51:19	0:02:28	68.6930524	71.3812876	67.731787	75.2395153	70.8457846
18:51:20	0:02:27	68.6930524	71.3812876	67.731787	73.2395153	70.8457846
18:51:21	0:02:26	69.6930524	70.3812876	68.731787	70.2395153	69.8457846
18:51:22	0:02:25	69.6930524	68.3812876	69.731787	68.2395153	68.8457846
18:51:23	0:02:24	69.6930524	68.3812876	69.731787	64.2395153	68.8457846
18:51:24	0:02:23	71.6930524	68.3812876	69.731787	63.2395153	67.8457846
18:51:25	0:02:22	72.6930524	69.3812876	68.731787	62.2395153	67.8457846
18:51:26	0:02:21	72.6930524	69.3812876	68.731787	62.2395153	67.8457846
18:51:27	0:02:20	71.6930524	70.3812876	68.731787	61.2395153	67.8457846
18:51:28	0:02:19	71.6930524	70.3812876	68.731787	60.2395153	68.8457846
18:51:29	0:02:18	71.6930524	70.3812876	68.731787	60.2395153	68.8457846
18:51:30	0:02:17	71.6930524	70.3812876	69.731787	60.2395153	67.8457846
18:51:31	0:02:16	71.6930524	70.3812876	69.731787	60.2395153	66.8457846
18:51:32	0:02:15	71.6930524	70.3812876	67.731787	61.2395153	64.8457846
18:51:33	0:02:14	71.6930524	72.3812876	66.731787	61.2395153	63.8457846
18:51:34	0:02:13	70.6930524	72.3812876	65.731787	61.2395153	63.8457846
18:51:35	0:02:12	70.6930524	73.3812876	64.731787	62.2395153	64.8457846
18:51:36	0:02:11	71.6930524	73.3812876	64.731787	63.2395153	65.8457846
18:51:37	0:02:10	71.6930524	73.3812876	64.731787	64.2395153	67.8457846
18:51:38	0:02:09	70.6930524	70.3812876	63.731787	64.2395153	68.8457846
18:51:39	0:02:08	69.6930524	70.3812876	64.731787	64.2395153	69.8457846
18:51:40	0:02:07	69.6930524	70.3812876	64.731787	65.2395153	70.8457846
18:51:41	0:02:06	69.6930524	69.3812876	64.731787	66.2395153	70.8457846
18:51:42	0:02:05	69.6930524	70.3812876	64.731787	66.2395153	70.8457846
18:51:43	0:02:04	70.6930524	70.3812876	64.731787	68.2395153	70.8457846
18:51:44	0:02:03	70.6930524	70.3812876	64.731787	68.2395153	69.8457846
18:51:45	0:02:02	71.6930524	70.3812876	64.731787	68.2395153	69.8457846
18:51:46	0:02:01	70.6930524	70.3812876	68.731787	68.2395153	69.8457846
18:51:47	0:02:00	70.6930524	71.3812876	69.731787	68.2395153	69.8457846
18:51:48	0:01:59	70.6930524	73.3812876	69.731787	68.2395153	69.8457846
18:51:49	0:01:58	70.6930524	73.3812876	70.731787	68.2395153	69.8457846
18:51:50	0:01:57	70.6930524	73.3812876	70.731787	67.2395153	68.8457846
18:51:51	0:01:56	70.6930524	73.3812876	70.731787	67.2395153	71.8457846
18:51:52	0:01:55	70.6930524	75.3812876	70.731787	65.2395153	75.8457846
18:51:53	0:01:54	70.6930524	78.3812876	70.731787	65.2395153	77.8457846
18:51:54	0:01:53	70.6930524	82.3812876	71.731787	64.2395153	78.8457846
18:51:55	0:01:52	70.6930524	72.7951505	72.731787	63.2395153	78.8457846
18:51:56	0:01:51	70.6930524	72.5451505	72.731787	64.2395153	76.8457846
18:51:57	0:01:50	71.6930524	72.6701505	73.731787	64.2395153	75.8457846

18:51:58	0:01:49	71.6930524	89.3812876	73.731787	66.2395153	75.8457846
18:51:59	0:01:48	71.6930524	89.3812876	74.731787	69.2395153	74.8457846
18:52:00	0:01:47	71.6930524	89.3812876	74.731787	71.2395153	72.8457846
18:52:01	0:01:46	71.6930524	86.3812876	74.731787	74.2395153	70.8457846
18:52:02	0:01:45	72.6930524	86.3812876	74.731787	74.2395153	70.8457846
18:52:03	0:01:44	72.6930524	86.3812876	75.731787	76.2395153	71.8457846
18:52:04	0:01:43	72.6930524	84.3812876	75.731787	78.2395153	72.8457846
18:52:05	0:01:42	72.6930524	84.3812876	76.731787	79.2395153	72.8457846
18:52:06	0:01:41	72.6930524	84.3812876	76.731787	79.2395153	72.8457846
18:52:07	0:01:40	72.6930524	84.3812876	76.731787	79.2395153	71.8457846
18:52:08	0:01:39	73.6930524	84.3812876	76.731787	79.2395153	69.8457846
18:52:09	0:01:38	73.6930524	84.3812876	78.731787	79.2395153	69.8457846
18:52:10	0:01:37	73.6930524	83.3812876	80.731787	79.2395153	69.8457846
18:52:11	0:01:36	73.6930524	82.3812876	80.731787	79.2395153	70.8457846
18:52:12	0:01:35	73.6930524	82.3812876	80.731787	79.2395153	70.8457846
18:52:13	0:01:34	72.6930524	81.3812876	81.731787	79.2395153	72.8457846
18:52:14	0:01:33	72.6930524	80.3812876	82.731787	80.2395153	72.8457846
18:52:15	0:01:32	68.6930524	80.3812876	83.731787	81.2395153	69.8457846
18:52:16	0:01:31	67.6930524	80.3812876	83.731787	82.2395153	66.8457846
18:52:17	0:01:30	66.6930524	78.3812876	83.731787	89.2395153	65.8457846
18:52:18	0:01:29	66.6930524	78.3812876	83.731787	89.2395153	67.8457846
18:52:19	0:01:28	66.6930524	78.3812876	83.731787	89.2395153	68.8457846
18:52:20	0:01:27	66.6930524	75.3812876	83.731787	89.2395153	69.8457846
18:52:21	0:01:26	66.6930524	75.3812876	82.731787	88.2395153	70.8457846
18:52:22	0:01:25	67.6930524	74.3812876	82.731787	88.2395153	69.8457846
18:52:23	0:01:24	67.6930524	71.3812876	82.731787	87.2395153	68.8457846
18:52:24	0:01:23	68.6930524	71.3812876	80.731787	86.2395153	65.8457846
18:52:25	0:01:22	68.6930524	66.3812876	80.731787	85.2395153	63.8457846
18:52:26	0:01:21	68.6930524	65.3812876	80.731787	82.2395153	63.8457846
18:52:27	0:01:20	68.6930524	65.3812876	81.731787	81.2395153	62.8457846
18:52:28	0:01:19	68.6930524	65.3812876	84.731787	80.2395153	63.8457846
18:52:29	0:01:18	68.6930524	66.3812876	85.731787	79.2395153	63.8457846
18:52:30	0:01:17	68.6930524	66.3812876	85.731787	79.2395153	65.8457846
18:52:31	0:01:16	68.6930524	66.3812876	71.0451505	79.2395153	65.8457846
18:52:32	0:01:15	67.6930524	65.3812876	71.0451505	79.2395153	65.8457846
18:52:33	0:01:14	67.6930524	65.3812876	71.1701505	79.2395153	65.8457846
18:52:34	0:01:13	66.6930524	65.3812876	71.5451505	79.2395153	66.8457846
18:52:35	0:01:12	66.6930524	65.3812876	70.7951505	75.2395153	66.8457846
18:52:36	0:01:11	65.6930524	66.3812876	70.7951505	75.2395153	66.8457846
18:52:37	0:01:10	65.6930524	66.3812876	70.7951505	74.2395153	67.8457846
18:52:38	0:01:09	65.6930524	66.3812876	71.1701505	74.2395153	70.8457846
18:52:39	0:01:08	65.6930524	67.3812876	71.5451505	73.2395153	71.8457846
18:52:40	0:01:07	65.6930524	67.3812876	71.4201505	73.2395153	68.8457846
18:52:41	0:01:06	65.6930524	69.3812876	71.4201505	72.2395153	64.8457846
18:52:42	0:01:05	65.6930524	73.3812876	71.7951505	69.2395153	62.8457846

18:52:43	0:01:04	65.6930524	74.3812876	72.0451505	66.2395153	63.8457846
18:52:44	0:01:03	66.6930524	68.3812876	71.7951505	66.2395153	64.8457846
18:52:45	0:01:02	65.6930524	68.3812876	71.1701505	63.2395153	63.8457846
18:52:46	0:01:01	65.6930524	66.3812876	70.6701505	63.2395153	62.8457846
18:52:47	0:01:00	64.6930524	63.3812876	69.9201505	63.2395153	61.8457846
18:52:48	0:00:59	64.6930524	62.3812876	69.2951505	59.2395153	61.8457846
18:52:49	0:00:58	65.6930524	62.3812876	69.5451505	59.2395153	61.8457846
18:52:50	0:00:57	65.6930524	61.3812876	68.6701505	58.2395153	61.8457846
18:52:51	0:00:56	65.6930524	61.3812876	68.5451505	57.2395153	61.8457846
18:52:52	0:00:55	64.6930524	61.3812876	67.4201505	56.2395153	61.8457846
18:52:53	0:00:54	64.6930524	59.3812876	66.9201505	56.2395153	61.8457846
18:52:54	0:00:53	64.6930524	59.3812876	66.6701505	55.2395153	61.8457846
18:52:55	0:00:52	64.6930524	59.3812876	66.2951505	55.2395153	62.8457846
18:52:56	0:00:51	65.6930524	59.3812876	66.4201505	55.2395153	63.8457846
18:52:57	0:00:50	66.6930524	59.3812876	67.0451505	55.2395153	67.8457846
18:52:58	0:00:49	67.6930524	59.3812876	67.5451505	54.2395153	70.8457846
18:52:59	0:00:48	67.6930524	58.3812876	80.731787	55.2395153	71.8457846
18:53:00	0:00:47	67.6930524	57.3812876	79.731787	55.2395153	66.8457846
18:53:01	0:00:46	63.6930524	57.3812876	78.731787	55.2395153	63.8457846
18:53:02	0:00:45	62.6930524	57.3812876	78.731787	55.2395153	61.8457846
18:53:03	0:00:44	62.6930524	57.3812876	79.731787	58.2395153	60.8457846
18:53:04	0:00:43	62.6930524	58.3812876	66.2951505	60.2395153	61.8457846
18:53:05	0:00:42	61.6930524	60.3812876	66.7951505	60.2395153	61.8457846
18:53:06	0:00:41	61.6930524	60.3812876	66.7951505	60.2395153	61.8457846
18:53:07	0:00:40	61.6930524	60.3812876	66.2951505	59.2395153	61.8457846
18:53:08	0:00:39	61.6930524	60.3812876	66.7951505	59.2395153	62.8457846
18:53:09	0:00:38	61.6930524	60.3812876	66.7951505	59.2395153	64.8457846
18:53:10	0:00:37	60.6930524	60.3812876	66.5451505	59.2395153	63.8457846
18:53:11	0:00:36	60.6930524	60.3812876	66.2951505	59.2395153	61.8457846
18:53:12	0:00:35	60.6930524	60.3812876	66.2951505	59.2395153	61.8457846
18:53:13	0:00:34	60.6930524	60.3812876	66.2951505	59.2395153	63.8457846
18:53:14	0:00:33	59.6930524	60.3812876	66.9201505	61.2395153	64.8457846
18:53:15	0:00:32	59.6930524	60.3812876	66.9201505	61.2395153	64.8457846
18:53:16	0:00:31	60.6930524	63.3812876	67.5451505	61.2395153	64.8457846
18:53:17	0:00:30	60.6930524	63.3812876	67.9201505	61.2395153	63.8457846
18:53:18	0:00:29	60.6930524	63.3812876	68.0451505	61.2395153	63.8457846
18:53:19	0:00:28	61.6930524	65.3812876	68.9201505	62.2395153	63.8457846
18:53:20	0:00:27	62.6930524	68.3812876	69.4201505	62.2395153	64.8457846
18:53:21	0:00:26	62.6930524	74.3812876	70.2951505	62.2395153	65.8457846
18:53:22	0:00:25	62.6930524	74.3812876	70.4201505	61.2395153	66.8457846
18:53:23	0:00:24	63.6930524	70.3812876	70.9201505	61.2395153	68.8457846
18:53:24	0:00:23	63.6930524	70.3812876	70.5451505	61.2395153	70.8457846
18:53:25	0:00:22	64.6930524	70.3812876	71.0451505	62.2395153	71.8457846
18:53:26	0:00:21	64.6930524	69.3812876	78.731787	65.2395153	70.8457846
18:53:27	0:00:20	63.6930524	69.3812876	72.731787	65.2395153	69.8457846

18:53:28	0:00:19	63.6930524	69.3812876	69.731787	65.2395153	68.8457846
18:53:29	0:00:18	65.6930524	69.3812876	68.731787	64.2395153	68.8457846
18:53:30	0:00:17	66.6930524	66.3812876	67.731787	63.2395153	68.8457846
18:53:31	0:00:16	66.6930524	66.3812876	67.731787	63.2395153	68.8457846
18:53:32	0:00:15	64.6930524	64.3812876	67.731787	63.2395153	69.8457846
18:53:33	0:00:14	61.6930524	62.3812876	68.731787	63.2395153	69.8457846
18:53:34	0:00:13	61.6930524	58.3812876	70.731787	63.2395153	69.8457846
18:53:35	0:00:12	61.6930524	57.3812876	70.731787	64.2395153	69.8457846
18:53:36	0:00:11	61.6930524	57.3812876	72.731787	64.2395153	68.8457846
18:53:37	0:00:10	61.6930524	56.3812876	73.731787	64.2395153	68.8457846
18:53:38	0:00:09	61.6930524	55.3812876	74.731787	65.2395153	68.8457846
18:53:39	0:00:08	61.6930524	54.3812876	73.731787	66.2395153	68.8457846
18:53:40	0:00:07	61.6930524	53.3812876	72.731787	68.2395153	69.8457846
18:53:41	0:00:06	61.6930524	53.3812876	72.731787	69.2395153	69.8457846
18:53:42	0:00:05	62.6930524	53.3812876	72.731787	69.2395153	69.8457846
18:53:43	0:00:04	62.6930524	53.3812876	72.731787	69.2395153	69.8457846
18:53:44	0:00:03	63.6930524	49.3812876	71.731787	70.2395153	69.8457846
18:53:45	0:00:02	63.6930524	54.3812876	71.731787	70.2395153	69.8457846
18:53:46	0:00:01	63.6930524	54.3812876	71.731787	70.2395153	69.8457846

End of Class

Person 9	Person 10	Person 12	Person 2	Activity Code	Average	annotation
77.3551261	81.2690501				75.9308004	
77.3551261	81.2690501				76.502229	
77.3551261	83.2690501				77.0736576	
77.3551261	83.2690501				76.6450862	
78.3551261	83.2690501				77.0736576	
78.3551261	81.2690501				77.2165147	
78.3551261	79.2690501				77.3593719	
79.3551261	79.2690501				77.7879433	
80.3551261	79.2690501				78.2165147	
80.3551261	79.2690501				78.502229	
80.3551261	79.2690501				78.7879433	
78.3551261	79.2690501				78.7879433	
79.3551261	79.2690501				79.2165147	
79.3551261	79.2690501				79.502229	
79.3551261	82.2690501				80.9308004	
78.3551261	84.2690501				81.7879433	
78.3551261	87.2690501				83.0736576	
77.3551261	89.2690501				82.7879433	
77.3551261	89.2690501				82.9308004	
77.3551261	88.2690501				82.9308004	
78.3551261	87.2690501				82.6450862	
80.3551261	87.2690501				82.3593719	
81.3551261	87.2690501				82.0736576	
81.3551261	87.2690501				81.6450862	
81.3551261	87.2690501				80.9308004	
82.3551261	87.2690501				80.7879433	
83.3551261	91.2690501				81.0736576	
82.3551261	92.2690501				81.2165147	
81.3551261	92.2690501				80.9308004	
81.3551261	92.2690501				80.9308004	
81.3551261	90.2690501	90.8456011			80.6450862	
80.3551261	90.2690501	90.8456011			81.7951505	
80.3551261	89.2690501	92.8456011			81.9201505	
80.3551261	89.2690501	94.8456011			81.9201505	
79.3551261	89.2690501	94.8456011			80.9201505	
79.3551261	89.2690501	94.8456011			80.5451505	
78.3551261	90.2690501	79.0451505			77.1950942	
78.3551261	92.2690501	78.5451505			76.6325942	
78.3551261	92.2690501	78.5451505			76.1325942	
78.3551261	92.2690501	79.1701505			76.2107192	
78.3551261	93.2690501	79.0451505			76.4450942	
77.3551261	93.2690501	79.4201505			76.3669692	

77.3551261	95.2690501	80.5451505			76.8825942
77.3551261	95.2690501	81.0451505			78.0700942
79.3551261	95.2690501	81.1701505			78.4607192
80.3551261	95.2690501	80.9201505			78.5544692
81.3551261	95.2690501	81.2951505			78.3513442
82.3551261	95.2690501	78.7879433			78.4129433
82.3551261	78.7879433	81.2329976	75.7357739		77.1193308
82.3551261	95.2690501	81.3441087	79.7357739		79.6325942
81.3551261	95.2690501	81.8456011	84.7357739		79.2951505
81.3551261	91.2690501	79.8456011	87.7357739		78.6701505
82.3551261	90.2690501	79.8456011	88.7357739		78.5451505
82.3551261	87.2690501	78.8456011	88.7357739		78.1701505
82.3551261	87.2690501	78.8456011	88.7357739		77.9201505
81.3551261	87.2690501	76.8456011	91.7357739		77.5451505
81.3551261	80.2690501	73.8456011	93.7357739		76.4201505
80.3551261	75.2690501	73.8456011	95.7357739		75.9201505
79.3551261	75.2690501	71.8456011	95.7357739		75.7951505
75.3551261	74.2690501	71.8456011	95.7357739		75.1701505
75.3551261	74.2690501	71.8456011	95.7357739		75.6701505
75.3551261	73.2690501	73.8456011	90.7357739		76.2951505
75.3551261	73.2690501	73.8456011	84.7357739		76.2951505
75.3551261	74.2690501	77.8456011	82.7357739		77.4201505
76.3551261	74.2690501	81.8456011	81.7357739		77.6701505
77.3551261	74.2690501	81.8456011	81.7357739		77.2951505
77.3551261	76.2690501	81.8456011	81.7357739		77.6701505
77.3551261	77.2690501	81.8456011	80.7357739		77.7951505
77.3551261	77.2690501	81.8456011	80.7357739		77.6701505
77.3551261	80.2690501	81.8456011	78.7357739		77.7951505
77.3551261	80.2690501	81.8456011	78.7357739		77.5451505
77.3551261	80.2690501	82.8456011	78.7357739		77.4201505
78.3551261	83.2690501	82.8456011	79.7357739		77.7951505
78.3551261	83.2690501	82.8456011	79.7357739		77.0451505
78.3551261	83.2690501	82.8456011	85.7357739		75.2951505
78.3551261	84.2690501	82.8456011	86.7357739		74.7951505
78.3551261	84.2690501	82.8456011	87.7357739		74.7951505
79.3551261	84.2690501	82.8456011	87.7357739		74.1701505
78.3551261	84.2690501	82.8456011	86.7357739		74.2951505
78.3551261	84.2690501	83.8456011	86.7357739		74.4201505
79.3551261	86.2690501	83.8456011	86.7357739		75.4201505
79.3551261	86.2690501	81.8456011	84.7357739		75.4201505
79.3551261	86.2690501	81.8456011	83.7357739		75.6701505
79.3551261	86.2690501	70.8456011	83.7357739		74.2951505
72.3551261	90.2690501	67.8456011	83.7357739		74.2951505
72.3551261	90.2690501	65.8456011	83.7357739		74.2951505
68.3551261	90.2690501	64.8456011	85.7357739		74.1701505

68.3551261	89.2690501	64.8456011	88.7357739	74.2951505
68.3551261	89.2690501	62.8456011	88.7357739	74.0451505
68.3551261	87.2690501	62.8456011	92.7357739	73.7951505
71.3551261	86.2690501	61.8456011	93.7357739	73.9201505
72.3551261	86.2690501	61.8456011	94.7357739	74.0451505
73.3551261	85.2690501	61.8456011	94.7357739	73.6701505
73.3551261	89.2690501	61.8456011	94.7357739	74.2951505
73.3551261	89.2690501	61.8456011	94.7357739	74.0451505
72.3551261	89.2690501	62.8456011	94.7357739	73.5451505
71.3551261	73.4201505	62.8456011	94.7357739	71.3140381
71.3551261	72.9201505	62.8456011	94.7357739	70.8765381
71.3551261	72.7951505	62.8456011	94.7357739	70.7359131
71.3551261	72.7951505	62.8456011	91.7357739	70.7359131
72.3551261	72.5451505	62.8456011	91.7357739	70.4546631
72.3551261	72.2951505	62.8456011	86.7357739	70.1734131
73.3551261	88.2690501	62.8456011	87.7357739	72.4201505
74.3551261	88.2690501	62.8456011	88.7357739	73.7951505
76.3551261	88.2690501	62.8456011	89.7357739	74.0451505
76.3551261	86.2690501	62.8456011	92.7357739	74.1701505
76.3551261	85.2690501	62.8456011	93.7357739	74.4201505
78.3551261	85.2690501	62.8456011	94.7357739	74.5451505
79.3551261	84.2690501	62.8456011	94.7357739	75.0451505
80.3551261	84.2690501	62.8456011	90.7357739	75.0451505
80.3551261	84.2690501	62.8456011	87.7357739	74.7951505
80.3551261	84.2690501	63.8456011	87.7357739	75.0451505
80.3551261	83.2690501	63.8456011	81.7357739	74.9201505
80.3551261	78.2690501	64.8456011	79.7357739	74.5451505
80.3551261	74.2690501	64.8456011	78.7357739	74.0451505
80.3551261	73.2690501	64.8456011	80.7357739	74.1701505
80.3551261	73.2690501	64.8456011	80.7357739	74.0451505
81.3551261	73.2690501	64.8456011	82.7357739	74.2951505
81.3551261	73.2690501	64.8456011	83.7357739	74.4201505
81.3551261	72.2690501	65.8456011	85.7357739	74.6701505
81.3551261	71.2690501	65.8456011	87.7357739	73.1701505
81.3551261	70.2690501	65.8456011	87.7357739	72.6701505
81.3551261	70.2690501	65.8456011	88.7357739	72.7951505
81.3551261	70.2690501	65.8456011	89.7357739	72.9201505
81.3551261	70.2690501	65.8456011	89.7357739	72.7951505
81.3551261	70.2690501	66.8456011	88.7357739	72.7951505
81.3551261	70.2690501	66.8456011	87.7357739	72.5451505
80.3551261	70.2690501	66.8456011	86.7357739	71.9201505
80.3551261	71.2690501	67.8456011	86.7357739	71.9201505
80.3551261	75.2690501	67.8456011	86.7357739	72.1701505
80.3551261	75.2690501	67.8456011	86.7357739	72.1701505
79.3551261	77.2690501	69.8456011	86.7357739	72.5451505

78.3551261	78.2690501	69.8456011	87.7357739	72.2951505
78.3551261	78.2690501	69.8456011	88.7357739	72.0451505
78.3551261	78.2690501	70.8456011	88.7357739	72.1701505
78.3551261	78.2690501	71.8456011	88.7357739	71.9201505
78.3551261	81.2690501	71.8456011	88.7357739	72.4201505
78.3551261	81.2690501	72.8456011	91.7357739	72.5451505
79.3551261	81.2690501	74.8456011	92.7357739	72.9201505
81.3551261	81.2690501	75.8456011	92.7357739	73.7951505
81.3551261	82.2690501	76.8456011	93.7357739	74.0451505
82.3551261	83.2690501	76.8456011	93.7357739	74.0451505
83.3551261	86.2690501	77.8456011	93.7357739	73.9201505
81.3551261	73.9201505	78.8456011	91.7357739	71.8765381
80.3551261	74.0451505	78.8456011	90.7357739	71.6421631
80.3551261	73.9201505	81.8456011	90.7357739	71.8765381
79.3551261	74.4201505	81.8456011	90.7357739	71.8140381
79.3551261	74.6701505	81.8456011	90.7357739	71.9702881
79.3551261	74.5451505	81.8456011	90.7357739	71.8296631
78.3551261	74.5451505	81.8456011	85.7357739	71.8296631
78.3551261	74.6701505	81.8456011	85.7357739	71.9702881
78.3551261	75.0451505	81.8456011	84.7357739	72.3921631
77.3551261	74.9201505	86.8456011	81.7357739	72.8765381
77.3551261	75.6701505	87.8456011	81.7357739	73.2202881
77.3551261	76.0451505	87.8456011	81.7357739	73.5171631
76.3551261	75.9201505	87.8456011	81.7357739	73.3765381
76.3551261	76.1701505	88.8456011	82.7357739	73.7827881
82.3551261	77.1701505	89.8456011	82.7357739	74.9077881
82.3551261	76.6701505	89.8456011	83.7357739	74.4702881
82.3551261	76.9201505	89.8456011	83.7357739	74.7515381
84.3551261	77.0451505	89.8456011	83.7357739	75.0171631
84.3551261	77.1701505	79.8456011	79.7357739	73.7827881
85.3551261	76.2951505	69.8456011	77.7357739	72.6734131
76.0451505	76.0451505	67.8456011	77.7357739	71.3534161
77.0451505	92.2690501	66.8456011	75.7357739	73.7564036
77.1701505	92.2690501	66.8456011	71.7357739	73.7720286
77.7951505	91.2690501	66.8456011	70.7357739	73.9751536
78.0451505	91.2690501	66.8456011	70.7357739	74.0064036
78.5451505	92.2690501	66.8456011	70.7357739	74.3189036
78.9201505	92.2690501	66.8456011	72.7357739	74.6157786
78.5451505	94.2690501	67.8456011	75.7357739	74.8189036
78.7951505	78.7951505	67.8456011	76.7357739	72.9159161
79.4201505	95.2690501	65.8456011	76.7357739	75.3032786
79.6701505	79.6701505	65.8456011	73.7357739	73.7596661
80.2951505	80.2951505	64.8456011	72.7357739	74.1659161
81.6701505	97.2690501	65.8456011	73.7357739	76.7095286
81.9201505	97.2690501	65.8456011	75.7357739	76.2407786

81.7951505	97.2690501	65.8456011	75.7357739	75.8501536
81.5451505	97.2690501	65.8456011	76.7357739	75.1939036
80.6701505	80.6701505	66.8456011	76.7357739	73.1346661
80.9201505	80.9201505	67.8456011	73.7357739	73.4471661
80.5451505	96.2690501	67.8456011	73.7357739	75.3189036
80.2951505	95.2690501	66.8456011	73.7357739	75.0376536
80.1701505	80.1701505	66.8456011	73.7357739	72.8846661
79.7951505	79.7951505	66.8456011	75.7357739	72.4159161
79.0451505	79.0451505	66.8456011	75.7357739	71.7284161
78.5451505	78.5451505	66.8456011	75.7357739	70.9784161
79.2951505	79.2951505	65.8456011	75.7357739	71.1659161
79.5451505	79.5451505	66.8456011	75.7357739	71.4784161
79.6701505	79.6701505	66.8456011	75.7357739	71.5096661
79.9201505	79.9201505	66.8456011	75.7357739	71.4471661
79.9201505	79.9201505	66.8456011	77.7357739	71.4471661
79.4201505	79.4201505	67.8456011	84.7357739	71.4471661
80.0451505	80.0451505	67.8456011	87.7357739	71.7284161
80.1701505	80.1701505	67.8456011	87.7357739	71.6346661
80.4201505	80.4201505	67.8456011	88.7357739	71.8221661
80.6701505	80.6701505	66.8456011	87.7357739	71.7596661
81.4201505	81.4201505	66.8456011	87.7357739	72.8221661
81.6701505	81.6701505	66.8456011	76.7357739	73.1346661
83.2951505	83.2951505	66.8456011	76.7357739	73.7909161
84.0451505	84.0451505	66.8456011	72.7357739	74.2284161
84.2951505	84.2951505	66.8456011	70.7357739	74.5409161
84.2951505	84.2951505	67.8456011	70.7357739	74.5409161
84.9201505	84.9201505	67.8456011	70.7357739	75.1971661
86.2951505	86.2951505	67.8456011	70.7357739	76.0409161
87.2951505	87.2951505	71.8456011	73.7357739	77.1659161
88.7951505	88.7951505	71.8456011	76.7357739	78.1659161
89.1701505	89.1701505	70.8456011	78.7357739	78.2596661
89.1701505	89.1701505	70.8456011	78.7357739	78.2596661
89.4201505	89.4201505	69.8456011	79.7357739	78.3221661
89.6701505	89.6701505	68.8456011	80.7357739	78.6346661
89.9201505	89.9201505	66.8456011	79.7357739	78.6971661
89.6701505	89.6701505	66.8456011	74.7357739	78.5096661
89.5451505	89.5451505	66.8456011	71.7357739	78.3534161
89.2951505	89.2951505	65.8456011	71.7357739	78.0409161
89.4201505	89.4201505	64.8456011	69.7357739	78.1971661
88.9201505	88.9201505	64.8456011	70.7357739	77.8221661
88.5451505	88.5451505	64.8456011	70.7357739	77.2284161
88.4201505	88.4201505	64.8456011	74.7357739	76.9471661
88.4201505	88.4201505	64.8456011	78.7357739	76.8221661
88.1701505	88.1701505	64.8456011	81.7357739	76.6346661
87.9201505	87.9201505	64.8456011	81.7357739	76.3221661

87.7951505	87.7951505	64.8456011	81.7357739		76.2909161
87.7951505	87.7951505	64.8456011	81.7357739		76.2909161
87.4201505	87.4201505	64.8456011	80.7357739		75.9471661
87.5451505	87.5451505	66.8456011	77.7357739		76.3534161
87.4201505	87.4201505	66.8456011	76.7357739		76.1971661
87.6701505	87.6701505	67.8456011	76.7357739		76.6346661
87.6701505	87.6701505	67.8456011	74.7357739		76.7596661
87.4201505	87.4201505	66.8456011	73.7357739	63	76.5721661
87.2951505	87.2951505	66.8456011	73.7357739	63	76.6659161
86.9201505	86.9201505	65.8456011	73.7357739	63	76.3221661
86.4201505	86.4201505	65.8456011	73.7357739	63	75.9471661
85.7951505	85.7951505	65.8456011	73.7357739	63	75.5409161
85.5451505	85.5451505	65.8456011	73.7357739	63	75.2284161
85.7951505	85.7951505	65.8456011	72.7357739	63	75.6659161
85.4201505	85.4201505	65.8456011	72.7357739	63	75.1971661
85.5451505	85.5451505	66.8456011	76.7357739	63	75.4784161
85.1701505	85.1701505	66.8456011	79.7357739	63	75.0096661
85.1701505	85.1701505	66.8456011	79.7357739	63	75.0096661
84.1701505	84.1701505	66.8456011	80.7357739	63	74.8846661
84.1701505	84.1701505	66.8456011	81.7357739	63	75.0096661
83.9201505	83.9201505	66.8456011	81.7357739	63	74.6971661
83.2951505	83.2951505	66.8456011	81.7357739	63	74.1659161
83.5451505	83.5451505	66.8456011	82.7357739	63	74.6034161
83.5451505	83.5451505	66.8456011	82.7357739	63	74.6034161
82.7951505	82.7951505	66.8456011	82.7357739	63	73.9159161
82.5451505	82.5451505	67.8456011	81.7357739	63	74.1034161
81.9201505	81.9201505	70.8456011	81.7357739	63	73.6971661
82.2951505	82.2951505	70.8456011	82.7357739	63	73.7909161
82.0451505	82.0451505	70.8456011	82.7357739	63	73.4784161
81.7951505	81.7951505	70.8456011	81.7357739	63	73.4159161
81.0451505	95.2690501	70.8456011	81.7357739	63	74.8814036
77.6701505	77.6701505	71.8456011	81.7357739	63	72.3846661
78.2951505	78.2951505	71.8456011	80.7357739	63	73.0409161
78.5451505	78.5451505	71.8456011	78.7357739	63	73.3534161
78.6701505	78.6701505	68.8456011	78.7357739	63	72.7596661
77.9201505	77.9201505	67.8456011	73.7357739	63	72.1971661
77.6701505	77.6701505	66.8456011	73.7357739	63	71.8846661
77.1701505	77.1701505	66.8456011	72.7357739	63	71.6346661
77.1701505	77.1701505	66.8456011	72.7357739	63	71.7596661
76.9201505	76.9201505	66.8456011	71.7357739	63	71.9471661
76.4201505	92.2690501	67.8456011	71.7357739	63	73.9282786
75.9201505	90.2690501	67.8456011	71.7357739	63	73.4907786
75.9201505	89.2690501	67.8456011	71.7357739	63	73.4907786
76.1701505	90.2690501	68.8456011	71.7357739	63	74.0220286
76.0451505	90.2690501	69.8456011	72.7357739	63	74.1314036

89.3551261	91.2690501	69.8456011	72.7357739	63	76.0451505
89.3551261	76.0451505	70.8456011	72.7357739	63	74.1421631
88.3551261	75.7951505	68.8456011	72.7357739	63	73.4859131
88.3551261	75.2951505	66.8456011	84.7357739	63	72.9234131
88.3551261	75.1701505	66.8456011	86.7357739	63	73.0327881
88.3551261	90.2690501	66.8456011	86.7357739	63	74.6701505
87.3551261	90.2690501	66.8456011	87.7357739	63	74.5451505
86.3551261	89.2690501	66.8456011	86.7357739	63	74.5451505
84.3551261	89.2690501	66.8456011	83.7357739	63	74.1701505
82.3551261	88.2690501	66.8456011	80.7357739	63	73.2951505
82.3551261	88.2690501	65.8456011	78.7357739	63	73.1701505
81.3551261	88.2690501	65.8456011	77.7357739	63	72.9201505
81.3551261	85.2690501	65.8456011	76.7357739	63	72.7951505
80.3551261	83.2690501	64.8456011	77.7357739	63	72.2951505
80.3551261	83.2690501	64.8456011	79.7357739	63	72.5451505
80.3551261	82.2690501	64.8456011	82.7357739	63	72.5451505
80.3551261	81.2690501	63.8456011	82.7357739	63	72.2951505
80.3551261	79.2690501	63.8456011	81.7357739	63	72.1701505
80.3551261	76.2690501	65.8456011	81.7357739	63	71.7951505
80.3551261	75.2690501	65.8456011	81.7357739	63	71.7951505
79.3551261	79.2690501	65.8456011	81.7357739	63	72.1701505
79.3551261	81.2690501	65.8456011	78.7357739	63	72.6701505
79.3551261	83.2690501	65.8456011	77.7357739	63	73.2951505
79.3551261	83.2690501	65.8456011	77.7357739	63	74.5451505
79.3551261	83.2690501	65.8456011	77.7357739	63	75.0451505
79.3551261	85.2690501	65.8456011	77.7357739	63	75.4201505
79.3551261	85.2690501	64.8456011	83.7357739	63	75.2951505
79.3551261	85.2690501	64.8456011	85.7357739	63	75.1701505
79.3551261	85.2690501	64.8456011	85.7357739	63	75.4201505
79.3551261	83.2690501	64.8456011	85.7357739	63	75.5451505
79.3551261	83.2690501	64.8456011	79.7357739	63	75.4201505
79.3551261	83.2690501	64.8456011	79.7357739	63	75.2951505
79.3551261	83.2690501	64.8456011	79.7357739	63	75.2951505
81.3551261	83.2690501	65.8456011	75.7357739	63	75.5451505
82.3551261	84.2690501	65.8456011	69.7357739	63	75.5451505
83.3551261	84.2690501	65.8456011	67.7357739	63	75.6701505
83.3551261	84.2690501	65.8456011	66.7357739	63	75.6701505
83.3551261	83.2690501	65.8456011	66.7357739	63	75.4201505
83.3551261	83.2690501	65.8456011	66.7357739	63	75.4201505
83.3551261	82.2690501	65.8456011	67.7357739	63	75.5451505
83.3551261	82.2690501	65.8456011	70.7357739	63	76.4201505
83.3551261	82.2690501	66.8456011	73.7357739	63	76.7951505
81.3551261	85.2690501	66.8456011	73.7357739	63	77.1701505
79.3551261	85.2690501	66.8456011	73.7357739	63	77.0451505
78.3551261	85.2690501	66.8456011	73.7357739	63	76.9201505

78.3551261	85.2690501	66.8456011	70.7357739	63	77.0451505
76.3551261	85.2690501	69.8456011	70.7357739	63	77.2951505
75.3551261	86.2690501	70.8456011	69.7357739	63	77.2951505
75.3551261	88.2690501	69.8456011	69.7357739	63	77.6701505
75.3551261	89.2690501	68.8456011	69.7357739	63	77.2951505
75.3551261	89.2690501	68.8456011	69.7357739	63	77.4201505
74.3551261	91.2690501	68.8456011	69.7357739	63	77.5451505
73.3551261	91.2690501	68.8456011	69.7357739	63	77.0451505
73.3551261	90.2690501	68.8456011	69.7357739	63	76.0451505
73.3551261	88.2690501	68.8456011	69.7357739	63	75.4201505
73.3551261	87.2690501	70.8456011	70.7357739	63	75.1701505
73.3551261	87.2690501	70.8456011	71.7357739	63	75.1701505
73.3551261	86.2690501	69.8456011	71.7357739	63	75.0451505
73.3551261	87.2690501	68.8456011	76.7357739	63	74.5451505
77.3551261	88.2690501	68.8456011	76.7357739	63	75.0451505
77.3551261	87.2690501	68.8456011	79.7357739	63	74.9201505
77.3551261	86.2690501	69.8456011	81.7357739	63	74.5451505
77.3551261	84.2690501	69.8456011	81.7357739	63	74.0451505
78.3551261	84.2690501	69.8456011	81.7357739	63	74.5451505
78.3551261	83.2690501	69.8456011	76.7357739	63	75.0451505
78.3551261	81.2690501	69.8456011	72.7357739	63	75.4201505
78.3551261	79.2690501	69.8456011	72.7357739	63	75.2951505
77.3551261	78.2690501	69.8456011	69.7357739	63	74.9201505
77.3551261	77.2690501	69.8456011	66.7357739	63	74.9201505
77.3551261	76.2690501	69.8456011	62.7357739	63	74.7951505
77.3551261	76.2690501	76.8456011	62.7357739	63	75.6701505
78.3551261	77.2690501	76.8456011	60.7357739	63	75.5451505
78.3551261	77.2690501	76.8456011	60.7357739	63	75.5451505
80.3551261	76.2690501	77.8456011	60.7357739	63	75.5451505
81.3551261	76.2690501	77.8456011	62.7357739	63	75.4201505
82.3551261	76.2690501	77.8456011	63.7357739	63	75.2951505
83.3551261	76.2690501	73.8456011	65.7357739	63	74.7951505
84.3551261	77.2690501	73.8456011	66.7357739	63	75.0451505
85.3551261	77.2690501	72.8456011	68.7357739	63	74.7951505
87.3551261	75.2690501	72.8456011	68.7357739	63	75.0451505
88.3551261	75.2690501	72.8456011	75.7357739	63	75.1701505
90.3551261	75.2690501	72.8456011	76.7357739	63	75.1701505
90.3551261	75.2690501	76.8456011	76.7357739	63	75.6701505
90.3551261	75.2690501	76.8456011	77.7357739	63	75.4201505
90.3551261	75.2690501	76.8456011	76.7357739	63	75.0451505
90.3551261	76.2690501	76.8456011	74.7357739	63	74.9201505
90.3551261	76.2690501	77.8456011	74.7357739	63	75.0451505
90.3551261	79.2690501	83.8456011	74.7357739	63	76.1701505
91.3551261	81.2690501	84.8456011	74.7357739	63	77.4201505
91.3551261	81.2690501	84.8456011	74.7357739	63	77.5451505

93.3551261	81.2690501	87.8456011	73.7357739	63	78.6701505
83.3551261	81.2690501	88.8456011	73.7357739	63	77.7951505
83.3551261	81.2690501	88.8456011	72.7357739	63	77.7951505
80.3551261	80.2690501	70.8456011	72.7357739	63	74.9201505
79.3551261	80.2690501	68.8456011	72.7357739	63	74.5451505
78.3551261	80.2690501	68.8456011	72.7357739	63	74.5451505
78.3551261	81.2690501	66.8456011	73.7357739	63	74.2951505
78.3551261	82.2690501	66.8456011	73.7357739	63	74.4201505
78.3551261	82.2690501	66.8456011	74.7357739	63	74.1701505
77.3551261	82.2690501	66.8456011	75.7357739	63	74.1701505
75.3551261	82.2690501	64.8456011	75.7357739	63	74.0451505
75.3551261	82.2690501	64.8456011	77.7357739	63	74.7951505
74.3551261	82.2690501	63.8456011	79.7357739	63	74.4201505
74.3551261	85.2690501	63.8456011	80.7357739	63	74.7951505
74.3551261	85.2690501	63.8456011	80.7357739	63	74.5451505
74.3551261	75.1701505	64.8456011	81.7357739	63	73.1577881
74.3551261	75.0451505	64.8456011	82.7357739	63	73.0171631
73.3551261	74.5451505	64.8456011	82.7357739	63	72.4546631
73.3551261	73.7951505	65.8456011	82.7357739	63	71.7359131
73.3551261	73.5451505	65.8456011	82.7357739	63	71.3296631
73.3551261	73.5451505	65.8456011	83.7357739	63	71.3296631
74.3551261	73.6701505	65.8456011	86.7357739	63	71.4702881
76.3551261	73.5451505	65.8456011	86.7357739	63	71.3296631
76.3551261	73.4201505	65.8456011	86.7357739	63	71.1890381
76.3551261	73.1701505	65.8456011	86.7357739	63	70.5327881
76.3551261	73.2951505	65.8456011	86.7357739	63	70.5484131
76.3551261	73.7951505	65.8456011	86.7357739	63	71.1109131
76.3551261	74.1701505	65.8456011	84.7357739	63	71.5327881
76.3551261	74.2951505	65.8456011	84.7357739	63	71.6734131
75.3551261	73.1701505	65.8456011	84.7357739	63	70.4077881
75.3551261	73.2951505	65.8456011	84.7357739	63	70.5484131
75.3551261	73.1701505	65.8456011	84.7357739	63	70.4077881
74.3551261	73.1701505	65.8456011	84.7357739	63	70.4077881
74.3551261	73.4201505	65.8456011	85.7357739	63	70.6890381
74.3551261	73.2951505	66.8456011	86.7357739	63	70.6734131
75.3551261	87.2690501	66.8456011	88.7357739	63	72.9201505
76.3551261	85.2690501	66.8456011	90.7357739	63	73.2951505
77.3551261	85.2690501	67.8456011	90.7357739	63	74.0451505
77.3551261	84.2690501	68.8456011	84.7357739	63	74.5451505
78.3551261	83.2690501	68.8456011	82.7357739	63	74.6701505
77.3551261	82.2690501	68.8456011	82.7357739	63	75.0451505
75.3551261	81.2690501	68.8456011	82.7357739	63	75.1701505
72.3551261	80.2690501	69.8456011	81.7357739	63	74.7951505
70.3551261	80.2690501	72.8456011	81.7357739	63	74.6701505
69.3551261	79.2690501	72.8456011	80.7357739	63	73.4583549

69.3551261	78.2690501	72.8456011	80.7357739	63	73.3333549
69.3551261	77.2690501	72.8456011	79.7357739	63	72.9271049
69.3551261	77.2690501	72.8456011	75.7357739	63	72.9427299
69.3551261	75.2690501	72.8456011	73.7357739	63	72.5208549
70.3551261	74.2690501	72.8456011	73.7357739	63	72.5208549
70.3551261	74.2690501	72.8456011	73.7357739	63	72.5208549
70.3551261	74.2690501	72.8456011	71.7357739	63	73.3646049
70.3551261	74.2690501	72.8456011	71.7357739	63	73.3646049
71.3551261	74.2690501	75.8456011	71.7357739	63	73.8802299
72.3551261	74.2690501	75.8456011	73.7357739	63	73.9271049
77.3551261	74.2690501	76.8456011	75.7357739	63	74.4739799
77.3551261	75.2690501	76.8456011	77.7357739	63	74.3177299
77.3551261	75.2690501	77.8456011	77.7357739	63	74.3021049
77.3551261	74.2690501	78.8456011	80.7357739	63	76.5451505
78.3551261	74.2690501	81.8456011	80.7357739	63	77.0451505
79.3551261	74.2690501	81.8456011	80.7357739	63	77.1701505
79.3551261	74.2690501	82.8456011	80.7357739	63	77.4201505
80.3551261	74.2690501	81.8456011	80.7357739	63	77.4201505
80.3551261	74.2690501	78.8456011	80.7357739	63	76.7951505
80.3551261	74.2690501	76.8456011	80.7357739	63	76.4201505
80.3551261	74.2690501	75.8456011	80.7357739	63	76.1701505
80.3551261	74.2690501	75.8456011	79.7357739	63	76.1701505
80.3551261	75.2690501	73.8456011	80.7357739	63	76.1701505
80.3551261	75.2690501	73.8456011	79.7357739	63	75.6701505
80.3551261	75.2690501	73.8456011	80.7357739	63	75.5451505
80.3551261	74.2690501	73.8456011	81.7357739	63	75.4201505
80.3551261	75.2690501	72.8456011	81.7357739	63	74.6701505
81.3551261	75.2690501	72.8456011	82.7357739	63	74.9201505
80.3551261	75.2690501	72.8456011	82.7357739	63	75.4201505
77.3551261	75.2690501	71.8456011	82.7357739	63	74.7951505
77.3551261	77.2690501	71.8456011	82.7357739	63	74.9201505
76.3551261	77.2690501	68.8456011	84.7357739	63	74.2951505
76.3551261	77.2690501	68.8456011	85.7357739	63	74.0451505
78.3551261	77.2690501	68.8456011	85.7357739	63	74.4201505
78.3551261	78.2690501	68.8456011	85.7357739	63	74.5451505
77.3551261	80.2690501	68.8456011	85.7357739	63	74.6701505
76.3551261	80.2690501	67.8456011	85.7357739	63	74.5451505
76.3551261	84.2690501	66.8456011	85.7357739	63	74.9201505
76.3551261	86.2690501	66.8456011	85.7357739	63	74.9201505
76.3551261	87.2690501	66.8456011	84.7357739	63	75.0451505
76.3551261	87.2690501	66.8456011	84.7357739	63	75.1701505
75.3551261	87.2690501	66.8456011	84.7357739	63	75.0451505
74.3551261	87.2690501	68.8456011	83.7357739	63	75.1701505
74.3551261	87.2690501	68.8456011	84.7357739	63	75.0451505
75.3551261	87.2690501	68.8456011	85.7357739	63	75.2951505

75.3551261	86.2690501	69.8456011	85.7357739	63	75.2951505
76.3551261	81.2690501	69.8456011	83.7357739	63	74.7951505
75.3551261	78.2690501	69.8456011	83.7357739	63	73.6701505
73.3551261	78.2690501	69.8456011	84.7357739	63	73.4201505
70.3551261	78.2690501	69.8456011	87.7357739	63	73.1701505
70.3551261	78.2690501	70.8456011	89.7357739	63	74.1701505
70.3551261	78.2690501	71.8456011	90.7357739	63	74.5451505
70.3551261	78.2690501	72.8456011	90.7357739	63	73.4201505
70.3551261	77.2690501	72.8456011	90.7357739	63	72.6701505
70.3551261	77.2690501	69.8456011	90.7357739	63	72.4201505
71.3551261	72.2690501	69.8456011	90.7357739	63	71.7951505
71.3551261	69.2690501	69.8456011	91.7357739	63	71.6701505
70.3551261	66.2690501	68.8456011	92.7357739	63	71.1701505
70.3551261	65.2690501	68.8456011	94.7357739	63	70.7951505
70.3551261	65.2690501	68.8456011	95.7357739	63	70.7951505
70.3551261	66.2690501	67.8456011	95.7357739	63	70.9201505
70.3551261	67.2690501	67.8456011	93.7357739	63	71.0451505
70.3551261	67.2690501	68.8456011	91.7357739	63	71.4201505
70.3551261	66.2690501	69.8456011	90.7357739	63	71.4201505
70.3551261	65.2690501	69.8456011	89.7357739	63	71.4201505
70.3551261	65.2690501	72.8456011	88.7357739	63	71.7951505
70.3551261	61.2690501	72.8456011	88.7357739	63	71.2951505
70.3551261	60.2690501	72.8456011	87.7357739	63	71.6701505
70.3551261	60.2690501	73.8456011	87.7357739	63	71.7951505
71.3551261	62.2690501	75.8456011	86.7357739	63	72.4201505
71.3551261	63.2690501	77.8456011	86.7357739	63	72.6701505
70.3551261	63.2690501	78.8456011	86.7357739	63	72.4201505
71.3551261	65.2690501	80.8456011	84.7357739	63	73.0451505
72.3551261	66.2690501	88.8456011	84.7357739	63	74.2951505
72.3551261	66.2690501	88.8456011	84.7357739	63	74.6701505
72.3551261	67.2690501	89.8456011	84.7357739	63	74.7951505
72.3551261	67.2690501	90.8456011	83.7357739	63	75.0451505
72.3551261	67.2690501	91.8456011	83.7357739	63	73.1013442
72.3551261	68.2690501	91.8456011	83.7357739	63	73.2419692
72.3551261	69.2690501	91.8456011	83.7357739	63	75.4201505
73.3551261	69.2690501	92.8456011	81.7357739	63	73.9763442
73.3551261	72.2690501	76.8996642	80.7357739	63	74.2419692
73.3551261	72.2690501	77.1218865	80.7357739	63	74.2732192
73.3551261	72.2690501	76.8996642	80.7357739	63	74.2419692
74.3551261	72.2690501	77.0107753	80.7357739	63	74.0075942
74.3551261	71.2690501	77.5663309	80.7357739	63	73.9607192
74.3551261	71.2690501	77.2329976	77.7357739	63	74.4607192
74.3551261	71.2690501	76.4552198	71.7357739	63	74.4450942
74.3551261	71.2690501	76.1218865	68.7357739	63	74.3200942
74.3551261	71.2690501	76.0107753	68.7357739	63	74.3044692

74.3551261	72.2690501	75.677442	66.7357739	63	74.2888442
74.3551261	71.2690501	75.4552198	64.7357739	63	74.1638442
75.3551261	71.2690501	75.7885531	63.7357739	63	74.2263442
78.3551261	71.2690501	75.7885531	63.7357739	63	74.7263442
78.3551261	70.2690501	76.1218865	63.7357739	63	74.7732192
79.3551261	70.2690501	76.677442	68.7357739	63	75.1482192
79.3551261	68.2690501	77.0107753	69.7357739	63	75.0544692
78.3551261	68.2690501	77.7885531	70.7357739	63	75.2732192
78.3551261	68.2690501	78.2329976	73.7357739	63	75.2888442
78.3551261	69.2690501	78.3441087	74.7357739	63	75.4138442
78.3551261	69.2690501	78.4552198	74.7357739	63	75.3044692
79.3551261	70.2690501	78.7885531	74.7357739	63	75.3513442
80.3551261	70.2690501	79.0107753	74.7357739	63	75.6325942
79.3551261	70.2690501	79.3441087	75.7357739	63	75.6638442
79.3551261	71.2690501	78.7885531	75.7357739	63	75.7107192
78.3551261	70.2690501	79.0107753	78.7357739	63	75.4450942
77.3551261	70.2690501	78.7885531	78.7357739	63	75.2888442
77.3551261	71.2690501	79.5663309	79.7357739	63	75.5075942
77.3551261	71.2690501	79.677442	79.7357739	63	76.1482192
77.3551261	74.2690501	79.7885531	79.7357739	63	76.1638442
77.3551261	74.2690501	79.677442	79.7357739	63	76.2732192
77.3551261	74.2690501	80.1218865	79.7357739	63	76.2107192
77.3551261	75.2690501	80.2329976	80.7357739	63	76.7107192
77.3551261	75.2690501	80.2329976	83.7357739	63	76.6638442
75.3551261	74.2690501	79.8996642	83.7357739	63	76.2419692
74.3551261	75.2690501	80.0107753	83.7357739	63	76.1325942
73.3551261	75.2690501	79.7885531	82.7357739	63	76.2419692
73.3551261	76.2690501	80.0107753	82.7357739	63	76.6482192
73.3551261	77.2690501	95.8456011	81.7357739	63	79.2951505
73.3551261	77.2690501	89.8456011	81.7357739	63	79.4201505
73.3551261	76.2690501	89.8456011	81.7357739	63	79.1701505
73.3551261	76.2690501	88.8456011	81.7357739	63	80.5451505
73.3551261	77.2690501	90.8456011	81.7357739	63	80.9201505
73.3551261	77.2690501	89.8456011	81.7357739	63	80.7951505
78.3551261	76.2690501	88.8456011	81.7357739	63	81.1701505
82.3551261	76.2690501	88.8456011	77.7357739	63	81.7951505
83.3551261	76.2690501	87.8456011	77.7357739	63	81.5451505
86.3551261	75.2690501	83.8456011	77.7357739	63	81.6701505
83.3551261	75.2690501	83.8456011	76.7357739	63	80.0451505
83.3551261	76.2690501	84.8456011	76.7357739	63	79.6701505
81.3551261	76.2690501	86.8456011	76.7357739	63	79.4201505
80.3551261	76.2690501	85.8456011	78.7357739	63	78.7951505
80.3551261	76.2690501	86.8456011	79.7357739	63	78.7951505
80.3551261	75.2690501	84.8456011	70.7357739	63	78.7951505
80.3551261	75.2690501	84.8456011	67.7357739	63	78.7951505

80.3551261	75.2690501	84.8456011	67.7357739	63	78.6701505
78.3551261	76.2690501	79.8456011	67.7357739	63	78.4201505
78.3551261	77.2690501	72.8456011	61.7357739	63	77.5451505
77.3551261	76.2690501	68.8456011	61.7357739	63	76.5451505
77.3551261	76.2690501	67.8456011	58.7357739	63	76.1701505
77.3551261	75.2690501	67.8456011	61.7357739	63	75.5451505
76.3551261	74.2690501	67.8456011	62.7357739	63	75.1701505
78.3551261	74.2690501	67.8456011	65.7357739	63	74.6701505
79.3551261	74.2690501	67.8456011	72.7357739	63	74.0451505
80.3551261	73.2690501	67.8456011	73.7357739	63	73.4201505
80.3551261	74.2690501	67.8456011	73.7357739	63	73.4201505
78.3551261	74.2690501	67.8456011	72.7357739	63	72.7951505
78.3551261	74.2690501	67.8456011	72.7357739	63	72.6701505
77.3551261	75.2690501	66.8456011	76.7357739	63	72.9201505
76.3551261	75.2690501	66.8456011	80.7357739	63	73.0451505
74.3551261	75.2690501	66.8456011	80.7357739	63	73.0451505
74.3551261	73.2690501	67.8456011	80.7357739	63	72.9201505
74.3551261	72.2690501	67.8456011	80.7357739	63	73.4201505
73.3551261	72.2690501	67.8456011	81.7357739	63	73.2951505
73.3551261	72.2690501	69.8456011	82.7357739	63	73.7951505
74.3551261	72.2690501	71.8456011	82.7357739	63	74.0451505
74.3551261	74.2690501	74.8456011	82.7357739	63	74.7951505
73.3551261	74.2690501	80.8456011	84.7357739	63	75.4201505
73.3551261	74.2690501	81.8456011	84.7357739	63	75.4201505
73.3551261	73.2690501	82.8456011	84.7357739	63	75.6701505
73.3551261	72.2690501	83.8456011	84.7357739	63	75.9201505
72.3551261	72.2690501	83.8456011	84.7357739	63	75.6701505
72.3551261	72.2690501	83.8456011	89.7357739	63	75.1701505
72.3551261	72.2690501	84.8456011	90.7357739	63	75.1701505
71.3551261	73.2690501	85.8456011	90.7357739	63	75.1701505
71.3551261	73.2690501	87.8456011	90.7357739	63	75.4201505
70.3551261	73.2690501	87.8456011	90.7357739	63	75.2951505
70.3551261	73.2690501	87.8456011	89.7357739	63	75.2951505
69.3551261	71.2690501	83.8456011	89.7357739	63	74.2951505
66.3551261	71.2690501	78.8456011	89.7357739	63	74.0451505
66.3551261	72.2690501	75.8456011	84.7357739	63	75.1701505
67.3551261	73.2690501	74.8456011	77.7357739	63	75.1701505
68.3551261	73.2690501	70.8456011	70.7357739	63	73.7951505
69.3551261	73.2690501	71.8456011	68.7357739	63	74.1701505
70.3551261	73.2690501	71.8456011	68.7357739	63	74.2951505
69.3551261	72.2690501	71.8456011	68.7357739	63	73.9201505
69.3551261	71.2690501	71.8456011	67.7357739	63	73.2951505
69.3551261	71.2690501	71.8456011	67.7357739	63	72.9201505
69.3551261	71.2690501	71.8456011	67.7357739	63	72.7951505
68.3551261	71.2690501	73.8456011	67.7357739	63	72.7951505

68.3551261	73.2690501	73.8456011	67.7357739	63	72.0451505
69.3551261	74.2690501	73.8456011	66.7357739	63	71.1701505
70.3551261	74.2690501	74.8456011	69.7357739	63	70.6701505
71.3551261	74.2690501	74.8456011	74.7357739	63	70.0451505
71.3551261	70.2690501	75.8456011	78.7357739	63	69.5451505
71.3551261	68.2690501	75.8456011	80.7357739	63	69.1701505
71.3551261	68.2690501	76.8456011	80.7357739	63	69.1701505
71.3551261	68.2690501	76.8456011	78.7357739	63	69.0451505
71.3551261	70.2690501	77.8456011	78.7357739	63	69.4201505
72.3551261	71.2690501	77.8456011	78.7357739	63	69.6701505
72.3551261	70.2690501	77.8456011	78.7357739	63	69.7951505
72.3551261	70.2690501	79.8456011	79.7357739	63	70.0451505
72.3551261	69.2690501	79.8456011	80.7357739	63	70.0451505
73.3551261	69.2690501	79.8456011	80.7357739	63	70.1701505
73.3551261	68.2690501	79.8456011	79.7357739	63	70.0451505
73.3551261	68.2690501	80.8456011	78.7357739	63	70.0451505
73.3551261	67.2690501	80.8456011	76.7357739	63	70.0451505
73.3551261	68.2690501	81.8456011	75.7357739	63	70.4201505
73.3551261	68.2690501	82.8456011	74.7357739	63	70.5451505
73.3551261	70.2690501	74.8456011	73.7357739	63	69.9201505
73.3551261	71.2690501	72.8456011	73.7357739	63	70.2951505
73.3551261	72.2690501	72.8456011	73.7357739	63	70.9201505
73.3551261	71.2690501	68.8456011	73.7357739	63	70.1701505
73.3551261	69.2690501	65.8456011	74.7357739	63	69.6701505
73.3551261	68.2690501	64.8456011	75.7357739	63	69.5451505
72.3551261	68.2690501	64.8456011	77.7357739	63	69.1701505
72.3551261	69.2690501	64.8456011	78.7357739	63	69.1701505
71.3551261	70.2690501	64.8456011	80.7357739	63	68.5451505
71.3551261	69.2690501	62.8456011	80.7357739	63	68.1701505
71.3551261	69.2690501	62.8456011	78.7357739	63	68.2951505
71.3551261	69.2690501	62.8456011	77.7357739	63	68.7951505
73.3551261	69.2690501	63.8456011	77.7357739	63	69.1701505
74.3551261	68.2690501	63.8456011	77.7357739	63	69.4201505
74.3551261	67.2690501	63.8456011	77.7357739	63	69.7951505
73.3551261	69.2690501	63.8456011	77.7357739	63	69.7951505
73.3551261	72.2690501	63.8456011	77.7357739	63	70.4201505
73.3551261	72.2690501	63.8456011	77.7357739	63	70.5451505
73.3551261	73.2690501	63.8456011	77.7357739	63	70.7951505
73.3551261	72.2690501	63.8456011	78.7357739	63	70.9201505
72.3551261	71.2690501	63.8456011	79.7357739	63	71.1701505
72.3551261	71.2690501	63.8456011	81.7357739	63	71.2951505
72.3551261	71.2690501	65.8456011	81.7357739	63	71.5451505
73.3551261	74.2690501	66.8456011	82.7357739	63	72.6701505
73.3551261	74.2690501	68.8456011	82.7357739	63	73.1701505
73.3551261	74.2690501	73.8456011	82.7357739	63	74.6701505

73.3551261	75.2690501	77.8456011	82.7357739	63	75.1701505
74.3551261	75.2690501	77.8456011	82.7357739	63	74.9201505
74.3551261	74.2690501	79.8456011	80.7357739	63	75.0451505
74.3551261	71.2690501	80.8456011	78.7357739	63	74.5451505
74.3551261	68.2690501	82.8456011	77.7357739	63	74.1701505
74.3551261	67.2690501	84.8456011	77.7357739	63	74.1701505
76.3551261	66.2690501	86.8456011	76.7357739	63	74.5451505
76.3551261	66.2690501	75.677442	76.7357739	63	73.1325942
76.3551261	66.2690501	76.3441087	74.7357739	63	73.1325942
77.3551261	66.2690501	76.2329976	72.7357739	63	73.1482192
76.3551261	67.2690501	76.1218865	70.7357739	63	72.5388442
75.3551261	67.2690501	76.677442	70.7357739	63	72.7419692
75.3551261	67.2690501	76.8996642	69.7357739	63	73.0388442
75.3551261	66.2690501	76.677442	69.7357739	63	73.0075942
75.3551261	65.2690501	77.0107753	69.7357739	63	72.8044692
74.3551261	65.2690501	77.2329976	69.7357739	63	73.2107192
74.3551261	65.2690501	77.677442	68.7357739	63	73.5388442
74.3551261	65.2690501	78.0107753	67.7357739	63	73.9763442
74.3551261	66.2690501	77.7885531	67.7357739	63	74.4450942
74.3551261	66.2690501	79.0107753	76.7357739	63	74.3513442
74.3551261	66.2690501	79.7885531	76.7357739	63	74.5857192
74.3551261	68.2690501	81.0107753	84.7357739	63	75.5075942
74.3551261	69.2690501	81.4552198	88.7357739	63	75.6325942
74.3551261	69.2690501	81.4552198	89.7357739	63	75.4919692
72.3551261	69.2690501	81.4552198	88.7357739	63	75.2575942
71.3551261	70.2690501	81.1218865	88.7357739	63	75.3357192
70.3551261	71.2690501	81.2329976	88.7357739	63	74.9763442
71.3551261	70.2690501	81.2329976	88.7357739	63	75.1013442
71.3551261	69.2690501	81.0107753	87.7357739	63	75.0857192
71.3551261	69.2690501	80.0107753	84.7357739	63	74.8669692
71.3551261	68.2690501	79.7885531	84.7357739	63	74.0857192
71.3551261	68.2690501	78.8996642	75.7357739	63	73.9763442
72.3551261	68.2690501	78.0107753	67.7357739	63	74.1013442
71.3551261	69.2690501	78.1218865	67.7357739	63	73.9919692
71.3551261	70.2690501	76.7885531	67.7357739	63	73.9294692
71.3551261	70.2690501	77.3441087	67.7357739	63	74.0075942
71.3551261	70.2690501	90.8456011	66.7357739	63	76.1701505
72.3551261	69.2690501	89.8456011	67.7357739	63	75.7951505
72.3551261	68.2690501	89.8456011	71.7357739	63	75.5451505
73.3551261	67.2690501	89.8456011	73.7357739	63	75.2951505
73.3551261	67.2690501	89.8456011	73.7357739	63	74.9201505
73.3551261	67.2690501	87.8456011	75.7357739	63	75.0451505
73.3551261	67.2690501	86.8456011	76.7357739	63	75.1701505
75.3551261	68.2690501	86.8456011	76.7357739	63	75.4201505
75.3551261	68.2690501	86.8456011	77.7357739	63	75.2951505

76.3551261	68.2690501	86.8456011	77.7357739	63	75.2951505
76.3551261	68.2690501	86.8456011	77.7357739	63	75.2951505
76.3551261	68.2690501	86.8456011	77.7357739	63	75.0451505
74.3551261	67.2690501	86.8456011	78.7357739	63	74.7951505
73.3551261	67.2690501	86.8456011	78.7357739	63	74.4201505
73.3551261	67.2690501	86.8456011	81.7357739	63	74.4201505
73.3551261	67.2690501	86.8456011	81.7357739	63	73.9201505
69.3551261	68.2690501	86.8456011	81.7357739	63	73.6701505
68.3551261	68.2690501	86.8456011	80.7357739	63	73.5451505
67.3551261	68.2690501	85.8456011	77.7357739	63	73.1701505
66.3551261	68.2690501	85.8456011	77.7357739	63	72.9201505
65.3551261	68.2690501	85.8456011	77.7357739	63	72.2951505
66.3551261	68.2690501	85.8456011	71.7357739	63	71.9201505
66.3551261	68.2690501	86.8456011	70.7357739	63	72.0451505
66.3551261	68.2690501	72.1218865	70.7357739	63	70.2263442
66.3551261	69.2690501	72.2329976	70.7357739	63	70.2419692
67.3551261	69.2690501	72.2329976	70.7357739	63	70.3669692
68.3551261	69.2690501	72.5663309	69.7357739	63	70.4294692
69.3551261	69.2690501	72.3441087	69.7357739	63	70.6482192
69.3551261	67.2690501	73.3441087	69.7357739	63	70.5388442
70.3551261	67.2690501	74.1218865	69.7357739	63	71.3982192
70.3551261	66.2690501	74.7885531	69.7357739	63	72.1169692
70.3551261	65.2690501	75.1218865	69.7357739	63	72.9138442
70.3551261	65.2690501	74.5663309	69.7357739	63	72.9607192
70.3551261	64.2690501	74.677442	69.7357739	63	72.3513442
70.3551261	64.2690501	75.0107753	71.7357739	63	72.4919692
70.3551261	64.2690501	75.3441087	74.7357739	63	72.6169692
71.3551261	66.2690501	75.1218865	74.7357739	63	72.5857192
71.3551261	66.2690501	74.7885531	73.7357739	63	72.3044692
71.3551261	66.2690501	74.7885531	73.7357739	63	72.0544692
73.3551261	66.2690501	74.677442	73.7357739	63	72.0388442
74.3551261	66.2690501	74.677442	73.7357739	63	71.9138442
75.3551261	66.2690501	73.7885531	73.7357739	63	71.7888442
77.3551261	65.2690501	83.8456011	73.7357739	63	72.9201505
78.3551261	65.2690501	76.8456011	74.7357739	63	72.2951505
78.3551261	65.2690501	71.8456011	74.7357739	63	71.7951505
80.3551261	65.2690501	69.8456011	76.7357739	63	71.9201505
79.3551261	64.2690501	68.8456011	76.7357739	63	71.6701505
79.3551261	66.2690501	68.8456011	78.7357739	63	71.6701505
78.3551261	66.2690501	68.8456011	78.7357739	63	71.5451505
78.3551261	66.2690501	68.8456011	78.7357739	63	72.1701505
78.3551261	66.2690501	67.8456011	78.7357739	63	72.2951505
78.3551261	66.2690501	68.8456011	78.7357739	63	73.0451505
78.3551261	66.2690501	67.8456011	78.7357739	63	73.1701505
78.3551261	66.2690501	66.8456011	78.7357739	63	73.5451505

78.3551261	66.2690501	66.8456011	77.7357739	63	73.7951505
78.3551261	66.2690501	65.8456011	76.7357739	63	74.0451505
78.3551261	66.2690501	65.8456011	76.7357739	63	74.1701505
78.3551261	67.2690501	65.8456011	76.7357739	63	74.2951505
79.3551261	66.2690501	66.8456011	75.7357739	63	74.4201505
80.3551261	64.2690501	67.8456011	75.7357739	63	74.0451505
80.3551261	63.2690501	68.8456011	74.7357739	63	73.9201505
80.3551261	62.2690501	69.8456011	74.7357739	63	73.9201505
79.3551261	62.2690501	70.8456011	74.7357739	63	73.7951505
79.3551261	62.2690501	70.8456011	75.7357739	63	73.6701505
77.3551261	62.2690501	71.8456011	75.7357739	63	73.6701505
77.3551261	62.2690501	71.8456011	76.7357739	63	75.1701505
77.3551261	62.2690501	70.8456011	77.7357739	63	75.4201505
74.3551261	62.2690501	70.8456011	77.7357739	63	73.5989799
74.3551261	62.2690501	69.8456011	78.7357739	63	73.4739799
74.3551261	62.2690501	69.8456011	77.7357739	63	73.5989799
73.3551261	62.2690501	68.8456011	77.7357739	63	73.1927299
74.3551261	62.2690501	68.8456011	77.7357739	63	73.5989799
75.3551261	63.2690501	68.8456011	78.7357739	63	76.0451505
75.3551261	63.2690501	68.8456011	78.7357739	63	73.8802299
75.3551261	63.2690501	70.8456011	79.7357739	63	74.1302299
74.3551261	64.2690501	70.8456011	79.7357739	63	73.5989799
74.3551261	65.2690501	70.8456011	79.7357739	63	73.1927299
74.3551261	65.2690501	70.8456011	78.7357739	63	71.4114799
73.3551261	66.2690501	70.8456011	79.7357739	63	70.8489799
73.3551261	66.2690501	70.8456011	79.7357739	63	70.9739799
72.3551261	66.2690501	70.8456011	83.7357739	63	70.9896049
71.3551261	66.2690501	70.8456011	83.7357739	63	71.1302299
71.3551261	66.2690501	70.8456011	84.7357739	63	71.3021049
72.3551261	66.2690501	71.8456011	77.7357739	63	71.5677299
72.3551261	63.2690501	71.8456011	71.7357739	63	71.3021049
71.3551261	62.2690501	71.8456011	71.7357739	63	70.8958549
70.3551261	62.2690501	71.8456011	66.7357739	63	70.5052299
70.3551261	62.2690501	71.8456011	66.7357739	63	70.3802299
71.3551261	62.2690501	71.8456011	65.7357739	63	69.9896049
71.3551261	62.2690501	73.8456011	65.7357739	63	71.6458549
71.3551261	63.2690501	73.8456011	65.7357739	63	72.6614799
71.3551261	64.2690501	73.8456011	65.7357739	63	73.2239799
71.3551261	64.2690501	73.8456011	65.7357739	63	73.5052299
71.3551261	65.2690501	74.8456011	68.7357739	63	74.6146049
71.3551261	65.2690501	74.8456011	68.7357739	63	74.7708549
71.3551261	64.2690501	73.8456011	72.7357739	63	75.9114799
71.3551261	63.2690501	73.8456011	72.7357739	63	76.4583549
72.3551261	62.2690501	73.8456011	72.7357739	63	76.7396049
73.3551261	62.2690501	71.8456011	72.7357739	63	76.4896049

73.3551261	61.2690501	68.8456011	72.7357739	63	73.7864799
73.3551261	61.2690501	68.8456011	72.7357739	63	74.1701505
73.3551261	62.2690501	66.8456011	71.7357739	63	73.0451505
73.3551261	63.2690501	66.8456011	71.7357739	63	72.9201505
73.3551261	62.2690501	66.8456011	71.7357739	63	72.4201505
73.3551261	62.2690501	66.8456011	71.7357739	63	72.4201505
73.3551261	61.2690501	66.8456011	71.7357739	63	72.2951505
73.3551261	62.2690501	66.8456011	71.7357739	63	72.2951505
73.3551261	63.2690501	66.8456011	71.7357739	63	72.2951505
73.3551261	63.2690501	65.8456011	71.7357739	63	72.2951505
71.3551261	63.2690501	65.8456011	72.7357739	63	71.9201505
71.3551261	63.2690501	65.8456011	72.7357739	63	71.9201505
71.3551261	65.2690501	66.8456011	73.7357739	63	71.6701505
71.3551261	66.2690501	66.8456011	73.7357739	63	71.5451505
72.3551261	66.2690501	68.8456011	73.7357739	63	71.5451505
72.3551261	67.2690501	68.8456011	73.7357739	63	71.7951505
73.3551261	70.2690501	68.8456011	75.7357739	63	71.9201505
73.3551261	70.2690501	69.8456011	76.7357739	63	72.1701505
72.3551261	69.2690501	69.8456011	77.7357739	63	72.1701505
72.3551261	67.2690501	69.8456011	78.7357739	63	72.2951505
70.3551261	67.2690501	69.8456011	78.7357739	63	72.4201505
69.3551261	67.2690501	69.8456011	79.7357739	63	72.4201505
69.3551261	66.2690501	69.8456011	79.7357739	63	72.7951505
69.3551261	66.2690501	67.8456011	82.7357739	63	72.5451505
69.3551261	66.2690501	67.8456011	82.7357739	63	73.0451505
69.3551261	66.2690501	67.8456011	82.7357739	63	72.9201505
69.3551261	66.2690501	67.8456011	82.7357739	63	72.4201505
69.3551261	65.2690501	67.8456011	83.7357739	63	72.1701505
69.3551261	66.2690501	66.8456011	83.7357739	63	71.9201505
69.3551261	66.2690501	66.8456011	85.7357739	63	71.5451505
69.3551261	66.2690501	66.8456011	85.7357739	63	71.4201505
69.3551261	67.2690501	66.8456011	86.7357739	63	70.6701505
68.3551261	67.2690501	67.8456011	89.7357739	63	70.5451505
68.3551261	67.2690501	69.8456011	89.7357739	63	70.7951505
70.3551261	68.2690501	70.8456011	89.7357739	63	71.0451505
70.3551261	68.2690501	70.8456011	87.7357739	63	70.9201505
76.3551261	68.2690501	71.8456011	85.7357739	63	72.1701505
77.3551261	69.2690501	71.8456011	79.7357739	63	72.4201505
78.3551261	69.2690501	72.8456011	79.7357739	63	72.7951505
78.3551261	69.2690501	72.8456011	75.7357739	63	72.6701505
77.3551261	70.2690501	72.8456011	72.7357739	63	72.1701505
77.3551261	70.2690501	72.8456011	72.7357739	63	71.9201505
77.3551261	70.2690501	72.8456011	71.7357739	63	72.0451505
77.3551261	70.2690501	73.8456011	71.7357739	63	72.1701505
77.3551261	70.2690501	74.8456011	71.7357739	63	72.1701505

77.3551261	71.2690501	74.8456011	70.7357739	63	70.5451505
79.3551261	70.2690501	73.8456011	70.7357739	63	70.4201505
79.3551261	71.2690501	73.8456011	72.7357739	63	70.2951505
80.3551261	73.2690501	74.8456011	73.7357739	63	70.6701505
79.3551261	73.2690501	74.8456011	73.7357739	63	70.9201505
77.3551261	73.2690501	74.8456011	75.7357739	63	71.6701505
76.3551261	73.2690501	74.8456011	80.7357739	63	71.7951505
76.3551261	72.2690501	76.8456011	81.7357739	63	72.4201505
75.3551261	72.2690501	77.8456011	82.7357739	63	72.4201505
75.3551261	72.2690501	77.8456011	83.7357739	63	72.5451505
74.3551261	71.2690501	75.2329976	85.7357739	63	72.0544692
74.3551261	71.2690501	75.1218865	85.7357739	63	71.4138442
73.3551261	71.2690501	76.5663309	85.7357739	63	71.3669692
73.3551261	78.2690501	76.677442	85.7357739	63	73.0075942
73.3551261	79.2690501	76.677442	85.7357739	63	73.2575942
71.3551261	79.2690501	75.677442	85.7357739	63	73.1169692
71.3551261	70.2690501	75.677442	85.7357739	63	71.9919692
71.3551261	67.2690501	75.3441087	85.7357739	63	71.9450942
71.3551261	65.2690501	75.3441087	85.7357739	63	71.6950942
71.3551261	65.2690501	75.2329976	84.7357739	63	71.6950942
71.3551261	65.2690501	75.1218865	84.7357739	63	71.6794692
71.3551261	65.2690501	74.8996642	83.7357739	63	71.5388442
71.3551261	65.2690501	74.677442	82.7357739	63	71.5232192
71.3551261	66.2690501	74.0107753	82.7357739	63	71.3044692
71.3551261	64.2690501	73.3441087	81.7357739	63	70.4763442
71.3551261	63.2690501	88.8456011	81.7357739	63	70.1325942
70.3551261	65.2690501	77.8456011	80.7357739	63	71.1701505
70.3551261	66.2690501	72.8456011	80.7357739	63	70.5451505
70.3551261	66.2690501	72.8456011	77.7357739	63	70.7951505
71.3551261	65.2690501	70.8456011	77.7357739	63	70.6701505
71.3551261	63.2690501	70.8456011	77.7357739	63	70.5451505
71.3551261	62.2690501	70.8456011	76.7357739	63	70.4201505
71.3551261	62.2690501	70.8456011	76.7357739	63	70.4201505
70.3551261	61.2690501	70.8456011	75.7357739	63	70.1701505
70.3551261	61.2690501	70.8456011	75.7357739	63	70.2951505
70.3551261	61.2690501	70.8456011	75.7357739	63	70.5451505
70.3551261	60.2690501	70.8456011	76.7357739	63	70.4201505
70.3551261	59.2690501	70.8456011	76.7357739	63	70.5451505
70.3551261	60.2690501	69.8456011	76.7357739	63	70.4201505
70.3551261	62.2690501	66.8456011	77.7357739	63	70.4201505
70.3551261	64.2690501	66.8456011	77.7357739	63	70.5451505
70.3551261	64.2690501	66.8456011	82.7357739	63	70.0451505
70.3551261	65.2690501	66.8456011	82.7357739	63	69.7951505
70.3551261	65.2690501	66.8456011	82.7357739	63	70.0451505
69.3551261	66.2690501	66.8456011	84.7357739	63	69.7951505

70.3551261	66.2690501	66.8456011	85.7357739	63	69.7951505
70.3551261	66.2690501	66.8456011	85.7357739	63	70.0451505
70.3551261	66.2690501	66.8456011	85.7357739	63	70.0451505
70.3551261	66.2690501	66.8456011	84.7357739	63	70.2951505
70.3551261	66.2690501	65.8456011	84.7357739	63	70.0451505
71.3551261	65.2690501	65.8456011	84.7357739	63	69.5451505
71.3551261	65.2690501	64.8456011	84.7357739	63	69.2951505
71.3551261	65.2690501	64.8456011	84.7357739	63	69.6701505
71.3551261	65.2690501	64.8456011	84.7357739	63	70.0451505
71.3551261	65.2690501	64.8456011	84.7357739	63	70.2951505
72.3551261	65.2690501	64.8456011	84.7357739	63	71.6701505
72.3551261	65.2690501	64.8456011	83.7357739	63	73.0451505
72.3551261	64.2690501	64.8456011	83.7357739	63	73.6701505
72.3551261	64.2690501	64.8456011	76.7357739	63	74.2951505
73.3551261	64.2690501	65.8456011	71.7357739	63	75.0451505
73.3551261	61.2690501	65.8456011	69.7357739	63	72.4583549
73.3551261	61.2690501	64.8456011	68.7357739	63	72.0677299
73.3551261	61.2690501	64.8456011	69.7357739	63	71.9114799
73.3551261	62.2690501	64.8456011	73.7357739	63	72.6701505
72.3551261	61.2690501	64.8456011	75.7357739	63	72.1701505
72.3551261	61.2690501	65.8456011	75.7357739	63	72.0451505
70.3551261	60.2690501	67.8456011	74.7357739	63	71.2951505
70.3551261	60.2690501	68.8456011	72.7357739	63	70.7951505
70.3551261	60.2690501	69.8456011	71.7357739	63	70.7951505
70.3551261	60.2690501	69.8456011	71.7357739	63	70.1701505
70.3551261	60.2690501	69.8456011	73.7357739	63	69.7951505
70.3551261	60.2690501	69.8456011	74.7357739	63	69.7951505
70.3551261	60.2690501	69.8456011	73.7357739	63	69.6701505
70.3551261	60.2690501	68.8456011	73.7357739	63	70.2951505
70.3551261	58.2690501	67.8456011	73.7357739	63	70.2951505
71.3551261	57.2690501	65.8456011	74.7357739	63	69.6701505
71.3551261	57.2690501	65.8456011	74.7357739	63	69.1701505
76.3551261	57.2690501	65.8456011	75.7357739	63	69.6701505
76.3551261	57.2690501	65.8456011	76.7357739	63	69.5451505
81.3551261	57.2690501	65.8456011	79.7357739	63	70.1701505
81.3551261	57.2690501	65.8456011	80.7357739	63	70.1701505
81.3551261	57.2690501	65.8456011	81.7357739	63	70.2951505
81.3551261	57.2690501	65.8456011	81.7357739	63	70.0451505
80.3551261	57.2690501	65.8456011	80.7357739	63	69.9201505
80.3551261	61.2690501	65.8456011	78.7357739	63	69.9201505
80.3551261	62.2690501	65.8456011	78.7357739	63	69.7951505
80.3551261	62.2690501	65.8456011	76.7357739	63	69.7951505
79.3551261	64.2690501	65.8456011	75.7357739	63	70.2951505
78.3551261	64.2690501	65.8456011	74.7357739	63	70.1701505
78.3551261	64.2690501	65.8456011	72.7357739	63	70.1701505

77.3551261	66.2690501	65.8456011	72.7357739	63	70.5451505
77.3551261	66.2690501	65.8456011	71.7357739	63	70.4201505
80.3551261	65.2690501	65.8456011	71.7357739	63	70.9201505
79.3551261	65.2690501	65.8456011	71.7357739	63	70.9201505
79.3551261	65.2690501	66.8456011	75.7357739	63	71.0451505
79.3551261	65.2690501	66.8456011	76.7357739	63	70.9201505
79.3551261	65.2690501	66.8456011	76.7357739	63	71.5451505
78.3551261	66.2690501	67.8456011	76.7357739	63	72.7951505
77.3551261	66.2690501	71.8456011	72.7357739	63	74.4201505
73.3551261	66.2690501	72.8456011	70.7357739	63	73.0451505
73.3551261	66.2690501	72.8456011	70.7357739	63	72.4201505
73.3551261	66.2690501	72.8456011	71.7357739	63	71.7951505
73.3551261	63.2690501	81.8456011	71.7357739	63	72.4201505
73.3551261	63.2690501	81.8456011	72.7357739	63	71.9201505
73.3551261	63.2690501	84.8456011	73.7357739	63	72.4201505
72.3551261	63.2690501	86.8456011	73.7357739	63	73.0451505
72.3551261	63.2690501	86.8456011	73.7357739	63	73.2951505
76.3551261	63.2690501	86.8456011	73.7357739	63	73.7951505
78.3551261	63.2690501	88.8456011	74.7357739	63	74.2951505
79.3551261	63.2690501	89.8456011	75.7357739	63	74.4201505
79.3551261	63.2690501	90.8456011	76.7357739	63	74.9201505
79.3551261	63.2690501	75.2329976	76.7357739	63	73.0700942
79.3551261	63.2690501	74.677442	75.7357739	63	72.8825942
79.3551261	63.2690501	73.7885531	71.7357739	63	72.3200942
79.3551261	64.2690501	76.8456011	69.7357739	63	72.2951505
81.3551261	64.2690501	67.8456011	69.7357739	63	71.6701505
81.3551261	63.2690501	67.8456011	70.7357739	63	71.7951505
81.3551261	62.2690501	67.8456011	70.7357739	63	71.5451505
81.3551261	62.2690501	67.8456011	70.7357739	63	71.2951505
81.3551261	62.2690501	67.8456011	70.7357739	63	71.1701505
80.3551261	61.2690501	66.8456011	72.7357739	63	70.7951505
80.3551261	60.2690501	66.8456011	74.7357739	63	70.4201505
80.3551261	63.2690501	66.8456011	75.7357739	63	70.6701505
80.3551261	63.2690501	66.8456011	73.7357739	63	70.9201505
80.3551261	65.2690501	66.8456011	73.7357739	63	71.2951505
79.3551261	67.2690501	66.8456011	73.7357739	63	71.7951505
79.3551261	69.2690501	65.8456011	74.7357739	63	72.2951505
79.3551261	68.2690501	65.8456011	74.7357739	63	72.7951505
79.3551261	67.2690501	65.8456011	74.7357739	63	73.7951505
79.3551261	66.2690501	65.8456011	74.7357739	63	73.7951505
76.3551261	66.2690501	65.8456011	74.7357739	63	73.2951505
71.3551261	66.2690501	65.8456011	72.7357739	63	72.5451505
70.3551261	66.2690501	65.8456011	70.7357739	63	72.2951505
69.3551261	66.2690501	68.8456011	69.7357739	63	72.7951505
69.3551261	67.2690501	70.8456011	69.7357739	63	73.4201505

69.3551261	67.2690501	71.8456011	69.7357739	63	73.7951505
69.3551261	68.2690501	71.8456011	71.7357739	63	73.7951505
68.3551261	68.2690501	71.8456011	72.7357739	63	73.7951505
63.3551261	65.2690501	71.8456011	73.7357739	63	71.7951505
61.3551261	59.2690501	71.8456011	73.7357739	63	70.6701505
61.3551261	59.2690501	71.8456011	75.7357739	63	70.6701505
60.3551261	56.2690501	71.8456011	75.7357739	63	66.8200713
60.3551261	56.2690501	71.8456011	76.7357739	63	66.5388213
60.3551261	56.2690501	71.8456011	77.7357739	63	66.6794463
60.3551261	56.2690501	71.8456011	77.7357739	63	66.3981963
62.3551261	56.2690501	71.8456011	78.7357739	63	66.6950713
62.3551261	56.2690501	72.8456011	78.7357739	63	66.9606963
62.3551261	57.2690501	72.8456011	79.7357739	63	68.9201505
62.3551261	57.2690501	73.8456011	80.7357739	63	68.5451505
63.3551261	63.2690501	75.8456011	83.7357739	63	69.6701505
63.3551261	63.2690501	75.8456011	83.7357739	63	69.6701505
63.3551261	66.2690501	77.8456011	83.7357739	63	70.7951505
64.3551261	68.2690501	77.8456011	80.7357739	63	71.4201505
64.3551261	70.2690501	76.8456011	79.7357739	63	71.6701505
63.3551261	72.2690501	76.8456011	78.7357739	63	71.7951505
64.3551261	74.2690501	76.8456011	77.7357739	63	72.0451505
65.3551261	75.2690501	77.8456011	77.7357739	63	72.2951505
65.3551261	79.2690501	77.8456011	77.7357739	63	72.7951505
66.3551261	79.2690501	78.8456011	77.7357739	63	73.0451505
67.3551261	83.2690501	79.7885531	77.7357739	63	73.9450942
68.3551261	83.2690501	84.0107753	77.7357739	63	74.7888442
70.3551261	84.2690501	86.2329976	78.7357739	63	75.9607192
70.3551261	83.2690501	86.4552198	78.7357739	63	76.1169692
69.3551261	83.2690501	86.677442	79.7357739	63	75.8825942
70.3551261	83.2690501	87.0107753	78.7357739	63	76.0700942
70.3551261	83.2690501	87.0107753	78.7357739	63	76.1950942
70.3551261	82.2690501	86.677442	77.7357739	63	76.0388442
68.3551261	82.2690501	86.5663309	77.7357739	63	75.6482192
68.3551261	81.2690501	86.8996642	77.7357739	63	75.3200942
68.3551261	82.2690501	86.677442	77.7357739	63	75.5388442
67.3551261	82.2690501	86.0107753	76.7357739	63	75.2107192
67.3551261	81.2690501	85.7885531	76.7357739	63	74.5544692
68.3551261	81.2690501	84.7885531	76.7357739	63	74.1638442
68.3551261	74.2690501	84.677442	76.7357739	63	72.8982192
68.3551261	72.2690501	84.2329976	76.7357739	63	72.7107192
68.3551261	71.2690501	83.677442	75.7357739	63	72.1482192
68.3551261	70.2690501	83.2329976	74.7357739	63	71.6013442
68.3551261	70.2690501	83.3441087	74.7357739	63	71.2419692
70.3551261	70.2690501	83.3441087	73.7357739	63	71.3825942
70.3551261	70.2690501	84.0107753	73.7357739	63	71.6013442

71.3551261	70.2690501	77.677442	73.7357739	63	71.4607192
70.3551261	70.2690501	73.2329976	73.7357739	63	70.8357192
70.3551261	70.2690501	83.8456011	73.7357739	63	72.4201505
70.3551261	74.2690501	83.8456011	73.7357739	63	73.2951505
70.3551261	76.2690501	79.8456011	72.7357739	63	73.1701505
71.3551261	76.2690501	76.8456011	72.7357739	63	72.9201505
72.3551261	75.2690501	73.8456011	73.7357739	63	72.1701505
72.3551261	73.2690501	71.8456011	73.7357739	63	71.6701505
76.3551261	70.2690501	68.8456011	73.7357739	63	71.2951505
76.3551261	69.2690501	67.8456011	74.7357739	63	71.0451505
77.3551261	68.2690501	67.8456011	74.7357739	63	70.9201505
76.3551261	68.2690501	67.8456011	74.7357739	63	71.0451505
76.3551261	69.2690501	67.8456011	75.7357739	63	71.5451505
75.3551261	69.2690501	67.8456011	74.7357739	63	71.7951505
75.3551261	69.2690501	67.8456011	74.7357739	63	71.7951505
75.3551261	69.2690501	67.8456011	72.7357739	63	71.7951505
75.3551261	68.2690501	67.8456011	71.7357739	63	71.5451505
75.3551261	67.2690501	67.8456011	69.7357739	63	71.4201505
75.3551261	67.2690501	67.8456011	68.7357739	63	71.4201505
76.3551261	67.2690501	66.8456011	67.7357739	63	71.2951505
76.3551261	67.2690501	65.8456011	67.7357739	63	71.0451505
76.3551261	67.2690501	64.8456011	67.7357739	63	70.9201505
76.3551261	68.2690501	64.8456011	67.7357739	63	71.1701505
76.3551261	68.2690501	64.8456011	68.7357739	63	71.0451505
78.3551261	69.2690501	64.8456011	68.7357739	63	71.1701505
78.3551261	71.2690501	64.8456011	68.7357739	63	71.0451505
76.3551261	72.2690501	64.8456011	68.7357739	63	70.5451505
76.3551261	73.2690501	66.8456011	68.7357739	63	70.7951505
74.3551261	74.2690501	66.8456011	69.7357739	63	70.1701505
73.3551261	73.2690501	66.8456011	69.7357739	63	70.1701505
72.3551261	72.2690501	64.8456011	70.7357739	63	69.4201505
72.3551261	71.2690501	63.8456011	71.7357739	63	67.8200713
72.3551261	71.2690501	63.8456011	73.7357739	63	67.9450713
73.3551261	71.2690501	63.8456011	78.7357739	63	69.9201505
73.3551261	70.2690501	62.8456011	78.7357739	63	70.0451505
73.3551261	70.2690501	62.8456011	79.7357739	63	70.1701505
74.3551261	71.2690501	62.8456011	78.7357739	63	70.5451505
74.3551261	72.2690501	62.8456011	78.7357739	63	70.9201505
74.3551261	72.2690501	62.8456011	77.7357739	63	71.5451505
74.3551261	71.2690501	62.8456011	77.7357739	63	71.2951505
74.3551261	71.2690501	62.8456011	77.7357739	63	71.6701505
74.3551261	71.2690501	63.8456011	77.7357739	63	71.7951505
74.3551261	71.2690501	64.8456011	76.7357739	63	72.0451505
74.3551261	67.2690501	64.8456011	76.7357739	63	71.5451505
74.3551261	65.2690501	63.8456011	76.7357739	63	71.1701505

76.3551261	64.2690501	64.8456011	76.7357739	63	71.1701505
76.3551261	65.2690501	64.8456011	75.7357739	63	71.1701505
77.3551261	66.2690501	64.8456011	75.7357739	63	71.4201505
79.3551261	67.2690501	64.8456011	75.7357739	63	71.6701505
79.3551261	67.2690501	64.8456011	74.7357739	63	71.7951505
79.3551261	68.2690501	66.8456011	73.7357739	63	72.2951505
79.3551261	68.2690501	66.8456011	69.7357739	63	72.2951505
79.3551261	68.2690501	66.8456011	66.7357739	63	72.0451505
81.3551261	70.2690501	66.8456011	65.7357739	63	72.6701505
83.3551261	69.2690501	65.8456011	60.7357739	63	72.5451505
83.3551261	69.2690501	65.8456011	60.7357739	63	72.5451505
83.3551261	69.2690501	65.8456011	59.7357739	63	72.5451505
84.3551261	66.2690501	65.8456011	58.7357739	63	72.5451505
83.3551261	65.2690501	65.8456011	58.7357739	63	72.1701505
83.3551261	63.2690501	66.8456011	61.7357739	63	72.0451505
80.3551261	64.2690501	67.8456011	61.7357739	63	71.9201505
77.3551261	64.2690501	67.8456011	69.7357739	63	72.0451505
73.3551261	64.2690501	67.8456011	70.7357739	63	72.0451505
72.3551261	64.2690501	67.8456011	71.7357739	63	72.1701505
71.3551261	64.2690501	67.8456011	72.7357739	63	72.5451505
71.3551261	64.2690501	68.8456011	72.7357739	63	72.6701505
70.3551261	61.2690501	68.8456011	72.7357739	63	72.4201505
69.3551261	61.2690501	68.8456011	72.7357739	63	72.7951505
69.3551261	61.2690501	68.8456011	72.7357739	63	72.7951505
69.3551261	61.2690501	68.8456011	72.7357739	63	73.2951505
68.3551261	61.2690501	68.8456011	71.7357739	63	73.4201505
68.3551261	61.2690501	68.8456011	72.7357739	63	73.6701505
68.3551261	62.2690501	68.8456011	72.7357739	63	74.1701505
68.3551261	64.2690501	68.8456011	72.7357739	63	74.2951505
68.3551261	68.2690501	75.8456011	72.7357739	63	75.2951505
68.3551261	69.2690501	83.8456011	73.7357739	63	76.7951505
68.3551261	68.2690501	87.8456011	73.7357739	63	77.0451505
68.3551261	68.2690501	77.2329976	76.7357739	63	74.7263442
67.3551261	68.2690501	77.677442	77.7357739	63	74.0232192
67.3551261	69.2690501	81.0107753	77.7357739	63	74.2419692
67.3551261	70.2690501	81.3441087	77.7357739	63	75.0388442
67.3551261	71.2690501	82.4552198	79.7357739	63	75.2888442
67.3551261	71.2690501	83.5663309	79.7357739	63	75.5700942
67.3551261	72.2690501	84.0107753	80.7357739	63	76.4919692
68.3551261	72.2690501	82.5663309	77.7357739	63	76.4607192
70.3551261	72.2690501	82.5663309	77.7357739	63	75.2107192
71.3551261	72.2690501	82.2329976	76.7357739	63	75.1794692
70.3551261	73.2690501	81.5663309	74.7357739	63	74.8669692
70.3551261	71.2690501	81.4552198	72.7357739	63	74.2575942
71.3551261	71.2690501	80.4552198	70.7357739	63	74.2732192

71.3551261	69.2690501	79.7885531	67.7357739	63	73.3513442
71.3551261	68.2690501	79.677442	66.7357739	63	72.9763442
71.3551261	68.2690501	79.5663309	66.7357739	63	72.9607192
71.3551261	68.2690501	79.8996642	66.7357739	63	72.7575942
71.3551261	68.2690501	79.8996642	68.7357739	63	73.1013442
71.3551261	68.2690501	80.2329976	68.7357739	63	72.8982192
72.3551261	68.2690501	81.2329976	73.7357739	63	73.3357192
72.3551261	68.2690501	81.2329976	72.7357739	63	73.8513442
72.3551261	68.2690501	80.8996642	70.7357739	63	73.9607192
71.3551261	68.2690501	80.5663309	69.7357739	63	73.8044692
70.3551261	68.2690501	81.0107753	69.7357739	63	73.6169692
70.3551261	69.2690501	81.3441087	67.7357739	63	74.0700942
70.3551261	69.2690501	80.677442	67.7357739	63	74.6013442
69.3551261	70.2690501	81.0107753	67.7357739	63	74.5232192
68.3551261	71.2690501	79.8996642	67.7357739	63	74.7419692
67.3551261	71.2690501	79.5663309	67.7357739	63	73.6950942
67.3551261	71.2690501	79.2329976	65.7357739	63	73.4294692
68.3551261	71.2690501	79.0107753	65.7357739	63	73.3982192
68.3551261	71.2690501	78.677442	65.7357739	63	73.1013442
68.3551261	71.2690501	78.3441087	65.7357739	63	72.6794692
68.3551261	71.2690501	78.2329976	65.7357739	63	72.2888442
68.3551261	71.2690501	78.0107753	65.7357739	63	72.1325942
68.3551261	72.2690501	78.1218865	64.7357739	63	71.9138442
68.3551261	72.2690501	78.2329976	64.7357739	63	72.1794692
69.3551261	72.2690501	79.677442	76.7357739	63	72.3200942
70.3551261	74.2690501	79.5663309	76.7357739	63	72.6794692
72.3551261	74.2690501	79.3441087	74.7357739	63	72.6794692
72.3551261	75.2690501	79.0107753	70.7357739	63	72.6950942
72.3551261	75.2690501	78.7885531	68.7357739	63	72.8200942
72.3551261	75.2690501	79.2329976	68.7357739	63	72.8825942
72.3551261	78.2690501	79.1218865	68.7357739	63	73.3669692
72.3551261	80.2690501	79.1218865	68.7357739	63	73.3669692
72.3551261	80.2690501	78.8996642	68.7357739	63	73.3357192
72.3551261	80.2690501	78.677442	68.7357739	63	73.0544692
72.3551261	81.2690501	79.0107753	68.7357739	63	72.9763442
72.3551261	82.2690501	79.1218865	68.7357739	63	73.2419692
72.3551261	82.2690501	80.1218865	70.7357739	63	73.4763442
72.3551261	83.2690501	77.2329976	70.7357739	63	73.8200942
72.3551261	83.2690501	90.8456011	70.7357739	63	76.2951505
71.3551261	83.2690501	89.8456011	66.7357739	63	76.5451505
71.3551261	83.2690501	89.8456011	64.7357739	63	76.7951505
73.3551261	83.2690501	88.8456011	64.7357739	63	77.1701505
73.3551261	82.2690501	88.8456011	64.7357739	63	76.9201505
74.3551261	80.2690501	88.8456011	64.7357739	63	76.6701505
76.3551261	79.2690501	88.8456011	65.7357739	63	76.7951505

75.3551261	78.2690501	88.8456011	65.7357739	63	76.5451505
75.3551261	78.2690501	85.8456011	64.7357739	63	75.7951505
75.3551261	75.2690501	85.8456011	64.7357739	63	75.0451505
75.3551261	74.2690501	74.8456011	63.7357739	63	73.4201505
75.3551261	73.2690501	73.8456011	63.7357739	63	72.7951505
75.3551261	74.2690501	73.8456011	63.7357739	63	72.6701505
75.3551261	75.2690501	73.8456011	63.7357739	63	72.5451505
75.3551261	75.2690501	73.8456011	63.7357739	63	72.5451505
75.3551261	74.2690501	74.8456011	63.7357739	63	72.6701505
75.3551261	73.2690501	74.8456011	63.7357739	63	72.5451505
75.3551261	72.2690501	74.8456011	63.7357739	63	72.5451505
75.3551261	73.2690501	74.8456011	64.7357739	63	72.5451505
75.3551261	73.2690501	74.8456011	65.7357739	63	72.9201505
74.3551261	72.2690501	74.8456011	65.7357739	63	73.2951505
74.3551261	72.2690501	74.8456011	66.7357739	63	73.1701505
74.3551261	71.2690501	74.8456011	73.7357739	63	73.2951505
74.3551261	70.2690501	75.8456011	73.7357739	63	73.4201505
74.3551261	68.2690501	75.8456011	75.7357739	63	71.3177299
74.3551261	68.2690501	76.8456011	75.7357739	63	71.3021049
74.3551261	67.2690501	76.8456011	77.7357739	63	71.3177299
74.3551261	68.2690501	76.8456011	77.7357739	63	71.8802299
73.3551261	68.2690501	76.8456011	77.7357739	63	72.0364799
73.3551261	68.2690501	76.8456011	77.7357739	63	72.1771049
73.3551261	68.2690501	77.8456011	73.7357739	63	72.0364799
73.3551261	70.2690501	77.8456011	74.7357739	63	72.3333549
73.3551261	71.2690501	77.8456011	74.7357739	63	72.6146049
74.3551261	71.2690501	78.8456011	75.7357739	63	72.8646049
74.3551261	71.2690501	78.8456011	75.7357739	63	75.1701505
74.3551261	71.2690501	78.8456011	76.7357739	63	75.5451505
74.3551261	70.2690501	79.8456011	76.7357739	63	75.7951505
74.3551261	70.2690501	79.8456011	77.7357739	63	75.7951505
74.3551261	69.2690501	79.8456011	78.7357739	63	75.5451505
74.3551261	69.2690501	79.8456011	79.7357739	63	75.7951505
73.3551261	69.2690501	80.8456011	79.7357739	63	75.7951505
73.3551261	67.2690501	80.8456011	78.7357739	63	75.9201505
73.3551261	65.2690501	80.8456011	78.7357739	63	76.0451505
72.3551261	64.2690501	80.8456011	78.7357739	63	75.6701505
72.3551261	64.2690501	80.8456011	77.7357739	63	75.9201505
70.3551261	64.2690501	82.8456011	77.7357739	63	76.0451505
69.3551261	64.2690501	82.8456011	77.7357739	63	75.5451505
68.3551261	64.2690501	82.8456011	77.7357739	63	74.6701505
68.3551261	64.2690501	83.8456011	78.7357739	63	74.7951505
68.3551261	67.2690501	84.8456011	79.7357739	63	74.0451505
68.3551261	68.2690501	84.8456011	79.7357739	63	73.0451505
68.3551261	68.2690501	82.8456011	79.7357739	63	72.5451505

67.3551261	68.2690501	76.8456011	79.7357739	63	71.5451505
67.3551261	69.2690501	76.8456011	79.7357739	63	71.4201505
66.3551261	69.2690501	75.8456011	81.7357739	63	70.6701505
66.3551261	69.2690501	75.8456011	82.7357739	63	70.6701505
67.3551261	69.2690501	75.8456011	83.7357739	63	71.1701505
67.3551261	70.2690501	75.8456011	83.7357739	63	71.7951505
67.3551261	70.2690501	76.8456011	83.7357739	63	71.7951505
67.3551261	71.2690501	76.8456011	83.7357739	63	71.6701505
67.3551261	72.2690501	76.8456011	83.7357739	63	71.6701505
67.3551261	72.2690501	76.8456011	89.7357739	63	71.6701505
69.3551261	72.2690501	76.8456011	90.7357739	63	72.2951505
69.3551261	71.2690501	76.8456011	91.7357739	63	72.4201505
69.3551261	71.2690501	76.8456011	90.7357739	63	72.6701505
69.3551261	71.2690501	77.8456011	90.7357739	63	73.0451505
69.3551261	70.2690501	73.8456011	90.7357739	63	72.7951505
69.3551261	73.2690501	72.8456011	90.7357739	63	73.0451505
69.3551261	73.2690501	72.8456011	89.7357739	63	73.1701505
71.3551261	72.2690501	72.8456011	88.7357739	63	74.1701505
71.3551261	68.2690501	72.8456011	87.7357739	63	74.0451505
72.3551261	66.2690501	72.8456011	87.7357739	63	73.7951505
72.3551261	65.2690501	72.8456011	84.7357739	63	73.5451505
71.3551261	65.2690501	72.8456011	76.7357739	63	73.1701505
71.3551261	60.2690501	72.8456011	75.7357739	63	72.1701505
71.3551261	58.2690501	72.8456011	75.7357739	63	71.4201505
71.3551261	58.2690501	72.8456011	75.7357739	63	71.0451505
72.3551261	59.2690501	72.8456011	75.7357739	63	70.7951505
73.3551261	59.2690501	72.8456011	75.7357739	63	70.7951505
73.3551261	62.2690501	76.8456011	81.7357739	63	71.7951505
73.3551261	65.2690501	78.8456011	81.7357739	63	72.5451505
75.3551261	65.2690501	78.8456011	81.7357739	63	73.4201505
75.3551261	66.2690501	78.8456011	81.7357739	63	73.5451505
76.3551261	65.2690501	78.8456011	79.7357739	63	73.7951505
76.3551261	65.2690501	78.8456011	66.7357739	63	74.1701505
77.3551261	65.2690501	78.8456011	60.7357739	63	74.5451505
77.3551261	65.2690501	78.8456011	58.7357739	63	74.7951505
77.3551261	65.2690501	76.8456011	58.7357739	63	74.2951505
77.3551261	65.2690501	74.8456011	58.7357739	63	73.7951505
77.3551261	69.2690501	73.8456011	61.7357739	63	74.0451505
78.3551261	71.2690501	72.8456011	64.7357739	63	74.5451505
78.3551261	71.2690501	72.8456011	67.7357739	63	74.6701505
79.3551261	69.2690501	72.8456011	67.7357739	63	75.0451505
79.3551261	66.2690501	72.8456011	69.7357739	63	74.9201505
79.3551261	66.2690501	72.8456011	68.7357739	63	73.4896049
79.3551261	66.2690501	72.8456011	68.7357739	63	73.5052299
79.3551261	68.2690501	72.8456011	68.7357739	63	74.4896049

79.3551261	68.2690501	71.8456011	71.7357739	63	74.6614799
79.3551261	69.2690501	71.8456011	72.7357739	63	74.6458549
77.3551261	70.2690501	71.8456011	74.7357739	63	74.5208549
77.3551261	70.2690501	72.8456011	76.7357739	63	74.9271049
78.3551261	70.2690501	72.8456011	78.7357739	63	75.3646049
78.3551261	70.2690501	72.8456011	80.7357739	63	75.3646049
78.3551261	70.2690501	72.8456011	80.7357739	63	75.5052299
78.3551261	71.2690501	72.8456011	80.7357739	63	76.4201505
78.3551261	71.2690501	72.8456011	76.7357739	63	75.0451505
78.3551261	71.2690501	72.8456011	76.7357739	63	75.4201505
78.3551261	71.2690501	72.8456011	72.7357739	63	75.7951505
79.3551261	72.2690501	72.8456011	72.7357739	63	76.0451505
78.3551261	72.2690501	72.8456011	73.7357739	63	75.1701505
78.3551261	73.2690501	72.8456011	74.7357739	63	73.0031334
78.3551261	74.2690501	73.8456011	74.7357739	63	72.9875084
78.3551261	74.2690501	73.8456011	74.7357739	63	72.8625084
78.3551261	74.2690501	73.8456011	74.7357739	63	73.0031334
65.3551261	74.2690501	74.8456011	74.7357739	63	71.1593834
65.3551261	74.2690501	74.8456011	74.7357739	63	70.9093834
62.3551261	67.2690501	75.8456011	74.7357739	63	69.3468834
62.3551261	67.2690501	78.8456011	74.7357739	63	69.5812584
62.3551261	67.2690501	80.8456011	74.7357739	63	70.3000084
62.3551261	66.2690501	81.8456011	75.7357739	63	70.5968834
65.3551261	66.2690501	81.8456011	76.7357739	63	72.6701505
68.3551261	66.2690501	81.8456011	76.7357739	63	72.5451505
68.3551261	67.2690501	83.8456011	77.7357739	63	72.7951505
68.3551261	68.2690501	84.8456011	78.7357739	63	73.6701505
68.3551261	66.2690501	83.8456011	79.7357739	63	73.4201505
68.3551261	66.2690501	85.8456011	78.7357739	63	73.5451505
69.3551261	66.2690501	85.8456011	78.7357739	63	73.5451505
69.3551261	66.2690501	85.8456011	77.7357739	63	73.7951505
70.3551261	64.2690501	87.8456011	76.7357739	63	72.7951505
71.3551261	64.2690501	87.8456011	76.7357739	63	72.2951505
72.3551261	64.2690501	87.8456011	75.7357739	63	72.0451505
72.3551261	64.2690501	87.8456011	75.7357739	63	69.7575942
73.3551261	64.2690501	86.8456011	75.7357739	63	71.4201505
74.3551261	64.2690501	85.8456011	80.7357739	63	70.6701505
74.3551261	64.2690501	85.8456011	83.7357739	63	71.1701505
75.3551261	63.2690501	83.8456011	83.7357739	63	70.9201505
74.3551261	63.2690501	82.8456011	84.7357739	63	70.6701505
74.3551261	64.2690501	82.8456011	84.7357739	63	70.0451505
74.3551261	65.2690501	83.8456011	84.7357739	63	69.9201505
74.3551261	65.2690501	83.8456011	83.7357739	63	69.4201505
74.3551261	67.2690501	84.8456011	83.7357739	63	69.9201505
73.3551261	68.2690501	84.8456011	77.7357739	63	70.0451505

73.3551261	68.2690501	85.8456011	77.7357739	63	71.2951505
73.3551261	68.2690501	87.8456011	72.7357739	63	72.4201505
73.3551261	69.2690501	88.8456011	71.7357739	63	72.6701505
76.3551261	73.2690501	88.8456011	73.7357739	63	73.7951505
76.3551261	73.2690501	89.8456011	74.7357739	63	74.0451505
77.3551261	70.2690501	73.7885531	74.7357739	63	71.7732192
76.3551261	68.2690501	73.8996642	78.7357739	63	71.3513442
75.3551261	68.2690501	74.0107753	79.7357739	63	70.6013442
75.3551261	68.2690501	74.3441087	80.7357739	63	70.6325942
75.3551261	69.2690501	76.7885531	80.7357739	63	71.2263442
75.3551261	71.2690501	77.0107753	80.7357739	63	73.2575942
75.3551261	72.2690501	77.4552198	80.7357739	63	73.4450942
75.3551261	72.2690501	76.8996642	81.7357739	63	73.9763442
75.3551261	72.2690501	77.1218865	83.7357739	63	73.2263442
75.3551261	72.2690501	77.3441087	83.7357739	63	73.2575942
75.3551261	74.2690501	76.7885531	83.7357739	63	73.4294692
76.3551261	74.2690501	75.8996642	83.7357739	63	72.6794692
76.3551261	72.2690501	75.8996642	83.7357739	63	71.6794692
76.3551261	72.2690501	76.1218865	83.7357739	63	71.7107192
76.3551261	72.2690501	76.2329976	83.7357739	63	71.7263442
76.3551261	72.2690501	76.1218865	83.7357739	63	71.8357192
75.3551261	72.2690501	76.4552198	81.7357739	63	71.6638442
75.3551261	76.2690501	74.677442	77.7357739	63	72.1013442
75.3551261	76.2690501	79.8456011	77.7357739	63	72.7951505
75.3551261	76.2690501	74.8456011	67.7357739	63	72.0451505
78.3551261	68.2690501	72.8456011	67.7357739	63	71.1701505
78.3551261	63.2690501	71.8456011	68.7357739	63	69.9201505
78.3551261	61.2690501	71.8456011	69.7357739	63	69.5451505
78.3551261	60.2690501	71.8456011	69.7357739	63	69.4201505
78.3551261	60.2690501	71.8456011	70.7357739	63	69.1701505
78.3551261	62.2690501	71.8456011	70.7357739	63	69.4201505
78.3551261	65.2690501	71.8456011	70.7357739	63	69.7951505
78.3551261	68.2690501	71.8456011	70.7357739	63	70.2951505
77.3551261	71.2690501	72.8456011	70.7357739	63	70.9201505
74.3551261	72.2690501	72.8456011	70.7357739	63	70.9201505
71.3551261	73.2690501	73.8456011	70.7357739	63	70.5451505
69.3551261	72.2690501	73.8456011	69.7357739	63	69.9201505
69.3551261	72.2690501	73.8456011	67.7357739	63	69.5451505
69.3551261	68.2690501	73.8456011	65.7357739	63	68.7951505
69.3551261	66.2690501	71.8456011	64.7357739	63	68.2951505
69.3551261	64.2690501	70.8456011	65.7357739	63	67.7951505
68.3551261	63.2690501	70.8456011	65.7357739	63	67.6701505
69.3551261	64.2690501	70.8456011	66.7357739	63	68.5451505
69.3551261	66.2690501	70.8456011	66.7357739	63	69.0451505
70.3551261	68.2690501	70.8456011	70.7357739	63	69.4201505

69.3551261	71.2690501	70.8456011	70.7357739	63	69.2951505
68.3551261	73.2690501	70.8456011	70.7357739	63	69.2951505
68.3551261	74.2690501	70.8456011	71.7357739	63	69.5451505
68.3551261	74.2690501	69.8456011	77.7357739	63	69.6701505
68.3551261	74.2690501	70.8456011	80.7357739	63	70.2951505
68.3551261	73.2690501	69.8456011	80.7357739	63	70.0451505
68.3551261	71.2690501	69.8456011	82.7357739	63	69.6701505
67.3551261	70.2690501	69.8456011	82.7357739	63	69.4201505
67.3551261	70.2690501	68.8456011	77.7357739	63	69.2951505
67.3551261	70.2690501	68.8456011	73.7357739	63	69.1701505
67.3551261	70.2690501	68.8456011	73.7357739	63	69.4201505
67.3551261	70.2690501	67.8456011	71.7357739	63	69.6701505
67.3551261	69.2690501	67.8456011	72.7357739	63	69.6701505
67.3551261	69.2690501	67.8456011	73.7357739	63	69.9201505
67.3551261	68.2690501	67.8456011	74.7357739	63	69.9201505
67.3551261	67.2690501	67.8456011	74.7357739	63	70.1701505
67.3551261	67.2690501	67.8456011	74.7357739	63	70.2951505
68.3551261	65.2690501	67.8456011	73.7357739	63	70.0451505
69.3551261	64.2690501	67.8456011	72.7357739	63	70.2951505
71.3551261	63.2690501	67.8456011	73.7357739	63	69.9201505
71.3551261	63.2690501	68.8456011	73.7357739	63	69.7951505
71.3551261	64.2690501	68.8456011	73.7357739	63	69.9201505
72.3551261	64.2690501	68.8456011	73.7357739	63	69.9201505
72.3551261	64.2690501	68.8456011	73.7357739	63	69.9201505
72.3551261	67.2690501	68.8456011	76.7357739	63	68.7951505
72.3551261	67.2690501	68.8456011	77.7357739	63	68.6701505
72.3551261	66.2690501	70.8456011	77.7357739	63	68.2951505
72.3551261	66.2690501	70.8456011	78.7357739	63	67.9201505
73.3551261	66.2690501	70.8456011	78.7357739	63	67.6701505
73.3551261	65.2690501	70.8456011	78.7357739	63	67.6701505
73.3551261	64.2690501	71.8456011	79.7357739	63	67.5451505
73.3551261	65.2690501	71.8456011	81.7357739	63	67.5451505
73.3551261	65.2690501	72.8456011	81.7357739	63	67.6701505
73.3551261	65.2690501	71.8456011	81.7357739	63	67.2951505
73.3551261	66.2690501	71.8456011	81.7357739	63	67.5451505
75.3551261	67.2690501	72.8456011	82.7357739	63	68.6701505
75.3551261	68.2690501	72.8456011	82.7357739	63	68.9201505
76.3551261	68.2690501	72.8456011	82.7357739	63	69.1701505
75.3551261	69.2690501	72.8456011	82.7357739	63	69.4201505
73.3551261	70.2690501	72.8456011	83.7357739	63	69.5451505
72.3551261	71.2690501	72.8456011	83.7357739	63	69.6701505
72.3551261	72.2690501	73.8456011	83.7357739	63	70.2951505
72.3551261	72.2690501	73.8456011	82.7357739	63	70.9201505
72.3551261	73.2690501	73.8456011	79.7357739	63	71.1701505
72.3551261	74.2690501	73.8456011	78.7357739	63	71.2951505

71.3551261	75.2690501	73.8456011	78.7357739	63	71.0451505	
70.3551261	74.2690501	73.8456011	76.7357739	63	70.6701505	
69.3551261	73.2690501	74.8456011	75.7357739	63	70.4201505	
69.3551261	73.2690501	76.8456011	73.7357739	63	69.6701505	
69.3551261	73.2690501	76.8456011	73.7357739	63	69.2951505	
69.3551261	74.2690501	76.8456011	73.7357739	63	69.1701505	
68.3551261	75.2690501	76.8456011	73.7357739	63	69.1701505	
68.3551261	75.2690501	76.8456011	72.7357739	63	69.2951505	Before
68.3551261	75.2690501	76.8456011	82.7357739	63	69.5451505	Before 1
68.3551261	75.2690501	76.8456011	81.7357739	63	70.2951505	Before 2
70.3551261	76.2690501	76.8456011	80.7357739	63	70.9201505	Before 3
71.3551261	73.2690501	76.8456011	80.7357739	63	71.7951505	Before 4
71.3551261	72.2690501	76.8456011	76.7357739	63	71.5451505	Before 5
71.3551261	72.2690501	76.8456011	74.7357739	63	69.6638213	Before 6
71.3551261	71.2690501	76.8456011	73.7357739	63	69.5544463	Before 7
71.3551261	72.2690501	77.8456011	73.7357739	63	69.8513213	Before 8
71.3551261	72.2690501	80.8456011	73.7357739	63	70.3981963	Before 9
71.3551261	71.2690501	80.8456011	73.7357739	63	70.5700713	Before 10
71.3551261	69.2690501	81.8456011	73.7357739	63	71.2575713	Before 11
71.3551261	67.2690501	82.8456011	72.7357739	63	71.3981963	Before 12
71.3551261	66.2690501	84.8456011	72.7357739	63	72.5451505	Before 13
71.3551261	65.2690501	86.8456011	74.7357739	63	71.9201505	Before 14
72.3551261	64.2690501	87.8456011	74.7357739	63	71.7951505	Before 15
72.3551261	64.2690501	87.8456011	74.7357739	63	71.7951505	Before 16
74.3551261	64.2690501	87.8456011	73.7357739	63	71.6701505	Before 17
75.3551261	65.2690501	86.8456011	73.7357739	63	71.7951505	Before 18
75.3551261	68.2690501	86.8456011	72.7357739	63	72.6701505	Before 19
76.3551261	69.2690501	86.8456011	72.7357739	63	72.7951505	Before 20
76.3551261	69.2690501	72.8996642	72.7357739	63	70.8044692	Before 21
76.3551261	68.2690501	72.7885531	72.7357739	63	70.7888442	Before 22
76.3551261	67.2690501	72.4552198	71.7357739	63	70.5075942	Before 23
76.3551261	66.2690501	72.4552198	71.7357739	63	70.1325942	Before 24
76.3551261	66.2690501	72.5663309	72.7357739	63	70.1325942	Before 25
74.3551261	66.2690501	72.7885531	76.7357739	63	70.1013442	Before 26
74.3551261	67.2690501	73.0107753	78.7357739	63	69.8513442	Before 27
73.3551261	69.2690501	72.7885531	79.7357739	63	69.8044692	Before 28
71.3551261	69.2690501	73.1218865	80.7357739	63	69.4607192	Before 29
70.3551261	72.2690501	73.1218865	80.7357739	63	69.7107192	Before 30
70.3551261	71.2690501	72.7885531	80.7357739	63	69.6638442	Before 31
69.3551261	69.2690501	72.7885531	80.7357739	63	69.2888442	Before 32
69.3551261	67.2690501	72.3441087	79.7357739	63	69.2419692	Before 33
68.3551261	65.2690501	72.3441087	79.7357739	63	68.8669692	Before 34
68.3551261	64.2690501	72.5663309	79.7357739	63	68.8982192	Before 35
68.3551261	65.2690501	72.4552198	79.7357739	63	69.1325942	Before 36
68.3551261	65.2690501	72.5663309	78.7357739	63	69.0388442	Before 37

68.3551261	66.2690501	72.7885531	78.7357739	63	69.0700942	Before 38
68.3551261	69.2690501	73.1218865	78.7357739	63	69.3669692	Before 39
67.3551261	70.2690501	72.677442	80.7357739	63	69.6482192	Before 40
67.3551261	69.2690501	72.677442	79.7357739	63	69.0388442	Before 41
67.3551261	67.2690501	72.7885531	78.7357739	63	69.1950942	Before 42
67.3551261	66.2690501	72.2329976	78.7357739	63	69.3669692	Before 43
67.3551261	64.2690501	71.8996642	77.7357739	63	68.7107192	Before 44
68.3551261	64.2690501	71.677442	76.7357739	63	68.3200942	Before 45
68.3551261	64.2690501	71.2329976	76.7357739	63	68.1325942	Before 46
68.3551261	63.2690501	71.3441087	75.7357739	63	67.6638442	Before 47
69.3551261	63.2690501	71.5663309	74.7357739	63	68.0857192	Before 48
69.3551261	64.2690501	72.1218865	75.7357739	63	68.5232192	Before 49
70.3551261	65.2690501	73.0107753	77.7357739	63	69.1169692	Before 50
71.3551261	65.2690501	73.4552198	77.7357739	63	69.8044692	Before 51
71.3551261	65.2690501	73.8996642	77.7357739	63	70.4919692	Before 52
72.3551261	65.2690501	83.8456011	78.7357739	63	72.2951505	Before 53
73.3551261	64.2690501	76.8456011	78.7357739	63	71.5451505	Before 54
73.3551261	65.2690501	71.8456011	78.7357739	63	70.7951505	Before 55
73.3551261	65.2690501	68.8456011	80.7357739	63	70.4201505	Before 56
73.3551261	66.2690501	68.8456011	79.7357739	63	70.6701505	Before 57
73.3551261	67.2690501	68.8456011	76.7357739	63	70.6701505	Before 58
73.3551261	68.2690501	68.8456011	77.7357739	63	70.4201505	Before 59
75.3551261	68.2690501	67.8456011	77.7357739	63	70.4201505	Before 60
75.3551261	69.2690501	67.8456011	77.7357739	63	70.6701505	Before 61
76.3551261	68.2690501	67.8456011	76.7357739	63	70.6701505	Before 62
74.3551261	67.2690501	67.8456011	76.7357739	63	70.2951505	Before 63
72.3551261	65.2690501	67.8456011	76.7357739	63	69.6701505	Before 64
70.3551261	64.2690501	67.8456011	76.7357739	63	69.5451505	Before 65
69.3551261	63.2690501	67.8456011	76.7357739	63	69.7951505	Before 66
68.3551261	62.2690501	67.8456011	76.7357739	63	69.7951505	Before 67
67.3551261	62.2690501	67.8456011	76.7357739	63	70.6701505	Before 68
67.3551261	63.2690501	63.8456011	76.7357739	63	70.4201505	Before 69
66.3551261	67.2690501	62.8456011	76.7357739	63	71.0451505	Before 70
66.3551261	69.2690501	61.8456011	76.7357739	63	71.2951505	Before 71
66.3551261	71.2690501	61.8456011	76.7357739	63	71.5451505	Before 72
66.3551261	74.2690501	65.8456011	76.7357739	63	72.2951505	Before 73
66.3551261	77.2690501	68.8456011	76.7357739	63	72.7951505	Before 74
68.3551261	78.2690501	68.8456011	71.7357739	63	72.5451505	Before 75
70.3551261	78.2690501	69.8456011	71.7357739	63	72.9201505	Before 76
69.3551261	78.2690501	69.8456011	70.7357739	63	72.7951505	Before 77
68.3551261	78.2690501	69.8456011	70.7357739	63	72.5451505	Before 78
68.3551261	78.2690501	69.8456011	70.7357739	63	72.5451505	Before 79
68.3551261	69.2690501	69.8456011	70.7357739	63	71.2951505	Before 80
68.3551261	67.2690501	69.8456011	70.7357739	69	70.7951505	Before 81
67.3551261	65.2690501	69.8456011	73.7357739	69	70.1701505	Before 82

67.3551261	64.2690501	69.8456011	73.7357739	69	70.1701505	Before 83
67.3551261	64.2690501	69.8456011	73.7357739	69	69.9201505	Before 84
67.3551261	64.2690501	72.8456011	72.7357739	69	70.0451505	Before 85
67.3551261	66.2690501	72.8456011	72.7357739	69	69.7951505	Before 86
68.3551261	66.2690501	72.8456011	73.7357739	69	69.9201505	Before 87
70.3551261	64.2690501	71.8456011	74.7357739	69	69.4201505	Before 88
70.3551261	63.2690501	71.8456011	75.7357739	69	69.9201505	Before 89
71.3551261	63.2690501	71.8456011	75.7357739	69	70.1701505	Before 90
71.3551261	62.2690501	71.8456011	77.7357739	69	70.2951505	Before 91
72.3551261	62.2690501	68.8456011	77.7357739	69	70.1701505	Before 92
72.3551261	61.2690501	67.8456011	77.7357739	69	70.0451505	Before 93
72.3551261	60.2690501	67.8456011	79.7357739	69	70.0451505	Before 94
72.3551261	60.2690501	67.8456011	79.7357739	69	70.6701505	Before 95
70.3551261	60.2690501	68.8456011	79.7357739	69	71.0451505	Before 96
70.3551261	61.2690501	69.8456011	79.7357739	69	71.2951505	Before 97
70.3551261	62.2690501	70.8456011	79.7357739	69	71.7951505	Before 98
70.3551261	65.2690501	76.8456011	78.7357739	69	72.0451505	Before 99
70.3551261	65.2690501	76.8456011	77.7357739	69	72.0451505	Before 100
71.3551261	64.2690501	77.8456011	77.7357739	69	71.9201505	Before 101
71.3551261	64.2690501	78.8456011	77.7357739	69	71.9201505	Before 102
71.3551261	64.2690501	79.8456011	77.7357739	69	72.4201505	Before 103
71.3551261	64.2690501	79.8456011	77.7357739	69	72.5451505	Before 104
71.3551261	64.2690501	88.8456011	78.7357739	69	73.7951505	Before 105
70.3551261	64.2690501	85.8456011	79.7357739	69	73.1701505	Before 106
70.3551261	64.2690501	83.8456011	80.7357739	69	72.9201505	Before 107
70.3551261	63.2690501	83.8456011	81.7357739	69	72.9201505	Before 108
69.3551261	63.2690501	83.8456011	82.7357739	69	72.9201505	Before 109
68.3551261	63.2690501	86.8456011	82.7357739	69	73.0451505	Before 110
68.3551261	63.2690501	86.8456011	82.7357739	69	72.6701505	Before 111
68.3551261	64.2690501	86.8456011	82.7357739	69	68.9607192	Before 112
67.3551261	64.2690501	72.5663309	83.7357739	69	68.2107192	Before 113
67.3551261	64.2690501	73.3441087	83.7357739	69	68.4450942	Before 114
67.3551261	63.2690501	73.0107753	83.7357739	69	68.2732192	Before 115
67.3551261	62.2690501	72.677442	82.7357739	69	67.8669692	Before 116
67.3551261	62.2690501	72.677442	82.7357739	69	67.4919692	Before 117
68.3551261	62.2690501	72.1218865	82.7357739	69	67.4138442	Before 118
68.3551261	61.2690501	71.3441087	81.7357739	69	66.5700942	Before 119
68.3551261	61.2690501	70.5663309	77.7357739	69	66.0232192	Before 120
68.3551261	61.2690501	70.2329976	73.7357739	69	65.6638442	Before 121
68.3551261	61.2690501	70.0107753	71.7357739	69	65.7888442	Before 122
68.3551261	61.2690501	69.8996642	70.7357739	69	65.7888442	Before 123
68.3551261	61.2690501	69.677442	70.7357739	69	65.7575942	Before 124
69.3551261	61.2690501	69.677442	70.7357739	69	65.5075942	Before 125
69.3551261	61.2690501	69.5663309	69.7357739	69	65.2575942	Before 126
69.3551261	61.2690501	70.0107753	70.7357739	69	65.3044692	Before 127

70.3551261	61.2690501	69.8996642	70.7357739	69	65.6638442	Before 128
70.3551261	61.2690501	70.1218865	71.7357739	69	65.5544692	Before 129
69.3551261	61.2690501	70.3441087	71.7357739	69	65.7107192	Before 130
69.3551261	61.2690501	70.3441087	72.7357739	69	65.9450942	Before 131
69.3551261	61.2690501	70.3441087	72.7357739	69	65.8200942	Before 132
69.3551261	61.2690501	71.2329976	72.7357739	69	65.9450942	Before 133
69.3551261	70.2690501	71.4552198	71.7357739	69	67.2419692	Before 134
69.3551261	75.2690501	71.4552198	70.7357739	69	67.6325942	Before 135
70.3551261	77.2690501	71.5663309	69.7357739	69	67.9138442	Before 136
71.3551261	79.2690501	71.2329976	62.7357739	69	68.3513442	Before 137
71.3551261	80.2690501	71.4552198	62.7357739	69	68.8825942	Before 138
71.3551261	80.2690501	71.3441087	61.7357739	69	69.1325942	Before 139
71.3551261	80.2690501	71.5663309	61.7357739	69	69.2888442	Before 140
72.3551261	80.2690501	71.5663309	61.7357739	69	69.5388442	Before 141
72.3551261	81.2690501	70.8996642	62.7357739	69	69.4294692	Before 142
72.3551261	80.2690501	70.3441087	62.7357739	69	68.9763442	Before 143
68.3551261	79.2690501	70.3441087	62.7357739	69	68.3513442	Before 144
66.3551261	78.2690501	70.8996642	65.7357739	69	68.3825942	Before 145
65.3551261	78.2690501	71.7885531	67.7357739	63	68.7263442	Before 146
65.3551261	79.2690501	72.677442	67.7357739	63	69.7263442	Before 147
65.3551261	80.2690501	73.2329976	67.7357739	63	70.8044692	Before 148
65.3551261	80.2690501	73.677442	66.7357739	63	71.3825942	Before 149
65.3551261	80.2690501	73.7885531	66.7357739	63	72.0232192	Before 150
65.3551261	80.2690501	74.1218865	66.7357739	63	72.0700942	Before 151
65.3551261	80.2690501	73.8996642	66.7357739	63	72.0388442	Before 152
65.3551261	81.2690501	74.0107753	66.7357739	63	71.8044692	Before 153
66.3551261	81.2690501	74.0107753	67.7357739	63	71.6638442	Before 154
66.3551261	81.2690501	74.1218865	67.7357739	63	71.5544692	Before 155
66.3551261	81.2690501	74.0107753	68.7357739	63	71.7732192	Before 156
66.3551261	81.2690501	74.5663309	71.7357739	63	71.5544692	Before 157
65.3551261	81.2690501	75.2329976	71.7357739	63	72.0232192	Before 158
65.3551261	81.2690501	75.1218865	69.7357739	63	73.1638442	Before 159
65.3551261	80.2690501	74.7885531	69.7357739	63	73.3669692	Before 160
65.3551261	76.2690501	75.0107753	69.7357739	63	73.0232192	Before 161
66.3551261	75.2690501	75.2329976	69.7357739	63	73.3044692	Before 162
66.3551261	76.2690501	75.8996642	68.7357739	63	75.9201505	Before 163
66.3551261	78.2690501	75.4552198	68.7357739	63	74.7263442	Before 164
66.3551261	79.2690501	75.3441087	68.7357739	63	76.1701505	Before 165
66.3551261	79.2690501	91.8456011	69.7357739	63	76.1701505	Before 166
66.3551261	79.2690501	90.8456011	69.7357739	63	76.2951505	Before 167
66.3551261	76.2690501	90.8456011	69.7357739	63	76.5451505	Before 168
66.3551261	75.2690501	90.8456011	69.7357739	63	76.7951505	Before 169
67.3551261	76.2690501	90.8456011	69.7357739	63	77.1701505	Before 170
67.3551261	76.2690501	88.8456011	69.7357739	63	76.9201505	Before 171
69.3551261	76.2690501	87.8456011	66.7357739	63	76.9201505	Before 172

69.3551261	76.2690501	87.8456011	66.7357739	63	77.1701505	Before 173
69.3551261	77.2690501	87.8456011	66.7357739	63	77.1701505	Before 174
69.3551261	76.2690501	89.8456011	64.7357739	63	77.0451505	Before 175
70.3551261	76.2690501	90.8456011	64.7357739	63	76.5451505	Before 176
70.3551261	77.2690501	74.8996642	66.7357739	63	74.3044692	Before 177
70.3551261	77.2690501	74.677442	66.7357739	63	73.7732192	Before 178
69.3551261	79.2690501	74.7885531	67.7357739	63	73.5232192	Before 179
69.3551261	79.2690501	74.3441087	69.7357739	63	73.4294692	Before 180
67.3551261	79.2690501	74.1218865	69.7357739	63	72.6482192	Before 181
67.3551261	78.2690501	74.0107753	69.7357739	63	72.3825942	Before 182
67.3551261	77.2690501	73.7885531	69.7357739	63	72.2263442	Before 183
67.3551261	77.2690501	74.0107753	74.7357739	63	71.9294692	Before 184
67.3551261	77.2690501	73.677442	74.7357739	63	71.5075942	Before 185
67.3551261	77.2690501	73.7885531	75.7357739	63	71.1325942	Before 186
67.3551261	77.2690501	72.8996642	74.7357739	63	71.0232192	Before 187
68.3551261	77.2690501	72.5663309	74.7357739	63	69.9763442	Before 188
68.3551261	76.2690501	72.3441087	74.7357739	63	69.4450942	Before 189
68.3551261	76.2690501	72.2329976	74.7357739	63	69.1794692	Before 190
68.3551261	76.2690501	71.8996642	74.7357739	63	69.0075942	Before 191
68.3551261	76.2690501	71.8996642	73.7357739	63	68.7732192	Before 192
68.3551261	78.2690501	72.0107753	73.7357739	63	68.9138442	Before 193
69.3551261	78.2690501	71.5663309	73.7357739	63	68.9763442	Before 194
69.3551261	77.2690501	71.8996642	74.7357739	63	68.5075942	Before 195
70.3551261	76.2690501	72.1218865	73.7357739	63	68.8044692	Before 196
70.3551261	76.2690501	72.677442	71.7357739	63	69.4138442	Before 197
72.3551261	76.2690501	73.2329976	71.7357739	63	70.3669692	Before 198
73.3551261	75.2690501	73.3441087	70.7357739	63	71.0232192	Before 199
74.3551261	75.2690501	73.8996642	70.7357739	63	71.3513442	Before 200
74.3551261	75.2690501	73.677442	70.7357739	63	71.8200942	Before 201
74.3551261	74.2690501	73.7885531	70.7357739	63	71.5857192	Before 202
73.3551261	74.2690501	73.2329976	67.7357739	63	71.6794692	Before 203
73.3551261	73.2690501	72.677442	66.7357739	63	71.2419692	Before 204
73.3551261	73.2690501	72.8996642	67.7357739	63	70.7575942	Before 205
72.3551261	72.2690501	73.4552198	67.7357739	63	70.8357192	Before 206
72.3551261	72.2690501	73.7885531	66.7357739	63	71.2732192	Before 207
72.3551261	72.2690501	74.1218865	66.7357739	63	71.8200942	Before 208
72.3551261	71.2690501	73.8996642	66.7357739	63	72.2888442	Before 209
71.3551261	71.2690501	73.5663309	67.7357739	63	72.1013442	Before 210
69.3551261	69.2690501	73.677442	70.7357739	63	71.5700942	Before 211
69.3551261	69.2690501	80.8456011	79.7357739	63	72.2951505	Before 212
69.3551261	68.2690501	80.8456011	82.7357739	63	72.1701505	Before 213
69.3551261	68.2690501	79.8456011	82.7357739	63	71.7951505	Before 214
69.3551261	68.2690501	79.8456011	82.7357739	63	71.6701505	Before 215
68.3551261	68.2690501	78.8456011	82.7357739	63	71.1701505	Before 216
69.3551261	68.2690501	77.8456011	82.7357739	63	71.1701505	Before 217

69.3551261	67.2690501	76.8456011	82.7357739	63	71.0451505	Before 218
71.3551261	67.2690501	76.8456011	82.7357739	63	71.4201505	Before 219
72.3551261	68.2690501	76.8456011	81.7357739	63	71.4201505	Before 220
72.3551261	69.2690501	76.8456011	81.7357739	63	70.7951505	Before 221
72.3551261	68.2690501	76.8456011	81.7357739	63	70.4201505	Before 222
72.3551261	64.2690501	78.8456011	81.7357739	63	70.0451505	Before 223
72.3551261	62.2690501	78.8456011	81.7357739	63	69.4201505	Before 224
72.3551261	61.2690501	78.8456011	81.7357739	63	69.4201505	Before 225
72.3551261	63.2690501	79.8456011	80.7357739	63	69.4201505	Before 226
71.3551261	65.2690501	79.8456011	80.7357739	63	69.2951505	Before 227
70.3551261	69.2690501	79.8456011	80.7357739	63	69.5451505	Before 228
70.3551261	71.2690501	79.8456011	80.7357739	63	69.4201505	Before 229
68.3551261	71.2690501	79.8456011	80.7357739	63	68.5451505	Before 230
68.3551261	67.2690501	79.8456011	80.7357739	63	67.7951505	Before 231
68.3551261	65.2690501	79.8456011	80.7357739	63	67.4201505	Before 232
69.3551261	64.2690501	80.8456011	79.7357739	63	67.5451505	Before 233
69.3551261	63.2690501	80.8456011	78.7357739	63	67.5451505	Before 234
70.3551261	63.2690501	72.8456011	78.7357739	63	66.6701505	Before 235
70.3551261	64.2690501	70.8456011	77.7357739	63	66.6701505	Before 236
70.3551261	65.2690501	69.8456011	77.7357739	63	66.9201505	Before 237
71.3551261	66.2690501	68.8456011	78.7357739	63	67.0451505	Before 238
71.3551261	66.2690501	68.8456011	78.7357739	63	67.5451505	Before 239
71.3551261	66.2690501	68.8456011	77.7357739	63	67.2951505	Before 240
71.3551261	66.2690501	68.8456011	77.7357739	63	67.2951505	minute 1
71.3551261	66.2690501	68.8456011	79.7357739	63	67.5451505	minute 2
70.3551261	66.2690501	68.8456011	79.7357739	63	66.9201505	minute 3
70.3551261	66.2690501	68.8456011	79.7357739	63	66.9201505	minute 4
70.3551261	64.2690501	65.8456011	78.7357739	63	66.4201505	minute 5
71.3551261	64.2690501	66.8456011	78.7357739	63	66.7951505	minute 6
71.3551261	65.2690501	65.8456011	78.7357739	63	66.7951505	minute 7
71.3551261	66.2690501	65.8456011	77.7357739	63	66.9201505	minute 8
73.3551261	66.2690501	64.8456011	77.7357739	63	67.0451505	minute 9
73.3551261	68.2690501	64.8456011	77.7357739	63	67.0451505	minute 10
74.3551261	69.2690501	64.8456011	75.7357739	63	67.9201505	minute 11
75.3551261	69.2690501	65.8456011	75.7357739	63	68.4201505	minute 12
75.3551261	69.2690501	65.8456011	75.7357739	63	68.2951505	minute 13
75.3551261	68.2690501	65.8456011	68.7357739	63	67.9201505	minute 14
76.3551261	68.2690501	65.8456011	68.7357739	63	68.1701505	minute 15
76.3551261	68.2690501	65.8456011	67.7357739	63	68.9201505	minute 16
79.3551261	69.2690501	65.8456011	67.7357739	63	70.2951505	minute 17
79.3551261	70.2690501	65.8456011	67.7357739	63	70.7951505	minute 18
80.3551261	70.2690501	65.8456011	65.7357739	63	71.0451505	minute 19
87.3551261	70.2690501	65.8456011	66.7357739	63	72.0451505	minute 20
72.9201505	70.2690501	65.8456011	67.7357739	63	70.6157786	minute 21
72.9201505	69.2690501	65.8456011	68.7357739	63	69.9907786	minute 22

72.6701505	68.2690501	65.8456011	68.7357739	63	69.5845286	minute 23
72.2951505	68.2690501	66.8456011	70.7357739	63	69.5376536	minute 24
72.5451505	68.2690501	67.8456011	72.7357739	63	69.8189036	minute 25
83.3551261	68.2690501	67.8456011	74.7357739	63	71.5451505	minute 26
75.3551261	68.2690501	69.8456011	75.7357739	63	70.6701505	minute 27
75.3551261	68.2690501	69.8456011	77.7357739	63	70.7951505	minute 28
75.3551261	68.2690501	69.8456011	78.7357739	63	70.7951505	minute 29
69.3551261	68.2690501	69.8456011	80.7357739	63	70.1701505	minute 30
69.3551261	70.2690501	69.8456011	80.7357739	63	70.5451505	minute 31
69.3551261	70.2690501	69.8456011	80.7357739	63	70.9201505	minute 32
68.3551261	70.2690501	69.8456011	80.7357739	63	71.0451505	minute 33
68.3551261	70.2690501	69.8456011	77.7357739	63	71.0451505	minute 34
68.3551261	70.2690501	69.8456011	75.7357739	63	70.7951505	minute 35
68.3551261	69.2690501	71.8456011	74.7357739	63	70.7951505	minute 36
68.3551261	70.2690501	71.8456011	73.7357739	63	70.1701505	minute 37
68.3551261	70.2690501	72.8456011	73.7357739	63	70.1701505	minute 38
69.3551261	71.2690501	72.8456011	73.7357739	63	70.2951505	minute 39
68.3551261	71.2690501	74.8456011	71.7357739	63	71.0451505	minute 40
68.3551261	72.2690501	74.8456011	70.7357739	63	71.4201505	minute 41
68.3551261	71.2690501	74.8456011	69.7357739	63	71.2951505	minute 42
68.3551261	71.2690501	75.8456011	69.7357739	63	71.4201505	minute 43
68.3551261	70.2690501	75.8456011	67.7357739	63	70.9201505	minute 44
68.3551261	69.2690501	72.8456011	67.7357739	63	69.7951505	minute 45
69.3551261	69.2690501	71.8456011	67.7357739	63	70.5451505	minute 46
69.3551261	71.2690501	70.8456011	67.7357739	63	68.2610378	minute 47
69.3551261	72.2690501	67.8456011	66.7357739	63	67.7922878	minute 48
69.3551261	72.2690501	67.8456011	67.7357739	63	67.6204128	minute 49
69.3551261	71.2690501	66.8456011	67.7357739	63	67.0891628	minute 50
69.3551261	71.2690501	67.8456011	67.7357739	63	67.0735378	minute 51
70.3551261	71.2690501	67.8456011	69.7357739	63	67.2454128	minute 52
71.3551261	69.2690501	67.8456011	72.7357739	63	67.3860378	minute 53
71.3551261	68.2690501	67.8456011	72.7357739	63	67.3704128	minute 54
71.3551261	68.2690501	67.8456011	71.7357739	63	67.3704128	minute 55
71.3551261	67.2690501	67.8456011	70.7357739	63	70.1701505	minute 56
71.3551261	67.2690501	67.8456011	70.7357739	63	70.4201505	minute 57
71.3551261	67.2690501	67.8456011	71.7357739	63	70.2951505	minute 58
71.3551261	67.2690501	67.8456011	73.7357739	63	70.6701505	minute 59
71.3551261	68.2690501	67.8456011	73.7357739	63	70.4201505	minute 60
69.3551261	68.2690501	67.8456011	73.7357739	73	70.2951505	Activity
68.3551261	68.2690501	68.8456011	76.7357739	73	70.0451505	Activity
68.3551261	68.2690501	73.8456011	76.7357739	73	69.9201505	Activity
68.3551261	68.2690501	73.8456011	76.7357739	73	69.7951505	Activity
67.3551261	69.2690501	74.8456011	77.7357739	73	69.9201505	Activity
67.3551261	70.2690501	75.8456011	77.7357739	73	70.1701505	Activity
67.3551261	70.2690501	75.8456011	79.7357739	73	70.5451505	Activity

67.3551261	70.2690501	75.8456011	78.7357739	73	70.5451505	Activity
67.3551261	70.2690501	74.8456011	77.7357739	73	71.9201505	Activity
66.3551261	70.2690501	73.8456011	76.7357739	73	71.4201505	Activity
66.3551261	68.2690501	72.8456011	74.7357739	73	69.0677299	Activity
66.3551261	68.2690501	72.8456011	74.7357739	73	69.2239799	Activity
66.3551261	68.2690501	72.8456011	72.7357739	73	69.8021049	Activity
66.3551261	68.2690501	72.8456011	72.7357739	73	69.7083549	Activity
65.3551261	68.2690501	73.8456011	72.7357739	73	69.7083549	Activity
64.3551261	67.2690501	73.8456011	72.7357739	73	69.3802299	Activity
63.3551261	65.2690501	73.8456011	72.7357739	73	71.2951505	Activity
63.3551261	67.2690501	74.8456011	72.7357739	73	70.9201505	Activity
63.3551261	67.2690501	74.8456011	72.7357739	73	71.2951505	Activity
63.3551261	67.2690501	74.8456011	73.7357739	73	71.6701505	Activity
63.3551261	67.2690501	75.8456011	78.7357739	73	71.5451505	Activity
63.3551261	66.2690501	75.8456011	85.7357739	73	71.2951505	Activity
65.3551261	66.2690501	75.8456011	87.7357739	73	71.2951505	Activity
66.3551261	66.2690501	76.8456011	86.7357739	73	71.4201505	Activity
66.3551261	66.2690501	76.8456011	83.7357739	73	71.4201505	Activity
64.3551261	67.2690501	77.8456011	81.7357739	73	72.0451505	Activity
63.3551261	67.2690501	77.8456011	81.7357739	73	72.6701505	Activity
64.3551261	67.2690501	77.8456011	77.7357739	73	73.0451505	Activity
65.3551261	67.2690501	76.8456011	77.7357739	73	73.2951505	Activity
69.3551261	66.2690501	76.8456011	76.7357739	73	73.5451505	Activity
70.3551261	67.2690501	76.8456011	76.7357739	73	73.7951505	Activity
70.3551261	65.2690501	76.8456011	76.7357739	73	73.2951505	Activity
73.3551261	65.2690501	76.8456011	75.7357739	73	72.7951505	Activity
74.3551261	64.2690501	76.8456011	75.7357739	73	72.7951505	Activity
75.3551261	64.2690501	76.8456011	75.7357739	73	72.7951505	Activity
76.3551261	64.2690501	76.8456011	74.7357739	73	72.1701505	Activity
76.3551261	63.2690501	77.8456011	74.7357739	73	71.9201505	Activity
79.3551261	63.2690501	78.8456011	74.7357739	73	72.5451505	Activity
80.3551261	63.2690501	79.8456011	74.7357739	73	72.1701505	Activity
81.3551261	63.2690501	79.8456011	74.7357739	73	72.1701505	Activity
81.3551261	64.2690501	80.8456011	75.7357739	73	72.6701505	Activity
81.3551261	63.2690501	80.8456011	75.7357739	73	72.4201505	Activity
82.3551261	63.2690501	79.8456011	76.7357739	73	72.9201505	Activity
81.3551261	63.2690501	73.8456011	78.7357739	73	72.6701505	Activity
81.3551261	63.2690501	69.8456011	79.7357739	73	72.4201505	Activity
80.3551261	63.2690501	69.8456011	80.7357739	73	73.6701505	Activity
79.3551261	63.2690501	69.8456011	82.7357739	73	72.0521049	Activity
79.3551261	65.2690501	69.8456011	83.7357739	73	72.7239799	Activity
79.3551261	65.2690501	69.8456011	83.7357739	73	72.7239799	Activity
79.3551261	66.2690501	69.8456011	84.7357739	73	72.7552299	Activity
78.3551261	66.2690501	69.8456011	84.7357739	73	72.4896049	Activity
79.3551261	66.2690501	69.8456011	85.7357739	73	72.6302299	Activity

79.3551261	66.2690501	69.8456011	86.7357739	73	72.7708549	Activity
79.3551261	68.2690501	69.8456011	86.7357739	73	73.0521049	Activity
80.3551261	68.2690501	69.8456011	87.7357739	73	73.6146049	Activity
80.3551261	68.2690501	67.8456011	87.7357739	73	74.0677299	Activity
80.3551261	68.2690501	66.8456011	87.7357739	73	73.3646049	Activity
80.3551261	68.2690501	66.8456011	87.7357739	73	73.2083549	Activity
79.3551261	68.2690501	66.8456011	87.7357739	73	73.2239799	Activity
79.3551261	68.2690501	66.8456011	87.7357739	73	73.5052299	Activity
79.3551261	70.2690501	66.8456011	87.7357739	73	74.4896049	Activity
79.3551261	71.2690501	67.8456011	88.7357739	73	75.5989799	Activity
79.3551261	73.2690501	68.8456011	88.7357739	73	76.8802299	Activity
79.3551261	73.2690501	68.8456011	89.7357739	73	78.8646049	Activity
79.3551261	74.2690501	68.8456011	88.7357739	73	77.4711172	Activity
80.3551261	74.2690501	68.8456011	86.7357739	73	77.4086172	Activity
80.3551261	73.2690501	68.8456011	84.7357739	73	77.4398672	Activity
80.3551261	72.2690501	68.8456011	84.7357739	73	78.0648672	Activity
80.3551261	71.2690501	68.8456011	83.7357739	73	79.0208549	Activity
80.3551261	71.2690501	68.8456011	83.7357739	73	78.5364799	Activity
80.3551261	71.2690501	69.8456011	83.7357739	73	77.4427299	Activity
84.3551261	71.2690501	69.8456011	83.7357739	73	76.1927299	Activity
84.3551261	70.2690501	69.8456011	83.7357739	73	75.2083549	Activity
84.3551261	69.2690501	69.8456011	83.7357739	73	74.5052299	Activity
84.3551261	68.2690501	68.8456011	83.7357739	73	74.5052299	Activity
85.3551261	68.2690501	69.8456011	82.7357739	73	74.7552299	Activity
86.3551261	68.2690501	69.8456011	82.7357739	73	74.7708549	Activity
87.3551261	68.2690501	69.8456011	82.7357739	73	75.1927299	Activity
88.3551261	68.2690501	69.8456011	79.7357739	73	74.9896049	Activity
89.3551261	69.2690501	69.8456011	77.7357739	73	74.7083549	Activity
90.3551261	69.2690501	69.8456011	77.7357739	73	74.9896049	Activity
94.3551261	68.2690501	69.8456011	77.7357739	73	75.3958549	Activity
96.3551261	69.2690501	68.8456011	77.7357739	73	75.6927299	Activity
80.0451505	68.2690501	68.8456011	77.7357739	73	73.357108	Activity
80.2951505	68.2690501	68.8456011	77.7357739	73	73.544608	Activity
80.6701505	68.2690501	71.8456011	77.7357739	73	74.513358	Activity
96.3551261	68.2690501	71.8456011	78.7357739	73	76.7708549	Activity
96.3551261	67.2690501	71.8456011	79.7357739	73	76.6146049	Activity
96.3551261	72.2690501	71.8456011	79.7357739	73	77.3177299	Activity
96.3551261	73.2690501	71.8456011	80.7357739	73	77.5677299	Activity
82.3551261	73.2690501	71.8456011	83.7357739	73	75.5989799	Activity
79.3551261	72.2690501	71.8456011	83.7357739	73	75.0364799	Activity
78.3551261	71.2690501	71.8456011	83.7357739	73	74.6146049	Activity
78.3551261	70.2690501	73.8456011	84.7357739	73	74.7239799	Activity
76.3551261	67.2690501	73.8456011	84.7357739	73	74.0364799	Activity
77.3551261	65.2690501	71.8456011	83.7357739	73	73.7864799	Activity
77.3551261	64.2690501	71.8456011	83.7357739	73	73.4583549	Activity

76.3551261	64.2690501	71.8456011	82.7357739	73	73.0208549	Activity
76.3551261	65.2690501	71.8456011	82.7357739	73	72.8802299	Activity
76.3551261	67.2690501	71.8456011	82.7357739	73	72.7396049	Activity
76.3551261	70.2690501	70.8456011	82.7357739	73	72.9271049	Activity
76.3551261	70.2690501	70.8456011	82.7357739	73	73.0833549	Activity
76.3551261	70.2690501	71.8456011	82.7357739	73	73.2083549	Activity
76.3551261	73.2690501	72.8456011	81.7357739	73	74.2239799	Activity
77.3551261	73.2690501	72.8456011	80.7357739	73	74.0989799	Activity
76.3551261	73.2690501	72.8456011	77.7357739	73	73.9583549	Activity
76.3551261	72.2690501	72.8456011	76.7357739	73	73.8021049	Activity
76.3551261	70.2690501	73.8456011	76.7357739	73	74.0677299	Activity
76.3551261	70.2690501	73.8456011	76.7357739	73	74.3646049	Activity
76.3551261	70.2690501	73.8456011	76.7357739	73	74.3646049	Activity
74.3551261	70.2690501	73.8456011	75.7357739	73	73.9739799	Activity
72.3551261	72.2690501	74.8456011	75.7357739	73	73.8177299	Activity
72.3551261	74.2690501	75.8456011	75.7357739	73	73.9583549	Activity
72.3551261	73.2690501	75.8456011	75.7357739	73	73.9739799	Activity
72.3551261	72.2690501	75.8456011	77.7357739	73	73.9583549	Activity
71.3551261	71.2690501	75.8456011	77.7357739	73	73.6771049	Activity
71.3551261	70.2690501	75.8456011	77.7357739	73	73.8177299	Activity
71.3551261	70.2690501	75.8456011	77.7357739	73	73.8177299	Activity
71.3551261	71.2690501	75.8456011	76.7357739	73	74.1927299	Activity
71.3551261	72.2690501	75.8456011	72.7357739	73	74.3021049	Activity
71.3551261	73.2690501	76.8456011	71.7357739	73	74.5521049	Activity
71.3551261	74.2690501	78.8456011	71.7357739	73	74.5364799	Activity
71.3551261	73.2690501	80.8456011	71.7357739	73	74.8021049	Activity
71.3551261	73.2690501	78.8456011	72.7357739	73	74.4427299	Activity
71.3551261	73.2690501	78.8456011	72.7357739	73	74.4114799	Activity
71.3551261	68.2690501	76.8456011	73.7357739	73	73.4583549	Activity
71.3551261	67.2690501	76.8456011	72.7357739	73	73.2864799	Activity
71.3551261	67.2690501	76.8456011	73.7357739	73	73.8333549	Activity
71.3551261	66.2690501	76.8456011	74.7357739	73	74.1146049	Activity
71.3551261	66.2690501	76.8456011	74.7357739	73	73.6927299	Activity
73.3551261	65.2690501	76.8456011	74.7357739	73	73.8333549	Activity
76.3551261	65.2690501	75.8456011	74.7357739	73	74.3958549	Activity
76.3551261	66.2690501	72.8456011	74.7357739	73	74.1458549	Activity
77.3551261	66.2690501	72.8456011	74.7357739	73	74.3958549	Activity
76.3551261	63.2690501	72.8456011	74.7357739	73	73.8489799	Activity
73.3551261	62.2690501	72.8456011	75.7357739	73	73.4271049	Activity
72.3551261	62.2690501	72.8456011	78.7357739	73	73.4427299	Activity
70.3551261	61.2690501	72.8456011	78.7357739	73	72.8802299	Activity
69.3551261	61.2690501	70.8456011	79.7357739	73	72.4896049	Activity
69.3551261	63.2690501	70.8456011	82.7357739	73	72.6458549	Activity
69.3551261	63.2690501	71.8456011	84.7357739	73	72.5208549	Activity
70.3551261	63.2690501	71.8456011	84.7357739	73	73.0052299	Activity

72.3551261	63.2690501	71.8456011	84.7357739	73	73.1614799	Activity
72.3551261	63.2690501	67.8456011	84.7357739	73	72.8177299	Activity
73.3551261	63.2690501	64.8456011	83.7357739	73	72.3958549	Activity
73.3551261	61.2690501	64.8456011	83.7357739	73	72.2083549	Activity
73.3551261	61.2690501	64.8456011	83.7357739	73	72.3802299	Activity
73.3551261	59.2690501	64.8456011	83.7357739	73	72.0989799	Activity
73.3551261	61.2690501	65.8456011	82.7357739	73	72.5052299	Activity
73.3551261	63.2690501	65.8456011	82.7357739	73	72.3958549	Activity
75.3551261	65.2690501	66.8456011	84.7357739	73	72.2239799	Activity
76.3551261	66.2690501	70.8456011	84.7357739	73	73.2864799	Activity
75.3551261	66.2690501	71.8456011	84.7357739	73	74.1771049	Activity
75.3551261	65.2690501	73.8456011	80.7357739	73	74.4427299	Activity
74.3551261	65.2690501	74.8456011	79.7357739	73	74.4583549	Activity
74.3551261	66.2690501	74.8456011	80.7357739	73	74.7552299	Activity
74.3551261	66.2690501	74.8456011	80.7357739	73	74.7552299	Activity
74.3551261	67.2690501	74.8456011	80.7357739	73	74.8958549	Activity
77.3551261	68.2690501	74.8456011	79.7357739	73	75.4583549	Activity
77.3551261	69.2690501	73.8456011	76.7357739	73	75.7552299	Activity
77.3551261	70.2690501	70.8456011	72.7357739	73	75.6458549	Activity
77.3551261	71.2690501	66.8456011	71.7357739	73	75.3802299	Activity
77.3551261	72.2690501	66.8456011	71.7357739	73	75.4583549	Activity
77.3551261	73.2690501	65.8456011	71.7357739	73	75.2083549	Activity
77.3551261	73.2690501	65.8456011	69.7357739	73	74.3489799	Activity
77.3551261	74.2690501	64.8456011	69.7357739	73	73.6614799	Activity
77.3551261	74.2690501	64.8456011	71.7357739	73	73.2239799	Activity
76.3551261	74.2690501	63.8456011	71.7357739	73	72.8177299	Activity
76.3551261	75.2690501	63.8456011	72.7357739	73	73.2239799	Activity
76.3551261	75.2690501	63.8456011	72.7357739	73	73.2239799	Activity
74.3551261	74.2690501	63.8456011	74.7357739	73	72.9427299	Activity
73.3551261	72.2690501	63.8456011	73.7357739	73	71.8021049	Activity
73.3551261	72.2690501	64.8456011	73.7357739	73	71.1771049	Activity
73.3551261	69.2690501	64.8456011	73.7357739	73	70.4271049	Activity
74.3551261	69.2690501	64.8456011	71.7357739	73	69.8177299	Activity
74.3551261	69.2690501	65.8456011	70.7357739	73	70.0677299	Activity
74.3551261	69.2690501	65.8456011	69.7357739	73	70.4896049	Activity
73.3551261	69.2690501	65.8456011	69.7357739	73	70.3489799	Activity
73.3551261	69.2690501	65.8456011	69.7357739	73	70.0677299	Activity
72.3551261	70.2690501	65.8456011	69.7357739	73	69.3802299	Activity
72.3551261	70.2690501	65.8456011	69.7357739	73	69.5208549	Activity
72.3551261	70.2690501	66.8456011	69.7357739	73	69.3646049	Activity
73.3551261	70.2690501	67.8456011	71.7357739	73	69.6458549	Activity
74.3551261	72.2690501	67.8456011	71.7357739	73	69.3802299	Activity
75.3551261	73.2690501	69.8456011	70.7357739	73	69.6146049	Activity
75.3551261	74.2690501	69.8456011	70.7357739	73	69.5052299	Activity
75.3551261	74.2690501	69.8456011	70.7357739	73	69.6458549	Activity

75.3551261	74.2690501	69.8456011	67.7357739	73	69.7864799	Activity
74.3551261	74.2690501	71.8456011	67.7357739	73	69.7552299	Activity
74.3551261	72.2690501	73.8456011	67.7357739	73	69.5208549	Activity
72.3551261	71.2690501	73.8456011	67.7357739	73	69.0052299	Activity
72.3551261	62.2690501	73.8456011	67.7357739	73	67.5989799	Activity
71.3551261	63.2690501	74.8456011	66.7357739	73	67.3021049	Activity
70.3551261	64.2690501	75.8456011	65.7357739	73	67.6146049	Activity
70.3551261	65.2690501	75.8456011	65.7357739	73	67.7708549	Activity
70.3551261	66.2690501	76.8456011	65.7357739	73	67.8333549	Activity
70.3551261	67.2690501	76.8456011	65.7357739	73	68.2396049	Activity
71.3551261	67.2690501	76.8456011	65.7357739	73	68.6614799	Activity
71.3551261	67.2690501	76.8456011	65.7357739	73	68.6458549	Activity
71.3551261	66.2690501	76.8456011	69.7357739	73	68.5052299	Activity
72.3551261	66.2690501	75.8456011	71.7357739	73	68.6458549	Activity
72.3551261	67.2690501	72.8456011	74.7357739	73	68.3646049	Activity
72.3551261	67.2690501	70.8456011	74.7357739	73	68.0521049	Activity
72.3551261	67.2690501	68.8456011	75.7357739	73	67.6302299	Activity
72.3551261	68.2690501	68.8456011	74.7357739	73	67.7396049	Activity
71.3551261	68.2690501	68.8456011	74.7357739	73	68.1771049	Activity
71.3551261	69.2690501	68.8456011	73.7357739	73	69.0364799	Activity
71.3551261	70.2690501	68.8456011	73.7357739	73	69.5989799	Activity
75.3551261	71.2690501	68.8456011	72.7357739	73	70.4427299	Activity
79.3551261	71.2690501	68.8456011	72.7357739	73	71.1458549	Activity
79.3551261	71.2690501	67.8456011	72.7357739	73	71.0208549	Activity
80.3551261	72.2690501	67.8456011	73.7357739	73	71.5677299	Activity
80.3551261	72.2690501	66.8456011	72.7357739	73	71.7239799	Activity
73.3551261	73.2690501	66.8456011	71.7357739	73	71.0052299	Activity
70.3551261	71.2690501	66.8456011	69.7357739	73	70.5833549	Activity
70.3551261	70.2690501	66.8456011	69.7357739	73	70.1614799	Activity
70.3551261	70.2690501	66.8456011	69.7357739	73	70.1458549	Activity
69.3551261	66.2690501	61.8456011	68.7357739	73	68.6771049	Activity
69.3551261	65.2690501	61.8456011	67.7357739	73	68.4583549	Activity
69.3551261	65.2690501	61.8456011	67.7357739	73	68.3021049	Activity
68.3551261	65.2690501	62.8456011	67.7357739	73	68.4114799	Activity
68.3551261	65.2690501	64.8456011	67.7357739	73	68.6771049	Activity
69.3551261	65.2690501	67.8456011	64.7357739	73	69.0521049	Activity
69.3551261	65.2690501	67.8456011	64.7357739	73	69.0989799	Activity
72.3551261	66.2690501	68.8456011	65.7357739	73	69.7708549	Activity
76.3551261	66.2690501	66.8456011	66.7357739	73	70.0989799	Activity
78.3551261	68.2690501	65.8456011	71.7357739	73	70.6458549	Activity
79.3551261	68.2690501	65.8456011	71.7357739	73	70.8802299	Activity
80.3551261	68.2690501	65.8456011	72.7357739	73	70.7396049	Activity
81.3551261	68.2690501	65.8456011	72.7357739	73	71.0052299	Activity
80.3551261	68.2690501	64.8456011	71.7357739	73	70.1614799	Activity
79.3551261	69.2690501	64.8456011	71.7357739	73	69.7083549	Activity

77.3551261	69.2690501	64.8456011	70.7357739	73	69.1146049	Activity
76.3551261	71.2690501	64.8456011	69.7357739	73	70.5451505	Activity
74.3551261	71.2690501	64.8456011	69.7357739	73	68.2951505	Activity
73.3551261	70.2690501	66.8456011	69.7357739	73	67.2951505	Activity
73.3551261	70.2690501	66.8456011	69.7357739	73	67.2951505	Activity
73.3551261	70.2690501	66.8456011	68.7357739	73	67.2951505	Activity
73.3551261	70.2690501	67.8456011	68.7357739	73	67.9201505	Activity
74.3551261	70.2690501	67.8456011	68.7357739	73	67.9201505	Activity
74.3551261	69.2690501	67.8456011	68.7357739	73	67.9201505	Activity
74.3551261	69.2690501	67.8456011	69.7357739	73	68.0451505	Activity
74.3551261	69.2690501	67.8456011	69.7357739	73	68.2951505	Activity
73.3551261	70.2690501	67.8456011	69.7357739	73	68.4201505	Activity
73.3551261	69.2690501	68.8456011	69.7357739	73	68.7951505	Activity
73.3551261	68.2690501	68.8456011	70.7357739	73	68.7951505	Activity
72.3551261	67.2690501	68.8456011	70.7357739	73	68.4201505	Activity
72.3551261	66.2690501	67.8456011	71.7357739	73	67.2951505	minute 1
72.3551261	65.2690501	67.8456011	71.7357739	69	67.1701505	minute 2
72.3551261	65.2690501	66.8456011	71.7357739	69	67.1701505	minute 3
71.3551261	65.2690501	66.8456011	71.7357739	69	67.1701505	minute 4
71.3551261	65.2690501	65.8456011	71.7357739	69	66.9201505	minute 5
71.3551261	66.2690501	65.8456011	71.7357739	69	67.1701505	minute 6
71.3551261	67.2690501	63.8456011	70.7357739	69	67.4201505	minute 7
72.3551261	69.2690501	62.8456011	69.7357739	69	68.0451505	minute 8
73.3551261	72.2690501	61.8456011	69.7357739	69	68.6701505	minute 9
73.3551261	76.2690501	61.8456011	69.7357739	69	69.5451505	minute 10
73.3551261	78.2690501	61.8456011	71.7357739	69	69.9201505	minute 11
73.3551261	78.2690501	61.8456011	72.7357739	69	70.0451505	minute 12
74.3551261	79.2690501	60.8456011	72.7357739	69	70.6701505	minute 13
75.3551261	78.2690501	61.8456011	72.7357739	69	70.7951505	minute 14
75.3551261	78.2690501	61.8456011	72.7357739	69	71.2951505	minute 15
75.3551261	77.2690501	61.8456011	72.7357739	69	71.1701505	minute 16
76.3551261	77.2690501	61.8456011	71.7357739	69	71.9201505	minute 17
76.3551261	77.2690501	62.8456011	71.7357739	69	72.0451505	minute 18
76.3551261	76.2690501	62.8456011	71.7357739	69	71.5451505	minute 19
77.3551261	76.2690501	62.8456011	70.7357739	69	71.2951505	minute 20
78.3551261	76.2690501	62.8456011	69.7357739	69	71.0451505	minute 21
78.3551261	75.2690501	64.8456011	69.7357739	69	71.1701505	minute 22
73.3551261	75.2690501	65.8456011	76.7357739	69	70.4201505	minute 23
72.3551261	75.2690501	66.8456011	77.7357739	69	70.7951505	minute 24
72.3551261	75.2690501	67.8456011	76.7357739	69	70.9201505	minute 25
72.3551261	73.2690501	67.8456011	76.7357739	69	70.4201505	minute 26
73.3551261	73.2690501	69.8456011	75.7357739	69	70.5451505	minute 27
73.3551261	71.2690501	70.8456011	75.7357739	69	70.0451505	minute 28
72.3551261	70.2690501	70.8456011	75.7357739	69	69.5451505	minute 29
72.3551261	69.2690501	70.8456011	73.7357739	69	69.5451505	minute 30

72.3551261	68.2690501	69.8456011	73.7357739	69	69.6701505	minute 31
72.3551261	67.2690501	69.8456011	72.7357739	69	69.6701505	minute 32
72.3551261	65.2690501	68.8456011	71.7357739	69	69.4201505	minute 33
73.3551261	64.2690501	67.8456011	71.7357739	69	69.6701505	minute 34
72.3551261	64.2690501	67.8456011	73.7357739	69	69.6701505	minute 35
72.3551261	63.2690501	66.8456011	73.7357739	69	69.5451505	minute 36
71.3551261	63.2690501	66.8456011	73.7357739	69	69.5451505	minute 37
71.3551261	63.2690501	66.8456011	72.7357739	69	69.7951505	minute 38
71.3551261	62.2690501	66.8456011	71.7357739	69	70.1701505	minute 39
71.3551261	62.2690501	66.8456011	68.7357739	69	68.240571	minute 40
71.3551261	62.2690501	66.8456011	68.7357739	69	68.099946	minute 41
71.3551261	63.2690501	66.8456011	65.7357739	69	70.7951505	minute 42
71.3551261	63.2690501	66.8456011	64.7357739	69	69.2951505	minute 43
71.3551261	62.2690501	66.8456011	64.7357739	69	69.1701505	minute 44
73.3551261	62.2690501	66.8456011	65.7357739	69	69.5451505	minute 45
75.3551261	61.2690501	66.8456011	67.7357739	69	69.6701505	minute 46
75.3551261	62.2690501	66.8456011	68.7357739	69	69.5451505	minute 47
75.3551261	62.2690501	66.8456011	68.7357739	69	69.7951505	minute 48
75.3551261	62.2690501	66.8456011	68.7357739	69	69.9201505	minute 49
73.3551261	62.2690501	66.8456011	69.7357739	69	69.5451505	minute 50
73.3551261	60.2690501	67.8456011	69.7357739	69	69.9201505	minute 51
73.3551261	60.2690501	69.8456011	69.7357739	69	70.5451505	minute 52
73.3551261	60.2690501	70.8456011	69.7357739	69	71.0451505	minute 53
74.3551261	61.2690501	70.8456011	69.7357739	69	71.6701505	minute 54
74.3551261	61.2690501	72.8456011	68.7357739	69	72.1701505	minute 55
74.3551261	61.2690501	72.8456011	68.7357739	69	71.9201505	minute 56
76.3551261	62.2690501	72.8456011	69.7357739	69	72.1701505	minute 57
76.3551261	61.2690501	75.8456011	69.7357739	69	72.4201505	minute 58
76.3551261	60.2690501	77.8456011	70.7357739	69	71.9201505	minute 59
76.3551261	58.2690501	77.8456011	71.7357739	69	71.5451505	minute 60
75.3551261	57.2690501	77.8456011	72.7357739	69	70.6701505	AFTER 1
75.3551261	57.2690501	76.8456011	72.7357739	69	70.0451505	AFTER 2
74.3551261	58.2690501	76.8456011	70.7357739	69	69.9201505	AFTER 3
74.3551261	58.2690501	74.8456011	69.7357739	69	69.0451505	AFTER 4
74.3551261	58.2690501	74.8456011	68.7357739	69	68.9201505	AFTER 5
75.3551261	59.2690501	74.8456011	68.7357739	69	69.1701505	AFTER 6
75.3551261	59.2690501	66.8456011	65.7357739	69	67.9201505	AFTER 7
75.3551261	60.2690501	64.8456011	66.7357739	69	67.7951505	AFTER 8
75.3551261	60.2690501	64.8456011	66.7357739	69	67.5451505	AFTER 9
76.3551261	58.2690501	64.8456011	67.7357739	69	67.9201505	AFTER 10
78.3551261	57.2690501	64.8456011	67.7357739	69	68.1701505	AFTER 11
78.3551261	57.2690501	62.8456011	68.7357739	69	67.9201505	AFTER 12
78.3551261	60.2690501	63.8456011	68.7357739	69	69.2951505	AFTER 13
81.3551261	60.2690501	63.8456011	69.7357739	69	69.6701505	AFTER 14
83.3551261	60.2690501	63.8456011	69.7357739	69	69.7951505	AFTER 15

84.3551261	59.2690501	63.8456011	69.7357739	69	70.2951505	AFTER 16
70.7951505	57.2690501	63.8456011	70.7357739	69	68.7251536	AFTER 17
71.0451505	57.2690501	63.8456011	71.7357739	69	68.6314036	AFTER 18
70.6701505	56.2690501	63.8456011	72.7357739	69	68.4595286	AFTER 19
81.3551261	56.2690501	63.8456011	72.7357739	69	70.0451505	AFTER 20
76.3551261	56.2690501	63.8456011	78.7357739	69	67.5989799	AFTER 21
76.3551261	56.2690501	63.8456011	78.7357739	69	67.7396049	AFTER 22
74.3551261	57.2690501	63.8456011	78.7357739	69	67.1771049	AFTER 23
74.3551261	57.2690501	63.8456011	93.7357739	69	67.1771049	AFTER 24
74.3551261	57.2690501	63.8456011	93.7357739	69	68.5451505	AFTER 25
74.3551261	57.2690501	63.8456011	92.7357739	69	68.6701505	AFTER 26
74.3551261	56.2690501	63.8456011	85.7357739	69	68.6701505	AFTER 27
74.3551261	56.2690501	64.8456011	85.7357739	69	68.6701505	AFTER 28
74.3551261	57.2690501	64.8456011	76.7357739	69	69.0451505	AFTER 29
74.3551261	59.2690501	64.8456011	73.7357739	69	69.0451505	AFTER 30
74.3551261	61.2690501	65.8456011	73.7357739	69	70.2951505	AFTER 31
74.3551261	62.2690501	65.8456011	70.7357739	69	69.1701505	AFTER 32
74.3551261	63.2690501	65.8456011	69.7357739	69	69.5451505	AFTER 33
74.3551261	63.2690501	65.8456011	68.7357739	69	69.1701505	AFTER 34
75.3551261	63.2690501	65.8456011	68.7357739	69	68.8704128	AFTER 35
76.3551261	63.2690501	65.8456011	67.7357739	69	69.2766628	AFTER 36
76.3551261	63.2690501	65.8456011	67.7357739	69	68.9172878	AFTER 37
76.3551261	63.2690501	65.8456011	67.7357739	69	69.2141628	AFTER 38
77.3551261	63.2690501	65.8456011	72.7357739	69	69.5266628	AFTER 39
77.3551261	64.2690501	65.8456011	75.7357739	69	69.5891628	AFTER 40
77.3551261	63.2690501	67.8456011	75.7357739	69	69.8704128	AFTER 41
78.3551261	63.2690501	70.8456011	77.7357739	69	71.5422878	AFTER 42
79.3551261	62.2690501	70.8456011	76.7357739	69	71.7297878	AFTER 43
77.3551261	62.2690501	70.8456011	71.7357739	69	71.5891628	AFTER 44
77.3551261	62.2690501	70.8456011	71.7357739	69	71.8704128	AFTER 45
78.3551261	62.2690501	71.8456011	71.7357739	63	72.2922878	AFTER 46
79.3551261	62.2690501	71.8456011	67.7357739	63	72.3079128	AFTER 47
79.3551261	62.2690501	71.8456011	67.7357739	63	72.2766628	AFTER 48
79.3551261	61.2690501	71.8456011	68.7357739	63	71.9797878	AFTER 49
79.3551261	61.2690501	71.8456011	69.7357739	63	71.2297878	AFTER 50
79.3551261	61.2690501	68.8456011	68.7357739	63	70.9485378	AFTER 51
78.3551261	61.2690501	68.8456011	66.7357739	63	70.6047878	AFTER 52
77.3551261	62.2690501	67.8456011	66.7357739	63	70.4172878	AFTER 53
77.3551261	62.2690501	66.8456011	66.7357739	63	72.7951505	AFTER 54
75.3551261	61.2690501	64.8456011	66.7357739	63	71.7951505	AFTER 55
75.3551261	61.2690501	64.8456011	66.7357739	63	71.7951505	AFTER 56
75.3551261	56.2690501	63.8456011	66.7357739	63	70.7951505	AFTER 57
75.3551261	56.2690501	63.8456011	67.7357739	63	70.2951505	AFTER 58
76.3551261	56.2690501	63.8456011	68.7357739	63	68.2454128	AFTER 59
77.3551261	56.2690501	63.8456011	68.7357739	63	70.6701505	AFTER 60

79.3551261	56.2690501	63.8456011	68.7357739	63	69.9641628	AFTER 61
77.3551261	56.2690501	63.8456011	68.7357739	63	70.5891628	AFTER 62
77.3551261	54.2690501	64.8456011	68.7357739	63	70.5891628	AFTER 63
77.3551261	54.2690501	64.8456011	68.7357739	63	72.1701505	AFTER 64
75.3551261	55.2690501	64.8456011	68.7357739	63	71.1701505	AFTER 65
75.3551261	55.2690501	64.8456011	68.7357739	63	71.1701505	AFTER 66
73.3551261	56.2690501	64.8456011	68.7357739	63	70.4201505	AFTER 67
73.3551261	58.2690501	64.8456011	79.7357739	63	70.4201505	AFTER 68
74.3551261	57.2690501	64.8456011	79.7357739	63	70.5451505	AFTER 69
74.3551261	57.2690501	64.8456011	79.7357739	63	70.0451505	AFTER 70
74.3551261	55.2690501	63.8456011	79.7357739	63	69.1701505	AFTER 71
74.3551261	55.2690501	63.8456011	79.7357739	63	68.9201505	AFTER 72
74.3551261	55.2690501	63.8456011	75.7357739	63	68.5451505	AFTER 73
75.3551261	55.2690501	64.8456011	74.7357739	63	68.9201505	AFTER 74
76.3551261	55.2690501	68.8456011	73.7357739	63	69.4201505	AFTER 75
76.3551261	55.2690501	68.8456011	72.7357739	63	69.2951505	AFTER 76
76.3551261	61.2690501	69.8456011	72.7357739	63	70.0451505	AFTER 77
76.3551261	61.2690501	70.8456011	71.7357739	63	70.1701505	AFTER 78
76.3551261	61.2690501	71.8456011	71.7357739	63	70.2951505	AFTER 79
77.3551261	62.2690501	71.8456011	71.7357739	63	70.5451505	AFTER 80
80.3551261	62.2690501	73.8456011	70.7357739	63	71.0451505	AFTER 81
82.3551261	59.2690501	74.8456011	71.7357739	63	71.0451505	AFTER 82
83.3551261	58.2690501	76.8456011	71.7357739	63	71.2951505	AFTER 83
82.3551261	58.2690501	77.8456011	71.7357739	63	71.5451505	AFTER 84
82.3551261	55.2690501	77.8456011	70.7357739	63	71.2951505	AFTER 85
83.3551261	54.2690501	79.8456011	70.7357739	63	71.6701505	AFTER 86
83.3551261	54.2690501	80.8456011	70.7357739	63	71.5451505	AFTER 87
83.3551261	54.2690501	80.8456011	69.7357739	63	71.4201505	AFTER 88
82.3551261	54.2690501	80.8456011	71.7357739	63	71.2951505	AFTER 89
82.3551261	55.2690501	80.8456011	71.7357739	63	71.7951505	AFTER 90
81.3551261	55.2690501	80.8456011	72.7357739	63	71.6701505	AFTER 91
81.3551261	55.2690501	80.8456011	73.7357739	63	71.4201505	AFTER 92
81.3551261	55.2690501	80.8456011	74.7357739	63	71.2951505	AFTER 93
81.3551261	57.2690501	78.8456011	74.7357739	63	71.1701505	AFTER 94
80.3551261	57.2690501	75.8456011	74.7357739	63	70.5451505	AFTER 95
80.3551261	57.2690501	75.8456011	74.7357739	63	70.5451505	AFTER 96
78.3551261	57.2690501	75.8456011	75.7357739	63	70.0451505	AFTER 97
79.3551261	57.2690501	75.8456011	75.7357739	63	70.1701505	AFTER 98
78.3551261	59.2690501	75.8456011	74.7357739	63	70.1701505	AFTER 99
78.3551261	59.2690501	74.8456011	74.7357739	63	69.7951505	AFTER 100
78.3551261	59.2690501	75.8456011	74.7357739	63	70.0451505	AFTER 101
78.3551261	59.2690501	75.8456011	76.7357739	63	70.4201505	AFTER 102
78.3551261	59.2690501	75.8456011	76.7357739	63	70.4201505	AFTER 103
80.3551261	59.2690501	75.8456011	76.7357739	63	70.2951505	AFTER 104
81.3551261	59.2690501	75.8456011	76.7357739	63	70.1701505	AFTER 105

80.3551261	59.2690501	75.8456011	73.7357739	63	69.9201505	AFTER 106
78.3551261	59.2690501	75.8456011	69.7357739	63	69.6701505	AFTER 107
78.3551261	60.2690501	75.8456011	66.7357739	63	69.6701505	AFTER 108
75.3551261	62.2690501	77.8456011	64.7357739	63	69.6701505	AFTER 109
74.3551261	62.2690501	79.8456011	63.7357739	63	70.0451505	AFTER 110
74.3551261	62.2690501	79.8456011	63.7357739	63	70.2951505	AFTER 111
74.3551261	62.2690501	79.8456011	63.7357739	63	69.6701505	AFTER 112
74.3551261	62.2690501	73.8456011	68.7357739	63	69.0451505	AFTER 113
74.3551261	63.2690501	73.8456011	70.7357739	63	68.5451505	AFTER 114
74.3551261	63.2690501	71.8456011	71.7357739	63	68.0451505	AFTER 115
75.3551261	63.2690501	70.8456011	73.7357739	63	67.9201505	AFTER 116
75.3551261	64.2690501	69.8456011	73.7357739	63	67.6701505	AFTER 117
75.3551261	67.2690501	69.8456011	73.7357739	63	67.9201505	AFTER 118
75.3551261	67.2690501	69.8456011	73.7357739	63	67.6701505	AFTER 119
75.3551261	67.2690501	69.8456011	73.7357739	63	67.6701505	AFTER 120
76.3551261	64.2690501	68.8456011	67.7357739	63	66.9201505	AFTER 121
76.3551261	62.2690501	68.8456011	65.7357739	63	66.6701505	AFTER 122
76.3551261	61.2690501	67.8456011	65.7357739	63	66.4201505	AFTER 123
76.3551261	61.2690501	67.8456011	65.7357739	63	66.5451505	AFTER 124
76.3551261	61.2690501	67.8456011	67.7357739	63	66.7951505	AFTER 125
76.3551261	63.2690501	67.8456011	68.7357739	63	67.4201505	AFTER 126
76.3551261	65.2690501	68.8456011	68.7357739	63	67.6701505	AFTER 127
76.3551261	66.2690501	69.8456011	69.7357739	63	68.0451505	AFTER 128
76.3551261	67.2690501	70.8456011	69.7357739	63	68.4201505	AFTER 129
76.3551261	67.2690501	70.8456011	69.7357739	63	68.4201505	AFTER 130
75.3551261	67.2690501	71.8456011	69.7357739	63	68.0451505	AFTER 131
74.3551261	66.2690501	71.8456011	70.7357739	63	69.2951505	AFTER 132
74.3551261	66.2690501	71.8456011	72.7357739	63	70.7951505	AFTER 133
74.3551261	64.2690501	71.8456011	72.7357739	63	70.5451505	AFTER 134
74.3551261	60.2690501	71.8456011	76.7357739	63	70.7951505	AFTER 135
74.3551261	60.2690501	70.8456011	79.7357739	63	70.9201505	AFTER 136
74.3551261	61.2690501	69.8456011	79.7357739	63	70.2951505	AFTER 137
74.3551261	61.2690501	69.8456011	80.7357739	63	69.9201505	AFTER 138
74.3551261	60.2690501	69.8456011	81.7357739	63	69.5451505	AFTER 139
71.3551261	60.2690501	68.8456011	81.7357739	63	69.0451505	AFTER 140
71.3551261	60.2690501	68.8456011	81.7357739	63	69.1701505	AFTER 141
71.3551261	59.2690501	68.8456011	81.7357739	63	69.0451505	AFTER 142
72.3551261	60.2690501	68.8456011	83.7357739	63	69.4201505	AFTER 143
72.3551261	62.2690501	68.8456011	84.7357739	63	69.7951505	AFTER 144
74.3551261	63.2690501	68.8456011	84.7357739	63	70.0451505	AFTER 145
78.3551261	64.2690501	68.8456011	85.7357739	63	70.2951505	AFTER 146
79.3551261	64.2690501	69.8456011	85.7357739	63	71.2951505	AFTER 147
80.3551261	64.2690501	70.8456011	85.7357739	63	71.4201505	AFTER 148
80.3551261	63.2690501	70.8456011	85.7357739	63	71.4201505	AFTER 149
81.3551261	63.2690501	71.8456011	85.7357739	63	70.9201505	AFTER 150

81.3551261	64.2690501	70.8456011	85.7357739	63	70.5451505	AFTER 151
81.3551261	64.2690501	68.8456011	85.7357739	63	70.1701505	AFTER 152
80.3551261	64.2690501	68.8456011	85.7357739	63	69.9201505	AFTER 153
80.3551261	64.2690501	68.8456011	84.7357739	63	69.7951505	AFTER 154
79.3551261	64.2690501	68.8456011	84.7357739	63	69.0451505	AFTER 155
78.3551261	63.2690501	68.8456011	86.7357739	63	68.5451505	AFTER 156
78.3551261	63.2690501	67.8456011	87.7357739	63	67.9201505	AFTER 157
76.3551261	62.2690501	66.8456011	87.7357739	63	66.6701505	AFTER 158
74.3551261	62.2690501	66.8456011	88.7357739	63	66.4201505	AFTER 159
74.3551261	62.2690501	65.8456011	89.7357739	63	66.0451505	AFTER 160
74.3551261	65.2690501	65.8456011	90.7357739	63	66.1701505	AFTER 161
74.3551261	66.2690501	65.8456011	90.7357739	63	66.2951505	AFTER 162
74.3551261	66.2690501	65.8456011	90.7357739	63	66.2951505	AFTER 163
75.3551261	65.2690501	65.8456011	90.7357739	63	66.5451505	AFTER 164
75.3551261	65.2690501	65.8456011	90.7357739	63	66.6701505	AFTER 165
75.3551261	65.2690501	65.8456011	90.7357739	63	67.0451505	AFTER 166
74.3551261	65.2690501	65.8456011	90.7357739	63	67.9201505	AFTER 167
73.3551261	65.2690501	65.8456011	85.7357739	63	67.9201505	AFTER 168
73.3551261	65.2690501	65.8456011	84.7357739	63	67.6701505	AFTER 169
73.3551261	65.2690501	67.8456011	84.7357739	63	68.0451505	AFTER 170
72.3551261	65.2690501	70.8456011	84.7357739	63	68.0451505	AFTER 171
71.3551261	66.2690501	71.8456011	85.7357739	63	68.0451505	AFTER 172
70.3551261	66.2690501	71.8456011	85.7357739	63	67.9201505	AFTER 173
70.3551261	66.2690501	72.8456011	85.7357739	63	68.0451505	AFTER 174
73.3551261	63.2690501	74.8456011	85.7357739	63	68.5451505	AFTER 175
73.3551261	61.2690501	74.8456011	85.7357739	63	68.4201505	AFTER 176
73.3551261	61.2690501	75.8456011	83.7357739	63	69.0451505	AFTER 177
72.3551261	61.2690501	76.8456011	83.7357739	63	69.5451505	AFTER 178
72.3551261	60.2690501	71.8456011	75.7357739	63	69.1701505	AFTER 179
72.3551261	59.2690501	69.8456011	72.7357739	63	69.1701505	AFTER 180
70.3551261	58.2690501	69.8456011	72.7357739	63	69.0451505	AFTER 181
70.3551261	57.2690501	68.8456011	70.7357739	63	69.1701505	AFTER 182
70.3551261	57.2690501	67.8456011	70.7357739	63	69.0451505	AFTER 183
70.3551261	57.2690501	67.8456011	70.7357739	63	69.1701505	AFTER 184
70.3551261	57.2690501	67.8456011	70.7357739	63	68.9201505	AFTER 185
70.3551261	57.2690501	67.8456011	70.7357739	63	68.6701505	AFTER 186
70.3551261	58.2690501	67.8456011	70.7357739	63	68.7951505	AFTER 187
70.3551261	57.2690501	66.8456011	70.7357739	63	68.5451505	AFTER 188
71.3551261	57.2690501	66.8456011	70.7357739	63	69.1701505	AFTER 189
71.3551261	57.2690501	65.8456011	70.7357739	63	68.7951505	AFTER 190
71.3551261	58.2690501	64.8456011	70.7357739	63	69.0451505	AFTER 191
71.3551261	58.2690501	64.8456011	70.7357739	63	68.9201505	AFTER 192
70.3551261	58.2690501	64.8456011	70.7357739	63	69.0451505	AFTER 193
70.3551261	58.2690501	64.8456011	69.7357739	63	69.5451505	AFTER 194
69.3551261	58.2690501	64.8456011	68.7357739	63	69.4201505	AFTER 195

69.3551261	58.2690501	64.8456011	68.7357739	63	69.2951505	AFTER 196
69.3551261	58.2690501	65.8456011	68.7357739	63	69.6701505	AFTER 197
70.3551261	58.2690501	65.8456011	69.7357739	63	70.2951505	AFTER 198
70.3551261	58.2690501	68.8456011	77.7357739	63	70.9201505	AFTER 199
71.3551261	58.2690501	68.8456011	83.7357739	63	71.4201505	AFTER 200
71.3551261	59.2690501	68.8456011	86.7357739	63	71.5451505	AFTER 201
71.3551261	59.2690501	70.8456011	88.7357739	63	68.3021049	AFTER 202
72.3551261	58.2690501	71.8456011	89.7357739	63	67.3802299	AFTER 203
72.3551261	58.2690501	71.8456011	89.7357739	63	67.2552299	AFTER 204
73.3551261	58.2690501	71.8456011	86.7357739	63	67.5364799	AFTER 205
72.3551261	58.2690501	71.8456011	80.7357739	63	67.5989799	AFTER 206
72.3551261	58.2690501	72.8456011	80.7357739	63	69.2951505	AFTER 207
72.3551261	59.2690501	73.8456011	80.7357739	63	69.4201505	AFTER 208
71.3551261	59.2690501	73.8456011	80.7357739	63	68.6701505	AFTER 209
71.3551261	59.2690501	74.8456011	80.7357739	63	68.6701505	AFTER 210
71.3551261	59.2690501	74.8456011	80.7357739	63	68.1701505	AFTER 211
71.3551261	59.2690501	74.8456011	80.7357739	63	67.7951505	AFTER 212
71.3551261	57.2690501	74.8456011	80.7357739	63	67.7951505	AFTER 213
71.3551261	57.2690501	74.8456011	80.7357739	63	68.0451505	AFTER 214
70.3551261	57.2690501	74.8456011	80.7357739	63	68.1701505	AFTER 215
70.3551261	57.2690501	76.8456011	80.7357739	63	68.7951505	AFTER 216
72.3551261	56.2690501	76.8456011	79.7357739	63	68.9201505	AFTER 217
72.3551261	54.2690501	76.8456011	79.7357739	63	69.0451505	AFTER 218
73.3551261	53.2690501	74.8456011	79.7357739	63	68.9201505	AFTER 219
73.3551261	53.2690501	68.8456011	78.7357739	63	68.1701505	AFTER 220
73.3551261	53.2690501	68.8456011	77.7357739	63	68.5451505	AFTER 221
73.3551261	53.2690501	67.8456011	77.7357739	63	68.2951505	AFTER 222
74.3551261	53.2690501	67.8456011	76.7357739	63	68.7951505	AFTER 223
74.3551261	54.2690501	67.8456011	74.7357739	63	68.9201505	AFTER 224
75.3551261	61.2690501	67.8456011	74.7357739	63	69.4201505	AFTER 225
76.3551261	63.2690501	67.8456011	74.7357739	63	69.5451505	AFTER 226
79.3551261	63.2690501	67.8456011	74.7357739	63	70.0451505	AFTER 227
79.3551261	63.2690501	66.8456011	75.7357739	63	69.9201505	AFTER 228
81.3551261	60.2690501	66.8456011	76.7357739	63	69.4201505	AFTER 229
81.3551261	60.2690501	65.8456011	79.7357739	63	69.1701505	AFTER 230
81.3551261	61.2690501	65.8456011	80.7357739	63	69.1701505	AFTER 231
81.3551261	63.2690501	64.8456011	82.7357739	63	69.2951505	AFTER 232
81.3551261	65.2690501	64.8456011	83.7357739	63	69.1701505	AFTER 233
81.3551261	66.2690501	63.8456011	83.7357739	63	69.1701505	AFTER 234
81.3551261	67.2690501	64.8456011	82.7357739	63	69.2951505	AFTER 235
81.3551261	67.2690501	64.8456011	82.7357739	63	69.1701505	AFTER 236
80.3551261	66.2690501	64.8456011	80.7357739	63	69.0451505	AFTER 237
79.3551261	65.2690501	64.8456011	76.7357739	63	69.0451505	AFTER 238
79.3551261	64.2690501	65.8456011	76.7357739	63	69.0451505	AFTER 239
78.3551261	65.2690501	65.8456011	72.7357739	63	69.4201505	AFTER 240

77.3551261	65.2690501	65.8456011	72.7357739	63	69.7951505
77.3551261	66.2690501	65.8456011	73.7357739	63	70.1701505
77.3551261	67.2690501	65.8456011	73.7357739	63	70.1701505
77.3551261	67.2690501	65.8456011	73.7357739	63	69.6701505
75.3551261	66.2690501	65.8456011	74.7357739	63	69.1701505
75.3551261	66.2690501	65.8456011	74.7357739	63	68.9201505
75.3551261	67.2690501	65.8456011	74.7357739	63	68.2951505
75.3551261	68.2690501	68.8456011	70.7357739	63	68.6701505
76.3551261	69.2690501	68.8456011	66.7357739	63	68.6701505
76.3551261	69.2690501	67.8456011	65.7357739	63	68.1701505
76.3551261	69.2690501	67.8456011	65.7357739	63	68.0451505
76.3551261	69.2690501	67.8456011	67.7357739	63	68.1701505
76.3551261	68.2690501	67.8456011	69.7357739	63	68.0451505
75.3551261	67.2690501	67.8456011	70.7357739	63	67.7951505
75.3551261	66.2690501	67.8456011	71.7357739	63	67.6701505
75.3551261	67.2690501	67.8456011	72.7357739	63	67.9201505
76.3551261	68.2690501	67.8456011	74.7357739	63	68.2951505
78.3551261	68.2690501	67.8456011	77.7357739	63	68.6701505
78.3551261	69.2690501	67.8456011	79.7357739	63	69.0451505
79.3551261	69.2690501	67.8456011	79.7357739	63	68.7951505
80.3551261	68.2690501	67.8456011	78.7357739	63	68.4201505
80.3551261	66.2690501	67.8456011	78.7357739	63	68.1701505
80.3551261	64.2690501	67.8456011	78.7357739	63	67.6701505
80.3551261	61.2690501	66.8456011	78.7357739	63	67.1701505
80.3551261	59.2690501	66.8456011	78.7357739	63	67.0451505
80.3551261	58.2690501	66.8456011	78.7357739	63	66.9201505
75.3551261	58.2690501	66.8456011	78.7357739	63	66.4201505
73.3551261	58.2690501	66.8456011	78.7357739	63	65.2951505
72.3551261	57.2690501	66.8456011	78.7357739	63	64.9201505
72.3551261	57.2690501	66.8456011	78.7357739	63	64.5451505
72.3551261	56.2690501	65.8456011	78.7357739	63	64.2951505
72.3551261	56.2690501	66.8456011	79.7357739	63	64.2951505
73.3551261	56.2690501	65.8456011	79.7357739	63	64.1701505
74.3551261	56.2690501	65.8456011	78.7357739	63	64.5451505
75.3551261	61.2690501	65.8456011	76.7357739	63	64.9201505
76.3551261	63.2690501	65.8456011	76.7357739	63	65.2951505
76.3551261	65.2690501	64.8456011	76.7357739	63	65.2951505
76.3551261	65.2690501	64.8456011	74.7357739	63	65.6701505
76.3551261	65.2690501	63.8456011	74.7357739	63	65.9201505
76.3551261	64.2690501	62.8456011	74.7357739	63	65.7951505
76.3551261	63.2690501	62.8456011	73.7357739	63	65.5451505
76.3551261	63.2690501	62.8456011	73.7357739	63	65.5451505
76.3551261	61.2690501	62.8456011	72.7357739	63	65.9201505
77.3551261	61.2690501	61.8456011	72.7357739	63	65.9201505
77.3551261	62.2690501	61.8456011	72.7357739	63	66.2951505

77.3551261	62.2690501	61.8456011	72.7357739	63	66.2951505
78.3551261	62.2690501	61.8456011	72.7357739	63	66.4201505
77.3551261	62.2690501	61.8456011	72.7357739	63	66.4201505
77.3551261	62.2690501	61.8456011	72.7357739	63	66.4201505
77.3551261	62.2690501	62.8456011	73.7357739	63	67.0451505
77.3551261	62.2690501	62.8456011	72.7357739	63	67.0451505
77.3551261	62.2690501	62.8456011	70.7357739	63	67.7951505
77.3551261	62.2690501	62.8456011	70.7357739	63	69.8547878
76.3551261	62.2690501	62.8456011	69.7357739	63	69.9641628
76.3551261	62.2690501	62.8456011	68.7357739	63	70.5110378
76.3551261	62.2690501	62.8456011	68.7357739	63	70.4172878
76.3551261	62.2690501	62.8456011	68.7357739	63	70.3547878
76.3551261	63.2690501	64.8456011	68.7357739	63	70.7922878
76.3551261	64.2690501	64.8456011	69.7357739	63	71.1204128
76.3551261	65.2690501	65.8456011	69.7357739	63	71.2610378
79.3551261	65.2690501	66.8456011	69.7357739	63	71.6985378
80.3551261	66.2690501	66.8456011	70.7357739	63	71.7141628
81.3551261	64.2690501	67.8456011	70.7357739	63	71.4172878
78.3551261	64.2690501	68.8456011	71.7357739	63	71.1360378
76.3551261	64.2690501	68.8456011	72.7357739	63	71.0266628
74.3551261	65.2690501	69.8456011	74.7357739	63	70.8704128
73.3551261	65.2690501	69.8456011	76.7357739	63	70.0422878
73.3551261	64.2690501	68.8456011	76.7357739	63	68.9485378
73.3551261	64.2690501	68.8456011	77.7357739	63	68.4954128
74.3551261	64.2690501	67.8456011	77.7357739	63	68.2141628
74.3551261	66.2690501	67.8456011	78.7357739	63	68.1829128
74.3551261	67.2690501	67.8456011	78.7357739	63	68.0266628
75.3551261	67.2690501	67.8456011	78.7357739	63	68.2610378
75.3551261	67.2690501	67.8456011	78.7357739	63	68.6360378
76.3551261	66.2690501	67.8456011	78.7357739	63	69.8704128
76.3551261	65.2690501	66.8456011	78.7357739	63	70.5266628
78.3551261	65.2690501	67.8456011	78.7357739	63	70.5422878
78.3551261	65.2690501	67.8456011	79.7357739	63	71.3547878
79.3551261	65.2690501	66.8456011	78.7357739	63	71.3547878
79.3551261	65.2690501	66.8456011	78.7357739	63	73.0451505
79.3551261	66.2690501	66.8456011	78.7357739	63	72.5451505
79.3551261	68.2690501	66.8456011	77.7357739	63	72.5451505
78.3551261	72.2690501	65.8456011	76.7357739	63	70.7552299
77.3551261	75.2690501	65.8456011	76.7357739	63	70.3802299
77.3551261	77.2690501	65.8456011	77.7357739	63	70.5208549
77.3551261	78.2690501	65.8456011	77.7357739	63	69.9739799
77.3551261	78.2690501	65.8456011	77.7357739	63	69.8489799
78.3551261	77.2690501	65.8456011	77.7357739	63	69.8489799
77.3551261	73.2690501	65.8456011	81.7357739	63	68.5677299
76.3551261	69.2690501	64.8456011	82.7357739	63	67.4427299

76.3551261	66.2690501	64.8456011	81.7357739	63	67.0052299
75.3551261	63.2690501	64.8456011	81.7357739	63	66.1458549
76.3551261	63.2690501	64.8456011	80.7357739	63	66.1458549
75.3551261	63.2690501	65.8456011	80.7357739	63	66.1302299
75.3551261	63.2690501	65.8456011	80.7357739	63	66.1458549
75.3551261	63.2690501	66.8456011	80.7357739	63	66.2708549
75.3551261	63.2690501	67.8456011	80.7357739	63	66.3489799
76.3551261	63.2690501	67.8456011	84.7357739	63	66.6302299
77.3551261	62.2690501	67.8456011	84.7357739	63	66.7552299
78.3551261	62.2690501	67.8456011	84.7357739	63	66.8802299
79.3551261	62.2690501	67.8456011	84.7357739	63	69.5451505
78.3551261	62.2690501	67.8456011	84.7357739	63	69.0451505
78.3551261	62.2690501	66.8456011	85.7357739	63	69.0451505
76.3551261	62.2690501	65.8456011	85.7357739	63	69.2951505
77.3551261	67.2690501	65.8456011	85.7357739	63	69.9201505
79.3551261	69.2690501	65.8456011	85.7357739	63	70.7951505
81.3551261	69.2690501	65.8456011	85.7357739	63	71.2951505
85.3551261	64.2690501	65.8456011	85.7357739	63	70.9201505
70.7951505	62.2690501	65.8456011	86.7357739	63	68.7251536
71.0451505	62.2690501	65.8456011	86.7357739	63	68.7564036
71.2951505	62.2690501	65.8456011	86.7357739	63	69.0376536
72.6701505	68.2690501	64.8456011	86.7357739	63	70.3345286
73.5451505	73.2690501	64.8456011	85.7357739	63	71.4439036
90.3551261	76.2690501	64.8456011	85.7357739	63	74.2951505
90.3551261	76.2690501	64.8456011	85.7357739	63	74.5451505
91.3551261	77.2690501	64.8456011	85.7357739	63	75.1701505
76.0451505	77.2690501	66.8456011	85.7357739	63	74.0064036
76.0451505	75.2690501	67.8456011	85.7357739	63	73.8814036
76.2951505	73.2690501	66.8456011	84.7357739	63	73.6626536
75.4201505	73.2690501	66.8456011	81.7357739	63	72.5532786
74.9201505	70.2690501	65.8456011	81.7357739	63	71.7407786
73.9201505	69.2690501	64.8456011	78.7357739	63	70.4907786
72.9201505	67.2690501	64.8456011	77.7357739	63	69.6157786
72.2951505	67.2690501	64.8456011	77.7357739	63	69.0376536
71.7951505	67.2690501	64.8456011	75.7357739	63	68.4751536
72.2951505	69.2690501	65.8456011	75.7357739	63	69.0376536
72.5451505	69.2690501	66.8456011	75.7357739	63	69.3189036
72.1701505	69.2690501	66.8456011	76.7357739	63	68.8970286
72.0451505	69.2690501	66.8456011	76.7357739	63	68.8814036
71.4201505	70.2690501	66.8456011	74.7357739	63	68.4282786
71.4201505	71.2690501	66.8456011	73.7357739	63	68.6782786
71.0451505	71.2690501	65.8456011	73.7357739	63	68.3814036
70.5451505	71.2690501	64.8456011	74.7357739	63	68.3189036
84.3551261	70.2690501	64.8456011	75.7357739	63	70.0451505
84.3551261	71.2690501	63.8456011	75.7357739	63	71.0451505

85.3551261	72.2690501	63.8456011	75.7357739	63	71.5451505
85.3551261	72.2690501	63.8456011	76.7357739	63	72.0451505
85.3551261	71.2690501	63.8456011	76.7357739	63	72.0451505
85.3551261	70.2690501	64.8456011	75.7357739	63	72.1701505
85.3551261	69.2690501	64.8456011	73.7357739	63	70.1927299
83.3551261	69.2690501	64.8456011	73.7357739	63	69.6458549
82.3551261	67.2690501	63.8456011	76.7357739	63	68.8177299
82.3551261	66.2690501	63.8456011	76.7357739	63	68.2552299
82.3551261	66.2690501	63.8456011	76.7357739	63	68.5364799
82.3551261	66.2690501	62.8456011	78.7357739	63	68.6927299
82.3551261	66.2690501	62.8456011	77.7357739	63	68.6927299
82.3551261	66.2690501	63.8456011	77.7357739	63	68.8177299
82.3551261	66.2690501	63.8456011	76.7357739	63	68.8489799
82.3551261	66.2690501	63.8456011	76.7357739	63	68.7083549
82.3551261	66.2690501	63.8456011	76.7357739	63	68.7083549
82.3551261	66.2690501	63.8456011	76.7357739	63	68.2396049
83.3551261	66.2690501	63.8456011	76.7357739	63	68.4739799
83.3551261	66.2690501	63.8456011	77.7357739	63	70.1701505
83.3551261	67.2690501	63.8456011	78.7357739	63	70.2951505
83.3551261	67.2690501	63.8456011	79.7357739	63	69.7951505
82.3551261	67.2690501	63.8456011	79.7357739	63	69.2951505
81.3551261	66.2690501	63.8456011	81.7357739	63	68.9201505
81.3551261	68.2690501	63.8456011	82.7357739	63	69.9201505
74.3551261	68.2690501	67.8456011	83.7357739	63	69.5451505
74.3551261	69.2690501	69.8456011	83.7357739	63	70.1701505
73.3551261	69.2690501	69.8456011	83.7357739	63	70.0451505
73.3551261	69.2690501	70.8456011	83.7357739	63	70.9201505
72.3551261	69.2690501	69.8456011	83.7357739	63	71.0451505
71.3551261	70.2690501	69.8456011	84.7357739	63	70.1701505
70.3551261	71.2690501	68.8456011	84.7357739	63	70.0451505
69.3551261	71.2690501	67.8456011	84.7357739	63	69.7951505
68.3551261	71.2690501	67.8456011	84.7357739	63	69.1701505
68.3551261	70.2690501	67.8456011	82.7357739	63	68.5451505
67.3551261	70.2690501	66.8456011	81.7357739	63	67.9201505
66.3551261	70.2690501	66.8456011	81.7357739	63	67.4201505
66.3551261	71.2690501	65.8456011	82.7357739	63	67.2951505
66.3551261	71.2690501	65.8456011	83.7357739	63	67.4201505
66.3551261	72.2690501	65.8456011	84.7357739	63	67.5451505
66.3551261	74.2690501	64.8456011	86.7357739	63	67.6701505
66.3551261	76.2690501	63.8456011	86.7357739	63	68.0451505
66.3551261	76.2690501	63.8456011	86.7357739	63	67.7951505
66.3551261	76.2690501	63.8456011	86.7357739	63	68.0451505
66.3551261	76.2690501	62.8456011	86.7357739	63	68.1701505
64.3551261	76.2690501	62.8456011	86.7357739	63	67.9201505
64.3551261	76.2690501	62.8456011	87.7357739	63	68.2951505

63.3551261	76.2690501	62.8456011	88.7357739	63	68.1701505
64.3551261	77.2690501	62.8456011	89.7357739	63	68.2951505
65.3551261	76.2690501	63.8456011	89.7357739	63	68.1701505
67.3551261	76.2690501	63.8456011	90.7357739	63	68.1701505
68.3551261	76.2690501	65.8456011	90.7357739	63	68.0451505
70.3551261	76.2690501	66.8456011	90.7357739	63	68.6701505
70.3551261	76.2690501	68.8456011	91.7357739	63	68.9201505
70.3551261	76.2690501	67.8456011	91.7357739	63	68.9201505
70.3551261	75.2690501	67.8456011	91.7357739	63	69.1701505
70.3551261	75.2690501	67.8456011	91.7357739	63	69.5451505
68.3551261	74.2690501	67.8456011	91.7357739	63	69.4201505
68.3551261	74.2690501	67.8456011	92.7357739	63	69.5451505
71.3551261	74.2690501	67.8456011	91.7357739	63	70.1701505
71.3551261	64.2690501	67.8456011	91.7357739	63	68.6701505
72.3551261	64.2690501	67.8456011	91.7357739	63	68.5451505
73.3551261	64.2690501	67.8456011	91.7357739	63	68.6701505
74.3551261	64.2690501	65.8456011	85.7357739	63	68.6701505
75.3551261	65.2690501	65.8456011	85.7357739	63	69.0451505
73.3551261	65.2690501	68.8456011	69.7357739	63	69.2951505
73.3551261	65.2690501	68.8456011	68.7357739	63	68.4201505
73.3551261	64.2690501	68.8456011	68.7357739	63	68.9201505
73.3551261	64.2690501	69.8456011	68.7357739	63	69.9201505
73.3551261	63.2690501	69.8456011	68.7357739	63	70.1701505
73.3551261	63.2690501	68.8456011	68.7357739	63	70.0451505
71.3551261	63.2690501	67.8456011	68.7357739	63	69.9201505
71.3551261	64.2690501	67.8456011	68.7357739	63	70.2951505
71.3551261	64.2690501	66.8456011	68.7357739	63	69.6701505
72.3551261	64.2690501	65.8456011	68.7357739	63	69.7951505
72.3551261	65.2690501	65.8456011	68.7357739	63	69.9201505
72.3551261	65.2690501	64.8456011	68.7357739	63	69.6701505
72.3551261	64.2690501	64.8456011	69.7357739	63	69.5451505
72.3551261	64.2690501	64.8456011	69.7357739	63	68.9201505
72.3551261	64.2690501	64.8456011	68.7357739	63	68.5451505
72.3551261	65.2690501	63.8456011	68.7357739	63	68.5451505
72.3551261	67.2690501	63.8456011	68.7357739	63	68.6701505
72.3551261	68.2690501	62.8456011	68.7357739	63	68.2951505
72.3551261	68.2690501	62.8456011	68.7357739	63	67.7951505
72.3551261	68.2690501	62.8456011	68.7357739	63	67.5451505
72.3551261	68.2690501	62.8456011	68.7357739	63	67.4201505
72.3551261	68.2690501	62.8456011	68.7357739	63	66.9201505
73.3551261	68.2690501	62.8456011	68.7357739	63	66.6701505
74.3551261	69.2690501	62.8456011	68.7357739	63	67.0451505
74.3551261	69.2690501	62.8456011	69.7357739	63	67.0451505
74.3551261	71.2690501	62.8456011	69.7357739	63	67.4201505
74.3551261	72.2690501	62.8456011	71.7357739	63	67.7951505

74.3551261	72.2690501	62.8456011	73.7357739	63	67.7951505
74.3551261	68.2690501	62.8456011	74.7357739	63	67.6701505
74.3551261	67.2690501	63.8456011	76.7357739	63	67.6701505
74.3551261	67.2690501	63.8456011	76.7357739	63	67.6701505
74.3551261	67.2690501	63.8456011	77.7357739	63	68.1701505
74.3551261	66.2690501	63.8456011	77.7357739	63	68.2951505
73.3551261	66.2690501	63.8456011	78.7357739	63	68.2951505
73.3551261	63.2690501	64.8456011	78.7357739	63	68.0451505
73.3551261	62.2690501	64.8456011	78.7357739	63	68.1701505
73.3551261	62.2690501	64.8456011	78.7357739	63	68.6701505
74.3551261	61.2690501	64.8456011	78.7357739	63	68.7951505
75.3551261	61.2690501	64.8456011	77.7357739	63	69.0451505
75.3551261	61.2690501	64.8456011	77.7357739	63	69.1701505
75.3551261	61.2690501	62.8456011	77.7357739	63	69.1701505
75.3551261	62.2690501	61.8456011	77.7357739	63	69.4201505
75.3551261	62.2690501	61.8456011	77.7357739	63	69.1701505
75.3551261	58.2690501	61.8456011	77.7357739	63	68.9201505
74.3551261	56.2690501	61.8456011	75.7357739	63	68.6701505
71.3551261	56.2690501	62.8456011	74.7357739	63	68.2951505
70.3551261	54.2690501	62.8456011	74.7357739	63	67.9201505
70.3551261	56.2690501	62.8456011	76.7357739	63	68.2951505
70.3551261	56.2690501	62.8456011	76.7357739	63	68.2951505
69.3551261	56.2690501	62.8456011	76.7357739	63	68.1701505
69.3551261	56.2690501	62.8456011	76.7357739	63	68.5451505
76.3551261	59.2690501	64.8456011	81.7357739	63	70.4201505
75.3551261	55.2690501	65.8456011	81.7357739	63	69.9201505
74.3551261	55.2690501	65.8456011	82.7357739	63	69.7951505
73.3551261	55.2690501	65.8456011	82.7357739	63	69.5451505
73.3551261	54.2690501	65.8456011	82.7357739	63	69.4201505
73.3551261	54.2690501	65.8456011	82.7357739	63	69.4201505
73.3551261	54.2690501	65.8456011	77.7357739	63	69.2951505
73.3551261	57.2690501	65.8456011	77.7357739	63	69.6701505
73.3551261	57.2690501	67.8456011	72.7357739	63	69.9201505
73.3551261	57.2690501	68.8456011	71.7357739	63	70.0451505
73.3551261	57.2690501	68.8456011	71.7357739	63	69.4201505
73.3551261	58.2690501	69.8456011	73.7357739	63	69.6701505
73.3551261	60.2690501	70.8456011	77.7357739	63	70.1701505
76.3551261	60.2690501	71.8456011	80.7357739	63	70.4201505
76.3551261	60.2690501	71.8456011	81.7357739	63	70.4201505
76.3551261	60.2690501	72.8456011	81.7357739	63	70.5451505
76.3551261	60.2690501	75.8456011	81.7357739	63	70.6701505
76.3551261	60.2690501	75.8456011	76.7357739	63	70.5451505
77.3551261	60.2690501	75.8456011	76.7357739	63	70.5451505
77.3551261	62.2690501	76.8456011	76.7357739	63	71.0451505
77.3551261	63.2690501	78.8456011	69.7357739	63	71.5451505

77.3551261	63.2690501	78.8456011	68.7357739	63	71.5451505
76.3551261	63.2690501	78.8456011	67.7357739	63	71.7951505
77.3551261	63.2690501	78.8456011	67.7357739	63	71.9201505
78.3551261	64.2690501	77.8456011	67.7357739	63	72.0451505
79.3551261	67.2690501	77.8456011	67.7357739	63	72.4201505
79.3551261	67.2690501	77.8456011	67.7357739	63	72.2951505
80.3551261	66.2690501	76.8456011	68.7357739	63	71.5451505
81.3551261	67.2690501	76.8456011	68.7357739	63	71.1701505
82.3551261	67.2690501	74.8456011	68.7357739	63	70.9201505
82.3551261	67.2690501	74.8456011	69.7357739	63	70.9201505
83.3551261	68.2690501	74.8456011	69.7357739	63	71.0451505
84.3551261	68.2690501	73.8456011	73.7357739	63	70.9201505
85.3551261	68.2690501	72.8456011	74.7357739	63	70.6701505
85.3551261	65.2690501	72.8456011	75.7357739	63	70.6701505
85.3551261	64.2690501	72.8456011	75.7357739	63	70.7951505
85.3551261	63.2690501	72.8456011	75.7357739	63	70.7951505
85.3551261	63.2690501	72.8456011	75.7357739	63	71.2951505
85.3551261	65.2690501	72.8456011	75.7357739	63	72.0451505
84.3551261	66.2690501	71.8456011	74.7357739	63	72.1701505
84.3551261	67.2690501	71.8456011	73.7357739	63	72.7951505
84.3551261	67.2690501	71.8456011	72.7357739	63	73.0451505
83.3551261	67.2690501	71.8456011	71.7357739	63	73.0451505
82.3551261	67.2690501	71.8456011	71.7357739	63	72.7951505
80.3551261	66.2690501	71.8456011	71.7357739	63	72.0451505
80.3551261	65.2690501	71.8456011	70.7357739	63	71.2951505
80.3551261	64.2690501	71.8456011	68.7357739	63	70.7951505
80.3551261	64.2690501	71.8456011	66.7357739	63	71.2951505
77.3551261	64.2690501	71.8456011	64.7357739	63	71.1701505
75.3551261	64.2690501	71.8456011	64.7357739	63	70.6701505
75.3551261	63.2690501	71.8456011	64.7357739	63	70.5451505
75.3551261	64.2690501	71.8456011	66.7357739	63	70.7951505
74.3551261	65.2690501	71.8456011	67.7357739	63	70.6701505
74.3551261	65.2690501	71.8456011	67.7357739	63	70.9201505
74.3551261	65.2690501	71.8456011	68.7357739	63	71.0451505
74.3551261	65.2690501	71.8456011	69.7357739	63	70.6701505
74.3551261	66.2690501	71.8456011	70.7357739	63	70.5451505
74.3551261	67.2690501	72.8456011	72.7357739	63	70.9201505
74.3551261	66.2690501	72.8456011	73.7357739	63	70.7951505
74.3551261	66.2690501	73.8456011	74.7357739	63	70.7951505
74.3551261	66.2690501	73.8456011	74.7357739	63	70.5451505
74.3551261	68.2690501	73.8456011	75.7357739	63	71.0451505
74.3551261	68.2690501	77.8456011	75.7357739	63	71.5451505
76.3551261	67.2690501	78.8456011	77.7357739	63	72.1701505
76.3551261	64.2690501	79.8456011	77.7357739	63	72.2951505
76.3551261	63.2690501	79.8456011	77.7357739	63	72.0451505

76.3551261	62.2690501	80.8456011	76.7357739	63	72.5451505
76.3551261	62.2690501	80.8456011	72.7357739	63	72.7951505
75.3551261	62.2690501	80.8456011	72.7357739	63	72.9201505
76.3551261	62.2690501	80.8456011	71.7357739	63	72.9201505
77.3551261	62.2690501	80.8456011	71.7357739	63	71.2951505
77.3551261	64.2690501	80.8456011	71.7357739	63	71.0451505
77.3551261	66.2690501	80.8456011	72.7357739	63	71.6701505
76.3551261	66.2690501	79.8456011	72.7357739	63	71.0451505
75.3551261	67.2690501	79.8456011	70.7357739	63	70.2951505
75.3551261	67.2690501	79.8456011	68.7357739	63	70.4201505
75.3551261	67.2690501	79.8456011	67.7357739	63	70.7951505
74.3551261	67.2690501	79.8456011	66.7357739	63	70.9201505
74.3551261	69.2690501	79.8456011	66.7357739	63	71.4201505
74.3551261	70.2690501	80.8456011	65.7357739	63	72.0451505
74.3551261	70.2690501	81.8456011	65.7357739	63	72.1701505
74.3551261	70.2690501	82.8456011	65.7357739	63	72.1701505
75.3551261	70.2690501	82.8456011	65.7357739	63	72.5451505
75.3551261	71.2690501	82.8456011	65.7357739	63	72.1701505
75.3551261	71.2690501	82.8456011	65.7357739	63	71.4201505
75.3551261	70.2690501	82.8456011	69.7357739	63	71.2951505
75.3551261	69.2690501	82.8456011	69.7357739	63	71.2951505
74.3551261	69.2690501	82.8456011	70.7357739	63	71.4201505
74.3551261	69.2690501	82.8456011	68.7357739	63	72.1701505
74.3551261	69.2690501	72.5663309	66.7357739	63	71.1013442
73.3551261	68.2690501	73.3441087	67.7357739	63	71.1950942
73.3551261	68.2690501	73.8996642	68.7357739	63	71.3825942
73.3551261	68.2690501	74.1218865	68.7357739	63	71.5388442
74.3551261	68.2690501	73.3441087	68.7357739	63	73.6701505
74.3551261	68.2690501	85.8456011	68.7357739	63	73.4201505
74.3551261	68.2690501	77.8456011	68.7357739	63	72.4201505
74.3551261	68.2690501	71.8456011	68.7357739	63	71.7951505
74.3551261	68.2690501	71.8456011	70.7357739	63	71.7951505
74.3551261	68.2690501	68.8456011	72.7357739	63	71.4201505
74.3551261	68.2690501	66.8456011	75.7357739	63	70.4201505
73.3551261	67.2690501	65.8456011	77.7357739	63	69.7951505
73.3551261	67.2690501	65.8456011	78.7357739	63	70.1701505
73.3551261	65.2690501	65.8456011	79.7357739	63	69.5451505
73.3551261	65.2690501	65.8456011	80.7357739	63	69.2951505
73.3551261	64.2690501	65.8456011	81.7357739	63	69.1701505
73.3551261	63.2690501	65.8456011	81.7357739	63	69.0451505
73.3551261	63.2690501	65.8456011	81.7357739	63	69.0451505
73.3551261	63.2690501	65.8456011	85.7357739	63	68.7951505
72.3551261	63.2690501	65.8456011	88.7357739	63	67.7951505
72.3551261	63.2690501	65.8456011	90.7357739	63	67.2951505
73.3551261	64.2690501	65.8456011	92.7357739	63	67.0451505

73.3551261	60.2690501	65.8456011	94.7357739	63	66.9201505
72.3551261	58.2690501	65.8456011	94.7357739	63	66.1701505
69.3551261	58.2690501	65.8456011	94.7357739	63	65.1701505
68.3551261	58.2690501	65.8456011	86.7357739	63	64.4201505
69.3551261	58.2690501	65.8456011	80.7357739	63	64.5451505
69.3551261	58.2690501	65.8456011	77.7357739	63	64.5451505
69.3551261	60.2690501	69.8456011	73.7357739	63	65.4201505
69.3551261	60.2690501	69.8456011	69.7357739	63	65.5451505
69.3551261	60.2690501	69.8456011	65.7357739	63	65.6701505
70.3551261	60.2690501	69.8456011	63.7357739	63	65.5451505
70.3551261	60.2690501	69.8456011	63.7357739	63	66.1701505
69.3551261	60.2690501	69.8456011	62.7357739	63	66.2951505
69.3551261	60.2690501	70.8456011	60.7357739	63	66.5451505
69.3551261	60.2690501	70.8456011	60.7357739	63	66.1701505
69.3551261	59.2690501	70.8456011	59.7357739	63	65.7951505
69.3551261	58.2690501	71.8456011	59.7357739	63	65.7951505
69.3551261	58.2690501	71.8456011	59.7357739	63	65.9201505
70.3551261	58.2690501	72.8456011	59.7357739	63	66.1701505
70.3551261	58.2690501	72.8456011	60.7357739	63	66.0451505
72.3551261	58.2690501	72.8456011	63.7357739	63	66.5451505
73.3551261	58.2690501	71.8456011	63.7357739	63	67.0451505
73.3551261	57.2690501	69.8456011	63.7357739	63	66.9201505
75.3551261	57.2690501	68.8456011	63.7357739	63	67.5451505
76.3551261	61.2690501	67.8456011	63.7357739	63	68.4201505
75.3551261	61.2690501	67.8456011	63.7357739	63	67.9201505
71.3551261	61.2690501	67.8456011	63.7357739	63	67.5451505
70.3551261	61.2690501	67.8456011	63.7357739	63	67.2951505
69.3551261	62.2690501	67.8456011	63.7357739	63	67.2951505
70.3551261	61.2690501	67.8456011	63.7357739	63	67.2951505
70.3551261	57.2690501	67.8456011	64.7357739	63	66.7951505
71.3551261	56.2690501	67.8456011	72.7357739	63	66.7951505
72.3551261	56.2690501	67.8456011	73.7357739	63	67.0451505
73.3551261	57.2690501	66.8456011	73.7357739	63	67.1701505
73.3551261	58.2690501	65.8456011	67.7357739	63	67.2951505
73.3551261	58.2690501	65.8456011	65.7357739	63	67.0451505
72.3551261	58.2690501	66.8456011	64.7357739	63	67.4201505
72.3551261	58.2690501	66.8456011	64.7357739	63	67.5451505
72.3551261	58.2690501	66.8456011	65.7357739	63	67.2951505
72.3551261	56.2690501	66.8456011	68.7357739	63	67.2951505
72.3551261	56.2690501	66.8456011	69.7357739	63	67.2951505
72.3551261	55.2690501	66.8456011	70.7357739	63	67.0451505
71.3551261	55.2690501	66.8456011	70.7357739	63	66.6701505
71.3551261	55.2690501	66.8456011	71.7357739	63	66.5451505
71.3551261	55.2690501	67.8456011	70.7357739	63	66.9201505
71.3551261	59.2690501	72.8456011	68.7357739	63	68.0451505

71.3551261	59.2690501	70.8456011	66.7357739	63	67.6701505
71.3551261	58.2690501	70.8456011	66.7357739	63	67.7951505
69.3551261	58.2690501	68.8456011	65.7357739	63	67.9201505
69.3551261	57.2690501	63.8456011	65.7357739	63	67.1701505
69.3551261	58.2690501	62.8456011	65.7357739	63	66.7951505
68.3551261	59.2690501	61.8456011	65.7357739	63	66.5451505
68.3551261	60.2690501	61.8456011	65.7357739	63	65.5451505
68.3551261	59.2690501	60.8456011	65.7357739	63	64.7951505
68.3551261	59.2690501	60.8456011	65.7357739	63	64.7951505
68.3551261	58.2690501	60.8456011	77.7357739	63	64.7951505
69.3551261	58.2690501	60.8456011	82.7357739	63	64.9201505
69.3551261	58.2690501	60.8456011	83.7357739	63	65.0451505
69.3551261	58.2690501	60.8456011	81.7357739	63	65.2951505
69.3551261	59.2690501	60.8456011	80.7357739	63	65.4201505
69.3551261	59.2690501	60.8456011	79.7357739	63	65.4201505
72.3551261	61.2690501	60.8456011	80.7357739	63	66.4201505
72.3551261	61.2690501	60.8456011	81.7357739	63	67.0451505
73.3551261	61.2690501	61.8456011	81.7357739	63	67.4201505
75.3551261	61.2690501	62.8456011	80.7357739	63	68.1701505
75.3551261	62.2690501	63.8456011	78.7357739	63	68.2951505
75.3551261	62.2690501	63.8456011	78.7357739	63	68.2951505
75.3551261	62.2690501	63.8456011	76.7357739	63	68.5451505
75.3551261	62.2690501	64.8456011	73.7357739	63	68.9201505
74.3551261	62.2690501	64.8456011	72.7357739	63	69.1701505
74.3551261	62.2690501	64.8456011	70.7357739	63	69.0451505
74.3551261	62.2690501	64.8456011	70.7357739	63	69.1701505
74.3551261	60.2690501	64.8456011	70.7357739	63	68.9201505
74.3551261	59.2690501	64.8456011	70.7357739	63	68.9201505
74.3551261	59.2690501	63.8456011	71.7357739	63	68.6701505
74.3551261	59.2690501	63.8456011	73.7357739	63	68.6701505
74.3551261	60.2690501	63.8456011	73.7357739	63	68.6701505
73.3551261	61.2690501	63.8456011	73.7357739	63	68.6701505
72.3551261	63.2690501	63.8456011	73.7357739	63	68.9201505
70.3551261	65.2690501	63.8456011	72.7357739	63	69.2951505
69.3551261	66.2690501	63.8456011	71.7357739	63	69.5451505
68.3551261	65.2690501	62.8456011	70.7357739	63	69.0451505
68.3551261	64.2690501	61.8456011	70.7357739	63	69.0451505
69.3551261	63.2690501	60.8456011	71.7357739	63	69.0451505
69.3551261	62.2690501	61.8456011	71.7357739	63	69.6701505
70.3551261	61.2690501	62.8456011	72.7357739	63	69.6701505
71.3551261	60.2690501	62.8456011	72.7357739	63	69.9201505
74.3551261	59.2690501	62.8456011	72.7357739	63	70.1701505
75.3551261	60.2690501	62.8456011	72.7357739	63	70.1701505
75.3551261	61.2690501	62.8456011	73.7357739	63	70.1701505
75.3551261	60.2690501	62.8456011	74.7357739	63	69.9201505

75.3551261	60.2690501	63.8456011	74.7357739	63	70.6701505
75.3551261	60.2690501	64.8456011	73.7357739	63	70.7951505
73.3551261	58.2690501	64.8456011	72.7357739	63	70.2951505
74.3551261	58.2690501	64.8456011	72.7357739	63	69.5451505
73.3551261	58.2690501	64.8456011	72.7357739	63	69.2951505
73.3551261	58.2690501	64.8456011	71.7357739	63	69.6701505
74.3551261	58.2690501	64.8456011	70.7357739	63	69.9201505
74.3551261	57.2690501	64.8456011	70.7357739	63	69.7951505
74.3551261	55.2690501	64.8456011	70.7357739	63	69.2951505
74.3551261	53.2690501	64.8456011	70.7357739	63	66.8625084
74.3551261	53.2690501	64.8456011	76.7357739	63	68.2951505
74.3551261	51.2690501	68.8456011	79.7357739	63	67.9201505
74.3551261	50.2690501	71.8456011	80.7357739	63	68.1701505
73.3551261	49.2690501	73.8456011	80.7357739	63	68.5451505
73.3551261	49.2690501	75.8456011	80.7357739	63	68.7951505
73.3551261	48.2690501	75.8456011	80.7357739	63	69.1701505
73.3551261	48.2690501	75.8456011	80.7357739	63	69.1701505
73.3551261	48.2690501	74.8456011	78.7357739	63	68.5451505
73.3551261	47.2690501	73.8456011	77.7357739	63	68.2951505
73.3551261	47.2690501	71.8456011	75.7357739	63	68.0451505
72.3551261	47.2690501	71.8456011	73.7357739	63	67.7951505
72.3551261	46.2690501	71.8456011	67.7357739	63	67.7951505
72.3551261	46.2690501	71.8456011	65.7357739	63	67.7951505
72.3551261	46.2690501	71.8456011	64.7357739	63	67.7951505
71.3551261	46.2690501	71.8456011	64.7357739	63	68.1701505
72.3551261	47.2690501	68.8456011	65.7357739	63	68.2951505
72.3551261	49.2690501	68.8456011	65.7357739	63	68.6701505
72.3551261	50.2690501	67.8456011	66.7357739	63	68.7951505
72.3551261	49.2690501	67.8456011	67.7357739	63	69.2951505
72.3551261	48.2690501	66.8456011	70.7357739	63	69.2951505
74.3551261	48.2690501	66.8456011	73.7357739	63	69.9201505
75.3551261	48.2690501	66.8456011	74.7357739	63	70.7951505
76.3551261	50.2690501	65.8456011	71.7357739	63	70.5451505
76.3551261	52.2690501	65.8456011	69.7357739	63	68.7708549
78.3551261	53.2690501	65.8456011	69.7357739	63	68.7864799
78.3551261	53.2690501	65.8456011	68.7357739	63	68.6458549
78.3551261	54.2690501	65.8456011	68.7357739	63	68.2239799
78.3551261	53.2690501	65.8456011	68.7357739	63	67.8021049
79.3551261	53.2690501	65.8456011	69.7357739	63	67.9427299
79.3551261	53.2690501	66.8456011	69.7357739	63	68.2239799
79.3551261	53.2690501	66.8456011	70.7357739	63	68.4114799
79.3551261	53.2690501	66.8456011	71.7357739	63	68.3958549
78.3551261	53.2690501	66.8456011	72.7357739	63	68.3958549
78.3551261	53.2690501	66.8456011	73.7357739	63	68.6771049
78.3551261	54.2690501	65.8456011	72.7357739	63	68.6614799

78.3551261	55.2690501	65.8456011	71.7357739	63	68.6458549
78.3551261	55.2690501	65.8456011	69.7357739	63	68.9271049
78.3551261	56.2690501	65.8456011	68.7357739	63	69.4896049
79.3551261	57.2690501	65.8456011	68.7357739	63	69.7708549
81.3551261	58.2690501	65.8456011	68.7357739	63	69.7864799
81.3551261	59.2690501	66.8456011	69.7357739	63	70.3333549
81.3551261	60.2690501	66.8456011	70.7357739	63	70.5052299
82.3551261	60.2690501	66.8456011	71.7357739	63	70.8021049
82.3551261	63.2690501	66.8456011	73.7357739	63	71.0833549
82.3551261	64.2690501	66.8456011	75.7357739	63	71.2239799
82.3551261	64.2690501	67.8456011	75.7357739	63	71.3646049
82.3551261	64.2690501	67.8456011	77.7357739	63	71.2396049
81.3551261	65.2690501	67.8456011	77.7357739	63	70.9583549
79.3551261	69.2690501	68.8456011	75.7357739	63	70.2552299
79.3551261	70.2690501	69.8456011	73.7357739	63	70.3958549
78.3551261	70.2690501	69.8456011	73.7357739	63	70.2552299
78.3551261	70.2690501	69.8456011	72.7357739	63	70.0989799
77.3551261	67.2690501	70.8456011	72.7357739	63	68.6614799
77.3551261	63.2690501	75.8456011	71.7357739	63	69.1614799
77.3551261	63.2690501	74.8456011	71.7357739	63	69.1146049
76.3551261	62.2690501	74.8456011	71.7357739	63	68.9427299
76.3551261	62.2690501	74.8456011	73.7357739	63	68.9583549
76.3551261	62.2690501	73.8456011	73.7357739	63	68.9896049
75.3551261	61.2690501	73.8456011	73.7357739	63	68.6771049
75.3551261	60.2690501	72.8456011	73.7357739	63	68.5364799
74.3551261	59.2690501	71.8456011	74.7357739	63	68.2552299
74.3551261	59.2690501	71.8456011	74.7357739	63	68.3802299
73.3551261	59.2690501	71.8456011	73.7357739	63	68.2083549
72.3551261	59.2690501	71.8456011	71.7357739	63	68.9114799
72.3551261	56.2690501	71.8456011	71.7357739	63	68.6458549
72.3551261	54.2690501	71.8456011	71.7357739	63	68.8177299
72.3551261	53.2690501	71.8456011	71.7357739	63	68.9583549
72.3551261	53.2690501	71.8456011	71.7357739	63	68.9583549
72.3551261	51.2690501	71.8456011	69.7357739	63	68.8177299
72.3551261	51.2690501	71.8456011	69.7357739	63	68.5677299
72.3551261	52.2690501	71.8456011	69.7357739	63	68.8646049
73.3551261	52.2690501	71.8456011	70.7357739	63	69.1458549
73.3551261	52.2690501	71.8456011	71.7357739	63	69.2864799
73.3551261	52.2690501	71.8456011	71.7357739	63	69.0364799
75.3551261	51.2690501	69.8456011	70.7357739	63	68.9271049
74.3551261	51.2690501	69.8456011	69.7357739	63	68.4739799
72.3551261	52.2690501	69.8456011	69.7357739	63	68.4739799
73.3551261	52.2690501	69.8456011	67.7357739	63	68.7396049
73.3551261	52.2690501	69.8456011	67.7357739	63	68.7239799
73.3551261	53.2690501	68.8456011	67.7357739	63	68.5989799

73.3551261	53.2690501	68.8456011	68.7357739	63	68.4583549
73.3551261	54.2690501	68.8456011	68.7357739	63	68.3333549
73.3551261	54.2690501	68.8456011	68.7357739	63	68.1146049
73.3551261	53.2690501	68.8456011	70.7357739	63	68.1302299
73.3551261	53.2690501	68.8456011	70.7357739	63	68.2708549
72.3551261	52.2690501	68.8456011	70.7357739	63	67.9896049
72.3551261	52.2690501	67.8456011	70.7357739	63	67.8646049
71.3551261	52.2690501	67.8456011	69.7357739	63	67.8489799
70.3551261	53.2690501	68.8456011	70.7357739	63	67.2708549
70.3551261	55.2690501	68.8456011	70.7357739	63	67.4427299
70.3551261	61.2690501	68.8456011	70.7357739	63	68.1771049
69.3551261	69.2690501	68.8456011	70.7357739	63	71.2951505
72.3551261	74.2690501	68.8456011	70.7357739	63	71.6701505
72.3551261	78.2690501	67.8456011	70.7357739	63	71.5451505
72.3551261	79.2690501	67.8456011	70.7357739	63	71.6701505
72.3551261	79.2690501	67.8456011	70.7357739	63	71.4201505
72.3551261	78.2690501	67.8456011	70.7357739	63	71.0451505
73.3551261	75.2690501	66.8456011	71.7357739	63	70.6701505
73.3551261	73.2690501	66.8456011	71.7357739	63	70.5451505
73.3551261	72.2690501	66.8456011	69.7357739	63	70.4201505
74.3551261	71.2690501	66.8456011	69.7357739	63	69.6701505
74.3551261	71.2690501	66.8456011	69.7357739	63	70.0451505
74.3551261	69.2690501	66.8456011	67.7357739	63	69.9201505
75.3551261	69.2690501	66.8456011	67.7357739	63	70.1701505
75.3551261	66.2690501	66.8456011	67.7357739	63	69.6701505
76.3551261	65.2690501	66.8456011	66.7357739	63	69.4201505
76.3551261	64.2690501	66.8456011	66.7357739	63	69.5451505
76.3551261	65.2690501	66.8456011	66.7357739	63	69.2951505
76.3551261	65.2690501	66.8456011	66.7357739	63	69.2951505
76.3551261	65.2690501	66.8456011	66.7357739	63	69.6701505
76.3551261	66.2690501	66.8456011	66.7357739	63	70.0451505
76.3551261	66.2690501	67.8456011	69.7357739	63	70.6701505
76.3551261	66.2690501	70.8456011	70.7357739	63	70.7951505
76.3551261	66.2690501	70.8456011	70.7357739	63	70.6701505
78.3551261	68.2690501	70.8456011	64.7357739	63	70.9201505
78.3551261	69.2690501	70.8456011	61.7357739	63	71.2951505
78.3551261	70.2690501	70.8456011	59.7357739	63	71.4201505
78.3551261	70.2690501	70.8456011	59.7357739	63	71.5451505
78.3551261	70.2690501	69.8456011	58.7357739	63	71.0451505
78.3551261	71.2690501	68.8456011	58.7357739	63	71.0451505
78.3551261	71.2690501	68.8456011	58.7357739	63	71.0451505
78.3551261	71.2690501	68.8456011	58.7357739	63	71.0451505
77.3551261	64.2690501	68.8456011	59.7357739	63	70.4201505
77.3551261	64.2690501	68.8456011	61.7357739	63	70.2951505
77.3551261	62.2690501	68.8456011	63.7357739	63	69.7951505

77.3551261	62.2690501	68.8456011	64.7357739	63	69.4201505
78.3551261	61.2690501	68.8456011	64.7357739	63	68.9201505
78.3551261	61.2690501	69.8456011	62.7357739	63	69.2951505
77.3551261	61.2690501	72.8456011	62.7357739	63	68.9201505
76.3551261	61.2690501	73.8456011	64.7357739	63	69.9201505
76.3551261	56.2690501	73.8456011	64.7357739	63	69.1701505
75.3551261	56.2690501	73.8456011	65.7357739	63	69.0451505
75.3551261	57.2690501	73.8456011	65.7357739	63	69.1701505
75.3551261	57.2690501	73.8456011	66.7357739	63	68.1701505
75.3551261	55.2690501	74.8456011	69.7357739	63	67.6701505
75.3551261	55.2690501	74.8456011	69.7357739	63	67.1701505
76.3551261	56.2690501	73.8456011	69.7357739	63	67.4201505
76.3551261	57.2690501	70.8456011	69.7357739	63	67.9201505
76.3551261	57.2690501	68.8456011	69.7357739	63	67.5451505
72.3551261	59.2690501	68.8456011	69.7357739	63	67.4201505
72.3551261	59.2690501	67.8456011	72.7357739	63	67.2951505
72.3551261	58.2690501	67.8456011	77.7357739	63	67.1701505
73.3551261	57.2690501	67.8456011	79.7357739	63	66.9201505
74.3551261	56.2690501	67.8456011	78.7357739	63	67.0451505
74.3551261	55.2690501	67.8456011	77.7357739	63	67.0451505
74.3551261	54.2690501	67.8456011	76.7357739	63	66.9201505
75.3551261	54.2690501	65.8456011	74.7357739	63	67.0451505
75.3551261	56.2690501	65.8456011	74.7357739	63	67.1701505
78.3551261	58.2690501	64.8456011	74.7357739	63	67.6701505
79.3551261	59.2690501	64.8456011	74.7357739	63	67.9201505
79.3551261	59.2690501	64.8456011	73.7357739	63	67.9201505
80.3551261	61.2690501	65.8456011	71.7357739	63	68.2951505
80.3551261	61.2690501	65.8456011	70.7357739	63	67.9201505
80.3551261	60.2690501	65.8456011	70.7357739	63	67.6701505
81.3551261	60.2690501	65.8456011	70.7357739	63	67.5451505
81.3551261	59.2690501	65.8456011	70.7357739	63	67.1701505
81.3551261	59.2690501	65.8456011	69.7357739	63	67.2951505
81.3551261	59.2690501	65.8456011	68.7357739	63	67.4201505
81.3551261	59.2690501	65.8456011	67.7357739	63	67.5451505
81.3551261	59.2690501	65.8456011	67.7357739	63	67.5451505
81.3551261	59.2690501	65.8456011	66.7357739	63	67.5451505
81.3551261	58.2690501	65.8456011	66.7357739	63	67.4201505
81.3551261	58.2690501	65.8456011	66.7357739	63	67.5451505
81.3551261	59.2690501	65.8456011	66.7357739	63	68.1701505
81.3551261	61.2690501	65.8456011	67.7357739	63	68.7951505
81.3551261	61.2690501	65.8456011	66.7357739	63	68.9201505
81.3551261	65.2690501	66.8456011	66.7357739	63	69.5451505
83.3551261	66.2690501	66.8456011	66.7357739	63	70.4201505
83.3551261	66.2690501	68.8456011	66.7357739	63	71.2951505
83.3551261	67.2690501	68.8456011	66.7357739	63	71.2951505

83.3551261	67.2690501	67.8456011	65.7357739	63	70.9201505
83.3551261	67.2690501	66.8456011	65.7357739	63	70.6701505
83.3551261	69.2690501	66.8456011	66.7357739	63	71.1701505
83.3551261	69.2690501	66.8456011	66.7357739	63	71.0451505
84.3551261	69.2690501	66.8456011	66.7357739	63	71.1701505
84.3551261	69.2690501	66.8456011	66.7357739	63	71.1701505
82.3551261	72.2690501	65.8456011	70.7357739	63	70.9201505
82.3551261	72.2690501	64.8456011	70.7357739	63	70.7951505
82.3551261	68.2690501	63.8456011	73.7357739	63	70.4201505
82.3551261	67.2690501	62.8456011	72.7357739	63	70.5451505
81.3551261	67.2690501	62.8456011	70.7357739	63	71.1701505
81.3551261	70.2690501	62.8456011	70.7357739	63	71.4201505
81.3551261	70.2690501	62.8456011	70.7357739	63	71.5451505
80.3551261	70.2690501	63.8456011	70.7357739	63	71.4201505
80.3551261	67.2690501	63.8456011	70.7357739	63	70.7951505
81.3551261	64.2690501	64.8456011	70.7357739	63	70.6701505
81.3551261	64.2690501	64.8456011	70.7357739	63	70.2951505
81.3551261	61.2690501	64.8456011	71.7357739	63	70.0451505
81.3551261	60.2690501	64.8456011	71.7357739	63	70.1701505
82.3551261	60.2690501	64.8456011	70.7357739	63	70.2951505
83.3551261	58.2690501	63.8456011	74.7357739	63	69.9201505
83.3551261	58.2690501	63.8456011	75.7357739	63	69.7951505
85.3551261	58.2690501	63.8456011	74.7357739	63	70.1701505
86.3551261	57.2690501	64.8456011	74.7357739	63	70.2951505
70.2951505	57.2690501	64.8456011	66.7357739	63	68.1626536
70.9201505	63.2690501	64.8456011	66.7357739	63	68.8657786
87.3551261	63.2690501	64.8456011	63.7357739	63	71.2951505
71.5451505	63.2690501	64.8456011	62.7357739	63	69.4439036
71.5451505	62.2690501	64.8456011	61.7357739	63	69.4439036
71.1701505	60.2690501	64.8456011	60.7357739	63	68.8970286
70.9201505	58.2690501	64.8456011	60.7357739	63	68.6157786
71.1701505	59.2690501	63.8456011	60.7357739	63	68.7720286
70.9201505	61.2690501	63.8456011	62.7357739	63	68.6157786
70.5451505	61.2690501	63.8456011	62.7357739	63	68.1939036
70.7951505	63.2690501	62.8456011	66.7357739	63	68.2251536
70.7951505	64.2690501	62.8456011	69.7357739	63	68.2251536
70.7951505	64.2690501	62.8456011	69.7357739	63	68.2251536
70.5451505	62.2690501	62.8456011	74.7357739	63	67.9439036
70.2951505	60.2690501	65.8456011	75.7357739	63	68.0376536
70.4201505	59.2690501	65.8456011	76.7357739	63	68.0532786
70.4201505	58.2690501	64.8456011	76.7357739	63	67.9282786
70.1701505	57.2690501	63.8456011	76.7357739	63	67.7720286
69.9201505	56.2690501	63.8456011	76.7357739	63	67.6157786
69.9201505	56.2690501	63.8456011	76.7357739	63	67.6157786
70.2951505	56.2690501	63.8456011	77.7357739	63	68.1626536

70.5451505	56.2690501	63.8456011	77.7357739	63	68.4439036
70.5451505	56.2690501	63.8456011	77.7357739	63	68.4439036
70.5451505	56.2690501	64.8456011	77.7357739	63	68.5689036
70.6701505	56.2690501	66.8456011	79.7357739	63	68.7095286
70.2951505	54.2690501	66.8456011	79.7357739	63	68.0376536
70.2951505	52.2690501	66.8456011	78.7357739	63	67.9126536
70.4201505	52.2690501	65.8456011	76.7357739	63	67.9282786
70.5451505	53.2690501	65.8456011	74.7357739	63	68.0689036
70.6701505	53.2690501	65.8456011	73.7357739	63	68.2095286
70.5451505	53.2690501	66.8456011	72.7357739	63	68.3189036
70.5451505	54.2690501	66.8456011	72.7357739	63	68.1939036
70.6701505	55.2690501	66.8456011	72.7357739	63	68.3345286
71.1701505	56.2690501	66.8456011	72.7357739	63	68.8970286
71.4201505	58.2690501	66.8456011	72.7357739	63	69.1782786
71.4201505	58.2690501	66.8456011	72.7357739	63	69.1782786
72.2951505	59.2690501	66.8456011	71.7357739	63	70.0376536
71.6701505	56.2690501	66.8456011	71.7357739	63	69.4595286
71.4201505	54.2690501	66.8456011	70.7357739	63	69.3032786
71.1701505	53.2690501	66.8456011	69.7357739	63	69.0220286
70.9201505	54.2690501	66.8456011	69.7357739	63	68.6157786
86.3551261	56.2690501	67.8456011	69.7357739	63	70.7951505
86.3551261	56.2690501	67.8456011	69.7357739	63	70.5451505
83.3551261	58.2690501	67.8456011	69.7357739	63	70.1701505
82.3551261	59.2690501	68.8456011	69.7357739	63	70.5451505
82.3551261	58.2690501	68.8456011	69.7357739	63	70.4201505
82.3551261	57.2690501	66.8456011	69.7357739	63	70.0451505
82.3551261	56.2690501	66.8456011	70.7357739	63	70.1701505
83.3551261	55.2690501	65.8456011	70.7357739	63	70.1701505
84.3551261	55.2690501	63.8456011	71.7357739	63	70.0451505
84.3551261	56.2690501	61.8456011	72.7357739	63	70.0451505
84.3551261	57.2690501	61.8456011	71.7357739	63	70.1701505
84.3551261	60.2690501	61.8456011	70.7357739	63	70.5451505
84.3551261	61.2690501	61.8456011	70.7357739	63	70.7951505
85.3551261	62.2690501	60.8456011	72.7357739	63	71.0451505
86.3551261	62.2690501	60.8456011	75.7357739	63	71.2951505
86.3551261	61.2690501	61.8456011	76.7357739	63	71.2951505
86.3551261	61.2690501	61.8456011	77.7357739	63	70.9201505
86.3551261	58.2690501	61.8456011	77.7357739	63	70.4201505
69.9201505	58.2690501	61.8456011	79.7357739	63	67.8657786
70.0451505	58.2690501	64.8456011	78.7357739	63	68.1314036
70.1701505	60.2690501	64.8456011	78.7357739	63	67.8970286
70.9201505	62.2690501	65.8456011	78.7357739	63	68.8657786
72.1701505	64.2690501	65.8456011	78.7357739	63	69.6470286
72.0451505	65.2690501	65.8456011	78.7357739	69	69.7564036
87.3551261	66.2690501	65.8456011	78.7357739	69	71.7951505

83.3551261	66.2690501	65.8456011	77.7357739	69	70.6701505
81.3551261	67.2690501	67.8456011	77.7357739	69	70.7951505
78.3551261	67.2690501	67.8456011	79.7357739	69	69.9201505
77.3551261	66.2690501	67.8456011	80.7357739	69	69.5451505
77.3551261	59.2690501	66.8456011	82.7357739	69	68.2951505
77.3551261	57.2690501	66.8456011	82.7357739	69	67.9201505
78.3551261	57.2690501	66.8456011	84.7357739	69	68.0451505
79.3551261	57.2690501	66.8456011	84.7357739	69	68.0451505
80.3551261	58.2690501	66.8456011	82.7357739	69	68.6701505
80.3551261	58.2690501	66.8456011	81.7357739	69	68.9201505
80.3551261	58.2690501	66.8456011	81.7357739	69	69.7951505
80.3551261	58.2690501	66.8456011	81.7357739	69	70.0451505
80.3551261	56.2690501	65.8456011	82.7357739	69	69.9201505
80.3551261	55.2690501	65.8456011	82.7357739	69	71.2951505
80.3551261	54.2690501	65.8456011	83.7357739	69	71.0451505
79.3551261	54.2690501	64.8456011	83.7357739	69	70.7951505
79.3551261	54.2690501	64.8456011	83.7357739	69	70.7951505
79.3551261	53.2690501	64.8456011	83.7357739	69	70.5451505
79.3551261	53.2690501	64.8456011	83.7357739	69	69.7951505
78.3551261	53.2690501	64.8456011	84.7357739	69	69.1701505
78.3551261	53.2690501	64.8456011	84.7357739	69	68.6701505
78.3551261	56.2690501	64.8456011	84.7357739	69	69.0451505
78.3551261	56.2690501	64.8456011	85.7357739	69	68.9201505
78.3551261	56.2690501	64.8456011	84.7357739	69	68.9201505
78.3551261	57.2690501	64.8456011	84.7357739	69	69.1701505
78.3551261	59.2690501	64.8456011	84.7357739	69	68.2951505
78.3551261	59.2690501	64.8456011	84.7357739	69	68.0451505
78.3551261	59.2690501	65.8456011	84.7357739	69	68.1701505
80.3551261	60.2690501	67.8456011	83.7357739	63	69.6701505
70.6701505	60.2690501	68.8456011	83.7357739	63	68.7095286
71.0451505	60.2690501	69.8456011	82.7357739	63	68.8814036
70.4201505	57.2690501	69.8456011	82.7357739	63	68.0532786
69.6701505	55.2690501	68.8456011	82.7357739	63	67.0845286
69.4201505	55.2690501	68.8456011	82.7357739	63	66.9282786
68.9201505	54.2690501	68.8456011	82.7357739	63	66.3657786
68.6701505	54.2690501	68.8456011	83.7357739	63	66.0845286
68.9201505	54.2690501	68.8456011	83.7357739	63	66.3657786
68.9201505	54.2690501	68.8456011	82.7357739	63	66.2407786
69.0451505	54.2690501	68.8456011	81.7357739	63	66.3814036
68.9201505	54.2690501	68.8456011	79.7357739	63	66.2407786
68.7951505	54.2690501	70.8456011	79.7357739	63	66.3501536
69.1701505	53.2690501	75.8456011	78.7357739	63	67.0220286
70.0451505	53.2690501	76.8456011	77.7357739	63	67.5064036
70.7951505	54.2690501	72.8456011	77.7357739	63	67.8501536
87.3551261	55.2690501	70.8456011	74.7357739	63	71.1701505

86.3551261	56.2690501	67.8456011	74.7357739	63	71.9201505
85.3551261	56.2690501	66.8456011	75.7357739	63	71.6701505
85.3551261	56.2690501	65.8456011	75.7357739	63	71.1701505
84.3551261	55.2690501	65.8456011	73.7357739	63	68.7951505
84.3551261	55.2690501	64.8456011	72.7357739	63	68.1701505
83.3551261	55.2690501	62.8456011	71.7357739	63	67.5451505
82.3551261	55.2690501	62.8456011	70.7357739	63	67.4201505
81.3551261	54.2690501	62.8456011	69.7357739	63	67.2951505
81.3551261	54.2690501	62.8456011	69.7357739	63	67.1701505
79.3551261	55.2690501	61.8456011	69.7357739	63	67.1701505
79.3551261	56.2690501	61.8456011	71.7357739	63	67.2951505
79.3551261	57.2690501	61.8456011	72.7357739	63	67.6701505
79.3551261	57.2690501	61.8456011	72.7357739	63	67.9201505
78.3551261	57.2690501	61.8456011	72.7357739	63	67.7951505
77.3551261	57.2690501	61.8456011	74.7357739	63	67.5451505
77.3551261	57.2690501	61.8456011	75.7357739	63	67.2951505
76.3551261	58.2690501	61.8456011	75.7357739	63	67.2951505
76.3551261	58.2690501	61.8456011	77.7357739	63	67.1701505
76.3551261	59.2690501	61.8456011	79.7357739	63	67.2951505
74.3551261	57.2690501	61.8456011	82.7357739	63	66.9201505
74.3551261	56.2690501	61.8456011	84.7357739	63	65.5451505
74.3551261	55.2690501	61.8456011	85.7357739	63	65.4201505
74.3551261	55.2690501	61.8456011	84.7357739	63	64.9201505
75.3551261	55.2690501	61.8456011	83.7357739	63	65.2951505
74.3551261	55.2690501	61.8456011	81.7357739	63	65.4201505
74.3551261	55.2690501	61.8456011	80.7357739	63	65.6701505
74.3551261	55.2690501	61.8456011	80.7357739	63	65.7951505
74.3551261	55.2690501	61.8456011	80.7357739	63	66.1701505
72.3551261	55.2690501	62.8456011	81.7357739	63	66.2951505
72.3551261	54.2690501	62.8456011	84.7357739	63	67.0451505
72.3551261	54.2690501	63.8456011	84.7357739	63	67.5451505
72.3551261	53.2690501	63.8456011	85.7357739	63	65.240571
72.3551261	53.2690501	64.8456011	85.7357739	63	65.365571
72.3551261	60.2690501	64.8456011	84.7357739	63	67.4201505
72.3551261	61.2690501	65.8456011	81.7357739	63	67.7951505
72.3551261	61.2690501	65.8456011	75.7357739	63	67.9201505
72.3551261	62.2690501	65.8456011	68.7357739	63	67.4201505
72.3551261	62.2690501	69.8456011	66.7357739	63	67.7951505
73.3551261	61.2690501	71.8456011	65.7357739	63	68.4201505
73.3551261	60.2690501	72.8456011	65.7357739	63	68.5451505
73.3551261	59.2690501	72.8456011	66.7357739	63	68.2951505
73.3551261	58.2690501	72.8456011	66.7357739	63	68.1701505
72.3551261	58.2690501	71.8456011	67.7357739	63	67.5451505
71.3551261	57.2690501	71.8456011	67.7357739	63	67.2951505
69.3551261	56.2690501	74.8456011	68.7357739	63	67.4201505

68.3551261	56.2690501	76.8456011	67.7357739	63	67.2951505
68.3551261	57.2690501	76.8456011	67.7357739	63	67.5451505
68.3551261	58.2690501	76.8456011	68.7357739	63	67.7951505
68.3551261	59.2690501	76.8456011	71.7357739	63	68.0451505
57.3551261	59.2690501	77.8456011	73.7357739	63	67.0451505
57.3551261	59.2690501	77.8456011	75.7357739	63	67.1701505
56.3551261	59.2690501	78.8456011	75.7357739	63	67.0451505
56.3551261	59.2690501	79.8456011	74.7357739	63	67.0451505
56.3551261	59.2690501	80.8456011	73.7357739	63	67.1701505
55.3551261	59.2690501	80.8456011	73.7357739	63	66.4201505
55.3551261	59.2690501	81.8456011	74.7357739	63	66.2951505
56.3551261	59.2690501	81.8456011	74.7357739	63	66.1701505
58.3551261	61.2690501	81.8456011	75.7357739	63	66.6701505
60.3551261	62.2690501	81.8456011	75.7357739	63	67.4201505
60.3551261	64.2690501	80.8456011	75.7357739	63	67.9201505
61.3551261	65.2690501	80.8456011	75.7357739	63	68.0451505
62.3551261	65.2690501	80.8456011	74.7357739	63	68.2951505
62.3551261	65.2690501	81.8456011	73.7357739	63	68.6701505
62.3551261	65.2690501	81.8456011	72.7357739	63	68.9201505
63.3551261	64.2690501	70.2329976	73.7357739	63	67.7888442
63.3551261	62.2690501	70.3441087	74.7357739	63	67.4138442
64.3551261	61.2690501	71.0107753	77.7357739	63	67.3357192
66.3551261	61.2690501	71.3441087	78.7357739	63	67.7419692
66.3551261	61.2690501	72.2329976	78.7357739	63	68.1169692
72.3551261	62.2690501	73.0107753	78.7357739	63	69.2263442
73.3551261	63.2690501	73.0107753	78.7357739	63	69.7263442
74.3551261	64.2690501	72.677442	78.7357739	63	69.5544692
74.3551261	64.2690501	72.7885531	78.7357739	63	69.1950942
74.3551261	63.2690501	72.677442	78.7357739	63	69.4294692
74.3551261	62.2690501	72.4552198	78.7357739	63	69.2732192
74.3551261	61.2690501	72.3441087	78.7357739	63	69.0075942
75.3551261	61.2690501	72.0107753	77.7357739	63	68.8513442
74.3551261	61.2690501	72.3441087	77.7357739	63	68.6482192
74.3551261	62.2690501	71.8996642	72.7357739	63	68.9138442
74.3551261	64.2690501	71.4552198	72.7357739	63	68.9763442
73.3551261	64.2690501	81.8456011	72.7357739	63	69.7951505
73.3551261	64.2690501	79.8456011	71.7357739	63	68.9201505
74.3551261	63.2690501	78.8456011	71.7357739	63	68.9201505
74.3551261	59.2690501	75.8456011	72.7357739	63	67.9201505
74.3551261	57.2690501	74.8456011	73.7357739	63	67.5451505
74.3551261	56.2690501	74.8456011	79.7357739	63	67.7951505
74.3551261	56.2690501	73.8456011	80.7357739	63	67.6701505
74.3551261	57.2690501	72.8456011	80.7357739	63	68.5451505
74.3551261	57.2690501	72.8456011	71.7357739	63	68.6701505
74.3551261	57.2690501	72.8456011	68.7357739	63	68.5451505

74.3551261	58.2690501	72.8456011	67.7357739	63	68.4201505
74.3551261	59.2690501	72.8456011	66.7357739	63	68.0451505
74.3551261	59.2690501	72.8456011	64.7357739	63	67.9201505
73.3551261	59.2690501	72.8456011	62.7357739	63	67.7951505
73.3551261	59.2690501	72.8456011	62.7357739	63	67.9201505
73.3551261	59.2690501	73.8456011	61.7357739	63	68.6701505
74.3551261	58.2690501	73.8456011	61.7357739	63	68.7951505
74.3551261	57.2690501	76.8456011	61.7357739	63	69.1701505
74.3551261	56.2690501	76.8456011	60.7357739	63	68.9201505
76.3551261	55.2690501	69.0107753	60.7357739	63	67.5700942
76.3551261	55.2690501	69.4552198	59.7357739	63	68.0232192
76.3551261	56.2690501	69.7885531	59.7357739	63	68.5700942
76.3551261	57.2690501	70.7885531	59.7357739	63	68.8357192
76.3551261	58.2690501	72.4552198	59.7357739	63	69.8200942
76.3551261	59.2690501	72.8996642	59.7357739	63	70.1325942
77.3551261	60.2690501	73.4552198	60.7357739	63	70.6950942
77.3551261	60.2690501	73.4552198	60.7357739	63	71.0700942
78.3551261	60.2690501	73.677442	61.7357739	63	71.0857192
78.3551261	59.2690501	73.677442	61.7357739	63	71.0857192
78.3551261	58.2690501	73.677442	61.7357739	63	70.8357192
78.3551261	58.2690501	74.7885531	61.7357739		70.8669692
78.3551261	59.2690501	74.3441087	61.7357739		71.9294692
77.3551261	60.2690501	74.5663309	61.7357739		71.3357192
76.3551261	63.2690501	76.3441087	68.7357739		71.6013442
76.3551261	66.2690501	73.7885531	68.7357739		72.1169692
76.3551261	67.2690501	81.8456011	69.7357739		72.6701505
75.3551261	67.2690501	81.8456011	69.7357739		72.5451505
75.3551261	66.2690501	74.8456011	69.7357739		71.4201505
75.3551261	62.2690501	73.8456011	69.7357739		70.7951505
75.3551261	60.2690501	72.8456011	70.7357739		70.2951505
75.3551261	60.2690501	71.8456011	71.7357739		70.1701505
75.3551261	60.2690501	70.8456011	70.7357739		70.1701505
76.3551261	60.2690501	69.8456011	69.7357739		70.1701505
77.3551261	60.2690501	69.8456011	68.7357739		70.9201505
79.3551261	60.2690501	69.8456011	69.7357739		71.1701505
78.3551261	59.2690501	69.8456011	71.7357739		71.0451505
78.3551261	59.2690501	69.8456011	72.7357739		71.0451505
78.3551261	61.2690501	69.8456011	72.7357739		71.2951505
77.3551261	61.2690501	71.8456011	72.7357739		71.1701505
77.3551261	63.2690501	71.8456011	71.7357739		71.6701505
77.3551261	64.2690501	72.8456011	69.7357739		72.0451505
76.3551261	64.2690501	75.8456011	68.7357739		72.4201505
76.3551261	65.2690501	76.8456011	68.7357739		72.9201505
76.3551261	66.2690501	78.8456011	68.7357739		72.9201505
75.3551261	66.2690501	82.8456011	69.7357739		72.9201505

75.3551261	66.2690501	82.8456011	69.7357739	72.5451505
75.3551261	68.2690501	85.8456011	72.7357739	72.7951505
75.3551261	68.2690501	85.8456011	72.7357739	72.7951505
75.3551261	67.2690501	85.8456011	73.7357739	72.6701505
75.3551261	65.2690501	85.8456011	75.7357739	72.6701505
75.3551261	65.2690501	86.8456011	78.7357739	72.7951505
75.3551261	63.2690501	73.1218865	80.7357739	70.5857192
75.3551261	60.2690501	72.677442	82.7357739	69.8669692
74.3551261	57.2690501	71.8996642	82.7357739	68.8825942
73.3551261	55.2690501	71.5663309	84.7357739	67.9294692
73.3551261	54.2690501	71.677442	84.7357739	67.3200942
74.3551261	53.2690501	71.4552198	83.7357739	67.3044692
74.3551261	52.2690501	84.8456011	81.7357739	69.0451505
74.3551261	51.2690501	76.8456011	78.7357739	67.9201505
75.3551261	51.2690501	74.8456011	78.7357739	67.6701505
75.3551261	51.2690501	74.8456011	77.7357739	67.6701505
76.3551261	52.2690501	74.8456011	77.7357739	67.9201505
75.3551261	52.2690501	73.8456011	77.7357739	67.6701505
75.3551261	52.2690501	73.8456011	77.7357739	67.5451505
75.3551261	52.2690501	73.8456011	76.7357739	67.1701505
75.3551261	52.2690501	72.8456011	76.7357739	67.0451505
75.3551261	51.2690501	71.8456011	76.7357739	66.5451505
75.3551261	51.2690501	71.8456011	77.7357739	66.7951505
75.3551261	51.2690501	70.8456011	77.7357739	67.0451505
76.3551261	51.2690501	69.8456011	78.7357739	67.4201505
77.3551261	51.2690501	69.8456011	78.7357739	67.0451505
77.3551261	51.2690501	69.8456011	79.7357739	67.1701505
77.3551261	51.2690501	68.8456011	79.7357739	67.2951505
77.3551261	51.2690501	68.8456011	81.7357739	67.2951505
77.3551261	51.2690501	67.8456011	79.7357739	67.2951505
77.3551261	50.2690501	67.8456011	79.7357739	67.5451505
77.3551261	49.2690501	66.8456011	78.7357739	67.1701505
77.3551261	50.2690501	65.8456011	78.7357739	67.2951505
77.3551261	50.2690501	65.8456011	78.7357739	67.6701505
78.3551261	50.2690501	65.8456011	76.7357739	68.0451505
78.3551261	51.2690501	65.8456011	76.7357739	68.4201505
78.3551261	52.2690501	65.8456011	76.7357739	68.6701505
78.3551261	56.2690501	65.8456011	76.7357739	68.9201505
78.3551261	59.2690501	65.8456011	76.7357739	69.6701505
77.3551261	59.2690501	65.8456011	76.7357739	70.0451505
77.3551261	59.2690501	65.8456011	73.7357739	70.6701505
77.3551261	58.2690501	71.8456011	72.7357739	71.9201505
77.3551261	58.2690501	71.8456011	72.7357739	70.7218834
77.3551261	57.2690501	71.8456011	70.7357739	70.4406334
77.3551261	57.2690501	71.8456011	70.7357739	70.5812584

80.3551261	58.2690501	70.8456011	69.7357739	73.2951505
80.3551261	61.2690501	70.8456011	69.7357739	74.0451505
79.3551261	61.2690501	68.8456011	69.7357739	73.6701505
78.3551261	61.2690501	67.8456011	69.7357739	73.1701505
78.3551261	61.2690501	67.8456011	69.7357739	73.2951505
80.3551261	61.2690501	66.8456011	69.7357739	73.9201505
80.3551261	62.2690501	66.8456011	69.7357739	74.1701505
79.3551261	62.2690501	66.8456011	68.7357739	74.2951505
79.3551261	61.2690501	66.8456011	68.7357739	74.1701505
78.3551261	57.2690501	66.8456011	68.7357739	73.4201505
78.3551261	57.2690501	66.8456011	68.7357739	73.2951505
77.3551261	56.2690501	66.8456011	68.7357739	73.2951505
77.3551261	56.2690501	66.8456011	68.7357739	73.4201505
77.3551261	56.2690501	66.8456011	68.7357739	73.4201505
77.3551261	56.2690501	66.8456011	68.7357739	73.4201505
76.3551261	61.2690501	67.8456011	68.7357739	74.1701505
76.3551261	65.2690501	67.8456011	67.7357739	74.7951505
76.3551261	65.2690501	71.8456011	67.7357739	74.6701505
76.3551261	64.2690501	71.8456011	68.7357739	74.1701505
75.3551261	62.2690501	71.8456011	69.7357739	74.1701505
75.3551261	60.2690501	70.8456011	69.7357739	74.0451505
75.3551261	58.2690501	70.8456011	68.7357739	73.9201505
75.3551261	56.2690501	70.8456011	67.7357739	73.4201505
75.3551261	56.2690501	71.8456011	67.7357739	73.4201505
76.3551261	56.2690501	71.8456011	66.7357739	73.4201505
76.3551261	57.2690501	71.8456011	66.7357739	72.9201505
76.3551261	57.2690501	70.8456011	66.7357739	72.1701505
76.3551261	57.2690501	70.8456011	66.7357739	71.1701505
77.3551261	56.2690501	70.8456011	66.7357739	70.6701505
77.3551261	55.2690501	70.8456011	66.7357739	70.4201505
76.3551261	54.2690501	70.8456011	66.7357739	70.5451505
76.3551261	54.2690501	70.8456011	65.7357739	70.6701505
76.3551261	54.2690501	69.8456011	65.7357739	70.7951505
76.3551261	54.2690501	69.8456011	66.7357739	68.959321
76.3551261	55.2690501	69.8456011	66.7357739	68.834321
76.3551261	55.2690501	69.8456011	66.7357739	68.849946
76.3551261	57.2690501	69.8456011	65.7357739	69.146821
75.3551261	56.2690501	69.8456011	65.7357739	68.303071
75.3551261	56.2690501	69.8456011	66.7357739	68.303071
74.3551261	56.2690501	69.8456011	66.7357739	68.178071
74.3551261	54.2690501	70.8456011	66.7357739	68.474946
74.3551261	55.2690501	71.8456011	66.7357739	68.896821
73.3551261	58.2690501	71.8456011	67.7357739	68.756196
73.3551261	61.2690501	71.8456011	67.7357739	68.756196
73.3551261	64.2690501	71.8456011	68.7357739	69.053071

73.3551261	65.2690501	71.8456011	68.7357739	69.084321
74.3551261	64.2690501	71.8456011	70.7357739	68.553071
74.3551261	62.2690501	71.8456011	71.7357739	67.599946
74.3551261	60.2690501	71.8456011	70.7357739	66.912446
74.3551261	59.2690501	72.8456011	70.7357739	66.193696
73.3551261	59.2690501	74.8456011	70.7357739	65.615571
74.3551261	58.2690501	73.8456011	70.7357739	65.646821
74.3551261	55.2690501	73.8456011	70.7357739	64.912446
74.3551261	55.2690501	70.8456011	70.7357739	64.396821
74.3551261	55.2690501	67.8456011	69.7357739	63.631196
74.3551261	56.2690501	66.8456011	69.7357739	63.318696
74.3551261	57.2690501	65.8456011	69.7357739	63.162446
73.3551261	57.2690501	64.8456011	72.7357739	62.990571
73.3551261	57.2690501	64.8456011	72.7357739	63.256196
75.3551261	57.2690501	64.8456011	79.7357739	64.209321
75.3551261	58.2690501	64.8456011	81.7357739	64.771821
75.3551261	58.2690501	64.8456011	81.7357739	66.5451505
75.3551261	58.2690501	64.8456011	80.7357739	65.6701505
77.3551261	59.2690501	64.8456011	73.7357739	65.0451505
78.3551261	59.2690501	64.8456011	69.7357739	64.7951505
78.3551261	60.2690501	65.8456011	67.7357739	65.4201505
78.3551261	59.2690501	67.8456011	65.7357739	64.365571
78.3551261	59.2690501	67.8456011	64.7357739	64.553071
78.3551261	59.2690501	66.8456011	63.7357739	64.428071
78.3551261	54.2690501	65.8456011	64.7357739	63.490571
78.3551261	52.2690501	64.8456011	64.7357739	63.303071
78.3551261	51.2690501	64.8456011	66.7357739	63.428071
77.3551261	51.2690501	64.8456011	65.7357739	63.021821
77.3551261	51.2690501	64.8456011	64.7357739	62.740571
76.3551261	52.2690501	63.8456011	63.7357739	62.615571
75.3551261	52.2690501	63.8456011	62.7357739	62.740571
75.3551261	54.2690501	63.8456011	62.7357739	63.318696
75.3551261	54.2690501	63.8456011	62.7357739	63.318696
75.3551261	54.2690501	64.8456011	63.7357739	64.021821
75.3551261	57.2690501	65.8456011	63.7357739	64.443696
74.3551261	57.2690501	69.8456011	64.7357739	64.834321
73.3551261	57.2690501	70.8456011	63.7357739	65.443696
72.3551261	56.2690501	70.8456011	63.7357739	65.881196
72.3551261	56.2690501	72.8456011	62.7357739	67.115571
72.3551261	56.2690501	76.8456011	62.7357739	67.631196
72.3551261	57.2690501	77.8456011	62.7357739	67.818696
72.3551261	57.2690501	78.8456011	63.7357739	68.146821
72.3551261	57.2690501	75.8456011	64.7357739	68.209321
73.3551261	56.2690501	70.8456011	64.7357739	68.6701505
73.3551261	56.2690501	68.8456011	64.7357739	67.4201505

73.3551261	56.2690501	67.8456011	64.7357739	66.7951505
77.3551261	55.2690501	66.8456011	64.7357739	67.0451505
77.3551261	54.2690501	65.8456011	64.7357739	66.2951505
77.3551261	54.2690501	64.8456011	64.7357739	66.1701505
77.3551261	52.2690501	64.8456011	69.7357739	65.5451505
77.3551261	52.2690501	64.8456011	72.7357739	65.0451505
77.3551261	52.2690501	64.8456011	73.7357739	64.7951505
77.3551261	52.2690501	64.8456011	71.7357739	64.7951505
77.3551261	52.2690501	64.8456011	70.7357739	64.9201505
76.3551261	52.2690501	64.8456011	68.7357739	64.7951505
76.3551261	53.2690501	64.8456011	68.7357739	65.0451505
76.3551261	53.2690501	64.8456011	67.7357739	64.9201505
76.3551261	53.2690501	64.8456011	67.7357739	65.0451505
76.3551261	53.2690501	64.8456011	68.7357739	65.1701505
76.3551261	53.2690501	64.8456011	69.7357739	65.2951505
76.3551261	53.2690501	64.8456011	70.7357739	65.2951505
76.3551261	53.2690501	64.8456011	70.7357739	64.9201505
76.3551261	55.2690501	64.8456011	71.7357739	65.7951505
76.3551261	55.2690501	64.8456011	71.7357739	65.7951505

Rolling Avg	Time AZ
78.1129433	10:59:31 AM
78.2700862	10:59:32 AM
78.427229	10:59:33 AM
78.577229	10:59:34 AM
78.7058004	10:59:35 AM
78.8058004	10:59:36 AM
78.8879433	10:59:37 AM
78.9629433	10:59:38 AM
79.0343719	10:59:39 AM
79.0950862	10:59:40 AM
79.1593719	10:59:41 AM
79.202229	10:59:42 AM
79.2738378	10:59:43 AM
79.3664287	10:59:44 AM
79.462591	10:59:45 AM
79.5373247	10:59:46 AM
79.6562549	10:59:47 AM
79.7200051	10:59:48 AM
79.76255	10:59:49 AM
79.7818805	10:59:50 AM
79.7888785	10:59:51 AM
79.8017358	10:59:52 AM
79.7983543	10:59:53 AM
79.7935778	10:59:54 AM
79.829203	10:59:55 AM
79.8638795	10:59:56 AM
79.8973284	10:59:57 AM
79.9221277	10:59:58 AM
79.9377527	10:59:59 AM
79.9103231	11:00:00 AM
79.9385822	11:00:01 AM
79.9512624	11:00:02 AM
79.9483176	11:00:03 AM
79.9315335	11:00:04 AM
79.8982315	11:00:05 AM
79.8229652	11:00:06 AM
79.7168954	11:00:07 AM
79.5505577	11:00:08 AM
79.3788629	11:00:09 AM
79.2004717	11:00:10 AM
79.0064554	11:00:11 AM
78.832082	11:00:12 AM

78.6804765	11:00:13 AM
78.5360138	11:00:14 AM
78.4303904	11:00:15 AM
78.3488742	11:00:16 AM
78.2615544	11:00:17 AM
78.1764667	11:00:18 AM
78.0909326	11:00:19 AM
78.0094163	11:00:20 AM
77.9310251	11:00:21 AM
77.8535267	11:00:22 AM
77.7441517	11:00:23 AM
77.6410267	11:00:24 AM
77.5191517	11:00:25 AM
77.3785267	11:00:26 AM
77.2347767	11:00:27 AM
77.1747781	11:00:28 AM
77.113217	11:00:29 AM
77.0672809	11:00:30 AM
77.0225167	11:00:31 AM
76.9968931	11:00:32 AM
76.9732226	11:00:33 AM
76.9429116	11:00:34 AM
76.848538	11:00:35 AM
76.7443987	11:00:36 AM
76.6379158	11:00:37 AM
76.5333859	11:00:38 AM
76.4304411	11:00:39 AM
76.3535866	11:00:40 AM
76.2076505	11:00:41 AM
76.0732755	11:00:42 AM
75.9576505	11:00:43 AM
75.8357755	11:00:44 AM
75.7389005	11:00:45 AM
75.6420255	11:00:46 AM
75.5420255	11:00:47 AM
75.4143727	11:00:48 AM
75.2882824	11:00:49 AM
75.1618015	11:00:50 AM
75.0509455	11:00:51 AM
74.9205583	11:00:52 AM
74.7675149	11:00:53 AM
74.6706399	11:00:54 AM
74.5800149	11:00:55 AM
74.4893899	11:00:56 AM
74.4112649	11:00:57 AM

74.3300149	11:00:58 AM
74.2487649	11:00:59 AM
74.1831399	11:01:00 AM
74.1143899	11:01:01 AM
74.0456399	11:01:02 AM
73.9862649	11:01:03 AM
73.9143899	11:01:04 AM
73.8518899	11:01:05 AM
73.8206399	11:01:06 AM
73.8050149	11:01:07 AM
73.7862649	11:01:08 AM
73.7893899	11:01:09 AM
73.7925149	11:01:10 AM
73.7987649	11:01:11 AM
73.7425149	11:01:12 AM
73.6737649	11:01:13 AM
73.6018899	11:01:14 AM
73.5675149	11:01:15 AM
73.5300149	11:01:16 AM
73.4925149	11:01:17 AM
73.4518899	11:01:18 AM
73.3925149	11:01:19 AM
73.3393899	11:01:20 AM
73.2987649	11:01:21 AM
73.2550149	11:01:22 AM
73.2175149	11:01:23 AM
73.1831399	11:01:24 AM
73.1268899	11:01:25 AM
73.0800149	11:01:26 AM
73.0393899	11:01:27 AM
73.0670427	11:01:28 AM
73.108758	11:01:29 AM
73.163364	11:01:30 AM
73.2398449	11:01:31 AM
73.3296071	11:01:32 AM
73.4264005	11:01:33 AM
73.4639005	11:01:34 AM
73.4159352	11:01:35 AM
73.3558605	11:01:36 AM
73.2985202	11:01:37 AM
73.2333674	11:01:38 AM
73.1689958	11:01:39 AM
73.0886087	11:01:40 AM
73.0082215	11:01:41 AM
72.9375999	11:01:42 AM

72.8712752	11:01:43 AM
72.8201849	11:01:44 AM
72.7870633	11:01:45 AM
72.7738637	11:01:46 AM
72.7540233	11:01:47 AM
72.7474643	11:01:48 AM
72.7627802	11:01:49 AM
72.7640337	11:01:50 AM
72.7660684	11:01:51 AM
72.8122437	11:01:52 AM
72.8400596	11:01:53 AM
72.8370162	11:01:54 AM
72.7978478	11:01:55 AM
72.8218791	11:01:56 AM
72.8463011	11:01:57 AM
72.8820512	11:01:58 AM
72.9342075	11:01:59 AM
72.9941763	11:02:00 AM
73.055317	11:02:01 AM
73.1215358	11:02:02 AM
73.130805	11:02:03 AM
73.2060082	11:02:04 AM
73.2488711	11:02:05 AM
73.2987652	11:02:06 AM
73.4184997	11:02:07 AM
73.5140154	11:02:08 AM
73.5966404	11:02:09 AM
73.6534843	11:02:10 AM
73.6369722	11:02:11 AM
73.6220225	11:02:12 AM
73.6538664	11:02:13 AM
73.681804	11:02:14 AM
73.7070072	11:02:15 AM
73.726351	11:02:16 AM
73.7226479	11:02:17 AM
73.7017574	11:02:18 AM
73.6816481	11:02:19 AM
73.6728669	11:02:20 AM
73.664867	11:02:21 AM
73.6517889	11:02:22 AM
73.628164	11:02:23 AM
73.5924297	11:02:24 AM
73.5551329	11:02:25 AM
73.5080705	11:02:26 AM
73.4692112	11:02:27 AM

73.4186331	11:02:28 AM
73.3664926	11:02:29 AM
73.333102	11:02:30 AM
73.3090865	11:02:31 AM
73.2893678	11:02:32 AM
73.308321	11:02:33 AM
73.3550086	11:02:34 AM
73.4511024	11:02:35 AM
73.5082152	11:02:36 AM
73.5930624	11:02:37 AM
73.6978314	11:02:38 AM
73.804163	11:02:39 AM
73.902682	11:02:40 AM
73.9953417	11:02:41 AM
74.0907358	11:02:42 AM
74.2352671	11:02:43 AM
74.3154267	11:02:44 AM
74.4302705	11:02:45 AM
74.5271455	11:02:46 AM
74.5643364	11:02:47 AM
74.6038711	11:02:48 AM
74.6383277	11:02:49 AM
74.6821592	11:02:50 AM
74.7743467	11:02:51 AM
74.8540342	11:02:52 AM
74.8791158	11:02:53 AM
74.9104474	11:02:54 AM
74.9956036	11:02:55 AM
75.0838849	11:02:56 AM
75.1995099	11:02:57 AM
75.3299786	11:02:58 AM
75.4666974	11:02:59 AM
75.5987286	11:03:00 AM
75.7252911	11:03:01 AM
75.8557599	11:03:02 AM
75.9776349	11:03:03 AM
76.0901349	11:03:04 AM
76.1854474	11:03:05 AM
76.2752911	11:03:06 AM
76.3713849	11:03:07 AM
76.4573224	11:03:08 AM
76.5237286	11:03:09 AM
76.5706036	11:03:10 AM
76.6010724	11:03:11 AM
76.6174786	11:03:12 AM

76.6291974	11:03:13 AM
76.6331036	11:03:14 AM
76.6073224	11:03:15 AM
76.5713849	11:03:16 AM
76.5073224	11:03:17 AM
76.4010724	11:03:18 AM
76.2971661	11:03:19 AM
76.1831036	11:03:20 AM
76.0698224	11:03:21 AM
75.9409161	11:03:22 AM
75.8088849	11:03:23 AM
75.7181783	11:03:24 AM
75.5689596	11:03:25 AM
75.4439596	11:03:26 AM
75.3228658	11:03:27 AM
75.1963033	11:03:28 AM
75.0705221	11:03:29 AM
74.9439596	11:03:30 AM
74.8142721	11:03:31 AM
74.6923971	11:03:32 AM
74.5830221	11:03:33 AM
74.5239561	11:03:34 AM
74.4539527	11:03:35 AM
74.392543	11:03:36 AM
74.3342583	11:03:37 AM
74.2826142	11:03:38 AM
74.2678763	11:03:39 AM
74.2024388	11:03:40 AM
74.1252824	11:03:41 AM
74.0317199	11:03:42 AM
73.9494854	11:03:43 AM
73.91756	11:03:44 AM
73.8926659	11:03:45 AM
73.8755842	11:03:46 AM
73.8381901	11:03:47 AM
73.7906397	11:03:48 AM
73.7329331	11:03:49 AM
73.6806952	11:03:50 AM
73.6253323	11:03:51 AM
73.5605944	11:03:52 AM
73.4989815	11:03:53 AM
73.4451811	11:03:54 AM
73.398412	11:03:55 AM
73.3375803	11:03:56 AM
73.2673737	11:03:57 AM

73.2143546	11:03:58 AM
73.1660229	11:03:59 AM
73.1403475	11:04:00 AM
73.1279534	11:04:01 AM
73.1546218	11:04:02 AM
73.1953526	11:04:03 AM
73.2088213	11:04:04 AM
73.2815834	11:04:05 AM
73.3348143	11:04:06 AM
73.3864826	11:04:07 AM
73.4561197	11:04:08 AM
73.5366943	11:04:09 AM
73.6219565	11:04:10 AM
73.7134686	11:04:11 AM
73.8081057	11:04:12 AM
73.8980553	11:04:13 AM
73.9416021	11:04:14 AM
73.9960864	11:04:15 AM
74.0443207	11:04:16 AM
74.0792737	11:04:17 AM
74.1146174	11:04:18 AM
74.1239924	11:04:19 AM
74.1903171	11:04:20 AM
74.282423	11:04:21 AM
74.3854665	11:04:22 AM
74.4826505	11:04:23 AM
74.5420255	11:04:24 AM
74.6107755	11:04:25 AM
74.6795255	11:04:26 AM
74.7670255	11:04:27 AM
74.8670255	11:04:28 AM
74.9732755	11:04:29 AM
75.0889005	11:04:30 AM
75.1951505	11:04:31 AM
75.2889005	11:04:32 AM
75.3607755	11:04:33 AM
75.4264005	11:04:34 AM
75.4982755	11:04:35 AM
75.5701505	11:04:36 AM
75.6389005	11:04:37 AM
75.7201505	11:04:38 AM
75.7889005	11:04:39 AM
75.8357755	11:04:40 AM
75.8545255	11:04:41 AM
75.8545255	11:04:42 AM

75.8545255	11:04:43 AM
75.8545255	11:04:44 AM
75.8545255	11:04:45 AM
75.8482755	11:04:46 AM
75.8357755	11:04:47 AM
75.8170255	11:04:48 AM
75.8232755	11:04:49 AM
75.8295255	11:04:50 AM
75.8357755	11:04:51 AM
75.8357755	11:04:52 AM
75.8326505	11:04:53 AM
75.8232755	11:04:54 AM
75.8014005	11:04:55 AM
75.7920255	11:04:56 AM
75.7764005	11:04:57 AM
75.7639005	11:04:58 AM
75.7326505	11:04:59 AM
75.6920255	11:05:00 AM
75.6545255	11:05:01 AM
75.6139005	11:05:02 AM
75.5670255	11:05:03 AM
75.5139005	11:05:04 AM
75.4576505	11:05:05 AM
75.4295255	11:05:06 AM
75.4232755	11:05:07 AM
75.4295255	11:05:08 AM
75.4607755	11:05:09 AM
75.4670255	11:05:10 AM
75.4857755	11:05:11 AM
75.4576505	11:05:12 AM
75.4357755	11:05:13 AM
75.4201505	11:05:14 AM
75.3982755	11:05:15 AM
75.3826505	11:05:16 AM
75.3732755	11:05:17 AM
75.3514005	11:05:18 AM
75.3295255	11:05:19 AM
75.3357755	11:05:20 AM
75.3451505	11:05:21 AM
75.3514005	11:05:22 AM
75.3389005	11:05:23 AM
75.2823415	11:05:24 AM
75.2253918	11:05:25 AM
75.1637546	11:05:26 AM
75.0841486	11:05:27 AM

74.9975115	11:05:28 AM
74.8889993	11:05:29 AM
74.7871277	11:05:30 AM
74.6817405	11:05:31 AM
74.5728377	11:05:32 AM
74.4506537	11:05:33 AM
74.3319852	11:05:34 AM
74.2398793	11:05:35 AM
74.1520702	11:05:36 AM
74.0740268	11:05:37 AM
73.9580927	11:05:38 AM
73.8425493	11:05:39 AM
73.7234902	11:05:40 AM
73.5919312	11:05:41 AM
73.4736534	11:05:42 AM
73.3643599	11:05:43 AM
73.3143599	11:05:44 AM
73.2706099	11:05:45 AM
73.2174849	11:05:46 AM
73.1456099	11:05:47 AM
73.0737349	11:05:48 AM
72.9831099	11:05:49 AM
72.9174849	11:05:50 AM
72.8424849	11:05:51 AM
72.8362349	11:05:52 AM
72.809065	11:05:53 AM
72.7787701	11:05:54 AM
72.744569	11:05:55 AM
72.7076335	11:05:56 AM
72.6664011	11:05:57 AM
72.6251687	11:05:58 AM
72.5870613	11:05:59 AM
72.5512977	11:06:00 AM
72.524909	11:06:01 AM
72.502036	11:06:02 AM
72.4865849	11:06:03 AM
72.5194897	11:06:04 AM
72.5520038	11:06:05 AM
72.5981899	11:06:06 AM
72.7184208	11:06:07 AM
72.861308	11:06:08 AM
73.0073202	11:06:09 AM
73.1560668	11:06:10 AM
73.3083289	11:06:11 AM
73.4484818	11:06:12 AM

73.5956658	11:06:13 AM
73.7362093	11:06:14 AM
73.8626902	11:06:15 AM
73.9786243	11:06:16 AM
74.0785427	11:06:17 AM
74.2069768	11:06:18 AM
74.3287702	11:06:19 AM
74.4353293	11:06:20 AM
74.5481383	11:06:21 AM
74.6664161	11:06:22 AM
74.7694596	11:06:23 AM
74.8194596	11:06:24 AM
74.8444596	11:06:25 AM
74.8444596	11:06:26 AM
74.8413346	11:06:27 AM
74.8382096	11:06:28 AM
74.8288346	11:06:29 AM
74.8132096	11:06:30 AM
74.8163346	11:06:31 AM
74.8225846	11:06:32 AM
74.8622544	11:06:33 AM
74.9081743	11:06:34 AM
74.9611255	11:06:35 AM
75.016811	11:06:36 AM
75.0799184	11:06:37 AM
75.1492758	11:06:38 AM
75.2186332	11:06:39 AM
75.2543968	11:06:40 AM
75.2620354	11:06:41 AM
75.2505335	11:06:42 AM
75.2316096	11:06:43 AM
75.2240139	11:06:44 AM
75.2296994	11:06:45 AM
75.2076505	11:06:46 AM
75.1107755	11:06:47 AM
74.9951505	11:06:48 AM
74.8607755	11:06:49 AM
74.7170255	11:06:50 AM
74.5607755	11:06:51 AM
74.4107755	11:06:52 AM
74.2701505	11:06:53 AM
74.1389005	11:06:54 AM
74.0107755	11:06:55 AM
73.8920255	11:06:56 AM
73.7857755	11:06:57 AM

73.6826505	11:06:58 AM
73.5920255	11:06:59 AM
73.5076505	11:07:00 AM
73.4264005	11:07:01 AM
73.3357755	11:07:02 AM
73.2764005	11:07:03 AM
73.2201505	11:07:04 AM
73.1732755	11:07:05 AM
73.1482755	11:07:06 AM
73.1451505	11:07:07 AM
73.1482755	11:07:08 AM
73.1514005	11:07:09 AM
73.1639005	11:07:10 AM
73.1184304	11:07:11 AM
73.0764758	11:07:12 AM
73.0858508	11:07:13 AM
73.0560057	11:07:14 AM
73.0359261	11:07:15 AM
73.0135029	11:07:16 AM
72.9934233	11:07:17 AM
72.9612344	11:07:18 AM
72.9278736	11:07:19 AM
72.9195128	11:07:20 AM
72.9388864	11:07:21 AM
72.961385	11:07:22 AM
72.989743	11:07:23 AM
72.9927103	11:07:24 AM
72.9831777	11:07:25 AM
73.0033325	11:07:26 AM
73.0547374	11:07:27 AM
73.1135641	11:07:28 AM
73.1973908	11:07:29 AM
73.2819988	11:07:30 AM
73.3845755	11:07:31 AM
73.4969178	11:07:32 AM
73.6123852	11:07:33 AM
73.7219931	11:07:34 AM
73.829648	11:07:35 AM
73.9349591	11:07:36 AM
74.0410514	11:07:37 AM
74.1483156	11:07:38 AM
74.2395642	11:07:39 AM
74.3394066	11:07:40 AM
74.4353427	11:07:41 AM
74.5441694	11:07:42 AM

74.6377617	11:07:43 AM
74.7278384	11:07:44 AM
74.8226026	11:07:45 AM
74.9142419	11:07:46 AM
74.9734592	11:07:47 AM
75.0127547	11:07:48 AM
75.0461908	11:07:49 AM
75.0761112	11:07:50 AM
75.1647831	11:07:51 AM
75.3161126	11:07:52 AM
75.4161126	11:07:53 AM
75.5459578	11:07:54 AM
75.7035373	11:07:55 AM
75.8697106	11:07:56 AM
76.0335401	11:07:57 AM
76.2126041	11:07:58 AM
76.4084648	11:07:59 AM
76.5855756	11:08:00 AM
76.766202	11:08:01 AM
76.9093284	11:08:02 AM
77.0434705	11:08:03 AM
77.1717531	11:08:04 AM
77.2875358	11:08:05 AM
77.4017559	11:08:06 AM
77.5034761	11:08:07 AM
77.6040244	11:08:08 AM
77.6920727	11:08:09 AM
77.7762147	11:08:10 AM
77.833013	11:08:11 AM
77.8644206	11:08:12 AM
77.8833283	11:08:13 AM
77.8893453	11:08:14 AM
77.8848155	11:08:15 AM
77.8607544	11:08:16 AM
77.8202871	11:08:17 AM
77.7630228	11:08:18 AM
77.7123992	11:08:19 AM
77.6500569	11:08:20 AM
77.5791208	11:08:21 AM
77.4984191	11:08:22 AM
77.4204518	11:08:23 AM
77.33975	11:08:24 AM
77.2574858	11:08:25 AM
77.1752216	11:08:26 AM
77.0910043	11:08:27 AM

77.0298338	11:08:28 AM
76.9776477	11:08:29 AM
76.9414772	11:08:30 AM
76.9107755	11:08:31 AM
76.8139005	11:08:32 AM
76.7201505	11:08:33 AM
76.6389005	11:08:34 AM
76.5170255	11:08:35 AM
76.3732755	11:08:36 AM
76.2326505	11:08:37 AM
76.0826505	11:08:38 AM
75.9232755	11:08:39 AM
75.7670255	11:08:40 AM
75.6076505	11:08:41 AM
75.4639005	11:08:42 AM
75.3232755	11:08:43 AM
75.2170255	11:08:44 AM
75.1264005	11:08:45 AM
75.0014005	11:08:46 AM
74.8857755	11:08:47 AM
74.7732755	11:08:48 AM
74.6545255	11:08:49 AM
74.5264005	11:08:50 AM
74.4107755	11:08:51 AM
74.3170255	11:08:52 AM
74.2326505	11:08:53 AM
74.1451505	11:08:54 AM
74.0451505	11:08:55 AM
73.9451505	11:08:56 AM
73.8451505	11:08:57 AM
73.7482755	11:08:58 AM
73.6420255	11:08:59 AM
73.5514005	11:09:00 AM
73.4607755	11:09:01 AM
73.3732755	11:09:02 AM
73.2889005	11:09:03 AM
73.2076505	11:09:04 AM
73.1357755	11:09:05 AM
73.0514005	11:09:06 AM
72.9732755	11:09:07 AM
72.8795255	11:09:08 AM
72.7795255	11:09:09 AM
72.6607755	11:09:10 AM
72.5357755	11:09:11 AM
72.4139005	11:09:12 AM

72.2701505	11:09:13 AM
72.1295255	11:09:14 AM
72.0107755	11:09:15 AM
71.8857755	11:09:16 AM
71.7482755	11:09:17 AM
71.6076505	11:09:18 AM
71.4514005	11:09:19 AM
71.2982755	11:09:20 AM
71.1295255	11:09:21 AM
70.9764005	11:09:22 AM
70.8326505	11:09:23 AM
70.6732755	11:09:24 AM
70.5232755	11:09:25 AM
70.4139005	11:09:26 AM
70.3045255	11:09:27 AM
70.1920255	11:09:28 AM
70.1045255	11:09:29 AM
70.0357755	11:09:30 AM
69.9826505	11:09:31 AM
69.9357755	11:09:32 AM
69.8951505	11:09:33 AM
69.8764005	11:09:34 AM
69.8857755	11:09:35 AM
69.9357755	11:09:36 AM
70.0139005	11:09:37 AM
70.1420255	11:09:38 AM
70.2920255	11:09:39 AM
70.4357755	11:09:40 AM
70.5857755	11:09:41 AM
70.7139005	11:09:42 AM
70.8264005	11:09:43 AM
70.9357755	11:09:44 AM
71.0482755	11:09:45 AM
71.1254616	11:09:46 AM
71.1995227	11:09:47 AM
71.2770994	11:09:48 AM
71.3394418	11:09:49 AM
71.4068622	11:09:50 AM
71.4723296	11:09:51 AM
71.5338907	11:09:52 AM
71.6059986	11:09:53 AM
71.6788878	11:09:54 AM
71.7443552	11:09:55 AM
71.83951	11:09:56 AM
71.9588836	11:09:57 AM

72.0790385	11:09:58 AM
72.2144277	11:09:59 AM
72.3728638	11:10:00 AM
72.5500499	11:10:01 AM
72.7330953	11:10:02 AM
72.9071564	11:10:03 AM
73.0706706	11:10:04 AM
73.2158255	11:10:05 AM
73.3578553	11:10:06 AM
73.4901195	11:10:07 AM
73.616915	11:10:08 AM
73.7085542	11:10:09 AM
73.7943341	11:10:10 AM
73.8769889	11:10:11 AM
73.9537844	11:10:12 AM
74.0227673	11:10:13 AM
74.0905784	11:10:14 AM
74.2062034	11:10:15 AM
74.2843284	11:10:16 AM
74.3437034	11:10:17 AM
74.3593284	11:10:18 AM
74.3530784	11:10:19 AM
74.3562034	11:10:20 AM
74.3593284	11:10:21 AM
74.3812034	11:10:22 AM
74.4093284	11:10:23 AM
74.4374534	11:10:24 AM
74.4562034	11:10:25 AM
74.5040173	11:10:26 AM
74.5455813	11:10:27 AM
74.5773795	11:10:28 AM
74.6244122	11:10:29 AM
74.6538667	11:10:30 AM
74.6696494	11:10:31 AM
74.6830883	11:10:32 AM
74.6922303	11:10:33 AM
74.6849661	11:10:34 AM
74.6538738	11:10:35 AM
74.6024689	11:10:36 AM
74.5424703	11:10:37 AM
74.4393453	11:10:38 AM
74.3307516	11:10:39 AM
74.202236	11:10:40 AM
74.0721578	11:10:41 AM
73.9510641	11:10:42 AM

73.8330953	11:10:43 AM
73.7346578	11:10:44 AM
73.6631735	11:10:45 AM
73.608486	11:10:46 AM
73.555361	11:10:47 AM
73.4924703	11:10:48 AM
73.4526266	11:10:49 AM
73.4186422	11:10:50 AM
73.3807516	11:10:51 AM
73.3385641	11:10:52 AM
73.2916891	11:10:53 AM
73.2424703	11:10:54 AM
73.1360627	11:10:55 AM
73.035905	11:10:56 AM
72.97028	11:10:57 AM
72.89528	11:10:58 AM
72.817155	11:10:59 AM
72.73903	11:11:00 AM
72.65153	11:11:01 AM
72.55778	11:11:02 AM
72.46403	11:11:03 AM
72.385905	11:11:04 AM
72.310905	11:11:05 AM
72.260905	11:11:06 AM
72.22028	11:11:07 AM
72.198405	11:11:08 AM
72.18278	11:11:09 AM
72.185905	11:11:10 AM
72.198405	11:11:11 AM
72.217155	11:11:12 AM
72.248405	11:11:13 AM
72.27653	11:11:14 AM
72.317155	11:11:15 AM
72.367155	11:11:16 AM
72.410905	11:11:17 AM
72.4970002	11:11:18 AM
72.5827047	11:11:19 AM
72.7027842	11:11:20 AM
72.8275513	11:11:21 AM
72.9013203	11:11:22 AM
72.9746987	11:11:23 AM
73.0297177	11:11:24 AM
73.0566117	11:11:25 AM
73.0737401	11:11:26 AM
73.1508509	11:11:27 AM

73.189073	11:11:28 AM
73.2300296	11:11:29 AM
73.2545798	11:11:30 AM
73.2697551	11:11:31 AM
73.2474304	11:11:32 AM
73.2172931	11:11:33 AM
73.1906715	11:11:34 AM
73.1675655	11:11:35 AM
73.1511002	11:11:36 AM
73.110649	11:11:37 AM
73.0924635	11:11:38 AM
73.0801374	11:11:39 AM
73.05453	11:11:40 AM
73.025407	11:11:41 AM
72.993159	11:11:42 AM
72.9542703	11:11:43 AM
72.9411629	11:11:44 AM
72.9503212	11:11:45 AM
72.9547919	11:11:46 AM
72.9631689	11:11:47 AM
72.9899053	11:11:48 AM
73.0142979	11:11:49 AM
73.0609561	11:11:50 AM
73.1181612	11:11:51 AM
73.1792726	11:11:52 AM
73.2310089	11:11:53 AM
73.2245422	11:11:54 AM
73.2307922	11:11:55 AM
73.2089172	11:11:56 AM
73.1870422	11:11:57 AM
73.1557922	11:11:58 AM
73.1245422	11:11:59 AM
73.0526672	11:12:00 AM
72.9745422	11:12:01 AM
72.9419464	11:12:02 AM
72.9124757	11:12:03 AM
72.870505	11:12:04 AM
72.8386905	11:12:05 AM
72.7904697	11:12:06 AM
72.6779697	11:12:07 AM
72.6195928	11:12:08 AM
72.5612158	11:12:09 AM
72.519245	11:12:10 AM
72.4936805	11:12:11 AM
72.5126473	11:12:12 AM

72.5488016	11:12:13 AM
72.5849558	11:12:14 AM
72.6207195	11:12:15 AM
72.6623425	11:12:16 AM
72.6934186	11:12:17 AM
72.7303542	11:12:18 AM
72.7708053	11:12:19 AM
72.8089127	11:12:20 AM
72.8505357	11:12:21 AM
72.8890337	11:12:22 AM
72.9279224	11:12:23 AM
72.9222797	11:12:24 AM
72.8724965	11:12:25 AM
72.8055258	11:12:26 AM
72.7377738	11:12:27 AM
72.6485374	11:12:28 AM
72.5522698	11:12:29 AM
72.4587366	11:12:30 AM
72.3577815	11:12:31 AM
72.2591701	11:12:32 AM
72.1636838	11:12:33 AM
72.1232755	11:12:34 AM
72.0670255	11:12:35 AM
72.0420255	11:12:36 AM
72.0232755	11:12:37 AM
72.0170255	11:12:38 AM
71.9701505	11:12:39 AM
71.9232755	11:12:40 AM
71.8732755	11:12:41 AM
71.8326505	11:12:42 AM
71.7982755	11:12:43 AM
71.7920255	11:12:44 AM
71.7889005	11:12:45 AM
71.8076505	11:12:46 AM
71.8295255	11:12:47 AM
71.8545255	11:12:48 AM
71.8610085	11:12:49 AM
71.8483508	11:12:50 AM
71.8282713	11:12:51 AM
71.8492074	11:12:52 AM
71.8732685	11:12:53 AM
71.8906889	11:12:54 AM
71.8799844	11:12:55 AM
71.858733	11:12:56 AM
71.8374816	11:12:57 AM

71.8037302	11:12:58 AM
71.7727132	11:12:59 AM
71.7506805	11:13:00 AM
71.7345072	11:13:01 AM
71.7191152	11:13:02 AM
71.692395	11:13:03 AM
71.6602061	11:13:04 AM
71.6727061	11:13:05 AM
71.6727061	11:13:06 AM
71.6727061	11:13:07 AM
71.6633311	11:13:08 AM
71.6539561	11:13:09 AM
71.6102061	11:13:10 AM
71.5602061	11:13:11 AM
71.4945811	11:13:12 AM
71.4352061	11:13:13 AM
71.3945811	11:13:14 AM
71.3570811	11:13:15 AM
71.3195811	11:13:16 AM
71.2758311	11:13:17 AM
71.2320811	11:13:18 AM
71.2320811	11:13:19 AM
71.2227061	11:13:20 AM
71.2102061	11:13:21 AM
71.1945811	11:13:22 AM
71.1664561	11:13:23 AM
71.1195811	11:13:24 AM
71.0758311	11:13:25 AM
71.0164561	11:13:26 AM
70.9633311	11:13:27 AM
70.9008311	11:13:28 AM
70.8380981	11:13:29 AM
70.7851308	11:13:30 AM
70.7427103	11:13:31 AM
70.6686492	11:13:32 AM
70.5945882	11:13:33 AM
70.5584177	11:13:34 AM
70.5847472	11:13:35 AM
70.6278736	11:13:36 AM
70.692875	11:13:37 AM
70.7766264	11:13:38 AM
70.7960986	11:13:39 AM
70.8093207	11:13:40 AM
70.8190272	11:13:41 AM
70.8531693	11:13:42 AM

70.8955144	11:13:43 AM
70.9433283	11:13:44 AM
70.9464533	11:13:45 AM
70.9527033	11:13:46 AM
70.9527033	11:13:47 AM
70.9402033	11:13:48 AM
70.9214533	11:13:49 AM
70.9058283	11:13:50 AM
70.8870783	11:13:51 AM
70.8902033	11:13:52 AM
70.8902033	11:13:53 AM
70.8683283	11:13:54 AM
70.8370783	11:13:55 AM
70.8152033	11:13:56 AM
70.7933283	11:13:57 AM
70.7870783	11:13:58 AM
70.7777033	11:13:59 AM
70.7839533	11:14:00 AM
70.7902033	11:14:01 AM
70.7870783	11:14:02 AM
70.7902033	11:14:03 AM
70.7902033	11:14:04 AM
70.7839533	11:14:05 AM
70.7902033	11:14:06 AM
70.7870783	11:14:07 AM
70.7902033	11:14:08 AM
70.8152033	11:14:09 AM
70.8433283	11:14:10 AM
70.8745783	11:14:11 AM
70.8964533	11:14:12 AM
70.9152033	11:14:13 AM
70.8964533	11:14:14 AM
70.8589533	11:14:15 AM
70.8370783	11:14:16 AM
70.8402033	11:14:17 AM
70.7902033	11:14:18 AM
70.7892482	11:14:19 AM
70.7824338	11:14:20 AM
70.7951505	11:14:21 AM
70.7764005	11:14:22 AM
70.7826505	11:14:23 AM
70.8076505	11:14:24 AM
70.8576505	11:14:25 AM
70.9326505	11:14:26 AM
71.0201505	11:14:27 AM

71.1264005	11:14:28 AM
71.2545255	11:14:29 AM
71.3363991	11:14:30 AM
71.4167102	11:14:31 AM
71.4673338	11:14:32 AM
71.5173338	11:14:33 AM
71.5673338	11:14:34 AM
71.6329588	11:14:35 AM
71.6798338	11:14:36 AM
71.7235838	11:14:37 AM
71.7485838	11:14:38 AM
71.7642088	11:14:39 AM
71.7673338	11:14:40 AM
71.7829588	11:14:41 AM
71.8079588	11:14:42 AM
71.8423338	11:14:43 AM
71.8923338	11:14:44 AM
71.9548338	11:14:45 AM
72.0173338	11:14:46 AM
72.1079588	11:14:47 AM
72.1985838	11:14:48 AM
72.2673338	11:14:49 AM
72.3204588	11:14:50 AM
72.3548338	11:14:51 AM
72.4017088	11:14:52 AM
72.4610838	11:14:53 AM
72.5329588	11:14:54 AM
72.5892088	11:14:55 AM
72.6142088	11:14:56 AM
72.5485838	11:14:57 AM
72.4892088	11:14:58 AM
72.4454588	11:14:59 AM
72.3210818	11:15:00 AM
72.1740486	11:15:01 AM
72.043031	11:15:02 AM
71.8924821	11:15:03 AM
71.7337301	11:15:04 AM
71.5753688	11:15:05 AM
71.4534938	11:15:06 AM
71.3097438	11:15:07 AM
71.1909938	11:15:08 AM
71.0597438	11:15:09 AM
71.0028702	11:15:10 AM
70.9663091	11:15:11 AM
70.9500605	11:15:12 AM

70.9375605	11:15:13 AM
70.9469355	11:15:14 AM
70.9594355	11:15:15 AM
70.9906855	11:15:16 AM
71.0344355	11:15:17 AM
71.1038091	11:15:18 AM
71.2036514	11:15:19 AM
71.3421657	11:15:20 AM
71.4783361	11:15:21 AM
71.6023972	11:15:22 AM
71.7217708	11:15:23 AM
71.8317694	11:15:24 AM
71.9253617	11:15:25 AM
71.9966885	11:15:26 AM
72.0348121	11:15:27 AM
72.0784044	11:15:28 AM
72.1262936	11:15:29 AM
72.1765266	11:15:30 AM
72.2232439	11:15:31 AM
72.2258206	11:15:32 AM
72.2080849	11:15:33 AM
72.1669116	11:15:34 AM
72.1120664	11:15:35 AM
72.0482369	11:15:36 AM
72.037923	11:15:37 AM
72.0612028	11:15:38 AM
72.080967	11:15:39 AM
72.1813582	11:15:40 AM
72.3283915	11:15:41 AM
72.4937841	11:15:42 AM
72.6630829	11:15:43 AM
72.8187099	11:15:44 AM
72.9489463	11:15:45 AM
73.0176963	11:15:46 AM
73.0864463	11:15:47 AM
73.1208213	11:15:48 AM
73.1520713	11:15:49 AM
73.1583213	11:15:50 AM
73.1614463	11:15:51 AM
73.1645713	11:15:52 AM
73.1645713	11:15:53 AM
73.1583213	11:15:54 AM
73.1395713	11:15:55 AM
73.1051963	11:15:56 AM
73.0645713	11:15:57 AM

72.9983227	11:15:58 AM
72.9047303	11:15:59 AM
72.7787161	11:16:00 AM
72.6550456	11:16:01 AM
72.5341096	11:16:02 AM
72.411611	11:16:03 AM
72.2828624	11:16:04 AM
72.14552	11:16:05 AM
72.0241933	11:16:06 AM
71.8954447	11:16:07 AM
71.7612274	11:16:08 AM
71.6164632	11:16:09 AM
71.4481032	11:16:10 AM
71.2926339	11:16:11 AM
71.2181822	11:16:12 AM
71.1515429	11:16:13 AM
71.1020912	11:16:14 AM
71.0756864	11:16:15 AM
71.0676409	11:16:16 AM
71.0717048	11:16:17 AM
71.06405	11:16:18 AM
71.0692858	11:16:19 AM
71.0932716	11:16:20 AM
71.0838966	11:16:21 AM
71.0401466	11:16:22 AM
70.9901466	11:16:23 AM
70.9463966	11:16:24 AM
70.9213966	11:16:25 AM
70.9151466	11:16:26 AM
70.9245216	11:16:27 AM
70.9432716	11:16:28 AM
70.9776466	11:16:29 AM
71.0088966	11:16:30 AM
71.0213966	11:16:31 AM
71.0432716	11:16:32 AM
71.0620216	11:16:33 AM
71.0807716	11:16:34 AM
71.1057716	11:16:35 AM
71.1338966	11:16:36 AM
71.1526466	11:16:37 AM
71.1713966	11:16:38 AM
71.1932716	11:16:39 AM
71.2213966	11:16:40 AM
71.2432716	11:16:41 AM
71.2713966	11:16:42 AM

71.3057716	11:16:43 AM
71.3463966	11:16:44 AM
71.3932716	11:16:45 AM
71.4432716	11:16:46 AM
71.5088966	11:16:47 AM
71.5870216	11:16:48 AM
71.6870216	11:16:49 AM
71.8332735	11:16:50 AM
71.9889005	11:16:51 AM
72.0982755	11:16:52 AM
72.2295255	11:16:53 AM
72.3951505	11:16:54 AM
72.5576505	11:16:55 AM
72.6528054	11:16:56 AM
72.7147571	11:16:57 AM
72.7884275	11:16:58 AM
72.8726449	11:16:59 AM
72.9599872	11:17:00 AM
73.0481108	11:17:01 AM
73.1717813	11:17:02 AM
73.3040455	11:17:03 AM
73.4050597	11:17:04 AM
73.5052927	11:17:05 AM
73.5914632	11:17:06 AM
73.6561492	11:17:07 AM
73.718101	11:17:08 AM
73.7445058	11:17:09 AM
73.7615356	11:17:10 AM
73.7844249	11:17:11 AM
73.786611	11:17:12 AM
73.8005158	11:17:13 AM
73.8093425	11:17:14 AM
73.8291067	11:17:15 AM
73.8617616	11:17:16 AM
73.9065258	11:17:17 AM
73.9505088	11:17:18 AM
73.9929292	11:17:19 AM
74.0435528	11:17:20 AM
74.1074577	11:17:21 AM
74.1662844	11:17:22 AM
74.2212048	11:17:23 AM
74.2468284	11:17:24 AM
74.2720614	11:17:25 AM
74.2871381	11:17:26 AM
74.294793	11:17:27 AM

74.2794009	11:17:28 AM
74.2511183	11:17:29 AM
74.2126794	11:17:30 AM
74.1562717	11:17:31 AM
74.1033797	11:17:32 AM
74.0290033	11:17:33 AM
73.9261112	11:17:34 AM
73.8169692	11:17:35 AM
73.766188	11:17:36 AM
73.7361098	11:17:37 AM
73.7021255	11:17:38 AM
73.6603286	11:17:39 AM
73.6122817	11:17:40 AM
73.5564223	11:17:41 AM
73.4704848	11:17:42 AM
73.3833755	11:17:43 AM
73.3341567	11:17:44 AM
73.2915786	11:17:45 AM
73.2654067	11:17:46 AM
73.3163456	11:17:47 AM
73.3731439	11:17:48 AM
73.4592391	11:17:49 AM
73.5640842	11:17:50 AM
73.66307	11:17:51 AM
73.7608839	11:17:52 AM
73.8532291	11:17:53 AM
73.9444023	11:17:54 AM
74.0058881	11:17:55 AM
74.0357333	11:17:56 AM
74.0222191	11:17:57 AM
73.9969861	11:17:58 AM
73.9733156	11:17:59 AM
73.935192	11:18:00 AM
73.8837872	11:18:01 AM
73.8374605	11:18:02 AM
73.78254	11:18:03 AM
73.7537914	11:18:04 AM
73.7316835	11:18:05 AM
73.7197317	11:18:06 AM
73.7245769	11:18:07 AM
73.7368439	11:18:08 AM
73.7620016	11:18:09 AM
73.7941905	11:18:10 AM
73.7792876	11:18:11 AM
73.7573535	11:18:12 AM

73.7322944	11:18:13 AM
73.7123134	11:18:14 AM
73.6962387	11:18:15 AM
73.683289	11:18:16 AM
73.6636986	11:18:17 AM
73.6499676	11:18:18 AM
73.6311585	11:18:19 AM
73.6185994	11:18:20 AM
73.6644602	11:18:21 AM
73.7267272	11:18:22 AM
73.7971974	11:18:23 AM
73.8610269	11:18:24 AM
73.9127471	11:18:25 AM
73.9621235	11:18:26 AM
73.9496235	11:18:27 AM
73.9339985	11:18:28 AM
73.9152485	11:18:29 AM
73.8777485	11:18:30 AM
73.8527485	11:18:31 AM
73.8371235	11:18:32 AM
73.8058735	11:18:33 AM
73.7589985	11:18:34 AM
73.7339985	11:18:35 AM
73.7089985	11:18:36 AM
73.6996235	11:18:37 AM
73.6933735	11:18:38 AM
73.6652485	11:18:39 AM
73.6371235	11:18:40 AM
73.5902485	11:18:41 AM
73.5402485	11:18:42 AM
73.5058735	11:18:43 AM
73.4871235	11:18:44 AM
73.4683735	11:18:45 AM
73.4371235	11:18:46 AM
73.3964985	11:18:47 AM
73.3589985	11:18:48 AM
73.3339985	11:18:49 AM
73.3089985	11:18:50 AM
73.342809	11:18:51 AM
73.3863851	11:18:52 AM
73.4233207	11:18:53 AM
73.4524437	11:18:54 AM
73.4807854	11:18:55 AM
73.5306116	11:18:56 AM
73.5808283	11:18:57 AM

73.6173732	11:18:58 AM
73.6406369	11:18:59 AM
73.6482755	11:19:00 AM
73.5732755	11:19:01 AM
73.4701505	11:19:02 AM
73.3514005	11:19:03 AM
73.2264005	11:19:04 AM
73.1076505	11:19:05 AM
73.0076505	11:19:06 AM
72.9264005	11:19:07 AM
72.8639005	11:19:08 AM
72.8014005	11:19:09 AM
72.7545255	11:19:10 AM
72.7107755	11:19:11 AM
72.6732755	11:19:12 AM
72.6545255	11:19:13 AM
72.6451505	11:19:14 AM
72.6201505	11:19:15 AM
72.6201505	11:19:16 AM
72.6576505	11:19:17 AM
72.7107755	11:19:18 AM
72.7982755	11:19:19 AM
72.8857755	11:19:20 AM
72.9562619	11:19:21 AM
73.0271389	11:19:22 AM
73.1101252	11:19:23 AM
73.1817835	11:19:24 AM
73.2530511	11:19:25 AM
73.3243187	11:19:26 AM
73.4057425	11:19:27 AM
73.4981039	11:19:28 AM
73.5748403	11:19:29 AM
73.6519672	11:19:30 AM
73.7457172	11:19:31 AM
73.7957172	11:19:32 AM
73.8613422	11:19:33 AM
73.9300922	11:19:34 AM
74.0019672	11:19:35 AM
74.0269672	11:19:36 AM
74.0009168	11:19:37 AM
73.9807258	11:19:38 AM
73.9636597	11:19:39 AM
73.9594843	11:19:40 AM
73.9342151	11:19:41 AM
73.9214459	11:19:42 AM

73.8789892	11:19:43 AM
73.8486419	11:19:44 AM
73.8362634	11:19:45 AM
73.8063067	11:19:46 AM
73.8094317	11:19:47 AM
73.7875567	11:19:48 AM
73.7688067	11:19:49 AM
73.7656817	11:19:50 AM
73.7469317	11:19:51 AM
73.7219317	11:19:52 AM
73.6906817	11:19:53 AM
73.6781817	11:19:54 AM
73.6531817	11:19:55 AM
73.6094317	11:19:56 AM
73.5469317	11:19:57 AM
73.4241178	11:19:58 AM
73.3334928	11:19:59 AM
73.2272428	11:20:00 AM
73.1692564	11:20:01 AM
73.1046295	11:20:02 AM
73.0091431	11:20:03 AM
72.8937349	11:20:04 AM
72.7755923	11:20:05 AM
72.6480746	11:20:06 AM
72.5229008	11:20:07 AM
72.3899144	11:20:08 AM
72.2881781	11:20:09 AM
72.2110511	11:20:10 AM
72.1173011	11:20:11 AM
72.0860511	11:20:12 AM
72.0516761	11:20:13 AM
71.9511278	11:20:14 AM
71.8337826	11:20:15 AM
71.7195625	11:20:16 AM
71.660299	11:20:17 AM
71.6162699	11:20:18 AM
71.626147	11:20:19 AM
71.6371961	11:20:20 AM
71.7076201	11:20:21 AM
71.7655441	11:20:22 AM
71.8633119	11:20:23 AM
71.9595171	11:20:24 AM
72.0190037	11:20:25 AM
72.0460683	11:20:26 AM
72.0220825	11:20:27 AM

72.0016124	11:20:28 AM
71.9776266	11:20:29 AM
71.9274689	11:20:30 AM
71.8944988	11:20:31 AM
71.8757488	11:20:32 AM
71.8382488	11:20:33 AM
71.7726238	11:20:34 AM
71.7007488	11:20:35 AM
71.6319988	11:20:36 AM
71.5663738	11:20:37 AM
71.5516877	11:20:38 AM
71.5016877	11:20:39 AM
71.4798127	11:20:40 AM
71.4579377	11:20:41 AM
71.4579377	11:20:42 AM
71.4641877	11:20:43 AM
71.4766877	11:20:44 AM
71.4766877	11:20:45 AM
71.4798127	11:20:46 AM
71.4516877	11:20:47 AM
71.4079377	11:20:48 AM
71.3204377	11:20:49 AM
71.2016877	11:20:50 AM
71.0985627	11:20:51 AM
70.9798127	11:20:52 AM
70.8641877	11:20:53 AM
70.802236	11:20:54 AM
70.7508311	11:20:55 AM
70.7244263	11:20:56 AM
70.7003652	11:20:57 AM
70.6770853	11:20:58 AM
70.5967742	11:20:59 AM
70.5024007	11:21:00 AM
70.3884958	11:21:01 AM
70.290216	11:21:02 AM
70.1880299	11:21:03 AM
70.0877969	11:21:04 AM
70.0125639	11:21:05 AM
69.962331	11:21:06 AM
69.9175668	11:21:07 AM
69.8724119	11:21:08 AM
69.8307727	11:21:09 AM
69.7965554	11:21:10 AM
69.7451505	11:21:11 AM
69.6826505	11:21:12 AM

69.6295255	11:21:13 AM
69.5951505	11:21:14 AM
69.5951505	11:21:15 AM
69.6045255	11:21:16 AM
69.6170255	11:21:17 AM
69.6076505	11:21:18 AM
69.5889005	11:21:19 AM
69.5514005	11:21:20 AM
69.4920255	11:21:21 AM
69.4107755	11:21:22 AM
69.3295255	11:21:23 AM
69.2545255	11:21:24 AM
69.1951505	11:21:25 AM
69.1482755	11:21:26 AM
69.1107755	11:21:27 AM
69.0920255	11:21:28 AM
69.1139005	11:21:29 AM
69.1451505	11:21:30 AM
69.1607755	11:21:31 AM
69.1701505	11:21:32 AM
69.1732755	11:21:33 AM
69.1826505	11:21:34 AM
69.2076505	11:21:35 AM
69.2420255	11:21:36 AM
69.2795255	11:21:37 AM
69.3045255	11:21:38 AM
69.3295255	11:21:39 AM
69.3545255	11:21:40 AM
69.3795255	11:21:41 AM
69.3889005	11:21:42 AM
69.3920255	11:21:43 AM
69.3857755	11:21:44 AM
69.3732755	11:21:45 AM
69.3639005	11:21:46 AM
69.3545255	11:21:47 AM
69.3639005	11:21:48 AM
69.3826505	11:21:49 AM
69.4201505	11:21:50 AM
69.4576505	11:21:51 AM
69.4418673	11:21:52 AM
69.4327247	11:21:53 AM
69.4341289	11:21:54 AM
69.4460801	11:21:55 AM
69.4623281	11:21:56 AM
69.4957636	11:21:57 AM

69.5608398	11:21:58 AM
69.6577148	11:21:59 AM
69.7483398	11:22:00 AM
69.8452148	11:22:01 AM
69.9483398	11:22:02 AM
70.0483398	11:22:03 AM
70.1545898	11:22:04 AM
70.2827148	11:22:05 AM
70.4108398	11:22:06 AM
70.4985727	11:22:07 AM
70.5796651	11:22:08 AM
70.6256012	11:22:09 AM
70.6559123	11:22:10 AM
70.6799734	11:22:11 AM
70.6970032	11:22:12 AM
70.704658	11:22:13 AM
70.708016	11:22:14 AM
70.6871552	11:22:15 AM
70.6569194	11:22:16 AM
70.6192618	11:22:17 AM
70.5691041	11:22:18 AM
70.5240246	11:22:19 AM
70.4789451	11:22:20 AM
70.4408968	11:22:21 AM
70.4274579	11:22:22 AM
70.4210502	11:22:23 AM
70.4185488	11:22:24 AM
70.4234693	11:22:25 AM
70.432296	11:22:26 AM
70.4196383	11:22:27 AM
70.3921369	11:22:28 AM
70.3533074	11:22:29 AM
70.2761966	11:22:30 AM
70.1955702	11:22:31 AM
70.1572895	11:22:32 AM
70.1100245	11:22:33 AM
70.0658844	11:22:34 AM
70.01901	11:22:35 AM
69.9826824	11:22:36 AM
69.9463549	11:22:37 AM
69.9236992	11:22:38 AM
69.9174492	11:22:39 AM
69.9080742	11:22:40 AM
69.8830742	11:22:41 AM
69.8486992	11:22:42 AM

69.8236992	11:22:43 AM
69.7955742	11:22:44 AM
69.7393242	11:22:45 AM
69.6799492	11:22:46 AM
69.6765912	11:22:47 AM
69.6736239	11:22:48 AM
69.6683128	11:22:49 AM
69.6567517	11:22:50 AM
69.6420656	11:22:51 AM
69.6344108	11:22:52 AM
69.6330059	11:22:53 AM
69.654648	11:22:54 AM
69.6786338	11:22:55 AM
69.7119945	11:22:56 AM
69.7527772	11:22:57 AM
69.8091849	11:22:58 AM
69.8855144	11:22:59 AM
69.9837189	11:23:00 AM
70.0748922	11:23:01 AM
70.1695811	11:23:02 AM
70.2634888	11:23:03 AM
70.3503652	11:23:04 AM
70.4298197	11:23:05 AM
70.470993	11:23:06 AM
70.5149007	11:23:07 AM
70.5392771	11:23:08 AM
70.5593566	11:23:09 AM
70.5895924	11:23:10 AM
70.6327188	11:23:11 AM
70.6742827	11:23:12 AM
70.7306904	11:23:13 AM
70.7640511	11:23:14 AM
70.7989744	11:23:15 AM
70.825304	11:23:16 AM
70.837571	11:23:17 AM
70.8295255	11:23:18 AM
70.7732755	11:23:19 AM
70.7357755	11:23:20 AM
70.7326505	11:23:21 AM
70.7482755	11:23:22 AM
70.7639005	11:23:23 AM
70.7920255	11:23:24 AM
70.8326505	11:23:25 AM
70.8732755	11:23:26 AM
70.9045255	11:23:27 AM

70.9357755	11:23:28 AM
70.9889005	11:23:29 AM
71.0607755	11:23:30 AM
71.1670255	11:23:31 AM
71.2514005	11:23:32 AM
71.3295255	11:23:33 AM
71.3857755	11:23:34 AM
71.4482755	11:23:35 AM
71.4982755	11:23:36 AM
71.5326505	11:23:37 AM
71.4680397	11:23:38 AM
71.365929	11:23:39 AM
71.2571775	11:23:40 AM
71.1503793	11:23:41 AM
71.0240497	11:23:42 AM
70.8914702	11:23:43 AM
70.7631875	11:23:44 AM
70.6138111	11:23:45 AM
70.4820128	11:23:46 AM
70.3537302	11:23:47 AM
70.2441975	11:23:48 AM
70.1346649	11:23:49 AM
70.030601	11:23:50 AM
69.9171621	11:23:51 AM
69.8037231	11:23:52 AM
69.6883311	11:23:53 AM
69.5944235	11:23:54 AM
69.4852814	11:23:55 AM
69.3737956	11:23:56 AM
69.2650442	11:23:57 AM
69.1562928	11:23:58 AM
69.0537914	11:23:59 AM
68.9837119	11:24:00 AM
68.907773	11:24:01 AM
68.8294903	11:24:02 AM
68.7558952	11:24:03 AM
68.6830813	11:24:04 AM
68.6102673	11:24:05 AM
68.5413597	11:24:06 AM
68.481827	11:24:07 AM
68.41956	11:24:08 AM
68.3334648	11:24:09 AM
68.2286197	11:24:10 AM
68.0933058	11:24:11 AM
67.9822106	11:24:12 AM

67.9023655	11:24:13 AM
67.8494734	11:24:14 AM
67.8110345	11:24:15 AM
67.7854862	11:24:16 AM
67.7704848	11:24:17 AM
67.847438	11:24:18 AM
67.9372817	11:24:19 AM
68.0177505	11:24:20 AM
68.0997817	11:24:21 AM
68.197438	11:24:22 AM
68.2990005	11:24:23 AM
68.4142348	11:24:24 AM
68.5790786	11:24:25 AM
68.7626723	11:24:26 AM
68.9466567	11:24:27 AM
69.1345473	11:24:28 AM
69.38783	11:24:29 AM
69.6120487	11:24:30 AM
69.8786126	11:24:31 AM
70.1514266	11:24:32 AM
70.4261936	11:24:33 AM
70.6982262	11:24:34 AM
70.9792433	11:24:35 AM
71.2657291	11:24:36 AM
71.5401055	11:24:37 AM
71.8176069	11:24:38 AM
72.0982333	11:24:39 AM
72.3464378	11:24:40 AM
72.5817517	11:24:41 AM
72.7975344	11:24:42 AM
72.9463625	11:24:43 AM
73.0686281	11:24:44 AM
73.1783938	11:24:45 AM
73.2819094	11:24:46 AM
73.3596438	11:24:47 AM
73.4334719	11:24:48 AM
73.5147219	11:24:49 AM
73.604175	11:24:50 AM
73.6823	11:24:51 AM
73.7424563	11:24:52 AM
73.7748781	11:24:53 AM
73.754175	11:24:54 AM
73.7057375	11:24:55 AM
73.6346438	11:24:56 AM
73.5580813	11:24:57 AM

73.4764406	11:24:58 AM
73.404175	11:24:59 AM
73.3369875	11:25:00 AM
73.2608156	11:25:01 AM
73.1865969	11:25:02 AM
73.1330813	11:25:03 AM
73.091675	11:25:04 AM
73.0381594	11:25:05 AM
72.9877688	11:25:06 AM
72.9576906	11:25:07 AM
72.9147219	11:25:08 AM
72.8087048	11:25:09 AM
72.7215955	11:25:10 AM
72.5862816	11:25:11 AM
72.4529208	11:25:12 AM
72.3273725	11:25:13 AM
72.2092461	11:25:14 AM
72.0965884	11:25:15 AM
71.9698683	11:25:16 AM
71.8361169	11:25:17 AM
71.7204919	11:25:18 AM
71.5954919	11:25:19 AM
71.4611169	11:25:20 AM
71.3267419	11:25:21 AM
71.1923669	11:25:22 AM
71.1140089	11:25:23 AM
71.0458072	11:25:24 AM
70.9932305	11:25:25 AM
70.9429975	11:25:26 AM
70.8966708	11:25:27 AM
70.8476097	11:25:28 AM
70.7930798	11:25:29 AM
70.7303469	11:25:30 AM
70.6781608	11:25:31 AM
70.6353497	11:25:32 AM
70.592148	11:25:33 AM
70.5813681	11:25:34 AM
70.5807445	11:25:35 AM
70.5648866	11:25:36 AM
70.5345755	11:25:37 AM
70.5007488	11:25:38 AM
70.4665314	11:25:39 AM
70.4307516	11:25:40 AM
70.3848155	11:25:41 AM
70.3314575	11:25:42 AM

70.2691152	11:25:43 AM
70.1860697	11:25:44 AM
70.099118	11:25:45 AM
69.9977132	11:25:46 AM
69.8845896	11:25:47 AM
69.7835753	11:25:48 AM
69.6645924	11:25:49 AM
69.5565469	11:25:50 AM
69.4481108	11:25:51 AM
69.3470966	11:25:52 AM
69.2351449	11:25:53 AM
69.1126463	11:25:54 AM
68.981554	11:25:55 AM
68.8551491	11:25:56 AM
68.7639005	11:25:57 AM
68.6670255	11:25:58 AM
68.5701505	11:25:59 AM
68.4732755	11:26:00 AM
68.3857755	11:26:01 AM
68.3295255	11:26:02 AM
68.3076505	11:26:03 AM
68.3014005	11:26:04 AM
68.2920255	11:26:05 AM
68.3076505	11:26:06 AM
68.3031662	11:26:07 AM
68.2924319	11:26:08 AM
68.2809164	11:26:09 AM
68.2838539	11:26:10 AM
68.2938228	11:26:11 AM
68.3469478	11:26:12 AM
68.3813228	11:26:13 AM
68.4125728	11:26:14 AM
68.4469478	11:26:15 AM
68.4875728	11:26:16 AM
68.5563228	11:26:17 AM
68.6438228	11:26:18 AM
68.7313228	11:26:19 AM
68.8188228	11:26:20 AM
68.9219478	11:26:21 AM
69.0250728	11:26:22 AM
69.1063228	11:26:23 AM
69.1844478	11:26:24 AM
69.2531978	11:26:25 AM
69.3469478	11:26:26 AM
69.4500728	11:26:27 AM

69.5438228	11:26:28 AM
69.6563228	11:26:29 AM
69.7563228	11:26:30 AM
69.8406978	11:26:31 AM
69.9344478	11:26:32 AM
69.971095	11:26:33 AM
69.9928984	11:26:34 AM
70.0072799	11:26:35 AM
70.0083803	11:26:36 AM
69.9872149	11:26:37 AM
69.9578465	11:26:38 AM
69.9351187	11:26:39 AM
69.9213752	11:26:40 AM
69.9013818	11:26:41 AM
69.9326318	11:26:42 AM
69.9357568	11:26:43 AM
69.9232568	11:26:44 AM
69.9138818	11:26:45 AM
69.8732568	11:26:46 AM
69.8652411	11:26:47 AM
69.8666004	11:26:48 AM
69.8749909	11:26:49 AM
69.8814284	11:26:50 AM
69.8839595	11:26:51 AM
69.8495845	11:26:52 AM
69.8464595	11:26:53 AM
69.8402095	11:26:54 AM
69.8683345	11:26:55 AM
69.8995845	11:26:56 AM
69.862649	11:26:57 AM
69.8202447	11:26:58 AM
69.7891686	11:26:59 AM
69.7557487	11:27:00 AM
69.7285788	11:27:01 AM
69.6932058	11:27:02 AM
69.7213308	11:27:03 AM
69.7400808	11:27:04 AM
69.7650808	11:27:05 AM
69.7807058	11:27:06 AM
69.7838308	11:27:07 AM
69.7838308	11:27:08 AM
69.7807058	11:27:09 AM
69.7932058	11:27:10 AM
69.8338308	11:27:11 AM
69.8713308	11:27:12 AM

69.9815586	11:27:13 AM
70.1128802	11:27:14 AM
70.2547486	11:27:15 AM
70.4161483	11:27:16 AM
70.5841887	11:27:17 AM
70.7354321	11:27:18 AM
70.8706599	11:27:19 AM
71.0062784	11:27:20 AM
71.1418968	11:27:21 AM
71.1918968	11:27:22 AM
71.2293968	11:27:23 AM
71.2856468	11:27:24 AM
71.3231468	11:27:25 AM
71.3668968	11:27:26 AM
71.4262718	11:27:27 AM
71.4856468	11:27:28 AM
71.5606468	11:27:29 AM
71.6325218	11:27:30 AM
71.6950218	11:27:31 AM
71.7825218	11:27:32 AM
71.8201957	11:27:33 AM
71.8746664	11:27:34 AM
71.8947621	11:27:35 AM
71.9281391	11:27:36 AM
72.013686	11:27:37 AM
72.0988422	11:27:38 AM
72.173061	11:27:39 AM
72.2566547	11:27:40 AM
72.354311	11:27:41 AM
72.4714985	11:27:42 AM
72.5232349	11:27:43 AM
72.58044	11:27:44 AM
72.6286607	11:27:45 AM
72.6745377	11:27:46 AM
72.748149	11:27:47 AM
72.8557448	11:27:48 AM
72.9953718	11:27:49 AM
73.1814831	11:27:50 AM
73.3327573	11:27:51 AM
73.466844	11:27:52 AM
73.5860869	11:27:53 AM
73.7115798	11:27:54 AM
73.8547224	11:27:55 AM
73.9795056	11:27:56 AM
74.0706951	11:27:57 AM

74.1431346	11:27:58 AM
74.2034647	11:27:59 AM
74.2462167	11:28:00 AM
74.2889687	11:28:01 AM
74.3535957	11:28:02 AM
74.4248633	11:28:03 AM
74.4910528	11:28:04 AM
74.5615391	11:28:05 AM
74.6249942	11:28:06 AM
74.6829806	11:28:07 AM
74.7573732	11:28:08 AM
74.8266877	11:28:09 AM
74.8438616	11:28:10 AM
74.8719731	11:28:11 AM
74.8930533	11:28:12 AM
75.011022	11:28:13 AM
75.1082876	11:28:14 AM
75.2231314	11:28:15 AM
75.3434439	11:28:16 AM
75.4211783	11:28:17 AM
75.4813345	11:28:18 AM
75.5274283	11:28:19 AM
75.5692251	11:28:20 AM
75.579772	11:28:21 AM
75.5727408	11:28:22 AM
75.5750845	11:28:23 AM
75.570397	11:28:24 AM
75.5618033	11:28:25 AM
75.5426626	11:28:26 AM
75.5036001	11:28:27 AM
75.4407095	11:28:28 AM
75.3489126	11:28:29 AM
75.232897	11:28:30 AM
75.1485936	11:28:31 AM
75.062337	11:28:32 AM
74.971393	11:28:33 AM
74.8714645	11:28:34 AM
74.7550583	11:28:35 AM
74.6507614	11:28:36 AM
74.5640426	11:28:37 AM
74.5046676	11:28:38 AM
74.4734176	11:28:39 AM
74.4601364	11:28:40 AM
74.4464645	11:28:41 AM
74.4195114	11:28:42 AM

74.3956833	11:28:43 AM
74.3613083	11:28:44 AM
74.3413864	11:28:45 AM
74.3312301	11:28:46 AM
74.3202926	11:28:47 AM
74.2988083	11:28:48 AM
74.2765426	11:28:49 AM
74.3036832	11:28:50 AM
74.325355	11:28:51 AM
74.2989799	11:28:52 AM
74.2118705	11:28:53 AM
74.1423393	11:28:54 AM
74.0622612	11:28:55 AM
73.9653862	11:28:56 AM
73.9212455	11:28:57 AM
73.9052299	11:28:58 AM
73.8935112	11:28:59 AM
73.885308	11:29:00 AM
73.8806205	11:29:01 AM
73.8716362	11:29:02 AM
73.8712455	11:29:03 AM
73.8677299	11:29:04 AM
73.8579643	11:29:05 AM
73.8556205	11:29:06 AM
73.8454643	11:29:07 AM
73.8435112	11:29:08 AM
73.8423393	11:29:09 AM
73.807183	11:29:10 AM
73.7646049	11:29:11 AM
73.7208549	11:29:12 AM
73.685308	11:29:13 AM
73.6360893	11:29:14 AM
73.5896049	11:29:15 AM
73.5403862	11:29:16 AM
73.4966362	11:29:17 AM
73.4833549	11:29:18 AM
73.4888237	11:29:19 AM
73.5005424	11:29:20 AM
73.5130424	11:29:21 AM
73.5399955	11:29:22 AM
73.563433	11:29:23 AM
73.5903862	11:29:24 AM
73.6220268	11:29:25 AM
73.6583549	11:29:26 AM
73.6856987	11:29:27 AM

73.7067924	11:29:28 AM
73.7231987	11:29:29 AM
73.7423393	11:29:30 AM
73.7407768	11:29:31 AM
73.7458549	11:29:32 AM
73.7442924	11:29:33 AM
73.7189018	11:29:34 AM
73.6966362	11:29:35 AM
73.6849174	11:29:36 AM
73.6626518	11:29:37 AM
73.597808	11:29:38 AM
73.5235893	11:29:39 AM
73.4243705	11:29:40 AM
73.3235893	11:29:41 AM
73.2396049	11:29:42 AM
73.1657768	11:29:43 AM
73.1024955	11:29:44 AM
73.0419487	11:29:45 AM
72.960308	11:29:46 AM
72.885308	11:29:47 AM
72.7942924	11:29:48 AM
72.7064018	11:29:49 AM
72.6204643	11:29:50 AM
72.550933	11:29:51 AM
72.4833549	11:29:52 AM
72.4149955	11:29:53 AM
72.357183	11:29:54 AM
72.288433	11:29:55 AM
72.216558	11:29:56 AM
72.1360893	11:29:57 AM
71.9939018	11:29:58 AM
71.8220268	11:29:59 AM
71.6513237	11:30:00 AM
71.4841362	11:30:01 AM
71.3110893	11:30:02 AM
71.1481987	11:30:03 AM
70.9923393	11:30:04 AM
70.8220268	11:30:05 AM
70.6407768	11:30:06 AM
70.4657768	11:30:07 AM
70.2903862	11:30:08 AM
70.1052299	11:30:09 AM
69.9157768	11:30:10 AM
69.7505424	11:30:11 AM
69.613433	11:30:12 AM

69.5087455	11:30:13 AM
69.4282768	11:30:14 AM
69.3587455	11:30:15 AM
69.3067924	11:30:16 AM
69.2587455	11:30:17 AM
69.2528862	11:30:18 AM
69.266558	11:30:19 AM
69.2810112	11:30:20 AM
69.3001518	11:30:21 AM
69.3024955	11:30:22 AM
69.2939018	11:30:23 AM
69.2521049	11:30:24 AM
69.2118705	11:30:25 AM
69.1849174	11:30:26 AM
69.157183	11:30:27 AM
69.1399955	11:30:28 AM
69.1251518	11:30:29 AM
69.1181205	11:30:30 AM
69.1220268	11:30:31 AM
69.1368705	11:30:32 AM
69.1618705	11:30:33 AM
69.1892143	11:30:34 AM
69.2138237	11:30:35 AM
69.250933	11:30:36 AM
69.2798393	11:30:37 AM
69.3325737	11:30:38 AM
69.3778862	11:30:39 AM
69.4511498	11:30:40 AM
69.4642572	11:30:41 AM
69.4508021	11:30:42 AM
69.4271907	11:30:43 AM
69.3930325	11:30:44 AM
69.3748899	11:30:45 AM
69.3602629	11:30:46 AM
69.3421203	11:30:47 AM
69.3341339	11:30:48 AM
69.3402101	11:30:49 AM
69.3599581	11:30:50 AM
69.3863467	11:30:51 AM
69.4017979	11:30:52 AM
69.3863896	11:30:53 AM
69.3287939	11:30:54 AM
69.2469794	11:30:55 AM
69.1475868	11:30:56 AM
69.0513192	11:30:57 AM

68.9351297	11:30:58 AM
68.821284	11:30:59 AM
68.731657	11:31:00 AM
68.6682019	11:31:01 AM
68.6309186	11:31:02 AM
68.615901	11:31:03 AM
68.6469772	11:31:04 AM
68.6866471	11:31:05 AM
68.7458482	11:31:06 AM
68.80544	11:31:07 AM
68.8708911	11:31:08 AM
68.9238422	11:31:09 AM
68.9943715	11:31:10 AM
69.0512289	11:31:11 AM
69.0873832	11:31:12 AM
69.1036156	11:31:13 AM
69.1077386	11:31:14 AM
69.1185022	11:31:15 AM
69.1038752	11:31:16 AM
69.119717	11:31:17 AM
69.1500119	11:31:18 AM
69.1826505	11:31:19 AM
69.1826505	11:31:20 AM
69.2264005	11:31:21 AM
69.2826505	11:31:22 AM
69.3389005	11:31:23 AM
69.3982755	11:31:24 AM
69.4420255	11:31:25 AM
69.4795255	11:31:26 AM
69.5232755	11:31:27 AM
69.5639005	11:31:28 AM
69.5951505	11:31:29 AM
69.6232755	11:31:30 AM
69.6482755	11:31:31 AM
69.6826505	11:31:32 AM
69.678161	11:31:33 AM
69.6982809	11:31:34 AM
69.7889059	11:31:35 AM
69.8420309	11:31:36 AM
69.8920309	11:31:37 AM
69.9576559	11:31:38 AM
70.0201559	11:31:39 AM
70.0732809	11:31:40 AM
70.1170309	11:31:41 AM
70.1482809	11:31:42 AM

70.1482809	11:31:43 AM
70.1482809	11:31:44 AM
70.1607809	11:31:45 AM
70.1701559	11:31:46 AM
70.1920309	11:31:47 AM
70.2139059	11:31:48 AM
70.2326559	11:31:49 AM
70.2389059	11:31:50 AM
70.2482809	11:31:51 AM
70.2576559	11:31:52 AM
70.2639059	11:31:53 AM
70.2545309	11:31:54 AM
70.2264059	11:31:55 AM
70.2139059	11:31:56 AM
70.1701559	11:31:57 AM
70.1201559	11:31:58 AM
70.0889059	11:31:59 AM
70.0232809	11:32:00 AM
69.9670309	11:32:01 AM
69.9170309	11:32:02 AM
69.8764059	11:32:03 AM
69.8389059	11:32:04 AM
69.7951559	11:32:05 AM
69.7920309	11:32:06 AM
69.7920309	11:32:07 AM
69.7951559	11:32:08 AM
69.8139059	11:32:09 AM
69.793406	11:32:10 AM
69.7643123	11:32:11 AM
69.7215468	11:32:12 AM
69.7666613	11:32:13 AM
69.7541371	11:32:14 AM
69.6777485	11:32:15 AM
69.6247973	11:32:16 AM
69.5749712	11:32:17 AM
69.5499712	11:32:18 AM
69.5249712	11:32:19 AM
69.5030962	11:32:20 AM
69.4749712	11:32:21 AM
69.4530962	11:32:22 AM
69.4405962	11:32:23 AM
69.4499712	11:32:24 AM
69.4155962	11:32:25 AM
69.3780962	11:32:26 AM
69.3155962	11:32:27 AM

69.2331027	11:32:28 AM
69.1670156	11:32:29 AM
69.085694	11:32:30 AM
69.0055443	11:32:31 AM
68.9457071	11:32:32 AM
68.8968074	11:32:33 AM
68.876814	11:32:34 AM
68.9142424	11:32:35 AM
68.9594833	11:32:36 AM
69.0230836	11:32:37 AM
69.0968402	11:32:38 AM
69.1748936	11:32:39 AM
69.2845877	11:32:40 AM
69.3966255	11:32:41 AM
69.5074914	11:32:42 AM
69.5902323	11:32:43 AM
69.659692	11:32:44 AM
69.726808	11:32:45 AM
69.7548614	11:32:46 AM
69.8329864	11:32:47 AM
69.8829864	11:32:48 AM
69.9204864	11:32:49 AM
69.9722363	11:32:50 AM
70.01383	11:32:51 AM
70.0084771	11:32:52 AM
70.0241021	11:32:53 AM
70.0832317	11:32:54 AM
70.1544706	11:32:55 AM
70.2397721	11:32:56 AM
70.3645982	11:32:57 AM
70.4302232	11:32:58 AM
70.4927232	11:32:59 AM
70.5364732	11:33:00 AM
70.5802232	11:33:01 AM
70.6177232	11:33:02 AM
70.6427232	11:33:03 AM
70.6145982	11:33:04 AM
70.6083482	11:33:05 AM
70.5833482	11:33:06 AM
70.5770982	11:33:07 AM
70.5908416	11:33:08 AM
70.5913038	11:33:09 AM
70.6195004	11:33:10 AM
70.6434001	11:33:11 AM
70.6626123	11:33:12 AM

70.686512	11:33:13 AM
70.7158804	11:33:14 AM
70.703452	11:33:15 AM
70.6925861	11:33:16 AM
70.6914858	11:33:17 AM
70.6771042	11:33:18 AM
70.6615508	11:33:19 AM
70.6424817	11:33:20 AM
70.6210689	11:33:21 AM
70.603953	11:33:22 AM
70.618087	11:33:23 AM
70.6361274	11:33:24 AM
70.6565114	11:33:25 AM
70.678458	11:33:26 AM
70.637833	11:33:27 AM
70.606583	11:33:28 AM
70.575333	11:33:29 AM
70.556583	11:33:30 AM
70.553458	11:33:31 AM
70.6015764	11:33:32 AM
70.5797014	11:33:33 AM
70.5817261	11:33:34 AM
70.5775008	11:33:35 AM
70.5732755	11:33:36 AM
70.5264005	11:33:37 AM
70.5014005	11:33:38 AM
70.4701505	11:33:39 AM
70.4514005	11:33:40 AM
70.4326505	11:33:41 AM
70.4107755	11:33:42 AM
70.4107755	11:33:43 AM
70.4389005	11:33:44 AM
70.4576505	11:33:45 AM
70.4701505	11:33:46 AM
70.4607755	11:33:47 AM
70.4264005	11:33:48 AM
70.3920255	11:33:49 AM
70.3326505	11:33:50 AM
70.2764005	11:33:51 AM
70.2107755	11:33:52 AM
70.1389005	11:33:53 AM
70.0357755	11:33:54 AM
69.9264005	11:33:55 AM
69.8045255	11:33:56 AM
69.6795255	11:33:57 AM

69.5670255	11:33:58 AM
69.4607755	11:33:59 AM
69.3639005	11:34:00 AM
69.2795255	11:34:01 AM
69.2076505	11:34:02 AM
69.1232755	11:34:03 AM
69.0326505	11:34:04 AM
68.9795255	11:34:05 AM
68.9670255	11:34:06 AM
68.9514005	11:34:07 AM
68.9576505	11:34:08 AM
68.9670255	11:34:09 AM
68.9732755	11:34:10 AM
68.9670255	11:34:11 AM
68.9514005	11:34:12 AM
68.9326505	11:34:13 AM
68.9107755	11:34:14 AM
68.8764005	11:34:15 AM
68.8514005	11:34:16 AM
68.8389005	11:34:17 AM
68.8357755	11:34:18 AM
68.8451505	11:34:19 AM
68.8857755	11:34:20 AM
68.9295255	11:34:21 AM
68.9732755	11:34:22 AM
68.9951505	11:34:23 AM
69.0014005	11:34:24 AM
69.0139005	11:34:25 AM
69.0357755	11:34:26 AM
69.0670255	11:34:27 AM
69.0920255	11:34:28 AM
69.1076505	11:34:29 AM
69.1139005	11:34:30 AM
69.0826505	11:34:31 AM
69.0514005	11:34:32 AM
69.0107755	11:34:33 AM
68.9920255	11:34:34 AM
68.9826505	11:34:35 AM
68.9795255	11:34:36 AM
68.9795255	11:34:37 AM
68.9764005	11:34:38 AM
68.9670255	11:34:39 AM
68.9732755	11:34:40 AM
68.9701505	11:34:41 AM
68.9514005	11:34:42 AM

68.9420255	11:34:43 AM
68.9420255	11:34:44 AM
68.9107755	11:34:45 AM
68.8389005	11:34:46 AM
68.7764005	11:34:47 AM
68.7201505	11:34:48 AM
68.6576505	11:34:49 AM
68.6264005	11:34:50 AM
68.6170255	11:34:51 AM
68.6076505	11:34:52 AM
68.6107755	11:34:53 AM
68.6076505	11:34:54 AM
68.6107755	11:34:55 AM
68.6014005	11:34:56 AM
68.5857755	11:34:57 AM
68.5576505	11:34:58 AM
68.5170255	11:34:59 AM
68.4545255	11:35:00 AM
68.3826505	11:35:01 AM
68.3264005	11:35:02 AM
68.2732755	11:35:03 AM
68.2357755	11:35:04 AM
68.2045255	11:35:05 AM
68.1826505	11:35:06 AM
68.1764005	11:35:07 AM
68.1857755	11:35:08 AM
68.2045255	11:35:09 AM
68.2482755	11:35:10 AM
68.3389005	11:35:11 AM
68.4514005	11:35:12 AM
68.5857755	11:35:13 AM
68.7201505	11:35:14 AM
68.7703244	11:35:15 AM
68.7974514	11:35:16 AM
68.8152033	11:35:17 AM
68.8368616	11:35:18 AM
68.8507073	11:35:19 AM
68.8850823	11:35:20 AM
68.9225823	11:35:21 AM
68.9475823	11:35:22 AM
68.9632073	11:35:23 AM
68.9663323	11:35:24 AM
68.9600823	11:35:25 AM
68.9569573	11:35:26 AM
68.9569573	11:35:27 AM

68.9475823	11:35:28 AM
68.9569573	11:35:29 AM
68.9538323	11:35:30 AM
68.9413323	11:35:31 AM
68.9350823	11:35:32 AM
68.9100823	11:35:33 AM
68.8975823	11:35:34 AM
68.8757073	11:35:35 AM
68.8694573	11:35:36 AM
68.8632073	11:35:37 AM
68.8757073	11:35:38 AM
68.8975823	11:35:39 AM
68.9288323	11:35:40 AM
68.9632073	11:35:41 AM
68.9694573	11:35:42 AM
68.9788323	11:35:43 AM
68.9819573	11:35:44 AM
68.9913323	11:35:45 AM
68.9944573	11:35:46 AM
68.9850823	11:35:47 AM
68.9819573	11:35:48 AM
68.9788323	11:35:49 AM
68.9632073	11:35:50 AM
68.9319573	11:35:51 AM
68.8850823	11:35:52 AM
68.8350823	11:35:53 AM
68.7913323	11:35:54 AM
68.8380335	11:35:55 AM
68.9077815	11:35:56 AM
68.9681545	11:35:57 AM
69.0089963	11:35:58 AM
69.0420255	11:35:59 AM
69.0170255	11:36:00 AM
68.9982755	11:36:01 AM
68.9982755	11:36:02 AM
68.9857755	11:36:03 AM
68.9826505	11:36:04 AM
68.9920255	11:36:05 AM
68.9982755	11:36:06 AM
68.9920255	11:36:07 AM
68.9795255	11:36:08 AM
68.9576505	11:36:09 AM
68.9420255	11:36:10 AM
68.9326505	11:36:11 AM
68.9357755	11:36:12 AM

68.9514005	11:36:13 AM
68.9482755	11:36:14 AM
68.9451505	11:36:15 AM
68.9170255	11:36:16 AM
68.8732755	11:36:17 AM
68.8139005	11:36:18 AM
68.7482755	11:36:19 AM
68.6576505	11:36:20 AM
68.5420255	11:36:21 AM
68.4295255	11:36:22 AM
68.3139005	11:36:23 AM
68.1920255	11:36:24 AM
68.0670255	11:36:25 AM
67.9420255	11:36:26 AM
67.8264005	11:36:27 AM
67.7170255	11:36:28 AM
67.6201505	11:36:29 AM
67.5264005	11:36:30 AM
67.4420255	11:36:31 AM
67.3639005	11:36:32 AM
67.2732755	11:36:33 AM
67.1670255	11:36:34 AM
67.0514005	11:36:35 AM
66.9451505	11:36:36 AM
66.8514005	11:36:37 AM
66.7795255	11:36:38 AM
66.7139005	11:36:39 AM
66.6670255	11:36:40 AM
66.6107755	11:36:41 AM
66.5545255	11:36:42 AM
66.5264005	11:36:43 AM
66.5014005	11:36:44 AM
66.4920255	11:36:45 AM
66.5372664	11:36:46 AM
66.5914918	11:36:47 AM
66.6625139	11:36:48 AM
66.7249424	11:36:49 AM
66.7764333	11:36:50 AM
66.8294867	11:36:51 AM
66.8813683	11:36:52 AM
66.9430155	11:36:53 AM
67.0249752	11:36:54 AM
67.1135755	11:36:55 AM
67.2072539	11:36:56 AM
67.3064011	11:36:57 AM

67.4059389	11:36:58 AM
67.5046954	11:36:59 AM
67.5952489	11:37:00 AM
67.6865835	11:37:01 AM
67.7759651	11:37:02 AM
67.8676904	11:37:03 AM
67.9648845	11:37:04 AM
68.0581723	11:37:05 AM
68.1604445	11:37:06 AM
68.2627166	11:37:07 AM
68.3864732	11:37:08 AM
68.517261	11:37:09 AM
68.6484394	11:37:10 AM
68.7905554	11:37:11 AM
68.9264213	11:37:12 AM
69.1076713	11:37:13 AM
69.2826713	11:37:14 AM
69.4576713	11:37:15 AM
69.5785483	11:37:16 AM
69.6900503	11:37:17 AM
69.7956929	11:37:18 AM
69.8876636	11:37:19 AM
69.9733843	11:37:20 AM
70.0591051	11:37:21 AM
70.1127946	11:37:22 AM
70.122734	11:37:23 AM
70.121736	11:37:24 AM
70.0805036	11:37:25 AM
69.9877803	11:37:26 AM
69.891932	11:37:27 AM
69.7828024	11:37:28 AM
69.6791416	11:37:29 AM
69.5789964	11:37:30 AM
69.474945	11:37:31 AM
69.3658154	11:37:32 AM
69.2562952	11:37:33 AM
69.2024605	11:37:34 AM
69.1357352	11:37:35 AM
69.0764318	11:37:36 AM
69.0304096	11:37:37 AM
69.0027468	11:37:38 AM
69.0008652	11:37:39 AM
69.0321868	11:37:40 AM
69.0814771	11:37:41 AM
69.0872206	11:37:42 AM

69.1007767	11:37:43 AM
69.1221452	11:37:44 AM
69.1798418	11:37:45 AM
69.2594135	11:37:46 AM
69.4008913	11:37:47 AM
69.5177597	11:37:48 AM
69.6338469	11:37:49 AM
69.7204498	11:37:50 AM
69.7836152	11:37:51 AM
69.8413119	11:37:52 AM
69.8290151	11:37:53 AM
69.8089058	11:37:54 AM
69.7575465	11:37:55 AM
69.7290602	11:37:56 AM
69.6954958	11:37:57 AM
69.6443532	11:37:58 AM
69.6209451	11:37:59 AM
69.6076932	11:38:00 AM
69.5838944	11:38:01 AM
69.5917362	11:38:02 AM
69.6163749	11:38:03 AM
69.6582012	11:38:04 AM
69.7140899	11:38:05 AM
69.7684161	11:38:06 AM
69.8662891	11:38:07 AM
69.9887715	11:38:08 AM
70.1206289	11:38:09 AM
70.2630332	11:38:10 AM
70.3984062	11:38:11 AM
70.5337792	11:38:12 AM
70.6165917	11:38:13 AM
70.6191093	11:38:14 AM
70.6134238	11:38:15 AM
70.5936758	11:38:16 AM
70.574709	11:38:17 AM
70.5440235	11:38:18 AM
70.491463	11:38:19 AM
70.4295275	11:38:20 AM
70.3777482	11:38:21 AM
70.3773282	11:38:22 AM
70.376127	11:38:23 AM
70.3561758	11:38:24 AM
70.3096621	11:38:25 AM
70.2778182	11:38:26 AM
70.1778182	11:38:27 AM

70.0590682	11:38:28 AM
69.9121932	11:38:29 AM
69.7850369	11:38:30 AM
69.6860056	11:38:31 AM
69.583068	11:38:32 AM
69.5234898	11:38:33 AM
69.4810991	11:38:34 AM
69.4918334	11:38:35 AM
69.5275677	11:38:36 AM
69.5558801	11:38:37 AM
69.5951301	11:38:38 AM
69.6140675	11:38:39 AM
69.6103487	11:38:40 AM
69.6015517	11:38:41 AM
69.5775204	11:38:42 AM
69.5523172	11:38:43 AM
69.517739	11:38:44 AM
69.4937077	11:38:45 AM
69.4743638	11:38:46 AM
69.4149888	11:38:47 AM
69.3399888	11:38:48 AM
69.2462388	11:38:49 AM
69.1462388	11:38:50 AM
69.0493638	11:38:51 AM
68.9431138	11:38:52 AM
68.8956743	11:38:53 AM
68.8587817	11:38:54 AM
68.8457172	11:38:55 AM
68.8435903	11:38:56 AM
68.834432	11:38:57 AM
68.8182425	11:38:58 AM
68.8176781	11:38:59 AM
68.8202386	11:39:00 AM
68.8220178	11:39:01 AM
68.8335627	11:39:02 AM
68.8544826	11:39:03 AM
68.8839963	11:39:04 AM
68.9107755	11:39:05 AM
68.9107755	11:39:06 AM
68.8701505	11:39:07 AM
68.8389005	11:39:08 AM
68.8232755	11:39:09 AM
68.8170255	11:39:10 AM
68.7951505	11:39:11 AM
68.7889005	11:39:12 AM

68.7451505	11:39:13 AM
68.7170255	11:39:14 AM
68.6920255	11:39:15 AM
68.6701505	11:39:16 AM
68.6670255	11:39:17 AM
68.6639005	11:39:18 AM
68.6764005	11:39:19 AM
68.6889005	11:39:20 AM
68.7201505	11:39:21 AM
68.7701505	11:39:22 AM
68.8264005	11:39:23 AM
68.8826505	11:39:24 AM
68.9201505	11:39:25 AM
68.9451505	11:39:26 AM
68.9670255	11:39:27 AM
68.9826505	11:39:28 AM
68.9951505	11:39:29 AM
68.9889005	11:39:30 AM
68.9732755	11:39:31 AM
68.9607755	11:39:32 AM
68.9264005	11:39:33 AM
68.8889005	11:39:34 AM
68.8576505	11:39:35 AM
68.8295255	11:39:36 AM
68.8107755	11:39:37 AM
68.8045255	11:39:38 AM
68.7826505	11:39:39 AM
68.7514005	11:39:40 AM
68.7201505	11:39:41 AM
68.6826505	11:39:42 AM
68.6482755	11:39:43 AM
68.6201505	11:39:44 AM
68.5889005	11:39:45 AM
68.5357755	11:39:46 AM
68.5232755	11:39:47 AM
68.5264005	11:39:48 AM
68.5295255	11:39:49 AM
68.5389005	11:39:50 AM
68.5420255	11:39:51 AM
68.5389005	11:39:52 AM
68.5639005	11:39:53 AM
68.5701505	11:39:54 AM
68.5451505	11:39:55 AM
68.5076505	11:39:56 AM
68.4639005	11:39:57 AM

68.4139005	11:39:58 AM
68.3639005	11:39:59 AM
68.3295255	11:40:00 AM
68.2889005	11:40:01 AM
68.2545255	11:40:02 AM
68.2732755	11:40:03 AM
68.2826505	11:40:04 AM
68.3045255	11:40:05 AM
68.3295255	11:40:06 AM
68.3514005	11:40:07 AM
68.3701505	11:40:08 AM
68.3951505	11:40:09 AM
68.4420255	11:40:10 AM
68.5014005	11:40:11 AM
68.5670255	11:40:12 AM
68.6295255	11:40:13 AM
68.7045255	11:40:14 AM
68.7826505	11:40:15 AM
68.8670255	11:40:16 AM
68.9420255	11:40:17 AM
69.0107755	11:40:18 AM
69.0826505	11:40:19 AM
69.1545255	11:40:20 AM
69.2264005	11:40:21 AM
69.3107755	11:40:22 AM
69.3951505	11:40:23 AM
69.4764005	11:40:24 AM
69.5639005	11:40:25 AM
69.6607755	11:40:26 AM
69.7576505	11:40:27 AM
69.8514005	11:40:28 AM
69.9389005	11:40:29 AM
70.0014005	11:40:30 AM
70.0514005	11:40:31 AM
70.0951505	11:40:32 AM
70.1326505	11:40:33 AM
70.1795255	11:40:34 AM
70.2295255	11:40:35 AM
70.2795255	11:40:36 AM
70.3389005	11:40:37 AM
70.4107755	11:40:38 AM
70.4732755	11:40:39 AM
70.5482755	11:40:40 AM
70.6451505	11:40:41 AM
70.7357755	11:40:42 AM

70.7951505	11:40:43 AM
70.8732755	11:40:44 AM
70.9545255	11:40:45 AM
71.0357755	11:40:46 AM
71.1014005	11:40:47 AM
71.1482755	11:40:48 AM
71.1857755	11:40:49 AM
71.2264005	11:40:50 AM
71.2576505	11:40:51 AM
71.2732755	11:40:52 AM
71.3014005	11:40:53 AM
71.3295255	11:40:54 AM
71.3420255	11:40:55 AM
71.3545255	11:40:56 AM
71.3701505	11:40:57 AM
71.3732755	11:40:58 AM
71.3701505	11:40:59 AM
71.3795255	11:41:00 AM
71.3857755	11:41:01 AM
71.3795255	11:41:02 AM
71.3545255	11:41:03 AM
71.3420255	11:41:04 AM
71.3357755	11:41:05 AM
71.3420255	11:41:06 AM
71.3482755	11:41:07 AM
71.3389005	11:41:08 AM
71.3451505	11:41:09 AM
71.3764005	11:41:10 AM
71.4201505	11:41:11 AM
71.4701505	11:41:12 AM
71.4795255	11:41:13 AM
71.4795255	11:41:14 AM
71.4982755	11:41:15 AM
71.5076505	11:41:16 AM
71.4982755	11:41:17 AM
71.4889005	11:41:18 AM
71.4889005	11:41:19 AM
71.4795255	11:41:20 AM
71.4639005	11:41:21 AM
71.4607755	11:41:22 AM
71.4451505	11:41:23 AM
71.4232755	11:41:24 AM
71.4107755	11:41:25 AM
71.3951505	11:41:26 AM
71.3795255	11:41:27 AM

71.3795255	11:41:28 AM
71.3920255	11:41:29 AM
71.3951505	11:41:30 AM
71.4201505	11:41:31 AM
71.4309304	11:41:32 AM
71.447179	11:41:33 AM
71.461865	11:41:34 AM
71.4835824	11:41:35 AM
71.5523324	11:41:36 AM
71.6117074	11:41:37 AM
71.6554574	11:41:38 AM
71.6867074	11:41:39 AM
71.7085824	11:41:40 AM
71.7242074	11:41:41 AM
71.7148324	11:41:42 AM
71.6960824	11:41:43 AM
71.6742074	11:41:44 AM
71.6242074	11:41:45 AM
71.5523324	11:41:46 AM
71.4742074	11:41:47 AM
71.3992074	11:41:48 AM
71.3117074	11:41:49 AM
71.2117074	11:41:50 AM
71.0835824	11:41:51 AM
70.9429574	11:41:52 AM
70.8367074	11:41:53 AM
70.7335824	11:41:54 AM
70.5960824	11:41:55 AM
70.4492074	11:41:56 AM
70.3023324	11:41:57 AM
70.1554574	11:41:58 AM
69.9992074	11:41:59 AM
69.8617074	11:42:00 AM
69.7148324	11:42:01 AM
69.5554574	11:42:02 AM
69.3898324	11:42:03 AM
69.2398324	11:42:04 AM
69.0835824	11:42:05 AM
68.9429574	11:42:06 AM
68.8117074	11:42:07 AM
68.6742074	11:42:08 AM
68.5367074	11:42:09 AM
68.3992074	11:42:10 AM
68.2492074	11:42:11 AM
68.1228025	11:42:12 AM

68.006554	11:42:13 AM
67.8981179	11:42:14 AM
67.7826505	11:42:15 AM
67.6295255	11:42:16 AM
67.5045255	11:42:17 AM
67.3920255	11:42:18 AM
67.2857755	11:42:19 AM
67.1732755	11:42:20 AM
67.0701505	11:42:21 AM
66.9920255	11:42:22 AM
66.9170255	11:42:23 AM
66.8326505	11:42:24 AM
66.7701505	11:42:25 AM
66.7170255	11:42:26 AM
66.6701505	11:42:27 AM
66.6201505	11:42:28 AM
66.5795255	11:42:29 AM
66.5482755	11:42:30 AM
66.5357755	11:42:31 AM
66.5357755	11:42:32 AM
66.5420255	11:42:33 AM
66.5451505	11:42:34 AM
66.5576505	11:42:35 AM
66.5920255	11:42:36 AM
66.6545255	11:42:37 AM
66.7420255	11:42:38 AM
66.8201505	11:42:39 AM
66.8795255	11:42:40 AM
66.9389005	11:42:41 AM
66.9764005	11:42:42 AM
67.0076505	11:42:43 AM
67.0170255	11:42:44 AM
66.9982755	11:42:45 AM
66.9545255	11:42:46 AM
66.9201505	11:42:47 AM
66.8951505	11:42:48 AM
66.8732755	11:42:49 AM
66.8514005	11:42:50 AM
66.8295255	11:42:51 AM
66.8139005	11:42:52 AM
66.7857755	11:42:53 AM
66.7701505	11:42:54 AM
66.7732755	11:42:55 AM
66.7701505	11:42:56 AM
66.7639005	11:42:57 AM

66.7732755	11:42:58 AM
66.7920255	11:42:59 AM
66.8232755	11:43:00 AM
66.8639005	11:43:01 AM
66.9107755	11:43:02 AM
66.9670255	11:43:03 AM
67.0264005	11:43:04 AM
67.0732755	11:43:05 AM
67.1170255	11:43:06 AM
67.1514005	11:43:07 AM
67.1920255	11:43:08 AM
67.2232755	11:43:09 AM
67.2514005	11:43:10 AM
67.2920255	11:43:11 AM
67.3420255	11:43:12 AM
67.3982755	11:43:13 AM
67.4482755	11:43:14 AM
67.5076505	11:43:15 AM
67.5701505	11:43:16 AM
67.6389005	11:43:17 AM
67.6795255	11:43:18 AM
67.7357755	11:43:19 AM
67.7951505	11:43:20 AM
67.8514005	11:43:21 AM
67.9264005	11:43:22 AM
68.0045255	11:43:23 AM
68.1076505	11:43:24 AM
68.2389005	11:43:25 AM
68.3764005	11:43:26 AM
68.4951505	11:43:27 AM
68.6076505	11:43:28 AM
68.7264005	11:43:29 AM
68.8482755	11:43:30 AM
68.9607755	11:43:31 AM
69.0576505	11:43:32 AM
69.0937095	11:43:33 AM
69.1405845	11:43:34 AM
69.1624595	11:43:35 AM
69.1812095	11:43:36 AM
69.1905845	11:43:37 AM
69.2030845	11:43:38 AM
69.2249595	11:43:39 AM
69.2405845	11:43:40 AM
69.2312095	11:43:41 AM
69.2093345	11:43:42 AM

69.1843345	11:43:43 AM
69.1499595	11:43:44 AM
69.1218345	11:43:45 AM
69.0937095	11:43:46 AM
69.0718345	11:43:47 AM
69.0593345	11:43:48 AM
69.0499595	11:43:49 AM
69.0499595	11:43:50 AM
69.0468345	11:43:51 AM
69.0468345	11:43:52 AM
69.0405845	11:43:53 AM
69.0624595	11:43:54 AM
69.1062095	11:43:55 AM
69.1437095	11:43:56 AM
69.1212271	11:43:57 AM
69.0991353	11:43:58 AM
69.0672779	11:43:59 AM
69.0186237	11:44:00 AM
68.9594225	11:44:01 AM
68.903737	11:44:02 AM
68.8613327	11:44:03 AM
68.804866	11:44:04 AM
68.7448836	11:44:05 AM
68.6974012	11:44:06 AM
68.6757001	11:44:07 AM
68.6598583	11:44:08 AM
68.6342509	11:44:09 AM
68.6094248	11:44:10 AM
68.6017861	11:44:11 AM
68.6136787	11:44:12 AM
68.686778	11:44:13 AM
68.7377331	11:44:14 AM
68.8023601	11:44:15 AM
68.868159	11:44:16 AM
68.9316141	11:44:17 AM
68.9923348	11:44:18 AM
69.0471962	11:44:19 AM
69.0989325	11:44:20 AM
69.1592626	11:44:21 AM
69.2082646	11:44:22 AM
69.2670322	11:44:23 AM
69.3285342	11:44:24 AM
69.38613	11:44:25 AM
69.4077882	11:44:26 AM
69.4419464	11:44:27 AM

69.4655578	11:44:28 AM
69.4817473	11:44:29 AM
69.4889524	11:44:30 AM
69.4938137	11:44:31 AM
69.4783626	11:44:32 AM
69.4593958	11:44:33 AM
69.4177728	11:44:34 AM
69.3573998	11:44:35 AM
69.2989799	11:44:36 AM
69.3024955	11:44:37 AM
69.2989799	11:44:38 AM
69.3032768	11:44:39 AM
69.3216362	11:44:40 AM
69.3505424	11:44:41 AM
69.3724174	11:44:42 AM
69.3810112	11:44:43 AM
69.3923393	11:44:44 AM
69.4110893	11:44:45 AM
69.4333549	11:44:46 AM
69.4423393	11:44:47 AM
69.4489799	11:44:48 AM
69.444683	11:44:49 AM
69.4333549	11:44:50 AM
69.4146049	11:44:51 AM
69.388433	11:44:52 AM
69.3587455	11:44:53 AM
69.3118705	11:44:54 AM
69.2575737	11:44:55 AM
69.1903862	11:44:56 AM
69.116558	11:44:57 AM
69.0427299	11:44:58 AM
68.9583549	11:44:59 AM
68.8739799	11:45:00 AM
68.7962455	11:45:01 AM
68.7216362	11:45:02 AM
68.647808	11:45:03 AM
68.5958549	11:45:04 AM
68.6257592	11:45:05 AM
68.700976	11:45:06 AM
68.7605677	11:45:07 AM
68.8244564	11:45:08 AM
68.8863919	11:45:09 AM
68.9385618	11:45:10 AM
68.9805754	11:45:11 AM
69.0272765	11:45:12 AM

69.0743683	11:45:13 AM
69.1097413	11:45:14 AM
69.1513643	11:45:15 AM
69.1941592	11:45:16 AM
69.225626	11:45:17 AM
69.2512334	11:45:18 AM
69.2662939	11:45:19 AM
69.2809638	11:45:20 AM
69.2893837	11:45:21 AM
69.3013192	11:45:22 AM
69.3288797	11:45:23 AM
69.3583933	11:45:24 AM
69.3965007	11:45:25 AM
69.4342175	11:45:26 AM
69.4750593	11:45:27 AM
69.5248854	11:45:28 AM
69.5954147	11:45:29 AM
69.6690689	11:45:30 AM
69.7392076	11:45:31 AM
69.7972368	11:45:32 AM
69.8583911	11:45:33 AM
69.923061	11:45:34 AM
69.9908559	11:45:35 AM
70.0484945	11:45:36 AM
70.1026175	11:45:37 AM
70.1407249	11:45:38 AM
70.1764886	11:45:39 AM
70.2028772	11:45:40 AM
70.2390315	11:45:41 AM
70.2802639	11:45:42 AM
70.3421994	11:45:43 AM
70.3670255	11:45:44 AM
70.3107755	11:45:45 AM
70.2482755	11:45:46 AM
70.1639005	11:45:47 AM
70.0639005	11:45:48 AM
69.9576505	11:45:49 AM
69.8670255	11:45:50 AM
69.7982755	11:45:51 AM
69.7232755	11:45:52 AM
69.6482755	11:45:53 AM
69.5889005	11:45:54 AM
69.5170255	11:45:55 AM
69.4420255	11:45:56 AM
69.3639005	11:45:57 AM

69.2982755	11:45:58 AM
69.2357755	11:45:59 AM
69.1732755	11:46:00 AM
69.1201505	11:46:01 AM
69.0795255	11:46:02 AM
69.0357755	11:46:03 AM
68.9826505	11:46:04 AM
68.9232755	11:46:05 AM
68.8514005	11:46:06 AM
68.7764005	11:46:07 AM
68.6920255	11:46:08 AM
68.5889005	11:46:09 AM
68.4857755	11:46:10 AM
68.3826505	11:46:11 AM
68.2951505	11:46:12 AM
68.2076505	11:46:13 AM
68.1201505	11:46:14 AM
68.0295255	11:46:15 AM
67.9576505	11:46:16 AM
67.9045255	11:46:17 AM
67.8795255	11:46:18 AM
67.8670255	11:46:19 AM
67.8826505	11:46:20 AM
67.9107755	11:46:21 AM
67.9701505	11:46:22 AM
68.0045255	11:46:23 AM
68.0482755	11:46:24 AM
68.0889005	11:46:25 AM
68.1389005	11:46:26 AM
68.2107755	11:46:27 AM
68.2982755	11:46:28 AM
68.3982755	11:46:29 AM
68.4857755	11:46:30 AM
68.5576505	11:46:31 AM
68.6295255	11:46:32 AM
68.7076505	11:46:33 AM
68.8045255	11:46:34 AM
68.9107755	11:46:35 AM
69.0264005	11:46:36 AM
69.1357755	11:46:37 AM
69.2295255	11:46:38 AM
69.3232755	11:46:39 AM
69.4045255	11:46:40 AM
69.4764005	11:46:41 AM
69.5389005	11:46:42 AM

69.5982755	11:46:43 AM
69.6482755	11:46:44 AM
69.6857755	11:46:45 AM
69.7420255	11:46:46 AM
69.8076505	11:46:47 AM
69.8230881	11:46:48 AM
69.8654788	11:46:49 AM
69.9654788	11:46:50 AM
70.0160726	11:46:51 AM
70.0635414	11:46:52 AM
70.0973384	11:46:53 AM
70.1241041	11:46:54 AM
70.1579011	11:46:55 AM
70.1846668	11:46:56 AM
70.1852606	11:46:57 AM
70.1710107	11:46:58 AM
70.1536357	11:46:59 AM
70.1206358	11:47:00 AM
70.0587296	11:47:01 AM
69.9772922	11:47:02 AM
69.8962454	11:47:03 AM
69.8214486	11:47:04 AM
69.7489956	11:47:05 AM
69.6601363	11:47:06 AM
69.574402	11:47:07 AM
69.4992145	11:47:08 AM
69.4310584	11:47:09 AM
69.3691522	11:47:10 AM
69.313496	11:47:11 AM
69.2707305	11:47:12 AM
69.2080431	11:47:13 AM
69.1266056	11:47:14 AM
69.0393088	11:47:15 AM
68.9524027	11:47:16 AM
68.8721371	11:47:17 AM
68.8102309	11:47:18 AM
68.7483248	11:47:19 AM
68.6993092	11:47:20 AM
68.6706062	11:47:21 AM
68.6458094	11:47:22 AM
68.6178876	11:47:23 AM
68.6208251	11:47:24 AM
68.6124346	11:47:25 AM
68.5907628	11:47:26 AM
68.5589347	11:47:27 AM

68.5702629	11:47:28 AM
68.6184972	11:47:29 AM
68.5997472	11:47:30 AM
68.6179033	11:47:31 AM
68.6454345	11:47:32 AM
68.6835126	11:47:33 AM
68.7192469	11:47:34 AM
68.7541999	11:47:35 AM
68.7930592	11:47:36 AM
68.8393404	11:47:37 AM
68.8848403	11:47:38 AM
68.9334652	11:47:39 AM
68.9914652	11:47:40 AM
69.0627463	11:47:41 AM
69.1379338	11:47:42 AM
69.2189806	11:47:43 AM
69.3031524	11:47:44 AM
69.3818554	11:47:45 AM
69.4519647	11:47:46 AM
69.4582147	11:47:47 AM
69.4574334	11:47:48 AM
69.4437616	11:47:49 AM
69.4543084	11:47:50 AM
69.4812616	11:47:51 AM
69.5074334	11:47:52 AM
69.6013709	11:47:53 AM
69.6703083	11:47:54 AM
69.7419801	11:47:55 AM
69.7882613	11:47:56 AM
69.8216518	11:47:57 AM
69.821058	11:47:58 AM
69.8142142	11:47:59 AM
69.8069797	11:48:00 AM
69.7856828	11:48:01 AM
69.7729796	11:48:02 AM
69.7665264	11:48:03 AM
69.7604638	11:48:04 AM
69.7751043	11:48:05 AM
69.7905261	11:48:06 AM
69.8473542	11:48:07 AM
69.9080885	11:48:08 AM
69.9080885	11:48:09 AM
69.9143385	11:48:10 AM
69.9237135	11:48:11 AM
69.9049635	11:48:12 AM

69.8737135	11:48:13 AM
69.8393385	11:48:14 AM
69.8112135	11:48:15 AM
69.7799635	11:48:16 AM
69.7518385	11:48:17 AM
69.7299635	11:48:18 AM
69.6830885	11:48:19 AM
69.6205885	11:48:20 AM
69.5549635	11:48:21 AM
69.5205885	11:48:22 AM
69.4559479	11:48:23 AM
69.3956043	11:48:24 AM
69.3239325	11:48:25 AM
69.2405419	11:48:26 AM
69.2171044	11:48:27 AM
69.1729638	11:48:28 AM
69.1276513	11:48:29 AM
69.0651513	11:48:30 AM
68.979995	11:48:31 AM
68.89562	11:48:32 AM
68.7567607	11:48:33 AM
68.6487608	11:48:34 AM
68.5544328	11:48:35 AM
68.4940891	11:48:36 AM
68.4517142	11:48:37 AM
68.5235892	11:48:38 AM
68.6235892	11:48:39 AM
68.7142142	11:48:40 AM
68.7923392	11:48:41 AM
68.7954642	11:48:42 AM
68.7767142	11:48:43 AM
68.7204642	11:48:44 AM
68.6548392	11:48:45 AM
68.5892142	11:48:46 AM
68.4860892	11:48:47 AM
68.3892142	11:48:48 AM
68.3017142	11:48:49 AM
68.2235892	11:48:50 AM
68.1579642	11:48:51 AM
68.1079642	11:48:52 AM
68.0673392	11:48:53 AM
68.0329642	11:48:54 AM
67.9892142	11:48:55 AM
67.9454642	11:48:56 AM
67.9048392	11:48:57 AM

67.8485892	11:48:58 AM
67.7798392	11:48:59 AM
67.7142142	11:49:00 AM
67.6329642	11:49:01 AM
67.5235892	11:49:02 AM
67.4413547	11:49:03 AM
67.3610734	11:49:04 AM
67.3046202	11:49:05 AM
67.2817607	11:49:06 AM
67.2659325	11:49:07 AM
67.2829168	11:49:08 AM
67.3194324	11:49:09 AM
67.2913022	11:49:10 AM
67.269422	11:49:11 AM
67.2953907	11:49:12 AM
67.33425	11:49:13 AM
67.3734999	11:49:14 AM
67.3834529	11:49:15 AM
67.3906716	11:49:16 AM
67.4049215	11:49:17 AM
67.3392965	11:49:18 AM
67.2486715	11:49:19 AM
67.1611715	11:49:20 AM
67.0705465	11:49:21 AM
67.0330465	11:49:22 AM
67.0142965	11:49:23 AM
67.0080465	11:49:24 AM
67.0111715	11:49:25 AM
67.0236715	11:49:26 AM
67.0455465	11:49:27 AM
67.0424215	11:49:28 AM
67.0392965	11:49:29 AM
67.0236715	11:49:30 AM
67.0017965	11:49:31 AM
66.9861715	11:49:32 AM
66.9580465	11:49:33 AM
66.9330465	11:49:34 AM
66.9049215	11:49:35 AM
66.8924215	11:49:36 AM
66.8955465	11:49:37 AM
66.9205465	11:49:38 AM
66.9830465	11:49:39 AM
67.0549215	11:49:40 AM
67.1486715	11:49:41 AM
67.2392965	11:49:42 AM

67.2985139	11:49:43 AM
67.3421062	11:49:44 AM
67.3806204	11:49:45 AM
67.4199159	11:49:46 AM
67.4654614	11:49:47 AM
67.5199912	11:49:48 AM
67.5745211	11:49:49 AM
67.6823685	11:49:50 AM
67.7781066	11:49:51 AM
67.8283396	11:49:52 AM
67.8652913	11:49:53 AM
67.8924774	11:49:54 AM
67.9282572	11:49:55 AM
67.9495839	11:49:56 AM
67.9619263	11:49:57 AM
67.9727061	11:49:58 AM
68.0102061	11:49:59 AM
68.0289561	11:50:00 AM
68.0633311	11:50:01 AM
68.0789561	11:50:02 AM
68.0820811	11:50:03 AM
68.0945811	11:50:04 AM
68.0977061	11:50:05 AM
68.1164561	11:50:06 AM
68.1320811	11:50:07 AM
68.1695811	11:50:08 AM
68.2008311	11:50:09 AM
68.2258311	11:50:10 AM
68.2477061	11:50:11 AM
68.2633311	11:50:12 AM
68.3008311	11:50:13 AM
68.3602061	11:50:14 AM
68.4258311	11:50:15 AM
68.4883311	11:50:16 AM
68.5258311	11:50:17 AM
68.5170797	11:50:18 AM
68.5165314	11:50:19 AM
68.523405	11:50:20 AM
68.5275442	11:50:21 AM
68.5500428	11:50:22 AM
68.6086366	11:50:23 AM
68.6906678	11:50:24 AM
68.7840272	11:50:25 AM
68.867621	11:50:26 AM
68.9418397	11:50:27 AM

68.9820741	11:50:28 AM
69.0105897	11:50:29 AM
69.0699647	11:50:30 AM
69.1234803	11:50:31 AM
69.1777772	11:50:32 AM
69.248871	11:50:33 AM
69.3404349	11:50:34 AM
69.43278	11:50:35 AM
69.5020783	11:50:36 AM
69.549111	11:50:37 AM
69.5820811	11:50:38 AM
69.5914561	11:50:39 AM
69.6227061	11:50:40 AM
69.6539561	11:50:41 AM
69.7289561	11:50:42 AM
69.8195811	11:50:43 AM
69.9008311	11:50:44 AM
69.9852061	11:50:45 AM
70.0539561	11:50:46 AM
70.1164561	11:50:47 AM
70.1945811	11:50:48 AM
70.2852061	11:50:49 AM
70.3945811	11:50:50 AM
70.5195811	11:50:51 AM
70.6477061	11:50:52 AM
70.7727061	11:50:53 AM
70.8695811	11:50:54 AM
70.9695811	11:50:55 AM
71.0602061	11:50:56 AM
71.1539561	11:50:57 AM
71.2814575	11:50:58 AM
71.4007558	11:50:59 AM
71.4511464	11:51:00 AM
71.4769277	11:51:01 AM
71.4534902	11:51:02 AM
71.3984121	11:51:03 AM
71.3140371	11:51:04 AM
71.2198964	11:51:05 AM
71.1688822	11:51:06 AM
71.089743	11:51:07 AM
71.0106038	11:51:08 AM
70.9306833	11:51:09 AM
70.8304503	11:51:10 AM
70.7388111	11:51:11 AM
70.6374063	11:51:12 AM

70.5137358	11:51:13 AM
70.3731108	11:51:14 AM
70.2231108	11:51:15 AM
70.1074858	11:51:16 AM
70.0137358	11:51:17 AM
69.9418608	11:51:18 AM
69.8637358	11:51:19 AM
69.7887358	11:51:20 AM
69.7168608	11:51:21 AM
69.6262358	11:51:22 AM
69.5293608	11:51:23 AM
69.4418608	11:51:24 AM
69.3449858	11:51:25 AM
69.2449858	11:51:26 AM
69.1574858	11:51:27 AM
69.0668608	11:51:28 AM
68.9762358	11:51:29 AM
68.8824858	11:51:30 AM
68.7824858	11:51:31 AM
68.7012358	11:51:32 AM
68.6293608	11:51:33 AM
68.5824858	11:51:34 AM
68.5606108	11:51:35 AM
68.5087791	11:51:36 AM
68.4530412	11:51:37 AM
68.4008189	11:51:38 AM
68.4133189	11:51:39 AM
68.4998047	11:51:40 AM
68.5948842	11:51:41 AM
68.7020731	11:51:42 AM
68.8362152	11:51:43 AM
69.0012166	11:51:44 AM
69.1728586	11:51:45 AM
69.3041086	11:51:46 AM
69.4603586	11:51:47 AM
69.6041086	11:51:48 AM
69.7447336	11:51:49 AM
69.8791086	11:51:50 AM
70.0228586	11:51:51 AM
70.1697336	11:51:52 AM
70.3259836	11:51:53 AM
70.5041086	11:51:54 AM
70.7103586	11:51:55 AM
70.9072336	11:51:56 AM
71.0853586	11:51:57 AM

71.2541086	11:51:58 AM
71.4291086	11:51:59 AM
71.5978586	11:52:00 AM
71.7509836	11:52:01 AM
71.9041086	11:52:02 AM
72.0572336	11:52:03 AM
72.1916086	11:52:04 AM
72.3166086	11:52:05 AM
72.4134836	11:52:06 AM
72.4884836	11:52:07 AM
72.5478586	11:52:08 AM
72.6009836	11:52:09 AM
72.6509836	11:52:10 AM
72.6978586	11:52:11 AM
72.6800879	11:52:12 AM
72.6498171	11:52:13 AM
72.604312	11:52:14 AM
72.5349788	11:52:15 AM
72.4745085	11:52:16 AM
72.4210694	11:52:17 AM
72.3609897	11:52:18 AM
72.2404846	11:52:19 AM
72.1117764	11:52:20 AM
71.9889275	11:52:21 AM
71.8785786	11:52:22 AM
71.7725267	11:52:23 AM
71.6516309	11:52:24 AM
71.5112039	11:52:25 AM
71.3438238	11:52:26 AM
71.1623812	11:52:27 AM
70.9817198	11:52:28 AM
70.7897303	11:52:29 AM
70.5985221	11:52:30 AM
70.3858295	11:52:31 AM
70.1602463	11:52:32 AM
69.9155224	11:52:33 AM
69.644236	11:52:34 AM
69.3534184	11:52:35 AM
69.0614289	11:52:36 AM
68.7885801	11:52:37 AM
68.5395593	11:52:38 AM
68.3077261	11:52:39 AM
68.1233511	11:52:40 AM
67.9296011	11:52:41 AM
67.7202261	11:52:42 AM

67.5046011	11:52:43 AM
67.3171011	11:52:44 AM
67.1219866	11:52:45 AM
66.9565596	11:52:46 AM
66.8005076	11:52:47 AM
66.6272681	11:52:48 AM
66.4462161	11:52:49 AM
66.2651641	11:52:50 AM
66.0708309	11:52:51 AM
65.9153622	11:52:52 AM
65.7598934	11:52:53 AM
65.607159	11:52:54 AM
65.4614559	11:52:55 AM
65.3368465	11:52:56 AM
65.2298153	11:52:57 AM
65.1364559	11:52:58 AM
65.0454403	11:52:59 AM
64.9591122	11:53:00 AM
64.8872372	11:53:01 AM
64.8462215	11:53:02 AM
64.8106747	11:53:03 AM
64.779034	11:53:04 AM
64.7688778	11:53:05 AM
64.7841122	11:53:06 AM
64.8280548	11:53:07 AM
64.8587161	11:53:08 AM
64.8882056	11:53:09 AM
64.9231639	11:53:10 AM
64.9577315	11:53:11 AM
65.0020647	11:53:12 AM
65.0499136	11:53:13 AM
65.0930749	11:53:14 AM
65.1338926	11:53:15 AM
65.179007	11:53:16 AM
65.2206059	11:53:17 AM
65.2352516	11:53:18 AM
65.2420849	11:53:19 AM
65.2014599	11:53:20 AM
65.1858349	11:53:21 AM
65.1889599	11:53:22 AM
65.2014599	11:53:23 AM
65.1983349	11:53:24 AM
65.2121994	11:53:25 AM
65.2432514	11:53:26 AM
65.2774284	11:53:27 AM

65.3245549	11:53:28 AM
65.3799403	11:53:29 AM
65.4464864	11:53:30 AM
65.5225655	11:53:31 AM
65.6056758	11:53:32 AM
65.6919111	11:53:33 AM
65.7958548	11:53:34 AM
65.9056089	11:53:35 AM
66.020363	11:53:36 AM
66.1452888	11:53:37 AM
66.2775834	11:53:38 AM
66.4209457	11:53:39 AM
66.5490736	11:53:40 AM
66.6617126	11:53:41 AM
66.7174022	11:53:42 AM
66.7105046	11:53:43 AM
66.5342459	11:53:44 AM
66.2729017	11:53:45 AM
66.009995	11:53:46 AM

		Person 12	Person 2	Person 4	Person 1	Person 13
Time						
18:02:22	0:00:01	70.101237	81.2842889	79.8195316	80.8813582	88.5172777
18:02:23	0:00:02	71.101237	81.2842889	79.8195316	80.8813582	87.5172777
18:02:24	0:00:03	74.101237	81.2842889	74.8195316	80.8813582	86.5172777
18:02:25	0:00:04	74.101237	81.2842889	73.8195316	80.8813582	86.5172777
18:02:26	0:00:05	74.101237	81.2842889	73.8195316	80.8813582	84.5172777
18:02:27	0:00:06	74.101237	81.2842889	73.8195316	81.8813582	83.5172777
18:02:28	0:00:07	73.101237	80.2842889	72.8195316	82.8813582	82.5172777
18:02:29	0:00:08	73.101237	80.2842889	72.8195316	82.8813582	82.5172777
18:02:30	0:00:09	73.101237	80.2842889	73.8195316	82.8813582	82.5172777
18:02:31	0:00:10	73.101237	79.2842889	74.8195316	82.8813582	82.5172777
18:02:32	0:00:11	73.101237	79.2842889	76.8195316	75.8813582	82.5172777
18:02:33	0:00:12	73.101237	78.2842889	76.8195316	75.8813582	82.5172777
18:02:34	0:00:13	73.101237	78.2842889	77.8195316	75.8813582	83.5172777
18:02:35	0:00:14	73.101237	78.2842889	78.8195316	74.8813582	83.5172777
18:02:36	0:00:15	74.101237	78.2842889	78.8195316	74.8813582	83.5172777
18:02:37	0:00:16	74.101237	77.2842889	78.8195316	74.8813582	84.5172777
18:02:38	0:00:17	75.101237	76.2842889	78.8195316	74.8813582	84.5172777
18:02:39	0:00:18	76.101237	76.2842889	78.8195316	75.8813582	85.5172777
18:02:40	0:00:19	78.101237	75.2842889	77.8195316	76.8813582	82.5172777
18:02:41	0:00:20	78.101237	75.2842889	77.8195316	76.8813582	79.5172777
18:02:42	0:00:21	78.101237	75.2842889	77.8195316	76.8813582	79.5172777
18:02:43	0:00:22	79.101237	75.2842889	76.8195316	76.8813582	79.5172777
18:02:44	0:00:23	75.101237	75.2842889	73.8195316	76.8813582	77.5172777
18:02:45	0:00:24	75.101237	75.2842889	72.8195316	81.8813582	77.5172777
18:02:46	0:00:25	75.101237	74.2842889	72.8195316	81.8813582	77.5172777
18:02:47	0:00:26	76.101237	73.2842889	69.8195316	80.8813582	77.5172777
18:02:48	0:00:27	74.101237	70.2842889	67.8195316	80.8813582	77.5172777
18:02:49	0:00:28	73.101237	70.2842889	65.8195316	80.8813582	77.5172777
18:02:50	0:00:29	73.101237	69.2842889	65.8195316	80.8813582	71.5172777
18:02:51	0:00:30	73.101237	69.2842889	64.8195316	80.8813582	69.5172777
18:02:52	0:00:31	72.101237	68.2842889	65.8195316	80.8813582	69.5172777
18:02:53	0:00:32	70.101237	68.2842889	65.8195316	79.8813582	67.5172777
18:02:54	0:00:33	70.101237	68.2842889	66.8195316	78.8813582	66.5172777
18:02:55	0:00:34	75.101237	68.2842889	66.8195316	78.8813582	66.5172777
18:02:56	0:00:35	76.101237	68.2842889	69.8195316	78.8813582	66.5172777
18:02:57	0:00:36	76.101237	68.2842889	69.8195316	78.8813582	66.5172777
18:02:58	0:00:37	75.101237	68.2842889	69.8195316	78.8813582	64.5172777
18:02:59	0:00:38	73.101237	68.2842889	70.8195316	79.8813582	64.5172777
18:03:00	0:00:39	73.101237	68.2842889	68.8195316	79.8813582	64.5172777
18:03:01	0:00:40	72.101237	68.2842889	68.8195316	79.8813582	64.5172777
18:03:02	0:00:41	73.101237	68.2842889	66.8195316	81.8813582	65.5172777
18:03:03	0:00:42	73.101237	68.2842889	66.8195316	83.8813582	64.5172777

18:03:04	0:00:43	74.101237	68.2842889	66.8195316	83.8813582	65.5172777
18:03:05	0:00:44	75.101237	68.2842889	66.8195316	82.8813582	65.5172777
18:03:06	0:00:45	75.101237	68.2842889	66.8195316	82.8813582	65.5172777
18:03:07	0:00:46	75.101237	69.2842889	64.8195316	82.8813582	65.5172777
18:03:08	0:00:47	75.101237	69.2842889	63.8195316	82.8813582	64.5172777
18:03:09	0:00:48	76.101237	69.2842889	63.8195316	82.8813582	63.5172777
18:03:10	0:00:49	77.101237	70.2842889	63.8195316	82.8813582	63.5172777
18:03:11	0:00:50	77.101237	70.2842889	63.8195316	82.8813582	63.5172777
18:03:12	0:00:51	78.101237	69.2842889	63.8195316	81.8813582	64.5172777
18:03:13	0:00:52	78.101237	69.2842889	63.8195316	79.8813582	64.5172777
18:03:14	0:00:53	78.101237	70.2842889	63.8195316	76.8813582	65.5172777
18:03:15	0:00:54	78.101237	71.2842889	63.8195316	74.8813582	65.5172777
18:03:16	0:00:55	78.101237	71.2842889	63.8195316	74.8813582	64.5172777
18:03:17	0:00:56	77.101237	74.2842889	63.8195316	72.8813582	64.5172777
18:03:18	0:00:57	77.101237	74.2842889	62.8195316	71.8813582	65.5172777
18:03:19	0:00:58	74.101237	74.2842889	62.8195316	70.8813582	65.5172777
18:03:20	0:00:59	74.101237	75.2842889	62.8195316	70.8813582	65.5172777
18:03:21	0:01:00	73.101237	75.2842889	62.8195316	70.8813582	65.5172777
18:03:22	0:01:01	73.101237	76.2842889	61.8195316	70.8813582	65.5172777
18:03:23	0:01:02	72.101237	75.2842889	61.8195316	69.8813582	65.5172777
18:03:24	0:01:03	69.101237	75.2842889	62.8195316	69.8813582	65.5172777
18:03:25	0:01:04	69.101237	75.2842889	62.8195316	69.8813582	65.5172777
18:03:26	0:01:05	66.101237	75.2842889	62.8195316	69.8813582	67.5172777
18:03:27	0:01:06	61.101237	75.2842889	62.8195316	69.8813582	69.5172777
18:03:28	0:01:07	60.101237	75.2842889	62.8195316	69.8813582	69.5172777
18:03:29	0:01:08	60.101237	74.2842889	62.8195316	69.8813582	72.5172777
18:03:30	0:01:09	61.101237	73.2842889	62.8195316	70.8813582	71.5172777
18:03:31	0:01:10	62.101237	73.2842889	62.8195316	71.8813582	68.5172777
18:03:32	0:01:11	64.101237	74.2842889	62.8195316	72.8813582	66.5172777
18:03:33	0:01:12	67.101237	74.2842889	62.8195316	72.8813582	65.5172777
18:03:34	0:01:13	68.101237	74.2842889	62.8195316	72.8813582	66.5172777
18:03:35	0:01:14	68.101237	74.2842889	62.8195316	72.8813582	66.5172777
18:03:36	0:01:15	68.101237	74.2842889	62.8195316	72.8813582	66.5172777
18:03:37	0:01:16	68.101237	70.2842889	62.8195316	72.8813582	64.5172777
18:03:38	0:01:17	67.101237	70.2842889	62.8195316	72.8813582	64.5172777
18:03:39	0:01:18	67.101237	70.2842889	63.8195316	70.8813582	63.5172777
18:03:40	0:01:19	67.101237	70.2842889	64.8195316	68.8813582	63.5172777
18:03:41	0:01:20	68.101237	69.2842889	64.8195316	68.8813582	63.5172777
18:03:42	0:01:21	68.101237	69.2842889	64.8195316	68.8813582	63.5172777
18:03:43	0:01:22	68.101237	69.2842889	63.8195316	68.8813582	63.5172777
18:03:44	0:01:23	69.101237	69.2842889	64.8195316	68.8813582	63.5172777
18:03:45	0:01:24	69.101237	69.2842889	67.8195316	68.8813582	63.5172777
18:03:46	0:01:25	70.101237	69.2842889	67.8195316	68.8813582	63.5172777
18:03:47	0:01:26	70.101237	68.2842889	67.8195316	69.8813582	63.5172777
18:03:48	0:01:27	70.101237	68.2842889	65.8195316	69.8813582	63.5172777

18:03:49	0:01:28	68.101237	68.2842889	65.8195316	69.8813582	63.5172777
18:03:50	0:01:29	68.101237	68.2842889	65.8195316	69.8813582	63.5172777
18:03:51	0:01:30	67.101237	68.2842889	66.8195316	69.8813582	63.5172777
18:03:52	0:01:31	68.101237	69.2842889	67.8195316	68.8813582	62.5172777
18:03:53	0:01:32	68.101237	69.2842889	67.8195316	67.8813582	62.5172777
18:03:54	0:01:33	68.101237	70.2842889	67.8195316	68.8813582	63.5172777
18:03:55	0:01:34	66.101237	70.2842889	67.8195316	68.8813582	63.5172777
18:03:56	0:01:35	66.101237	70.2842889	67.8195316	68.8813582	63.5172777
18:03:57	0:01:36	65.101237	70.2842889	66.8195316	68.8813582	63.5172777
18:03:58	0:01:37	65.101237	68.2842889	66.8195316	67.8813582	64.5172777
18:03:59	0:01:38	65.101237	66.2842889	66.8195316	66.8813582	65.5172777
18:04:00	0:01:39	65.101237	66.2842889	65.8195316	66.8813582	66.5172777
18:04:01	0:01:40	65.101237	66.2842889	65.8195316	65.8813582	67.5172777
18:04:02	0:01:41	65.101237	66.2842889	66.8195316	65.8813582	67.5172777
18:04:03	0:01:42	65.101237	66.2842889	66.8195316	65.8813582	73.5172777
18:04:04	0:01:43	65.101237	67.2842889	66.8195316	64.8813582	73.5172777
18:04:05	0:01:44	65.101237	67.2842889	65.8195316	65.8813582	73.5172777
18:04:06	0:01:45	66.101237	67.2842889	64.8195316	65.8813582	74.5172777
18:04:07	0:01:46	73.101237	67.2842889	64.8195316	65.8813582	74.5172777
18:04:08	0:01:47	74.101237	67.2842889	65.8195316	65.8813582	76.5172777
18:04:09	0:01:48	74.101237	67.2842889	66.8195316	63.8813582	78.5172777
18:04:10	0:01:49	74.101237	67.2842889	67.8195316	64.8813582	78.5172777
18:04:11	0:01:50	74.101237	64.2842889	67.8195316	64.8813582	78.5172777
18:04:12	0:01:51	74.101237	64.2842889	67.8195316	64.8813582	78.5172777
18:04:13	0:01:52	75.101237	63.2842889	68.8195316	65.8813582	75.5172777
18:04:14	0:01:53	74.101237	63.2842889	68.8195316	66.8813582	74.5172777
18:04:15	0:01:54	73.101237	63.2842889	68.8195316	66.8813582	74.5172777
18:04:16	0:01:55	72.101237	63.2842889	68.8195316	66.8813582	75.5172777
18:04:17	0:01:56	72.101237	63.2842889	69.8195316	66.8813582	76.5172777
18:04:18	0:01:57	73.101237	63.2842889	69.8195316	67.8813582	76.5172777
18:04:19	0:01:58	73.101237	63.2842889	68.8195316	67.8813582	77.5172777
18:04:20	0:01:59	73.101237	63.2842889	68.8195316	67.8813582	78.5172777
18:04:21	0:02:00	74.101237	64.2842889	68.8195316	66.8813582	78.5172777
18:04:22	0:02:01	74.101237	64.2842889	68.8195316	66.8813582	78.5172777
18:04:23	0:02:02	75.101237	65.2842889	69.8195316	66.8813582	72.5172777
18:04:24	0:02:03	75.101237	65.2842889	69.8195316	66.8813582	70.5172777
18:04:25	0:02:04	75.101237	65.2842889	69.8195316	66.8813582	69.5172777
18:04:26	0:02:05	75.101237	69.2842889	69.8195316	67.8813582	69.5172777
18:04:27	0:02:06	75.101237	68.2842889	69.8195316	67.8813582	69.5172777
18:04:28	0:02:07	75.101237	66.2842889	69.8195316	68.8813582	67.5172777
18:04:29	0:02:08	74.101237	64.2842889	69.8195316	67.8813582	66.5172777
18:04:30	0:02:09	74.101237	64.2842889	69.8195316	67.8813582	66.5172777
18:04:31	0:02:10	74.101237	64.2842889	70.8195316	67.8813582	66.5172777
18:04:32	0:02:11	73.101237	64.2842889	69.8195316	67.8813582	66.5172777
18:04:33	0:02:12	73.101237	63.2842889	69.8195316	67.8813582	66.5172777

18:04:34	0:02:13	71.101237	64.2842889	69.8195316	67.8813582	67.5172777
18:04:35	0:02:14	71.101237	64.2842889	71.8195316	69.8813582	66.5172777
18:04:36	0:02:15	70.101237	64.2842889	72.8195316	69.8813582	66.5172777
18:04:37	0:02:16	71.101237	64.2842889	72.8195316	70.8813582	66.5172777
18:04:38	0:02:17	75.101237	65.2842889	73.8195316	71.8813582	68.5172777
18:04:39	0:02:18	78.101237	65.2842889	73.8195316	71.8813582	68.5172777
18:04:40	0:02:19	79.101237	65.2842889	73.8195316	71.8813582	68.5172777
18:04:41	0:02:20	79.101237	64.2842889	73.8195316	71.8813582	67.5172777
18:04:42	0:02:21	78.101237	64.2842889	75.8195316	73.8813582	68.5172777
18:04:43	0:02:22	77.101237	63.2842889	76.8195316	73.8813582	68.5172777
18:04:44	0:02:23	76.101237	64.2842889	77.8195316	73.8813582	67.5172777
18:04:45	0:02:24	75.101237	64.2842889	77.8195316	70.8813582	67.5172777
18:04:46	0:02:25	75.101237	64.2842889	76.8195316	68.8813582	67.5172777
18:04:47	0:02:26	75.101237	64.2842889	76.8195316	68.8813582	66.5172777
18:04:48	0:02:27	75.101237	64.2842889	75.8195316	68.8813582	67.5172777
18:04:49	0:02:28	77.101237	64.2842889	75.8195316	68.8813582	68.5172777
18:04:50	0:02:29	77.101237	64.2842889	75.8195316	68.8813582	68.5172777
18:04:51	0:02:30	80.101237	64.2842889	73.8195316	67.8813582	68.5172777
18:04:52	0:02:31	81.101237	64.2842889	73.8195316	66.8813582	68.5172777
18:04:53	0:02:32	81.101237	64.2842889	73.8195316	67.8813582	69.5172777
18:04:54	0:02:33	75.101237	65.2842889	72.8195316	67.8813582	69.5172777
18:04:55	0:02:34	75.101237	65.2842889	69.8195316	66.8813582	68.5172777
18:04:56	0:02:35	75.101237	64.2842889	69.8195316	66.8813582	67.5172777
18:04:57	0:02:36	75.101237	63.2842889	69.8195316	66.8813582	66.5172777
18:04:58	0:02:37	63.101237	62.2842889	69.8195316	67.8813582	66.5172777
18:04:59	0:02:38	53.101237	62.2842889	69.8195316	67.8813582	65.5172777
18:05:00	0:02:39	53.101237	62.2842889	69.8195316	67.8813582	65.5172777
18:05:01	0:02:40	53.101237	62.2842889	70.8195316	67.8813582	65.5172777
18:05:02	0:02:41	54.101237	62.2842889	71.8195316	67.8813582	65.5172777
18:05:03	0:02:42	55.101237	62.2842889	75.8195316	66.8813582	67.5172777
18:05:04	0:02:43	56.101237	64.2842889	75.8195316	66.8813582	67.5172777
18:05:05	0:02:44	57.101237	65.2842889	75.8195316	66.8813582	67.5172777
18:05:06	0:02:45	58.101237	65.2842889	73.8195316	66.8813582	67.5172777
18:05:07	0:02:46	58.101237	65.2842889	73.8195316	66.8813582	66.5172777
18:05:08	0:02:47	58.101237	65.2842889	72.8195316	66.8813582	66.5172777
18:05:09	0:02:48	63.101237	66.2842889	73.8195316	66.8813582	66.5172777
18:05:10	0:02:49	63.101237	66.2842889	74.8195316	66.8813582	65.5172777
18:05:11	0:02:50	65.101237	66.2842889	74.8195316	66.8813582	65.5172777
18:05:12	0:02:51	65.101237	66.2842889	75.8195316	65.8813582	65.5172777
18:05:13	0:02:52	59.101237	68.2842889	75.8195316	64.8813582	66.5172777
18:05:14	0:02:53	56.101237	69.2842889	76.8195316	63.8813582	67.5172777
18:05:15	0:02:54	58.101237	70.2842889	76.8195316	63.8813582	67.5172777
18:05:16	0:02:55	60.101237	71.2842889	76.8195316	63.8813582	67.5172777
18:05:17	0:02:56	60.101237	71.2842889	77.8195316	64.8813582	66.5172777
18:05:18	0:02:57	62.101237	71.2842889	78.8195316	64.8813582	66.5172777

18:05:19	0:02:58	63.101237	72.2842889	77.8195316	65.8813582	66.5172777
18:05:20	0:02:59	66.101237	72.2842889	76.8195316	65.8813582	67.5172777
18:05:21	0:03:00	66.101237	72.2842889	76.8195316	65.8813582	67.5172777
18:05:22	0:03:01	66.101237	72.2842889	76.8195316	65.8813582	67.5172777
18:05:23	0:03:02	75.101237	72.2842889	76.8195316	65.8813582	67.5172777
18:05:24	0:03:03	75.101237	72.2842889	76.8195316	65.8813582	67.5172777
18:05:25	0:03:04	76.101237	71.2842889	76.8195316	67.8813582	65.5172777
18:05:26	0:03:05	75.101237	69.2842889	76.8195316	68.8813582	66.5172777
18:05:27	0:03:06	73.101237	69.2842889	76.8195316	68.8813582	65.5172777
18:05:28	0:03:07	72.101237	69.2842889	80.8195316	69.8813582	65.5172777
18:05:29	0:03:08	72.101237	69.2842889	84.8195316	69.8813582	65.5172777
18:05:30	0:03:09	72.101237	67.2842889	84.8195316	68.8813582	65.5172777
18:05:31	0:03:10	65.101237	66.2842889	72.3428504	66.8813582	65.5172777
18:05:32	0:03:11	64.101237	67.2842889	72.7428504	66.8813582	66.5172777
18:05:33	0:03:12	64.101237	67.2842889	72.9428504	66.8813582	66.5172777
18:05:34	0:03:13	65.101237	67.2842889	73.5428504	65.8813582	65.5172777
18:05:35	0:03:14	67.101237	67.2842889	74.2428504	65.8813582	65.5172777
18:05:36	0:03:15	68.101237	66.2842889	74.4428504	66.8813582	65.5172777
18:05:37	0:03:16	70.101237	66.2842889	85.8195316	66.8813582	63.5172777
18:05:38	0:03:17	71.101237	70.2842889	75.8195316	66.8813582	63.5172777
18:05:39	0:03:18	72.101237	71.2842889	75.8195316	66.8813582	64.5172777
18:05:40	0:03:19	72.101237	70.2842889	75.8195316	65.8813582	65.5172777
18:05:41	0:03:20	72.101237	69.2842889	74.8195316	65.8813582	66.5172777
18:05:42	0:03:21	73.101237	69.2842889	73.8195316	65.8813582	66.5172777
18:05:43	0:03:22	73.101237	69.2842889	71.8195316	66.8813582	66.5172777
18:05:44	0:03:23	75.101237	70.2842889	71.8195316	66.8813582	66.5172777
18:05:45	0:03:24	76.101237	70.2842889	68.8195316	68.8813582	66.5172777
18:05:46	0:03:25	76.101237	70.2842889	67.8195316	68.8813582	67.5172777
18:05:47	0:03:26	74.101237	70.2842889	66.8195316	66.8813582	68.5172777
18:05:48	0:03:27	74.101237	70.2842889	66.8195316	65.8813582	68.5172777
18:05:49	0:03:28	74.101237	70.2842889	65.8195316	65.8813582	68.5172777
18:05:50	0:03:29	75.101237	68.2842889	65.8195316	66.8813582	71.5172777
18:05:51	0:03:30	75.101237	68.2842889	65.8195316	67.8813582	72.5172777
18:05:52	0:03:31	76.101237	68.2842889	67.8195316	67.8813582	72.5172777
18:05:53	0:03:32	78.101237	67.2842889	68.8195316	68.8813582	73.5172777
18:05:54	0:03:33	78.101237	67.2842889	69.8195316	68.8813582	75.5172777
18:05:55	0:03:34	78.101237	66.2842889	69.8195316	68.8813582	75.5172777
18:05:56	0:03:35	74.101237	66.2842889	69.8195316	67.8813582	76.5172777
18:05:57	0:03:36	69.101237	66.2842889	69.8195316	66.8813582	76.5172777
18:05:58	0:03:37	66.101237	66.2842889	69.8195316	66.8813582	76.5172777
18:05:59	0:03:38	66.101237	66.2842889	69.8195316	65.8813582	76.5172777
18:06:00	0:03:39	65.101237	66.2842889	69.8195316	65.8813582	76.5172777
18:06:01	0:03:40	65.101237	66.2842889	69.8195316	65.8813582	75.5172777
18:06:02	0:03:41	66.101237	66.2842889	69.8195316	65.8813582	75.5172777
18:06:03	0:03:42	70.101237	67.2842889	70.8195316	65.8813582	75.5172777

18:06:04	0:03:43	71.101237	67.2842889	71.8195316	66.8813582	75.5172777
18:06:05	0:03:44	71.101237	67.2842889	71.8195316	66.8813582	75.5172777
18:06:06	0:03:45	70.101237	67.2842889	72.8195316	66.8813582	75.5172777
18:06:07	0:03:46	70.101237	68.2842889	71.8195316	67.8813582	75.5172777
18:06:08	0:03:47	71.101237	68.2842889	70.8195316	67.8813582	75.5172777
18:06:09	0:03:48	71.101237	69.2842889	70.8195316	68.8813582	76.5172777
18:06:10	0:03:49	71.101237	67.2842889	70.8195316	69.8813582	76.5172777
18:06:11	0:03:50	70.101237	67.2842889	69.8195316	69.8813582	78.5172777
18:06:12	0:03:51	71.101237	66.2842889	69.8195316	70.8813582	81.5172777
18:06:13	0:03:52	72.101237	67.2842889	70.8195316	70.8813582	81.5172777
18:06:14	0:03:53	73.101237	67.2842889	70.8195316	70.8813582	81.5172777
18:06:15	0:03:54	74.101237	67.2842889	72.8195316	70.8813582	81.5172777
18:06:16	0:03:55	74.101237	68.2842889	71.8195316	70.8813582	81.5172777
18:06:17	0:03:56	76.101237	68.2842889	71.8195316	70.8813582	80.5172777
18:06:18	0:03:57	76.101237	68.2842889	71.8195316	70.8813582	80.5172777
18:06:19	0:03:58	76.101237	67.2842889	71.8195316	70.8813582	75.5172777
18:06:20	0:03:59	74.101237	67.2842889	70.8195316	69.8813582	75.5172777
18:06:21	0:04:00	74.101237	67.2842889	70.8195316	68.8813582	75.5172777
18:06:22	0:04:01	74.101237	66.2842889	70.8195316	68.8813582	74.5172777
18:06:23	0:04:02	73.101237	65.2842889	70.8195316	68.8813582	74.5172777
18:06:24	0:04:03	72.101237	65.2842889	70.8195316	68.8813582	74.5172777
18:06:25	0:04:04	72.101237	65.2842889	70.8195316	68.8813582	72.5172777
18:06:26	0:04:05	71.101237	66.2842889	73.8195316	70.8813582	71.5172777
18:06:27	0:04:06	71.101237	65.2842889	76.8195316	70.8813582	70.5172777
18:06:28	0:04:07	71.101237	64.2842889	76.8195316	69.8813582	69.5172777
18:06:29	0:04:08	70.101237	64.2842889	76.8195316	67.8813582	69.5172777
18:06:30	0:04:09	70.101237	64.2842889	76.8195316	66.8813582	69.5172777
18:06:31	0:04:10	70.101237	64.2842889	76.8195316	66.8813582	69.5172777
18:06:32	0:04:11	70.101237	64.2842889	76.8195316	65.8813582	68.5172777
18:06:33	0:04:12	70.101237	64.2842889	80.8195316	65.8813582	68.5172777
18:06:34	0:04:13	70.101237	64.2842889	81.8195316	65.8813582	68.5172777
18:06:35	0:04:14	68.101237	64.2842889	81.8195316	65.8813582	69.5172777
18:06:36	0:04:15	68.101237	64.2842889	80.8195316	64.8813582	69.5172777
18:06:37	0:04:16	68.101237	63.2842889	80.8195316	64.8813582	69.5172777
18:06:38	0:04:17	68.101237	63.2842889	77.8195316	65.8813582	69.5172777
18:06:39	0:04:18	67.101237	63.2842889	76.8195316	65.8813582	66.5172777
18:06:40	0:04:19	67.101237	63.2842889	74.8195316	65.8813582	63.5172777
18:06:41	0:04:20	68.101237	63.2842889	73.8195316	65.8813582	63.5172777
18:06:42	0:04:21	68.101237	63.2842889	73.8195316	65.8813582	62.5172777
18:06:43	0:04:22	75.101237	63.2842889	73.8195316	65.8813582	62.5172777
18:06:44	0:04:23	76.101237	63.2842889	72.8195316	65.8813582	62.5172777
18:06:45	0:04:24	76.101237	63.2842889	71.8195316	65.8813582	62.5172777
18:06:46	0:04:25	76.101237	64.2842889	71.8195316	65.8813582	62.5172777
18:06:47	0:04:26	78.101237	64.2842889	71.8195316	65.8813582	62.5172777
18:06:48	0:04:27	79.101237	64.2842889	69.8195316	65.8813582	63.5172777

18:06:49	0:04:28	79.101237	64.2842889	68.8195316	65.8813582	63.5172777
18:06:50	0:04:29	79.101237	63.2842889	68.8195316	65.8813582	63.5172777
18:06:51	0:04:30	79.101237	63.2842889	68.8195316	65.8813582	63.5172777
18:06:52	0:04:31	77.101237	63.2842889	68.8195316	65.8813582	63.5172777
18:06:53	0:04:32	76.101237	63.2842889	69.8195316	66.8813582	63.5172777
18:06:54	0:04:33	76.101237	63.2842889	69.8195316	65.8813582	63.5172777
18:06:55	0:04:34	77.101237	63.2842889	69.8195316	65.8813582	63.5172777
18:06:56	0:04:35	77.101237	63.2842889	69.8195316	66.8813582	63.5172777
18:06:57	0:04:36	77.101237	63.2842889	69.8195316	66.8813582	64.5172777
18:06:58	0:04:37	77.101237	63.2842889	68.8195316	65.8813582	65.5172777
18:06:59	0:04:38	78.101237	63.2842889	68.8195316	66.8813582	65.5172777
18:07:00	0:04:39	78.101237	66.2842889	68.8195316	65.8813582	67.5172777
18:07:01	0:04:40	77.101237	66.2842889	67.8195316	66.8813582	67.5172777
18:07:02	0:04:41	77.101237	69.2842889	67.8195316	66.8813582	67.5172777
18:07:03	0:04:42	77.101237	71.2842889	67.8195316	65.8813582	67.5172777
18:07:04	0:04:43	77.101237	71.2842889	67.8195316	66.8813582	67.5172777
18:07:05	0:04:44	77.101237	71.2842889	68.8195316	66.8813582	67.5172777
18:07:06	0:04:45	77.101237	70.2842889	68.8195316	66.8813582	66.5172777
18:07:07	0:04:46	77.101237	69.2842889	69.8195316	65.8813582	66.5172777
18:07:08	0:04:47	78.101237	69.2842889	70.8195316	66.8813582	66.5172777
18:07:09	0:04:48	77.101237	69.2842889	70.8195316	66.8813582	67.5172777
18:07:10	0:04:49	76.101237	69.2842889	70.8195316	65.8813582	67.5172777
18:07:11	0:04:50	76.101237	69.2842889	70.8195316	66.8813582	67.5172777
18:07:12	0:04:51	76.101237	69.2842889	70.8195316	66.8813582	67.5172777
18:07:13	0:04:52	76.101237	69.2842889	70.8195316	66.8813582	65.5172777
18:07:14	0:04:53	76.101237	68.2842889	70.8195316	67.8813582	65.5172777
18:07:15	0:04:54	76.101237	67.2842889	70.8195316	67.8813582	64.5172777
18:07:16	0:04:55	75.101237	67.2842889	69.8195316	68.8813582	64.5172777
18:07:17	0:04:56	75.101237	67.2842889	70.8195316	68.8813582	63.5172777
18:07:18	0:04:57	74.101237	68.2842889	71.8195316	68.8813582	63.5172777
18:07:19	0:04:58	74.101237	68.2842889	73.8195316	69.8813582	63.5172777
18:07:20	0:04:59	74.101237	68.2842889	73.8195316	70.8813582	63.5172777
18:07:21	0:05:00	74.101237	68.2842889	74.8195316	70.8813582	63.5172777
18:07:22	0:05:01	73.101237	68.2842889	74.8195316	71.8813582	64.5172777
18:07:23	0:05:02	73.101237	68.2842889	74.8195316	73.8813582	65.5172777
18:07:24	0:05:03	72.101237	68.2842889	74.8195316	73.8813582	65.5172777
18:07:25	0:05:04	70.101237	67.2842889	76.8195316	72.8813582	66.5172777
18:07:26	0:05:05	69.101237	67.2842889	76.8195316	71.8813582	67.5172777
18:07:27	0:05:06	67.101237	67.2842889	75.8195316	71.8813582	67.5172777
18:07:28	0:05:07	67.101237	66.2842889	75.8195316	71.8813582	68.5172777
18:07:29	0:05:08	67.101237	65.2842889	76.8195316	70.8813582	71.5172777
18:07:30	0:05:09	65.101237	65.2842889	78.8195316	71.8813582	71.5172777
18:07:31	0:05:10	67.101237	64.2842889	79.8195316	71.8813582	76.5172777
18:07:32	0:05:11	68.101237	64.2842889	79.8195316	71.8813582	77.5172777
18:07:33	0:05:12	68.101237	63.2842889	77.8195316	71.8813582	77.5172777

18:07:34	0:05:13	69.101237	63.2842889	74.8195316	71.8813582	77.5172777
18:07:35	0:05:14	70.101237	63.2842889	74.8195316	71.8813582	77.5172777
18:07:36	0:05:15	71.101237	62.2842889	71.8195316	71.8813582	77.5172777
18:07:37	0:05:16	71.101237	62.2842889	71.8195316	73.8813582	76.5172777
18:07:38	0:05:17	71.101237	62.2842889	71.8195316	73.8813582	76.5172777
18:07:39	0:05:18	72.101237	62.2842889	71.8195316	73.8813582	76.5172777
18:07:40	0:05:19	73.101237	62.2842889	73.8195316	71.8813582	77.5172777
18:07:41	0:05:20	74.101237	62.2842889	74.8195316	69.8813582	79.5172777
18:07:42	0:05:21	75.101237	62.2842889	74.8195316	69.8813582	80.5172777
18:07:43	0:05:22	76.101237	62.2842889	73.8195316	69.8813582	80.5172777
18:07:44	0:05:23	76.101237	62.2842889	73.8195316	69.8813582	81.5172777
18:07:45	0:05:24	76.101237	62.2842889	73.8195316	69.8813582	84.5172777
18:07:46	0:05:25	77.101237	62.2842889	72.8195316	68.8813582	83.5172777
18:07:47	0:05:26	77.101237	62.2842889	72.8195316	68.8813582	81.5172777
18:07:48	0:05:27	77.101237	65.2842889	72.8195316	68.8813582	81.5172777
18:07:49	0:05:28	77.101237	66.2842889	72.8195316	68.8813582	80.5172777
18:07:50	0:05:29	76.101237	68.2842889	72.8195316	68.8813582	79.5172777
18:07:51	0:05:30	76.101237	69.2842889	72.8195316	68.8813582	78.5172777
18:07:52	0:05:31	72.101237	68.2842889	71.8195316	67.8813582	78.5172777
18:07:53	0:05:32	71.101237	67.2842889	72.8195316	66.8813582	78.5172777
18:07:54	0:05:33	71.101237	67.2842889	73.8195316	66.8813582	78.5172777
18:07:55	0:05:34	71.101237	66.2842889	74.8195316	66.8813582	78.5172777
18:07:56	0:05:35	71.101237	66.2842889	74.8195316	66.8813582	78.5172777
18:07:57	0:05:36	71.101237	66.2842889	74.8195316	66.8813582	79.5172777
18:07:58	0:05:37	71.101237	66.2842889	74.8195316	65.8813582	79.5172777
18:07:59	0:05:38	71.101237	66.2842889	74.8195316	66.8813582	79.5172777
18:08:00	0:05:39	71.101237	66.2842889	73.8195316	66.8813582	81.5172777
18:08:01	0:05:40	71.101237	65.2842889	73.8195316	66.8813582	82.5172777
18:08:02	0:05:41	71.101237	64.2842889	73.8195316	65.8813582	82.5172777
18:08:03	0:05:42	72.101237	64.2842889	73.8195316	65.8813582	83.5172777
18:08:04	0:05:43	72.101237	64.2842889	73.8195316	65.8813582	83.5172777
18:08:05	0:05:44	72.101237	64.2842889	73.8195316	66.8813582	81.5172777
18:08:06	0:05:45	72.101237	64.2842889	73.8195316	66.8813582	81.5172777
18:08:07	0:05:46	72.101237	65.2842889	72.8195316	67.8813582	79.5172777
18:08:08	0:05:47	72.101237	65.2842889	72.8195316	67.8813582	77.5172777
18:08:09	0:05:48	72.101237	65.2842889	72.8195316	67.8813582	77.5172777
18:08:10	0:05:49	78.101237	65.2842889	72.8195316	68.8813582	78.5172777
18:08:11	0:05:50	78.101237	65.2842889	72.8195316	67.8813582	78.5172777
18:08:12	0:05:51	76.101237	66.2842889	71.8195316	66.8813582	78.5172777
18:08:13	0:05:52	67.101237	66.2842889	71.8195316	66.8813582	78.5172777
18:08:14	0:05:53	64.101237	66.2842889	71.8195316	65.8813582	76.5172777
18:08:15	0:05:54	64.101237	66.2842889	71.8195316	64.8813582	75.5172777
18:08:16	0:05:55	64.101237	67.2842889	71.8195316	64.8813582	75.5172777
18:08:17	0:05:56	64.101237	67.2842889	71.8195316	65.8813582	74.5172777
18:08:18	0:05:57	65.101237	67.2842889	71.8195316	65.8813582	70.5172777

18:08:19	0:05:58	65.101237	67.2842889	71.8195316	66.8813582	69.5172777
18:08:20	0:05:59	67.101237	67.2842889	71.8195316	67.8813582	69.5172777
18:08:21	0:06:00	68.101237	68.2842889	72.8195316	67.8813582	69.5172777
18:08:22	0:06:01	68.101237	68.2842889	72.8195316	67.8813582	70.5172777
18:08:23	0:06:02	70.101237	68.2842889	78.8195316	67.8813582	70.5172777
18:08:24	0:06:03	70.101237	69.2842889	81.8195316	67.8813582	70.5172777
18:08:25	0:06:04	73.101237	69.2842889	82.8195316	67.8813582	72.5172777
18:08:26	0:06:05	73.101237	69.2842889	82.8195316	65.8813582	74.5172777
18:08:27	0:06:06	74.101237	69.2842889	83.8195316	66.8813582	75.5172777
18:08:28	0:06:07	75.101237	69.2842889	82.8195316	66.8813582	75.5172777
18:08:29	0:06:08	75.101237	69.2842889	80.8195316	66.8813582	76.5172777
18:08:30	0:06:09	75.101237	69.2842889	79.8195316	66.8813582	76.5172777
18:08:31	0:06:10	75.101237	69.2842889	79.8195316	68.8813582	77.5172777
18:08:32	0:06:11	75.101237	69.2842889	77.8195316	68.8813582	78.5172777
18:08:33	0:06:12	74.101237	68.2842889	77.8195316	68.8813582	78.5172777
18:08:34	0:06:13	75.101237	67.2842889	77.8195316	69.8813582	79.5172777
18:08:35	0:06:14	78.101237	66.2842889	77.8195316	69.8813582	79.5172777
18:08:36	0:06:15	78.101237	66.2842889	77.8195316	69.8813582	78.5172777
18:08:37	0:06:16	78.101237	66.2842889	76.8195316	69.8813582	77.5172777
18:08:38	0:06:17	78.101237	66.2842889	73.8195316	69.8813582	75.5172777
18:08:39	0:06:18	78.101237	66.2842889	72.8195316	70.8813582	75.5172777
18:08:40	0:06:19	77.101237	66.2842889	71.8195316	69.8813582	73.5172777
18:08:41	0:06:20	78.101237	67.2842889	71.8195316	69.8813582	72.5172777
18:08:42	0:06:21	78.101237	67.2842889	73.8195316	69.8813582	71.5172777
18:08:43	0:06:22	77.101237	67.2842889	73.8195316	69.8813582	72.5172777
18:08:44	0:06:23	77.101237	67.2842889	73.8195316	69.8813582	73.5172777
18:08:45	0:06:24	77.101237	67.2842889	73.8195316	69.8813582	73.5172777
18:08:46	0:06:25	77.101237	67.2842889	68.8195316	69.8813582	70.5172777
18:08:47	0:06:26	77.101237	67.2842889	67.8195316	71.8813582	68.5172777
18:08:48	0:06:27	76.101237	67.2842889	67.8195316	71.8813582	67.5172777
18:08:49	0:06:28	75.101237	66.2842889	67.8195316	71.8813582	67.5172777
18:08:50	0:06:29	75.101237	66.2842889	67.8195316	71.8813582	65.5172777
18:08:51	0:06:30	75.101237	66.2842889	67.8195316	72.8813582	66.5172777
18:08:52	0:06:31	75.101237	67.2842889	67.8195316	72.8813582	67.5172777
18:08:53	0:06:32	75.101237	67.2842889	67.8195316	73.8813582	67.5172777
18:08:54	0:06:33	75.101237	67.2842889	67.8195316	74.8813582	67.5172777
18:08:55	0:06:34	74.101237	67.2842889	68.8195316	74.8813582	66.5172777
18:08:56	0:06:35	73.101237	67.2842889	68.8195316	75.8813582	65.5172777
18:08:57	0:06:36	72.101237	67.2842889	68.8195316	75.8813582	63.5172777
18:08:58	0:06:37	69.101237	67.2842889	68.8195316	75.8813582	63.5172777
18:08:59	0:06:38	68.101237	67.2842889	68.8195316	77.8813582	63.5172777
18:09:00	0:06:39	68.101237	67.2842889	68.8195316	77.8813582	63.5172777
18:09:01	0:06:40	65.101237	70.2842889	69.8195316	79.8813582	65.5172777
18:09:02	0:06:41	65.101237	70.2842889	69.8195316	78.8813582	65.5172777
18:09:03	0:06:42	65.101237	76.2842889	70.8195316	76.8813582	66.5172777

18:09:04	0:06:43	65.101237	76.2842889	71.8195316	76.8813582	66.5172777
18:09:05	0:06:44	65.101237	78.2842889	71.8195316	73.8813582	68.5172777
18:09:06	0:06:45	65.101237	77.2842889	71.8195316	70.8813582	68.5172777
18:09:07	0:06:46	65.101237	72.2842889	71.8195316	70.8813582	69.5172777
18:09:08	0:06:47	66.101237	69.2842889	71.8195316	67.8813582	70.5172777
18:09:09	0:06:48	79.101237	67.2842889	71.8195316	66.8813582	70.5172777
18:09:10	0:06:49	79.101237	66.2842889	71.8195316	66.8813582	70.5172777
18:09:11	0:06:50	83.101237	66.2842889	70.8195316	66.8813582	75.5172777
18:09:12	0:06:51	83.101237	65.2842889	70.8195316	66.8813582	77.5172777
18:09:13	0:06:52	83.101237	65.2842889	70.8195316	66.8813582	79.5172777
18:09:14	0:06:53	83.101237	65.2842889	70.8195316	66.8813582	79.5172777
18:09:15	0:06:54	82.101237	65.2842889	71.8195316	66.8813582	79.5172777
18:09:16	0:06:55	82.101237	63.2842889	71.8195316	66.8813582	78.5172777
18:09:17	0:06:56	81.101237	62.2842889	71.8195316	66.8813582	76.5172777
18:09:18	0:06:57	80.101237	62.2842889	70.8195316	67.8813582	70.5172777
18:09:19	0:06:58	79.101237	62.2842889	70.8195316	67.8813582	69.5172777
18:09:20	0:06:59	79.101237	62.2842889	68.8195316	67.8813582	69.5172777
18:09:21	0:07:00	78.101237	62.2842889	68.8195316	68.8813582	69.5172777
18:09:22	0:07:01	77.101237	62.2842889	68.8195316	68.8813582	69.5172777
18:09:23	0:07:02	69.101237	62.2842889	69.8195316	68.8813582	69.5172777
18:09:24	0:07:03	67.101237	62.2842889	70.8195316	68.8813582	67.5172777
18:09:25	0:07:04	66.101237	62.2842889	70.8195316	68.8813582	67.5172777
18:09:26	0:07:05	67.101237	62.2842889	70.8195316	71.8813582	66.5172777
18:09:27	0:07:06	68.101237	62.2842889	68.8195316	71.8813582	65.5172777
18:09:28	0:07:07	69.101237	62.2842889	66.8195316	71.8813582	64.5172777
18:09:29	0:07:08	71.101237	60.2842889	66.8195316	71.8813582	64.5172777
18:09:30	0:07:09	73.101237	60.2842889	66.8195316	71.8813582	65.5172777
18:09:31	0:07:10	73.101237	60.2842889	69.8195316	73.8813582	66.5172777
18:09:32	0:07:11	73.101237	59.2842889	69.8195316	74.8813582	68.5172777
18:09:33	0:07:12	73.101237	60.2842889	68.8195316	74.8813582	68.5172777
18:09:34	0:07:13	74.101237	61.2842889	68.8195316	75.8813582	68.5172777
18:09:35	0:07:14	75.101237	61.2842889	72.8195316	75.8813582	68.5172777
18:09:36	0:07:15	75.101237	61.2842889	72.8195316	76.8813582	65.5172777
18:09:37	0:07:16	75.101237	61.2842889	72.8195316	79.8813582	65.5172777
18:09:38	0:07:17	75.101237	61.2842889	70.8195316	72.4872373	64.5172777
18:09:39	0:07:18	76.101237	61.2842889	70.8195316	71.9417828	64.5172777
18:09:40	0:07:19	77.101237	60.2842889	70.8195316	71.5781464	65.5172777
18:09:41	0:07:20	82.101237	60.2842889	71.8195316	71.8508737	66.5172777
18:09:42	0:07:21	82.101237	61.2842889	72.8195316	72.0326919	67.5172777
18:09:43	0:07:22	71.8508737	62.2842889	71.8195316	82.8813582	67.5172777
18:09:44	0:07:23	71.5781464	62.2842889	71.8195316	77.8813582	70.5172777
18:09:45	0:07:24	71.2145101	62.2842889	71.8195316	74.8813582	71.5172777
18:09:46	0:07:25	70.8508737	62.2842889	71.8195316	72.8813582	72.5172777
18:09:47	0:07:26	71.123601	65.2842889	71.8195316	71.8813582	73.5172777
18:09:48	0:07:27	71.3963282	65.2842889	71.8195316	72.8813582	73.5172777

18:09:49	0:07:28	71.6690555	68.2842889	71.8195316	72.8813582	73.5172777
18:09:50	0:07:29	72.4872373	70.2842889	74.8195316	73.8813582	73.5172777
18:09:51	0:07:30	72.6690555	71.2842889	74.8195316	74.8813582	72.5172777
18:09:52	0:07:31	72.8508737	71.2842889	74.8195316	74.8813582	72.5172777
18:09:53	0:07:32	73.0326919	73.2842889	76.8195316	73.8813582	71.5172777
18:09:54	0:07:33	73.0326919	73.2842889	76.8195316	73.8813582	71.5172777
18:09:55	0:07:34	72.3963282	73.2842889	76.8195316	73.8813582	68.5172777
18:09:56	0:07:35	72.5781464	73.2842889	77.8195316	73.8813582	68.5172777
18:09:57	0:07:36	83.101237	73.2842889	77.8195316	72.8813582	68.5172777
18:09:58	0:07:37	83.101237	73.2842889	77.8195316	72.8813582	68.5172777
18:09:59	0:07:38	83.101237	73.2842889	78.8195316	72.8813582	68.5172777
18:10:00	0:07:39	83.101237	73.2842889	78.8195316	72.8813582	68.5172777
18:10:01	0:07:40	83.101237	73.2842889	77.8195316	71.8813582	68.5172777
18:10:02	0:07:41	84.101237	73.2842889	76.8195316	70.8813582	71.5172777
18:10:03	0:07:42	84.101237	73.2842889	76.8195316	69.8813582	72.5172777
18:10:04	0:07:43	85.101237	73.2842889	74.8195316	69.8813582	73.5172777
18:10:05	0:07:44	85.101237	75.2842889	72.8195316	69.8813582	73.5172777
18:10:06	0:07:45	86.101237	76.2842889	71.8195316	67.8813582	73.5172777
18:10:07	0:07:46	87.101237	76.2842889	71.8195316	67.8813582	73.5172777
18:10:08	0:07:47	87.101237	76.2842889	71.8195316	67.8813582	72.5172777
18:10:09	0:07:48	87.101237	75.2842889	70.8195316	67.8813582	72.5172777
18:10:10	0:07:49	87.101237	75.2842889	70.8195316	67.8813582	72.5172777
18:10:11	0:07:50	87.101237	75.2842889	70.8195316	67.8813582	72.5172777
18:10:12	0:07:51	74.8508737	75.2842889	71.8195316	67.8813582	70.5172777
18:10:13	0:07:52	75.123601	74.2842889	71.8195316	67.8813582	70.5172777
18:10:14	0:07:53	75.2145101	74.2842889	71.8195316	67.8813582	70.5172777
18:10:15	0:07:54	75.0326919	73.2842889	72.8195316	67.8813582	70.5172777
18:10:16	0:07:55	74.7599646	73.2842889	70.8195316	66.8813582	70.5172777
18:10:17	0:07:56	75.0326919	73.2842889	70.8195316	66.8813582	70.5172777
18:10:18	0:07:57	75.123601	73.2842889	70.8195316	66.8813582	70.5172777
18:10:19	0:07:58	87.101237	75.2842889	69.8195316	66.8813582	69.5172777
18:10:20	0:07:59	84.101237	75.2842889	69.8195316	67.8813582	69.5172777
18:10:21	0:08:00	83.101237	75.2842889	69.8195316	67.8813582	69.5172777
18:10:22	0:08:01	81.101237	75.2842889	72.8195316	78.8813582	70.5172777
18:10:23	0:08:02	80.101237	75.2842889	73.8195316	81.8813582	70.5172777
18:10:24	0:08:03	79.101237	75.2842889	73.8195316	85.8813582	71.5172777
18:10:25	0:08:04	79.101237	75.2842889	73.8195316	86.8813582	71.5172777
18:10:26	0:08:05	80.101237	74.2842889	72.8195316	86.8813582	71.5172777
18:10:27	0:08:06	79.101237	72.2842889	71.8195316	79.8813582	70.5172777
18:10:28	0:08:07	79.101237	69.2842889	71.8195316	75.8813582	70.5172777
18:10:29	0:08:08	77.101237	68.2842889	70.8195316	72.8813582	70.5172777
18:10:30	0:08:09	74.101237	68.2842889	70.8195316	71.8813582	70.5172777
18:10:31	0:08:10	71.101237	68.2842889	70.8195316	70.8813582	70.5172777
18:10:32	0:08:11	71.101237	66.2842889	70.8195316	70.8813582	71.5172777
18:10:33	0:08:12	71.101237	66.2842889	70.8195316	70.8813582	71.5172777

18:10:34	0:08:13	73.101237	65.2842889	71.8195316	69.8813582	71.5172777
18:10:35	0:08:14	75.101237	65.2842889	71.8195316	68.8813582	71.5172777
18:10:36	0:08:15	76.101237	65.2842889	71.8195316	67.8813582	71.5172777
18:10:37	0:08:16	76.101237	68.2842889	71.8195316	67.8813582	71.5172777
18:10:38	0:08:17	76.101237	68.2842889	70.8195316	66.8813582	70.5172777
18:10:39	0:08:18	76.101237	67.2842889	70.8195316	66.8813582	70.5172777
18:10:40	0:08:19	76.101237	66.2842889	70.8195316	64.8813582	69.5172777
18:10:41	0:08:20	76.101237	66.2842889	70.8195316	64.8813582	69.5172777
18:10:42	0:08:21	76.101237	65.2842889	70.8195316	64.8813582	69.5172777
18:10:43	0:08:22	76.101237	65.2842889	72.8195316	64.8813582	69.5172777
18:10:44	0:08:23	76.101237	64.2842889	72.8195316	64.8813582	70.5172777
18:10:45	0:08:24	77.101237	64.2842889	72.8195316	64.8813582	71.5172777
18:10:46	0:08:25	79.101237	65.2842889	72.8195316	64.8813582	71.5172777
18:10:47	0:08:26	79.101237	65.2842889	71.8195316	64.8813582	72.5172777
18:10:48	0:08:27	79.101237	65.2842889	71.8195316	64.8813582	72.5172777
18:10:49	0:08:28	79.101237	66.2842889	71.8195316	62.8813582	71.5172777
18:10:50	0:08:29	79.101237	66.2842889	71.8195316	62.8813582	72.5172777
18:10:51	0:08:30	80.101237	66.2842889	72.8195316	62.8813582	74.5172777
18:10:52	0:08:31	80.101237	66.2842889	76.8195316	61.8813582	74.5172777
18:10:53	0:08:32	76.101237	67.2842889	76.8195316	61.8813582	76.5172777
18:10:54	0:08:33	76.101237	67.2842889	77.8195316	62.8813582	76.5172777
18:10:55	0:08:34	70.101237	67.2842889	78.8195316	64.8813582	74.5172777
18:10:56	0:08:35	69.101237	67.2842889	78.8195316	65.8813582	75.5172777
18:10:57	0:08:36	69.101237	67.2842889	82.8195316	66.8813582	75.5172777
18:10:58	0:08:37	69.101237	67.2842889	83.8195316	66.8813582	75.5172777
18:10:59	0:08:38	69.101237	67.2842889	82.8195316	66.8813582	75.5172777
18:11:00	0:08:39	69.101237	68.2842889	78.8195316	65.8813582	72.5172777
18:11:01	0:08:40	68.101237	70.2842889	75.8195316	65.8813582	72.5172777
18:11:02	0:08:41	68.101237	70.2842889	73.8195316	65.8813582	73.5172777
18:11:03	0:08:42	68.101237	74.2842889	72.8195316	65.8813582	74.5172777
18:11:04	0:08:43	68.101237	74.2842889	71.8195316	65.8813582	74.5172777
18:11:05	0:08:44	65.101237	74.2842889	70.8195316	66.8813582	75.5172777
18:11:06	0:08:45	65.101237	77.2842889	70.8195316	65.8813582	75.5172777
18:11:07	0:08:46	65.101237	77.2842889	69.8195316	65.8813582	75.5172777
18:11:08	0:08:47	65.101237	77.2842889	67.8195316	65.8813582	72.5172777
18:11:09	0:08:48	67.101237	77.2842889	66.8195316	65.8813582	71.5172777
18:11:10	0:08:49	71.101237	77.2842889	66.8195316	65.8813582	70.5172777
18:11:11	0:08:50	71.101237	77.2842889	66.8195316	65.8813582	69.5172777
18:11:12	0:08:51	71.101237	77.2842889	66.8195316	65.8813582	70.5172777
18:11:13	0:08:52	73.101237	71.2842889	65.8195316	64.8813582	70.5172777
18:11:14	0:08:53	74.101237	71.2842889	65.8195316	65.8813582	70.5172777
18:11:15	0:08:54	74.101237	69.2842889	64.8195316	65.8813582	72.5172777
18:11:16	0:08:55	75.101237	68.2842889	64.8195316	65.8813582	73.5172777
18:11:17	0:08:56	75.101237	68.2842889	64.8195316	64.8813582	72.5172777
18:11:18	0:08:57	76.101237	68.2842889	64.8195316	63.8813582	72.5172777

18:11:19	0:08:58	76.101237	68.2842889	65.8195316	63.8813582	72.5172777
18:11:20	0:08:59	77.101237	68.2842889	66.8195316	63.8813582	73.5172777
18:11:21	0:09:00	77.101237	68.2842889	66.8195316	63.8813582	72.5172777
18:11:22	0:09:01	77.101237	68.2842889	66.8195316	63.8813582	71.5172777
18:11:23	0:09:02	77.101237	68.2842889	67.8195316	63.8813582	71.5172777
18:11:24	0:09:03	75.101237	68.2842889	67.8195316	63.8813582	70.5172777
18:11:25	0:09:04	75.101237	69.2842889	67.8195316	63.8813582	71.5172777
18:11:26	0:09:05	74.101237	68.2842889	67.8195316	63.8813582	71.5172777
18:11:27	0:09:06	74.101237	68.2842889	67.8195316	62.8813582	72.5172777
18:11:28	0:09:07	75.101237	68.2842889	67.8195316	62.8813582	74.5172777
18:11:29	0:09:08	75.101237	69.2842889	67.8195316	63.8813582	75.5172777
18:11:30	0:09:09	75.101237	69.2842889	67.8195316	64.8813582	75.5172777
18:11:31	0:09:10	76.101237	68.2842889	67.8195316	67.8813582	75.5172777
18:11:32	0:09:11	79.101237	68.2842889	67.8195316	67.8813582	76.5172777
18:11:33	0:09:12	79.101237	68.2842889	67.8195316	68.8813582	76.5172777
18:11:34	0:09:13	79.101237	68.2842889	68.8195316	68.8813582	76.5172777
18:11:35	0:09:14	80.101237	68.2842889	68.8195316	68.8813582	75.5172777
18:11:36	0:09:15	82.101237	68.2842889	67.8195316	67.8813582	72.5172777
18:11:37	0:09:16	82.101237	68.2842889	67.8195316	67.8813582	71.5172777
18:11:38	0:09:17	83.101237	66.2842889	69.8195316	67.8813582	71.5172777
18:11:39	0:09:18	84.101237	66.2842889	71.8195316	67.8813582	71.5172777
18:11:40	0:09:19	84.101237	65.2842889	70.8195316	67.8813582	74.5172777
18:11:41	0:09:20	84.101237	65.2842889	70.8195316	66.8813582	79.5172777
18:11:42	0:09:21	84.101237	65.2842889	70.8195316	66.8813582	79.5172777
18:11:43	0:09:22	84.101237	65.2842889	71.8195316	67.8813582	82.5172777
18:11:44	0:09:23	84.101237	65.2842889	71.8195316	67.8813582	83.5172777
18:11:45	0:09:24	84.101237	67.2842889	71.8195316	67.8813582	84.5172777
18:11:46	0:09:25	84.101237	67.2842889	72.8195316	67.8813582	84.5172777
18:11:47	0:09:26	85.101237	67.2842889	71.8195316	68.8813582	84.5172777
18:11:48	0:09:27	85.101237	67.2842889	71.8195316	68.8813582	80.5172777
18:11:49	0:09:28	84.101237	67.2842889	71.8195316	68.8813582	80.5172777
18:11:50	0:09:29	84.101237	67.2842889	71.8195316	67.8813582	72.5172777
18:11:51	0:09:30	83.101237	67.2842889	71.8195316	67.8813582	71.5172777
18:11:52	0:09:31	83.101237	67.2842889	72.8195316	67.8813582	70.5172777
18:11:53	0:09:32	83.101237	67.2842889	72.8195316	67.8813582	70.5172777
18:11:54	0:09:33	83.101237	67.2842889	73.8195316	67.8813582	69.5172777
18:11:55	0:09:34	79.101237	67.2842889	73.8195316	68.8813582	68.5172777
18:11:56	0:09:35	75.101237	66.2842889	73.8195316	67.8813582	67.5172777
18:11:57	0:09:36	70.101237	69.2842889	74.8195316	67.8813582	67.5172777
18:11:58	0:09:37	67.101237	69.2842889	75.8195316	67.8813582	67.5172777
18:11:59	0:09:38	65.101237	73.2842889	75.8195316	67.8813582	68.5172777
18:12:00	0:09:39	65.101237	75.2842889	75.8195316	67.8813582	68.5172777
18:12:01	0:09:40	63.101237	75.2842889	74.8195316	67.8813582	68.5172777
18:12:02	0:09:41	61.101237	73.2842889	74.8195316	67.8813582	68.5172777
18:12:03	0:09:42	61.101237	73.2842889	74.8195316	67.8813582	68.5172777

18:12:04	0:09:43	61.101237	71.2842889	74.8195316	67.8813582	68.5172777
18:12:05	0:09:44	60.101237	70.2842889	74.8195316	67.8813582	68.5172777
18:12:06	0:09:45	60.101237	69.2842889	75.8195316	67.8813582	69.5172777
18:12:07	0:09:46	60.101237	69.2842889	75.8195316	67.8813582	69.5172777
18:12:08	0:09:47	60.101237	69.2842889	74.8195316	67.8813582	69.5172777
18:12:09	0:09:48	60.101237	69.2842889	76.8195316	67.8813582	69.5172777
18:12:10	0:09:49	60.101237	69.2842889	76.8195316	67.8813582	70.5172777
18:12:11	0:09:50	60.101237	68.2842889	78.8195316	67.8813582	70.5172777
18:12:12	0:09:51	60.101237	68.2842889	83.8195316	67.8813582	71.5172777
18:12:13	0:09:52	60.101237	68.2842889	83.8195316	67.8813582	71.5172777
18:12:14	0:09:53	60.101237	68.2842889	85.8195316	67.8813582	71.5172777
18:12:15	0:09:54	60.101237	67.2842889	86.8195316	67.8813582	72.5172777
18:12:16	0:09:55	60.101237	67.2842889	86.8195316	67.8813582	72.5172777
18:12:17	0:09:56	60.101237	69.2842889	78.8195316	68.8813582	73.5172777
18:12:18	0:09:57	60.101237	69.2842889	78.8195316	68.8813582	73.5172777
18:12:19	0:09:58	60.101237	69.2842889	77.8195316	68.8813582	73.5172777
18:12:20	0:09:59	60.101237	69.2842889	76.8195316	68.8813582	70.5172777
18:12:21	0:10:00	62.101237	68.2842889	75.8195316	68.8813582	70.5172777
18:12:22	0:10:01	62.101237	68.2842889	75.8195316	69.8813582	71.5172777
18:12:23	0:10:02	62.101237	67.2842889	75.8195316	69.8813582	71.5172777
18:12:24	0:10:03	62.101237	67.2842889	76.8195316	68.8813582	71.5172777
18:12:25	0:10:04	63.101237	67.2842889	76.8195316	68.8813582	70.5172777
18:12:26	0:10:05	63.101237	67.2842889	76.8195316	68.8813582	69.5172777
18:12:27	0:10:06	63.101237	67.2842889	73.8195316	68.8813582	69.5172777
18:12:28	0:10:07	64.101237	67.2842889	71.8195316	66.8813582	68.5172777
18:12:29	0:10:08	64.101237	67.2842889	70.8195316	66.8813582	68.5172777
18:12:30	0:10:09	63.101237	67.2842889	69.8195316	65.8813582	70.5172777
18:12:31	0:10:10	63.101237	67.2842889	69.8195316	65.8813582	71.5172777
18:12:32	0:10:11	63.101237	66.2842889	69.8195316	64.8813582	72.5172777
18:12:33	0:10:12	62.101237	66.2842889	69.8195316	64.8813582	72.5172777
18:12:34	0:10:13	62.101237	65.2842889	69.8195316	64.8813582	73.5172777
18:12:35	0:10:14	61.101237	65.2842889	69.8195316	65.8813582	73.5172777
18:12:36	0:10:15	61.101237	65.2842889	69.8195316	65.8813582	73.5172777
18:12:37	0:10:16	59.101237	65.2842889	69.8195316	65.8813582	72.5172777
18:12:38	0:10:17	59.101237	66.2842889	68.8195316	66.8813582	72.5172777
18:12:39	0:10:18	59.101237	66.2842889	67.8195316	66.8813582	73.5172777
18:12:40	0:10:19	59.101237	67.2842889	67.8195316	66.8813582	72.5172777
18:12:41	0:10:20	59.101237	67.2842889	65.8195316	65.8813582	70.5172777
18:12:42	0:10:21	59.101237	67.2842889	65.8195316	64.8813582	69.5172777
18:12:43	0:10:22	58.101237	67.2842889	65.8195316	64.8813582	67.5172777
18:12:44	0:10:23	57.101237	67.2842889	65.8195316	65.8813582	67.5172777
18:12:45	0:10:24	57.101237	67.2842889	64.8195316	66.8813582	68.5172777
18:12:46	0:10:25	57.101237	66.2842889	65.8195316	66.8813582	69.5172777
18:12:47	0:10:26	57.101237	66.2842889	64.8195316	66.8813582	69.5172777
18:12:48	0:10:27	57.101237	66.2842889	64.8195316	65.8813582	71.5172777

18:12:49	0:10:28	58.101237	65.2842889	64.8195316	65.8813582	72.5172777
18:12:50	0:10:29	58.101237	65.2842889	64.8195316	66.8813582	73.5172777
18:12:51	0:10:30	61.101237	64.2842889	64.8195316	66.8813582	73.5172777
18:12:52	0:10:31	61.101237	64.2842889	65.8195316	66.8813582	74.5172777
18:12:53	0:10:32	62.101237	64.2842889	65.8195316	66.8813582	74.5172777
18:12:54	0:10:33	62.101237	64.2842889	65.8195316	66.8813582	75.5172777
18:12:55	0:10:34	62.101237	64.2842889	66.8195316	66.8813582	75.5172777
18:12:56	0:10:35	62.101237	64.2842889	66.8195316	66.8813582	75.5172777
18:12:57	0:10:36	63.101237	64.2842889	66.8195316	66.8813582	74.5172777
18:12:58	0:10:37	63.101237	64.2842889	66.8195316	66.8813582	74.5172777
18:12:59	0:10:38	63.101237	64.2842889	66.8195316	66.8813582	74.5172777
18:13:00	0:10:39	64.101237	65.2842889	67.8195316	66.8813582	74.5172777
18:13:01	0:10:40	64.101237	65.2842889	68.8195316	66.8813582	74.5172777
18:13:02	0:10:41	64.101237	65.2842889	69.8195316	66.8813582	74.5172777
18:13:03	0:10:42	64.101237	65.2842889	69.8195316	68.8813582	74.5172777
18:13:04	0:10:43	64.101237	65.2842889	70.8195316	70.8813582	73.5172777
18:13:05	0:10:44	64.101237	65.2842889	71.8195316	71.8813582	71.5172777
18:13:06	0:10:45	65.101237	65.2842889	71.8195316	71.8813582	72.5172777
18:13:07	0:10:46	65.101237	65.2842889	71.8195316	71.8813582	72.5172777
18:13:08	0:10:47	69.101237	65.2842889	70.8195316	71.8813582	72.5172777
18:13:09	0:10:48	69.101237	65.2842889	70.8195316	71.8813582	71.5172777
18:13:10	0:10:49	69.101237	65.2842889	70.8195316	75.8813582	71.5172777
18:13:11	0:10:50	68.101237	65.2842889	70.8195316	73.8813582	70.5172777
18:13:12	0:10:51	67.101237	65.2842889	70.8195316	69.8813582	70.5172777
18:13:13	0:10:52	67.101237	65.2842889	69.8195316	67.8813582	71.5172777
18:13:14	0:10:53	65.101237	65.2842889	69.8195316	67.8813582	71.5172777
18:13:15	0:10:54	65.101237	65.2842889	69.8195316	66.8813582	71.5172777
18:13:16	0:10:55	65.101237	66.2842889	70.8195316	67.8813582	71.5172777
18:13:17	0:10:56	65.101237	66.2842889	71.8195316	67.8813582	72.5172777
18:13:18	0:10:57	64.101237	66.2842889	71.8195316	67.8813582	74.5172777
18:13:19	0:10:58	64.101237	66.2842889	71.8195316	67.8813582	73.5172777
18:13:20	0:10:59	63.101237	66.2842889	71.8195316	67.8813582	73.5172777
18:13:21	0:11:00	62.101237	66.2842889	71.8195316	67.8813582	74.5172777
18:13:22	0:11:01	62.101237	66.2842889	70.8195316	67.8813582	74.5172777
18:13:23	0:11:02	59.101237	66.2842889	70.8195316	67.8813582	74.5172777
18:13:24	0:11:03	58.101237	66.2842889	70.8195316	67.8813582	75.5172777
18:13:25	0:11:04	58.101237	66.2842889	70.8195316	67.8813582	76.5172777
18:13:26	0:11:05	57.101237	64.2842889	69.8195316	67.8813582	76.5172777
18:13:27	0:11:06	57.101237	64.2842889	69.8195316	67.8813582	75.5172777
18:13:28	0:11:07	57.101237	64.2842889	69.8195316	67.8813582	73.5172777
18:13:29	0:11:08	57.101237	64.2842889	69.8195316	67.8813582	72.5172777
18:13:30	0:11:09	57.101237	64.2842889	69.8195316	67.8813582	72.5172777
18:13:31	0:11:10	56.101237	65.2842889	69.8195316	67.8813582	72.5172777
18:13:32	0:11:11	53.101237	65.2842889	69.8195316	67.8813582	68.5172777
18:13:33	0:11:12	53.101237	65.2842889	69.8195316	67.8813582	68.5172777

18:13:34	0:11:13	53.101237	67.2842889	70.8195316	67.8813582	68.5172777
18:13:35	0:11:14	52.101237	71.2842889	71.8195316	67.8813582	68.5172777
18:13:36	0:11:15	51.101237	71.2842889	71.8195316	67.8813582	69.5172777
18:13:37	0:11:16	51.101237	72.2842889	72.8195316	67.8813582	69.5172777
18:13:38	0:11:17	51.101237	72.2842889	72.8195316	67.8813582	69.5172777
18:13:39	0:11:18	51.101237	72.2842889	72.8195316	67.8813582	68.5172777
18:13:40	0:11:19	51.101237	72.2842889	72.8195316	67.8813582	68.5172777
18:13:41	0:11:20	52.101237	71.2842889	72.8195316	68.8813582	69.5172777
18:13:42	0:11:21	53.101237	72.2842889	73.8195316	68.8813582	69.5172777
18:13:43	0:11:22	56.101237	72.2842889	73.8195316	68.8813582	71.5172777
18:13:44	0:11:23	60.101237	72.2842889	73.8195316	68.8813582	71.5172777
18:13:45	0:11:24	62.101237	72.2842889	73.8195316	69.8813582	72.5172777
18:13:46	0:11:25	64.101237	72.2842889	75.8195316	69.8813582	72.5172777
18:13:47	0:11:26	65.101237	72.2842889	77.8195316	69.8813582	71.5172777
18:13:48	0:11:27	67.101237	72.2842889	78.8195316	70.8813582	70.5172777
18:13:49	0:11:28	68.101237	72.2842889	79.8195316	70.8813582	70.5172777
18:13:50	0:11:29	69.101237	71.2842889	81.8195316	69.8813582	69.5172777
18:13:51	0:11:30	69.101237	66.2842889	81.8195316	68.8813582	69.5172777
18:13:52	0:11:31	70.101237	66.2842889	81.8195316	68.8813582	69.5172777
18:13:53	0:11:32	71.101237	66.2842889	81.8195316	68.8813582	69.5172777
18:13:54	0:11:33	73.101237	66.2842889	78.8195316	68.8813582	69.5172777
18:13:55	0:11:34	74.101237	66.2842889	77.8195316	69.8813582	70.5172777
18:13:56	0:11:35	76.101237	66.2842889	76.8195316	70.8813582	70.5172777
18:13:57	0:11:36	76.101237	66.2842889	76.8195316	70.8813582	70.5172777
18:13:58	0:11:37	77.101237	66.2842889	76.8195316	71.8813582	70.5172777
18:13:59	0:11:38	77.101237	65.2842889	76.8195316	72.8813582	73.5172777
18:14:00	0:11:39	77.101237	65.2842889	76.8195316	72.8813582	73.5172777
18:14:01	0:11:40	76.101237	64.2842889	76.8195316	72.8813582	76.5172777
18:14:02	0:11:41	75.101237	64.2842889	76.8195316	72.8813582	76.5172777
18:14:03	0:11:42	74.101237	64.2842889	76.8195316	71.8813582	78.5172777
18:14:04	0:11:43	73.101237	64.2842889	73.8195316	70.8813582	79.5172777
18:14:05	0:11:44	70.101237	64.2842889	72.8195316	70.8813582	79.5172777
18:14:06	0:11:45	69.101237	61.2842889	72.8195316	70.8813582	78.5172777
18:14:07	0:11:46	69.101237	61.2842889	71.8195316	70.8813582	77.5172777
18:14:08	0:11:47	69.101237	62.2842889	71.8195316	70.8813582	76.5172777
18:14:09	0:11:48	68.101237	62.2842889	73.8195316	70.8813582	76.5172777
18:14:10	0:11:49	72.101237	62.2842889	73.8195316	71.8813582	74.5172777
18:14:11	0:11:50	72.101237	62.2842889	72.8195316	72.8813582	74.5172777
18:14:12	0:11:51	71.101237	62.2842889	71.8195316	71.8813582	75.5172777
18:14:13	0:11:52	70.101237	61.2842889	71.8195316	72.8813582	75.5172777
18:14:14	0:11:53	70.101237	61.2842889	71.8195316	72.8813582	75.5172777
18:14:15	0:11:54	70.101237	61.2842889	72.8195316	72.8813582	76.5172777
18:14:16	0:11:55	70.101237	61.2842889	71.8195316	72.8813582	76.5172777
18:14:17	0:11:56	69.101237	61.2842889	71.8195316	73.8813582	76.5172777
18:14:18	0:11:57	69.101237	61.2842889	73.8195316	73.8813582	76.5172777

18:14:19	0:11:58	68.101237	61.2842889	74.8195316	73.8813582	75.5172777
18:14:20	0:11:59	68.101237	62.2842889	74.8195316	72.8813582	73.5172777
18:14:21	0:12:00	67.101237	62.2842889	79.8195316	72.8813582	73.5172777
18:14:22	0:12:01	68.101237	63.2842889	84.8195316	72.8813582	69.5172777
18:14:23	0:12:02	72.101237	64.2842889	87.8195316	71.8813582	68.5172777
18:14:24	0:12:03	72.101237	64.2842889	87.8195316	71.8813582	68.5172777
18:14:25	0:12:04	76.101237	64.2842889	87.8195316	74.8813582	68.5172777
18:14:26	0:12:05	77.101237	71.2842889	87.8195316	74.8813582	68.5172777
18:14:27	0:12:06	77.101237	72.2842889	80.8195316	76.8813582	70.5172777
18:14:28	0:12:07	77.101237	72.2842889	77.8195316	78.8813582	70.5172777
18:14:29	0:12:08	77.101237	72.2842889	74.8195316	77.8813582	70.5172777
18:14:30	0:12:09	76.101237	73.2842889	74.8195316	76.8813582	75.5172777
18:14:31	0:12:10	76.101237	73.2842889	71.8195316	74.8813582	75.5172777
18:14:32	0:12:11	75.101237	73.2842889	70.8195316	73.8813582	75.5172777
18:14:33	0:12:12	75.101237	73.2842889	69.8195316	73.8813582	75.5172777
18:14:34	0:12:13	75.101237	73.2842889	68.8195316	72.8813582	78.5172777
18:14:35	0:12:14	75.101237	72.2842889	68.8195316	71.8813582	78.5172777
18:14:36	0:12:15	75.101237	70.2842889	66.8195316	72.8813582	80.5172777
18:14:37	0:12:16	75.101237	69.2842889	65.8195316	72.8813582	80.5172777
18:14:38	0:12:17	75.101237	69.2842889	66.8195316	72.8813582	81.5172777
18:14:39	0:12:18	75.101237	69.2842889	66.8195316	72.8813582	82.5172777
18:14:40	0:12:19	75.101237	68.2842889	66.8195316	72.8813582	82.5172777
18:14:41	0:12:20	75.101237	68.2842889	66.8195316	72.8813582	79.5172777
18:14:42	0:12:21	76.101237	69.2842889	68.8195316	72.8813582	79.5172777
18:14:43	0:12:22	76.101237	68.2842889	70.8195316	72.8813582	75.5172777
18:14:44	0:12:23	78.101237	67.2842889	70.8195316	72.8813582	74.5172777
18:14:45	0:12:24	78.101237	66.2842889	73.8195316	73.8813582	74.5172777
18:14:46	0:12:25	78.101237	65.2842889	73.8195316	73.8813582	75.5172777
18:14:47	0:12:26	78.101237	64.2842889	73.8195316	73.8813582	74.5172777
18:14:48	0:12:27	77.101237	63.2842889	71.8195316	73.8813582	74.5172777
18:14:49	0:12:28	77.101237	63.2842889	70.8195316	71.8813582	73.5172777
18:14:50	0:12:29	77.101237	63.2842889	70.8195316	70.8813582	73.5172777
18:14:51	0:12:30	77.101237	63.2842889	70.8195316	70.8813582	72.5172777
18:14:52	0:12:31	77.101237	65.2842889	69.8195316	70.8813582	71.5172777
18:14:53	0:12:32	77.101237	65.2842889	69.8195316	70.8813582	71.5172777
18:14:54	0:12:33	76.101237	65.2842889	69.8195316	70.8813582	71.5172777
18:14:55	0:12:34	75.101237	65.2842889	68.8195316	70.8813582	72.5172777
18:14:56	0:12:35	74.101237	65.2842889	68.8195316	69.8813582	73.5172777
18:14:57	0:12:36	74.101237	65.2842889	68.8195316	69.8813582	72.5172777
18:14:58	0:12:37	73.101237	65.2842889	68.8195316	67.8813582	72.5172777
18:14:59	0:12:38	71.101237	65.2842889	68.8195316	67.8813582	72.5172777
18:15:00	0:12:39	69.101237	65.2842889	68.8195316	67.8813582	71.5172777
18:15:01	0:12:40	69.101237	66.2842889	68.8195316	66.8813582	71.5172777
18:15:02	0:12:41	68.101237	67.2842889	69.8195316	66.8813582	71.5172777
18:15:03	0:12:42	68.101237	68.2842889	69.8195316	66.8813582	71.5172777

18:15:04	0:12:43	69.101237	68.2842889	69.8195316	66.8813582	72.5172777
18:15:05	0:12:44	66.101237	68.2842889	69.8195316	66.8813582	73.5172777
18:15:06	0:12:45	65.101237	68.2842889	69.8195316	67.8813582	75.5172777
18:15:07	0:12:46	64.101237	66.2842889	69.8195316	67.8813582	75.5172777
18:15:08	0:12:47	63.101237	66.2842889	69.8195316	68.8813582	75.5172777
18:15:09	0:12:48	63.101237	66.2842889	69.8195316	67.8813582	75.5172777
18:15:10	0:12:49	63.101237	66.2842889	70.8195316	67.8813582	73.5172777
18:15:11	0:12:50	64.101237	66.2842889	71.8195316	67.8813582	71.5172777
18:15:12	0:12:51	66.101237	66.2842889	71.8195316	67.8813582	70.5172777
18:15:13	0:12:52	66.101237	67.2842889	72.8195316	67.8813582	70.5172777
18:15:14	0:12:53	67.101237	67.2842889	72.8195316	67.8813582	69.5172777
18:15:15	0:12:54	67.101237	67.2842889	73.8195316	68.8813582	70.5172777
18:15:16	0:12:55	68.101237	67.2842889	73.8195316	68.8813582	70.5172777
18:15:17	0:12:56	68.101237	67.2842889	74.8195316	68.8813582	70.5172777
18:15:18	0:12:57	68.101237	67.2842889	74.8195316	68.8813582	70.5172777
18:15:19	0:12:58	68.101237	66.2842889	74.8195316	68.8813582	70.5172777
18:15:20	0:12:59	75.101237	66.2842889	73.8195316	68.8813582	70.5172777
18:15:21	0:13:00	75.101237	65.2842889	72.8195316	68.8813582	70.5172777
18:15:22	0:13:01	76.101237	65.2842889	72.8195316	68.8813582	70.5172777
18:15:23	0:13:02	76.101237	65.2842889	72.8195316	67.8813582	70.5172777
18:15:24	0:13:03	75.101237	65.2842889	73.8195316	67.8813582	70.5172777
18:15:25	0:13:04	75.101237	66.2842889	73.8195316	67.8813582	70.5172777
18:15:26	0:13:05	75.101237	67.2842889	74.8195316	66.8813582	70.5172777
18:15:27	0:13:06	75.101237	66.2842889	73.8195316	66.8813582	70.5172777
18:15:28	0:13:07	73.101237	66.2842889	73.8195316	66.8813582	71.5172777
18:15:29	0:13:08	73.101237	66.2842889	71.8195316	66.8813582	71.5172777
18:15:30	0:13:09	73.101237	65.2842889	71.8195316	66.8813582	71.5172777
18:15:31	0:13:10	74.101237	65.2842889	70.8195316	66.8813582	71.5172777
18:15:32	0:13:11	75.101237	65.2842889	70.8195316	65.8813582	70.5172777
18:15:33	0:13:12	78.101237	66.2842889	70.8195316	65.8813582	68.5172777
18:15:34	0:13:13	78.101237	66.2842889	70.8195316	65.8813582	68.5172777
18:15:35	0:13:14	78.101237	66.2842889	70.8195316	66.8813582	66.5172777
18:15:36	0:13:15	77.101237	66.2842889	70.8195316	66.8813582	65.5172777
18:15:37	0:13:16	77.101237	66.2842889	70.8195316	66.8813582	64.5172777
18:15:38	0:13:17	75.101237	67.2842889	71.8195316	66.8813582	64.5172777
18:15:39	0:13:18	72.101237	67.2842889	71.8195316	67.8813582	64.5172777
18:15:40	0:13:19	71.101237	67.2842889	71.8195316	67.8813582	67.5172777
18:15:41	0:13:20	71.101237	67.2842889	71.8195316	67.8813582	68.5172777
18:15:42	0:13:21	71.101237	67.2842889	72.8195316	67.8813582	69.5172777
18:15:43	0:13:22	70.101237	67.2842889	73.8195316	68.8813582	69.5172777
18:15:44	0:13:23	70.101237	67.2842889	74.8195316	68.8813582	70.5172777
18:15:45	0:13:24	70.101237	67.2842889	74.8195316	68.8813582	72.5172777
18:15:46	0:13:25	71.101237	65.2842889	75.8195316	68.8813582	73.5172777
18:15:47	0:13:26	72.101237	63.2842889	74.8195316	68.8813582	74.5172777
18:15:48	0:13:27	72.101237	62.2842889	74.8195316	68.8813582	74.5172777

18:15:49	0:13:28	72.101237	62.2842889	74.8195316	69.8813582	73.5172777
18:15:50	0:13:29	73.101237	61.2842889	74.8195316	69.8813582	73.5172777
18:15:51	0:13:30	73.101237	61.2842889	74.8195316	69.8813582	74.5172777
18:15:52	0:13:31	73.101237	62.2842889	75.8195316	69.8813582	75.5172777
18:15:53	0:13:32	73.101237	63.2842889	75.8195316	69.8813582	75.5172777
18:15:54	0:13:33	67.101237	64.2842889	75.8195316	69.8813582	76.5172777
18:15:55	0:13:34	68.101237	64.2842889	75.8195316	69.8813582	77.5172777
18:15:56	0:13:35	68.101237	64.2842889	74.8195316	69.8813582	77.5172777
18:15:57	0:13:36	69.101237	66.2842889	73.8195316	69.8813582	77.5172777
18:15:58	0:13:37	69.101237	67.2842889	73.8195316	69.8813582	75.5172777
18:15:59	0:13:38	69.101237	67.2842889	72.8195316	68.8813582	69.5172777
18:16:00	0:13:39	69.101237	68.2842889	73.8195316	68.8813582	67.5172777
18:16:01	0:13:40	69.101237	68.2842889	73.8195316	68.8813582	66.5172777
18:16:02	0:13:41	70.101237	68.2842889	73.8195316	67.8813582	66.5172777
18:16:03	0:13:42	73.101237	71.2842889	75.8195316	67.8813582	65.5172777
18:16:04	0:13:43	74.101237	71.2842889	74.8195316	67.8813582	66.5172777
18:16:05	0:13:44	73.101237	75.2842889	74.8195316	67.8813582	67.5172777
18:16:06	0:13:45	72.101237	75.2842889	74.8195316	67.8813582	68.5172777
18:16:07	0:13:46	70.101237	74.2842889	74.8195316	67.8813582	69.5172777
18:16:08	0:13:47	64.101237	74.2842889	74.8195316	67.8813582	70.5172777
18:16:09	0:13:48	64.101237	74.2842889	74.8195316	67.8813582	69.5172777
18:16:10	0:13:49	64.101237	74.2842889	75.8195316	67.8813582	68.5172777
18:16:11	0:13:50	64.101237	75.2842889	75.8195316	68.8813582	67.5172777
18:16:12	0:13:51	64.101237	75.2842889	75.8195316	68.8813582	67.5172777
18:16:13	0:13:52	64.101237	75.2842889	73.8195316	68.8813582	66.5172777
18:16:14	0:13:53	65.101237	76.2842889	73.8195316	68.8813582	65.5172777
18:16:15	0:13:54	65.101237	76.2842889	74.8195316	68.8813582	66.5172777
18:16:16	0:13:55	68.101237	77.2842889	76.8195316	68.8813582	66.5172777
18:16:17	0:13:56	70.101237	77.2842889	76.8195316	68.8813582	67.5172777
18:16:18	0:13:57	69.101237	77.2842889	76.8195316	68.8813582	67.5172777
18:16:19	0:13:58	68.101237	78.2842889	78.8195316	68.8813582	68.5172777
18:16:20	0:13:59	64.101237	78.2842889	80.8195316	67.8813582	68.5172777
18:16:21	0:14:00	63.101237	78.2842889	80.8195316	67.8813582	69.5172777
18:16:22	0:14:01	65.101237	78.2842889	82.8195316	67.8813582	69.5172777
18:16:23	0:14:02	65.101237	78.2842889	83.8195316	67.8813582	69.5172777
18:16:24	0:14:03	65.101237	80.2842889	83.8195316	67.8813582	69.5172777
18:16:25	0:14:04	67.101237	80.2842889	85.8195316	68.8813582	71.5172777
18:16:26	0:14:05	67.101237	80.2842889	86.8195316	68.8813582	71.5172777
18:16:27	0:14:06	67.101237	77.2842889	86.8195316	68.8813582	70.5172777
18:16:28	0:14:07	67.101237	76.2842889	86.8195316	67.8813582	69.5172777
18:16:29	0:14:08	67.101237	75.2842889	86.8195316	67.8813582	69.5172777
18:16:30	0:14:09	70.101237	75.2842889	86.8195316	66.8813582	67.5172777
18:16:31	0:14:10	70.101237	75.2842889	83.8195316	66.8813582	66.5172777
18:16:32	0:14:11	72.101237	75.2842889	83.8195316	66.8813582	67.5172777
18:16:33	0:14:12	72.101237	75.2842889	83.8195316	66.8813582	67.5172777

18:16:34	0:14:13	72.101237	75.2842889	81.8195316	66.8813582	68.5172777
18:16:35	0:14:14	72.101237	75.2842889	81.8195316	66.8813582	69.5172777
18:16:36	0:14:15	73.101237	75.2842889	75.8195316	66.8813582	70.5172777
18:16:37	0:14:16	76.101237	76.2842889	75.8195316	66.8813582	71.5172777
18:16:38	0:14:17	76.101237	76.2842889	74.8195316	66.8813582	71.5172777
18:16:39	0:14:18	74.101237	76.2842889	74.8195316	66.8813582	76.5172777
18:16:40	0:14:19	73.101237	76.2842889	72.8195316	66.8813582	77.5172777
18:16:41	0:14:20	74.101237	76.2842889	70.8195316	66.8813582	77.5172777
18:16:42	0:14:21	76.101237	77.2842889	70.8195316	66.8813582	77.5172777
18:16:43	0:14:22	76.101237	78.2842889	70.8195316	66.8813582	77.5172777
18:16:44	0:14:23	76.101237	79.2842889	70.8195316	66.8813582	77.5172777
18:16:45	0:14:24	76.101237	79.2842889	67.8195316	66.8813582	76.5172777
18:16:46	0:14:25	76.101237	78.2842889	66.8195316	67.8813582	75.5172777
18:16:47	0:14:26	76.101237	78.2842889	66.8195316	67.8813582	75.5172777
18:16:48	0:14:27	75.101237	76.2842889	66.8195316	67.8813582	73.5172777
18:16:49	0:14:28	74.101237	71.2842889	66.8195316	67.8813582	73.5172777
18:16:50	0:14:29	73.101237	69.2842889	67.8195316	67.8813582	72.5172777
18:16:51	0:14:30	74.101237	69.2842889	68.8195316	67.8813582	70.5172777
18:16:52	0:14:31	74.101237	68.2842889	68.8195316	67.8813582	69.5172777
18:16:53	0:14:32	73.101237	68.2842889	68.8195316	67.8813582	69.5172777
18:16:54	0:14:33	73.101237	68.2842889	69.8195316	67.8813582	68.5172777
18:16:55	0:14:34	73.101237	69.2842889	69.8195316	71.8813582	68.5172777
18:16:56	0:14:35	74.101237	70.2842889	69.8195316	72.8813582	68.5172777
18:16:57	0:14:36	75.101237	70.2842889	69.8195316	72.8813582	71.5172777
18:16:58	0:14:37	75.101237	69.2842889	70.8195316	71.8813582	71.5172777
18:16:59	0:14:38	76.101237	69.2842889	70.8195316	70.8813582	70.5172777
18:17:00	0:14:39	77.101237	68.2842889	71.8195316	69.8813582	70.5172777
18:17:01	0:14:40	77.101237	68.2842889	71.8195316	69.8813582	71.5172777
18:17:02	0:14:41	77.101237	68.2842889	71.8195316	68.8813582	72.5172777
18:17:03	0:14:42	77.101237	68.2842889	71.8195316	68.8813582	72.5172777
18:17:04	0:14:43	76.101237	68.2842889	71.8195316	68.8813582	72.5172777
18:17:05	0:14:44	76.101237	68.2842889	73.8195316	67.8813582	74.5172777
18:17:06	0:14:45	76.101237	68.2842889	73.8195316	67.8813582	74.5172777
18:17:07	0:14:46	74.101237	69.2842889	73.8195316	66.8813582	74.5172777
18:17:08	0:14:47	74.101237	69.2842889	76.8195316	66.8813582	75.5172777
18:17:09	0:14:48	75.101237	68.2842889	76.8195316	66.8813582	75.5172777
18:17:10	0:14:49	74.101237	68.2842889	76.8195316	67.8813582	75.5172777
18:17:11	0:14:50	73.101237	68.2842889	75.8195316	66.8813582	74.5172777
18:17:12	0:14:51	73.101237	68.2842889	73.8195316	67.8813582	70.5172777
18:17:13	0:14:52	73.101237	69.2842889	72.8195316	67.8813582	67.5172777
18:17:14	0:14:53	73.101237	70.2842889	72.8195316	67.8813582	65.5172777
18:17:15	0:14:54	73.101237	71.2842889	71.8195316	67.8813582	65.5172777
18:17:16	0:14:55	73.101237	71.2842889	71.8195316	67.8813582	65.5172777
18:17:17	0:14:56	73.101237	71.2842889	71.8195316	67.8813582	66.5172777
18:17:18	0:14:57	76.101237	71.2842889	71.8195316	67.8813582	68.5172777

18:17:19	0:14:58	76.101237	71.2842889	71.8195316	67.8813582	72.5172777
18:17:20	0:14:59	76.101237	72.2842889	71.8195316	68.8813582	73.5172777
18:17:21	0:15:00	75.101237	73.2842889	71.8195316	69.8813582	73.5172777
18:17:22	0:15:01	75.101237	71.2842889	71.8195316	69.8813582	73.5172777
18:17:23	0:15:02	75.101237	71.2842889	71.8195316	68.8813582	71.5172777
18:17:24	0:15:03	75.101237	72.2842889	71.8195316	67.8813582	70.5172777
18:17:25	0:15:04	76.101237	72.2842889	71.8195316	67.8813582	69.5172777
18:17:26	0:15:05	77.101237	72.2842889	71.8195316	67.8813582	69.5172777
18:17:27	0:15:06	78.101237	71.2842889	71.8195316	67.8813582	68.5172777
18:17:28	0:15:07	79.101237	71.2842889	72.8195316	67.8813582	68.5172777
18:17:29	0:15:08	79.101237	71.2842889	74.8195316	67.8813582	68.5172777
18:17:30	0:15:09	80.101237	71.2842889	73.8195316	67.8813582	69.5172777
18:17:31	0:15:10	80.101237	70.2842889	73.8195316	67.8813582	70.5172777
18:17:32	0:15:11	81.101237	70.2842889	73.8195316	67.8813582	70.5172777
18:17:33	0:15:12	81.101237	70.2842889	73.8195316	67.8813582	70.5172777
18:17:34	0:15:13	81.101237	69.2842889	73.8195316	67.8813582	70.5172777
18:17:35	0:15:14	81.101237	68.2842889	73.8195316	68.8813582	70.5172777
18:17:36	0:15:15	71.101237	68.2842889	73.8195316	68.8813582	69.5172777
18:17:37	0:15:16	63.101237	68.2842889	73.8195316	68.8813582	68.5172777
18:17:38	0:15:17	59.101237	68.2842889	73.8195316	68.8813582	68.5172777
18:17:39	0:15:18	58.101237	68.2842889	72.8195316	67.8813582	67.5172777
18:17:40	0:15:19	58.101237	68.2842889	72.8195316	67.8813582	68.5172777
18:17:41	0:15:20	58.101237	68.2842889	72.8195316	66.8813582	69.5172777
18:17:42	0:15:21	58.101237	68.2842889	73.8195316	66.8813582	69.5172777
18:17:43	0:15:22	57.101237	68.2842889	73.8195316	65.8813582	71.5172777
18:17:44	0:15:23	56.101237	68.2842889	73.8195316	64.8813582	72.5172777
18:17:45	0:15:24	57.101237	68.2842889	73.8195316	64.8813582	72.5172777
18:17:46	0:15:25	58.101237	68.2842889	73.8195316	64.8813582	72.5172777
18:17:47	0:15:26	60.101237	68.2842889	72.8195316	64.8813582	72.5172777
18:17:48	0:15:27	63.101237	68.2842889	71.8195316	63.8813582	73.5172777
18:17:49	0:15:28	65.101237	68.2842889	70.8195316	63.8813582	73.5172777
18:17:50	0:15:29	66.101237	68.2842889	70.8195316	64.8813582	70.5172777
18:17:51	0:15:30	66.101237	68.2842889	70.8195316	65.8813582	69.5172777
18:17:52	0:15:31	67.101237	68.2842889	70.8195316	65.8813582	67.5172777
18:17:53	0:15:32	67.101237	68.2842889	70.8195316	64.8813582	67.5172777
18:17:54	0:15:33	67.101237	68.2842889	70.8195316	64.8813582	67.5172777
18:17:55	0:15:34	68.101237	68.2842889	70.8195316	64.8813582	67.5172777
18:17:56	0:15:35	69.101237	68.2842889	72.8195316	65.8813582	67.5172777
18:17:57	0:15:36	69.101237	68.2842889	72.8195316	65.8813582	67.5172777
18:17:58	0:15:37	69.101237	68.2842889	73.8195316	65.8813582	67.5172777
18:17:59	0:15:38	70.101237	68.2842889	73.8195316	66.8813582	66.5172777
18:18:00	0:15:39	70.101237	68.2842889	73.8195316	66.8813582	66.5172777
18:18:01	0:15:40	73.101237	68.2842889	72.8195316	66.8813582	67.5172777
18:18:02	0:15:41	74.101237	68.2842889	72.8195316	65.8813582	67.5172777
18:18:03	0:15:42	74.101237	68.2842889	71.8195316	64.8813582	67.5172777

18:18:04	0:15:43	74.101237	68.2842889	73.8195316	64.8813582	67.5172777
18:18:05	0:15:44	74.101237	68.2842889	73.8195316	64.8813582	67.5172777
18:18:06	0:15:45	75.101237	67.2842889	73.8195316	65.8813582	69.5172777
18:18:07	0:15:46	75.101237	67.2842889	73.8195316	65.8813582	74.5172777
18:18:08	0:15:47	75.101237	67.2842889	72.8195316	65.8813582	75.5172777
18:18:09	0:15:48	73.101237	66.2842889	71.8195316	64.8813582	74.5172777
18:18:10	0:15:49	71.101237	66.2842889	71.8195316	65.8813582	73.5172777
18:18:11	0:15:50	71.101237	66.2842889	71.8195316	65.8813582	73.5172777
18:18:12	0:15:51	70.101237	66.2842889	71.8195316	65.8813582	72.5172777
18:18:13	0:15:52	70.101237	66.2842889	70.8195316	65.8813582	72.5172777
18:18:14	0:15:53	71.101237	66.2842889	70.8195316	65.8813582	72.5172777
18:18:15	0:15:54	70.101237	66.2842889	70.8195316	65.8813582	72.5172777
18:18:16	0:15:55	70.101237	66.2842889	72.8195316	65.8813582	74.5172777
18:18:17	0:15:56	69.101237	66.2842889	72.8195316	64.8813582	75.5172777
18:18:18	0:15:57	69.101237	66.2842889	74.8195316	63.8813582	75.5172777
18:18:19	0:15:58	69.101237	66.2842889	77.8195316	63.8813582	75.5172777
18:18:20	0:15:59	68.101237	66.2842889	77.8195316	64.8813582	71.5172777
18:18:21	0:16:00	68.101237	66.2842889	77.8195316	64.8813582	69.5172777
18:18:22	0:16:01	69.101237	66.2842889	76.8195316	65.8813582	69.5172777
18:18:23	0:16:02	70.101237	66.2842889	75.8195316	65.8813582	70.5172777
18:18:24	0:16:03	72.101237	66.2842889	73.8195316	65.8813582	73.5172777
18:18:25	0:16:04	73.101237	67.2842889	73.8195316	65.8813582	73.5172777
18:18:26	0:16:05	73.101237	67.2842889	73.8195316	65.8813582	81.5172777
18:18:27	0:16:06	73.101237	67.2842889	74.8195316	65.8813582	84.5172777
18:18:28	0:16:07	74.101237	67.2842889	76.8195316	67.8813582	84.5172777
18:18:29	0:16:08	74.101237	68.2842889	78.8195316	67.8813582	73.4872373
18:18:30	0:16:09	74.101237	70.2842889	78.8195316	67.8813582	73.5781464
18:18:31	0:16:10	74.101237	71.2842889	78.8195316	68.8813582	85.5172777
18:18:32	0:16:11	74.101237	70.2842889	76.8195316	68.8813582	84.5172777
18:18:33	0:16:12	74.101237	68.2842889	74.8195316	68.8813582	83.5172777
18:18:34	0:16:13	74.101237	67.2842889	72.8195316	68.8813582	83.5172777
18:18:35	0:16:14	74.101237	67.2842889	72.8195316	68.8813582	82.5172777
18:18:36	0:16:15	74.101237	67.2842889	72.8195316	68.8813582	82.5172777
18:18:37	0:16:16	74.101237	67.2842889	71.8195316	68.8813582	82.5172777
18:18:38	0:16:17	74.101237	67.2842889	71.8195316	68.8813582	75.5172777
18:18:39	0:16:18	74.101237	69.2842889	71.8195316	68.8813582	72.5172777
18:18:40	0:16:19	75.101237	69.2842889	70.8195316	68.8813582	71.5172777
18:18:41	0:16:20	75.101237	69.2842889	69.8195316	67.8813582	71.5172777
18:18:42	0:16:21	75.101237	69.2842889	69.8195316	66.8813582	70.5172777
18:18:43	0:16:22	74.101237	69.2842889	68.8195316	66.8813582	70.5172777
18:18:44	0:16:23	74.101237	69.2842889	68.8195316	66.8813582	70.5172777
18:18:45	0:16:24	72.101237	69.2842889	69.8195316	66.8813582	70.5172777
18:18:46	0:16:25	70.101237	69.2842889	69.8195316	66.8813582	70.5172777
18:18:47	0:16:26	68.101237	69.2842889	70.8195316	67.8813582	70.5172777
18:18:48	0:16:27	68.101237	69.2842889	70.8195316	67.8813582	69.5172777

18:18:49	0:16:28	67.101237	69.2842889	70.8195316	68.8813582	69.5172777
18:18:50	0:16:29	65.101237	68.2842889	70.8195316	68.8813582	70.5172777
18:18:51	0:16:30	65.101237	68.2842889	70.8195316	68.8813582	72.5172777
18:18:52	0:16:31	65.101237	68.2842889	70.8195316	68.8813582	73.5172777
18:18:53	0:16:32	64.101237	68.2842889	71.8195316	68.8813582	74.5172777
18:18:54	0:16:33	64.101237	68.2842889	71.8195316	68.8813582	74.5172777
18:18:55	0:16:34	63.101237	66.2842889	71.8195316	68.8813582	75.5172777
18:18:56	0:16:35	63.101237	65.2842889	71.8195316	68.8813582	76.5172777
18:18:57	0:16:36	63.101237	66.2842889	72.8195316	68.8813582	77.5172777
18:18:58	0:16:37	64.101237	66.2842889	72.8195316	68.8813582	77.5172777
18:18:59	0:16:38	64.101237	66.2842889	72.8195316	69.8813582	76.5172777
18:19:00	0:16:39	64.101237	66.2842889	71.8195316	69.8813582	75.5172777
18:19:01	0:16:40	64.101237	66.2842889	71.8195316	69.8813582	75.5172777
18:19:02	0:16:41	62.101237	67.2842889	71.8195316	69.8813582	74.5172777
18:19:03	0:16:42	58.101237	67.2842889	72.8195316	69.8813582	74.5172777
18:19:04	0:16:43	58.101237	67.2842889	73.8195316	70.8813582	74.5172777
18:19:05	0:16:44	58.101237	67.2842889	73.8195316	70.8813582	79.5172777
18:19:06	0:16:45	60.101237	68.2842889	73.8195316	71.8813582	81.5172777
18:19:07	0:16:46	61.101237	69.2842889	73.8195316	70.8813582	81.5172777
18:19:08	0:16:47	62.101237	69.2842889	73.8195316	70.8813582	80.5172777
18:19:09	0:16:48	62.101237	69.2842889	73.8195316	69.8813582	76.5172777
18:19:10	0:16:49	63.101237	69.2842889	73.8195316	69.8813582	73.5172777
18:19:11	0:16:50	64.101237	70.2842889	73.8195316	69.8813582	72.5172777
18:19:12	0:16:51	65.101237	70.2842889	73.8195316	66.8813582	72.5172777
18:19:13	0:16:52	65.101237	70.2842889	73.8195316	67.8813582	74.5172777
18:19:14	0:16:53	65.101237	70.2842889	72.8195316	67.8813582	74.5172777
18:19:15	0:16:54	66.101237	68.2842889	72.8195316	66.8813582	75.5172777
18:19:16	0:16:55	68.101237	68.2842889	72.8195316	65.8813582	75.5172777
18:19:17	0:16:56	70.101237	68.2842889	72.8195316	65.8813582	74.5172777
18:19:18	0:16:57	70.101237	68.2842889	72.8195316	65.8813582	73.5172777
18:19:19	0:16:58	70.101237	68.2842889	73.8195316	65.8813582	68.5172777
18:19:20	0:16:59	66.101237	68.2842889	74.8195316	65.8813582	66.5172777
18:19:21	0:17:00	65.101237	68.2842889	76.8195316	66.8813582	65.5172777
18:19:22	0:17:01	66.101237	68.2842889	78.8195316	66.8813582	64.5172777
18:19:23	0:17:02	66.101237	68.2842889	80.8195316	66.8813582	64.5172777
18:19:24	0:17:03	68.101237	68.2842889	82.8195316	66.8813582	65.5172777
18:19:25	0:17:04	69.101237	69.2842889	82.8195316	65.8813582	66.5172777
18:19:26	0:17:05	70.101237	70.2842889	79.8195316	65.8813582	66.5172777
18:19:27	0:17:06	70.101237	71.2842889	79.8195316	65.8813582	66.5172777
18:19:28	0:17:07	71.101237	71.2842889	77.8195316	65.8813582	69.5172777
18:19:29	0:17:08	73.101237	71.2842889	77.8195316	65.8813582	69.5172777
18:19:30	0:17:09	75.101237	71.2842889	76.8195316	65.8813582	69.5172777
18:19:31	0:17:10	75.101237	71.2842889	76.8195316	65.8813582	70.5172777
18:19:32	0:17:11	75.101237	71.2842889	70.8195316	65.8813582	70.5172777
18:19:33	0:17:12	72.101237	71.2842889	68.8195316	65.8813582	70.5172777

18:19:34	0:17:13	71.101237	71.2842889	68.8195316	65.8813582	69.5172777
18:19:35	0:17:14	70.101237	72.2842889	68.8195316	66.8813582	68.5172777
18:19:36	0:17:15	67.101237	72.2842889	70.8195316	67.8813582	67.5172777
18:19:37	0:17:16	66.101237	73.2842889	71.8195316	68.8813582	68.5172777
18:19:38	0:17:17	66.101237	72.2842889	71.8195316	69.8813582	68.5172777
18:19:39	0:17:18	66.101237	72.2842889	70.8195316	68.8813582	71.5172777
18:19:40	0:17:19	67.101237	70.2842889	69.8195316	67.8813582	72.5172777
18:19:41	0:17:20	67.101237	68.2842889	69.8195316	67.8813582	73.5172777
18:19:42	0:17:21	69.101237	67.2842889	68.8195316	66.8813582	68.5172777
18:19:43	0:17:22	69.101237	67.2842889	67.8195316	65.8813582	68.5172777
18:19:44	0:17:23	69.101237	67.2842889	67.8195316	65.8813582	67.5172777
18:19:45	0:17:24	69.101237	67.2842889	67.8195316	65.8813582	67.5172777
18:19:46	0:17:25	68.101237	67.2842889	68.8195316	65.8813582	68.5172777
18:19:47	0:17:26	68.101237	67.2842889	68.8195316	65.8813582	68.5172777
18:19:48	0:17:27	68.101237	67.2842889	68.8195316	65.8813582	68.5172777
18:19:49	0:17:28	66.101237	67.2842889	68.8195316	65.8813582	69.5172777
18:19:50	0:17:29	66.101237	67.2842889	69.8195316	65.8813582	69.5172777
18:19:51	0:17:30	63.101237	67.2842889	69.8195316	65.8813582	69.5172777
18:19:52	0:17:31	69.101237	67.2842889	69.8195316	66.8813582	69.5172777
18:19:53	0:17:32	69.101237	67.2842889	70.8195316	66.8813582	68.5172777
18:19:54	0:17:33	69.101237	67.2842889	70.8195316	66.8813582	68.5172777
18:19:55	0:17:34	70.101237	67.2842889	71.8195316	66.8813582	68.5172777
18:19:56	0:17:35	70.101237	67.2842889	71.8195316	67.8813582	68.5172777
18:19:57	0:17:36	70.101237	67.2842889	71.8195316	67.8813582	68.5172777
18:19:58	0:17:37	76.101237	66.2842889	71.8195316	67.8813582	68.5172777
18:19:59	0:17:38	76.101237	66.2842889	71.8195316	67.8813582	68.5172777
18:20:00	0:17:39	80.101237	66.2842889	72.8195316	68.8813582	68.5172777
18:20:01	0:17:40	80.101237	66.2842889	72.8195316	68.8813582	68.5172777
18:20:02	0:17:41	80.101237	67.2842889	71.8195316	67.8813582	68.5172777
18:20:03	0:17:42	81.101237	66.2842889	71.8195316	67.8813582	68.5172777
18:20:04	0:17:43	81.101237	67.2842889	71.8195316	67.8813582	68.5172777
18:20:05	0:17:44	81.101237	67.2842889	71.8195316	67.8813582	68.5172777
18:20:06	0:17:45	79.101237	67.2842889	71.8195316	68.8813582	68.5172777
18:20:07	0:17:46	79.101237	68.2842889	71.8195316	68.8813582	68.5172777
18:20:08	0:17:47	79.101237	68.2842889	72.8195316	69.8813582	69.5172777
18:20:09	0:17:48	79.101237	68.2842889	72.8195316	68.8813582	69.5172777
18:20:10	0:17:49	77.101237	68.2842889	72.8195316	67.8813582	69.5172777
18:20:11	0:17:50	77.101237	67.2842889	71.8195316	66.8813582	70.5172777
18:20:12	0:17:51	77.101237	68.2842889	71.8195316	66.8813582	70.5172777
18:20:13	0:17:52	77.101237	68.2842889	71.8195316	66.8813582	70.5172777
18:20:14	0:17:53	76.101237	68.2842889	71.8195316	66.8813582	71.5172777
18:20:15	0:17:54	76.101237	68.2842889	71.8195316	66.8813582	71.5172777
18:20:16	0:17:55	76.101237	67.2842889	71.8195316	66.8813582	71.5172777
18:20:17	0:17:56	75.101237	67.2842889	71.8195316	67.8813582	73.5172777
18:20:18	0:17:57	75.101237	67.2842889	72.8195316	67.8813582	73.5172777

18:20:19	0:17:58	74.101237	67.2842889	72.8195316	66.8813582	80.5172777
18:20:20	0:17:59	73.101237	67.2842889	73.8195316	67.8813582	80.5172777
18:20:21	0:18:00	72.101237	67.2842889	73.8195316	68.8813582	82.5172777
18:20:22	0:18:01	72.101237	67.2842889	73.8195316	68.8813582	85.5172777
18:20:23	0:18:02	73.101237	68.2842889	74.8195316	69.8813582	86.5172777
18:20:24	0:18:03	73.101237	67.2842889	74.8195316	69.8813582	86.5172777
18:20:25	0:18:04	73.101237	67.2842889	75.8195316	69.8813582	86.5172777
18:20:26	0:18:05	73.101237	67.2842889	75.8195316	69.8813582	85.5172777
18:20:27	0:18:06	73.101237	67.2842889	79.8195316	70.8813582	85.5172777
18:20:28	0:18:07	73.101237	67.2842889	80.8195316	70.8813582	81.5172777
18:20:29	0:18:08	73.101237	67.2842889	79.8195316	69.8813582	79.5172777
18:20:30	0:18:09	73.101237	67.2842889	79.8195316	69.8813582	79.5172777
18:20:31	0:18:10	72.101237	67.2842889	80.8195316	70.8813582	79.5172777
18:20:32	0:18:11	72.101237	67.2842889	80.8195316	69.8813582	76.5172777
18:20:33	0:18:12	73.101237	67.2842889	79.8195316	69.8813582	75.5172777
18:20:34	0:18:13	74.101237	68.2842889	77.8195316	68.8813582	75.5172777
18:20:35	0:18:14	74.101237	68.2842889	77.8195316	68.8813582	74.5172777
18:20:36	0:18:15	77.101237	68.2842889	73.8195316	68.8813582	74.5172777
18:20:37	0:18:16	78.101237	68.2842889	73.8195316	68.8813582	72.5172777
18:20:38	0:18:17	81.101237	70.2842889	73.8195316	68.8813582	72.5172777
18:20:39	0:18:18	84.101237	70.2842889	72.8195316	68.8813582	71.5172777
18:20:40	0:18:19	85.101237	70.2842889	72.8195316	68.8813582	71.5172777
18:20:41	0:18:20	85.101237	70.2842889	72.8195316	69.8813582	69.5172777
18:20:42	0:18:21	85.101237	70.2842889	72.8195316	69.8813582	69.5172777
18:20:43	0:18:22	85.101237	72.2842889	70.8195316	70.8813582	69.5172777
18:20:44	0:18:23	86.101237	71.2842889	69.8195316	70.8813582	69.5172777
18:20:45	0:18:24	86.101237	70.2842889	69.8195316	70.8813582	70.5172777
18:20:46	0:18:25	85.101237	70.2842889	69.8195316	70.8813582	70.5172777
18:20:47	0:18:26	85.101237	70.2842889	70.8195316	69.8813582	71.5172777
18:20:48	0:18:27	68.101237	70.2842889	70.8195316	69.8813582	72.5172777
18:20:49	0:18:28	64.101237	69.2842889	70.8195316	69.8813582	71.5172777
18:20:50	0:18:29	64.101237	69.2842889	70.8195316	69.8813582	72.5172777
18:20:51	0:18:30	61.101237	69.2842889	69.8195316	70.8813582	73.5172777
18:20:52	0:18:31	61.101237	69.2842889	69.8195316	70.8813582	73.5172777
18:20:53	0:18:32	62.101237	68.2842889	69.8195316	71.8813582	73.5172777
18:20:54	0:18:33	63.101237	68.2842889	69.8195316	71.8813582	73.5172777
18:20:55	0:18:34	63.101237	68.2842889	69.8195316	71.8813582	74.5172777
18:20:56	0:18:35	67.101237	68.2842889	69.8195316	72.8813582	75.5172777
18:20:57	0:18:36	70.101237	68.2842889	71.8195316	73.8813582	74.5172777
18:20:58	0:18:37	72.101237	68.2842889	73.8195316	74.8813582	74.5172777
18:20:59	0:18:38	72.101237	68.2842889	73.8195316	74.8813582	72.5172777
18:21:00	0:18:39	74.101237	68.2842889	73.8195316	74.8813582	72.5172777
18:21:01	0:18:40	75.101237	68.2842889	73.8195316	75.8813582	71.5172777
18:21:02	0:18:41	75.101237	68.2842889	73.8195316	74.8813582	71.5172777
18:21:03	0:18:42	76.101237	68.2842889	72.8195316	74.8813582	72.5172777

18:21:04	0:18:43	76.101237	69.2842889	72.8195316	74.8813582	72.5172777
18:21:05	0:18:44	75.101237	69.2842889	73.8195316	74.8813582	73.5172777
18:21:06	0:18:45	75.101237	70.2842889	79.8195316	74.8813582	74.5172777
18:21:07	0:18:46	71.101237	70.2842889	83.8195316	73.8813582	74.5172777
18:21:08	0:18:47	68.101237	70.2842889	83.8195316	73.8813582	74.5172777
18:21:09	0:18:48	68.101237	72.2842889	75.2145101	72.8813582	74.5172777
18:21:10	0:18:49	69.101237	72.2842889	75.3963282	72.8813582	74.5172777
18:21:11	0:18:50	71.101237	72.2842889	75.8508737	71.8813582	71.5172777
18:21:12	0:18:51	71.101237	72.2842889	75.6690555	71.8813582	68.5172777
18:21:13	0:18:52	72.101237	73.2842889	75.5781464	72.8813582	67.5172777
18:21:14	0:18:53	72.101237	73.2842889	75.8508737	72.8813582	67.5172777
18:21:15	0:18:54	72.101237	73.2842889	75.4872373	71.8813582	68.5172777
18:21:16	0:18:55	71.101237	73.2842889	75.5781464	71.8813582	69.5172777
18:21:17	0:18:56	72.101237	73.2842889	82.8195316	71.8813582	70.5172777
18:21:18	0:18:57	73.101237	73.2842889	79.8195316	71.8813582	70.5172777
18:21:19	0:18:58	74.101237	73.2842889	72.8195316	71.8813582	72.5172777
18:21:20	0:18:59	75.101237	73.2842889	69.8195316	71.8813582	72.5172777
18:21:21	0:19:00	74.101237	73.2842889	68.8195316	72.8813582	72.5172777
18:21:22	0:19:01	74.101237	72.2842889	68.8195316	75.8813582	72.5172777
18:21:23	0:19:02	74.101237	72.2842889	66.8195316	75.8813582	73.5172777
18:21:24	0:19:03	73.0258374	72.2842889	65.8195316	77.8813582	73.5172777
18:21:25	0:19:04	73.1258374	72.2842889	65.8195316	77.8813582	73.5172777
18:21:26	0:19:05	72.8258374	72.2842889	65.8195316	78.8813582	74.5172777
18:21:27	0:19:06	73.0258374	71.2842889	66.8195316	79.8813582	73.5172777
18:21:28	0:19:07	73.4258374	75.2842889	66.8195316	80.8813582	73.5172777
18:21:29	0:19:08	73.2258374	75.2842889	66.8195316	80.8813582	73.5172777
18:21:30	0:19:09	73.2258374	74.2842889	67.8195316	80.8813582	73.5172777
18:21:31	0:19:10	73.4258374	74.2842889	68.8195316	80.8813582	73.5172777
18:21:32	0:19:11	73.9258374	74.2842889	69.8195316	80.8813582	75.5172777
18:21:33	0:19:12	74.3258374	74.2842889	69.8195316	80.8813582	76.5172777
18:21:34	0:19:13	74.2258374	74.2842889	69.8195316	80.8813582	76.5172777
18:21:35	0:19:14	74.6258374	74.2842889	70.8195316	81.8813582	75.5172777
18:21:36	0:19:15	74.6258374	74.2842889	70.8195316	81.8813582	75.5172777
18:21:37	0:19:16	74.3258374	73.2842889	70.8195316	82.8813582	75.5172777
18:21:38	0:19:17	73.4258374	71.2842889	70.8195316	82.8813582	68.5172777
18:21:39	0:19:18	72.9258374	70.2842889	70.8195316	82.8813582	66.5172777
18:21:40	0:19:19	73.0258374	70.2842889	70.8195316	82.8813582	67.5172777
18:21:41	0:19:20	73.0258374	70.2842889	70.8195316	82.8813582	68.5172777
18:21:42	0:19:21	73.1258374	70.2842889	70.8195316	82.8813582	68.5172777
18:21:43	0:19:22	73.0258374	70.2842889	69.8195316	81.8813582	69.5172777
18:21:44	0:19:23	73.2258374	70.2842889	70.8195316	79.8813582	69.5172777
18:21:45	0:19:24	73.1258374	71.2842889	70.8195316	78.8813582	69.5172777
18:21:46	0:19:25	72.7258374	71.2842889	70.8195316	76.8813582	69.5172777
18:21:47	0:19:26	72.8258374	74.2842889	72.8195316	74.8813582	69.5172777
18:21:48	0:19:27	72.6258374	74.2842889	71.8195316	73.8813582	69.5172777

18:21:49	0:19:28	73.2258374	72.2842889	71.8195316	72.8813582	69.5172777
18:21:50	0:19:29	73.3258374	69.2842889	71.8195316	72.8813582	69.5172777
18:21:51	0:19:30	73.2258374	68.2842889	72.8195316	71.8813582	69.5172777
18:21:52	0:19:31	73.5258374	68.2842889	73.8195316	71.8813582	69.5172777
18:21:53	0:19:32	74.6258374	68.2842889	73.8195316	71.8813582	70.5172777
18:21:54	0:19:33	74.7258374	68.2842889	73.8195316	71.8813582	71.5172777
18:21:55	0:19:34	74.3258374	68.2842889	75.8195316	72.8813582	72.5172777
18:21:56	0:19:35	74.4258374	69.2842889	76.8195316	73.8813582	72.5172777
18:21:57	0:19:36	73.8258374	69.2842889	76.8195316	74.8813582	72.5172777
18:21:58	0:19:37	73.6258374	69.2842889	83.8195316	74.8813582	72.5172777
18:21:59	0:19:38	73.6258374	69.2842889	85.8195316	74.8813582	72.5172777
18:22:00	0:19:39	73.7258374	69.2842889	73.7258374	74.8813582	72.5172777
18:22:01	0:19:40	74.0258374	69.2842889	74.0258374	74.8813582	71.5172777
18:22:02	0:19:41	74.0258374	69.2842889	74.0258374	74.8813582	72.5172777
18:22:03	0:19:42	74.2258374	69.2842889	74.2258374	75.8813582	72.5172777
18:22:04	0:19:43	74.1258374	69.2842889	74.1258374	73.8813582	72.5172777
18:22:05	0:19:44	74.0258374	70.2842889	74.0258374	73.8813582	73.5172777
18:22:06	0:19:45	73.8258374	70.2842889	73.8258374	72.8813582	74.5172777
18:22:07	0:19:46	73.1258374	70.2842889	73.1258374	72.8813582	74.5172777
18:22:08	0:19:47	72.9258374	70.2842889	72.9258374	72.8813582	74.5172777
18:22:09	0:19:48	72.2258374	70.2842889	84.8195316	72.8813582	76.5172777
18:22:10	0:19:49	71.4258374	70.2842889	80.8195316	71.8813582	77.5172777
18:22:11	0:19:50	71.4258374	70.2842889	78.8195316	71.8813582	79.5172777
18:22:12	0:19:51	71.4258374	70.2842889	77.8195316	71.8813582	81.5172777
18:22:13	0:19:52	71.3258374	70.2842889	77.8195316	70.8813582	81.5172777
18:22:14	0:19:53	71.7258374	69.2842889	78.8195316	70.8813582	81.5172777
18:22:15	0:19:54	71.6258374	69.2842889	77.8195316	70.8813582	80.5172777
18:22:16	0:19:55	72.6258374	69.2842889	77.8195316	73.8813582	77.5172777
18:22:17	0:19:56	73.2258374	72.2842889	77.8195316	76.8813582	77.5172777
18:22:18	0:19:57	73.4258374	72.2842889	76.8195316	76.8813582	78.5172777
18:22:19	0:19:58	73.7258374	75.2842889	75.8195316	77.8813582	77.5172777
18:22:20	0:19:59	74.0258374	75.2842889	75.8195316	77.8813582	76.5172777
18:22:21	0:20:00	74.2258374	75.2842889	71.8195316	77.8813582	76.5172777
18:22:22	0:20:01	74.1258374	75.2842889	71.8195316	77.8813582	74.5172777
18:22:23	0:20:02	74.3258374	75.2842889	71.8195316	79.8813582	73.5172777
18:22:24	0:20:03	74.3258374	75.2842889	70.8195316	80.8813582	72.5172777
18:22:25	0:20:04	74.1258374	75.2842889	69.8195316	77.8813582	73.5172777
18:22:26	0:20:05	74.8258374	75.2842889	69.8195316	77.8813582	75.5172777
18:22:27	0:20:06	74.9258374	75.2842889	69.8195316	77.8813582	77.5172777
18:22:28	0:20:07	75.0258374	75.2842889	70.8195316	77.8813582	77.5172777
18:22:29	0:20:08	75.0258374	75.2842889	70.8195316	76.8813582	76.5172777
18:22:30	0:20:09	75.0258374	75.2842889	70.8195316	76.8813582	76.5172777
18:22:31	0:20:10	75.1258374	75.2842889	72.8195316	74.8813582	74.5172777
18:22:32	0:20:11	75.3258374	74.2842889	73.8195316	73.8813582	74.5172777
18:22:33	0:20:12	75.1258374	73.2842889	73.8195316	73.8813582	73.5172777

18:22:34	0:20:13	74.7258374	73.2842889	73.8195316	73.8813582	72.5172777
18:22:35	0:20:14	74.4258374	73.2842889	74.8195316	72.8813582	72.5172777
18:22:36	0:20:15	74.3258374	72.2842889	75.8195316	73.8813582	71.5172777
18:22:37	0:20:16	74.9258374	72.2842889	75.8195316	73.8813582	72.5172777
18:22:38	0:20:17	74.5258374	73.2842889	74.8195316	71.8813582	71.5172777
18:22:39	0:20:18	75.2258374	72.2842889	73.8195316	71.8813582	71.5172777
18:22:40	0:20:19	75.7258374	72.2842889	73.8195316	71.8813582	71.5172777
18:22:41	0:20:20	75.6258374	72.2842889	75.8195316	71.8813582	70.5172777
18:22:42	0:20:21	76.0258374	72.2842889	78.8195316	71.8813582	71.5172777
18:22:43	0:20:22	76.5258374	72.2842889	81.8195316	71.8813582	71.5172777
18:22:44	0:20:23	76.4258374	72.2842889	81.8195316	70.8813582	73.5172777
18:22:45	0:20:24	76.9258374	72.2842889	81.8195316	69.8813582	75.5172777
18:22:46	0:20:25	77.1258374	71.2842889	80.8195316	69.8813582	78.5172777
18:22:47	0:20:26	76.8258374	71.2842889	79.8195316	69.8813582	78.5172777
18:22:48	0:20:27	76.4258374	70.2842889	79.8195316	69.8813582	79.5172777
18:22:49	0:20:28	76.6258374	69.2842889	83.8195316	68.8813582	78.5172777
18:22:50	0:20:29	76.6258374	68.2842889	86.8195316	68.8813582	77.5172777
18:22:51	0:20:30	76.0258374	66.2842889	88.8195316	69.8813582	77.5172777
18:22:52	0:20:31	75.7258374	65.2842889	75.7258374	70.8813582	77.5172777
18:22:53	0:20:32	76.0258374	65.2842889	88.8195316	70.8813582	77.5172777
18:22:54	0:20:33	76.3258374	66.2842889	76.3258374	69.8813582	77.5172777
18:22:55	0:20:34	76.3258374	66.2842889	76.3258374	69.8813582	77.5172777
18:22:56	0:20:35	76.1258374	69.2842889	76.1258374	69.8813582	74.5172777
18:22:57	0:20:36	75.9258374	69.2842889	75.9258374	69.8813582	73.5172777
18:22:58	0:20:37	74.5258374	70.2842889	74.5258374	69.8813582	72.5172777
18:22:59	0:20:38	73.3258374	71.2842889	73.3258374	69.8813582	73.5172777
18:23:00	0:20:39	73.1258374	72.2842889	73.1258374	70.8813582	75.5172777
18:23:01	0:20:40	73.0258374	72.2842889	73.0258374	72.8813582	77.5172777
18:23:02	0:20:41	73.0258374	74.2842889	73.0258374	71.8813582	77.5172777
18:23:03	0:20:42	72.9258374	74.2842889	72.9258374	69.8813582	76.5172777
18:23:04	0:20:43	72.7258374	73.2842889	72.7258374	69.8813582	75.5172777
18:23:05	0:20:44	72.4258374	73.2842889	80.8195316	69.8813582	75.5172777
18:23:06	0:20:45	71.9258374	73.2842889	79.8195316	69.8813582	70.5172777
18:23:07	0:20:46	72.6258374	73.2842889	79.8195316	72.8813582	69.5172777
18:23:08	0:20:47	72.8258374	73.2842889	79.8195316	72.8813582	68.5172777
18:23:09	0:20:48	73.3258374	74.2842889	77.8195316	73.8813582	68.5172777
18:23:10	0:20:49	73.5258374	74.2842889	77.8195316	73.8813582	69.5172777
18:23:11	0:20:50	73.6258374	74.2842889	76.8195316	73.8813582	72.5172777
18:23:12	0:20:51	73.8258374	74.2842889	75.8195316	73.8813582	74.5172777
18:23:13	0:20:52	74.0258374	74.2842889	75.8195316	74.8813582	74.5172777
18:23:14	0:20:53	75.1258374	74.2842889	79.8195316	75.8813582	75.5172777
18:23:15	0:20:54	75.2258374	74.2842889	79.8195316	75.8813582	76.5172777
18:23:16	0:20:55	75.1258374	74.2842889	79.8195316	75.8813582	76.5172777
18:23:17	0:20:56	74.1258374	73.2842889	75.8195316	75.8813582	75.5172777
18:23:18	0:20:57	73.6258374	73.2842889	73.8195316	76.8813582	75.5172777

18:23:19	0:20:58	73.8258374	74.2842889	72.8195316	76.8813582	75.5172777
18:23:20	0:20:59	73.9258374	74.2842889	71.8195316	76.8813582	76.5172777
18:23:21	0:21:00	74.3258374	76.2842889	71.8195316	76.8813582	77.5172777
18:23:22	0:21:01	74.5258374	76.2842889	71.8195316	76.8813582	78.5172777
18:23:23	0:21:02	75.0258374	76.2842889	73.8195316	77.8813582	79.5172777
18:23:24	0:21:03	75.0258374	76.2842889	73.8195316	77.8813582	79.5172777
18:23:25	0:21:04	74.4258374	69.2842889	73.8195316	76.8813582	79.5172777
18:23:26	0:21:05	74.7258374	68.2842889	77.8195316	74.8813582	78.5172777
18:23:27	0:21:06	75.3258374	67.2842889	79.8195316	74.8813582	78.5172777
18:23:28	0:21:07	74.9258374	67.2842889	81.8195316	73.8813582	72.5172777
18:23:29	0:21:08	74.8258374	67.2842889	81.8195316	73.8813582	71.5172777
18:23:30	0:21:09	74.5258374	67.2842889	82.8195316	73.8813582	69.5172777
18:23:31	0:21:10	74.2258374	67.2842889	81.8195316	73.8813582	69.5172777
18:23:32	0:21:11	74.4258374	66.2842889	84.8195316	73.8813582	70.5172777
18:23:33	0:21:12	74.5258374	66.2842889	86.8195316	73.8813582	70.5172777
18:23:34	0:21:13	74.7258374	66.2842889	86.8195316	73.8813582	72.5172777
18:23:35	0:21:14	74.9258374	65.2842889	74.9258374	72.8813582	72.5172777
18:23:36	0:21:15	75.3258374	65.2842889	75.3258374	72.8813582	76.5172777
18:23:37	0:21:16	74.6258374	64.2842889	74.6258374	71.8813582	76.5172777
18:23:38	0:21:17	73.9258374	64.2842889	73.9258374	70.8813582	75.5172777
18:23:39	0:21:18	73.5258374	64.2842889	73.5258374	69.8813582	74.5172777
18:23:40	0:21:19	73.3258374	64.2842889	73.3258374	69.8813582	74.5172777
18:23:41	0:21:20	72.3258374	65.2842889	72.3258374	68.8813582	69.5172777
18:23:42	0:21:21	72.1258374	65.2842889	72.1258374	68.8813582	67.5172777
18:23:43	0:21:22	72.0258374	65.2842889	72.0258374	67.8813582	67.5172777
18:23:44	0:21:23	72.1258374	65.2842889	72.1258374	68.8813582	67.5172777
18:23:45	0:21:24	72.5258374	67.2842889	72.5258374	70.8813582	69.5172777
18:23:46	0:21:25	72.4258374	66.2842889	81.8195316	72.8813582	71.5172777
18:23:47	0:21:26	72.1258374	66.2842889	76.8195316	72.8813582	71.5172777
18:23:48	0:21:27	71.9258374	66.2842889	73.8195316	72.8813582	70.5172777
18:23:49	0:21:28	71.2258374	66.2842889	69.8195316	70.8813582	69.5172777
18:23:50	0:21:29	71.4258374	66.2842889	68.8195316	69.8813582	68.5172777
18:23:51	0:21:30	71.7258374	66.2842889	68.8195316	69.8813582	68.5172777
18:23:52	0:21:31	71.9258374	66.2842889	67.8195316	69.8813582	68.5172777
18:23:53	0:21:32	71.8258374	66.2842889	67.8195316	68.8813582	69.5172777
18:23:54	0:21:33	72.1258374	66.2842889	67.8195316	68.8813582	71.5172777
18:23:55	0:21:34	74.7258374	66.2842889	67.8195316	68.8813582	73.5172777
18:23:56	0:21:35	74.9258374	66.2842889	67.8195316	69.8813582	73.5172777
18:23:57	0:21:36	75.5258374	65.2842889	67.8195316	70.8813582	72.5172777
18:23:58	0:21:37	75.4258374	64.2842889	67.8195316	71.8813582	69.5172777
18:23:59	0:21:38	75.3258374	64.2842889	67.8195316	70.8813582	69.5172777
18:24:00	0:21:39	75.3258374	65.2842889	67.8195316	70.8813582	69.5172777
18:24:01	0:21:40	75.1258374	65.2842889	67.8195316	71.8813582	69.5172777
18:24:02	0:21:41	75.3258374	65.2842889	68.8195316	72.8813582	70.5172777
18:24:03	0:21:42	73.4258374	66.2842889	69.8195316	72.8813582	71.5172777

18:24:04	0:21:43	74.0258374	66.2842889	69.8195316	73.8813582	73.5172777
18:24:05	0:21:44	74.0258374	66.2842889	72.8195316	73.8813582	73.5172777
18:24:06	0:21:45	73.9258374	66.2842889	75.8195316	73.8813582	77.5172777
18:24:07	0:21:46	73.9258374	66.2842889	75.8195316	73.8813582	80.5172777
18:24:08	0:21:47	74.1258374	67.2842889	74.8195316	73.8813582	81.5172777
18:24:09	0:21:48	73.5258374	67.2842889	74.8195316	73.8813582	82.5172777
18:24:10	0:21:49	73.5258374	67.2842889	73.8195316	73.8813582	82.5172777
18:24:11	0:21:50	73.2258374	67.2842889	71.8195316	73.8813582	81.5172777
18:24:12	0:21:51	73.7258374	67.2842889	71.8195316	73.8813582	81.5172777
18:24:13	0:21:52	73.2258374	65.2842889	70.8195316	73.8813582	80.5172777
18:24:14	0:21:53	73.3258374	64.2842889	70.8195316	73.8813582	79.5172777
18:24:15	0:21:54	73.5258374	64.2842889	74.8195316	73.8813582	78.5172777
18:24:16	0:21:55	73.4258374	64.2842889	74.8195316	73.8813582	76.5172777
18:24:17	0:21:56	73.9258374	64.2842889	78.8195316	73.8813582	75.5172777
18:24:18	0:21:57	73.9258374	64.2842889	78.8195316	73.8813582	75.5172777
18:24:19	0:21:58	74.4258374	65.2842889	82.8195316	73.8813582	76.5172777
18:24:20	0:21:59	75.4258374	65.2842889	86.8195316	73.8813582	78.5172777
18:24:21	0:22:00	75.4258374	65.2842889	86.8195316	73.8813582	79.5172777
18:24:22	0:22:01	75.6258374	65.2842889	75.6258374	73.8813582	79.5172777
18:24:23	0:22:02	76.1258374	65.2842889	76.1258374	73.8813582	77.5172777
18:24:24	0:22:03	75.9258374	65.2842889	75.9258374	73.8813582	76.5172777
18:24:25	0:22:04	76.1258374	65.2842889	76.1258374	73.8813582	76.5172777
18:24:26	0:22:05	76.3258374	65.2842889	76.3258374	73.8813582	77.5172777
18:24:27	0:22:06	76.1258374	67.2842889	84.8195316	72.8813582	78.5172777
18:24:28	0:22:07	75.8258374	67.2842889	79.8195316	72.8813582	78.5172777
18:24:29	0:22:08	75.4258374	66.2842889	76.8195316	72.8813582	76.5172777
18:24:30	0:22:09	75.4258374	66.2842889	77.8195316	71.8813582	74.5172777
18:24:31	0:22:10	75.1258374	66.2842889	76.8195316	71.8813582	74.5172777
18:24:32	0:22:11	74.9258374	66.2842889	75.8195316	70.8813582	75.5172777
18:24:33	0:22:12	74.2258374	65.2842889	73.8195316	70.8813582	75.5172777
18:24:34	0:22:13	74.0258374	65.2842889	72.8195316	70.8813582	76.5172777
18:24:35	0:22:14	73.6258374	65.2842889	72.8195316	69.8813582	77.5172777
18:24:36	0:22:15	73.5258374	65.2842889	72.8195316	69.8813582	77.5172777
18:24:37	0:22:16	73.9258374	66.2842889	72.8195316	69.8813582	76.5172777
18:24:38	0:22:17	73.7258374	66.2842889	71.8195316	68.8813582	75.5172777
18:24:39	0:22:18	74.1258374	66.2842889	70.8195316	68.8813582	75.5172777
18:24:40	0:22:19	74.1258374	67.2842889	69.8195316	68.8813582	73.5172777
18:24:41	0:22:20	74.0258374	67.2842889	69.8195316	67.8813582	72.5172777
18:24:42	0:22:21	73.6258374	66.2842889	69.8195316	67.8813582	70.5172777
18:24:43	0:22:22	73.5258374	66.2842889	69.8195316	66.8813582	70.5172777
18:24:44	0:22:23	73.5258374	66.2842889	69.8195316	66.8813582	70.5172777
18:24:45	0:22:24	73.5258374	65.2842889	70.8195316	66.8813582	70.5172777
18:24:46	0:22:25	73.5258374	65.2842889	70.8195316	66.8813582	70.5172777
18:24:47	0:22:26	73.3258374	65.2842889	70.8195316	65.8813582	70.5172777
18:24:48	0:22:27	73.0258374	65.2842889	70.8195316	65.8813582	71.5172777

18:24:49	0:22:28	72.5258374	65.2842889	70.8195316	65.8813582	70.5172777
18:24:50	0:22:29	72.6258374	65.2842889	71.8195316	66.8813582	71.5172777
18:24:51	0:22:30	73.3258374	65.2842889	72.8195316	66.8813582	73.5172777
18:24:52	0:22:31	72.9258374	67.2842889	71.8195316	66.8813582	73.5172777
18:24:53	0:22:32	72.6258374	68.2842889	71.8195316	66.8813582	73.5172777
18:24:54	0:22:33	72.3258374	68.2842889	71.8195316	66.8813582	73.5172777
18:24:55	0:22:34	72.4258374	68.2842889	72.8195316	67.8813582	72.5172777
18:24:56	0:22:35	72.6258374	68.2842889	74.8195316	67.8813582	72.5172777
18:24:57	0:22:36	72.8258374	68.2842889	74.8195316	67.8813582	72.5172777
18:24:58	0:22:37	72.9258374	67.2842889	73.8195316	67.8813582	74.5172777
18:24:59	0:22:38	73.0258374	67.2842889	72.8195316	67.8813582	75.5172777
18:25:00	0:22:39	73.2258374	67.2842889	72.8195316	68.8813582	74.5172777
18:25:01	0:22:40	72.5258374	67.2842889	71.8195316	69.8813582	73.5172777
18:25:02	0:22:41	72.1258374	67.2842889	70.8195316	70.8813582	72.5172777
18:25:03	0:22:42	72.4258374	67.2842889	70.8195316	70.8813582	72.5172777
18:25:04	0:22:43	72.3258374	67.2842889	70.8195316	70.8813582	71.5172777
18:25:05	0:22:44	73.3258374	67.2842889	71.8195316	70.8813582	71.5172777
18:25:06	0:22:45	73.7258374	67.2842889	71.8195316	74.8813582	71.5172777
18:25:07	0:22:46	73.5258374	67.2842889	72.8195316	74.8813582	70.5172777
18:25:08	0:22:47	73.6258374	70.2842889	72.8195316	74.8813582	70.5172777
18:25:09	0:22:48	73.5258374	70.2842889	72.8195316	75.8813582	70.5172777
18:25:10	0:22:49	73.1258374	70.2842889	73.8195316	75.8813582	70.5172777
18:25:11	0:22:50	73.4258374	70.2842889	75.8195316	75.8813582	71.5172777
18:25:12	0:22:51	73.4258374	70.2842889	75.8195316	75.8813582	71.5172777
18:25:13	0:22:52	74.5258374	70.2842889	74.5258374	75.8813582	73.5172777
18:25:14	0:22:53	74.6258374	70.2842889	74.6258374	76.8813582	75.5172777
18:25:15	0:22:54	75.1258374	70.2842889	75.1258374	76.8813582	75.5172777
18:25:16	0:22:55	75.1258374	70.2842889	75.1258374	73.8813582	76.5172777
18:25:17	0:22:56	75.5258374	70.2842889	75.5258374	73.8813582	77.5172777
18:25:18	0:22:57	75.1258374	70.2842889	75.1258374	73.8813582	77.5172777
18:25:19	0:22:58	74.4258374	71.2842889	84.8195316	74.8813582	77.5172777
18:25:20	0:22:59	74.6258374	74.2842889	81.8195316	74.8813582	78.5172777
18:25:21	0:23:00	74.8258374	75.2842889	80.8195316	75.8813582	78.5172777
18:25:22	0:23:01	74.6258374	74.2842889	79.8195316	77.8813582	78.5172777
18:25:23	0:23:02	74.5258374	73.2842889	78.8195316	77.8813582	80.5172777
18:25:24	0:23:03	74.6258374	73.2842889	77.8195316	77.8813582	80.5172777
18:25:25	0:23:04	74.2258374	73.2842889	75.8195316	75.8813582	79.5172777
18:25:26	0:23:05	73.8258374	73.2842889	73.8195316	74.8813582	79.5172777
18:25:27	0:23:06	73.4258374	73.2842889	72.8195316	72.8813582	80.5172777
18:25:28	0:23:07	73.1258374	73.2842889	70.8195316	72.8813582	81.5172777
18:25:29	0:23:08	72.6258374	73.2842889	69.8195316	71.8813582	82.5172777
18:25:30	0:23:09	72.4258374	72.2842889	69.8195316	71.8813582	82.5172777
18:25:31	0:23:10	71.9258374	72.2842889	68.8195316	70.8813582	83.5172777
18:25:32	0:23:11	72.4258374	72.2842889	68.8195316	70.8813582	84.5172777
18:25:33	0:23:12	72.1258374	72.2842889	68.8195316	70.8813582	84.5172777

18:25:34	0:23:13	71.5258374	71.2842889	68.8195316	70.8813582	82.5172777
18:25:35	0:23:14	71.1258374	68.2842889	68.8195316	70.8813582	81.5172777
18:25:36	0:23:15	70.9258374	67.2842889	69.8195316	70.8813582	80.5172777
18:25:37	0:23:16	70.9258374	67.2842889	70.8195316	70.8813582	78.5172777
18:25:38	0:23:17	70.9258374	66.2842889	73.8195316	70.8813582	78.5172777
18:25:39	0:23:18	71.0258374	66.2842889	74.8195316	70.8813582	78.5172777
18:25:40	0:23:19	71.3258374	66.2842889	76.8195316	70.8813582	78.5172777
18:25:41	0:23:20	71.9258374	67.2842889	79.8195316	70.8813582	78.5172777
18:25:42	0:23:21	71.9258374	67.2842889	79.8195316	70.8813582	78.5172777
18:25:43	0:23:22	72.0258374	67.2842889	80.8195316	69.8813582	77.5172777
18:25:44	0:23:23	71.6258374	67.2842889	77.8195316	69.8813582	76.5172777
18:25:45	0:23:24	71.6258374	68.2842889	76.8195316	69.8813582	74.5172777
18:25:46	0:23:25	71.7258374	69.2842889	75.8195316	69.8813582	73.5172777
18:25:47	0:23:26	71.9258374	69.2842889	75.8195316	69.8813582	73.5172777
18:25:48	0:23:27	72.3258374	73.2842889	74.8195316	69.8813582	74.5172777
18:25:49	0:23:28	72.1258374	73.2842889	73.8195316	69.8813582	74.5172777
18:25:50	0:23:29	72.3258374	73.2842889	73.8195316	68.8813582	73.5172777
18:25:51	0:23:30	71.9258374	73.2842889	72.8195316	68.8813582	72.5172777
18:25:52	0:23:31	71.5258374	73.2842889	72.8195316	68.8813582	70.5172777
18:25:53	0:23:32	71.6258374	73.2842889	72.8195316	69.8813582	69.5172777
18:25:54	0:23:33	71.4258374	73.2842889	71.8195316	69.8813582	69.5172777
18:25:55	0:23:34	70.9258374	73.2842889	70.8195316	69.8813582	69.5172777
18:25:56	0:23:35	70.3258374	73.2842889	71.8195316	69.8813582	70.5172777
18:25:57	0:23:36	70.8258374	73.2842889	73.8195316	70.8813582	72.5172777
18:25:58	0:23:37	70.0258374	62.2842889	73.8195316	70.8813582	73.5172777
18:25:59	0:23:38	70.1258374	62.2842889	75.8195316	70.8813582	74.5172777
18:26:00	0:23:39	70.7258374	62.2842889	77.8195316	71.8813582	75.5172777
18:26:01	0:23:40	71.8258374	62.2842889	71.8258374	73.8813582	75.5172777
18:26:02	0:23:41	72.1258374	62.2842889	72.1258374	73.8813582	73.5172777
18:26:03	0:23:42	72.0258374	62.2842889	72.0258374	73.8813582	70.5172777
18:26:04	0:23:43	71.7258374	62.2842889	71.7258374	70.8813582	69.5172777
18:26:05	0:23:44	71.7258374	62.2842889	71.7258374	68.8813582	68.5172777
18:26:06	0:23:45	71.7258374	62.2842889	71.7258374	68.8813582	68.5172777
18:26:07	0:23:46	71.9258374	67.2842889	71.9258374	68.8813582	67.5172777
18:26:08	0:23:47	71.4258374	67.2842889	81.8195316	67.8813582	67.5172777
18:26:09	0:23:48	71.8258374	67.2842889	76.8195316	67.8813582	70.5172777
18:26:10	0:23:49	72.4258374	67.2842889	74.8195316	67.8813582	70.5172777
18:26:11	0:23:50	72.4258374	67.2842889	73.8195316	67.8813582	70.5172777
18:26:12	0:23:51	72.5258374	67.2842889	73.8195316	67.8813582	70.5172777
18:26:13	0:23:52	72.6258374	67.2842889	72.8195316	67.8813582	67.5172777
18:26:14	0:23:53	72.0258374	67.2842889	70.8195316	67.8813582	65.5172777
18:26:15	0:23:54	71.3258374	67.2842889	68.8195316	67.8813582	65.5172777
18:26:16	0:23:55	71.2258374	68.2842889	67.8195316	69.8813582	64.5172777
18:26:17	0:23:56	71.0258374	68.2842889	67.8195316	70.8813582	63.5172777
18:26:18	0:23:57	71.0258374	68.2842889	66.8195316	71.8813582	63.5172777

18:26:19	0:23:58	70.2258374	68.2842889	66.8195316	69.8813582	63.5172777
18:26:20	0:23:59	69.9258374	68.2842889	66.8195316	68.8813582	64.5172777
18:26:21	0:24:00	70.3258374	69.2842889	67.8195316	68.8813582	65.5172777
18:26:22	0:24:01	70.7258374	70.2842889	69.8195316	68.8813582	65.5172777
18:26:23	0:24:02	70.9258374	70.2842889	69.8195316	67.8813582	65.5172777
18:26:24	0:24:03	71.2258374	70.2842889	72.8195316	67.8813582	66.5172777
18:26:25	0:24:04	71.2258374	69.2842889	73.8195316	67.8813582	65.5172777
18:26:26	0:24:05	70.8258374	69.2842889	73.8195316	67.8813582	65.5172777
18:26:27	0:24:06	70.3258374	69.2842889	72.8195316	67.8813582	65.5172777
18:26:28	0:24:07	70.1258374	69.2842889	72.8195316	67.8813582	64.5172777
18:26:29	0:24:08	70.1258374	70.2842889	70.8195316	67.8813582	64.5172777
18:26:30	0:24:09	70.0258374	70.2842889	70.8195316	67.8813582	64.5172777
18:26:31	0:24:10	69.7258374	68.2842889	70.8195316	67.8813582	64.5172777
18:26:32	0:24:11	70.3258374	67.2842889	70.8195316	68.8813582	64.5172777
18:26:33	0:24:12	70.2258374	65.2842889	70.8195316	69.8813582	64.5172777
18:26:34	0:24:13	70.4258374	64.2842889	69.8195316	70.8813582	65.5172777
18:26:35	0:24:14	70.6258374	64.2842889	69.8195316	71.8813582	66.5172777
18:26:36	0:24:15	70.9258374	64.2842889	68.8195316	71.8813582	67.5172777
18:26:37	0:24:16	70.8258374	64.2842889	68.8195316	70.8813582	67.5172777
18:26:38	0:24:17	71.1258374	65.2842889	67.8195316	70.8813582	67.5172777
18:26:39	0:24:18	71.4258374	66.2842889	67.8195316	70.8813582	68.5172777
18:26:40	0:24:19	71.4258374	66.2842889	67.8195316	70.8813582	68.5172777
18:26:41	0:24:20	71.4258374	68.2842889	68.8195316	70.8813582	67.5172777
18:26:42	0:24:21	71.3258374	67.2842889	68.8195316	69.8813582	67.5172777
18:26:43	0:24:22	71.2258374	66.2842889	69.8195316	68.8813582	67.5172777
18:26:44	0:24:23	71.0258374	66.2842889	69.8195316	67.8813582	67.5172777
18:26:45	0:24:24	71.0258374	66.2842889	69.8195316	67.8813582	67.5172777
18:26:46	0:24:25	71.1258374	66.2842889	69.8195316	66.8813582	67.5172777
18:26:47	0:24:26	71.2258374	66.2842889	68.8195316	66.8813582	67.5172777
18:26:48	0:24:27	71.5258374	66.2842889	68.8195316	67.8813582	67.5172777
18:26:49	0:24:28	71.4258374	66.2842889	69.8195316	67.8813582	67.5172777
18:26:50	0:24:29	71.1258374	66.2842889	69.8195316	68.8813582	67.5172777
18:26:51	0:24:30	71.4258374	66.2842889	69.8195316	68.8813582	67.5172777
18:26:52	0:24:31	71.1258374	66.2842889	71.8195316	68.8813582	65.5172777
18:26:53	0:24:32	71.2258374	66.2842889	72.8195316	68.8813582	65.5172777
18:26:54	0:24:33	71.7258374	68.2842889	73.8195316	68.8813582	66.5172777
18:26:55	0:24:34	71.7258374	69.2842889	72.8195316	69.8813582	66.5172777
18:26:56	0:24:35	71.6258374	68.2842889	73.8195316	69.8813582	66.5172777
18:26:57	0:24:36	71.4258374	68.2842889	73.8195316	69.8813582	66.5172777
18:26:58	0:24:37	70.7258374	68.2842889	72.8195316	69.8813582	66.5172777
18:26:59	0:24:38	71.2258374	68.2842889	72.8195316	69.8813582	66.5172777
18:27:00	0:24:39	71.2258374	68.2842889	72.8195316	69.8813582	66.5172777
18:27:01	0:24:40	71.8258374	68.2842889	71.8195316	69.8813582	65.5172777
18:27:02	0:24:41	71.9258374	68.2842889	71.8195316	69.8813582	66.5172777
18:27:03	0:24:42	72.2258374	68.2842889	71.8195316	69.8813582	68.5172777

18:27:04	0:24:43	72.1258374	68.2842889	71.8195316	69.8813582	68.5172777
18:27:05	0:24:44	72.8258374	68.2842889	71.8195316	70.8813582	68.5172777
18:27:06	0:24:45	73.6258374	67.2842889	71.8195316	71.8813582	71.5172777
18:27:07	0:24:46	74.0258374	66.2842889	71.8195316	75.8813582	72.5172777
18:27:08	0:24:47	73.9258374	66.2842889	72.8195316	75.8813582	72.5172777
18:27:09	0:24:48	73.8258374	66.2842889	72.8195316	75.8813582	73.5172777
18:27:10	0:24:49	74.0258374	66.2842889	72.8195316	73.8813582	73.5172777
18:27:11	0:24:50	73.9258374	66.2842889	71.8195316	71.8813582	73.5172777
18:27:12	0:24:51	74.3258374	66.2842889	71.8195316	70.8813582	72.5172777
18:27:13	0:24:52	74.2258374	66.2842889	71.8195316	69.8813582	71.5172777
18:27:14	0:24:53	73.9258374	66.2842889	71.8195316	69.8813582	71.5172777
18:27:15	0:24:54	73.7258374	65.2842889	71.8195316	69.8813582	72.5172777
18:27:16	0:24:55	73.7258374	64.2842889	71.8195316	69.8813582	73.5172777
18:27:17	0:24:56	74.7258374	64.2842889	72.8195316	68.8813582	73.5172777
18:27:18	0:24:57	75.2258374	64.2842889	72.8195316	68.8813582	73.5172777
18:27:19	0:24:58	75.4258374	64.2842889	72.8195316	68.8813582	74.5172777
18:27:20	0:24:59	75.2258374	64.2842889	72.8195316	68.8813582	74.5172777
18:27:21	0:25:00	74.9258374	64.2842889	72.8195316	68.8813582	72.5172777
18:27:22	0:25:01	74.1258374	63.2842889	72.8195316	68.8813582	69.5172777
18:27:23	0:25:02	72.7258374	63.2842889	72.8195316	68.8813582	69.5172777
18:27:24	0:25:03	72.3258374	64.2842889	72.8195316	67.8813582	69.5172777
18:27:25	0:25:04	72.2258374	64.2842889	73.8195316	67.8813582	70.5172777
18:27:26	0:25:05	71.8258374	64.2842889	74.8195316	67.8813582	71.5172777
18:27:27	0:25:06	71.5258374	65.2842889	74.8195316	67.8813582	70.5172777
18:27:28	0:25:07	71.0258374	65.2842889	75.8195316	67.8813582	68.5172777
18:27:29	0:25:08	70.7258374	65.2842889	75.8195316	67.8813582	68.5172777
18:27:30	0:25:09	70.7258374	65.2842889	75.8195316	67.8813582	69.5172777
18:27:31	0:25:10	70.9258374	65.2842889	75.8195316	67.8813582	69.5172777
18:27:32	0:25:11	70.9258374	64.2842889	75.8195316	68.8813582	69.5172777
18:27:33	0:25:12	70.7258374	63.2842889	75.8195316	68.8813582	69.5172777
18:27:34	0:25:13	70.6258374	64.2842889	75.8195316	68.8813582	69.5172777
18:27:35	0:25:14	69.8258374	65.2842889	74.8195316	68.8813582	69.5172777
18:27:36	0:25:15	69.5258374	66.2842889	73.8195316	68.8813582	69.5172777
18:27:37	0:25:16	69.2258374	66.2842889	73.8195316	67.8813582	67.5172777
18:27:38	0:25:17	69.2258374	66.2842889	73.8195316	67.8813582	67.5172777
18:27:39	0:25:18	69.4258374	66.2842889	73.8195316	67.8813582	67.5172777
18:27:40	0:25:19	70.0258374	66.2842889	75.8195316	67.8813582	67.5172777
18:27:41	0:25:20	70.4258374	66.2842889	75.8195316	68.8813582	67.5172777
18:27:42	0:25:21	70.6258374	68.2842889	75.8195316	68.8813582	67.5172777
18:27:43	0:25:22	70.7258374	69.2842889	75.8195316	69.8813582	68.5172777
18:27:44	0:25:23	70.3258374	70.2842889	74.8195316	69.8813582	68.5172777
18:27:45	0:25:24	70.0258374	70.2842889	75.8195316	69.8813582	68.5172777
18:27:46	0:25:25	70.6258374	69.2842889	75.8195316	69.8813582	70.5172777
18:27:47	0:25:26	70.6258374	69.2842889	75.8195316	69.8813582	71.5172777
18:27:48	0:25:27	70.4258374	68.2842889	75.8195316	69.8813582	71.5172777

18:27:49	0:25:28	70.5258374	68.2842889	75.8195316	69.8813582	73.5172777
18:27:50	0:25:29	70.9258374	68.2842889	76.8195316	69.8813582	75.5172777
18:27:51	0:25:30	71.0258374	68.2842889	76.8195316	69.8813582	76.5172777
18:27:52	0:25:31	70.7258374	68.2842889	75.8195316	69.8813582	76.5172777
18:27:53	0:25:32	70.8258374	68.2842889	75.8195316	69.8813582	78.5172777
18:27:54	0:25:33	70.8258374	68.2842889	75.8195316	69.8813582	78.5172777
18:27:55	0:25:34	70.5258374	68.2842889	72.8195316	71.8813582	78.5172777
18:27:56	0:25:35	70.6258374	67.2842889	71.8195316	71.8813582	79.5172777
18:27:57	0:25:36	71.1258374	67.2842889	71.8195316	71.8813582	79.5172777
18:27:58	0:25:37	71.1258374	67.2842889	70.8195316	71.8813582	78.5172777
18:27:59	0:25:38	71.5258374	67.2842889	71.8195316	71.8813582	78.5172777
18:28:00	0:25:39	71.4258374	67.2842889	71.8195316	70.8813582	78.5172777
18:28:01	0:25:40	71.6258374	67.2842889	72.8195316	70.8813582	78.5172777
18:28:02	0:25:41	71.8258374	65.2842889	72.8195316	70.8813582	79.5172777
18:28:03	0:25:42	72.1258374	64.2842889	72.8195316	70.8813582	79.5172777
18:28:04	0:25:43	72.1258374	64.2842889	72.8195316	71.8813582	78.5172777
18:28:05	0:25:44	73.2258374	65.2842889	71.8195316	71.8813582	76.5172777
18:28:06	0:25:45	73.8258374	65.2842889	71.8195316	71.8813582	74.5172777
18:28:07	0:25:46	74.0258374	65.2842889	70.8195316	71.8813582	74.5172777
18:28:08	0:25:47	73.2258374	66.2842889	70.8195316	70.8813582	71.5172777
18:28:09	0:25:48	73.0258374	66.2842889	70.8195316	70.8813582	69.5172777
18:28:10	0:25:49	72.5258374	66.2842889	71.8195316	70.8813582	68.5172777
18:28:11	0:25:50	73.0258374	66.2842889	72.8195316	70.8813582	68.5172777
18:28:12	0:25:51	73.6258374	66.2842889	73.8195316	69.8813582	68.5172777
18:28:13	0:25:52	73.6258374	66.2842889	73.8195316	69.8813582	69.5172777
18:28:14	0:25:53	74.0258374	66.2842889	76.8195316	69.8813582	71.5172777
18:28:15	0:25:54	74.2258374	66.2842889	78.8195316	68.8813582	73.5172777
18:28:16	0:25:55	74.1258374	66.2842889	78.8195316	68.8813582	74.5172777
18:28:17	0:25:56	74.6258374	66.2842889	78.8195316	68.8813582	73.5172777
18:28:18	0:25:57	74.7258374	66.2842889	78.8195316	68.8813582	72.5172777
18:28:19	0:25:58	74.2258374	66.2842889	76.8195316	68.8813582	70.5172777
18:28:20	0:25:59	73.6258374	69.2842889	76.8195316	68.8813582	68.5172777
18:28:21	0:26:00	74.0258374	69.2842889	76.8195316	74.8813582	67.5172777
18:28:22	0:26:01	74.0258374	70.2842889	76.8195316	76.8813582	67.5172777
18:28:23	0:26:02	74.4258374	70.2842889	77.8195316	77.8813582	68.5172777
18:28:24	0:26:03	74.8258374	70.2842889	77.8195316	79.8813582	69.5172777
18:28:25	0:26:04	75.3258374	70.2842889	81.8195316	80.8813582	71.5172777
18:28:26	0:26:05	74.3258374	71.2842889	81.8195316	76.8813582	71.5172777
18:28:27	0:26:06	73.8258374	71.2842889	80.8195316	75.8813582	70.5172777
18:28:28	0:26:07	73.6258374	72.2842889	80.8195316	74.8813582	70.5172777
18:28:29	0:26:08	72.5258374	72.2842889	76.8195316	72.8813582	70.5172777
18:28:30	0:26:09	72.1258374	72.2842889	74.8195316	71.8813582	70.5172777
18:28:31	0:26:10	72.6258374	73.2842889	74.8195316	70.8813582	71.5172777
18:28:32	0:26:11	72.5258374	73.2842889	73.8195316	70.8813582	71.5172777
18:28:33	0:26:12	72.6258374	73.2842889	73.8195316	70.8813582	71.5172777

18:28:34	0:26:13	73.1258374	73.2842889	73.8195316	71.8813582	74.5172777
18:28:35	0:26:14	73.3258374	73.2842889	74.8195316	70.8813582	76.5172777
18:28:36	0:26:15	73.0258374	73.2842889	73.8195316	70.8813582	76.5172777
18:28:37	0:26:16	74.0258374	80.2842889	73.8195316	70.8813582	75.5172777
18:28:38	0:26:17	73.2258374	80.2842889	71.8195316	69.8813582	71.5172777
18:28:39	0:26:18	73.3258374	82.2842889	71.8195316	69.8813582	69.5172777
18:28:40	0:26:19	72.9258374	83.2842889	70.8195316	69.8813582	67.5172777
18:28:41	0:26:20	72.6258374	83.2842889	70.8195316	69.8813582	66.5172777
18:28:42	0:26:21	72.6258374	84.2842889	70.8195316	69.8813582	66.5172777
18:28:43	0:26:22	72.6258374	84.2842889	70.8195316	69.8813582	67.5172777
18:28:44	0:26:23	72.8258374	83.2842889	70.8195316	69.8813582	67.5172777
18:28:45	0:26:24	72.4258374	79.2842889	70.8195316	69.8813582	68.5172777
18:28:46	0:26:25	71.9258374	71.2842889	71.8195316	70.8813582	68.5172777
18:28:47	0:26:26	72.6258374	71.2842889	71.8195316	72.8813582	69.5172777
18:28:48	0:26:27	72.6258374	71.2842889	71.8195316	72.8813582	69.5172777
18:28:49	0:26:28	72.5258374	71.2842889	70.8195316	73.8813582	70.5172777
18:28:50	0:26:29	72.1258374	70.2842889	70.8195316	74.8813582	70.5172777
18:28:51	0:26:30	72.4258374	70.2842889	70.8195316	74.8813582	71.5172777
18:28:52	0:26:31	72.6258374	73.2842889	70.8195316	74.8813582	70.5172777
18:28:53	0:26:32	72.6258374	73.2842889	70.8195316	74.8813582	70.5172777
18:28:54	0:26:33	72.8258374	73.2842889	71.8195316	74.8813582	69.5172777
18:28:55	0:26:34	73.2258374	74.2842889	73.8195316	74.8813582	69.5172777
18:28:56	0:26:35	73.2258374	73.2842889	73.8195316	74.8813582	69.5172777
18:28:57	0:26:36	73.4258374	73.2842889	76.8195316	74.8813582	69.5172777
18:28:58	0:26:37	73.6258374	73.2842889	77.8195316	74.8813582	69.5172777
18:28:59	0:26:38	73.3258374	73.2842889	76.8195316	74.8813582	70.5172777
18:29:00	0:26:39	73.0258374	73.2842889	75.8195316	74.8813582	70.5172777
18:29:01	0:26:40	72.5258374	73.2842889	75.8195316	74.8813582	70.5172777
18:29:02	0:26:41	72.3258374	70.2842889	75.8195316	73.8813582	70.5172777
18:29:03	0:26:42	71.9258374	69.2842889	73.8195316	71.8813582	70.5172777
18:29:04	0:26:43	71.5258374	69.2842889	73.8195316	71.8813582	69.5172777
18:29:05	0:26:44	71.1258374	68.2842889	72.8195316	69.8813582	68.5172777
18:29:06	0:26:45	70.4258374	65.2842889	72.8195316	69.8813582	67.5172777
18:29:07	0:26:46	70.3258374	64.2842889	72.8195316	70.8813582	66.5172777
18:29:08	0:26:47	70.6258374	64.2842889	73.8195316	70.8813582	66.5172777
18:29:09	0:26:48	70.7258374	63.2842889	73.8195316	70.8813582	66.5172777
18:29:10	0:26:49	71.1258374	63.2842889	72.8195316	70.8813582	66.5172777
18:29:11	0:26:50	71.3258374	63.2842889	72.8195316	70.8813582	66.5172777
18:29:12	0:26:51	71.2258374	64.2842889	71.8195316	70.8813582	66.5172777
18:29:13	0:26:52	71.2258374	65.2842889	71.8195316	69.8813582	67.5172777
18:29:14	0:26:53	71.3258374	65.2842889	71.8195316	69.8813582	66.5172777
18:29:15	0:26:54	71.1258374	65.2842889	70.8195316	69.8813582	66.5172777
18:29:16	0:26:55	71.1258374	65.2842889	70.8195316	69.8813582	65.5172777
18:29:17	0:26:56	71.2258374	67.2842889	70.8195316	69.8813582	65.5172777
18:29:18	0:26:57	71.0258374	67.2842889	70.8195316	69.8813582	65.5172777

18:29:19	0:26:58	71.0258374	66.2842889	70.8195316	69.8813582	66.5172777
18:29:20	0:26:59	70.8258374	66.2842889	70.8195316	69.8813582	66.5172777
18:29:21	0:27:00	70.5258374	66.2842889	70.8195316	69.8813582	67.5172777
18:29:22	0:27:01	70.6258374	67.2842889	70.8195316	69.8813582	67.5172777
18:29:23	0:27:02	70.8258374	68.2842889	69.8195316	70.8813582	68.5172777
18:29:24	0:27:03	70.6258374	68.2842889	69.8195316	70.8813582	68.5172777
18:29:25	0:27:04	70.6258374	68.2842889	69.8195316	70.8813582	68.5172777
18:29:26	0:27:05	70.4258374	68.2842889	69.8195316	69.8813582	68.5172777
18:29:27	0:27:06	70.4258374	69.2842889	69.8195316	69.8813582	68.5172777
18:29:28	0:27:07	70.4258374	69.2842889	69.8195316	69.8813582	68.5172777
18:29:29	0:27:08	71.1258374	69.2842889	72.8195316	69.8813582	69.5172777
18:29:30	0:27:09	71.7258374	69.2842889	74.8195316	69.8813582	70.5172777
18:29:31	0:27:10	72.1258374	69.2842889	74.8195316	69.8813582	73.5172777
18:29:32	0:27:11	73.1258374	70.2842889	77.8195316	69.8813582	74.5172777
18:29:33	0:27:12	73.2258374	70.2842889	79.8195316	69.8813582	74.5172777
18:29:34	0:27:13	73.0258374	69.2842889	79.8195316	70.8813582	75.5172777
18:29:35	0:27:14	73.0258374	69.2842889	79.8195316	70.8813582	74.5172777
18:29:36	0:27:15	73.3258374	69.2842889	79.8195316	70.8813582	73.5172777
18:29:37	0:27:16	73.1258374	69.2842889	78.8195316	70.8813582	72.5172777
18:29:38	0:27:17	73.2258374	69.2842889	78.8195316	71.8813582	72.5172777
18:29:39	0:27:18	72.7258374	69.2842889	77.8195316	72.8813582	71.5172777
18:29:40	0:27:19	72.3258374	63.2842889	74.8195316	73.8813582	71.5172777
18:29:41	0:27:20	72.6258374	63.2842889	74.8195316	73.8813582	70.5172777
18:29:42	0:27:21	72.6258374	63.2842889	74.8195316	73.8813582	70.5172777
18:29:43	0:27:22	72.4258374	63.2842889	74.8195316	73.8813582	69.5172777
18:29:44	0:27:23	71.8258374	63.2842889	74.8195316	72.8813582	69.5172777
18:29:45	0:27:24	71.8258374	63.2842889	73.8195316	72.8813582	69.5172777
18:29:46	0:27:25	71.9258374	63.2842889	73.8195316	72.8813582	68.5172777
18:29:47	0:27:26	71.9258374	63.2842889	74.8195316	72.8813582	68.5172777
18:29:48	0:27:27	72.2258374	64.2842889	76.8195316	72.8813582	68.5172777
18:29:49	0:27:28	72.1258374	64.2842889	76.8195316	71.8813582	68.5172777
18:29:50	0:27:29	72.1258374	64.2842889	76.8195316	72.8813582	69.5172777
18:29:51	0:27:30	71.7258374	64.2842889	73.8195316	73.8813582	69.5172777
18:29:52	0:27:31	72.0258374	64.2842889	73.8195316	73.8813582	69.5172777
18:29:53	0:27:32	71.9258374	64.2842889	72.8195316	72.8813582	69.5172777
18:29:54	0:27:33	71.7258374	64.2842889	71.8195316	71.8813582	69.5172777
18:29:55	0:27:34	71.6258374	64.2842889	70.8195316	70.8813582	69.5172777
18:29:56	0:27:35	72.0258374	64.2842889	70.8195316	70.8813582	69.5172777
18:29:57	0:27:36	72.1258374	63.2842889	70.8195316	70.8813582	69.5172777
18:29:58	0:27:37	72.0258374	63.2842889	69.8195316	71.8813582	68.5172777
18:29:59	0:27:38	71.7258374	63.2842889	69.8195316	71.8813582	69.5172777
18:30:00	0:27:39	71.7258374	63.2842889	69.8195316	72.8813582	69.5172777
18:30:01	0:27:40	71.7258374	63.2842889	67.8195316	72.8813582	69.5172777
18:30:02	0:27:41	71.4258374	62.2842889	67.8195316	72.8813582	69.5172777
18:30:03	0:27:42	71.7258374	62.2842889	66.8195316	72.8813582	69.5172777

18:30:04	0:27:43	71.9258374	62.2842889	67.8195316	72.8813582	69.5172777
18:30:05	0:27:44	72.0258374	62.2842889	67.8195316	72.8813582	69.5172777
18:30:06	0:27:45	72.1258374	64.2842889	67.8195316	73.8813582	70.5172777
18:30:07	0:27:46	72.6258374	63.2842889	68.8195316	75.8813582	71.5172777
18:30:08	0:27:47	72.4258374	64.2842889	68.8195316	75.8813582	71.5172777
18:30:09	0:27:48	72.4258374	64.2842889	68.8195316	75.8813582	71.5172777
18:30:10	0:27:49	72.6258374	64.2842889	68.8195316	75.8813582	73.5172777
18:30:11	0:27:50	72.6258374	64.2842889	68.8195316	75.8813582	73.5172777
18:30:12	0:27:51	72.4258374	64.2842889	68.8195316	75.8813582	73.5172777
18:30:13	0:27:52	72.2258374	64.2842889	65.8195316	75.8813582	73.5172777
18:30:14	0:27:53	72.4258374	65.2842889	65.8195316	75.8813582	73.5172777
18:30:15	0:27:54	72.1258374	65.2842889	65.8195316	75.8813582	72.5172777
18:30:16	0:27:55	72.0258374	65.2842889	65.8195316	75.8813582	72.5172777
18:30:17	0:27:56	71.8258374	66.2842889	65.8195316	75.8813582	71.5172777
18:30:18	0:27:57	72.0258374	66.2842889	66.8195316	75.8813582	71.5172777
18:30:19	0:27:58	72.5258374	65.2842889	68.8195316	75.8813582	71.5172777
18:30:20	0:27:59	72.8258374	65.2842889	69.8195316	75.8813582	71.5172777
18:30:21	0:28:00	73.2258374	66.2842889	69.8195316	75.8813582	70.5172777
18:30:22	0:28:01	73.3258374	66.2842889	69.8195316	73.8813582	70.5172777
18:30:23	0:28:02	73.3258374	66.2842889	69.8195316	71.8813582	72.5172777
18:30:24	0:28:03	73.4258374	65.2842889	68.8195316	70.8813582	74.5172777
18:30:25	0:28:04	73.3258374	62.2842889	68.8195316	69.8813582	75.5172777
18:30:26	0:28:05	73.1258374	62.2842889	68.8195316	69.8813582	73.5172777
18:30:27	0:28:06	73.1258374	63.2842889	69.8195316	69.8813582	72.5172777
18:30:28	0:28:07	72.8258374	64.2842889	68.8195316	69.8813582	72.5172777
18:30:29	0:28:08	72.4258374	64.2842889	68.8195316	69.8813582	71.5172777
18:30:30	0:28:09	71.9258374	64.2842889	68.8195316	70.8813582	71.5172777
18:30:31	0:28:10	71.5258374	66.2842889	67.8195316	70.8813582	71.5172777
18:30:32	0:28:11	71.4258374	69.2842889	66.8195316	70.8813582	70.5172777
18:30:33	0:28:12	71.7258374	69.2842889	67.8195316	70.8813582	70.5172777
18:30:34	0:28:13	71.8258374	70.2842889	68.8195316	70.8813582	70.5172777
18:30:35	0:28:14	71.9258374	70.2842889	68.8195316	70.8813582	71.5172777
18:30:36	0:28:15	71.9258374	70.2842889	68.8195316	70.8813582	71.5172777
18:30:37	0:28:16	72.3258374	72.2842889	68.8195316	70.8813582	71.5172777
18:30:38	0:28:17	72.5258374	72.2842889	69.8195316	70.8813582	71.5172777
18:30:39	0:28:18	72.5258374	72.2842889	69.8195316	70.8813582	71.5172777
18:30:40	0:28:19	72.2258374	72.2842889	69.8195316	70.8813582	71.5172777
18:30:41	0:28:20	72.0258374	72.2842889	69.8195316	70.8813582	72.5172777
18:30:42	0:28:21	72.1258374	73.2842889	69.8195316	70.8813582	74.5172777
18:30:43	0:28:22	71.7258374	69.2842889	69.8195316	70.8813582	74.5172777
18:30:44	0:28:23	72.3258374	69.2842889	69.8195316	70.8813582	76.5172777
18:30:45	0:28:24	72.3258374	69.2842889	68.8195316	70.8813582	77.5172777
18:30:46	0:28:25	72.2258374	68.2842889	68.8195316	70.8813582	78.5172777
18:30:47	0:28:26	72.1258374	68.2842889	68.8195316	70.8813582	78.5172777
18:30:48	0:28:27	71.9258374	67.2842889	68.8195316	71.8813582	78.5172777

18:30:49	0:28:28	71.6258374	67.2842889	67.8195316	71.8813582	77.5172777
18:30:50	0:28:29	71.4258374	67.2842889	67.8195316	70.8813582	77.5172777
18:30:51	0:28:30	70.9258374	67.2842889	67.8195316	70.8813582	74.5172777
18:30:52	0:28:31	70.7258374	67.2842889	66.8195316	70.8813582	73.5172777
18:30:53	0:28:32	70.9258374	67.2842889	66.8195316	71.8813582	72.5172777
18:30:54	0:28:33	71.1258374	67.2842889	66.8195316	70.8813582	72.5172777
18:30:55	0:28:34	71.1258374	66.2842889	65.8195316	71.8813582	72.5172777
18:30:56	0:28:35	71.5258374	66.2842889	65.8195316	72.8813582	72.5172777
18:30:57	0:28:36	71.7258374	66.2842889	66.8195316	72.8813582	72.5172777
18:30:58	0:28:37	72.0258374	66.2842889	67.8195316	72.8813582	73.5172777
18:30:59	0:28:38	72.4258374	66.2842889	70.8195316	72.8813582	73.5172777
18:31:00	0:28:39	72.6258374	66.2842889	70.8195316	72.8813582	73.5172777
18:31:01	0:28:40	72.7258374	66.2842889	70.8195316	73.8813582	71.5172777
18:31:02	0:28:41	72.3258374	66.2842889	70.8195316	74.8813582	69.5172777
18:31:03	0:28:42	71.9258374	65.2842889	69.8195316	74.8813582	68.5172777
18:31:04	0:28:43	72.5258374	65.2842889	69.8195316	74.8813582	66.5172777
18:31:05	0:28:44	73.0258374	65.2842889	69.8195316	73.8813582	66.5172777
18:31:06	0:28:45	72.8258374	65.2842889	68.8195316	73.8813582	65.5172777
18:31:07	0:28:46	73.0258374	66.2842889	67.8195316	72.8813582	65.5172777
18:31:08	0:28:47	73.5258374	71.2842889	67.8195316	72.8813582	65.5172777
18:31:09	0:28:48	73.8258374	72.2842889	67.8195316	72.8813582	65.5172777
18:31:10	0:28:49	74.1258374	71.2842889	67.8195316	72.8813582	65.5172777
18:31:11	0:28:50	74.4258374	71.2842889	68.8195316	72.8813582	66.5172777
18:31:12	0:28:51	74.6258374	71.2842889	68.8195316	72.8813582	67.5172777
18:31:13	0:28:52	74.5258374	70.2842889	68.8195316	72.8813582	67.5172777
18:31:14	0:28:53	74.3258374	70.2842889	67.8195316	72.8813582	68.5172777
18:31:15	0:28:54	74.3258374	70.2842889	66.8195316	73.8813582	68.5172777
18:31:16	0:28:55	74.5258374	69.2842889	66.8195316	74.8813582	68.5172777
18:31:17	0:28:56	74.6258374	69.2842889	67.8195316	74.8813582	68.5172777
18:31:18	0:28:57	74.1258374	69.2842889	68.8195316	74.8813582	68.5172777
18:31:19	0:28:58	73.8258374	69.2842889	68.8195316	74.8813582	69.5172777
18:31:20	0:28:59	73.5258374	68.2842889	68.8195316	74.8813582	69.5172777
18:31:21	0:29:00	73.6258374	68.2842889	68.8195316	74.8813582	70.5172777
18:31:22	0:29:01	74.3258374	68.2842889	70.8195316	74.8813582	70.5172777
18:31:23	0:29:02	74.0258374	68.2842889	70.8195316	74.8813582	71.5172777
18:31:24	0:29:03	73.5258374	68.2842889	70.8195316	74.8813582	71.5172777
18:31:25	0:29:04	73.2258374	69.2842889	70.8195316	73.8813582	71.5172777
18:31:26	0:29:05	73.0258374	69.2842889	70.8195316	73.8813582	71.5172777
18:31:27	0:29:06	73.4258374	70.2842889	70.8195316	72.8813582	70.5172777
18:31:28	0:29:07	73.0258374	70.2842889	69.8195316	71.8813582	70.5172777
18:31:29	0:29:08	72.7258374	70.2842889	69.8195316	71.8813582	70.5172777
18:31:30	0:29:09	72.7258374	70.2842889	69.8195316	71.8813582	70.5172777
18:31:31	0:29:10	72.4258374	70.2842889	70.8195316	72.8813582	70.5172777
18:31:32	0:29:11	72.3258374	71.2842889	70.8195316	72.8813582	70.5172777
18:31:33	0:29:12	72.5258374	73.2842889	70.8195316	71.8813582	70.5172777

18:31:34	0:29:13	72.3258374	74.2842889	71.8195316	70.8813582	70.5172777
18:31:35	0:29:14	71.9258374	74.2842889	71.8195316	70.8813582	70.5172777
18:31:36	0:29:15	71.8258374	75.2842889	71.8195316	69.8813582	70.5172777
18:31:37	0:29:16	71.6258374	76.2842889	71.8195316	68.8813582	70.5172777
18:31:38	0:29:17	71.7258374	76.2842889	71.8195316	68.8813582	70.5172777
18:31:39	0:29:18	72.0258374	76.2842889	71.8195316	68.8813582	71.5172777
18:31:40	0:29:19	72.2258374	76.2842889	71.8195316	68.8813582	71.5172777
18:31:41	0:29:20	71.5258374	72.2842889	71.8195316	68.8813582	71.5172777
18:31:42	0:29:21	71.2258374	70.2842889	71.8195316	68.8813582	71.5172777
18:31:43	0:29:22	71.0258374	70.2842889	72.8195316	69.8813582	71.5172777
18:31:44	0:29:23	71.1258374	70.2842889	74.8195316	70.8813582	72.5172777
18:31:45	0:29:24	70.3258374	69.2842889	74.8195316	70.8813582	72.5172777
18:31:46	0:29:25	69.9258374	69.2842889	74.8195316	70.8813582	71.5172777
18:31:47	0:29:26	70.2258374	69.2842889	73.8195316	70.8813582	71.5172777
18:31:48	0:29:27	70.3258374	69.2842889	71.8195316	70.8813582	70.5172777
18:31:49	0:29:28	70.2258374	69.2842889	71.8195316	70.8813582	69.5172777
18:31:50	0:29:29	70.6258374	69.2842889	71.8195316	71.8813582	69.5172777
18:31:51	0:29:30	70.8258374	69.2842889	70.8195316	72.8813582	69.5172777
18:31:52	0:29:31	70.8258374	68.2842889	71.8195316	72.8813582	69.5172777
18:31:53	0:29:32	71.0258374	68.2842889	71.8195316	73.8813582	69.5172777
18:31:54	0:29:33	70.9258374	68.2842889	71.8195316	73.8813582	70.5172777
18:31:55	0:29:34	70.5258374	67.2842889	71.8195316	74.8813582	70.5172777
18:31:56	0:29:35	70.7258374	67.2842889	71.8195316	74.8813582	70.5172777
18:31:57	0:29:36	70.9258374	67.2842889	72.8195316	74.8813582	71.5172777
18:31:58	0:29:37	71.5258374	67.2842889	72.8195316	74.8813582	71.5172777
18:31:59	0:29:38	71.7258374	67.2842889	72.8195316	74.8813582	72.5172777
18:32:00	0:29:39	72.1258374	67.2842889	73.8195316	75.8813582	73.5172777
18:32:01	0:29:40	72.2258374	68.2842889	73.8195316	75.8813582	73.5172777
18:32:02	0:29:41	71.9258374	68.2842889	75.8195316	75.8813582	72.5172777
18:32:03	0:29:42	71.9258374	67.2842889	75.8195316	75.8813582	72.5172777
18:32:04	0:29:43	72.2258374	67.2842889	74.8195316	75.8813582	73.5172777
18:32:05	0:29:44	72.7258374	67.2842889	74.8195316	78.8813582	74.5172777
18:32:06	0:29:45	72.6258374	67.2842889	73.8195316	80.8813582	74.5172777
18:32:07	0:29:46	73.0258374	67.2842889	72.8195316	81.8813582	76.5172777
18:32:08	0:29:47	73.4258374	66.2842889	72.8195316	81.8813582	78.5172777
18:32:09	0:29:48	73.7258374	66.2842889	72.8195316	81.8813582	80.5172777
18:32:10	0:29:49	73.7258374	66.2842889	72.8195316	81.8813582	81.5172777
18:32:11	0:29:50	73.9258374	66.2842889	73.8195316	80.8813582	82.5172777
18:32:12	0:29:51	75.9258374	66.2842889	73.8195316	80.8813582	82.5172777
18:32:13	0:29:52	76.1258374	65.2842889	74.8195316	80.8813582	82.5172777
18:32:14	0:29:53	76.3258374	65.2842889	74.8195316	79.8813582	84.5172777
18:32:15	0:29:54	76.2258374	64.2842889	73.8195316	78.8813582	85.5172777
18:32:16	0:29:55	76.5258374	64.2842889	71.8195316	78.8813582	88.5172777
18:32:17	0:29:56	76.8258374	64.2842889	70.8195316	78.8813582	89.5172777
18:32:18	0:29:57	76.9258374	64.2842889	70.8195316	77.8813582	76.9258374

18:32:19	0:29:58	77.1258374	64.2842889	69.8195316	77.8813582	77.1258374
18:32:20	0:29:59	76.7258374	64.2842889	67.8195316	77.8813582	76.7258374
18:32:21	0:30:00	75.9258374	64.2842889	67.8195316	76.8813582	75.9258374
18:32:22	0:30:01	75.8258374	64.2842889	67.8195316	76.8813582	75.8258374
18:32:23	0:30:02	75.7258374	64.2842889	67.8195316	76.8813582	75.7258374
18:32:24	0:30:03	75.7258374	64.2842889	67.8195316	76.8813582	75.7258374
18:32:25	0:30:04	73.8258374	63.2842889	68.8195316	76.8813582	73.8258374
18:32:26	0:30:05	73.6258374	62.2842889	67.8195316	76.8813582	73.6258374
18:32:27	0:30:06	73.6258374	62.2842889	67.8195316	76.8813582	85.5172777
18:32:28	0:30:07	73.5258374	62.2842889	67.8195316	76.8813582	83.5172777
18:32:29	0:30:08	73.3258374	62.2842889	67.8195316	77.8813582	80.5172777
18:32:30	0:30:09	72.8258374	62.2842889	66.8195316	77.8813582	79.5172777
18:32:31	0:30:10	71.8258374	62.2842889	65.8195316	77.8813582	78.5172777
18:32:32	0:30:11	71.4258374	62.2842889	65.8195316	77.8813582	78.5172777
18:32:33	0:30:12	71.3258374	62.2842889	65.8195316	77.8813582	78.5172777
18:32:34	0:30:13	70.6258374	62.2842889	65.8195316	78.8813582	78.5172777
18:32:35	0:30:14	70.0258374	62.2842889	65.8195316	78.8813582	76.5172777
18:32:36	0:30:15	69.1258374	62.2842889	65.8195316	78.8813582	75.5172777
18:32:37	0:30:16	68.9258374	62.2842889	65.8195316	78.8813582	74.5172777
18:32:38	0:30:17	68.9258374	62.2842889	66.8195316	80.8813582	74.5172777
18:32:39	0:30:18	68.9258374	63.2842889	66.8195316	80.8813582	73.5172777
18:32:40	0:30:19	69.0258374	63.2842889	66.8195316	81.8813582	73.5172777
18:32:41	0:30:20	69.4258374	64.2842889	65.8195316	81.8813582	75.5172777
18:32:42	0:30:21	69.8258374	64.2842889	66.8195316	81.8813582	76.5172777
18:32:43	0:30:22	70.4258374	64.2842889	65.8195316	81.8813582	79.5172777
18:32:44	0:30:23	71.1258374	65.2842889	66.8195316	81.8813582	80.5172777
18:32:45	0:30:24	71.6258374	66.2842889	66.8195316	80.8813582	81.5172777
18:32:46	0:30:25	71.5258374	66.2842889	66.8195316	79.8813582	79.5172777
18:32:47	0:30:26	71.6258374	66.2842889	67.8195316	79.8813582	79.5172777
18:32:48	0:30:27	71.7258374	64.2842889	67.8195316	79.8813582	80.5172777
18:32:49	0:30:28	71.8258374	62.2842889	67.8195316	80.8813582	80.5172777
18:32:50	0:30:29	72.0258374	62.2842889	67.8195316	80.8813582	80.5172777
18:32:51	0:30:30	72.0258374	62.2842889	68.8195316	79.8813582	77.5172777
18:32:52	0:30:31	71.6258374	62.2842889	69.8195316	79.8813582	77.5172777
18:32:53	0:30:32	71.1258374	62.2842889	71.8195316	79.8813582	71.5172777
18:32:54	0:30:33	71.6258374	62.2842889	72.8195316	79.8813582	71.5172777
18:32:55	0:30:34	71.4258374	63.2842889	71.8195316	79.8813582	68.5172777
18:32:56	0:30:35	71.6258374	63.2842889	71.8195316	79.8813582	69.5172777
18:32:57	0:30:36	71.3258374	62.2842889	70.8195316	79.8813582	69.5172777
18:32:58	0:30:37	71.0258374	62.2842889	69.8195316	79.8813582	69.5172777
18:32:59	0:30:38	70.7258374	62.2842889	68.8195316	79.8813582	69.5172777
18:33:00	0:30:39	70.9258374	62.2842889	68.8195316	80.8813582	69.5172777
18:33:01	0:30:40	71.0258374	62.2842889	68.8195316	80.8813582	69.5172777
18:33:02	0:30:41	71.2258374	61.2842889	68.8195316	80.8813582	68.5172777
18:33:03	0:30:42	70.5258374	62.2842889	68.8195316	80.8813582	68.5172777

18:33:04	0:30:43	70.4258374	62.2842889	68.8195316	81.8813582	68.5172777
18:33:05	0:30:44	71.3258374	62.2842889	70.8195316	80.8813582	68.5172777
18:33:06	0:30:45	71.6258374	63.2842889	70.8195316	80.8813582	68.5172777
18:33:07	0:30:46	72.6258374	63.2842889	72.8195316	81.8813582	68.5172777
18:33:08	0:30:47	73.0258374	63.2842889	73.8195316	81.8813582	69.5172777
18:33:09	0:30:48	72.9258374	63.2842889	73.8195316	80.8813582	69.5172777
18:33:10	0:30:49	73.0258374	67.2842889	75.8195316	80.8813582	71.5172777
18:33:11	0:30:50	73.4258374	69.2842889	75.8195316	81.8813582	72.5172777
18:33:12	0:30:51	73.5258374	69.2842889	76.8195316	83.8813582	72.5172777
18:33:13	0:30:52	73.4258374	70.2842889	76.8195316	84.8813582	72.5172777
18:33:14	0:30:53	73.7258374	70.2842889	80.8195316	84.8813582	73.5172777
18:33:15	0:30:54	73.3258374	70.2842889	80.8195316	84.8813582	73.5172777
18:33:16	0:30:55	73.7258374	70.2842889	82.8195316	84.8813582	73.5172777
18:33:17	0:30:56	73.5258374	70.2842889	82.8195316	83.8813582	73.5172777
18:33:18	0:30:57	73.5258374	70.2842889	82.8195316	83.8813582	73.5172777
18:33:19	0:30:58	73.1258374	70.2842889	79.8195316	82.8813582	73.5172777
18:33:20	0:30:59	73.2258374	69.2842889	78.8195316	82.8813582	73.5172777
18:33:21	0:31:00	73.4258374	69.2842889	78.8195316	82.8813582	73.5172777
18:33:22	0:31:01	72.8258374	68.2842889	76.8195316	82.8813582	72.5172777
18:33:23	0:31:02	72.4258374	67.2842889	75.8195316	82.8813582	71.5172777
18:33:24	0:31:03	71.2258374	67.2842889	74.8195316	82.8813582	69.5172777
18:33:25	0:31:04	70.5258374	66.2842889	73.8195316	80.8813582	68.5172777
18:33:26	0:31:05	69.5258374	65.2842889	72.8195316	80.8813582	68.5172777
18:33:27	0:31:06	69.3258374	65.2842889	72.8195316	80.8813582	71.5172777
18:33:28	0:31:07	68.9258374	65.2842889	69.8195316	79.8813582	71.5172777
18:33:29	0:31:08	68.9258374	65.2842889	69.8195316	79.8813582	72.5172777
18:33:30	0:31:09	68.7258374	65.2842889	69.8195316	68.7258374	71.5172777
18:33:31	0:31:10	68.7258374	65.2842889	68.8195316	68.7258374	71.5172777
18:33:32	0:31:11	68.8258374	65.2842889	68.8195316	68.8258374	69.5172777
18:33:33	0:31:12	68.6258374	65.2842889	68.8195316	68.6258374	68.5172777
18:33:34	0:31:13	69.1258374	65.2842889	68.8195316	69.1258374	68.5172777
18:33:35	0:31:14	69.2258374	65.2842889	67.8195316	69.2258374	68.5172777
18:33:36	0:31:15	69.3258374	65.2842889	67.8195316	69.3258374	68.5172777
18:33:37	0:31:16	69.2258374	65.2842889	67.8195316	69.2258374	68.5172777
18:33:38	0:31:17	69.8258374	66.2842889	68.8195316	69.8258374	69.5172777
18:33:39	0:31:18	69.8258374	69.2842889	68.8195316	81.8813582	69.5172777
18:33:40	0:31:19	69.5258374	69.2842889	67.8195316	80.8813582	69.5172777
18:33:41	0:31:20	69.6258374	69.2842889	67.8195316	80.8813582	70.5172777
18:33:42	0:31:21	69.0258374	69.2842889	67.8195316	77.8813582	70.5172777
18:33:43	0:31:22	68.6258374	66.2842889	67.8195316	77.8813582	70.5172777
18:33:44	0:31:23	68.0258374	65.2842889	67.8195316	76.8813582	70.5172777
18:33:45	0:31:24	68.1258374	64.2842889	69.8195316	76.8813582	70.5172777
18:33:46	0:31:25	68.5258374	64.2842889	70.8195316	76.8813582	70.5172777
18:33:47	0:31:26	68.7258374	63.2842889	71.8195316	76.8813582	69.5172777
18:33:48	0:31:27	68.8258374	63.2842889	73.8195316	77.8813582	69.5172777

18:33:49	0:31:28	68.8258374	63.2842889	73.8195316	77.8813582	69.5172777
18:33:50	0:31:29	68.9258374	63.2842889	74.8195316	77.8813582	69.5172777
18:33:51	0:31:30	69.0258374	63.2842889	73.8195316	76.8813582	69.5172777
18:33:52	0:31:31	69.3258374	63.2842889	73.8195316	76.8813582	69.5172777
18:33:53	0:31:32	69.2258374	63.2842889	73.8195316	75.8813582	69.5172777
18:33:54	0:31:33	69.8258374	63.2842889	75.8195316	75.8813582	69.5172777
18:33:55	0:31:34	70.6258374	63.2842889	76.8195316	75.8813582	72.5172777
18:33:56	0:31:35	71.6258374	67.2842889	77.8195316	74.8813582	77.5172777
18:33:57	0:31:36	71.9258374	70.2842889	76.8195316	74.8813582	77.5172777
18:33:58	0:31:37	72.4258374	70.2842889	75.8195316	74.8813582	80.5172777
18:33:59	0:31:38	72.4258374	70.2842889	74.8195316	74.8813582	81.5172777
18:34:00	0:31:39	72.4258374	71.2842889	74.8195316	74.8813582	81.5172777
18:34:01	0:31:40	72.3258374	71.2842889	71.8195316	73.8813582	83.5172777
18:34:02	0:31:41	72.2258374	71.2842889	70.8195316	73.8813582	84.5172777
18:34:03	0:31:42	72.0258374	72.2842889	70.8195316	73.8813582	84.5172777
18:34:04	0:31:43	71.1258374	71.2842889	68.8195316	73.8813582	81.5172777
18:34:05	0:31:44	71.0258374	71.2842889	68.8195316	73.8813582	81.5172777
18:34:06	0:31:45	71.0258374	71.2842889	68.8195316	73.8813582	81.5172777
18:34:07	0:31:46	71.6258374	71.2842889	68.8195316	74.8813582	81.5172777
18:34:08	0:31:47	71.5258374	71.2842889	68.8195316	72.8813582	81.5172777
18:34:09	0:31:48	71.0258374	69.2842889	68.8195316	71.8813582	81.5172777
18:34:10	0:31:49	71.4258374	68.2842889	68.8195316	72.8813582	81.5172777
18:34:11	0:31:50	70.9258374	67.2842889	66.8195316	72.8813582	80.5172777
18:34:12	0:31:51	70.9258374	67.2842889	66.8195316	72.8813582	80.5172777
18:34:13	0:31:52	70.9258374	67.2842889	65.8195316	74.8813582	80.5172777
18:34:14	0:31:53	70.8258374	68.2842889	65.8195316	74.8813582	80.5172777
18:34:15	0:31:54	71.4258374	69.2842889	64.8195316	74.8813582	80.5172777
18:34:16	0:31:55	71.6258374	69.2842889	64.8195316	74.8813582	80.5172777
18:34:17	0:31:56	71.3258374	69.2842889	64.8195316	74.8813582	76.5172777
18:34:18	0:31:57	70.9258374	69.2842889	65.8195316	73.8813582	74.5172777
18:34:19	0:31:58	70.3258374	68.2842889	66.8195316	73.8813582	72.5172777
18:34:20	0:31:59	70.2258374	67.2842889	66.8195316	73.8813582	69.5172777
18:34:21	0:32:00	70.1258374	67.2842889	66.8195316	73.8813582	68.5172777
18:34:22	0:32:01	70.0258374	66.2842889	67.8195316	73.8813582	67.5172777
18:34:23	0:32:02	69.7258374	64.2842889	68.8195316	73.8813582	67.5172777
18:34:24	0:32:03	69.7258374	65.2842889	68.8195316	72.8813582	68.5172777
18:34:25	0:32:04	69.8258374	65.2842889	68.8195316	72.8813582	68.5172777
18:34:26	0:32:05	70.4258374	67.2842889	68.8195316	70.8813582	69.5172777
18:34:27	0:32:06	70.4258374	67.2842889	68.8195316	70.8813582	69.5172777
18:34:28	0:32:07	69.5258374	67.2842889	67.8195316	69.8813582	67.5172777
18:34:29	0:32:08	70.1258374	68.2842889	67.8195316	69.8813582	67.5172777
18:34:30	0:32:09	70.2258374	68.2842889	66.8195316	68.8813582	67.5172777
18:34:31	0:32:10	70.4258374	69.2842889	66.8195316	68.8813582	66.5172777
18:34:32	0:32:11	70.1258374	69.2842889	66.8195316	68.8813582	65.5172777
18:34:33	0:32:12	70.4258374	69.2842889	66.8195316	68.8813582	64.5172777

18:34:34	0:32:13	70.7258374	73.2842889	67.8195316	68.8813582	63.5172777
18:34:35	0:32:14	70.4258374	73.2842889	68.8195316	68.8813582	63.5172777
18:34:36	0:32:15	69.6258374	73.2842889	68.8195316	67.8813582	62.5172777
18:34:37	0:32:16	68.9258374	68.2842889	68.8195316	67.8813582	63.5172777
18:34:38	0:32:17	68.7258374	67.2842889	69.8195316	67.8813582	64.5172777
18:34:39	0:32:18	68.4258374	67.2842889	69.8195316	67.8813582	64.5172777
18:34:40	0:32:19	68.4258374	67.2842889	69.8195316	67.8813582	68.5172777
18:34:41	0:32:20	68.5258374	66.2842889	69.8195316	68.8813582	70.5172777
18:34:42	0:32:21	68.1258374	66.2842889	69.8195316	68.8813582	71.5172777
18:34:43	0:32:22	68.1258374	65.2842889	69.8195316	68.8813582	71.5172777
18:34:44	0:32:23	67.9258374	65.2842889	69.8195316	68.8813582	71.5172777
18:34:45	0:32:24	68.2258374	63.2842889	69.8195316	69.8813582	71.5172777
18:34:46	0:32:25	68.0258374	62.2842889	70.8195316	69.8813582	70.5172777
18:34:47	0:32:26	68.0258374	62.2842889	70.8195316	71.8813582	70.5172777
18:34:48	0:32:27	67.9258374	62.2842889	70.8195316	71.8813582	71.5172777
18:34:49	0:32:28	68.4258374	62.2842889	73.8195316	71.8813582	72.5172777
18:34:50	0:32:29	68.5258374	62.2842889	73.8195316	71.8813582	72.5172777
18:34:51	0:32:30	68.8258374	62.2842889	74.8195316	69.8813582	72.5172777
18:34:52	0:32:31	69.1258374	62.2842889	74.8195316	69.8813582	71.5172777
18:34:53	0:32:32	69.0258374	61.2842889	74.8195316	69.8813582	69.5172777
18:34:54	0:32:33	68.7258374	62.2842889	73.8195316	68.8813582	69.5172777
18:34:55	0:32:34	68.8258374	62.2842889	73.8195316	68.8813582	68.5172777
18:34:56	0:32:35	68.9258374	62.2842889	72.8195316	70.8813582	67.5172777
18:34:57	0:32:36	68.8258374	62.2842889	72.8195316	70.8813582	67.5172777
18:34:58	0:32:37	68.9258374	62.2842889	72.8195316	70.8813582	67.5172777
18:34:59	0:32:38	69.1258374	62.2842889	73.8195316	70.8813582	68.5172777
18:35:00	0:32:39	69.2258374	62.2842889	73.8195316	70.8813582	69.5172777
18:35:01	0:32:40	69.9258374	62.2842889	73.8195316	69.8813582	69.5172777
18:35:02	0:32:41	70.2258374	62.2842889	72.8195316	69.8813582	69.5172777
18:35:03	0:32:42	70.5258374	62.2842889	72.8195316	68.8813582	69.5172777
18:35:04	0:32:43	70.5258374	62.2842889	70.8195316	68.8813582	70.5172777
18:35:05	0:32:44	70.5258374	62.2842889	69.8195316	68.8813582	70.5172777
18:35:06	0:32:45	70.5258374	62.2842889	69.8195316	68.8813582	70.5172777
18:35:07	0:32:46	70.2258374	63.2842889	69.8195316	68.8813582	70.5172777
18:35:08	0:32:47	71.0258374	63.2842889	67.8195316	73.8813582	70.5172777
18:35:09	0:32:48	70.7258374	63.2842889	67.8195316	75.8813582	70.5172777
18:35:10	0:32:49	70.6258374	64.2842889	67.8195316	76.8813582	70.5172777
18:35:11	0:32:50	70.6258374	64.2842889	68.8195316	77.8813582	70.5172777
18:35:12	0:32:51	70.3258374	63.2842889	67.8195316	78.8813582	71.5172777
18:35:13	0:32:52	70.1258374	63.2842889	67.8195316	79.8813582	71.5172777
18:35:14	0:32:53	69.9258374	63.2842889	67.8195316	79.8813582	71.5172777
18:35:15	0:32:54	70.3258374	63.2842889	67.8195316	81.8813582	73.5172777
18:35:16	0:32:55	70.8258374	63.2842889	67.8195316	70.8258374	74.5172777
18:35:17	0:32:56	70.7258374	64.2842889	67.8195316	70.7258374	74.5172777
18:35:18	0:32:57	70.8258374	64.2842889	68.8195316	70.8258374	75.5172777

18:35:19	0:32:58	71.0258374	64.2842889	68.8195316	71.0258374	75.5172777
18:35:20	0:32:59	69.5258374	65.2842889	68.8195316	78.8813582	75.5172777
18:35:21	0:33:00	69.6258374	67.2842889	68.8195316	76.8813582	75.5172777
18:35:22	0:33:01	69.4258374	67.2842889	69.8195316	75.8813582	73.5172777
18:35:23	0:33:02	69.7258374	67.2842889	70.8195316	75.8813582	72.5172777
18:35:24	0:33:03	69.7258374	67.2842889	70.8195316	76.8813582	71.5172777
18:35:25	0:33:04	69.7258374	67.2842889	71.8195316	76.8813582	72.5172777
18:35:26	0:33:05	69.3258374	66.2842889	71.8195316	76.8813582	71.5172777
18:35:27	0:33:06	69.2258374	66.2842889	71.8195316	76.8813582	71.5172777
18:35:28	0:33:07	68.8258374	66.2842889	70.8195316	76.8813582	71.5172777
18:35:29	0:33:08	68.7258374	66.2842889	69.8195316	75.8813582	72.5172777
18:35:30	0:33:09	68.7258374	66.2842889	69.8195316	74.8813582	72.5172777
18:35:31	0:33:10	68.7258374	66.2842889	68.8195316	72.8813582	71.5172777
18:35:32	0:33:11	68.7258374	66.2842889	68.8195316	69.8813582	70.5172777
18:35:33	0:33:12	68.9258374	67.2842889	68.8195316	68.8813582	70.5172777
18:35:34	0:33:13	72.0258374	68.2842889	68.8195316	68.8813582	70.5172777
18:35:35	0:33:14	73.2258374	68.2842889	68.8195316	68.8813582	71.5172777
18:35:36	0:33:15	73.0258374	69.2842889	69.8195316	68.8813582	71.5172777
18:35:37	0:33:16	73.7258374	69.2842889	69.8195316	68.8813582	71.5172777
18:35:38	0:33:17	73.5258374	68.2842889	69.8195316	68.8813582	71.5172777
18:35:39	0:33:18	73.8258374	68.2842889	69.8195316	68.8813582	74.5172777
18:35:40	0:33:19	73.8258374	68.2842889	69.8195316	68.8813582	73.5172777
18:35:41	0:33:20	71.4258374	68.2842889	68.8195316	68.8813582	73.5172777
18:35:42	0:33:21	71.5258374	68.2842889	68.8195316	67.8813582	72.5172777
18:35:43	0:33:22	72.3258374	68.2842889	68.8195316	67.8813582	72.5172777
18:35:44	0:33:23	71.0258374	68.2842889	68.8195316	67.8813582	72.5172777
18:35:45	0:33:24	70.7258374	68.2842889	69.8195316	67.8813582	71.5172777
18:35:46	0:33:25	70.3258374	68.2842889	69.8195316	67.8813582	71.5172777
18:35:47	0:33:26	69.9258374	68.2842889	69.8195316	66.8813582	71.5172777
18:35:48	0:33:27	69.7258374	68.2842889	69.8195316	66.8813582	70.5172777
18:35:49	0:33:28	69.5258374	68.2842889	69.8195316	66.8813582	70.5172777
18:35:50	0:33:29	69.6258374	68.2842889	69.8195316	66.8813582	70.5172777
18:35:51	0:33:30	69.5258374	68.2842889	69.8195316	65.8813582	70.5172777
18:35:52	0:33:31	69.5258374	68.2842889	69.8195316	65.8813582	70.5172777
18:35:53	0:33:32	69.7258374	68.2842889	69.8195316	66.8813582	71.5172777
18:35:54	0:33:33	69.6258374	69.2842889	69.8195316	66.8813582	70.5172777
18:35:55	0:33:34	69.9258374	69.2842889	69.8195316	66.8813582	70.5172777
18:35:56	0:33:35	70.4258374	69.2842889	71.8195316	66.8813582	71.5172777
18:35:57	0:33:36	71.0258374	69.2842889	71.8195316	68.8813582	71.5172777
18:35:58	0:33:37	71.9258374	69.2842889	72.8195316	68.8813582	71.5172777
18:35:59	0:33:38	72.1258374	69.2842889	72.8195316	70.8813582	71.5172777
18:36:00	0:33:39	72.1258374	69.2842889	72.8195316	70.8813582	71.5172777
18:36:01	0:33:40	72.1258374	68.2842889	74.8195316	70.8813582	71.5172777
18:36:02	0:33:41	72.2258374	72.2842889	75.8195316	68.8813582	71.5172777
18:36:03	0:33:42	72.0258374	74.2842889	75.8195316	68.8813582	71.5172777

18:36:04	0:33:43	71.8258374	77.2842889	75.8195316	68.8813582	72.5172777
18:36:05	0:33:44	71.2258374	78.2842889	72.8195316	68.8813582	71.5172777
18:36:06	0:33:45	70.6258374	78.2842889	71.8195316	68.8813582	71.5172777
18:36:07	0:33:46	70.2258374	76.2842889	70.8195316	68.8813582	71.5172777
18:36:08	0:33:47	69.0258374	67.2842889	69.8195316	68.8813582	70.5172777
18:36:09	0:33:48	69.1258374	67.2842889	69.8195316	68.8813582	70.5172777
18:36:10	0:33:49	69.6258374	66.2842889	69.8195316	71.8813582	69.5172777
18:36:11	0:33:50	70.0258374	66.2842889	70.8195316	72.8813582	69.5172777
18:36:12	0:33:51	69.9258374	66.2842889	70.8195316	72.8813582	67.5172777
18:36:13	0:33:52	70.2258374	66.2842889	70.8195316	72.8813582	68.5172777
18:36:14	0:33:53	69.8258374	66.2842889	70.8195316	72.8813582	68.5172777
18:36:15	0:33:54	69.6258374	65.2842889	69.8195316	71.8813582	68.5172777
18:36:16	0:33:55	70.7258374	64.2842889	69.8195316	71.8813582	69.5172777
18:36:17	0:33:56	71.3258374	65.2842889	69.8195316	71.8813582	70.5172777
18:36:18	0:33:57	72.2258374	65.2842889	69.8195316	71.8813582	73.5172777
18:36:19	0:33:58	72.3258374	66.2842889	69.8195316	70.8813582	72.5172777
18:36:20	0:33:59	73.1258374	66.2842889	67.8195316	69.8813582	71.5172777
18:36:21	0:34:00	73.5258374	66.2842889	67.8195316	69.8813582	71.5172777
18:36:22	0:34:01	74.0258374	68.2842889	66.8195316	70.8813582	71.5172777
18:36:23	0:34:02	74.0258374	68.2842889	66.8195316	70.8813582	71.5172777
18:36:24	0:34:03	73.6258374	67.2842889	66.8195316	69.8813582	71.5172777
18:36:25	0:34:04	73.2258374	64.2842889	66.8195316	69.8813582	71.5172777
18:36:26	0:34:05	73.2258374	64.2842889	66.8195316	69.8813582	71.5172777
18:36:27	0:34:06	72.9258374	64.2842889	67.8195316	68.8813582	70.5172777
18:36:28	0:34:07	73.0258374	64.2842889	67.8195316	68.8813582	70.5172777
18:36:29	0:34:08	73.4258374	64.2842889	67.8195316	68.8813582	70.5172777
18:36:30	0:34:09	73.5258374	68.2842889	67.8195316	68.8813582	70.5172777
18:36:31	0:34:10	73.4258374	67.2842889	67.8195316	69.8813582	70.5172777
18:36:32	0:34:11	73.4258374	67.2842889	67.8195316	69.8813582	71.5172777
18:36:33	0:34:12	73.2258374	65.2842889	67.8195316	68.8813582	72.5172777
18:36:34	0:34:13	73.3258374	64.2842889	68.8195316	68.8813582	74.5172777
18:36:35	0:34:14	73.0258374	64.2842889	68.8195316	68.8813582	74.5172777
18:36:36	0:34:15	72.6258374	64.2842889	68.8195316	67.8813582	76.5172777
18:36:37	0:34:16	72.3258374	64.2842889	68.8195316	67.8813582	75.5172777
18:36:38	0:34:17	72.3258374	64.2842889	68.8195316	67.8813582	76.5172777
18:36:39	0:34:18	72.7258374	64.2842889	69.8195316	67.8813582	76.5172777
18:36:40	0:34:19	73.1258374	63.2842889	69.8195316	67.8813582	77.5172777
18:36:41	0:34:20	73.5258374	61.2842889	69.8195316	66.8813582	78.5172777
18:36:42	0:34:21	73.8258374	61.2842889	69.8195316	67.8813582	79.5172777
18:36:43	0:34:22	73.9258374	61.2842889	69.8195316	68.8813582	80.5172777
18:36:44	0:34:23	74.0258374	60.2842889	69.8195316	68.8813582	80.5172777
18:36:45	0:34:24	74.1258374	60.2842889	69.8195316	68.8813582	79.5172777
18:36:46	0:34:25	74.4258374	60.2842889	69.8195316	68.8813582	79.5172777
18:36:47	0:34:26	74.7258374	60.2842889	70.8195316	69.8813582	78.5172777
18:36:48	0:34:27	75.0258374	60.2842889	71.8195316	69.8813582	78.5172777

18:36:49	0:34:28	74.1258374	60.2842889	71.8195316	69.8813582	76.5172777
18:36:50	0:34:29	73.9258374	60.2842889	71.8195316	70.8813582	75.5172777
18:36:51	0:34:30	74.3258374	60.2842889	76.8195316	71.8813582	75.5172777
18:36:52	0:34:31	74.1258374	60.2842889	76.8195316	71.8813582	74.5172777
18:36:53	0:34:32	74.0258374	60.2842889	76.8195316	71.8813582	74.5172777
18:36:54	0:34:33	74.0258374	60.2842889	76.8195316	71.8813582	74.5172777
18:36:55	0:34:34	74.1258374	60.2842889	77.8195316	71.8813582	76.5172777
18:36:56	0:34:35	74.3258374	60.2842889	77.8195316	71.8813582	76.5172777
18:36:57	0:34:36	74.0258374	60.2842889	77.8195316	71.8813582	73.5172777
18:36:58	0:34:37	73.8258374	61.2842889	77.8195316	70.8813582	71.5172777
18:36:59	0:34:38	73.3258374	61.2842889	76.8195316	68.8813582	70.5172777
18:37:00	0:34:39	72.8258374	61.2842889	76.8195316	67.8813582	69.5172777
18:37:01	0:34:40	71.6258374	68.2842889	75.8195316	67.8813582	70.5172777
18:37:02	0:34:41	70.7258374	68.2842889	73.8195316	67.8813582	71.5172777
18:37:03	0:34:42	70.9258374	68.2842889	71.8195316	67.8813582	74.5172777
18:37:04	0:34:43	71.0258374	68.2842889	70.8195316	67.8813582	77.5172777
18:37:05	0:34:44	70.5258374	68.2842889	70.8195316	68.8813582	77.5172777
18:37:06	0:34:45	70.7258374	68.2842889	70.8195316	68.8813582	76.5172777
18:37:07	0:34:46	70.6258374	68.2842889	70.8195316	68.8813582	75.5172777
18:37:08	0:34:47	69.8258374	69.2842889	70.8195316	68.8813582	75.5172777
18:37:09	0:34:48	69.5258374	68.2842889	70.8195316	67.8813582	75.5172777
18:37:10	0:34:49	69.2258374	67.2842889	71.8195316	67.8813582	75.5172777
18:37:11	0:34:50	69.5258374	67.2842889	72.8195316	67.8813582	75.5172777
18:37:12	0:34:51	70.5258374	67.2842889	72.8195316	67.8813582	75.5172777
18:37:13	0:34:52	71.1258374	67.2842889	72.8195316	67.8813582	76.5172777
18:37:14	0:34:53	71.1258374	69.2842889	72.8195316	66.8813582	76.5172777
18:37:15	0:34:54	71.3258374	69.2842889	72.8195316	66.8813582	77.5172777
18:37:16	0:34:55	71.8258374	69.2842889	73.8195316	66.8813582	77.5172777
18:37:17	0:34:56	72.3258374	69.2842889	75.8195316	66.8813582	78.5172777
18:37:18	0:34:57	72.2258374	69.2842889	75.8195316	66.8813582	77.5172777
18:37:19	0:34:58	71.9258374	69.2842889	75.8195316	66.8813582	77.5172777
18:37:20	0:34:59	71.7258374	69.2842889	75.8195316	66.8813582	77.5172777
18:37:21	0:35:00	71.6258374	69.2842889	75.8195316	66.8813582	76.5172777
18:37:22	0:35:01	71.3258374	69.2842889	76.8195316	66.8813582	74.5172777
18:37:23	0:35:02	71.5258374	69.2842889	76.8195316	66.8813582	74.5172777
18:37:24	0:35:03	71.3258374	69.2842889	76.8195316	66.8813582	73.5172777
18:37:25	0:35:04	71.1258374	71.2842889	76.8195316	66.8813582	72.5172777
18:37:26	0:35:05	71.1258374	71.2842889	74.8195316	66.8813582	73.5172777
18:37:27	0:35:06	71.3258374	72.2842889	74.8195316	66.8813582	73.5172777
18:37:28	0:35:07	71.6258374	72.2842889	74.8195316	66.8813582	73.5172777
18:37:29	0:35:08	71.8258374	74.2842889	73.8195316	66.8813582	72.5172777
18:37:30	0:35:09	71.4258374	74.2842889	72.8195316	65.8813582	72.5172777
18:37:31	0:35:10	70.9258374	74.2842889	72.8195316	65.8813582	71.5172777
18:37:32	0:35:11	70.4258374	74.2842889	72.8195316	64.8813582	71.5172777
18:37:33	0:35:12	70.5258374	74.2842889	72.8195316	64.8813582	71.5172777

18:37:34	0:35:13	70.3258374	74.2842889	72.8195316	64.8813582	70.5172777
18:37:35	0:35:14	70.0258374	74.2842889	69.8195316	63.8813582	70.5172777
18:37:36	0:35:15	70.2258374	77.2842889	68.8195316	63.8813582	70.5172777
18:37:37	0:35:16	71.2258374	71.2258374	68.8195316	63.8813582	70.5172777
18:37:38	0:35:17	71.5258374	84.2842889	68.8195316	63.8813582	70.5172777
18:37:39	0:35:18	71.7258374	84.2842889	68.8195316	63.8813582	71.5172777
18:37:40	0:35:19	71.6258374	84.2842889	69.8195316	63.8813582	71.5172777
18:37:41	0:35:20	72.0258374	84.2842889	69.8195316	64.8813582	71.5172777
18:37:42	0:35:21	72.1258374	84.2842889	69.8195316	68.8813582	71.5172777
18:37:43	0:35:22	72.3258374	84.2842889	69.8195316	68.8813582	72.5172777
18:37:44	0:35:23	72.8258374	85.2842889	70.8195316	67.8813582	72.5172777
18:37:45	0:35:24	73.2258374	85.2842889	71.8195316	67.8813582	73.5172777
18:37:46	0:35:25	73.4258374	85.2842889	71.8195316	67.8813582	73.5172777
18:37:47	0:35:26	73.5258374	85.2842889	71.8195316	68.8813582	73.5172777
18:37:48	0:35:27	73.5258374	85.2842889	71.8195316	68.8813582	73.5172777
18:37:49	0:35:28	73.4258374	85.2842889	70.8195316	70.8813582	73.5172777
18:37:50	0:35:29	73.4258374	85.2842889	71.8195316	71.8813582	73.5172777
18:37:51	0:35:30	73.2258374	85.2842889	71.8195316	72.8813582	73.5172777
18:37:52	0:35:31	73.4258374	84.2842889	73.8195316	72.8813582	74.5172777
18:37:53	0:35:32	73.3258374	83.2842889	72.8195316	72.8813582	75.5172777
18:37:54	0:35:33	72.3258374	75.2842889	71.8195316	72.8813582	75.5172777
18:37:55	0:35:34	72.6258374	75.2842889	71.8195316	73.8813582	75.5172777
18:37:56	0:35:35	73.6258374	75.2842889	71.8195316	73.8813582	76.5172777
18:37:57	0:35:36	73.8258374	74.2842889	71.8195316	73.8813582	76.5172777
18:37:58	0:35:37	74.1258374	73.2842889	71.8195316	74.8813582	76.5172777
18:37:59	0:35:38	74.0258374	73.2842889	70.8195316	74.8813582	76.5172777
18:38:00	0:35:39	74.3258374	73.2842889	70.8195316	74.8813582	76.5172777
18:38:01	0:35:40	74.6258374	72.2842889	70.8195316	74.8813582	75.5172777
18:38:02	0:35:41	74.9258374	72.2842889	70.8195316	74.8813582	75.5172777
18:38:03	0:35:42	75.1258374	71.2842889	70.8195316	75.8813582	74.5172777
18:38:04	0:35:43	75.4258374	70.2842889	75.8195316	75.8813582	74.5172777
18:38:05	0:35:44	75.0258374	70.2842889	75.8195316	75.8813582	73.5172777
18:38:06	0:35:45	74.5258374	70.2842889	75.8195316	74.8813582	73.5172777
18:38:07	0:35:46	74.3258374	70.2842889	74.8195316	75.8813582	73.5172777
18:38:08	0:35:47	74.5258374	70.2842889	73.8195316	75.8813582	74.5172777
18:38:09	0:35:48	74.4258374	70.2842889	72.8195316	75.8813582	73.5172777
18:38:10	0:35:49	74.5258374	70.2842889	72.8195316	74.8813582	73.5172777
18:38:11	0:35:50	74.3258374	69.2842889	71.8195316	72.8813582	74.5172777
18:38:12	0:35:51	74.0258374	69.2842889	70.8195316	71.8813582	73.5172777
18:38:13	0:35:52	74.0258374	69.2842889	70.8195316	71.8813582	72.5172777
18:38:14	0:35:53	74.2258374	69.2842889	71.8195316	70.8813582	72.5172777
18:38:15	0:35:54	74.8258374	69.2842889	73.8195316	70.8813582	72.5172777
18:38:16	0:35:55	74.8258374	69.2842889	73.8195316	71.8813582	71.5172777
18:38:17	0:35:56	75.2258374	69.2842889	73.8195316	71.8813582	71.5172777
18:38:18	0:35:57	75.1258374	69.2842889	73.8195316	71.8813582	72.5172777

18:38:19	0:35:58	75.1258374	69.2842889	72.8195316	71.8813582	73.5172777
18:38:20	0:35:59	75.2258374	69.2842889	72.8195316	72.8813582	73.5172777
18:38:21	0:36:00	74.9258374	69.2842889	71.8195316	72.8813582	73.5172777
18:38:22	0:36:01	74.7258374	69.2842889	71.8195316	71.8813582	72.5172777
18:38:23	0:36:02	74.7258374	69.2842889	71.8195316	71.8813582	72.5172777
18:38:24	0:36:03	75.5258374	69.2842889	71.8195316	71.8813582	72.5172777
18:38:25	0:36:04	75.3258374	69.2842889	71.8195316	72.8813582	72.5172777
18:38:26	0:36:05	74.7258374	69.2842889	71.8195316	72.8813582	73.5172777
18:38:27	0:36:06	74.0258374	69.2842889	71.8195316	72.8813582	73.5172777
18:38:28	0:36:07	73.9258374	68.2842889	71.8195316	73.8813582	74.5172777
18:38:29	0:36:08	73.6258374	67.2842889	71.8195316	73.8813582	73.5172777
18:38:30	0:36:09	73.7258374	67.2842889	72.8195316	73.8813582	73.5172777
18:38:31	0:36:10	74.3258374	68.2842889	72.8195316	76.8813582	73.5172777
18:38:32	0:36:11	74.3258374	68.2842889	72.8195316	76.8813582	72.5172777
18:38:33	0:36:12	74.3258374	68.2842889	71.8195316	77.8813582	72.5172777
18:38:34	0:36:13	74.4258374	68.2842889	71.8195316	77.8813582	72.5172777
18:38:35	0:36:14	74.5258374	67.2842889	71.8195316	78.8813582	72.5172777
18:38:36	0:36:15	74.5258374	67.2842889	71.8195316	78.8813582	72.5172777
18:38:37	0:36:16	74.6258374	67.2842889	72.8195316	77.8813582	71.5172777
18:38:38	0:36:17	73.8258374	67.2842889	73.8195316	76.8813582	70.5172777
18:38:39	0:36:18	73.4258374	67.2842889	73.8195316	75.8813582	70.5172777
18:38:40	0:36:19	73.6258374	67.2842889	73.8195316	75.8813582	70.5172777
18:38:41	0:36:20	73.7258374	67.2842889	73.8195316	75.8813582	70.5172777
18:38:42	0:36:21	73.7258374	67.2842889	73.8195316	75.8813582	70.5172777
18:38:43	0:36:22	72.9258374	66.2842889	73.8195316	76.8813582	70.5172777
18:38:44	0:36:23	72.4258374	66.2842889	70.8195316	75.8813582	70.5172777
18:38:45	0:36:24	72.0258374	66.2842889	70.8195316	75.8813582	70.5172777
18:38:46	0:36:25	72.0258374	66.2842889	71.8195316	75.8813582	71.5172777
18:38:47	0:36:26	72.0258374	66.2842889	71.8195316	76.8813582	71.5172777
18:38:48	0:36:27	72.0258374	66.2842889	71.8195316	78.8813582	71.5172777
18:38:49	0:36:28	71.5258374	66.2842889	71.8195316	79.8813582	71.5172777
18:38:50	0:36:29	71.6258374	66.2842889	73.8195316	80.8813582	71.5172777
18:38:51	0:36:30	71.1258374	66.2842889	75.8195316	81.8813582	70.5172777
18:38:52	0:36:31	71.2258374	67.2842889	75.8195316	81.8813582	71.5172777
18:38:53	0:36:32	71.1258374	67.2842889	75.8195316	80.8813582	73.5172777
18:38:54	0:36:33	71.2258374	66.2842889	75.8195316	80.8813582	74.5172777
18:38:55	0:36:34	71.0258374	65.2842889	75.8195316	79.8813582	74.5172777
18:38:56	0:36:35	70.7258374	65.2842889	74.8195316	76.8813582	74.5172777
18:38:57	0:36:36	70.3258374	65.2842889	73.8195316	75.8813582	71.5172777
18:38:58	0:36:37	70.4258374	64.2842889	72.8195316	74.8813582	69.5172777
18:38:59	0:36:38	70.2258374	63.2842889	70.8195316	73.8813582	68.5172777
18:39:00	0:36:39	70.5258374	63.2842889	70.8195316	71.8813582	67.5172777
18:39:01	0:36:40	71.0258374	68.2842889	70.8195316	68.8813582	67.5172777
18:39:02	0:36:41	71.0258374	68.2842889	70.8195316	67.8813582	67.5172777
18:39:03	0:36:42	70.6258374	69.2842889	69.8195316	66.8813582	67.5172777

18:39:04	0:36:43	70.9258374	70.2842889	69.8195316	66.8813582	68.5172777
18:39:05	0:36:44	70.8258374	69.2842889	69.8195316	66.8813582	68.5172777
18:39:06	0:36:45	70.0258374	68.2842889	69.8195316	67.8813582	67.5172777
18:39:07	0:36:46	69.2258374	67.2842889	68.8195316	70.8813582	67.5172777
18:39:08	0:36:47	69.0258374	67.2842889	68.8195316	70.8813582	66.5172777
18:39:09	0:36:48	68.8258374	67.2842889	68.8195316	70.8813582	67.5172777
18:39:10	0:36:49	68.9258374	67.2842889	68.8195316	70.8813582	68.5172777
18:39:11	0:36:50	68.9258374	66.2842889	68.8195316	69.8813582	69.5172777
18:39:12	0:36:51	68.8258374	65.2842889	68.8195316	69.8813582	72.5172777
18:39:13	0:36:52	69.3258374	64.2842889	68.8195316	68.8813582	75.5172777
18:39:14	0:36:53	69.4258374	64.2842889	68.8195316	68.8813582	77.5172777
18:39:15	0:36:54	69.1258374	64.2842889	69.8195316	67.8813582	77.5172777
18:39:16	0:36:55	68.9258374	63.2842889	69.8195316	67.8813582	77.5172777
18:39:17	0:36:56	68.8258374	63.2842889	69.8195316	67.8813582	77.5172777
18:39:18	0:36:57	69.1258374	63.2842889	70.8195316	71.8813582	77.5172777
18:39:19	0:36:58	69.7258374	63.2842889	71.8195316	73.8813582	78.5172777
18:39:20	0:36:59	69.8258374	63.2842889	71.8195316	76.8813582	78.5172777
18:39:21	0:37:00	69.6258374	63.2842889	71.8195316	78.8813582	77.5172777
18:39:22	0:37:01	69.6258374	63.2842889	70.8195316	77.8813582	77.5172777
18:39:23	0:37:02	69.4258374	63.2842889	70.8195316	75.8813582	77.5172777
18:39:24	0:37:03	69.4258374	65.2842889	70.8195316	75.8813582	74.5172777
18:39:25	0:37:04	69.6258374	67.2842889	70.8195316	75.8813582	72.5172777
18:39:26	0:37:05	69.6258374	68.2842889	69.8195316	75.8813582	69.5172777
18:39:27	0:37:06	69.9258374	68.2842889	69.8195316	74.8813582	68.5172777
18:39:28	0:37:07	70.3258374	69.2842889	70.8195316	74.8813582	68.5172777
18:39:29	0:37:08	70.4258374	69.2842889	70.8195316	75.8813582	68.5172777
18:39:30	0:37:09	71.1258374	68.2842889	71.8195316	76.8813582	69.5172777
18:39:31	0:37:10	70.9258374	68.2842889	71.8195316	76.8813582	69.5172777
18:39:32	0:37:11	70.9258374	68.2842889	71.8195316	76.8813582	69.5172777
18:39:33	0:37:12	71.5258374	68.2842889	72.8195316	76.8813582	69.5172777
18:39:34	0:37:13	72.4258374	68.2842889	73.8195316	76.8813582	70.5172777
18:39:35	0:37:14	73.1258374	68.2842889	74.8195316	76.8813582	70.5172777
18:39:36	0:37:15	73.1258374	68.2842889	74.8195316	76.8813582	70.5172777
18:39:37	0:37:16	72.7258374	66.2842889	75.8195316	77.8813582	72.5172777
18:39:38	0:37:17	72.7258374	66.2842889	75.8195316	78.8813582	73.5172777
18:39:39	0:37:18	72.9258374	65.2842889	75.8195316	78.8813582	73.5172777
18:39:40	0:37:19	73.1258374	65.2842889	76.8195316	79.8813582	73.5172777
18:39:41	0:37:20	72.5258374	65.2842889	76.8195316	78.8813582	72.5172777
18:39:42	0:37:21	72.2258374	65.2842889	76.8195316	76.8813582	71.5172777
18:39:43	0:37:22	71.6258374	65.2842889	75.8195316	75.8813582	72.5172777
18:39:44	0:37:23	71.4258374	64.2842889	74.8195316	74.8813582	75.5172777
18:39:45	0:37:24	71.5258374	64.2842889	73.8195316	74.8813582	76.5172777
18:39:46	0:37:25	71.5258374	64.2842889	73.8195316	74.8813582	77.5172777
18:39:47	0:37:26	71.6258374	64.2842889	73.8195316	74.8813582	77.5172777
18:39:48	0:37:27	72.0258374	64.2842889	73.8195316	74.8813582	77.5172777

18:39:49	0:37:28	72.1258374	64.2842889	72.8195316	74.8813582	77.5172777
18:39:50	0:37:29	72.5258374	64.2842889	72.8195316	74.8813582	77.5172777
18:39:51	0:37:30	72.8258374	63.2842889	71.8195316	75.8813582	76.5172777
18:39:52	0:37:31	73.1258374	63.2842889	71.8195316	76.8813582	76.5172777
18:39:53	0:37:32	73.1258374	63.2842889	71.8195316	79.8813582	76.5172777
18:39:54	0:37:33	72.6258374	63.2842889	68.8195316	80.8813582	75.5172777
18:39:55	0:37:34	72.2258374	63.2842889	67.8195316	81.8813582	74.5172777
18:39:56	0:37:35	72.4258374	63.2842889	67.8195316	81.8813582	73.5172777
18:39:57	0:37:36	72.9258374	64.2842889	66.8195316	81.8813582	72.5172777
18:39:58	0:37:37	73.1258374	64.2842889	66.8195316	81.8813582	73.5172777
18:39:59	0:37:38	72.1258374	64.2842889	67.8195316	77.8813582	73.5172777
18:40:00	0:37:39	71.8258374	64.2842889	67.8195316	74.8813582	74.5172777
18:40:01	0:37:40	71.5258374	63.2842889	67.8195316	72.8813582	74.5172777
18:40:02	0:37:41	71.4258374	63.2842889	69.8195316	71.8813582	73.5172777
18:40:03	0:37:42	71.6258374	63.2842889	69.8195316	71.8813582	72.5172777
18:40:04	0:37:43	71.8258374	63.2842889	69.8195316	72.8813582	71.5172777
18:40:05	0:37:44	72.0258374	63.2842889	69.8195316	72.8813582	71.5172777
18:40:06	0:37:45	71.9258374	64.2842889	69.8195316	72.8813582	71.5172777
18:40:07	0:37:46	71.2258374	64.2842889	69.8195316	73.8813582	71.5172777
18:40:08	0:37:47	70.5258374	64.2842889	69.8195316	73.8813582	71.5172777
18:40:09	0:37:48	69.6258374	63.2842889	69.8195316	71.8813582	71.5172777
18:40:10	0:37:49	68.8258374	63.2842889	69.8195316	70.8813582	71.5172777
18:40:11	0:37:50	68.3258374	63.2842889	69.8195316	69.8813582	71.5172777
18:40:12	0:37:51	68.2258374	64.2842889	69.8195316	68.8813582	72.5172777
18:40:13	0:37:52	68.1258374	64.2842889	69.8195316	69.8813582	72.5172777
18:40:14	0:37:53	68.4258374	64.2842889	69.8195316	69.8813582	76.5172777
18:40:15	0:37:54	68.1258374	64.2842889	69.8195316	69.8813582	76.5172777
18:40:16	0:37:55	67.9258374	64.2842889	70.8195316	70.8813582	73.5172777
18:40:17	0:37:56	68.2258374	64.2842889	70.8195316	70.8813582	73.5172777
18:40:18	0:37:57	68.2258374	64.2842889	70.8195316	70.8813582	72.5172777
18:40:19	0:37:58	68.1258374	64.2842889	71.8195316	69.8813582	72.5172777
18:40:20	0:37:59	67.8258374	64.2842889	71.8195316	69.8813582	70.5172777
18:40:21	0:38:00	68.1258374	65.2842889	70.8195316	70.8813582	70.5172777
18:40:22	0:38:01	68.0258374	65.2842889	70.8195316	69.8813582	72.5172777
18:40:23	0:38:02	68.5258374	66.2842889	70.8195316	69.8813582	72.5172777
18:40:24	0:38:03	69.0258374	67.2842889	70.8195316	69.8813582	72.5172777
18:40:25	0:38:04	69.3258374	67.2842889	70.8195316	69.8813582	71.5172777
18:40:26	0:38:05	70.3258374	68.2842889	72.8195316	69.8813582	69.5172777
18:40:27	0:38:06	70.9258374	68.2842889	72.8195316	69.8813582	69.5172777
18:40:28	0:38:07	71.3258374	68.2842889	72.8195316	69.8813582	69.5172777
18:40:29	0:38:08	71.2258374	68.2842889	72.8195316	69.8813582	68.5172777
18:40:30	0:38:09	71.2258374	68.2842889	72.8195316	69.8813582	68.5172777
18:40:31	0:38:10	71.5258374	68.2842889	72.8195316	69.8813582	69.5172777
18:40:32	0:38:11	71.5258374	68.2842889	73.8195316	68.8813582	69.5172777
18:40:33	0:38:12	71.2258374	67.2842889	74.8195316	68.8813582	69.5172777

18:40:34	0:38:13	69.8258374	65.2842889	73.8195316	68.8813582	70.5172777
18:40:35	0:38:14	69.4258374	65.2842889	73.8195316	69.8813582	70.5172777
18:40:36	0:38:15	69.3258374	64.2842889	72.8195316	70.8813582	71.5172777
18:40:37	0:38:16	70.2258374	63.2842889	72.8195316	71.8813582	71.5172777
18:40:38	0:38:17	70.4258374	62.2842889	73.8195316	71.8813582	71.5172777
18:40:39	0:38:18	70.3258374	62.2842889	73.8195316	72.8813582	71.5172777
18:40:40	0:38:19	70.5258374	62.2842889	75.8195316	73.8813582	72.5172777
18:40:41	0:38:20	70.2258374	62.2842889	75.8195316	73.8813582	72.5172777
18:40:42	0:38:21	70.5258374	62.2842889	73.8195316	73.8813582	72.5172777
18:40:43	0:38:22	70.8258374	63.2842889	72.8195316	71.8813582	74.5172777
18:40:44	0:38:23	71.1258374	62.2842889	71.8195316	72.8813582	74.5172777
18:40:45	0:38:24	71.1258374	62.2842889	71.8195316	72.8813582	74.5172777
18:40:46	0:38:25	70.7258374	62.2842889	71.8195316	72.8813582	73.5172777
18:40:47	0:38:26	69.8258374	62.2842889	71.8195316	72.8813582	72.5172777
18:40:48	0:38:27	68.6258374	62.2842889	71.8195316	71.8813582	71.5172777
18:40:49	0:38:28	69.0258374	62.2842889	71.8195316	70.8813582	71.5172777
18:40:50	0:38:29	69.1258374	62.2842889	71.8195316	70.8813582	71.5172777
18:40:51	0:38:30	69.3258374	62.2842889	71.8195316	70.8813582	71.5172777
18:40:52	0:38:31	69.4258374	62.2842889	70.8195316	71.8813582	70.5172777
18:40:53	0:38:32	69.4258374	62.2842889	71.8195316	71.8813582	70.5172777
18:40:54	0:38:33	69.4258374	62.2842889	71.8195316	71.8813582	70.5172777
18:40:55	0:38:34	69.3258374	62.2842889	72.8195316	71.8813582	69.5172777
18:40:56	0:38:35	69.4258374	62.2842889	72.8195316	71.8813582	69.5172777
18:40:57	0:38:36	69.1258374	62.2842889	72.8195316	71.8813582	69.5172777
18:40:58	0:38:37	69.2258374	62.2842889	72.8195316	71.8813582	69.5172777
18:40:59	0:38:38	69.1258374	62.2842889	72.8195316	71.8813582	67.5172777
18:41:00	0:38:39	69.2258374	62.2842889	72.8195316	71.8813582	67.5172777
18:41:01	0:38:40	69.2258374	62.2842889	71.8195316	70.8813582	68.5172777
18:41:02	0:38:41	68.9258374	62.2842889	71.8195316	68.8813582	69.5172777
18:41:03	0:38:42	69.4258374	62.2842889	72.8195316	67.8813582	69.5172777
18:41:04	0:38:43	69.9258374	62.2842889	72.8195316	67.8813582	71.5172777
18:41:05	0:38:44	70.0258374	62.2842889	73.8195316	68.8813582	72.5172777
18:41:06	0:38:45	70.1258374	62.2842889	72.8195316	68.8813582	75.5172777
18:41:07	0:38:46	70.2258374	62.2842889	72.8195316	68.8813582	75.5172777
18:41:08	0:38:47	70.9258374	63.2842889	73.8195316	69.8813582	77.5172777
18:41:09	0:38:48	71.7258374	64.2842889	73.8195316	72.8813582	79.5172777
18:41:10	0:38:49	72.3258374	64.2842889	74.8195316	72.8813582	79.5172777
18:41:11	0:38:50	72.6258374	64.2842889	74.8195316	72.8813582	79.5172777
18:41:12	0:38:51	73.5258374	64.2842889	74.8195316	72.8813582	79.5172777
18:41:13	0:38:52	73.9258374	64.2842889	74.8195316	72.8813582	77.5172777
18:41:14	0:38:53	73.7258374	64.2842889	73.8195316	72.8813582	74.5172777
18:41:15	0:38:54	73.4258374	65.2842889	73.8195316	71.8813582	72.5172777
18:41:16	0:38:55	72.9258374	65.2842889	73.8195316	70.8813582	70.5172777
18:41:17	0:38:56	72.1258374	65.2842889	73.8195316	68.8813582	70.5172777
18:41:18	0:38:57	72.0258374	70.2842889	73.8195316	68.8813582	70.5172777

18:41:19	0:38:58	72.5258374	78.2842889	72.8195316	68.8813582	70.5172777
18:41:20	0:38:59	72.4258374	78.2842889	72.8195316	68.8813582	71.5172777
18:41:21	0:39:00	73.1258374	73.1258374	72.8195316	68.8813582	72.5172777
18:41:22	0:39:01	73.1258374	73.1258374	71.8195316	68.8813582	72.5172777
18:41:23	0:39:02	72.9258374	72.9258374	71.8195316	68.8813582	73.5172777
18:41:24	0:39:03	72.9258374	72.9258374	71.8195316	68.8813582	73.5172777
18:41:25	0:39:04	72.6258374	72.6258374	71.8195316	68.8813582	72.5172777
18:41:26	0:39:05	75.3258374	75.3258374	72.8195316	71.8813582	72.5172777
18:41:27	0:39:06	77.0258374	77.0258374	74.8195316	72.8813582	70.5172777
18:41:28	0:39:07	77.9258374	89.2842889	74.8195316	72.8813582	70.5172777
18:41:29	0:39:08	75.7258374	75.7258374	76.8195316	71.8813582	70.5172777
18:41:30	0:39:09	75.1258374	75.1258374	76.8195316	70.8813582	70.5172777
18:41:31	0:39:10	74.3258374	74.3258374	76.8195316	71.8813582	70.5172777
18:41:32	0:39:11	74.6258374	74.6258374	76.8195316	71.8813582	69.5172777
18:41:33	0:39:12	74.3258374	74.3258374	75.8195316	70.8813582	69.5172777
18:41:34	0:39:13	73.9258374	73.9258374	75.8195316	70.8813582	69.5172777
18:41:35	0:39:14	73.2258374	83.2842889	75.8195316	70.8813582	70.5172777
18:41:36	0:39:15	73.0258374	81.2842889	74.8195316	70.8813582	70.5172777
18:41:37	0:39:16	72.7258374	80.2842889	74.8195316	70.8813582	70.5172777
18:41:38	0:39:17	71.9258374	80.2842889	73.8195316	70.8813582	69.5172777
18:41:39	0:39:18	71.5258374	81.2842889	73.8195316	69.8813582	69.5172777
18:41:40	0:39:19	71.6258374	84.2842889	73.8195316	69.8813582	70.5172777
18:41:41	0:39:20	72.0258374	72.0258374	74.8195316	69.8813582	70.5172777
18:41:42	0:39:21	72.0258374	72.0258374	74.8195316	68.8813582	70.5172777
18:41:43	0:39:22	71.9258374	71.9258374	75.8195316	68.8813582	70.5172777
18:41:44	0:39:23	73.0258374	73.0258374	75.8195316	68.8813582	71.5172777
18:41:45	0:39:24	73.2258374	73.2258374	75.8195316	69.8813582	71.5172777
18:41:46	0:39:25	73.5258374	73.5258374	74.8195316	68.8813582	73.5172777
18:41:47	0:39:26	74.0258374	74.0258374	74.8195316	69.8813582	74.5172777
18:41:48	0:39:27	74.1258374	74.1258374	74.8195316	69.8813582	75.5172777
18:41:49	0:39:28	74.1258374	74.1258374	75.8195316	69.8813582	75.5172777
18:41:50	0:39:29	75.4258374	75.4258374	75.8195316	69.8813582	77.5172777
18:41:51	0:39:30	76.4258374	76.4258374	75.8195316	69.8813582	78.5172777
18:41:52	0:39:31	77.2258374	77.2258374	75.8195316	70.8813582	78.5172777
18:41:53	0:39:32	77.2258374	77.2258374	74.8195316	71.8813582	78.5172777
18:41:54	0:39:33	77.2258374	77.2258374	73.8195316	71.8813582	76.5172777
18:41:55	0:39:34	76.1258374	76.1258374	73.8195316	72.8813582	77.5172777
18:41:56	0:39:35	75.8258374	75.8258374	73.8195316	73.8813582	78.5172777
18:41:57	0:39:36	75.6258374	75.6258374	74.8195316	73.8813582	78.5172777
18:41:58	0:39:37	75.1258374	75.1258374	73.8195316	73.8813582	77.5172777
18:41:59	0:39:38	75.1258374	75.1258374	73.8195316	73.8813582	77.5172777
18:42:00	0:39:39	75.1258374	75.1258374	74.8195316	73.8813582	76.5172777
18:42:01	0:39:40	74.8258374	74.8258374	74.8195316	73.8813582	74.5172777
18:42:02	0:39:41	74.9258374	74.9258374	74.8195316	74.8813582	73.5172777
18:42:03	0:39:42	75.0258374	75.0258374	73.8195316	74.8813582	72.5172777

18:42:04	0:39:43	75.0258374	75.0258374	73.8195316	73.8813582	72.5172777
18:42:05	0:39:44	74.6258374	74.6258374	74.8195316	72.8813582	72.5172777
18:42:06	0:39:45	74.5258374	74.5258374	74.8195316	72.8813582	73.5172777
18:42:07	0:39:46	74.2258374	74.2258374	74.8195316	72.8813582	74.5172777
18:42:08	0:39:47	74.3258374	74.3258374	74.8195316	72.8813582	74.5172777
18:42:09	0:39:48	74.3258374	74.3258374	74.8195316	73.8813582	74.5172777
18:42:10	0:39:49	74.7258374	74.7258374	75.8195316	73.8813582	74.5172777
18:42:11	0:39:50	74.6258374	74.6258374	75.8195316	72.8813582	74.5172777
18:42:12	0:39:51	73.8258374	82.2842889	77.8195316	72.8813582	75.5172777
18:42:13	0:39:52	74.5258374	82.2842889	77.8195316	70.8813582	77.5172777
18:42:14	0:39:53	75.3258374	81.2842889	77.8195316	70.8813582	78.5172777
18:42:15	0:39:54	75.0258374	81.2842889	77.8195316	69.8813582	78.5172777
18:42:16	0:39:55	74.9258374	81.2842889	77.8195316	69.8813582	80.5172777
18:42:17	0:39:56	74.2258374	79.2842889	77.8195316	68.8813582	79.5172777
18:42:18	0:39:57	74.2258374	79.2842889	77.8195316	68.8813582	79.5172777
18:42:19	0:39:58	72.7258374	72.2842889	77.8195316	68.8813582	79.5172777
18:42:20	0:39:59	72.3258374	72.2842889	76.8195316	68.8813582	77.5172777
18:42:21	0:40:00	72.1258374	72.2842889	74.8195316	68.8813582	75.5172777
18:42:22	0:40:01	72.0258374	73.2842889	73.8195316	69.8813582	75.5172777
18:42:23	0:40:02	72.0258374	73.2842889	73.8195316	70.8813582	75.5172777
18:42:24	0:40:03	71.6258374	73.2842889	72.8195316	70.8813582	75.5172777
18:42:25	0:40:04	71.4258374	72.2842889	72.8195316	70.8813582	76.5172777
18:42:26	0:40:05	71.5258374	72.2842889	72.8195316	70.8813582	78.5172777
18:42:27	0:40:06	71.0258374	70.2842889	73.8195316	70.8813582	79.5172777
18:42:28	0:40:07	71.0258374	69.2842889	73.8195316	70.8813582	80.5172777
18:42:29	0:40:08	71.0258374	67.2842889	72.8195316	70.8813582	82.5172777
18:42:30	0:40:09	71.1258374	66.2842889	72.8195316	71.8813582	82.5172777
18:42:31	0:40:10	71.2258374	66.2842889	72.8195316	73.8813582	83.5172777
18:42:32	0:40:11	71.3258374	66.2842889	72.8195316	73.8813582	82.5172777
18:42:33	0:40:12	71.5258374	66.2842889	71.8195316	73.8813582	81.5172777
18:42:34	0:40:13	71.8258374	66.2842889	71.8195316	78.8813582	80.5172777
18:42:35	0:40:14	72.2258374	66.2842889	71.8195316	80.8813582	79.5172777
18:42:36	0:40:15	72.2258374	67.2842889	71.8195316	80.8813582	78.5172777
18:42:37	0:40:16	72.8258374	67.2842889	72.8195316	84.8813582	76.5172777
18:42:38	0:40:17	72.4258374	67.2842889	72.8195316	84.8813582	74.5172777
18:42:39	0:40:18	72.1258374	67.2842889	72.8195316	82.8813582	73.5172777
18:42:40	0:40:19	71.8258374	67.2842889	72.8195316	78.8813582	73.5172777
18:42:41	0:40:20	72.5258374	67.2842889	72.8195316	78.8813582	73.5172777
18:42:42	0:40:21	72.4258374	68.2842889	73.8195316	78.8813582	74.5172777
18:42:43	0:40:22	71.2258374	68.2842889	72.8195316	76.8813582	75.5172777
18:42:44	0:40:23	71.9258374	69.2842889	73.8195316	75.8813582	75.5172777
18:42:45	0:40:24	72.1258374	69.2842889	73.8195316	76.8813582	76.5172777
18:42:46	0:40:25	71.8258374	70.2842889	74.8195316	76.8813582	76.5172777
18:42:47	0:40:26	71.9258374	71.2842889	74.8195316	76.8813582	76.5172777
18:42:48	0:40:27	71.4258374	69.2842889	74.8195316	77.8813582	76.5172777

18:42:49	0:40:28	71.2258374	69.2842889	74.8195316	77.8813582	76.5172777
18:42:50	0:40:29	71.3258374	69.2842889	74.8195316	77.8813582	76.5172777
18:42:51	0:40:30	71.7258374	69.2842889	73.8195316	77.8813582	75.5172777
18:42:52	0:40:31	71.8258374	69.2842889	73.8195316	78.8813582	75.5172777
18:42:53	0:40:32	71.6258374	69.2842889	73.8195316	78.8813582	73.5172777
18:42:54	0:40:33	71.3258374	69.2842889	72.8195316	78.8813582	72.5172777
18:42:55	0:40:34	71.4258374	70.2842889	71.8195316	77.8813582	72.5172777
18:42:56	0:40:35	71.7258374	69.2842889	72.8195316	78.8813582	72.5172777
18:42:57	0:40:36	71.5258374	69.2842889	72.8195316	78.8813582	73.5172777
18:42:58	0:40:37	71.2258374	68.2842889	73.8195316	78.8813582	74.5172777
18:42:59	0:40:38	71.5258374	69.2842889	73.8195316	79.8813582	76.5172777
18:43:00	0:40:39	72.8258374	69.2842889	73.8195316	72.8258374	76.5172777
18:43:01	0:40:40	73.3258374	69.2842889	73.8195316	73.3258374	76.5172777
18:43:02	0:40:41	73.7258374	69.2842889	73.8195316	73.7258374	76.5172777
18:43:03	0:40:42	74.7258374	69.2842889	73.8195316	74.7258374	77.5172777
18:43:04	0:40:43	75.3258374	68.2842889	74.8195316	75.3258374	77.5172777
18:43:05	0:40:44	75.6258374	68.2842889	74.8195316	75.6258374	77.5172777
18:43:06	0:40:45	75.5258374	67.2842889	74.8195316	75.5258374	77.5172777
18:43:07	0:40:46	76.0258374	66.2842889	75.8195316	76.0258374	79.5172777
18:43:08	0:40:47	75.8258374	65.2842889	75.8195316	75.8258374	79.5172777
18:43:09	0:40:48	75.6258374	65.2842889	75.8195316	75.6258374	79.5172777
18:43:10	0:40:49	76.0258374	64.2842889	76.8195316	76.0258374	79.5172777
18:43:11	0:40:50	76.1258374	64.2842889	76.8195316	76.1258374	79.5172777
18:43:12	0:40:51	76.1258374	64.2842889	76.8195316	76.1258374	79.5172777
18:43:13	0:40:52	76.9258374	64.2842889	77.8195316	76.9258374	78.5172777
18:43:14	0:40:53	78.3258374	65.2842889	77.8195316	78.3258374	77.5172777
18:43:15	0:40:54	79.0258374	65.2842889	76.8195316	79.0258374	77.5172777
18:43:16	0:40:55	79.3258374	65.2842889	76.8195316	79.3258374	76.5172777
18:43:17	0:40:56	79.3258374	65.2842889	75.8195316	79.3258374	75.5172777
18:43:18	0:40:57	79.3258374	66.2842889	75.8195316	79.3258374	75.5172777
18:43:19	0:40:58	76.8258374	66.2842889	74.8195316	76.8258374	74.5172777
18:43:20	0:40:59	75.7258374	66.2842889	74.8195316	83.8813582	74.5172777
18:43:21	0:41:00	75.5258374	66.2842889	75.8195316	80.8813582	75.5172777
18:43:22	0:41:01	75.0258374	66.2842889	75.8195316	78.8813582	75.5172777
18:43:23	0:41:02	74.6258374	66.2842889	75.8195316	78.8813582	74.5172777
18:43:24	0:41:03	74.5258374	66.2842889	75.8195316	78.8813582	73.5172777
18:43:25	0:41:04	74.2258374	66.2842889	74.8195316	78.8813582	74.5172777
18:43:26	0:41:05	73.4258374	66.2842889	72.8195316	78.8813582	75.5172777
18:43:27	0:41:06	73.1258374	66.2842889	72.8195316	78.8813582	74.5172777
18:43:28	0:41:07	72.5258374	66.2842889	72.8195316	77.8813582	73.5172777
18:43:29	0:41:08	71.9258374	66.2842889	72.8195316	76.8813582	72.5172777
18:43:30	0:41:09	70.9258374	66.2842889	72.8195316	75.8813582	72.5172777
18:43:31	0:41:10	70.2258374	66.2842889	72.8195316	74.8813582	71.5172777
18:43:32	0:41:11	69.6258374	66.2842889	72.8195316	71.8813582	71.5172777
18:43:33	0:41:12	69.6258374	67.2842889	73.8195316	69.8813582	72.5172777

18:43:34	0:41:13	69.5258374	67.2842889	76.8195316	68.8813582	73.5172777
18:43:35	0:41:14	69.8258374	67.2842889	76.8195316	68.8813582	73.5172777
18:43:36	0:41:15	70.3258374	67.2842889	76.8195316	68.8813582	73.5172777
18:43:37	0:41:16	70.2258374	68.2842889	73.8195316	69.8813582	74.5172777
18:43:38	0:41:17	70.7258374	67.2842889	73.8195316	69.8813582	75.5172777
18:43:39	0:41:18	71.2258374	67.2842889	74.8195316	69.8813582	76.5172777
18:43:40	0:41:19	71.7258374	66.2842889	74.8195316	69.8813582	76.5172777
18:43:41	0:41:20	72.0258374	65.2842889	76.8195316	69.8813582	77.5172777
18:43:42	0:41:21	72.5258374	65.2842889	76.8195316	69.8813582	79.5172777
18:43:43	0:41:22	72.2258374	65.2842889	75.8195316	69.8813582	82.5172777
18:43:44	0:41:23	72.0258374	65.2842889	75.8195316	68.8813582	84.5172777
18:43:45	0:41:24	71.9258374	65.2842889	75.8195316	68.8813582	71.9258374
18:43:46	0:41:25	71.3258374	65.2842889	75.8195316	68.8813582	71.3258374
18:43:47	0:41:26	71.4258374	66.2842889	75.8195316	68.8813582	71.4258374
18:43:48	0:41:27	71.7258374	66.2842889	77.8195316	68.8813582	71.7258374
18:43:49	0:41:28	71.9258374	66.2842889	77.8195316	68.8813582	71.9258374
18:43:50	0:41:29	72.2258374	66.2842889	77.8195316	68.8813582	72.2258374
18:43:51	0:41:30	72.0258374	66.2842889	77.8195316	68.8813582	72.0258374
18:43:52	0:41:31	72.0258374	66.2842889	77.8195316	67.8813582	72.0258374
18:43:53	0:41:32	71.7258374	66.2842889	77.8195316	67.8813582	71.7258374
18:43:54	0:41:33	71.6258374	66.2842889	77.8195316	67.8813582	71.6258374
18:43:55	0:41:34	71.8258374	66.2842889	79.8195316	66.8813582	71.8258374
18:43:56	0:41:35	71.5258374	67.2842889	80.8195316	65.8813582	83.5172777
18:43:57	0:41:36	71.3258374	67.2842889	80.8195316	64.8813582	83.5172777
18:43:58	0:41:37	70.4258374	67.2842889	79.8195316	64.8813582	74.5172777
18:43:59	0:41:38	70.3258374	67.2842889	78.8195316	64.8813582	71.5172777
18:44:00	0:41:39	69.9258374	67.2842889	77.8195316	64.8813582	69.5172777
18:44:01	0:41:40	70.3258374	68.2842889	77.8195316	63.8813582	68.5172777
18:44:02	0:41:41	70.4258374	69.2842889	77.8195316	64.8813582	67.5172777
18:44:03	0:41:42	70.8258374	74.2842889	76.8195316	65.8813582	66.5172777
18:44:04	0:41:43	70.7258374	74.2842889	75.8195316	65.8813582	66.5172777
18:44:05	0:41:44	71.0258374	74.2842889	75.8195316	66.8813582	66.5172777
18:44:06	0:41:45	71.0258374	74.2842889	75.8195316	66.8813582	66.5172777
18:44:07	0:41:46	70.6258374	72.2842889	73.8195316	66.8813582	66.5172777
18:44:08	0:41:47	71.1258374	71.2842889	74.8195316	66.8813582	69.5172777
18:44:09	0:41:48	71.1258374	69.2842889	74.8195316	66.8813582	70.5172777
18:44:10	0:41:49	71.3258374	68.2842889	77.8195316	66.8813582	70.5172777
18:44:11	0:41:50	71.1258374	68.2842889	79.8195316	66.8813582	71.5172777
18:44:12	0:41:51	70.5258374	66.2842889	79.8195316	66.8813582	71.5172777
18:44:13	0:41:52	69.4258374	66.2842889	76.8195316	66.8813582	71.5172777
18:44:14	0:41:53	68.8258374	66.2842889	75.8195316	66.8813582	71.5172777
18:44:15	0:41:54	68.8258374	66.2842889	74.8195316	67.8813582	70.5172777
18:44:16	0:41:55	68.9258374	66.2842889	75.8195316	67.8813582	70.5172777
18:44:17	0:41:56	68.8258374	66.2842889	75.8195316	67.8813582	70.5172777
18:44:18	0:41:57	68.9258374	65.2842889	75.8195316	67.8813582	70.5172777

18:44:19	0:41:58	69.2258374	64.2842889	76.8195316	67.8813582	69.5172777
18:44:20	0:41:59	69.7258374	63.2842889	76.8195316	66.8813582	69.5172777
18:44:21	0:42:00	69.6258374	61.2842889	76.8195316	66.8813582	69.5172777
18:44:22	0:42:01	69.6258374	61.2842889	76.8195316	66.8813582	67.5172777
18:44:23	0:42:02	70.0258374	61.2842889	76.8195316	66.8813582	67.5172777
18:44:24	0:42:03	70.6258374	61.2842889	76.8195316	67.8813582	67.5172777
18:44:25	0:42:04	70.8258374	61.2842889	76.8195316	67.8813582	67.5172777
18:44:26	0:42:05	71.3258374	61.2842889	76.8195316	68.8813582	67.5172777
18:44:27	0:42:06	71.6258374	62.2842889	76.8195316	68.8813582	67.5172777
18:44:28	0:42:07	72.0258374	62.2842889	76.8195316	71.8813582	68.5172777
18:44:29	0:42:08	72.4258374	62.2842889	77.8195316	72.8813582	69.5172777
18:44:30	0:42:09	72.9258374	63.2842889	77.8195316	73.8813582	69.5172777
18:44:31	0:42:10	73.0258374	64.2842889	77.8195316	72.8813582	69.5172777
18:44:32	0:42:11	71.6258374	64.2842889	77.8195316	70.8813582	69.5172777
18:44:33	0:42:12	71.7258374	66.2842889	76.8195316	70.8813582	70.5172777
18:44:34	0:42:13	71.5258374	69.2842889	76.8195316	68.8813582	70.5172777
18:44:35	0:42:14	71.2258374	68.2842889	76.8195316	68.8813582	70.5172777
18:44:36	0:42:15	70.8258374	68.2842889	76.8195316	68.8813582	69.5172777
18:44:37	0:42:16	70.8258374	68.2842889	77.8195316	68.8813582	69.5172777
18:44:38	0:42:17	70.8258374	68.2842889	77.8195316	69.8813582	69.5172777
18:44:39	0:42:18	70.8258374	68.2842889	77.8195316	69.8813582	69.5172777
18:44:40	0:42:19	70.9258374	68.2842889	77.8195316	69.8813582	69.5172777
18:44:41	0:42:20	71.3258374	68.2842889	77.8195316	68.8813582	71.5172777
18:44:42	0:42:21	70.9258374	67.2842889	77.8195316	68.8813582	72.5172777
18:44:43	0:42:22	70.6258374	67.2842889	77.8195316	68.8813582	73.5172777
18:44:44	0:42:23	70.3258374	67.2842889	77.8195316	68.8813582	74.5172777
18:44:45	0:42:24	70.0258374	66.2842889	76.8195316	68.8813582	74.5172777
18:44:46	0:42:25	70.0258374	66.2842889	76.8195316	68.8813582	73.5172777
18:44:47	0:42:26	70.1258374	65.2842889	76.8195316	68.8813582	73.5172777
18:44:48	0:42:27	69.9258374	65.2842889	75.8195316	68.8813582	71.5172777
18:44:49	0:42:28	69.8258374	65.2842889	74.8195316	68.8813582	71.5172777
18:44:50	0:42:29	69.7258374	65.2842889	74.8195316	68.8813582	71.5172777
18:44:51	0:42:30	69.5258374	65.2842889	73.8195316	69.8813582	70.5172777
18:44:52	0:42:31	69.2258374	65.2842889	73.8195316	69.8813582	69.5172777
18:44:53	0:42:32	68.9258374	64.2842889	72.8195316	69.8813582	69.5172777
18:44:54	0:42:33	69.0258374	64.2842889	72.8195316	69.8813582	71.5172777
18:44:55	0:42:34	68.7258374	64.2842889	73.8195316	68.8813582	73.5172777
18:44:56	0:42:35	69.3258374	65.2842889	73.8195316	68.8813582	75.5172777
18:44:57	0:42:36	69.4258374	65.2842889	73.8195316	68.8813582	76.5172777
18:44:58	0:42:37	69.3258374	66.2842889	72.8195316	68.8813582	76.5172777
18:44:59	0:42:38	69.3258374	66.2842889	72.8195316	68.8813582	76.5172777
18:45:00	0:42:39	69.3258374	66.2842889	72.8195316	68.8813582	76.5172777
18:45:01	0:42:40	69.1258374	66.2842889	72.8195316	67.8813582	77.5172777
18:45:02	0:42:41	69.2258374	66.2842889	69.8195316	67.8813582	77.5172777
18:45:03	0:42:42	69.3258374	66.2842889	69.8195316	67.8813582	77.5172777

18:45:04	0:42:43	69.3258374	66.2842889	70.8195316	67.8813582	77.5172777
18:45:05	0:42:44	69.1258374	66.2842889	72.8195316	67.8813582	79.5172777
18:45:06	0:42:45	69.4258374	66.2842889	72.8195316	67.8813582	69.4258374
18:45:07	0:42:46	69.9258374	66.2842889	73.8195316	67.8813582	69.9258374
18:45:08	0:42:47	70.5258374	66.2842889	77.8195316	67.8813582	70.5258374
18:45:09	0:42:48	70.7258374	66.2842889	77.8195316	67.8813582	70.7258374
18:45:10	0:42:49	70.7258374	66.2842889	77.8195316	68.8813582	70.7258374
18:45:11	0:42:50	70.6258374	67.2842889	77.8195316	68.8813582	70.6258374
18:45:12	0:42:51	70.6258374	67.2842889	77.8195316	68.8813582	70.6258374
18:45:13	0:42:52	70.6258374	67.2842889	78.8195316	68.8813582	70.6258374
18:45:14	0:42:53	70.6258374	66.2842889	78.8195316	68.8813582	70.6258374
18:45:15	0:42:54	70.2258374	66.2842889	78.8195316	68.8813582	70.2258374
18:45:16	0:42:55	70.2258374	66.2842889	78.8195316	68.8813582	70.2258374
18:45:17	0:42:56	69.7258374	66.2842889	77.8195316	68.8813582	69.7258374
18:45:18	0:42:57	69.5258374	66.2842889	75.8195316	68.8813582	69.5258374
18:45:19	0:42:58	69.9258374	66.2842889	75.8195316	68.8813582	69.9258374
18:45:20	0:42:59	70.3258374	66.2842889	74.8195316	68.8813582	70.3258374
18:45:21	0:43:00	70.7258374	66.2842889	72.8195316	68.8813582	70.7258374
18:45:22	0:43:01	70.7258374	66.2842889	72.8195316	68.8813582	70.7258374
18:45:23	0:43:02	71.0258374	66.2842889	72.8195316	68.8813582	71.0258374
18:45:24	0:43:03	71.3258374	67.2842889	72.8195316	68.8813582	71.3258374
18:45:25	0:43:04	70.9258374	67.2842889	72.8195316	68.8813582	70.9258374
18:45:26	0:43:05	69.8258374	67.2842889	73.8195316	68.8813582	69.8258374
18:45:27	0:43:06	70.1258374	67.2842889	73.8195316	68.8813582	70.1258374
18:45:28	0:43:07	69.3258374	68.2842889	76.8195316	68.8813582	77.5172777
18:45:29	0:43:08	69.4258374	68.2842889	76.8195316	68.8813582	75.5172777
18:45:30	0:43:09	69.3258374	69.2842889	76.8195316	68.8813582	74.5172777
18:45:31	0:43:10	69.3258374	70.2842889	77.8195316	68.8813582	73.5172777
18:45:32	0:43:11	69.4258374	71.2842889	77.8195316	67.8813582	74.5172777
18:45:33	0:43:12	69.3258374	71.2842889	77.8195316	67.8813582	74.5172777
18:45:34	0:43:13	69.7258374	71.2842889	77.8195316	67.8813582	74.5172777
18:45:35	0:43:14	69.4258374	71.2842889	76.8195316	66.8813582	74.5172777
18:45:36	0:43:15	69.5258374	72.2842889	76.8195316	66.8813582	74.5172777
18:45:37	0:43:16	68.8258374	71.2842889	73.8195316	66.8813582	74.5172777
18:45:38	0:43:17	68.2258374	71.2842889	71.8195316	66.8813582	74.5172777
18:45:39	0:43:18	67.8258374	71.2842889	69.8195316	67.8813582	74.5172777
18:45:40	0:43:19	67.3258374	70.2842889	69.8195316	67.8813582	75.5172777
18:45:41	0:43:20	66.5258374	70.2842889	68.8195316	67.8813582	74.5172777
18:45:42	0:43:21	66.0258374	69.2842889	67.8195316	67.8813582	74.5172777
18:45:43	0:43:22	66.1258374	68.2842889	67.8195316	68.8813582	75.5172777
18:45:44	0:43:23	66.6258374	67.2842889	67.8195316	68.8813582	75.5172777
18:45:45	0:43:24	67.3258374	66.2842889	67.8195316	69.8813582	67.3258374
18:45:46	0:43:25	67.3258374	66.2842889	68.8195316	69.8813582	67.3258374
18:45:47	0:43:26	67.3258374	65.2842889	68.8195316	70.8813582	67.3258374
18:45:48	0:43:27	67.6258374	65.2842889	68.8195316	70.8813582	67.6258374

18:45:49	0:43:28	68.0258374	65.2842889	68.8195316	70.8813582	68.0258374
18:45:50	0:43:29	67.7258374	65.2842889	68.8195316	70.8813582	67.7258374
18:45:51	0:43:30	67.9258374	65.2842889	68.8195316	72.8813582	67.9258374
18:45:52	0:43:31	67.0258374	64.2842889	68.8195316	72.8813582	78.5172777
18:45:53	0:43:32	66.4258374	64.2842889	67.8195316	73.8813582	77.5172777
18:45:54	0:43:33	66.4258374	64.2842889	67.8195316	73.8813582	77.5172777
18:45:55	0:43:34	66.2258374	63.2842889	68.8195316	72.8813582	77.5172777
18:45:56	0:43:35	65.9258374	63.2842889	69.8195316	72.8813582	73.5172777
18:45:57	0:43:36	65.8258374	63.2842889	71.8195316	72.8813582	72.5172777
18:45:58	0:43:37	65.4258374	64.2842889	71.8195316	72.8813582	71.5172777
18:45:59	0:43:38	65.4258374	64.2842889	73.8195316	70.8813582	71.5172777
18:46:00	0:43:39	65.4258374	64.2842889	73.8195316	70.8813582	72.5172777
18:46:01	0:43:40	65.4258374	64.2842889	74.8195316	70.8813582	71.5172777
18:46:02	0:43:41	65.4258374	64.2842889	74.8195316	70.8813582	71.5172777
18:46:03	0:43:42	65.7258374	64.2842889	74.8195316	70.8813582	71.5172777
18:46:04	0:43:43	66.0258374	64.2842889	74.8195316	70.8813582	70.5172777
18:46:05	0:43:44	66.6258374	64.2842889	75.8195316	70.8813582	70.5172777
18:46:06	0:43:45	67.0258374	66.2842889	76.8195316	69.8813582	68.5172777
18:46:07	0:43:46	67.1258374	66.2842889	76.8195316	69.8813582	69.5172777
18:46:08	0:43:47	66.7258374	66.2842889	74.8195316	69.8813582	69.5172777
18:46:09	0:43:48	66.7258374	65.2842889	72.8195316	69.8813582	70.5172777
18:46:10	0:43:49	67.1258374	65.2842889	71.8195316	69.8813582	72.5172777
18:46:11	0:43:50	67.2258374	64.2842889	71.8195316	69.8813582	73.5172777
18:46:12	0:43:51	67.3258374	64.2842889	71.8195316	67.8813582	73.5172777
18:46:13	0:43:52	67.6258374	64.2842889	70.8195316	67.8813582	74.5172777
18:46:14	0:43:53	67.8258374	64.2842889	70.8195316	67.8813582	74.5172777
18:46:15	0:43:54	67.5258374	64.2842889	71.8195316	67.8813582	73.5172777
18:46:16	0:43:55	67.6258374	64.2842889	71.8195316	67.8813582	73.5172777
18:46:17	0:43:56	67.4258374	64.2842889	71.8195316	67.8813582	74.5172777
18:46:18	0:43:57	67.3258374	64.2842889	71.8195316	67.8813582	74.5172777
18:46:19	0:43:58	67.2258374	65.2842889	71.8195316	67.8813582	75.5172777
18:46:20	0:43:59	67.3258374	65.2842889	71.8195316	67.8813582	75.5172777
18:46:21	0:44:00	67.0258374	65.2842889	69.8195316	67.8813582	75.5172777
18:46:22	0:44:01	66.6258374	64.2842889	67.8195316	67.8813582	75.5172777
18:46:23	0:44:02	66.9258374	64.2842889	67.8195316	67.8813582	75.5172777
18:46:24	0:44:03	67.1258374	64.2842889	67.8195316	67.8813582	75.5172777
18:46:25	0:44:04	66.9258374	64.2842889	67.8195316	67.8813582	74.5172777
18:46:26	0:44:05	66.9258374	64.2842889	68.8195316	67.8813582	74.5172777
18:46:27	0:44:06	66.9258374	64.2842889	68.8195316	67.8813582	78.5172777
18:46:28	0:44:07	66.8258374	63.2842889	68.8195316	67.8813582	66.8258374
18:46:29	0:44:08	66.5258374	63.2842889	68.8195316	67.8813582	66.5258374
18:46:30	0:44:09	66.2258374	63.2842889	67.8195316	67.8813582	66.2258374
18:46:31	0:44:10	65.7258374	63.2842889	67.8195316	67.8813582	65.7258374
18:46:32	0:44:11	65.6258374	63.2842889	66.8195316	67.8813582	77.5172777
18:46:33	0:44:12	65.6258374	64.2842889	66.8195316	67.8813582	77.5172777

18:46:34	0:44:13	65.9258374	65.2842889	66.8195316	67.8813582	76.5172777
18:46:35	0:44:14	65.6258374	64.2842889	66.8195316	67.8813582	74.5172777
18:46:36	0:44:15	65.9258374	64.2842889	67.8195316	67.8813582	72.5172777
18:46:37	0:44:16	65.8258374	63.2842889	68.8195316	68.8813582	73.5172777
18:46:38	0:44:17	65.9258374	64.2842889	69.8195316	68.8813582	73.5172777
18:46:39	0:44:18	66.1258374	64.2842889	69.8195316	70.8813582	72.5172777
18:46:40	0:44:19	66.3258374	64.2842889	69.8195316	70.8813582	72.5172777
18:46:41	0:44:20	66.4258374	63.2842889	70.8195316	70.8813582	72.5172777
18:46:42	0:44:21	66.7258374	63.2842889	70.8195316	70.8813582	72.5172777
18:46:43	0:44:22	66.7258374	63.2842889	70.8195316	71.8813582	72.5172777
18:46:44	0:44:23	66.9258374	63.2842889	70.8195316	72.8813582	73.5172777
18:46:45	0:44:24	66.8258374	63.2842889	70.8195316	72.8813582	73.5172777
18:46:46	0:44:25	67.6258374	63.2842889	70.8195316	72.8813582	74.5172777
18:46:47	0:44:26	68.3258374	64.2842889	72.8195316	73.8813582	72.5172777
18:46:48	0:44:27	68.3258374	64.2842889	72.8195316	73.8813582	72.5172777
18:46:49	0:44:28	68.6258374	64.2842889	74.8195316	73.8813582	71.5172777
18:46:50	0:44:29	68.2258374	64.2842889	74.8195316	73.8813582	69.5172777
18:46:51	0:44:30	68.4258374	64.2842889	74.8195316	73.8813582	69.5172777
18:46:52	0:44:31	68.4258374	63.2842889	74.8195316	73.8813582	69.5172777
18:46:53	0:44:32	69.3258374	63.2842889	74.8195316	73.8813582	71.5172777
18:46:54	0:44:33	69.4258374	64.2842889	75.8195316	73.8813582	72.5172777
18:46:55	0:44:34	69.7258374	65.2842889	75.8195316	73.8813582	75.5172777
18:46:56	0:44:35	70.0258374	66.2842889	76.8195316	73.8813582	75.5172777
18:46:57	0:44:36	69.8258374	66.2842889	76.8195316	73.8813582	75.5172777
18:46:58	0:44:37	70.3258374	66.2842889	78.8195316	73.8813582	75.5172777
18:46:59	0:44:38	70.8258374	66.2842889	78.8195316	72.8813582	75.5172777
18:47:00	0:44:39	71.0258374	66.2842889	77.8195316	72.8813582	75.5172777
18:47:01	0:44:40	71.2258374	67.2842889	77.8195316	72.8813582	75.5172777
18:47:02	0:44:41	71.9258374	67.2842889	77.8195316	72.8813582	77.5172777
18:47:03	0:44:42	72.1258374	66.2842889	77.8195316	72.8813582	78.5172777
18:47:04	0:44:43	73.0258374	66.2842889	78.8195316	78.8813582	78.5172777
18:47:05	0:44:44	72.9258374	66.2842889	78.8195316	79.8813582	78.5172777
18:47:06	0:44:45	72.6258374	67.2842889	78.8195316	78.8813582	78.5172777
18:47:07	0:44:46	72.5258374	67.2842889	79.8195316	78.8813582	77.5172777
18:47:08	0:44:47	72.7258374	66.2842889	81.8195316	78.8813582	77.5172777
18:47:09	0:44:48	72.8258374	67.2842889	81.8195316	78.8813582	77.5172777
18:47:10	0:44:49	72.8258374	66.2842889	82.8195316	77.8813582	77.5172777
18:47:11	0:44:50	72.0258374	66.2842889	81.8195316	74.8813582	76.5172777
18:47:12	0:44:51	71.9258374	67.2842889	80.8195316	74.8813582	76.5172777
18:47:13	0:44:52	71.6258374	67.2842889	79.8195316	74.8813582	76.5172777
18:47:14	0:44:53	71.3258374	67.2842889	78.8195316	74.8813582	76.5172777
18:47:15	0:44:54	71.1258374	67.2842889	77.8195316	74.8813582	76.5172777
18:47:16	0:44:55	71.0258374	67.2842889	76.8195316	74.8813582	76.5172777
18:47:17	0:44:56	71.0258374	67.2842889	76.8195316	74.8813582	75.5172777
18:47:18	0:44:57	71.1258374	67.2842889	76.8195316	74.8813582	75.5172777

18:47:19	0:44:58	71.1258374	67.2842889	75.8195316	74.8813582	75.5172777
18:47:20	0:44:59	71.3258374	67.2842889	75.8195316	74.8813582	76.5172777
18:47:21	0:45:00	71.2258374	67.2842889	74.8195316	74.8813582	75.5172777
18:47:22	0:45:01	70.9258374	67.2842889	74.8195316	73.8813582	75.5172777
18:47:23	0:45:02	70.8258374	67.2842889	75.8195316	73.8813582	75.5172777
18:47:24	0:45:03	70.8258374	65.2842889	75.8195316	73.8813582	75.5172777
18:47:25	0:45:04	70.3258374	65.2842889	77.8195316	72.8813582	77.5172777
18:47:26	0:45:05	70.2258374	65.2842889	77.8195316	72.8813582	77.5172777
18:47:27	0:45:06	70.3258374	65.2842889	77.8195316	72.8813582	76.5172777
18:47:28	0:45:07	70.3258374	65.2842889	77.8195316	71.8813582	76.5172777
18:47:29	0:45:08	70.6258374	64.2842889	79.8195316	71.8813582	77.5172777
18:47:30	0:45:09	70.9258374	64.2842889	80.8195316	72.8813582	77.5172777
18:47:31	0:45:10	70.6258374	63.2842889	79.8195316	72.8813582	79.5172777
18:47:32	0:45:11	69.8258374	63.2842889	79.8195316	71.8813582	80.5172777
18:47:33	0:45:12	69.8258374	63.2842889	79.8195316	71.8813582	81.5172777
18:47:34	0:45:13	69.9258374	62.2842889	79.8195316	72.8813582	81.5172777
18:47:35	0:45:14	70.1258374	62.2842889	79.8195316	73.8813582	81.5172777
18:47:36	0:45:15	70.8258374	62.2842889	79.8195316	73.8813582	82.5172777
18:47:37	0:45:16	71.0258374	62.2842889	79.8195316	73.8813582	71.0258374
18:47:38	0:45:17	71.0258374	64.2842889	75.8195316	74.8813582	71.0258374
18:47:39	0:45:18	70.9258374	64.2842889	75.8195316	74.8813582	70.9258374
18:47:40	0:45:19	70.6258374	64.2842889	73.8195316	74.8813582	70.6258374
18:47:41	0:45:20	71.0258374	65.2842889	73.8195316	75.8813582	83.5172777
18:47:42	0:45:21	71.0258374	65.2842889	73.8195316	75.8813582	83.5172777
18:47:43	0:45:22	71.9258374	65.2842889	73.8195316	75.8813582	83.5172777
18:47:44	0:45:23	71.9258374	65.2842889	72.8195316	75.8813582	84.5172777
18:47:45	0:45:24	72.3258374	65.2842889	70.8195316	75.8813582	84.5172777
18:47:46	0:45:25	72.2258374	65.2842889	68.8195316	75.8813582	82.5172777
18:47:47	0:45:26	71.8258374	65.2842889	66.8195316	77.8813582	81.5172777
18:47:48	0:45:27	72.2258374	66.2842889	67.8195316	77.8813582	79.5172777
18:47:49	0:45:28	72.4258374	67.2842889	67.8195316	77.8813582	79.5172777
18:47:50	0:45:29	72.4258374	67.2842889	67.8195316	77.8813582	78.5172777
18:47:51	0:45:30	72.0258374	66.2842889	68.8195316	77.8813582	78.5172777
18:47:52	0:45:31	72.2258374	66.2842889	68.8195316	77.8813582	78.5172777
18:47:53	0:45:32	72.8258374	66.2842889	68.8195316	77.8813582	78.5172777
18:47:54	0:45:33	73.3258374	67.2842889	70.8195316	76.8813582	77.5172777
18:47:55	0:45:34	73.2258374	67.2842889	70.8195316	76.8813582	75.5172777
18:47:56	0:45:35	73.5258374	67.2842889	74.8195316	76.8813582	73.5172777
18:47:57	0:45:36	73.4258374	67.2842889	73.8195316	76.8813582	72.5172777
18:47:58	0:45:37	74.2258374	71.2842889	73.8195316	80.8813582	72.5172777
18:47:59	0:45:38	74.0258374	69.2842889	72.8195316	80.8813582	72.5172777
18:48:00	0:45:39	74.0258374	69.2842889	72.8195316	80.8813582	72.5172777
18:48:01	0:45:40	74.0258374	70.2842889	72.8195316	78.8813582	72.5172777
18:48:02	0:45:41	73.8258374	69.2842889	72.8195316	78.8813582	73.5172777
18:48:03	0:45:42	73.8258374	69.2842889	72.8195316	77.8813582	74.5172777

18:48:04	0:45:43	73.5258374	69.2842889	72.8195316	76.8813582	75.5172777
18:48:05	0:45:44	72.9258374	68.2842889	72.8195316	73.8813582	75.5172777
18:48:06	0:45:45	72.4258374	67.2842889	72.8195316	73.8813582	75.5172777
18:48:07	0:45:46	72.0258374	67.2842889	72.8195316	70.8813582	75.5172777
18:48:08	0:45:47	72.0258374	67.2842889	73.8195316	70.8813582	75.5172777
18:48:09	0:45:48	71.8258374	67.2842889	73.8195316	70.8813582	75.5172777
18:48:10	0:45:49	71.6258374	67.2842889	73.8195316	70.8813582	74.5172777
18:48:11	0:45:50	71.4258374	67.2842889	72.8195316	70.8813582	73.5172777
18:48:12	0:45:51	71.1258374	67.2842889	71.8195316	69.8813582	73.5172777
18:48:13	0:45:52	70.4258374	64.2842889	71.8195316	69.8813582	72.5172777
18:48:14	0:45:53	70.4258374	64.2842889	71.8195316	69.8813582	72.5172777
18:48:15	0:45:54	70.5258374	64.2842889	71.8195316	69.8813582	72.5172777
18:48:16	0:45:55	70.6258374	65.2842889	72.8195316	70.8813582	72.5172777
18:48:17	0:45:56	70.7258374	65.2842889	72.8195316	71.8813582	72.5172777
18:48:18	0:45:57	71.4258374	66.2842889	72.8195316	71.8813582	74.5172777
18:48:19	0:45:58	71.9258374	66.2842889	74.8195316	71.8813582	75.5172777
18:48:20	0:45:59	71.9258374	66.2842889	73.8195316	71.8813582	74.5172777
18:48:21	0:46:00	72.8258374	66.2842889	72.8195316	70.8813582	73.5172777
18:48:22	0:46:01	73.0258374	66.2842889	70.8195316	70.8813582	73.5172777
18:48:23	0:46:02	72.6258374	66.2842889	70.8195316	70.8813582	72.5172777
18:48:24	0:46:03	72.4258374	64.2842889	68.8195316	70.8813582	72.5172777
18:48:25	0:46:04	73.5258374	64.2842889	67.8195316	70.8813582	74.5172777
18:48:26	0:46:05	73.7258374	64.2842889	66.8195316	69.8813582	76.5172777
18:48:27	0:46:06	73.7258374	64.2842889	66.8195316	69.8813582	76.5172777
18:48:28	0:46:07	73.6258374	64.2842889	66.8195316	69.8813582	76.5172777
18:48:29	0:46:08	74.0258374	63.2842889	66.8195316	69.8813582	77.5172777
18:48:30	0:46:09	74.1258374	64.2842889	66.8195316	69.8813582	77.5172777
18:48:31	0:46:10	74.7258374	65.2842889	66.8195316	70.8813582	78.5172777
18:48:32	0:46:11	75.1258374	65.2842889	66.8195316	70.8813582	79.5172777
18:48:33	0:46:12	75.4258374	65.2842889	66.8195316	69.8813582	80.5172777
18:48:34	0:46:13	75.4258374	65.2842889	67.8195316	69.8813582	80.5172777
18:48:35	0:46:14	75.5258374	64.2842889	68.8195316	69.8813582	82.5172777
18:48:36	0:46:15	75.5258374	64.2842889	68.8195316	69.8813582	82.5172777
18:48:37	0:46:16	75.5258374	64.2842889	68.8195316	68.8813582	85.5172777
18:48:38	0:46:17	75.1258374	63.2842889	68.8195316	68.8813582	85.5172777
18:48:39	0:46:18	74.9258374	63.2842889	68.8195316	68.8813582	84.5172777
18:48:40	0:46:19	74.1258374	63.2842889	68.8195316	68.8813582	80.5172777
18:48:41	0:46:20	73.5258374	63.2842889	68.8195316	69.8813582	76.5172777
18:48:42	0:46:21	73.8258374	63.2842889	68.8195316	70.8813582	76.5172777
18:48:43	0:46:22	73.6258374	63.2842889	69.8195316	70.8813582	76.5172777
18:48:44	0:46:23	73.8258374	63.2842889	69.8195316	70.8813582	77.5172777
18:48:45	0:46:24	74.2258374	63.2842889	68.8195316	70.8813582	77.5172777
18:48:46	0:46:25	74.5258374	63.2842889	68.8195316	71.8813582	78.5172777
18:48:47	0:46:26	74.6258374	62.2842889	68.8195316	71.8813582	78.5172777
18:48:48	0:46:27	75.0258374	62.2842889	69.8195316	72.8813582	78.5172777

18:48:49	0:46:28	75.7258374	62.2842889	69.8195316	72.8813582	77.5172777
18:48:50	0:46:29	76.5258374	63.2842889	70.8195316	72.8813582	77.5172777
18:48:51	0:46:30	76.4258374	63.2842889	70.8195316	72.8813582	75.5172777
18:48:52	0:46:31	76.4258374	63.2842889	70.8195316	73.8813582	75.5172777
18:48:53	0:46:32	77.0258374	64.2842889	70.8195316	73.8813582	77.5172777
18:48:54	0:46:33	76.9258374	64.2842889	71.8195316	74.8813582	78.5172777
18:48:55	0:46:34	77.5258374	64.2842889	71.8195316	74.8813582	79.5172777
18:48:56	0:46:35	77.8258374	64.2842889	73.8195316	73.8813582	79.5172777
18:48:57	0:46:36	77.6258374	64.2842889	74.8195316	73.8813582	79.5172777
18:48:58	0:46:37	78.1258374	64.2842889	74.8195316	73.8813582	80.5172777
18:48:59	0:46:38	77.7258374	67.2842889	74.8195316	73.8813582	80.5172777
18:49:00	0:46:39	76.9258374	67.2842889	74.8195316	71.8813582	80.5172777
18:49:01	0:46:40	76.0258374	66.2842889	74.8195316	71.8813582	80.5172777
18:49:02	0:46:41	75.8258374	66.2842889	74.8195316	71.8813582	79.5172777
18:49:03	0:46:42	76.0258374	66.2842889	74.8195316	72.8813582	77.5172777
18:49:04	0:46:43	76.1258374	66.2842889	74.8195316	72.8813582	77.5172777
18:49:05	0:46:44	76.0258374	66.2842889	74.8195316	72.8813582	77.5172777
18:49:06	0:46:45	75.6258374	66.2842889	73.8195316	71.8813582	74.5172777
18:49:07	0:46:46	75.1258374	66.2842889	73.8195316	71.8813582	72.5172777
18:49:08	0:46:47	74.7258374	66.2842889	72.8195316	71.8813582	71.5172777
18:49:09	0:46:48	74.5258374	66.2842889	72.8195316	72.8813582	71.5172777
18:49:10	0:46:49	74.4258374	67.2842889	71.8195316	72.8813582	71.5172777
18:49:11	0:46:50	74.2258374	66.2842889	71.8195316	72.8813582	72.5172777
18:49:12	0:46:51	74.2258374	66.2842889	71.8195316	73.8813582	73.5172777
18:49:13	0:46:52	74.1258374	66.2842889	71.8195316	74.8813582	73.5172777
18:49:14	0:46:53	74.0258374	67.2842889	71.8195316	74.8813582	74.5172777
18:49:15	0:46:54	74.4258374	67.2842889	71.8195316	74.8813582	75.5172777
18:49:16	0:46:55	74.8258374	67.2842889	71.8195316	75.8813582	76.5172777
18:49:17	0:46:56	75.2258374	68.2842889	71.8195316	75.8813582	76.5172777
18:49:18	0:46:57	75.8258374	68.2842889	71.8195316	75.8813582	77.5172777
18:49:19	0:46:58	76.4258374	72.2842889	71.8195316	75.8813582	77.5172777
18:49:20	0:46:59	76.8258374	72.2842889	72.8195316	76.8813582	77.5172777
18:49:21	0:47:00	76.8258374	71.2842889	73.8195316	76.8813582	77.5172777
18:49:22	0:47:01	76.6258374	68.2842889	73.8195316	76.8813582	77.5172777

Person 6	Person 3	Person 5	Person 10	Person 11	Person 8
75.3734324	64.9289015	86.421441		75.928496	
75.3734324	63.9289015	86.421441		74.928496	
75.3734324	63.9289015	83.421441		74.928496	
74.3734324	63.9289015	83.421441		74.928496	
74.3734324	63.9289015	83.421441		73.928496	
74.3734324	63.9289015	83.421441		72.928496	
74.3734324	63.9289015	83.421441		72.928496	
74.3734324	63.9289015	82.421441		72.928496	
73.3734324	63.9289015	83.421441		72.928496	
73.3734324	63.9289015	84.421441		72.928496	
74.3734324	62.9289015	84.421441		71.928496	
74.3734324	62.9289015	84.421441		71.928496	
72.3734324	62.9289015	84.421441		71.928496	
73.3734324	62.9289015	84.421441	70.1725398	71.928496	
73.3734324	62.9289015	84.421441	71.1725398	71.928496	
73.3734324	62.9289015	85.421441	71.1725398	71.928496	
73.3734324	63.9289015	85.421441	72.1725398	69.928496	
73.3734324	63.9289015	86.421441	72.1725398	69.928496	
73.3734324	64.9289015	76.1428504	72.1725398	69.928496	
73.3734324	65.9289015	75.4428504	68.1725398	68.928496	
73.3734324	65.9289015	75.4428504	68.1725398	68.928496	
74.3734324	68.9289015	76.2428504	68.1725398	68.928496	
74.3734324	68.9289015	75.8428504	67.1725398	68.928496	
74.3734324	68.9289015	76.2428504	67.1725398	68.928496	
74.3734324	68.9289015	76.1428504	67.1725398	68.928496	
75.3734324	71.9289015	76.0428504	68.1725398	68.928496	
75.3734324	71.9289015	75.3428504	68.1725398	68.928496	
75.3734324	71.9289015	74.7428504	67.1725398	67.928496	
75.3734324	71.9289015	74.0428504	67.1725398	67.928496	
75.3734324	71.9289015	74.0428504	67.1725398	70.928496	
77.3734324	69.9289015	82.421441	69.1725398	74.928496	
77.3734324	68.9289015	81.421441	69.1725398	74.928496	
76.3734324	68.9289015	81.421441	71.1725398	75.928496	
76.3734324	68.9289015	80.421441	72.1725398	75.928496	
75.3734324	68.9289015	78.421441	72.1725398	75.928496	
75.3734324	67.9289015	73.421441	72.1725398	71.928496	
75.3734324	67.9289015	72.421441	72.1725398	70.928496	
75.3734324	67.9289015	72.421441	72.1725398	70.928496	
75.3734324	76.9289015	71.421441	72.1725398	70.928496	
75.3734324	80.9289015	71.421441	72.1725398	70.928496	
75.3734324	80.9289015	71.421441	72.1725398	72.928496	
74.3734324	78.9289015	71.421441	71.1725398	72.928496	

74.3734324	78.9289015	71.421441	72.1725398	71.928496
73.3734324	76.9289015	70.421441	72.1725398	71.928496
68.3734324	76.9289015	70.421441	73.1725398	71.928496
69.3734324	76.9289015	70.421441	73.1725398	71.928496
69.3734324	76.9289015	72.421441	73.1725398	71.928496
69.3734324	76.9289015	73.421441	73.1725398	70.928496
69.3734324	79.9289015	73.421441	73.1725398	69.928496
65.3734324	80.9289015	75.421441	73.1725398	66.928496
65.3734324	80.9289015	76.421441	73.1725398	66.928496
65.3734324	80.9289015	76.421441	73.1725398	65.928496
66.3734324	80.9289015	75.421441	71.1725398	65.928496
66.3734324	80.9289015	75.421441	70.1725398	65.928496
66.3734324	80.9289015	75.421441	70.1725398	65.928496
64.3734324	80.9289015	75.421441	70.1725398	66.928496
64.3734324	81.9289015	76.421441	70.1725398	67.928496
64.3734324	81.9289015	76.421441	70.1725398	68.928496
66.3734324	81.9289015	76.421441	70.1725398	69.928496
66.3734324	81.9289015	77.421441	69.1725398	73.928496
66.3734324	82.9289015	77.421441	69.1725398	74.928496
66.3734324	82.9289015	80.421441	71.1725398	74.928496
65.3734324	82.9289015	81.421441	71.1725398	74.928496
65.3734324	83.9289015	81.421441	71.1725398	74.928496
65.3734324	83.9289015	82.421441	69.1725398	72.928496
66.3734324	81.9289015	82.421441	69.1725398	72.928496
66.3734324	76.9289015	70.6428504	69.1725398	71.928496
66.3734324	76.9289015	71.0428504	69.1725398	72.928496
65.3734324	75.9289015	70.9428504	69.1725398	73.928496
64.3734324	74.9289015	78.421441	68.1725398	73.928496
64.3734324	73.9289015	78.421441	68.1725398	73.928496
64.3734324	73.9289015	77.421441	67.1725398	73.928496
64.3734324	73.9289015	77.421441	67.1725398	73.928496
64.3734324	72.9289015	78.421441	68.1725398	73.928496
64.3734324	72.9289015	78.421441	68.1725398	73.928496
63.3734324	71.9289015	78.421441	69.1725398	75.928496
62.3734324	71.9289015	78.421441	69.1725398	76.928496
62.3734324	71.9289015	78.421441	69.1725398	76.928496
62.3734324	70.9289015	78.421441	68.1725398	76.928496
62.3734324	70.9289015	78.421441	68.1725398	77.928496
62.3734324	71.9289015	77.421441	68.1725398	78.928496
63.3734324	74.9289015	77.421441	68.1725398	77.928496
64.3734324	74.9289015	77.421441	72.1725398	75.928496
64.3734324	74.9289015	78.421441	73.1725398	73.928496
64.3734324	74.9289015	79.421441	73.1725398	72.928496
65.3734324	74.9289015	78.421441	74.1725398	72.928496
66.3734324	73.9289015	79.421441	74.1725398	72.928496

66.3734324	73.9289015	79.421441	74.1725398	72.928496
66.3734324	75.9289015	80.421441	74.1725398	72.928496
66.3734324	73.9289015	79.421441	77.1725398	71.928496
67.3734324	72.9289015	79.421441	77.1725398	70.928496
70.3734324	72.9289015	78.421441	77.1725398	69.928496
70.3734324	72.9289015	78.421441	78.1725398	69.928496
70.3734324	73.9289015	78.421441	78.1725398	68.928496
80.3734324	73.9289015	77.421441	78.1725398	67.928496
81.3734324	73.9289015	77.421441	77.1725398	66.928496
81.3734324	72.9289015	77.421441	77.1725398	65.928496
81.3734324	72.9289015	77.421441	77.1725398	65.928496
79.3734324	72.9289015	77.421441	77.1725398	65.928496
77.3734324	72.9289015	77.421441	77.1725398	65.928496
76.3734324	72.9289015	76.421441	77.1725398	66.928496
76.3734324	72.9289015	72.421441	77.1725398	67.928496
75.3734324	72.9289015	72.421441	77.1725398	67.928496
75.3734324	72.9289015	71.421441	77.1725398	69.928496
75.3734324	73.9289015	71.421441	77.1725398	69.928496
75.3734324	73.9289015	70.421441	78.1725398	70.928496
75.3734324	73.9289015	70.421441	82.1725398	70.928496
74.3734324	73.9289015	69.421441	82.1725398	69.928496
74.3734324	72.9289015	69.421441	83.1725398	69.928496
73.3734324	72.9289015	69.421441	83.1725398	68.928496
72.3734324	72.9289015	69.421441	83.1725398	68.928496
72.3734324	73.9289015	69.421441	82.1725398	67.928496
71.3734324	80.9289015	70.421441	81.1725398	67.928496
71.3734324	80.9289015	70.421441	80.1725398	68.928496
71.3734324	81.9289015	73.421441	78.1725398	68.928496
69.3734324	81.9289015	76.421441	77.1725398	70.928496
68.3734324	81.9289015	76.421441	76.1725398	72.928496
68.3734324	83.9289015	76.421441	76.1725398	75.928496
65.3734324	83.9289015	76.421441	74.1725398	78.928496
65.3734324	81.9289015	75.421441	74.1725398	78.928496
65.3734324	78.9289015	73.421441	72.1725398	78.928496
65.3734324	75.9289015	72.421441	72.1725398	74.928496
65.3734324	73.9289015	72.421441	71.1725398	72.928496
65.3734324	73.9289015	72.421441	71.1725398	72.928496
66.3734324	72.9289015	73.421441	72.1725398	72.928496
66.3734324	70.9289015	73.421441	72.1725398	72.928496
66.3734324	70.9289015	75.421441	72.1725398	72.928496
66.3734324	71.9289015	75.421441	72.1725398	73.928496
66.3734324	71.9289015	75.421441	72.1725398	73.928496
66.3734324	69.9289015	74.421441	71.1725398	68.928496
67.3734324	70.9289015	73.421441	70.1725398	68.928496
67.3734324	70.9289015	74.421441	70.1725398	68.928496

68.3734324	71.9289015	74.421441	70.1725398	69.928496
70.3734324	73.9289015	76.421441	70.1725398	69.928496
70.3734324	75.9289015	75.421441	75.1725398	69.928496
72.3734324	79.9289015	75.421441	76.1725398	69.928496
73.3734324	80.9289015	77.421441	77.1725398	68.928496
73.3734324	80.9289015	76.421441	77.1725398	67.928496
74.3734324	79.9289015	76.421441	77.1725398	66.928496
77.3734324	77.9289015	76.421441	77.1725398	66.928496
77.3734324	76.9289015	76.421441	77.1725398	66.928496
78.3734324	75.9289015	76.421441	77.1725398	66.928496
79.3734324	75.9289015	76.421441	77.1725398	65.928496
79.3734324	75.9289015	76.421441	78.1725398	65.928496
79.3734324	75.9289015	76.421441	77.1725398	66.928496
79.3734324	75.9289015	76.421441	76.1725398	67.928496
79.3734324	74.9289015	76.421441	74.1725398	67.928496
79.3734324	77.9289015	80.421441	74.1725398	68.928496
79.3734324	77.9289015	81.421441	73.1725398	69.928496
79.3734324	77.9289015	82.421441	73.1725398	72.928496
78.3734324	76.9289015	82.421441	74.1725398	75.928496
77.3734324	76.9289015	82.421441	75.1725398	75.928496
73.3734324	76.9289015	81.421441	75.1725398	77.928496
73.3734324	76.9289015	81.421441	75.1725398	77.928496
73.3734324	76.9289015	81.421441	75.1725398	75.928496
73.3734324	76.9289015	81.421441	75.1725398	74.928496
73.3734324	76.9289015	81.421441	75.1725398	74.928496
73.3734324	74.9289015	81.421441	77.1725398	74.928496
73.3734324	73.9289015	82.421441	77.1725398	74.928496
73.3734324	71.9289015	82.421441	77.1725398	74.928496
73.3734324	70.9289015	82.421441	76.1725398	71.928496
73.3734324	70.9289015	82.421441	76.1725398	71.928496
73.3734324	69.9289015	77.421441	74.1725398	71.928496
74.3734324	69.9289015	77.421441	71.1725398	70.928496
72.3734324	69.9289015	77.421441	71.1725398	70.928496
71.3734324	69.9289015	74.421441	72.1725398	70.928496
71.3734324	69.9289015	74.421441	76.1725398	69.928496
71.3734324	69.9289015	74.421441	76.1725398	68.928496
71.3734324	69.9289015	74.421441	77.1725398	68.928496
71.3734324	69.9289015	74.421441	76.1725398	67.928496
74.3734324	69.9289015	73.421441	70.1725398	66.928496
74.3734324	69.9289015	73.421441	68.1725398	66.928496
76.3734324	69.9289015	72.421441	68.1725398	66.928496
76.3734324	69.9289015	72.421441	67.1725398	64.928496
76.3734324	75.9289015	72.421441	69.1725398	65.928496
76.3734324	75.9289015	72.421441	70.1725398	66.928496
76.3734324	78.9289015	72.421441	70.1725398	69.928496

76.3734324	79.9289015	73.421441	70.1725398	70.928496
75.3734324	81.9289015	74.421441	70.1725398	70.928496
75.3734324	81.9289015	73.421441	70.1725398	70.928496
75.3734324	81.9289015	73.421441	70.1725398	70.928496
75.3734324	81.9289015	73.421441	70.1725398	70.928496
75.3734324	81.9289015	73.421441	70.1725398	70.928496
75.3734324	81.9289015	73.421441	71.1725398	70.928496
71.3734324	81.9289015	73.421441	72.1725398	73.928496
70.3734324	81.9289015	73.421441	70.1725398	73.928496
69.3734324	80.9289015	73.421441	70.1725398	73.928496
68.3734324	80.9289015	71.421441	69.1725398	74.928496
69.3734324	78.9289015	71.421441	69.1725398	75.928496
69.3734324	78.9289015	71.421441	72.1725398	80.928496
69.3734324	78.9289015	71.421441	72.1725398	81.928496
71.3734324	78.9289015	70.421441	72.1725398	81.928496
72.3734324	79.9289015	70.421441	74.1725398	83.928496
72.3734324	81.9289015	71.421441	76.1725398	83.928496
73.3734324	81.9289015	72.421441	75.1725398	83.928496
72.3734324	81.9289015	72.421441	76.1725398	83.928496
72.3734324	81.9289015	71.421441	76.1725398	83.928496
69.3734324	81.9289015	70.421441	76.1725398	82.928496
69.3734324	80.9289015	69.421441	77.1725398	81.928496
68.3734324	80.9289015	70.421441	77.1725398	81.928496
67.3734324	80.9289015	70.421441	76.1725398	81.928496
67.3734324	80.9289015	70.421441	75.1725398	81.928496
67.3734324	80.9289015	70.421441	75.1725398	77.928496
67.3734324	80.9289015	70.421441	75.1725398	75.928496
67.3734324	79.9289015	70.421441	75.1725398	73.928496
67.3734324	79.9289015	70.421441	74.1725398	73.928496
66.3734324	79.9289015	70.421441	73.1725398	74.928496
65.3734324	80.9289015	70.421441	75.1725398	74.928496
65.3734324	80.9289015	71.421441	75.1725398	80.928496
67.3734324	80.9289015	71.421441	76.1725398	81.928496
67.3734324	80.9289015	71.421441	77.1725398	81.928496
67.3734324	80.9289015	71.421441	76.1725398	81.928496
66.3734324	79.9289015	72.421441	73.1725398	81.928496
66.3734324	79.9289015	74.421441	73.1725398	76.928496
66.3734324	79.9289015	74.421441	72.1725398	76.928496
64.3734324	79.9289015	74.421441	72.1725398	72.928496
64.3734324	78.9289015	74.421441	72.1725398	72.928496
62.3734324	77.9289015	74.421441	72.1725398	72.928496
62.3734324	77.9289015	74.421441	72.1725398	72.928496
61.3734324	76.9289015	75.421441	72.1725398	72.928496
61.3734324	73.9289015	75.421441	71.1725398	72.928496
61.3734324	73.9289015	76.421441	65.1725398	71.928496

61.3734324	71.9289015	79.421441	62.1725398	70.928496	
58.3734324	70.9289015	80.421441	62.1725398	69.928496	
59.3734324	72.9289015	79.421441	62.1725398	69.928496	
60.3734324	72.9289015	79.421441	63.1725398	67.928496	
60.3734324	72.9289015	79.421441	63.1725398	67.928496	
61.3734324	72.9289015	79.421441	63.1725398	67.928496	
62.3734324	72.9289015	78.421441	63.1725398	67.928496	
62.3734324	72.9289015	76.421441	63.1725398	65.928496	
61.3734324	72.9289015	76.421441	66.1725398	65.928496	
61.3734324	73.9289015	76.421441	66.1725398	65.928496	
62.3734324	75.9289015	76.421441	66.1725398	66.928496	
62.3734324	75.9289015	76.421441	66.1725398	66.928496	
62.3734324	72.9289015	76.421441	69.1725398	67.928496	
62.3734324	72.9289015	76.421441	73.1725398	67.928496	
62.3734324	72.9289015	76.421441	73.1725398	67.928496	
62.3734324	72.9289015	76.421441	76.1725398	67.928496	
62.3734324	72.9289015	76.421441	76.1725398	67.928496	
71.3734324	72.9289015	76.421441	75.1725398	67.928496	73.7599646
71.3734324	72.9289015	77.421441	73.1725398	74.928496	74.123601
71.3734324	72.9289015	77.421441	74.1725398	78.928496	74.3054192
74.3734324	73.9289015	77.421441	74.1725398	80.928496	74.5781464
74.3734324	73.9289015	77.421441	74.1725398	80.928496	74.3963282
76.3734324	71.9289015	78.421441	76.1725398	78.928496	80.9311067
76.3734324	71.9289015	78.421441	76.1725398	77.928496	79.9311067
76.3734324	70.9289015	78.421441	76.1725398	76.928496	79.9311067
76.3734324	70.9289015	78.421441	76.1725398	75.928496	79.9311067
75.3734324	71.9289015	78.421441	76.1725398	75.928496	79.9311067
77.3734324	71.9289015	77.421441	75.1725398	75.928496	78.9311067
77.3734324	71.9289015	78.421441	75.1725398	78.928496	78.9311067
77.3734324	71.9289015	78.421441	77.1725398	81.928496	78.9311067
77.3734324	71.9289015	78.421441	78.1725398	81.928496	78.9311067
77.3734324	72.9289015	78.421441	78.1725398	81.928496	78.9311067
77.3734324	73.9289015	78.421441	78.1725398	80.928496	77.9311067
77.3734324	73.9289015	78.421441	78.1725398	73.928496	75.9311067
73.3734324	73.9289015	78.421441	78.1725398	70.928496	75.9311067
72.3734324	73.9289015	78.421441	77.1725398	68.928496	75.9311067
72.3734324	75.9289015	78.421441	76.1725398	67.928496	76.9311067
72.3734324	75.9289015	78.421441	76.1725398	67.928496	78.9311067
72.3734324	71.9289015	78.421441	76.1725398	67.928496	78.9311067
72.3734324	71.9289015	78.421441	77.1725398	67.928496	78.9311067
70.3734324	71.9289015	77.421441	77.1725398	67.928496	78.9311067
70.3734324	70.9289015	77.421441	76.1725398	67.928496	78.9311067
70.3734324	70.9289015	77.421441	74.1725398	68.928496	78.9311067
68.3734324	70.9289015	77.421441	73.1725398	68.928496	77.9311067
68.3734324	70.9289015	77.421441	73.1725398	68.928496	77.9311067

68.3734324	70.9289015	77.421441	73.1725398	67.928496	77.9311067
68.3734324	70.9289015	77.421441	73.1725398	66.928496	77.9311067
69.3734324	62.9289015	77.421441	74.1725398	65.928496	76.9311067
70.3734324	61.9289015	78.421441	74.1725398	64.928496	76.9311067
70.3734324	61.9289015	78.421441	74.1725398	64.928496	76.9311067
70.3734324	61.9289015	78.421441	73.1725398	65.928496	76.9311067
71.3734324	61.9289015	79.421441	71.1725398	66.928496	76.9311067
70.3734324	61.9289015	79.421441	71.1725398	67.928496	76.9311067
70.3734324	60.9289015	79.421441	72.1725398	67.928496	76.9311067
70.3734324	55.9289015	79.421441	74.1725398	68.928496	76.9311067
70.3734324	55.9289015	79.421441	75.1725398	68.928496	76.9311067
70.3734324	52.9289015	78.421441	74.1725398	67.928496	76.9311067
69.3734324	50.9289015	76.421441	74.1725398	65.928496	76.9311067
69.3734324	46.9289015	72.421441	74.1725398	65.928496	71.9311067
69.3734324	44.9289015	72.421441	74.1725398	64.928496	71.9311067
70.3734324	43.9289015	70.421441	74.1725398	64.928496	71.9311067
70.3734324	44.9289015	71.421441	73.1725398	65.928496	71.9311067
70.3734324	51.9289015	71.421441	73.1725398	66.928496	71.9311067
70.3734324	58.9289015	71.421441	73.1725398	67.928496	71.9311067
70.3734324	60.9289015	71.421441	74.1725398	68.928496	71.9311067
70.3734324	62.9289015	71.421441	75.1725398	68.928496	71.9311067
70.3734324	63.9289015	71.421441	75.1725398	71.928496	72.9311067
70.3734324	63.9289015	72.421441	75.1725398	74.928496	73.9311067
70.3734324	63.9289015	72.421441	75.1725398	74.928496	73.9311067
71.3734324	65.9289015	74.421441	75.1725398	74.928496	73.9311067
72.3734324	69.9289015	75.421441	74.1725398	73.928496	73.9311067
72.3734324	70.9289015	75.421441	74.1725398	70.928496	73.9311067
72.3734324	71.9289015	74.421441	71.1725398	69.928496	73.9311067
73.3734324	71.9289015	72.421441	71.1725398	69.928496	75.9311067
75.3734324	72.9289015	72.421441	75.1725398	69.928496	76.9311067
75.3734324	74.9289015	72.421441	75.1725398	69.928496	77.9311067
75.3734324	74.9289015	72.421441	74.1725398	69.928496	77.9311067
75.3734324	76.9289015	72.421441	72.1725398	69.928496	77.9311067
74.3734324	75.9289015	72.421441	70.1725398	69.928496	77.9311067
73.3734324	75.9289015	70.421441	70.1725398	69.928496	75.9311067
73.3734324	74.9289015	70.421441	68.1725398	69.928496	75.9311067
73.3734324	74.9289015	73.421441	68.1725398	69.928496	75.9311067
72.3734324	74.9289015	73.421441	69.1725398	69.928496	75.9311067
73.3734324	74.9289015	76.421441	71.1725398	71.928496	75.9311067
75.3734324	74.9289015	78.421441	72.1725398	74.928496	75.9311067
75.3734324	74.9289015	79.421441	72.1725398	77.928496	75.9311067
75.3734324	73.9289015	79.421441	73.1725398	79.928496	76.9311067
76.3734324	71.9289015	79.421441	72.1725398	79.928496	76.9311067
76.3734324	71.9289015	79.421441	72.1725398	80.928496	76.9311067
76.3734324	71.9289015	79.421441	72.1725398	81.928496	70.9311067

76.3734324	70.9289015	78.421441	72.1725398	81.928496	70.9311067
76.3734324	71.9289015	78.421441	72.1725398	81.928496	68.9311067
78.3734324	71.9289015	77.421441	72.1725398	82.928496	68.9311067
78.3734324	71.9289015	76.421441	72.1725398	84.928496	68.9311067
78.3734324	71.9289015	76.421441	72.1725398	84.928496	68.9311067
78.3734324	71.9289015	76.421441	73.1725398	84.928496	68.9311067
75.3734324	72.9289015	75.421441	73.1725398	83.928496	67.9311067
74.3734324	73.9289015	81.421441	73.1725398	83.928496	68.9311067
72.3734324	74.9289015	85.421441	73.1725398	83.928496	68.9311067
72.3734324	74.9289015	75.4872373	79.1725398	83.928496	68.9311067
70.3734324	74.9289015	75.7599646	79.1725398	83.928496	68.9311067
69.3734324	75.9289015	76.123601	80.1725398	83.928496	66.9311067
69.3734324	78.9289015	76.123601	80.1725398	83.928496	65.9311067
69.3734324	79.9289015	76.2145101	80.1725398	83.928496	66.9311067
69.3734324	79.9289015	77.0326919	80.1725398	84.928496	66.9311067
69.3734324	79.9289015	77.4872373	81.1725398	84.928496	66.9311067
69.3734324	79.9289015	77.123601	82.1725398	84.928496	66.9311067
68.3734324	78.9289015	77.0326919	83.1725398	84.928496	67.9311067
68.3734324	78.9289015	76.123601	81.1725398	83.928496	68.9311067
68.3734324	78.9289015	75.6690555	78.1725398	83.928496	68.9311067
67.3734324	78.9289015	75.6690555	78.1725398	83.928496	68.9311067
66.3734324	77.9289015	75.4872373	77.1725398	82.928496	70.9311067
66.3734324	73.9289015	74.6690555	74.1725398	80.928496	70.9311067
66.3734324	73.9289015	74.3054192	73.1725398	71.928496	71.9311067
66.3734324	68.9289015	73.3963282	73.1725398	67.928496	71.9311067
66.3734324	68.9289015	73.3963282	73.1725398	66.928496	71.9311067
66.3734324	68.9289015	74.123601	78.1725398	67.928496	71.9311067
65.3734324	67.9289015	73.8508737	82.1725398	68.928496	71.9311067
65.3734324	67.9289015	73.7599646	82.1725398	68.928496	72.9311067
65.3734324	67.9289015	77.421441	83.1725398	69.928496	72.9311067
65.3734324	71.9289015	74.421441	83.1725398	70.928496	72.9311067
65.3734324	74.9289015	74.421441	77.1725398	73.928496	72.9311067
65.3734324	74.9289015	74.421441	77.1725398	73.928496	72.9311067
67.3734324	72.9289015	73.421441	73.1725398	73.928496	72.9311067
67.3734324	72.9289015	73.421441	73.1725398	74.928496	72.9311067
67.3734324	72.9289015	73.421441	73.1725398	74.928496	72.9311067
67.3734324	72.9289015	73.421441	76.1725398	67.928496	73.9311067
68.3734324	72.9289015	72.421441	76.1725398	65.928496	73.9311067
68.3734324	72.9289015	71.421441	78.1725398	65.928496	72.9311067
68.3734324	72.9289015	71.421441	78.1725398	65.928496	72.9311067
67.3734324	72.9289015	71.421441	77.1725398	64.928496	73.9311067
65.3734324	72.9289015	71.421441	75.1725398	64.928496	74.9311067
65.3734324	72.9289015	72.421441	74.1725398	63.928496	75.9311067
65.3734324	72.9289015	74.421441	72.1725398	66.928496	75.9311067
65.3734324	75.9289015	75.421441	72.1725398	66.928496	75.9311067

67.3734324	77.9289015	75.421441	73.1725398	66.928496	75.9311067
67.3734324	78.9289015	75.421441	73.1725398	68.928496	75.9311067
67.3734324	78.9289015	75.421441	73.1725398	69.928496	76.9311067
68.3734324	78.9289015	75.421441	75.1725398	69.928496	76.9311067
69.3734324	79.9289015	75.421441	75.1725398	72.928496	77.9311067
69.3734324	81.9289015	74.421441	76.1725398	73.928496	77.9311067
70.3734324	83.9289015	74.421441	74.1725398	71.928496	78.9311067
70.3734324	83.9289015	72.421441	70.1725398	71.928496	78.9311067
71.3734324	83.9289015	72.421441	69.1725398	70.928496	78.9311067
71.3734324	83.9289015	73.421441	68.1725398	70.928496	77.9311067
70.3734324	83.9289015	73.421441	68.1725398	70.928496	77.9311067
72.3734324	84.9289015	74.421441	68.1725398	70.928496	76.9311067
72.3734324	86.9289015	72.421441	71.1725398	70.928496	76.9311067
72.3734324	86.9289015	72.421441	73.1725398	70.928496	76.9311067
71.3734324	86.9289015	68.421441	73.1725398	70.928496	76.9311067
71.3734324	85.9289015	68.421441	73.1725398	70.928496	75.9311067
70.3734324	85.9289015	68.421441	73.1725398	70.928496	75.9311067
70.3734324	84.9289015	68.421441	70.1725398	70.928496	75.9311067
70.3734324	84.9289015	70.421441	69.1725398	71.928496	75.9311067
72.3734324	80.9289015	72.421441	66.1725398	74.928496	72.9311067
72.3734324	81.9289015	73.421441	64.1725398	74.928496	72.9311067
72.3734324	80.9289015	72.421441	63.1725398	74.928496	72.9311067
73.3734324	79.9289015	72.421441	63.1725398	75.928496	72.9311067
73.3734324	79.9289015	70.421441	64.1725398	74.928496	72.9311067
74.3734324	79.9289015	70.421441	65.1725398	73.928496	70.9311067
72.3734324	79.9289015	71.421441	68.1725398	72.928496	71.9311067
72.3734324	76.9289015	71.421441	69.1725398	72.928496	80.9311067
72.3734324	75.9289015	73.421441	69.1725398	72.928496	80.9311067
72.3734324	75.9289015	75.421441	69.1725398	72.928496	85.9311067
72.3734324	74.9289015	79.421441	73.1725398	72.928496	84.9311067
72.3734324	75.9289015	80.421441	73.1725398	73.928496	78.9311067
72.3734324	75.9289015	80.421441	73.1725398	74.928496	75.9311067
70.3734324	77.9289015	84.421441	71.1725398	73.928496	74.9311067
65.3734324	79.9289015	72.8508737	71.1725398	72.928496	74.9311067
64.3734324	79.9289015	72.7599646	71.1725398	71.928496	74.9311067
64.3734324	81.9289015	85.421441	71.1725398	70.928496	74.9311067
64.3734324	81.9289015	81.421441	71.1725398	70.928496	74.9311067
62.3734324	81.9289015	75.421441	77.1725398	71.928496	74.9311067
62.3734324	80.9289015	75.421441	80.1725398	71.928496	74.9311067
62.3734324	80.9289015	73.421441	81.1725398	74.928496	75.9311067
63.3734324	80.9289015	71.421441	81.1725398	76.928496	75.9311067
63.3734324	80.9289015	71.421441	81.1725398	76.928496	75.9311067
63.3734324	79.9289015	69.421441	81.1725398	76.928496	75.9311067
72.3734324	79.9289015	69.421441	80.1725398	77.928496	76.9311067
74.3734324	78.9289015	69.421441	79.1725398	75.928496	76.9311067

75.3734324	78.9289015	68.421441	79.1725398	76.928496	76.9311067
75.3734324	79.9289015	67.421441	71.1725398	76.928496	77.9311067
75.3734324	79.9289015	67.421441	67.1725398	76.928496	77.9311067
75.3734324	84.9289015	67.421441	67.1725398	76.928496	77.9311067
75.3734324	72.3963282	67.421441	66.1725398	77.928496	77.9311067
78.3734324	85.9289015	68.421441	67.1725398	75.928496	76.9311067
78.3734324	85.9289015	68.421441	67.1725398	74.928496	76.9311067
79.3734324	85.9289015	68.421441	66.1725398	73.928496	74.9311067
80.3734324	85.9289015	68.421441	66.1725398	70.928496	74.9311067
80.3734324	85.9289015	75.421441	64.1725398	67.928496	74.9311067
81.3734324	85.9289015	75.4872373	63.1725398	66.928496	73.9311067
81.3734324	84.9289015	75.8508737	63.1725398	65.928496	73.9311067
80.3734324	85.9289015	75.5781464	63.1725398	65.928496	73.9311067
80.3734324	85.9289015	76.0326919	65.1725398	66.928496	74.9311067
81.3734324	85.9289015	75.7599646	65.1725398	66.928496	74.9311067
81.3734324	86.9289015	76.6690555	68.1725398	73.928496	74.9311067
81.3734324	86.9289015	76.5781464	68.1725398	73.928496	75.9311067
81.3734324	86.9289015	76.5781464	68.1725398	74.928496	75.9311067
79.3734324	86.9289015	76.123601	67.1725398	73.928496	75.9311067
78.3734324	84.9289015	73.3054192	67.1725398	72.928496	76.9311067
78.3734324	72.0326919	75.421441	67.1725398	71.928496	77.9311067
76.3734324	71.6690555	75.421441	67.1725398	69.928496	78.9311067
76.3734324	71.8508737	75.421441	66.1725398	68.928496	79.9311067
76.3734324	83.9289015	76.421441	66.1725398	69.928496	81.9311067
76.3734324	82.9289015	76.421441	66.1725398	69.928496	81.9311067
76.3734324	79.9289015	77.421441	66.1725398	70.928496	80.9311067
76.3734324	79.9289015	76.421441	66.1725398	70.928496	80.9311067
75.3734324	74.9289015	76.421441	66.1725398	70.928496	80.9311067
73.3734324	71.9289015	75.421441	66.1725398	71.928496	80.9311067
74.3734324	70.9289015	75.421441	66.1725398	74.928496	80.9311067
74.3734324	70.9289015	75.421441	66.1725398	77.928496	80.9311067
74.3734324	70.9289015	75.421441	68.1725398	79.928496	80.9311067
75.3734324	69.9289015	74.421441	68.1725398	80.928496	73.9311067
76.3734324	69.9289015	72.421441	67.1725398	80.928496	73.9311067
76.3734324	69.9289015	72.421441	66.1725398	80.928496	73.9311067
76.3734324	69.9289015	70.421441	66.1725398	75.928496	72.9311067
76.3734324	70.9289015	70.421441	67.1725398	71.928496	69.9311067
76.3734324	68.9289015	70.421441	67.1725398	70.928496	68.9311067
76.3734324	66.9289015	69.421441	69.1725398	70.928496	68.9311067
76.3734324	66.9289015	69.421441	69.1725398	69.928496	68.9311067
76.3734324	64.9289015	69.421441	69.1725398	69.928496	67.9311067
75.3734324	64.9289015	68.421441	69.1725398	69.928496	67.9311067
76.3734324	64.9289015	67.421441	69.1725398	68.928496	64.9311067
77.3734324	64.9289015	66.421441	67.1725398	68.928496	64.9311067
77.3734324	64.9289015	66.421441	67.1725398	69.928496	64.9311067

77.3734324	64.9289015	66.421441	67.1725398	69.928496	64.9311067
79.3734324	64.9289015	66.421441	68.1725398	69.928496	64.9311067
79.3734324	64.9289015	65.421441	68.1725398	68.928496	68.9311067
79.3734324	64.9289015	65.421441	70.1725398	69.928496	68.9311067
79.3734324	64.9289015	65.421441	70.1725398	69.928496	68.9311067
79.3734324	64.9289015	65.421441	70.1725398	69.928496	68.9311067
76.3734324	67.9289015	65.421441	70.1725398	67.928496	67.9311067
75.3734324	67.9289015	65.421441	72.1725398	67.928496	68.9311067
75.3734324	68.9289015	65.421441	75.1725398	67.928496	67.9311067
75.3734324	70.9289015	65.421441	76.1725398	68.928496	67.9311067
74.3734324	70.9289015	65.421441	76.1725398	69.928496	66.9311067
74.3734324	71.9289015	65.421441	77.1725398	70.928496	65.9311067
73.3734324	72.9289015	65.421441	77.1725398	72.928496	65.9311067
73.3734324	72.9289015	74.421441	81.1725398	72.928496	65.9311067
72.3734324	73.9289015	74.421441	83.1725398	75.928496	65.9311067
71.3734324	73.9289015	74.421441	84.1725398	76.928496	65.9311067
71.3734324	73.9289015	77.421441	85.1725398	76.928496	64.9311067
70.3734324	73.9289015	77.421441	85.1725398	75.928496	64.9311067
69.3734324	73.9289015	81.421441	85.1725398	73.928496	67.9311067
66.3734324	74.9289015	81.421441	85.1725398	71.928496	69.9311067
66.3734324	72.9289015	81.421441	85.1725398	69.928496	69.9311067
66.3734324	70.9289015	80.421441	85.1725398	69.928496	70.9311067
66.3734324	70.9289015	80.421441	85.1725398	68.928496	78.9311067
66.3734324	70.9289015	78.421441	85.1725398	68.928496	78.9311067
66.3734324	71.9289015	78.421441	85.1725398	68.928496	81.9311067
66.3734324	72.9289015	78.421441	84.1725398	68.928496	81.9311067
68.3734324	72.9289015	75.421441	84.1725398	67.928496	81.9311067
69.3734324	72.9289015	74.421441	84.1725398	67.928496	81.9311067
69.3734324	74.9289015	75.421441	84.1725398	67.928496	81.9311067
69.3734324	75.9289015	76.421441	83.1725398	67.928496	81.9311067
68.3734324	75.9289015	76.421441	83.1725398	67.928496	81.9311067
67.3734324	74.9289015	78.421441	81.1725398	68.928496	81.9311067
67.3734324	73.9289015	78.421441	81.1725398	68.928496	83.9311067
67.3734324	74.9289015	77.421441	81.1725398	67.928496	83.9311067
66.3734324	74.9289015	77.421441	81.1725398	67.928496	83.9311067
66.3734324	74.9289015	77.421441	81.1725398	66.928496	83.9311067
63.3734324	75.9289015	74.421441	81.1725398	66.928496	83.9311067
63.3734324	75.9289015	74.421441	80.1725398	67.928496	83.9311067
63.3734324	75.9289015	74.421441	80.1725398	67.928496	83.9311067
62.3734324	75.9289015	73.421441	78.1725398	73.928496	83.9311067
62.3734324	75.9289015	73.421441	77.1725398	79.928496	83.9311067
62.3734324	75.9289015	73.421441	77.1725398	81.928496	83.9311067
62.3734324	75.9289015	73.421441	77.1725398	80.928496	77.9311067
62.3734324	75.9289015	73.421441	77.1725398	76.928496	77.9311067
62.3734324	75.9289015	73.421441	76.1725398	73.928496	77.9311067

62.3734324	75.9289015	77.421441	75.1725398	70.928496	77.9311067
62.3734324	75.9289015	78.421441	75.1725398	69.928496	77.9311067
64.3734324	76.9289015	79.421441	75.1725398	69.928496	76.9311067
64.3734324	76.9289015	80.421441	75.1725398	69.928496	75.9311067
64.3734324	76.9289015	79.421441	75.1725398	69.928496	75.9311067
66.3734324	78.9289015	78.421441	77.1725398	69.928496	75.9311067
67.3734324	79.9289015	78.421441	76.1725398	68.928496	75.9311067
67.3734324	79.9289015	78.421441	76.1725398	68.928496	75.9311067
67.3734324	79.9289015	78.421441	76.1725398	68.928496	76.9311067
67.3734324	80.9289015	80.421441	78.1725398	68.928496	78.9311067
67.3734324	80.9289015	80.421441	79.1725398	67.928496	78.9311067
67.3734324	80.9289015	81.421441	79.1725398	66.928496	77.9311067
67.3734324	80.9289015	80.421441	79.1725398	66.928496	77.9311067
67.3734324	79.9289015	80.421441	78.1725398	65.928496	77.9311067
67.3734324	79.9289015	80.421441	78.1725398	65.928496	77.9311067
67.3734324	81.9289015	80.421441	77.1725398	65.928496	77.9311067
68.3734324	81.9289015	78.421441	77.1725398	64.928496	77.9311067
68.3734324	81.9289015	78.421441	79.1725398	64.928496	77.9311067
68.3734324	80.9289015	79.421441	79.1725398	64.928496	77.9311067
68.3734324	80.9289015	79.421441	79.1725398	64.928496	77.9311067
67.3734324	81.9289015	79.421441	79.1725398	64.928496	78.9311067
68.3734324	79.9289015	78.421441	79.1725398	64.928496	78.9311067
68.3734324	77.9289015	77.421441	79.1725398	65.928496	79.9311067
69.3734324	75.9289015	78.421441	80.1725398	65.928496	79.9311067
69.3734324	75.9289015	78.421441	80.1725398	66.928496	78.9311067
69.3734324	74.9289015	79.421441	80.1725398	67.928496	78.9311067
69.3734324	74.9289015	81.421441	80.1725398	66.928496	77.9311067
70.3734324	74.9289015	81.421441	80.1725398	66.928496	77.9311067
72.3734324	74.9289015	82.421441	80.1725398	67.928496	77.9311067
74.3734324	74.9289015	81.421441	78.1725398	67.928496	77.9311067
74.3734324	74.9289015	80.421441	78.1725398	67.928496	77.9311067
74.3734324	74.9289015	80.421441	77.1725398	67.928496	76.9311067
74.3734324	75.9289015	77.421441	76.1725398	67.928496	76.9311067
74.3734324	75.9289015	74.421441	76.1725398	68.928496	76.9311067
74.3734324	75.9289015	74.421441	74.1725398	68.928496	76.9311067
73.3734324	75.9289015	74.421441	75.1725398	68.928496	78.9311067
72.3734324	75.9289015	74.421441	76.1725398	68.928496	78.9311067
72.3734324	75.9289015	74.421441	77.1725398	69.928496	78.9311067
72.3734324	75.9289015	74.421441	78.1725398	74.928496	78.9311067
70.3734324	75.9289015	71.421441	78.1725398	77.928496	78.9311067
70.3734324	75.9289015	70.421441	78.1725398	78.928496	78.9311067
71.3734324	78.9289015	70.421441	74.1725398	78.928496	82.9311067
71.3734324	82.9289015	70.421441	72.1725398	78.928496	83.9311067
71.3734324	81.9289015	71.421441	70.1725398	78.928496	83.9311067
70.3734324	79.9289015	71.421441	69.1725398	78.928496	84.9311067

70.3734324	79.9289015	71.421441	70.1725398	78.928496	73.0326919
70.3734324	73.9289015	71.421441	72.1725398	79.928496	82.9311067
70.3734324	69.9289015	71.421441	73.1725398	79.928496	82.9311067
70.3734324	67.9289015	71.421441	75.1725398	80.928496	81.9311067
68.3734324	67.9289015	71.421441	76.1725398	80.928496	79.9311067
68.3734324	69.9289015	71.421441	76.1725398	68.928496	79.9311067
65.3734324	70.9289015	70.421441	76.1725398	66.928496	77.9311067
64.3734324	70.9289015	67.421441	74.1725398	65.928496	77.9311067
64.3734324	72.9289015	65.421441	74.1725398	65.928496	77.9311067
64.3734324	74.9289015	65.421441	71.1725398	65.928496	77.9311067
64.3734324	75.9289015	59.421441	70.1725398	64.928496	77.9311067
64.3734324	75.9289015	58.421441	70.1725398	63.928496	77.9311067
64.3734324	74.9289015	57.421441	71.1725398	63.928496	77.9311067
64.3734324	74.9289015	57.421441	71.1725398	63.928496	77.9311067
64.3734324	74.9289015	57.421441	72.1725398	63.928496	77.9311067
64.3734324	74.9289015	58.421441	74.1725398	63.928496	77.9311067
64.3734324	74.9289015	56.421441	76.1725398	63.928496	79.9311067
72.3734324	74.9289015	56.421441	77.1725398	63.928496	79.9311067
73.3734324	74.9289015	56.421441	77.1725398	63.928496	79.9311067
73.3734324	74.9289015	55.421441	75.1725398	63.928496	79.9311067
73.3734324	74.9289015	56.421441	75.1725398	68.928496	80.9311067
74.3734324	74.9289015	56.421441	74.1725398	69.928496	80.9311067
75.3734324	74.9289015	58.421441	74.1725398	71.928496	82.9311067
75.3734324	74.9289015	58.421441	74.1725398	71.928496	82.9311067
75.3734324	75.9289015	60.421441	74.1725398	76.928496	74.9417828
75.3734324	75.9289015	63.421441	73.1725398	77.928496	75.3963282
74.3734324	77.9289015	63.421441	73.1725398	75.928496	75.5781464
74.3734324	77.9289015	67.421441	73.1725398	75.928496	76.0326919
74.3734324	77.9289015	68.421441	73.1725398	75.928496	76.3054192
74.3734324	77.9289015	68.421441	72.1725398	75.928496	75.8508737
73.3734324	77.9289015	73.421441	72.1725398	73.928496	75.9417828
73.3734324	78.9289015	73.421441	72.1725398	73.928496	75.2145101
72.3734324	78.9289015	73.421441	72.1725398	72.928496	74.5781464
72.3734324	78.9289015	74.421441	72.1725398	72.928496	74.6690555
72.3734324	78.9289015	74.421441	72.1725398	72.928496	74.6690555
73.3734324	78.9289015	75.421441	71.1725398	72.928496	74.6690555
73.3734324	78.9289015	77.421441	69.1725398	72.928496	86.9311067
74.3734324	77.9289015	80.421441	68.1725398	73.928496	73.9417828
75.3734324	77.9289015	80.421441	68.1725398	74.928496	74.0326919
75.3734324	75.9289015	81.421441	68.1725398	77.928496	73.9417828
75.3734324	74.9289015	81.421441	68.1725398	79.928496	86.9311067
75.3734324	73.9289015	81.421441	68.1725398	79.928496	86.9311067
76.3734324	73.9289015	82.421441	68.1725398	79.928496	86.9311067
76.3734324	73.9289015	82.421441	69.1725398	74.928496	73.5781464
76.3734324	73.9289015	84.421441	69.1725398	73.928496	73.6690555

76.3734324	74.9289015	85.421441	70.1725398	71.928496	73.5781464
76.3734324	74.9289015	85.421441	73.1725398	70.928496	73.5781464
76.3734324	73.9289015	73.7599646	73.1725398	70.928496	73.7599646
75.3734324	73.9289015	73.8508737	74.1725398	71.928496	73.8508737
74.3734324	74.9289015	85.421441	74.1725398	71.928496	73.6690555
74.3734324	77.9289015	79.421441	74.1725398	72.928496	73.7599646
74.3734324	82.9289015	78.421441	73.1725398	72.928496	74.123601
75.3734324	85.9289015	78.421441	73.1725398	75.928496	74.8508737
74.3734324	85.9289015	78.421441	73.1725398	77.928496	75.5781464
74.3734324	86.9289015	78.421441	73.1725398	78.928496	86.9311067
74.3734324	86.9289015	79.421441	72.1725398	80.928496	84.9311067
74.3734324	86.9289015	80.421441	72.1725398	81.928496	84.9311067
71.3734324	75.8508737	80.421441	72.1725398	81.928496	84.9311067
71.3734324	75.3054192	80.421441	73.1725398	78.928496	82.9311067
71.3734324	74.9417828	79.421441	75.1725398	75.928496	81.9311067
71.3734324	74.6690555	80.421441	75.1725398	74.928496	81.9311067
71.3734324	74.123601	80.421441	75.1725398	74.928496	79.9311067
71.3734324	85.9289015	81.421441	76.1725398	76.928496	79.9311067
71.3734324	85.9289015	84.421441	76.1725398	78.928496	80.9311067
71.3734324	85.9289015	84.421441	76.1725398	79.928496	81.9311067
76.3734324	85.9289015	85.421441	76.1725398	79.928496	77.9311067
79.3734324	85.9289015	85.421441	76.1725398	79.928496	77.9311067
81.3734324	85.9289015	84.421441	75.1725398	77.928496	75.9311067
81.3734324	85.9289015	82.421441	76.1725398	77.928496	75.9311067
82.3734324	86.9289015	82.421441	76.1725398	77.928496	75.9311067
83.3734324	86.9289015	80.421441	81.1725398	75.928496	73.9311067
83.3734324	74.0326919	79.421441	81.1725398	72.928496	73.9311067
85.3734324	73.8508737	78.421441	79.1725398	70.928496	73.9311067
85.3734324	73.5781464	78.421441	79.1725398	69.928496	72.9311067
85.3734324	72.9417828	76.421441	78.1725398	68.928496	70.9311067
85.3734324	72.7599646	74.421441	78.1725398	67.928496	70.9311067
72.3963282	72.3963282	72.421441	78.1725398	66.928496	69.9311067
72.3963282	72.3963282	72.421441	78.1725398	66.928496	69.9311067
83.3734324	71.2145101	70.421441	77.1725398	64.928496	69.9311067
81.3734324	81.9289015	70.421441	79.1725398	64.928496	69.9311067
81.3734324	75.9289015	67.421441	78.1725398	64.928496	69.9311067
81.3734324	69.9289015	66.421441	76.1725398	64.928496	69.9311067
80.3734324	68.9289015	66.421441	76.1725398	64.928496	68.9311067
80.3734324	69.9289015	66.421441	75.1725398	69.928496	66.9311067
80.3734324	71.9289015	66.421441	75.1725398	74.928496	64.9311067
79.3734324	73.9289015	66.421441	75.1725398	74.928496	63.9311067
79.3734324	73.9289015	66.421441	75.1725398	78.928496	63.9311067
79.3734324	74.9289015	67.421441	75.1725398	78.928496	64.9311067
79.3734324	76.9289015	67.421441	75.1725398	74.928496	64.9311067
81.3734324	77.9289015	67.421441	75.1725398	71.928496	63.9311067

81.3734324	77.9289015	67.421441	75.1725398	70.928496	63.9311067
81.3734324	76.9289015	67.421441	72.1725398	69.928496	63.9311067
80.3734324	76.9289015	68.421441	71.1725398	69.928496	66.9311067
80.3734324	76.9289015	69.421441	70.1725398	69.928496	66.9311067
80.3734324	76.9289015	68.421441	69.1725398	69.928496	67.9311067
80.3734324	76.9289015	68.421441	69.1725398	69.928496	67.9311067
80.3734324	76.9289015	68.421441	69.1725398	71.928496	67.9311067
80.3734324	76.9289015	68.421441	67.1725398	74.928496	67.9311067
80.3734324	76.9289015	71.421441	67.1725398	75.928496	67.9311067
79.3734324	75.9289015	72.421441	67.1725398	75.928496	67.9311067
79.3734324	75.9289015	72.421441	67.1725398	75.928496	72.9311067
74.3734324	75.9289015	72.421441	69.1725398	73.928496	72.9311067
73.3734324	77.9289015	73.421441	69.1725398	72.928496	72.9311067
73.3734324	77.9289015	74.421441	69.1725398	72.928496	72.9311067
73.3734324	77.9289015	74.421441	70.1725398	72.928496	72.9311067
72.3734324	77.9289015	74.421441	70.1725398	71.928496	72.9311067
72.3734324	77.9289015	69.421441	71.1725398	70.928496	70.9311067
72.3734324	77.9289015	69.421441	73.1725398	71.928496	68.9311067
72.3734324	79.9289015	65.421441	75.1725398	71.928496	67.9311067
72.3734324	79.9289015	65.421441	74.1725398	71.928496	67.9311067
72.3734324	80.9289015	66.421441	74.1725398	71.928496	66.9311067
72.3734324	80.9289015	66.421441	73.1725398	71.928496	66.9311067
72.3734324	78.9289015	66.421441	73.1725398	71.928496	66.9311067
72.3734324	78.9289015	66.421441	72.1725398	72.928496	66.9311067
72.3734324	76.9289015	66.421441	71.1725398	72.928496	66.9311067
72.3734324	76.9289015	67.421441	71.1725398	73.928496	66.9311067
72.3734324	76.9289015	67.421441	71.1725398	73.928496	69.9311067
72.3734324	79.9289015	67.421441	74.1725398	74.928496	69.9311067
72.3734324	79.9289015	68.421441	77.1725398	75.928496	67.9311067
72.3734324	79.9289015	69.421441	77.1725398	78.928496	67.9311067
70.3734324	78.9289015	71.421441	75.1725398	80.928496	67.9311067
70.3734324	78.9289015	71.421441	74.1725398	82.928496	67.9311067
70.3734324	75.9289015	72.421441	74.1725398	83.928496	67.9311067
70.3734324	75.9289015	73.421441	73.1725398	82.928496	69.9311067
72.3734324	75.9289015	73.421441	73.1725398	75.928496	70.9311067
73.3734324	74.9289015	81.421441	73.1725398	73.928496	70.9311067
73.3734324	76.9289015	81.421441	73.1725398	73.928496	70.9311067
72.3734324	76.9289015	81.421441	75.1725398	72.928496	70.9311067
72.3734324	76.9289015	80.421441	76.1725398	71.928496	70.9311067
73.3734324	76.9289015	80.421441	75.1725398	71.928496	71.9311067
73.3734324	76.9289015	80.421441	75.1725398	71.928496	73.9311067
73.3734324	76.9289015	80.421441	75.1725398	71.928496	73.9311067
73.3734324	72.9289015	80.421441	74.1725398	71.928496	73.9311067
74.3734324	72.9289015	80.421441	74.1725398	71.928496	72.9311067
74.3734324	73.9289015	80.421441	74.1725398	71.928496	71.9311067

74.3734324	73.9289015	80.421441	73.1725398	71.928496	71.9311067
74.3734324	74.9289015	79.421441	74.1725398	70.928496	72.9311067
75.3734324	76.9289015	79.421441	75.1725398	71.928496	73.9311067
75.3734324	76.9289015	79.421441	77.1725398	71.928496	73.9311067
75.3734324	76.9289015	79.421441	77.1725398	75.928496	74.9311067
75.3734324	77.9289015	79.421441	77.1725398	76.928496	74.9311067
76.3734324	77.9289015	79.421441	76.1725398	76.928496	74.9311067
76.3734324	77.9289015	78.421441	74.1725398	73.928496	74.9311067
80.3734324	76.9289015	77.421441	73.1725398	72.928496	74.9311067
81.3734324	77.9289015	77.421441	74.1725398	72.928496	74.9311067
83.3734324	78.9289015	76.421441	74.1725398	72.928496	74.9311067
83.3734324	78.9289015	76.421441	74.1725398	72.928496	74.9311067
74.5781464	78.9289015	76.421441	75.1725398	71.928496	74.9311067
74.7599646	78.9289015	76.421441	75.1725398	71.928496	73.9311067
74.8508737	78.9289015	76.421441	75.1725398	71.928496	71.9311067
74.9417828	77.9289015	76.421441	76.1725398	71.928496	69.9311067
75.0326919	77.9289015	75.421441	76.1725398	71.928496	69.9311067
74.7599646	78.9289015	76.421441	76.1725398	72.928496	69.9311067
75.0326919	78.9289015	76.421441	76.1725398	72.928496	69.9311067
75.3054192	78.9289015	76.421441	78.1725398	72.928496	69.9311067
75.3054192	78.9289015	73.421441	78.1725398	73.928496	70.9311067
75.2145101	77.9289015	73.421441	79.1725398	71.928496	69.9311067
75.2145101	77.9289015	71.421441	79.1725398	69.928496	69.9311067
75.2145101	77.9289015	71.421441	77.1725398	69.928496	70.9311067
75.2145101	77.9289015	71.421441	76.1725398	69.928496	69.9311067
75.3054192	77.9289015	71.421441	74.1725398	69.928496	69.9311067
75.4872373	76.9289015	71.421441	74.1725398	70.928496	70.9311067
75.6690555	78.9289015	71.421441	74.1725398	70.928496	69.9311067
75.6690555	78.9289015	71.421441	73.1725398	70.928496	70.9311067
75.6690555	79.9289015	71.421441	73.1725398	69.928496	70.9311067
75.2145101	79.9289015	71.421441	73.1725398	69.928496	69.9311067
74.5781464	79.9289015	69.421441	73.1725398	70.928496	69.9311067
73.7599646	79.9289015	66.421441	73.1725398	70.928496	68.9311067
73.4872373	79.9289015	64.421441	72.1725398	72.928496	69.9311067
74.123601	79.9289015	69.421441	71.1725398	73.928496	72.9311067
74.2145101	79.9289015	69.421441	71.1725398	73.928496	72.9311067
74.7599646	80.9289015	73.421441	71.1725398	71.928496	72.9311067
75.0326919	80.9289015	73.421441	72.1725398	71.928496	74.9311067
74.6690555	82.9289015	72.421441	72.1725398	68.928496	74.9311067
74.6690555	84.9289015	71.421441	72.1725398	68.928496	74.9311067
75.123601	84.9289015	71.421441	73.1725398	70.928496	76.9311067
75.3054192	80.9289015	72.421441	73.1725398	72.928496	77.9311067
76.0326919	79.9289015	75.421441	74.1725398	75.928496	81.9311067
76.2145101	77.9289015	75.421441	77.1725398	76.928496	81.9311067
76.2145101	77.9289015	76.421441	77.1725398	78.928496	83.9311067

86.3734324	77.9289015	77.421441	76.1725398	79.928496	84.9311067
84.3734324	77.9289015	77.421441	74.1725398	80.928496	84.9311067
84.3734324	77.9289015	78.421441	72.1725398	80.928496	84.9311067
84.3734324	77.9289015	78.421441	72.1725398	79.928496	84.9311067
83.3734324	76.9289015	79.421441	71.1725398	74.928496	83.9311067
83.3734324	76.9289015	79.421441	71.1725398	74.928496	83.9311067
83.3734324	76.9289015	79.421441	72.1725398	70.928496	80.9311067
82.3734324	82.9289015	80.421441	72.1725398	69.928496	80.9311067
82.3734324	84.9289015	79.421441	72.1725398	69.928496	78.9311067
82.3734324	84.9289015	79.421441	70.1725398	69.928496	75.9311067
84.3734324	84.9289015	79.421441	69.1725398	69.928496	73.9311067
84.3734324	84.9289015	78.421441	68.1725398	69.928496	73.9311067
84.3734324	84.9289015	78.421441	69.1725398	68.928496	70.9311067
85.3734324	84.9289015	78.421441	69.1725398	68.928496	70.9311067
85.3734324	83.9289015	78.421441	71.1725398	68.928496	71.9311067
85.3734324	83.9289015	78.421441	72.1725398	69.928496	71.9311067
85.3734324	83.9289015	77.421441	73.1725398	69.928496	71.9311067
85.3734324	83.9289015	79.421441	74.1725398	69.928496	71.9311067
76.3054192	84.9289015	82.421441	74.1725398	68.928496	72.9311067
76.5781464	85.9289015	82.421441	74.1725398	66.928496	74.9311067
76.8508737	85.9289015	85.421441	74.1725398	65.928496	74.9311067
77.2145101	85.9289015	88.421441	74.1725398	64.928496	77.9311067
77.3054192	86.9289015	87.421441	76.1725398	63.928496	80.9311067
77.7599646	86.9289015	86.421441	76.1725398	63.928496	82.9311067
77.0326919	86.9289015	80.421441	77.1725398	63.928496	82.9311067
77.0326919	86.9289015	80.421441	76.1725398	63.928496	84.9311067
76.7599646	85.9289015	75.421441	76.1725398	63.928496	84.9311067
86.3734324	85.9289015	74.421441	76.1725398	63.928496	85.9311067
78.3734324	85.9289015	74.421441	76.1725398	63.928496	85.9311067
77.3734324	86.9289015	73.421441	75.1725398	63.928496	85.9311067
77.3734324	74.5781464	72.421441	75.1725398	64.928496	85.9311067
76.3734324	74.3963282	71.421441	75.1725398	64.928496	86.9311067
76.3734324	74.8508737	71.421441	76.1725398	67.928496	74.8508737
76.3734324	74.7599646	70.421441	76.1725398	67.928496	74.7599646
76.3734324	74.8508737	71.421441	75.1725398	67.928496	74.8508737
75.3734324	74.9417828	72.421441	76.1725398	68.928496	74.9417828
77.3734324	75.3963282	73.421441	76.1725398	68.928496	75.3963282
77.3734324	75.5781464	73.421441	77.1725398	68.928496	75.5781464
78.3734324	76.2145101	73.421441	79.1725398	69.928496	76.2145101
79.3734324	76.0326919	72.421441	80.1725398	69.928496	76.0326919
79.3734324	76.123601	72.421441	80.1725398	71.928496	76.123601
79.3734324	75.7599646	72.421441	80.1725398	72.928496	75.7599646
79.3734324	75.7599646	73.421441	80.1725398	72.928496	75.7599646
79.3734324	75.5781464	73.421441	80.1725398	72.928496	75.5781464
79.3734324	87.9289015	73.421441	76.1725398	72.928496	75.2145101

80.3734324	85.9289015	73.421441	76.1725398	72.928496	75.3054192
80.3734324	83.9289015	73.421441	77.1725398	73.928496	75.123601
80.3734324	82.9289015	73.421441	77.1725398	75.928496	75.3054192
80.3734324	81.9289015	73.421441	75.1725398	75.928496	74.6690555
82.3734324	81.9289015	73.421441	75.1725398	76.928496	74.9417828
82.3734324	81.9289015	73.421441	74.1725398	73.928496	74.3963282
82.3734324	82.9289015	72.421441	74.1725398	73.928496	74.3054192
80.3734324	81.9289015	72.421441	73.1725398	73.928496	74.123601
79.3734324	80.9289015	72.421441	73.1725398	70.928496	73.7599646
79.3734324	79.9289015	72.421441	74.1725398	70.928496	73.9417828
78.3734324	78.9289015	71.421441	74.1725398	71.928496	73.8508737
76.3734324	78.9289015	71.421441	75.1725398	70.928496	73.9417828
76.3734324	79.9289015	70.421441	75.1725398	70.928496	74.0326919
76.3734324	80.9289015	70.421441	75.1725398	69.928496	83.9311067
76.3734324	80.9289015	70.421441	75.1725398	69.928496	83.9311067
75.3734324	80.9289015	70.421441	75.1725398	68.928496	77.9311067
75.3734324	79.9289015	69.421441	75.1725398	68.928496	76.9311067
75.3734324	79.9289015	69.421441	75.1725398	73.928496	75.9311067
75.3734324	80.9289015	69.421441	75.1725398	74.928496	74.9311067
75.3734324	80.9289015	68.421441	75.1725398	74.928496	74.9311067
75.3734324	80.9289015	68.421441	75.1725398	74.928496	74.9311067
75.3734324	80.9289015	68.421441	75.1725398	74.928496	74.9311067
76.3734324	80.9289015	68.421441	74.1725398	74.928496	74.9311067
75.3734324	80.9289015	68.421441	73.1725398	74.928496	74.9311067
75.3734324	80.9289015	72.421441	73.1725398	71.928496	74.9311067
75.3734324	80.9289015	72.421441	73.1725398	69.928496	75.9311067
75.3734324	81.9289015	73.421441	72.1725398	68.928496	75.9311067
75.3734324	81.9289015	75.421441	72.1725398	68.928496	76.9311067
75.3734324	82.9289015	75.421441	72.1725398	68.928496	75.9311067
76.3734324	82.9289015	77.421441	72.1725398	68.928496	75.9311067
76.3734324	84.9289015	77.421441	73.1725398	68.928496	75.9311067
76.3734324	84.9289015	77.421441	73.1725398	68.928496	75.9311067
76.3734324	84.9289015	77.421441	75.1725398	68.928496	78.9311067
76.3734324	84.9289015	77.421441	81.1725398	69.928496	79.9311067
76.3734324	82.9289015	75.421441	81.1725398	69.928496	79.9311067
76.3734324	82.9289015	75.421441	81.1725398	71.928496	78.9311067
76.3734324	82.9289015	75.421441	77.1725398	72.928496	78.9311067
77.3734324	83.9289015	74.421441	76.1725398	73.928496	75.9311067
77.3734324	83.9289015	74.421441	75.1725398	73.928496	75.9311067
77.3734324	84.9289015	72.421441	75.1725398	73.928496	76.9311067
77.3734324	84.9289015	72.421441	75.1725398	72.928496	76.9311067
77.3734324	84.9289015	71.421441	75.1725398	69.928496	76.9311067
77.3734324	84.9289015	71.421441	77.1725398	69.928496	76.9311067
77.3734324	82.9289015	71.421441	78.1725398	69.928496	76.9311067
78.3734324	81.9289015	71.421441	78.1725398	69.928496	76.9311067

78.3734324	81.9289015	71.421441	78.1725398	69.928496	77.9311067
77.3734324	81.9289015	72.421441	78.1725398	69.928496	77.9311067
72.3734324	78.9289015	72.421441	78.1725398	69.928496	82.9311067
72.3734324	76.9289015	72.421441	77.1725398	70.928496	83.9311067
71.3734324	75.9289015	73.421441	78.1725398	75.928496	85.9311067
71.3734324	75.9289015	73.421441	79.1725398	77.928496	85.9311067
71.3734324	74.9289015	73.421441	79.1725398	77.928496	85.9311067
71.3734324	74.9289015	73.421441	78.1725398	77.928496	85.9311067
70.3734324	75.9289015	74.421441	75.1725398	77.928496	84.9311067
70.3734324	77.9289015	71.421441	74.1725398	76.928496	84.9311067
67.3734324	79.9289015	71.421441	74.1725398	75.928496	80.9311067
68.3734324	80.9289015	71.421441	74.1725398	75.928496	71.9311067
70.3734324	79.9289015	71.421441	73.1725398	73.928496	70.9311067
71.3734324	77.9289015	71.421441	73.1725398	73.928496	70.9311067
71.3734324	76.9289015	71.421441	74.1725398	73.928496	70.9311067
79.3734324	76.9289015	71.421441	73.1725398	73.928496	70.9311067
80.3734324	75.9289015	71.421441	73.1725398	73.928496	70.9311067
80.3734324	75.9289015	71.421441	67.1725398	73.928496	70.9311067
80.3734324	75.9289015	72.421441	66.1725398	74.928496	70.9311067
80.3734324	75.9289015	72.421441	65.1725398	76.928496	70.9311067
80.3734324	75.9289015	72.421441	65.1725398	76.928496	70.9311067
80.3734324	75.9289015	72.421441	65.1725398	76.928496	70.9311067
80.3734324	77.9289015	72.421441	64.1725398	76.928496	71.9311067
80.3734324	77.9289015	72.421441	64.1725398	76.928496	71.9311067
82.3734324	77.9289015	72.421441	66.1725398	73.928496	73.9311067
82.3734324	79.9289015	73.421441	66.1725398	71.928496	73.9311067
81.3734324	77.9289015	73.421441	67.1725398	71.928496	74.9311067
81.3734324	74.9289015	73.421441	68.1725398	71.928496	74.9311067
81.3734324	72.9289015	75.421441	69.1725398	71.928496	74.9311067
81.3734324	72.9289015	77.421441	71.1725398	71.928496	74.9311067
80.3734324	72.9289015	79.421441	72.1725398	71.928496	74.9311067
80.3734324	72.9289015	79.421441	75.1725398	71.928496	75.9311067
79.3734324	72.9289015	79.421441	77.1725398	71.928496	75.9311067
79.3734324	72.9289015	75.421441	77.1725398	71.928496	74.9311067
79.3734324	72.9289015	74.421441	76.1725398	71.928496	74.9311067
77.3734324	78.9289015	74.421441	75.1725398	70.928496	72.9311067
80.3734324	78.9289015	73.421441	75.1725398	70.928496	72.9311067
85.3734324	79.9289015	73.421441	76.1725398	70.928496	72.9311067
85.3734324	79.9289015	74.421441	76.1725398	71.928496	72.9311067
87.3734324	82.9289015	74.421441	76.1725398	73.928496	70.9311067
87.3734324	83.9289015	74.421441	76.1725398	74.928496	70.9311067
88.3734324	85.9289015	73.421441	76.1725398	75.928496	69.9311067
76.123601	86.9289015	73.421441	77.1725398	75.928496	69.9311067
76.3054192	87.9289015	71.421441	76.1725398	76.928496	68.9311067
76.3054192	87.9289015	70.421441	77.1725398	76.928496	68.9311067

76.3054192	86.9289015	70.421441	77.1725398	77.928496	69.9311067
76.3054192	85.9289015	70.421441	76.1725398	77.928496	69.9311067
75.4872373	81.9289015	70.421441	75.1725398	77.928496	69.9311067
76.0326919	80.9289015	70.421441	75.1725398	78.928496	69.9311067
75.9417828	80.9289015	70.421441	75.1725398	78.928496	69.9311067
76.3963282	78.9289015	70.421441	79.1725398	78.928496	70.9311067
76.0326919	78.9289015	70.421441	79.1725398	76.928496	71.9311067
75.9417828	78.9289015	70.421441	79.1725398	76.928496	71.9311067
76.3054192	78.9289015	70.421441	79.1725398	78.928496	71.9311067
76.4872373	78.9289015	69.421441	78.1725398	79.928496	73.9311067
76.3963282	78.9289015	68.421441	77.1725398	80.928496	73.9311067
75.9417828	78.9289015	68.421441	75.1725398	81.928496	73.9311067
75.5781464	78.9289015	67.421441	75.1725398	81.928496	73.9311067
75.5781464	78.9289015	66.421441	75.1725398	80.928496	74.9311067
74.4872373	78.9289015	66.421441	77.1725398	72.928496	73.9311067
73.8508737	78.9289015	66.421441	77.1725398	72.928496	72.9311067
73.3054192	78.9289015	66.421441	78.1725398	68.928496	72.9311067
82.3734324	78.9289015	66.421441	78.1725398	68.928496	72.9311067
78.3734324	78.9289015	66.421441	78.1725398	67.928496	72.9311067
78.3734324	77.9289015	66.421441	78.1725398	67.928496	71.9311067
78.3734324	77.9289015	66.421441	78.1725398	67.928496	71.9311067
77.3734324	75.9289015	66.421441	77.1725398	67.928496	71.9311067
76.3734324	73.9289015	66.421441	77.1725398	68.928496	71.9311067
75.3734324	72.9289015	66.421441	76.1725398	68.928496	70.9311067
74.3734324	72.9289015	66.421441	76.1725398	68.928496	70.9311067
74.3734324	71.9289015	66.421441	78.1725398	68.928496	69.9311067
74.3734324	71.9289015	66.421441	78.1725398	68.928496	68.9311067
74.3734324	71.9289015	66.421441	78.1725398	69.928496	67.9311067
75.3734324	71.9289015	66.421441	79.1725398	71.928496	67.9311067
75.3734324	71.9289015	65.421441	79.1725398	74.928496	63.9311067
76.3734324	71.9289015	65.421441	76.1725398	74.928496	61.9311067
76.3734324	69.9289015	66.421441	76.1725398	74.928496	61.9311067
76.3734324	69.9289015	66.421441	76.1725398	74.928496	61.9311067
77.3734324	69.9289015	67.421441	74.1725398	72.928496	60.9311067
77.3734324	67.9289015	67.421441	74.1725398	71.928496	60.9311067
77.3734324	67.9289015	68.421441	74.1725398	72.928496	60.9311067
79.3734324	67.9289015	68.421441	76.1725398	72.928496	60.9311067
82.3734324	67.9289015	68.421441	79.1725398	73.928496	61.9311067
82.3734324	68.9289015	68.421441	79.1725398	79.928496	61.9311067
82.3734324	69.9289015	69.421441	79.1725398	80.928496	63.9311067
82.3734324	70.9289015	69.421441	77.1725398	81.928496	66.9311067
83.3734324	69.9289015	70.421441	77.1725398	82.928496	70.9311067
83.3734324	69.9289015	72.421441	74.1725398	82.928496	71.9311067
83.3734324	69.9289015	75.421441	74.1725398	81.928496	71.9311067
83.3734324	70.9289015	75.421441	75.1725398	77.928496	72.9311067

84.3734324	72.9289015	76.421441	77.1725398	77.928496	73.9311067
85.3734324	73.9289015	79.421441	78.1725398	74.928496	73.9311067
85.3734324	73.9289015	80.421441	78.1725398	73.928496	74.9311067
81.3734324	73.9289015	80.421441	78.1725398	73.928496	74.9311067
82.3734324	73.9289015	80.421441	78.1725398	75.928496	77.9311067
82.3734324	75.9289015	79.421441	78.1725398	75.928496	81.9311067
80.3734324	76.9289015	74.421441	78.1725398	76.928496	81.9311067
78.3734324	76.9289015	74.421441	79.1725398	72.928496	81.9311067
78.3734324	74.9289015	74.421441	79.1725398	72.928496	81.9311067
78.3734324	74.9289015	73.421441	85.1725398	68.928496	80.9311067
78.3734324	74.9289015	73.421441	84.1725398	67.928496	80.9311067
77.3734324	74.9289015	73.421441	83.1725398	66.928496	81.9311067
77.3734324	74.9289015	73.421441	82.1725398	67.928496	81.9311067
76.3734324	74.9289015	74.421441	81.1725398	68.928496	81.9311067
76.3734324	74.9289015	75.421441	81.1725398	69.928496	81.9311067
76.3734324	75.9289015	76.421441	81.1725398	69.928496	81.9311067
76.3734324	76.9289015	76.421441	81.1725398	69.928496	80.9311067
76.3734324	76.9289015	81.421441	82.1725398	69.928496	80.9311067
76.3734324	76.9289015	85.421441	82.1725398	69.928496	79.9311067
76.3734324	69.9289015	73.2145101	82.1725398	69.928496	79.9311067
76.3734324	69.9289015	73.0326919	83.1725398	69.928496	79.9311067
76.3734324	68.9289015	73.123601	83.1725398	69.928496	79.9311067
76.3734324	69.9289015	72.8508737	83.1725398	69.928496	79.9311067
75.3734324	70.9289015	72.8508737	82.1725398	69.928496	79.9311067
75.3734324	70.9289015	85.421441	83.1725398	69.928496	80.9311067
77.3734324	74.9289015	85.421441	83.1725398	68.928496	80.9311067
77.3734324	77.9289015	85.421441	83.1725398	68.928496	80.9311067
78.3734324	79.9289015	85.421441	81.1725398	68.928496	82.9311067
78.3734324	77.9289015	85.421441	81.1725398	68.928496	83.9311067
79.3734324	77.9289015	85.421441	81.1725398	69.928496	83.9311067
79.3734324	76.9289015	85.421441	80.1725398	70.928496	85.9311067
79.3734324	76.9289015	84.421441	80.1725398	70.928496	85.9311067
74.7599646	76.9289015	83.421441	75.1725398	71.928496	85.9311067
74.5781464	76.9289015	82.421441	75.1725398	71.928496	85.9311067
74.5781464	76.9289015	82.421441	76.1725398	71.928496	85.9311067
74.2145101	75.9289015	78.421441	75.1725398	72.928496	86.9311067
74.2145101	75.9289015	77.421441	76.1725398	73.928496	85.9311067
85.3734324	75.9289015	75.421441	76.1725398	78.928496	81.9311067
84.3734324	74.9289015	75.421441	76.1725398	81.928496	74.9311067
84.3734324	74.9289015	75.421441	76.1725398	82.928496	74.9311067
81.3734324	73.9289015	75.421441	76.1725398	82.928496	74.9311067
81.3734324	73.9289015	75.421441	76.1725398	82.928496	74.9311067
81.3734324	72.9289015	75.421441	77.1725398	82.928496	72.9311067
80.3734324	72.9289015	74.421441	76.1725398	81.928496	67.9311067
81.3734324	72.9289015	74.421441	75.1725398	77.928496	67.9311067

81.3734324	71.9289015	74.421441	75.1725398	73.928496	64.9311067
81.3734324	71.9289015	74.421441	75.1725398	72.928496	64.9311067
81.3734324	70.9289015	74.421441	76.1725398	71.928496	63.9311067
80.3734324	70.9289015	75.421441	76.1725398	71.928496	62.9311067
80.3734324	70.9289015	75.421441	76.1725398	71.928496	62.9311067
79.3734324	70.9289015	75.421441	75.1725398	72.928496	62.9311067
77.3734324	69.9289015	75.421441	74.1725398	72.928496	61.9311067
77.3734324	69.9289015	76.421441	74.1725398	72.928496	63.9311067
77.3734324	69.9289015	76.421441	74.1725398	73.928496	66.9311067
76.3734324	71.9289015	76.421441	75.1725398	73.928496	69.9311067
75.3734324	71.9289015	76.421441	75.1725398	73.928496	69.9311067
75.3734324	72.9289015	76.421441	75.1725398	72.928496	66.9311067
75.3734324	73.9289015	74.421441	74.1725398	69.928496	66.9311067
75.3734324	74.9289015	73.421441	74.1725398	67.928496	66.9311067
75.3734324	75.9289015	73.421441	74.1725398	65.928496	65.9311067
75.3734324	75.9289015	73.421441	71.1725398	65.928496	64.9311067
74.3734324	75.9289015	71.421441	70.1725398	65.928496	62.9311067
74.3734324	75.9289015	71.421441	70.1725398	65.928496	62.9311067
74.3734324	73.9289015	71.421441	70.1725398	65.928496	61.9311067
74.3734324	73.9289015	71.421441	69.1725398	65.928496	59.9311067
74.3734324	72.9289015	71.421441	70.1725398	66.928496	58.9311067
78.3734324	71.9289015	71.421441	74.1725398	66.928496	58.9311067
78.3734324	72.9289015	71.421441	74.1725398	66.928496	56.9311067
81.3734324	72.9289015	71.421441	74.1725398	66.928496	56.9311067
84.3734324	74.9289015	71.421441	74.1725398	66.928496	56.9311067
73.4872373	74.9289015	71.421441	74.1725398	66.928496	56.9311067
73.5781464	74.9289015	71.421441	73.1725398	66.928496	56.9311067
74.0326919	74.9289015	75.421441	73.1725398	66.928496	56.9311067
73.7599646	74.9289015	74.421441	73.1725398	66.928496	57.9311067
73.3963282	75.9289015	73.421441	74.1725398	66.928496	57.9311067
73.3963282	75.9289015	73.421441	76.1725398	66.928496	58.9311067
74.123601	75.9289015	72.421441	77.1725398	68.928496	65.9311067
73.9417828	74.9289015	70.421441	78.1725398	67.928496	66.9311067
73.6690555	74.9289015	70.421441	75.1725398	66.928496	67.9311067
72.8508737	74.9289015	68.421441	75.1725398	66.928496	67.9311067
72.7599646	74.9289015	68.421441	74.1725398	66.928496	67.9311067
72.6690555	74.9289015	68.421441	74.1725398	66.928496	67.9311067
73.0326919	74.9289015	68.421441	75.1725398	68.928496	67.9311067
73.2145101	74.9289015	69.421441	75.1725398	71.928496	67.9311067
73.6690555	74.9289015	69.421441	75.1725398	75.928496	67.9311067
73.6690555	76.9289015	70.421441	72.1725398	76.928496	68.9311067
73.4872373	76.9289015	71.421441	71.1725398	76.928496	68.9311067
73.3054192	76.9289015	71.421441	71.1725398	75.928496	69.9311067
73.2145101	75.9289015	71.421441	70.1725398	76.928496	69.9311067
73.123601	74.9289015	70.421441	70.1725398	77.928496	70.9311067

72.8508737	73.9289015	68.421441	70.1725398	78.928496	70.9311067
72.3054192	72.9289015	65.421441	70.1725398	78.928496	70.9311067
72.4872373	74.9289015	65.421441	71.1725398	78.928496	70.9311067
72.5781464	74.9289015	64.421441	71.1725398	79.928496	70.9311067
72.6690555	74.9289015	63.421441	71.1725398	81.928496	70.9311067
72.6690555	74.9289015	63.421441	71.1725398	81.928496	70.9311067
72.3054192	75.9289015	61.421441	72.1725398	80.928496	69.9311067
72.3054192	75.9289015	61.421441	75.1725398	79.928496	69.9311067
72.123601	75.9289015	59.421441	72.1725398	79.928496	69.9311067
72.2145101	75.9289015	58.421441	71.1725398	80.928496	70.9311067
72.4872373	75.9289015	59.421441	72.1725398	81.928496	70.9311067
72.5781464	76.9289015	59.421441	72.1725398	82.928496	71.9311067
80.3734324	75.9289015	57.421441	75.1725398	84.928496	71.9311067
77.3734324	75.9289015	57.421441	77.1725398	72.0326919	71.9311067
77.3734324	75.9289015	57.421441	78.1725398	72.0326919	72.9311067
77.3734324	74.9289015	57.421441	80.1725398	72.3963282	72.9311067
75.3734324	74.9289015	58.421441	80.1725398	72.7599646	72.9311067
74.3734324	80.9289015	58.421441	81.1725398	77.928496	72.9311067
74.3734324	80.9289015	59.421441	81.1725398	71.928496	72.9311067
74.3734324	80.9289015	59.421441	78.1725398	71.928496	71.9311067
74.3734324	81.9289015	62.421441	76.1725398	64.928496	71.9311067
74.3734324	81.9289015	62.421441	76.1725398	62.928496	71.9311067
74.3734324	81.9289015	62.421441	75.1725398	61.928496	73.9311067
71.3734324	81.9289015	64.421441	75.1725398	60.928496	74.9311067
71.3734324	81.9289015	64.421441	74.1725398	60.928496	74.9311067
71.3734324	80.9289015	65.421441	74.1725398	60.928496	73.9311067
71.3734324	80.9289015	66.421441	74.1725398	60.928496	73.9311067
71.3734324	80.9289015	67.421441	74.1725398	60.928496	72.9311067
71.3734324	81.9289015	67.421441	73.1725398	60.928496	73.9311067
70.3734324	80.9289015	71.421441	73.1725398	61.928496	73.9311067
70.3734324	80.9289015	71.421441	72.1725398	62.928496	74.9311067
73.3734324	79.9289015	72.421441	72.1725398	62.928496	74.9311067
74.3734324	79.9289015	72.421441	72.1725398	61.928496	74.9311067
74.3734324	78.9289015	73.421441	73.1725398	60.928496	75.9311067
74.3734324	78.9289015	73.421441	73.1725398	59.928496	75.9311067
75.3734324	79.9289015	73.421441	73.1725398	59.928496	73.9311067
75.3734324	77.9289015	72.421441	72.1725398	61.928496	72.9311067
74.3734324	77.9289015	72.421441	72.1725398	62.928496	70.9311067
74.3734324	77.9289015	72.421441	72.1725398	64.928496	70.9311067
74.3734324	78.9289015	71.421441	73.1725398	65.928496	68.9311067
74.3734324	79.9289015	71.421441	72.1725398	65.928496	66.9311067
74.3734324	80.9289015	71.421441	74.1725398	66.928496	66.9311067
74.3734324	80.9289015	71.421441	75.1725398	67.928496	67.9311067
74.3734324	79.9289015	71.421441	74.1725398	68.928496	67.9311067
74.3734324	78.9289015	71.421441	74.1725398	72.928496	67.9311067

74.3734324	78.9289015	71.421441	73.1725398	75.928496	68.9311067
74.3734324	77.9289015	71.421441	73.1725398	75.928496	68.9311067
74.3734324	76.9289015	71.421441	73.1725398	75.928496	69.9311067
75.3734324	76.9289015	71.421441	73.1725398	75.928496	69.9311067
76.3734324	76.9289015	71.421441	73.1725398	76.928496	69.9311067
76.3734324	77.9289015	70.421441	74.1725398	76.928496	70.9311067
76.3734324	78.9289015	70.421441	74.1725398	76.928496	70.9311067
76.3734324	78.9289015	70.421441	74.1725398	72.928496	70.9311067
78.3734324	79.9289015	70.421441	74.1725398	69.928496	70.9311067
78.3734324	80.9289015	70.421441	73.1725398	68.928496	71.9311067
77.3734324	81.9289015	70.421441	74.1725398	68.928496	71.9311067
76.3734324	81.9289015	70.421441	74.1725398	68.928496	71.9311067
75.3734324	81.9289015	70.421441	73.1725398	70.928496	71.9311067
75.3734324	81.9289015	70.421441	72.1725398	70.928496	71.9311067
75.3734324	81.9289015	70.421441	72.1725398	70.928496	71.9311067
75.3734324	80.9289015	68.421441	72.1725398	69.928496	71.9311067
75.3734324	79.9289015	66.421441	72.1725398	68.928496	71.9311067
75.3734324	79.9289015	66.421441	72.1725398	68.928496	71.9311067
75.3734324	79.9289015	66.421441	72.1725398	69.928496	73.9311067
75.3734324	79.9289015	65.421441	75.1725398	72.928496	73.9311067
75.3734324	79.9289015	65.421441	75.1725398	72.928496	74.9311067
73.3734324	77.9289015	65.421441	75.1725398	72.928496	74.9311067
68.3734324	76.9289015	65.421441	74.1725398	73.928496	73.9311067
66.3734324	76.9289015	65.421441	74.1725398	76.928496	73.9311067
66.3734324	74.9289015	65.421441	72.1725398	79.928496	72.9311067
66.3734324	74.9289015	65.421441	72.1725398	82.928496	73.9311067
65.3734324	74.9289015	65.421441	72.1725398	83.928496	74.9311067
67.3734324	73.9289015	67.421441	73.1725398	84.928496	74.9311067
67.3734324	73.9289015	67.421441	73.1725398	84.928496	74.9311067
67.3734324	73.9289015	67.421441	72.1725398	84.928496	75.9311067
67.3734324	74.9289015	67.421441	72.1725398	72.9417828	77.9311067
69.3734324	75.9289015	69.421441	72.1725398	85.928496	78.9311067
72.3734324	75.9289015	69.421441	72.1725398	85.928496	78.9311067
73.3734324	75.9289015	71.421441	72.1725398	84.928496	80.9311067
73.3734324	75.9289015	71.421441	73.1725398	84.928496	83.9311067
73.3734324	75.9289015	71.421441	76.1725398	86.928496	83.9311067
73.3734324	76.9289015	71.421441	77.1725398	86.928496	83.9311067
73.3734324	76.9289015	71.421441	76.1725398	86.928496	84.9311067
73.3734324	76.9289015	71.421441	76.1725398	85.928496	84.9311067
72.3734324	77.9289015	72.421441	75.1725398	84.928496	83.9311067
69.3734324	78.9289015	73.421441	73.1725398	83.928496	82.9311067
68.3734324	78.9289015	73.421441	71.1725398	82.928496	82.9311067
68.3734324	80.9289015	73.421441	71.1725398	78.928496	82.9311067
68.3734324	80.9289015	73.421441	72.1725398	77.928496	82.9311067
69.3734324	81.9289015	73.421441	72.1725398	76.928496	80.9311067

69.3734324	83.9289015	73.421441	75.1725398	77.928496	77.9311067
69.3734324	84.9289015	73.421441	77.1725398	80.928496	77.9311067
69.3734324	84.9289015	70.421441	73.1725398	72.928496	73.9311067
70.3734324	85.9289015	70.421441	72.1725398	69.928496	73.9311067
70.3734324	85.9289015	70.421441	72.1725398	69.928496	73.9311067
70.3734324	86.9289015	70.421441	72.1725398	69.928496	73.9311067
70.3734324	74.0326919	70.421441	71.1725398	69.928496	72.9311067
71.3734324	74.3963282	70.421441	72.1725398	71.928496	72.9311067
70.3734324	75.0326919	70.421441	73.1725398	73.928496	72.9311067
70.3734324	75.123601	70.421441	74.1725398	74.928496	72.9311067
70.3734324	74.5781464	70.421441	74.1725398	75.928496	69.9311067
69.3734324	74.6690555	70.421441	75.1725398	75.928496	70.9311067
71.3734324	75.123601	70.421441	76.1725398	75.928496	70.9311067
72.3734324	75.123601	70.421441	77.1725398	75.928496	71.9311067
78.3734324	75.8508737	70.421441	78.1725398	76.928496	71.9311067
78.3734324	75.7599646	70.421441	78.1725398	76.928496	71.9311067
81.3734324	76.4872373	71.421441	78.1725398	81.928496	71.9311067
81.3734324	76.3963282	71.421441	76.1725398	81.928496	73.9311067
81.3734324	76.2145101	71.421441	75.1725398	80.928496	73.9311067
82.3734324	82.9289015	70.421441	74.1725398	80.928496	73.9311067
83.3734324	81.9289015	70.421441	74.1725398	80.928496	73.9311067
83.3734324	81.9289015	71.421441	74.1725398	80.928496	73.9311067
83.3734324	81.9289015	70.421441	74.1725398	79.928496	74.9311067
83.3734324	81.9289015	70.421441	74.1725398	79.928496	74.9311067
81.3734324	79.9289015	67.421441	74.1725398	78.928496	74.9311067
80.3734324	79.9289015	67.421441	74.1725398	77.928496	75.9311067
80.3734324	78.9289015	66.421441	74.1725398	77.928496	75.9311067
80.3734324	78.9289015	65.421441	73.1725398	76.928496	75.9311067
80.3734324	78.9289015	65.421441	73.1725398	76.928496	75.9311067
80.3734324	75.9289015	64.421441	73.1725398	76.928496	75.9311067
76.3734324	73.9289015	63.421441	74.1725398	74.928496	75.9311067
76.3734324	72.9289015	63.421441	76.1725398	73.928496	75.9311067
71.3734324	72.9289015	63.421441	76.1725398	70.928496	75.9311067
71.3734324	72.9289015	63.421441	76.1725398	68.928496	76.9311067
71.3734324	72.9289015	62.421441	76.1725398	69.928496	75.9311067
71.3734324	72.9289015	62.421441	76.1725398	69.928496	75.9311067
70.3734324	72.9289015	62.421441	76.1725398	69.928496	75.9311067
70.3734324	72.9289015	62.421441	75.1725398	69.928496	75.9311067
70.3734324	72.9289015	62.421441	74.1725398	69.928496	75.9311067
70.3734324	73.9289015	64.421441	74.1725398	76.928496	75.9311067
70.3734324	73.9289015	64.421441	74.1725398	81.928496	75.9311067
69.3734324	73.9289015	64.421441	73.1725398	81.928496	75.9311067
70.3734324	73.9289015	65.421441	73.1725398	80.928496	75.9311067
70.3734324	74.9289015	65.421441	72.1725398	76.928496	76.9311067
70.3734324	73.9289015	65.421441	72.1725398	76.928496	78.9311067

70.3734324	73.9289015	65.421441	73.1725398	74.928496	80.9311067
70.3734324	74.9289015	66.421441	74.1725398	73.928496	81.9311067
70.3734324	74.9289015	68.421441	74.1725398	72.928496	82.9311067
70.3734324	74.9289015	71.421441	74.1725398	72.928496	82.9311067
67.3734324	75.9289015	71.421441	74.1725398	73.928496	82.9311067
67.3734324	75.9289015	71.421441	75.1725398	73.928496	83.9311067
67.3734324	74.9289015	71.421441	75.1725398	73.928496	83.9311067
71.3734324	74.9289015	71.421441	77.1725398	74.928496	83.9311067
72.3734324	74.9289015	69.421441	77.1725398	76.928496	83.9311067
72.3734324	72.9289015	69.421441	77.1725398	77.928496	83.9311067
73.3734324	72.9289015	69.421441	77.1725398	80.928496	83.9311067
73.3734324	72.9289015	67.421441	75.1725398	82.928496	82.9311067
76.3734324	71.9289015	67.421441	74.1725398	82.928496	82.9311067
76.3734324	74.9289015	64.421441	74.1725398	82.928496	81.9311067
76.3734324	74.9289015	64.421441	76.1725398	81.928496	81.9311067
76.3734324	73.9289015	64.421441	77.1725398	82.928496	74.9311067
76.3734324	73.9289015	63.421441	79.1725398	78.928496	72.9311067
77.3734324	73.9289015	63.421441	81.1725398	76.928496	71.9311067
76.3734324	73.9289015	63.421441	82.1725398	75.928496	71.9311067
75.3734324	73.9289015	63.421441	81.1725398	76.928496	70.9311067
75.3734324	73.9289015	64.421441	78.1725398	77.928496	70.9311067
75.3734324	74.9289015	64.421441	78.1725398	78.928496	69.9311067
76.3734324	74.9289015	64.421441	75.1725398	78.928496	66.9311067
76.3734324	77.9289015	65.421441	73.1725398	78.928496	66.9311067
76.3734324	78.9289015	66.421441	71.1725398	77.928496	66.9311067
76.3734324	79.9289015	66.421441	71.1725398	76.928496	64.9311067
77.3734324	79.9289015	67.421441	71.1725398	74.928496	64.9311067
77.3734324	80.9289015	67.421441	72.1725398	73.928496	64.9311067
77.3734324	80.9289015	67.421441	73.1725398	73.928496	65.9311067
78.3734324	80.9289015	68.421441	74.1725398	73.928496	65.9311067
77.3734324	80.9289015	70.421441	74.1725398	71.928496	65.9311067
77.3734324	81.9289015	70.421441	75.1725398	71.928496	66.9311067
77.3734324	81.9289015	70.421441	75.1725398	71.928496	66.9311067
77.3734324	78.9289015	70.421441	74.1725398	71.928496	67.9311067
78.3734324	75.9289015	71.421441	73.1725398	72.928496	68.9311067
78.3734324	73.9289015	71.421441	73.1725398	72.928496	68.9311067
78.3734324	71.9289015	71.421441	73.1725398	72.928496	70.9311067
78.3734324	70.9289015	71.421441	73.1725398	71.928496	71.9311067
78.3734324	69.9289015	71.421441	73.1725398	71.928496	73.9311067
78.3734324	69.9289015	71.421441	74.1725398	70.928496	73.9311067
78.3734324	71.9289015	71.421441	76.1725398	69.928496	73.9311067
76.3734324	71.9289015	71.421441	76.1725398	69.928496	74.9311067
76.3734324	74.9289015	70.421441	75.1725398	66.928496	74.9311067
77.3734324	74.9289015	70.421441	72.1725398	66.928496	74.9311067
77.3734324	74.9289015	70.421441	72.1725398	66.928496	74.9311067

76.3734324	73.9289015	73.421441	71.1725398	67.928496	82.9311067
76.3734324	72.9289015	73.421441	71.1725398	68.928496	73.3258374
76.3734324	71.9289015	73.421441	72.1725398	68.928496	73.2258374
76.3734324	70.9289015	73.421441	73.1725398	71.928496	85.9311067
78.3734324	70.9289015	76.421441	74.1725398	75.928496	85.9311067
78.3734324	70.9289015	80.421441	74.1725398	75.928496	81.9311067
78.3734324	70.9289015	80.421441	74.1725398	76.928496	72.9311067
78.3734324	70.9289015	82.421441	72.1725398	74.928496	72.9311067
78.3734324	70.9289015	83.421441	72.1725398	74.928496	64.9311067
78.3734324	70.9289015	81.421441	68.1725398	71.928496	64.9311067
78.3734324	70.9289015	80.421441	68.1725398	70.928496	64.9311067
78.3734324	70.9289015	79.421441	67.1725398	70.928496	65.9311067
82.3734324	70.9289015	79.421441	67.1725398	70.928496	65.9311067
82.3734324	70.9289015	79.421441	67.1725398	70.928496	63.9311067
82.3734324	70.9289015	79.421441	69.1725398	70.928496	62.9311067
81.3734324	70.9289015	78.421441	71.1725398	70.928496	61.9311067
78.3734324	71.9289015	75.421441	71.1725398	70.928496	61.9311067
78.3734324	71.9289015	74.421441	71.1725398	70.928496	60.9311067
72.3734324	71.9289015	74.421441	72.1725398	70.928496	58.9311067
71.3734324	71.9289015	73.421441	73.1725398	70.928496	58.9311067
71.3734324	69.9289015	73.421441	74.1725398	70.928496	57.9311067
71.3734324	67.9289015	71.421441	74.1725398	70.928496	57.9311067
71.3734324	67.9289015	70.421441	74.1725398	71.928496	57.9311067
71.3734324	67.9289015	69.421441	75.1725398	71.928496	56.9311067
71.3734324	67.9289015	68.421441	75.1725398	72.928496	56.9311067
71.3734324	69.9289015	68.421441	76.1725398	72.928496	57.9311067
71.3734324	70.9289015	69.421441	76.1725398	71.928496	57.9311067
73.3734324	73.9289015	75.421441	76.1725398	71.928496	56.9311067
73.3734324	75.9289015	75.421441	75.1725398	70.928496	56.9311067
73.3734324	77.9289015	75.421441	75.1725398	70.928496	56.9311067
73.3734324	77.9289015	75.421441	75.1725398	71.928496	56.9311067
73.3734324	78.9289015	75.421441	79.1725398	71.928496	55.9311067
77.3734324	78.9289015	76.421441	80.1725398	71.928496	55.9311067
77.3734324	79.9289015	76.421441	80.1725398	71.928496	55.9311067
76.3734324	80.9289015	77.421441	79.1725398	71.928496	56.9311067
76.3734324	80.9289015	77.421441	79.1725398	72.928496	56.9311067
76.3734324	80.9289015	77.421441	73.1725398	79.928496	56.9311067
76.3734324	80.9289015	77.421441	72.1725398	83.928496	58.9311067
76.3734324	79.9289015	77.421441	72.1725398	83.928496	58.9311067
76.3734324	78.9289015	77.421441	72.1725398	83.928496	59.9311067
76.3734324	78.9289015	77.421441	73.1725398	84.928496	59.9311067
76.3734324	78.9289015	77.421441	73.1725398	84.928496	59.9311067
75.3734324	77.9289015	77.421441	74.1725398	85.928496	62.9311067
75.3734324	77.9289015	78.421441	74.1725398	87.928496	62.9311067
75.3734324	77.9289015	78.421441	74.1725398	85.928496	64.9311067

75.3734324	75.9289015	78.421441	74.1725398	84.928496	64.9311067
73.3734324	74.9289015	77.421441	75.1725398	84.928496	64.9311067
73.3734324	74.9289015	76.421441	75.1725398	84.928496	64.9311067
73.3734324	73.9289015	76.421441	79.1725398	85.928496	65.9311067
72.3734324	70.9289015	75.421441	79.1725398	85.928496	69.9311067
72.3734324	70.9289015	75.421441	83.1725398	85.928496	74.9311067
72.3734324	70.9289015	76.421441	83.1725398	86.928496	77.9311067
71.3734324	70.9289015	76.421441	82.1725398	86.928496	77.9311067
71.3734324	69.9289015	76.421441	83.1725398	85.928496	78.9311067
72.3734324	68.9289015	76.421441	85.1725398	85.928496	78.9311067
74.3734324	66.9289015	76.421441	85.1725398	82.928496	79.9311067
74.3734324	67.9289015	76.421441	87.1725398	82.928496	80.9311067
74.3734324	68.9289015	76.421441	87.1725398	82.928496	80.9311067
74.3734324	67.9289015	76.421441	87.1725398	81.928496	80.9311067
72.3734324	67.9289015	76.421441	85.1725398	81.928496	80.9311067
72.3734324	67.9289015	77.421441	85.1725398	81.928496	80.9311067
72.3734324	67.9289015	79.421441	82.1725398	81.928496	80.9311067
70.3734324	67.9289015	79.421441	82.1725398	76.928496	80.9311067
70.3734324	69.9289015	79.421441	81.1725398	70.928496	82.9311067
70.3734324	71.9289015	80.421441	81.1725398	69.928496	83.9311067
70.3734324	72.9289015	81.421441	80.1725398	68.928496	84.9311067
70.3734324	72.9289015	81.421441	80.1725398	68.928496	84.9311067
70.3734324	72.9289015	80.421441	79.1725398	68.928496	84.9311067
70.3734324	71.9289015	80.421441	79.1725398	68.928496	84.9311067
69.3734324	68.9289015	77.421441	75.1725398	68.928496	81.9311067
64.3734324	66.9289015	76.421441	73.1725398	68.928496	79.9311067
63.3734324	66.9289015	76.421441	71.1725398	68.928496	76.9311067
63.3734324	66.9289015	75.421441	69.1725398	68.928496	75.9311067
63.3734324	66.9289015	75.421441	69.1725398	68.928496	75.9311067
63.3734324	69.9289015	75.421441	68.1725398	68.928496	75.9311067
63.3734324	70.9289015	77.421441	67.1725398	68.928496	74.9311067
63.3734324	70.9289015	78.421441	68.1725398	68.928496	74.9311067
63.3734324	70.9289015	78.421441	68.1725398	69.928496	74.9311067
63.3734324	70.9289015	78.421441	71.1725398	73.928496	72.9311067
63.3734324	71.9289015	78.421441	71.1725398	75.928496	72.9311067
64.3734324	72.9289015	78.421441	71.1725398	78.928496	72.9311067
65.3734324	72.9289015	78.421441	71.1725398	79.928496	71.9311067
64.3734324	72.9289015	77.421441	71.1725398	81.928496	70.9311067
64.3734324	72.9289015	77.421441	72.1725398	81.928496	70.9311067
64.3734324	72.9289015	77.421441	74.1725398	81.928496	69.9311067
64.3734324	72.9289015	77.421441	75.1725398	83.928496	71.9311067
64.3734324	72.9289015	77.421441	75.1725398	83.928496	71.9311067
66.3734324	72.9289015	76.421441	73.1725398	83.928496	71.9311067
66.3734324	73.9289015	76.421441	69.1725398	82.928496	71.9311067
67.3734324	75.9289015	75.421441	69.1725398	75.928496	72.9311067

74.3734324	75.9289015	75.421441	69.1725398	70.928496	72.9311067
74.3734324	77.9289015	75.421441	69.1725398	68.928496	73.9311067
74.3734324	78.9289015	75.421441	69.1725398	68.928496	73.9311067
74.3734324	79.9289015	75.421441	69.1725398	68.928496	73.9311067
74.3734324	79.9289015	75.421441	69.1725398	68.928496	74.9311067
74.3734324	79.9289015	75.421441	69.1725398	68.928496	74.9311067
74.3734324	80.9289015	75.421441	68.1725398	69.928496	75.9311067
75.3734324	80.9289015	76.421441	68.1725398	70.928496	75.9311067
75.3734324	80.9289015	76.421441	68.1725398	76.928496	74.9311067
75.3734324	81.9289015	76.421441	68.1725398	76.928496	74.9311067
75.3734324	81.9289015	80.421441	68.1725398	73.928496	73.9311067
75.3734324	81.9289015	79.421441	68.1725398	72.928496	73.9311067
75.3734324	80.9289015	79.421441	68.1725398	71.928496	73.9311067
75.3734324	81.9289015	77.421441	67.1725398	72.928496	73.9311067
75.3734324	81.9289015	76.421441	67.1725398	72.928496	73.9311067
76.3734324	81.9289015	76.421441	67.1725398	71.928496	73.9311067
77.3734324	82.9289015	73.421441	67.1725398	71.928496	76.9311067
75.3734324	80.9289015	72.421441	67.1725398	70.928496	79.9311067
72.3734324	80.9289015	73.421441	67.1725398	69.928496	78.9311067
69.3734324	81.9289015	73.421441	68.1725398	68.928496	76.9311067
67.3734324	82.9289015	75.421441	68.1725398	68.928496	73.9311067
66.3734324	81.9289015	75.421441	69.1725398	68.928496	73.9311067
66.3734324	81.9289015	75.421441	69.1725398	67.928496	71.9311067
66.3734324	81.9289015	75.421441	69.1725398	67.928496	71.9311067
66.3734324	81.9289015	75.421441	71.1725398	66.928496	71.9311067
65.3734324	81.9289015	75.421441	71.1725398	66.928496	72.9311067
65.3734324	81.9289015	75.421441	69.1725398	67.928496	71.9311067
65.3734324	81.9289015	75.421441	70.1725398	67.928496	70.9311067
68.3734324	81.9289015	75.421441	70.1725398	67.928496	69.9311067
71.3734324	80.9289015	75.421441	70.1725398	67.928496	69.9311067
71.3734324	79.9289015	75.421441	71.1725398	67.928496	69.9311067
76.3734324	79.9289015	75.421441	71.1725398	67.928496	69.9311067
76.3734324	79.9289015	75.421441	75.1725398	66.928496	69.9311067
77.3734324	79.9289015	74.421441	76.1725398	66.928496	71.9311067
77.3734324	79.9289015	74.421441	76.1725398	66.928496	70.9311067
78.3734324	79.9289015	74.421441	76.1725398	68.928496	68.9311067
78.3734324	79.9289015	74.421441	74.7258374	71.928496	65.9311067
78.3734324	79.9289015	74.421441	74.9258374	72.928496	65.9311067
78.3734324	79.9289015	74.421441	75.5258374	74.928496	65.9311067
79.3734324	80.9289015	73.421441	75.4258374	74.928496	66.9311067
79.3734324	80.9289015	73.421441	75.3258374	74.928496	66.9311067
80.3734324	81.9289015	73.421441	75.3258374	74.928496	66.9311067
80.3734324	81.9289015	72.421441	75.1258374	74.928496	65.9311067
80.3734324	81.9289015	71.421441	75.3258374	74.928496	65.9311067
76.3734324	67.9289015	71.421441	73.4258374	74.928496	65.9311067

76.3734324	68.9289015	70.421441	74.0258374	77.928496	65.9311067
72.3734324	69.9289015	69.421441	74.0258374	78.928496	65.9311067
72.3734324	69.9289015	69.421441	73.9258374	76.928496	65.9311067
72.3734324	69.9289015	69.421441	73.9258374	75.928496	66.9311067
72.3734324	69.9289015	69.421441	74.1258374	76.928496	66.9311067
73.3734324	68.9289015	69.421441	81.1725398	76.928496	66.9311067
73.3734324	68.9289015	70.421441	81.1725398	76.928496	66.9311067
73.3734324	68.9289015	70.421441	82.1725398	76.928496	65.9311067
73.3734324	68.9289015	76.421441	81.1725398	76.928496	65.9311067
73.3734324	67.9289015	74.421441	80.1725398	80.928496	64.9311067
73.3734324	67.9289015	75.421441	80.1725398	80.928496	66.9311067
75.3734324	67.9289015	75.421441	77.1725398	79.928496	67.9311067
76.3734324	67.9289015	77.421441	76.1725398	78.928496	67.9311067
76.3734324	67.9289015	78.421441	75.1725398	80.928496	67.9311067
76.3734324	67.9289015	78.421441	75.1725398	80.928496	67.9311067
76.3734324	67.9289015	78.421441	73.1725398	81.928496	67.9311067
77.3734324	69.9289015	78.421441	73.1725398	82.928496	67.9311067
77.3734324	70.9289015	78.421441	71.1725398	82.928496	67.9311067
77.3734324	71.9289015	78.421441	70.1725398	82.928496	67.9311067
78.3734324	72.9289015	78.421441	70.1725398	83.928496	68.9311067
78.3734324	72.9289015	78.421441	69.1725398	83.928496	68.9311067
78.3734324	73.9289015	78.421441	69.1725398	83.928496	69.9311067
79.3734324	73.9289015	78.421441	69.1725398	83.928496	69.9311067
80.3734324	73.9289015	78.421441	66.1725398	88.928496	69.9311067
81.3734324	76.9289015	77.421441	66.1725398	75.8258374	68.9311067
81.3734324	78.9289015	77.421441	66.1725398	75.4258374	68.9311067
82.3734324	78.9289015	76.421441	70.1725398	87.928496	67.9311067
82.3734324	79.9289015	74.421441	72.1725398	85.928496	66.9311067
81.3734324	79.9289015	74.421441	71.1725398	82.928496	70.9311067
81.3734324	79.9289015	72.421441	70.1725398	79.928496	72.9311067
81.3734324	79.9289015	71.421441	66.1725398	79.928496	75.9311067
81.3734324	79.9289015	71.421441	63.1725398	78.928496	75.9311067
81.3734324	79.9289015	70.421441	63.1725398	78.928496	75.9311067
81.3734324	81.9289015	70.421441	64.1725398	78.928496	76.9311067
81.3734324	81.9289015	71.421441	64.1725398	78.928496	76.9311067
82.3734324	83.9289015	71.421441	65.1725398	77.928496	78.9311067
82.3734324	83.9289015	72.421441	67.1725398	76.928496	78.9311067
82.3734324	83.9289015	73.421441	68.1725398	75.928496	78.9311067
82.3734324	83.9289015	73.421441	71.1725398	75.928496	74.9311067
82.3734324	83.9289015	73.421441	73.1725398	74.928496	73.9311067
82.3734324	83.9289015	73.421441	73.1725398	74.928496	73.9311067
82.3734324	84.9289015	72.421441	73.1725398	74.928496	73.9311067
82.3734324	84.9289015	72.421441	73.1725398	74.928496	73.9311067
82.3734324	84.9289015	72.421441	73.1725398	74.928496	72.9311067
82.3734324	77.9289015	75.421441	73.1725398	74.928496	72.9311067

82.3734324	70.9289015	75.421441	73.1725398	77.928496	72.9311067
81.3734324	65.9289015	76.421441	73.1725398	80.928496	72.9311067
81.3734324	63.9289015	77.421441	77.1725398	80.928496	73.9311067
81.3734324	63.9289015	77.421441	78.1725398	74.928496	73.9311067
81.3734324	63.9289015	76.421441	78.1725398	71.928496	73.9311067
81.3734324	63.9289015	76.421441	78.1725398	69.928496	72.9311067
81.3734324	63.9289015	76.421441	80.1725398	68.928496	71.9311067
80.3734324	63.9289015	74.421441	82.1725398	69.928496	71.9311067
79.3734324	63.9289015	74.421441	83.1725398	71.928496	71.9311067
79.3734324	63.9289015	73.421441	83.1725398	72.928496	72.9311067
79.3734324	64.9289015	73.421441	83.1725398	72.928496	72.9311067
79.3734324	66.9289015	73.421441	83.1725398	71.928496	73.9311067
73.3734324	66.9289015	73.421441	83.1725398	71.928496	73.9311067
68.3734324	67.9289015	73.421441	72.1258374	71.928496	72.9311067
68.3734324	67.9289015	73.421441	72.4258374	71.928496	72.9311067
67.3734324	69.9289015	73.421441	72.3258374	71.928496	71.9311067
66.3734324	76.9289015	74.421441	73.3258374	71.928496	73.9311067
66.3734324	76.9289015	74.421441	73.7258374	72.928496	73.9311067
66.3734324	75.9289015	74.421441	86.1725398	72.928496	73.9311067
66.3734324	75.9289015	74.421441	84.1725398	72.928496	73.9311067
66.3734324	75.9289015	74.421441	82.1725398	72.928496	73.9311067
66.3734324	75.9289015	72.421441	80.1725398	71.928496	73.9311067
65.3734324	75.9289015	72.421441	79.1725398	73.928496	73.9311067
65.3734324	75.9289015	72.421441	79.1725398	73.928496	73.9311067
65.3734324	75.9289015	70.421441	80.1725398	76.928496	68.9311067
66.3734324	75.9289015	69.421441	80.1725398	76.928496	66.9311067
69.3734324	76.9289015	66.421441	80.1725398	76.928496	66.9311067
69.3734324	76.9289015	66.421441	80.1725398	77.928496	66.9311067
71.3734324	75.9289015	64.421441	81.1725398	77.928496	68.9311067
71.3734324	75.9289015	64.421441	81.1725398	77.928496	68.9311067
71.3734324	65.9289015	64.421441	84.1725398	76.928496	72.9311067
74.3734324	61.9289015	64.421441	84.1725398	76.928496	74.9311067
74.3734324	61.9289015	65.421441	84.1725398	76.928496	74.9311067
74.3734324	62.9289015	65.421441	83.1725398	75.928496	73.9311067
74.3734324	62.9289015	65.421441	83.1725398	74.928496	73.9311067
75.3734324	62.9289015	68.421441	81.1725398	74.928496	73.9311067
75.3734324	62.9289015	70.421441	80.1725398	74.928496	73.9311067
75.3734324	62.9289015	71.421441	80.1725398	73.928496	72.9311067
73.3734324	62.9289015	72.421441	79.1725398	73.928496	72.9311067
71.3734324	63.9289015	72.421441	79.1725398	73.928496	71.9311067
70.3734324	63.9289015	71.421441	78.1725398	73.928496	70.9311067
70.3734324	67.9289015	71.421441	73.1725398	73.928496	70.9311067
70.3734324	67.9289015	70.421441	69.1725398	74.928496	70.9311067
69.3734324	75.9289015	69.421441	67.1725398	75.928496	69.9311067
68.3734324	77.9289015	69.421441	67.1725398	75.928496	65.9311067

67.3734324	76.9289015	69.421441	66.1725398	75.928496	65.9311067
67.3734324	76.9289015	68.421441	66.1725398	76.928496	65.9311067
67.3734324	76.9289015	67.421441	66.1725398	76.928496	65.9311067
68.3734324	76.9289015	67.421441	67.1725398	75.928496	65.9311067
68.3734324	76.9289015	67.421441	68.1725398	72.928496	65.9311067
69.3734324	76.9289015	67.421441	68.1725398	71.928496	65.9311067
70.3734324	75.9289015	68.421441	68.1725398	71.928496	65.9311067
72.3734324	75.9289015	68.421441	68.1725398	71.928496	65.9311067
72.3734324	75.9289015	68.421441	68.1725398	71.928496	65.9311067
71.3734324	75.9289015	68.421441	70.1725398	71.928496	66.9311067
71.3734324	75.9289015	67.421441	70.1725398	72.928496	66.9311067
71.3734324	75.9289015	67.421441	71.1725398	73.928496	66.9311067
71.3734324	75.9289015	67.421441	74.1725398	72.928496	66.9311067
71.3734324	75.9289015	67.421441	75.1725398	71.928496	68.9311067
71.3734324	75.9289015	67.421441	76.1725398	70.928496	68.9311067
71.3734324	75.9289015	67.421441	78.1725398	67.928496	68.9311067
72.3734324	78.9289015	67.421441	78.1725398	66.928496	69.9311067
72.3734324	76.9289015	67.421441	78.1725398	66.928496	69.9311067
72.3734324	76.9289015	67.421441	78.1725398	66.928496	67.9311067
72.3734324	75.9289015	69.421441	79.1725398	66.928496	66.9311067
72.3734324	74.9289015	69.421441	79.1725398	66.928496	66.9311067
72.3734324	71.9289015	69.421441	78.1725398	66.928496	66.9311067
71.3734324	67.9289015	69.421441	75.1725398	66.928496	66.9311067
71.3734324	67.9289015	68.421441	75.1725398	67.928496	66.9311067
71.3734324	67.9289015	68.421441	75.1725398	68.928496	67.9311067
70.3734324	67.9289015	70.421441	75.1725398	65.928496	67.9311067
73.3734324	67.9289015	70.421441	75.1725398	64.928496	67.9311067
73.3734324	67.9289015	70.421441	76.1725398	64.928496	68.9311067
75.3734324	67.9289015	69.421441	76.1725398	65.928496	68.9311067
75.3734324	66.9289015	69.421441	76.1725398	65.928496	68.9311067
76.3734324	66.9289015	69.421441	75.1725398	66.928496	68.9311067
77.3734324	66.9289015	68.421441	74.1725398	67.928496	69.9311067
77.3734324	66.9289015	68.421441	74.1725398	67.928496	69.9311067
79.3734324	65.9289015	68.421441	74.1725398	68.928496	69.9311067
80.3734324	65.9289015	68.421441	74.1725398	69.928496	70.9311067
80.3734324	65.9289015	68.421441	74.1725398	70.928496	75.9311067
72.4258374	66.9289015	67.421441	76.1725398	71.928496	75.9311067
72.4258374	66.9289015	67.421441	76.1725398	71.928496	76.9311067
85.3734324	65.9289015	67.421441	77.1725398	71.928496	77.9311067
85.3734324	65.9289015	68.421441	79.1725398	71.928496	79.9311067
83.3734324	64.9289015	69.421441	81.1725398	68.928496	80.9311067
83.3734324	63.9289015	69.421441	81.1725398	65.928496	79.9311067
82.3734324	63.9289015	70.421441	81.1725398	63.928496	79.9311067
81.3734324	62.9289015	70.421441	81.1725398	63.928496	79.9311067
81.3734324	62.9289015	70.421441	81.1725398	63.928496	79.9311067

76.3734324	62.9289015	70.421441	79.1725398	64.928496	79.9311067
75.3734324	63.9289015	70.421441	75.1725398	65.928496	79.9311067
75.3734324	63.9289015	70.421441	72.1725398	69.928496	79.9311067
75.3734324	63.9289015	70.421441	72.1725398	70.928496	79.9311067
75.3734324	64.9289015	70.421441	72.1725398	70.928496	81.9311067
75.3734324	64.9289015	71.421441	72.1725398	69.928496	80.9311067
75.3734324	65.9289015	72.421441	74.1725398	67.928496	79.9311067
75.3734324	64.9289015	72.421441	74.1725398	67.928496	76.9311067
75.3734324	64.9289015	71.421441	74.1725398	66.928496	74.9311067
75.3734324	65.9289015	70.421441	74.1725398	66.928496	73.9311067
75.3734324	65.9289015	70.421441	73.1725398	68.928496	73.9311067
75.3734324	65.9289015	70.421441	71.1725398	69.928496	73.9311067
75.3734324	65.9289015	71.421441	69.1725398	70.928496	72.9311067
78.3734324	64.9289015	72.421441	70.1725398	72.928496	72.9311067
78.3734324	64.9289015	72.421441	70.1725398	72.928496	72.9311067
79.3734324	66.9289015	72.421441	70.1725398	72.928496	71.9311067
79.3734324	67.9289015	71.421441	71.1725398	71.928496	71.9311067
80.3734324	66.9289015	71.421441	72.1725398	72.928496	72.9311067
81.3734324	65.9289015	71.421441	72.1725398	72.928496	72.9311067
82.3734324	65.9289015	71.421441	73.1725398	72.928496	73.9311067
82.3734324	64.9289015	71.421441	75.1725398	72.928496	73.9311067
83.3734324	64.9289015	69.421441	76.1725398	72.928496	73.9311067
80.3734324	64.9289015	69.421441	77.1725398	72.928496	73.9311067
80.3734324	64.9289015	69.421441	76.1725398	72.928496	75.9311067
80.3734324	64.9289015	70.421441	76.1725398	71.928496	75.9311067
78.3734324	63.9289015	71.421441	76.1725398	71.928496	76.9311067
78.3734324	62.9289015	71.421441	76.1725398	71.928496	77.9311067
79.3734324	63.9289015	72.421441	76.1725398	70.928496	77.9311067
81.3734324	63.9289015	72.421441	76.1725398	69.928496	78.9311067
80.3734324	66.9289015	72.421441	77.1725398	69.928496	77.9311067
80.3734324	66.9289015	72.421441	78.1725398	69.928496	74.9311067
78.3734324	66.9289015	72.421441	78.1725398	70.928496	71.9311067
78.3734324	69.9289015	72.421441	77.1725398	71.928496	71.9311067
75.3734324	69.9289015	71.421441	77.1725398	74.928496	69.9311067
75.3734324	69.9289015	71.421441	77.1725398	74.928496	69.9311067
75.3734324	69.9289015	71.421441	77.1725398	75.928496	69.9311067
74.3734324	69.9289015	71.421441	77.1725398	75.928496	69.9311067
73.3734324	69.9289015	71.421441	76.1725398	76.928496	69.9311067
73.3734324	68.9289015	72.421441	76.1725398	75.928496	68.9311067
72.3734324	68.9289015	72.421441	76.1725398	70.928496	68.9311067
73.3734324	68.9289015	77.421441	76.1725398	69.928496	68.9311067
73.3734324	68.9289015	77.421441	76.1725398	69.928496	68.9311067
74.3734324	71.9289015	78.421441	76.1725398	69.928496	71.9311067
74.3734324	71.9289015	78.421441	75.1725398	69.928496	72.9311067
74.3734324	72.9289015	78.421441	75.1725398	70.928496	71.9311067

75.3734324	72.9289015	78.421441	75.1725398	70.928496	69.9311067
75.3734324	74.9289015	78.421441	75.1725398	75.928496	68.9311067
75.3734324	74.9289015	78.421441	77.1725398	78.928496	68.9311067
75.3734324	73.9289015	79.421441	76.1725398	79.928496	68.9311067
74.3734324	73.9289015	81.421441	75.1725398	77.928496	68.9311067
74.3734324	73.9289015	79.421441	75.1725398	77.928496	68.9311067
74.3734324	75.9289015	79.421441	78.1725398	76.928496	68.9311067
74.3734324	75.9289015	79.421441	80.1725398	76.928496	68.9311067
74.3734324	80.9289015	79.421441	80.1725398	77.928496	68.9311067
74.3734324	80.9289015	79.421441	80.1725398	78.928496	68.9311067
74.3734324	80.9289015	79.421441	79.1725398	79.928496	65.9311067
74.3734324	80.9289015	79.421441	77.1725398	79.928496	65.9311067
74.3734324	80.9289015	79.421441	77.1725398	79.928496	65.9311067
82.3734324	82.9289015	79.421441	76.1725398	80.928496	65.9311067
82.3734324	82.9289015	79.421441	78.1725398	83.928496	65.9311067
82.3734324	82.9289015	79.421441	78.1725398	82.928496	67.9311067
82.3734324	82.9289015	79.421441	78.1725398	80.928496	67.9311067
82.3734324	82.9289015	80.421441	77.1725398	79.928496	67.9311067
82.3734324	85.9289015	80.421441	75.1725398	78.928496	63.9311067
72.3734324	72.7258374	80.421441	72.1725398	78.928496	62.9311067
70.3734324	72.3258374	79.421441	70.1725398	78.928496	62.9311067
70.3734324	72.2258374	79.421441	69.1725398	77.928496	61.9311067
71.3734324	83.9289015	77.421441	69.1725398	75.928496	61.9311067
72.3734324	80.9289015	77.421441	69.1725398	73.928496	62.9311067
72.3734324	78.9289015	77.421441	68.1725398	71.928496	63.9311067
72.3734324	77.9289015	78.421441	67.1725398	69.928496	63.9311067
72.3734324	77.9289015	78.421441	67.1725398	68.928496	63.9311067
72.3734324	77.9289015	79.421441	67.1725398	69.928496	63.9311067
72.3734324	77.9289015	79.421441	67.1725398	69.928496	63.9311067
72.3734324	77.9289015	78.421441	67.1725398	69.928496	63.9311067
72.3734324	76.9289015	78.421441	67.1725398	68.928496	63.9311067
72.3734324	72.9289015	76.421441	66.1725398	67.928496	63.9311067
73.3734324	70.9289015	74.421441	66.1725398	66.928496	64.9311067
73.3734324	69.9289015	74.421441	65.1725398	66.928496	66.9311067
73.3734324	68.9289015	74.421441	65.1725398	67.928496	66.9311067
73.3734324	68.9289015	74.421441	65.1725398	67.928496	68.9311067
74.3734324	68.9289015	74.421441	65.1725398	67.928496	71.9311067
74.3734324	68.9289015	74.421441	65.1725398	68.928496	73.9311067
74.3734324	69.9289015	73.421441	65.1725398	68.928496	73.9311067
74.3734324	69.9289015	73.421441	65.1725398	67.928496	72.9311067
73.3734324	69.9289015	73.421441	66.1725398	68.928496	67.9311067
73.3734324	69.9289015	71.421441	66.1725398	68.928496	65.9311067
73.3734324	71.9289015	73.421441	68.1725398	67.928496	65.9311067
73.3734324	71.9289015	73.421441	68.1725398	66.928496	65.9311067
73.3734324	71.9289015	73.421441	68.1725398	65.928496	65.9311067

73.3734324	71.9289015	73.421441	67.1725398	65.928496	65.9311067
73.3734324	71.9289015	73.421441	68.1725398	65.928496	65.9311067
73.3734324	70.9289015	73.421441	68.1725398	66.928496	65.9311067
74.3734324	66.9289015	73.421441	69.1725398	66.928496	65.9311067
74.3734324	65.9289015	73.421441	69.1725398	66.928496	65.9311067
74.3734324	65.9289015	73.421441	69.1725398	66.928496	65.9311067
74.3734324	65.9289015	73.421441	69.1725398	65.928496	64.9311067
75.3734324	65.9289015	74.421441	69.1725398	65.928496	64.9311067
75.3734324	65.9289015	75.421441	69.1725398	66.928496	67.9311067
75.3734324	65.9289015	75.421441	70.1725398	66.928496	68.9311067
75.3734324	65.9289015	76.421441	70.1725398	66.928496	70.9311067
75.3734324	65.9289015	76.421441	70.1725398	66.928496	70.9311067
76.3734324	65.9289015	74.421441	69.1725398	67.928496	72.9311067
76.3734324	64.9289015	72.421441	69.1725398	67.928496	78.9311067
77.3734324	64.9289015	72.421441	69.1725398	68.928496	80.9311067
77.3734324	61.9289015	72.421441	69.1725398	71.928496	80.9311067
78.3734324	60.9289015	72.421441	68.1725398	79.928496	73.2258374
78.3734324	59.9289015	73.421441	70.1725398	85.928496	73.8258374
78.3734324	59.9289015	73.421441	70.1725398	74.0258374	74.0258374
77.3734324	55.9289015	73.421441	70.1725398	73.2258374	73.2258374
76.3734324	55.9289015	73.421441	70.1725398	73.0258374	73.0258374
75.3734324	55.9289015	73.421441	67.1725398	72.5258374	72.5258374
75.3734324	58.9289015	74.421441	67.1725398	73.0258374	73.0258374
75.3734324	63.9289015	74.421441	66.1725398	73.6258374	73.6258374
75.3734324	63.9289015	74.421441	66.1725398	73.6258374	73.6258374
74.3734324	63.9289015	74.421441	66.1725398	74.0258374	74.0258374
74.3734324	62.9289015	74.421441	66.1725398	74.2258374	74.2258374
74.3734324	62.9289015	74.421441	66.1725398	85.928496	74.1258374
73.3734324	65.9289015	76.421441	66.1725398	84.928496	74.6258374
74.3734324	66.9289015	76.421441	66.1725398	84.928496	74.7258374
74.3734324	65.9289015	76.421441	66.1725398	84.928496	74.2258374
74.3734324	64.9289015	76.421441	65.1725398	82.928496	73.6258374
74.3734324	64.9289015	76.421441	65.1725398	82.928496	74.0258374
74.3734324	64.9289015	74.421441	65.1725398	82.928496	74.0258374
75.3734324	64.9289015	74.421441	65.1725398	82.928496	86.9311067
77.3734324	64.9289015	74.421441	65.1725398	81.928496	86.9311067
76.3734324	64.9289015	73.421441	65.1725398	81.928496	86.9311067
76.3734324	64.9289015	72.421441	65.1725398	77.928496	84.9311067
76.3734324	64.9289015	72.421441	65.1725398	76.928496	83.9311067
76.3734324	64.9289015	70.421441	65.1725398	76.928496	83.9311067
77.3734324	64.9289015	70.421441	65.1725398	74.928496	79.9311067
77.3734324	65.9289015	70.421441	65.1725398	74.928496	77.9311067
77.3734324	69.9289015	72.421441	65.1725398	74.928496	75.9311067
77.3734324	70.9289015	72.421441	65.1725398	73.928496	75.9311067
77.3734324	71.9289015	72.421441	65.1725398	73.928496	75.9311067

76.3734324	72.9289015	72.421441	66.1725398	73.928496	75.9311067
76.3734324	72.9289015	72.421441	66.1725398	73.928496	75.9311067
74.3734324	72.9289015	72.421441	66.1725398	73.928496	75.9311067
74.3734324	72.9289015	72.421441	67.1725398	76.928496	75.9311067
73.3734324	71.9289015	73.421441	67.1725398	76.928496	75.9311067
73.3734324	71.9289015	73.421441	68.1725398	76.928496	75.9311067
73.3734324	71.9289015	74.421441	69.1725398	72.928496	75.9311067
73.3734324	71.9289015	74.421441	69.1725398	70.928496	75.9311067
73.3734324	70.9289015	74.421441	69.1725398	70.928496	75.9311067
73.3734324	72.9289015	73.421441	69.1725398	70.928496	73.9311067
74.3734324	76.9289015	73.421441	68.1725398	70.928496	72.9311067
75.3734324	77.9289015	71.421441	67.1725398	70.928496	72.9311067
75.3734324	79.9289015	72.421441	67.1725398	71.928496	69.9311067
81.3734324	79.9289015	72.421441	66.1725398	69.928496	70.9311067
81.3734324	79.9289015	72.421441	66.1725398	69.928496	70.9311067
79.3734324	79.9289015	72.421441	66.1725398	69.928496	70.9311067
79.3734324	77.9289015	71.421441	65.1725398	69.928496	70.9311067
79.3734324	76.9289015	74.421441	65.1725398	69.928496	70.9311067
79.3734324	75.9289015	74.421441	65.1725398	69.928496	71.9311067
79.3734324	75.9289015	74.421441	65.1725398	69.928496	71.9311067
79.3734324	75.9289015	74.421441	65.1725398	71.928496	71.9311067
79.3734324	75.9289015	74.421441	66.1725398	71.928496	71.9311067
79.3734324	75.9289015	74.421441	66.1725398	72.928496	71.9311067
79.3734324	74.9289015	73.421441	68.1725398	71.928496	71.9311067
78.3734324	74.9289015	73.421441	68.1725398	72.928496	72.9311067
78.3734324	73.9289015	73.421441	67.1725398	72.928496	71.9311067
78.3734324	73.9289015	73.421441	65.1725398	72.928496	71.9311067
77.3734324	72.9289015	70.421441	64.1725398	72.928496	72.9311067
76.3734324	71.9289015	70.421441	64.1725398	76.928496	72.9311067
76.3734324	69.9289015	70.421441	64.1725398	79.928496	72.9311067
75.3734324	67.9289015	68.421441	64.1725398	81.928496	72.9311067
73.3734324	67.9289015	68.421441	64.1725398	83.928496	73.9311067
73.3734324	65.9289015	68.421441	64.1725398	82.928496	73.9311067
73.3734324	65.9289015	68.421441	64.1725398	82.928496	73.9311067
74.3734324	66.9289015	69.421441	64.1725398	81.928496	73.9311067
74.3734324	67.9289015	69.421441	64.1725398	81.928496	74.9311067
74.3734324	69.9289015	69.421441	65.1725398	81.928496	76.9311067
74.3734324	70.9289015	69.421441	65.1725398	81.928496	77.9311067
74.3734324	70.9289015	69.421441	65.1725398	79.928496	78.9311067
74.3734324	69.9289015	69.421441	65.1725398	77.928496	80.9311067
74.3734324	68.9289015	70.421441	65.1725398	79.928496	80.9311067
74.3734324	67.9289015	70.421441	65.1725398	79.928496	80.9311067
76.3734324	66.9289015	70.421441	65.1725398	79.928496	80.9311067
76.3734324	66.9289015	70.421441	65.1725398	79.928496	79.9311067
76.3734324	66.9289015	71.421441	66.1725398	78.928496	76.9311067

74.3734324	66.9289015	72.421441	67.1725398	78.928496	76.9311067
73.3734324	66.9289015	72.421441	66.1725398	78.928496	76.9311067
71.3734324	66.9289015	72.421441	66.1725398	78.928496	74.9311067
71.3734324	66.9289015	73.421441	66.1725398	77.928496	74.9311067
71.3734324	67.9289015	73.421441	66.1725398	77.928496	73.9311067
70.3734324	67.9289015	73.421441	66.1725398	76.928496	73.9311067
70.3734324	69.9289015	73.421441	66.1725398	76.928496	71.9311067
70.3734324	69.9289015	73.421441	66.1725398	76.928496	70.9311067
70.3734324	69.9289015	73.421441	67.1725398	76.928496	68.9311067
70.3734324	70.9289015	73.421441	67.1725398	76.928496	67.9311067
70.3734324	71.9289015	73.421441	68.1725398	76.928496	68.9311067
71.3734324	72.9289015	73.421441	70.1725398	75.928496	68.9311067
71.3734324	72.9289015	72.421441	70.1725398	77.928496	68.9311067
71.3734324	73.9289015	72.421441	74.1725398	77.928496	68.9311067
70.3734324	73.9289015	72.421441	75.1725398	76.928496	68.9311067
68.3734324	72.9289015	72.421441	75.1725398	76.928496	68.9311067
69.3734324	71.9289015	72.421441	76.1725398	76.928496	68.9311067
70.3734324	71.9289015	72.421441	78.1725398	77.928496	68.9311067
70.3734324	71.9289015	73.421441	78.1725398	76.928496	68.9311067
70.3734324	71.9289015	73.421441	78.1725398	76.928496	68.9311067
68.3734324	70.9289015	73.421441	78.1725398	75.928496	68.9311067
71.3734324	71.9289015	73.421441	77.1725398	74.928496	70.9311067
72.3734324	72.9289015	74.421441	76.1725398	73.928496	73.9311067
72.3734324	72.9289015	74.421441	76.1725398	73.928496	73.9311067
72.3734324	73.9289015	74.421441	74.1725398	73.928496	73.9311067
71.3734324	74.9289015	73.421441	73.1725398	73.928496	70.9311067
72.3734324	74.9289015	73.421441	72.1725398	73.928496	71.9311067
75.3734324	75.9289015	73.421441	70.1725398	73.928496	71.9311067
76.3734324	75.9289015	73.421441	69.1725398	73.928496	70.9311067
76.3734324	76.9289015	74.421441	67.1725398	73.928496	70.9311067
76.3734324	76.9289015	74.421441	67.1725398	73.928496	70.9311067
77.3734324	75.9289015	74.421441	65.1725398	73.928496	70.9311067
77.3734324	74.9289015	74.421441	64.1725398	73.928496	70.9311067
79.3734324	73.9289015	75.421441	64.1725398	74.928496	70.9311067
79.3734324	73.9289015	75.421441	64.1725398	75.928496	70.9311067
79.3734324	73.9289015	75.421441	64.1725398	75.928496	70.9311067
79.3734324	74.9289015	75.421441	64.1725398	75.928496	70.9311067
79.3734324	75.9289015	76.421441	64.1725398	75.928496	72.9311067
79.3734324	76.9289015	76.421441	63.1725398	75.928496	74.9311067
79.3734324	76.9289015	76.421441	63.1725398	74.928496	75.9311067
78.3734324	76.9289015	75.421441	63.1725398	71.928496	76.9311067
77.3734324	75.9289015	75.421441	63.1725398	71.928496	77.9311067
76.3734324	74.9289015	75.421441	67.1725398	71.928496	77.9311067
74.3734324	75.9289015	75.421441	67.1725398	70.928496	77.9311067
74.3734324	75.9289015	75.421441	67.1725398	73.928496	78.9311067

73.3734324	76.9289015	75.421441	67.1725398	74.928496	78.9311067
72.3734324	76.9289015	75.421441	67.1725398	79.928496	75.9311067
72.3734324	75.9289015	73.421441	67.1725398	81.928496	73.9311067
72.3734324	75.9289015	73.421441	69.1725398	81.928496	73.9311067
72.3734324	75.9289015	70.421441	69.1725398	81.928496	73.9311067
72.3734324	75.9289015	70.421441	69.1725398	81.928496	73.9311067
72.3734324	75.9289015	70.421441	69.1725398	81.928496	73.9311067
72.3734324	75.9289015	70.421441	69.1725398	81.928496	73.9311067
72.3734324	75.9289015	69.421441	70.1725398	79.928496	73.9311067
72.3734324	75.9289015	70.421441	70.1725398	79.928496	73.9311067
72.3734324	76.9289015	70.421441	71.1725398	79.928496	72.9311067
72.3734324	76.9289015	70.421441	72.1725398	76.928496	72.9311067
71.3734324	76.9289015	70.421441	72.1725398	76.928496	72.9311067
71.3734324	76.9289015	70.421441	71.1725398	75.928496	72.9311067
71.3734324	78.9289015	71.421441	70.1725398	74.928496	72.9311067
72.3734324	82.9289015	72.421441	69.1725398	73.928496	72.9311067
73.3734324	84.9289015	72.421441	68.1725398	73.928496	72.9311067
76.3734324	84.9289015	74.421441	68.1725398	72.928496	72.9311067
78.3734324	84.9289015	74.421441	68.1725398	72.928496	73.9311067
79.3734324	84.9289015	74.421441	68.1725398	70.928496	74.9311067
79.3734324	84.9289015	76.421441	68.1725398	70.928496	74.9311067
82.3734324	83.9289015	76.421441	68.1725398	70.928496	74.9311067
82.3734324	83.9289015	76.421441	68.1725398	70.928496	74.9311067
82.3734324	82.9289015	76.421441	68.1725398	70.928496	74.9311067
82.3734324	80.9289015	73.421441	70.1725398	70.928496	74.9311067
82.3734324	78.9289015	73.421441	70.1725398	69.928496	74.9311067
82.3734324	71.9289015	73.421441	70.1725398	69.928496	75.9311067
79.3734324	68.9289015	73.421441	71.1725398	69.928496	75.9311067
79.3734324	65.9289015	73.421441	71.1725398	70.928496	75.9311067
76.3734324	65.9289015	76.421441	71.1725398	72.928496	75.9311067
75.3734324	63.9289015	77.421441	71.1725398	73.928496	75.9311067
76.3734324	62.9289015	77.421441	72.1725398	73.928496	74.9311067
76.3734324	62.9289015	77.421441	72.1725398	73.928496	74.9311067
77.3734324	61.9289015	78.421441	71.1725398	74.928496	75.9311067
77.3734324	61.9289015	78.421441	72.1725398	74.928496	75.9311067
77.3734324	61.9289015	78.421441	72.1725398	74.928496	75.9311067
77.3734324	61.9289015	80.421441	71.1725398	70.928496	75.9311067
77.3734324	60.9289015	80.421441	70.1725398	69.928496	75.9311067
76.3734324	60.9289015	80.421441	70.1725398	68.928496	75.9311067
76.3734324	62.9289015	80.421441	69.1725398	69.928496	73.9311067
76.3734324	64.9289015	80.421441	69.1725398	75.928496	69.9311067
75.3734324	64.9289015	79.421441	69.1725398	78.928496	68.9311067
75.3734324	62.9289015	79.421441	69.1725398	80.928496	67.9311067
75.3734324	61.9289015	79.421441	70.1725398	78.928496	68.9311067
75.3734324	61.9289015	78.421441	70.1725398	77.928496	68.9311067

78.3734324	61.9289015	78.421441	68.1725398	75.928496	68.9311067
78.3734324	62.9289015	78.421441	67.1725398	74.928496	68.9311067
78.3734324	62.9289015	78.421441	67.1725398	72.928496	68.9311067
80.3734324	63.9289015	77.421441	66.1725398	71.928496	68.9311067
80.3734324	64.9289015	79.421441	66.1725398	70.928496	68.9311067
80.3734324	64.9289015	79.421441	66.1725398	70.928496	71.9311067
78.3734324	64.9289015	80.421441	67.1725398	71.928496	71.9311067
78.3734324	64.9289015	82.421441	68.1725398	71.928496	71.9311067
77.3734324	63.9289015	83.421441	69.1725398	72.928496	71.9311067
77.3734324	63.9289015	83.421441	70.1725398	72.928496	71.9311067
76.3734324	65.9289015	83.421441	70.1725398	72.928496	71.9311067
76.3734324	66.9289015	83.421441	68.1725398	73.928496	73.9311067
74.3734324	67.9289015	85.421441	67.1725398	74.928496	74.9311067
68.3734324	70.9289015	72.3258374	66.1725398	75.928496	74.9311067
63.3734324	72.9289015	83.421441	66.1725398	79.928496	74.9311067
63.3734324	77.9289015	83.421441	67.1725398	82.928496	73.9311067
63.3734324	81.9289015	82.421441	68.1725398	84.928496	73.9311067
63.3734324	81.9289015	81.421441	69.1725398	84.928496	73.9311067
63.3734324	82.9289015	81.421441	69.1725398	73.0258374	74.9311067
63.3734324	82.9289015	81.421441	68.1725398	85.928496	75.9311067
63.3734324	83.9289015	81.421441	68.1725398	73.8258374	75.9311067
63.3734324	84.9289015	81.421441	70.1725398	74.1258374	75.9311067
63.3734324	85.9289015	81.421441	70.1725398	74.4258374	75.9311067
63.3734324	74.6258374	81.421441	70.1725398	74.6258374	73.9311067
63.3734324	74.5258374	81.421441	70.1725398	74.5258374	72.9311067
63.3734324	74.3258374	81.421441	68.1725398	74.3258374	72.9311067
63.3734324	74.3258374	80.421441	67.1725398	74.3258374	72.9311067
63.3734324	74.5258374	80.421441	67.1725398	74.5258374	72.9311067
63.3734324	74.6258374	80.421441	66.1725398	74.6258374	72.9311067
63.3734324	74.1258374	80.421441	65.1725398	82.928496	72.9311067
63.3734324	73.8258374	80.421441	66.1725398	81.928496	68.9311067
63.3734324	73.5258374	80.421441	66.1725398	79.928496	68.9311067
64.3734324	73.6258374	80.421441	66.1725398	79.928496	67.9311067
67.3734324	74.3258374	80.421441	66.1725398	79.928496	71.9311067
66.3734324	74.0258374	82.421441	66.1725398	79.928496	71.9311067
66.3734324	84.9289015	82.421441	66.1725398	77.928496	71.9311067
66.3734324	82.9289015	83.421441	66.1725398	75.928496	71.9311067
65.3734324	82.9289015	83.421441	66.1725398	75.928496	70.9311067
65.3734324	82.9289015	73.4258374	67.1725398	74.928496	71.9311067
64.3734324	81.9289015	73.0258374	67.1725398	73.928496	72.9311067
64.3734324	80.9289015	72.7258374	66.1725398	72.928496	72.9311067
64.3734324	80.9289015	72.7258374	66.1725398	72.928496	72.9311067
64.3734324	78.9289015	72.4258374	65.1725398	71.928496	71.9311067
63.3734324	78.9289015	72.3258374	64.1725398	71.928496	71.9311067
63.3734324	80.9289015	72.5258374	64.1725398	71.928496	70.9311067

62.3734324	80.9289015	72.3258374	65.1725398	70.928496	69.9311067
62.3734324	80.9289015	84.421441	65.1725398	70.928496	67.9311067
62.3734324	82.9289015	84.421441	66.1725398	70.928496	63.9311067
62.3734324	82.9289015	83.421441	66.1725398	70.928496	62.9311067
64.3734324	82.9289015	83.421441	65.1725398	70.928496	62.9311067
64.3734324	82.9289015	83.421441	65.1725398	71.928496	63.9311067
65.3734324	82.9289015	83.421441	65.1725398	71.928496	64.9311067
64.3734324	81.9289015	80.421441	65.1725398	72.928496	65.9311067
64.3734324	78.9289015	80.421441	66.1725398	73.928496	65.9311067
64.3734324	76.9289015	78.421441	66.1725398	73.928496	65.9311067
63.3734324	74.9289015	78.421441	66.1725398	73.928496	65.9311067
63.3734324	72.9289015	70.421441	66.1725398	77.928496	64.9311067
63.3734324	71.9289015	69.421441	65.1725398	77.928496	64.9311067
66.3734324	71.9289015	69.421441	65.1725398	78.928496	64.9311067
67.3734324	72.9289015	69.421441	66.1725398	79.928496	64.9311067
67.3734324	73.9289015	69.421441	66.1725398	79.928496	63.9311067
67.3734324	75.9289015	70.421441	66.1725398	79.928496	63.9311067
67.3734324	75.9289015	70.421441	66.1725398	79.928496	65.9311067
68.3734324	75.9289015	70.421441	66.1725398	79.928496	64.9311067
69.3734324	74.9289015	72.421441	66.1725398	79.928496	63.9311067
69.3734324	72.9289015	72.421441	66.1725398	79.928496	63.9311067
69.3734324	70.9289015	72.421441	66.1725398	79.928496	61.9311067
70.3734324	68.9289015	72.421441	68.1725398	79.928496	62.9311067
70.3734324	69.9289015	72.421441	69.1725398	77.928496	62.9311067
70.3734324	70.9289015	73.421441	72.1725398	77.928496	63.9311067
70.3734324	72.9289015	73.421441	72.1725398	76.928496	63.9311067
70.3734324	73.9289015	74.421441	72.1725398	76.928496	62.9311067
70.3734324	73.9289015	74.421441	72.1725398	76.928496	62.9311067
70.3734324	73.9289015	74.421441	72.1725398	71.928496	63.9311067
70.3734324	73.9289015	75.421441	72.1725398	71.928496	63.9311067
71.3734324	73.9289015	75.421441	72.1725398	71.928496	65.9311067
71.3734324	73.9289015	75.421441	72.1725398	70.928496	67.9311067
71.3734324	70.9289015	75.421441	72.1725398	70.928496	68.9311067
71.3734324	69.9289015	77.421441	72.1725398	70.928496	69.9311067
73.3734324	68.9289015	78.421441	73.1725398	70.928496	69.9311067
74.3734324	68.9289015	78.421441	73.1725398	70.928496	69.9311067
74.3734324	68.9289015	78.421441	74.1725398	70.928496	67.9311067
74.3734324	68.9289015	78.421441	75.1725398	70.928496	67.9311067
74.3734324	68.9289015	78.421441	76.1725398	75.9258374	67.9311067
73.3734324	68.9289015	78.421441	77.1725398	76.1258374	67.9311067
73.3734324	68.9289015	78.421441	78.1725398	76.3258374	67.9311067
73.3734324	68.9289015	79.421441	78.1725398	76.2258374	66.9311067
73.3734324	68.9289015	81.421441	78.1725398	76.5258374	66.9311067
74.3734324	69.9289015	81.421441	78.1725398	76.8258374	66.9311067
76.3734324	68.9289015	81.421441	79.1725398	76.9258374	64.9311067

76.3734324	68.9289015	84.421441	79.1725398	77.1258374	63.9311067
75.3734324	68.9289015	84.421441	79.1725398	76.7258374	63.9311067
76.3734324	69.9289015	78.421441	78.1725398	75.9258374	62.9311067
78.3734324	69.9289015	78.421441	78.1725398	75.8258374	60.9311067
80.3734324	69.9289015	77.421441	78.1725398	75.7258374	58.9311067
80.3734324	69.9289015	77.421441	78.1725398	75.7258374	58.9311067
80.3734324	70.9289015	77.421441	78.1725398	73.928496	57.9311067
81.3734324	70.9289015	79.421441	78.1725398	72.928496	57.9311067
81.3734324	73.9289015	78.421441	78.1725398	72.928496	58.9311067
81.3734324	74.9289015	78.421441	78.1725398	71.928496	59.9311067
81.3734324	74.9289015	78.421441	77.1725398	71.928496	60.9311067
78.3734324	74.9289015	79.421441	75.1725398	71.928496	61.9311067
77.3734324	74.9289015	79.421441	73.1725398	65.928496	62.9311067
76.3734324	72.9289015	81.421441	71.1725398	64.928496	62.9311067
76.3734324	71.9289015	81.421441	70.1725398	64.928496	63.9311067
76.3734324	66.9289015	81.421441	70.1725398	62.928496	62.9311067
76.3734324	66.9289015	79.421441	69.1725398	60.928496	63.9311067
72.3734324	65.9289015	77.421441	68.1725398	60.928496	63.9311067
72.3734324	65.9289015	76.421441	68.1725398	60.928496	63.9311067
72.3734324	65.9289015	76.421441	67.1725398	59.928496	62.9311067
71.3734324	66.9289015	76.421441	66.1725398	60.928496	62.9311067
70.3734324	67.9289015	75.421441	67.1725398	60.928496	62.9311067
70.3734324	67.9289015	75.421441	68.1725398	61.928496	62.9311067
70.3734324	67.9289015	75.421441	68.1725398	63.928496	62.9311067
70.3734324	68.9289015	75.421441	70.1725398	64.928496	62.9311067
70.3734324	70.9289015	74.421441	73.1725398	64.928496	62.9311067
70.3734324	70.9289015	74.421441	75.1725398	65.928496	63.9311067
70.3734324	72.9289015	74.421441	75.1725398	65.928496	63.9311067
71.3734324	72.9289015	73.421441	75.1725398	65.928496	63.9311067
71.3734324	74.9289015	73.421441	75.1725398	65.928496	63.9311067
71.3734324	76.9289015	72.421441	72.1725398	69.928496	63.9311067
71.3734324	78.9289015	72.421441	70.1725398	71.928496	63.9311067
74.3734324	77.9289015	71.421441	70.1725398	72.928496	64.9311067
74.3734324	75.9289015	70.421441	70.1725398	72.928496	62.9311067
74.3734324	74.9289015	70.421441	71.1725398	72.928496	61.9311067
77.3734324	74.9289015	70.421441	71.1725398	73.928496	61.9311067
78.3734324	73.9289015	70.421441	71.1725398	74.928496	61.9311067
78.3734324	73.9289015	70.421441	70.1725398	76.928496	61.9311067
78.3734324	73.9289015	69.421441	67.1725398	79.928496	61.9311067
77.3734324	72.9289015	69.421441	67.1725398	79.928496	61.9311067
76.3734324	70.9289015	68.421441	68.1725398	80.928496	61.9311067
76.3734324	70.9289015	67.421441	68.1725398	82.928496	61.9311067
76.3734324	70.9289015	67.421441	69.1725398	71.0258374	60.9311067
76.3734324	70.9289015	67.421441	73.1725398	83.928496	60.9311067
76.3734324	69.9289015	68.421441	73.1725398	75.928496	60.9311067

77.3734324	69.9289015	68.421441	73.1725398	70.928496	62.9311067
77.3734324	69.9289015	68.421441	72.1725398	69.928496	72.9311067
77.3734324	68.9289015	69.421441	70.1725398	69.928496	76.9311067
77.3734324	68.9289015	69.421441	69.1725398	73.928496	80.9311067
76.3734324	70.9289015	69.421441	69.1725398	73.928496	81.9311067
76.3734324	70.9289015	69.421441	69.1725398	73.928496	81.9311067
73.3734324	70.9289015	68.421441	69.1725398	70.928496	81.9311067
73.3734324	70.9289015	69.421441	68.1725398	70.928496	81.9311067
71.3734324	70.9289015	70.421441	68.1725398	69.928496	81.9311067
69.3734324	71.9289015	70.421441	67.1725398	68.928496	81.9311067
69.3734324	71.9289015	70.421441	66.1725398	67.928496	81.9311067
69.3734324	71.9289015	68.421441	66.1725398	66.928496	80.9311067
69.3734324	73.9289015	68.421441	67.1725398	65.928496	80.9311067
69.3734324	73.9289015	68.421441	67.1725398	65.928496	79.9311067
69.3734324	73.9289015	68.421441	67.1725398	65.928496	79.9311067
69.3734324	74.9289015	67.421441	67.1725398	65.928496	79.9311067
69.3734324	75.9289015	68.421441	67.1725398	65.928496	80.9311067
68.3734324	78.9289015	69.421441	67.1725398	64.928496	80.9311067
67.3734324	78.9289015	69.421441	66.1725398	64.928496	80.9311067
67.3734324	78.9289015	70.421441	66.1725398	64.928496	78.9311067
66.3734324	75.9289015	70.421441	65.1725398	63.928496	75.9311067
65.3734324	75.9289015	72.421441	66.1725398	63.928496	71.9311067
64.3734324	72.9289015	73.421441	67.1725398	63.928496	65.9311067
61.3734324	67.9289015	73.421441	71.1725398	64.928496	63.9311067
61.3734324	66.9289015	72.421441	73.1725398	65.928496	62.9311067
60.3734324	66.9289015	72.421441	73.1725398	65.928496	62.9311067
60.3734324	66.9289015	71.421441	74.1725398	65.928496	59.9311067
60.3734324	68.9289015	71.421441	73.1725398	65.928496	59.9311067
59.3734324	68.9289015	71.421441	73.1725398	66.928496	59.9311067
59.3734324	70.9289015	70.421441	72.1725398	66.928496	58.9311067
64.3734324	70.9289015	71.421441	70.1725398	66.928496	58.9311067
66.3734324	70.9289015	71.421441	70.1725398	66.928496	58.9311067
66.3734324	70.9289015	71.421441	70.1725398	67.928496	58.9311067
64.3734324	70.9289015	71.421441	70.1725398	68.928496	58.9311067
64.3734324	70.9289015	72.421441	70.1725398	68.928496	61.9311067
63.3734324	70.9289015	72.421441	70.1725398	68.928496	62.9311067
63.3734324	70.9289015	72.421441	70.1725398	68.928496	61.9311067
63.3734324	70.9289015	72.421441	69.1725398	69.928496	61.9311067
62.3734324	69.9289015	72.421441	68.1725398	69.928496	61.9311067
62.3734324	68.9289015	72.421441	68.1725398	69.928496	61.9311067
62.3734324	66.9289015	71.421441	67.1725398	69.928496	61.9311067
62.3734324	66.9289015	71.421441	67.1725398	69.928496	61.9311067
65.3734324	66.9289015	71.421441	67.1725398	69.928496	61.9311067
66.3734324	66.9289015	71.421441	70.1725398	68.928496	61.9311067
66.3734324	66.9289015	69.421441	70.1725398	67.928496	62.9311067

68.3734324	64.9289015	68.421441	70.1725398	67.928496	63.9311067
68.3734324	64.9289015	68.421441	70.1725398	67.928496	63.9311067
68.3734324	66.9289015	69.421441	69.1725398	67.928496	64.9311067
69.3734324	66.9289015	69.421441	69.1725398	68.928496	65.9311067
71.3734324	65.9289015	69.421441	66.1725398	68.928496	67.9311067
73.3734324	65.9289015	70.421441	66.1725398	69.928496	67.9311067
73.3734324	65.9289015	70.421441	66.1725398	71.928496	69.9311067
73.3734324	65.9289015	70.421441	67.1725398	71.928496	69.9311067
73.3734324	65.9289015	70.421441	69.1725398	70.928496	69.9311067
72.3734324	65.9289015	70.421441	72.1725398	69.928496	71.9311067
72.3734324	61.9289015	71.421441	74.1725398	68.928496	73.9311067
72.3734324	60.9289015	71.421441	75.1725398	67.928496	73.9311067
72.3734324	61.9289015	71.421441	74.1725398	67.928496	74.9311067
72.3734324	61.9289015	71.421441	74.1725398	66.928496	74.9311067
72.3734324	61.9289015	71.421441	72.1725398	66.928496	73.9311067
71.3734324	61.9289015	71.421441	70.1725398	66.928496	73.9311067
71.3734324	62.9289015	72.421441	68.1725398	66.928496	72.9311067
71.3734324	62.9289015	72.421441	67.1725398	67.928496	72.9311067
70.3734324	65.9289015	72.421441	68.1725398	67.928496	74.9311067
70.3734324	65.9289015	72.421441	69.1725398	67.928496	74.9311067
68.3734324	65.9289015	72.421441	69.1725398	67.928496	74.9311067
68.3734324	67.9289015	72.421441	69.1725398	68.928496	75.9311067
68.3734324	67.9289015	71.421441	69.1725398	68.928496	75.9311067
68.3734324	67.9289015	71.421441	69.1725398	68.928496	75.9311067
70.3734324	68.9289015	66.421441	69.1725398	69.928496	75.9311067
70.3734324	68.9289015	62.421441	71.1725398	69.928496	75.9311067
70.3734324	71.9289015	62.421441	71.1725398	72.928496	75.9311067
69.3734324	71.9289015	61.421441	72.1725398	74.928496	76.9311067
69.3734324	70.9289015	60.421441	72.1725398	76.928496	77.9311067
69.3734324	70.9289015	58.421441	72.1725398	76.928496	77.9311067
65.3734324	69.9289015	57.421441	72.1725398	76.928496	79.9311067
65.3734324	69.9289015	57.421441	72.1725398	76.928496	82.9311067
65.3734324	69.9289015	54.421441	75.1725398	76.928496	82.9311067
64.3734324	69.9289015	54.421441	75.1725398	76.928496	70.0258374
64.3734324	70.9289015	53.421441	73.1725398	76.928496	69.7258374
64.3734324	71.9289015	55.421441	73.1725398	78.928496	77.9311067
64.3734324	71.9289015	57.421441	73.1725398	78.928496	76.9311067
64.3734324	72.9289015	67.421441	68.1725398	78.928496	75.9311067
68.3734324	72.9289015	70.421441	68.1725398	72.928496	74.9311067
68.3734324	72.9289015	72.421441	66.1725398	72.928496	69.9311067
68.3734324	72.9289015	79.421441	67.1725398	69.928496	69.9311067
70.3734324	72.9289015	80.421441	68.1725398	69.928496	68.9311067
70.3734324	72.9289015	81.421441	68.1725398	70.928496	68.9311067
70.3734324	71.9289015	80.421441	68.1725398	70.928496	68.9311067
73.3734324	71.9289015	80.421441	70.1725398	69.928496	68.9311067

72.3734324	71.9289015	79.421441	71.1725398	69.928496	68.9311067
71.3734324	71.9289015	79.421441	71.1725398	68.928496	66.9311067
71.3734324	71.9289015	76.421441	70.1725398	67.928496	65.9311067
70.3734324	70.9289015	75.421441	70.1725398	67.928496	65.9311067
70.3734324	69.9289015	74.421441	70.1725398	66.928496	65.9311067
69.3734324	68.9289015	74.421441	70.1725398	66.928496	64.9311067
68.3734324	65.9289015	74.421441	69.1725398	67.928496	64.9311067
67.3734324	65.9289015	74.421441	69.1725398	67.928496	64.9311067
66.3734324	63.9289015	74.421441	68.1725398	67.928496	63.9311067
65.3734324	63.9289015	75.421441	68.1725398	67.928496	64.9311067
63.3734324	63.9289015	75.421441	69.1725398	66.928496	64.9311067
63.3734324	64.9289015	75.421441	70.1725398	66.928496	66.9311067
62.3734324	66.9289015	74.421441	70.1725398	67.928496	64.9311067
63.3734324	68.9289015	72.421441	71.1725398	67.928496	60.9311067
64.3734324	68.9289015	70.421441	71.1725398	67.928496	59.9311067
64.3734324	69.9289015	70.421441	71.1725398	67.928496	59.9311067
65.3734324	70.9289015	69.421441	70.1725398	68.928496	59.9311067
66.3734324	70.9289015	69.421441	69.1725398	69.928496	62.9311067
67.3734324	70.9289015	69.421441	69.1725398	69.928496	65.9311067
67.3734324	70.9289015	69.421441	70.1725398	69.928496	66.9311067
67.3734324	71.9289015	69.421441	70.1725398	69.928496	63.9311067
67.3734324	72.9289015	69.421441	71.1725398	69.928496	63.9311067
68.3734324	71.9289015	69.421441	72.1725398	69.928496	63.9311067
68.3734324	70.9289015	69.421441	73.1725398	70.928496	61.9311067
68.3734324	71.9289015	69.421441	73.1725398	71.928496	60.9311067
68.3734324	71.9289015	71.421441	72.1725398	71.928496	59.9311067
68.3734324	71.9289015	71.421441	71.1725398	71.928496	60.9311067
68.3734324	71.9289015	77.421441	72.1725398	72.928496	60.9311067
68.3734324	71.9289015	79.421441	72.1725398	73.928496	61.9311067
68.3734324	71.9289015	81.421441	72.1725398	73.928496	63.9311067
68.3734324	71.9289015	81.421441	72.1725398	73.928496	64.9311067
68.3734324	71.9289015	81.421441	72.1725398	74.928496	64.9311067
68.3734324	71.9289015	81.421441	72.1725398	74.928496	64.9311067
68.3734324	70.9289015	77.421441	73.1725398	74.928496	64.9311067
68.3734324	70.9289015	76.421441	74.1725398	79.928496	64.9311067
68.3734324	69.9289015	76.421441	74.1725398	75.928496	64.9311067
68.3734324	69.9289015	73.421441	74.1725398	75.928496	64.9311067
68.3734324	73.9289015	72.421441	73.1725398	71.928496	64.9311067
68.3734324	73.9289015	71.421441	72.1725398	70.928496	64.9311067
68.3734324	72.9289015	71.421441	72.1725398	68.928496	64.9311067
68.3734324	71.9289015	71.421441	72.1725398	67.928496	64.9311067
68.3734324	71.9289015	71.421441	71.1725398	67.928496	65.9311067
69.3734324	70.9289015	71.421441	70.1725398	65.928496	69.9311067
68.3734324	70.9289015	71.421441	69.1725398	65.928496	69.9311067
68.3734324	69.9289015	71.421441	69.1725398	65.928496	69.9311067

68.3734324	69.9289015	70.421441	69.1725398	65.928496	72.9311067
67.3734324	69.9289015	70.421441	68.1725398	65.928496	64.9311067
67.3734324	69.9289015	71.421441	68.1725398	65.928496	64.9311067
67.3734324	69.9289015	71.421441	68.1725398	65.928496	64.9311067
68.3734324	69.9289015	71.421441	68.1725398	66.928496	65.9311067
68.3734324	69.9289015	71.421441	68.1725398	66.928496	65.9311067
67.3734324	69.9289015	71.421441	67.1725398	66.928496	65.9311067
67.3734324	67.9289015	71.421441	68.1725398	66.928496	64.9311067
67.3734324	67.9289015	70.421441	68.1725398	66.928496	64.9311067
66.3734324	66.9289015	68.421441	69.1725398	65.928496	65.9311067
66.3734324	67.9289015	67.421441	68.1725398	65.928496	66.9311067
67.3734324	67.9289015	67.421441	68.1725398	65.928496	66.9311067
70.3734324	67.9289015	67.421441	68.1725398	65.928496	67.9311067
73.3734324	67.9289015	67.421441	68.1725398	66.928496	67.9311067
73.3734324	68.9289015	67.421441	69.1725398	66.928496	67.9311067
72.0258374	70.9289015	67.421441	72.1725398	65.928496	67.9311067
73.2258374	70.9289015	67.421441	73.1725398	65.928496	67.9311067
73.0258374	69.9289015	64.421441	73.1725398	65.928496	67.9311067
73.7258374	68.9289015	63.421441	73.1725398	65.928496	67.9311067
73.5258374	67.9289015	62.421441	69.1725398	65.928496	67.9311067
73.8258374	67.9289015	63.421441	67.1725398	65.928496	67.9311067
73.8258374	68.9289015	63.421441	66.1725398	64.928496	69.9311067
71.4258374	69.9289015	66.421441	65.1725398	65.928496	71.9311067
71.5258374	70.9289015	72.421441	65.1725398	65.928496	73.9311067
72.3258374	71.9289015	76.421441	66.1725398	65.928496	75.9311067
76.3734324	72.9289015	76.421441	66.1725398	65.928496	74.9311067
73.3734324	72.9289015	76.421441	66.1725398	65.928496	74.9311067
70.3734324	71.9289015	76.421441	66.1725398	65.928496	74.9311067
68.3734324	70.9289015	77.421441	66.1725398	65.928496	73.9311067
68.3734324	70.9289015	77.421441	66.1725398	65.928496	72.9311067
67.3734324	69.9289015	78.421441	66.1725398	65.928496	71.9311067
67.3734324	69.9289015	79.421441	66.1725398	65.928496	71.9311067
67.3734324	69.9289015	81.421441	65.1725398	64.928496	71.9311067
68.3734324	69.9289015	81.421441	64.1725398	65.928496	70.9311067
68.3734324	69.9289015	81.421441	64.1725398	65.928496	70.9311067
68.3734324	68.9289015	81.421441	64.1725398	65.928496	70.9311067
68.3734324	69.9289015	81.421441	65.1725398	65.928496	71.9311067
68.3734324	69.9289015	81.421441	66.1725398	65.928496	72.9311067
68.3734324	69.9289015	81.421441	66.1725398	65.928496	76.9311067
69.3734324	74.9289015	81.421441	66.1725398	66.928496	77.9311067
69.3734324	75.9289015	81.421441	66.1725398	66.928496	76.9311067
69.3734324	75.9289015	81.421441	66.1725398	66.928496	76.9311067
69.3734324	76.9289015	77.421441	69.1725398	67.928496	74.9311067
69.3734324	75.9289015	75.421441	70.1725398	67.928496	74.9311067
69.3734324	75.9289015	71.421441	70.1725398	67.928496	74.9311067

69.3734324	74.9289015	66.421441	69.1725398	68.928496	74.9311067
69.3734324	74.9289015	65.421441	68.1725398	68.928496	73.9311067
69.3734324	74.9289015	64.421441	65.1725398	67.928496	73.9311067
69.3734324	73.9289015	64.421441	65.1725398	67.928496	73.9311067
69.3734324	73.9289015	63.421441	65.1725398	68.928496	72.9311067
69.3734324	71.9289015	63.421441	67.1725398	68.928496	73.9311067
71.3734324	70.9289015	63.421441	70.1725398	68.928496	73.9311067
72.3734324	70.9289015	63.421441	70.1725398	69.928496	73.9311067
72.3734324	71.9289015	63.421441	70.1725398	69.928496	73.9311067
72.3734324	72.9289015	63.421441	70.1725398	69.928496	74.9311067
72.3734324	72.9289015	63.421441	65.1725398	70.928496	74.9311067
72.3734324	72.9289015	63.421441	63.1725398	70.928496	77.9311067
75.3734324	72.9289015	64.421441	63.1725398	77.928496	77.9311067
75.3734324	72.9289015	64.421441	63.1725398	80.928496	78.9311067
75.3734324	73.9289015	64.421441	66.1725398	82.928496	78.9311067
75.3734324	73.9289015	65.421441	66.1725398	82.928496	79.9311067
75.3734324	77.9289015	65.421441	72.1725398	85.928496	78.9311067
75.3734324	79.9289015	65.421441	73.1725398	73.5258374	78.9311067
75.3734324	80.9289015	66.421441	73.1725398	74.0258374	77.9311067
75.3734324	80.9289015	67.421441	73.1725398	74.0258374	73.9311067
76.3734324	81.9289015	67.421441	70.1725398	73.6258374	72.9311067
76.3734324	81.9289015	67.421441	68.1725398	73.2258374	73.9311067
77.3734324	81.9289015	67.421441	68.1725398	73.2258374	74.9311067
76.3734324	81.9289015	67.421441	68.1725398	72.9258374	74.9311067
76.3734324	82.9289015	67.421441	68.1725398	73.0258374	74.9311067
76.3734324	81.9289015	68.421441	72.1725398	73.4258374	74.9311067
76.3734324	77.9289015	68.421441	74.1725398	73.5258374	74.9311067
76.3734324	74.9289015	68.421441	74.1725398	73.4258374	76.9311067
74.3734324	74.9289015	68.421441	75.1725398	73.4258374	76.9311067
74.3734324	74.9289015	68.421441	75.1725398	73.2258374	76.9311067
74.3734324	73.9289015	68.421441	75.1725398	73.3258374	76.9311067
74.3734324	72.9289015	68.421441	73.1725398	73.0258374	76.9311067
74.3734324	70.9289015	68.421441	70.1725398	72.6258374	76.9311067
74.3734324	68.9289015	68.421441	70.1725398	72.3258374	76.9311067
75.3734324	66.9289015	68.421441	70.1725398	72.3258374	76.9311067
75.3734324	65.9289015	68.421441	70.1725398	72.7258374	75.9311067
75.3734324	64.9289015	68.421441	70.1725398	73.1258374	75.9311067
74.3734324	65.9289015	68.421441	70.1725398	73.5258374	81.9311067
74.3734324	67.9289015	68.421441	68.1725398	73.8258374	81.9311067
74.3734324	67.9289015	69.421441	67.1725398	73.9258374	80.9311067
74.3734324	70.9289015	69.421441	67.1725398	74.0258374	79.9311067
74.3734324	73.9289015	69.421441	66.1725398	74.1258374	78.9311067
74.3734324	75.9289015	70.421441	65.1725398	74.4258374	78.9311067
74.3734324	75.9289015	70.421441	66.1725398	74.7258374	77.9311067
74.3734324	74.9289015	69.421441	67.1725398	75.0258374	76.9311067

69.3734324	72.9289015	68.421441	68.1725398	74.1258374	75.9311067
66.3734324	72.9289015	68.421441	69.1725398	73.9258374	75.9311067
65.3734324	70.9289015	65.421441	72.1725398	74.3258374	74.9311067
65.3734324	69.9289015	64.421441	73.1725398	74.1258374	74.9311067
65.3734324	69.9289015	63.421441	73.1725398	74.0258374	74.9311067
65.3734324	69.9289015	63.421441	73.1725398	74.0258374	74.9311067
65.3734324	72.9289015	63.421441	72.1725398	74.1258374	71.9311067
65.3734324	72.9289015	65.421441	73.1725398	74.3258374	70.9311067
65.3734324	73.9289015	66.421441	74.1725398	74.0258374	68.9311067
65.3734324	72.9289015	67.421441	74.1725398	73.8258374	68.9311067
65.3734324	71.9289015	67.421441	75.1725398	73.3258374	67.9311067
66.3734324	70.9289015	67.421441	74.1725398	72.8258374	67.9311067
66.3734324	68.9289015	67.421441	74.1725398	71.6258374	67.9311067
66.3734324	68.9289015	66.421441	72.1725398	70.7258374	67.9311067
68.3734324	67.9289015	66.421441	72.1725398	70.9258374	67.9311067
72.3734324	67.9289015	65.421441	72.1725398	81.928496	65.9311067
72.3734324	66.9289015	63.421441	69.1725398	81.928496	65.9311067
73.3734324	66.9289015	65.421441	70.1725398	81.928496	64.9311067
74.3734324	65.9289015	65.421441	70.1725398	81.928496	64.9311067
74.3734324	65.9289015	65.421441	69.1725398	73.928496	64.9311067
74.3734324	65.9289015	65.421441	68.1725398	73.928496	64.9311067
74.3734324	65.9289015	65.421441	68.1725398	69.928496	65.9311067
74.3734324	68.9289015	66.421441	68.1725398	67.928496	65.9311067
75.3734324	76.9289015	68.421441	68.1725398	66.928496	65.9311067
77.3734324	78.9289015	70.421441	68.1725398	65.928496	65.9311067
77.3734324	79.9289015	70.421441	66.1725398	65.928496	65.9311067
77.3734324	79.9289015	70.421441	65.1725398	67.928496	65.9311067
77.3734324	80.9289015	71.421441	65.1725398	68.928496	66.9311067
76.3734324	79.9289015	72.421441	66.1725398	70.928496	66.9311067
75.3734324	78.9289015	74.421441	65.1725398	71.928496	66.9311067
71.3734324	76.9289015	77.421441	65.1725398	71.928496	66.9311067
71.3734324	74.9289015	79.421441	65.1725398	69.928496	66.9311067
71.3734324	72.9289015	79.421441	66.1725398	67.928496	69.9311067
71.3734324	71.9289015	79.421441	66.1725398	66.928496	69.9311067
71.3734324	71.9289015	79.421441	68.1725398	66.928496	69.9311067
71.3734324	71.9289015	79.421441	68.1725398	65.928496	69.9311067
70.3734324	71.9289015	77.421441	68.1725398	65.928496	69.9311067
70.3734324	71.9289015	76.421441	68.1725398	66.928496	70.9311067
68.3734324	71.9289015	76.421441	68.1725398	69.928496	70.9311067
67.3734324	71.9289015	72.421441	68.1725398	70.928496	77.9311067
67.3734324	71.9289015	70.421441	73.1725398	70.928496	76.9311067
67.3734324	71.9289015	69.421441	74.1725398	68.928496	76.9311067
66.3734324	70.9289015	69.421441	74.1725398	65.928496	77.9311067
66.3734324	69.9289015	69.421441	72.1725398	64.928496	77.9311067
66.3734324	69.9289015	69.421441	72.1725398	64.928496	78.9311067

66.3734324	68.9289015	70.421441	69.1725398	66.928496	78.9311067
66.3734324	69.9289015	71.421441	68.1725398	67.928496	77.9311067
66.3734324	69.9289015	71.421441	68.1725398	68.928496	76.9311067
66.3734324	69.9289015	72.421441	70.1725398	68.928496	76.9311067
66.3734324	69.9289015	72.421441	72.1725398	68.928496	77.9311067
66.3734324	69.9289015	71.421441	74.1725398	67.928496	78.9311067
66.3734324	68.9289015	69.421441	74.1725398	67.928496	79.9311067
68.3734324	68.9289015	69.421441	74.1725398	68.928496	79.9311067
68.3734324	67.9289015	68.421441	72.1725398	69.928496	79.9311067
68.3734324	66.9289015	69.421441	70.1725398	71.928496	80.9311067
70.3734324	66.9289015	69.421441	69.1725398	72.928496	82.9311067
70.3734324	66.9289015	69.421441	69.1725398	72.928496	84.9311067
70.3734324	67.9289015	69.421441	70.1725398	72.928496	84.9311067
70.3734324	67.9289015	67.421441	71.1725398	72.928496	85.9311067
70.3734324	67.9289015	67.421441	71.1725398	72.928496	85.9311067
69.3734324	67.9289015	67.421441	70.1725398	71.928496	73.4258374
70.3734324	67.9289015	66.421441	69.1725398	70.928496	73.4258374
70.3734324	67.9289015	67.421441	68.1725398	68.928496	85.9311067
70.3734324	67.9289015	68.421441	68.1725398	67.928496	85.9311067
70.3734324	67.9289015	68.421441	68.1725398	67.928496	85.9311067
70.3734324	67.9289015	68.421441	68.1725398	66.928496	72.3258374
70.3734324	67.9289015	69.421441	68.1725398	66.928496	72.6258374
70.3734324	68.9289015	69.421441	68.1725398	66.928496	73.6258374
72.3734324	69.9289015	69.421441	68.1725398	66.928496	73.8258374
72.3734324	69.9289015	70.421441	68.1725398	67.928496	74.1258374
73.3734324	68.9289015	70.421441	68.1725398	67.928496	74.0258374
74.3734324	68.9289015	70.421441	69.1725398	68.928496	74.3258374
76.3734324	68.9289015	71.421441	69.1725398	68.928496	74.6258374
76.3734324	68.9289015	71.421441	70.1725398	70.928496	74.9258374
78.3734324	68.9289015	71.421441	70.1725398	71.928496	75.1258374
78.3734324	68.9289015	70.421441	70.1725398	71.928496	75.4258374
78.3734324	68.9289015	70.421441	69.1725398	69.928496	75.0258374
78.3734324	68.9289015	70.421441	69.1725398	69.928496	74.5258374
79.3734324	68.9289015	70.421441	70.1725398	67.928496	74.3258374
79.3734324	67.9289015	71.421441	73.1725398	66.928496	74.5258374
80.3734324	67.9289015	71.421441	73.1725398	66.928496	74.4258374
79.3734324	67.9289015	71.421441	76.1725398	67.928496	74.5258374
78.3734324	67.9289015	71.421441	76.1725398	68.928496	74.3258374
78.3734324	67.9289015	71.421441	76.1725398	68.928496	74.0258374
78.3734324	67.9289015	71.421441	77.1725398	68.928496	74.0258374
79.3734324	68.9289015	71.421441	77.1725398	68.928496	74.2258374
79.3734324	71.9289015	71.421441	77.1725398	68.928496	74.8258374
79.3734324	72.9289015	71.421441	77.1725398	67.928496	74.8258374
79.3734324	75.9289015	71.421441	77.1725398	67.928496	75.2258374
77.3734324	75.9289015	72.421441	76.1725398	67.928496	75.1258374

76.3734324	75.9289015	72.421441	77.1725398	67.928496	75.1258374
76.3734324	71.9289015	72.421441	79.1725398	68.928496	75.2258374
74.3734324	71.9289015	72.421441	79.1725398	68.928496	74.9258374
74.3734324	72.9289015	72.421441	79.1725398	68.928496	74.7258374
73.3734324	72.9289015	72.421441	80.1725398	68.928496	74.7258374
73.3734324	73.9289015	71.421441	78.1725398	78.928496	75.5258374
73.3734324	72.9289015	71.421441	74.1725398	79.928496	75.3258374
73.3734324	71.9289015	71.421441	70.1725398	79.928496	74.7258374
73.3734324	69.9289015	69.421441	70.1725398	76.928496	74.0258374
73.3734324	68.9289015	69.421441	71.1725398	74.928496	73.9258374
73.3734324	67.9289015	69.421441	73.1725398	73.928496	73.6258374
73.3734324	67.9289015	68.421441	75.1725398	72.928496	73.7258374
74.3734324	67.9289015	68.421441	76.1725398	72.928496	74.3258374
75.3734324	67.9289015	68.421441	76.1725398	72.928496	74.3258374
75.3734324	67.9289015	68.421441	75.1725398	73.928496	74.3258374
77.3734324	66.9289015	69.421441	74.1725398	74.928496	74.4258374
77.3734324	66.9289015	70.421441	73.1725398	76.928496	74.5258374
77.3734324	66.9289015	70.421441	73.1725398	78.928496	74.5258374
77.3734324	66.9289015	70.421441	73.1725398	80.928496	74.6258374
77.3734324	67.9289015	70.421441	73.1725398	81.928496	78.9311067
76.3734324	67.9289015	71.421441	74.1725398	81.928496	74.9311067
76.3734324	67.9289015	70.421441	74.1725398	84.928496	74.9311067
76.3734324	67.9289015	71.421441	74.1725398	84.928496	74.9311067
76.3734324	67.9289015	71.421441	74.1725398	84.928496	74.9311067
74.3734324	67.9289015	71.421441	69.1725398	83.928496	74.9311067
74.3734324	69.9289015	70.421441	67.1725398	83.928496	74.9311067
73.3734324	68.9289015	70.421441	66.1725398	82.928496	74.9311067
71.3734324	68.9289015	70.421441	66.1725398	82.928496	74.9311067
70.3734324	68.9289015	70.421441	66.1725398	82.928496	74.9311067
69.3734324	68.9289015	70.421441	67.1725398	82.928496	72.9311067
69.3734324	68.9289015	70.421441	67.1725398	82.928496	66.9311067
69.3734324	70.9289015	71.421441	67.1725398	77.928496	66.9311067
69.3734324	71.9289015	71.421441	67.1725398	76.928496	59.9311067
69.3734324	71.9289015	72.421441	67.1725398	74.928496	59.9311067
69.3734324	71.9289015	72.421441	66.1725398	73.928496	59.9311067
69.3734324	71.9289015	73.421441	66.1725398	73.928496	59.9311067
70.3734324	71.9289015	73.421441	66.1725398	73.928496	58.9311067
70.3734324	71.9289015	74.421441	66.1725398	73.928496	58.9311067
70.3734324	72.9289015	73.421441	66.1725398	75.928496	57.9311067
73.3734324	72.9289015	71.421441	68.1725398	78.928496	57.9311067
74.3734324	72.9289015	70.421441	68.1725398	80.928496	58.9311067
74.3734324	72.9289015	70.421441	70.1725398	81.928496	61.9311067
74.3734324	72.9289015	70.421441	71.1725398	81.928496	63.9311067
73.3734324	72.9289015	68.421441	74.1725398	82.928496	63.9311067
72.3734324	76.9289015	60.421441	76.1725398	82.928496	63.9311067

71.3734324	78.9289015	60.421441	76.1725398	81.928496	64.9311067
71.3734324	78.9289015	60.421441	75.1725398	80.928496	66.9311067
71.3734324	78.9289015	58.421441	71.1725398	78.928496	67.9311067
68.3734324	79.9289015	58.421441	70.1725398	71.928496	68.9311067
68.3734324	79.9289015	56.421441	71.1725398	71.928496	68.9311067
68.3734324	79.9289015	56.421441	72.1725398	66.928496	69.9311067
67.3734324	79.9289015	55.421441	74.1725398	66.928496	69.9311067
67.3734324	79.9289015	55.421441	75.1725398	66.928496	69.9311067
67.3734324	76.9289015	54.421441	75.1725398	66.928496	70.9311067
67.3734324	76.9289015	53.421441	77.1725398	67.928496	72.9311067
67.3734324	74.9289015	53.421441	77.1725398	68.928496	72.9311067
67.3734324	73.9289015	52.421441	76.1725398	68.928496	72.9311067
69.3734324	72.9289015	52.421441	74.1725398	68.928496	72.9311067
69.3734324	71.9289015	53.421441	73.1725398	68.928496	72.9311067
69.3734324	71.9289015	55.421441	70.1725398	67.928496	72.9311067
69.3734324	71.9289015	59.421441	69.1725398	66.928496	72.9311067
69.3734324	70.9289015	59.421441	68.1725398	66.928496	72.9311067
69.3734324	69.9289015	59.421441	67.1725398	65.928496	72.9311067
70.3734324	69.9289015	60.421441	68.1725398	64.928496	72.9311067
71.3734324	70.9289015	60.421441	68.1725398	63.928496	71.9311067
71.3734324	70.9289015	60.421441	70.1725398	63.928496	70.9311067
71.3734324	70.9289015	60.421441	73.1725398	63.928496	69.9311067
71.3734324	71.9289015	61.421441	74.1725398	63.928496	69.9311067
75.3734324	69.9289015	64.421441	74.1725398	64.928496	68.9311067
75.3734324	69.9289015	65.421441	74.1725398	65.928496	68.9311067
76.3734324	70.9289015	65.421441	71.1725398	66.928496	68.9311067
76.3734324	70.9289015	70.421441	71.1725398	66.928496	68.9311067
75.3734324	70.9289015	71.421441	68.1725398	67.928496	68.9311067
71.3734324	70.9289015	73.421441	67.1725398	70.928496	68.9311067
71.3734324	70.9289015	73.421441	68.1725398	73.928496	69.9311067
71.3734324	72.9289015	73.421441	71.1725398	74.928496	70.9311067
72.3734324	72.9289015	73.421441	76.1725398	74.928496	70.9311067
72.3734324	72.9289015	73.421441	76.1725398	74.928496	70.9311067
72.3734324	72.9289015	65.421441	75.1725398	73.928496	74.9311067
72.3734324	72.9289015	64.421441	72.1725398	73.928496	76.9311067
72.3734324	72.9289015	64.421441	72.1725398	73.928496	79.9311067
71.3734324	72.9289015	64.421441	69.1725398	73.928496	83.9311067
71.3734324	71.9289015	64.421441	66.1725398	73.928496	83.9311067
71.3734324	71.9289015	64.421441	64.1725398	73.928496	72.2258374
71.3734324	68.9289015	64.421441	64.1725398	73.928496	83.9311067
71.3734324	66.9289015	64.421441	64.1725398	73.928496	83.9311067
71.3734324	65.9289015	64.421441	66.1725398	73.928496	83.9311067
71.3734324	65.9289015	64.421441	66.1725398	73.928496	82.9311067
71.3734324	65.9289015	64.421441	67.1725398	74.928496	81.9311067
71.3734324	65.9289015	66.421441	69.1725398	74.928496	81.9311067

71.3734324	65.9289015	66.421441	70.1725398	75.928496	81.9311067
71.3734324	65.9289015	67.421441	70.1725398	75.928496	84.9311067
71.3734324	66.9289015	68.421441	71.1725398	76.928496	72.8258374
69.3734324	70.9289015	68.421441	70.1725398	77.928496	85.9311067
67.3734324	72.9289015	70.421441	65.1725398	77.928496	85.9311067
67.3734324	71.9289015	71.421441	65.1725398	77.928496	83.9311067
65.3734324	71.9289015	71.421441	65.1725398	77.928496	82.9311067
64.3734324	71.9289015	71.421441	65.1725398	81.928496	82.9311067
65.3734324	71.9289015	73.421441	67.1725398	84.928496	80.9311067
66.3734324	71.9289015	73.421441	70.1725398	84.928496	77.9311067
66.3734324	70.9289015	73.421441	70.1725398	84.928496	71.9311067
66.3734324	71.9289015	73.421441	70.1725398	71.8258374	69.9311067
67.3734324	71.9289015	73.421441	70.1725398	71.5258374	68.9311067
67.3734324	68.9289015	73.421441	70.1725398	71.4258374	68.9311067
70.3734324	67.9289015	73.421441	71.1725398	71.6258374	66.9311067
71.3734324	67.9289015	73.421441	73.1725398	71.8258374	66.9311067
71.3734324	67.9289015	73.421441	75.1725398	72.0258374	66.9311067
72.3734324	67.9289015	72.421441	76.1725398	71.9258374	66.9311067
72.3734324	67.9289015	72.421441	72.1725398	81.928496	65.9311067
71.3734324	65.9289015	72.421441	70.1725398	80.928496	64.9311067
71.3734324	65.9289015	71.421441	68.1725398	78.928496	63.9311067
71.3734324	64.9289015	70.421441	68.1725398	73.928496	63.9311067
71.3734324	64.9289015	69.421441	68.1725398	70.928496	63.9311067
70.3734324	64.9289015	69.421441	69.1725398	69.928496	62.9311067
70.3734324	63.9289015	69.421441	69.1725398	68.928496	62.9311067
69.3734324	63.9289015	69.421441	69.1725398	68.928496	62.9311067
69.3734324	63.9289015	69.421441	68.1725398	68.928496	60.9311067
69.3734324	62.9289015	69.421441	68.1725398	68.928496	60.9311067
70.3734324	61.9289015	69.421441	68.1725398	69.928496	62.9311067
70.3734324	61.9289015	69.421441	68.1725398	69.928496	63.9311067
70.3734324	57.9289015	69.421441	69.1725398	68.928496	66.9311067
70.3734324	55.9289015	69.421441	70.1725398	67.928496	67.9311067
71.3734324	55.9289015	69.421441	70.1725398	67.928496	68.9311067
71.3734324	54.9289015	69.421441	70.1725398	65.928496	69.9311067
75.3734324	54.9289015	71.421441	68.1725398	65.928496	69.9311067
76.3734324	54.9289015	71.421441	66.1725398	68.928496	71.9311067
76.3734324	54.9289015	72.421441	66.1725398	68.928496	74.9311067
77.3734324	55.9289015	72.421441	66.1725398	75.928496	74.9311067
77.3734324	57.9289015	72.421441	67.1725398	77.928496	75.9311067
77.3734324	58.9289015	72.421441	69.1725398	77.928496	76.9311067
77.3734324	58.9289015	72.421441	69.1725398	77.928496	76.9311067
77.3734324	58.9289015	72.421441	69.1725398	77.928496	76.9311067
77.3734324	60.9289015	73.421441	69.1725398	73.928496	79.9311067
77.3734324	60.9289015	73.421441	70.1725398	72.928496	79.9311067
77.3734324	60.9289015	73.421441	70.1725398	70.928496	78.9311067

70.3734324	59.9289015	73.421441	69.1725398	67.928496	78.9311067
70.3734324	59.9289015	71.421441	68.1725398	65.928496	78.9311067
70.3734324	59.9289015	69.421441	68.1725398	65.928496	79.9311067
73.3734324	65.9289015	69.421441	67.1725398	66.928496	79.9311067
73.3734324	66.9289015	69.421441	67.1725398	67.928496	79.9311067
73.3734324	66.9289015	67.421441	66.1725398	68.928496	79.9311067
73.3734324	65.9289015	66.421441	67.1725398	67.928496	79.9311067
72.3734324	65.9289015	66.421441	68.1725398	65.928496	78.9311067
72.3734324	65.9289015	66.421441	74.1725398	64.928496	78.9311067
72.3734324	65.9289015	65.421441	75.1725398	64.928496	81.9311067
72.3734324	65.9289015	65.421441	75.1725398	65.928496	71.1258374
71.3734324	65.9289015	65.421441	75.1725398	65.928496	71.1258374
70.3734324	65.9289015	63.421441	75.1725398	66.928496	70.7258374
68.3734324	66.9289015	62.421441	71.1725398	66.928496	69.8258374
68.3734324	66.9289015	60.421441	70.1725398	64.928496	77.9311067
68.3734324	66.9289015	68.421441	70.1725398	62.928496	76.9311067
68.3734324	66.9289015	68.421441	71.1725398	62.928496	76.9311067
68.3734324	67.9289015	69.421441	73.1725398	61.928496	75.9311067
68.3734324	67.9289015	69.421441	75.1725398	61.928496	75.9311067
68.3734324	68.9289015	69.421441	75.1725398	60.928496	74.9311067
68.3734324	69.9289015	69.421441	75.1725398	59.928496	74.9311067
67.3734324	69.9289015	69.421441	74.1725398	60.928496	74.9311067
69.3734324	69.9289015	69.421441	73.1725398	60.928496	74.9311067
68.3734324	68.9289015	69.421441	70.1725398	62.928496	74.9311067
68.3734324	68.9289015	69.421441	69.1725398	63.928496	75.9311067
68.3734324	68.9289015	70.421441	68.1725398	64.928496	75.9311067
68.3734324	68.9289015	70.421441	68.1725398	64.928496	76.9311067
68.3734324	68.9289015	71.421441	68.1725398	64.928496	76.9311067
68.3734324	66.9289015	71.421441	68.1725398	64.928496	76.9311067
69.3734324	65.9289015	71.421441	71.1725398	65.928496	77.9311067
69.3734324	65.9289015	71.421441	72.1725398	66.928496	78.9311067
69.3734324	65.9289015	70.421441	72.1725398	67.928496	76.9311067
69.3734324	65.9289015	69.421441	72.1725398	68.928496	75.9311067
69.3734324	65.9289015	69.421441	72.1725398	68.928496	76.9311067
69.3734324	65.9289015	69.421441	72.1725398	68.928496	78.9311067
70.3734324	65.9289015	70.421441	71.1725398	68.928496	79.9311067
71.3734324	65.9289015	70.421441	73.1725398	68.928496	81.9311067
71.3734324	66.9289015	69.421441	74.1725398	68.928496	83.9311067
72.3734324	66.9289015	68.421441	75.1725398	76.928496	83.9311067
72.3734324	68.9289015	68.421441	75.1725398	80.928496	83.9311067
72.3734324	68.9289015	68.421441	75.1725398	82.928496	83.9311067
72.3734324	68.9289015	68.421441	74.1725398	82.928496	83.9311067
72.3734324	68.9289015	68.421441	72.1725398	82.928496	83.9311067
72.3734324	68.9289015	68.421441	72.1725398	79.928496	80.9311067
71.3734324	68.9289015	71.421441	72.1725398	78.928496	73.9311067

71.3734324	68.9289015	71.421441	72.1725398	77.928496	72.9311067
71.3734324	68.9289015	71.421441	72.1725398	75.928496	72.9311067
71.3734324	68.9289015	71.421441	71.1725398	72.928496	73.9311067
71.3734324	70.9289015	68.421441	69.1725398	72.928496	76.9311067
71.3734324	69.9289015	67.421441	69.1725398	70.928496	76.9311067
71.3734324	69.9289015	67.421441	69.1725398	70.928496	76.9311067
69.3734324	70.9289015	67.421441	69.1725398	68.928496	76.9311067
75.3258374	72.9289015	67.421441	70.1725398	67.928496	75.9311067
77.0258374	72.9289015	67.421441	70.1725398	66.928496	75.9311067
77.9258374	72.9289015	67.421441	70.1725398	65.928496	75.9311067
75.7258374	71.9289015	67.421441	70.1725398	64.928496	75.9311067
75.1258374	68.9289015	66.421441	70.1725398	64.928496	74.9311067
74.3258374	67.9289015	66.421441	70.1725398	64.928496	73.9311067
74.6258374	67.9289015	71.421441	71.1725398	64.928496	73.9311067
74.3258374	66.9289015	71.421441	73.1725398	64.928496	73.9311067
86.3734324	65.9289015	69.421441	74.1725398	64.928496	73.9311067
84.3734324	64.9289015	69.421441	74.1725398	64.928496	73.9311067
84.3734324	62.9289015	69.421441	74.1725398	65.928496	75.9311067
81.3734324	61.9289015	69.421441	73.1725398	66.928496	77.9311067
77.3734324	61.9289015	67.421441	72.1725398	67.928496	77.9311067
77.3734324	60.9289015	65.421441	70.1725398	69.928496	76.9311067
76.3734324	60.9289015	65.421441	69.1725398	69.928496	75.9311067
75.3734324	60.9289015	65.421441	70.1725398	70.928496	75.9311067
75.3734324	59.9289015	67.421441	70.1725398	70.928496	74.9311067
74.3734324	59.9289015	68.421441	71.1725398	65.928496	67.9311067
75.3734324	58.9289015	69.421441	76.1725398	65.928496	64.9311067
75.3734324	57.9289015	69.421441	76.1725398	63.928496	64.9311067
76.3734324	57.9289015	69.421441	77.1725398	63.928496	63.9311067
77.3734324	57.9289015	70.421441	77.1725398	62.928496	64.9311067
79.3734324	57.9289015	69.421441	74.1725398	62.928496	66.9311067
79.3734324	57.9289015	69.421441	72.1725398	62.928496	66.9311067
79.3734324	64.9289015	71.421441	72.1725398	64.928496	66.9311067
79.3734324	66.9289015	77.421441	69.1725398	67.928496	67.9311067
79.3734324	68.9289015	80.421441	70.1725398	68.928496	67.9311067
79.3734324	70.9289015	80.421441	70.1725398	67.928496	66.9311067
79.3734324	71.9289015	81.421441	71.1725398	67.928496	66.9311067
79.3734324	71.9289015	72.421441	70.1725398	67.928496	65.9311067
80.3734324	71.9289015	68.421441	69.1725398	67.928496	65.9311067
80.3734324	69.9289015	67.421441	69.1725398	67.928496	65.9311067
80.3734324	68.9289015	67.421441	67.1725398	67.928496	65.9311067
81.3734324	67.9289015	69.421441	67.1725398	65.928496	65.9311067
81.3734324	67.9289015	71.421441	66.1725398	64.928496	65.9311067
81.3734324	67.9289015	71.421441	66.1725398	64.928496	65.9311067
81.3734324	67.9289015	72.421441	66.1725398	64.928496	65.9311067
83.3734324	67.9289015	72.421441	66.1725398	65.928496	65.9311067

83.3734324	67.9289015	73.421441	66.1725398	65.928496	65.9311067
78.3734324	67.9289015	73.421441	66.1725398	66.928496	65.9311067
76.3734324	68.9289015	73.421441	66.1725398	66.928496	64.9311067
72.3734324	68.9289015	73.421441	66.1725398	66.928496	64.9311067
72.3734324	68.9289015	72.421441	68.1725398	66.928496	64.9311067
72.3734324	69.9289015	71.421441	68.1725398	66.928496	64.9311067
72.3734324	69.9289015	71.421441	68.1725398	67.928496	69.9311067
72.3734324	69.9289015	71.421441	69.1725398	72.928496	69.9311067
72.3734324	68.9289015	72.421441	70.1725398	72.928496	72.9311067
72.3734324	68.9289015	72.421441	70.1725398	78.928496	73.9311067
72.3734324	73.9289015	72.421441	72.1725398	79.928496	73.9311067
72.3734324	75.9289015	69.421441	71.1725398	78.928496	74.9311067
72.3734324	76.9289015	67.421441	69.1725398	78.928496	74.9311067
69.3734324	76.9289015	67.421441	68.1725398	78.928496	75.9311067
69.3734324	76.9289015	67.421441	68.1725398	78.928496	75.9311067
64.3734324	75.9289015	64.421441	67.1725398	78.928496	77.9311067
64.3734324	75.9289015	63.421441	67.1725398	78.928496	77.9311067
65.3734324	75.9289015	63.421441	67.1725398	79.928496	77.9311067
65.3734324	73.9289015	63.421441	67.1725398	79.928496	77.9311067
65.3734324	70.9289015	63.421441	68.1725398	80.928496	77.9311067
65.3734324	69.9289015	62.421441	67.1725398	80.928496	77.9311067
65.3734324	69.9289015	61.421441	66.1725398	80.928496	77.9311067
65.3734324	68.9289015	60.421441	67.1725398	80.928496	77.9311067
61.3734324	68.9289015	60.421441	67.1725398	80.928496	76.9311067
59.3734324	67.9289015	61.421441	69.1725398	81.928496	75.9311067
57.3734324	67.9289015	61.421441	70.1725398	71.0258374	75.9311067
57.3734324	66.9289015	62.421441	71.1725398	83.928496	75.9311067
54.3734324	65.9289015	62.421441	72.1725398	71.2258374	74.9311067
54.3734324	65.9289015	62.421441	73.1725398	71.3258374	74.9311067
54.3734324	65.9289015	62.421441	74.1725398	71.5258374	75.9311067
53.3734324	65.9289015	62.421441	74.1725398	71.8258374	75.9311067
53.3734324	65.9289015	62.421441	75.1725398	72.2258374	76.9311067
53.3734324	65.9289015	61.421441	76.1725398	72.2258374	75.9311067
53.3734324	66.9289015	62.421441	76.1725398	72.8258374	75.9311067
52.3734324	66.9289015	62.421441	76.1725398	72.4258374	74.9311067
51.3734324	66.9289015	62.421441	77.1725398	72.1258374	74.9311067
50.3734324	66.9289015	64.421441	77.1725398	71.8258374	74.9311067
50.3734324	70.9289015	65.421441	80.1725398	72.5258374	74.9311067
50.3734324	70.9289015	65.421441	82.1725398	72.4258374	73.9311067
50.3734324	71.9289015	65.421441	82.1725398	78.928496	69.9311067
49.3734324	72.9289015	73.421441	82.1725398	78.928496	67.9311067
49.3734324	72.9289015	75.421441	82.1725398	76.928496	67.9311067
50.3734324	72.9289015	76.421441	81.1725398	71.928496	66.9311067
51.3734324	72.9289015	77.421441	81.1725398	70.928496	65.9311067
54.3734324	71.9289015	77.421441	80.1725398	71.928496	59.9311067

54.3734324	71.9289015	77.421441	80.1725398	71.928496	57.9311067
55.3734324	70.9289015	77.421441	80.1725398	72.928496	57.9311067
62.3734324	69.9289015	79.421441	78.1725398	72.928496	57.9311067
62.3734324	70.9289015	78.421441	77.1725398	73.928496	57.9311067
62.3734324	71.9289015	78.421441	76.1725398	74.928496	56.9311067
63.3734324	72.9289015	77.421441	74.1725398	74.928496	56.9311067
64.3734324	72.9289015	77.421441	74.1725398	75.928496	56.9311067
64.3734324	72.9289015	76.421441	73.1725398	79.928496	56.9311067
64.3734324	71.9289015	75.421441	72.1725398	79.928496	56.9311067
63.3734324	70.9289015	75.421441	72.1725398	79.928496	54.9311067
63.3734324	70.9289015	75.421441	71.1725398	79.928496	54.9311067
63.3734324	70.9289015	75.421441	70.1725398	80.928496	54.9311067
64.3734324	70.9289015	75.421441	70.1725398	80.928496	54.9311067
64.3734324	70.9289015	75.421441	69.1725398	80.928496	54.9311067
64.3734324	72.9289015	75.421441	68.1725398	80.928496	61.9311067
65.3734324	73.9289015	75.421441	69.1725398	80.928496	63.9311067
66.3734324	73.9289015	75.421441	71.1725398	79.928496	64.9311067
66.3734324	74.9289015	75.421441	71.1725398	78.928496	64.9311067
69.3734324	74.9289015	75.421441	72.1725398	77.928496	64.9311067
69.3734324	73.9289015	75.421441	72.1725398	77.928496	64.9311067
69.3734324	71.9289015	75.421441	72.1725398	76.928496	65.9311067
70.3734324	71.9289015	75.421441	73.1725398	76.928496	66.9311067
69.3734324	71.9289015	75.421441	74.1725398	75.928496	66.9311067
69.3734324	71.9289015	75.421441	74.1725398	75.928496	66.9311067
69.3734324	73.9289015	79.421441	74.1725398	72.928496	70.9311067
68.3734324	73.9289015	79.421441	89.1725398	71.928496	71.9311067
68.3734324	74.9289015	82.421441	79.0258374	70.928496	71.9311067
71.3734324	73.9289015	82.421441	79.3258374	70.928496	72.9311067
71.3734324	72.9289015	84.421441	79.3258374	68.928496	71.9311067
71.3734324	70.9289015	84.421441	79.3258374	67.928496	71.9311067
72.3734324	68.9289015	84.421441	76.8258374	67.928496	71.9311067
72.3734324	66.9289015	83.421441	75.7258374	67.928496	71.9311067
72.3734324	66.9289015	83.421441	75.5258374	66.928496	72.9311067
72.3734324	66.9289015	80.421441	75.0258374	66.928496	72.9311067
72.3734324	66.9289015	80.421441	74.6258374	67.928496	72.9311067
71.3734324	68.9289015	80.421441	74.5258374	67.928496	72.9311067
70.3734324	68.9289015	80.421441	74.2258374	68.928496	69.9311067
68.3734324	70.9289015	79.421441	85.1725398	68.928496	67.9311067
68.3734324	70.9289015	79.421441	83.1725398	68.928496	67.9311067
68.3734324	70.9289015	79.421441	81.1725398	66.928496	67.9311067
68.3734324	70.9289015	78.421441	79.1725398	65.928496	67.9311067
68.3734324	69.9289015	73.421441	76.1725398	65.928496	67.9311067
68.3734324	69.9289015	68.421441	76.1725398	65.928496	67.9311067
68.3734324	69.9289015	68.421441	71.1725398	65.928496	69.9311067
67.3734324	69.9289015	68.421441	71.1725398	65.928496	69.9311067

67.3734324	69.9289015	68.421441	67.1725398	65.928496	69.9311067
68.3734324	69.9289015	68.421441	67.1725398	66.928496	70.9311067
71.3734324	69.9289015	68.421441	67.1725398	67.928496	71.9311067
71.3734324	67.9289015	69.421441	67.1725398	66.928496	72.9311067
72.3734324	66.9289015	69.421441	71.1725398	66.928496	73.9311067
72.3734324	66.9289015	69.421441	73.1725398	65.928496	75.9311067
72.3734324	67.9289015	69.421441	74.1725398	65.928496	79.9311067
72.3734324	67.9289015	70.421441	73.1725398	65.928496	80.9311067
70.3734324	69.9289015	72.421441	73.1725398	65.928496	81.9311067
69.3734324	71.9289015	72.421441	68.1725398	64.928496	81.9311067
69.3734324	71.9289015	72.421441	65.1725398	64.928496	81.9311067
68.3734324	70.9289015	72.421441	65.1725398	64.928496	81.9311067
66.3734324	69.9289015	71.421441	63.1725398	64.928496	81.9311067
66.3734324	69.9289015	71.421441	63.1725398	64.928496	81.9311067
66.3734324	68.9289015	71.421441	63.1725398	65.928496	81.9311067
66.3734324	69.9289015	71.421441	63.1725398	65.928496	81.9311067
66.3734324	69.9289015	71.421441	64.1725398	66.928496	81.9311067
65.3734324	69.9289015	71.421441	64.1725398	66.928496	81.9311067
65.3734324	69.9289015	71.421441	65.1725398	67.928496	80.9311067
64.3734324	68.9289015	69.421441	65.1725398	68.928496	80.9311067
64.3734324	68.9289015	71.421441	65.1725398	68.928496	77.9311067
64.3734324	68.9289015	71.421441	66.1725398	68.928496	77.9311067
64.3734324	68.9289015	71.421441	67.1725398	68.928496	76.9311067
64.3734324	67.9289015	71.421441	67.1725398	68.928496	76.9311067
64.3734324	67.9289015	71.421441	68.1725398	68.928496	76.9311067
64.3734324	68.9289015	71.421441	70.1725398	68.928496	76.9311067
65.3734324	67.9289015	70.421441	70.1725398	68.928496	76.9311067
65.3734324	67.9289015	70.421441	74.1725398	68.928496	77.9311067
65.3734324	67.9289015	70.421441	74.1725398	68.928496	77.9311067
65.3734324	67.9289015	70.421441	74.1725398	68.928496	77.9311067
66.3734324	67.9289015	69.421441	74.1725398	68.928496	77.9311067
68.3734324	68.9289015	67.421441	72.1725398	71.928496	77.9311067
68.3734324	68.9289015	67.421441	72.1725398	71.928496	77.9311067
70.3734324	69.9289015	67.421441	69.1725398	72.928496	76.9311067
70.3734324	69.9289015	67.421441	70.1725398	72.928496	77.9311067
70.3734324	68.9289015	68.421441	70.1725398	72.928496	78.9311067
71.3734324	66.9289015	67.421441	72.1725398	72.928496	78.9311067
71.3734324	66.9289015	65.421441	71.1725398	70.928496	78.9311067
72.3734324	66.9289015	64.421441	71.1725398	66.928496	78.9311067
72.3734324	66.9289015	62.421441	66.1725398	65.928496	78.9311067
72.3734324	66.9289015	59.421441	64.1725398	65.928496	78.9311067
72.3734324	66.9289015	59.421441	64.1725398	66.928496	78.9311067
72.3734324	67.9289015	60.421441	65.1725398	66.928496	75.9311067
72.3734324	68.9289015	60.421441	65.1725398	66.928496	73.9311067
72.3734324	68.9289015	60.421441	66.1725398	68.928496	72.9311067

75.3734324	68.9289015	60.421441	66.1725398	69.928496	72.9311067
75.3734324	69.9289015	61.421441	67.1725398	73.928496	72.9311067
75.3734324	69.9289015	61.421441	67.1725398	74.928496	72.9311067
74.3734324	70.9289015	63.421441	67.1725398	74.928496	72.9311067
74.3734324	72.9289015	66.421441	67.1725398	73.928496	72.9311067
74.3734324	74.9289015	68.421441	67.1725398	73.928496	73.9311067
74.3734324	75.9289015	68.421441	67.1725398	73.928496	74.9311067
74.3734324	76.9289015	70.421441	67.1725398	73.928496	75.9311067
74.3734324	77.9289015	71.421441	66.1725398	74.928496	75.9311067
74.3734324	77.9289015	71.421441	66.1725398	74.928496	75.9311067
74.3734324	78.9289015	71.421441	66.1725398	74.928496	75.9311067
74.3734324	78.9289015	71.421441	69.1725398	74.928496	75.9311067
74.3734324	78.9289015	71.421441	71.1725398	74.928496	74.9311067
74.3734324	72.9289015	71.421441	72.1725398	73.928496	68.9311067
74.3734324	72.9289015	71.421441	73.1725398	73.928496	66.9311067
73.3734324	68.9289015	71.421441	74.1725398	74.928496	66.9311067
73.3734324	66.9289015	71.421441	74.1725398	74.928496	66.9311067
73.3734324	65.9289015	70.421441	73.1725398	74.928496	66.9311067
74.3734324	64.9289015	70.421441	72.1725398	74.928496	66.9311067
74.3734324	63.9289015	70.421441	71.1725398	75.928496	66.9311067
74.3734324	63.9289015	70.421441	70.1725398	76.928496	66.9311067
74.3734324	63.9289015	70.421441	70.1725398	77.928496	66.9311067
74.3734324	63.9289015	70.421441	71.1725398	78.928496	67.9311067
74.3734324	62.9289015	66.421441	72.1725398	78.928496	67.9311067
74.3734324	61.9289015	62.421441	74.1725398	78.928496	66.9311067
75.3734324	61.9289015	61.421441	75.1725398	78.928496	61.9311067
75.3734324	61.9289015	61.421441	76.1725398	78.928496	59.9311067
75.3734324	61.9289015	62.421441	77.1725398	78.928496	58.9311067
75.3734324	63.9289015	62.421441	78.1725398	77.928496	58.9311067
76.3734324	64.9289015	62.421441	78.1725398	76.928496	58.9311067
77.3734324	64.9289015	62.421441	77.1725398	76.928496	58.9311067
77.3734324	64.9289015	63.421441	74.1725398	76.928496	59.9311067
77.3734324	63.9289015	63.421441	73.1725398	77.928496	59.9311067
77.3734324	63.9289015	64.421441	72.1725398	77.928496	57.9311067
76.3734324	63.9289015	65.421441	71.1725398	77.928496	57.9311067
75.3734324	63.9289015	65.421441	72.1725398	76.928496	57.9311067
74.3734324	64.9289015	65.421441	72.1725398	71.928496	57.9311067
74.3734324	65.9289015	66.421441	74.1725398	70.928496	57.9311067
74.3734324	64.9289015	67.421441	75.1725398	68.928496	58.9311067
74.3734324	63.9289015	66.421441	76.1725398	68.928496	58.9311067
74.3734324	62.9289015	66.421441	76.1725398	68.928496	59.9311067
74.3734324	62.9289015	66.421441	76.1725398	68.928496	59.9311067
72.3734324	61.9289015	66.421441	74.1725398	70.928496	60.9311067
71.3734324	61.9289015	66.421441	74.1725398	71.928496	64.9311067
69.3734324	61.9289015	66.421441	74.1725398	73.928496	65.9311067

69.3734324	59.9289015	66.421441	75.1725398	73.928496	65.9311067
69.3734324	58.9289015	65.421441	75.1725398	73.928496	61.9311067
68.3734324	59.9289015	63.421441	75.1725398	76.928496	59.9311067
70.3734324	64.9289015	62.421441	75.1725398	76.928496	57.9311067
70.3734324	64.9289015	62.421441	75.1725398	73.928496	57.9311067
73.3734324	64.9289015	60.421441	75.1725398	72.928496	57.9311067
74.3734324	64.9289015	60.421441	75.1725398	69.928496	58.9311067
74.3734324	64.9289015	60.421441	75.1725398	66.928496	59.9311067
74.3734324	65.9289015	60.421441	75.1725398	64.928496	59.9311067
75.3734324	65.9289015	60.421441	74.1725398	63.928496	58.9311067
76.3734324	65.9289015	60.421441	75.1725398	63.928496	57.9311067
76.3734324	65.9289015	56.421441	75.1725398	63.928496	57.9311067
76.3734324	66.9289015	55.421441	75.1725398	63.928496	57.9311067
76.3734324	66.9289015	53.421441	77.1725398	63.928496	56.9311067
76.3734324	66.9289015	53.421441	77.1725398	63.928496	57.9311067
78.3734324	66.9289015	51.421441	79.1725398	63.928496	57.9311067
78.3734324	65.9289015	54.421441	79.1725398	63.928496	59.9311067
77.3734324	65.9289015	58.421441	80.1725398	63.928496	61.9311067
77.3734324	65.9289015	58.421441	80.1725398	63.928496	61.9311067
77.3734324	65.9289015	61.421441	80.1725398	63.928496	61.9311067
77.3734324	65.9289015	62.421441	78.1725398	65.928496	62.9311067
76.3734324	65.9289015	62.421441	77.1725398	65.928496	62.9311067
70.3734324	66.9289015	62.421441	75.1725398	65.928496	62.9311067
70.3734324	66.9289015	62.421441	72.1725398	67.928496	66.9311067
65.3734324	66.9289015	62.421441	70.1725398	69.928496	66.9311067
65.3734324	69.9289015	62.421441	69.1725398	70.928496	66.9311067
65.3734324	69.9289015	61.421441	69.1725398	70.928496	66.9311067
63.3734324	69.9289015	61.421441	69.1725398	70.928496	67.9311067
63.3734324	69.9289015	61.421441	70.1725398	69.928496	67.9311067
62.3734324	70.9289015	61.421441	70.1725398	68.928496	67.9311067
62.3734324	73.9289015	61.421441	71.1725398	68.928496	67.9311067
61.3734324	73.9289015	61.421441	71.1725398	68.928496	67.9311067
61.3734324	73.9289015	60.421441	72.1725398	68.928496	67.9311067
61.3734324	73.9289015	60.421441	72.1725398	67.928496	65.9311067
60.3734324	73.9289015	60.421441	72.1725398	67.928496	62.9311067
60.3734324	73.9289015	60.421441	71.1725398	67.928496	60.9311067
57.3734324	72.9289015	59.421441	71.1725398	67.928496	60.9311067
56.3734324	69.9289015	59.421441	71.1725398	64.928496	61.9311067
56.3734324	68.9289015	59.421441	71.1725398	63.928496	60.9311067
56.3734324	67.9289015	59.421441	72.1725398	63.928496	60.9311067
54.3734324	68.9289015	59.421441	75.1725398	65.928496	62.9311067
53.3734324	67.9289015	59.421441	75.1725398	67.928496	62.9311067
51.3734324	67.9289015	57.421441	75.1725398	68.928496	61.9311067
50.3734324	66.9289015	57.421441	75.1725398	69.928496	60.9311067
49.3734324	66.9289015	57.421441	75.1725398	73.928496	60.9311067

49.3734324	65.9289015	57.421441	75.1725398	76.928496	62.9311067
47.3734324	64.9289015	57.421441	76.1725398	76.928496	63.9311067
47.3734324	63.9289015	59.421441	77.1725398	77.928496	64.9311067
46.3734324	62.9289015	59.421441	78.1725398	73.928496	64.9311067
46.3734324	62.9289015	59.421441	78.1725398	69.928496	63.9311067
46.3734324	62.9289015	59.421441	78.1725398	69.928496	63.9311067
45.3734324	63.9289015	60.421441	78.1725398	67.928496	63.9311067
45.3734324	64.9289015	60.421441	76.1725398	67.928496	64.9311067
46.3734324	64.9289015	57.421441	76.1725398	67.928496	64.9311067
46.3734324	64.9289015	56.421441	74.1725398	66.928496	64.9311067
46.3734324	64.9289015	57.421441	74.1725398	65.928496	64.9311067
47.3734324	63.9289015	57.421441	74.1725398	64.928496	64.9311067
47.3734324	62.9289015	57.421441	74.1725398	65.928496	64.9311067
47.3734324	62.9289015	58.421441	74.1725398	65.928496	63.9311067
48.3734324	62.9289015	59.421441	74.1725398	66.928496	63.9311067
48.3734324	64.9289015	61.421441	74.1725398	65.928496	64.9311067
50.3734324	66.9289015	61.421441	74.1725398	66.928496	64.9311067
53.3734324	66.9289015	61.421441	75.1725398	66.928496	64.9311067
55.3734324	66.9289015	60.421441	75.1725398	67.928496	62.9311067
54.3734324	66.9289015	60.421441	75.1725398	67.928496	61.9311067
54.3734324	69.9289015	61.421441	74.1725398	67.928496	60.9311067
54.3734324	72.9289015	63.421441	72.1725398	67.928496	60.9311067
55.3734324	72.9289015	63.421441	71.1725398	68.928496	60.9311067
56.3734324	72.9289015	63.421441	72.1725398	69.928496	60.9311067
56.3734324	73.9289015	64.421441	73.1725398	69.928496	60.9311067
59.3734324	70.9289015	64.421441	75.1725398	69.928496	60.9311067
59.3734324	67.9289015	64.421441	75.1725398	69.928496	60.9311067
57.3734324	66.9289015	66.421441	77.1725398	69.928496	60.9311067
56.3734324	64.9289015	66.421441	77.1725398	69.928496	60.9311067
56.3734324	63.9289015	66.421441	77.1725398	69.928496	60.9311067
56.3734324	63.9289015	65.421441	75.1725398	69.928496	60.9311067
57.3734324	63.9289015	65.421441	75.1725398	69.928496	60.9311067
57.3734324	63.9289015	64.421441	75.1725398	69.928496	60.9311067
58.3734324	62.9289015	64.421441	74.1725398	69.928496	60.9311067
61.3734324	62.9289015	64.421441	71.1725398	71.928496	61.9311067
64.3734324	62.9289015	65.421441	70.1725398	71.928496	60.9311067
64.3734324	62.9289015	65.421441	70.1725398	72.928496	58.9311067
64.3734324	62.9289015	65.421441	70.1725398	71.928496	58.9311067
63.3734324	62.9289015	63.421441	69.1725398	71.928496	58.9311067
63.3734324	62.9289015	61.421441	69.1725398	71.928496	58.9311067
62.3734324	61.9289015	61.421441	69.1725398	70.928496	58.9311067
60.3734324	61.9289015	61.421441	69.1725398	70.928496	58.9311067
59.3734324	56.9289015	61.421441	70.1725398	71.928496	58.9311067
59.3734324	56.9289015	62.421441	71.1725398	71.928496	58.9311067
59.3734324	55.9289015	62.421441	71.1725398	71.928496	58.9311067

59.3734324	55.9289015	62.421441	72.1725398	72.928496	59.9311067
58.3734324	55.9289015	62.421441	72.1725398	73.928496	59.9311067
58.3734324	55.9289015	66.421441	72.1725398	73.928496	59.9311067
56.3734324	55.9289015	66.421441	72.1725398	72.928496	59.9311067
55.3734324	56.9289015	66.421441	72.1725398	71.928496	59.9311067
55.3734324	56.9289015	68.421441	72.1725398	70.928496	59.9311067
54.3734324	59.9289015	69.421441	73.1725398	68.928496	59.9311067
54.3734324	60.9289015	69.421441	73.1725398	68.928496	59.9311067
54.3734324	60.9289015	67.421441	77.1725398	68.928496	60.9311067
54.3734324	59.9289015	66.421441	77.1725398	69.928496	60.9311067
54.3734324	59.9289015	65.421441	77.1725398	69.928496	61.9311067
54.3734324	59.9289015	63.421441	77.1725398	69.928496	62.9311067
59.3734324	59.9289015	63.421441	77.1725398	71.928496	62.9311067
60.3734324	62.9289015	62.421441	78.1725398	72.928496	62.9311067
60.3734324	62.9289015	62.421441	78.1725398	72.928496	62.9311067
62.3734324	62.9289015	60.421441	80.1725398	72.928496	62.9311067
62.3734324	62.9289015	59.421441	80.1725398	70.928496	63.9311067
62.3734324	62.9289015	59.421441	68.4258374	69.928496	64.9311067
62.3734324	62.9289015	59.421441	68.4258374	68.928496	66.9311067
63.3734324	68.9289015	59.421441	82.1725398	67.928496	67.9311067
63.3734324	68.9289015	59.421441	80.1725398	67.928496	67.9311067
63.3734324	71.9289015	58.421441	77.1725398	68.928496	66.9311067
63.3734324	72.9289015	58.421441	77.1725398	68.928496	66.9311067
63.3734324	72.9289015	58.421441	75.1725398	68.928496	66.9311067
63.3734324	76.9289015	58.421441	74.1725398	67.928496	67.9311067
64.3734324	77.9289015	63.421441	73.1725398	67.928496	67.9311067
64.3734324	77.9289015	66.421441	73.1725398	67.928496	67.9311067
66.3734324	76.9289015	66.421441	74.1725398	67.928496	66.9311067
72.3734324	76.9289015	67.421441	74.1725398	66.928496	65.9311067
75.3734324	76.9289015	67.421441	74.1725398	66.928496	64.9311067
75.3734324	76.9289015	69.421441	75.1725398	66.928496	63.9311067
73.3734324	75.9289015	70.421441	74.1725398	67.928496	63.9311067
72.3734324	73.9289015	70.421441	74.1725398	68.928496	62.9311067
72.3734324	71.9289015	70.421441	75.1725398	68.928496	62.9311067
72.3734324	70.9289015	70.421441	76.1725398	69.928496	62.9311067
72.3734324	70.9289015	70.421441	76.1725398	69.928496	62.9311067
73.3734324	70.9289015	70.421441	77.1725398	68.928496	62.9311067
73.3734324	70.9289015	68.421441	77.1725398	67.928496	62.9311067
73.3734324	69.9289015	68.421441	77.1725398	67.928496	62.9311067
73.3734324	66.9289015	68.421441	78.1725398	67.928496	62.9311067
73.3734324	64.9289015	68.421441	78.1725398	67.928496	62.9311067
73.3734324	62.9289015	68.421441	78.1725398	67.928496	63.9311067
74.3734324	62.9289015	68.421441	76.1725398	68.928496	63.9311067
74.3734324	62.9289015	68.421441	77.1725398	68.928496	63.9311067
74.3734324	62.9289015	67.421441	78.1725398	69.928496	63.9311067

74.3734324	63.9289015	66.421441	78.1725398	70.928496	63.9311067
74.3734324	63.9289015	66.421441	77.1725398	72.928496	63.9311067
75.3734324	62.9289015	65.421441	77.1725398	74.928496	63.9311067
75.3734324	62.9289015	65.421441	75.1725398	75.928496	62.9311067
75.3734324	63.9289015	63.421441	75.1725398	74.928496	62.9311067
75.3734324	64.9289015	63.421441	75.1725398	74.928496	63.9311067
64.3734324	65.9289015	63.421441	75.1725398	74.928496	65.9311067
64.3734324	66.9289015	63.421441	75.1725398	73.928496	64.9311067
65.3734324	66.9289015	63.421441	77.1725398	73.928496	63.9311067
66.3734324	66.9289015	64.421441	77.1725398	72.928496	63.9311067
66.3734324	66.9289015	63.421441	78.1725398	72.928496	64.9311067
65.3734324	68.9289015	63.421441	78.1725398	72.928496	64.9311067
65.3734324	68.9289015	63.421441	76.1725398	71.928496	64.9311067
62.3734324	68.9289015	61.421441	74.1725398	70.928496	64.9311067
62.3734324	67.9289015	61.421441	74.1725398	70.928496	64.9311067
62.3734324	67.9289015	61.421441	75.1725398	70.928496	64.9311067
63.3734324	67.9289015	61.421441	75.1725398	70.928496	64.9311067
63.3734324	68.9289015	66.421441	75.1725398	70.928496	64.9311067
63.3734324	68.9289015	66.421441	74.1725398	70.928496	65.9311067
64.3734324	71.9289015	64.421441	73.1725398	70.928496	65.9311067
64.3734324	71.9289015	63.421441	73.1725398	70.928496	65.9311067
64.3734324	70.9289015	62.421441	73.1725398	70.928496	67.9311067
64.3734324	70.9289015	62.421441	74.1725398	70.928496	68.9311067
64.3734324	70.9289015	62.421441	74.1725398	70.928496	68.9311067
67.3734324	69.9289015	63.421441	77.1725398	70.928496	71.9311067
67.3734324	69.9289015	63.421441	77.1725398	70.928496	71.9311067
72.3734324	69.9289015	63.421441	77.1725398	70.928496	72.9311067
72.3734324	69.9289015	64.421441	77.1725398	70.928496	74.9311067
72.3734324	69.9289015	64.421441	74.1725398	70.928496	74.9311067
73.3734324	70.9289015	66.421441	73.1725398	70.928496	75.9311067
73.3734324	70.9289015	67.421441	73.1725398	70.928496	75.9311067
73.3734324	69.9289015	67.421441	73.1725398	71.928496	76.9311067
73.3734324	68.9289015	66.421441	73.1725398	71.928496	74.9311067
74.3734324	67.9289015	67.421441	74.1725398	70.928496	75.9311067
80.3734324	66.9289015	67.421441	74.1725398	70.928496	76.9311067
81.3734324	65.9289015	70.421441	74.1725398	70.928496	77.9311067
83.3734324	64.9289015	70.421441	74.1725398	70.928496	77.9311067
83.3734324	64.9289015	70.421441	75.1725398	70.928496	77.9311067
83.3734324	65.9289015	70.421441	75.1725398	70.928496	77.9311067
83.3734324	65.9289015	70.421441	75.1725398	70.928496	77.9311067
83.3734324	65.9289015	70.421441	75.1725398	72.928496	76.9311067
82.3734324	67.9289015	70.421441	74.1725398	72.928496	76.9311067
82.3734324	68.9289015	70.421441	74.1725398	72.928496	76.9311067
82.3734324	68.9289015	70.421441	74.1725398	71.928496	75.9311067
81.3734324	69.9289015	70.421441	75.1725398	70.928496	75.9311067

80.3734324	69.9289015	70.421441	75.1725398	69.928496	74.9311067
80.3734324	69.9289015	70.421441	73.1725398	69.928496	74.9311067
80.3734324	68.9289015	70.421441	71.1725398	69.928496	73.9311067
80.3734324	68.9289015	71.421441	71.1725398	69.928496	71.9311067
80.3734324	68.9289015	69.421441	71.1725398	69.928496	72.9311067
78.3734324	68.9289015	68.421441	71.1725398	69.928496	73.9311067
78.3734324	67.9289015	68.421441	71.1725398	69.928496	73.9311067
78.3734324	67.9289015	68.421441	70.1725398	69.928496	74.9311067
78.3734324	66.9289015	68.421441	71.1725398	69.928496	73.9311067
78.3734324	66.9289015	67.421441	70.1725398	68.928496	73.9311067
78.3734324	66.9289015	67.421441	70.1725398	68.928496	73.9311067
78.3734324	66.9289015	67.421441	70.1725398	68.928496	74.9311067
77.3734324	66.9289015	66.421441	70.1725398	68.928496	74.9311067
77.3734324	66.9289015	66.421441	70.1725398	68.928496	74.9311067
77.3734324	66.9289015	67.421441	72.1725398	68.928496	75.9311067
77.3734324	65.9289015	69.421441	73.1725398	68.928496	75.9311067
77.3734324	65.9289015	69.421441	74.1725398	69.928496	75.9311067
77.3734324	75.9289015	70.421441	73.1725398	69.928496	77.9311067
77.3734324	79.9289015	71.421441	72.1725398	69.928496	77.9311067
74.3734324	79.9289015	71.421441	73.1725398	69.928496	76.9311067
73.3734324	79.9289015	71.421441	75.1725398	70.928496	76.9311067
73.3734324	77.9289015	79.421441	77.1725398	70.928496	78.9311067
73.3734324	74.9289015	80.421441	79.1725398	72.928496	78.9311067
73.3734324	72.9289015	82.421441	78.1725398	72.928496	79.9311067
73.3734324	71.9289015	82.421441	77.1725398	72.928496	80.9311067
73.3734324	71.9289015	85.421441	76.1725398	73.928496	81.9311067
73.3734324	71.9289015	85.421441	75.1725398	74.928496	81.9311067
73.3734324	71.9289015	87.421441	74.1725398	75.928496	82.9311067
74.3734324	71.9289015	87.421441	74.1725398	75.928496	84.9311067
74.3734324	71.9289015	87.421441	75.1725398	75.928496	86.9311067
74.3734324	71.9289015	85.421441	75.1725398	76.928496	86.9311067
74.3734324	70.9289015	85.421441	76.1725398	76.928496	85.9311067
74.3734324	70.9289015	85.421441	76.1725398	76.928496	85.9311067
74.3734324	70.9289015	84.421441	76.1725398	75.928496	85.9311067
74.3734324	69.9289015	82.421441	76.1725398	75.928496	85.9311067
74.3734324	69.9289015	83.421441	75.1725398	74.928496	85.9311067
74.3734324	69.9289015	83.421441	73.1725398	74.928496	83.9311067
74.3734324	69.9289015	83.421441	72.1725398	72.928496	83.9311067
74.3734324	71.9289015	83.421441	72.1725398	72.928496	83.9311067
74.3734324	71.9289015	83.421441	72.1725398	69.928496	83.9311067
75.3734324	71.9289015	84.421441	72.1725398	68.928496	83.9311067
75.3734324	73.9289015	84.421441	75.1725398	68.928496	83.9311067
76.3734324	73.9289015	84.421441	75.1725398	68.928496	83.9311067
78.3734324	73.9289015	84.421441	75.1725398	68.928496	83.9311067
78.3734324	76.9289015	84.421441	74.1725398	68.928496	83.9311067

80.3734324	80.9289015	87.421441	74.1725398	69.928496	81.9311067
80.3734324	80.9289015	76.5258374	74.1725398	69.928496	81.9311067
81.3734324	81.9289015	76.4258374	73.1725398	69.928496	76.9311067
81.3734324	81.9289015	76.4258374	73.1725398	69.928496	75.9311067
83.3734324	81.9289015	77.0258374	76.1725398	69.928496	73.9311067
82.3734324	81.9289015	76.9258374	79.1725398	69.928496	71.9311067
82.3734324	81.9289015	77.5258374	79.1725398	75.928496	70.9311067
81.3734324	81.9289015	77.8258374	80.1725398	78.928496	69.9311067
79.3734324	81.9289015	77.6258374	79.1725398	78.928496	69.9311067
78.3734324	83.9289015	78.1258374	79.1725398	82.928496	69.9311067
77.3734324	78.9289015	77.7258374	79.1725398	82.928496	69.9311067
77.3734324	76.9289015	76.9258374	78.1725398	79.928496	70.9311067
76.3734324	74.9289015	76.0258374	78.1725398	73.928496	71.9311067
76.3734324	74.9289015	75.8258374	78.1725398	72.928496	71.9311067
76.3734324	74.9289015	76.0258374	82.1725398	71.928496	73.9311067
77.3734324	73.9289015	88.421441	82.1725398	71.928496	75.9311067
77.3734324	73.9289015	88.421441	81.1725398	71.928496	75.9311067
75.3734324	72.9289015	88.421441	81.1725398	73.928496	77.9311067
73.3734324	72.9289015	87.421441	79.1725398	75.928496	77.9311067
73.3734324	70.9289015	87.421441	77.1725398	77.928496	77.9311067
72.3734324	69.9289015	74.5258374	75.1725398	78.928496	77.9311067
71.3734324	69.9289015	74.4258374	74.1725398	75.928496	79.9311067
71.3734324	69.9289015	74.2258374	73.1725398	75.928496	76.9311067
71.3734324	69.9289015	74.2258374	73.1725398	73.928496	76.9311067
70.3734324	69.9289015	74.1258374	73.1725398	73.928496	75.9311067
66.3734324	70.9289015	74.0258374	73.1725398	72.928496	73.9311067
66.3734324	73.9289015	74.4258374	73.1725398	72.928496	73.9311067
66.3734324	74.9289015	74.8258374	73.1725398	73.928496	73.9311067
66.3734324	74.9289015	75.2258374	74.1725398	73.928496	73.9311067
67.3734324	74.9289015	75.8258374	77.1725398	74.928496	73.9311067
66.3734324	73.9289015	76.4258374	79.1725398	74.928496	73.9311067
66.3734324	72.9289015	76.8258374	81.1725398	74.928496	73.9311067
66.3734324	72.9289015	76.8258374	81.1725398	75.928496	72.9311067
66.3734324	72.9289015	76.6258374	82.1725398	75.928496	72.9311067

Behavior		Activity	
	average of all		moving average
	78.1395516	63	72.3738001
	77.9173294	63	72.5700121
	77.2506627	63	72.7517797
	77.0284405	63	72.9046584
	76.6951071	63	73.0697593
	76.583996	63	73.2318047
	76.2506627	63	73.3963501
	76.1395516	63	73.5395065
	76.2506627	63	73.6671075
	76.3617738	63	73.7733195
	75.6951071	63	73.9073093
	75.583996	63	74.0784619
	75.583996	63	74.23017
	75.1428504	63	74.3568781
	75.3428504	63	74.4974751
	75.4428504	63	74.6130721
	75.4428504	63	74.7008913
	75.8428504	63	74.6155389
	74.7149913	63	74.5346786
	73.9449913	63	74.4733497
	73.9449913	63	74.4225872
	74.4249913	63	74.2901697
	73.3849913	63	74.1558077
	73.8249913	63	74.0431124
	73.7149913	63	73.9259727
	73.8049913	63	73.8071663
	73.0349913	63	73.6911376
	72.4749913	63	73.5834423
	71.7049913	63	73.4785248
	71.7049913	63	73.3808295
	73.0428504	63	73.2703564
	72.3428504	63	73.17905
	72.4428504	63	73.0830213
	72.9428504	63	72.9794927
	73.0428504	63	72.8819927
	72.0428504	63	72.7769927
	71.5428504	63	72.6669927
	71.5428504	63	72.5619927
	72.1428504	63	72.4394927
	72.4428504	63	72.3551892
	72.8428504	63	72.2976356
	72.5428504	63	72.2450821

72.7428504	63	72.1855286
72.3428504	63	72.1469751
71.9428504	63	72.0999215
71.9428504	63	72.045618
71.9428504	63	71.9790645
71.9428504	63	71.8848145
72.3428504	63	71.8130645
71.9428504	63	71.7578145
72.0428504	63	71.711261
71.7428504	63	71.633761
71.4428504	63	71.573761
71.2428504	63	71.516261
71.1428504	63	71.448761
71.0428504	63	71.378761
71.2428504	63	71.321261
70.9428504	63	71.273761
71.3428504	63	71.221261
71.6428504	63	71.146261
71.8428504	63	71.066261
72.0428504	63	70.978761
71.8428504	63	70.903761
71.9428504	63	70.836261
71.5428504	63	70.786261
71.1428504	63	70.748761
69.2649913	63	70.713761
69.6049913	63	70.676261
69.4949913	63	70.633761
69.8428504	63	70.588761
69.9428504	63	70.551261
69.9428504	63	70.511261
70.1428504	63	70.478761
70.2428504	63	70.463761
70.2428504	63	70.448761
69.7428504	63	70.456261
69.6428504	63	70.458761
69.4428504	63	70.446261
69.1428504	63	70.436261
69.2428504	63	70.411261
69.3428504	63	70.373761
69.5428504	63	70.331261
70.0428504	63	70.291261
70.3428504	63	70.253761
70.4428504	63	70.216261
70.5428504	63	70.193761
70.4428504	63	70.201261

70.2428504	63	70.2757074
70.5428504	63	70.3366539
70.4428504	63	70.4053504
70.4428504	63	70.4528504
70.4428504	63	70.4953504
70.8428504	63	70.5328504
70.6428504	63	70.5778504
71.4428504	63	70.6178504
71.1428504	63	70.6628504
70.7428504	63	70.7303504
70.5428504	63	70.8053504
70.3428504	63	70.8978504
70.1428504	63	70.9953504
70.1428504	63	71.0853504
70.4428504	63	71.1553504
70.3428504	63	71.1928504
70.4428504	63	71.2003504
70.6428504	63	71.1978504
71.4428504	63	71.2103504
72.2428504	63	71.2128504
72.0428504	63	71.2153504
72.2428504	63	71.2153504
71.7428504	63	71.2078504
71.6428504	63	71.1828504
71.4428504	63	71.1528504
71.9428504	63	71.1228504
71.8428504	63	71.0903504
72.0428504	63	71.0853504
72.4428504	63	71.0753504
72.6428504	63	71.0953504
73.1428504	63	71.1578504
73.0428504	63	71.2278504
72.8428504	63	71.3028504
72.1428504	63	71.3803504
71.0428504	63	71.4653504
70.3428504	63	71.5403504
70.2428504	63	71.6178504
70.9428504	63	71.6853504
70.6428504	63	71.7403504
70.5428504	63	71.7728504
70.2428504	63	71.7778504
70.2428504	63	71.8153504
69.4428504	63	71.8503504
69.2428504	71	71.9078504
69.2428504	71	71.9728504

69.5428504	71	72.0478504
70.4428504	71	72.0878504
71.0428504	71	72.1178504
71.9428504	71	72.1328504
73.2428504	71	72.1303504
73.3428504	71	72.0928504
73.3428504	71	72.0153504
73.2428504	71	71.9403504
73.5428504	71	71.8678504
73.4428504	71	71.8053504
73.4428504	71	71.7853504
73.1428504	71	71.7703504
72.8428504	71	71.7553504
72.7428504	71	71.7153504
72.4428504	71	71.6728504
73.5428504	71	71.6378504
73.6428504	71	71.6253504
74.0428504	71	71.6153504
74.2428504	71	71.6253504
74.4428504	71	71.6278504
73.5428504	71	71.6153504
73.0428504	71	71.5953504
72.6428504	71	71.5528504
72.3428504	71	71.5253504
71.1428504	71	71.4828504
70.0428504	71	71.4303504
70.0428504	71	71.3878504
69.9428504	71	71.3578504
69.6428504	71	71.3278504
70.2428504	71	71.2903504
69.7428504	71	71.2778504
69.6428504	71	71.2653504
69.3428504	71	71.2628504
68.9428504	71	71.2653504
69.1428504	71	71.2553504
69.7428504	71	71.2578504
69.8428504	71	71.2353504
69.8428504	63	71.2028504
69.3428504	63	71.1241587
68.7428504	63	71.046467
68.7428504	63	70.9667753
68.7428504	63	70.9235836
69.9428504	63	70.9121419
70.2428504	63	70.9162002
71.1428504	63	70.9562002

71.6428504	63	71.0112002
72.1428504	63	71.0887002
72.0428504	63	71.1587002
72.0428504	63	71.2287002
72.9428504	63	71.3012002
72.9428504	63	71.3537002
73.0428504	63	71.4162002
72.9428504	63	71.4762002
72.3428504	63	71.5362002
72.5428504	63	71.5937002
72.6428504	63	71.6412002
72.3428504	63	71.6762002
70.8951823	63	71.7337002
71.1351823	63	71.8062002
71.2551823	63	71.9012002
71.8151823	63	72.0187002
72.5851823	63	72.1337002
72.8051823	63	72.2387002
73.9428504	63	72.3012002
73.3428504	63	72.3262002
73.1428504	63	72.3187002
72.8428504	63	72.2887002
72.7428504	63	72.2437002
72.5428504	63	72.1962002
72.3428504	63	72.1412002
72.2428504	63	72.0637002
72.0428504	63	71.9862002
71.7428504	63	71.8962002
71.2428504	63	71.8137002
71.0428504	63	71.7487002
71.1428504	63	71.6787002
72.1428504	63	71.6162002
72.7428504	63	71.5587002
73.1428504	63	71.5273919
73.4428504	63	71.5050836
73.3428504	63	71.4897753
72.9428504	63	71.472967
72.4428504	63	71.4444087
71.2428504	63	71.4128504
70.8428504	63	71.3653504
70.4428504	63	71.3328504
70.3428504	63	71.2978504
70.1428504	63	71.2603504
69.8428504	63	71.2467529
69.8428504	63	71.2480728

69.8428504	63	71.2616241
69.4428504	63	71.2896589
69.6428504	63	71.317735
69.7428504	63	71.3795265
69.7428504	63	71.4515452
70.1428504	63	71.5172003
70.0428504	63	71.5712646
69.6428504	63	71.5980561
70.2428504	63	71.6075748
70.6428504	63	71.611639
71.1428504	63	71.6286578
71.4428504	63	71.652722
71.5428504	63	71.6867862
72.0428504	63	71.7265323
72.0428504	63	71.773551
71.7428504	63	71.8101153
71.3428504	63	71.8362249
72.1989517	63	71.8557437
72.5956459	63	71.8848079
72.8849021	63	71.9100085
73.3642409	63	71.9533909
73.1658938	63	71.9899551
74.2145101	63	72.0297012
74.123601	63	72.0644472
73.6690555	63	72.0921478
73.3054192	63	72.1198484
73.2145101	63	72.1330035
73.123601	63	72.1441132
73.3054192	63	72.147041
74.123601	63	72.1304234
74.3054192	63	72.1060785
74.3054192	63	72.0669609
74.0326919	63	72.0248888
73.123601	63	71.9825894
72.3054192	63	71.9300627
71.4872373	63	71.8707178
71.123601	63	71.8256911
71.3054192	63	71.786119
70.8508737	63	71.7069625
71.5781464	63	71.5951614
71.3054192	63	71.4715834
71.0326919	63	71.3337492
71.0326919	63	71.2076919
70.8508737	63	71.0690555
70.8508737	63	70.948601

70.6690555	63	70.8554192
70.4872373	63	70.7781464
69.7599646	63	70.7099646
69.5781464	63	70.6576919
69.6690555	63	70.6008737
69.5781464	63	70.5304192
69.7599646	63	70.4645101
69.8508737	63	70.3895101
69.9417828	63	70.3099646
69.6690555	63	70.2554192
69.9417828	63	70.2417828
69.7599646	63	70.2622373
69.0326919	63	70.2917828
68.123601	63	70.3190555
67.9417828	63	70.3508737
67.8508737	63	70.3599646
68.123601	63	70.3667828
68.6690555	63	70.3849646
69.3054192	63	70.4008737
69.9417828	63	70.4326919
70.2145101	63	70.4826919
70.4872373	63	70.5508737
71.0326919	63	70.6326919
71.0326919	63	70.7440555
71.3054192	63	70.8667828
71.6690555	63	70.9690555
71.3054192	63	71.0645101
70.8508737	63	71.1554192
70.9417828	63	71.2417828
71.7599646	63	71.3304192
72.3054192	63	71.4258737
72.3054192	63	71.5190555
72.3963282	63	71.6099646
72.123601	63	71.7395101
71.9417828	63	71.9031464
71.5781464	63	72.0623869
71.7599646	63	72.2222472
71.6690555	63	72.3806612
72.123601	63	72.5254388
72.8508737	63	72.6545139
73.3963282	63	72.7786304
73.7599646	63	72.8992345
74.2145101	63	73.0144667
74.4872373	63	73.1158559
73.7599646	63	73.1947244

73.3963282	63	73.2543781
73.3963282	63	73.3049409
73.3054192	63	73.3596359
73.4872373	63	73.4033804
73.4872373	63	73.425844
73.6690555	63	73.4030597
73.3963282	63	73.366639
74.2145101	63	73.3477802
74.6690555	63	73.3328471
74.3114006	63	73.3222529
74.2452849	63	73.3336165
74.460161	63	73.3586165
74.460161	63	73.3767983
74.4684254	63	73.3972529
74.906442	63	73.3927074
75.0386734	63	73.3677074
75.0965246	63	73.3290711
75.0882601	63	73.2927074
74.1874337	63	73.238162
73.6915659	63	73.1699801
73.6915659	63	73.0995256
73.4932188	63	73.0199801
72.6006568	63	72.9290711
71.8403263	63	72.8427074
70.8485907	63	72.7586165
70.8485907	63	72.6767983
71.5510701	63	72.6017983
71.7990039	63	72.5472529
71.6998304	63	72.4836165
72.3963282	63	72.4177074
72.5781464	63	72.3925579
72.4872373	63	72.3849703
72.4872373	63	72.3856472
71.9417828	63	72.3726878
71.8508737	63	72.3663399
71.8508737	63	72.3467689
72.3054192	63	72.3193466
72.0326919	63	72.2950235
71.7599646	63	72.2845434
70.9417828	63	72.2988567
70.2145101	63	72.3096576
69.7599646	63	72.3204584
69.8508737	63	72.3384907
70.123601	63	72.3674734
70.2145101	63	72.4154644

70.6690555	63	72.4700669
71.2145101	63	72.5246694
71.6690555	63	72.5435281
72.0326919	63	72.560734
73.3054192	63	72.5781464
73.9417828	63	72.5758737
74.4872373	63	72.5758737
73.9417828	63	72.5940555
74.2145101	63	72.5963282
74.123601	63	72.6258737
73.9417828	63	72.6667828
74.123601	63	72.6963282
74.6690555	63	72.7054192
74.7599646	63	72.7258737
74.123601	63	72.7223042
74.123601	63	72.73671
74.2145101	63	72.8026191
73.7599646	63	72.8685281
73.7599646	63	72.9276191
73.0326919	63	72.9776191
73.0326919	63	73.0253463
72.3054192	63	73.0662554
72.4872373	63	73.0935281
72.3963282	63	73.1139827
72.3054192	63	73.1458009
72.5781464	63	73.1548918
73.2145101	63	73.1526191
72.5781464	63	73.1230736
73.123601	63	73.0889827
73.4872373	63	73.0503463
73.0326919	63	72.9764087
72.6690555	63	72.965045
72.8508737	63	72.9445905
71.6171858	63	72.9218632
71.5180122	63	72.8945905
72.8508737	63	72.8923178
72.3963282	63	72.8856491
72.2145101	63	72.8729887
72.123601	63	72.864254
72.123601	63	72.8565523
72.3054192	63	72.8527762
72.3054192	63	72.8715208
72.4872373	63	72.9059678
73.3054192	63	72.9381421
73.6690555	63	72.9601925

73.8508737	63	72.9553834
73.3054192	63	72.9124374
72.5781464	63	72.8436651
72.6690555	63	72.7957605
71.1660943	63	72.766215
73.4872373	63	72.7207605
73.3054192	63	72.6821241
73.7599646	63	72.6571241
73.6690555	63	72.6275786
74.0326919	63	72.6220572
73.8568552	63	72.6258333
73.7080949	63	72.6099242
73.4105742	63	72.6258333
73.4518965	63	72.6258333
72.8816486	63	72.6303787
73.782475	63	72.6090285
73.6833015	63	72.565984
73.7742106	63	72.5130221
73.2783428	63	72.4629528
72.1130535	63	72.3973877
70.8603092	63	72.3218187
70.463615	63	72.2297208
70.6619621	63	72.1413418
71.9417828	63	72.0589546
71.6690555	63	71.9771873
71.4872373	63	71.9381592
71.6690555	63	71.8485407
71.6690555	63	71.7857816
71.3963282	63	71.7188902
71.6690555	63	71.6615031
72.2145101	63	71.5999836
73.0326919	63	71.5428601
72.2145101	63	71.474373
72.3054192	63	71.4205553
71.26959	63	71.3941661
70.5836396	63	71.391124
70.1869454	63	71.3655612
70.484466	63	71.3470232
70.6828131	63	71.3262125
70.6462952	63	71.3518895
70.1669564	63	71.4180622
69.7702622	63	71.5178263
69.2826589	63	71.634326
69.3983613	63	71.7390488
69.6049729	63	71.8231397

69.9024936	63	71.9072306
70.7950555	63	71.9822306
71.0843117	63	72.0481397
71.3735679	63	72.1299579
71.5719151	63	72.1839343
71.5719151	63	72.2385306
70.9686093	63	72.2796971
71.2578655	63	72.2954504
72.3963282	63	72.3242203
72.7599646	63	72.3581554
72.7599646	63	72.4204656
72.9417828	63	72.5248737
72.9417828	63	72.632381
74.3054192	63	72.7324503
74.7599646	63	72.8548336
74.8508737	63	72.9826754
75.123601	63	73.1293188
74.8508737	63	73.2767886
75.3054192	63	73.4341758
75.0326919	63	73.5613977
74.4872373	63	73.6720907
74.3054192	63	73.7708002
74.9417828	63	73.8426502
73.5553861	63	73.8822687
73.8529068	63	73.9032923
73.8611712	63	73.9102663
73.6628241	63	73.919513
73.3653035	63	73.946115
73.6628241	63	73.9723039
73.7619977	63	73.9768493
74.7599646	63	73.963213
74.4872373	63	73.9586675
74.4872373	63	73.9404857
75.5781464	63	73.9223039
75.7599646	63	73.8700311
76.0326919	63	73.8268493
75.6690555	63	73.7813948
75.5781464	63	73.7313948
74.4872373	63	73.6927584
74.0326919	63	73.6359402
73.8508737	63	73.5859402
73.6690555	63	73.5473039
72.6690555	63	73.5109402
72.2145101	63	73.4723039
71.8508737	63	73.4751456

71.9417828	63	73.4682766
72.0326919	63	73.4680192
72.3054192	63	73.4568113
72.5781464	63	73.4530415
72.2145101	63	73.45547
72.5781464	63	73.4576919
72.2145101	63	73.4349646
72.2145101	63	73.4031464
72.2145101	63	73.3690555
73.0326919	63	73.3145101
73.0326919	63	73.2622373
73.123601	63	73.198601
73.3054192	63	73.1349646
73.0326919	63	73.0713282
73.0326919	63	73.0281464
72.9417828	63	72.9804192
72.8508737	63	72.9417828
73.3963282	63	72.9145101
73.6690555	63	72.9145101
73.5781464	63	72.9417828
73.8508737	63	72.9599646
73.2145101	63	72.9804192
73.2145101	63	73.0054192
73.7599646	63	73.0326919
73.8508737	63	73.0440555
73.8508737	63	73.0576919
73.2145101	63	73.0397409
73.123601	63	73.0533773
73.3963282	63	73.0579228
73.6690555	63	73.0601955
73.4872373	63	73.0374682
73.123601	63	72.9851955
73.0326919	63	72.9192864
72.7599646	63	72.8283773
72.123601	63	72.7442864
72.3054192	63	72.6647409
72.5781464	63	72.5783773
72.6690555	63	72.4920137
73.3054192	63	72.3965591
72.5781464	63	72.3033773
72.7599646	63	72.2170137
73.0326919	63	72.1329228
73.3963282	63	72.0692864
73.0326919	63	72.0192864
72.7599646	63	71.95565

71.8601087	63	71.8851955
72.7599646	63	71.8374682
72.3963282	63	71.8101955
72.3054192	63	71.8101955
72.123601	63	71.8033773
70.9417828	63	71.8011288
70.4872373	63	71.8135498
69.6690555	63	71.8400203
69.6690555	63	71.8811603
69.8508737	63	71.9342837
69.4872373	63	71.9909196
69.3963282	63	72.0454893
69.5781464	63	72.0734061
69.9417828	63	72.088513
70.123601	63	72.0901902
70.4872373	63	72.1100492
70.6690555	63	72.1276354
71.2145101	63	72.1571809
71.2145101	63	72.1390233
71.0326919	63	72.132436
71.9417828	63	72.1324603
72.123601	63	72.193593
73.123601	63	72.2345021
73.123601	63	72.2822294
73.579117	63	72.2836999
73.9840757	63	72.2921953
74.1824228	63	72.3277567
74.6782905	63	72.3746818
74.8849021	63	72.4159713
74.3890343	63	72.4599468
74.4882079	63	72.5229876
73.6948195	63	72.5953259
73.2733319	63	72.6798543
73.3725054	63	72.7796719
73.3725054	63	72.8879607
73.4634145	63	73.0220516
74.2145101	63	73.151597
72.6700261	63	73.2834152
72.7691996	63	73.3696015
72.7609352	63	73.4363663
74.3054192	63	73.500032
74.3963282	63	73.5380778
74.3054192	63	73.5567021
72.3642409	63	73.5862475
72.4634145	63	73.6339748

72.3642409	63	73.6725869
72.3642409	63	73.7056205
71.3206357	63	73.7405136
71.4280737	63	73.7516463
72.3725054	63	73.7485229
72.3807699	63	73.7532507
72.7774641	63	73.7532264
73.5708525	63	73.7323637
74.2733319	63	73.7170794
75.4872373	63	73.6918778
75.6690555	63	73.6493209
75.9417828	63	73.5972598
74.6619621	63	73.4870105
73.8851026	63	73.4153733
73.5793175	63	73.345337
73.463615	63	73.3021309
72.8685737	63	73.1975855
74.3054192	63	73.0703128
75.0326919	63	72.9271309
75.123601	63	72.8347513
75.3054192	63	72.7444378
75.5781464	63	72.6566036
75.123601	63	72.5801331
74.7599646	63	72.5388436
74.5781464	63	72.4880499
74.4872373	63	72.4136455
72.8603092	63	72.3390345
72.6619621	63	72.2476879
72.3644414	63	72.1455975
71.6702265	63	72.0304906
71.3809703	63	71.885036
69.8045394	63	71.7373088
69.8045394	63	71.5895815
69.9677472	63	71.4761225
71.0326919	63	71.3911758
70.123601	63	71.3116011
69.3054192	63	71.2462826
68.5781464	63	71.1912946
68.6690555	63	71.104931
68.8508737	63	71.004931
68.8508737	63	70.9094764
69.3054192	63	70.8094764
69.6690555	63	70.6867492
69.3963282	63	70.5822037
69.3963282	63	70.4844764

69.3963282	63	70.3958401
69.123601	63	70.3094764
69.4872373	63	70.2706042
69.6690555	63	70.2230543
69.6690555	63	70.1715787
69.7599646	63	70.1260949
70.0326919	63	70.0878425
70.123601	63	70.0935463
70.4872373	63	70.1219774
70.3963282	63	70.1576919
70.8508737	63	70.1781464
70.6690555	63	70.2167828
70.8508737	63	70.2758737
71.0326919	63	70.3508737
71.3054192	63	70.423601
71.3054192	63	70.4758737
70.6690555	63	70.5417828
70.9417828	63	70.6031464
70.8508737	63	70.6463282
71.0326919	63	70.6917828
71.0326919	63	70.7349646
71.3054192	63	70.7804192
70.7599646	63	70.8326919
70.3054192	63	70.8645101
69.8508737	63	70.8758737
69.8508737	63	70.8872373
70.0326919	63	70.9008737
70.9417828	63	70.9190555
71.3963282	63	70.948601
71.8508737	63	70.9781464
71.6690555	63	71.0213282
71.6690555	63	71.0554192
71.5781464	63	71.0940555
71.5781464	63	71.1190555
70.9417828	63	71.1463282
71.4872373	63	71.1849646
71.7599646	63	71.2372373
71.3963282	63	71.3145101
71.2145101	63	71.3740662
71.123601	63	71.4385811
71.2145101	63	71.5010298
71.2145101	63	71.5636851
70.7599646	63	71.6174562
70.123601	63	71.6774256
70.123601	63	71.7516512

70.3054192	63	71.8446784
70.7599646	63	71.9331602
71.3054192	63	72.0146172
71.6690555	63	72.0688015
72.123601	63	72.1093494
72.2145101	63	72.1385336
72.2145101	63	72.1747427
71.8508737	63	72.2136377
72.123601	63	72.2597641
72.8508737	63	72.3036178
73.3963282	63	72.3633806
73.7599646	63	72.3971103
73.3240295	63	72.4112119
73.4314675	63	72.4120904
73.5306411	63	72.4123491
73.5389056	63	72.4345085
73.4562609	63	72.4568746
73.1587403	63	72.4941168
73.2744427	63	72.5501605
73.5719634	63	72.612196
73.3901452	63	72.6742315
73.2909717	63	72.7441183
73.1091535	63	72.8076001
73.0182444	63	72.8795529
73.0182444	63	72.9473736
73.117418	63	73.0197397
73.224856	63	73.1151942
73.4232031	63	73.1992851
73.332294	63	73.2992851
73.332294	63	73.3970124
72.8364262	63	73.4720124
72.3240295	63	73.533376
71.4314675	63	73.5879215
71.224856	63	73.6829108
72.0099799	63	73.7683959
72.1091535	63	73.8377654
72.7041948	63	73.8955646
73.0017155	63	73.9599753
72.6050213	63	74.0136423
72.6050213	63	74.0598713
73.100889	63	74.1009349
73.2992361	63	74.1533622
74.1835337	63	74.2037234
74.3818808	63	74.2631755
75.0182444	63	74.3088367

76.0326919	63	74.3619358
75.5781464	63	74.4199937
75.8508737	63	74.485283
76.0326919	63	74.5480929
75.8508737	63	74.6255722
75.8508737	63	74.6832168
75.9417828	63	74.7555309
77.123601	63	74.8332168
76.8508737	63	74.9550656
76.3054192	63	75.0593524
75.8508737	63	75.1303748
76.0326919	63	75.161757
75.3054192	63	75.1733045
75.123601	63	75.1578101
75.2145101	63	75.1495471
75.4872373	63	75.1416974
75.3054192	63	75.1241369
75.4872373	63	75.1082293
74.8446907	63	75.0710408
75.1422113	63	75.0386042
75.439732	63	74.9848867
75.8364262	63	74.9062213
75.9355998	63	74.8327211
76.4314675	63	74.7546755
75.6380791	63	74.6735307
75.7289882	63	74.6173566
75.4314675	63	74.5636618
76.3054192	63	74.5027357
75.3963282	63	74.4194955
74.8508737	63	74.3279908
73.3644414	63	74.2575605
73.1660943	63	74.1858906
72.3819409	63	74.1071958
72.2745029	63	74.0371788
72.2910318	63	73.9617898
72.3984698	63	73.8890868
72.6629326	63	73.8048136
72.6959905	63	73.7275651
73.0844202	63	73.6482505
72.8695442	63	73.6097687
72.8860731	63	73.5638489
72.6381392	63	73.4900366
72.7290483	63	73.413125
72.7868996	63	73.3382796
73.6039104	63	73.2555829

73.7030839	63	73.1881755
73.5047368	63	73.1184954
73.793993	63	73.058526
73.1906872	63	72.9789805
73.4882079	63	72.9130714
72.9840757	63	72.8607987
72.8849021	63	72.8388686
72.5047368	63	72.8196245
72.1080426	63	72.8268023
72.3063897	63	72.8343934
72.1163071	63	72.8529349
72.2154806	63	72.8756087
72.3146542	63	72.8893981
73.3054192	63	72.9091793
73.3054192	63	72.9351588
72.4872373	63	72.9574193
72.7599646	63	72.9769938
72.9417828	63	73.000494
73.123601	63	73.0194488
72.9417828	63	73.0392299
72.9417828	63	73.0408586
73.0326919	63	73.0422806
73.123601	63	73.0441158
72.7599646	63	73.0455378
72.7599646	63	73.057497
72.4872373	63	73.0597455
72.3963282	63	73.07687
72.6690555	63	73.0964738
72.5781464	63	73.1210363
73.0326919	63	73.1600617
73.3054192	63	73.2123101
73.2145101	63	73.2670379
73.4872373	63	73.3215591
74.123601	63	73.3690555
73.7599646	63	73.3895101
73.6690555	63	73.4008737
73.5781464	63	73.4008737
73.4872373	63	73.3781464
73.5781464	63	73.3417828
73.6690555	63	73.298601
73.7599646	63	73.2758737
73.5781464	63	73.2713282
73.8508737	63	73.273601
73.6690555	63	73.2599646
73.5781464	63	73.2531464

73.6690555	63	73.2372373
73.6690555	63	73.2258737
73.4872373	63	73.2167828
73.6690555	63	73.2076919
74.3963282	63	73.2008737
74.3054192	63	73.1826919
74.3963282	63	73.1622373
74.2145101	63	73.1463282
74.123601	63	73.1326919
73.7599646	63	73.1122373
72.4872373	63	73.1076919
71.8508737	63	73.1167828
71.4872373	63	73.1304192
71.3963282	63	73.148601
72.0326919	63	73.1622373
72.7599646	63	73.1713282
73.123601	63	73.1826919
72.5781464	63	73.2190555
72.4872373	63	73.2667828
72.123601	63	73.3145101
72.0326919	63	73.3690555
72.0326919	63	73.423601
72.3054192	63	73.4826919
72.3054192	63	73.5139423
72.3054192	63	73.5433331
72.4872373	63	73.5545422
72.5781464	63	73.568024
72.9417828	63	73.5769603
73.3054192	63	73.5681281
73.5781464	63	73.5741718
74.0326919	63	73.5868272
74.123601	63	73.6459701
74.2145101	63	73.7133775
74.123601	63	73.7873965
74.0326919	63	73.8758783
74.2145101	63	73.9534097
75.0326919	63	74.0125526
75.7599646	63	74.050208
75.5781464	63	74.093855
75.7599646	63	74.1375021
75.8508737	63	74.160488
76.0326919	63	74.1683913
74.7372527	63	74.1614186
74.8446907	63	74.1682367
74.8446907	63	74.1591458

74.8446907	63	74.1432367
74.7537816	63	74.1227822
73.8612196	63	74.1023276
74.3653518	63	74.0750549
74.2661783	63	74.0386913
74.8529551	63	73.990964
74.54717	63	73.9296004
74.4479965	63	73.8636913
74.9355998	63	73.7977822
75.1339469	63	73.7432367
75.1256824	63	73.6864186
74.6298146	63	73.6136913
74.3240295	63	73.5250549
74.2331204	63	73.4182367
73.0430378	63	73.3046004
72.3488229	63	73.1886913
71.7537816	63	73.0750549
72.5781464	63	72.965964
71.9417828	63	72.89835
71.6690555	63	72.8325955
71.6690555	63	72.7713864
71.7599646	63	72.7147228
71.8508737	63	72.6739683
71.8508737	63	72.6555278
71.6690555	63	72.6313022
71.5781464	63	72.6186469
71.4872373	63	72.6163221
71.5781464	63	72.6352784
71.9417828	63	72.6612594
71.7599646	63	72.661414
71.3054192	63	72.663428
71.4872373	63	72.6747396
71.4872373	63	72.6870843
71.0326919	63	72.6979827
71.123601	63	72.7043356
71.3054192	63	72.744986
71.6690555	63	72.8029919
72.0326919	63	72.873601
72.2145101	63	72.923601
72.3963282	63	72.9917828
72.5781464	63	73.0713282
73.123601	63	73.1531464
73.123601	63	73.2326919
73.3963282	63	73.298601
73.7599646	63	73.3508737

	74.7599646	63	73.3549489
	75.3054192	63	73.3540653
	75.4872373	63	73.3556612
	74.9417828	63	73.3566371
	75.2145101	63	73.3485222
	75.5781464	63	73.3780677
	75.123601	63	73.4280677
	74.7599646	63	73.4826131
	74.4872373	63	73.5462495
	74.6690555	63	73.6212495
	74.6690555	63	73.7030677
	74.5781464	63	73.7848858
	74.5781464	63	73.8530677
	74.6690555	63	73.8903098
	74.8508737	63	73.9180478
	74.9417828	63	73.9412403
	74.9417828	63	73.94997
	74.4872373	63	73.9473361
	73.9417828	63	73.9768816
BEFORE	71.8320618	63	73.9859725
BEFORE	71.5428056	63	73.9905179
BEFORE	71.5510701	63	73.9632452
BEFORE	71.6171858	63	73.9223361
BEFORE	71.6171858	63	73.8791543
BEFORE	72.9417828	63	73.8291543
BEFORE	73.3054192	63	73.7586997
BEFORE	73.6690555	63	73.6655179
BEFORE	74.0326919	63	73.581427
BEFORE	74.0326919	63	73.5086997
BEFORE	74.3963282	63	73.4518816
BEFORE	74.5781464	63	73.3905179
BEFORE	74.3963282	63	73.3132452
BEFORE	73.5223766	63	73.2223361
BEFORE	73.3240295	63	73.1382452
BEFORE	73.3240295	63	73.056427
BEFORE	72.9273353	63	72.9791543
BEFORE	73.0182444	63	72.8996088
BEFORE	74.3054192	63	72.8109725
BEFORE	73.7599646	63	72.731427
BEFORE	73.9417828	63	72.6586997
BEFORE	73.6690555	63	72.6364428
BEFORE	73.6690555	63	72.6191445
BEFORE	73.7599646	63	72.5789123
BEFORE	72.9417828	63	72.5324818
BEFORE	72.3963282	63	72.4815058

BEFORE	71.8508737	63	72.3928694
BEFORE	71.7599646	63	72.3019603
BEFORE	71.8508737	63	72.2224149
BEFORE	72.2145101	63	72.1496876
BEFORE	72.2145101	63	72.0928694
BEFORE	71.5781464	63	72.0496876
BEFORE	70.9417828	63	71.9589689
BEFORE	71.2145101	63	71.8754816
BEFORE	71.3963282	63	71.8556469
BEFORE	71.7599646	63	71.8310601
BEFORE	71.7599646	63	71.796556
BEFORE	71.3963282	63	71.7719692
BEFORE	71.3054192	63	71.7649444
BEFORE	71.0326919	63	71.7207815
BEFORE	70.9417828	63	71.6805443
BEFORE	70.8508737	63	71.6134476
BEFORE	69.9417828	63	71.548417
BEFORE	69.7599646	63	71.480907
BEFORE	69.5781464	63	71.4142235
BEFORE	69.3963282	63	71.3729533
BEFORE	69.6690555	63	71.3508979
BEFORE	70.4872373	63	71.3470243
BEFORE	71.123601	63	71.3427375
BEFORE	71.7599646	63	71.3312193
BEFORE	72.6690555	63	71.3081308
BEFORE	70.9493978	63	71.282563
BEFORE	71.0568358	63	71.267739
BEFORE	72.7289882	63	71.2539481
BEFORE	72.3405584	63	71.2451158
BEFORE	71.9438642	63	71.2342174
BEFORE	71.9438642	63	71.2189802
BEFORE	72.7372527	63	71.203743
BEFORE	72.5389056	63	71.1876793
BEFORE	72.1504758	63	71.1784338
BEFORE	71.2579138	63	71.1710479
BEFORE	71.0678312	63	71.1684139
BEFORE	70.9686576	63	71.1754908
BEFORE	71.0926246	63	71.2077742
BEFORE	71.2909717	63	71.2668651
BEFORE	71.5141122	63	71.2943747
BEFORE	71.6959303	63	71.3241569
BEFORE	71.5884923	63	71.3547656
BEFORE	71.3901452	63	71.3748371
BEFORE	71.2909717	63	71.418019
BEFORE	71.1917981	63	71.4362008

BEFORE	70.9851865	63	71.4225644
BEFORE	70.3901452	63	71.4291922
BEFORE	70.8612196	63	71.4240432
BEFORE	70.9603932	63	71.379363
BEFORE	71.1504758	63	71.3375755
BEFORE	71.1504758	63	71.3102507
BEFORE	70.7537816	63	71.2783805
BEFORE	70.9355998	63	71.2266756
BEFORE	70.7372527	63	71.182202
BEFORE	70.8364262	63	71.1519847
BEFORE	71.1339469	63	71.1486269
BEFORE	71.2331204	63	71.1432029
BEFORE	72.123601	63	71.1357129
BEFORE	70.6785279	63	71.1273964
BEFORE	70.5876188	63	71.1209394
BEFORE	70.8934039	63	71.1111766
BEFORE	71.2900981	63	71.108232
BEFORE	72.8508737	63	71.1057006
BEFORE	72.4872373	63	71.1013098
BEFORE	72.123601	63	71.1062164
BEFORE	71.2145101	63	71.1181478
BEFORE	70.8508737	63	71.1352446
BEFORE	70.9417828	63	71.178581
BEFORE	70.6690555	63	71.2192314
BEFORE	70.8508737	63	71.2414934
BEFORE	70.6690555	63	71.2544579
BEFORE	70.6690555	63	71.2696951
BEFORE	70.7599646	63	71.292577
BEFORE	70.9417828	63	71.3086406
BEFORE	71.123601	63	71.3387539
BEFORE	70.8508737	63	71.3709333
BEFORE	70.6690555	63	71.4024928
BEFORE	70.7599646	63	71.4293003
BEFORE	71.0326919	63	71.4224821
BEFORE	71.123601	63	71.4381544
BEFORE	71.5781464	63	71.4515539
BEFORE	71.4872373	63	71.4573089
BEFORE	71.2145101	63	71.4508737
BEFORE	71.4872373	63	71.4076919
BEFORE	71.6690555	63	71.3713282
BEFORE	71.6690555	63	71.3440555
BEFORE	72.123601	63	71.3281464
BEFORE	72.4872373	63	71.3145101
BEFORE	71.8508737	63	71.2917828
BEFORE	71.6690555	63	71.298601

BEFORE	71.7599646	63	71.3122373
BEFORE	71.6690555	63	71.3326919
BEFORE	71.5781464	63	71.348601
BEFORE	71.9417828	63	71.348601
BEFORE	72.123601	63	71.3463282
BEFORE	72.3963282	63	71.3463282
BEFORE	72.3054192	63	71.3622373
BEFORE	71.8508737	63	71.398601
BEFORE	71.3054192	63	71.4440555
BEFORE	71.123601	63	71.4804192
BEFORE	71.123601	63	71.5145101
BEFORE	71.0326919	63	71.5190857
BEFORE	71.123601	63	71.5690857
BEFORE	71.0326919	63	71.6304494
BEFORE	71.0326919	63	71.6963584
BEFORE	70.5781464	63	71.7736312
BEFORE	70.3054192	63	71.8599948
BEFORE	70.0326919	63	71.9327221
BEFORE	70.9417828	63	71.991813
BEFORE	71.3963282	63	72.066813
BEFORE	71.4872373	63	72.141813
BEFORE	71.3054192	63	72.2031766
BEFORE	70.7599646	63	72.2577221
BEFORE	70.8508737	63	72.3077221
BEFORE	71.123601	63	72.3531766
BEFORE	71.4872373	63	72.3940857
BEFORE	72.123601	63	72.4463584
BEFORE	72.5781464	63	72.516813
BEFORE	72.4872373	63	72.5599948
BEFORE	72.4872373	63	72.6190857
BEFORE	71.7611725	63	72.6940857
BEFORE	73.4872373	63	72.7690857
BEFORE	73.6690555	63	72.8147761
BEFORE	74.123601	63	72.8658385
BEFORE	74.7599646	63	72.9365289
BEFORE	75.123601	63	73.0051532
BEFORE	75.0326919	63	73.0702652
BEFORE	74.8508737	63	73.1446746
BEFORE	74.8508737	63	73.2360262
BEFORE	74.6690555	63	73.3023778
BEFORE	74.2145101	63	73.3749277
BEFORE	73.8508737	63	73.4427256
BEFORE	73.5781464	63	73.5349036
BEFORE	73.7599646	63	73.6382387
BEFORE	73.7599646	63	73.7320696

BEFORE	74.4872373	63	73.8434332
BEFORE	75.123601	63	73.9479787
BEFORE	73.5781464	63	74.0411605
BEFORE	73.6690555	63	74.1184332
BEFORE	74.123601	63	74.1979787
BEFORE	74.123601	63	74.2616151
BEFORE	72.8603092	63	74.3388576
BEFORE	73.1660943	63	74.368403
BEFORE	73.8603092	63	74.3843121
BEFORE	73.7776646	63	74.3911303
BEFORE	73.1826232	63	74.3365849
BEFORE	73.2817968	63	74.2411303
BEFORE	73.6867555	63	74.1502212
BEFORE	73.5958464	63	74.0411303
BEFORE	74.2983257	63	73.9297667
BEFORE	74.1991522	63	73.8229485
BEFORE	74.9925406	63	73.7297667
BEFORE	74.893367	63	73.6456758
BEFORE	74.6041108	63	73.5797667
BEFORE	75.5781464	63	73.518403
BEFORE	75.6690555	63	73.4911303
BEFORE	75.8508737	63	73.452494
BEFORE	75.6690555	63	73.3979485
BEFORE	75.6690555	63	73.3865849
BEFORE	75.0326919	63	73.3638576
BEFORE	74.8508737	63	73.3343121
BEFORE	74.6690555	63	73.3093121
BEFORE	74.3054192	63	73.3249853
BEFORE	74.3963282	63	73.3557412
BEFORE	72.5781464	63	73.3736871
BEFORE	71.3054192	63	73.3846083
BEFORE	71.3963282	63	73.3976667
BEFORE	70.4872373	63	73.4086591
BEFORE	70.3963282	63	73.4219242
BEFORE	70.3963282	63	73.4325034
BEFORE	70.4872373	63	73.4275866
BEFORE	70.4872373	63	73.43486
BEFORE	70.9417828	63	73.414654
BEFORE	71.3054192	63	73.3994067
BEFORE	72.6690555	63	73.4101212
BEFORE	72.9417828	63	73.3942122
BEFORE	72.9417828	63	73.3533031
BEFORE	73.123601	63	73.2919394
BEFORE	72.7599646	63	73.2328485
BEFORE	72.9417828	63	73.1760303

BEFORE	73.123601	63	73.1283031
BEFORE	73.4872373	63	73.0826772
BEFORE	74.3963282	63	73.0440967
BEFORE	74.5781464	63	73.0071072
BEFORE	74.2145101	63	72.9728449
BEFORE	73.7049626	63	72.9940372
BEFORE	73.7214916	63	73.0420476
BEFORE	74.2173593	63	73.0877853
BEFORE	74.0190122	63	73.1612503
BEFORE	74.1016569	63	73.2494881
BEFORE	74.4900866	63	73.3477258
BEFORE	74.1843015	63	73.4411908
BEFORE	74.283475	63	73.5446558
BEFORE	75.0326919	63	73.6367572
BEFORE	74.9417828	63	73.7122676
BEFORE	74.0326919	63	73.7311872
BEFORE	73.3963282	63	73.7307885
BEFORE	73.3054192	63	73.7328899
BEFORE	73.3963282	63	73.7304458
BEFORE	73.123601	63	73.7395926
BEFORE	73.0258374	63	73.741694
BEFORE	73.1258374	63	73.7442499
BEFORE	72.8258374	63	73.7352149
BEFORE	73.0258374	63	73.6934526
BEFORE	73.4258374	63	73.6496449
BEFORE	73.2258374	63	73.6099281
BEFORE	73.2258374	63	73.5979499
BEFORE	73.4258374	63	73.5571375
BEFORE	73.9258374	63	73.5012011
BEFORE	74.3258374	63	73.4888718
BEFORE	74.2258374	63	73.5019763
BEFORE	74.6258374	63	73.50787
BEFORE	74.6258374	63	73.5114084
BEFORE	74.3258374	63	73.5149675
BEFORE	73.4258374	63	73.4847961
Minute	72.9258374	63	73.4518975
Minute	73.0258374	63	73.4417261
Minute	73.0258374	63	73.4179327
Minute	73.1258374	63	73.4045939
Minute	73.0258374	63	73.3867096
Minute	73.2258374	63	73.381098
Minute	73.1258374	63	73.3706578
Minute	72.7258374	63	73.3504449
Minute	72.8258374	63	73.3322774
Minute	72.6258374	63	73.290019

Minute	73.2258374	63	73.2345788
Minute	72.0889947	63	73.2095788
Minute	71.9799038	63	73.1645788
Minute	73.5258374	63	73.1145788
Minute	74.6258374	63	73.0520788
Minute	74.7258374	63	72.9770788
Minute	74.3258374	63	72.9145788
Minute	74.4258374	63	72.8395788
Minute	73.8258374	63	72.7895788
Minute	73.6258374	63	72.7620788
Minute	73.6258374	63	72.7620788
Minute	72.4445924	63	72.7820788
Minute	72.7718652	63	72.8070788
Minute	72.6809561	63	72.8370788
Minute	72.8991379	63	72.8620788
Minute	72.6082288	63	72.8945788
Minute	72.3173197	63	72.9220788
Minute	72.0991379	63	72.9470788
Minute	71.3355015	63	72.9995788
Minute	71.2082288	63	73.0520788
Minute	72.2258374	63	73.1120788
Minute	71.4258374	63	73.1570788
Minute	71.4258374	63	73.2304998
Minute	71.4258374	63	73.3091482
Minute	71.3258374	63	73.3541482
Minute	71.7258374	63	73.3666482
Minute	71.6258374	63	73.3666482
Minute	72.6258374	63	73.3691482
Minute	73.2258374	63	73.3666482
Minute	73.4258374	63	73.3941482
Minute	73.7258374	63	73.4166482
Minute	74.0258374	63	73.4566482
Minute	74.2258374	63	73.5386793
Minute	74.1258374	63	73.6100286
Minute	74.3258374	63	73.6936506
Minute	74.3258374	63	73.7843181
Minute	74.1258374	63	73.8797583
Minute	74.8258374	63	73.9949713
Minute	74.9258374	63	74.1206388
Minute	75.0258374	63	74.2578972
Minute	75.0258374	63	74.3883374
Minute	75.0258374	63	74.4983374
Minute	75.1258374	63	74.6283374
Minute	75.3258374	63	74.7433374
Minute	75.1258374	63	74.821079

Minute	74.7258374	63	74.938579
Minute	74.4258374	63	75.0206388
Minute	74.3258374	63	75.1051985
Minute	74.9258374	63	75.1593038
Minute	74.5258374	63	75.1929545
Activity	75.2258374	73	75.1834234
Activity	75.7258374	73	75.1382104
Activity	75.6258374	73	75.0800429
Activity	76.0258374	73	75.0164209
Activity	76.5258374	73	74.9575716
Activity	76.4258374	73	74.890995
Activity	76.9258374	73	74.8212366
Activity	77.1258374	73	74.7787366
Activity	76.8258374	73	74.7062366
Activity	76.4258374	73	74.6487366
Activity	76.6258374	73	74.5937366
Activity	76.6258374	73	74.5512366
Activity	76.0258374	73	74.5137366
Activity	74.5355015	73	74.4762366
Activity	76.0258374	73	74.4387366
Activity	75.0082288	73	74.4112366
Activity	75.0082288	73	74.4212366
Activity	74.790047	73	74.4412366
Activity	74.5718652	73	74.4612366
Activity	73.0445924	73	74.4412366
Activity	71.9173197	73	74.4187366
Activity	71.6991379	73	74.3837366
Activity	71.6809561	73	74.3387366
Activity	71.7718652	73	74.3062366
Activity	71.6627743	73	74.2687366
Activity	71.5355015	73	74.2312366
Activity	72.4258374	73	74.1962366
Activity	71.9258374	73	74.1337366
Activity	72.6258374	73	74.0737366
Activity	72.8258374	73	74.0362366
Activity	73.3258374	73	73.9987366
Activity	73.5258374	73	73.9537366
Activity	73.6258374	73	73.9012366
Activity	73.8258374	73	73.8562366
Activity	74.0258374	73	73.853495
Activity	75.1258374	73	73.815995
Activity	75.2258374	73	73.8089352
Activity	75.1258374	73	73.7752989
Activity	74.1258374	73	73.751208
Activity	73.6258374	73	73.7157534

Activity	73.8258374	73	73.7016625
Activity	73.9258374	73	73.7048443
Activity	74.3258374	73	73.7102989
Activity	74.5258374	73	73.6934807
Activity	75.0258374	73	73.6689352
Activity	75.0258374	73	73.6466625
Activity	74.4258374	73	73.6302989
Activity	74.7258374	73	73.6025859
Activity	75.3258374	73	73.6150859
Activity	74.9258374	73	73.6025859
Activity	74.8258374	73	73.5800859
Activity	74.5258374	73	73.5275859
Activity	74.2258374	73	73.4750859
Activity	74.4258374	73	73.4275859
Activity	74.5258374	73	73.3800859
Activity	74.7258374	73	73.3250859
Activity	73.6627743	73	73.2500859
Activity	73.8264106	73	73.1797525
Activity	73.1536833	73	73.1173736
Activity	72.4809561	73	73.0849948
Activity	72.0445924	73	73.0623886
Activity	71.9173197	73	73.0320552
Activity	71.0082288	73	73.00604
Activity	70.790047	73	72.9668429
Activity	70.7718652	73	72.9281004
Activity	70.8809561	73	72.8341306
Activity	71.3173197	73	72.7565245
Activity	72.4258374	73	72.6939184
Activity	72.1258374	73	72.6347213
Activity	71.9258374	73	72.5673424
Activity	71.2258374	73	72.5154181
Activity	71.4258374	73	72.4829181
Activity	71.7258374	73	72.4579181
Activity	71.9258374	73	72.4329181
Activity	71.8258374	73	72.4154181
Activity	72.1258374	73	72.3829181
Activity	72.4125008	73	72.3479181
Activity	72.6306826	73	72.3444947
Activity	72.8306826	73	72.3344804
Activity	72.7215917	73	72.3537842
Activity	72.6125008	73	72.3899062
Activity	72.8852281	73	72.4494374
Activity	72.7579553	73	72.5371503
Activity	72.9761371	73	72.6475905
Activity	71.2670462	73	72.7384996

Activity	71.9215917	73	72.8366814
Activity	71.9215917	73	72.9266814
Activity	72.3579553	73	73.0112269
Activity	72.6306826	73	73.0735139
Activity	72.8488644	73	73.1735139
Activity	73.5258374	73	73.2412352
Activity	73.5258374	73	73.3155473
Activity	73.2258374	73	73.4155473
Activity	73.7258374	73	73.5005473
Activity	73.2258374	73	73.5755473
Activity	73.3258374	73	73.6355473
Activity	73.5258374	73	73.6830473
Activity	73.4258374	73	73.7133807
Activity	73.9258374	73	73.7357596
Activity	73.9258374	73	73.7631385
Activity	74.4258374	73	73.7882446
Activity	75.4258374	73	73.826078
Activity	75.4258374	73	73.8570933
Activity	74.4264106	73	73.8887903
Activity	74.6991379	73	73.9050328
Activity	74.4809561	73	73.9615026
Activity	74.6991379	73	74.0016087
Activity	74.9173197	73	74.0417149
Activity	76.1258374	73	74.0709119
Activity	74.6346866	73	74.0882908
Activity	74.198323	73	74.0927151
Activity	75.4258374	73	74.0677151
Activity	75.1258374	73	74.0452151
Activity	74.9258374	73	74.0477151
Activity	74.2258374	73	74.0277151
Activity	74.0258374	73	74.0127151
Activity	73.6258374	73	73.9877151
Activity	73.5258374	73	73.9602151
Activity	73.9258374	73	73.9402151
Activity	73.7258374	73	73.9127151
Activity	74.1258374	73	73.8877151
Activity	74.1258374	73	73.8527151
Activity	74.0258374	73	73.7977151
Activity	73.6258374	73	73.7252151
Activity	73.5258374	73	73.6380492
Activity	73.5258374	73	73.5454287
Activity	73.5258374	73	73.4555355
Activity	73.5258374	73	73.3874605
Activity	73.3258374	73	73.3271128
Activity	73.0258374	73	73.2621128

Activity	72.5258374	73	73.2368915
Activity	72.6258374	73	73.2200794
Activity	73.3258374	73	73.1625794
Activity	72.9258374	73	73.1200794
Activity	72.6258374	73	73.0825794
Activity	72.3258374	73	73.0598664
Activity	72.4258374	73	73.0448808
Activity	72.6258374	73	73.0444406
Activity	72.8258374	73	73.0442276
Activity	72.9258374	73	73.042651
Activity	73.0258374	73	73.0442563
Activity	73.2258374	73	73.0517563
Activity	72.5258374	73	73.0642563
Activity	70.9397735	73	73.0842563
Activity	70.994319	73	73.1092563
Activity	70.8852281	73	73.1342563
Activity	71.9761371	73	73.1617563
Activity	72.5034099	73	73.1792563
Activity	73.5258374	73	73.1867563
Activity	73.6258374	73	73.1892563
Activity	73.5258374	73	73.1917563
Activity	73.1258374	73	73.1942563
Activity	73.4258374	73	73.1892563
Activity	73.4258374	73	73.1542563
Activity	73.3173197	73	73.1417563
Activity	73.4264106	73	73.1292563
Activity	73.6082288	73	73.1092563
Activity	73.5173197	73	73.0767563
Activity	73.8627743	73	73.0342563
Activity	73.790047	73	72.9867563
Activity	74.4258374	73	72.9367563
Activity	74.6258374	73	72.8867563
Activity	74.8258374	73	72.8392563
Activity	74.6258374	73	72.8242563
Activity	74.5258374	73	72.8489079
Activity	74.6258374	73	72.8746958
Activity	74.2258374	73	72.8932111
Activity	73.8258374	73	72.8844536
Activity	73.4258374	73	72.8650143
Activity	73.1258374	73	72.8250143
Activity	72.6258374	73	72.7925143
Activity	72.4258374	73	72.7575143
Activity	71.9258374	73	72.7375143
Activity	72.4258374	73	72.7000143
Activity	72.1258374	73	72.6525143

Activity	71.5258374	73	72.6102272
Activity	71.1258374	73	72.5602129
Activity	70.9258374	73	72.4931531
Activity	70.9258374	73	72.413366
Activity	70.9258374	73	72.3374426
Activity	71.0258374	73	72.2433374
Activity	71.3258374	73	72.1358374
Activity	71.9258374	73	72.0383374
Activity	71.9258374	73	71.9338062
Activity	72.0258374	73	71.8356388
Activity	71.6258374	73	71.7304258
Activity	71.6258374	73	71.6145311
Activity	71.7258374	73	71.5040908
Activity	71.9258374	73	71.4036506
Activity	72.3258374	73	71.3277559
Activity	72.1258374	73	71.2852559
Activity	72.3258374	73	71.2652559
Activity	71.9258374	73	71.2358295
Activity	71.5258374	73	71.2189032
Activity	71.6258374	73	71.2214032
minute	71.4258374	63	71.2339032
minute	70.9258374	63	71.2464032
minute	70.3258374	63	71.2514032
minute	70.8258374	63	71.2589032
minute	70.0258374	63	71.2614032
minute	70.1258374	63	71.2639032
minute	70.7258374	63	71.2439032
minute	70.6445924	63	71.2089032
minute	70.6991379	63	71.1689032
minute	70.3173197	63	71.1389032
minute	69.990047	63	71.1114032
minute	69.8082288	63	71.1014032
minute	69.8082288	63	71.0914032
minute	70.390047	63	71.0689032
minute	71.4258374	63	71.0289032
minute	71.8258374	63	70.9739032
minute	71.2487833	63	70.9239032
minute	71.2487833	63	70.8664032
minute	72.5258374	63	70.8114032
minute	72.6258374	63	70.7814032
minute	72.0258374	63	70.7464032
minute	71.3258374	63	70.7214032
minute	71.2258374	63	70.7139032
minute	71.0258374	63	70.7289032
minute	71.0258374	63	70.7289032

minute	70.2258374	63	70.7564032
minute	69.9258374	63	70.7889032
minute	70.3258374	63	70.8064032
minute	70.7258374	63	70.8259343
minute	70.9258374	63	70.8416018
minute	71.2258374	63	70.8643147
minute	71.2258374	63	70.8902095
minute	70.8258374	63	70.9206497
minute	70.3258374	63	70.9535899
minute	70.1258374	63	70.9744847
minute	70.1258374	63	70.9769847
minute	70.0258374	63	70.9669847
minute	69.7258374	63	70.963911
minute	70.3258374	63	70.9683374
minute	70.2258374	63	70.9333374
minute	70.4258374	63	70.8983374
minute	70.6258374	63	70.8908374
minute	70.9258374	63	70.9008374
minute	70.8258374	63	70.9108374
minute	71.1258374	63	70.9208374
minute	71.4258374	63	70.9133374
minute	71.4258374	63	70.9383374
minute	71.4258374	63	70.9708374
minute	71.3258374	63	71.0083374
minute	71.2258374	63	71.0383374
minute	71.0258374	63	71.0708374
minute	71.0258374	63	71.0933374
minute	71.1258374	63	71.1333374
minute	71.2258374	63	71.2033374
minute	71.5258374	63	71.2958374
minute	71.4258374	63	71.3908374
minute	71.1258374	63	71.4833374
minute	71.4258374	63	71.5833374
minute	71.1258374	63	71.6883374
minute	71.2258374	63	71.7883374
AFTER	71.7258374	63	71.8883374
AFTER	71.7258374	63	71.9758374
AFTER	71.6258374	63	72.0533374
AFTER	71.4258374	63	72.1233374
AFTER	70.7258374	63	72.2208374
AFTER	71.2258374	63	72.3233374
AFTER	71.2258374	63	72.4233374
AFTER	71.8258374	63	72.5183374
AFTER	71.9258374	63	72.6058374
AFTER	72.2258374	63	72.6758374

AFTER	72.1258374	63	72.6833304
AFTER	72.8258374	63	72.6826416
AFTER	73.6258374	63	72.6792256
AFTER	74.0258374	63	72.6967256
AFTER	73.9258374	63	72.7042256
AFTER	73.8258374	63	72.6917256
AFTER	74.0258374	63	72.6742256
AFTER	73.9258374	63	72.6642256
AFTER	74.3258374	63	72.6517256
AFTER	74.2258374	63	72.6467256
AFTER	73.9258374	63	72.6342256
AFTER	73.7258374	63	72.6067256
AFTER	73.7258374	63	72.5592256
AFTER	74.7258374	63	72.5067256
AFTER	75.2258374	63	72.4517256
AFTER	75.4258374	63	72.4142256
AFTER	75.2258374	63	72.3692256
AFTER	74.9258374	63	72.3392256
AFTER	74.1258374	63	72.3042256
AFTER	71.5255588	63	72.2717256
AFTER	70.9982861	63	72.2342256
AFTER	70.8891952	63	72.1892256
AFTER	71.8258374	63	72.1192256
AFTER	71.5258374	63	72.0442256
AFTER	71.0258374	63	71.9592256
AFTER	70.7258374	63	71.8717256
AFTER	70.7258374	63	71.7892256
AFTER	70.9258374	63	71.7117256
AFTER	70.9258374	63	71.6392256
AFTER	70.7258374	63	71.5492256
AFTER	70.6258374	63	71.4642256
AFTER	69.8258374	63	71.3867256
AFTER	69.5258374	63	71.3067256
AFTER	69.2258374	63	71.2292256
AFTER	69.2258374	63	71.1392256
AFTER	69.4258374	63	71.0367256
AFTER	70.0258374	63	70.9392256
AFTER	70.4258374	63	70.8442256
AFTER	70.6258374	63	70.7617256
AFTER	70.7258374	63	70.7042256
AFTER	70.3258374	63	70.7192325
AFTER	70.0258374	63	70.7474213
AFTER	70.6258374	63	70.774689
AFTER	70.6258374	63	70.7949043
AFTER	70.4258374	63	70.7942045

AFTER	70.5258374	63	70.7823683
AFTER	70.9258374	63	70.7698503
AFTER	71.0258374	63	70.7448323
AFTER	70.7258374	63	70.729587
AFTER	70.8258374	63	70.7275235
AFTER	70.8258374	63	70.7327327
AFTER	70.5258374	63	70.7522602
AFTER	70.6258374	63	70.7976967
AFTER	71.1258374	63	70.8790484
AFTER	71.1258374	63	70.9747182
AFTER	71.5258374	63	71.0731153
AFTER	71.4258374	63	71.1528761
AFTER	71.6258374	63	71.2080914
AFTER	71.8258374	63	71.2664885
AFTER	72.1258374	63	71.3221583
AFTER	72.1258374	63	71.4146583
AFTER	71.9799038	63	71.5271583
AFTER	72.6344492	63	71.6596583
AFTER	71.4978439	63	71.7521583
AFTER	70.5523894	63	71.8321583
AFTER	70.2251167	63	71.9121583
AFTER	69.7251167	63	71.9621583
AFTER	70.3160257	63	71.9921583
AFTER	70.8432985	63	72.0321583
AFTER	70.9342076	63	72.0771583
AFTER	71.4069348	63	72.1221583
AFTER	71.6432985	63	72.1796583
AFTER	72.7799038	63	72.2496583
AFTER	73.0526311	63	72.3096583
AFTER	73.161722	63	72.3821583
AFTER	72.6162674	63	72.4346583
AFTER	72.2344492	63	72.4796583
AFTER	72.761722	63	72.5171583
AFTER	72.8526311	63	72.5421583
AFTER	74.4258374	63	72.5621583
AFTER	74.8258374	63	72.5746583
AFTER	75.3258374	63	72.5921583
AFTER	74.3258374	63	72.6033067
AFTER	73.8258374	63	72.5855914
AFTER	73.6258374	63	72.6137912
AFTER	72.5258374	63	72.6656274
AFTER	72.1258374	63	72.7231454
AFTER	72.6258374	63	72.7831634
AFTER	72.5258374	63	72.8359087
AFTER	72.6258374	63	72.8804722

AFTER	73.1258374	63	72.922763
AFTER	73.3258374	63	72.9582355
AFTER	73.0258374	63	72.997799
AFTER	74.0258374	63	73.0089473
AFTER	73.2258374	63	73.0182775
AFTER	73.3258374	63	73.0298804
AFTER	72.9258374	63	73.0476196
AFTER	72.6258374	63	73.0674043
AFTER	72.6258374	63	73.0615072
AFTER	72.6258374	63	73.0483374
AFTER	72.8258374	63	72.9858374
AFTER	72.4258374	63	72.9033374
AFTER	71.9258374	63	72.7983374
AFTER	72.6258374	63	72.7008374
AFTER	72.6258374	63	72.6133374
AFTER	72.5258374	63	72.5383374
AFTER	72.1258374	63	72.4933374
AFTER	72.4258374	63	72.4683374
AFTER	72.6258374	63	72.4358374
AFTER	72.6258374	63	72.4033374
AFTER	72.8258374	63	72.3683374
AFTER	73.2258374	63	72.3233374
AFTER	73.2258374	63	72.2683374
AFTER	73.4258374	63	72.2208374
AFTER	73.6258374	63	72.1508374
AFTER	73.3258374	63	72.0958374
AFTER	73.0258374	63	72.0383374
AFTER	72.5258374	63	71.9858374
AFTER	72.3258374	63	71.9333374
AFTER	71.9258374	63	71.8833374
AFTER	71.5258374	63	71.8383374
AFTER	71.1258374	63	71.7833374
AFTER	70.4258374	63	71.7383374
AFTER	70.3258374	63	71.7008374
AFTER	70.6258374	63	71.6458374
AFTER	70.7258374	63	71.5908374
AFTER	71.1258374	63	71.5558374
AFTER	71.3258374	63	71.5458374
AFTER	71.2258374	63	71.5383374
AFTER	71.2258374	63	71.5508374
AFTER	71.3258374	63	71.5658374
AFTER	71.1258374	63	71.5708374
AFTER	71.1258374	63	71.5658374
AFTER	71.2258374	63	71.5683374
AFTER	71.0258374	63	71.5608374

AFTER	71.0258374	63	71.5508374
AFTER	70.8258374	63	71.5358374
AFTER	70.5258374	63	71.5183374
AFTER	70.6258374	63	71.5208374
AFTER	70.8258374	63	71.5283374
AFTER	70.6258374	63	71.5408374
AFTER	70.6258374	63	71.5483374
AFTER	70.4258374	63	71.5658374
AFTER	70.4258374	63	71.6033374
AFTER	70.4258374	63	71.6433374
AFTER	71.1258374	63	71.6833374
AFTER	71.7258374	63	71.7183374
AFTER	72.1258374	63	71.7433374
AFTER	73.1258374	63	71.7533374
AFTER	73.2258374	63	71.7733374
AFTER	73.0258374	63	71.7908374
AFTER	73.0258374	63	71.8008374
AFTER	73.3258374	63	71.8133374
AFTER	73.1258374	63	71.8358374
AFTER	73.2258374	63	71.8583374
AFTER	72.7258374	63	71.8833374
AFTER	72.3258374	63	71.9008374
AFTER	72.6258374	63	71.9233374
AFTER	72.6258374	63	71.9533374
AFTER	72.4258374	63	71.9733374
AFTER	71.8258374	63	71.9958374
AFTER	71.8258374	63	72.0283374
AFTER	71.9258374	63	72.0633374
AFTER	71.9258374	63	72.1058374
AFTER	72.2258374	63	72.1608374
AFTER	72.1258374	63	72.2108374
AFTER	72.1258374	63	72.2433374
AFTER	71.7258374	63	72.2658374
AFTER	72.0258374	63	72.2783374
AFTER	71.9258374	63	72.2608374
AFTER	71.7258374	63	72.2358374
AFTER	71.6258374	63	72.2208374
AFTER	72.0258374	63	72.1983374
AFTER	72.1258374	63	72.1658374
AFTER	72.0258374	63	72.1333374
AFTER	71.7258374	63	72.1033374
AFTER	71.7258374	63	72.0983374
AFTER	71.7258374	63	72.1108374
AFTER	71.4258374	63	72.1258374
AFTER	71.7258374	63	72.1433374

AFTER	71.9258374	63	72.1658374
AFTER	72.0258374	63	72.2058374
AFTER	72.1258374	63	72.2433374
AFTER	72.6258374	63	72.2733374
AFTER	72.4258374	63	72.3033374
AFTER	72.4258374	63	72.3183374
AFTER	72.6258374	63	72.3258374
AFTER	72.6258374	63	72.3208374
AFTER	72.4258374	63	72.3158374
AFTER	72.2258374	63	72.3008374
AFTER	72.4258374	63	72.2958374
AFTER	72.1258374	63	72.2983374
AFTER	72.0258374	63	72.3058374
AFTER	71.8258374	63	72.3033374
AFTER	72.0258374	63	72.3083374
AFTER	72.5258374	63	72.3208374
AFTER	72.8258374	63	72.3408374
AFTER	73.2258374	63	72.3533374
AFTER	73.3258374	63	72.3608374
AFTER	73.3258374	63	72.3783374
AFTER	73.4258374	63	72.3783374
AFTER	73.3258374	63	72.3883374
AFTER	73.1258374	63	72.3958374
AFTER	73.1258374	63	72.3983374
AFTER	72.8258374	63	72.3858374
AFTER	72.4258374	63	72.3733374
AFTER	71.9258374	63	72.3533374
AFTER	71.5258374	63	72.3233374
AFTER	71.4258374	63	72.2808374
AFTER	71.7258374	63	72.2383374
AFTER	71.8258374	63	72.2058374
AFTER	71.9258374	63	72.1733374
AFTER	71.9258374	63	72.1483374
AFTER	72.3258374	63	72.1358374
AFTER	72.5258374	63	72.1333374
AFTER	72.5258374	63	72.1333374
AFTER	72.2258374	63	72.1308374
AFTER	72.0258374	63	72.1258374
AFTER	72.1258374	63	72.1133374
AFTER	71.7258374	63	72.0585746
AFTER	72.3258374	63	72.0235746
AFTER	72.3258374	63	72.0010746
AFTER	72.2258374	63	71.9935746
AFTER	72.1258374	63	71.9860746
AFTER	71.9258374	63	71.9542504

AFTER	71.6258374	63	71.9717504
AFTER	71.4258374	63	71.9769716
AFTER	70.9258374	63	72.000602
AFTER	70.7258374	63	72.0424141
AFTER	70.9258374	63	72.0596738
AFTER	71.1258374	63	72.0642063
	71.1258374	63	72.0603296
	71.5258374	63	72.0494075
	71.7258374	63	72.0398491
	72.0258374	63	72.0209724
	72.4258374	63	72.0136927
	72.6258374	63	71.9982312
	72.7258374	63	71.9820879
	71.1353279	63	71.9736718
	71.9258374	63	71.9863922
	72.5258374	63	72.0122943
	73.0258374	63	72.0422943
	72.8258374	63	72.0647943
	71.8528684	63	72.0847943
	73.5258374	63	72.0854861
	72.6346866	63	72.0802688
	72.8710502	63	72.0743697
	73.198323	63	72.0734706
	72.1162262	63	72.0768897
	71.9071353	63	72.0825815
	71.6707717	63	72.0887279
	71.4889535	63	72.0866924
	71.5434989	63	72.1066924
	71.5707717	63	72.1141924
	72.2346497	63	72.1116924
	71.907377	63	72.1041924
	71.5801043	63	72.0941924
	71.6891952	63	72.0841924
	72.6346497	63	72.0541924
	72.7619224	63	72.0564551
	73.5258374	63	72.0339551
	73.2258374	63	71.9989551
	73.0258374	63	71.9314551
	72.1535098	63	71.8589551
	71.7171461	63	71.8182794
	71.3898734	63	71.7382794
	71.3898734	63	71.6780581
	71.0626007	63	71.6219278
	70.9535098	63	71.5626157
	71.1716916	63	71.5303559

71.0444189	63	71.5083235
71.9258374	63	71.4897001
71.8258374	63	71.4656222
71.6258374	63	71.4451807
71.7258374	63	71.4290573
72.0258374	63	71.411337
72.2258374	63	71.4067985
71.5258374	63	71.4204419
71.2258374	63	71.4338579
71.0258374	63	71.4161376
71.1258374	63	71.3952355
70.3258374	63	71.3627355
69.9258374	63	71.3502355
70.2258374	63	71.3402355
70.3258374	63	71.3620437
70.2258374	63	71.404761
70.6258374	63	71.4631601
70.8258374	63	71.5215592
70.8258374	63	71.5931401
71.0258374	63	71.685624
70.9258374	63	71.7735625
70.5258374	63	71.8701374
70.7258374	63	71.9396768
70.9258374	63	72.0198981
71.5258374	63	72.1110284
71.7258374	63	72.1692236
72.1258374	63	72.2235552
72.2258374	63	72.2633413
71.9258374	63	72.2992639
71.9258374	63	72.3420045
72.2258374	63	72.3867907
72.7258374	63	72.4290768
72.6258374	63	72.4786417
73.0258374	63	72.5372975
73.4258374	63	72.6222975
73.7258374	69	72.7022975
73.7258374	69	72.7797975
73.9258374	69	72.8347975
74.6528684	69	72.8597975
74.689232	69	72.8747975
74.9074139	69	72.8822975
74.7074139	69	72.8747975
75.0346866	69	72.8622975
75.2710502	69	72.8222975
74.0536466	69	72.7722975

74.1991011	69	72.7072975
73.8172829	69	72.6372975
72.9627375	69	72.5597975
72.9354647	69	72.4897975
72.8172829	69	72.4372975
72.8172829	69	72.3997975
72.3084337	69	72.3722975
72.2720701	69	72.3447975
73.6258374	69	72.3172975
73.5258374	69	72.2822975
73.3258374	69	72.2397975
72.8258374	69	72.1922975
71.8258374	69	72.1497975
71.4258374	69	72.1022975
71.3258374	69	72.0266218
70.6258374	69	71.9375369
70.0258374	69	71.8554975
69.1258374	69	71.7734581
68.9258374	69	71.6882368
68.9258374	69	71.5896065
68.9258374	69	71.5139113
69.0258374	69	71.4270797
69.4258374	69	71.3547936
69.8258374	69	71.2770468
70.4258374	69	71.2343061
71.1258374	69	71.17702
71.6258374	69	71.1172339
71.5258374	69	71.092669
71.6258374	69	71.0765131
71.7258374	69	71.0515131
71.8258374	69	71.0390131
72.0258374	69	71.0290131
72.0258374	69	71.0340131
71.6258374	69	71.0740131
71.1258374	69	71.1265131
71.6258374	69	71.1790131
71.4258374	69	71.2565131
71.6258374	69	71.3390131
71.3258374	69	71.4540131
71.0258374	69	71.5690131
70.7258374	69	71.6840131
70.9258374	69	71.7890131
69.8528684	69	71.8940131
71.2258374	69	71.9940131
70.5258374	69	72.0690131

70.4258374	69	72.1190131
71.3258374	69	72.1215131
71.6258374	69	72.0940131
72.6258374	69	72.0440131
73.0258374	69	71.9865131
72.9258374	69	71.9165131
73.0258374	69	71.8440131
73.4258374	69	71.7316142
73.5258374	69	71.6192153
73.4258374	69	71.5127255
73.7258374	69	71.4132811
73.3258374	69	71.3127004
73.7258374	69	71.219847
73.5258374	69	71.1247208
73.5258374	69	71.0343673
73.1258374	69	70.9701502
73.2258374	69	70.9476502
73.4258374	69	70.9126502
72.8258374	69	70.9069744
72.4258374	69	70.8519744
71.2258374	69	70.8044744
70.5258374	69	70.7444744
69.5258374	69	70.6644744
69.3258374	69	70.5869744
68.9258374	69	70.4894744
68.9258374	69	70.3844744
67.5298809	69	70.2819744
67.5298809	69	70.1794744
67.3662446	69	70.0694744
67.1480627	69	69.9644744
67.6026082	69	69.8594744
67.7116991	69	69.7619744
67.82079	69	69.6944744
67.7116991	69	69.6419744
68.4571537	69	69.6019744
69.8258374	69	69.5744744
69.5258374	69	69.5569744
69.6258374	69	69.5369744
69.0258374	69	69.5094744
68.6258374	69	69.4944744
68.0258374	69	69.4844744
68.1258374	69	69.4819744
68.5258374	69	69.4944744
68.7258374	69	69.5319744
68.8258374	69	69.5894744

68.8258374	69	69.6544744
68.9258374	69	69.7069744
69.0258374	69	69.8043734
69.3258374	69	69.8892723
69.2258374	69	69.9782621
69.8258374	69	70.0727064
70.6258374	69	70.1532872
71.6258374	69	70.2461406
71.9258374	69	70.3412668
72.4258374	69	70.4316203
72.4258374	69	70.4933374
72.4258374	69	70.5058374
72.3258374	69	70.5233374
72.2258374	69	70.5358374
72.0258374	69	70.5292345
71.1258374	69	70.5244498
71.0258374	69	70.5669498
71.0258374	69	70.6094498
71.6258374	69	70.6569498
71.5258374	69	70.6994498
71.0258374	69	70.7169498
71.4258374	69	70.7494498
70.9258374	69	70.7819498
70.9258374	69	70.8169498
70.9258374	69	70.8369498
70.8258374	69	70.8669498
71.4258374	69	70.8894498
71.6258374	69	70.8844498
71.3258374	69	70.8344498
70.9258374	69	70.7594498
70.3258374	69	70.6669498
70.2258374	69	70.5669498
70.1258374	69	70.4669498
68.761722	69	70.3719498
68.4344492	69	70.2694498
69.7258374	69	70.1719498
69.8258374	69	70.0919498
70.4258374	69	70.0219498
70.4258374	69	69.9469498
69.5258374	69	69.8569498
70.1258374	69	69.7669498
70.2258374	69	69.7019498
70.4258374	69	69.6294498
70.1258374	69	69.5769498
70.4258374	69	69.5319498

70.7258374	69	69.4844498
70.4258374	69	69.4319498
69.6258374	69	69.3669498
68.9258374	69	69.2994498
68.7258374	69	69.2369498
68.4258374	69	69.1869498
68.4258374	69	69.1569498
68.5258374	69	69.1319498
68.1258374	69	69.1269498
68.1258374	69	69.1635527
67.9258374	69	69.2158374
68.2258374	69	69.2358374
68.0258374	69	69.2533374
68.0258374	69	69.2558374
67.9258374	69	69.2508374
68.4258374	69	69.2883374
68.5258374	69	69.3033374
68.8258374	69	69.3133374
69.1258374	69	69.3183374
69.0258374	69	69.3233374
68.7258374	69	69.3158374
68.8258374	69	69.2958374
68.9258374	69	69.2933374
68.8258374	69	69.291393
68.9258374	69	69.3042214
69.1258374	69	69.324777
69.2258374	69	69.3582872
69.9258374	69	69.3857872
70.2258374	69	69.4132872
70.5258374	69	69.4457872
70.5258374	69	69.4857872
70.5258374	69	69.5307872
70.5258374	69	69.5682872
70.2258374	69	69.6007872
71.0258374	69	69.6307872
70.7258374	69	69.6532872
70.6258374	69	69.6607872
70.6258374	69	69.6657872
70.3258374	69	69.6632872
70.1258374	69	69.6532872
69.9258374	69	69.6507872
70.3258374	69	69.6711336
69.5480627	69	69.6989799
69.4389718	69	69.7188718
69.5480627	69	69.7399

69.7662446	69	69.74161
69.5258374	69	69.7442291
69.6258374	69	69.7443482
69.4258374	69	69.7274218
69.7258374	69	69.7193591
69.7258374	69	69.7256146
69.7258374	69	69.7381146
69.3258374	69	69.7431146
69.2258374	69	69.7381146
68.8258374	69	69.7306146
68.7258374	69	69.6981146
68.7258374	69	69.6681146
68.7258374	69	69.6431146
68.7258374	69	69.6156146
68.9258374	69	69.5956146
69.5396924	69	69.5856146
69.9396924	69	69.5781146
69.7215106	69	69.5681146
69.6669651	69	69.5900589
68.9942378	69	69.6297306
69.2306015	63	69.6891749
69.2306015	63	69.7481648
69.2487833	63	69.8131648
69.9033287	63	69.8756648
70.776056	63	69.9456648
71.0258374	63	70.0031648
70.7258374	63	70.0556648
70.3258374	63	70.0931648
69.9258374	63	70.1256648
69.7258374	63	70.1506648
69.5258374	63	70.1556648
69.6258374	63	70.1656648
69.5258374	63	70.1881648
69.5258374	63	70.2206648
69.7258374	63	70.2506648
69.6258374	63	70.2831648
69.9258374	63	70.2903184
70.4258374	63	70.282472
71.0258374	63	70.3075802
71.9258374	63	70.349052
72.1258374	63	70.429842
72.1258374	63	70.5072229
72.1258374	63	70.6046038
72.2258374	63	70.6810695
72.0258374	63	70.7502626

71.8258374	63	70.7908192
71.2258374	63	70.8142223
70.6258374	63	70.8342162
70.2258374	63	70.8687557
69.0258374	63	70.907386
69.1258374	63	70.9537436
69.6258374	63	71.0160103
70.0258374	63	71.0807769
69.9258374	63	71.1453164
70.2258374	63	71.2098558
69.8258374	63	71.2639406
69.6258374	63	71.3232528
70.7258374	63	71.3668831
71.3258374	63	71.3871043
72.2258374	63	71.3841437
72.3258374	63	71.3586832
73.1258374	63	71.327768
72.3074139	63	71.2963983
72.6710502	63	71.2759378
72.398323	63	71.2588863
71.9619593	63	71.249562
71.5255957	63	71.2479651
71.7074139	63	71.2618227
71.4710502	63	71.2965894
71.5801411	63	71.3449924
72.0165048	63	71.4224864
72.2165048	63	71.4706621
72.1074139	63	71.5008834
72.1074139	63	71.5274682
71.889232	63	71.5510986
71.998323	63	71.5645016
71.6710502	63	71.5879047
71.2346866	63	71.6213077
70.9074139	63	71.6326653
70.9074139	63	71.6231138
70.889232	63	71.5856078
70.8710502	63	71.5319654
71.3074139	63	71.449232
71.5437775	63	71.3928684
71.6528684	63	71.314232
71.7619593	63	71.2478684
71.7801411	63	71.2244654
72.0165048	63	71.1994714
72.1619593	63	71.174932
72.1255957	63	71.1538017

71.0528684	63	71.1099441
70.8346866	63	71.0476774
71.089232	63	70.9729107
70.8710502	63	70.9083713
70.7619593	63	70.8688319
70.7619593	63	70.849747
70.9619593	63	70.8279349
71.1801411	63	70.8193045
70.9437775	63	70.8340833
70.7255957	63	70.8695439
70.1801411	63	70.9025045
69.8165048	63	70.9284196
70.0528684	63	70.9497893
69.5255957	63	70.9577499
69.7437775	63	70.9523014
71.0258374	63	70.9491256
70.5258374	63	70.9382226
70.7258374	63	70.921865
70.6258374	63	70.8995983
69.8258374	63	70.8786952
69.5258374	63	70.8662013
69.2258374	63	70.8855255
69.5258374	63	70.9003043
70.5258374	63	70.8962194
71.1258374	63	70.8850891
71.1258374	63	70.879186
71.3258374	63	70.868283
71.8258374	63	70.8448799
72.3258374	63	70.8210223
72.2258374	63	70.7983955
71.9258374	63	70.8184016
71.7258374	63	70.857044
71.6258374	63	70.9022773
71.3258374	63	70.9516015
71.5258374	63	71.0166076
71.3258374	63	71.0811591
71.1258374	63	71.1261591
71.1258374	63	71.1936591
71.3258374	63	71.2611591
71.6258374	63	71.3336591
71.8258374	63	71.4261591
71.4258374	63	71.4929653
70.9258374	63	71.5672715
70.4258374	63	71.6597715
70.5258374	63	71.7322715

70.3258374	63	71.7872715
70.0258374	63	71.7863504
70.2258374	63	71.7863384
70.0387054	63	71.7829174
71.5258374	63	71.7724509
71.7258374	63	71.7703934
71.6258374	63	71.7731087
72.0258374	63	71.7890058
72.1258374	63	71.8110393
72.3258374	63	71.8487546
72.8258374	63	71.8869245
73.2258374	63	71.9382761
73.4258374	63	71.9837187
73.5258374	63	72.0246158
73.5258374	63	72.0596038
72.1980856	63	72.0925464
72.1980856	63	72.1177617
73.2258374	63	72.157977
73.4258374	63	72.202965
73.3258374	63	72.2522712
71.0889947	63	72.2990774
71.3253583	63	72.3563382
71.6889947	63	72.4351898
71.9071765	63	72.5090415
72.1435402	63	72.5962078
72.0344492	63	72.6434686
72.361722	63	72.6857293
72.5071765	63	72.7309446
72.8344492	63	72.7579781
73.0526311	63	72.7793298
73.3799038	63	72.7956814
72.9435402	63	72.8213513
72.761722	63	72.8292938
72.7253583	63	72.8204182
72.9435402	63	72.7899517
72.8344492	63	72.7567579
73.0344492	63	72.7508488
72.7253583	63	72.747667
72.3980856	63	72.735155
72.3980856	63	72.717643
72.6162674	63	72.7026311
73.1799038	63	72.7485402
73.1799038	63	72.7935402
73.5253583	63	72.8339947
73.4162674	63	72.871722

73.4162674	63	72.9137794
73.4344492	63	72.9485641
73.1071765	63	72.980167
72.9799038	63	73.0106335
72.9799038	63	73.0329182
73.8526311	63	73.0297484
73.5435402	63	73.0058967
73.0708129	63	72.9829541
72.3071765	63	72.964557
72.1980856	63	72.947069
71.961722	63	72.9241264
72.0708129	63	72.8914111
72.7253583	63	72.8561958
72.7253583	63	72.8162078
72.7253583	63	72.7869016
72.9253583	63	72.7550954
73.1253583	63	72.7203347
73.3071765	63	72.666483
73.4162674	63	72.6051313
73.8258374	63	72.5251433
73.4258374	63	72.4503826
73.6258374	63	72.3706218
73.7258374	63	72.2979065
73.7258374	63	72.245873
72.9258374	63	72.1970214
72.4258374	63	72.1381697
72.0258374	63	72.0649999
72.0258374	63	71.9970573
72.0258374	63	71.9209329
72.0258374	63	71.8438994
71.5258374	63	71.7645932
71.6258374	63	71.6861961
71.1258374	63	71.6075717
71.2258374	63	71.5125837
71.1258374	63	71.4150957
71.2258374	63	71.3301076
71.0258374	63	71.2426196
70.7258374	63	71.1426316
70.3258374	63	71.0330981
70.4258374	63	70.9183374
70.2258374	63	70.8008374
70.5258374	63	70.7083374
71.0258374	63	70.6133374
71.0258374	63	70.5108374
70.6258374	63	70.4083374

	70.9258374	63	70.3208374
	70.8258374	63	70.2458374
	70.0258374	63	70.1858374
	69.2258374	63	70.1258374
	69.0258374	63	70.0733374
	68.8258374	63	70.0308374
	68.9258374	63	70.0033374
	68.9258374	63	69.9908374
	68.8258374	63	69.9858374
	69.3258374	63	69.9783374
	69.4258374	63	69.9883374
	69.1258374	63	70.0183374
	68.9258374	63	70.0708374
	68.8258374	63	70.1308374
	69.1258374	63	70.1908374
	69.7258374	63	70.2483374
	69.8258374	63	70.3158374
	69.6258374	63	70.3808374
	69.6258374	63	70.4183374
	69.4258374	63	70.417189
	69.4258374	63	70.442189
	69.6258374	63	70.454689
	69.6258374	63	70.472189
	69.9258374	63	70.509689
	70.3258374	63	70.569689
	70.4258374	63	70.644689
	71.1258374	63	70.727189
	70.9258374	63	70.817189
	70.9258374	63	70.8849043
	71.5258374	63	70.9924043
Before 1	72.4258374	63	71.0874043
Before 2	73.1258374	63	71.1674043
Before 3	73.1258374	63	71.2449043
Before 4	72.7258374	63	71.3324043
Before 5	72.7258374	63	71.4349043
Before 6	72.9258374	63	71.5349043
Before 7	73.1258374	63	71.5949043
Before 8	72.5258374	63	71.6151256
Before 9	70.9799038	63	71.632165
Before 10	71.6258374	63	71.6419317
Before 11	71.4258374	63	71.6576074
Before 12	71.5258374	63	71.6810105
Before 13	71.5258374	63	71.7048681
Before 14	71.6258374	63	71.7328166
Before 15	72.0258374	63	71.7653166

Before 16	72.1258374	63	71.7703166
Before 17	72.5258374	63	71.7503166
Before 18	71.6344492	63	71.6928166
Before 19	73.1258374	63	71.6278166
Before 20	73.1258374	63	71.5603166
Before 21	72.6258374	63	71.4753166
Before 22	72.2258374	63	71.3753166
Before 23	72.4258374	63	71.2503166
Before 24	72.9258374	63	71.1203166
Before 25	73.1258374	63	71.0078166
Before 26	72.1258374	63	70.8953166
Before 27	70.6346866	63	70.7753166
Before 28	70.3074139	63	70.6428166
Before 29	70.0165048	63	70.5328166
Before 30	70.0528684	63	70.4589649
Before 31	70.3619593	63	70.3814649
Before 32	70.5801411	63	70.3214649
Before 33	70.7437775	63	70.2664649
Before 34	71.2258374	63	70.2364649
Before 35	70.5258374	63	70.2189649
Before 36	69.6258374	63	70.2014649
Before 37	68.8258374	63	70.1789649
Before 38	68.3258374	63	70.1464649
Before 39	68.2258374	63	70.1437496
Before 40	68.1258374	63	70.1037496
Before 41	68.4258374	63	70.0562496
Before 42	68.1258374	63	69.9862496
Before 43	67.9258374	63	69.9162496
Before 44	68.2258374	63	69.8387496
Before 45	68.2258374	63	69.7712496
Before 46	68.1258374	63	69.7037496
Before 47	67.8258374	63	69.6587496
Before 48	68.1258374	63	69.6560284
Before 49	68.0258374	63	69.653989
Before 50	68.5258374	63	69.6667223
Before 51	69.0258374	63	69.6860465
Before 52	69.3258374	63	69.6737679
Before 53	70.3258374	63	69.6537619
Before 54	70.9258374	63	69.6210287
Before 55	71.3258374	63	69.556244
Before 56	71.2258374	63	69.508744
Before 57	71.2258374	63	69.493744
Before 58	71.5258374	63	69.501244
Before 59	71.5258374	63	69.526244
Before 60	71.2258374	63	69.556244

Before 61	69.8258374	63	69.588744
Before 62	69.4258374	63	69.613744
Before 63	69.3258374	63	69.643744
Before 64	70.2258374	63	69.681244
Before 65	70.4258374	63	69.703744
Before 66	70.3258374	63	69.728744
Before 67	70.5258374	63	69.753744
Before 68	70.2258374	63	69.788744
Before 69	70.5258374	63	69.816244
Before 70	70.8258374	63	69.838744
Before 71	69.8708129	63	69.861244
Before 72	69.7799038	63	69.883744
Before 73	69.4344492	63	69.901244
Before 74	68.6344492	63	69.896244
Before 75	68.6258374	63	69.878744
Before 76	69.0258374	63	69.868744
Before 77	69.1258374	63	69.881244
Before 78	69.3258374	63	69.908744
Before 79	69.4258374	63	69.936244
Before 80	69.4258374	63	69.986244
Before 81	69.4258374	63	70.053744
Before 82	69.3258374	63	70.151244
Before 83	69.4258374	63	70.251244
Before 84	69.1258374	63	70.341244
Before 85	69.2258374	63	70.388744
Before 86	69.1258374	63	70.428744
Before 87	69.2258374	63	70.483744
Before 88	69.2258374	63	70.531244
Before 89	68.9258374	63	70.5715657
Before 90	69.4258374	63	70.6021147
Before 91	69.9258374	63	70.6174364
Before 92	70.0258374	63	70.6566337
Before 93	70.1258374	63	70.6876492
Before 94	70.2258374	63	70.7644656
Before 95	70.9258374	63	70.8728729
Before 96	71.7258374	63	71.0111739
Before 97	72.3258374	63	71.0963874
Before 98	72.6258374	63	71.1613736
Before 99	73.5258374	63	71.2136326
Before 100	73.9258374	63	71.2768007
Before 101	73.7258374	69	71.3356506
Before 102	73.4258374	69	71.4155178
Before 103	72.9258374	69	71.5130178
Before 104	72.1258374	69	71.6030178
Before 105	72.0258374	69	71.6930178

Before 106	72.5258374	69	71.7605178
Before 107	72.4258374	69	71.8205178
Before 108	71.8387054	69	71.8805178
Before 109	71.7477963	69	71.9181122
Before 110	71.4387054	69	71.9609339
Before 111	71.4387054	69	71.9680738
Before 112	71.0205236	69	71.9768046
Before 113	72.5071059	69	71.979399
Before 114	72.9707422	69	71.9831298
Before 115	74.1578742	69	71.9957242
Before 116	72.4343786	69	71.9935459
Before 117	71.7252877	69	71.9690949
Before 118	71.4161968	69	71.9650984
Before 119	71.9525604	69	71.9808746
Before 120	71.7798331	69	71.9959691
Before 121	72.6205236	69	72.0010635
Before 122	73.2258374	69	72.0111579
Before 123	73.0258374	69	72.0032978
Before 124	72.7258374	69	72.0020286
Before 125	71.9258374	69	72.0153049
Before 126	71.5258374	69	72.0174447
Before 127	71.6258374	69	72.0070846
Before 128	70.7296145	69	71.9992245
Before 129	70.6387054	69	72.0001336
Before 130	69.7114327	69	72.0060427
Before 131	70.275069	69	72.0224063
Before 132	70.1296145	69	72.03877
Before 133	70.275069	69	72.054679
Before 134	70.7296145	69	72.0306963
Before 135	70.8387054	69	71.9869408
Before 136	70.7477963	63	71.9162344
Before 137	72.1659781	63	71.890888
Before 138	73.2568872	63	71.9009961
Before 139	74.1296145	63	71.9297407
Before 140	74.1296145	63	71.9765726
Before 141	74.1296145	63	72.0452227
Before 142	73.1114327	63	72.1128556
Before 143	72.875069	63	72.1578556
Before 144	72.6568872	63	72.2053556
Before 145	72.1114327	63	72.2428556
Before 146	72.1114327	63	72.3003556
Before 147	72.1114327	63	72.3303556
Before 148	71.875069	63	72.3478556
Before 149	71.98416	63	72.3827611
Before 150	72.0932509	63	72.4174394

Before 151	72.0932509	63	72.4752995
Before 152	71.6568872	63	72.5090688
Before 153	71.5477963	63	72.5414743
Before 154	71.2205236	63	72.5727435
Before 155	71.3296145	63	72.5801491
Before 156	71.4205236	63	72.5848274
Before 157	72.1296145	63	72.5624542
Before 158	72.5659781	63	72.5364507
Before 159	73.8258374	63	72.4522593
Before 160	74.5258374	63	72.3467043
Before 161	75.3258374	63	72.2420584
Before 162	75.0258374	63	72.1455943
Before 163	74.9258374	63	72.0832211
Before 164	74.2258374	63	72.0244842
Before 165	74.2258374	63	71.9852928
Before 166	72.7258374	63	71.9488287
Before 167	72.3258374	63	71.9041828
Before 168	72.1258374	63	71.8513551
Before 169	72.0258374	63	71.8258001
Before 170	72.0258374	63	71.8061541
Before 171	71.6258374	63	71.7844688
Before 172	71.4258374	63	71.7802835
Before 173	71.5258374	63	71.7920072
Before 174	71.0258374	63	71.7989582
Before 175	71.0258374	63	71.8165911
Before 176	69.8528684	63	71.8189966
Before 177	71.1258374	63	71.8141295
Before 178	69.889232	63	71.7940351
Before 179	69.9074139	63	71.7730315
Before 180	69.9437775	63	71.7230315
Before 181	70.2710502	63	71.6505315
Before 182	70.6165048	63	71.5505315
Before 183	70.5255957	63	71.4605315
Before 184	71.089232	63	71.3805315
Before 185	70.6528684	63	71.3130315
Before 186	70.3255957	63	71.2380315
Before 187	69.998323	63	71.2080315
Before 188	70.8528684	63	71.1749508
Before 189	71.198323	63	71.1514155
Before 190	71.2258374	63	71.1299257
Before 191	71.9258374	63	71.1334359
Before 192	72.1258374	63	71.161037
Before 193	71.8258374	63	71.2018199
Before 194	71.9258374	63	71.2373755
Before 195	71.4258374	63	71.2990675

Before 196	71.2258374	63	71.355305
Before 197	71.3258374	63	71.4354121
Before 198	71.7258374	63	71.4923314
Before 199	71.8258374	63	71.5783476
Before 200	71.6258374	63	71.6639093
Before 201	71.3258374	63	71.7681073
Before 202	71.4258374	63	71.9023054
Before 203	71.7258374	63	72.0125336
Before 204	71.5258374	63	72.1316255
Before 205	71.2258374	63	72.2275356
Before 206	71.5258374	63	72.3298093
Before 207	71.0026082	63	72.4164012
Before 208	71.1844264	63	72.515392
Before 209	71.1662446	63	72.5898374
Before 210	72.1662446	63	72.64201
Before 211	72.7298809	63	72.6916766
Before 212	73.0571537	63	72.7233886
Before 213	72.9480627	63	72.7419189
Before 214	73.4935173	63	72.7819189
Before 215	73.2753355	63	72.8119189
Before 216	73.0571537	63	72.8394189
Before 217	73.4026082	63	72.8569189
Before 218	73.3298809	63	72.8469189
Before 219	73.3298809	63	72.8094189
Before 220	74.1116991	63	72.7544189
Before 221	75.6389718	63	72.7044189
Before 222	75.0256353	63	72.6594189
Before 223	75.2892716	63	72.6194189
Before 224	74.9256353	63	72.5844189
Before 225	74.7438171	63	72.5519189
Before 226	73.7892716	63	72.5394189
Before 227	73.9579553	63	72.5319189
Before 228	73.8306826	63	72.5499996
Before 229	73.2852281	63	72.5710349
Before 230	73.2125008	63	72.6050247
Before 231	73.194319	63	72.6065145
Before 232	72.8670462	63	72.5889134
Before 233	73.4258374	63	72.5297409
Before 234	73.1258374	63	72.456932
Before 235	72.5258374	63	72.373214
Before 236	71.9258374	63	72.3008596
Before 237	70.9258374	63	72.2371416
Before 238	70.2258374	63	72.1706963
Before 239	69.6258374	63	72.1028874
Before 240	69.6258374	63	72.0350785

Minute 1	69.5258374	63	71.9395423
Minute 2	69.8258374	63	71.8030971
Minute 3	70.3258374	63	71.6874398
Minute 4	70.2258374	63	71.5933539
Minute 5	70.7258374	63	71.5033589
Minute 6	71.2258374	63	71.3954095
Minute 7	71.7258374	63	71.3088236
Minute 8	72.0258374	63	71.2080207
Minute 9	72.5258374	63	71.1203995
Minute 10	72.2258374	63	71.0489148
Minute 11	72.0258374	63	70.9892482
Minute 12	70.6902519	63	70.9275361
Minute 13	70.0357064	63	70.8815059
Minute 14	70.1447973	63	70.8215059
Minute 15	70.381161	63	70.7590059
Minute 16	70.5084337	63	70.7240059
Minute 17	70.7447973	63	70.7040059
Minute 18	70.6175246	63	70.7140059
Minute 19	70.6175246	63	70.7365059
Minute 20	70.2902519	63	70.7590059
Minute 21	70.181161	63	70.7540059
Minute 22	70.3993428	63	70.7365059
Minute 23	71.5258374	63	70.7115059
Minute 24	71.3258374	63	70.6765059
Minute 25	70.4258374	63	70.6415059
Minute 26	70.3258374	63	70.5965059
Minute 27	69.9258374	63	70.5465059
Minute 28	70.3258374	63	70.4965059
Minute 29	70.4258374	63	70.4365059
Minute 30	70.8258374	63	70.3640059
Minute 31	70.7258374	63	70.3090059
Minute 32	71.0258374	63	70.2740059
Minute 33	71.0258374	63	70.2773955
Minute 34	70.6258374	63	70.3096488
Minute 35	71.1258374	63	70.3466748
Minute 36	71.1258374	63	70.3877917
Minute 37	71.3258374	63	70.4357268
Minute 38	71.1258374	63	70.4902528
Minute 39	70.5258374	63	70.5504606
Minute 40	69.4258374	63	70.5756685
Minute 41	68.8258374	63	70.6115581
Minute 42	68.8258374	63	70.645175
Minute 43	68.9258374	63	70.6658374
Minute 44	68.8258374	63	70.6483374
Minute 45	68.9258374	63	70.6358374

Minute 46	69.2258374	63	70.6458374
Minute 47	69.7258374	63	70.6583374
Minute 48	69.6258374	63	70.6833374
Minute 49	69.6258374	63	70.7083374
Minute 50	70.0258374	63	70.7208374
Minute 51	70.6258374	63	70.7158374
Minute 52	70.8258374	63	70.7058374
Minute 53	71.3258374	63	70.6808374
Minute 54	71.6258374	63	70.6558374
Minute 55	72.0258374	63	70.6433374
Minute 56	72.4258374	63	70.6133374
Minute 57	72.9258374	63	70.5808374
Minute 58	73.0258374	63	70.5408374
Minute 59	71.6258374	63	70.5008374
Minute 60	71.7258374	63	70.4683374
Movie 1	71.5258374	65	70.4558374
Movie 2	71.2258374	65	70.4608374
Movie 3	70.8258374	65	70.4583374
Movie 4	70.8258374	65	70.4683374
Movie 5	70.8258374	65	70.4833374
Movie 6	70.8258374	65	70.4933374
Movie 7	70.9258374	65	70.4958374
Movie 8	71.3258374	65	70.4858374
Movie 9	70.9258374	65	70.4733374
Movie 10	70.6258374	65	70.4633374
Movie 11	70.3258374	65	70.4458374
Movie 12	70.0258374	65	70.4133374
Movie 13	70.0258374	65	70.3708374
Movie 14	70.1258374	65	70.2913114
Movie 15	69.9258374	65	70.2179217
Movie 16	69.8258374	65	70.1395321
Movie 17	69.7258374	65	70.0520515
Movie 18	69.5258374	65	69.952071
Movie 19	69.2258374	65	69.8468632
Movie 20	68.9258374	65	69.7743826
Movie 21	69.0258374	65	69.6971294
Movie 22	68.7258374	65	69.6248761
Movie 23	69.3258374	65	69.5492137
Movie 24	69.4258374	65	69.4835514
Movie 25	69.3258374	65	69.4110708
Movie 26	69.3258374	65	69.3354084
Movie 27	69.3258374	65	69.2661097
Movie 28	69.1258374	65	69.2029474
Movie 29	69.2258374	65	69.1406941
Movie 30	69.3258374	65	69.0884408

Movie 31	69.3258374	65	69.0518694
Movie 32	69.1258374	65	69.0309797
Movie 33	68.1447973	65	69.0112264
Movie 34	68.6902519	65	68.9728368
Movie 35	68.8902519	65	68.940129
Movie 36	68.9266155	65	68.925129
Movie 37	68.9266155	65	68.915129
Movie 38	68.8175246	65	68.905129
Movie 39	68.7266155	65	68.900129
Movie 40	68.6357064	65	68.905129
Movie 41	68.6357064	65	68.915129
Movie 42	68.1993428	65	68.932629
Movie 43	68.1993428	65	68.950129
Movie 44	67.9266155	65	68.955129
Movie 45	67.7993428	65	68.940129
Movie 46	68.0538882	65	68.912629
Movie 47	68.3993428	65	68.875129
Movie 48	68.8357064	65	68.825129
Movie 49	68.8357064	65	68.760129
Movie 50	69.1629792	65	68.680129
Movie 51	69.4902519	65	68.600129
Movie 52	69.2357064	65	68.532629
Movie 53	68.4902519	65	68.453103
Movie 54	68.8175246	65	68.3912848
Movie 55	69.3258374	65	68.3112848
Movie 56	69.4258374	65	68.2344666
Movie 57	69.3258374	65	68.1676484
Movie 58	69.3258374	65	68.0971939
Movie 59	69.4258374	65	68.0440121
Movie 60	69.3258374	65	68.0014926
Movie 61	69.7258374	65	67.9462459
Movie 62	69.4258374	65	67.8909992
Movie 63	69.5258374	65	67.8416615
Movie 64	68.8258374	65	67.7848239
Movie 65	68.2258374	65	67.7323044
Movie 66	67.8258374	65	67.6729668
Movie 67	67.3258374	65	67.6072655
Movie 68	66.5258374	65	67.5329279
Movie 69	66.0258374	65	67.4476812
Movie 70	66.1258374	65	67.3624345
Movie 71	66.6258374	65	67.2765059
Movie 72	65.9447973	65	67.1898955
Movie 73	65.6720701	65	67.1246488
Movie 74	65.4902519	65	67.0880385
Movie 75	65.8175246	65	67.0457463

Movie 76	66.2538882	65	66.9807463
Movie 77	66.1084337	65	66.9132463
Movie 78	66.6902519	65	66.8582463
Movie 79	67.0258374	65	66.8057463
Movie 80	66.4258374	65	66.7532463
Movie 81	66.4258374	65	66.7107463
Movie 82	66.2258374	65	66.6632463
Movie 83	65.9258374	65	66.6157463
Movie 84	65.8258374	65	66.5682463
Movie 85	65.4258374	65	66.5332463
Movie 86	65.4258374	65	66.5107463
Movie 87	65.4258374	65	66.4957463
Movie 88	65.4258374	65	66.4957463
Movie 89	65.4258374	65	66.5082463
Movie 90	65.7258374	65	66.5232463
Movie 91	66.0258374	65	66.5432463
Movie 92	66.6258374	65	66.5557463
Movie 93	67.0258374	65	66.5802723
Movie 94	67.1258374	65	66.6116165
Movie 95	66.7258374	65	66.6475061
Movie 96	66.7258374	65	66.641597
Movie 97	67.1258374	65	66.616597
Movie 98	67.2258374	65	66.5870515
Movie 99	67.3258374	65	66.531597
Movie 100	67.6258374	65	66.496597
Movie 101	67.8258374	65	66.476597
Movie 102	67.5258374	65	66.464097
Movie 103	67.6258374	65	66.449097
Movie 104	67.4258374	65	66.449097
Movie 105	67.3258374	65	66.449097
Movie 106	67.2258374	65	66.461597
Movie 107	67.3258374	65	66.479097
Movie 108	67.0258374	65	66.501597
Movie 109	66.6258374	65	66.526597
Movie 110	66.9258374	65	66.559097
Movie 111	67.1258374	65	66.584097
Movie 112	66.9258374	65	66.606597
Movie 113	66.9258374	65	66.611597
Movie 114	66.9258374	65	66.626597
Movie 115	65.581161	65	66.656597
Movie 116	65.2538882	65	66.696597
Movie 117	64.9266155	65	66.744097
Movie 118	64.4720701	65	66.771597
Movie 119	65.6258374	65	66.7703545
Movie 120	65.6258374	65	66.766612

Movie 121	65.9258374	65	66.809112
Movie 122	65.6258374	65	66.849112
Movie 123	65.9258374	65	66.904112
Movie 124	65.8258374	65	66.964112
Movie 125	65.9258374	65	67.024112
Movie 126	66.1258374	65	67.099112
Movie 127	66.3258374	65	67.189112
Movie 128	66.4258374	65	67.281612
Movie 129	66.7258374	65	67.386612
Movie 130	66.7258374	65	67.519112
Movie 131	66.9258374	65	67.649112
Movie 132	66.8258374	65	67.796612
Movie 133	67.6258374	65	67.946612
Movie 134	68.3258374	65	68.089112
Movie 135	68.3258374	65	68.229112
Movie 136	68.6258374	65	68.4077289
Movie 137	68.2258374	65	68.5970276
Movie 138	67.1761371	65	68.7945082
Movie 139	67.1761371	65	68.9833524
Movie 140	69.3258374	65	69.1408524
Movie 141	69.4258374	65	69.2908524
Movie 142	69.7258374	65	69.4258524
Movie 143	70.0258374	65	69.5633524
Movie 144	69.8258374	65	69.6908524
Movie 145	70.3258374	65	69.8208524
Movie 146	70.8258374	65	69.9508524
Movie 147	71.0258374	65	70.0758524
Movie 148	71.2258374	65	70.2008524
Movie 149	71.9258374	65	70.3208524
Movie 150	72.1258374	65	70.4258524
Minute 1	73.0258374	63	70.5283524
Minute 2	72.9258374	63	70.6258524
Minute 3	72.6258374	63	70.7133524
Minute 4	72.5258374	63	70.7783524
Minute 5	72.7258374	63	70.8283524
Minute 6	72.8258374	63	70.8783524
Minute 7	72.8258374	63	70.9283524
Minute 8	72.0258374	63	70.9958524
Minute 9	71.9258374	63	71.0820949
Minute 10	71.6258374	63	71.1483374
Minute 11	71.3258374	63	71.1608374
Minute 12	71.1258374	63	71.1733374
Minute 13	71.0258374	63	71.1833374
Minute 14	71.0258374	63	71.2033374
Minute 15	71.1258374	63	71.202675

Minute 16	71.1258374	63	71.1895126
Minute 17	71.3258374	63	71.161123
Minute 18	71.2258374	63	71.1218243
Minute 19	70.9258374	63	71.1168243
Minute 20	70.8258374	63	71.0943243
Minute 21	70.8258374	63	71.0893243
Minute 22	70.3258374	63	71.0618243
Minute 23	70.2258374	63	71.0468243
Minute 24	70.3258374	63	71.0368243
Minute 25	70.3258374	63	71.0193243
Minute 26	70.6258374	63	71.0068243
Minute 27	70.9258374	63	70.9968243
Minute 28	70.6258374	63	70.9868243
Minute 29	69.8258374	63	70.9868243
Minute 30	69.8258374	63	70.9943243
Minute 31	69.9258374	63	71.0243243
Minute 32	70.1258374	63	71.0743243
Minute 33	70.8258374	63	71.1268243
Minute 34	69.7993428	63	71.1893243
Minute 35	69.7993428	63	71.2493243
Minute 36	69.6902519	63	71.3268243
Minute 37	69.4538882	63	71.3993243
Minute 38	71.0258374	63	71.4668243
Minute 39	71.0258374	63	71.5368243
Minute 40	71.9258374	63	71.6093243
Minute 41	71.9258374	63	71.6843243
Minute 42	72.3258374	63	71.7518243
Minute 43	72.2258374	63	71.8168243
Minute 44	71.8258374	63	71.8718243
Minute 45	72.2258374	63	71.9143243
Minute 46	72.4258374	63	71.9568243
Minute 47	72.4258374	63	71.9868243
Minute 48	72.0258374	63	72.0043243
Minute 49	72.2258374	63	72.0243243
Minute 50	72.8258374	63	72.0568243
Minute 51	73.3258374	63	72.0718243
Minute 52	73.2258374	63	72.0843243
Minute 53	73.5258374	63	72.0943243
Minute 54	73.4258374	63	72.0893243
Minute 55	74.2258374	63	72.1124866
Minute 56	74.0258374	63	72.153149
Minute 57	74.0258374	63	72.2090386
Minute 58	74.0258374	63	72.2708374
Minute 59	73.8258374	63	72.3158374
Minute 60	73.8258374	63	72.3658374

After 1	73.5258374	63	72.3833374
After 2	72.9258374	63	72.3958374
After 3	72.4258374	63	72.4258374
After 4	72.0258374	63	72.4633374
After 5	72.0258374	63	72.5108374
After 6	71.8258374	63	72.5458374
After 7	71.6258374	63	72.5858374
After 8	71.4258374	63	72.6283374
After 9	71.1258374	63	72.6958374
After 10	70.4258374	63	72.7683374
After 11	70.4258374	63	72.8333374
After 12	70.5258374	63	72.8858374
After 13	70.6258374	63	72.9433374
After 14	70.7258374	63	72.9933374
After 15	71.4258374	63	73.0458374
After 16	71.9258374	63	73.0683374
After 17	71.9258374	63	73.0908374
After 18	72.8258374	63	73.0933374
After 19	73.0258374	63	73.0808374
After 20	72.6258374	63	73.0808374
After 21	72.4258374	63	73.0758374
After 22	73.5258374	63	73.0833374
After 23	73.7258374	63	73.1158374
After 24	73.7258374	63	73.1683374
After 25	73.6258374	63	73.2333374
After 26	74.0258374	63	73.3083374
After 27	74.1258374	63	73.4058374
After 28	74.7258374	63	73.4899383
After 29	75.1258374	63	73.5649483
After 30	75.4258374	63	73.6474583
After 31	75.4258374	63	73.7638319
After 32	75.5258374	63	73.8865691
After 33	75.5258374	63	74.02317
After 34	75.5258374	63	74.1654528
After 35	75.1258374	63	74.2997809
After 36	74.9258374	63	74.4325182
After 37	74.1258374	63	74.5441191
After 38	73.5258374	63	74.6361746
After 39	73.8258374	63	74.6811845
After 40	73.6258374	63	74.71574
After 41	73.8258374	63	74.7702954
After 42	74.2258374	63	74.8627954
After 43	74.5258374	63	74.9252954
After 44	74.6258374	63	74.9727954
After 45	75.0258374	63	75.0077954

After 46	75.7258374	63	75.0352954
After 47	74.9898734	63	75.0184872
After 48	74.426237	63	74.9919063
After 49	74.426237	63	74.9403254
After 50	75.0807825	63	74.8787445
After 51	75.3353279	63	74.8069363
After 52	75.9898734	63	74.7255827
After 53	76.3171461	63	74.6526381
After 54	76.0989643	63	74.5906026
After 55	76.7353279	63	74.5349308
After 56	76.3898734	63	74.5056226
After 57	75.6080552	63	74.4931326
After 58	74.626237	63	74.509279
After 59	74.4080552	63	74.5404253
After 60	74.8080552	63	74.5586171
After 61	76.1258374	63	74.5825346
After 62	76.0258374	63	74.5332569
After 63	75.6258374	63	74.5413469
After 64	75.1258374	63	74.5417661
After 65	74.7258374	63	74.5394308
After 66	73.3535098	63	74.5255334
After 67	73.0626007	63	74.4902304
After 68	72.6626007	63	74.4750897
After 69	72.6626007	63	74.5545811
After 70	72.5535098	63	74.5585918
After 71	72.1716916	63	74.8640011
After 72	72.6080552	63	75.1614559
After 73	73.0444189	63	75.4509561
After 74	73.2989643	63	75.7302291
After 75	73.9535098	63	76.0163202
After 76	74.426237	63	76.288775
After 77	74.7716916	63	76.634378
After 78	74.7716916	63	77.0044128
After 79	74.5535098	63	77.4051294

		Person 2	Person 1	Person 7	Person 5	Person 10
Time						
20:02:50	0:00:01		72.8813582		88.421441	87.1725398
20:02:51	0:00:02		72.8813582		88.421441	87.1725398
20:02:52	0:00:03		72.8813582		88.421441	88.1725398
20:02:53	0:00:04		72.8813582		88.421441	88.1725398
20:02:54	0:00:05		72.8813582		88.421441	88.1725398
20:02:55	0:00:06		73.8813582		87.421441	89.1725398
20:02:56	0:00:07	95.4256152	73.8813582		87.421441	90.1725398
20:02:57	0:00:08	95.4256152	73.8813582		87.421441	90.1725398
20:02:58	0:00:09	95.4256152	75.8813582		87.421441	89.1725398
20:02:59	0:00:10	95.7589485	77.8813582		87.421441	89.1725398
20:03:00	0:00:11	96.0922819	79.8813582		87.421441	89.1725398
20:03:01	0:00:12	95.7589485	79.8813582		86.421441	89.1725398
20:03:02	0:00:13	95.7589485	79.8813582		86.421441	89.1725398
20:03:03	0:00:14	96.0922819	81.8813582		86.421441	89.1725398
20:03:04	0:00:15	96.4256152	81.8813582		86.421441	90.1725398
20:03:05	0:00:16	96.2589485	81.8813582		86.421441	90.1725398
20:03:06	0:00:17	98.0922819	82.8813582		86.421441	95.1725398
20:03:07	0:00:18	103.258949	82.8813582		87.421441	109.17254
20:03:08	0:00:19	104.425615	82.8813582		88.421441	112.17254
20:03:09	0:00:20	104.592282	82.8813582		88.421441	112.17254
20:03:10	0:00:21	105.925615	82.8813582		89.421441	115.17254
20:03:11	0:00:22	106.258949	81.8813582		89.421441	117.17254
20:03:12	0:00:23	106.925615	81.8813582		90.421441	118.17254
20:03:13	0:00:24	107.925615	81.8813582		91.421441	120.17254
20:03:14	0:00:25	108.758949	81.8813582		91.421441	120.17254
20:03:15	0:00:26	108.258949	75.8813582		91.421441	120.17254
20:03:16	0:00:27	107.925615	71.8813582		91.421441	120.17254
20:03:17	0:00:28	108.425615	71.8813582		91.421441	120.17254
20:03:18	0:00:29	108.925615	71.8813582		91.421441	120.17254
20:03:19	0:00:30	109.258949	69.8813582		91.421441	121.17254
20:03:20	0:00:31	109.592282	69.8813582		92.421441	121.17254
20:03:21	0:00:32	110.258949	71.8813582		92.421441	121.17254
20:03:22	0:00:33	110.092282	72.8813582		92.421441	120.17254
20:03:23	0:00:34	109.592282	73.8813582		91.421441	119.17254
20:03:24	0:00:35	109.425615	73.8813582		90.421441	119.17254
20:03:25	0:00:36	108.758949	72.8813582		90.421441	117.17254
20:03:26	0:00:37	108.758949	72.8813582		90.421441	117.17254
20:03:27	0:00:38	107.258949	70.8813582		91.421441	112.17254
20:03:28	0:00:39	107.925615	69.8813582		93.421441	112.17254
20:03:29	0:00:40	107.592282	69.8813582		94.421441	110.17254
20:03:30	0:00:41	107.925615	69.8813582		95.421441	110.17254
20:03:31	0:00:42	107.925615	69.8813582		95.421441	110.17254

20:03:32	0:00:43	107.925615	69.8813582		95.421441	110.17254
20:03:33	0:00:44	108.425615	69.8813582		95.421441	111.17254
20:03:34	0:00:45	108.092282	69.8813582		94.421441	111.17254
20:03:35	0:00:46	108.092282	69.8813582		94.421441	111.17254
20:03:36	0:00:47	107.925615	69.8813582		93.421441	111.17254
20:03:37	0:00:48	107.592282	69.8813582		93.421441	110.17254
20:03:38	0:00:49	107.258949	69.8813582		93.421441	109.17254
20:03:39	0:00:50	106.925615	69.8813582		93.421441	108.17254
20:03:40	0:00:51	106.758949	68.8813582		93.421441	108.17254
20:03:41	0:00:52	106.425615	68.8813582		92.421441	108.17254
20:03:42	0:00:53	106.258949	68.8813582		91.421441	108.17254
20:03:43	0:00:54	106.758949	68.8813582		93.421441	107.17254
20:03:44	0:00:55	107.592282	71.8813582		93.421441	108.17254
20:03:45	0:00:56	108.592282	71.8813582		93.421441	110.17254
20:03:46	0:00:57	108.592282	71.8813582		93.421441	110.17254
20:03:47	0:00:58	108.592282	71.8813582		93.421441	110.17254
20:03:48	0:00:59	108.758949	72.8813582		93.421441	110.17254
20:03:49	0:01:00	109.258949	75.8813582		93.421441	110.17254
20:03:50	0:01:01	109.925615	79.8813582		93.421441	110.17254
20:03:51	0:01:02	109.425615	79.8813582		95.421441	107.17254
20:03:52	0:01:03	109.925615	82.8813582		95.421441	107.17254
20:03:53	0:01:04	110.425615	83.8813582		97.421441	106.17254
20:03:54	0:01:05	109.925615	82.8813582		97.421441	105.17254
20:03:55	0:01:06	109.925615	80.8813582		98.421441	105.17254
20:03:56	0:01:07	108.592282	74.8813582		98.421441	104.17254
20:03:57	0:01:08	107.925615	74.8813582		99.421441	103.17254
20:03:58	0:01:09	106.092282	73.8813582		99.421441	98.1725398
20:03:59	0:01:10	105.592282	73.8813582		99.421441	98.1725398
20:04:00	0:01:11	104.425615	73.8813582		98.421441	96.1725398
20:04:01	0:01:12	102.758949	73.8813582		96.421441	93.1725398
20:04:02	0:01:13	102.092282	73.8813582		96.421441	91.1725398
20:04:03	0:01:14	100.925615	73.8813582		94.421441	90.1725398
20:04:04	0:01:15	100.425615	72.8813582		93.421441	90.1725398
20:04:05	0:01:16	100.425615	72.8813582		93.421441	90.1725398
20:04:06	0:01:17	99.2589485	69.8813582		91.421441	90.1725398
20:04:07	0:01:18	99.2589485	69.8813582		91.421441	90.1725398
20:04:08	0:01:19	98.0922819	68.8813582		89.421441	89.1725398
20:04:09	0:01:20	97.9256152	67.8813582		89.421441	89.1725398
20:04:10	0:01:21	97.9256152	67.8813582		89.421441	89.1725398
20:04:11	0:01:22	97.5922819	67.8813582		88.421441	89.1725398
20:04:12	0:01:23	97.5922819	67.8813582		88.421441	89.1725398
20:04:13	0:01:24	93.4256152	68.8813582		88.421441	76.1725398
20:04:14	0:01:25	92.9256152	68.8813582		87.421441	76.1725398
20:04:15	0:01:26	93.2589485	70.8813582		88.421441	75.1725398
20:04:16	0:01:27	93.9256152	70.8813582		89.421441	76.1725398

20:04:17	0:01:28	93.4256152	71.8813582	89.421441	75.1725398
20:04:18	0:01:29	93.7589485	73.8813582	89.421441	75.1725398
20:04:19	0:01:30	94.2589485	74.8813582	91.421441	75.1725398
20:04:20	0:01:31	93.4256152	74.8813582	91.421441	73.1725398
20:04:21	0:01:32	93.5922819	75.8813582	91.421441	73.1725398
20:04:22	0:01:33	92.7589485	75.8813582	91.421441	73.1725398
20:04:23	0:01:34	92.4256152	75.8813582	90.421441	73.1725398
20:04:24	0:01:35	92.7589485	77.8813582	90.421441	73.1725398
20:04:25	0:01:36	92.2589485	77.8813582	90.421441	73.1725398
20:04:26	0:01:37	92.5922819	77.8813582	90.421441	74.1725398
20:04:27	0:01:38	93.5922819	76.8813582	92.421441	74.1725398
20:04:28	0:01:39	93.9256152	76.8813582	92.421441	74.1725398
20:04:29	0:01:40	93.2589485	74.8813582	91.421441	74.1725398
20:04:30	0:01:41	92.7589485	73.8813582	91.421441	73.1725398
20:04:31	0:01:42	92.4256152	73.8813582	91.421441	72.1725398
20:04:32	0:01:43	92.2589485	72.8813582	91.421441	72.1725398
20:04:33	0:01:44	92.0922819	71.8813582	90.421441	72.1725398
20:04:34	0:01:45	92.0922819	71.8813582	90.421441	72.1725398
20:04:35	0:01:46	92.4256152	71.8813582	90.421441	73.1725398
20:04:36	0:01:47	92.7589485	71.8813582	91.421441	73.1725398
20:04:37	0:01:48	93.2589485	71.8813582	92.421441	73.1725398
20:04:38	0:01:49	93.0922819	70.8813582	92.421441	73.1725398
20:04:39	0:01:50	93.4256152	70.8813582	93.421441	73.1725398
20:04:40	0:01:51	93.9256152	70.8813582	94.421441	73.1725398
20:04:41	0:01:52	93.5922819	70.8813582	94.421441	71.1725398
20:04:42	0:01:53	93.2589485	70.8813582	93.421441	71.1725398
20:04:43	0:01:54	93.4256152	70.8813582	93.421441	71.1725398
20:04:44	0:01:55	92.7589485	70.8813582	92.421441	70.1725398
20:04:45	0:01:56	91.9256152	69.8813582	91.421441	69.1725398
20:04:46	0:01:57	91.0922819	68.8813582	89.421441	69.1725398
20:04:47	0:01:58	90.7589485	68.8813582	88.421441	69.1725398
20:04:48	0:01:59	90.5922819	67.8813582	88.421441	69.1725398
20:04:49	0:02:00	89.9256152	67.8813582	86.421441	69.1725398
20:04:50	0:02:01	89.5922819	67.8813582	85.421441	69.1725398
20:04:51	0:02:02	89.9256152	68.8813582	85.421441	70.1725398
20:04:52	0:02:03	89.5922819	68.8813582	83.421441	71.1725398
20:04:53	0:02:04	89.0922819	68.8813582	83.421441	71.1725398
20:04:54	0:02:05	89.7589485	68.8813582	83.421441	73.1725398
20:04:55	0:02:06	89.9256152	68.8813582	83.421441	74.1725398
20:04:56	0:02:07	89.9256152	68.8813582	83.421441	74.1725398
20:04:57	0:02:08	89.5922819	68.8813582	82.421441	74.1725398
20:04:58	0:02:09	89.7589485	68.8813582	82.421441	75.1725398
20:04:59	0:02:10	89.5922819	68.8813582	82.421441	75.1725398
20:05:00	0:02:11	88.9256152	68.8813582	80.421441	75.1725398
20:05:01	0:02:12	89.0922819	70.8813582	80.421441	75.1725398

20:05:02	0:02:13	91.2589485	71.8813582		82.421441	79.1725398
20:05:03	0:02:14	91.2589485	71.8813582		82.421441	79.1725398
20:05:04	0:02:15	91.0922819	72.8813582		81.421441	79.1725398
20:05:05	0:02:16	92.5922819	71.8813582		81.421441	84.1725398
20:05:06	0:02:17	92.5922819	71.8813582		81.421441	84.1725398
20:05:07	0:02:18	92.5922819	71.8813582		81.421441	84.1725398
20:05:08	0:02:19	92.9256152	72.8813582		81.421441	84.1725398
20:05:09	0:02:20	93.5922819	73.8813582		81.421441	85.1725398
20:05:10	0:02:21	94.9256152	73.8813582		82.421441	88.1725398
20:05:11	0:02:22	96.9256152	75.8813582		85.421441	90.1725398
20:05:12	0:02:23	97.0922819	75.8813582		85.421441	91.1725398
20:05:13	0:02:24	97.0922819	75.8813582		85.421441	91.1725398
20:05:14	0:02:25	97.5922819	76.8813582		86.421441	91.1725398
20:05:15	0:02:26	98.0922819	77.8813582		87.421441	91.1725398
20:05:16	0:02:27	97.7589485	77.8813582		87.421441	90.1725398
20:05:17	0:02:28	97.0922819	77.8813582		88.421441	87.1725398
20:05:18	0:02:29	97.5922819	78.8813582		89.421441	87.1725398
20:05:19	0:02:30	97.5922819	78.8813582		89.421441	87.1725398
20:05:20	0:02:31	97.7589485	79.8813582		89.421441	87.1725398
20:05:21	0:02:32	96.5922819	80.8813582		89.421441	83.1725398
20:05:22	0:02:33	96.5922819	80.8813582		89.421441	83.1725398
20:05:23	0:02:34	96.4256152	77.8813582		90.421441	83.1725398
20:05:24	0:02:35	95.7589485	77.8813582		90.421441	81.1725398
20:05:25	0:02:36	94.7589485	76.8813582		90.421441	79.1725398
20:05:26	0:02:37	94.9256152	76.8813582		90.421441	79.1725398
20:05:27	0:02:38	94.4256152	74.8813582		89.421441	79.1725398
20:05:28	0:02:39	93.7589485	74.8813582		87.421441	79.1725398
20:05:29	0:02:40	93.9256152	74.8813582		87.421441	79.1725398
20:05:30	0:02:41	93.9256152	74.8813582		87.421441	79.1725398
20:05:31	0:02:42	93.5922819	73.8813582		87.421441	79.1725398
20:05:32	0:02:43	92.9256152	73.8813582		87.421441	77.1725398
20:05:33	0:02:44	93.2589485	77.8813582		87.421441	76.1725398
20:05:34	0:02:45	90.7589485	77.8813582		87.421441	69.1725398
20:05:35	0:02:46	90.0922819	77.8813582		87.421441	67.1725398
20:05:36	0:02:47	90.0922819	77.8813582		87.421441	67.1725398
20:05:37	0:02:48	89.9256152	80.8813582		87.421441	66.1725398
20:05:38	0:02:49	89.7589485	78.8813582		88.421441	66.1725398
20:05:39	0:02:50	90.2589485	78.8813582		89.421441	67.1725398
20:05:40	0:02:51	90.9256152	78.8813582		91.421441	67.1725398
20:05:41	0:02:52	90.5922819	78.8813582		91.421441	66.1725398
20:05:42	0:02:53	90.9256152	78.8813582		92.421441	66.1725398
20:05:43	0:02:54	91.2589485	78.8813582		93.421441	66.1725398
20:05:44	0:02:55	92.9256152	80.8813582		97.421441	66.1725398
20:05:45	0:02:56	91.7589485	81.8813582		97.421441	62.1725398
20:05:46	0:02:57	91.7589485	81.8813582		97.421441	62.1725398

20:05:47	0:02:58	90.9256152	81.8813582		97.421441	60.1725398
20:05:48	0:02:59	91.2842889	81.8813582		96.421441	60.1725398
20:05:49	0:03:00	78.2842889	78.8813582		93.421441	60.1725398
20:05:50	0:03:01	73.2842889	76.8813582		92.421441	60.1725398
20:05:51	0:03:02	70.2842889	76.8813582		92.421441	60.1725398
20:05:52	0:03:03	68.2842889	76.8813582		92.421441	59.1725398
20:05:53	0:03:04	68.2842889	76.8813582		92.421441	60.1725398
20:05:54	0:03:05	67.2842889	75.8813582		92.421441	60.1725398
20:05:55	0:03:06	67.2842889	75.8813582		91.421441	60.1725398
20:05:56	0:03:07	66.2842889	74.8813582		91.421441	60.1725398
20:05:57	0:03:08	66.2842889	74.8813582		92.421441	60.1725398
20:05:58	0:03:09	66.2842889	74.8813582		91.421441	60.1725398
20:05:59	0:03:10	65.2842889	73.8813582		91.421441	60.1725398
20:06:00	0:03:11	65.2842889	73.8813582		91.421441	60.1725398
20:06:01	0:03:12	65.2842889	73.8813582		91.421441	59.1725398
20:06:02	0:03:13	65.2842889	73.8813582		91.421441	59.1725398
20:06:03	0:03:14	64.2842889	73.8813582		89.421441	59.1725398
20:06:04	0:03:15	64.2842889	71.8813582		86.421441	59.1725398
20:06:05	0:03:16	63.2842889	70.8813582		86.421441	59.1725398
20:06:06	0:03:17	64.2842889	70.8813582		86.421441	59.1725398
20:06:07	0:03:18	64.2842889	69.8813582		82.421441	59.1725398
20:06:08	0:03:19	65.2842889	67.8813582		81.421441	59.1725398
20:06:09	0:03:20	65.2842889	67.8813582		81.421441	59.1725398
20:06:10	0:03:21	65.2842889	67.8813582		82.421441	58.1725398
20:06:11	0:03:22	65.2842889	67.8813582		83.421441	58.1725398
20:06:12	0:03:23	65.2842889	67.8813582		84.421441	58.1725398
20:06:13	0:03:24	65.2842889	67.8813582		84.421441	58.1725398
20:06:14	0:03:25	65.2842889	67.8813582		84.421441	58.1725398
20:06:15	0:03:26	65.2842889	67.8813582		83.421441	59.1725398
20:06:16	0:03:27	65.2842889	68.8813582		82.421441	59.1725398
20:06:17	0:03:28	65.2842889	68.8813582		82.421441	59.1725398
20:06:18	0:03:29	65.2842889	70.8813582		82.421441	59.1725398
20:06:19	0:03:30	65.2842889	70.8813582		82.421441	59.1725398
20:06:20	0:03:31	64.2842889	71.8813582		82.421441	59.1725398
20:06:21	0:03:32	65.2842889	71.8813582		81.421441	60.1725398
20:06:22	0:03:33	66.2842889	73.8813582		82.421441	60.1725398
20:06:23	0:03:34	66.2842889	75.8813582		83.421441	61.1725398
20:06:24	0:03:35	66.2842889	79.8813582		84.421441	64.1725398
20:06:25	0:03:36	63.2842889	79.8813582		84.421441	64.1725398
20:06:26	0:03:37	63.2842889	79.8813582		85.421441	64.1725398
20:06:27	0:03:38	62.2842889	76.8813582		86.421441	64.1725398
20:06:28	0:03:39	63.2842889	71.8813582		86.421441	64.1725398
20:06:29	0:03:40	63.2842889	69.8813582		87.421441	64.1725398
20:06:30	0:03:41	63.2842889	69.8813582		87.421441	65.1725398
20:06:31	0:03:42	63.2842889	67.8813582		88.421441	65.1725398

20:06:32	0:03:43	64.2842889	67.8813582		89.421441	65.1725398
20:06:33	0:03:44	65.2842889	67.8813582		89.421441	65.1725398
20:06:34	0:03:45	65.2842889	67.8813582		89.421441	66.1725398
20:06:35	0:03:46	67.2842889	67.8813582		89.421441	66.1725398
20:06:36	0:03:47	68.2842889	67.8813582		88.421441	66.1725398
20:06:37	0:03:48	68.2842889	67.8813582		88.421441	67.1725398
20:06:38	0:03:49	68.2842889	67.8813582		86.421441	67.1725398
20:06:39	0:03:50	68.2842889	67.8813582		85.421441	67.1725398
20:06:40	0:03:51	69.2842889	68.8813582		85.421441	68.1725398
20:06:41	0:03:52	70.2842889	69.8813582	75.846326	85.421441	68.1725398
20:06:42	0:03:53	70.2842889	70.8813582	75.846326	84.421441	69.1725398
20:06:43	0:03:54	70.2842889	70.8813582	74.846326	86.421441	70.1725398
20:06:44	0:03:55	71.2842889	70.8813582	76.846326	86.421441	69.1725398
20:06:45	0:03:56	71.2842889	71.8813582	75.846326	87.421441	68.1725398
20:06:46	0:03:57	71.2842889	71.8813582	74.846326	87.421441	68.1725398
20:06:47	0:03:58	71.2842889	71.8813582	74.846326	88.421441	68.1725398
20:06:48	0:03:59	71.2842889	70.8813582	74.846326	89.421441	68.1725398
20:06:49	0:04:00	69.2842889	69.8813582	74.846326	90.421441	68.1725398
20:06:50	0:04:01	66.2842889	69.8813582	74.846326	90.421441	68.1725398
20:06:51	0:04:02	65.2842889	69.8813582	74.846326	92.421441	78.1725398
20:06:52	0:04:03	64.2842889	69.8813582	73.846326	93.421441	95.1725398
20:06:53	0:04:04	63.2842889	70.8813582	73.846326	94.421441	95.1725398
20:06:54	0:04:05	63.2842889	70.8813582	73.846326	94.421441	95.1725398
20:06:55	0:04:06	63.2842889	70.8813582	73.846326	96.421441	100.17254
20:06:56	0:04:07	63.2842889	70.8813582	72.846326	96.421441	100.17254
20:06:57	0:04:08	63.2842889	71.8813582	71.846326	96.421441	100.17254
20:06:58	0:04:09	63.2842889	70.8813582	71.846326	96.421441	101.17254
20:06:59	0:04:10	62.2842889	69.8813582	71.846326	96.421441	101.17254
20:07:00	0:04:11	62.2842889	69.8813582	71.846326	96.421441	101.17254
20:07:01	0:04:12	62.2842889	70.8813582	72.846326	96.421441	102.17254
20:07:02	0:04:13	61.2842889	69.8813582	72.846326	95.421441	102.17254
20:07:03	0:04:14	61.2842889	70.8813582	72.846326	95.421441	102.17254
20:07:04	0:04:15	61.2842889	70.8813582	72.846326	87.421441	103.17254
20:07:05	0:04:16	61.2842889	70.8813582	73.846326	87.421441	103.17254
20:07:06	0:04:17	61.2842889	72.8813582	74.846326	86.421441	103.17254
20:07:07	0:04:18	61.2842889	73.8813582	78.846326	86.421441	103.17254
20:07:08	0:04:19	61.2842889	74.8813582	78.846326	86.421441	103.17254
20:07:09	0:04:20	61.2842889	74.8813582	78.846326	86.421441	104.17254
20:07:10	0:04:21	61.2842889	75.8813582	77.846326	84.421441	105.17254
20:07:11	0:04:22	61.2842889	75.8813582	77.846326	84.421441	105.17254
20:07:12	0:04:23	61.2842889	78.8813582	75.846326	82.421441	106.17254
20:07:13	0:04:24	61.2842889	79.8813582	74.846326	80.421441	106.17254
20:07:14	0:04:25	61.2842889	79.8813582	74.846326	69.421441	81.3428596
20:07:15	0:04:26	61.2842889	79.8813582	74.846326	69.421441	81.3428596
20:07:16	0:04:27	61.2842889	82.8813582	74.846326	69.421441	81.771431

20:07:17	0:04:28	61.2842889	82.8813582	74.846326	68.421441	104.17254
20:07:18	0:04:29	61.2842889	82.8813582	76.846326	68.421441	104.17254
20:07:19	0:04:30	61.2842889	82.8813582	77.846326	68.421441	104.17254
20:07:20	0:04:31	61.2842889	82.8813582	76.846326	68.421441	87.1725398
20:07:21	0:04:32	61.2842889	83.8813582	76.846326	68.421441	84.1725398
20:07:22	0:04:33	61.2842889	83.8813582	75.846326	68.421441	82.1725398
20:07:23	0:04:34	61.2842889	88.8813582	73.846326	66.421441	82.1725398
20:07:24	0:04:35	61.2842889	93.8813582	72.846326	66.421441	80.1725398
20:07:25	0:04:36	62.2842889	95.8813582	72.846326	64.421441	80.1725398
20:07:26	0:04:37	62.2842889	95.8813582	72.846326	65.421441	80.1725398
20:07:27	0:04:38	62.2842889	95.8813582	74.846326	66.421441	80.1725398
20:07:28	0:04:39	63.2842889	99.8813582	77.846326	66.421441	83.1725398
20:07:29	0:04:40	63.2842889	100.881358	77.846326	67.421441	84.1725398
20:07:30	0:04:41	62.2842889	100.881358	77.846326	70.421441	85.1725398
20:07:31	0:04:42	62.2842889	88.8813582	77.846326	69.421441	85.1725398
20:07:32	0:04:43	62.2842889	80.8813582	77.846326	69.421441	86.1725398
20:07:33	0:04:44	63.2842889	77.8813582	77.846326	69.421441	86.1725398
20:07:34	0:04:45	64.2842889	77.8813582	76.846326	69.421441	87.1725398
20:07:35	0:04:46	64.2842889	73.8813582	76.846326	82.421441	87.1725398
20:07:36	0:04:47	64.2842889	72.8813582	79.846326	86.421441	86.1725398
20:07:37	0:04:48	64.2842889	72.8813582	79.846326	86.421441	84.1725398
20:07:38	0:04:49	64.2842889	70.8813582	86.846326	86.421441	83.1725398
20:07:39	0:04:50	64.2842889	70.8813582	87.846326	87.421441	83.1725398
20:07:40	0:04:51	64.2842889	70.8813582	87.846326	87.421441	83.1725398
20:07:41	0:04:52	64.2842889	69.8813582	87.846326	86.421441	83.1725398
20:07:42	0:04:53	65.2842889	69.8813582	88.846326	86.421441	83.1725398
20:07:43	0:04:54	64.2842889	69.8813582	88.846326	85.421441	83.1725398
20:07:44	0:04:55	64.2842889	69.8813582	88.846326	85.421441	82.1725398
20:07:45	0:04:56	64.2842889	69.8813582	89.846326	86.421441	84.1725398
20:07:46	0:04:57	64.2842889	69.8813582	89.846326	86.421441	84.1725398
20:07:47	0:04:58	64.2842889	69.8813582	91.846326	86.421441	83.1725398
20:07:48	0:04:59	62.2842889	69.8813582	91.846326	86.421441	83.1725398
20:07:49	0:05:00	62.2842889	69.8813582	93.846326	86.421441	80.1725398
20:07:50	0:05:01	62.2842889	67.8813582	93.846326	81.421441	80.1725398
20:07:51	0:05:02	62.2842889	67.8813582	94.846326	81.421441	80.1725398
20:07:52	0:05:03	62.2842889	67.8813582	94.846326	80.421441	80.1725398
20:07:53	0:05:04	61.2842889	67.8813582	95.846326	79.421441	79.1725398
20:07:54	0:05:05	61.2842889	68.8813582	95.846326	79.421441	78.1725398
20:07:55	0:05:06	61.2842889	68.8813582	95.846326	85.421441	78.1725398
20:07:56	0:05:07	61.2842889	68.8813582	95.846326	88.421441	78.1725398
20:07:57	0:05:08	61.2842889	68.8813582	94.846326	92.421441	79.1725398
20:07:58	0:05:09	61.2842889	68.8813582	94.846326	93.421441	78.1725398
20:07:59	0:05:10	61.2842889	68.8813582	94.846326	94.421441	78.1725398
20:08:00	0:05:11	61.2842889	68.8813582	94.846326	94.421441	79.1725398
20:08:01	0:05:12	61.2842889	69.8813582	94.846326	94.421441	79.1725398

20:08:02	0:05:13	61.2842889	69.8813582	90.846326	93.421441	79.1725398
20:08:03	0:05:14	62.2842889	68.8813582	90.846326	90.421441	79.1725398
20:08:04	0:05:15	62.2842889	68.8813582	90.846326	87.421441	79.1725398
20:08:05	0:05:16	61.2842889	68.8813582	90.846326	86.421441	80.1725398
20:08:06	0:05:17	61.2842889	68.8813582	90.846326	86.421441	80.1725398
20:08:07	0:05:18	61.2842889	68.8813582	88.846326	83.421441	80.1725398
20:08:08	0:05:19	61.2842889	69.8813582	87.846326	82.421441	80.1725398
20:08:09	0:05:20	61.2842889	70.8813582	87.846326	83.421441	78.1725398
20:08:10	0:05:21	61.2842889	70.8813582	86.846326	83.421441	78.1725398
20:08:11	0:05:22	61.2842889	72.8813582	85.846326	83.421441	77.1725398
20:08:12	0:05:23	61.2842889	72.8813582	84.846326	81.421441	77.1725398
20:08:13	0:05:24	61.2842889	74.8813582	84.846326	80.421441	78.1725398
20:08:14	0:05:25	60.2842889	75.8813582	80.846326	80.421441	78.1725398
20:08:15	0:05:26	60.2842889	75.8813582	79.846326	79.421441	78.1725398
20:08:16	0:05:27	61.2842889	74.8813582	79.846326	79.421441	77.1725398
20:08:17	0:05:28	61.2842889	74.8813582	79.846326	78.421441	77.1725398
20:08:18	0:05:29	61.2842889	74.8813582	83.846326	78.421441	76.1725398
20:08:19	0:05:30	62.2842889	74.8813582	83.846326	79.421441	75.1725398
20:08:20	0:05:31	62.2842889	73.8813582	83.846326	79.421441	75.1725398
20:08:21	0:05:32	62.2842889	73.8813582	83.846326	79.421441	75.1725398
20:08:22	0:05:33	61.2842889	72.8813582	83.846326	81.421441	75.1725398
20:08:23	0:05:34	61.2842889	72.8813582	84.846326	80.421441	75.1725398
20:08:24	0:05:35	60.2842889	72.8813582	84.846326	80.421441	75.1725398
20:08:25	0:05:36	60.2842889	76.8813582	85.846326	81.421441	75.1725398
20:08:26	0:05:37	61.2842889	78.8813582	87.846326	82.421441	73.1725398
20:08:27	0:05:38	61.2842889	78.8813582	87.846326	83.421441	73.1725398
20:08:28	0:05:39	61.2842889	78.8813582	86.846326	83.421441	73.1725398
20:08:29	0:05:40	61.2842889	79.8813582	86.846326	84.421441	73.1725398
20:08:30	0:05:41	61.2842889	80.8813582	85.846326	85.421441	75.1725398
20:08:31	0:05:42	61.2842889	81.8813582	82.846326	87.421441	78.1725398
20:08:32	0:05:43	61.2842889	81.8813582	82.846326	87.421441	79.1725398
20:08:33	0:05:44	61.2842889	81.8813582	81.846326	88.421441	79.1725398
20:08:34	0:05:45	62.2842889	81.8813582	78.846326	88.421441	77.1725398
20:08:35	0:05:46	62.2842889	81.8813582	78.846326	91.421441	74.1725398
20:08:36	0:05:47	62.2842889	81.8813582	78.846326	91.421441	74.1725398
20:08:37	0:05:48	62.2842889	81.8813582	78.846326	91.421441	74.1725398
20:08:38	0:05:49	62.2842889	81.8813582	80.846326	92.421441	77.1725398
20:08:39	0:05:50	62.2842889	81.8813582	83.846326	93.421441	80.1725398
20:08:40	0:05:51	62.2842889	81.8813582	83.846326	93.421441	80.1725398
20:08:41	0:05:52	62.2842889	78.8813582	83.846326	94.421441	80.1725398
20:08:42	0:05:53	61.2842889	78.8813582	83.846326	94.421441	80.1725398
20:08:43	0:05:54	61.2842889	75.8813582	82.846326	95.421441	78.1725398
20:08:44	0:05:55	61.2842889	74.8813582	79.846326	95.421441	78.1725398
20:08:45	0:05:56	61.2842889	74.8813582	79.846326	92.421441	76.1725398
20:08:46	0:05:57	61.2842889	73.8813582	79.846326	90.421441	76.1725398

20:08:47	0:05:58	61.2842889	73.8813582	79.846326	83.421441	74.1725398
20:08:48	0:05:59	61.2842889	73.8813582	79.846326	83.421441	74.1725398
20:08:49	0:06:00	60.2842889	73.8813582	77.846326	82.421441	73.1725398
20:08:50	0:06:01	60.2842889	73.8813582	76.846326	81.421441	73.1725398
20:08:51	0:06:02	60.2842889	74.8813582	76.846326	80.421441	73.1725398
20:08:52	0:06:03	60.2842889	74.8813582	76.846326	80.421441	74.1725398
20:08:53	0:06:04	60.2842889	75.8813582	76.846326	84.421441	75.1725398
20:08:54	0:06:05	60.2842889	75.8813582	76.846326	85.421441	74.1725398
20:08:55	0:06:06	60.2842889	76.8813582	76.846326	85.421441	74.1725398
20:08:56	0:06:07	60.2842889	76.8813582	77.846326	85.421441	72.1725398
20:08:57	0:06:08	61.2842889	76.8813582	77.846326	85.421441	72.1725398
20:08:58	0:06:09	61.2842889	76.8813582	78.846326	85.421441	72.1725398
20:08:59	0:06:10	61.2842889	77.8813582	78.846326	85.421441	73.1725398
20:09:00	0:06:11	61.2842889	78.8813582	77.846326	84.421441	73.1725398
20:09:01	0:06:12	61.2842889	79.8813582	76.846326	84.421441	73.1725398
20:09:02	0:06:13	61.2842889	83.8813582	76.846326	82.421441	73.1725398
20:09:03	0:06:14	61.2842889	83.8813582	76.846326	82.421441	74.1725398
20:09:04	0:06:15	61.2842889	85.8813582	75.846326	82.421441	74.1725398
20:09:05	0:06:16	62.2842889	86.8813582	75.846326	82.421441	74.1725398
20:09:06	0:06:17	62.2842889	83.8813582	73.846326	82.421441	76.1725398
20:09:07	0:06:18	64.2842889	77.8813582	72.846326	82.421441	74.1725398
20:09:08	0:06:19	66.2842889	74.8813582	72.846326	82.421441	73.1725398
20:09:09	0:06:20	67.2842889	72.8813582	72.846326	83.421441	73.1725398
20:09:10	0:06:21	62.2842889	71.8813582	71.846326	84.421441	73.1725398
20:09:11	0:06:22	61.2842889	71.8813582	71.846326	86.421441	73.1725398
20:09:12	0:06:23	61.2842889	70.8813582	71.846326	85.421441	74.1725398
20:09:13	0:06:24	61.2842889	70.8813582	69.846326	84.421441	74.1725398
20:09:14	0:06:25	61.2842889	69.8813582	67.846326	84.421441	73.1725398
20:09:15	0:06:26	60.2842889	68.8813582	65.846326	82.421441	73.1725398
20:09:16	0:06:27	61.2842889	67.8813582	65.846326	82.421441	73.1725398
20:09:17	0:06:28	61.2842889	67.8813582	68.846326	81.421441	73.1725398
20:09:18	0:06:29	61.2842889	68.8813582	69.846326	81.421441	73.1725398
20:09:19	0:06:30	61.2842889	68.8813582	69.846326	79.421441	75.1725398
20:09:20	0:06:31	60.2842889	67.8813582	70.846326	79.421441	75.1725398
20:09:21	0:06:32	60.2842889	67.8813582	70.846326	78.421441	75.1725398
20:09:22	0:06:33	60.2842889	67.8813582	70.846326	78.421441	75.1725398
20:09:23	0:06:34	60.2842889	66.8813582	70.846326	78.421441	75.1725398
20:09:24	0:06:35	60.2842889	66.8813582	71.846326	76.421441	75.1725398
20:09:25	0:06:36	59.2842889	66.8813582	72.846326	76.421441	73.1725398
20:09:26	0:06:37	58.2842889	66.8813582	72.846326	75.421441	72.1725398
20:09:27	0:06:38	58.2842889	66.8813582	75.846326	75.421441	74.1725398
20:09:28	0:06:39	59.2842889	66.8813582	76.846326	76.421441	76.1725398
20:09:29	0:06:40	63.2842889	67.8813582	76.846326	76.421441	77.1725398
20:09:30	0:06:41	63.2842889	67.8813582	76.846326	77.421441	77.1725398
20:09:31	0:06:42	65.2842889	67.8813582	76.846326	77.421441	77.1725398

20:09:32	0:06:43	65.2842889	67.8813582	74.846326	78.421441	78.1725398
20:09:33	0:06:44	65.2842889	67.8813582	73.846326	78.421441	79.1725398
20:09:34	0:06:45	64.2842889	68.8813582	72.846326	78.421441	79.1725398
20:09:35	0:06:46	64.2842889	68.8813582	69.846326	78.421441	79.1725398
20:09:36	0:06:47	64.2842889	68.8813582	69.846326	78.421441	79.1725398
20:09:37	0:06:48	64.2842889	68.8813582	76.846326	79.421441	80.1725398
20:09:38	0:06:49	63.2842889	69.8813582	79.846326	79.421441	80.1725398
20:09:39	0:06:50	62.2842889	69.8813582	79.846326	79.421441	80.1725398
20:09:40	0:06:51	62.2842889	69.8813582	80.846326	78.421441	82.1725398
20:09:41	0:06:52	61.2842889	69.8813582	80.846326	78.421441	81.1725398
20:09:42	0:06:53	60.2842889	70.8813582	80.846326	77.421441	77.1725398
20:09:43	0:06:54	60.2842889	70.8813582	80.846326	77.421441	74.1725398
20:09:44	0:06:55	60.2842889	69.8813582	80.846326	77.421441	73.1725398
20:09:45	0:06:56	60.2842889	69.8813582	80.846326	77.421441	73.1725398
20:09:46	0:06:57	60.2842889	69.8813582	79.846326	78.421441	72.1725398
20:09:47	0:06:58	60.2842889	69.8813582	79.846326	78.421441	72.1725398
20:09:48	0:06:59	60.2842889	68.8813582	79.846326	78.421441	72.1725398
20:09:49	0:07:00	60.2842889	68.8813582	79.846326	78.421441	72.1725398
20:09:50	0:07:01	61.2842889	68.8813582	79.846326	78.421441	72.1725398
20:09:51	0:07:02	61.2842889	68.8813582	78.846326	78.421441	70.1725398
20:09:52	0:07:03	61.2842889	69.8813582	78.846326	78.421441	70.1725398
20:09:53	0:07:04	61.2842889	69.8813582	78.846326	78.421441	69.1725398
20:09:54	0:07:05	60.2842889	69.8813582	79.846326	78.421441	68.1725398
20:09:55	0:07:06	60.2842889	69.8813582	79.846326	76.421441	68.1725398
20:09:56	0:07:07	60.2842889	69.8813582	79.846326	75.421441	68.1725398
20:09:57	0:07:08	61.2842889	70.8813582	82.846326	75.421441	69.1725398
20:09:58	0:07:09	60.2842889	75.8813582	82.846326	75.421441	70.1725398
20:09:59	0:07:10	60.2842889	75.8813582	85.846326	75.421441	71.1725398
20:10:00	0:07:11	59.2842889	75.8813582	85.846326	74.421441	71.1725398
20:10:01	0:07:12	59.2842889	82.8813582	86.846326	74.421441	71.1725398
20:10:02	0:07:13	59.2842889	83.8813582	84.846326	74.421441	71.1725398
20:10:03	0:07:14	58.2842889	83.8813582	84.846326	74.421441	72.1725398
20:10:04	0:07:15	58.2842889	87.8813582	84.846326	73.421441	72.1725398
20:10:05	0:07:16	58.2842889	91.8813582	79.846326	74.421441	72.1725398
20:10:06	0:07:17	58.2842889	100.881358	79.846326	79.421441	72.1725398
20:10:07	0:07:18	58.2842889	78.2000025	78.846326	81.421441	72.1725398
20:10:08	0:07:19	57.2842889	77.771431	78.846326	81.421441	71.1725398
20:10:09	0:07:20	57.2842889	79.6285739	78.846326	84.421441	71.1725398
20:10:10	0:07:21	57.2842889	79.6285739	78.846326	84.421441	71.1725398
20:10:11	0:07:22	57.2842889	79.6285739	78.846326	84.421441	71.1725398
20:10:12	0:07:23	57.2842889	79.6285739	78.846326	84.421441	71.1725398
20:10:13	0:07:24	60.2842889	80.3428596	78.846326	84.421441	71.1725398
20:10:14	0:07:25	61.2842889	81.3428596	78.846326	86.421441	72.1725398
20:10:15	0:07:26	61.2842889	81.6285739	78.846326	86.421441	73.1725398
20:10:16	0:07:27	61.2842889	100.881358	77.846326	86.421441	73.1725398

20:10:17	0:07:28	61.2842889	96.8813582	76.846326	86.421441	75.1725398
20:10:18	0:07:29	61.2842889	94.8813582	76.846326	86.421441	75.1725398
20:10:19	0:07:30	61.2842889	93.8813582	77.846326	86.421441	75.1725398
20:10:20	0:07:31	61.2842889	91.8813582	77.846326	86.421441	76.1725398
20:10:21	0:07:32	61.2842889	91.8813582	77.846326	85.421441	76.1725398
20:10:22	0:07:33	62.2842889	89.8813582	77.846326	85.421441	77.1725398
20:10:23	0:07:34	62.2842889	81.8813582	77.846326	83.421441	78.1725398
20:10:24	0:07:35	62.2842889	76.8813582	77.846326	83.421441	78.1725398
20:10:25	0:07:36	62.2842889	73.8813582	78.846326	85.421441	73.1725398
20:10:26	0:07:37	62.2842889	71.8813582	75.846326	84.421441	71.1725398
20:10:27	0:07:38	62.2842889	70.8813582	75.846326	83.421441	71.1725398
20:10:28	0:07:39	62.2842889	70.8813582	75.846326	83.421441	70.1725398
20:10:29	0:07:40	61.2842889	68.8813582	75.846326	81.421441	69.1725398
20:10:30	0:07:41	61.2842889	68.8813582	75.846326	81.421441	69.1725398
20:10:31	0:07:42	62.2842889	68.8813582	75.846326	82.421441	69.1725398
20:10:32	0:07:43	61.2842889	67.8813582	76.846326	82.421441	71.1725398
20:10:33	0:07:44	61.2842889	67.8813582	76.846326	88.421441	71.1725398
20:10:34	0:07:45	61.2842889	67.8813582	74.846326	91.421441	72.1725398
20:10:35	0:07:46	61.2842889	67.8813582	74.846326	93.421441	72.1725398
20:10:36	0:07:47	61.2842889	67.8813582	74.846326	96.421441	73.1725398
20:10:37	0:07:48	61.2842889	67.8813582	74.846326	96.421441	74.1725398
20:10:38	0:07:49	62.2842889	67.8813582	74.846326	99.421441	74.1725398
20:10:39	0:07:50	62.2842889	67.8813582	74.846326	103.421441	75.1725398
20:10:40	0:07:51	62.2842889	67.8813582	74.846326	81.771431	75.1725398
20:10:41	0:07:52	62.2842889	67.8813582	76.846326	82.6285739	75.1725398
20:10:42	0:07:53	62.2842889	67.8813582	76.846326	82.9142882	76.1725398
20:10:43	0:07:54	62.2842889	67.8813582	76.846326	83.4857167	77.1725398
20:10:44	0:07:55	62.2842889	68.8813582	75.846326	83.771431	78.1725398
20:10:45	0:07:56	62.2842889	68.8813582	75.846326	83.771431	78.1725398
20:10:46	0:07:57	62.2842889	68.8813582	75.846326	84.6285739	77.1725398
20:10:47	0:07:58	62.2842889	67.8813582	75.846326	83.9142882	75.1725398
20:10:48	0:07:59	62.2842889	67.8813582	75.846326	84.4857167	75.1725398
20:10:49	0:08:00	62.2842889	67.8813582	77.846326	85.0571453	76.1725398
20:10:50	0:08:01	62.2842889	67.8813582	79.846326	85.6285739	77.1725398
20:10:51	0:08:02	62.2842889	67.8813582	79.846326	85.3428596	76.1725398
20:10:52	0:08:03	63.2842889	67.8813582	79.846326	85.2000025	76.1725398
20:10:53	0:08:04	63.2842889	67.8813582	77.846326	84.6285739	75.1725398
20:10:54	0:08:05	63.2842889	67.8813582	77.846326	84.6285739	75.1725398
20:10:55	0:08:06	63.2842889	67.8813582	77.846326	84.3428596	75.1725398
20:10:56	0:08:07	63.2842889	68.8813582	77.846326	84.4857167	75.1725398
20:10:57	0:08:08	63.2842889	68.8813582	77.846326	84.771431	76.1725398
20:10:58	0:08:09	65.2842889	68.8813582	77.846326	84.771431	76.1725398
20:10:59	0:08:10	65.2842889	68.8813582	76.846326	84.0571453	75.1725398
20:11:00	0:08:11	67.2842889	70.8813582	75.846326	83.9142882	73.1725398
20:11:01	0:08:12	70.2842889	72.8813582	74.846326	83.9142882	72.1725398

20:11:02	0:08:13	70.2842889	73.8813582	76.846326	84.0571453	72.1725398
20:11:03	0:08:14	70.2842889	73.8813582	76.846326	84.0571453	72.1725398
20:11:04	0:08:15	70.2842889	74.8813582	76.846326	85.3428596	73.1725398
20:11:05	0:08:16	70.2842889	75.8813582	74.846326	86.0571453	74.1725398
20:11:06	0:08:17	70.2842889	74.8813582	74.846326	87.0571453	74.1725398
20:11:07	0:08:18	71.2842889	72.8813582	73.846326	87.6285739	74.1725398
20:11:08	0:08:19		70.8813582	73.846326	87.771431	74.1725398
20:11:09	0:08:20		70.8813582	73.846326	87.771431	74.1725398
20:11:10	0:08:21		70.8813582	73.846326	87.771431	73.1725398
20:11:11	0:08:22		70.8813582	75.846326	89.0571453	73.1725398
20:11:12	0:08:23		70.8813582	77.846326	89.771431	73.1725398
20:11:13	0:08:24		70.8813582	79.846326	90.771431	73.1725398
20:11:14	0:08:25		70.8813582	79.846326	90.771431	73.1725398
20:11:15	0:08:26		69.8813582	79.846326	90.6285739	73.1725398
20:11:16	0:08:27		70.8813582	80.846326	92.4857167	76.1725398
20:11:17	0:08:28		70.8813582	80.846326	93.6285739	78.1725398
20:11:18	0:08:29		71.8813582	80.846326	93.9142882	77.1725398
20:11:19	0:08:30		71.8813582	80.846326	94.9142882	78.1725398
20:11:20	0:08:31		69.8813582	80.846326	95.4857167	79.1725398
20:11:21	0:08:32		69.8813582	82.846326	95.9142882	79.1725398
20:11:22	0:08:33		72.8813582	81.846326	96.4857167	79.1725398
20:11:23	0:08:34		72.8813582	81.846326	96.771431	79.1725398
20:11:24	0:08:35		72.8813582	81.846326	96.771431	79.1725398
20:11:25	0:08:36		72.8813582	80.846326	96.9142882	80.1725398
20:11:26	0:08:37		71.8813582	79.846326	96.771431	80.1725398
20:11:27	0:08:38		71.8813582	80.846326	97.4857167	83.1725398
20:11:28	0:08:39		70.8813582	80.846326	95.2000025	84.1725398
20:11:29	0:08:40		70.8813582	80.846326	95.771431	86.1725398
20:11:30	0:08:41		70.8813582	80.846326	95.771431	87.1725398
20:11:31	0:08:42		70.8813582	82.846326	96.0571453	87.1725398
20:11:32	0:08:43		70.8813582	83.846326	95.771431	86.1725398
20:11:33	0:08:44		70.8813582	81.846326	84.421441	83.1725398
20:11:34	0:08:45		70.8813582	80.846326	75.421441	82.1725398
20:11:35	0:08:46		70.8813582	80.846326	73.421441	82.1725398
20:11:36	0:08:47		71.8813582	79.846326	73.421441	81.1725398
20:11:37	0:08:48		70.8813582	79.846326	73.421441	80.1725398
20:11:38	0:08:49		70.8813582	79.846326	73.421441	78.1725398
20:11:39	0:08:50		69.8813582	79.846326	73.421441	77.1725398
20:11:40	0:08:51		67.8813582	78.846326	73.421441	77.1725398
20:11:41	0:08:52		67.8813582	77.846326	73.421441	77.1725398
20:11:42	0:08:53		67.8813582	77.846326	73.421441	77.1725398
20:11:43	0:08:54		66.8813582	77.846326	72.421441	77.1725398
20:11:44	0:08:55		66.8813582	76.846326	72.421441	77.1725398
20:11:45	0:08:56		66.8813582	76.846326	72.421441	77.1725398
20:11:46	0:08:57		67.8813582	76.846326	72.421441	77.1725398

20:11:47	0:08:58	67.8813582	77.846326	72.421441	78.1725398
20:11:48	0:08:59	67.8813582	77.846326	72.421441	79.1725398
20:11:49	0:09:00	68.8813582	77.846326	72.421441	79.1725398
20:11:50	0:09:01	69.8813582	76.846326	72.421441	78.1725398
20:11:51	0:09:02	69.8813582	76.846326	72.421441	75.1725398
20:11:52	0:09:03	71.8813582	76.846326	72.421441	76.1725398
20:11:53	0:09:04	71.8813582	79.846326	72.421441	77.1725398
20:11:54	0:09:05	70.8813582	80.846326	71.421441	77.1725398
20:11:55	0:09:06	69.8813582	80.846326	70.421441	77.1725398
20:11:56	0:09:07	69.8813582	80.846326	69.421441	77.1725398
20:11:57	0:09:08	69.8813582	80.846326	69.421441	77.1725398
20:11:58	0:09:09	68.8813582	80.846326	70.421441	76.1725398
20:11:59	0:09:10	68.8813582	79.846326	70.421441	75.1725398
20:12:00	0:09:11	69.8813582	79.846326	70.421441	74.1725398
20:12:01	0:09:12	69.8813582	79.846326	70.421441	73.1725398
20:12:02	0:09:13	70.8813582	80.846326	71.421441	73.1725398
20:12:03	0:09:14	70.8813582	79.846326	72.421441	73.1725398
20:12:04	0:09:15	69.8813582	79.846326	73.421441	73.1725398
20:12:05	0:09:16	69.8813582	79.846326	75.421441	73.1725398
20:12:06	0:09:17	69.8813582	78.846326	77.421441	74.1725398
20:12:07	0:09:18	70.8813582	78.846326	77.421441	74.1725398
20:12:08	0:09:19	69.8813582	77.846326	78.421441	74.1725398
20:12:09	0:09:20	69.8813582	78.846326	80.421441	75.1725398
20:12:10	0:09:21	69.8813582	78.846326	81.421441	74.1725398
20:12:11	0:09:22	69.8813582	78.846326	81.421441	74.1725398
20:12:12	0:09:23	69.8813582	78.846326	81.421441	72.1725398
20:12:13	0:09:24	69.8813582	78.846326	82.421441	73.1725398
20:12:14	0:09:25	69.8813582	79.846326	83.421441	75.1725398
20:12:15	0:09:26	69.8813582	80.846326	84.421441	75.1725398
20:12:16	0:09:27	69.8813582	80.846326	85.421441	76.1725398
20:12:17	0:09:28	69.8813582	81.846326	85.421441	75.1725398
20:12:18	0:09:29	69.8813582	81.846326	85.421441	75.1725398
20:12:19	0:09:30	70.8813582	80.846326	85.421441	75.1725398
20:12:20	0:09:31	69.8813582	80.846326	85.421441	74.1725398
20:12:21	0:09:32	69.8813582	79.846326	85.421441	72.1725398
20:12:22	0:09:33	69.8813582	78.846326	85.421441	72.1725398
20:12:23	0:09:34	69.8813582	78.846326	85.421441	72.1725398
20:12:24	0:09:35	69.8813582	78.846326	85.421441	73.1725398
20:12:25	0:09:36	68.8813582	78.846326	86.421441	74.1725398
20:12:26	0:09:37	68.8813582	78.846326	86.421441	74.1725398
20:12:27	0:09:38	68.8813582	76.846326	86.421441	74.1725398
20:12:28	0:09:39	68.8813582	75.846326	87.421441	74.1725398
20:12:29	0:09:40	68.8813582	75.846326	88.421441	74.1725398
20:12:30	0:09:41	68.8813582	74.846326	87.421441	74.1725398
20:12:31	0:09:42	68.8813582	72.846326	87.421441	74.1725398

20:12:32	0:09:43	66.8813582	71.846326	87.421441	73.1725398
20:12:33	0:09:44	66.8813582	71.846326	87.421441	74.1725398
20:12:34	0:09:45	67.8813582	73.846326	87.421441	74.1725398
20:12:35	0:09:46	69.8813582	73.846326	86.421441	75.1725398
20:12:36	0:09:47	71.8813582	73.846326	86.421441	74.1725398
20:12:37	0:09:48	71.8813582	75.846326	85.421441	74.1725398
20:12:38	0:09:49	72.8813582	75.846326	85.421441	72.1725398
20:12:39	0:09:50	72.8813582	75.846326	83.421441	72.1725398
20:12:40	0:09:51	73.8813582	75.846326	82.421441	72.1725398
20:12:41	0:09:52	73.8813582	76.846326	82.421441	72.1725398
20:12:42	0:09:53	73.8813582	76.846326	84.421441	72.1725398
20:12:43	0:09:54	73.8813582	76.846326	84.421441	72.1725398
20:12:44	0:09:55	73.8813582	76.846326	89.421441	74.1725398
20:12:45	0:09:56	73.8813582	77.846326	90.421441	75.1725398
20:12:46	0:09:57	73.8813582	77.846326	92.421441	75.1725398
20:12:47	0:09:58	74.8813582	74.846326	79.421441	77.1725398
20:12:48	0:09:59	73.8813582	72.846326	76.421441	77.1725398
20:12:49	0:10:00	73.8813582	72.846326	72.421441	77.1725398
20:12:50	0:10:01	74.8813582	71.846326	69.421441	77.1725398
20:12:51	0:10:02	74.8813582	72.846326	69.421441	77.1725398
20:12:52	0:10:03	75.8813582	74.846326	67.421441	77.1725398
20:12:53	0:10:04	76.8813582	74.846326	66.421441	76.1725398
20:12:54	0:10:05	77.8813582	79.846326	65.421441	75.1725398
20:12:55	0:10:06	78.8813582	81.846326	64.421441	75.1725398
20:12:56	0:10:07	76.8813582	82.846326	64.421441	75.1725398
20:12:57	0:10:08	75.8813582	81.846326	64.421441	76.1725398
20:12:58	0:10:09	75.8813582	81.846326	63.421441	78.1725398
20:12:59	0:10:10	74.8813582	78.846326	62.421441	78.1725398
20:13:00	0:10:11	75.8813582	76.846326	61.421441	83.1725398
20:13:01	0:10:12	75.8813582	75.846326	61.421441	83.1725398
20:13:02	0:10:13	75.8813582	74.846326	63.421441	83.1725398
20:13:03	0:10:14	76.8813582	74.846326	63.421441	83.1725398
20:13:04	0:10:15	76.8813582	75.846326	67.421441	82.1725398
20:13:05	0:10:16	77.8813582	75.846326	67.421441	82.1725398
20:13:06	0:10:17	77.8813582	76.846326	71.421441	81.1725398
20:13:07	0:10:18	77.8813582	77.846326	72.421441	80.1725398
20:13:08	0:10:19	76.8813582	78.846326	72.421441	79.1725398
20:13:09	0:10:20	74.8813582	78.846326	74.421441	78.1725398
20:13:10	0:10:21	74.8813582	78.846326	89.421441	78.1725398
20:13:11	0:10:22	73.8813582	78.846326	90.421441	78.1725398
20:13:12	0:10:23	73.8813582	78.846326	90.421441	78.1725398
20:13:13	0:10:24	72.8813582	77.846326	88.421441	78.1725398
20:13:14	0:10:25	70.8813582	77.846326	85.421441	79.1725398
20:13:15	0:10:26	70.8813582	77.846326	81.421441	78.1725398
20:13:16	0:10:27	70.8813582	77.846326	80.421441	77.1725398

20:13:17	0:10:28	70.8813582	77.846326	79.421441	76.1725398
20:13:18	0:10:29	70.8813582	80.846326	80.421441	75.1725398
20:13:19	0:10:30	69.8813582	80.846326	80.421441	74.1725398
20:13:20	0:10:31	69.8813582	79.846326	80.421441	74.1725398
20:13:21	0:10:32	69.8813582	79.846326	78.421441	74.1725398
20:13:22	0:10:33	70.8813582	79.846326	77.421441	73.1725398
20:13:23	0:10:34	70.8813582	79.846326	76.421441	73.1725398
20:13:24	0:10:35	71.8813582	79.846326	75.421441	73.1725398
20:13:25	0:10:36	71.8813582	79.846326	77.421441	73.1725398
20:13:26	0:10:37	71.8813582	79.846326	77.421441	73.1725398
20:13:27	0:10:38	71.8813582	79.846326	78.421441	73.1725398
20:13:28	0:10:39	70.8813582	79.846326	79.421441	74.1725398
20:13:29	0:10:40	69.8813582	78.846326	79.421441	75.1725398
20:13:30	0:10:41	69.8813582	79.846326	79.421441	75.1725398
20:13:31	0:10:42	69.8813582	79.846326	78.421441	74.1725398
20:13:32	0:10:43	69.8813582	80.846326	78.421441	74.1725398
20:13:33	0:10:44	68.8813582	78.846326	78.421441	73.1725398
20:13:34	0:10:45	65.8813582	78.846326	78.421441	73.1725398
20:13:35	0:10:46	64.8813582	77.846326	78.421441	73.1725398
20:13:36	0:10:47	65.8813582	78.846326	78.421441	73.1725398
20:13:37	0:10:48	65.8813582	78.846326	77.421441	74.1725398
20:13:38	0:10:49	65.8813582	78.846326	78.421441	74.1725398
20:13:39	0:10:50	67.8813582	79.846326	78.421441	74.1725398
20:13:40	0:10:51	67.8813582	79.846326	78.421441	74.1725398
20:13:41	0:10:52	70.8813582	79.846326	78.421441	76.1725398
20:13:42	0:10:53	70.8813582	80.846326	78.421441	78.1725398
20:13:43	0:10:54	70.8813582	80.846326	78.421441	78.1725398
20:13:44	0:10:55	69.8813582	80.846326	78.421441	76.1725398
20:13:45	0:10:56	69.8813582	78.846326	79.421441	75.1725398
20:13:46	0:10:57	69.8813582	78.846326	81.421441	75.1725398
20:13:47	0:10:58	69.8813582	78.846326	82.421441	75.1725398
20:13:48	0:10:59	70.8813582	77.846326	83.421441	75.1725398
20:13:49	0:11:00	78.8813582	77.846326	83.421441	74.1725398
20:13:50	0:11:01	82.8813582	77.846326	84.421441	74.1725398
20:13:51	0:11:02	86.8813582	77.846326	85.421441	74.1725398
20:13:52	0:11:03	87.8813582	77.846326	85.421441	75.1725398
20:13:53	0:11:04	88.8813582	77.846326	84.421441	76.1725398
20:13:54	0:11:05	89.8813582	79.846326	84.421441	78.1725398
20:13:55	0:11:06	87.8813582	80.846326	84.421441	78.1725398
20:13:56	0:11:07	81.8813582	81.846326	83.421441	78.1725398
20:13:57	0:11:08	75.8813582	81.846326	82.421441	77.1725398
20:13:58	0:11:09	75.8813582	82.846326	80.421441	75.1725398
20:13:59	0:11:10	72.8813582	82.846326	80.421441	73.1725398
20:14:00	0:11:11	71.8813582	84.846326	81.421441	72.1725398
20:14:01	0:11:12	71.8813582	84.846326	82.421441	72.1725398

20:14:02	0:11:13	72.8813582	83.846326	82.421441	72.1725398
20:14:03	0:11:14	72.8813582	80.846326	85.421441	72.1725398
20:14:04	0:11:15	72.8813582	78.846326	86.421441	73.1725398
20:14:05	0:11:16	72.8813582	77.846326	88.421441	73.1725398
20:14:06	0:11:17	72.8813582	77.846326	88.421441	73.1725398
20:14:07	0:11:18	71.8813582	79.846326	91.421441	72.1725398
20:14:08	0:11:19	70.8813582	79.846326	91.421441	73.1725398
20:14:09	0:11:20	69.8813582	79.846326	90.421441	73.1725398
20:14:10	0:11:21	69.8813582	79.846326	90.421441	73.1725398
20:14:11	0:11:22	69.8813582	79.846326	89.421441	76.1725398
20:14:12	0:11:23	71.8813582	78.846326	88.421441	76.1725398
20:14:13	0:11:24	72.8813582	77.846326	88.421441	75.1725398
20:14:14	0:11:25	72.8813582	75.846326	88.421441	75.1725398
20:14:15	0:11:26	73.8813582	75.846326	89.421441	70.1725398
20:14:16	0:11:27	74.8813582	73.846326	89.421441	69.1725398
20:14:17	0:11:28	74.8813582	71.846326	89.421441	69.1725398
20:14:18	0:11:29	73.8813582	71.846326	89.421441	68.1725398
20:14:19	0:11:30	72.8813582	71.846326	88.421441	69.1725398
20:14:20	0:11:31	72.8813582	71.846326	85.421441	71.1725398
20:14:21	0:11:32	73.8813582	71.846326	82.421441	71.1725398
20:14:22	0:11:33	73.8813582	71.846326	81.421441	71.1725398
20:14:23	0:11:34	74.8813582	71.846326	83.421441	72.1725398
20:14:24	0:11:35	74.8813582	71.846326	83.421441	72.1725398
20:14:25	0:11:36	74.8813582	71.846326	84.421441	71.1725398
20:14:26	0:11:37	74.8813582	73.846326	85.421441	71.1725398
20:14:27	0:11:38	74.8813582	76.846326	85.421441	71.1725398
20:14:28	0:11:39	74.8813582	76.846326	85.421441	71.1725398
20:14:29	0:11:40	74.8813582	76.846326	81.421441	70.1725398
20:14:30	0:11:41	73.8813582	76.846326	81.421441	69.1725398
20:14:31	0:11:42	74.8813582	77.846326	81.421441	69.1725398
20:14:32	0:11:43	74.8813582	80.846326	81.421441	67.1725398
20:14:33	0:11:44	74.8813582	80.846326	81.421441	67.1725398
20:14:34	0:11:45	74.8813582	82.846326	80.421441	68.1725398
20:14:35	0:11:46	75.8813582	83.846326	79.421441	68.1725398
20:14:36	0:11:47	76.8813582	83.846326	79.421441	69.1725398
20:14:37	0:11:48	76.8813582	83.846326	79.421441	70.1725398
20:14:38	0:11:49	76.8813582	83.846326	78.421441	70.1725398
20:14:39	0:11:50	76.8813582	83.846326	78.421441	68.1725398
20:14:40	0:11:51	76.8813582	82.846326	79.421441	68.1725398
20:14:41	0:11:52	74.8813582	82.846326	79.421441	68.1725398
20:14:42	0:11:53	74.8813582	82.846326	79.421441	67.1725398
20:14:43	0:11:54	70.8813582	82.846326	79.421441	68.1725398
20:14:44	0:11:55	69.8813582	78.846326	79.421441	69.1725398
20:14:45	0:11:56	70.8813582	78.846326	79.421441	70.1725398
20:14:46	0:11:57	71.8813582	70.846326	79.421441	70.1725398

20:14:47	0:11:58		72.8813582	69.846326	79.421441	70.1725398
20:14:48	0:11:59		72.8813582	68.846326	80.421441	70.1725398
20:14:49	0:12:00		74.8813582	68.846326	81.421441	70.1725398
20:14:50	0:12:01		75.8813582	68.846326	81.421441	70.1725398
20:14:51	0:12:02		75.8813582	68.846326	82.421441	69.1725398
20:14:52	0:12:03		73.8813582	69.846326	83.421441	67.1725398
20:14:53	0:12:04		72.8813582	69.846326	84.421441	66.1725398
20:14:54	0:12:05		71.8813582	69.846326	84.421441	66.1725398
20:14:55	0:12:06		70.8813582	69.846326	83.421441	65.1725398
20:14:56	0:12:07		69.8813582	69.846326	83.421441	65.1725398
20:14:57	0:12:08		69.8813582	69.846326	83.421441	65.1725398
20:14:58	0:12:09		69.8813582	69.846326	83.421441	65.1725398
20:14:59	0:12:10		69.8813582	69.846326	83.421441	65.1725398
20:15:00	0:12:11		69.8813582	69.846326	83.421441	65.1725398
20:15:01	0:12:12		69.8813582	69.846326	82.421441	67.1725398
20:15:02	0:12:13		69.8813582	70.846326	81.421441	67.1725398
20:15:03	0:12:14		69.8813582	71.846326	81.421441	70.1725398
20:15:04	0:12:15		69.8813582	72.846326	80.421441	71.1725398
20:15:05	0:12:16		69.8813582	73.846326	79.421441	68.1725398
20:15:06	0:12:17		69.8813582	73.846326	76.421441	69.1725398
20:15:07	0:12:18		69.8813582	74.846326	76.421441	69.1725398
20:15:08	0:12:19		69.8813582	74.846326	74.421441	71.1725398
20:15:09	0:12:20		68.8813582	74.846326	70.421441	71.1725398
20:15:10	0:12:21		68.8813582	74.846326	70.421441	71.1725398
20:15:11	0:12:22		68.8813582	74.846326	70.421441	71.1725398
20:15:12	0:12:23		68.8813582	74.846326	73.421441	69.1725398
20:15:13	0:12:24		68.8813582	72.846326	75.421441	69.1725398
20:15:14	0:12:25		68.8813582	70.846326	75.421441	68.1725398
20:15:15	0:12:26		69.8813582	70.846326	76.421441	68.1725398
20:15:16	0:12:27		72.8813582	70.846326	76.421441	69.1725398
20:15:17	0:12:28		73.8813582	70.846326	78.421441	71.1725398
20:15:18	0:12:29		74.8813582	71.846326	78.421441	71.1725398
20:15:19	0:12:30		75.8813582	73.846326	77.421441	71.1725398
20:15:20	0:12:31		75.8813582	73.846326	77.421441	73.1725398
20:15:21	0:12:32		75.8813582	74.846326	77.421441	73.1725398
20:15:22	0:12:33		74.8813582	74.846326	77.421441	73.1725398
20:15:23	0:12:34		74.8813582	74.846326	78.421441	73.1725398
20:15:24	0:12:35		74.8813582	74.846326	81.421441	74.1725398
20:15:25	0:12:36		73.8813582	73.846326	82.421441	75.1725398
20:15:26	0:12:37		72.8813582	73.846326	82.421441	75.1725398
20:15:27	0:12:38		73.8813582	74.846326	82.421441	75.1725398
20:15:28	0:12:39		74.8813582	74.846326	82.421441	74.1725398
20:15:29	0:12:40		74.8813582	76.846326	82.421441	74.1725398
20:15:30	0:12:41		73.8813582	72.846326	82.421441	71.1725398
20:15:31	0:12:42		73.8813582	71.846326	81.421441	70.1725398

20:15:32	0:12:43	73.8813582	71.846326	81.421441	70.1725398
20:15:33	0:12:44	72.8813582	71.846326	82.421441	70.1725398
20:15:34	0:12:45	71.8813582	69.846326	81.421441	70.1725398
20:15:35	0:12:46	71.8813582	68.846326	81.421441	70.1725398
20:15:36	0:12:47	71.8813582	67.846326	81.421441	70.1725398
20:15:37	0:12:48	71.8813582	67.846326	82.421441	70.1725398
20:15:38	0:12:49	71.8813582	67.846326	83.421441	71.1725398
20:15:39	0:12:50	70.8813582	67.846326	83.421441	71.1725398
20:15:40	0:12:51	70.8813582	66.846326	83.421441	71.1725398
20:15:41	0:12:52	70.8813582	67.846326	82.421441	71.1725398
20:15:42	0:12:53	70.8813582	67.846326	82.421441	70.1725398
20:15:43	0:12:54	70.8813582	68.846326	82.421441	69.1725398
20:15:44	0:12:55	71.8813582	69.846326	82.421441	69.1725398
20:15:45	0:12:56	72.8813582	69.846326	82.421441	69.1725398
20:15:46	0:12:57	72.8813582	69.846326	81.421441	68.1725398
20:15:47	0:12:58	72.8813582	70.846326	81.421441	68.1725398
20:15:48	0:12:59	72.8813582	70.846326	80.421441	67.1725398
20:15:49	0:13:00	72.8813582	70.846326	79.421441	67.1725398
20:15:50	0:13:01	72.8813582	70.846326	79.421441	67.1725398
20:15:51	0:13:02	72.8813582	70.846326	79.421441	67.1725398
20:15:52	0:13:03	73.8813582	70.846326	80.421441	67.1725398
20:15:53	0:13:04	73.8813582	69.846326	80.421441	67.1725398
20:15:54	0:13:05	73.8813582	69.846326	80.421441	67.1725398
20:15:55	0:13:06	69.8813582	69.846326	82.421441	67.1725398
20:15:56	0:13:07	69.8813582	69.846326	82.421441	67.1725398
20:15:57	0:13:08	69.8813582	69.846326	82.421441	67.1725398
20:15:58	0:13:09	69.8813582	69.846326	82.421441	67.1725398
20:15:59	0:13:10	69.8813582	69.846326	81.421441	68.1725398
20:16:00	0:13:11	69.8813582	69.846326	81.421441	68.1725398
20:16:01	0:13:12	69.8813582	69.846326	81.421441	69.1725398
20:16:02	0:13:13	68.8813582	69.846326	81.421441	69.1725398
20:16:03	0:13:14	69.8813582	69.846326	82.421441	69.1725398
20:16:04	0:13:15	70.8813582	69.846326	83.421441	69.1725398
20:16:05	0:13:16	72.8813582	69.846326	83.421441	69.1725398
20:16:06	0:13:17	72.8813582	69.846326	83.421441	69.1725398
20:16:07	0:13:18	75.8813582	69.846326	85.421441	69.1725398
20:16:08	0:13:19	77.8813582	69.846326	85.421441	70.1725398
20:16:09	0:13:20	78.8813582	70.846326	85.421441	70.1725398
20:16:10	0:13:21	80.8813582	71.846326	85.421441	70.1725398
20:16:11	0:13:22	76.8813582	72.846326	85.421441	70.1725398
20:16:12	0:13:23	72.8813582	72.846326	85.421441	71.1725398
20:16:13	0:13:24	69.8813582	72.846326	85.421441	71.1725398
20:16:14	0:13:25	68.8813582	72.846326	86.421441	70.1725398
20:16:15	0:13:26	68.8813582	72.846326	86.421441	71.1725398
20:16:16	0:13:27	67.8813582	72.846326	87.421441	74.1725398

20:16:17	0:13:28		67.8813582	72.846326	87.421441	75.1725398
20:16:18	0:13:29		69.8813582	72.846326	88.421441	75.1725398
20:16:19	0:13:30		69.8813582	71.846326	89.421441	76.1725398
20:16:20	0:13:31		70.8813582	71.846326	90.421441	75.1725398
20:16:21	0:13:32		70.8813582	70.846326	86.421441	75.1725398
20:16:22	0:13:33		69.8813582	70.846326	90.421441	71.1725398
20:16:23	0:13:34		69.8813582	69.846326	94.421441	69.1725398
20:16:24	0:13:35		69.8813582	68.846326	95.421441	69.1725398
20:16:25	0:13:36		69.8813582	68.846326	95.421441	70.1725398
20:16:26	0:13:37		69.8813582	70.846326	96.421441	71.1725398
20:16:27	0:13:38		69.8813582	70.846326	96.421441	71.1725398
20:16:28	0:13:39		69.8813582	69.846326	96.421441	71.1725398
20:16:29	0:13:40		69.8813582	69.846326	96.421441	71.1725398
20:16:30	0:13:41		69.8813582	69.846326	96.421441	69.1725398
20:16:31	0:13:42		69.8813582	68.846326	96.421441	69.1725398
20:16:32	0:13:43		69.8813582	68.846326	96.421441	68.1725398
20:16:33	0:13:44		69.8813582	68.846326	97.421441	69.1725398
20:16:34	0:13:45		70.8813582	68.846326	97.421441	69.1725398
20:16:35	0:13:46		70.8813582	68.846326	98.421441	69.1725398
20:16:36	0:13:47		69.8813582	69.846326	102.421441	71.1725398
20:16:37	0:13:48		69.8813582	69.846326	102.421441	71.1725398
20:16:38	0:13:49		69.8813582	68.846326	102.421441	71.1725398
20:16:39	0:13:50		69.8813582	68.846326	103.421441	71.1725398
20:16:40	0:13:51		70.8813582	68.846326	103.421441	71.1725398
20:16:41	0:13:52		70.8813582	68.846326	103.421441	70.1725398
20:16:42	0:13:53		70.8813582	69.846326	103.421441	70.1725398
20:16:43	0:13:54		70.8813582	69.846326	103.421441	71.1725398
20:16:44	0:13:55		69.8813582	71.846326	101.421441	73.1725398
20:16:45	0:13:56		69.8813582	71.846326	101.421441	75.1725398
20:16:46	0:13:57		69.8813582	71.846326	101.421441	78.1725398
20:16:47	0:13:58		69.8813582	71.846326	100.421441	78.1725398
20:16:48	0:13:59		69.8813582	71.846326	100.421441	78.1725398
20:16:49	0:14:00		69.8813582	71.846326	101.421441	77.1725398
20:16:50	0:14:01		69.8813582	74.846326	101.421441	76.1725398
20:16:51	0:14:02		69.8813582	75.846326	101.421441	75.1725398
20:16:52	0:14:03		69.8813582	77.846326	101.421441	72.1725398
20:16:53	0:14:04		69.8813582	79.846326	101.421441	71.1725398
20:16:54	0:14:05		69.8813582	79.846326	101.421441	73.1725398
20:16:55	0:14:06		70.8813582	81.846326	101.421441	74.1725398
20:16:56	0:14:07		70.8813582	85.846326	101.421441	74.1725398
20:16:57	0:14:08		70.8813582	87.846326	101.421441	74.1725398
20:16:58	0:14:09		71.8813582	87.846326	101.421441	74.1725398
20:16:59	0:14:10		71.8813582	83.846326	103.421441	75.1725398
20:17:00	0:14:11		70.8813582	76.846326	103.421441	76.1725398
20:17:01	0:14:12		69.8813582	73.846326	103.421441	76.1725398

20:17:02	0:14:13	69.8813582	73.846326	103.421441	75.1725398
20:17:03	0:14:14	70.8813582	72.846326	103.421441	73.1725398
20:17:04	0:14:15	71.8813582	67.846326	103.421441	73.1725398
20:17:05	0:14:16	71.8813582	64.846326	102.421441	73.1725398
20:17:06	0:14:17	71.8813582	64.846326	101.421441	73.1725398
20:17:07	0:14:18	71.8813582	63.846326	102.421441	73.1725398
20:17:08	0:14:19	71.8813582	63.846326	103.421441	74.1725398
20:17:09	0:14:20	69.8813582	63.846326	103.421441	74.1725398
20:17:10	0:14:21	68.8813582	64.846326	103.421441	78.1725398
20:17:11	0:14:22	67.8813582	64.846326	103.421441	78.1725398
20:17:12	0:14:23	67.8813582	65.846326	103.421441	79.1725398
20:17:13	0:14:24	67.8813582	66.846326	103.421441	77.1725398
20:17:14	0:14:25	68.8813582	66.846326	104.421441	75.1725398
20:17:15	0:14:26	69.8813582	66.846326	104.421441	75.1725398
20:17:16	0:14:27	69.8813582	66.846326	104.421441	75.1725398
20:17:17	0:14:28	70.8813582	66.846326	104.421441	75.1725398
20:17:18	0:14:29	70.8813582	68.846326	104.421441	77.1725398
20:17:19	0:14:30	71.8813582	69.846326	104.421441	77.1725398
20:17:20	0:14:31	73.8813582	69.846326	103.421441	78.1725398
20:17:21	0:14:32	73.8813582	68.846326	102.421441	78.1725398
20:17:22	0:14:33	73.8813582	68.846326	103.421441	78.1725398
20:17:23	0:14:34	73.8813582	67.846326	103.421441	78.1725398
20:17:24	0:14:35	73.8813582	67.846326	103.421441	77.1725398
20:17:25	0:14:36	72.8813582	67.846326	102.421441	75.1725398
20:17:26	0:14:37	71.8813582	65.846326	102.421441	74.1725398
20:17:27	0:14:38	70.8813582	66.846326	102.421441	76.1725398
20:17:28	0:14:39	69.8813582	66.846326	101.421441	80.1725398
20:17:29	0:14:40	69.8813582	66.846326	99.421441	80.1725398
20:17:30	0:14:41	69.8813582	66.846326	99.421441	80.1725398
20:17:31	0:14:42	68.8813582	66.846326	99.421441	80.1725398
20:17:32	0:14:43	68.8813582	66.846326	97.421441	78.1725398
20:17:33	0:14:44	70.8813582	66.846326	97.421441	76.1725398
20:17:34	0:14:45	71.8813582	66.846326	97.421441	76.1725398
20:17:35	0:14:46	70.8813582	66.846326	96.421441	75.1725398
20:17:36	0:14:47	70.8813582	66.846326	96.421441	76.1725398
20:17:37	0:14:48	70.8813582	67.846326	96.421441	76.1725398
20:17:38	0:14:49	70.8813582	67.846326	99.421441	78.1725398
20:17:39	0:14:50	69.8813582	66.846326	99.421441	78.1725398
20:17:40	0:14:51	69.8813582	66.846326	100.421441	80.1725398
20:17:41	0:14:52	69.8813582	66.846326	101.421441	80.1725398
20:17:42	0:14:53	69.8813582	66.846326	101.421441	80.1725398
20:17:43	0:14:54	69.8813582	65.846326	104.421441	84.1725398
20:17:44	0:14:55	69.8813582	65.846326	105.421441	85.1725398
20:17:45	0:14:56	69.8813582	68.846326	105.421441	87.1725398
20:17:46	0:14:57	69.8813582	68.846326	109.421441	88.1725398

20:17:47	0:14:58	70.8813582	68.846326	109.421441	88.1725398
20:17:48	0:14:59	73.8813582	68.846326	110.421441	88.1725398
20:17:49	0:15:00	73.8813582	68.846326	110.421441	88.1725398
20:17:50	0:15:01	75.8813582	68.846326	110.421441	88.1725398
20:17:51	0:15:02	75.8813582	68.846326	110.421441	88.1725398
20:17:52	0:15:03	77.8813582	68.846326	110.421441	88.1725398
20:17:53	0:15:04	77.8813582	68.846326	110.421441	87.1725398
20:17:54	0:15:05	78.8813582	70.846326	109.421441	86.1725398
20:17:55	0:15:06	78.8813582	72.846326	109.421441	86.1725398
20:17:56	0:15:07	77.8813582	72.846326	109.421441	84.1725398
20:17:57	0:15:08	77.8813582	75.846326	108.421441	82.1725398
20:17:58	0:15:09	77.8813582	75.846326	107.421441	80.1725398
20:17:59	0:15:10	77.8813582	74.846326	107.421441	82.1725398
20:18:00	0:15:11	76.8813582	74.846326	107.421441	82.1725398
20:18:01	0:15:12	76.8813582	73.846326	105.421441	82.1725398
20:18:02	0:15:13	76.8813582	71.846326	105.421441	81.1725398
20:18:03	0:15:14	76.8813582	70.846326	105.421441	77.1725398
20:18:04	0:15:15	78.8813582	69.846326	102.421441	77.1725398
20:18:05	0:15:16	80.8813582	69.846326	102.421441	75.1725398
20:18:06	0:15:17	82.8813582	69.846326	102.421441	75.1725398
20:18:07	0:15:18	82.8813582	68.846326	103.421441	75.1725398
20:18:08	0:15:19	85.8813582	67.846326	103.421441	76.1725398
20:18:09	0:15:20	85.8813582	66.846326	103.421441	76.1725398
20:18:10	0:15:21	85.8813582	66.846326	101.421441	76.1725398
20:18:11	0:15:22	85.8813582	66.846326	101.421441	76.1725398
20:18:12	0:15:23	86.8813582	66.846326	101.421441	76.1725398
20:18:13	0:15:24	89.8813582	66.846326	101.421441	75.1725398
20:18:14	0:15:25	91.8813582	67.846326	101.421441	75.1725398
20:18:15	0:15:26	92.8813582	67.846326	101.421441	75.1725398
20:18:16	0:15:27	93.8813582	67.846326	100.421441	75.1725398
20:18:17	0:15:28	91.8813582	66.846326	100.421441	74.1725398
20:18:18	0:15:29	84.8813582	66.846326	99.421441	74.1725398
20:18:19	0:15:30	83.8813582	66.846326	98.421441	75.1725398
20:18:20	0:15:31	83.8813582	65.846326	98.421441	74.1725398
20:18:21	0:15:32	82.8813582	65.846326	98.421441	74.1725398
20:18:22	0:15:33	82.8813582	64.846326	96.421441	73.1725398
20:18:23	0:15:34	82.8813582	64.846326	96.421441	71.1725398
20:18:24	0:15:35	79.8813582	66.846326	96.421441	71.1725398
20:18:25	0:15:36	78.8813582	67.846326	93.421441	71.1725398
20:18:26	0:15:37	77.8813582	67.846326	92.421441	71.1725398
20:18:27	0:15:38	77.8813582	67.846326	92.421441	70.1725398
20:18:28	0:15:39	77.8813582	69.846326	92.421441	70.1725398
20:18:29	0:15:40	79.8813582	69.846326	92.421441	67.1725398
20:18:30	0:15:41	79.8813582	69.846326	92.421441	67.1725398
20:18:31	0:15:42	79.8813582	69.846326	92.421441	67.1725398

20:18:32	0:15:43	80.8813582	67.846326	92.421441	66.1725398
20:18:33	0:15:44	80.8813582	66.846326	92.421441	65.1725398
20:18:34	0:15:45	80.8813582	66.846326	92.421441	64.1725398
20:18:35	0:15:46	79.8813582	66.846326	91.421441	64.1725398
20:18:36	0:15:47	79.8813582	66.846326	91.421441	64.1725398
20:18:37	0:15:48	74.8813582	66.846326	91.421441	63.1725398
20:18:38	0:15:49	74.8813582	67.846326	91.421441	64.1725398
20:18:39	0:15:50	73.8813582	71.846326	91.421441	64.1725398
20:18:40	0:15:51	73.8813582	73.846326	91.421441	64.1725398
20:18:41	0:15:52	73.8813582	73.846326	91.421441	64.1725398
20:18:42	0:15:53	73.8813582	74.846326	92.421441	63.1725398
20:18:43	0:15:54	71.8813582	74.846326	92.421441	63.1725398
20:18:44	0:15:55	70.8813582	74.846326	94.421441	63.1725398
20:18:45	0:15:56	69.8813582	74.846326	95.421441	64.1725398
20:18:46	0:15:57	68.8813582	74.846326	96.421441	64.1725398
20:18:47	0:15:58	69.8813582	68.846326	98.421441	64.1725398
20:18:48	0:15:59	70.8813582	68.846326	98.421441	69.1725398
20:18:49	0:16:00	70.8813582	67.846326	98.421441	70.1725398
20:18:50	0:16:01	70.8813582	65.846326	98.421441	70.1725398
20:18:51	0:16:02	71.8813582	65.846326	98.421441	71.1725398
20:18:52	0:16:03	71.8813582	65.846326	100.421441	71.1725398
20:18:53	0:16:04	70.8813582	65.846326	100.421441	71.1725398
20:18:54	0:16:05	70.8813582	65.846326	100.421441	72.1725398
20:18:55	0:16:06	71.8813582	65.846326	99.421441	73.1725398
20:18:56	0:16:07	71.8813582	65.846326	99.421441	73.1725398
20:18:57	0:16:08	72.8813582	66.846326	99.421441	73.1725398
20:18:58	0:16:09	71.8813582	67.846326	99.421441	73.1725398
20:18:59	0:16:10	71.8813582	67.846326	99.421441	73.1725398
20:19:00	0:16:11	71.8813582	65.846326	99.421441	73.1725398
20:19:01	0:16:12	71.8813582	63.846326	97.421441	73.1725398
20:19:02	0:16:13	71.8813582	63.846326	97.421441	75.1725398
20:19:03	0:16:14	70.8813582	63.846326	97.421441	75.1725398
20:19:04	0:16:15	70.8813582	64.846326	97.421441	82.1725398
20:19:05	0:16:16	70.8813582	64.846326	98.421441	83.1725398
20:19:06	0:16:17	69.8813582	66.846326	97.421441	80.1725398
20:19:07	0:16:18	68.8813582	67.846326	97.421441	80.1725398
20:19:08	0:16:19	68.8813582	67.846326	98.421441	80.1725398
20:19:09	0:16:20	67.8813582	67.846326	98.421441	77.1725398
20:19:10	0:16:21	67.8813582	67.846326	98.421441	76.1725398
20:19:11	0:16:22	67.8813582	68.846326	100.421441	76.1725398
20:19:12	0:16:23	67.8813582	68.846326	101.421441	75.1725398
20:19:13	0:16:24	67.8813582	67.846326	101.421441	75.1725398
20:19:14	0:16:25	68.8813582	67.846326	101.421441	71.1725398
20:19:15	0:16:26	68.8813582	66.846326	101.421441	71.1725398
20:19:16	0:16:27	68.8813582	66.846326	101.421441	70.1725398

20:19:17	0:16:28	69.8813582	66.846326	100.421441	70.1725398
20:19:18	0:16:29	69.8813582	66.846326	97.421441	72.1725398
20:19:19	0:16:30	69.8813582	68.846326	96.421441	72.1725398
20:19:20	0:16:31	69.8813582	70.846326	94.421441	72.1725398
20:19:21	0:16:32	69.8813582	70.846326	94.421441	74.1725398
20:19:22	0:16:33	69.8813582	71.846326	90.421441	74.1725398
20:19:23	0:16:34	68.8813582	70.846326	88.421441	74.1725398
20:19:24	0:16:35	67.8813582	67.846326	88.421441	72.1725398
20:19:25	0:16:36	67.8813582	67.846326	88.421441	71.1725398
20:19:26	0:16:37	67.8813582	64.846326	87.421441	72.1725398
20:19:27	0:16:38	67.8813582	65.846326	87.421441	73.1725398
20:19:28	0:16:39	67.8813582	66.846326	87.421441	73.1725398
20:19:29	0:16:40	67.8813582	66.846326	87.421441	76.1725398
20:19:30	0:16:41	67.8813582	66.846326	87.421441	76.1725398
20:19:31	0:16:42	67.8813582	68.846326	86.421441	77.1725398
20:19:32	0:16:43	67.8813582	68.846326	85.421441	77.1725398
20:19:33	0:16:44	67.8813582	68.846326	84.421441	77.1725398
20:19:34	0:16:45	67.8813582	67.846326	85.421441	77.1725398
20:19:35	0:16:46	67.8813582	67.846326	85.421441	77.1725398
20:19:36	0:16:47	67.8813582	67.846326	85.421441	77.1725398
20:19:37	0:16:48	67.8813582	67.846326	87.421441	77.1725398
20:19:38	0:16:49	67.8813582	68.846326	87.421441	76.1725398
20:19:39	0:16:50	67.8813582	68.846326	87.421441	75.1725398
20:19:40	0:16:51	67.8813582	68.846326	87.421441	74.1725398
20:19:41	0:16:52	67.8813582	69.846326	96.421441	73.1725398
20:19:42	0:16:53	67.8813582	69.846326	97.421441	71.1725398
20:19:43	0:16:54	67.8813582	69.846326	98.421441	70.1725398
20:19:44	0:16:55	67.8813582	69.846326	98.421441	70.1725398
20:19:45	0:16:56	66.8813582	69.846326	96.421441	70.1725398
20:19:46	0:16:57	66.8813582	69.846326	96.421441	70.1725398
20:19:47	0:16:58	66.8813582	69.846326	96.421441	72.1725398
20:19:48	0:16:59	66.8813582	69.846326	95.421441	71.1725398
20:19:49	0:17:00	66.8813582	70.846326	95.421441	70.1725398
20:19:50	0:17:01	66.8813582	71.846326	92.421441	70.1725398
20:19:51	0:17:02	66.8813582	71.846326	91.421441	70.1725398
20:19:52	0:17:03	66.8813582	70.846326	90.421441	70.1725398
20:19:53	0:17:04	66.8813582	70.846326	87.421441	70.1725398
20:19:54	0:17:05	66.8813582	70.846326	82.421441	70.1725398
20:19:55	0:17:06	66.8813582	69.846326	82.421441	70.1725398
20:19:56	0:17:07	66.8813582	69.846326	79.421441	71.1725398
20:19:57	0:17:08	66.8813582	69.846326	74.421441	71.1725398
20:19:58	0:17:09	66.8813582	69.846326	74.421441	71.1725398
20:19:59	0:17:10	66.8813582	69.846326	74.421441	71.1725398
20:20:00	0:17:11	67.8813582	70.846326	75.421441	71.1725398
20:20:01	0:17:12	68.8813582	70.846326	76.421441	71.1725398

20:20:02	0:17:13	69.8813582	70.846326	76.421441	71.1725398
20:20:03	0:17:14	70.8813582	71.846326	77.421441	71.1725398
20:20:04	0:17:15	71.8813582	72.846326	78.421441	71.1725398
20:20:05	0:17:16	72.8813582	73.846326	78.421441	71.1725398
20:20:06	0:17:17	73.8813582	73.846326	80.421441	72.1725398
20:20:07	0:17:18	73.8813582	73.846326	86.421441	76.1725398
20:20:08	0:17:19	73.8813582	72.846326	86.421441	76.1725398
20:20:09	0:17:20	74.8813582	72.846326	94.421441	76.1725398
20:20:10	0:17:21	73.8813582	71.846326	100.421441	75.1725398
20:20:11	0:17:22	73.8813582	71.846326	100.421441	75.1725398
20:20:12	0:17:23	73.8813582	70.846326	101.421441	74.1725398
20:20:13	0:17:24	73.8813582	69.846326	102.421441	74.1725398
20:20:14	0:17:25	73.8813582	69.846326	100.421441	76.1725398
20:20:15	0:17:26	73.8813582	69.846326	100.421441	80.1725398
20:20:16	0:17:27	74.8813582	73.846326	99.421441	80.1725398
20:20:17	0:17:28	74.8813582	79.846326	99.421441	80.1725398
20:20:18	0:17:29	74.8813582	79.846326	89.421441	82.1725398
20:20:19	0:17:30	74.8813582	79.846326	89.421441	83.1725398
20:20:20	0:17:31	73.8813582	80.846326	89.421441	83.1725398
20:20:21	0:17:32	73.8813582	80.846326	88.421441	81.1725398
20:20:22	0:17:33	73.8813582	80.846326	88.421441	81.1725398
20:20:23	0:17:34	72.8813582	81.846326	88.421441	81.1725398
20:20:24	0:17:35	72.8813582	81.846326	88.421441	81.1725398
20:20:25	0:17:36	72.8813582	81.846326	88.421441	79.1725398
20:20:26	0:17:37	71.8813582	80.846326	88.421441	78.1725398
20:20:27	0:17:38	71.8813582	76.846326	88.421441	78.1725398
20:20:28	0:17:39	71.8813582	75.846326	87.421441	79.1725398
20:20:29	0:17:40	71.8813582	75.846326	86.421441	79.1725398
20:20:30	0:17:41	69.8813582	75.846326	86.421441	78.1725398
20:20:31	0:17:42	69.8813582	74.846326	87.421441	78.1725398
20:20:32	0:17:43	69.8813582	74.846326	87.421441	76.1725398
20:20:33	0:17:44	69.8813582	72.846326	87.421441	76.1725398
20:20:34	0:17:45	68.8813582	72.846326	88.421441	76.1725398
20:20:35	0:17:46	68.8813582	72.846326	88.421441	76.1725398
20:20:36	0:17:47	68.8813582	72.846326	88.421441	76.1725398
20:20:37	0:17:48	68.8813582	71.846326	88.421441	76.1725398
20:20:38	0:17:49	68.8813582	71.846326	88.421441	76.1725398
20:20:39	0:17:50	68.8813582	71.846326	85.421441	76.1725398
20:20:40	0:17:51	70.8813582	69.846326	83.421441	76.1725398
20:20:41	0:17:52	69.8813582	69.846326	83.421441	76.1725398
20:20:42	0:17:53	69.8813582	66.846326	83.421441	79.1725398
20:20:43	0:17:54	69.8813582	65.846326	83.421441	79.1725398
20:20:44	0:17:55	69.8813582	65.846326	79.421441	78.1725398
20:20:45	0:17:56	68.8813582	64.846326	76.421441	77.1725398
20:20:46	0:17:57	68.8813582	62.846326	75.421441	77.1725398

20:20:47	0:17:58	68.8813582	60.846326	75.421441	77.1725398
20:20:48	0:17:59	69.8813582	60.846326	75.421441	77.1725398
20:20:49	0:18:00	69.8813582	60.846326	76.421441	77.1725398
20:20:50	0:18:01	69.8813582	60.846326	78.421441	76.1725398
20:20:51	0:18:02	69.8813582	60.846326	78.421441	76.1725398
20:20:52	0:18:03	69.8813582	62.846326	77.421441	75.1725398
20:20:53	0:18:04	69.8813582	62.846326	77.421441	74.1725398
20:20:54	0:18:05	69.8813582	64.846326	77.421441	74.1725398
20:20:55	0:18:06	69.8813582	64.846326	77.421441	74.1725398
20:20:56	0:18:07	69.8813582	67.846326	72.421441	74.1725398
20:20:57	0:18:08	70.8813582	68.846326	72.421441	74.1725398
20:20:58	0:18:09	70.8813582	68.846326	73.421441	73.1725398
20:20:59	0:18:10	70.8813582	69.846326	73.421441	72.1725398
20:21:00	0:18:11	71.8813582	69.846326	73.421441	72.1725398
20:21:01	0:18:12	70.8813582	69.846326	73.421441	70.1725398
20:21:02	0:18:13	70.8813582	69.846326	74.421441	70.1725398
20:21:03	0:18:14	70.8813582	72.846326	74.421441	69.1725398
20:21:04	0:18:15	70.8813582	72.846326	72.421441	69.1725398
20:21:05	0:18:16	70.8813582	73.846326	72.421441	69.1725398
20:21:06	0:18:17	71.8813582	73.846326	74.421441	71.1725398
20:21:07	0:18:18	71.8813582	73.846326	75.421441	71.1725398
20:21:08	0:18:19	71.8813582	74.846326	75.421441	77.1725398
20:21:09	0:18:20	72.8813582	74.846326	76.421441	83.1725398
20:21:10	0:18:21	73.8813582	75.846326	73.421441	85.1725398
20:21:11	0:18:22	74.8813582	75.846326	73.421441	85.1725398
20:21:12	0:18:23	74.8813582	75.846326	72.421441	84.1725398
20:21:13	0:18:24	76.8813582	74.846326	72.421441	82.1725398
20:21:14	0:18:25	76.8813582	73.846326	72.421441	81.1725398
20:21:15	0:18:26	76.8813582	73.846326	72.421441	81.1725398
20:21:16	0:18:27	76.8813582	71.846326	75.421441	81.1725398
20:21:17	0:18:28	76.8813582	70.846326	78.421441	81.1725398
20:21:18	0:18:29	76.8813582	70.846326	78.421441	81.1725398
20:21:19	0:18:30	77.8813582	70.846326	82.421441	82.1725398
20:21:20	0:18:31	77.8813582	70.846326	82.421441	80.1725398
20:21:21	0:18:32	77.8813582	70.846326	82.421441	78.1725398
20:21:22	0:18:33	77.8813582	70.846326	83.421441	77.1725398
20:21:23	0:18:34	78.8813582	70.846326	84.421441	78.1725398
20:21:24	0:18:35	79.8813582	70.846326	84.421441	79.1725398
20:21:25	0:18:36	76.8813582	70.846326	85.421441	81.1725398
20:21:26	0:18:37	75.8813582	69.846326	86.421441	81.1725398
20:21:27	0:18:38	75.8813582	69.846326	86.421441	80.1725398
20:21:28	0:18:39	74.8813582	68.846326	86.421441	79.1725398
20:21:29	0:18:40	73.8813582	69.846326	84.421441	78.1725398
20:21:30	0:18:41	73.8813582	72.846326	84.421441	78.1725398
20:21:31	0:18:42	72.8813582	72.846326	84.421441	78.1725398

20:21:32	0:18:43	71.8813582	73.846326	84.421441	80.1725398
20:21:33	0:18:44	71.8813582	72.846326	84.421441	80.1725398
20:21:34	0:18:45	72.8813582	72.846326	84.421441	81.1725398
20:21:35	0:18:46	72.8813582	71.846326	84.421441	82.1725398
20:21:36	0:18:47	72.8813582	71.846326	84.421441	81.1725398
20:21:37	0:18:48	71.8813582	71.846326	84.421441	79.1725398
20:21:38	0:18:49	71.8813582	71.846326	83.421441	77.1725398
20:21:39	0:18:50	70.8813582	74.846326	84.421441	76.1725398
20:21:40	0:18:51	69.8813582	74.846326	85.421441	77.1725398
20:21:41	0:18:52	69.8813582	75.846326	85.421441	77.1725398
20:21:42	0:18:53	68.8813582	79.846326	86.421441	78.1725398
20:21:43	0:18:54	68.8813582	78.846326	86.421441	78.1725398
20:21:44	0:18:55	68.8813582	78.846326	87.421441	78.1725398
20:21:45	0:18:56	68.8813582	78.846326	89.421441	78.1725398
20:21:46	0:18:57	68.8813582	76.846326	89.421441	84.1725398
20:21:47	0:18:58	67.8813582	76.846326	89.421441	83.1725398
20:21:48	0:18:59	68.8813582	76.846326	89.421441	80.1725398
20:21:49	0:19:00	69.8813582	70.846326	89.421441	80.1725398
20:21:50	0:19:01	70.8813582	66.846326	89.421441	77.1725398
20:21:51	0:19:02	69.8813582	64.846326	88.421441	76.1725398
20:21:52	0:19:03	69.8813582	64.846326	88.421441	75.1725398
20:21:53	0:19:04	70.8813582	64.846326	86.421441	75.1725398
20:21:54	0:19:05	70.8813582	65.846326	86.421441	74.1725398
20:21:55	0:19:06	70.8813582	66.846326	88.421441	72.1725398
20:21:56	0:19:07	70.8813582	66.846326	88.421441	72.1725398
20:21:57	0:19:08	70.8813582	66.846326	89.421441	71.1725398
20:21:58	0:19:09	71.8813582	67.846326	92.421441	71.1725398
20:21:59	0:19:10	71.8813582	67.846326	93.421441	72.1725398
20:22:00	0:19:11	71.8813582	67.846326	93.421441	73.1725398
20:22:01	0:19:12	71.8813582	67.846326	93.421441	73.1725398
20:22:02	0:19:13	71.8813582	67.846326	93.421441	73.1725398
20:22:03	0:19:14	71.8813582	67.846326	93.421441	73.1725398
20:22:04	0:19:15	70.8813582	67.846326	93.421441	71.1725398
20:22:05	0:19:16	70.8813582	68.846326	93.421441	70.1725398
20:22:06	0:19:17	71.8813582	68.846326	93.421441	70.1725398
20:22:07	0:19:18	72.8813582	69.846326	92.421441	70.1725398
20:22:08	0:19:19	72.8813582	72.846326	78.421441	70.1725398
20:22:09	0:19:20	72.8813582	72.846326	78.421441	71.1725398
20:22:10	0:19:21	72.8813582	74.846326	78.421441	70.1725398
20:22:11	0:19:22	72.8813582	80.846326	78.421441	69.1725398
20:22:12	0:19:23	72.8813582	80.846326	78.421441	69.1725398
20:22:13	0:19:24	72.8813582	80.846326	79.421441	69.1725398
20:22:14	0:19:25	72.8813582	84.846326	79.421441	70.1725398
20:22:15	0:19:26	73.8813582	85.846326	80.421441	70.1725398
20:22:16	0:19:27	72.8813582	85.846326	80.421441	70.1725398

20:22:17	0:19:28	70.8813582	82.846326	80.421441	71.1725398
20:22:18	0:19:29	69.8813582	81.846326	79.421441	71.1725398
20:22:19	0:19:30	68.8813582	80.846326	79.421441	72.1725398
20:22:20	0:19:31	68.8813582	82.846326	79.421441	72.1725398
20:22:21	0:19:32	68.8813582	82.846326	79.421441	71.1725398
20:22:22	0:19:33	68.8813582	84.846326	79.421441	72.1725398
20:22:23	0:19:34	68.8813582	84.846326	79.421441	71.1725398
20:22:24	0:19:35	67.8813582	83.846326	78.421441	71.1725398
20:22:25	0:19:36	67.8813582	79.846326	76.421441	71.1725398
20:22:26	0:19:37	68.8813582	79.846326	76.421441	71.1725398
20:22:27	0:19:38	68.8813582	74.846326	76.421441	72.1725398
20:22:28	0:19:39	68.8813582	72.846326	75.421441	72.1725398
20:22:29	0:19:40	68.8813582	71.846326	74.421441	71.1725398
20:22:30	0:19:41	69.8813582	71.846326	74.421441	71.1725398
20:22:31	0:19:42	72.8813582	71.846326	75.421441	71.1725398
20:22:32	0:19:43	73.8813582	71.846326	75.421441	71.1725398
20:22:33	0:19:44	74.8813582	71.846326	74.421441	71.1725398
20:22:34	0:19:45	75.8813582	71.846326	72.421441	72.1725398
20:22:35	0:19:46	75.8813582	69.846326	71.421441	75.1725398
20:22:36	0:19:47	75.8813582	68.846326	71.421441	76.1725398
20:22:37	0:19:48	75.8813582	68.846326	71.421441	76.1725398
20:22:38	0:19:49	75.8813582	68.846326	70.421441	73.1725398
20:22:39	0:19:50	75.8813582	68.846326	70.421441	69.1725398
20:22:40	0:19:51	73.8813582	68.846326	70.421441	68.1725398
20:22:41	0:19:52	72.8813582	68.846326	70.421441	69.1725398
20:22:42	0:19:53	72.8813582	68.846326	70.421441	70.1725398
20:22:43	0:19:54	72.8813582	70.846326	72.421441	72.1725398
20:22:44	0:19:55	71.8813582	70.846326	72.421441	73.1725398
20:22:45	0:19:56	71.8813582	73.846326	72.421441	73.1725398
20:22:46	0:19:57	72.8813582	74.846326	73.421441	74.1725398
20:22:47	0:19:58	72.8813582	74.846326	73.421441	74.1725398
20:22:48	0:19:59	72.8813582	74.846326	85.421441	74.1725398
20:22:49	0:20:00	71.8813582	74.846326	86.421441	75.1725398
20:22:50	0:20:01	71.8813582	73.846326	86.421441	75.1725398
20:22:51	0:20:02	70.8813582	73.846326	86.421441	75.1725398
20:22:52	0:20:03	70.8813582	73.846326	86.421441	75.1725398
20:22:53	0:20:04	70.8813582	73.846326	88.421441	75.1725398
20:22:54	0:20:05	70.8813582	75.846326	89.421441	74.1725398
20:22:55	0:20:06	70.8813582	75.846326	89.421441	74.1725398
20:22:56	0:20:07	70.8813582	76.846326	90.421441	76.1725398
20:22:57	0:20:08	71.8813582	76.846326	89.421441	77.1725398
20:22:58	0:20:09	71.8813582	77.846326	88.421441	78.1725398
20:22:59	0:20:10	71.8813582	78.846326	88.421441	79.1725398
20:23:00	0:20:11	72.8813582	79.846326	86.421441	79.1725398
20:23:01	0:20:12	73.8813582	79.846326	85.421441	78.1725398

20:23:02	0:20:13	74.8813582	81.846326	85.421441	78.1725398
20:23:03	0:20:14	73.8813582	78.846326	85.421441	78.1725398
20:23:04	0:20:15	70.8813582	77.846326	85.421441	78.1725398
20:23:05	0:20:16	69.8813582	76.846326	84.421441	77.1725398
20:23:06	0:20:17	69.8813582	76.846326	84.421441	77.1725398
20:23:07	0:20:18	69.8813582	74.846326	88.421441	80.1725398
20:23:08	0:20:19	71.8813582	74.846326	88.421441	79.1725398
20:23:09	0:20:20	72.8813582	73.846326	88.421441	73.1725398
20:23:10	0:20:21	72.8813582	73.846326	88.421441	69.1725398
20:23:11	0:20:22	73.8813582	73.846326	89.421441	69.1725398
20:23:12	0:20:23	73.8813582	73.846326	90.421441	68.1725398
20:23:13	0:20:24	73.8813582	72.846326	91.421441	69.1725398
20:23:14	0:20:25	72.8813582	72.846326	91.421441	69.1725398
20:23:15	0:20:26	72.8813582	72.846326	90.421441	69.1725398
20:23:16	0:20:27	72.8813582	72.846326	89.421441	69.1725398
20:23:17	0:20:28	74.8813582	72.846326	88.421441	69.1725398
20:23:18	0:20:29	73.8813582	71.846326	89.421441	69.1725398
20:23:19	0:20:30	73.8813582	72.846326	89.421441	69.1725398
20:23:20	0:20:31	73.8813582	72.846326	89.421441	69.1725398
20:23:21	0:20:32	74.8813582	72.846326	89.421441	69.1725398
20:23:22	0:20:33	74.8813582	74.846326	89.421441	69.1725398
20:23:23	0:20:34	74.8813582	75.846326	89.421441	68.1725398
20:23:24	0:20:35	74.8813582	76.846326	88.421441	68.1725398
20:23:25	0:20:36	74.8813582	76.846326	81.421441	69.1725398
20:23:26	0:20:37	74.8813582	75.846326	80.421441	69.1725398
20:23:27	0:20:38	74.8813582	74.846326	81.421441	70.1725398
20:23:28	0:20:39	74.8813582	73.846326	81.421441	71.1725398
20:23:29	0:20:40	73.8813582	73.846326	81.421441	71.1725398
20:23:30	0:20:41	72.8813582	74.846326	82.421441	71.1725398
20:23:31	0:20:42	72.8813582	73.846326	83.421441	71.1725398
20:23:32	0:20:43	72.8813582	73.846326	84.421441	71.1725398
20:23:33	0:20:44	72.8813582	72.846326	84.421441	71.1725398
20:23:34	0:20:45	72.8813582	70.846326	84.421441	70.1725398
20:23:35	0:20:46	71.8813582	65.846326	84.421441	70.1725398
20:23:36	0:20:47	71.8813582	65.846326	84.421441	69.1725398
20:23:37	0:20:48	71.8813582	61.846326	83.421441	69.1725398
20:23:38	0:20:49	69.8813582	61.846326	83.421441	69.1725398
20:23:39	0:20:50	68.8813582	67.846326	83.421441	71.1725398
20:23:40	0:20:51	68.8813582	67.846326	84.421441	71.1725398
20:23:41	0:20:52	68.8813582	67.846326	84.421441	70.1725398
20:23:42	0:20:53	68.8813582	66.846326	84.421441	68.1725398
20:23:43	0:20:54	68.8813582	66.846326	84.421441	65.1725398
20:23:44	0:20:55	69.8813582	66.846326	84.421441	64.1725398
20:23:45	0:20:56	69.8813582	66.846326	84.421441	64.1725398
20:23:46	0:20:57	69.8813582	67.846326	84.421441	64.1725398

20:23:47	0:20:58	70.8813582	67.846326	84.421441	65.1725398
20:23:48	0:20:59	70.8813582	68.846326	84.421441	66.1725398
20:23:49	0:21:00	70.8813582	68.846326	84.421441	67.1725398
20:23:50	0:21:01	71.8813582	68.846326	84.421441	67.1725398
20:23:51	0:21:02	71.8813582	70.846326	83.421441	66.1725398
20:23:52	0:21:03	71.8813582	70.846326	83.421441	66.1725398
20:23:53	0:21:04	71.8813582	70.846326	84.421441	65.1725398
20:23:54	0:21:05	69.8813582	69.846326	86.421441	65.1725398
20:23:55	0:21:06	67.8813582	69.846326	86.421441	65.1725398
20:23:56	0:21:07	67.8813582	69.846326	87.421441	65.1725398
20:23:57	0:21:08	66.8813582	69.846326	88.421441	67.1725398
20:23:58	0:21:09	66.8813582	69.846326	88.421441	68.1725398
20:23:59	0:21:10	66.8813582	70.846326	88.421441	70.1725398
20:24:00	0:21:11	66.8813582	70.846326	88.421441	70.1725398
20:24:01	0:21:12	67.8813582	70.846326	88.421441	70.1725398
20:24:02	0:21:13	67.8813582	72.846326	90.421441	70.1725398
20:24:03	0:21:14	67.8813582	73.846326	92.421441	70.1725398
20:24:04	0:21:15	67.8813582	73.846326	91.421441	70.1725398
20:24:05	0:21:16	67.8813582	74.846326	91.421441	70.1725398
20:24:06	0:21:17	67.8813582	74.846326	91.421441	70.1725398
20:24:07	0:21:18	67.8813582	75.846326	91.421441	71.1725398
20:24:08	0:21:19	67.8813582	75.846326	91.421441	71.1725398
20:24:09	0:21:20	67.8813582	75.846326	91.421441	72.1725398
20:24:10	0:21:21	67.8813582	77.846326	91.421441	73.1725398
20:24:11	0:21:22	67.8813582	77.846326	91.421441	73.1725398
20:24:12	0:21:23	67.8813582	78.846326	91.421441	73.1725398
20:24:13	0:21:24	67.8813582	77.846326	91.421441	74.1725398
20:24:14	0:21:25	67.8813582	76.846326	91.421441	74.1725398
20:24:15	0:21:26	67.8813582	74.846326	90.421441	74.1725398
20:24:16	0:21:27	67.8813582	74.846326	90.421441	72.1725398
20:24:17	0:21:28	67.8813582	76.846326	90.421441	71.1725398
20:24:18	0:21:29	67.8813582	76.846326	89.421441	71.1725398
20:24:19	0:21:30	68.8813582	76.846326	89.421441	71.1725398
20:24:20	0:21:31	67.8813582	74.846326	89.421441	75.1725398
20:24:21	0:21:32	67.8813582	75.846326	89.421441	80.1725398
20:24:22	0:21:33	68.8813582	75.846326	89.421441	80.1725398
20:24:23	0:21:34	68.8813582	75.846326	88.421441	77.1725398
20:24:24	0:21:35	68.8813582	75.846326	88.421441	75.1725398
20:24:25	0:21:36	67.8813582	75.846326	86.421441	73.1725398
20:24:26	0:21:37	67.8813582	75.846326	86.421441	74.1725398
20:24:27	0:21:38	68.8813582	76.846326	82.421441	74.1725398
20:24:28	0:21:39	68.8813582	76.846326	82.421441	73.1725398
20:24:29	0:21:40	68.8813582	76.846326	81.421441	70.1725398
20:24:30	0:21:41	68.8813582	76.846326	82.421441	70.1725398
20:24:31	0:21:42	68.8813582	74.846326	82.421441	68.1725398

20:24:32	0:21:43	68.8813582	74.846326	82.421441	67.1725398
20:24:33	0:21:44	69.8813582	72.846326	82.421441	67.1725398
20:24:34	0:21:45	70.8813582	72.846326	82.421441	67.1725398
20:24:35	0:21:46	70.8813582	72.846326	82.421441	66.1725398
20:24:36	0:21:47	71.8813582	72.846326	81.421441	66.1725398
20:24:37	0:21:48	71.8813582	72.846326	81.421441	66.1725398
20:24:38	0:21:49	71.8813582	75.846326	82.421441	66.1725398
20:24:39	0:21:50	71.8813582	76.846326	81.421441	68.1725398
20:24:40	0:21:51	71.8813582	76.846326	80.421441	68.1725398
20:24:41	0:21:52	71.8813582	76.846326	80.421441	68.1725398
20:24:42	0:21:53	71.8813582	74.846326	80.421441	66.1725398
20:24:43	0:21:54	71.8813582	72.846326	81.421441	66.1725398
20:24:44	0:21:55	71.8813582	71.846326	81.421441	66.1725398
20:24:45	0:21:56	70.8813582	71.846326	81.421441	66.1725398
20:24:46	0:21:57	70.8813582	71.846326	81.421441	63.1725398
20:24:47	0:21:58	70.8813582	71.846326	81.421441	63.1725398
20:24:48	0:21:59	70.8813582	72.846326	81.421441	63.1725398
20:24:49	0:22:00	70.8813582	72.846326	84.421441	63.1725398
20:24:50	0:22:01	70.8813582	72.846326	84.421441	65.1725398
20:24:51	0:22:02	70.8813582	72.846326	83.421441	68.1725398
20:24:52	0:22:03	70.8813582	72.846326	83.421441	69.1725398
20:24:53	0:22:04	71.8813582	72.846326	83.421441	69.1725398
20:24:54	0:22:05	71.8813582	72.846326	83.421441	69.1725398
20:24:55	0:22:06	71.8813582	71.846326	83.421441	71.1725398
20:24:56	0:22:07	72.8813582	71.846326	85.421441	73.1725398
20:24:57	0:22:08	73.8813582	72.846326	86.421441	75.1725398
20:24:58	0:22:09	73.8813582	72.846326	85.421441	75.1725398
20:24:59	0:22:10	74.8813582	72.846326	85.421441	76.1725398
20:25:00	0:22:11	74.8813582	75.846326	85.421441	77.1725398
20:25:01	0:22:12	74.8813582	75.846326	85.421441	69.1725398
20:25:02	0:22:13	74.8813582	75.846326	85.421441	61.1725398
20:25:03	0:22:14	74.8813582	76.846326	86.421441	61.1725398
20:25:04	0:22:15	73.8813582	74.846326	86.421441	62.1725398
20:25:05	0:22:16	73.8813582	76.846326	86.421441	65.1725398
20:25:06	0:22:17	74.8813582	77.846326	89.421441	66.1725398
20:25:07	0:22:18	74.8813582	77.846326	90.421441	66.1725398
20:25:08	0:22:19	73.8813582	77.846326	90.421441	66.1725398
20:25:09	0:22:20	73.8813582	77.846326	89.421441	66.1725398
20:25:10	0:22:21	73.8813582	78.846326	88.421441	67.1725398
20:25:11	0:22:22	73.8813582	78.846326	88.421441	73.1725398
20:25:12	0:22:23	73.8813582	77.846326	88.421441	75.1725398
20:25:13	0:22:24	72.8813582	75.846326	90.421441	75.1725398
20:25:14	0:22:25	71.8813582	75.846326	90.421441	74.1725398
20:25:15	0:22:26	70.8813582	70.846326	90.421441	74.1725398
20:25:16	0:22:27	70.8813582	68.846326	90.421441	74.1725398

20:25:17	0:22:28	72.8813582	68.846326	90.421441	74.1725398
20:25:18	0:22:29	71.8813582	68.846326	90.421441	74.1725398
20:25:19	0:22:30	70.8813582	68.846326	90.421441	74.1725398
20:25:20	0:22:31	71.8813582	68.846326	87.421441	73.1725398
20:25:21	0:22:32	71.8813582	68.846326	87.421441	73.1725398
20:25:22	0:22:33	70.8813582	68.846326	87.421441	73.1725398
20:25:23	0:22:34	70.8813582	68.846326	87.421441	72.1725398
20:25:24	0:22:35	70.8813582	68.846326	86.421441	72.1725398
20:25:25	0:22:36	71.8813582	66.846326	86.421441	72.1725398
20:25:26	0:22:37	71.8813582	66.846326	86.421441	72.1725398
20:25:27	0:22:38	71.8813582	66.846326	87.421441	73.1725398
20:25:28	0:22:39	71.8813582	66.846326	87.421441	78.1725398
20:25:29	0:22:40	72.8813582	66.846326	88.421441	78.1725398
20:25:30	0:22:41	72.8813582	67.846326	88.421441	78.1725398
20:25:31	0:22:42	72.8813582	67.846326	88.421441	78.1725398
20:25:32	0:22:43	72.8813582	67.846326	86.421441	76.1725398
20:25:33	0:22:44	72.8813582	67.846326	86.421441	77.1725398
20:25:34	0:22:45	73.8813582	67.846326	85.421441	79.1725398
20:25:35	0:22:46	73.8813582	66.846326	85.421441	79.1725398
20:25:36	0:22:47	74.8813582	66.846326	84.421441	79.1725398
20:25:37	0:22:48	75.8813582	66.846326	84.421441	80.1725398
20:25:38	0:22:49	77.8813582	67.846326	83.421441	80.1725398
20:25:39	0:22:50	79.8813582	67.846326	83.421441	80.1725398
20:25:40	0:22:51	78.8813582	67.846326	83.421441	78.1725398
20:25:41	0:22:52	78.8813582	67.846326	83.421441	78.1725398
20:25:42	0:22:53	77.8813582	67.846326	83.421441	76.1725398
20:25:43	0:22:54	75.8813582	67.846326	82.421441	76.1725398
20:25:44	0:22:55	72.8813582	64.846326	80.421441	75.1725398
20:25:45	0:22:56	72.8813582	66.846326	81.421441	75.1725398
20:25:46	0:22:57	70.8813582	69.846326	81.421441	75.1725398
20:25:47	0:22:58	70.8813582	69.846326	82.421441	75.1725398
20:25:48	0:22:59	70.8813582	69.846326	82.421441	75.1725398
20:25:49	0:23:00	69.8813582	70.846326	83.421441	75.1725398
20:25:50	0:23:01	70.8813582	68.846326	83.421441	75.1725398
20:25:51	0:23:02	70.8813582	68.846326	83.421441	75.1725398
20:25:52	0:23:03	70.8813582	67.846326	84.421441	77.1725398
20:25:53	0:23:04	70.8813582	68.846326	85.421441	73.1725398
20:25:54	0:23:05	69.8813582	68.846326	88.421441	71.1725398
20:25:55	0:23:06	69.8813582	68.846326	90.421441	71.1725398
20:25:56	0:23:07	70.8813582	68.846326	91.421441	70.1725398
20:25:57	0:23:08	71.8813582	68.846326	92.421441	69.1725398
20:25:58	0:23:09	70.8813582	68.846326	92.421441	69.1725398
20:25:59	0:23:10	69.8813582	69.846326	93.421441	69.1725398
20:26:00	0:23:11	68.8813582	70.846326	93.421441	69.1725398
20:26:01	0:23:12	68.8813582	72.846326	93.421441	69.1725398

20:26:02	0:23:13	67.8813582	72.846326	93.421441	72.1725398
20:26:03	0:23:14	68.8813582	74.846326	93.421441	72.1725398
20:26:04	0:23:15	68.8813582	73.846326	94.421441	72.1725398
20:26:05	0:23:16	69.8813582	73.846326	95.421441	72.1725398
20:26:06	0:23:17	70.8813582	73.846326	95.421441	71.1725398
20:26:07	0:23:18	70.8813582	73.846326	96.421441	71.1725398
20:26:08	0:23:19	70.8813582	73.846326	98.421441	70.1725398
20:26:09	0:23:20	70.8813582	70.846326	102.421441	71.1725398
20:26:10	0:23:21	70.8813582	70.846326	103.421441	71.1725398
20:26:11	0:23:22	69.8813582	70.846326	103.421441	72.1725398
20:26:12	0:23:23	69.8813582	70.846326	105.421441	71.1725398
20:26:13	0:23:24	69.8813582	72.846326	104.421441	69.1725398
20:26:14	0:23:25	69.8813582	73.846326	104.421441	68.1725398
20:26:15	0:23:26	69.8813582	74.846326	103.421441	67.1725398
20:26:16	0:23:27	69.8813582	73.846326	103.421441	67.1725398
20:26:17	0:23:28	69.8813582	73.846326	97.421441	68.1725398
20:26:18	0:23:29	70.8813582	69.846326	97.421441	68.1725398
20:26:19	0:23:30	70.8813582	67.846326	97.421441	70.1725398
20:26:20	0:23:31	70.8813582	65.846326	97.421441	72.1725398
20:26:21	0:23:32	70.8813582	65.846326	95.421441	75.1725398
20:26:22	0:23:33	71.8813582	65.846326	94.421441	76.1725398
20:26:23	0:23:34	71.8813582	65.846326	93.421441	76.1725398
20:26:24	0:23:35	70.8813582	65.846326	93.421441	72.1725398
20:26:25	0:23:36	69.8813582	65.846326	93.421441	71.1725398
20:26:26	0:23:37	69.8813582	66.846326	93.421441	70.1725398
20:26:27	0:23:38	68.8813582	67.846326	93.421441	70.1725398
20:26:28	0:23:39	68.8813582	67.846326	93.421441	71.1725398
20:26:29	0:23:40	68.8813582	68.846326	91.421441	72.1725398
20:26:30	0:23:41	67.8813582	68.846326	88.421441	75.1725398
20:26:31	0:23:42	67.8813582	68.846326	88.421441	77.1725398
20:26:32	0:23:43	67.8813582	67.846326	86.421441	76.1725398
20:26:33	0:23:44	67.8813582	67.846326	85.421441	75.1725398
20:26:34	0:23:45	67.8813582	67.846326	84.421441	75.1725398
20:26:35	0:23:46	68.8813582	67.846326	84.421441	75.1725398
20:26:36	0:23:47	68.8813582	70.846326	80.421441	75.1725398
20:26:37	0:23:48	68.8813582	70.846326	78.421441	75.1725398
20:26:38	0:23:49	68.8813582	70.846326	78.421441	78.1725398
20:26:39	0:23:50	68.8813582	70.846326	78.421441	78.1725398
20:26:40	0:23:51	68.8813582	68.846326	81.421441	78.1725398
20:26:41	0:23:52	68.8813582	68.846326	83.421441	78.1725398
20:26:42	0:23:53	68.8813582	68.846326	83.421441	78.1725398
20:26:43	0:23:54	68.8813582	68.846326	83.421441	74.1725398
20:26:44	0:23:55	69.8813582	69.846326	84.421441	69.1725398
20:26:45	0:23:56	69.8813582	69.846326	86.421441	69.1725398
20:26:46	0:23:57	69.8813582	70.846326	86.421441	69.1725398

20:26:47	0:23:58	69.8813582	70.846326	87.421441	69.1725398
20:26:48	0:23:59	69.8813582	70.846326	87.421441	69.1725398
20:26:49	0:24:00	69.8813582	70.846326	87.421441	69.1725398
20:26:50	0:24:01	69.8813582	70.846326	87.421441	70.1725398
20:26:51	0:24:02	69.8813582	71.846326	87.421441	70.1725398
20:26:52	0:24:03	69.8813582	71.846326	87.421441	71.1725398
20:26:53	0:24:04	71.8813582	72.846326	87.421441	71.1725398
20:26:54	0:24:05	72.8813582	72.846326	87.421441	70.1725398
20:26:55	0:24:06	72.8813582	72.846326	87.421441	70.1725398
20:26:56	0:24:07	73.8813582	72.846326	87.421441	70.1725398
20:26:57	0:24:08	73.8813582	73.846326	86.421441	70.1725398
20:26:58	0:24:09	73.8813582	73.846326	77.421441	70.1725398
20:26:59	0:24:10	73.8813582	73.846326	77.421441	71.1725398
20:27:00	0:24:11	73.8813582	73.846326	75.421441	73.1725398
20:27:01	0:24:12	73.8813582	73.846326	72.421441	75.1725398
20:27:02	0:24:13	73.8813582	71.846326	71.421441	76.1725398
20:27:03	0:24:14	71.8813582	70.846326	71.421441	77.1725398
20:27:04	0:24:15	70.8813582	71.846326	72.421441	77.1725398
20:27:05	0:24:16	70.8813582	71.846326	75.421441	77.1725398
20:27:06	0:24:17	71.8813582	71.846326	76.421441	76.1725398
20:27:07	0:24:18	72.8813582	71.846326	76.421441	76.1725398
20:27:08	0:24:19	73.8813582	71.846326	76.421441	75.1725398
20:27:09	0:24:20	73.8813582	71.846326	78.421441	75.1725398
20:27:10	0:24:21	74.8813582	71.846326	78.421441	78.1725398
20:27:11	0:24:22	74.8813582	71.846326	78.421441	80.1725398
20:27:12	0:24:23	74.8813582	71.846326	78.421441	81.1725398
20:27:13	0:24:24	73.8813582	70.846326	78.421441	81.1725398
20:27:14	0:24:25	74.8813582	74.846326	78.421441	81.1725398
20:27:15	0:24:26	74.8813582	74.846326	78.421441	81.1725398
20:27:16	0:24:27	74.8813582	76.846326	79.421441	75.1725398
20:27:17	0:24:28	74.8813582	75.846326	82.421441	72.1725398
20:27:18	0:24:29	74.8813582	75.846326	81.421441	71.1725398
20:27:19	0:24:30	75.8813582	73.846326	81.421441	71.1725398
20:27:20	0:24:31	75.8813582	72.846326	81.421441	71.1725398
20:27:21	0:24:32	75.8813582	72.846326	81.421441	70.1725398
20:27:22	0:24:33	77.8813582	72.846326	81.421441	70.1725398
20:27:23	0:24:34	79.8813582	73.846326	81.421441	70.1725398
20:27:24	0:24:35	79.8813582	73.846326	79.421441	71.1725398
20:27:25	0:24:36	78.8813582	71.846326	80.421441	71.1725398
20:27:26	0:24:37	78.8813582	70.846326	81.421441	72.1725398
20:27:27	0:24:38	76.8813582	69.846326	79.421441	71.1725398
20:27:28	0:24:39	76.8813582	69.846326	78.421441	71.1725398
20:27:29	0:24:40	77.8813582	69.846326	78.421441	70.1725398
20:27:30	0:24:41	78.8813582	68.846326	79.421441	70.1725398
20:27:31	0:24:42	78.8813582	68.846326	80.421441	71.1725398

20:27:32	0:24:43	78.8813582	67.846326	81.421441	71.1725398
20:27:33	0:24:44	78.8813582	68.846326	81.421441	72.1725398
20:27:34	0:24:45	79.8813582	68.846326	82.421441	72.1725398
20:27:35	0:24:46	78.8813582	68.846326	82.421441	72.1725398
20:27:36	0:24:47	78.8813582	68.846326	82.421441	72.1725398
20:27:37	0:24:48	78.8813582	68.846326	83.421441	72.1725398
20:27:38	0:24:49	78.8813582	68.846326	82.421441	73.1725398
20:27:39	0:24:50	78.8813582	68.846326	82.421441	73.1725398
20:27:40	0:24:51	78.8813582	68.846326	82.421441	73.1725398
20:27:41	0:24:52	78.8813582	68.846326	82.421441	73.1725398
20:27:42	0:24:53	78.8813582	68.846326	83.421441	74.1725398
20:27:43	0:24:54	80.8813582	68.846326	83.421441	74.1725398
20:27:44	0:24:55	81.8813582	66.846326	82.421441	74.1725398
20:27:45	0:24:56	83.8813582	67.846326	82.421441	74.1725398
20:27:46	0:24:57	84.8813582	67.846326	83.421441	74.1725398
20:27:47	0:24:58	85.8813582	67.846326	83.421441	74.1725398
20:27:48	0:24:59	87.8813582	67.846326	83.421441	76.1725398
20:27:49	0:25:00	87.8813582	66.846326	83.421441	84.1725398
20:27:50	0:25:01	89.8813582	66.846326	83.421441	86.1725398
20:27:51	0:25:02	90.8813582	69.846326	83.421441	86.1725398
20:27:52	0:25:03	91.8813582	69.846326	83.421441	84.1725398
20:27:53	0:25:04	91.8813582	69.846326	83.421441	78.1725398
20:27:54	0:25:05	92.8813582	69.846326	83.421441	75.1725398
20:27:55	0:25:06	94.8813582	69.846326	83.421441	73.1725398
20:27:56	0:25:07	94.8813582	69.846326	82.421441	71.1725398
20:27:57	0:25:08	94.8813582	69.846326	80.421441	70.1725398
20:27:58	0:25:09	94.8813582	69.846326	80.421441	70.1725398
20:27:59	0:25:10	95.8813582	69.846326	80.421441	69.1725398
20:28:00	0:25:11	95.8813582	70.846326	78.421441	69.1725398
20:28:01	0:25:12	95.8813582	70.846326	78.421441	69.1725398
20:28:02	0:25:13	95.8813582	70.846326	76.421441	69.1725398
20:28:03	0:25:14	97.8813582	70.846326	75.421441	70.1725398
20:28:04	0:25:15	98.8813582	70.846326	75.421441	70.1725398
20:28:05	0:25:16	93.8813582	69.846326	73.421441	66.1725398
20:28:06	0:25:17	90.8813582	69.846326	73.421441	65.1725398
20:28:07	0:25:18	84.8813582	68.846326	73.421441	66.1725398
20:28:08	0:25:19	78.8813582	68.846326	73.421441	66.1725398
20:28:09	0:25:20	75.8813582	70.846326	72.421441	68.1725398
20:28:10	0:25:21	73.8813582	70.846326	72.421441	68.1725398
20:28:11	0:25:22	71.8813582	70.846326	70.421441	68.1725398
20:28:12	0:25:23	70.8813582	69.846326	70.421441	68.1725398
20:28:13	0:25:24	69.8813582	69.846326	70.421441	72.1725398
20:28:14	0:25:25	69.8813582	69.846326	71.421441	75.1725398
20:28:15	0:25:26	70.8813582	68.846326	72.421441	76.1725398
20:28:16	0:25:27	72.8813582	69.846326	73.421441	76.1725398

20:28:17	0:25:28	76.8813582	69.846326	81.421441	75.1725398
20:28:18	0:25:29	79.8813582	71.846326	83.421441	74.1725398
20:28:19	0:25:30	80.8813582	72.846326	83.421441	74.1725398
20:28:20	0:25:31	81.8813582	72.846326	84.421441	74.1725398
20:28:21	0:25:32	81.8813582	72.846326	84.421441	74.1725398
20:28:22	0:25:33	81.8813582	72.846326	84.421441	74.1725398
20:28:23	0:25:34	83.8813582	71.846326	84.421441	74.1725398
20:28:24	0:25:35	84.8813582	71.846326	84.421441	74.1725398
20:28:25	0:25:36	90.8813582	69.846326	85.421441	73.1725398
20:28:26	0:25:37	93.8813582	69.846326	84.421441	71.1725398
20:28:27	0:25:38	92.8813582	69.846326	84.421441	70.1725398
20:28:28	0:25:39	90.8813582	69.846326	83.421441	69.1725398
20:28:29	0:25:40	89.8813582	71.846326	83.421441	69.1725398
20:28:30	0:25:41	89.8813582	71.846326	83.421441	69.1725398
20:28:31	0:25:42	87.8813582	72.846326	82.421441	70.1725398
20:28:32	0:25:43	87.8813582	73.846326	82.421441	70.1725398
20:28:33	0:25:44	87.8813582	71.846326	81.421441	75.1725398
20:28:34	0:25:45	85.8813582	70.846326	81.421441	75.1725398
20:28:35	0:25:46	84.8813582	70.846326	80.421441	75.1725398
20:28:36	0:25:47	81.8813582	70.846326	80.421441	75.1725398
20:28:37	0:25:48	77.8813582	70.846326	79.421441	74.1725398
20:28:38	0:25:49	75.8813582	70.846326	79.421441	74.1725398
20:28:39	0:25:50	75.8813582	71.846326	79.421441	73.1725398
20:28:40	0:25:51	75.8813582	70.846326	79.421441	72.1725398
20:28:41	0:25:52	75.8813582	70.846326	79.421441	72.1725398
20:28:42	0:25:53	75.8813582	70.846326	79.421441	72.1725398
20:28:43	0:25:54	75.8813582	70.846326	81.421441	72.1725398
20:28:44	0:25:55	75.8813582	69.846326	85.421441	71.1725398
20:28:45	0:25:56	75.8813582	68.846326	85.421441	71.1725398
20:28:46	0:25:57	75.8813582	67.846326	87.421441	71.1725398
20:28:47	0:25:58	75.8813582	67.846326	88.421441	70.1725398
20:28:48	0:25:59	74.8813582	67.846326	89.421441	71.1725398
20:28:49	0:26:00	74.8813582	66.846326	89.421441	73.1725398
20:28:50	0:26:01	74.8813582	70.846326	89.421441	75.1725398
20:28:51	0:26:02	74.8813582	70.846326	89.421441	75.1725398
20:28:52	0:26:03	74.8813582	70.846326	90.421441	73.1725398
20:28:53	0:26:04	75.8813582	70.846326	90.421441	70.1725398
20:28:54	0:26:05	75.8813582	70.846326	90.421441	69.1725398
20:28:55	0:26:06	75.8813582	70.846326	93.421441	69.1725398
20:28:56	0:26:07	76.8813582	70.846326	93.421441	71.1725398
20:28:57	0:26:08	77.8813582	70.846326	93.421441	73.1725398
20:28:58	0:26:09	78.8813582	70.846326	88.421441	74.1725398
20:28:59	0:26:10	78.8813582	70.846326	88.421441	73.1725398
20:29:00	0:26:11	78.8813582	70.846326	87.421441	72.1725398
20:29:01	0:26:12	77.8813582	67.846326	87.421441	71.1725398

20:29:02	0:26:13	77.8813582	66.846326	87.421441	71.1725398
20:29:03	0:26:14	77.8813582	66.846326	87.421441	71.1725398
20:29:04	0:26:15	77.8813582	66.846326	87.421441	71.1725398
20:29:05	0:26:16	77.8813582	68.846326	87.421441	70.1725398
20:29:06	0:26:17	77.8813582	68.846326	86.421441	70.1725398
20:29:07	0:26:18	77.8813582	68.846326	86.421441	71.1725398
20:29:08	0:26:19	76.8813582	68.846326	86.421441	71.1725398
20:29:09	0:26:20	74.8813582	68.846326	85.421441	72.1725398
20:29:10	0:26:21	72.8813582	67.846326	84.421441	72.1725398
20:29:11	0:26:22	70.8813582	67.846326	84.421441	73.1725398
20:29:12	0:26:23	68.8813582	67.846326	84.421441	74.1725398
20:29:13	0:26:24	68.8813582	64.846326	84.421441	74.1725398
20:29:14	0:26:25	68.8813582	64.846326	83.421441	75.1725398
20:29:15	0:26:26	68.8813582	63.846326	83.421441	75.1725398
20:29:16	0:26:27	68.8813582	63.846326	81.421441	75.1725398
20:29:17	0:26:28	68.8813582	64.846326	83.421441	76.1725398
20:29:18	0:26:29	68.8813582	65.846326	85.421441	76.1725398
20:29:19	0:26:30	68.8813582	65.846326	85.421441	76.1725398
20:29:20	0:26:31	68.8813582	70.846326	86.421441	72.1725398
20:29:21	0:26:32	68.8813582	72.846326	85.421441	70.1725398
20:29:22	0:26:33	68.8813582	73.846326	86.421441	70.1725398
20:29:23	0:26:34	69.8813582	72.846326	86.421441	71.1725398
20:29:24	0:26:35	69.8813582	72.846326	86.421441	71.1725398
20:29:25	0:26:36	70.8813582	71.846326	88.421441	71.1725398
20:29:26	0:26:37	69.8813582	71.846326	88.421441	71.1725398
20:29:27	0:26:38	68.8813582	70.846326	88.421441	71.1725398
20:29:28	0:26:39	68.8813582	70.846326	86.421441	72.1725398
20:29:29	0:26:40	68.8813582	70.846326	86.421441	73.1725398
20:29:30	0:26:41	69.8813582	71.846326	86.421441	73.1725398
20:29:31	0:26:42	70.8813582	72.846326	86.421441	74.1725398
20:29:32	0:26:43	71.8813582	72.846326	86.421441	74.1725398
20:29:33	0:26:44	71.8813582	72.846326	86.421441	75.1725398
20:29:34	0:26:45	71.8813582	72.846326	86.421441	77.1725398
20:29:35	0:26:46	71.8813582	70.846326	86.421441	77.1725398
20:29:36	0:26:47	70.8813582	69.846326	86.421441	78.1725398
20:29:37	0:26:48	69.8813582	69.846326	84.421441	78.1725398
20:29:38	0:26:49	69.8813582	71.846326	82.421441	78.1725398
20:29:39	0:26:50	69.8813582	72.846326	81.421441	78.1725398
20:29:40	0:26:51	69.8813582	73.846326	80.421441	78.1725398
20:29:41	0:26:52	68.8813582	74.846326	80.421441	78.1725398
20:29:42	0:26:53	68.8813582	74.846326	81.421441	78.1725398
20:29:43	0:26:54	68.8813582	73.846326	82.421441	77.1725398
20:29:44	0:26:55	68.8813582	73.846326	83.421441	75.1725398
20:29:45	0:26:56	68.8813582	73.846326	82.421441	75.1725398
20:29:46	0:26:57	68.8813582	73.846326	81.421441	74.1725398

20:29:47	0:26:58	68.8813582	73.846326	81.421441	73.1725398
20:29:48	0:26:59	68.8813582	72.846326	81.421441	74.1725398
20:29:49	0:27:00	68.8813582	71.846326	81.421441	74.1725398
20:29:50	0:27:01	68.8813582	71.846326	81.421441	74.1725398
20:29:51	0:27:02	68.8813582	71.846326	79.421441	74.1725398
20:29:52	0:27:03	68.8813582	71.846326	79.421441	73.1725398
20:29:53	0:27:04	69.8813582	71.846326	79.421441	73.1725398
20:29:54	0:27:05	69.8813582	71.846326	79.421441	73.1725398
20:29:55	0:27:06	72.8813582	71.846326	79.421441	72.1725398
20:29:56	0:27:07	72.8813582	71.846326	79.421441	72.1725398
20:29:57	0:27:08	72.8813582	71.846326	79.421441	72.1725398
20:29:58	0:27:09	73.8813582	72.846326	79.421441	71.1725398
20:29:59	0:27:10	73.8813582	72.846326	79.421441	73.1725398
20:30:00	0:27:11	73.8813582	72.846326	80.421441	74.1725398
20:30:01	0:27:12	75.8813582	69.846326	80.421441	75.1725398
20:30:02	0:27:13	76.8813582	69.846326	81.421441	75.1725398
20:30:03	0:27:14	76.8813582	69.846326	83.421441	77.1725398
20:30:04	0:27:15	77.8813582	69.846326	85.421441	76.1725398
20:30:05	0:27:16	78.8813582	68.846326	85.421441	76.1725398
20:30:06	0:27:17	78.8813582	68.846326	88.421441	76.1725398
20:30:07	0:27:18	79.8813582	68.846326	89.421441	76.1725398
20:30:08	0:27:19	79.8813582	68.846326	89.421441	76.1725398
20:30:09	0:27:20	79.8813582	68.846326	89.421441	77.1725398
20:30:10	0:27:21	79.8813582	67.846326	88.421441	77.1725398
20:30:11	0:27:22	75.8813582	67.846326	88.421441	77.1725398
20:30:12	0:27:23	75.8813582	67.846326	87.421441	77.1725398
20:30:13	0:27:24	75.8813582	68.846326	87.421441	74.1725398
20:30:14	0:27:25	75.8813582	68.846326	86.421441	74.1725398
20:30:15	0:27:26	75.8813582	68.846326	86.421441	73.1725398
20:30:16	0:27:27	75.8813582	68.846326	85.421441	72.1725398
20:30:17	0:27:28	75.8813582	69.846326	86.421441	72.1725398
20:30:18	0:27:29	75.8813582	69.846326	86.421441	72.1725398
20:30:19	0:27:30	72.8813582	72.846326	85.421441	72.1725398
20:30:20	0:27:31	72.8813582	72.846326	85.421441	72.1725398
20:30:21	0:27:32	72.8813582	72.846326	86.421441	73.1725398
20:30:22	0:27:33	73.8813582	76.846326	85.421441	74.1725398
20:30:23	0:27:34	72.8813582	77.846326	85.421441	74.1725398
20:30:24	0:27:35	72.8813582	77.846326	83.421441	74.1725398
20:30:25	0:27:36	69.8813582	77.846326	83.421441	74.1725398
20:30:26	0:27:37	69.8813582	73.846326	82.421441	74.1725398
20:30:27	0:27:38	69.8813582	72.846326	82.421441	74.1725398
20:30:28	0:27:39	69.8813582	70.846326	83.421441	74.1725398
20:30:29	0:27:40	69.8813582	70.846326	85.421441	75.1725398
20:30:30	0:27:41	69.8813582	70.846326	85.421441	76.1725398
20:30:31	0:27:42	70.8813582	70.846326	84.421441	77.1725398

20:30:32	0:27:43		70.8813582	71.846326	85.421441	77.1725398
20:30:33	0:27:44		70.8813582	71.846326	85.421441	77.1725398
20:30:34	0:27:45		70.8813582	71.846326	85.421441	77.1725398
20:30:35	0:27:46		71.8813582	72.846326	87.421441	77.1725398
20:30:36	0:27:47		71.8813582	72.846326	88.421441	80.1725398
20:30:37	0:27:48		71.8813582	72.846326	88.421441	81.1725398
20:30:38	0:27:49		71.8813582	71.846326	88.421441	81.1725398
20:30:39	0:27:50		71.8813582	71.846326	90.421441	82.1725398
20:30:40	0:27:51		71.8813582	71.846326	93.421441	82.1725398
20:30:41	0:27:52		71.8813582	71.846326	93.421441	85.1725398
20:30:42	0:27:53		72.8813582	70.846326	99.421441	81.1725398
20:30:43	0:27:54		73.8813582	69.846326	100.421441	81.1725398
20:30:44	0:27:55		73.8813582	69.846326	100.421441	79.1725398
20:30:45	0:27:56		73.8813582	69.846326	101.421441	78.1725398
20:30:46	0:27:57		73.8813582	68.846326	104.421441	78.1725398
20:30:47	0:27:58		73.8813582	67.846326	104.421441	77.1725398
20:30:48	0:27:59		73.8813582	67.846326	108.421441	77.1725398
20:30:49	0:28:00		73.8813582	64.846326	108.421441	77.1725398
20:30:50	0:28:01		73.8813582	64.846326	108.421441	78.1725398
20:30:51	0:28:02		73.8813582	64.846326	107.421441	78.1725398
20:30:52	0:28:03		72.8813582	64.846326	106.421441	78.1725398
20:30:53	0:28:04		72.8813582	65.846326	106.421441	78.1725398
20:30:54	0:28:05		72.8813582	66.846326	106.421441	78.1725398
20:30:55	0:28:06		73.8813582	66.846326	104.421441	78.1725398
20:30:56	0:28:07		73.8813582	67.846326	104.421441	77.1725398
20:30:57	0:28:08		73.8813582	67.846326	103.421441	77.1725398
20:30:58	0:28:09		73.8813582	67.846326	98.421441	75.1725398
20:30:59	0:28:10		74.8813582	67.846326	98.421441	71.1725398
20:31:00	0:28:11		74.8813582	67.846326	94.421441	71.1725398
20:31:01	0:28:12		74.8813582	67.846326	94.421441	68.1725398
20:31:02	0:28:13		74.8813582	67.846326	94.421441	66.1725398
20:31:03	0:28:14		74.8813582	67.846326	94.421441	66.1725398
20:31:04	0:28:15		74.8813582	67.846326	94.421441	65.1725398
20:31:05	0:28:16		74.8813582	67.846326	94.421441	65.1725398
20:31:06	0:28:17		74.8813582	67.846326	92.421441	65.1725398
20:31:07	0:28:18		73.8813582	67.846326	89.421441	65.1725398
20:31:08	0:28:19		73.8813582	69.846326	89.421441	64.1725398
20:31:09	0:28:20		73.8813582	69.846326	86.421441	64.1725398
20:31:10	0:28:21		73.8813582	69.846326	86.421441	64.1725398
20:31:11	0:28:22		73.8813582	69.846326	86.421441	68.1725398
20:31:12	0:28:23		73.8813582	69.846326	86.421441	68.1725398
20:31:13	0:28:24		73.8813582	69.846326	86.421441	71.1725398
20:31:14	0:28:25		73.8813582	69.846326	86.421441	71.1725398
20:31:15	0:28:26		75.8813582	68.846326	88.421441	71.1725398
20:31:16	0:28:27		75.8813582	69.846326	88.421441	71.1725398

20:31:17	0:28:28	75.8813582	69.846326	88.421441	70.1725398
20:31:18	0:28:29	75.8813582	69.846326	86.421441	69.1725398
20:31:19	0:28:30	75.8813582	69.846326	85.421441	68.1725398
20:31:20	0:28:31	75.8813582	70.846326	85.421441	69.1725398
20:31:21	0:28:32	75.8813582	71.846326	83.421441	69.1725398
20:31:22	0:28:33	75.8813582	70.846326	83.421441	69.1725398
20:31:23	0:28:34	75.8813582	70.846326	82.421441	68.1725398
20:31:24	0:28:35	74.8813582	70.846326	81.421441	68.1725398
20:31:25	0:28:36	74.8813582	70.846326	81.421441	68.1725398
20:31:26	0:28:37	73.8813582	69.846326	80.421441	67.1725398
20:31:27	0:28:38	73.8813582	69.846326	79.421441	66.1725398
20:31:28	0:28:39	73.8813582	68.846326	79.421441	66.1725398
20:31:29	0:28:40	72.8813582	69.846326	79.421441	66.1725398
20:31:30	0:28:41	71.8813582	69.846326	78.421441	66.1725398
20:31:31	0:28:42	71.8813582	69.846326	79.421441	67.1725398
20:31:32	0:28:43	70.8813582	74.846326	80.421441	67.1725398
20:31:33	0:28:44	70.8813582	74.846326	83.421441	67.1725398
20:31:34	0:28:45	69.8813582	74.846326	83.421441	67.1725398
20:31:35	0:28:46	69.8813582	73.846326	83.421441	68.1725398
20:31:36	0:28:47	69.8813582	73.846326	83.421441	68.1725398
20:31:37	0:28:48	70.8813582	67.846326	83.421441	69.1725398
20:31:38	0:28:49	70.8813582	65.846326	82.421441	66.1725398
20:31:39	0:28:50	70.8813582	64.846326	80.421441	64.1725398
20:31:40	0:28:51	70.8813582	64.846326	80.421441	64.1725398
20:31:41	0:28:52	71.8813582	63.846326	80.421441	65.1725398
20:31:42	0:28:53	71.8813582	63.846326	80.421441	67.1725398
20:31:43	0:28:54	72.8813582	62.846326	80.421441	68.1725398
20:31:44	0:28:55	73.8813582	62.846326	80.421441	69.1725398
20:31:45	0:28:56	73.8813582	62.846326	80.421441	70.1725398
20:31:46	0:28:57	73.8813582	65.846326	80.421441	71.1725398
20:31:47	0:28:58	73.8813582	66.846326	80.421441	71.1725398
20:31:48	0:28:59	73.8813582	66.846326	83.421441	71.1725398
20:31:49	0:29:00	72.8813582	65.846326	85.421441	71.1725398
20:31:50	0:29:01	71.8813582	65.846326	86.421441	72.1725398
20:31:51	0:29:02	71.8813582	64.846326	86.421441	71.1725398
20:31:52	0:29:03	72.8813582	66.846326	86.421441	70.1725398
20:31:53	0:29:04	73.8813582	67.846326	86.421441	70.1725398
20:31:54	0:29:05	73.8813582	69.846326	86.421441	70.1725398
20:31:55	0:29:06	74.8813582	69.846326	87.421441	70.1725398
20:31:56	0:29:07	75.8813582	70.846326	87.421441	69.1725398
20:31:57	0:29:08	76.8813582	70.846326	86.421441	68.1725398
20:31:58	0:29:09	77.8813582	70.846326	80.421441	68.1725398
20:31:59	0:29:10	77.8813582	70.846326	80.421441	68.1725398
20:32:00	0:29:11	77.8813582	69.846326	77.421441	69.1725398
20:32:01	0:29:12	77.8813582	68.846326	71.421441	69.1725398

20:32:02	0:29:13	78.8813582	67.846326	71.421441	71.1725398
20:32:03	0:29:14	80.8813582	67.846326	70.421441	71.1725398
20:32:04	0:29:15	87.8813582	67.846326	81.421441	71.1725398
20:32:05	0:29:16	97.8813582	67.846326	86.421441	71.1725398
20:32:06	0:29:17	97.8813582	67.846326	86.421441	71.1725398
20:32:07	0:29:18	101.881358	67.846326	88.421441	73.1725398
20:32:08	0:29:19	101.881358	67.846326	89.421441	75.1725398
20:32:09	0:29:20	101.881358	67.846326	88.421441	75.1725398
20:32:10	0:29:21	102.881358	67.846326	88.421441	75.1725398
20:32:11	0:29:22	102.881358	68.846326	85.421441	79.1725398
20:32:12	0:29:23	102.881358	68.846326	84.421441	79.1725398
20:32:13	0:29:24	83.0192881	68.846326	82.421441	78.1725398
20:32:14	0:29:25	104.881358	67.846326	79.421441	78.1725398
20:32:15	0:29:26	80.8526214	66.846326	78.421441	78.1725398
20:32:16	0:29:27	80.5192881	64.846326	78.421441	78.1725398
20:32:17	0:29:28	80.5192881	64.846326	77.421441	78.1725398
20:32:18	0:29:29	82.0192881	64.846326	75.421441	80.1725398
20:32:19	0:29:30	82.8526214	64.846326	74.421441	82.1725398
20:32:20	0:29:31	83.0192881	64.846326	73.421441	82.1725398
20:32:21	0:29:32	83.1859547	64.846326	73.421441	82.1725398
20:32:22	0:29:33	83.6859547	64.846326	73.421441	83.1725398
20:32:23	0:29:34	84.5192881	66.846326	74.421441	83.1725398
20:32:24	0:29:35	84.5192881	68.846326	74.421441	82.1725398
20:32:25	0:29:36	87.0192881	68.846326	77.421441	86.1725398
20:32:26	0:29:37	90.0192881	68.846326	79.421441	93.1725398
20:32:27	0:29:38	92.1859547	67.846326	79.421441	100.17254
20:32:28	0:29:39	93.6859547	67.846326	84.421441	100.17254
20:32:29	0:29:40	85.6859547	67.846326	84.421441	79.1725398
20:32:30	0:29:41	84.1859547	65.846326	83.421441	77.1725398
20:32:31	0:29:42	83.6859547	65.846326	82.421441	77.1725398
20:32:32	0:29:43	83.5192881	65.846326	82.421441	77.1725398
20:32:33	0:29:44	82.3526214	65.846326	80.421441	77.1725398
20:32:34	0:29:45	83.1859547	65.846326	79.421441	81.1725398
20:32:35	0:29:46	82.3526214	65.846326	77.421441	81.1725398
20:32:36	0:29:47	83.5192881	66.846326	77.421441	84.1725398
20:32:37	0:29:48	82.3526214	67.846326	74.421441	84.1725398
20:32:38	0:29:49	78.1859547	67.846326	72.421441	74.1725398
20:32:39	0:29:50	77.3526214	70.846326	70.421441	72.1725398
20:32:40	0:29:51	76.5192881	70.846326	68.421441	72.1725398
20:32:41	0:29:52	76.8526214	71.846326	68.421441	72.1725398
20:32:42	0:29:53	76.0192881	72.846326	67.421441	70.1725398
20:32:43	0:29:54	76.0192881	72.846326	70.421441	70.1725398
20:32:44	0:29:55	75.1859547	69.846326	70.421441	70.1725398
20:32:45	0:29:56	99.8813582	69.846326	83.421441	69.1725398
20:32:46	0:29:57	97.8813582	68.846326	85.421441	68.1725398

20:32:47	0:29:58	88.8813582	68.846326	85.421441	68.1725398
20:32:48	0:29:59	83.8813582	67.846326	85.421441	66.1725398
20:32:49	0:30:00	80.8813582	67.846326	85.421441	66.1725398
20:32:50	0:30:01	77.8813582	67.846326	85.421441	66.1725398
20:32:51	0:30:02	74.8813582	67.846326	84.421441	66.1725398
20:32:52	0:30:03	73.8813582	67.846326	84.421441	66.1725398
20:32:53	0:30:04	74.8813582	67.846326	85.421441	66.1725398
20:32:54	0:30:05	79.8813582	67.846326	86.421441	66.1725398
20:32:55	0:30:06	84.8813582	67.846326	86.421441	66.1725398
20:32:56	0:30:07	86.8813582	67.846326	87.421441	67.1725398
20:32:57	0:30:08	88.8813582	67.846326	88.421441	69.1725398
20:32:58	0:30:09	93.8813582	66.846326	88.421441	73.1725398
20:32:59	0:30:10	98.8813582	66.846326	92.421441	74.1725398
20:33:00	0:30:11	98.8813582	66.846326	92.421441	74.1725398
20:33:01	0:30:12	102.881358	66.846326	94.421441	72.1725398
20:33:02	0:30:13	103.881358	66.846326	93.421441	71.1725398
20:33:03	0:30:14	103.881358	66.846326	92.421441	71.1725398
20:33:04	0:30:15	103.881358	66.846326	91.421441	71.1725398
20:33:05	0:30:16	102.881358	66.846326	91.421441	72.1725398
20:33:06	0:30:17	103.881358	66.846326	91.421441	72.1725398
20:33:07	0:30:18	104.881358	66.846326	91.421441	73.1725398
20:33:08	0:30:19	104.881358	66.846326	91.421441	75.1725398
20:33:09	0:30:20	103.881358	71.846326	88.421441	75.1725398
20:33:10	0:30:21	102.881358	72.846326	88.421441	75.1725398
20:33:11	0:30:22	100.881358	72.846326	88.421441	75.1725398
20:33:12	0:30:23	98.8813582	72.846326	87.421441	75.1725398
20:33:13	0:30:24	96.8813582	76.846326	87.421441	75.1725398
20:33:14	0:30:25	96.8813582	76.846326	86.421441	75.1725398
20:33:15	0:30:26	95.8813582	76.846326	86.421441	75.1725398
20:33:16	0:30:27	83.8813582	73.846326	86.421441	77.1725398
20:33:17	0:30:28	83.8813582	70.846326	86.421441	76.1725398
20:33:18	0:30:29	74.8813582	70.846326	86.421441	76.1725398
20:33:19	0:30:30	70.8813582	69.846326	86.421441	75.1725398
20:33:20	0:30:31	68.8813582	68.846326	90.421441	75.1725398
20:33:21	0:30:32	67.8813582	68.846326	95.421441	77.1725398
20:33:22	0:30:33	66.8813582	68.846326	95.421441	79.1725398
20:33:23	0:30:34	66.8813582	67.846326	95.421441	79.1725398
20:33:24	0:30:35	66.8813582	65.846326	95.421441	80.1725398
20:33:25	0:30:36	66.8813582	65.846326	95.421441	81.1725398
20:33:26	0:30:37	66.8813582	65.846326	95.421441	81.1725398
20:33:27	0:30:38	67.8813582	65.846326	95.421441	81.1725398
20:33:28	0:30:39	67.8813582	65.846326	95.421441	81.1725398
20:33:29	0:30:40	67.8813582	65.846326	95.421441	82.1725398
20:33:30	0:30:41	67.8813582	65.846326	96.421441	82.1725398
20:33:31	0:30:42	67.8813582	65.846326	96.421441	82.1725398

20:33:32	0:30:43	68.8813582	65.846326	96.421441	81.1725398
20:33:33	0:30:44	69.8813582	65.846326	96.421441	79.1725398
20:33:34	0:30:45	71.8813582	65.846326	96.421441	78.1725398
20:33:35	0:30:46	72.8813582	65.846326	95.421441	77.1725398
20:33:36	0:30:47	70.8813582	65.846326	94.421441	77.1725398
20:33:37	0:30:48	69.8813582	66.846326	95.421441	77.1725398
20:33:38	0:30:49	68.8813582	66.846326	95.421441	75.1725398
20:33:39	0:30:50	68.8813582	66.846326	96.421441	75.1725398
20:33:40	0:30:51	68.8813582	66.846326	98.421441	75.1725398
20:33:41	0:30:52	69.8813582	64.846326	98.421441	75.1725398
20:33:42	0:30:53	69.8813582	64.846326	98.421441	75.1725398
20:33:43	0:30:54	69.8813582	64.846326	98.421441	75.1725398
20:33:44	0:30:55	69.8813582	64.846326	98.421441	75.1725398
20:33:45	0:30:56	69.8813582	66.846326	95.421441	77.1725398
20:33:46	0:30:57	69.8813582	66.846326	95.421441	78.1725398
20:33:47	0:30:58	71.8813582	66.846326	95.421441	79.1725398
20:33:48	0:30:59	71.8813582	65.846326	95.421441	79.1725398
20:33:49	0:31:00	72.8813582	66.846326	95.421441	81.1725398
20:33:50	0:31:01	73.8813582	67.846326	95.421441	81.1725398
20:33:51	0:31:02	74.8813582	67.846326	95.421441	81.1725398
20:33:52	0:31:03	74.8813582	67.846326	95.421441	78.1725398
20:33:53	0:31:04	75.8813582	67.846326	95.421441	78.1725398
20:33:54	0:31:05	75.8813582	67.846326	95.421441	78.1725398
20:33:55	0:31:06	80.8813582	67.846326	95.421441	73.1725398
20:33:56	0:31:07	81.8813582	66.846326	88.421441	74.1725398
20:33:57	0:31:08	82.8813582	66.846326	87.421441	74.1725398
20:33:58	0:31:09	82.8813582	66.846326	86.421441	74.1725398
20:33:59	0:31:10	82.8813582	66.846326	86.421441	74.1725398
20:34:00	0:31:11	79.8813582	66.846326	86.421441	75.1725398
20:34:01	0:31:12	78.8813582	66.846326	86.421441	75.1725398
20:34:02	0:31:13	78.8813582	66.846326	89.421441	74.1725398
20:34:03	0:31:14	77.8813582	66.846326	89.421441	74.1725398
20:34:04	0:31:15	75.8813582	66.846326	89.421441	73.1725398
20:34:05	0:31:16	75.8813582	66.846326	89.421441	73.1725398
20:34:06	0:31:17	72.8813582	66.846326	89.421441	73.1725398
20:34:07	0:31:18	70.8813582	66.846326	91.421441	73.1725398
20:34:08	0:31:19	71.8813582	66.846326	91.421441	73.1725398
20:34:09	0:31:20	72.8813582	66.846326	92.421441	73.1725398
20:34:10	0:31:21	73.8813582	66.846326	92.421441	73.1725398
20:34:11	0:31:22	75.8813582	65.846326	97.421441	73.1725398
20:34:12	0:31:23	77.8813582	65.846326	97.421441	79.1725398
20:34:13	0:31:24	77.8813582	67.846326	97.421441	85.1725398
20:34:14	0:31:25	79.8813582	68.846326	97.421441	86.1725398
20:34:15	0:31:26	79.8813582	68.846326	97.421441	86.1725398
20:34:16	0:31:27	80.8813582	67.846326	97.421441	86.1725398

20:34:17	0:31:28	80.8813582	67.846326	97.421441	87.1725398
20:34:18	0:31:29	81.8813582	67.846326	96.421441	87.1725398
20:34:19	0:31:30	79.8813582	65.846326	94.421441	87.1725398
20:34:20	0:31:31	78.8813582	66.846326	94.421441	88.1725398
20:34:21	0:31:32	77.8813582	66.846326	91.421441	88.1725398
20:34:22	0:31:33	77.8813582	66.846326	87.421441	88.1725398
20:34:23	0:31:34	76.8813582	66.846326	87.421441	86.1725398
20:34:24	0:31:35	76.8813582	66.846326	85.421441	84.1725398
20:34:25	0:31:36	76.8813582	66.846326	85.421441	83.1725398
20:34:26	0:31:37	77.8813582	67.846326	82.421441	82.1725398
20:34:27	0:31:38	76.8813582	69.846326	81.421441	80.1725398
20:34:28	0:31:39	75.8813582	71.846326	80.421441	79.1725398
20:34:29	0:31:40	73.8813582	71.846326	80.421441	79.1725398
20:34:30	0:31:41	73.8813582	72.846326	79.421441	79.1725398
20:34:31	0:31:42	71.8813582	72.846326	78.421441	79.1725398
20:34:32	0:31:43	69.8813582	72.846326	78.421441	79.1725398
20:34:33	0:31:44	68.8813582	72.846326	78.421441	78.1725398
20:34:34	0:31:45	68.8813582	72.846326	79.421441	78.1725398
20:34:35	0:31:46	68.8813582	71.846326	79.421441	77.1725398
20:34:36	0:31:47	68.8813582	71.846326	79.421441	75.1725398
20:34:37	0:31:48	69.8813582	70.846326	80.421441	73.1725398
20:34:38	0:31:49	69.8813582	70.846326	80.421441	73.1725398
20:34:39	0:31:50	70.8813582	69.846326	81.421441	73.1725398
20:34:40	0:31:51	70.8813582	67.846326	80.421441	73.1725398
20:34:41	0:31:52	70.8813582	67.846326	79.421441	75.1725398
20:34:42	0:31:53	70.8813582	66.846326	79.421441	76.1725398
20:34:43	0:31:54	69.8813582	66.846326	79.421441	76.1725398
20:34:44	0:31:55	69.8813582	65.846326	79.421441	78.1725398
20:34:45	0:31:56	69.8813582	65.846326	80.421441	78.1725398
20:34:46	0:31:57	70.8813582	64.846326	80.421441	79.1725398
20:34:47	0:31:58	70.8813582	64.846326	79.421441	79.1725398
20:34:48	0:31:59	70.8813582	64.846326	79.421441	79.1725398
20:34:49	0:32:00	72.8813582	64.846326	80.421441	75.1725398
20:34:50	0:32:01	73.8813582	65.846326	80.421441	74.1725398
20:34:51	0:32:02	73.8813582	65.846326	81.421441	74.1725398
20:34:52	0:32:03	71.8813582	67.846326	81.421441	74.1725398
20:34:53	0:32:04	70.8813582	66.846326	81.421441	73.1725398
20:34:54	0:32:05	70.8813582	65.846326	77.421441	73.1725398
20:34:55	0:32:06	69.8813582	63.846326	75.421441	72.1725398
20:34:56	0:32:07	69.8813582	61.846326	68.421441	72.1725398
20:34:57	0:32:08	69.8813582	61.846326	66.421441	72.1725398
20:34:58	0:32:09	69.8813582	60.846326	61.421441	72.1725398
20:34:59	0:32:10	70.8813582	57.846326	60.421441	70.1725398
20:35:00	0:32:11	70.8813582	56.846326	60.421441	70.1725398
20:35:01	0:32:12	71.8813582	56.846326	60.421441	70.1725398

20:35:02	0:32:13	77.8813582	65.846326	59.421441	70.1725398
20:35:03	0:32:14	75.8813582	72.846326	59.421441	70.1725398
20:35:04	0:32:15	73.8813582	79.846326	59.421441	70.1725398
20:35:05	0:32:16	72.8813582	79.846326	59.421441	70.1725398
20:35:06	0:32:17	69.8813582	79.846326	61.421441	69.1725398
20:35:07	0:32:18	69.8813582	79.846326	63.421441	69.1725398
20:35:08	0:32:19	67.8813582	79.846326	63.421441	69.1725398
20:35:09	0:32:20	66.8813582	79.846326	63.421441	69.1725398
20:35:10	0:32:21	66.8813582	79.846326	65.421441	70.1725398
20:35:11	0:32:22	66.8813582	82.846326	65.421441	71.1725398
20:35:12	0:32:23	67.8813582	82.846326	65.421441	71.1725398
20:35:13	0:32:24	67.8813582	81.846326	66.421441	70.1725398
20:35:14	0:32:25	67.8813582	81.846326	66.421441	69.1725398
20:35:15	0:32:26	66.8813582	81.846326	66.421441	69.1725398
20:35:16	0:32:27	66.8813582	81.846326	64.421441	69.1725398
20:35:17	0:32:28	66.8813582	81.846326	64.421441	69.1725398
20:35:18	0:32:29	66.8813582	81.846326	64.421441	69.1725398
20:35:19	0:32:30	67.8813582	81.846326	63.421441	69.1725398
20:35:20	0:32:31	67.8813582	79.846326	63.421441	69.1725398
20:35:21	0:32:32	68.8813582	72.846326	63.421441	69.1725398
20:35:22	0:32:33	69.8813582	70.846326	63.421441	69.1725398
20:35:23	0:32:34	70.8813582	68.846326	64.421441	69.1725398
20:35:24	0:32:35	71.8813582	68.846326	64.421441	70.1725398
20:35:25	0:32:36	71.8813582	68.846326	65.421441	71.1725398
20:35:26	0:32:37	71.8813582	68.846326	65.421441	79.1725398
20:35:27	0:32:38	74.8813582	69.846326	65.421441	79.1725398
20:35:28	0:32:39	74.8813582	70.846326	65.421441	82.1725398
20:35:29	0:32:40	76.8813582	71.846326	66.421441	82.1725398
20:35:30	0:32:41	76.8813582	71.846326	67.421441	82.1725398
20:35:31	0:32:42	75.8813582	71.846326	67.421441	82.1725398
20:35:32	0:32:43	74.8813582	71.846326	67.421441	82.1725398
20:35:33	0:32:44	72.8813582	70.846326	67.421441	82.1725398
20:35:34	0:32:45	72.8813582	70.846326	66.421441	81.1725398
20:35:35	0:32:46	72.8813582	70.846326	66.421441	81.1725398
20:35:36	0:32:47	71.8813582	71.846326	66.421441	81.1725398
20:35:37	0:32:48	69.8813582	69.846326	66.421441	81.1725398
20:35:38	0:32:49	69.8813582	69.846326	66.421441	81.1725398
20:35:39	0:32:50	69.8813582	68.846326	66.421441	81.1725398
20:35:40	0:32:51	68.8813582	67.846326	67.421441	80.1725398
20:35:41	0:32:52	69.8813582	67.846326	68.421441	79.1725398
20:35:42	0:32:53	69.8813582	67.846326	68.421441	79.1725398
20:35:43	0:32:54	69.8813582	64.846326	71.421441	76.1725398
20:35:44	0:32:55	69.8813582	64.846326	71.421441	75.1725398
20:35:45	0:32:56	68.8813582	63.846326	73.421441	75.1725398
20:35:46	0:32:57	68.8813582	63.846326	74.421441	74.1725398

20:35:47	0:32:58	67.8813582	64.846326	74.421441	75.1725398
20:35:48	0:32:59	67.8813582	65.846326	74.421441	76.1725398
20:35:49	0:33:00	66.8813582	65.846326	74.421441	76.1725398
20:35:50	0:33:01	66.8813582	66.846326	74.421441	75.1725398
20:35:51	0:33:02	66.8813582	66.846326	74.421441	74.1725398
20:35:52	0:33:03	67.8813582	66.846326	77.421441	73.1725398
20:35:53	0:33:04	67.8813582	66.846326	77.421441	71.1725398
20:35:54	0:33:05	68.8813582	66.846326	77.421441	70.1725398
20:35:55	0:33:06	69.8813582	67.846326	77.421441	70.1725398
20:35:56	0:33:07	69.8813582	69.846326	77.421441	74.1725398
20:35:57	0:33:08	71.8813582	69.846326	77.421441	74.1725398
20:35:58	0:33:09	72.8813582	70.846326	77.421441	74.1725398
20:35:59	0:33:10	72.8813582	69.846326	77.421441	73.1725398
20:36:00	0:33:11	72.8813582	69.846326	78.421441	73.1725398
20:36:01	0:33:12	72.8813582	68.846326	78.421441	74.1725398
20:36:02	0:33:13	73.8813582	68.846326	78.421441	75.1725398
20:36:03	0:33:14	73.8813582	68.846326	81.421441	77.1725398
20:36:04	0:33:15	74.8813582	68.846326	81.421441	78.1725398
20:36:05	0:33:16	74.8813582	68.846326	81.421441	78.1725398
20:36:06	0:33:17	75.8813582	68.846326	81.421441	78.1725398
20:36:07	0:33:18	76.8813582	68.846326	82.421441	78.1725398
20:36:08	0:33:19	76.8813582	68.846326	82.421441	77.1725398
20:36:09	0:33:20	77.8813582	67.846326	85.421441	76.1725398
20:36:10	0:33:21	77.8813582	76.846326	85.421441	76.1725398
20:36:11	0:33:22	77.8813582	77.846326	85.421441	75.1725398
20:36:12	0:33:23	77.8813582	77.846326	85.421441	76.1725398
20:36:13	0:33:24	77.8813582	78.846326	86.421441	77.1725398
20:36:14	0:33:25	77.8813582	79.846326	86.421441	77.1725398
20:36:15	0:33:26	76.8813582	79.846326	87.421441	77.1725398
20:36:16	0:33:27	76.8813582	79.846326	87.421441	77.1725398
20:36:17	0:33:28	73.8813582	77.846326	87.421441	77.1725398
20:36:18	0:33:29	72.8813582	75.846326	87.421441	77.1725398
20:36:19	0:33:30	71.8813582	73.846326	86.421441	81.1725398
20:36:20	0:33:31	71.8813582	71.846326	86.421441	80.1725398
20:36:21	0:33:32	71.8813582	71.846326	86.421441	79.1725398
20:36:22	0:33:33	71.8813582	67.846326	86.421441	79.1725398
20:36:23	0:33:34	71.8813582	67.846326	92.421441	77.1725398
20:36:24	0:33:35	72.8813582	66.846326	95.421441	76.1725398
20:36:25	0:33:36	71.8813582	65.846326	94.421441	74.1725398
20:36:26	0:33:37	70.8813582	65.846326	94.421441	74.1725398
20:36:27	0:33:38	70.8813582	64.846326	94.421441	74.1725398
20:36:28	0:33:39	70.8813582	64.846326	93.421441	74.1725398
20:36:29	0:33:40	69.8813582	64.846326	93.421441	74.1725398
20:36:30	0:33:41	68.8813582	67.846326	93.421441	74.1725398
20:36:31	0:33:42	68.8813582	67.846326	92.421441	75.1725398

20:36:32	0:33:43	67.8813582	67.846326	90.421441	75.1725398
20:36:33	0:33:44	67.8813582	67.846326	90.421441	74.1725398
20:36:34	0:33:45	66.8813582	68.846326	89.421441	71.1725398
20:36:35	0:33:46	66.8813582	69.846326	89.421441	70.1725398
20:36:36	0:33:47	66.8813582	69.846326	89.421441	70.1725398
20:36:37	0:33:48	66.8813582	69.846326	88.421441	70.1725398
20:36:38	0:33:49	66.8813582	71.846326	88.421441	73.1725398
20:36:39	0:33:50	66.8813582	70.846326	88.421441	73.1725398
20:36:40	0:33:51	65.8813582	69.846326	88.421441	74.1725398
20:36:41	0:33:52	65.8813582	71.846326	88.421441	74.1725398
20:36:42	0:33:53	65.8813582	72.846326	88.421441	74.1725398
20:36:43	0:33:54	66.8813582	74.846326	88.421441	73.1725398
20:36:44	0:33:55	66.8813582	76.846326	87.421441	72.1725398
20:36:45	0:33:56	66.8813582	76.846326	87.421441	71.1725398
20:36:46	0:33:57	66.8813582	77.846326	87.421441	69.1725398
20:36:47	0:33:58	66.8813582	76.846326	87.421441	68.1725398
20:36:48	0:33:59	65.8813582	74.846326	87.421441	68.1725398
20:36:49	0:34:00	66.8813582	69.846326	87.421441	68.1725398
20:36:50	0:34:01	66.8813582	69.846326	87.421441	68.1725398
20:36:51	0:34:02	66.8813582	68.846326	87.421441	68.1725398
20:36:52	0:34:03	66.8813582	67.846326	87.421441	67.1725398
20:36:53	0:34:04	66.8813582	69.846326	87.421441	67.1725398
20:36:54	0:34:05	66.8813582	69.846326	87.421441	67.1725398
20:36:55	0:34:06	66.8813582	69.846326	88.421441	66.1725398
20:36:56	0:34:07	66.8813582	69.846326	88.421441	66.1725398
20:36:57	0:34:08	67.8813582	69.846326	88.421441	66.1725398
20:36:58	0:34:09	67.8813582	69.846326	88.421441	66.1725398
20:36:59	0:34:10	67.8813582	67.846326	88.421441	66.1725398
20:37:00	0:34:11	69.8813582	65.846326	88.421441	65.1725398
20:37:01	0:34:12	68.8813582	65.846326	89.421441	65.1725398
20:37:02	0:34:13	68.8813582	64.846326	90.421441	67.1725398
20:37:03	0:34:14	69.8813582	64.846326	95.421441	67.1725398
20:37:04	0:34:15	69.8813582	65.846326	96.421441	67.1725398
20:37:05	0:34:16	69.8813582	65.846326	96.421441	67.1725398
20:37:06	0:34:17	69.8813582	65.846326	98.421441	67.1725398
20:37:07	0:34:18	70.8813582	65.846326	99.421441	67.1725398
20:37:08	0:34:19	70.8813582	64.846326	99.421441	67.1725398
20:37:09	0:34:20	69.8813582	64.846326	76.2953836	67.1725398
20:37:10	0:34:21	69.8813582	64.846326	76.2953836	67.1725398
20:37:11	0:34:22	69.8813582	65.846326	97.421441	70.1725398
20:37:12	0:34:23	69.8813582	65.846326	97.421441	71.1725398
20:37:13	0:34:24	68.8813582	67.846326	89.421441	71.1725398
20:37:14	0:34:25	68.8813582	67.846326	89.421441	71.1725398
20:37:15	0:34:26	69.8813582	68.846326	87.421441	71.1725398
20:37:16	0:34:27	69.8813582	70.846326	90.421441	69.1725398

20:37:17	0:34:28	68.8813582	70.846326	91.421441	68.1725398
20:37:18	0:34:29	68.8813582	74.846326	91.421441	69.1725398
20:37:19	0:34:30	69.8813582	73.846326	90.421441	70.1725398
20:37:20	0:34:31	69.8813582	73.846326	87.421441	70.1725398
20:37:21	0:34:32	69.8813582	72.846326	86.421441	70.1725398
20:37:22	0:34:33	69.8813582	71.846326	84.421441	72.1725398
20:37:23	0:34:34	69.8813582	71.846326	83.421441	71.1725398
20:37:24	0:34:35	69.8813582	71.846326	83.421441	71.1725398
20:37:25	0:34:36	69.8813582	70.846326	83.421441	71.1725398
20:37:26	0:34:37	69.8813582	70.846326	83.421441	71.1725398
20:37:27	0:34:38	69.8813582	70.846326	83.421441	71.1725398
20:37:28	0:34:39	70.8813582	69.846326	83.421441	71.1725398
20:37:29	0:34:40	71.8813582	68.846326	83.421441	75.1725398
20:37:30	0:34:41	71.8813582	68.846326	83.421441	75.1725398
20:37:31	0:34:42	70.8813582	70.846326	83.421441	80.1725398
20:37:32	0:34:43	69.8813582	70.846326	83.421441	80.1725398
20:37:33	0:34:44	68.8813582	70.846326	88.421441	83.1725398
20:37:34	0:34:45	68.8813582	71.846326	92.421441	83.1725398
20:37:35	0:34:46	68.8813582	71.846326	94.421441	83.1725398
20:37:36	0:34:47	68.8813582	73.846326	95.421441	82.1725398
20:37:37	0:34:48	69.8813582	73.846326	98.421441	79.1725398
20:37:38	0:34:49	69.8813582	71.846326	98.421441	78.1725398
20:37:39	0:34:50	69.8813582	69.846326	97.421441	76.1725398
20:37:40	0:34:51	70.8813582	68.846326	93.421441	76.1725398
20:37:41	0:34:52	71.8813582	68.846326	92.421441	73.1725398
20:37:42	0:34:53	71.8813582	67.846326	91.421441	71.1725398
20:37:43	0:34:54	72.8813582	67.846326	90.421441	71.1725398
20:37:44	0:34:55	72.8813582	67.846326	89.421441	72.1725398
20:37:45	0:34:56	72.8813582	67.846326	89.421441	72.1725398
20:37:46	0:34:57	71.8813582	67.846326	89.421441	73.1725398
20:37:47	0:34:58	71.8813582	68.846326	89.421441	74.1725398
20:37:48	0:34:59	71.8813582	68.846326	90.421441	73.1725398
20:37:49	0:35:00	71.8813582	68.846326	91.421441	73.1725398
20:37:50	0:35:01	71.8813582	68.846326	91.421441	73.1725398
20:37:51	0:35:02	70.8813582	69.846326	91.421441	73.1725398
20:37:52	0:35:03	70.8813582	69.846326	91.421441	77.1725398
20:37:53	0:35:04	70.8813582	71.846326	91.421441	79.1725398
20:37:54	0:35:05	70.8813582	71.846326	91.421441	80.1725398
20:37:55	0:35:06	70.8813582	70.846326	91.421441	80.1725398
20:37:56	0:35:07	70.8813582	69.846326	91.421441	81.1725398
20:37:57	0:35:08	71.8813582	70.846326	91.421441	81.1725398
20:37:58	0:35:09	71.8813582	70.846326	90.421441	78.1725398
20:37:59	0:35:10	71.8813582	70.846326	89.421441	76.1725398
20:38:00	0:35:11	72.8813582	70.846326	89.421441	76.1725398
20:38:01	0:35:12	72.8813582	71.846326	88.421441	75.1725398

20:38:02	0:35:13	73.8813582	71.846326	88.421441	75.1725398
20:38:03	0:35:14	73.8813582	71.846326	87.421441	75.1725398
20:38:04	0:35:15	72.8813582	71.846326	87.421441	78.1725398
20:38:05	0:35:16	71.8813582	70.846326	87.421441	80.1725398
20:38:06	0:35:17	71.8813582	70.846326	87.421441	80.1725398
20:38:07	0:35:18	71.8813582	68.846326	87.421441	80.1725398
20:38:08	0:35:19	70.8813582	68.846326	88.421441	79.1725398
20:38:09	0:35:20	70.8813582	68.846326	88.421441	79.1725398
20:38:10	0:35:21	70.8813582	67.846326	89.421441	79.1725398
20:38:11	0:35:22	70.8813582	67.846326	89.421441	78.1725398
20:38:12	0:35:23	71.8813582	67.846326	89.421441	77.1725398
20:38:13	0:35:24	72.8813582	67.846326	89.421441	75.1725398
20:38:14	0:35:25	72.8813582	68.846326	89.421441	75.1725398
20:38:15	0:35:26	72.8813582	69.846326	89.421441	75.1725398
20:38:16	0:35:27	73.8813582	71.846326	89.421441	74.1725398
20:38:17	0:35:28	72.8813582	71.846326	89.421441	74.1725398
20:38:18	0:35:29	72.8813582	71.846326	89.421441	70.1725398
20:38:19	0:35:30	72.8813582	70.846326	89.421441	69.1725398
20:38:20	0:35:31	72.8813582	68.846326	89.421441	68.1725398
20:38:21	0:35:32	72.8813582	68.846326	90.421441	69.1725398
20:38:22	0:35:33	72.8813582	68.846326	90.421441	69.1725398
20:38:23	0:35:34	72.8813582	69.846326	90.421441	69.1725398
20:38:24	0:35:35	72.8813582	69.846326	90.421441	69.1725398
20:38:25	0:35:36	72.8813582	72.846326	89.421441	69.1725398
20:38:26	0:35:37	73.8813582	73.846326	89.421441	69.1725398
20:38:27	0:35:38	74.8813582	73.846326	89.421441	69.1725398
20:38:28	0:35:39	74.8813582	73.846326	89.421441	73.1725398
20:38:29	0:35:40	74.8813582	74.846326	89.421441	73.1725398
20:38:30	0:35:41	74.8813582	74.846326	88.421441	73.1725398
20:38:31	0:35:42	74.8813582	73.846326	88.421441	76.1725398
20:38:32	0:35:43	74.8813582	72.846326	88.421441	74.1725398
20:38:33	0:35:44	75.8813582	70.846326	88.421441	73.1725398
20:38:34	0:35:45	76.8813582	69.846326	88.421441	74.1725398
20:38:35	0:35:46	73.8813582	69.846326	88.421441	74.1725398
20:38:36	0:35:47	72.8813582	69.846326	88.421441	75.1725398
20:38:37	0:35:48	72.8813582	68.846326	88.421441	75.1725398
20:38:38	0:35:49	72.8813582	68.846326	88.421441	76.1725398
20:38:39	0:35:50	72.8813582	71.846326	85.421441	75.1725398
20:38:40	0:35:51	72.8813582	71.846326	85.421441	75.1725398
20:38:41	0:35:52	72.8813582	71.846326	84.421441	75.1725398
20:38:42	0:35:53	72.8813582	72.846326	84.421441	75.1725398
20:38:43	0:35:54	72.8813582	72.846326	83.421441	75.1725398
20:38:44	0:35:55	72.8813582	72.846326	83.421441	75.1725398
20:38:45	0:35:56	73.8813582	72.846326	83.421441	75.1725398
20:38:46	0:35:57	73.8813582	72.846326	82.421441	76.1725398

20:38:47	0:35:58	74.8813582	72.846326	79.421441	74.1725398
20:38:48	0:35:59	74.8813582	72.846326	79.421441	70.1725398
20:38:49	0:36:00	75.8813582	72.846326	79.421441	69.1725398
20:38:50	0:36:01	76.8813582	72.846326	79.421441	69.1725398
20:38:51	0:36:02	77.8813582	72.846326	79.421441	69.1725398
20:38:52	0:36:03	78.8813582	71.846326	79.421441	69.1725398
20:38:53	0:36:04	79.8813582	71.846326	78.421441	69.1725398
20:38:54	0:36:05	78.8813582	71.846326	78.421441	69.1725398
20:38:55	0:36:06	77.8813582	70.846326	78.421441	69.1725398
20:38:56	0:36:07	78.8813582	69.846326	78.421441	68.1725398
20:38:57	0:36:08	79.8813582	67.846326	79.421441	66.1725398
20:38:58	0:36:09	79.8813582	67.846326	79.421441	66.1725398
20:38:59	0:36:10	82.8813582	67.846326	79.421441	66.1725398
20:39:00	0:36:11	83.8813582	67.846326	79.421441	66.1725398
20:39:01	0:36:12	83.8813582	67.846326	80.421441	66.1725398
20:39:02	0:36:13	89.8813582	66.846326	80.421441	66.1725398
20:39:03	0:36:14	91.8813582	66.846326	80.421441	66.1725398
20:39:04	0:36:15	95.8813582	67.846326	80.421441	66.1725398
20:39:05	0:36:16	79.8953836	67.846326	79.421441	66.1725398
20:39:06	0:36:17	80.0953836	67.846326	79.421441	66.1725398
20:39:07	0:36:18	80.6953836	68.846326	78.421441	67.1725398
20:39:08	0:36:19	81.0953836	68.846326	78.421441	68.1725398
20:39:09	0:36:20	80.6953836	68.846326	76.421441	68.1725398
20:39:10	0:36:21	80.6953836	68.846326	76.421441	68.1725398
20:39:11	0:36:22	81.8953836	68.846326	76.421441	70.1725398
20:39:12	0:36:23	96.8813582	70.846326	76.421441	73.1725398
20:39:13	0:36:24	93.8813582	70.846326	76.421441	73.1725398
20:39:14	0:36:25	83.8813582	72.846326	77.421441	69.1725398
20:39:15	0:36:26	81.8813582	71.846326	77.421441	69.1725398
20:39:16	0:36:27	79.8813582	71.846326	77.421441	67.1725398
20:39:17	0:36:28	78.8813582	71.846326	77.421441	66.1725398
20:39:18	0:36:29	78.8813582	71.846326	77.421441	66.1725398
20:39:19	0:36:30	76.8813582	70.846326	78.421441	66.1725398
20:39:20	0:36:31	76.8813582	70.846326	79.421441	65.1725398
20:39:21	0:36:32	75.8813582	70.846326	79.421441	65.1725398
20:39:22	0:36:33	74.8813582	70.846326	80.421441	65.1725398
20:39:23	0:36:34	74.8813582	70.846326	80.421441	65.1725398
20:39:24	0:36:35	74.8813582	70.846326	80.421441	65.1725398
20:39:25	0:36:36	74.8813582	70.846326	81.421441	72.1725398
20:39:26	0:36:37	75.8813582	70.846326	81.421441	73.1725398
20:39:27	0:36:38	76.8813582	69.846326	81.421441	73.1725398
20:39:28	0:36:39	76.8813582	67.846326	81.421441	73.1725398
20:39:29	0:36:40	76.8813582	67.846326	81.421441	73.1725398
20:39:30	0:36:41	74.8813582	67.846326	81.421441	73.1725398
20:39:31	0:36:42	71.8813582	67.846326	81.421441	73.1725398

20:39:32	0:36:43	70.8813582	67.846326	81.421441	73.1725398
20:39:33	0:36:44	70.8813582	67.846326	80.421441	73.1725398
20:39:34	0:36:45	70.8813582	67.846326	81.421441	72.1725398
20:39:35	0:36:46	70.8813582	68.846326	82.421441	72.1725398
20:39:36	0:36:47	70.8813582	68.846326	82.421441	74.1725398
20:39:37	0:36:48	71.8813582	69.846326	82.421441	79.1725398
20:39:38	0:36:49	71.8813582	69.846326	82.421441	80.1725398
20:39:39	0:36:50	69.8813582	69.846326	82.421441	80.1725398
20:39:40	0:36:51	69.8813582	70.846326	82.421441	79.1725398
20:39:41	0:36:52	69.8813582	70.846326	82.421441	81.1725398
20:39:42	0:36:53	68.8813582	70.846326	82.421441	82.1725398
20:39:43	0:36:54	67.8813582	70.846326	82.421441	84.1725398
20:39:44	0:36:55	67.8813582	70.846326	82.421441	84.1725398
20:39:45	0:36:56	66.8813582	70.846326	82.421441	84.1725398
20:39:46	0:36:57	66.8813582	71.846326	82.421441	83.1725398
20:39:47	0:36:58	66.8813582	72.846326	83.421441	79.1725398
20:39:48	0:36:59	67.8813582	73.846326	84.421441	79.1725398
20:39:49	0:37:00	67.8813582	73.846326	84.421441	76.1725398
20:39:50	0:37:01	66.8813582	73.846326	84.421441	77.1725398
20:39:51	0:37:02	66.8813582	73.846326	85.421441	77.1725398
20:39:52	0:37:03	66.8813582	73.846326	84.421441	78.1725398
20:39:53	0:37:04	66.8813582	72.846326	84.421441	79.1725398
20:39:54	0:37:05	66.8813582	71.846326	82.421441	78.1725398
20:39:55	0:37:06	66.8813582	71.846326	82.421441	77.1725398
20:39:56	0:37:07	66.8813582	70.846326	83.421441	74.1725398
20:39:57	0:37:08	66.8813582	71.846326	85.421441	73.1725398
20:39:58	0:37:09	66.8813582	72.846326	85.421441	73.1725398
20:39:59	0:37:10	66.8813582	72.846326	86.421441	73.1725398
20:40:00	0:37:11	66.8813582	73.846326	86.421441	72.1725398
20:40:01	0:37:12	66.8813582	75.846326	86.421441	74.1725398
20:40:02	0:37:13	66.8813582	75.846326	86.421441	75.1725398
20:40:03	0:37:14	66.8813582	75.846326	89.421441	75.1725398
20:40:04	0:37:15	66.8813582	74.846326	89.421441	73.1725398
20:40:05	0:37:16	67.8813582	73.846326	89.421441	70.1725398
20:40:06	0:37:17	67.8813582	73.846326	89.421441	70.1725398
20:40:07	0:37:18	67.8813582	72.846326	90.421441	68.1725398
20:40:08	0:37:19	68.8813582	73.846326	89.421441	68.1725398
20:40:09	0:37:20	68.8813582	73.846326	89.421441	69.1725398
20:40:10	0:37:21	68.8813582	73.846326	89.421441	70.1725398
20:40:11	0:37:22	68.8813582	73.846326	88.421441	70.1725398
20:40:12	0:37:23	68.8813582	73.846326	88.421441	70.1725398
20:40:13	0:37:24	67.8813582	74.846326	88.421441	70.1725398
20:40:14	0:37:25	68.8813582	74.846326	88.421441	71.1725398
20:40:15	0:37:26	68.8813582	74.846326	87.421441	71.1725398
20:40:16	0:37:27	69.8813582	74.846326	87.421441	72.1725398

20:40:17	0:37:28	68.8813582	74.846326	87.421441	74.1725398
20:40:18	0:37:29	67.8813582	74.846326	87.421441	74.1725398
20:40:19	0:37:30	67.8813582	74.846326	86.421441	74.1725398
20:40:20	0:37:31	69.8813582	71.846326	85.421441	74.1725398
20:40:21	0:37:32	70.8813582	71.846326	83.421441	74.1725398
20:40:22	0:37:33	71.8813582	69.846326	82.421441	74.1725398
20:40:23	0:37:34	71.8813582	67.846326	82.421441	74.1725398
20:40:24	0:37:35	72.8813582	67.846326	81.421441	73.1725398
20:40:25	0:37:36	72.8813582	66.846326	81.421441	74.1725398
20:40:26	0:37:37	72.8813582	65.846326	80.421441	73.1725398
20:40:27	0:37:38	73.8813582	64.846326	80.421441	72.1725398
20:40:28	0:37:39	72.8813582	64.846326	80.421441	72.1725398
20:40:29	0:37:40	69.8813582	64.846326	80.421441	72.1725398
20:40:30	0:37:41	68.8813582	64.846326	82.421441	72.1725398
20:40:31	0:37:42	67.8813582	64.846326	81.421441	71.1725398
20:40:32	0:37:43	67.8813582	64.846326	81.421441	70.1725398
20:40:33	0:37:44	67.8813582	64.846326	81.421441	70.1725398
20:40:34	0:37:45	67.8813582	65.846326	84.421441	70.1725398
20:40:35	0:37:46	66.8813582	66.846326	84.421441	70.1725398
20:40:36	0:37:47	66.8813582	68.846326	84.421441	70.1725398
20:40:37	0:37:48	67.8813582	68.846326	84.421441	71.1725398
20:40:38	0:37:49	67.8813582	70.846326	83.421441	71.1725398
20:40:39	0:37:50	67.8813582	74.846326	83.421441	70.1725398
20:40:40	0:37:51	69.8813582	74.846326	83.421441	69.1725398
20:40:41	0:37:52	70.8813582	74.846326	83.421441	69.1725398
20:40:42	0:37:53	71.8813582	72.846326	83.421441	69.1725398
20:40:43	0:37:54	72.8813582	70.846326	83.421441	71.1725398
20:40:44	0:37:55	70.8813582	70.846326	81.421441	72.1725398
20:40:45	0:37:56	69.8813582	70.846326	81.421441	72.1725398
20:40:46	0:37:57	67.8813582	69.846326	81.421441	74.1725398
20:40:47	0:37:58	66.8813582	69.846326	81.421441	73.1725398
20:40:48	0:37:59	66.8813582	69.846326	81.421441	73.1725398
20:40:49	0:38:00	66.8813582	71.846326	84.421441	72.1725398
20:40:50	0:38:01	66.8813582	71.846326	87.421441	71.1725398
20:40:51	0:38:02	67.8813582	71.846326	87.421441	71.1725398
20:40:52	0:38:03	67.8813582	71.846326	89.421441	71.1725398
20:40:53	0:38:04	68.8813582	71.846326	87.421441	71.1725398
20:40:54	0:38:05	68.8813582	71.846326	87.421441	72.1725398
20:40:55	0:38:06	69.8813582	71.846326	88.421441	73.1725398
20:40:56	0:38:07	69.8813582	71.846326	88.421441	72.1725398
20:40:57	0:38:08	69.8813582	71.846326	89.421441	71.1725398
20:40:58	0:38:09	69.8813582	71.846326	89.421441	71.1725398
20:40:59	0:38:10	69.8813582	72.846326	88.421441	71.1725398
20:41:00	0:38:11	68.8813582	72.846326	88.421441	70.1725398
20:41:01	0:38:12	69.8813582	73.846326	88.421441	69.1725398

20:41:02	0:38:13	70.8813582	74.846326	88.421441	68.1725398
20:41:03	0:38:14	70.8813582	75.846326	89.421441	68.1725398
20:41:04	0:38:15	71.8813582	75.846326	88.421441	67.1725398
20:41:05	0:38:16	71.8813582	75.846326	88.421441	68.1725398
20:41:06	0:38:17	71.8813582	75.846326	88.421441	68.1725398
20:41:07	0:38:18	71.8813582	72.846326	89.421441	68.1725398
20:41:08	0:38:19	71.8813582	70.846326	89.421441	68.1725398
20:41:09	0:38:20	71.8813582	69.846326	89.421441	68.1725398
20:41:10	0:38:21	70.8813582	69.846326	89.421441	68.1725398
20:41:11	0:38:22	70.8813582	67.846326	89.421441	69.1725398
20:41:12	0:38:23	69.8813582	67.846326	89.421441	68.1725398
20:41:13	0:38:24	68.8813582	67.846326	89.421441	68.1725398
20:41:14	0:38:25	68.8813582	67.846326	89.421441	68.1725398
20:41:15	0:38:26	67.8813582	67.846326	89.421441	69.1725398
20:41:16	0:38:27	67.8813582	67.846326	88.421441	68.1725398
20:41:17	0:38:28	68.8813582	66.846326	88.421441	66.1725398
20:41:18	0:38:29	69.8813582	66.846326	88.421441	66.1725398
20:41:19	0:38:30	69.8813582	63.846326	88.421441	66.1725398
20:41:20	0:38:31	69.8813582	62.846326	88.421441	67.1725398
20:41:21	0:38:32	69.8813582	62.846326	92.421441	69.1725398
20:41:22	0:38:33	69.8813582	62.846326	92.421441	71.1725398
20:41:23	0:38:34	69.8813582	61.846326	92.421441	71.1725398
20:41:24	0:38:35	69.8813582	61.846326	96.421441	71.1725398
20:41:25	0:38:36	69.8813582	61.846326	98.421441	69.1725398
20:41:26	0:38:37	68.8813582	60.846326	74.8953836	67.1725398
20:41:27	0:38:38	68.8813582	60.846326	74.4953836	66.1725398
20:41:28	0:38:39	68.8813582	60.846326	74.6953836	66.1725398
20:41:29	0:38:40	68.8813582	60.846326	74.8953836	67.1725398
20:41:30	0:38:41	69.8813582	60.846326	96.421441	67.1725398
20:41:31	0:38:42	69.8813582	61.846326	96.421441	69.1725398
20:41:32	0:38:43	69.8813582	61.846326	95.421441	68.1725398
20:41:33	0:38:44	69.8813582	71.846326	89.421441	68.1725398
20:41:34	0:38:45	69.8813582	72.846326	89.421441	66.1725398
20:41:35	0:38:46	69.8813582	72.846326	87.421441	66.1725398
20:41:36	0:38:47	69.8813582	72.846326	86.421441	66.1725398
20:41:37	0:38:48	68.8813582	74.846326	86.421441	66.1725398
20:41:38	0:38:49	67.8813582	74.846326	92.421441	66.1725398
20:41:39	0:38:50	67.8813582	77.846326	97.421441	66.1725398
20:41:40	0:38:51	67.8813582	77.846326	97.421441	67.1725398
20:41:41	0:38:52	67.8813582	79.846326	97.421441	70.1725398
20:41:42	0:38:53	67.8813582	79.846326	101.421441	72.1725398
20:41:43	0:38:54	68.8813582	76.846326	103.421441	72.1725398
20:41:44	0:38:55	68.8813582	76.846326	103.421441	71.1725398
20:41:45	0:38:56	69.8813582	73.846326	79.2953836	68.1725398
20:41:46	0:38:57	69.8813582	71.846326	78.4953836	67.1725398

20:41:47	0:38:58	68.8813582	71.846326	78.6953836	68.1725398
20:41:48	0:38:59	68.8813582	69.846326	78.6953836	69.1725398
20:41:49	0:39:00	68.8813582	69.846326	78.8953836	70.1725398
20:41:50	0:39:01	68.8813582	68.846326	78.6953836	70.1725398
20:41:51	0:39:02	68.8813582	68.846326	78.4953836	70.1725398
20:41:52	0:39:03	69.8813582	67.846326	78.2953836	70.1725398
20:41:53	0:39:04	69.8813582	67.846326	77.8953836	69.1725398
20:41:54	0:39:05	69.8813582	67.846326	101.421441	69.1725398
20:41:55	0:39:06	69.8813582	68.846326	98.421441	69.1725398
20:41:56	0:39:07	70.8813582	67.846326	98.421441	69.1725398
20:41:57	0:39:08	71.8813582	67.846326	98.421441	69.1725398
20:41:58	0:39:09	69.8813582	67.846326	98.421441	69.1725398
20:41:59	0:39:10	69.8813582	66.846326	99.421441	69.1725398
20:42:00	0:39:11	69.8813582	66.846326	99.421441	69.1725398
20:42:01	0:39:12	68.8813582	66.846326	99.421441	67.1725398
20:42:02	0:39:13	68.8813582	66.846326	76.4953836	67.1725398
20:42:03	0:39:14	68.8813582	66.846326	76.8953836	68.1725398
20:42:04	0:39:15	68.8813582	65.846326	86.421441	71.1725398
20:42:05	0:39:16	67.8813582	65.846326	86.421441	72.1725398
20:42:06	0:39:17	67.8813582	65.846326	85.421441	73.1725398
20:42:07	0:39:18	67.8813582	65.846326	85.421441	73.1725398
20:42:08	0:39:19	68.8813582	65.846326	85.421441	74.1725398
20:42:09	0:39:20	69.8813582	67.846326	87.421441	75.1725398
20:42:10	0:39:21	69.8813582	67.846326	89.421441	75.1725398
20:42:11	0:39:22	69.8813582	69.846326	89.421441	77.1725398
20:42:12	0:39:23	69.8813582	69.846326	91.421441	77.1725398
20:42:13	0:39:24	71.8813582	69.846326	92.421441	77.1725398
20:42:14	0:39:25	74.8813582	69.846326	97.421441	76.1725398
20:42:15	0:39:26	74.8813582	73.846326	97.421441	75.1725398
20:42:16	0:39:27	74.8813582	73.846326	99.421441	74.1725398
20:42:17	0:39:28	74.8813582	72.846326	100.421441	73.1725398
20:42:18	0:39:29	74.8813582	71.846326	100.421441	73.1725398
20:42:19	0:39:30	74.8813582	71.846326	101.421441	73.1725398
20:42:20	0:39:31	74.8813582	71.846326	103.421441	72.1725398
20:42:21	0:39:32	74.8813582	72.846326	105.421441	72.1725398
20:42:22	0:39:33	74.8813582	72.846326	81.8953836	72.1725398
20:42:23	0:39:34	74.8813582	72.846326	82.0953836	72.1725398
20:42:24	0:39:35	76.8813582	69.846326	81.0953836	70.1725398
20:42:25	0:39:36	77.8813582	69.846326	81.0953836	69.1725398
20:42:26	0:39:37	75.8813582	69.846326	80.4953836	68.1725398
20:42:27	0:39:38	74.8813582	69.846326	80.2953836	68.1725398
20:42:28	0:39:39	73.8813582	69.846326	79.2953836	68.1725398
20:42:29	0:39:40	71.8813582	78.846326	101.421441	69.1725398
20:42:30	0:39:41	71.8813582	80.846326	96.421441	68.1725398
20:42:31	0:39:42	71.8813582	80.846326	94.421441	68.1725398

20:42:32	0:39:43	72.8813582	82.846326	94.421441	68.1725398
20:42:33	0:39:44	72.8813582	82.846326	91.421441	68.1725398
20:42:34	0:39:45	72.8813582	82.846326	90.421441	67.1725398
20:42:35	0:39:46	73.8813582	82.846326	90.421441	67.1725398
20:42:36	0:39:47	73.8813582	82.846326	89.421441	67.1725398
20:42:37	0:39:48	74.8813582	86.846326	89.421441	68.1725398
20:42:38	0:39:49	74.8813582	88.846326	88.421441	69.1725398
20:42:39	0:39:50	74.8813582	90.846326	85.421441	69.1725398
20:42:40	0:39:51	74.8813582	90.846326	85.421441	68.1725398
20:42:41	0:39:52	74.8813582	91.846326	80.421441	68.1725398
20:42:42	0:39:53	74.8813582	93.846326	76.421441	68.1725398
20:42:43	0:39:54	71.8813582	94.846326	74.421441	67.1725398
20:42:44	0:39:55	71.8813582	94.846326	74.421441	67.1725398
20:42:45	0:39:56	71.8813582	82.846326	72.421441	67.1725398
20:42:46	0:39:57	72.8813582	74.846326	71.421441	68.1725398
20:42:47	0:39:58	72.8813582	72.846326	71.421441	69.1725398
20:42:48	0:39:59	72.8813582	72.846326	71.421441	69.1725398
20:42:49	0:40:00	72.8813582	72.846326	69.421441	70.1725398
20:42:50	0:40:01	71.8813582	73.846326	69.421441	70.1725398
20:42:51	0:40:02	71.8813582	73.846326	67.421441	70.1725398
20:42:52	0:40:03	72.8813582	71.846326	67.421441	69.1725398
20:42:53	0:40:04	72.8813582	71.846326	65.421441	69.1725398
20:42:54	0:40:05	72.8813582	71.846326	65.421441	69.1725398
20:42:55	0:40:06	72.8813582	70.846326	65.421441	69.1725398
20:42:56	0:40:07	72.8813582	69.846326	59.421441	69.1725398
20:42:57	0:40:08	72.8813582	69.846326	58.421441	69.1725398
20:42:58	0:40:09	72.8813582	70.846326	50.421441	70.1725398
20:42:59	0:40:10	72.8813582	70.846326	49.421441	70.1725398
20:43:00	0:40:11	72.8813582	71.846326	49.421441	70.1725398
20:43:01	0:40:12	71.8813582	71.846326	48.421441	71.1725398
20:43:02	0:40:13	70.8813582	70.846326	48.421441	70.1725398
20:43:03	0:40:14	70.8813582	70.846326	49.421441	71.1725398
20:43:04	0:40:15	69.8813582	70.846326	57.421441	72.1725398
20:43:05	0:40:16	66.8813582	70.846326	57.421441	73.1725398
20:43:06	0:40:17	65.8813582	70.846326	58.421441	75.1725398
20:43:07	0:40:18	65.8813582	70.846326	58.421441	75.1725398
20:43:08	0:40:19	66.8813582	70.846326	58.421441	76.1725398
20:43:09	0:40:20	66.8813582	70.846326	58.421441	74.1725398
20:43:10	0:40:21	67.8813582	69.846326	59.421441	72.1725398
20:43:11	0:40:22	67.8813582	68.846326	60.421441	70.1725398
20:43:12	0:40:23	68.8813582	68.846326	60.421441	70.1725398
20:43:13	0:40:24	68.8813582	68.846326	59.421441	70.1725398
20:43:14	0:40:25	69.8813582	69.846326	58.421441	70.1725398
20:43:15	0:40:26	71.8813582	69.846326	58.421441	69.1725398
20:43:16	0:40:27	72.8813582	69.846326	58.421441	67.1725398

20:43:17	0:40:28	73.8813582	70.846326	58.421441	67.1725398
20:43:18	0:40:29	74.8813582	71.846326	58.421441	65.1725398
20:43:19	0:40:30	74.8813582	73.846326	58.421441	65.1725398
20:43:20	0:40:31	74.8813582	73.846326	57.421441	66.1725398
20:43:21	0:40:32	74.8813582	71.846326	57.421441	68.1725398
20:43:22	0:40:33	75.8813582	69.846326	57.421441	70.1725398
20:43:23	0:40:34	75.8813582	69.846326	57.421441	71.1725398
20:43:24	0:40:35	74.8813582	68.846326	57.421441	71.1725398
20:43:25	0:40:36	74.8813582	68.846326	56.421441	71.1725398
20:43:26	0:40:37	73.8813582	70.846326	56.421441	71.1725398
20:43:27	0:40:38	72.8813582	72.846326	56.421441	71.1725398
20:43:28	0:40:39	72.8813582	72.846326	56.421441	70.1725398
20:43:29	0:40:40	72.8813582	72.846326	56.421441	69.1725398
20:43:30	0:40:41	72.8813582	73.846326	57.421441	69.1725398
20:43:31	0:40:42	72.8813582	80.846326	57.421441	71.1725398
20:43:32	0:40:43	72.8813582	83.846326	57.421441	71.1725398
20:43:33	0:40:44	72.8813582	86.846326	57.421441	71.1725398
20:43:34	0:40:45	72.8813582	86.846326	57.421441	71.1725398
20:43:35	0:40:46	73.8813582	86.846326	57.421441	71.1725398
20:43:36	0:40:47	73.8813582	86.846326	57.421441	71.1725398
20:43:37	0:40:48	73.8813582	83.846326	57.421441	70.1725398
20:43:38	0:40:49	73.8813582	78.846326	57.421441	70.1725398
20:43:39	0:40:50	74.8813582	72.846326	57.421441	70.1725398
20:43:40	0:40:51	74.8813582	71.846326	57.421441	71.1725398
20:43:41	0:40:52	76.8813582	71.846326	57.421441	71.1725398
20:43:42	0:40:53	76.8813582	71.846326	57.421441	71.1725398
20:43:43	0:40:54	76.8813582	72.846326	57.421441	71.1725398
20:43:44	0:40:55	76.8813582	72.846326	59.421441	71.1725398
20:43:45	0:40:56	76.8813582	71.846326	60.421441	71.1725398
20:43:46	0:40:57	76.8813582	72.846326	62.421441	70.1725398
20:43:47	0:40:58	73.8813582	72.846326	62.421441	69.1725398
20:43:48	0:40:59	73.8813582	72.846326	62.421441	69.1725398
20:43:49	0:41:00	73.8813582	74.846326	62.421441	70.1725398
20:43:50	0:41:01	72.8813582	74.846326	62.421441	70.1725398
20:43:51	0:41:02	72.8813582	74.846326	62.421441	70.1725398
20:43:52	0:41:03	73.8813582	73.846326	62.421441	70.1725398
20:43:53	0:41:04	73.8813582	71.846326	62.421441	70.1725398
20:43:54	0:41:05	72.8813582	71.846326	62.421441	68.1725398
20:43:55	0:41:06	72.8813582	70.846326	62.421441	68.1725398
20:43:56	0:41:07	72.8813582	69.846326	62.421441	68.1725398
20:43:57	0:41:08	72.8813582	69.846326	62.421441	68.1725398
20:43:58	0:41:09	71.8813582	69.846326	62.421441	67.1725398
20:43:59	0:41:10	72.8813582	69.846326	66.421441	66.1725398
20:44:00	0:41:11	72.8813582	69.846326	66.421441	66.1725398
20:44:01	0:41:12	73.8813582	68.846326	67.421441	66.1725398

20:44:02	0:41:13	73.8813582	67.846326	67.421441	67.1725398
20:44:03	0:41:14	73.8813582	67.846326	68.421441	67.1725398
20:44:04	0:41:15	73.8813582	67.846326	70.421441	67.1725398
20:44:05	0:41:16	74.8813582	67.846326	71.421441	66.1725398
20:44:06	0:41:17	74.8813582	67.846326	72.421441	65.1725398
20:44:07	0:41:18	73.8813582	68.846326	73.421441	65.1725398
20:44:08	0:41:19	74.8813582	67.846326	73.421441	65.1725398
20:44:09	0:41:20	74.8813582	67.846326	73.421441	64.1725398
20:44:10	0:41:21	74.8813582	67.846326	74.421441	65.1725398
20:44:11	0:41:22	74.8813582	69.846326	74.421441	65.1725398
20:44:12	0:41:23	74.8813582	69.846326	75.421441	65.1725398
20:44:13	0:41:24	73.8813582	73.846326	78.421441	65.1725398
20:44:14	0:41:25	73.8813582	77.846326	81.421441	65.1725398
20:44:15	0:41:26	73.8813582	77.846326	85.421441	65.1725398
20:44:16	0:41:27	73.8813582	79.846326	86.421441	65.1725398
20:44:17	0:41:28	73.8813582	77.846326	86.421441	67.1725398
20:44:18	0:41:29	73.8813582	75.846326	85.421441	67.1725398
20:44:19	0:41:30	73.8813582	72.846326	85.421441	68.1725398
20:44:20	0:41:31	73.8813582	71.846326	85.421441	69.1725398
20:44:21	0:41:32	73.8813582	70.846326	85.421441	68.1725398
20:44:22	0:41:33	73.8813582	69.846326	85.421441	68.1725398
20:44:23	0:41:34	73.8813582	69.846326	84.421441	68.1725398
20:44:24	0:41:35	73.8813582	69.846326	85.421441	68.1725398
20:44:25	0:41:36	72.8813582	70.846326	86.421441	70.1725398
20:44:26	0:41:37	72.8813582	70.846326	86.421441	73.1725398
20:44:27	0:41:38	72.8813582	72.846326	87.421441	73.1725398
20:44:28	0:41:39	70.8813582	78.846326	86.421441	73.1725398
20:44:29	0:41:40	69.8813582	81.846326	84.421441	71.1725398
20:44:30	0:41:41	69.8813582	82.846326	89.421441	68.1725398
20:44:31	0:41:42	69.8813582	82.846326	93.421441	68.1725398
20:44:32	0:41:43	69.8813582	82.846326	95.421441	68.1725398
20:44:33	0:41:44	70.8813582	82.846326	95.421441	69.1725398
20:44:34	0:41:45	70.8813582	84.846326	83.421441	70.1725398
20:44:35	0:41:46	70.8813582	84.846326	79.421441	70.1725398
20:44:36	0:41:47	70.8813582	85.846326	77.421441	69.1725398
20:44:37	0:41:48	71.8813582	85.846326	75.421441	69.1725398
20:44:38	0:41:49	71.8813582	87.846326	73.421441	65.1725398
20:44:39	0:41:50	72.8813582	87.846326	73.421441	63.1725398
20:44:40	0:41:51	72.8813582	87.846326	72.421441	63.1725398
20:44:41	0:41:52	72.8813582	88.846326	73.421441	63.1725398
20:44:42	0:41:53	72.8813582	88.846326	73.421441	63.1725398
20:44:43	0:41:54	72.8813582	88.846326	73.421441	64.1725398
20:44:44	0:41:55	72.8813582	74.846326	76.421441	66.1725398
20:44:45	0:41:56	72.8813582	74.846326	78.421441	67.1725398
20:44:46	0:41:57	73.8813582	68.846326	78.421441	67.1725398

20:44:47	0:41:58	73.8813582	67.846326	81.421441	66.1725398
20:44:48	0:41:59	73.8813582	67.846326	83.421441	66.1725398
20:44:49	0:42:00	71.8813582	65.846326	86.421441	66.1725398
20:44:50	0:42:01	71.8813582	65.846326	86.421441	65.1725398
20:44:51	0:42:02	71.8813582	65.846326	86.421441	64.1725398
20:44:52	0:42:03	71.8813582	64.846326	84.421441	65.1725398
20:44:53	0:42:04	70.8813582	63.846326	84.421441	66.1725398
20:44:54	0:42:05	70.8813582	66.846326	84.421441	66.1725398
20:44:55	0:42:06	70.8813582	67.846326	84.421441	66.1725398
20:44:56	0:42:07	71.8813582	67.846326	84.421441	67.1725398
20:44:57	0:42:08	71.8813582	66.846326	88.421441	68.1725398
20:44:58	0:42:09	71.8813582	67.846326	90.421441	67.1725398
20:44:59	0:42:10	72.8813582	68.846326	90.421441	67.1725398
20:45:00	0:42:11	72.8813582	69.846326	90.421441	67.1725398
20:45:01	0:42:12	71.8813582	69.846326	91.421441	66.1725398
20:45:02	0:42:13	71.8813582	69.846326	91.421441	67.1725398
20:45:03	0:42:14	71.8813582	69.846326	91.421441	68.1725398
20:45:04	0:42:15	71.8813582	69.846326	92.421441	69.1725398
20:45:05	0:42:16	73.8813582	69.846326	92.421441	68.1725398
20:45:06	0:42:17	74.8813582	69.846326	93.421441	66.1725398
20:45:07	0:42:18	75.8813582	69.846326	93.421441	64.1725398
20:45:08	0:42:19	75.8813582	69.846326	97.421441	62.1725398
20:45:09	0:42:20	75.8813582	69.846326	98.421441	62.1725398
20:45:10	0:42:21	75.8813582	69.846326	76.2953836	62.1725398
20:45:11	0:42:22	75.8813582	69.846326	101.421441	65.1725398
20:45:12	0:42:23	75.8813582	70.846326	101.421441	67.1725398
20:45:13	0:42:24	75.8813582	73.846326	101.421441	68.1725398
20:45:14	0:42:25	75.8813582	73.846326	101.421441	68.1725398
20:45:15	0:42:26	76.8813582	75.846326	101.421441	68.1725398
20:45:16	0:42:27	78.8813582	75.846326	101.421441	66.1725398
20:45:17	0:42:28	78.8813582	74.846326	100.421441	66.1725398
20:45:18	0:42:29	77.8813582	74.846326	98.421441	66.1725398
20:45:19	0:42:30	76.8813582	74.846326	98.421441	66.1725398
20:45:20	0:42:31	75.8813582	74.846326	92.421441	68.1725398
20:45:21	0:42:32	74.8813582	74.846326	89.421441	69.1725398
20:45:22	0:42:33	72.8813582	74.846326	87.421441	70.1725398
20:45:23	0:42:34	71.8813582	73.846326	86.421441	71.1725398
20:45:24	0:42:35	71.8813582	70.846326	85.421441	72.1725398
20:45:25	0:42:36	70.8813582	70.846326	84.421441	73.1725398
20:45:26	0:42:37	70.8813582	70.846326	84.421441	73.1725398
20:45:27	0:42:38	70.8813582	71.846326	83.421441	73.1725398
20:45:28	0:42:39	69.8813582	71.846326	81.421441	73.1725398
20:45:29	0:42:40	68.8813582	70.846326	81.421441	73.1725398
20:45:30	0:42:41	68.8813582	70.846326	81.421441	71.1725398
20:45:31	0:42:42	68.8813582	70.846326	80.421441	71.1725398

20:45:32	0:42:43	68.8813582	71.846326	80.421441	71.1725398
20:45:33	0:42:44	68.8813582	72.846326	79.421441	71.1725398
20:45:34	0:42:45	68.8813582	74.846326	79.421441	74.1725398
20:45:35	0:42:46	68.8813582	74.846326	79.421441	74.1725398
20:45:36	0:42:47	68.8813582	74.846326	79.421441	74.1725398
20:45:37	0:42:48	69.8813582	73.846326	79.421441	74.1725398
20:45:38	0:42:49	69.8813582	73.846326	79.421441	74.1725398
20:45:39	0:42:50	69.8813582	73.846326	79.421441	70.1725398
20:45:40	0:42:51	69.8813582	73.846326	79.421441	69.1725398
20:45:41	0:42:52	69.8813582	73.846326	81.421441	69.1725398
20:45:42	0:42:53	68.8813582	71.846326	81.421441	69.1725398
20:45:43	0:42:54	68.8813582	69.846326	81.421441	71.1725398
20:45:44	0:42:55	68.8813582	70.846326	80.421441	71.1725398
20:45:45	0:42:56	68.8813582	70.846326	78.421441	73.1725398
20:45:46	0:42:57	70.8813582	70.846326	78.421441	72.1725398
20:45:47	0:42:58	71.8813582	70.846326	78.421441	72.1725398
20:45:48	0:42:59	70.8813582	78.846326	77.421441	68.1725398
20:45:49	0:43:00	70.8813582	78.846326	75.421441	66.1725398
20:45:50	0:43:01	70.8813582	78.846326	75.421441	66.1725398
20:45:51	0:43:02	70.8813582	75.846326	74.421441	66.1725398
20:45:52	0:43:03	70.8813582	73.846326	74.421441	66.1725398
20:45:53	0:43:04	71.8813582	73.846326	74.421441	66.1725398
20:45:54	0:43:05	71.8813582	70.846326	74.421441	66.1725398
20:45:55	0:43:06	73.8813582	71.846326	75.421441	66.1725398
20:45:56	0:43:07	73.8813582	71.846326	75.421441	65.1725398
20:45:57	0:43:08	74.8813582	72.846326	75.421441	66.1725398
20:45:58	0:43:09	74.8813582	72.846326	75.421441	66.1725398
20:45:59	0:43:10	73.8813582	71.846326	81.421441	67.1725398
20:46:00	0:43:11	73.8813582	71.846326	83.421441	67.1725398
20:46:01	0:43:12	74.8813582	71.846326	83.421441	67.1725398
20:46:02	0:43:13	75.8813582	71.846326	84.421441	69.1725398
20:46:03	0:43:14	76.8813582	71.846326	84.421441	70.1725398
20:46:04	0:43:15	74.8813582	71.846326	84.421441	70.1725398
20:46:05	0:43:16	73.8813582	71.846326	84.421441	70.1725398
20:46:06	0:43:17	73.8813582	71.846326	84.421441	70.1725398
20:46:07	0:43:18	73.8813582	70.846326	84.421441	70.1725398
20:46:08	0:43:19	73.8813582	70.846326	86.421441	73.1725398
20:46:09	0:43:20	74.8813582	73.846326	83.421441	73.1725398
20:46:10	0:43:21	76.8813582	77.846326	81.421441	73.1725398
20:46:11	0:43:22	77.8813582	77.846326	81.421441	73.1725398
20:46:12	0:43:23	76.8813582	77.846326	80.421441	73.1725398
20:46:13	0:43:24	72.8813582	77.846326	80.421441	73.1725398
20:46:14	0:43:25	69.8813582	77.846326	80.421441	74.1725398
20:46:15	0:43:26	69.8813582	77.846326	80.421441	74.1725398
20:46:16	0:43:27	67.8813582	76.846326	81.421441	80.1725398

20:46:17	0:43:28	68.8813582	75.846326	81.421441	81.1725398
20:46:18	0:43:29	68.8813582	75.846326	81.421441	81.1725398
20:46:19	0:43:30	68.8813582	73.846326	81.421441	81.1725398
20:46:20	0:43:31	68.8813582	70.846326	81.421441	96.1725398
20:46:21	0:43:32	68.8813582	70.846326	82.421441	96.1725398
20:46:22	0:43:33	69.8813582	67.846326	82.421441	96.1725398
20:46:23	0:43:34	69.8813582	65.846326	82.421441	96.1725398
20:46:24	0:43:35	69.8813582	65.846326	82.421441	89.1725398
20:46:25	0:43:36	69.8813582	64.846326	82.421441	89.1725398
20:46:26	0:43:37	70.8813582	64.846326	82.421441	89.1725398
20:46:27	0:43:38	75.8813582	64.846326	82.421441	71.1725398
20:46:28	0:43:39	79.8813582	67.846326	82.421441	65.1725398
20:46:29	0:43:40	80.8813582	68.846326	82.421441	64.1725398
20:46:30	0:43:41	80.8813582	68.846326	82.421441	64.1725398
20:46:31	0:43:42	83.8813582	72.846326	84.421441	63.1725398
20:46:32	0:43:43	84.8813582	74.846326	83.421441	64.1725398
20:46:33	0:43:44	84.8813582	74.846326	83.421441	64.1725398
20:46:34	0:43:45	83.8813582	74.846326	83.421441	65.1725398
20:46:35	0:43:46	80.8813582	72.846326	83.421441	65.1725398
20:46:36	0:43:47	78.8813582	72.846326	83.421441	64.1725398
20:46:37	0:43:48	78.8813582	70.846326	83.421441	64.1725398
20:46:38	0:43:49	78.8813582	68.846326	83.421441	65.1725398
20:46:39	0:43:50	78.8813582	65.846326	90.421441	65.1725398
20:46:40	0:43:51	77.8813582	65.846326	91.421441	65.1725398
20:46:41	0:43:52	76.8813582	65.846326	92.421441	65.1725398
20:46:42	0:43:53	76.8813582	65.846326	92.421441	66.1725398
20:46:43	0:43:54	74.8813582	66.846326	92.421441	67.1725398
20:46:44	0:43:55	74.8813582	66.846326	90.421441	67.1725398
20:46:45	0:43:56	74.8813582	66.846326	86.421441	67.1725398
20:46:46	0:43:57	73.8813582	69.846326	86.421441	67.1725398
20:46:47	0:43:58	73.8813582	71.846326	87.421441	66.1725398
20:46:48	0:43:59	73.8813582	72.846326	88.421441	66.1725398
20:46:49	0:44:00	73.8813582	73.846326	88.421441	65.1725398
20:46:50	0:44:01	73.8813582	73.846326	88.421441	65.1725398
20:46:51	0:44:02	73.8813582	73.846326	88.421441	65.1725398
20:46:52	0:44:03	73.8813582	73.846326	85.421441	66.1725398
20:46:53	0:44:04	75.8813582	72.846326	84.421441	66.1725398
20:46:54	0:44:05	75.8813582	72.846326	82.421441	67.1725398
20:46:55	0:44:06	75.8813582	72.846326	82.421441	67.1725398
20:46:56	0:44:07	75.8813582	76.846326	82.421441	69.1725398
20:46:57	0:44:08	75.8813582	76.846326	81.421441	69.1725398
20:46:58	0:44:09	76.8813582	76.846326	81.421441	74.1725398
20:46:59	0:44:10	77.8813582	76.846326	81.421441	78.1725398
20:47:00	0:44:11	78.8813582	73.846326	81.421441	78.1725398
20:47:01	0:44:12	75.8813582	70.846326	82.421441	80.1725398

20:47:02	0:44:13	72.8813582	68.846326	85.421441	81.1725398
20:47:03	0:44:14	71.8813582	68.846326	88.421441	85.1725398
20:47:04	0:44:15	71.8813582	69.846326	88.421441	87.1725398
20:47:05	0:44:16	71.8813582	72.846326	88.421441	87.1725398
20:47:06	0:44:17	71.8813582	72.846326	89.421441	87.1725398
20:47:07	0:44:18	71.8813582	72.846326	89.421441	87.1725398
20:47:08	0:44:19	71.8813582	72.846326	88.421441	87.1725398
20:47:09	0:44:20	72.8813582	70.846326	88.421441	87.1725398
20:47:10	0:44:21	73.8813582	69.846326	88.421441	86.1725398
20:47:11	0:44:22	75.8813582	70.846326	88.421441	86.1725398
20:47:12	0:44:23	76.8813582	70.846326	89.421441	85.1725398
20:47:13	0:44:24	76.8813582	67.846326	89.421441	84.1725398
20:47:14	0:44:25	77.8813582	66.846326	90.421441	84.1725398
20:47:15	0:44:26	77.8813582	66.846326	89.421441	84.1725398
20:47:16	0:44:27	77.8813582	66.846326	89.421441	84.1725398
20:47:17	0:44:28	77.8813582	69.846326	89.421441	84.1725398
20:47:18	0:44:29	77.8813582	70.846326	89.421441	84.1725398
20:47:19	0:44:30	76.8813582	72.846326	89.421441	83.1725398
20:47:20	0:44:31	75.8813582	73.846326	91.421441	83.1725398
20:47:21	0:44:32	75.8813582	73.846326	91.421441	83.1725398
20:47:22	0:44:33	75.8813582	72.846326	92.421441	83.1725398
20:47:23	0:44:34	76.8813582	69.846326	92.421441	83.1725398
20:47:24	0:44:35	76.8813582	69.846326	92.421441	83.1725398
20:47:25	0:44:36	78.8813582	64.846326	93.421441	90.1725398
20:47:26	0:44:37	78.8813582	64.846326	94.421441	92.1725398
20:47:27	0:44:38	78.8813582	65.846326	94.421441	93.1725398
20:47:28	0:44:39	78.8813582	65.846326	94.421441	93.1725398
20:47:29	0:44:40	79.8813582	67.846326	96.421441	93.1725398
20:47:30	0:44:41	80.8813582	68.846326	95.421441	93.1725398
20:47:31	0:44:42	81.8813582	68.846326	95.421441	94.1725398
20:47:32	0:44:43	81.8813582	71.846326	95.421441	93.1725398
20:47:33	0:44:44	81.8813582	72.846326	95.421441	93.1725398
20:47:34	0:44:45	81.8813582	72.846326	95.421441	93.1725398
20:47:35	0:44:46	81.8813582	71.846326	95.421441	92.1725398
20:47:36	0:44:47	79.8813582	70.846326	95.421441	80.1725398
20:47:37	0:44:48	79.8813582	70.846326	95.421441	72.1725398
20:47:38	0:44:49	78.8813582	70.846326	91.421441	71.1725398
20:47:39	0:44:50	79.8813582	73.846326	90.421441	71.1725398
20:47:40	0:44:51	79.8813582	74.846326	89.421441	71.1725398
20:47:41	0:44:52	82.8813582	75.846326	88.421441	70.1725398
20:47:42	0:44:53	81.8813582	74.846326	88.421441	69.1725398
20:47:43	0:44:54	76.8813582	74.846326	86.421441	69.1725398
20:47:44	0:44:55	72.8813582	72.846326	86.421441	70.1725398
20:47:45	0:44:56	72.8813582	71.846326	84.421441	71.1725398
20:47:46	0:44:57	69.8813582	71.846326	83.421441	70.1725398

20:47:47	0:44:58		69.8813582	70.846326	81.421441	69.1725398
20:47:48	0:44:59		69.8813582	70.846326	79.421441	68.1725398
20:47:49	0:45:00		69.8813582	71.846326	79.421441	67.1725398
20:47:50	0:45:01		69.8813582	71.846326	78.421441	68.1725398
20:47:51	0:45:02		69.8813582	71.846326	77.421441	69.1725398
20:47:52	0:45:03		69.8813582	72.846326	76.421441	70.1725398
20:47:53	0:45:04		69.8813582	72.846326	76.421441	70.1725398
20:47:54	0:45:05		69.8813582	72.846326	76.421441	70.1725398
20:47:55	0:45:06		70.8813582	70.846326	76.421441	69.1725398
20:47:56	0:45:07		70.8813582	68.846326	76.421441	68.1725398
20:47:57	0:45:08		70.8813582	68.846326	76.421441	68.1725398
20:47:58	0:45:09		70.8813582	66.846326	76.421441	68.1725398
20:47:59	0:45:10		70.8813582	64.846326	76.421441	70.1725398
20:48:00	0:45:11		71.8813582	64.846326	76.421441	71.1725398
20:48:01	0:45:12		72.8813582	64.846326	77.421441	74.1725398
20:48:02	0:45:13		72.8813582	61.846326	77.421441	77.1725398
20:48:03	0:45:14		71.8813582	61.846326	78.421441	78.1725398
20:48:04	0:45:15		70.8813582	64.846326	78.421441	81.1725398
20:48:05	0:45:16		69.8813582	65.846326	76.421441	81.1725398
20:48:06	0:45:17		69.8813582	65.846326	76.421441	81.1725398
20:48:07	0:45:18		69.8813582	67.846326	76.421441	82.1725398
20:48:08	0:45:19		69.8813582	68.846326	76.421441	84.1725398
20:48:09	0:45:20		69.8813582	68.846326	75.421441	85.1725398
20:48:10	0:45:21		69.8813582	68.846326	74.421441	86.1725398
20:48:11	0:45:22		69.8813582	68.846326	73.421441	86.1725398
20:48:12	0:45:23		68.8813582	68.846326	73.421441	88.1725398
20:48:13	0:45:24		68.8813582	68.846326	72.421441	88.1725398
20:48:14	0:45:25		68.8813582	68.846326	72.421441	91.1725398
20:48:15	0:45:26		68.8813582	69.846326	72.421441	92.1725398
20:48:16	0:45:27		67.8813582	69.846326	71.421441	94.1725398
20:48:17	0:45:28		67.8813582	69.846326	71.421441	95.1725398
20:48:18	0:45:29		67.8813582	69.846326	71.421441	95.1725398
20:48:19	0:45:30		67.8813582	70.846326	72.421441	95.1725398
20:48:20	0:45:31		67.8813582	70.846326	75.421441	96.1725398
20:48:21	0:45:32		68.8813582	70.846326	75.421441	93.1725398
20:48:22	0:45:33		70.8813582	70.846326	75.421441	90.1725398
20:48:23	0:45:34		70.8813582	72.846326	75.421441	90.1725398
20:48:24	0:45:35		71.8813582	73.846326	74.421441	87.1725398
20:48:25	0:45:36		72.8813582	75.846326	72.421441	84.1725398
20:48:26	0:45:37		75.8813582	75.846326	72.421441	83.1725398
20:48:27	0:45:38		77.8813582	76.846326	72.421441	83.1725398
20:48:28	0:45:39		77.8813582	76.846326	71.421441	83.1725398
20:48:29	0:45:40		76.8813582	76.846326	71.421441	83.1725398
20:48:30	0:45:41		75.8813582	79.846326	71.421441	83.1725398
20:48:31	0:45:42		74.8813582	79.846326	72.421441	83.1725398

20:48:32	0:45:43	73.8813582	79.846326	71.421441	84.1725398
20:48:33	0:45:44	72.8813582	79.846326	70.421441	84.1725398
20:48:34	0:45:45	72.8813582	80.846326	70.421441	84.1725398
20:48:35	0:45:46	72.8813582	81.846326	68.421441	84.1725398
20:48:36	0:45:47	71.8813582	81.846326	68.421441	84.1725398
20:48:37	0:45:48	71.8813582	81.846326	68.421441	83.1725398
20:48:38	0:45:49	71.8813582	81.846326	68.421441	81.1725398
20:48:39	0:45:50	71.8813582	81.846326	68.421441	81.1725398
20:48:40	0:45:51	69.8813582	81.846326	69.421441	82.1725398
20:48:41	0:45:52	67.8813582	81.846326	71.421441	82.1725398
20:48:42	0:45:53	67.8813582	82.846326	72.421441	82.1725398
20:48:43	0:45:54	70.8813582	82.846326	72.421441	82.1725398
20:48:44	0:45:55	72.8813582	82.846326	72.421441	82.1725398
20:48:45	0:45:56	73.8813582	82.846326	72.421441	83.1725398
20:48:46	0:45:57	74.8813582	82.846326	72.421441	86.1725398
20:48:47	0:45:58	74.8813582	82.846326	72.421441	87.1725398
20:48:48	0:45:59	74.8813582	83.846326	73.421441	88.1725398
20:48:49	0:46:00	75.8813582	84.846326	73.421441	88.1725398
20:48:50	0:46:01	75.8813582	84.846326	73.421441	88.1725398
20:48:51	0:46:02	76.8813582	84.846326	73.421441	88.1725398
20:48:52	0:46:03	76.8813582	85.846326	73.421441	89.1725398
20:48:53	0:46:04	76.8813582	82.846326	72.421441	90.1725398
20:48:54	0:46:05	75.8813582	77.846326	72.421441	91.1725398
20:48:55	0:46:06	73.8813582	77.846326	71.421441	92.1725398
20:48:56	0:46:07	72.8813582	77.846326	71.421441	92.1725398
20:48:57	0:46:08	71.8813582	76.846326	72.421441	92.1725398
20:48:58	0:46:09	71.8813582	75.846326	73.421441	92.1725398
20:48:59	0:46:10	71.8813582	73.846326	74.421441	93.1725398
20:49:00	0:46:11	72.8813582	73.846326	74.421441	92.1725398
20:49:01	0:46:12	72.8813582	69.846326	75.421441	86.1725398
20:49:02	0:46:13	72.8813582	69.846326	75.421441	83.1725398
20:49:03	0:46:14	73.8813582	69.846326	77.421441	80.1725398
20:49:04	0:46:15	73.8813582	69.846326	77.421441	77.1725398
20:49:05	0:46:16	74.8813582	68.846326	77.421441	73.1725398
20:49:06	0:46:17	76.8813582	64.846326	77.421441	73.1725398
20:49:07	0:46:18	77.8813582	61.846326	77.421441	73.1725398
20:49:08	0:46:19	77.8813582	60.846326	79.421441	69.1725398
20:49:09	0:46:20	76.8813582	60.846326	79.421441	69.1725398
20:49:10	0:46:21	74.8813582	60.846326	81.421441	67.1725398
20:49:11	0:46:22	73.8813582	60.846326	81.421441	68.1725398
20:49:12	0:46:23	73.8813582	60.846326	81.421441	68.1725398
20:49:13	0:46:24	72.8813582	59.846326	80.421441	68.1725398
20:49:14	0:46:25	72.8813582	59.846326	80.421441	68.1725398
20:49:15	0:46:26	73.8813582	59.846326	80.421441	72.1725398
20:49:16	0:46:27	73.8813582	60.846326	81.421441	72.1725398

20:49:17	0:46:28	73.8813582	60.846326	81.421441	71.1725398
20:49:18	0:46:29	74.8813582	60.846326	82.421441	70.1725398
20:49:19	0:46:30	76.8813582	60.846326	82.421441	69.1725398
20:49:20	0:46:31	76.8813582	60.846326	83.421441	69.1725398
20:49:21	0:46:32	77.8813582	60.846326	83.421441	69.1725398
20:49:22	0:46:33	77.8813582	61.846326	83.421441	69.1725398
20:49:23	0:46:34	72.8813582	61.846326	83.421441	70.1725398
20:49:24	0:46:35	68.8813582	64.846326	83.421441	70.1725398
20:49:25	0:46:36	66.8813582	66.846326	84.421441	69.1725398
20:49:26	0:46:37	65.8813582	66.846326	84.421441	69.1725398
20:49:27	0:46:38	64.8813582	66.846326	85.421441	68.1725398
20:49:28	0:46:39	63.8813582	62.846326	85.421441	69.1725398
20:49:29	0:46:40	63.8813582	61.846326	85.421441	69.1725398
20:49:30	0:46:41	63.8813582	62.846326	90.421441	72.1725398
20:49:31	0:46:42	63.8813582	63.846326	92.421441	72.1725398
20:49:32	0:46:43	63.8813582	63.846326	93.421441	71.1725398
20:49:33	0:46:44	63.8813582	66.846326	96.421441	69.1725398
20:49:34	0:46:45	63.8813582	67.846326	96.421441	69.1725398
20:49:35	0:46:46	63.8813582	68.846326	98.421441	69.1725398
20:49:36	0:46:47	62.8813582	68.846326	98.421441	73.1725398
20:49:37	0:46:48	62.8813582	68.846326	98.421441	74.1725398
20:49:38	0:46:49	62.8813582	69.846326	98.421441	74.1725398
20:49:39	0:46:50	62.8813582	69.846326	98.421441	73.1725398
20:49:40	0:46:51	62.8813582	69.846326	98.421441	73.1725398
20:49:41	0:46:52	62.8813582	69.846326	99.421441	73.1725398
20:49:42	0:46:53	62.8813582	69.846326	101.421441	73.1725398
20:49:43	0:46:54	62.8813582	69.846326	78.4953836	73.1725398
20:49:44	0:46:55	62.8813582	69.846326	78.4953836	73.1725398
20:49:45	0:46:56	62.8813582	69.846326	78.4953836	73.1725398
20:49:46	0:46:57	62.8813582	74.846326	102.421441	72.1725398
20:49:47	0:46:58	62.8813582	75.846326	79.4953836	72.1725398
20:49:48	0:46:59	63.8813582	77.846326	103.421441	72.1725398
20:49:49	0:47:00	63.8813582	77.846326	103.421441	73.1725398
20:49:50	0:47:01	63.8813582	77.846326	101.421441	73.1725398
20:49:51	0:47:02	63.8813582	78.846326	100.421441	74.1725398
20:49:52	0:47:03	63.8813582	78.846326	100.421441	73.1725398
20:49:53	0:47:04	63.8813582	78.846326	100.421441	73.1725398
20:49:54	0:47:05	63.8813582	78.846326	100.421441	72.1725398
20:49:55	0:47:06	64.8813582	79.846326	100.421441	72.1725398
20:49:56	0:47:07	65.8813582	79.846326	98.421441	72.1725398
20:49:57	0:47:08	65.8813582	79.846326	98.421441	72.1725398
20:49:58	0:47:09	64.8813582	80.846326	98.421441	75.1725398
20:49:59	0:47:10	64.8813582	80.846326	98.421441	76.1725398
20:50:00	0:47:11	64.8813582	80.846326	97.421441	77.1725398
20:50:01	0:47:12	64.8813582	80.846326	97.421441	76.1725398

20:50:02	0:47:13	64.8813582	81.846326	97.421441	75.1725398
20:50:03	0:47:14	64.8813582	81.846326	93.421441	72.1725398
20:50:04	0:47:15	64.8813582	81.846326	90.421441	70.1725398
20:50:05	0:47:16	63.8813582	83.846326	90.421441	69.1725398
20:50:06	0:47:17	63.8813582	83.846326	90.421441	69.1725398
20:50:07	0:47:18	64.8813582	83.846326	88.421441	70.1725398
20:50:08	0:47:19	64.8813582	83.846326	87.421441	70.1725398
20:50:09	0:47:20	64.8813582	83.846326	87.421441	70.1725398
20:50:10	0:47:21	63.8813582	83.846326	87.421441	70.1725398
20:50:11	0:47:22	63.8813582	83.846326	80.421441	70.1725398
20:50:12	0:47:23	63.8813582	83.846326	79.421441	72.1725398
20:50:13	0:47:24	63.8813582	83.846326	79.421441	74.1725398
20:50:14	0:47:25	63.8813582	83.846326	79.421441	76.1725398
20:50:15	0:47:26	63.8813582	86.846326	77.421441	76.1725398
20:50:16	0:47:27	63.8813582	90.846326	76.421441	76.1725398
20:50:17	0:47:28	62.8813582	91.846326	76.421441	74.1725398
20:50:18	0:47:29	62.8813582	91.846326	74.421441	74.1725398
20:50:19	0:47:30	61.8813582	91.846326	73.421441	74.1725398
20:50:20	0:47:31	61.8813582	91.846326	73.421441	74.1725398
20:50:21	0:47:32	61.8813582	93.846326	73.421441	78.1725398
20:50:22	0:47:33	61.8813582	85.846326	73.421441	80.1725398
20:50:23	0:47:34	61.8813582	81.846326	73.421441	81.1725398
20:50:24	0:47:35	61.8813582	80.846326	82.421441	81.1725398
20:50:25	0:47:36	59.8813582	80.846326	86.421441	83.1725398
20:50:26	0:47:37	59.8813582	80.846326	88.421441	85.1725398
20:50:27	0:47:38	59.8813582	80.846326	87.421441	86.1725398
20:50:28	0:47:39	58.8813582	79.846326	86.421441	88.1725398
20:50:29	0:47:40	58.8813582	78.846326	85.421441	88.1725398
20:50:30	0:47:41	59.8813582	78.846326	85.421441	90.1725398
20:50:31	0:47:42	59.8813582	78.846326	83.421441	91.1725398
20:50:32	0:47:43	59.8813582	79.846326	82.421441	91.1725398
20:50:33	0:47:44	60.8813582	79.846326	80.421441	91.1725398
20:50:34	0:47:45	60.8813582	78.846326	80.421441	90.1725398
20:50:35	0:47:46	60.8813582	78.846326	80.421441	90.1725398
20:50:36	0:47:47	60.8813582	69.846326	79.421441	89.1725398
20:50:37	0:47:48	60.8813582	65.846326	79.421441	89.1725398
20:50:38	0:47:49	60.8813582	64.846326	79.421441	88.1725398
20:50:39	0:47:50	60.8813582	63.846326	79.421441	87.1725398
20:50:40	0:47:51	60.8813582	63.846326	79.421441	85.1725398
20:50:41	0:47:52	60.8813582	63.846326	79.421441	83.1725398
20:50:42	0:47:53	60.8813582	63.846326	79.421441	82.1725398
20:50:43	0:47:54	60.8813582	63.846326	79.421441	77.1725398
20:50:44	0:47:55	60.8813582	63.846326	79.421441	73.1725398
20:50:45	0:47:56	60.8813582	63.846326	79.421441	71.1725398
20:50:46	0:47:57	60.8813582	63.846326	86.421441	70.1725398

20:50:47	0:47:58	60.8813582	63.846326	88.421441	69.1725398
20:50:48	0:47:59	61.8813582	62.846326	88.421441	68.1725398
20:50:49	0:48:00	62.8813582	62.846326	89.421441	68.1725398
20:50:50	0:48:01	62.8813582	61.846326	89.421441	68.1725398
20:50:51	0:48:02	62.8813582	61.846326	89.421441	67.1725398
20:50:52	0:48:03	62.8813582	60.846326	89.421441	67.1725398
20:50:53	0:48:04	62.8813582	60.846326	89.421441	67.1725398
20:50:54	0:48:05	62.8813582	60.846326	89.421441	67.1725398
20:50:55	0:48:06	62.8813582	63.846326	89.421441	67.1725398
20:50:56	0:48:07	62.8813582	64.846326	89.421441	67.1725398
20:50:57	0:48:08	63.8813582	64.846326	88.421441	67.1725398
20:50:58	0:48:09	63.8813582	64.846326	88.421441	69.1725398
20:50:59	0:48:10	64.8813582	63.846326	88.421441	71.1725398
20:51:00	0:48:11	64.8813582	63.846326	88.421441	71.1725398
20:51:01	0:48:12	64.8813582	62.846326	88.421441	71.1725398
20:51:02	0:48:13	64.8813582	62.846326	88.421441	70.1725398
20:51:03	0:48:14	64.8813582	62.846326	88.421441	72.1725398
20:51:04	0:48:15	64.8813582	61.846326	88.421441	73.1725398
20:51:05	0:48:16	64.8813582	62.846326	88.421441	74.1725398
20:51:06	0:48:17	64.8813582	62.846326	88.421441	75.1725398
20:51:07	0:48:18	64.8813582	63.846326	88.421441	76.1725398
20:51:08	0:48:19	64.8813582	64.846326	88.421441	76.1725398
20:51:09	0:48:20	64.8813582	64.846326	88.421441	76.1725398
20:51:10	0:48:21	64.8813582	64.846326	89.421441	76.1725398
20:51:11	0:48:22	64.8813582	66.846326	89.421441	77.1725398
20:51:12	0:48:23	64.8813582	67.846326	89.421441	80.1725398
20:51:13	0:48:24	64.8813582	67.846326	89.421441	82.1725398
20:51:14	0:48:25	64.8813582	67.846326	91.421441	83.1725398
20:51:15	0:48:26	64.8813582	63.846326	91.421441	83.1725398
20:51:16	0:48:27		63.846326	91.421441	85.1725398
20:51:17	0:48:28		62.846326	90.421441	85.1725398
20:51:18	0:48:29		62.846326	90.421441	85.1725398
20:51:19	0:48:30		62.846326	86.421441	85.1725398
20:51:20	0:48:31		62.846326	86.421441	83.1725398
20:51:21	0:48:32		61.846326	86.421441	83.1725398
20:51:22	0:48:33		61.846326	87.421441	83.1725398
20:51:23	0:48:34		61.846326	87.421441	85.1725398
20:51:24	0:48:35		61.846326	87.421441	85.1725398
20:51:25	0:48:36		61.846326	87.421441	86.1725398
20:51:26	0:48:37		61.846326	89.421441	86.1725398
20:51:27	0:48:38		61.846326	89.421441	88.1725398
20:51:28	0:48:39		60.846326	89.421441	89.1725398
20:51:29	0:48:40		61.846326	88.421441	89.1725398
20:51:30	0:48:41		62.846326	87.421441	88.1725398
20:51:31	0:48:42		62.846326	87.421441	86.1725398

20:51:32	0:48:43		62.846326	87.421441	84.1725398
20:51:33	0:48:44		63.846326	88.421441	82.1725398
20:51:34	0:48:45		63.846326	90.421441	82.1725398
20:51:35	0:48:46		64.846326	90.421441	82.1725398
20:51:36	0:48:47		64.846326	90.421441	82.1725398
20:51:37	0:48:48		64.846326	92.421441	81.1725398
20:51:38	0:48:49		65.846326	92.421441	80.1725398
20:51:39	0:48:50		68.846326	92.421441	78.1725398
20:51:40	0:48:51		69.846326	92.421441	76.1725398
20:51:41	0:48:52		69.846326	92.421441	75.1725398
20:51:42	0:48:53		69.846326	91.421441	73.1725398
20:51:43	0:48:54		68.846326	90.421441	73.1725398
20:51:44	0:48:55		68.846326	88.421441	73.1725398
20:51:45	0:48:56		68.846326	88.421441	73.1725398
20:51:46	0:48:57		68.846326	88.421441	73.1725398
20:51:47	0:48:58		68.846326	88.421441	73.1725398
20:51:48	0:48:59		70.846326	88.421441	72.1725398
20:51:49	0:49:00		73.846326	88.421441	74.1725398
20:51:50	0:49:01		72.846326	88.421441	74.1725398
20:51:51	0:49:02		72.846326	88.421441	73.1725398
20:51:52	0:49:03		72.846326	89.421441	72.1725398
20:51:53	0:49:04		71.846326	89.421441	72.1725398
20:51:54	0:49:05		71.846326	89.421441	72.1725398
20:51:55	0:49:06		71.846326	89.421441	75.1725398
20:51:56	0:49:07	64.8813582	71.846326	89.421441	76.1725398
20:51:57	0:49:08	64.8813582	71.846326	89.421441	76.1725398
20:51:58	0:49:09	64.8813582	70.846326	89.421441	77.1725398
20:51:59	0:49:10	64.8813582	70.846326	89.421441	78.1725398
20:52:00	0:49:11	64.8813582	70.846326	89.421441	78.1725398
20:52:01	0:49:12	78.8813582	70.846326	88.421441	76.1725398
20:52:02	0:49:13	79.8813582	70.846326	86.421441	75.1725398
20:52:03	0:49:14	79.8813582	69.846326	86.421441	75.1725398
20:52:04	0:49:15	79.8813582	69.846326	86.421441	74.1725398
20:52:05	0:49:16	79.8813582	69.846326	86.421441	75.1725398
20:52:06	0:49:17	79.8813582	68.846326	86.421441	75.1725398
20:52:07	0:49:18	79.8813582	68.846326	86.421441	77.1725398
20:52:08	0:49:19	80.8813582	68.846326	86.421441	77.1725398
20:52:09	0:49:20	80.8813582	68.846326	86.421441	79.1725398
20:52:10	0:49:21	80.8813582	68.846326	86.421441	79.1725398
20:52:11	0:49:22	81.8813582	68.846326	86.421441	79.1725398
20:52:12	0:49:23	81.8813582	71.846326	85.421441	79.1725398
20:52:13	0:49:24	81.8813582	72.846326	85.421441	76.1725398
20:52:14	0:49:25	85.8813582	72.846326	85.421441	74.1725398
20:52:15	0:49:26	85.8813582	72.846326	85.421441	74.1725398
20:52:16	0:49:27	85.8813582	72.846326	84.421441	74.1725398

20:52:17	0:49:28	85.8813582	73.846326	84.421441	74.1725398
20:52:18	0:49:29	85.8813582	73.846326	80.421441	74.1725398
20:52:19	0:49:30	85.8813582	72.846326	80.421441	78.1725398
20:52:20	0:49:31	88.8813582	71.846326	80.421441	79.1725398
20:52:21	0:49:32	88.8813582	69.846326	80.421441	79.1725398
20:52:22	0:49:33	89.8813582	67.846326	80.421441	79.1725398
20:52:23	0:49:34	90.8813582	67.846326	80.421441	79.1725398
20:52:24	0:49:35	91.8813582	65.846326	79.421441	79.1725398
20:52:25	0:49:36	91.8813582	64.846326	81.421441	82.1725398
20:52:26	0:49:37	93.8813582	64.846326	82.421441	82.1725398
20:52:27	0:49:38	93.8813582	64.846326	83.421441	81.1725398
20:52:28	0:49:39	93.8813582	64.846326	83.421441	80.1725398
20:52:29	0:49:40	95.8813582	68.846326	83.421441	80.1725398
20:52:30	0:49:41	96.8813582	68.846326	88.421441	80.1725398
20:52:31	0:49:42	98.8813582	68.846326	88.421441	80.1725398
20:52:32	0:49:43	94.8813582	67.846326	89.421441	80.1725398
20:52:33	0:49:44	93.8813582	66.846326	89.421441	80.1725398
20:52:34	0:49:45	88.8813582	66.846326	89.421441	79.1725398
20:52:35	0:49:46	85.8813582	65.846326	89.421441	79.1725398
20:52:36	0:49:47	87.8813582	65.846326	89.421441	79.1725398
20:52:37	0:49:48	91.8813582	65.846326	91.421441	78.1725398
20:52:38	0:49:49	95.8813582	64.846326	91.421441	76.1725398
20:52:39	0:49:50	99.8813582	64.846326	91.421441	76.1725398
20:52:40	0:49:51	92.8813582	64.846326	92.421441	76.1725398
20:52:41	0:49:52	89.8813582	64.846326	91.421441	76.1725398
20:52:42	0:49:53	87.8813582	64.846326	91.421441	77.1725398
20:52:43	0:49:54	85.8813582	64.846326	91.421441	77.1725398
20:52:44	0:49:55	84.8813582	63.846326	90.421441	77.1725398
20:52:45	0:49:56	84.8813582	63.846326	93.421441	77.1725398
20:52:46	0:49:57	83.8813582	63.846326	94.421441	77.1725398
20:52:47	0:49:58	83.8813582	70.846326	94.421441	77.1725398
20:52:48	0:49:59	83.8813582	70.846326	94.421441	77.1725398
20:52:49	0:50:00	82.8813582	70.846326	96.421441	78.1725398
20:52:50	0:50:01	83.8813582	74.846326	96.421441	77.1725398
20:52:51	0:50:02	83.8813582	74.846326	93.421441	77.1725398
20:52:52	0:50:03	85.8813582	74.846326	93.421441	75.1725398
20:52:53	0:50:04	85.8813582	73.846326	92.421441	75.1725398
20:52:54	0:50:05	85.8813582	73.846326	91.421441	75.1725398
20:52:55	0:50:06	85.8813582	73.846326	91.421441	75.1725398
20:52:56	0:50:07	85.8813582	73.846326	90.421441	75.1725398
20:52:57	0:50:08	85.8813582	73.846326	90.421441	75.1725398
20:52:58	0:50:09	85.8813582	73.846326	91.421441	75.1725398
20:52:59	0:50:10	82.8813582	74.846326	100.421441	75.1725398
20:53:00	0:50:11	83.8813582	74.846326	101.421441	74.1725398
20:53:01	0:50:12	82.8813582	75.846326	101.421441	74.1725398

20:53:02	0:50:13	79.8813582	75.846326	101.421441	74.1725398
20:53:03	0:50:14	78.8813582	76.846326	101.421441	73.1725398
20:53:04	0:50:15	76.8813582	78.846326	102.421441	73.1725398
20:53:05	0:50:16	74.8813582	78.846326	103.421441	74.1725398
20:53:06	0:50:17	69.8813582	78.846326	103.421441	74.1725398
20:53:07	0:50:18	68.8813582	78.846326	103.421441	73.1725398
20:53:08	0:50:19	67.8813582	78.846326	103.421441	73.1725398
20:53:09	0:50:20	67.8813582	79.846326	102.421441	73.1725398
20:53:10	0:50:21	67.8813582	79.846326	101.421441	72.1725398
20:53:11	0:50:22	67.8813582	79.846326	101.421441	71.1725398
20:53:12	0:50:23	67.8813582	80.846326	101.421441	71.1725398
20:53:13	0:50:24	66.8813582	74.846326	99.421441	71.1725398
20:53:14	0:50:25	66.8813582	73.846326	96.421441	71.1725398
20:53:15	0:50:26	66.8813582	70.846326	93.421441	72.1725398
20:53:16	0:50:27	66.8813582	70.846326	93.421441	72.1725398
20:53:17	0:50:28	66.8813582	68.846326	92.421441	75.1725398
20:53:18	0:50:29	66.8813582	65.846326	92.421441	76.1725398
20:53:19	0:50:30	66.8813582	65.846326	93.421441	76.1725398
20:53:20	0:50:31	65.8813582	65.846326	94.421441	76.1725398
20:53:21	0:50:32	66.8813582	64.846326	94.421441	76.1725398
20:53:22	0:50:33	66.8813582	63.846326	94.421441	75.1725398
20:53:23	0:50:34	66.8813582	62.846326	95.421441	75.1725398
20:53:24	0:50:35	66.8813582	62.846326	96.421441	75.1725398
20:53:25	0:50:36	66.8813582	62.846326	96.421441	75.1725398
20:53:26	0:50:37	66.8813582	61.846326	96.421441	75.1725398
20:53:27	0:50:38	66.8813582	61.846326	97.421441	75.1725398
20:53:28	0:50:39	65.8813582	61.846326	98.421441	76.1725398
20:53:29	0:50:40	64.8813582	63.846326	98.421441	76.1725398
20:53:30	0:50:41	65.8813582	64.846326	98.421441	76.1725398
20:53:31	0:50:42	65.8813582	64.846326	96.421441	80.1725398
20:53:32	0:50:43	65.8813582	64.846326	95.421441	80.1725398
20:53:33	0:50:44	66.8813582	65.846326	92.421441	77.1725398
20:53:34	0:50:45	65.8813582	65.846326	92.421441	74.1725398
20:53:35	0:50:46	65.8813582	64.846326	91.421441	74.1725398
20:53:36	0:50:47	65.8813582	62.846326	89.421441	71.1725398
20:53:37	0:50:48	64.8813582	63.846326	89.421441	71.1725398
20:53:38	0:50:49	64.8813582	62.846326	89.421441	72.1725398
20:53:39	0:50:50	63.8813582	62.846326	89.421441	73.1725398
20:53:40	0:50:51	63.8813582	62.846326	88.421441	73.1725398
20:53:41	0:50:52	63.8813582	62.846326	88.421441	73.1725398
20:53:42	0:50:53	63.8813582	62.846326	88.421441	73.1725398
20:53:43	0:50:54	63.8813582	62.846326	88.421441	73.1725398
20:53:44	0:50:55	63.8813582	62.846326	87.421441	72.1725398
20:53:45	0:50:56	64.8813582	61.846326	86.421441	72.1725398
20:53:46	0:50:57	64.8813582	60.846326	87.421441	71.1725398

20:53:47	0:50:58	64.8813582	60.846326	87.421441	72.1725398
20:53:48	0:50:59	63.8813582	60.846326	88.421441	72.1725398
20:53:49	0:51:00	63.8813582	61.846326	88.421441	92.1725398
20:53:50	0:51:01	64.8813582	61.846326	88.421441	95.1725398
20:53:51	0:51:02	64.8813582	61.846326	88.421441	96.1725398
20:53:52	0:51:03	66.8813582	61.846326	89.421441	96.1725398
20:53:53	0:51:04	66.8813582	63.846326	88.421441	97.1725398
20:53:54	0:51:05	66.8813582	65.846326	88.421441	98.1725398
20:53:55	0:51:06	65.8813582	66.846326	88.421441	99.1725398
20:53:56	0:51:07	65.8813582	66.846326	88.421441	99.1725398
20:53:57	0:51:08	65.8813582	68.846326	88.421441	102.17254
20:53:58	0:51:09	65.8813582	69.846326	92.421441	102.17254
20:53:59	0:51:10	65.8813582	68.846326	94.421441	102.17254
20:54:00	0:51:11	65.8813582	68.846326	94.421441	102.17254
20:54:01	0:51:12	65.8813582	64.846326	87.421441	102.17254
20:54:02	0:51:13	65.8813582	63.846326	86.421441	103.17254
20:54:03	0:51:14	64.8813582	62.846326	86.421441	104.17254
20:54:04	0:51:15	63.8813582	60.846326	86.421441	104.17254
20:54:05	0:51:16	63.8813582	60.846326	86.421441	105.17254
20:54:06	0:51:17	63.8813582	59.846326	86.421441	105.17254
20:54:07	0:51:18	63.8813582	59.846326	86.421441	105.17254
20:54:08	0:51:19	63.8813582	59.846326	83.421441	105.17254
20:54:09	0:51:20	63.8813582	59.846326	83.421441	105.17254
20:54:10	0:51:21	63.8813582	59.846326	82.421441	105.17254
20:54:11	0:51:22	63.8813582	60.846326	83.421441	104.17254
20:54:12	0:51:23	63.8813582	61.846326	85.421441	103.17254
20:54:13	0:51:24	65.8813582	61.846326	85.421441	103.17254
20:54:14	0:51:25	65.8813582	62.846326	86.421441	103.17254
20:54:15	0:51:26	65.8813582	62.846326	86.421441	100.17254
20:54:16	0:51:27	65.8813582	62.846326	86.421441	96.1725398
20:54:17	0:51:28	67.8813582	62.846326	86.421441	94.1725398
20:54:18	0:51:29	68.8813582	63.846326	87.421441	91.1725398
20:54:19	0:51:30	68.8813582	63.846326	87.421441	89.1725398
20:54:20	0:51:31	68.8813582	64.846326	87.421441	89.1725398
20:54:21	0:51:32	68.8813582	64.846326	87.421441	88.1725398
20:54:22	0:51:33	69.8813582	65.846326	85.421441	87.1725398
20:54:23	0:51:34	69.8813582	65.846326	86.421441	85.1725398
20:54:24	0:51:35	70.8813582	66.846326	86.421441	84.1725398
20:54:25	0:51:36	72.8813582	67.846326	89.421441	85.1725398
20:54:26	0:51:37	73.8813582	67.846326	91.421441	84.1725398
20:54:27	0:51:38	74.8813582	67.846326	92.421441	83.1725398
20:54:28	0:51:39	74.8813582	67.846326	91.421441	84.1725398
20:54:29	0:51:40	77.8813582	66.846326	91.421441	84.1725398
20:54:30	0:51:41	77.8813582	67.846326	91.421441	86.1725398
20:54:31	0:51:42	81.8813582	69.846326	90.421441	86.1725398

20:54:32	0:51:43	84.8813582	71.846326	90.421441	88.1725398
20:54:33	0:51:44	82.8813582	72.846326	90.421441	90.1725398
20:54:34	0:51:45	81.8813582	72.846326	91.421441	92.1725398
20:54:35	0:51:46	81.8813582	72.846326	91.421441	93.1725398
20:54:36	0:51:47	81.8813582	72.846326	91.421441	93.1725398
20:54:37	0:51:48	79.8813582	72.846326	91.421441	93.1725398
20:54:38	0:51:49	79.8813582	72.846326	89.421441	92.1725398
20:54:39	0:51:50	79.8813582	65.846326	88.421441	92.1725398
20:54:40	0:51:51	79.8813582	65.846326	87.421441	88.1725398
20:54:41	0:51:52	79.8813582	63.846326	87.421441	85.1725398
20:54:42	0:51:53	79.8813582	63.846326	83.421441	84.1725398
20:54:43	0:51:54	79.8813582	63.846326	82.421441	82.1725398
20:54:44	0:51:55	79.8813582	64.846326	80.421441	81.1725398
20:54:45	0:51:56	79.8813582	65.846326	78.421441	81.1725398
20:54:46	0:51:57	81.8813582	65.846326	78.421441	80.1725398
20:54:47	0:51:58	82.8813582	67.846326	77.421441	79.1725398
20:54:48	0:51:59	82.8813582	69.846326	77.421441	78.1725398
20:54:49	0:52:00	83.8813582	70.846326	77.421441	78.1725398
20:54:50	0:52:01	83.8813582	69.846326	77.421441	78.1725398
20:54:51	0:52:02	83.8813582	69.846326	77.421441	75.1725398
20:54:52	0:52:03	83.8813582	69.846326	77.421441	75.1725398
20:54:53	0:52:04	83.8813582	68.846326	78.421441	75.1725398
20:54:54	0:52:05	84.8813582	66.846326	78.421441	75.1725398
20:54:55	0:52:06	84.8813582	65.846326	79.421441	75.1725398
20:54:56	0:52:07	84.8813582	65.846326	79.421441	76.1725398
20:54:57	0:52:08	84.8813582	66.846326	81.421441	76.1725398
20:54:58	0:52:09	84.8813582	66.846326	81.421441	78.1725398
20:54:59	0:52:10	85.8813582	66.846326	81.421441	78.1725398
20:55:00	0:52:11	84.8813582	66.846326	81.421441	78.1725398
20:55:01	0:52:12	83.8813582	66.846326	82.421441	79.1725398
20:55:02	0:52:13	83.8813582	66.846326	82.421441	78.1725398
20:55:03	0:52:14	82.8813582	66.846326	82.421441	76.1725398
20:55:04	0:52:15	81.8813582	66.846326	85.421441	72.1725398
20:55:05	0:52:16	80.8813582	67.846326	85.421441	72.1725398
20:55:06	0:52:17	80.8813582	67.846326	86.421441	73.1725398
20:55:07	0:52:18	80.8813582	67.846326	86.421441	73.1725398
20:55:08	0:52:19	79.8813582	68.846326	89.421441	73.1725398
20:55:09	0:52:20	79.8813582	68.846326	89.421441	73.1725398
20:55:10	0:52:21	77.8813582	69.846326	89.421441	72.1725398
20:55:11	0:52:22	74.8813582	70.846326	89.421441	70.1725398
20:55:12	0:52:23	73.8813582	70.846326	89.421441	69.1725398
20:55:13	0:52:24	74.8813582	75.846326	89.421441	69.1725398
20:55:14	0:52:25	74.8813582	75.846326	91.421441	69.1725398
20:55:15	0:52:26	76.8813582	73.846326	94.421441	69.1725398
20:55:16	0:52:27	75.8813582	71.846326	96.421441	69.1725398

20:55:17	0:52:28		73.8813582	71.846326	96.421441	76.1725398
20:55:18	0:52:29		71.8813582	71.846326	97.421441	79.1725398
20:55:19	0:52:30		69.8813582	71.846326	97.421441	80.1725398
20:55:20	0:52:31		69.8813582	71.846326	96.421441	79.1725398
20:55:21	0:52:32		69.8813582	72.846326	93.421441	77.1725398
20:55:22	0:52:33		68.8813582	73.846326	93.421441	75.1725398
20:55:23	0:52:34		68.8813582	75.846326	92.421441	73.1725398
20:55:24	0:52:35		67.8813582	75.846326	93.421441	73.1725398
20:55:25	0:52:36		67.8813582	76.846326	93.421441	73.1725398
20:55:26	0:52:37		67.8813582	77.846326	93.421441	73.1725398
20:55:27	0:52:38		68.8813582	77.846326	93.421441	73.1725398
20:55:28	0:52:39		69.8813582	77.846326	93.421441	73.1725398
20:55:29	0:52:40		69.8813582	77.846326	93.421441	73.1725398
20:55:30	0:52:41		69.8813582	77.846326	93.421441	73.1725398
20:55:31	0:52:42		71.8813582	76.846326	100.421441	74.1725398
20:55:32	0:52:43		71.8813582	76.846326	103.421441	73.1725398
20:55:33	0:52:44		70.8813582	76.846326	103.421441	73.1725398
20:55:34	0:52:45		70.8813582	76.846326	105.421441	73.1725398
20:55:35	0:52:46		70.8813582	76.846326	104.421441	73.1725398
20:55:36	0:52:47		69.8813582	77.846326	101.421441	72.1725398
20:55:37	0:52:48		69.8813582	78.846326	101.421441	71.1725398
20:55:38	0:52:49		69.8813582	79.846326	91.421441	72.1725398
20:55:39	0:52:50		69.8813582	79.846326	89.421441	74.1725398
20:55:40	0:52:51		68.8813582	80.846326	87.421441	74.1725398
20:55:41	0:52:52		68.8813582	81.846326	87.421441	74.1725398
20:55:42	0:52:53		68.8813582	82.846326	87.421441	73.1725398
20:55:43	0:52:54		68.8813582	85.846326	86.421441	71.1725398
20:55:44	0:52:55		68.8813582	85.846326	84.421441	71.1725398
20:55:45	0:52:56		68.8813582	88.846326	84.421441	71.1725398
20:55:46	0:52:57		68.8813582	89.846326	83.421441	70.1725398
20:55:47	0:52:58		68.8813582	90.846326	83.421441	70.1725398
20:55:48	0:52:59		68.8813582	90.846326	83.421441	71.1725398
20:55:49	0:53:00		68.8813582	90.846326	83.421441	72.1725398
20:55:50	0:53:01		70.8813582	90.846326	84.421441	72.1725398
20:55:51	0:53:02		70.8813582	91.846326	84.421441	77.1725398
20:55:52	0:53:03		71.8813582	86.846326	85.421441	76.1725398
20:55:53	0:53:04		71.8813582	76.846326	85.421441	75.1725398
20:55:54	0:53:05		72.8813582	76.846326	84.421441	75.1725398
20:55:55	0:53:06		73.8813582	71.846326	85.421441	76.1725398
20:55:56	0:53:07		72.8813582	71.846326	85.421441	76.1725398
20:55:57	0:53:08		72.8813582	71.846326	85.421441	75.1725398
20:55:58	0:53:09		72.8813582	72.846326	85.421441	74.1725398
20:55:59	0:53:10		73.8813582	74.846326	85.421441	72.1725398
20:56:00	0:53:11		73.8813582	76.846326	85.421441	70.1725398
20:56:01	0:53:12		74.8813582	76.846326	86.421441	71.1725398

20:56:02	0:53:13	74.8813582	74.846326	86.421441	72.1725398
20:56:03	0:53:14	75.8813582	73.846326	86.421441	72.1725398
20:56:04	0:53:15	76.8813582	71.846326	86.421441	73.1725398
20:56:05	0:53:16	77.8813582	69.846326	86.421441	73.1725398
20:56:06	0:53:17	78.8813582	69.846326	86.421441	71.1725398
20:56:07	0:53:18	77.8813582	67.846326	86.421441	71.1725398
20:56:08	0:53:19	76.8813582	67.846326	86.421441	70.1725398
20:56:09	0:53:20	75.8813582	67.846326	85.421441	69.1725398
20:56:10	0:53:21	75.8813582	67.846326	85.421441	70.1725398
20:56:11	0:53:22	75.8813582	67.846326	83.421441	71.1725398
20:56:12	0:53:23	75.8813582	68.846326	83.421441	73.1725398
20:56:13	0:53:24	75.8813582	68.846326	83.421441	74.1725398
20:56:14	0:53:25	75.8813582	68.846326	83.421441	74.1725398
20:56:15	0:53:26	75.8813582	68.846326	83.421441	74.1725398
20:56:16	0:53:27	75.8813582	68.846326	82.421441	73.1725398
20:56:17	0:53:28	75.8813582	67.846326	82.421441	71.1725398
20:56:18	0:53:29	75.8813582	67.846326	82.421441	71.1725398
20:56:19	0:53:30	75.8813582	67.846326	83.421441	70.1725398
20:56:20	0:53:31	75.8813582	68.846326	84.421441	70.1725398
20:56:21	0:53:32	73.8813582	69.846326	84.421441	70.1725398
20:56:22	0:53:33	72.8813582	69.846326	84.421441	72.1725398
20:56:23	0:53:34	72.8813582	69.846326	83.421441	73.1725398
20:56:24	0:53:35	72.8813582	69.846326	83.421441	73.1725398
20:56:25	0:53:36	70.8813582	72.846326	83.421441	74.1725398
20:56:26	0:53:37	69.8813582	72.846326	83.421441	73.1725398
20:56:27	0:53:38	69.8813582	72.846326	83.421441	71.1725398
20:56:28	0:53:39	68.8813582	71.846326	81.421441	68.1725398
20:56:29	0:53:40	68.8813582	70.846326	81.421441	66.1725398
20:56:30	0:53:41	68.8813582	71.846326	81.421441	66.1725398
20:56:31	0:53:42	70.8813582	71.846326	81.421441	67.1725398
20:56:32	0:53:43	70.8813582	71.846326	81.421441	67.1725398
20:56:33	0:53:44	70.8813582	68.846326	81.421441	68.1725398
20:56:34	0:53:45	69.8813582	66.846326	80.421441	69.1725398
20:56:35	0:53:46	69.8813582	65.846326	80.421441	69.1725398
20:56:36	0:53:47	69.8813582	65.846326	81.421441	69.1725398
20:56:37	0:53:48	68.8813582	65.846326	81.421441	70.1725398
20:56:38	0:53:49	67.8813582	65.846326	81.421441	70.1725398
20:56:39	0:53:50	67.8813582	65.846326	82.421441	69.1725398
20:56:40	0:53:51	66.8813582	64.846326	82.421441	69.1725398
20:56:41	0:53:52	66.8813582	64.846326	82.421441	69.1725398
20:56:42	0:53:53	66.8813582	64.846326	82.421441	69.1725398
20:56:43	0:53:54	66.8813582	64.846326	83.421441	70.1725398
20:56:44	0:53:55	66.8813582	65.846326	84.421441	71.1725398
20:56:45	0:53:56	66.8813582	65.846326	84.421441	71.1725398
20:56:46	0:53:57	66.8813582	66.846326	84.421441	71.1725398

20:56:47	0:53:58	65.8813582	66.846326	84.421441	72.1725398
20:56:48	0:53:59	65.8813582	66.846326	84.421441	72.1725398
20:56:49	0:54:00	66.8813582	67.846326	84.421441	70.1725398
20:56:50	0:54:01	66.8813582	67.846326	84.421441	70.1725398
20:56:51	0:54:02	65.8813582	67.846326	84.421441	70.1725398
20:56:52	0:54:03	65.8813582	67.846326	84.421441	72.1725398
20:56:53	0:54:04	63.8813582	67.846326	84.421441	72.1725398
20:56:54	0:54:05	60.8813582	68.846326	84.421441	72.1725398
20:56:55	0:54:06	60.8813582	68.846326	83.421441	73.1725398
20:56:56	0:54:07	60.8813582	68.846326	81.421441	74.1725398
20:56:57	0:54:08	60.8813582	67.846326	80.421441	74.1725398
20:56:58	0:54:09	60.8813582	66.846326	80.421441	74.1725398
20:56:59	0:54:10	60.8813582	64.846326	80.421441	74.1725398
20:57:00	0:54:11	60.8813582	61.846326	81.421441	73.1725398
20:57:01	0:54:12	60.8813582	61.846326	83.421441	71.1725398
20:57:02	0:54:13	60.8813582	61.846326	85.421441	71.1725398
20:57:03	0:54:14	59.8813582	64.846326	85.421441	70.1725398
20:57:04	0:54:15	59.8813582	64.846326	85.421441	69.1725398
20:57:05	0:54:16	59.8813582	64.846326	85.421441	70.1725398
20:57:06	0:54:17	58.8813582	64.846326	86.421441	71.1725398
20:57:07	0:54:18	58.8813582	66.846326	87.421441	72.1725398
20:57:08	0:54:19	58.8813582	70.846326	87.421441	73.1725398
20:57:09	0:54:20	58.8813582	72.846326	87.421441	73.1725398
20:57:10	0:54:21	58.8813582	72.846326	88.421441	74.1725398
20:57:11	0:54:22	57.8813582	71.846326	88.421441	74.1725398
20:57:12	0:54:23	57.8813582	71.846326	89.421441	71.1725398
20:57:13	0:54:24	57.8813582	72.846326	89.421441	71.1725398
20:57:14	0:54:25	57.8813582	72.846326	89.421441	66.1725398
20:57:15	0:54:26	57.8813582	72.846326	89.421441	66.1725398
20:57:16	0:54:27	59.8813582	72.846326	89.421441	65.1725398
20:57:17	0:54:28	59.8813582	72.846326	89.421441	65.1725398
20:57:18	0:54:29	59.8813582	72.846326	89.421441	65.1725398
20:57:19	0:54:30	60.8813582	75.846326	81.421441	68.1725398
20:57:20	0:54:31	60.8813582	76.846326	80.421441	68.1725398
20:57:21	0:54:32	61.8813582	76.846326	80.421441	68.1725398
20:57:22	0:54:33	62.8813582	76.846326	77.421441	65.1725398
20:57:23	0:54:34	63.8813582	76.846326	77.421441	62.1725398
20:57:24	0:54:35	64.8813582	75.846326	77.421441	62.1725398
20:57:25	0:54:36	64.8813582	74.846326	77.421441	62.1725398
20:57:26	0:54:37	64.8813582	73.846326	77.421441	61.1725398
20:57:27	0:54:38	66.8813582	73.846326	76.421441	61.1725398
20:57:28	0:54:39	66.8813582	73.846326	76.421441	61.1725398
20:57:29	0:54:40	67.8813582	74.846326	76.421441	61.1725398
20:57:30	0:54:41	66.8813582	74.846326	75.421441	61.1725398
20:57:31	0:54:42	67.8813582	74.846326	75.421441	61.1725398

20:57:32	0:54:43	67.8813582	74.846326	74.421441	61.1725398
20:57:33	0:54:44	68.8813582	74.846326	74.421441	62.1725398
20:57:34	0:54:45	68.8813582	74.846326	74.421441	62.1725398
20:57:35	0:54:46	69.8813582	74.846326	73.421441	62.1725398
20:57:36	0:54:47	70.8813582	74.846326	73.421441	65.1725398
20:57:37	0:54:48	69.8813582	75.846326	73.421441	77.1725398
20:57:38	0:54:49	68.8813582	75.846326	73.421441	77.1725398
20:57:39	0:54:50	67.8813582	76.846326	72.421441	76.1725398
20:57:40	0:54:51	66.8813582	76.846326	73.421441	74.1725398
20:57:41	0:54:52	66.8813582	77.846326	73.421441	73.1725398
20:57:42	0:54:53	66.8813582	89.846326	74.421441	71.1725398
20:57:43	0:54:54	66.8813582	89.846326	74.421441	69.1725398
20:57:44	0:54:55	67.8813582	94.846326	74.421441	67.1725398
20:57:45	0:54:56	68.8813582	95.846326	79.421441	66.1725398
20:57:46	0:54:57	69.8813582	95.846326	81.421441	67.1725398
20:57:47	0:54:58	71.8813582	100.846326	83.421441	69.1725398
20:57:48	0:54:59	73.8813582	102.846326	83.421441	69.1725398
20:57:49	0:55:00	73.8813582	102.846326	83.421441	69.1725398
20:57:50	0:55:01	73.8813582	102.846326	83.421441	68.1725398
20:57:51	0:55:02	72.8813582	102.846326	83.421441	68.1725398
20:57:52	0:55:03	72.8813582	102.846326	83.421441	67.1725398
20:57:53	0:55:04	70.8813582	102.846326	83.421441	65.1725398
20:57:54	0:55:05	68.8813582	78.8953836	83.421441	64.1725398
20:57:55	0:55:06	67.8813582	78.2953836	81.421441	64.1725398
20:57:56	0:55:07	67.8813582	78.6953836	81.421441	65.1725398
20:57:57	0:55:08	66.8813582	78.4953836	80.421441	65.1725398
20:57:58	0:55:09	67.8813582	77.6953836	73.421441	66.1725398
20:57:59	0:55:10	67.8813582	77.8953836	73.421441	67.1725398
20:58:00	0:55:11	67.8813582	102.846326	73.421441	74.1725398
20:58:01	0:55:12	67.8813582	98.846326	71.421441	74.1725398
20:58:02	0:55:13	67.8813582	97.846326	71.421441	78.1725398
20:58:03	0:55:14	67.8813582	97.846326	71.421441	79.1725398
20:58:04	0:55:15	68.8813582	97.846326	75.421441	79.1725398
20:58:05	0:55:16	69.8813582	97.846326	83.421441	77.1725398
20:58:06	0:55:17	69.8813582	98.846326	84.421441	70.1725398
20:58:07	0:55:18	68.8813582	97.846326	84.421441	67.1725398
20:58:08	0:55:19	67.8813582	93.846326	83.421441	67.1725398
20:58:09	0:55:20	67.8813582	91.846326	84.421441	66.1725398
20:58:10	0:55:21	67.8813582	88.846326	84.421441	66.1725398
20:58:11	0:55:22	66.8813582	87.846326	84.421441	66.1725398
20:58:12	0:55:23	66.8813582	87.846326	84.421441	66.1725398
20:58:13	0:55:24	66.8813582	87.846326	81.421441	66.1725398
20:58:14	0:55:25	66.8813582	84.846326	81.421441	66.1725398
20:58:15	0:55:26	66.8813582	84.846326	79.421441	66.1725398
20:58:16	0:55:27	66.8813582	83.846326	80.421441	66.1725398

20:58:17	0:55:28	66.8813582	82.846326	81.421441	69.1725398
20:58:18	0:55:29	66.8813582	81.846326	81.421441	69.1725398
20:58:19	0:55:30	66.8813582	81.846326	80.421441	69.1725398
20:58:20	0:55:31	66.8813582	81.846326	80.421441	69.1725398
20:58:21	0:55:32	65.8813582	81.846326	79.421441	69.1725398
20:58:22	0:55:33	65.8813582	81.846326	80.421441	67.1725398
20:58:23	0:55:34	66.8813582	81.846326	80.421441	74.1725398
20:58:24	0:55:35	66.8813582	81.846326	80.421441	74.1725398
20:58:25	0:55:36	66.8813582	81.846326	80.421441	73.1725398
20:58:26	0:55:37	66.8813582	81.846326	81.421441	72.1725398
20:58:27	0:55:38	66.8813582	81.846326	81.421441	72.1725398
20:58:28	0:55:39	67.8813582	80.846326	82.421441	72.1725398
20:58:29	0:55:40	67.8813582	79.846326	83.421441	72.1725398
20:58:30	0:55:41	67.8813582	79.846326	84.421441	72.1725398
20:58:31	0:55:42	67.8813582	79.846326	85.421441	72.1725398
20:58:32	0:55:43	66.8813582	79.846326	85.421441	70.1725398
20:58:33	0:55:44	66.8813582	79.846326	86.421441	69.1725398
20:58:34	0:55:45	66.8813582	79.846326	85.421441	69.1725398
20:58:35	0:55:46	66.8813582	79.846326	85.421441	69.1725398
20:58:36	0:55:47	66.8813582	79.846326	85.421441	68.1725398
20:58:37	0:55:48	66.8813582	78.846326	85.421441	68.1725398
20:58:38	0:55:49	65.8813582	78.846326	84.421441	67.1725398
20:58:39	0:55:50	65.8813582	78.846326	82.421441	67.1725398
20:58:40	0:55:51	65.8813582	78.846326	82.421441	67.1725398
20:58:41	0:55:52	65.8813582	78.846326	82.421441	67.1725398
20:58:42	0:55:53	65.8813582	80.846326	82.421441	67.1725398
20:58:43	0:55:54	65.8813582	77.846326	82.421441	66.1725398
20:58:44	0:55:55	66.8813582	77.846326	82.421441	66.1725398
20:58:45	0:55:56	66.8813582	76.846326	82.421441	66.1725398
20:58:46	0:55:57	66.8813582	75.846326	82.421441	64.1725398
20:58:47	0:55:58	67.8813582	75.846326	82.421441	64.1725398
20:58:48	0:55:59	67.8813582	75.846326	82.421441	64.1725398
20:58:49	0:56:00	67.8813582	77.846326	82.421441	64.1725398
20:58:50	0:56:01	68.8813582	77.846326	82.421441	64.1725398
20:58:51	0:56:02	68.8813582	77.846326	81.421441	64.1725398
20:58:52	0:56:03	69.8813582	76.846326	81.421441	64.1725398
20:58:53	0:56:04	70.8813582	75.846326	81.421441	64.1725398
20:58:54	0:56:05	70.8813582	74.846326	81.421441	66.1725398
20:58:55	0:56:06	72.8813582	74.846326	81.421441	66.1725398
20:58:56	0:56:07	71.8813582	73.846326	81.421441	63.1725398
20:58:57	0:56:08	71.8813582	72.846326	81.421441	63.1725398
20:58:58	0:56:09	67.8813582	72.846326	80.421441	63.1725398
20:58:59	0:56:10	67.8813582	72.846326	79.421441	68.1725398
20:59:00	0:56:11	67.8813582	72.846326	79.421441	68.1725398
20:59:01	0:56:12	67.8813582	71.846326	79.421441	68.1725398

20:59:02	0:56:13	67.8813582	71.846326	83.421441	74.1725398
20:59:03	0:56:14	67.8813582	71.846326	84.421441	74.1725398
20:59:04	0:56:15	66.8813582	71.846326	85.421441	71.1725398
20:59:05	0:56:16	66.8813582	70.846326	85.421441	71.1725398
20:59:06	0:56:17	66.8813582	70.846326	85.421441	71.1725398
20:59:07	0:56:18	66.8813582	70.846326	85.421441	70.1725398
20:59:08	0:56:19	66.8813582	67.846326	86.421441	70.1725398
20:59:09	0:56:20	66.8813582	70.846326	87.421441	70.1725398
20:59:10	0:56:21	67.8813582	70.846326	87.421441	70.1725398
20:59:11	0:56:22	67.8813582	71.846326	87.421441	70.1725398
20:59:12	0:56:23	67.8813582	71.846326	87.421441	75.1725398
20:59:13	0:56:24	67.8813582	71.846326	87.421441	76.1725398
20:59:14	0:56:25	68.8813582	71.846326	87.421441	72.1725398
20:59:15	0:56:26	68.8813582	74.846326	87.421441	72.1725398
20:59:16	0:56:27	68.8813582	76.846326	89.421441	69.1725398
20:59:17	0:56:28	68.8813582	76.846326	91.421441	69.1725398
20:59:18	0:56:29	68.8813582	77.846326	91.421441	66.1725398
20:59:19	0:56:30	68.8813582	82.846326	91.421441	66.1725398
20:59:20	0:56:31	69.8813582	82.846326	90.421441	66.1725398
20:59:21	0:56:32	69.8813582	96.846326	90.421441	70.1725398
20:59:22	0:56:33	69.8813582	99.846326	90.421441	71.1725398
20:59:23	0:56:34	70.8813582	99.846326	90.421441	71.1725398
20:59:24	0:56:35	71.8813582	102.846326	88.421441	72.1725398
20:59:25	0:56:36	70.8813582	102.846326	88.421441	73.1725398
20:59:26	0:56:37	69.8813582	102.846326	90.421441	73.1725398
20:59:27	0:56:38	69.8813582	103.846326	89.421441	72.1725398
20:59:28	0:56:39	68.8813582	103.846326	89.421441	71.1725398
20:59:29	0:56:40	68.8813582	103.846326	89.421441	71.1725398
20:59:30	0:56:41	67.8813582	103.846326	89.421441	71.1725398
20:59:31	0:56:42	67.8813582	99.846326	89.421441	71.1725398
20:59:32	0:56:43	66.8813582	99.846326	89.421441	71.1725398
20:59:33	0:56:44	66.8813582	98.846326	90.421441	75.1725398
20:59:34	0:56:45	66.8813582	98.846326	89.421441	75.1725398
20:59:35	0:56:46	67.8813582	98.846326	89.421441	75.1725398
20:59:36	0:56:47	67.8813582	96.846326	88.421441	70.1725398
20:59:37	0:56:48	67.8813582	95.846326	89.421441	69.1725398
20:59:38	0:56:49	67.8813582	95.846326	94.421441	69.1725398
20:59:39	0:56:50	67.8813582	94.846326	94.421441	71.1725398
20:59:40	0:56:51	68.8813582	94.846326	95.421441	76.1725398
20:59:41	0:56:52	67.8813582	100.846326	96.421441	79.1725398
20:59:42	0:56:53	67.8813582	102.846326	96.421441	80.1725398
20:59:43	0:56:54	67.8813582	89.846326	95.421441	79.1725398
20:59:44	0:56:55	67.8813582	87.846326	95.421441	76.1725398
20:59:45	0:56:56	67.8813582	87.846326	95.421441	72.1725398
20:59:46	0:56:57	67.8813582	87.846326	94.421441	71.1725398

20:59:47	0:56:58	67.8813582	85.846326	92.421441	71.1725398
20:59:48	0:56:59	67.8813582	79.846326	92.421441	71.1725398
20:59:49	0:57:00	67.8813582	76.846326	90.421441	70.1725398
20:59:50	0:57:01	67.8813582	72.846326	90.421441	72.1725398
20:59:51	0:57:02	66.8813582	66.846326	90.421441	73.1725398
20:59:52	0:57:03	66.8813582	66.846326	92.421441	72.1725398
20:59:53	0:57:04	65.8813582	65.846326	93.421441	70.1725398
20:59:54	0:57:05	65.8813582	64.846326	94.421441	69.1725398
20:59:55	0:57:06	66.8813582	64.846326	94.421441	69.1725398
20:59:56	0:57:07	66.8813582	63.846326	94.421441	70.1725398
20:59:57	0:57:08	66.8813582	63.846326	94.421441	70.1725398
20:59:58	0:57:09	66.8813582	63.846326	94.421441	71.1725398
20:59:59	0:57:10	66.8813582	63.846326	94.421441	70.1725398
21:00:00	0:57:11	66.8813582	63.846326	94.421441	70.1725398
21:00:01	0:57:12	65.8813582	61.846326	94.421441	69.1725398
21:00:02	0:57:13	65.8813582	61.846326	93.421441	68.1725398
21:00:03	0:57:14	65.8813582	61.846326	93.421441	69.1725398
21:00:04	0:57:15	64.8813582	63.846326	93.421441	69.1725398
21:00:05	0:57:16	64.8813582	63.846326	93.421441	71.1725398
21:00:06	0:57:17	64.8813582	66.846326	93.421441	71.1725398
21:00:07	0:57:18	64.8813582	67.846326	93.421441	71.1725398
21:00:08	0:57:19	65.8813582	67.846326	93.421441	69.1725398
21:00:09	0:57:20	66.8813582	67.846326	93.421441	68.1725398
21:00:10	0:57:21	66.8813582	71.846326	94.421441	67.1725398
21:00:11	0:57:22	66.8813582	74.846326	95.421441	66.1725398
21:00:12	0:57:23	66.8813582	74.846326	96.421441	67.1725398
21:00:13	0:57:24	65.8813582	74.846326	96.421441	67.1725398
21:00:14	0:57:25	65.8813582	74.846326	96.421441	68.1725398
21:00:15	0:57:26	65.8813582	72.846326	96.421441	69.1725398
21:00:16	0:57:27	65.8813582	71.846326	96.421441	69.1725398
21:00:17	0:57:28	65.8813582	70.846326	96.421441	70.1725398
21:00:18	0:57:29	66.8813582	70.846326	96.421441	68.1725398
21:00:19	0:57:30	68.8813582	70.846326	95.421441	67.1725398
21:00:20	0:57:31	68.8813582	70.846326	93.421441	67.1725398
21:00:21	0:57:32	68.8813582	71.846326	91.421441	66.1725398
21:00:22	0:57:33	69.8813582	71.846326	90.421441	66.1725398
21:00:23	0:57:34	70.8813582	72.846326	89.421441	66.1725398
21:00:24	0:57:35	70.8813582	73.846326	88.421441	66.1725398
21:00:25	0:57:36	69.8813582	75.846326	86.421441	66.1725398
21:00:26	0:57:37	69.8813582	73.846326	83.421441	66.1725398
21:00:27	0:57:38	69.8813582	73.846326	82.421441	65.1725398
21:00:28	0:57:39	69.8813582	73.846326	81.421441	65.1725398
21:00:29	0:57:40	69.8813582	74.846326	80.421441	65.1725398
21:00:30	0:57:41	69.8813582	74.846326	80.421441	65.1725398
21:00:31	0:57:42	68.8813582	73.846326	79.421441	65.1725398

21:00:32	0:57:43	68.8813582	68.846326	79.421441	65.1725398
21:00:33	0:57:44	67.8813582	66.846326	78.421441	65.1725398
21:00:34	0:57:45	67.8813582	66.846326	78.421441	65.1725398
21:00:35	0:57:46	66.8813582	66.846326	78.421441	64.1725398
21:00:36	0:57:47	67.8813582	65.846326	77.421441	65.1725398
21:00:37	0:57:48	67.8813582	62.846326	77.421441	66.1725398
21:00:38	0:57:49	67.8813582	62.846326	78.421441	67.1725398
21:00:39	0:57:50	67.8813582	62.846326	78.421441	68.1725398
21:00:40	0:57:51	67.8813582	62.846326	78.421441	68.1725398
21:00:41	0:57:52	67.8813582	62.846326	79.421441	68.1725398
21:00:42	0:57:53	67.8813582	62.846326	78.421441	67.1725398
21:00:43	0:57:54	67.8813582	62.846326	78.421441	66.1725398
21:00:44	0:57:55	67.8813582	62.846326	77.421441	65.1725398
21:00:45	0:57:56	67.8813582	62.846326	77.421441	65.1725398
21:00:46	0:57:57	67.8813582	62.846326	77.421441	66.1725398
21:00:47	0:57:58	67.8813582	62.846326	78.421441	66.1725398
21:00:48	0:57:59	67.8813582	62.846326	80.421441	66.1725398
21:00:49	0:58:00	67.8813582	62.846326	80.421441	67.1725398
21:00:50	0:58:01	67.8813582	62.846326	81.421441	68.1725398
21:00:51	0:58:02	67.8813582	62.846326	81.421441	68.1725398
21:00:52	0:58:03	67.8813582	62.846326	79.421441	66.1725398
21:00:53	0:58:04	67.8813582	62.846326	79.421441	65.1725398
21:00:54	0:58:05	67.8813582	62.846326	79.421441	65.1725398
21:00:55	0:58:06	67.8813582	62.846326	77.421441	65.1725398
21:00:56	0:58:07	67.8813582	62.846326	79.421441	67.1725398
21:00:57	0:58:08	69.8813582	62.846326	80.421441	70.1725398
21:00:58	0:58:09	69.8813582	61.846326	80.421441	72.1725398
21:00:59	0:58:10	69.8813582	62.846326	80.421441	72.1725398
21:01:00	0:58:11	71.8813582	62.846326	79.421441	73.1725398
21:01:01	0:58:12	71.8813582	62.846326	79.421441	73.1725398
21:01:02	0:58:13	72.8813582	62.846326	80.421441	74.1725398
21:01:03	0:58:14	72.8813582	62.846326	80.421441	76.1725398
21:01:04	0:58:15	72.8813582	62.846326	80.421441	76.1725398
21:01:05	0:58:16	71.8813582	62.846326	81.421441	76.1725398
21:01:06	0:58:17	69.8813582	62.846326	82.421441	74.1725398
21:01:07	0:58:18	68.8813582	61.846326	83.421441	73.1725398
21:01:08	0:58:19	67.8813582	62.846326	83.421441	73.1725398
21:01:09	0:58:20	66.8813582	62.846326	85.421441	75.1725398
21:01:10	0:58:21	66.8813582	62.846326	86.421441	76.1725398
21:01:11	0:58:22	65.8813582	62.846326	86.421441	77.1725398
21:01:12	0:58:23	66.8813582	65.846326	86.421441	78.1725398
21:01:13	0:58:24	66.8813582	65.846326	87.421441	78.1725398
21:01:14	0:58:25	66.8813582	65.846326	87.421441	79.1725398
21:01:15	0:58:26	66.8813582	65.846326	86.421441	79.1725398
21:01:16	0:58:27	66.8813582	69.846326	85.421441	78.1725398

21:01:17	0:58:28	66.8813582	78.846326	85.421441	78.1725398
21:01:18	0:58:29	66.8813582	79.846326	85.421441	76.1725398
21:01:19	0:58:30	66.8813582	79.846326	84.421441	75.1725398
21:01:20	0:58:31	66.8813582	81.846326	84.421441	76.1725398
21:01:21	0:58:32	66.8813582	81.846326	83.421441	77.1725398
21:01:22	0:58:33	66.8813582	81.846326	82.421441	77.1725398
21:01:23	0:58:34	65.8813582	81.846326	81.421441	78.1725398
21:01:24	0:58:35	65.8813582	81.846326	82.421441	78.1725398
21:01:25	0:58:36	65.8813582	81.846326	82.421441	78.1725398
21:01:26	0:58:37	65.8813582	80.846326	82.421441	77.1725398
21:01:27	0:58:38	65.8813582	80.846326	82.421441	77.1725398
21:01:28	0:58:39	65.8813582	80.846326	82.421441	77.1725398
21:01:29	0:58:40	65.8813582	79.846326	83.421441	80.1725398
21:01:30	0:58:41	63.8813582	79.846326	83.421441	80.1725398
21:01:31	0:58:42	63.8813582	79.846326	84.421441	80.1725398
21:01:32	0:58:43	62.8813582	78.846326	84.421441	79.1725398
21:01:33	0:58:44	63.8813582	76.846326	85.421441	78.1725398
21:01:34	0:58:45	63.8813582	75.846326	85.421441	76.1725398
21:01:35	0:58:46	63.8813582	75.846326	86.421441	76.1725398
21:01:36	0:58:47	63.8813582	75.846326	86.421441	77.1725398
21:01:37	0:58:48	62.8813582	72.846326	86.421441	78.1725398
21:01:38	0:58:49	63.8813582	71.846326	85.421441	78.1725398
21:01:39	0:58:50	63.8813582	71.846326	86.421441	78.1725398
21:01:40	0:58:51	64.8813582	71.846326	86.421441	77.1725398
21:01:41	0:58:52	64.8813582	71.846326	86.421441	77.1725398
21:01:42	0:58:53	64.8813582	69.846326	86.421441	77.1725398
21:01:43	0:58:54	64.8813582	69.846326	86.421441	77.1725398
21:01:44	0:58:55	64.8813582	68.846326	86.421441	76.1725398
21:01:45	0:58:56	64.8813582	67.846326	86.421441	76.1725398
21:01:46	0:58:57	64.8813582	67.846326	86.421441	76.1725398
21:01:47	0:58:58	64.8813582	66.846326	87.421441	76.1725398
21:01:48	0:58:59	64.8813582	66.846326	87.421441	76.1725398
21:01:49	0:59:00	64.8813582	67.846326	87.421441	76.1725398
21:01:50	0:59:01	65.8813582	67.846326	87.421441	77.1725398
21:01:51	0:59:02	65.8813582	67.846326	87.421441	77.1725398
21:01:52	0:59:03	65.8813582	68.846326	87.421441	80.1725398
21:01:53	0:59:04	65.8813582	68.846326	87.421441	80.1725398
21:01:54	0:59:05	65.8813582	67.846326	87.421441	79.1725398
21:01:55	0:59:06	66.8813582	67.846326	90.421441	79.1725398
21:01:56	0:59:07	66.8813582	67.846326	90.421441	78.1725398
21:01:57	0:59:08	67.8813582	67.846326	92.421441	75.1725398
21:01:58	0:59:09	67.8813582	67.846326	90.421441	68.1725398
21:01:59	0:59:10	67.8813582	68.846326	90.421441	66.1725398
21:02:00	0:59:11	67.8813582	68.846326	89.421441	66.1725398
21:02:01	0:59:12	67.8813582	71.846326	88.421441	66.1725398

21:02:02	0:59:13	67.8813582	72.846326	87.421441	67.1725398
21:02:03	0:59:14	67.8813582	72.846326	86.421441	68.1725398
21:02:04	0:59:15	67.8813582	72.846326	86.421441	68.1725398
21:02:05	0:59:16	66.8813582	72.846326	84.421441	68.1725398
21:02:06	0:59:17	66.8813582	73.846326	84.421441	65.1725398
21:02:07	0:59:18	66.8813582	74.846326	84.421441	63.1725398
21:02:08	0:59:19	66.8813582	77.846326	84.421441	61.1725398
21:02:09	0:59:20	64.8813582	79.846326	84.421441	61.1725398
21:02:10	0:59:21	64.8813582	82.846326	83.421441	61.1725398
21:02:11	0:59:22	63.8813582	82.846326	83.421441	62.1725398
21:02:12	0:59:23	64.8813582	82.846326	83.421441	63.1725398
21:02:13	0:59:24	65.8813582	83.846326	82.421441	63.1725398
21:02:14	0:59:25	65.8813582	83.846326	82.421441	64.1725398
21:02:15	0:59:26	65.8813582	83.846326	81.421441	65.1725398
21:02:16	0:59:27	65.8813582	83.846326	81.421441	67.1725398
21:02:17	0:59:28	65.8813582	83.846326	78.421441	68.1725398
21:02:18	0:59:29	66.8813582	86.846326	77.421441	68.1725398
21:02:19	0:59:30	65.8813582	82.846326	76.421441	67.1725398
21:02:20	0:59:31	65.8813582	81.846326	76.421441	67.1725398
21:02:21	0:59:32	65.8813582	81.846326	76.421441	66.1725398
21:02:22	0:59:33	65.8813582	81.846326	76.421441	65.1725398
21:02:23	0:59:34	65.8813582	81.846326	75.421441	65.1725398
21:02:24	0:59:35	65.8813582	80.846326	75.421441	66.1725398
21:02:25	0:59:36	65.8813582	80.846326	75.421441	69.1725398
21:02:26	0:59:37	65.8813582	80.846326	75.421441	70.1725398
21:02:27	0:59:38	65.8813582	80.846326	73.421441	68.1725398
21:02:28	0:59:39	65.8813582	80.846326	73.421441	67.1725398
21:02:29	0:59:40	64.8813582	80.846326	73.421441	66.1725398
21:02:30	0:59:41	64.8813582	80.846326	72.421441	66.1725398
21:02:31	0:59:42	64.8813582	80.846326	72.421441	67.1725398
21:02:32	0:59:43	64.8813582	80.846326	72.421441	67.1725398
21:02:33	0:59:44	64.8813582	80.846326	72.421441	68.1725398
21:02:34	0:59:45	65.8813582	80.846326	72.421441	68.1725398
21:02:35	0:59:46	65.8813582	79.846326	72.421441	67.1725398
21:02:36	0:59:47	65.8813582	75.846326	74.421441	68.1725398
21:02:37	0:59:48	65.8813582	71.846326	75.421441	69.1725398
21:02:38	0:59:49	65.8813582	67.846326	75.421441	69.1725398
21:02:39	0:59:50	65.8813582	67.846326	76.421441	70.1725398
21:02:40	0:59:51	65.8813582	66.846326	76.421441	71.1725398
21:02:41	0:59:52	65.8813582	66.846326	76.421441	72.1725398
21:02:42	0:59:53	65.8813582	66.846326	76.421441	72.1725398
21:02:43	0:59:54	66.8813582	66.846326	80.421441	71.1725398
21:02:44	0:59:55	66.8813582	66.846326	80.421441	71.1725398
21:02:45	0:59:56	65.8813582	67.846326	80.421441	71.1725398
21:02:46	0:59:57	66.8813582	67.846326	80.421441	70.1725398

21:02:47	0:59:58	66.8813582	68.846326	79.421441	70.1725398
21:02:48	0:59:59	67.8813582	68.846326	78.421441	70.1725398
21:02:49	1:00:00	66.8813582	68.846326	78.421441	70.1725398
21:02:50	1:00:01	67.8813582	69.846326	78.421441	70.1725398
21:02:51	1:00:02	67.8813582	69.846326	78.421441	69.1725398
21:02:52	1:00:03	67.8813582	69.846326	78.421441	68.1725398
21:02:53	1:00:04	68.8813582	69.846326	78.421441	68.1725398
21:02:54	1:00:05	69.8813582	69.846326	81.421441	67.1725398
21:02:55	1:00:06	70.8813582	69.846326	83.421441	66.1725398
21:02:56	1:00:07	68.8813582	68.846326	85.421441	66.1725398
21:02:57	1:00:08	67.8813582	68.846326	85.421441	67.1725398
21:02:58	1:00:09	67.8813582	68.846326	85.421441	68.1725398
21:02:59	1:00:10	67.8813582	68.846326	86.421441	69.1725398
21:03:00	1:00:11	67.8813582	69.846326	87.421441	70.1725398
21:03:01	1:00:12	67.8813582	70.846326	87.421441	70.1725398
21:03:02	1:00:13	66.8813582	71.846326	87.421441	70.1725398
21:03:03	1:00:14	66.8813582	71.846326	87.421441	70.1725398
21:03:04	1:00:15	66.8813582	72.846326	88.421441	70.1725398
21:03:05	1:00:16	66.8813582	72.846326	88.421441	71.1725398
21:03:06	1:00:17	66.8813582	72.846326	91.421441	71.1725398
21:03:07	1:00:18	66.8813582	73.846326	91.421441	71.1725398
21:03:08	1:00:19	66.8813582	73.846326	94.421441	72.1725398
21:03:09	1:00:20	65.8813582	73.846326	94.421441	72.1725398
21:03:10	1:00:21	65.8813582	69.846326	97.421441	72.1725398
21:03:11	1:00:22	65.8813582	69.846326	97.421441	75.1725398
21:03:12	1:00:23	65.8813582	68.846326	95.421441	76.1725398
21:03:13	1:00:24	65.8813582	68.846326	95.421441	76.1725398
21:03:14	1:00:25	65.8813582	67.846326	95.421441	76.1725398
21:03:15	1:00:26	65.8813582	66.846326	94.421441	76.1725398
21:03:16	1:00:27	65.8813582	66.846326	93.421441	76.1725398
21:03:17	1:00:28	65.8813582	66.846326	92.421441	76.1725398
21:03:18	1:00:29	65.8813582	66.846326	92.421441	76.1725398
21:03:19	1:00:30	65.8813582	66.846326	92.421441	76.1725398
21:03:20	1:00:31	66.8813582	66.846326	91.421441	74.1725398
21:03:21	1:00:32	65.8813582	66.846326	91.421441	68.1725398
21:03:22	1:00:33	65.8813582	64.846326	88.421441	67.1725398
21:03:23	1:00:34	65.8813582	64.846326	87.421441	66.1725398
21:03:24	1:00:35	65.8813582	63.846326	84.421441	65.1725398
21:03:25	1:00:36	65.8813582	63.846326	84.421441	65.1725398
21:03:26	1:00:37	65.8813582	62.846326	84.421441	64.1725398
21:03:27	1:00:38	64.8813582	62.846326	84.421441	64.1725398
21:03:28	1:00:39	64.8813582	61.846326	84.421441	66.1725398
21:03:29	1:00:40	64.8813582	61.846326	83.421441	68.1725398
21:03:30	1:00:41	64.8813582	60.846326	83.421441	67.1725398
21:03:31	1:00:42	64.8813582	60.846326	83.421441	67.1725398

21:03:32	1:00:43	64.8813582	60.846326	83.421441	65.1725398
21:03:33	1:00:44	64.8813582	60.846326	82.421441	64.1725398
21:03:34	1:00:45	64.8813582	60.846326	82.421441	64.1725398
21:03:35	1:00:46	64.8813582	61.846326	82.421441	64.1725398
21:03:36	1:00:47	64.8813582	61.846326	82.421441	64.1725398
21:03:37	1:00:48	65.8813582	64.846326	82.421441	67.1725398
21:03:38	1:00:49	65.8813582	62.846326	82.421441	67.1725398
21:03:39	1:00:50	65.8813582	61.846326	83.421441	67.1725398
21:03:40	1:00:51	65.8813582	61.846326	84.421441	65.1725398
21:03:41	1:00:52	66.8813582	61.846326	84.421441	65.1725398
21:03:42	1:00:53	67.8813582	62.846326	84.421441	65.1725398
21:03:43	1:00:54	67.8813582	62.846326	83.421441	65.1725398
21:03:44	1:00:55	66.8813582	64.846326	86.421441	65.1725398
21:03:45	1:00:56	66.8813582	65.846326	82.421441	65.1725398
21:03:46	1:00:57	66.8813582	65.846326	80.421441	66.1725398
21:03:47	1:00:58	66.8813582	65.846326	80.421441	66.1725398
21:03:48	1:00:59	67.8813582	66.846326	80.421441	68.1725398
21:03:49	1:01:00	68.8813582	66.846326	80.421441	71.1725398
21:03:50	1:01:01	68.8813582	66.846326	80.421441	71.1725398
21:03:51	1:01:02	68.8813582	67.846326	80.421441	69.1725398
21:03:52	1:01:03	68.8813582	69.846326	80.421441	69.1725398
21:03:53	1:01:04	70.8813582	71.846326	80.421441	68.1725398
21:03:54	1:01:05	71.8813582	71.846326	80.421441	68.1725398
21:03:55	1:01:06	72.8813582	71.846326	81.421441	68.1725398
21:03:56	1:01:07	72.8813582	71.846326	81.421441	69.1725398
21:03:57	1:01:08	73.8813582	71.846326	81.421441	70.1725398
21:03:58	1:01:09	73.8813582	70.846326	82.421441	64.1725398
21:03:59	1:01:10	73.8813582	70.846326	82.421441	62.1725398
21:04:00	1:01:11	72.8813582	70.846326	82.421441	62.1725398
21:04:01	1:01:12	69.8813582	70.846326	82.421441	62.1725398
21:04:02	1:01:13	69.8813582	69.846326	82.421441	62.1725398
21:04:03	1:01:14	69.8813582	69.846326	82.421441	63.1725398
21:04:04	1:01:15	68.8813582	69.846326	83.421441	65.1725398
21:04:05	1:01:16	68.8813582	68.846326	83.421441	67.1725398
21:04:06	1:01:17	68.8813582	68.846326	83.421441	73.1725398
21:04:07	1:01:18	68.8813582	68.846326	81.421441	73.1725398
21:04:08	1:01:19	67.8813582	67.846326	79.421441	73.1725398
21:04:09	1:01:20	67.8813582	67.846326	78.421441	73.1725398
21:04:10	1:01:21	67.8813582	78.846326	78.421441	72.1725398
21:04:11	1:01:22	66.8813582	81.846326	78.421441	71.1725398
21:04:12	1:01:23	66.8813582	80.846326	78.421441	71.1725398
21:04:13	1:01:24	66.8813582	77.846326	78.421441	71.1725398
[truncated: 356,702 more chars]
